# Inferring copy number and genotype in tumour exome data

**DOI:** 10.1186/1471-2164-15-732

**Published:** 2014-08-28

**Authors:** Kaushalya C Amarasinghe, Jason Li, Sally M Hunter, Georgina L Ryland, Prue A Cowin, Ian G Campbell, Saman K Halgamuge

**Affiliations:** Optimisation and Pattern Recognition group, Mechanical Engineering Department, Melbourne School of Engineering, The University of Melbourne, Parkville, Victoria 3010 Australia; Bioinformatics Core Facility, Peter MacCallum Cancer Centre, East Melbourne, Victoria 3002 Australia; Cancer Genetics Laboratory, Peter MacCallum Cancer Centre, East Melbourne, Victoria 3002 Australia; Cancer Genomics and Genetics Laboratory, Peter MacCallum Cancer Centre, East Melbourne, Victoria 3002 Australia; Sir Peter MacCallum Department of Oncology, The University of Melbourne, Parkville, Victoria 3010 Australia; Department of Pathology, The University of Melbourne, Parkville, Victoria 3010 Australia

## Abstract

**Background:**

Using whole exome sequencing to predict aberrations in tumours is a cost effective alternative to whole genome sequencing, however is predominantly used for variant detection and infrequently utilised for detection of somatic copy number variation.

**Results:**

We propose a new method to infer copy number and genotypes using whole exome data from paired tumour/normal samples. Our algorithm uses two Hidden Markov Models to predict copy number and genotypes and computationally resolves polyploidy/aneuploidy, normal cell contamination and signal baseline shift. Our method makes explicit detection on chromosome arm level events, which are commonly found in tumour samples. The methods are combined into a package named ADTEx (Aberration Detection in Tumour Exome). We applied our algorithm to a cohort of 17 in-house generated and 18 TCGA paired ovarian cancer/normal exomes and evaluated the performance by comparing against the copy number variations and genotypes predicted using Affymetrix SNP 6.0 data of the same samples. Further, we carried out a comparison study to show that ADTEx outperformed its competitors in terms of precision and F-measure.

**Conclusions:**

Our proposed method, ADTEx, uses both depth of coverage ratios and B allele frequencies calculated from whole exome sequencing data, to predict copy number variations along with their genotypes. ADTEx is implemented as a user friendly software package using Python and R statistical language. Source code and sample data are freely available under GNU license (GPLv3) at http://adtex.sourceforge.net/.

**Electronic supplementary material:**

The online version of this article (doi:10.1186/1471-2164-15-732) contains supplementary material, which is available to authorized users.

## Background

Tumourigenesis is associated with the acquisition of genomic aberrations [1, 2] including copy number alterations (CNAs) and loss of heterozygosity (LOH), which activate oncogenes or deactivate various classes of genes that play crucial roles in cancer development [1]. Previously, such data has been generated from array comparative genomic hybridisation (aCGH) and single nucleotide polymorphism (SNP) genotyping arrays [3–5] but the implementation of massively parallel sequencing (MPS) technologies has provided novel opportunities for using sequencing data to generate equivalent genomic aberration information. In the cancer genomics field it has become a routine to perform whole genome sequencing (WGS) or whole exome sequencing (WES) on DNA extracted from tumour tissues [2, 6, 7]. WES is particularly popular for large sequencing projects seeking to identify disease-specific mutations since it is significantly cheaper than WGS and involves reduced analytical complexity, but typically seeks only to identify single nucleotide variants and small insertions-deletions [8, 9]. CNAs have been successfully detected in gene panel targeted resequencing projects [10], however, the bioinformatics tools for upscaling this to exomes are lacking. With the efforts of large sequencing consortia, such as The Cancer Genome Atlas (TCGA) network and International Cancer Genome Consortium (ICGC) [11] and individual research groups, many whole exome sequencing projects involving thousands of tumours are currently underway. However, somatic CNA identification by means of WES data is still in its early stages and needs the development of new robust computational methods and algorithms.

Computational methods have been published for detecting CNAs in targeted resequencing data including whole exome sequencing [7, 12–19], although majority of these methods are designed for analysing variations in germline DNA and perform poorly when applied to the detection of somatic CNAs in tumour samples. Control-FREEC [20] is a method developed for WGS tumour data and more recently supports the application to WES data. ExomeCNV [7] and VarScan 2 [14] are designed for CNA identification in tumour WES data, however, they do not predict absolute copy number in non-diploid samples. Other methods can predict absolute copy numbers in non-diploid tumour samples, but only if the ploidy is known *a priori* [12, 20] which is rarely the case or is impractical to obtain.

Simultaneous generation of depth of coverage (DOC) ratios and B allele frequencies (BAFs) would facilitate the identification of aneuploidy and polyploidy present in cancer samples. A diploid genome would only have BAFs of 0, 0.5 and 1, corresponding to AA, AB and BB genotypes, whereas, for example, a triploid genome, assuming no tumour heterogeneity or normal contamination, would have allele ratios of 0, 0.33, 0.67 and 1 regions corresponding to AAA, AAB, ABB and BBB genotypes and a baseline DOC ratio of one which is similar to a diploid tumour genome. To generate ratios and allele frequencies from WES data, we need to align them to the reference genome and identify the SNPs. Another issue impeding the use of WGS and WES for somatic CNA identification is non-tumour cell contamination, which is present in the majority of tumour tissues. Normal DNA contamination attenuates the signal-to-noise ratio in BAF and coverage ratio signals by altering their values towards a normal diploid genome pattern. Consequently, a high normal cell contamination would make it impossible to differentially detect somatic variations in tumour cells. Among the previously published methods, ExomeCNV [7] requires tumour purity to be known *a priori* while Control-FREEC [20] does not. Previous studies on SNP genotyping array data [4, 21] suggested the use of Hidden Markov Models (HMM) to predict CNA and LOH events with a parameter training procedure, which inherently models the normal contamination.

When analysing exome sequencing data, it is important to overcome the intrinsic noise present in data itself, which hinders its ability to accurately predict CNAs. Programs such as XHMM [13] and CoNIFER [15], which are applicable to CNA detection in germline DNA studies, perform principle component analysis and singular value decomposition, to remove the noise present in WES data and normalise the read counts. However, these methods are not applicable in single tumour/matched normal sample pairs. A potential way to overcome this issue is to implement discrete wavelet transformation (DWT) in normalising exome ratios as we have reported previously [12]. DWT normalisation can achieve higher precision (that is, lower number of false positives) while maintaining a comparable or superior sensitivity compared to other methods.

Overall, the three above mentioned issues, (i) noise in WES data, (ii) ploidy and (iii) normal cell contamination in tumour samples have not been simultaneously evaluated by any of the computational methods applicable to WES tumour data. Although our previously proposed method [12] considers these three issues, it requires prior knowledge of contamination and tumour ploidy. Therefore, in the current study, we propose a new approach named Aberration Detection in Tumour Exome (ADTEx), which automatically estimates the three aspects important to WES tumour data and predicts CNA events and genotypes of SNPs associated with these regions. Further, ADTEx makes explicit predictions on chromosome arm level CNA events, which is a pattern commonly observed across many tumour types.

## Results and discussion

### ADTEx for aberration detection in tumour exome

ADTEx consists of two HMMs to predict copy number alterations and genotypes in WES data of paired tumour/normal samples. Two types of signals were generated from the exome data, DOC ratios and BAFs. Copy number analysis using ratios can be complemented by the computation of BAFs to determine ploidy and zygosity. Here, we propose to apply these two types of signals to predict the zygosity state of segments in the genome targeted by exome capture.

One HMM is used to predict CNAs, which in combination with BAF signal can be used to estimate ploidy of the tumour and predict the absolute copy numbers. A second HMM is used to predict zygosity or genotype of each CNA segment. The overall framework of the method is given in Additional file 1: Figure S1. We applied our method to 17 in-house tumour samples derived from ovarian cancer patients to assess the performance of the method. We selected ten samples with different aberration types from those 17 samples to compare the performance of ADTEx against existing somatic CNA prediction methods. Further, we evaluated the performance of ADTEx on publicly available 18 paired ovarian cancer/normal samples downloaded from the TCGA project.

The parameters in our two HMMs were trained using an expectation maximisation (EM) algorithm [22]. Given these estimated parameters, the maximum likelihood of the hidden state sequence is determined using Viterbi algorithm [23]. In the first HMM, to detect copy number, we applied EM algorithm for each chromosome separately, while in the second HMM, we pooled all chromosomes and estimated parameters jointly. However, in the second HMM, the initial state distribution was computed separately for each chromosome.

### Aberrations detection in 17 ovarian cancer samples

We used 17 paired ovarian tumour/normal samples to evaluate the performance of our proposed method (Table 1 and Additional file 1: Table S1). The samples were sequenced on an Illumina HiSeq 2000 (one pair) and the Illumina Genome Analyzer IIx (16 pairs). Exon capture was performed using the 51 Mb Agilent SureSelect Human All Exon V4 (one pair), the 36.5 Mb Roche NimbleGen EZ Exome SeqCap V2 (11 pairs) and the 26 Mb Roche NimbleGen EZ Exome SeqCap V1 platform (five pairs). Each WES sample was aligned to the reference genome, GRCh37, using BWA [24]. The predicted aberrations in exome samples were validated by Affymetrix SNP 6.0 data generated for the same samples. ASCAT [25] was used to predict CNAs from the SNP array data.

**Table 1:** **Summary of the exome sequencing data**

Exome platform	Agilent SureSelect Human All Exon Version 4	Roche NimbleGen EZ Exome SeqCap Version 2	Roche NimbleGen EZ Exome SeqCap Version 1
**No of paired samples**	1 × 2	11 × 2	6 × 2
**Target size**	51Mbp	36Mbp	26Mbp
**Sequencing platform**	Illumina HiSeq	Illumina GAIIx	Illumina GAIIx
**Read length**	101 bp	100 bp	79 bp, 100 bp and 101 bp
Avg. mapped reads (BWA)*	102,082,760	86,607,431	77,433,963
Avg. on target reads*	95,930,467	78,038,985	70,758,116
Avg. bases mapped to target regions*	9.7Gbp	7.8Gbp	6.8Gbp
**Average coverage per targeted base**	189.28	216.91	204.68

*Per sample.

### Correlation between SNP array data and WES data

Manual inspection of the ratio plots between SNP 6.0 array data and whole exome sequencing data proved to be highly consistent. We also observed statistically significant positive correlation (Additional file 1: Figure S2) between SNP 6.0 data ratios and exome depth of coverage ratios. To obtain these, we partitioned the exome into windows containing five exons and computed the mean normalised DOC ratios in each partitioned window. Mean SNP 6.0 intensity ratios were calculated from the probes overlapping the exonic windows. The Spearman’s rank correlation was calculated between these two data sets for three different tumour samples (Additional file 1: Figure S2) and the Spearman’s rho ranged from 0.63 to 0.81 (p value <0.001). This evaluation demonstrates that WES is comparable with SNP 6.0 array data for the analysis of CNAs.

#### Polyploidy detection in exome data

To predict the copy number of each exonic locus, we first needed to establish a method for predicting the polyploidy status of each tumour sample. Additional file 1: Figure S3 shows the properties of BAF to detect correct ploidy by our method. In each case, ADTEx accurately determined the copy number status of the regions corresponding to baseline ratio, by comparing BAFs and predicted copy number. This estimation is only possible when the BAFs of the tumour sample at normal heterozygous loci are available, DOC ratios alone would not allow correcting for this.

**Figure 1:**
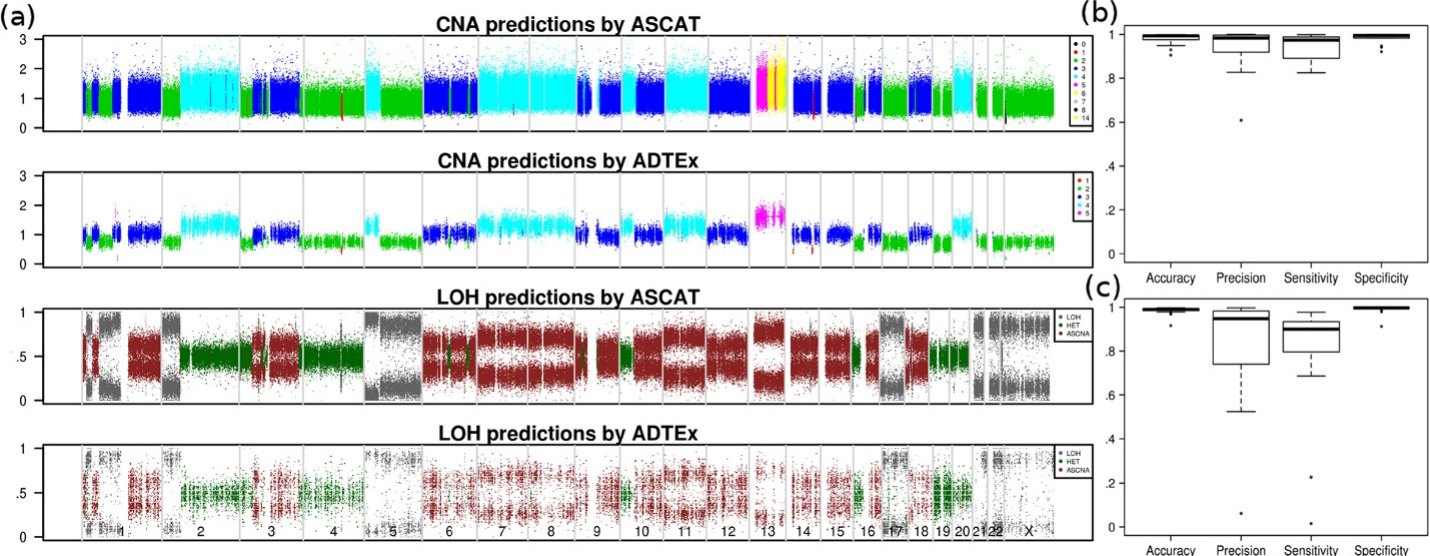
**Performance comparison and evaluation of ADTEx predictions against data from Affymetrix SNP 6.0 genotyping arrays. (a)** Comparison of predictions on sample OV12. Copy number predictions by ASCAT (top panel), copy number predictions by ADTEx (second panel), LOH predictions by ASCAT (third panel) and LOH predictions by ADTEx (bottom panel). The top two panels show the exon level depth of coverage ratios and each colour represents the predicted copy number, while bottom two panels show the tumour BAF of heterozygous loci in the matched normal sample with colours representing the predicted LOH status. **(b)** Performance metrics of ADTEx copy number predictions computed as accuracy, precision, sensitivity and specificity and **(c)** performance metrics of ADTEx LOH predictions on all samples.

Overall, prediction accuracies of the ploidy detection were measured by calling copy number at each exonic locus and validating them against the calls made by ASCAT on SNP 6.0 array data (Figure 1 and Additional file 1: Table S2).

#### Comparison with SNP genotyping array data

CNA were evident in 14 of the 17 samples, which were therefore used to evaluate the performance of CNA predictions. LOH was predicted in all 17 samples (including three samples with copy neutral LOH), which were therefore used in the genotype prediction evaluation. A representative comparison between exome results and SNP 6.0 results are shown in Figure 1a for the sample OV12.

We evaluated ADTEx predicted CNAs against those predicted by ASCAT on SNP 6.0 array data, which we assumed as the ground truths. Sensitivity, specificity, precision and accuracy were computed for each sample (Figure 1 and Additional file 1: Table S2). Each exon was treated as a point of measure for the performance calculation with true positives (TPs) considered those exons identified by both ASCAT and ADTEx as gains/losses and false positives (FPs) considered those exons predicted by ADTEx as gains/losses and copy neutral by ASCAT. True negatives (TN) and false negatives (FN) were recognised in the same manner. ADTEx had median values of 94.1% sensitivity, 98.3% specificity, 94% precision and 98% accuracy for detecting CNAs. Triploidy and tetraploidy were each present in 2 of 14 samples while most of other samples were aneuploid (Additional file 1: Figure S4 and Additional file 2). As shown in Additional file 1: Figure S5a, a high proportion of the genome was affected by duplications with an average of 26.6% of the genome amplified compared with just 4% deleted. The smallest deletion and amplification detected by ADTEx were 100pb and 80 bp long respectively, while the largest deletion and amplification were 181Mbp and 243Mbp in length respectively. The resolution of the smallest CNA detected was restricted to the smallest exon detected as a CNA.

For large scale (>0.1 of the chromosome) CNA events, we further assessed the performance of ADTEx based on the number of events detected by the method. According to the results from SNP arrays, there were 150 large scale events in all samples (Additional file 1: Table S11). ADTEx detected 145 events, which is a sensitivity of 96.7%. For the assessment, an event predicted by ASCAT is considered to be correctly detected by ADTEx when there is more than 50% overlap between the predictions made by two methods.

Figure 1c shows the performance measurements of ADTEx on LOH predictions, evaluated considering ASCAT predictions as ground truths (Additional file 1: Table S3). The heterozygous SNP loci in matched normal sample were retained for this analysis. Further, we filtered out the SNP loci that fell outside of the regions of the predicted copy number variant and copy neutral segments for the relevant sample. Each SNP locus was considered as a performance measurement point with true positives considered those SNP loci identified as having LOH by both ASCAT and ADTEx and false positive events considered those SNP loci defined by ASCAT as non-LOH but predicted by ADTEx as LOH. Median values of sensitivity and specificity were 90.1% and 99.7%.

The distribution of the total length of LOH of a sample ranged from a minimum of 0.1 Mb to maximum of 1,577 Mb with a mean of 273 Mb. Additional file 1: Figure S5b shows the distribution of the genomic proportion of allelic imbalance presents in each sample. Additional file 1: Figure S6 shows different types of LOH events identified using ADTEx on whole exome sequencing data.

Performance metrics for detecting allele specific copy number alterations (ASCNA) were reported as median sensitivity of 96.8% and specificity of 98.2% and are summarised in Additional file 1: Table S4 in terms of sensitivity, specificity, precision and accuracy.

### Performance evaluation on TCGA data

Next, we evaluated the performance of ADTEx on high-grade serous ovarian adenocarcinoma samples sequenced as part of TCGA project [26]. We downloaded BAM files of 18 paired tumour/normal samples sequenced at Washington University from the Cancer Genomics Hub (CGHub). These were sequenced using Illumina Genome Analyzer IIx and target capture was performed by Agilent SureSelect Human exome platform. All samples have been aligned to the GRCh37-lite. The number of reads mapped to the targeted regions ranged from 57,215,953 to 118,126,167 (Additional file 1: Table S5).

To evaluate the somatic CNA detection of ADTEx, we also downloaded the raw Affymetrix SNP 6.0 files of the same samples from TCGA data portal. Then, as before, we carried out CNA detection on SNP 6.0 data using ASCAT algorithm [25]. These results were treated as the ground truths for the evaluation. Each sample has very high aberration rate with focal and large scale CNAs, typical of this tumour type.

In detecting somatic CNA, ADTEx showed median sensitivity of 93.7%, precision 79.3% and F-measure of 83.0%. F measure was computed using the following equation.


The largest detected CNA segment was 242,433,351 bp long in length and smallest detected CNA was 120 bp long. On average 1,035 CNA segments per tumour were detected by ADTEx (Additional file 3). In all samples, 76,341 exons were identified as losses and 2,357,365 exons were identified as gains. Therefore, for each tumour sample there were about 135,000 altered exons and this number is consistent with the reported values [14] for ovarian cancer samples. Sensitivity, precision and F-measure for detecting LOH were computed as 92.9%, 96.3% and 94.5% respectively, relative to ASCAT predictions.

### Comparison with other copy number predicting algorithms

#### Methods compared

In order to demonstrate the effectiveness of our proposed method, we carried out a comparison between ADTEx and other somatic CNA detecting algorithms. We selected ExomeCNV [7], VarScan 2 [14] and Control-FREEC [20] for the comparison as they were developed for WES data generated from paired tumour/normal samples. Further, we evaluated the performance of our previous work described in Amarasinghe *et al*., 2013 [12] and details of the evaluation are given below separately in the section “Comparison with previous work”. We selected ten samples from a in-house data set, with different copy number aberrations to compare the competing methods. These samples contain focal aberrations, chromosome arm-level and full chromosomal events. In all cases we used SAMtools pileup/mpileup [27] to generate coverage files as inputs for the three competing exome based methods. Supplementary Methods section in Additional file 1 describes the parameter settings used with each method.

#### Results from the comparison study

**Figure 2:**
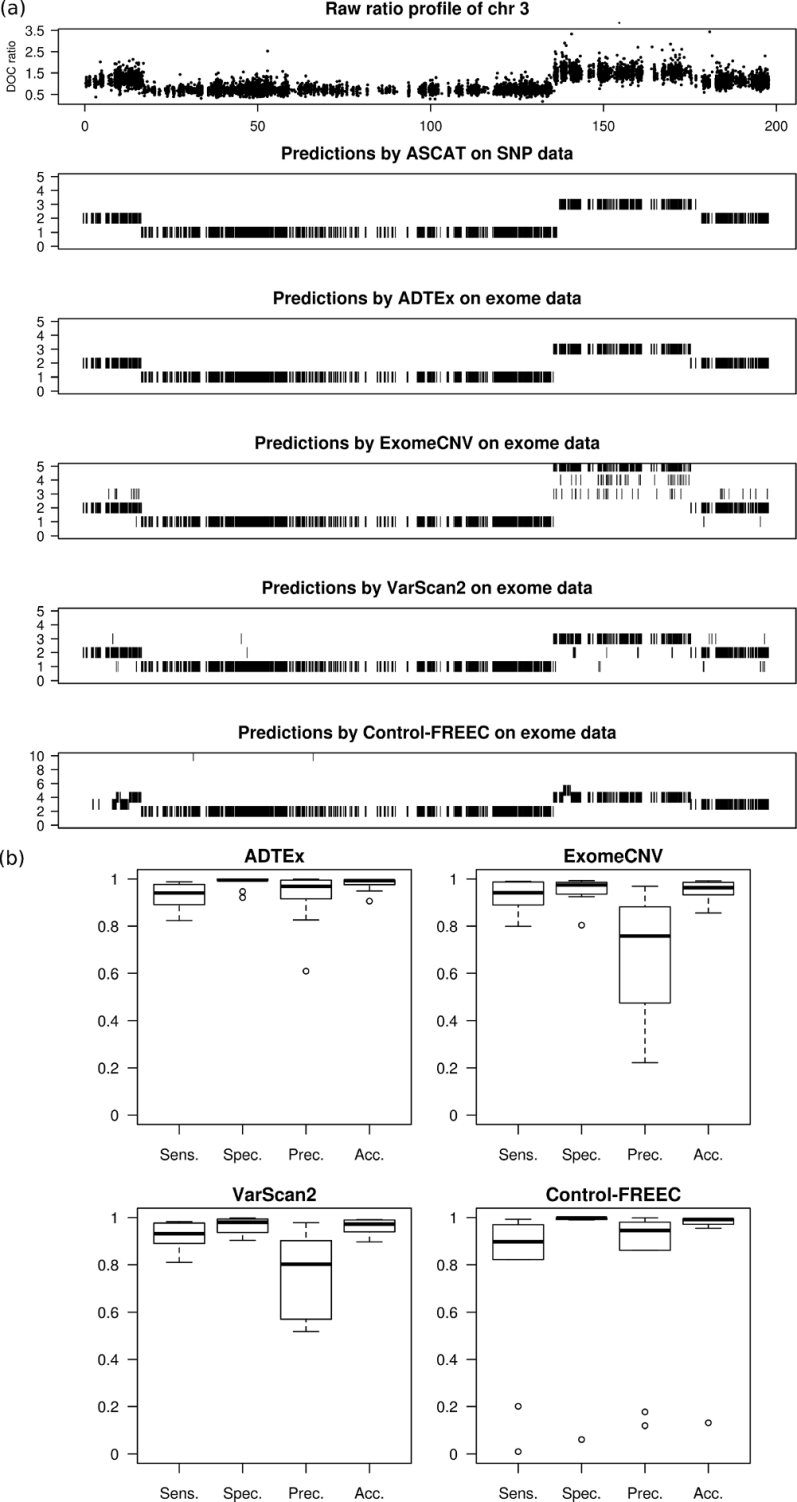
**Results from the performance comparison of ADTEx with existing methods. (a)** Top panel shows the DOC ratios of chromosome 3 of sample OV1. Second panel shows the predictions by ASCAT on SNP 6.0 data. Bottom 4 panels show copy number predictions by each of the methods. **(b)** Performance metrics of ADTEx, ExomeCNV, VarScan2 and Control-FREEC on nearly diploid tumour samples. The results are based on comparison against predictions by ASCAT on SNP 6.0 data as ground truths.

The overall performance of the four methods is shown in Figure 2 and Table 2. Additional file 1: Table S6 gives the performance matrix of each method on each sample. VarScan2 does not predict the absolute copy number, instead it predicts gains/losses and copy neutral regions. Accordingly, in Figure 2a, VarScan2 result shows losses, neutral regions, and gains as having copy 1, 2 and 3. Figure 2b shows the performance metrics of each method in terms of sensitivity, specificity, precision and accuracy. We were particularly interested in sensitivity [no. of TP/(no. of TP + no. of FN)] and precision [no. of TP/(no. of TP + no. of FP)] as the performance measures to base our comparison. Therefore, we report the F measure values here.

**Table 2:** O**verall performance for CNA detection of each method in terms of mean / median values**

Method	ADTEx	ExomeCNV	VarScan 2	Control-FREEC	Method [ [12]]
Sensitivity	**92.5%** / 94.1%	92.3% / **94.2%**	91.9% / 93.2%	75.5% / 89.8%	86.9% / 88.3%
Specificity	**98.5%** / 99.5%	95.1% / 97.5%	96.3% / 98.0%	90.3% / **99.8%**	93.8% / 99.6%
Precision	**91.9%** / **96.8%**	68.2% / 75.8%	76.4% / 80.2%	78.7% / 94.6%	84.6% / 92.9%
Accuracy	**97.9%** / **99.3%**	95.3% / 96.4%	96.0% / 97.2%	90.1% / 99.2%	94.5% / 98.9%
F-Measure	**91.5%** / **92.3%**	76.5% / 90.6%	82.0% / 84.7%	76.0% / 82.5%	85.2% / 87.8%

In the table, bold value in each line represents the best value of each performance measure.

The calculated mean (median) F-measure values for ADTEx, ExomeCNV, VarScan2 and Control-FREEC were 91.5% (92.3%), 76.5% (90.6%), 82.0% (84.7%) and 76.0% (82.5%) respectively. Overall, both mean and median performance scores of ADTEx were better compared to other methods. VarScan2 ranked second and ADTEx showed 12% increase in terms of mean F measure over VarScan2. As per the Table 2, ADTEx showed superior or comparable performance in terms of mean and median values of all performance measures compared to other competing methods. The main reason for superior results produced by ADTEx compared to other methods is that it simultaneously evaluates specific characteristics of tumour WES data, namely (i) noise, (ii) ploidy and (iii) normal cell contamination.

Figure 2a shows the copy number predictions of the four methods on chromosome 3 for the sample OV1. Interestingly, Control-FREEC appeared to misidentify the normal regions as it misjudged the baseline of the ratios, resulting in the prediction of copy neutral regions as amplifications and deletions as copy neutral.

#### Comparison of small CNAs

SNP genotyping arrays cannot be used to detect smaller-size CNAs (<1Mbp) due to limited resolution. To assess the performance at this level, we compared smaller (<1Mbp) CNAs detected by ADTEx with three other exome based methods. Overall, ADTEx, ExomeCNV, VarScan 2 and Control-FREEC detected 448, 7167, 4618 and 1494 CNA events, respectively, of which 79%, 94%, 93% and 82% were identified as smaller than 1Mb. Identification of high percentages of smaller events by all four methods can be attributed to the sparse nature of WES data. As ADTEx performs noise reduction in WES data, many of the smaller CNAs predicted by other three methods are integrated into larger CNAs predicted by ADTEx. The overlap between other methods and 354 smaller CNAs identified by ADTEx is given in Additional file 1: Table S12. VarScan 2 identified 164 of those events, which has the highest concordance rate.

#### Comparison of LOH and ASCNA predictions

Control-FREEC predicts LOH and ASCNA events similar to ADTEx, hence we compared ADTEx LOH and ASCNA predictions against the results from Control-FREEC (Table 3 and Additional file 1: Table S7 and Table S8). Five samples with different variations were chosen to carry out the comparison. ADTEx outperformed Control-FREEC in terms of precision and F-measure. The mean F-measure and precision of ADTEx were calculated respectively as 89.5% and 89.1% while that of Control-FREEC were 46.3% and 37.0% with respect to LOH predictions. The mean F-measure and precision values for ASCNA predictions were 83.7% and 82.0% for ADTEx and 66.1% and 68.1% for Control-FREEC respectively.

**Table 3:** **Comparison between LOH predictions of ADTEx and Control-FREEC**

Sample	Sensitivity		Precision		F-measure	
	ADTEx	C-FR*	ADTEx	C-FR*	ADTEx	C-FR*
OV1	97.8%	95.3%	98.0%	94.1%	97.9%	94.7%
OV2	79.1%	93.1%	59.6%	2.5%	68.0%	4.9%
OV4	93.7%	100%	98.3%	45.6%	95.9%	62.6%
OV7	92.6%	94.9%	93.8%	26.2%	93.2%	41.1%
OV11	89.5%	100%	96.0%	16.5%	92.6%	28.3%

*Control-FREEC.

#### Comparison with previous work

We described a copy number predicting algorithm in Amarasinghe et al., 2013 [12]. The main differences between current work and the algorithm in Amarasinghe *et al*., 2013 [12] are i) no *a priori* knowledge of contamination or ploidy is required and ii) genotype status of SNPs in each of the CNA segment predicted by ADTEx. We evaluated the performance of Amarasinghe et al., 2013 [12] using the ten samples in the comparison study and also applied the method on triploid sample OV8 (Table 2 on diploid samples and Additional file 1: Table S9). The ploidy and contamination values were chosen based on manual confirmation and prediction made by ASCAT [25] on SNP 6.0 data of the same samples. For the ten diploid samples we saw 4% and 5% increases in median precision and F-measure values respectively for ADTEx compared to Amarasinghe et al., 2013 [12]. Further, for the OV8 sample ADTEx showed clear improvements in terms of sensitivity, specificity, precision and accuracy.

### Evaluation of the effect of normal cell contamination

We carried out computer simulations to evaluate the effect of normal cell contamination on CNA and LOH predictions by ADTEx. We use OV1 data sample to generate different combination of data sets with 0.1 to 0.7 normal cell contamination and read depths of 150X (original coverage), 60X and 400X. The original normal cell contamination is predicted as 25%, therefore the expected contamination levels can be calculated as 0.325, 0.4, 0.475, 0.55, 0.625, 0.7 and 0.775 respectively. For original coverage, ADTEx accurately predicted the normal cell contamination up to 0.7 (Pearson correlation 0.99). Additional file 1: Figure S7 shows the changes in the predicted values. The F measure performance on CNA predictions (Additional file 1: Figure S8) was reported taking the SNP 6.0 results as ground truths. Relatively high F measure value is maintained (F measure 0.66) at the 0.55 contamination level for CNA detection. The F measure performance of LOH predictions (Additional file 1: Figure S9) suggests ADTEx performed well even at 0.625 normal cell contamination level (F measure 0.96). At high levels of contamination (>0.7), prediction of CNA is difficult due to the very low level of variation in the depth of coverage ratios. Further, 150X and 400X coverage showed better performance compared to 60X coverage.

### Chromosome arm level copy number aberrations

Chromosome arm level or full chromosomal CNAs are commonly observed across many tumour types [28, 29]. Different tumour types have been reported to have recurrent arm level events on different chromosomes. For example gain in chromosome 3 or 3q are more common in cervical cancer while loss of chromosome 10 is common in glioblastomas [28].

ADTEx explicitly predicts chromosome arm level events based on the results generated by exon level copy number predictions. The distribution of DOC ratios of copy neutral regions is calculated from the exon level result. Then, statistical confidence level (assuming no CNA) for each chromosome arm considering the mean DOC ratio is produced based on the calculated distribution. At the 0.05 confidence level we were able to detect 44 chromosome p/q level events (15 gains and 29 losses) and 30 full chromosome CNA events (15 of each gains and losses) in the 17 in-house ovarian tumour samples. According to the carefully curated list of chromosome arm level CNAs (Additional file 1: Table S10) in those ovarian cancer samples, ADTEx showed 96% sensitivity and 99% specificity. Four single copy losses and one single copy gain residing in samples with higher ploidy were not detected as significant compared to copy neutral level.

## Conclusion

We have described a new approach to infer somatic CNAs and genotype states in WES data from tumour samples. Our method both models and evaluates tumour related attributes in WES data. Further, the proposed method explicitly predicts chromosome arm level CNA events, which are commonly found in many tumour types. We implemented this approach in a software called “Aberration Detection in Tumour Exome (ADTEx)”, which is freely available under GNU General Public Licence v3 (GPLv3). To our knowledge, ADTEx is the first attempt to computationally derive absolute copy numbers and genotypes using WES data from tumour samples without any *a priori* knowledge of levels of normal DNA contamination or ploidy of the tumour samples. The algorithm takes DOC ratios and BAFs as inputs and models them using Gaussian distribution. Prior to applying HMM to derive CNAs and genotypes, the DOC ratios are smoothed by discrete wavelet transformation techniques. We applied the algorithm to 35 (in-house and public data) paired ovarian tumour/normal samples captured using three different targeted capture platforms and sequenced using Illumina Genome Analyzer II or HiSeq2000 sequencers. Further, to our knowledge ADTEx is the only method that predicts chromosome arm level CNA in WES data.

We demonstrated the superior performance of ADTEx compared to existing methods. Most importantly, we compared the performance of ADTEx against the results generated by ASCAT on Affymetrix SNP 6.0 data and showed that our method can produce results consistent with SNP array data, the gold standard for detecting CNAs. We believe that the integrated CNA and LOH predictions in ADTEx will greatly improve the type and usefulness of the data generated in large WES studies.

However, We have not addressed the issue of tumour heterogeneity where some components of the tumour biopsy will have different clonal outgrows harbouring different genetic alterations. Clonal heterogeneity will result in an amalgamation of the signals present in both subpopulations which may result in a reduced sensitivity of ADTEx. This could be a future research direction that can be pursued using WES data.

## Methods

ADTEx processing pipeline is shown in Additional file 1: Figure S1. Overall, the method consists of two HMMs. One HMM predicts copy number alterations using noise reduced DOC ratios and the other HMM predicts tumour zygosity states at heterozygous SNP loci in normal samples. We have proposed a two stage HMM algorithm, which ensures:

i) Increased performance in predicting CNAs by taking all data points that are available (depth of coverage ratios) for the evaluation of first HMM and then calling zygosity on SNP loci in the second HMM.
ii) Computational efficiency, achieved through dimensionality reduction in the first stage of HMM.

### Calculation of DOC ratio and BAF

DOC ratios are calculated as the ratios between average coverage per base of exonic regions in tumour and matched normal samples using the following steps: (i) coverage per base at targeted regions are calculated by BEDTools software [30], (ii) average coverage per base is calculated for each exonic region, (iii) regions with lower average coverage than a predefined threshold (=10 reads) are excluded from the analysis, (iv) mean coverage normalisation is performed for each sample and (v) ratio between mean normalised DOC of tumour and matched normal samples are calculated. These ratios showed extensive intrinsic noise particularly in low coverage regions. We applied DWT denoising on ratios generated from low coverage regions. DWT denoising helps to achieve a higher sensitivity and precision [12].

BAF is calculated as the ratio between number of B alleles and total number of A and B alleles. Here, ‘A allele’ refers to the reference allele and ‘B allele’ refers to the non-reference allele in DNA sequencing data when the sequence reads are aligned to the reference genome. We calculated SNP loci in both tumour and matched normal samples by applying The Genome Analysis Toolkit (GATK) software [31]. BAF at each SNP is then calculated applying the following formula,


For example, based on the above formula BAF should be around 0, 0.5 and 1 for genotypes AA, AB and BB, respectively. If a BAF is deviated from these values, it would mean that there is a possibility of copy number alteration. For instance, a BAF value of 0.25 indicates a genotype of AAAB.

### DOC ratio baseline evaluation

DOC ratios can be used as an indication of the relevant copy number present in tumour sample compared to the matched normal sample. Copy number of a particular region and the DOC ratio has a direct relationship in non-cancerous samples. For example, ratios of one and two represent copy number two and four respectively. However, due to the presence of extensive abnormalities in tumour samples, mean DOC ratio will differ from the nominal ratio of one. This change in the signal is known as the baseline shift (Additional file 1: Figure S3). We corrected for the baseline by identifying the peaks of the distribution of the DOC ratios. Ratios are normalised based on the value of the peak closest to one.

On the other hand, the baseline ratio would not correspond to copy number of two in most of the tumour samples due to the presence of polyploidy and aneuploidy. We successfully identified the copy number of the baseline by evaluating BAF of each tumour sample. Correct identification of the copy number of the baseline made it possible to predict the absolute copy number in the tumour samples. Our approach, which detects the baseline ploidy is explained in the following section.

### HMM to predict copy number variations and identification of baseline ploidy

The definitions of hidden states are described in detail in Additional file 1: Supplementary Methods. We model the emission probability of DOC ratios by a Gaussian distribution. The mean of the distribution depends on the hidden state while standard deviation remains constant for all states. Standard deviation was kept constant after analysing the data and observing that there is not much impact on the end result. In the current work, we used expectation maximisation (EM) algorithm during HMM parameter training step. We trained initial state distribution, stationary transition matrix and mean of the Gaussian distributions. We selected one chromosome at a time during the training step to clearly and accurately capture the initial states and ratio variations in chromosomes. This approach helped to achieve faster computational time as well due to the reduced number of data points. Finally, we applied Viterbi algorithm [23] to predict the sequence of hidden states.

When the BAFs of tumour samples are present, we fitted HMM for different base ploidy values (reflecting different copy number 2, 3, and 4 states) and then applied the following steps to determine the base ploidy: (i) Select the SNPs which overlaps with the captured exonic regions, (ii) segment BAFs (b_i_ as given in Equation (1)) of SNPs using DNACopy [5] circular binary segmentation algorithm, (iii) estimate B allele count (N_B,i_) for different values of contamination () as given in Equation (2), (iv) calculate the cost of each estimation by taking the distance between estimated N_B,i_ and rounded value of N_B,i_ (as B allele counts cannot be fractions) and (v) calculate the minimum summation of distances that would give the best fit for the base ploidy.
1

In Equation (1), C_T_ represents the copy number of the tumour that is predicted by the HMM. From Equation (1) we can estimate N_B,i_:
2

This procedure identifies the absolute copy numbers when base ploidy of the tumour sample is not known a priori.

### HMM to identify zygosity states

#### Definition of hidden states

The definitions of the hidden states to predict tumour zygosity are shown in Table 4. ADTEx analyses positions, , with heterozygous SNPs in the matched normal sample. SNPs having BAF within 0.3 and 0.7 in normal sample were selected as heterozygous regions (thresholds were selected as in [25]). The removal of homozygous loci in the normal sample ensured the detection of tumour-specific somatic LOH events.

**Table 4:** **Definitions of hidden states in ADTEx zygosity detection HMM**

State	Copy	Copy number alteration status	Genotype	BAF	Zygosity
1	0/1	Deletion	A,B	0,1	LOH
2	2	Copy neutral with LOH	AA,BB	0,1	LOH
3	2	Normal	AB	0.5	HET
4	3	Three copies with LOH	AAA,BBB	0,1	LOH
5	3	Three copies with duplication of one allele	AAB, ABB	0.33,0.67	ASCNA
6	4	Four copies with LOH	AAAA,BBBB	0,1	LOH
7	4	Four copies with duplication of both alleles	AABB	0.5	HET
8	4	Four copies with duplication of one allele	AAAB,ABBB	0.25,0.75	ASCNA
9	5	Five copies with LOH	AAAAA,BBBBB	0,1	LOH
10	5	Five copies with duplication of one allele	AAAAB,ABBBB	0.2,0.8	ASCNA
11	5	Five copies with duplication of both alleles	AAABB,AABBB	0.4,0.6	ASCNA
12	6	Six copies with LOH	AAAAAA,BBBBBB	0,1	LOH
13	6	Six copies with duplication in one allele	AAAAAB,ABBBBB	0.17,0.83	ASCNA
14	6	Six copies with duplication in both alleles	AAABBB	0.5	HET
15	6	Six copies with duplication in both alleles	AAAABB,AABBBB	0.33,0.67	ASCNA

As depicted in Table 4, each hidden state can be uniquely identified with a copy number and zygosity state. The copy number of each SNP locus is calculated from the previous HMM predicting copy number.

#### Probability density function of observations

Mirrored BAFs (as defined below) around 0.5 are used as the observations in the proposed HMM.


Normal cell proportion () and standard deviation (_b_) of BAF signal are considered as main parameters of the observation distribution. We assumed mirrored BAF signal is normally distributed with state specific mean and a constant _b_ for all states. If BAF of l^th^ SNP is *b*_*l*_, then observation probability distribution function (pdf) for a hidden state S_k_ with associated copy number C_T_ can be formulated as:
3

Here, state specific mean () is given by:
4

where, N_B_(S_k_) is the expected B allele count for state S_k_.

#### Non-stationary transition matrix

The state transition matrix is considered to be non-stationary as described in [6, 32]. The genomic distance (d) between two SNPs is non-uniform and hence we calculated the transition probabilities based on exponential function utilizing *d.* Further, the transition probability depends on the state specific copy number. For example, if the current observation *j*, is assigned with a copy number of two, then the model can only transit to ‘hidden state 2’ and ‘hidden state 3’ (Table 4) from previous state *i*.


Where,
56

In equation (5), L is chosen to be 2 Mb.

#### Expectation maximisation (EM) algorithm

EM algorithm has been used in various parameter estimation tasks including HMM [23]. In the following text we describe the EM algorithm that is used to estimate HMM parameters. We pooled data points from all the chromosomes for the parameter training. The pooled training procedure helped to make sure that all hidden states would be covered during parameter selection. However, the joint estimation of parameters did not favour the determination of initial state distribution () as it can vary at the beginning of each chromosome. Therefore, we trained a separate initial state distribution for each chromosome.

We can derive the partial log likelihood function for observation distribution as:
7

In the above Equation (7), _l_ (S_k_) is the posterior probability of l^th^ SNP to be in state S_k_, which is calculated using forward-backward algorithm [23]. By taking the derivative with respect to  and setting it to zero, we derive the following equation regarding normal cell contamination estimation:
8

Where *P*_*T*_ = 2*α* + (1 − *α*)*C*_*T*_(*S*_*k*_) and *t* = EM iteration number. The algorithm is constrained to identify  in the interval of 0 ≤  ≤ 0.7, so if ^α(t+1)^ is less than 0 or greater than 0.7, it will be set to 0 or 0.7 respectively.

Estimated  as per Equation (8) is used to update state dependent means. Although, the presence of normal cell contamination, shrinks BAFs to 0.5, it does not affect BAFs in the ‘HET’ states of the HMM. Hence, mean of these ‘HET’ states are calculated as per the typical EM training step described in [22].

## Electronic supplementary material

Additional file 1: **Contains Supplementary Methods, Supplementary Figures S1 - S9 and Supplementary Tables S1 - S12.** (PDF 1 MB)

Additional file 2: **Copy number and genotype profiles of the in-house generated ovarian cancer samples predicted by ADTEx.** (PDF 1 MB)

Additional file 3: **Supplementary Table containing CNA predictions of the TCGA samples made by ADTEx.** (ZIP 5 MB)

## Abbreviations

ADTEx: Aberration detection in tumour exome
TCGA: The cancer ganome Atlas
SNP: Single nucleotide polymorphism
GPL: General public license
CNA: Copy number alteration
LOH: Loss of heterozygosity
aCGH: Array comparative hybridization
MPS: Massively parallel sequencing
WGS: Whole genome sequencing
WES: Whole exome sequencing
ICGS: International cancer genome consortium
DOC: Depth of coverage
BAF: B allele frequency
HMM: Hidden Markov model
XHMM: exome hidden Markov model
CoNIFER: Copy number inference from exome reads
DWT: Discrete wavelet transformation
EM: Expectation maximisation
BWA: Burrow-Wheeler aligner
ASCAT: Allele specific copy number analysis of tumours
TP: True positive
FP: False positive
TN: True negative
FN: False negative
ASCNA: Allele specific copy number alterations
CGHub: Cancer genomics hub
GATK: The genome analysis toolkit.
