# Supplementary material for: Inferring copy number and genotype in tumour exome data
Source: BMC Genomics. 2014 Aug 28;15(1):732. doi: 10.1186/1471-2164-15-732 (PMC4162913; doi:10.1186/1471-2164-15-732)
Supplement: Supplementary file 1 — Additional file 1: Contains Supplementary Methods, Supplementary Figures S1 - S9 and Supplementary Tables S1 - S12. (PDF 1 MB) [file 12864_2014_6426_MOESM1_ESM.pdf]

Inferring copy number and genotype in tumour exome data

Kaushalya C Amarasinghe[1], Jason Li [1,2], Sally M Hunter[3], Georgina L Ryland[3], Prue A Cowin[4], Ian G Campbell[3,5,6], Saman K Halgamuge[1]

[1]Optimisation and Pattern Recognition group, Mechanical Engineering Department, Melbourne School of Engineering, The University of Melbourne, Parkville, Victoria 3010, Australia

[2]Bioinformatics Core Facility, Peter MacCallum Cancer Centre, East Melbourne, Victoria 3002, Australia

[3]Cancer Genetics Laboratory, Peter MacCallum Cancer Centre, East Melbourne, Victoria 3002, Australia

[4]Cancer Genomics and Genetics Laboratory, Peter MacCallum Cancer Centre, East Melbourne, Victoria 3002, Australia

[5]Sir Peter MacCallum Department of Oncology, The University of Melbourne, Parkville, Victoria 3010, Australia

[6]Department of Pathology, The University of Melbourne, Parkville, Victoria 3010, Australia

*Corresponding author

Email addresses:

KCA: kca@student.unimelb.edu.au

JL: jason.li@petermac.org

SMH: sally.hunter@petermac.org

GLR: georgina.ryland@petermac.org

PAC: prue.cowin@gmail.com

IGC: ian.campbell@petermac.org

SKH: saman@unimelb.edu.au

Contents

1 Supplementary Methods 3
1.1 HMM to predict copy number alterations 3
1.1.1 Emission distribution 3
1.2 Parameters used in the comparison of each method 4
2 Supplementary Figures 5
Figure S1 5
Figure S2 6
Figure S3 6
Figure S4 7
Figure S5 8
Figure S6 8
Figure S7 9
Figure S8 9
Figure S9 10
3 Supplementary Tables 11
Table S1. 11
Table S2. 12
Table S3. 12
Table S4. 12
Table S5. 13
Table S6. 14
Table S7. 15
Table S8. 15
Table S9. 15
Table S10. 16
Table S11. 19
Table S12. 19
4 References 19

1 Supplementary Methods

1.1 HMM to predict copy number alterations

This step predicts the unknown copy number state sequence from the observed depth of coverage (DOC) ratios. The hidden states of the HMM represent the copy number states of each targeted region. In the default settings we have five different copy number states representing 0 to 4 copy numbers. These states are interpreted in biological context as homozygous deletion (copy 0), hemizygous deletion (copy 1), no CNV or copy neutral (copy 2), 1 copy gain (copy 3), and copy amplifications (copy 4 and above).

The observed DOC ratios (R_{ij}) are smoothed by applying DWT denoising before feeding them to the HMM. Here, R_{ij} represents the DOC ratio of the ith targeted region in j^{th} chromosome. Each chromosome j of each tumour-control samples pair is considered separately for copy number identification. The fitted discrete time HMM is given below:

1. The total number of hidden states in the model is given by K and those are denoted by S = $S_1, S_2, ..., S_K$. If there are L exons in the sample of consideration, the state of lth exon (e_l) equals to S_k where $1 \leq l \leq L$ and $1 \leq k \leq K$.
2. The initial state distribution $\pi = \{\pi_k\}$ where

   $$\pi_k = P(e_1 = S_k), \qquad 1 \leq k \leq K$$

3. The state transition probability distribution $A = a_{mp}$ where

   $$a_{mp} = P(e_{l+1} = S_p \mid e_l = S_m), \qquad 1 \leq m, p \leq K$$

4. The emission probability distribution is given by $B = \{b_k(\mathbf{O})\}$ where

   $$\{b_k(\mathbf{O})\} = \mathcal{N}(\mathbf{O}_l, \mu_k, \sigma^2), \qquad 1 \leq l \leq L \text{ and } 1 \leq k \leq K$$

   Here, $\mathcal{N}$ represents the Gaussian distribution. Mean (μ_k) of that distribution vary with different states and the normal cell contamination percentage and ploidy. We used a common standard deviation, σ, for all states.

Above HMM can be represented compactly as $\lambda = (A, B, \pi)$ where A, B and π represent transition probability matrix, emission probability distribution and initial state distribution.

1.1.1 Emission distribution

A 100% pure diploid tumour sample would have DOC ratios (0,0.5,1,1.5) representing copy numbers (0,1,2,3) correspondingly. However, this is not essentially the case when there is normal contamination, aneuploidy and polyploidy present in the data. A polyploid sample with normal

contamination will see a deviation in the relationship between copy number and DOC ratio. This relationship is given by,

$$R_{ij} = \frac{2\alpha + (1-\alpha)P_{Tij}}{P_T} \quad \text{-------------------->} \ (1)$$

Where α, P_{Tij} and P_T represent normal contamination, copy number of considered tumour region and ploidy of tumour sample respectively.

Therefore, we modelled the mean of emission distribution by equation 1, where P_{Tij} depends on the copy number of each hidden state. P_T either can be given as an input or a best fit can be computed if B allele frequencies (BAF) are present. This computation is described in the main article.

1.2 Parameters used in the comparison of each method

ExomeCNV (Sathirapongsasuti, et al., 2011): We observed that this method performs better with their segment merging step, which utilizes circular binary segmentation algorithm in DNACopy package (Olshen, et al., 2004). Hence, we used the CNV segments output after the merging step and converted this to exon level copy number. We then compared its performance against copy number predictions by ASCAT on SNP 6 array data. We applied ExomeCNV version 1.2 with its default parameters, except for the read length and normal cell contamination. We applied sample specific normal cell contamination values as input to ExomeCNV based on manual inspection of the data and predictions made by ASCAT.

VarScan 2 (Koboldt, et al., 2012): This method (VarScan 2 version 3.1) segments the exome based on the differences between the read depths of tumour and normal samples. We followed the recommended workflow provided in the VarScan project page in SourceForge website (http://varscan.sourceforge.net/copy-number-calling.html): 1) Run VarScan 'copynumber' routine on pileup files generated by SAMtools (Li, et al., 2009), 2) Run VarScan 'copyCaller' on the results from step (1), 3) Apply circular binary segmentation (CBS) from DNAcopy package(Olshen, et al., 2004), 4) Visualise the results and adjust baseline if necessary. If baseline is adjusted, then apply steps (3) and (4) again and 5) Merge segments and classify events using 'mergeSegments.pl' script. The result contained segmented exome based on the predicted CNV status of each genomic locus. Since we calculated the performance based on exon level predictions, we converted the output to exon level predictions by referring to the overlap of each exon with the predicted CNV segments. If an exon has two different CNV status predicted by consequent CNV segments, then the CNV status which overlaps with the most number of bases of the exon is taken as the correct call.

Control-FREEC (Boeva, et al., 2012): All the default parameters specified by the authors of the method (version 6.2) in their example on exome sequencing data analysis were used in our comparison study. The samples were tested with the 'contaminationAdjustment = TRUE' and 'ploidy = 2' options.

2 Supplementary Figures

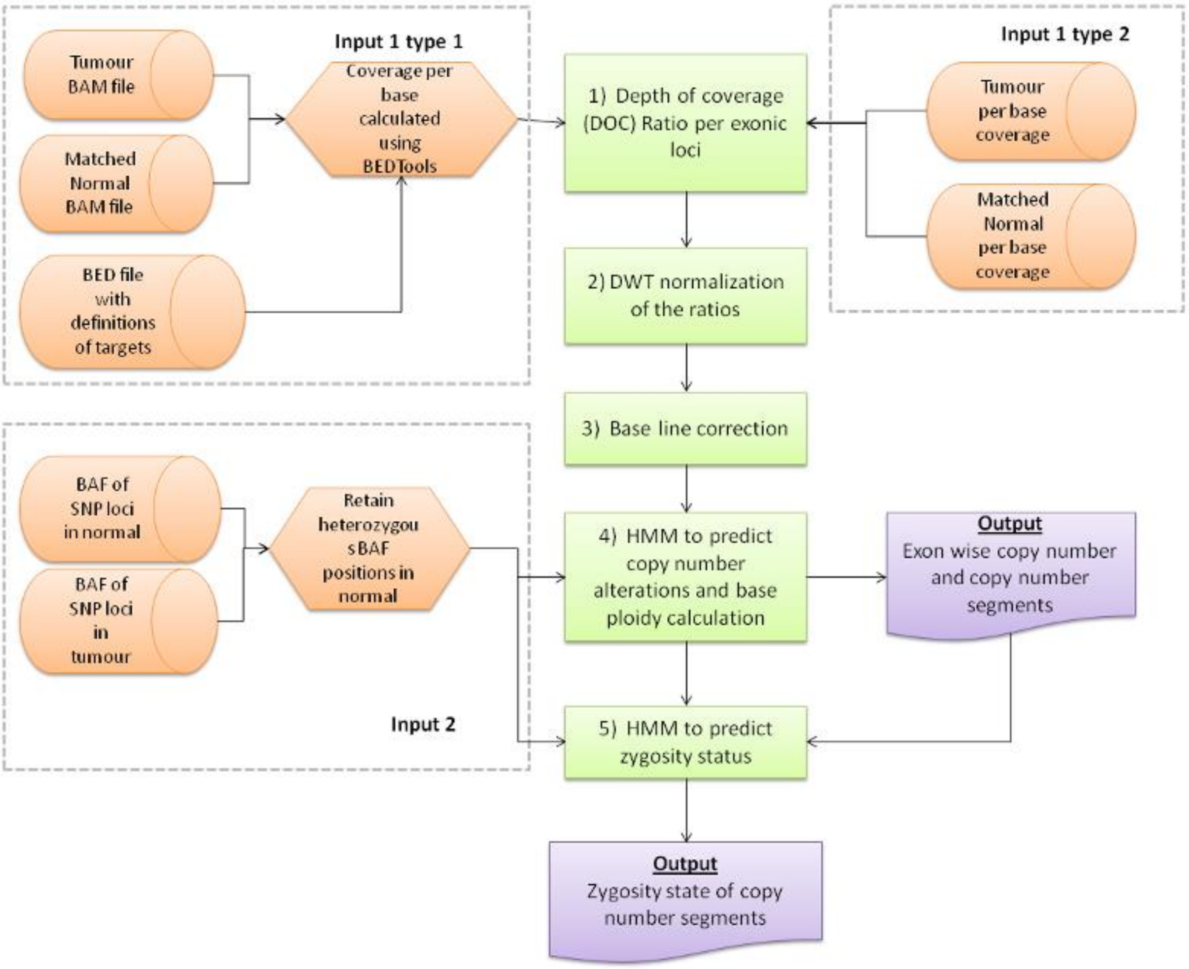

Figure S1. Overall workflow of the proposed algorithm ADTEx. More details with example commands can be found in the user manual distributed with the free software package. The method accepts two inputs. 1) BAM files of tumour and matched normal samples with targeted regions definition in BED format or per base coverage files for tumour and matched normal samples generated by BED Tools. 2) B allele frequencies of tumour and normal samples at tumour SNP loci.

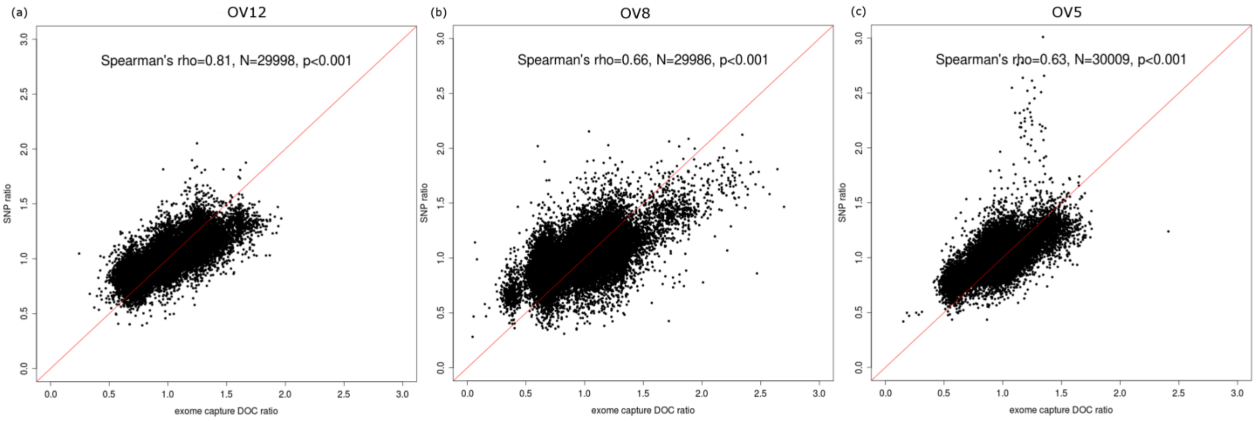

Figure S2. Concordance of ratio data between SNP6 ratios (vertical axis) and WES ratios (horizontal axis) in (a) OV12, (b) OV8 and (c) OV5 tumour samples. For each sample we observed statistically significant positive correlation. (OV12: 0.81, OV8: 0.66 and OV5: 0.63 with P value < 0.001).

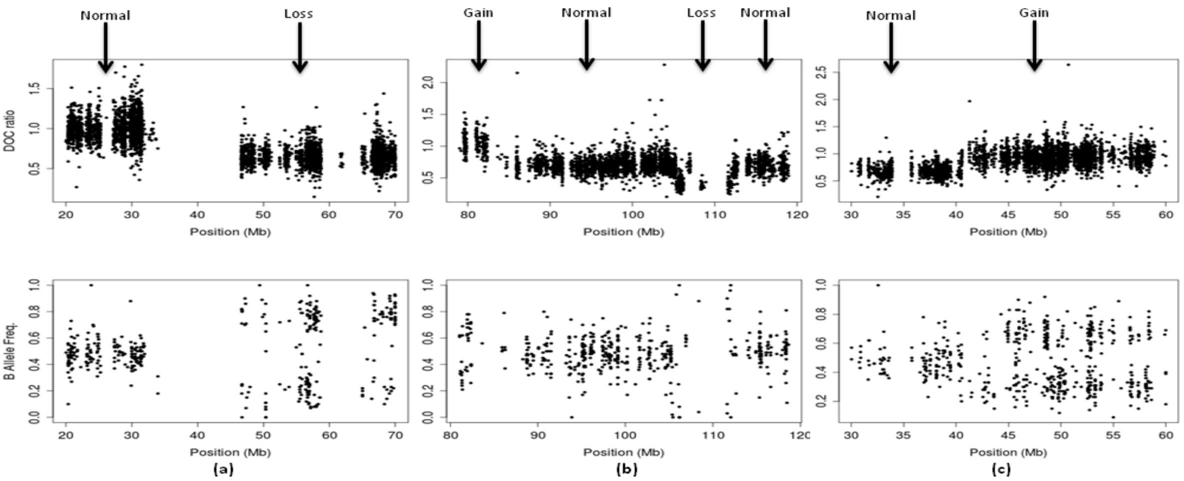

Figure S3. Estimating copy status of the ratios around 1. (a) OV4 (chr16) (b) OV8 (chr10) and (c) OV12 (chr3). OV4, OV8 and OV12 have copy 2, copy 3 and copy 4 states around ratio 1. These can be identified using the variation in BAFs as shown in the bottom panel.

(a)

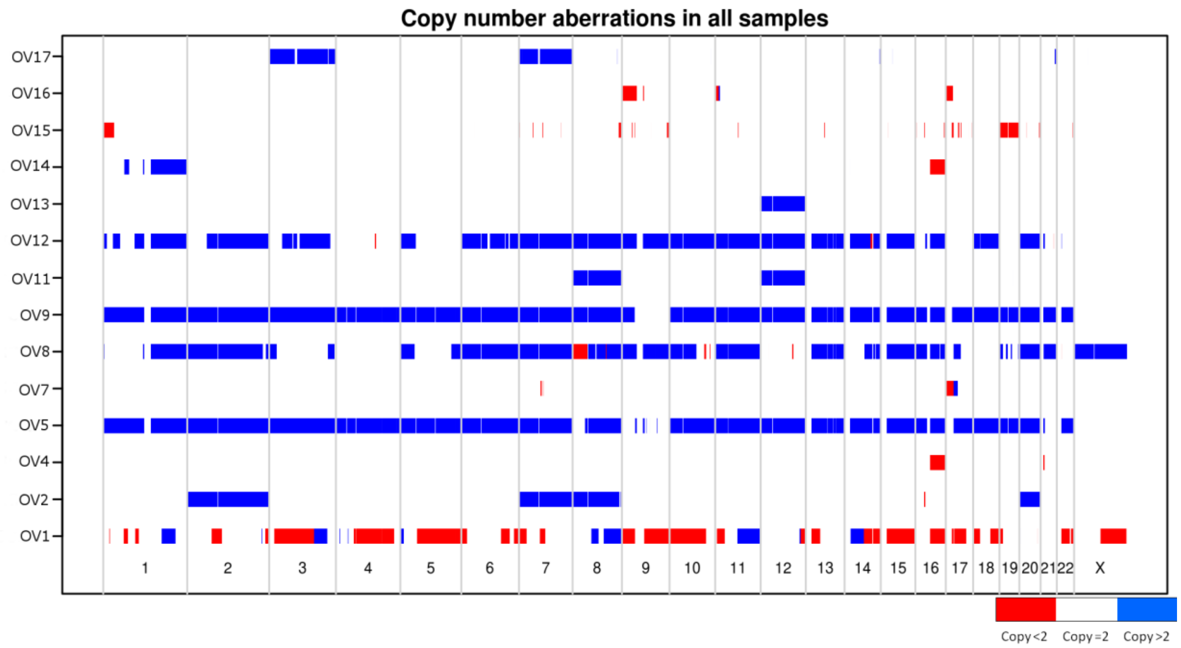

(b)

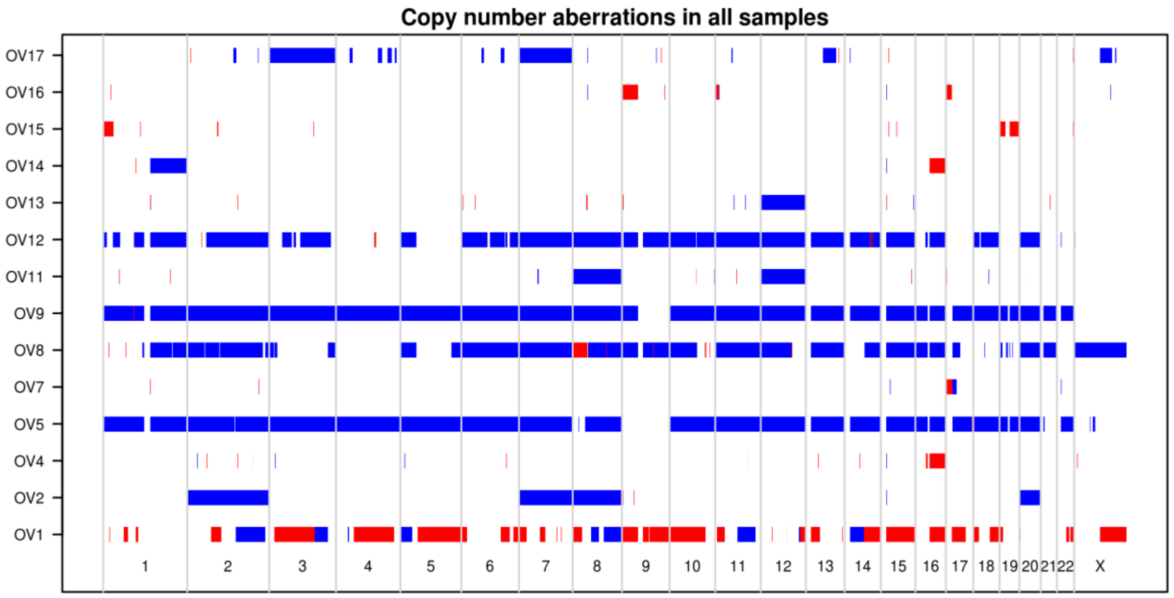

Figure S4. Copy number alterations predicted by (a) ADTEx and (b) ASCAT for all tumour samples with copy number alterations.

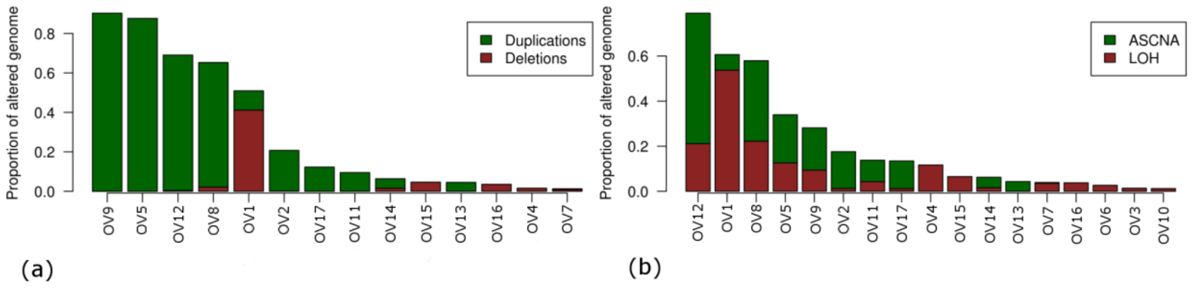

Figure S5. Distribution of the proportion of genome altered. Proportion of the genome altered by (a) copy number aberrations and (b) LOH as per the predictions made by ADTEx for each tumour sample.

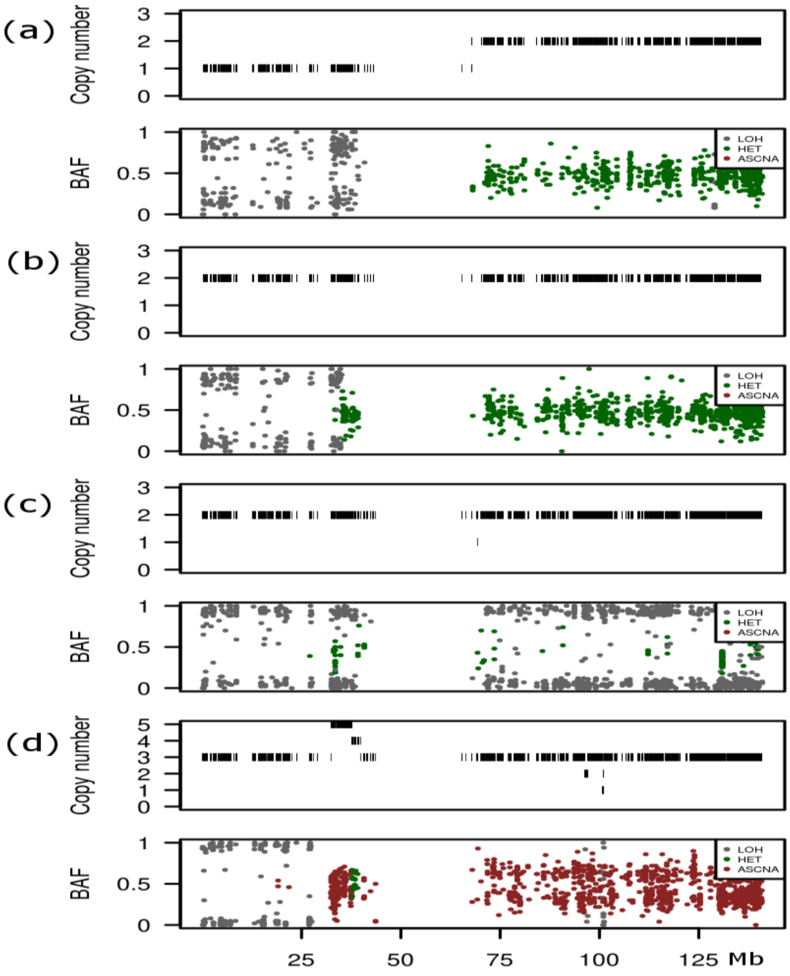

Figure S6. Examples of LOH event detections in the ovarian cancer samples by ADTEx. (a) Copy deletion LOH in OV16, (b) copy neutral LOH in OV17, (c) full chromosomal copy neutral LOH in OV11 and (d) copy amplified LOH in OV8.

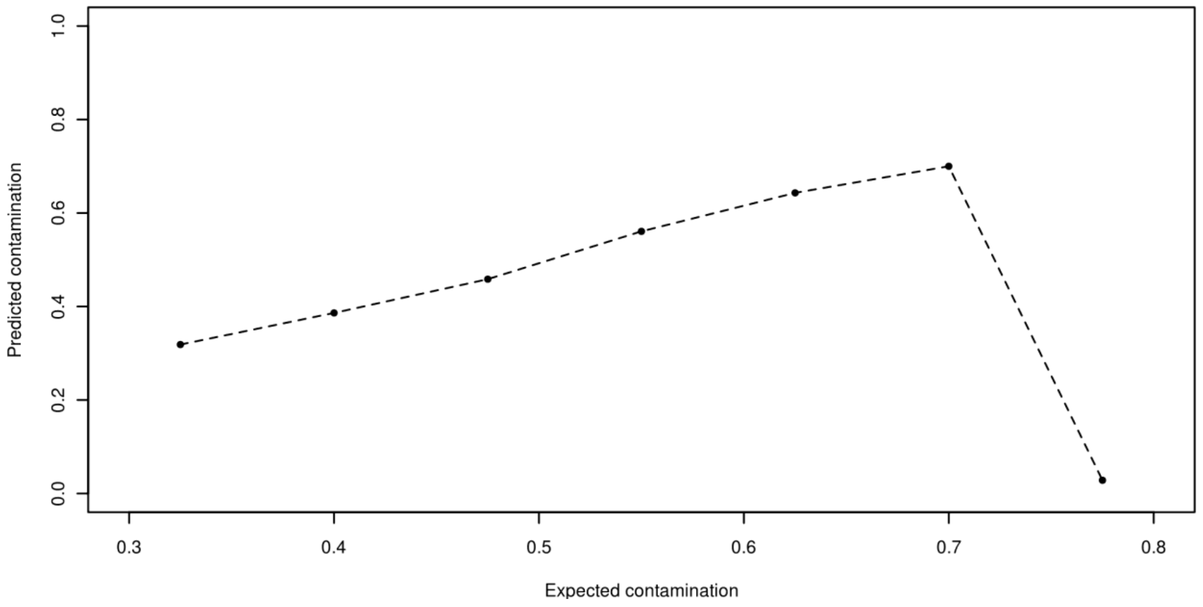

Figure S7. Estimated vs actual contamination levels of the simulation study at original coverage (150X).

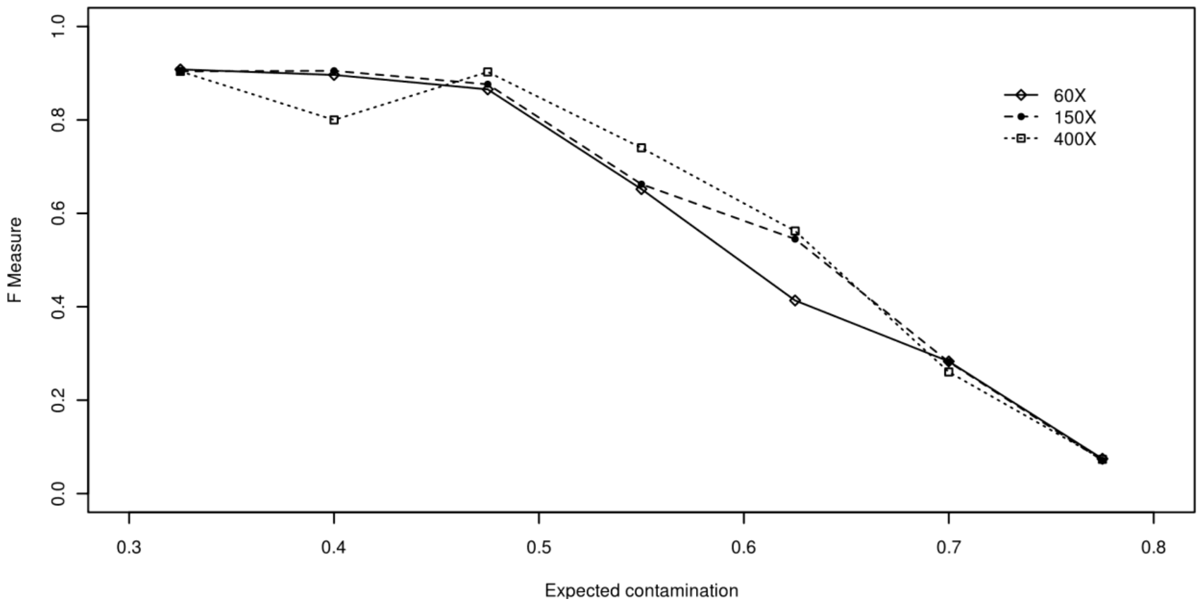

Figure S8. Performance of ADTEx of detecting copy number alterations in terms of F measure at each contamination level.

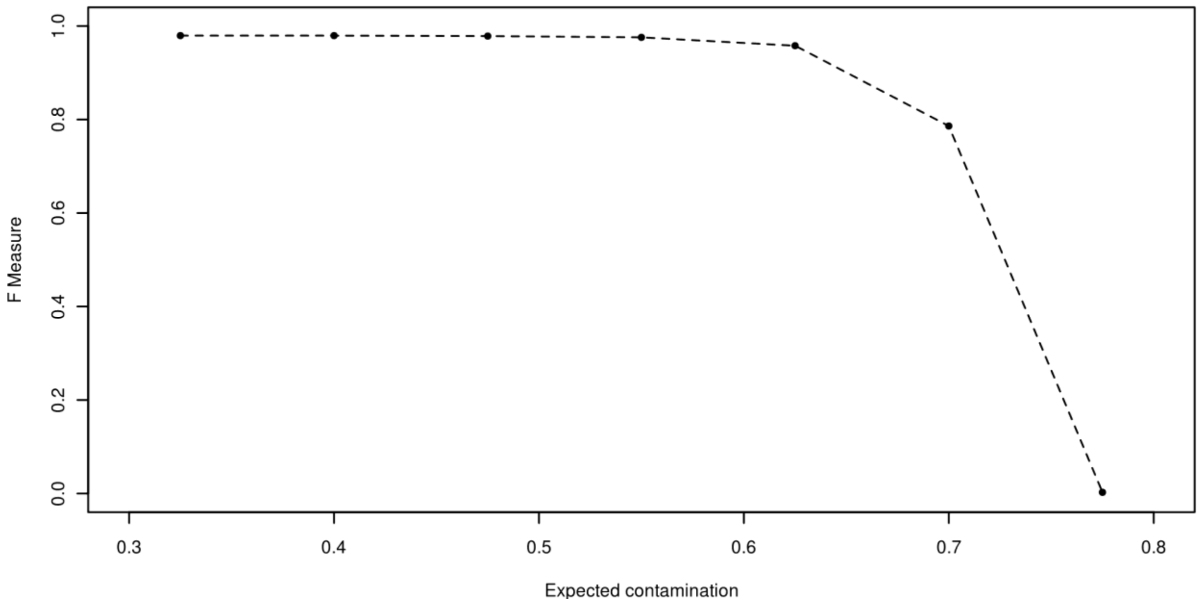

Figure S9. Performance of ADTEx of detecting LOH at 150X coverage.

3 Supplementary Tables

Table S1. Sample wise coverage statistics for the 17 ovarian tumours

Sample Name	Tumour / Normal	Exome Platform	On target reads	Read length(bp)	Total on-target Sequenced length (Mb)	Avg. coverage per targeted base
OV1	Tumour	Agilent SureSelect Human All Exon Version 4	95,108,011	101	9,606	188
OV1	Normal		96,752,923	101	9,772	191
OV2	Tumour	Roche NimbleGen EZ Exome SeqCap Version 2	130,959,793	100	13,096	364
OV2	Normal		81,102,091	100	8,110	225
OV3	Tumour	Roche NimbleGen EZ Exome SeqCap Version 2	63,118,103	100	6,312	175
OV3	Normal		71,684,515	100	7,168	199
OV4	Tumour	Roche NimbleGen EZ Exome SeqCap Version 2	61,769,014	100	6,177	172
OV4	Normal		89,363,191	100	8,936	248
OV5	Tumour	Roche NimbleGen EZ Exome SeqCap Version 2	87,472,518	100	8,747	243
OV5	Normal		83,619,445	100	8,362	232
OV6	Tumour	Roche NimbleGen EZ Exome SeqCap Version 2	83,945,445	100	8,395	233
OV6	Normal		83,177,569	100	8,318	231
OV7	Tumour	Roche NimbleGen EZ Exome SeqCap Version 2	73,602,087	100	7,360	205
OV7	Normal		103,046,749	100	10,305	286
OV8	Tumour	Roche NimbleGen EZ Exome SeqCap Version 2	76,919,416	100	7,692	214
OV8	Normal		73,266,587	100	7,327	204
OV9	Tumour	Roche NimbleGen EZ Exome SeqCap Version 2	54,170,665	100	5,417	151
OV9	Normal		75,610,361	100	7,561	210
OV10	Tumour	Roche NimbleGen EZ Exome SeqCap Version 2	66,934,752	100	6,693	186
OV10	Normal		81,516,449	100	8,152	227
OV11	Tumour	Roche NimbleGen EZ Exome SeqCap Version 2	65,833,549	100	6,583	183
OV11	Normal		63,483,035	100	6,348	176
OV12	Tumour	Roche NimbleGen EZ Exome SeqCap Version 2	68,826,106	100	6,883	191
OV12	Normal		77,436,221	100	7,744	215
OV13	Tumour	Roche NimbleGen EZ Exome SeqCap Version 2	97,608,354	100	9,761	271
OV13	Normal		104,017,548	100	10,402	289
OV14	Tumour	Roche NimbleGen EZ Exome SeqCap Version 2	70,466,147	101	7,117	198
OV14	Normal		85,090,300	101	8,594	239
OV15	Tumour	Roche NimbleGen EZ Exome SeqCap Version 2	78,912,754	101	7,970	222
OV15	Normal		92,089,982	101	9,301	259
OV16	Tumour	Roche NimbleGen EZ Exome SeqCap Version 1	37,876,598	79	2,992	114
OV16	Normal		36,527,280	100	3,653	139
OV17	Tumour	Roche NimbleGen EZ Exome SeqCap Version 1	52,558,469	79	4,152	158
OV17	Normal		52,433,723	79	4,142	158

Table S2. Performance summary for each sample compared with ASCAT (Van Loo, et al., 2010) copy number alteration results on SNP 6.0 data

Sample Name	Sensitivity	Specificity	Precision	Accuracy
OV1	89.1%	92.1%	91.9%	90.6%
OV2	97.3%	99.5%	97.7%	99.1%
OV4	82.4%	99.98%	98.96%	99.6%
OV5	99.8%	98.3%	99.8%	99.6%
OV7	98.8%	99.4%	82.6%	99.4%
OV8	92.4%	94.2%	96.9%	93.0%
OV9	99.9%	99.8%	99.99%	99.9%
OV11	97.5%	99.99%	99.7%	99.7%
OV12	99.6%	99.4%	99.7%	99.5%
OV16	90.8%	99.98%	99.5%	99.6%
OV13	89.1%	99.99%	99.92%	99.3%
OV17	84.1%	99.5%	96.0%	97.6%
OV14	98.6%	99.4%	91.6%	99.3%
OV15	97.7%	94.7%	60.9%	94.9%

Table S3. Performance summary for each sample compared with ASCAT (Van Loo, et al., 2010) LOH prediction results on SNP 6.0 data

Sample Name	Sensitivity	Specificity	Precision	Accuracy
OV1	97.8%	98.0%	98.0%	97.9%
OV2	79.1%	99.5%	59.6%	99.3%
OV3	68.7%	99.9%	94.8%	98.9%
OV4	93.7%	99.8%	98.3%	99.0%
OV5	93.5%	99.96%	99.7%	99.1%
OV6	89.8%	99.97%	98.4%	99.7%
OV7	92.7%	99.6%	93.8%	99.2%
OV8	91.4%	98.7%	95.5%	96.9%
OV9	91.7%	99.9%	99.4%	99.2%
OV10	22.7%	99.7%	68.9%	97.2%
OV11	89.5%	99.8%	96.0%	99.4%
OV12	96.2%	99.8%	99.0%	99.0%
OV16	90.1%	99.6%	92.5%	99.0%
OV13	1.5%	99.7%	6.1%	98.5%
OV17	82.6%	99.7%	74.0%	99.5%
OV14	79.6%	99.9%	92.2%	99.4%
OV15	95.99%	91.3%	52.4%	91.7%

Table S4. Performance summary for each sample compared with ASCAT (Van Loo, et al., 2010) ASCNA prediction results on SNP 6.0 data

Sample Name	Sensitivity	Specificity	Precision	Accuracy
OV1	51.4%	97.0%	58.5%	93.5%
OV2	96.0%	98.2%	89.5%	97.9%
OV3	NA			

OV4	NA			
OV5	96.8%	97.5%	89.4%	97.3%
OV6	NA			
OV7	97.0%	99.9%	79.9%	99.9%
OV8	70.0%	94.2%	91.0%	83.2%
OV9	85.1%	98.1%	92.8%	95.2%
OV10	NA			
OV11	99.5%	99.99%	99.96%	99.95%
OV12	98.6%	99.0%	99.2%	98.8%
OV16	NA			
OV13	97.7%	99.99%	99.95%	99.9%
OV17	89.2%	98.2%	84.9%	97.3%
OV14	100.0%	99.7%	95.1%	99.7%
OV15	NA			

Table S5. Summary of TCGA samples included in this study (2011)

Tumor Sample	Total Reads on Targets	Total Gb	Normal Sample	Total Reads on Targets	Total Gb
TCGA-04-1336-01A-01W-0488-09	65615059	6.11	TCGA-04-1336-11A-01W-0489-09	67197808	6.26
TCGA-04-1343-01A-01W-0488-09	107840152	10.04	TCGA-04-1343-10A-01W-0489-09	98148440	9.14
TCGA-04-1347-01A-01W-0488-09	76407526	7.12	TCGA-04-1347-11A-01W-0489-09	58341013	5.43
TCGA-04-1348-01A-01W-0494-09	74926564	6.98	TCGA-04-1348-11A-01W-0494-09	72110127	6.72
TCGA-04-1349-01A-01W-0494-09	118126167	11.00	TCGA-04-1349-11A-01W-0494-09	107016932	9.97
TCGA-04-1361-01A-01W-0494-09	58789066	5.48	TCGA-04-1361-11A-01W-0494-09	58940962	5.49
TCGA-04-1362-01A-01W-0494-09	57215953	5.33	TCGA-04-1362-10A-01W-0494-09	62141132	5.79
TCGA-04-1542-01A-01W-0553-09	83570502	7.78	TCGA-04-1542-10A-01W-0553-09	60831262	5.67
TCGA-09-0365-01A-02W-0372-09	98814914	9.20	TCGA-09-0365-10A-01W-0372-09	92979525	8.66
TCGA-09-0366-01A-01W-0372-09	82369580	7.67	TCGA-09-0366-10B-01W-0372-09	91671450	8.54
TCGA-09-0369-01A-01W-0372-09	92566190	8.62	TCGA-09-0369-10C-01W-0372-09	90091112	8.39
TCGA-09-1664-01A-01W-0639-09	76194803	7.10	TCGA-09-1664-11A-01W-0639-09	102202707	9.52
TCGA-09-1672-01A-01W-0633-09	73395933	6.84	TCGA-09-1672-10A-01W-0633-09	65659694	6.12
TCGA-10-0930-01A-02W-0421-09	80187835	7.47	TCGA-10-0930-11A-01W-0977-09	88957759	8.28
TCGA-10-0933-01A-01W-0421-09	88627046	8.25	TCGA-10-0933-11A-01W-0421-09	88125067	8.21
TCGA-10-0935-01A-03W-0421-09	89582634	8.34	TCGA-10-0935-11A-01W-0421-09	92004187	8.57
TCGA-13-0723-01A-02W-0372-09	96888212	9.02	TCGA-13-0723-10B-01W-0372-09	88874383	8.28
TCGA-13-0724-01A-01W-0372-09	89506994	8.34	TCGA-13-0724-10B-01W-0372-09	85414091	7.95

Table S6. Sample wise performance summary for comparison of other methods

ExomeCNV (Sathirapongsasuti, et al., 2011):

Sample Name	Sensitivity	Specificity	Precision	Accuracy
OV1	90.9%	80.4%	82.2%	85.6%
OV2	98.99%	99.3%	96.9%	99.2%
OV4	82.4%	93.7%	22.2%	93.5%
OV7	98.9%	98.6%	65.3%	98.6%
OV11	97.4%	98.6%	87.2%	98.5%
OV16	89.1%	92.5%	34.1%	92.3%
OV13	89.0%	93.6%	47.5%	93.3%
OV17	79.9%	98.5%	88.2%	96.2%
OV14	98.7%	99.2%	89.4%	99.2%
OV15	97.7%	96.4%	69.4%	96.5%

VarScan2 (Koboldt, et al., 2012):

Sample Name	Sensitivity	Specificity	Precision	Accuracy
OV1	89.10%	90.35%	90.25%	89.72%
OV2	97.88%	91.55%	72.81%	92.74%
OV4	82.45%	99.38%	74.84%	99.00%
OV7	98.34%	97.69%	53.39%	97.71%
OV11	95.69%	98.36%	85.63%	98.11%
OV16	90.65%	99.47%	88.59%	99.10%
OV13	89.03%	99.87%	97.85%	99.19%
OV17	81.04%	98.94%	91.29%	96.77%
OV14	97.70%	93.72%	51.83%	93.98%
OV15	96.97%	93.72%	57.04%	93.98%

Control-FREEC (Boeva, et al., 2012):

Sample Name	Sensitivity	Specificity	Precision	Accuracy
OV1	20.1%	6.1%	17.7%	13.1%
OV2	95.6%	99.9%	99.9%	99.2%
OV4	82.4%	99.9%	95.6%	99.5%
OV7	98.0%	99.6%	86.1%	99.5%
OV11	97.0%	99.9%	99.2%	99.7%
OV16	1.0%	99.8%	11.9%	95.5%
OV13	88.8%	99.9%	98.1%	99.2%
OV17	82.2%	99.2%	93.5%	97.2%
OV14	99.3%	99.8%	97.0%	99.7%
OV15	90.8%	99.0%	88.3%	98.3%

Table S7. Sample wise performance summary for LOH prediction for ADTEx and Control-FREEC (Boeva, et al., 2012)

	Sensitivity		Specificity		Precision		Accuracy	
	ADTEx	Control-FREEC	ADTEx	Control-FREEC	ADTEx	Control-FREEC	ADTEx	Control-FREEC
OV1	97.8%	95.3%	98.0%	93.5%	98.0%	94.1%	97.9%	94.4%
OV2	79.1%	93.1%	99.5%	40.8%	59.6%	2.5%	99.3%	41.6%
OV4	93.7%	100%	99.8%	0.05%	98.3%	45.6%	99.0%	45.6%
OV7	92.6%	94.9%	99.6%	2.1%	93.8%	26.2%	99.2%	27.0%
OV11	89.5%	100%	99.8%	28.0%	96.0%	16.5%	99.4%	36.9%

Table S8. Sample wise performance summary for ASCNA prediction for ADTEx and Control-FREEC (Boeva, et al., 2012)

	Sensitivity		Specificity		Precision		Accuracy	
	ADTEx	Control-FREEC	ADTEx	Control-FREEC	ADTEx	Control-FREEC	ADTEx	Control-FREEC
OV1	51.4%	60.9%	97.0%	86.3%	58.5%	29.2%	93.5%	84.1%
OV2	96.0%	81.8%	98.2%	98.6%	89.5%	97.1%	97.9%	92.5%
OV7	97.0%	38.7%	99.9%	98.4%	79.9%	46.0%	99.9%	96.3%
OV11	99.5%	88.8%	100%	100%	100%	99.9%	100%	96.9%

Table S9. Sample wise performance summary for CNV predictions made by (Amarasinghe, et al., 2013)

Sample Name	Sensitivity	Specificity	Precision	Accuracy
OV1	87.4%	61.0%	69.3%	74.2%
OV2	74.5%	80.5%	46.9%	79.4%
OV4	82.4%	99.8%	91.2%	99.4%
OV7	70.2%	99.4%	76.5%	98.7%
OV8	91.3%	77.4%	88.7%	86.6%
OV11	97.6%	99.9%	98.7%	99.7%
OV16	90.5%	99.9%	96.5%	99.5%
OV13	89.1%	99.8%	97.2%	99.1%

OV17	84.1%	99.3%	94.5%	97.5%
OV14	97.0%	98.1%	78.3%	98.1%
OV15	96.3%	99.8%	97.0%	99.5%

Table S10. Arm level copy number aberrations.

Sample	Chr	Arm	CN	Status	Detected/Not Detected
IC050	2	2p	3	Whole chromosomal amplification	Detected
IC050	2	2q	3	Whole chromosomal amplification	Detected
IC050	7	7p	3	Whole chromosomal amplification	Detected
IC050	7	7q	3	Whole chromosomal amplification	Detected
IC050	8	8p	3	Whole chromosomal amplification	Detected
IC050	8	8q	3	Whole chromosomal amplification	Detected
IC050	20	20p	3	Whole chromosomal amplification	Detected
IC050	20	20q	3	Whole chromosomal amplification	Detected
IC121	16	16q	1	q arm deletion	Detected
IC138	6	6p	3	Whole chromosomal deletion	Not Detected
IC138	6	6q	3	Whole chromosomal deletion	Not Detected
IC138	8	8p	3	p arm deletion	Detected
IC138	8	8q	5	q arm amplification	Detected
IC138	9	9p	2	Whole chromosomal deletion	Detected
IC138	9	9q	2	Whole chromosomal deletion	Detected
IC138	11	11p	3	Whole chromosomal deletion	Detected
IC138	11	11q	3	Whole chromosomal deletion	Detected
IC138	15	15q	5	q arm amplification	Detected
IC138	17	17p	3	p arm deletion	Detected
IC138	21	21q	3	q arm deletion	Detected
IC138	X	Xp	3	Whole chromosomal deletion	Detected
IC138	X	Xq	3	Whole chromosomal deletion	Detected
IC257	17	17p	1	p arm deletion	Detected
IC321	1	1p	2	p arm deletion	Detected
IC321	3	3p	2	Whole chromosomal deletion	Detected
IC321	3	3q	2	Whole chromosomal deletion	Detected
IC321	4	4p	2	Whole chromosomal deletion	Detected
IC321	4	4q	2	Whole chromosomal deletion	Detected
IC321	5	5q	2	q arm deletion	Detected
IC321	6	6p	4	Whole chromosomal amplification with differential arm CN(not in SNP)	Detected
IC321	6	6q	5	Whole chromosomal amplification with differential arm CN(not in SNP)	Detected
IC321	7	7p	4	Whole chromosomal amplification	Detected
IC321	7	7q	4	Whole chromosomal amplification	Detected
IC321	8	8p	1	p arm deletion	Detected
IC321	10	10p	4	p arm amplification	Not Detected

IC321	12	12p	2	Whole chromosomal deletion(not in SNP)	Detected
IC321	12	12q	2	Whole chromosomal deletion(not in SNP)	Detected
IC321	13	13q	5	q arm amplification	Detected
IC321	15	15q	4	q arm amplification	Detected
IC321	17	17p	2	p arm deletion	Detected
IC321	18	18p	2	Whole chromosomal deletion	Detected
IC321	18	18q	2	Whole chromosomal deletion	Detected
IC321	19	19q	2	q arm deletion	Detected
IC321	20	20q	4	q arm amplification	Detected
IC321	21	21q	4	q arm amplification	Detected
IC321	22	22q	2	q arm deletion	Detected
IC321	X	Xp	4	Whole chromosomal amplification	Detected
IC321	X	Xq	4	Whole chromosomal amplification	Detected
IC343	5	5p	3	Whole chromosomal deletion	Not Detected
IC343	5	5q	3	Whole chromosomal deletion	Not Detected
IC343	9	9p	3	Whole chromosomal deletion with differential arm CN	Detected
IC343	9	9q	2	Whole chromosomal deletion with differential arm CN	Detected
IC343	15	15q	3	q arm deletion	Detected
IC343	17	17p	2	p arm deletion	Detected
IC343	X	Xp	2	Whole chromosomal deletion	Detected
IC343	X	Xq	2	Whole chromosomal deletion	Detected
IC400	8	8p	3	Whole chromosomal amplification	Detected
IC400	8	8q	3	Whole chromosomal amplification	Detected
IC400	12	12p	3	Whole chromosomal amplification	Detected
IC400	12	12q	3	Whole chromosomal amplification	Detected
IC403	2	2q	4	q arm amplification	Detected
IC403	4	4p	2	Whole chromosomal deletion	Detected
IC403	4	4q	2	Whole chromosomal deletion	Detected
IC403	5	5p	5	p arm amplification	Detected
IC403	5	5q	2	q arm deletion	Detected
IC403	7	7p	4	Whole chromosomal amplification	Detected
IC403	7	7q	4	Whole chromosomal amplification	Detected
IC403	8	8p	4	Whole chromosomal amplification	Detected
IC403	8	8q	4	Whole chromosomal amplification	Detected
IC403	10	10p	4	p arm amplification	Detected
IC403	11	11p	4	Whole chromosomal amplification	Detected
IC403	11	11q	4	Whole chromosomal amplification	Detected
IC403	13	13q	5	q arm amplification	Detected
IC403	16	16p	2	p arm deletion	Detected
IC403	17	17p	2	Whole chromosomal deletion	Detected
IC403	17	17q	2	Whole chromosomal deletion	Detected
IC403	19	19p	2	Whole chromosomal deletion	Detected
IC403	19	19q	2	Whole chromosomal deletion	Detected
IC403	20	20p	4	Whole chromosomal amplification	Detected

IC403	20	20q	4	Whole chromosomal amplification	Detected
IC403	21	21q	2	q arm deletion	Detected
IC403	22	22q	2	q arm deletion	Detected
IC403	X	Xp	2	Whole chromosomal deletion	Detected
IC403	X	Xq	2	Whole chromosomal deletion	Detected
S276	9	9p	1	p arm deletion	Detected
S276	17	17p	1	p arm deletion	Detected
S508	12	12p	3	Whole chromosomal amplification	Detected
S508	12	12q	3	Whole chromosomal amplification	Detected
S531	3	3p	3	Whole chromosomal amplification	Detected
S531	3	3q	3	Whole chromosomal amplification	Detected
S531	7	7p	3	Whole chromosomal amplification	Detected
S531	7	7q	3	Whole chromosomal amplification	Detected
S60136	1	1p	3	p arm amplification	Detected
S60136	16	16q	1	q arm deletion	Detected
S60267	19	19p	1	Whole chromosomal deletion	Detected
S60267	19	19q	1	Whole chromosomal deletion	Detected
AOCS34	3	3p	1	p arm deletion	Detected
AOCS34	4	4q	1	q arm deletion	Detected
AOCS34	5	5q	1	q arm deletion	Detected
AOCS34	8	8q	4	q arm amplification	Detected
AOCS34	9	9p	1	Whole chromosomal deletion	Detected
AOCS34	9	9q	1	Whole chromosomal deletion	Detected
AOCS34	10	10p	1	Whole chromosomal deletion	Detected
AOCS34	10	10q	1	Whole chromosomal deletion	Detected
AOCS34	11	11q	4	q arm amplification	Detected
AOCS34	15	15q	1	q arm deletion	Detected
AOCS34	16	16q	1	q arm deletion	Detected
AOCS34	18	18p	1	p arm deletion	Detected
AOCS34	22	22q	1	q arm deletion	Detected

Table S11. Sensitivity of larger CNAs detected by ADTEx.

Sample	Events detected	Events not detected	Sensitivity
OV1	29	1	96.7%
OV2	4	0	100%
OV4	1	0	100%
OV5	15	0	100%
OV7	2	0	100%
OV8	39	2	95.1%
OV9	11	0	100%
OV11	2	0	100%
OV12	27	0	100%
OV13	1	0	100%
OV14	3	0	100%
OV15	3	0	100%
OV16	2	0	100%
OV17	6	2	75.0%

Table S12. Comparison of smaller CNAs identified by ADTEx with other WES methods.

Sample	ADTEx	ADTEx Vs VarScan 2	ADTEx Vs ExomeCNV	ADTEx Vs FREEC
OV1	10	9	9	5
OV2	17	6	5	2
OV4	3	3	2	1
OV7	46	18	21	3
OV11	9	6	2	1
OV13	1	1	1	0
OV14	21	2	2	1
OV15	211	116	91	15
OV16	4	2	0	0
OV17	32	1	1	2

4 References

(2011) Integrated genomic analyses of ovarian carcinoma, *Nature*, **474**, 609-615.

Amarasinghe, K., Li, J. and Halgamuge, S. (2013) CoNVEX: copy number variation estimation in exome sequencing data using HMM, *BMC Bioinformatics*, **14**, S2.

Boeva, V., *et al.* (2012) Control-FREEC: a tool for assessing copy number and allelic content using next-generation sequencing data, *Bioinformatics*, **28**, 423-425.

Koboldt, D.C., *et al.* (2012) VarScan 2: Somatic mutation and copy number alteration discovery in cancer by exome sequencing, *Genome Research*, **22**, 568-576.

Li, H., *et al.* (2009) The Sequence Alignment/Map format and SAMtools, *Bioinformatics*, **25**, 2078-2079.

Olshen, A.B., *et al.* (2004) Circular binary segmentation for the analysis of array-based DNA copy number data, *Biostatistics*, **5**, 557-572.

Sathirapongsasuti, J.F.*, et al.* (2011) Exome Sequencing-Based Copy-Number Variation and Loss of Heterozygosity Detection: ExomeCNV, *Bioinformatics*.
Van Loo, P.*, et al.* (2010) Allele-specific copy number analysis of tumors, *Proceedings of the National Academy of Sciences*, **107**, 16910-16915.
