# Supplementary material for: Inferring copy number and genotype in tumour exome data
Source: BMC Genomics. 2014 Aug 28;15(1):732. doi: 10.1186/1471-2164-15-732 (PMC4162913; doi:10.1186/1471-2164-15-732)
Supplement: Supplementary file 2 — Additional file 2: Copy number and genotype profiles of the in-house generated ovarian cancer samples predicted by ADTEx. (PDF 1 MB) [file 12864_2014_6426_MOESM2_ESM.pdf]

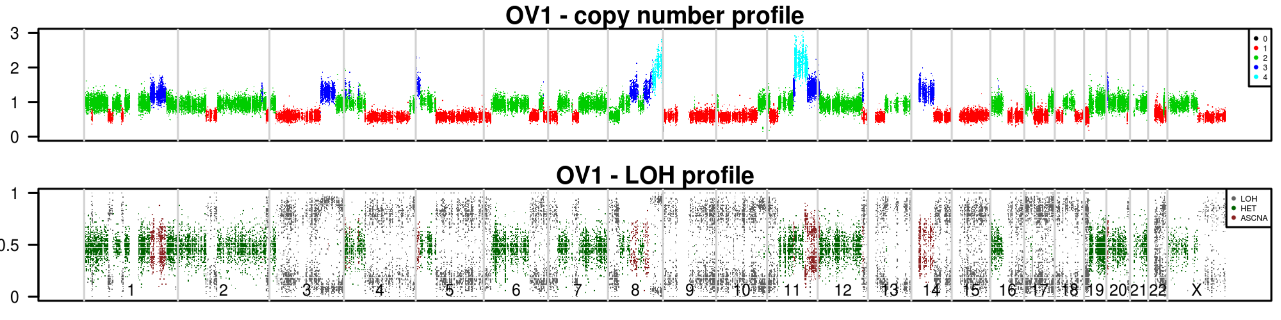
OV1 - copy number profile
0
1
2
3
4
OV1 - LOH profile
LOH
HET
ASCNA
1
2
3
4
5
6
7
8
9
10
11
12
13
14
15
16
17
18
19
20
21
22
X

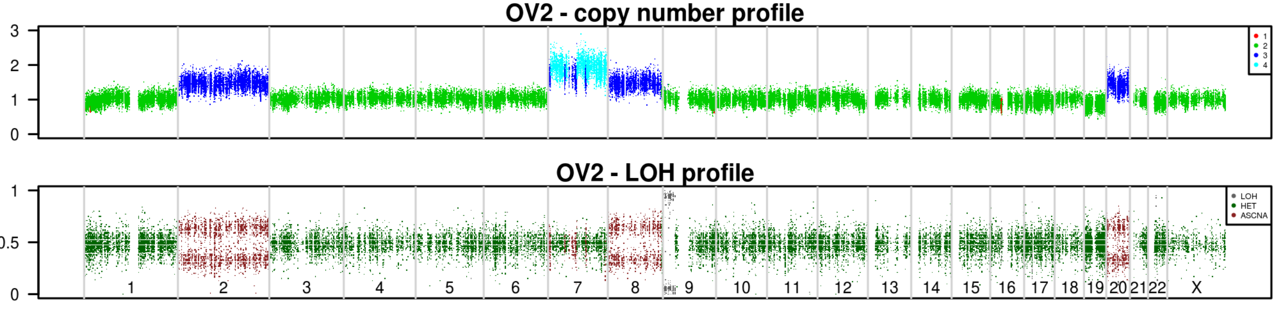

OV2 - copy number profile
3
2
1
0
1
2
3
4
OV2 - LOH profile
1
0.5
0
LOH
HET
ASCNA
1
2
3
4
5
6
7
8
9
10
11
12
13
14
15
16
17
18
19
20
21
22
X

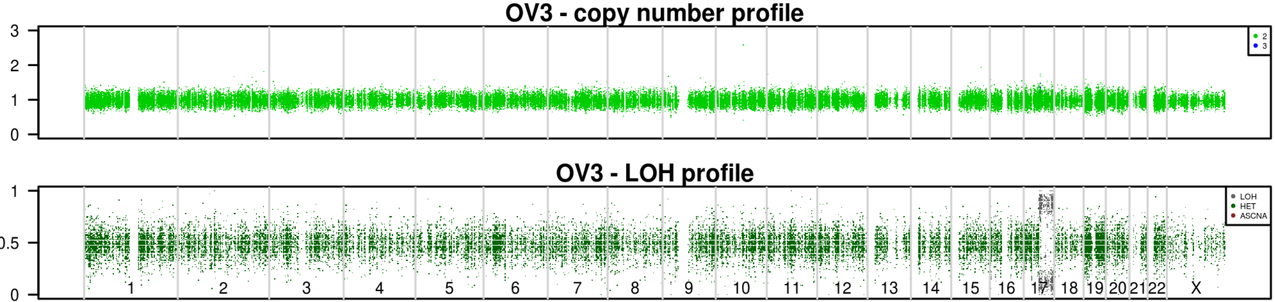
OV3 - copy number profile
3
2
1
0
2
3
OV3 - LOH profile
1
0.5
0
LOH
HET
ASCNA
1
2
3
4
5
6
7
8
9
10
11
12
13
14
15
16
17
18
19
20
21
22
X

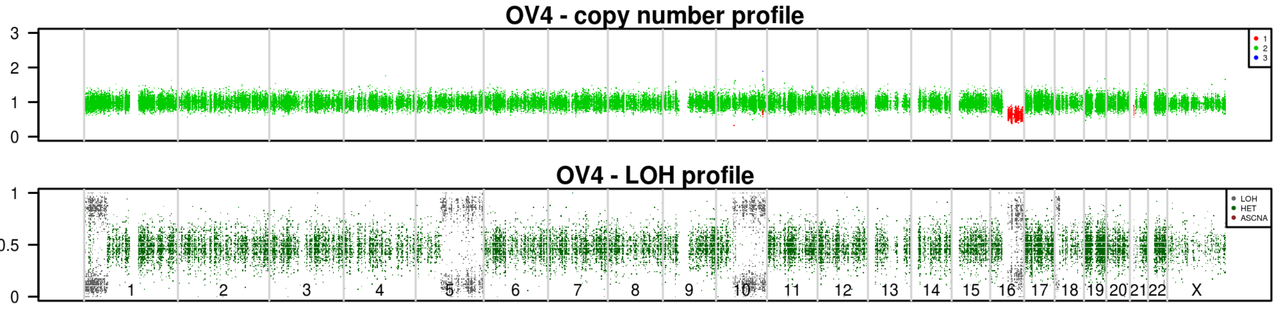

OV4 - copy number profile
3
2
1
0
1
2
3
OV4 - LOH profile
1
0.5
0
LOH
HET
ASCNA
1
2
3
4
5
6
7
8
9
10
11
12
13
14
15
16
17
18
19
20
21
22
X

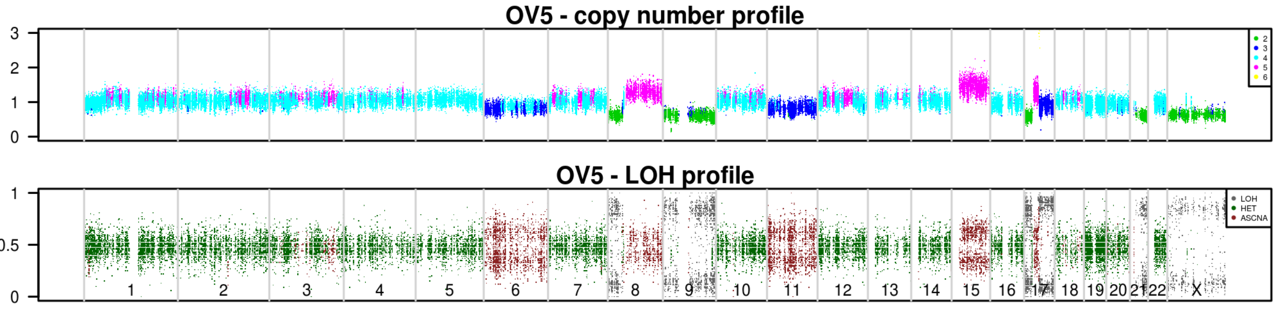

OV5 - copy number profile
3
2
1
0
2
3
4
5
6
OV5 - LOH profile
1
0.5
0
LOH
HET
ASCNA
1
2
3
4
5
6
7
8
9
10
11
12
13
14
15
16
17
18
19
20
21
22
X

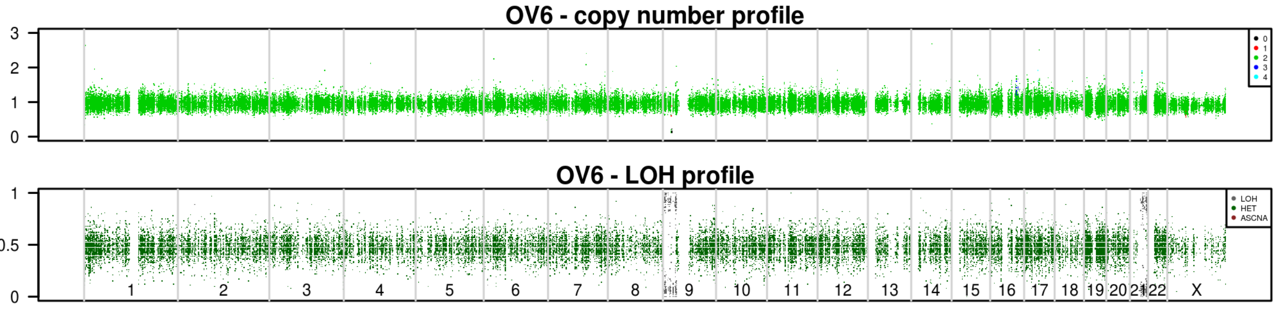
OV6 - copy number profile
3
2
1
0
0
1
2
3
4
OV6 - LOH profile
1
0.5
0
LOH
HET
ASCNA
1
2
3
4
5
6
7
8
9
10
11
12
13
14
15
16
17
18
19
20
21
22
X

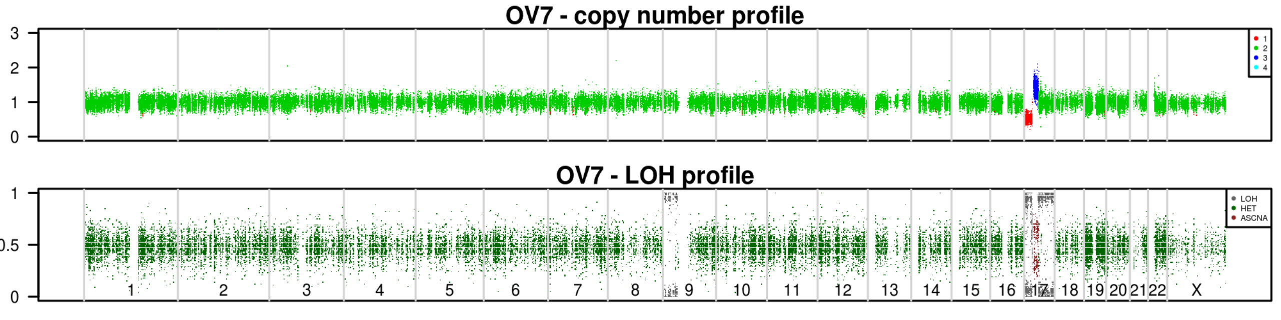

OV7 - copy number profile
3
2
1
0
1
2
3
4
OV7 - LOH profile
1
0.5
0
LOH
HET
ASCNA
1
2
3
4
5
6
7
8
9
10
11
12
13
14
15
16
17
18
19
20
21
22
X

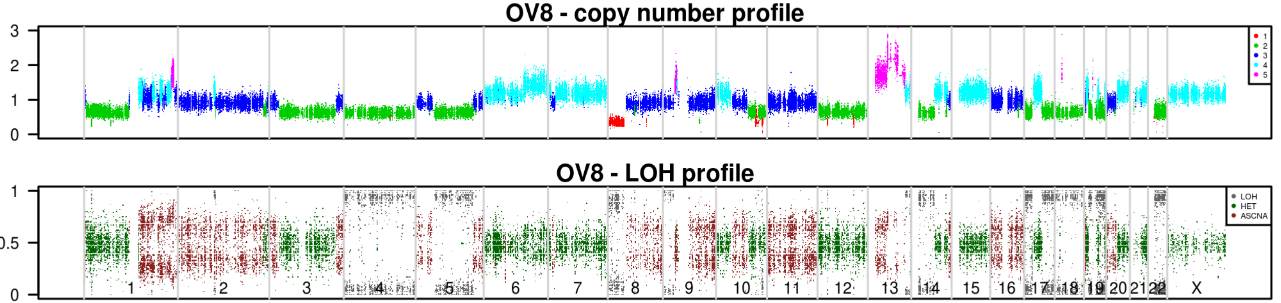
OV8 - copy number profile
OV8 - LOH profile
LOH
HET
ASCNA
1 2 3 4 5 6 7 8 9 10 11 12 13 14 15 16 17 18 19 20 21 22 X

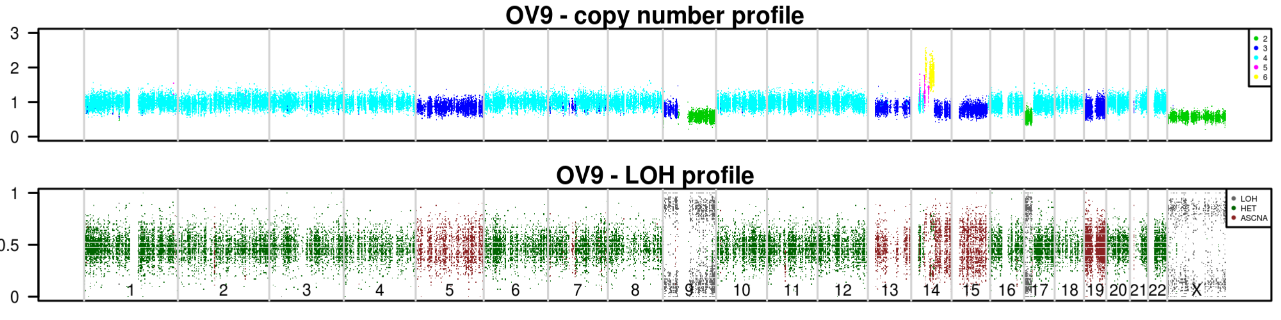

OV9 - copy number profile
3
2
1
0
2
3
4
5
6
OV9 - LOH profile
1
0.5
0
LOH
HET
ASCNA
1
2
3
4
5
6
7
8
9
10
11
12
13
14
15
16
17
18
19
20
21
22
X

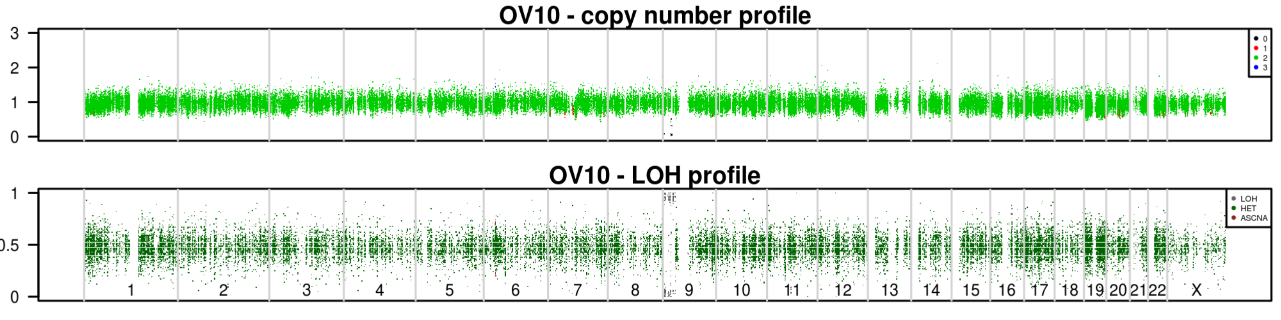
OV10 - copy number profile
0
1
2
3
OV10 - LOH profile
LOH
HET
ASCNA
0
0.5
1
1
2
3
4
5
6
7
8
9
10
11
12
13
14
15
16
17
18
19
20
21
22
X

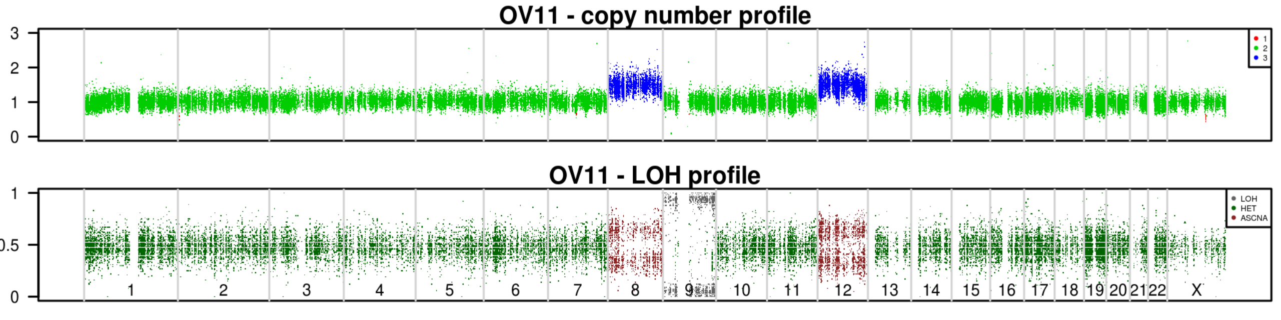
OV11 - copy number profile
3
2
1
0
1
2
3
OV11 - LOH profile
1
0.5
0
LOH
HET
ASCNA
1
2
3
4
5
6
7
8
9
10
11
12
13
14
15
16
17
18
19
20
21
22
X

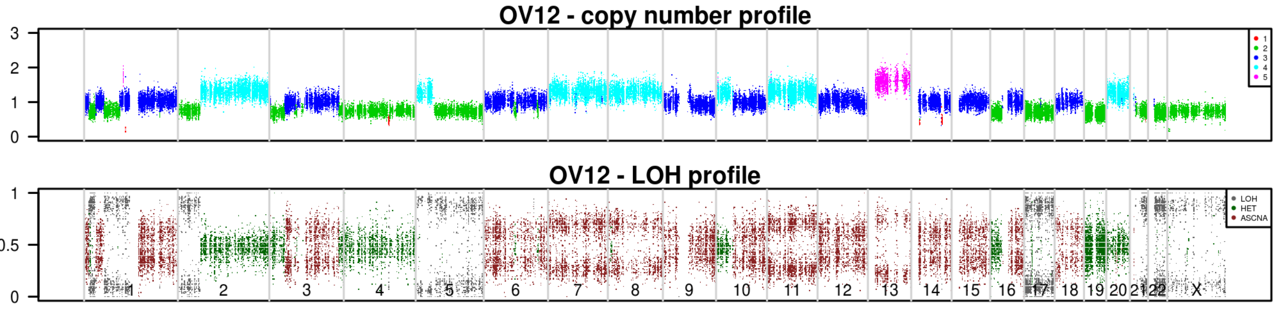
OV12 - copy number profile
3
2
1
0
1
2
3
4
5
OV12 - LOH profile
1
0.5
0
LOH
HET
ASCNA
1
2
3
4
5
6
7
8
9
10
11
12
13
14
15
16
17
18
19
20
21
22
X

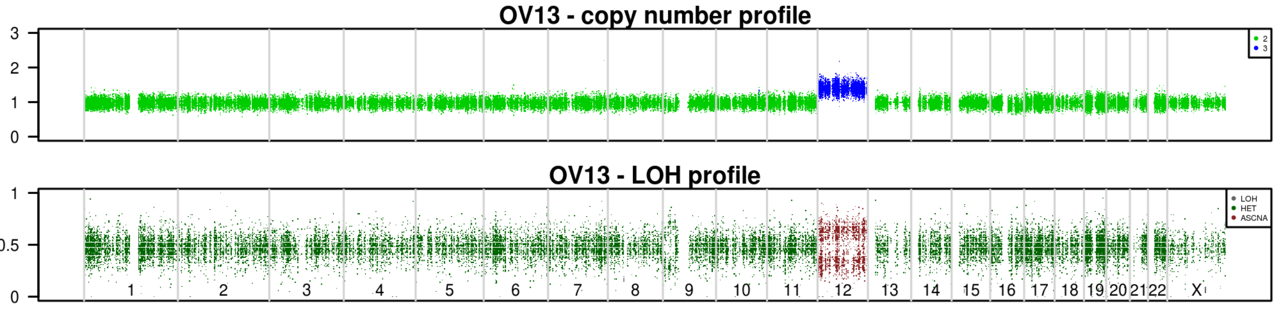
OV13 - copy number profile
3
2
1
0
2
3
OV13 - LOH profile
1
0.5
0
LOH
HET
ASCNA
1
2
3
4
5
6
7
8
9
10
11
12
13
14
15
16
17
18
19
20
21
22
X

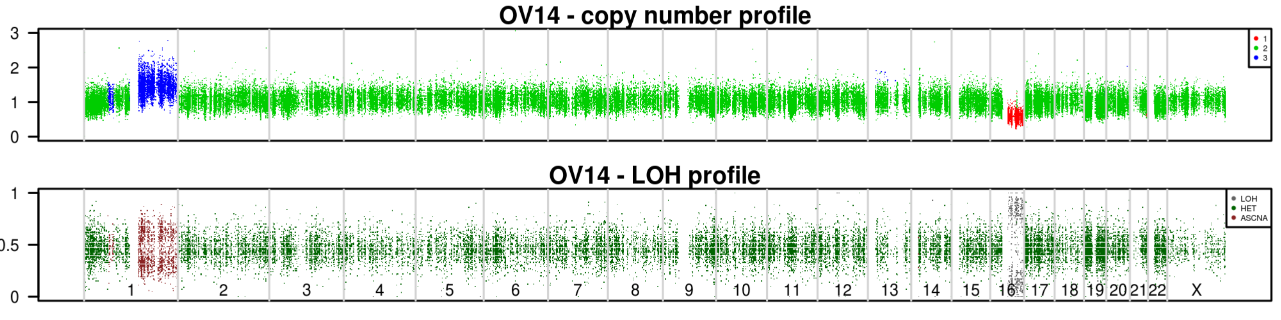

OV14 - copy number profile
3
2
1
0
1
2
3
OV14 - LOH profile
1
0.5
0
LOH
HET
ASCNA
1
2
3
4
5
6
7
8
9
10
11
12
13
14
15
16
17
18
19
20
21
22
X

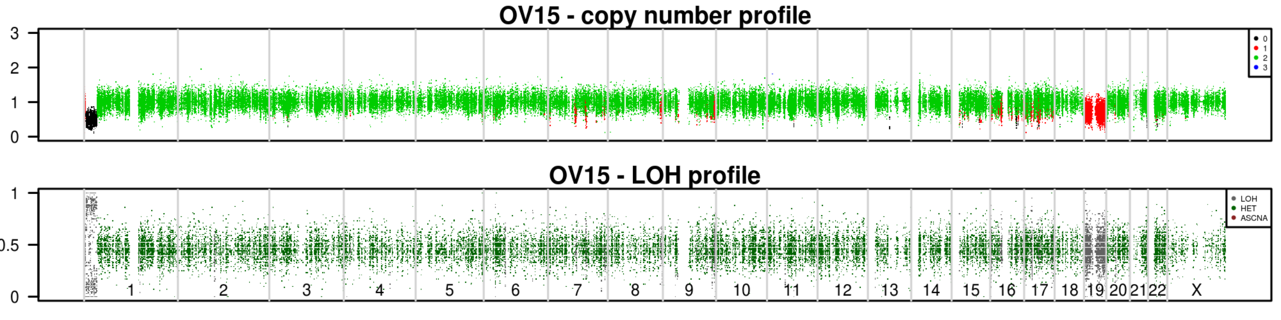
OV15 - copy number profile
0
1
2
3
OV15 - LOH profile
LOH
HET
ASCNA
0
0.5
1
1
2
3
4
5
6
7
8
9
10
11
12
13
14
15
16
17
18
19
20
21
22
X

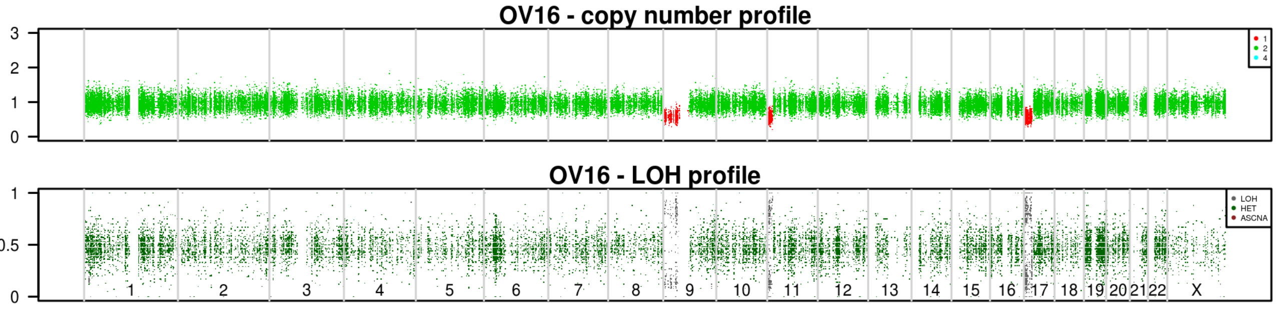
OV16 - copy number profile
3
2
1
0
1
2
4
OV16 - LOH profile
1
0.5
0
LOH
HET
ASCNA
1
2
3
4
5
6
7
8
9
10
11
12
13
14
15
16
17
18
19
20
21
22
X

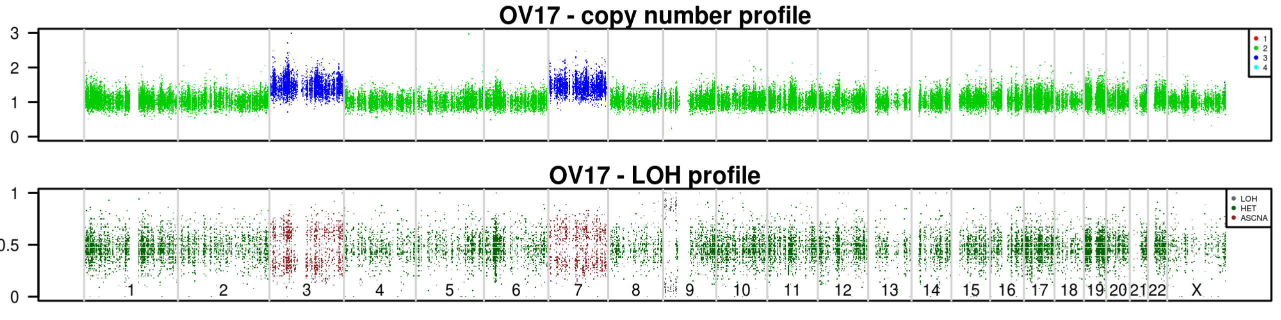
OV17 - copy number profile
3
2
1
0
1
2
3
4
OV17 - LOH profile
1
0.5
0
LOH
HET
ASCNA
1
2
3
4
5
6
7
8
9
10
11
12
13
14
15
16
17
18
19
20
21
22
X
