# Supplementary material for: Inferring copy number and genotype in tumour exome data
Source: BMC Genomics. 2014 Aug 28;15(1):732. doi: 10.1186/1471-2164-15-732 (PMC4162913; doi:10.1186/1471-2164-15-732)
Supplement: Supplementary file 3 — Additional file 3: Supplementary Table containing CNA predictions of the TCGA samples made by ADTEx. (ZIP 5 MB) [file 12864_2014_6426_MOESM3_ESM.zip › Additional_File_3.pdf]

| Sample_bar_code | Chromosome | Segment_start | Segment_end | Mean_ratio | CNV | CNV_type |
|---|---|---|---|---|---|---|
| TCGA-04-1336 | 1 | 16834 | 9995737 | 1.001661257 | 4 | amp |
| TCGA-04-1336 | 1 | 10093680 | 10182136 | 1.062940098 | 4 | amp |
| TCGA-04-1336 | 1 | 10186833 | 10479866 | 0.83642751 | 3 | amp |
| TCGA-04-1336 | 1 | 10493840 | 11141336 | 0.960940794 | 4 | amp |
| TCGA-04-1336 | 1 | 11142719 | 11177161 | 0.766897185 | 3 | amp |
| TCGA-04-1336 | 1 | 11181213 | 12294431 | 0.986886456 | 4 | amp |
| TCGA-04-1336 | 1 | 12302518 | 12338128 | 0.770918068 | 3 | amp |
| TCGA-04-1336 | 1 | 12339511 | 12785995 | 0.949624921 | 4 | amp |
| TCGA-04-1336 | 1 | 12806373 | 13351804 | 0.76972605 | 3 | amp |
| TCGA-04-1336 | 1 | 13450512 | 13522295 | 1.137580245 | 5 | amp |
| TCGA-04-1336 | 1 | 13668883 | 15871105 | 0.953522718 | 4 | amp |
| TCGA-04-1336 | 1 | 15873253 | 15976326 | 0.746857534 | 3 | amp |
| TCGA-04-1336 | 1 | 15978175 | 19488415 | 0.997971614 | 4 | amp |
| TCGA-04-1336 | 1 | 19488889 | 19585369 | 0.841752248 | 3 | amp |
| TCGA-04-1336 | 1 | 19593754 | 25599232 | 0.96658523 | 4 | amp |
| TCGA-04-1336 | 1 | 25611036 | 25815843 | 0.835780367 | 3 | amp |
| TCGA-04-1336 | 1 | 25817867 | 28887254 | 0.970940834 | 4 | amp |
| TCGA-04-1336 | 1 | 28887823 | 29543208 | 0.845539686 | 3 | amp |
| TCGA-04-1336 | 1 | 29581732 | 32860069 | 0.983088056 | 4 | amp |
| TCGA-04-1336 | 1 | 33058523 | 33160765 | 0.777103008 | 3 | amp |
| TCGA-04-1336 | 1 | 33233322 | 35791043 | 0.964503395 | 4 | amp |
| TCGA-04-1336 | 1 | 35824486 | 36226810 | 0.857795528 | 3 | amp |
| TCGA-04-1336 | 1 | 36227574 | 36748352 | 0.959317124 | 4 | amp |
| TCGA-04-1336 | 1 | 36751939 | 36904553 | 0.810283154 | 3 | amp |
| TCGA-04-1336 | 1 | 36909559 | 44134990 | 0.966111659 | 4 | amp |
| TCGA-04-1336 | 1 | 44137120 | 44422181 | 0.845652012 | 3 | amp |
| TCGA-04-1336 | 1 | 44432219 | 45219514 | 1.044745085 | 4 | amp |
| TCGA-04-1336 | 1 | 45224798 | 45974079 | 1.00943911 | 4 | amp |
| TCGA-04-1336 | 1 | 45974407 | 46083318 | 0.822864568 | 3 | amp |
| TCGA-04-1336 | 1 | 46083712 | 47776029 | 0.953459421 | 4 | amp |
| TCGA-04-1336 | 1 | 47834093 | 48688619 | 0.824682754 | 3 | amp |
| TCGA-04-1336 | 1 | 48699320 | 78044566 | 0.958784528 | 4 | amp |
| TCGA-04-1336 | 1 | 78045202 | 78207487 | 0.821239077 | 3 | amp |
| TCGA-04-1336 | 1 | 78248919 | 78381864 | 1.059005157 | 4 | amp |
| TCGA-04-1336 | 1 | 78383226 | 78433354 | 0.8173732 | 3 | amp |
| TCGA-04-1336 | 1 | 78433807 | 89298889 | 0.999278619 | 4 | amp |
| TCGA-04-1336 | 1 | 89298909 | 89849321 | 0.873642873 | 3 | amp |
| TCGA-04-1336 | 1 | 89849628 | 109740305 | 0.983895913 | 4 | amp |
| TCGA-04-1336 | 1 | 109740588 | 109780551 | 0.746376949 | 3 | amp |
| TCGA-04-1336 | 1 | 109792753 | 114951390 | 0.967466479 | 4 | amp |
| TCGA-04-1336 | 1 | 114952751 | 115231599 | 0.859840106 | 3 | amp |
| TCGA-04-1336 | 1 | 115236002 | 144158263 | 0.973077173 | 4 | amp |
| TCGA-04-1336 | 1 | 144158836 | 144922060 | 0.805577632 | 3 | amp |
| TCGA-04-1336 | 1 | 144922172 | 145304006 | 0.988745738 | 4 | amp |
| TCGA-04-1336 | 1 | 145304429 | 145528686 | 0.844376836 | 3 | amp |
| TCGA-04-1336 | 1 | 145539344 | 146024395 | 0.980762813 | 4 | amp |
| TCGA-04-1336 | 1 | 146034106 | 146232708 | 0.847035143 | 3 | amp |
| TCGA-04-1336 | 1 | 146233255 | 146419429 | 0.992510638 | 4 | amp |
| TCGA-04-1336 | 1 | 146419985 | 146466179 | 0.817771972 | 3 | amp |

| | | | | | | |
|---|---|---|---|---|---|---|
| TCGA-04-1336 | 1 | 146491172 | 148004849 | 0.934027246 | 4 | amp |
| TCGA-04-1336 | 1 | 148005301 | 148891635 | 0.817673293 | 3 | amp |
| TCGA-04-1336 | 1 | 148903066 | 149898838 | 0.958413355 | 4 | amp |
| TCGA-04-1336 | 1 | 149899001 | 150460019 | 0.860767161 | 3 | amp |
| TCGA-04-1336 | 1 | 150476770 | 150900500 | 0.916818803 | 4 | amp |
| TCGA-04-1336 | 1 | 150902368 | 150922043 | 0.82012315 | 3 | amp |
| TCGA-04-1336 | 1 | 150970057 | 151345175 | 0.857821145 | 3 | amp |
| TCGA-04-1336 | 1 | 151371983 | 153363060 | 0.96952708 | 4 | amp |
| TCGA-04-1336 | 1 | 153390508 | 153732105 | 0.829270321 | 3 | amp |
| TCGA-04-1336 | 1 | 153732736 | 153984902 | 0.943078593 | 4 | amp |
| TCGA-04-1336 | 1 | 154018503 | 154116107 | 0.997367102 | 4 | amp |
| TCGA-04-1336 | 1 | 154125159 | 154285049 | 0.777316235 | 3 | amp |
| TCGA-04-1336 | 1 | 154285426 | 154548442 | 0.954957129 | 4 | amp |
| TCGA-04-1336 | 1 | 154557220 | 154965285 | 0.854536589 | 3 | amp |
| TCGA-04-1336 | 1 | 154965325 | 155167471 | 0.965946522 | 4 | amp |
| TCGA-04-1336 | 1 | 155172001 | 155314078 | 0.992049933 | 4 | amp |
| TCGA-04-1336 | 1 | 155349810 | 155585473 | 0.985901907 | 4 | amp |
| TCGA-04-1336 | 1 | 155585509 | 155701236 | 0.772864004 | 3 | amp |
| TCGA-04-1336 | 1 | 155701719 | 155721668 | 1.072820632 | 4 | amp |
| TCGA-04-1336 | 1 | 155721704 | 155784300 | 0.755121346 | 3 | amp |
| TCGA-04-1336 | 1 | 155785583 | 155933028 | 0.979180142 | 4 | amp |
| TCGA-04-1336 | 1 | 155934728 | 156006964 | 0.715383437 | 3 | amp |
| TCGA-04-1336 | 1 | 156011248 | 157106181 | 0.969551974 | 4 | amp |
| TCGA-04-1336 | 1 | 157485419 | 158047975 | 0.849360598 | 3 | amp |
| TCGA-04-1336 | 1 | 158057507 | 158152986 | 1.048106078 | 4 | amp |
| TCGA-04-1336 | 1 | 158323717 | 159796768 | 0.938075442 | 4 | amp |
| TCGA-04-1336 | 1 | 159799608 | 159889657 | 0.808649197 | 3 | amp |
| TCGA-04-1336 | 1 | 159890059 | 160094272 | 0.988531138 | 4 | amp |
| TCGA-04-1336 | 1 | 160167321 | 160314623 | 0.858672736 | 3 | amp |
| TCGA-04-1336 | 1 | 160340497 | 160924583 | 0.890327123 | 3 | amp |
| TCGA-04-1336 | 1 | 160990771 | 161179743 | 0.994858166 | 4 | amp |
| TCGA-04-1336 | 1 | 161205595 | 161683202 | 0.819635555 | 3 | amp |
| TCGA-04-1336 | 1 | 161692387 | 175092847 | 0.944534232 | 4 | amp |
| TCGA-04-1336 | 1 | 175096035 | 176740329 | 0.861066592 | 3 | amp |
| TCGA-04-1336 | 1 | 176758908 | 178858863 | 0.975831727 | 4 | amp |
| TCGA-04-1336 | 1 | 178861343 | 179091045 | 0.783547949 | 3 | amp |
| TCGA-04-1336 | 1 | 179095479 | 180974634 | 1.012954714 | 4 | amp |
| TCGA-04-1336 | 1 | 181003086 | 181693699 | 1.243497966 | 5 | amp |
| TCGA-04-1336 | 1 | 181695189 | 181708395 | 1.935789755 | 6 | amp |
| TCGA-04-1336 | 1 | 181714430 | 181754961 | 1.312680269 | 5 | amp |
| TCGA-04-1336 | 1 | 181759576 | 182357909 | 1.611213573 | 6 | amp |
| TCGA-04-1336 | 1 | 182368759 | 182555981 | 1.236761977 | 5 | amp |
| TCGA-04-1336 | 1 | 182569387 | 182787869 | 1.539026586 | 6 | amp |
| TCGA-04-1336 | 1 | 182787910 | 182856573 | 1.362607347 | 5 | amp |
| TCGA-04-1336 | 1 | 182869063 | 182920647 | 1.630336357 | 6 | amp |
| TCGA-04-1336 | 1 | 183072404 | 183192484 | 1.250777579 | 5 | amp |
| TCGA-04-1336 | 1 | 183194708 | 183212588 | 1.653005434 | 6 | amp |
| TCGA-04-1336 | 1 | 183221736 | 183487008 | 1.204095354 | 5 | amp |
| TCGA-04-1336 | 1 | 183495666 | 183503008 | 1.628579822 | 6 | amp |
| TCGA-04-1336 | 1 | 183506254 | 185144294 | 1.315201906 | 5 | amp |

| | | | | | | |
|---|---|---|---|---|---|---|
| TCGA-04-1336 | 1 | 185149492 | 185159787 | 1.59942056 | 6 | amp |
| TCGA-04-1336 | 1 | 185171738 | 185878674 | 1.397157947 | 5 | amp |
| TCGA-04-1336 | 1 | 185880768 | 185947135 | 1.588392141 | 6 | amp |
| TCGA-04-1336 | 1 | 185950067 | 186645845 | 1.350663411 | 5 | amp |
| TCGA-04-1336 | 1 | 186645916 | 186648631 | 1.904558927 | 6 | amp |
| TCGA-04-1336 | 1 | 186823442 | 192628680 | 1.310157935 | 5 | amp |
| TCGA-04-1336 | 1 | 192778196 | 192997306 | 1.846425288 | 6 | amp |
| TCGA-04-1336 | 1 | 192998253 | 197094368 | 1.34380381 | 5 | amp |
| TCGA-04-1336 | 1 | 197097587 | 197115478 | 1.593181399 | 6 | amp |
| TCGA-04-1336 | 1 | 197128456 | 197411478 | 1.38495162 | 5 | amp |
| TCGA-04-1336 | 1 | 197446751 | 198248197 | 1.546262455 | 6 | amp |
| TCGA-04-1336 | 1 | 198262061 | 198713354 | 1.382350549 | 5 | amp |
| TCGA-04-1336 | 1 | 198717222 | 200378388 | 1.648439635 | 6 | amp |
| TCGA-04-1336 | 1 | 200522435 | 200827214 | 1.383861191 | 5 | amp |
| TCGA-04-1336 | 1 | 200867388 | 201336976 | 1.569830456 | 6 | amp |
| TCGA-04-1336 | 1 | 201337232 | 201382264 | 1.328455056 | 5 | amp |
| TCGA-04-1336 | 1 | 201383591 | 202128707 | 0.948900526 | 4 | amp |
| TCGA-04-1336 | 1 | 202166135 | 202746243 | 0.868983709 | 3 | amp |
| TCGA-04-1336 | 1 | 202850048 | 203472882 | 1.018220124 | 4 | amp |
| TCGA-04-1336 | 1 | 203652279 | 203834267 | 0.807335759 | 3 | amp |
| TCGA-04-1336 | 1 | 203838972 | 205053484 | 1.002993978 | 4 | amp |
| TCGA-04-1336 | 1 | 205057838 | 205240435 | 0.818909318 | 3 | amp |
| TCGA-04-1336 | 1 | 205240916 | 205351043 | 0.955172978 | 4 | amp |
| TCGA-04-1336 | 1 | 205388298 | 205498564 | 1.358099044 | 5 | amp |
| TCGA-04-1336 | 1 | 205498579 | 205779623 | 0.984747334 | 4 | amp |
| TCGA-04-1336 | 1 | 205797689 | 206650220 | 0.861067577 | 3 | amp |
| TCGA-04-1336 | 1 | 206651472 | 207685016 | 0.931029673 | 4 | amp |
| TCGA-04-1336 | 1 | 207696914 | 207783073 | 0.826326097 | 3 | amp |
| TCGA-04-1336 | 1 | 207784989 | 223949980 | 0.925248752 | 4 | amp |
| TCGA-04-1336 | 1 | 223951820 | 225600371 | 0.845736399 | 3 | amp |
| TCGA-04-1336 | 1 | 225602946 | 230891177 | 0.932722459 | 4 | amp |
| TCGA-04-1336 | 1 | 230895196 | 230991528 | 0.788389633 | 3 | amp |
| TCGA-04-1336 | 1 | 231042617 | 231126052 | 1.004637965 | 4 | amp |
| TCGA-04-1336 | 1 | 231131497 | 234541866 | 0.878752706 | 3 | amp |
| TCGA-04-1336 | 1 | 234546173 | 236390052 | 0.928882775 | 4 | amp |
| TCGA-04-1336 | 1 | 236393476 | 237060960 | 0.877922841 | 3 | amp |
| TCGA-04-1336 | 1 | 237433796 | 241749931 | 0.981934478 | 4 | amp |
| TCGA-04-1336 | 1 | 241749977 | 242162341 | 0.842188009 | 3 | amp |
| TCGA-04-1336 | 1 | 242253103 | 249231325 | 0.938556064 | 4 | amp |
| TCGA-04-1336 | 10 | 92880 | 1046980 | 0.996763613 | 4 | amp |
| TCGA-04-1336 | 10 | 1051712 | 1065795 | 1.252525085 | 5 | amp |
| TCGA-04-1336 | 10 | 1066621 | 7697675 | 0.975031986 | 4 | amp |
| TCGA-04-1336 | 10 | 7745349 | 7786263 | 0.712505469 | 3 | amp |
| TCGA-04-1336 | 10 | 7786719 | 11574263 | 0.922038155 | 4 | amp |
| TCGA-04-1336 | 10 | 11789288 | 13717097 | 1.218038289 | 5 | amp |
| TCGA-04-1336 | 10 | 13735818 | 27337970 | 0.897369896 | 4 | amp |
| TCGA-04-1336 | 10 | 27342224 | 27512383 | 0.793243885 | 3 | amp |
| TCGA-04-1336 | 10 | 27520652 | 37425626 | 0.909519106 | 4 | amp |
| TCGA-04-1336 | 10 | 37430631 | 38246530 | 0.788138875 | 3 | amp |
| TCGA-04-1336 | 10 | 38260571 | 70229948 | 0.938777491 | 4 | amp |

| | | | | | | |
|---|---|---|---|---|---|---|
| TCGA-04-1336 | 10 | 70243146 | 70703023 | 0.769317781 | 3 | amp |
| TCGA-04-1336 | 10 | 70706066 | 75160645 | 0.943549019 | 4 | amp |
| TCGA-04-1336 | 10 | 75184300 | 75530902 | 0.792162386 | 3 | amp |
| TCGA-04-1336 | 10 | 75532871 | 95157238 | 0.924399997 | 4 | amp |
| TCGA-04-1336 | 10 | 95159140 | 95400791 | 0.800454366 | 3 | amp |
| TCGA-04-1336 | 10 | 95405700 | 97174674 | 0.93434424 | 4 | amp |
| TCGA-04-1336 | 10 | 97181683 | 98744939 | 0.846639768 | 3 | amp |
| TCGA-04-1336 | 10 | 98762394 | 101998003 | 0.895378596 | 4 | amp |
| TCGA-04-1336 | 10 | 102003404 | 102089084 | 0.737763688 | 3 | amp |
| TCGA-04-1336 | 10 | 102089592 | 103989035 | 0.922434207 | 4 | amp |
| TCGA-04-1336 | 10 | 104018653 | 104130660 | 0.744905294 | 3 | amp |
| TCGA-04-1336 | 10 | 104135108 | 104170719 | 1.139990072 | 5 | amp |
| TCGA-04-1336 | 10 | 104170783 | 104486948 | 0.979255532 | 4 | amp |
| TCGA-04-1336 | 10 | 104488146 | 104620336 | 0.70705019 | 3 | amp |
| TCGA-04-1336 | 10 | 104621836 | 112362791 | 0.933026997 | 4 | amp |
| TCGA-04-1336 | 10 | 112362934 | 112771591 | 0.797709425 | 3 | amp |
| TCGA-04-1336 | 11 | 86637 | 294772 | 0.929844618 | 4 | amp |
| TCGA-04-1336 | 11 | 308105 | 428676 | 0.765841162 | 3 | amp |
| TCGA-04-1336 | 11 | 460177 | 3697983 | 0.913571691 | 4 | amp |
| TCGA-04-1336 | 11 | 3716607 | 4148495 | 0.817571707 | 3 | amp |
| TCGA-04-1336 | 11 | 4150262 | 4389532 | 1.056881369 | 4 | amp |
| TCGA-04-1336 | 11 | 4470561 | 5618195 | 0.830097038 | 3 | amp |
| TCGA-04-1336 | 11 | 5699428 | 6078950 | 0.836667213 | 3 | amp |
| TCGA-04-1336 | 11 | 6079190 | 6453301 | 0.958935687 | 4 | amp |
| TCGA-04-1336 | 11 | 6462091 | 6704560 | 0.936199729 | 4 | amp |
| TCGA-04-1336 | 11 | 6789232 | 7507290 | 0.840146913 | 3 | amp |
| TCGA-04-1336 | 11 | 7509321 | 7985077 | 0.934461899 | 4 | amp |
| TCGA-04-1336 | 11 | 8008790 | 8161746 | 0.867906242 | 3 | amp |
| TCGA-04-1336 | 11 | 8246155 | 8953891 | 0.939831322 | 4 | amp |
| TCGA-04-1336 | 11 | 8959135 | 9161429 | 0.818659678 | 3 | amp |
| TCGA-04-1336 | 11 | 9215014 | 9917583 | 0.842021272 | 3 | amp |
| TCGA-04-1336 | 11 | 9983458 | 10786306 | 0.930893686 | 4 | amp |
| TCGA-04-1336 | 11 | 10787902 | 10800678 | 0.818328775 | 3 | amp |
| TCGA-04-1336 | 11 | 10819305 | 10824031 | 1.239742073 | 5 | amp |
| TCGA-04-1336 | 11 | 10824555 | 10828881 | 0.989904696 | 4 | amp |
| TCGA-04-1336 | 11 | 11292643 | 12886475 | 0.878823607 | 3 | amp |
| TCGA-04-1336 | 11 | 12951676 | 13388390 | 0.992479424 | 4 | amp |
| TCGA-04-1336 | 11 | 13391119 | 13743461 | 0.828464091 | 3 | amp |
| TCGA-04-1336 | 11 | 13749046 | 16838907 | 0.940951511 | 4 | amp |
| TCGA-04-1336 | 11 | 16996218 | 17519823 | 0.873628131 | 3 | amp |
| TCGA-04-1336 | 11 | 17522587 | 18339411 | 0.94627374 | 4 | amp |
| TCGA-04-1336 | 11 | 18354578 | 18741842 | 0.854317032 | 3 | amp |
| TCGA-04-1336 | 11 | 18742999 | 19914179 | 0.96699473 | 4 | amp |
| TCGA-04-1336 | 11 | 19954647 | 20129388 | 0.778839003 | 3 | amp |
| TCGA-04-1336 | 11 | 20136119 | 33038168 | 0.933454242 | 4 | amp |
| TCGA-04-1336 | 11 | 33047159 | 34167744 | 0.851054772 | 3 | amp |
| TCGA-04-1336 | 11 | 34175778 | 34664285 | 0.974621586 | 4 | amp |
| TCGA-04-1336 | 11 | 34667898 | 34680488 | 1.888875653 | 6 | amp |
| TCGA-04-1336 | 11 | 34904253 | 35202007 | 0.942633708 | 4 | amp |
| TCGA-04-1336 | 11 | 35208322 | 36655030 | 0.849678939 | 3 | amp |

| | | | | | | |
|---|---|---|---|---|---|---|
| TCGA-04-1336 | 11 | 36657543 | 43345202 | 1.190998192 | 5 | amp |
| TCGA-04-1336 | 11 | 43348048 | 43472833 | 0.842228214 | 3 | amp |
| TCGA-04-1336 | 11 | 43511698 | 46430279 | 0.936558503 | 4 | amp |
| TCGA-04-1336 | 11 | 46431781 | 46666988 | 0.845261553 | 3 | amp |
| TCGA-04-1336 | 11 | 46693783 | 46804930 | 0.918897266 | 4 | amp |
| TCGA-04-1336 | 11 | 46806010 | 46907742 | 0.81352057 | 3 | amp |
| TCGA-04-1336 | 11 | 46907859 | 47282272 | 0.940866593 | 4 | amp |
| TCGA-04-1336 | 11 | 47282745 | 47303334 | 0.800957777 | 3 | amp |
| TCGA-04-1336 | 11 | 47330135 | 47602581 | 0.950069834 | 4 | amp |
| TCGA-04-1336 | 11 | 47603582 | 47732115 | 0.769612022 | 3 | amp |
| TCGA-04-1336 | 11 | 47735807 | 47753139 | 1.122084029 | 5 | amp |
| TCGA-04-1336 | 11 | 47786770 | 48486305 | 0.871724357 | 3 | amp |
| TCGA-04-1336 | 11 | 48510329 | 57135589 | 0.967722583 | 4 | amp |
| TCGA-04-1336 | 11 | 57135787 | 57177620 | 0.783924817 | 3 | amp |
| TCGA-04-1336 | 11 | 57182025 | 58602345 | 0.933984966 | 4 | amp |
| TCGA-04-1336 | 11 | 58604448 | 58723531 | 1.216048488 | 5 | amp |
| TCGA-04-1336 | 11 | 58877078 | 59419125 | 0.918410015 | 4 | amp |
| TCGA-04-1336 | 11 | 59419878 | 59599329 | 0.848534389 | 3 | amp |
| TCGA-04-1336 | 11 | 59623316 | 59949246 | 0.849543029 | 3 | amp |
| TCGA-04-1336 | 11 | 60048078 | 60541426 | 0.995446461 | 4 | amp |
| TCGA-04-1336 | 11 | 60563022 | 60901713 | 0.963488329 | 4 | amp |
| TCGA-04-1336 | 11 | 60906194 | 61018798 | 0.772668987 | 3 | amp |
| TCGA-04-1336 | 11 | 61026075 | 61071541 | 1.005004447 | 4 | amp |
| TCGA-04-1336 | 11 | 61089027 | 61960945 | 0.959644493 | 4 | amp |
| TCGA-04-1336 | 11 | 61976140 | 62361549 | 0.864473789 | 3 | amp |
| TCGA-04-1336 | 11 | 62363719 | 62374620 | 0.869476578 | 3 | amp |
| TCGA-04-1336 | 11 | 62376737 | 62393401 | 0.901588654 | 3 | amp |
| TCGA-04-1336 | 11 | 62413992 | 62496619 | 0.951015675 | 4 | amp |
| TCGA-04-1336 | 11 | 62533882 | 62549462 | 0.815290095 | 3 | amp |
| TCGA-04-1336 | 11 | 62549574 | 62560202 | 1.07070843 | 4 | amp |
| TCGA-04-1336 | 11 | 62564622 | 63326177 | 0.94969114 | 4 | amp |
| TCGA-04-1336 | 11 | 63396773 | 63525807 | 0.814559468 | 3 | amp |
| TCGA-04-1336 | 11 | 63585231 | 64402953 | 0.998061685 | 4 | amp |
| TCGA-04-1336 | 11 | 64427755 | 64460358 | 0.813155564 | 3 | amp |
| TCGA-04-1336 | 11 | 64465195 | 64694401 | 0.966231643 | 4 | amp |
| TCGA-04-1336 | 11 | 64695234 | 64726886 | 0.760343747 | 3 | amp |
| TCGA-04-1336 | 11 | 64756552 | 64990125 | 0.973673574 | 4 | amp |
| TCGA-04-1336 | 11 | 64990933 | 65111321 | 0.787325113 | 3 | amp |
| TCGA-04-1336 | 11 | 65123237 | 66264978 | 0.977851012 | 4 | amp |
| TCGA-04-1336 | 11 | 66287033 | 67829520 | 0.946697193 | 4 | amp |
| TCGA-04-1336 | 11 | 67831984 | 67957583 | 0.818811361 | 3 | amp |
| TCGA-04-1336 | 11 | 68029089 | 71729620 | 0.983870341 | 4 | amp |
| TCGA-04-1336 | 11 | 71729827 | 71907260 | 0.816444186 | 3 | amp |
| TCGA-04-1336 | 11 | 71927895 | 75439221 | 0.962442626 | 4 | amp |
| TCGA-04-1336 | 11 | 75511355 | 76956556 | 0.962528687 | 4 | amp |
| TCGA-04-1336 | 11 | 76969358 | 77911894 | 0.868623534 | 3 | amp |
| TCGA-04-1336 | 11 | 77916834 | 86108843 | 0.956806311 | 4 | amp |
| TCGA-04-1336 | 11 | 86111661 | 86161047 | 0.786792742 | 3 | amp |
| TCGA-04-1336 | 11 | 86161331 | 89596111 | 0.942559866 | 4 | amp |
| TCGA-04-1336 | 11 | 89644704 | 89666103 | 0.761646329 | 3 | amp |

| | | | | | | |
|---|---|---|---|---|---|---|
| TCGA-04-1336 | 11 | 89714734 | 89892515 | 0.93378674 | 4 | amp |
| TCGA-04-1336 | 11 | 89896099 | 89903330 | 1.266707095 | 5 | amp |
| TCGA-04-1336 | 11 | 89906977 | 102487846 | 0.952014499 | 4 | amp |
| TCGA-04-1336 | 11 | 102495837 | 102595595 | 0.806704322 | 3 | amp |
| TCGA-04-1336 | 11 | 102641513 | 104825780 | 1.004368079 | 4 | amp |
| TCGA-04-1336 | 11 | 104839158 | 104916080 | 0.81382143 | 3 | amp |
| TCGA-04-1336 | 11 | 104970028 | 107526725 | 0.947645496 | 4 | amp |
| TCGA-04-1336 | 11 | 107535716 | 108047828 | 0.848012325 | 3 | amp |
| TCGA-04-1336 | 11 | 108055912 | 108139356 | 0.983904966 | 4 | amp |
| TCGA-04-1336 | 11 | 108141771 | 108412578 | 0.862534945 | 3 | amp |
| TCGA-04-1336 | 11 | 108464083 | 112101436 | 0.94456744 | 4 | amp |
| TCGA-04-1336 | 11 | 112103830 | 113076953 | 1.24364002 | 5 | amp |
| TCGA-04-1336 | 11 | 113077981 | 116707914 | 0.958712447 | 4 | amp |
| TCGA-04-1336 | 11 | 116717070 | 117053345 | 0.84960546 | 3 | amp |
| TCGA-04-1336 | 11 | 117053390 | 117073939 | 0.971775822 | 4 | amp |
| TCGA-04-1336 | 11 | 117073991 | 117117714 | 0.866003424 | 3 | amp |
| TCGA-04-1336 | 11 | 117160276 | 117261942 | 0.815652267 | 3 | amp |
| TCGA-04-1336 | 11 | 117262890 | 117965610 | 0.953246213 | 4 | amp |
| TCGA-04-1336 | 11 | 118007728 | 118076711 | 0.989070481 | 4 | amp |
| TCGA-04-1336 | 11 | 118081194 | 118123032 | 0.800458897 | 3 | amp |
| TCGA-04-1336 | 11 | 118175601 | 118257359 | 0.819888784 | 3 | amp |
| TCGA-04-1336 | 11 | 118260409 | 118362670 | 1.002935428 | 4 | amp |
| TCGA-04-1336 | 11 | 118363708 | 118370648 | 1.243149684 | 5 | amp |
| TCGA-04-1336 | 11 | 118371661 | 118495832 | 0.799989911 | 3 | amp |
| TCGA-04-1336 | 11 | 118497943 | 118900125 | 0.970935977 | 4 | amp |
| TCGA-04-1336 | 11 | 118916275 | 118955819 | 0.75990219 | 3 | amp |
| TCGA-04-1336 | 11 | 118958901 | 119991386 | 0.942480161 | 4 | amp |
| TCGA-04-1336 | 11 | 119993652 | 121037507 | 0.851293671 | 3 | amp |
| TCGA-04-1336 | 11 | 121038724 | 121483552 | 0.957204534 | 4 | amp |
| TCGA-04-1336 | 11 | 121485551 | 123516496 | 0.828832239 | 3 | amp |
| TCGA-04-1336 | 11 | 123524391 | 125525227 | 0.953704094 | 4 | amp |
| TCGA-04-1336 | 11 | 125769233 | 126081558 | 0.930248266 | 4 | amp |
| TCGA-04-1336 | 11 | 126131289 | 128426377 | 0.870579131 | 3 | amp |
| TCGA-04-1336 | 11 | 128442930 | 134051085 | 0.985416195 | 4 | amp |
| TCGA-04-1336 | 11 | 134054499 | 134087030 | 0.79031165 | 3 | amp |
| TCGA-04-1336 | 11 | 134090422 | 134257557 | 0.963370629 | 4 | amp |
| TCGA-04-1336 | 12 | 42862409 | 43825336 | 0.770409308 | 3 | amp |
| TCGA-04-1336 | 12 | 43826068 | 45173553 | 0.967036334 | 4 | amp |
| TCGA-04-1336 | 12 | 45173598 | 45568178 | 1.280916793 | 5 | amp |
| TCGA-04-1336 | 12 | 45695776 | 48516659 | 0.989513502 | 4 | amp |
| TCGA-04-1336 | 12 | 48524122 | 49099734 | 0.805672156 | 3 | amp |
| TCGA-04-1336 | 12 | 49108206 | 49392944 | 0.984162223 | 4 | amp |
| TCGA-04-1336 | 12 | 49406778 | 49422795 | 1.043437213 | 4 | amp |
| TCGA-04-1336 | 12 | 49422811 | 49438757 | 1.198193574 | 5 | amp |
| TCGA-04-1336 | 12 | 49439666 | 51128944 | 0.96491849 | 4 | amp |
| TCGA-04-1336 | 12 | 51130765 | 51510210 | 0.806924629 | 3 | amp |
| TCGA-04-1336 | 12 | 51511431 | 52387951 | 0.987228334 | 4 | amp |
| TCGA-04-1336 | 12 | 52402908 | 52779396 | 1.156111968 | 5 | amp |
| TCGA-04-1336 | 12 | 52788718 | 53043787 | 0.996924113 | 4 | amp |
| TCGA-04-1336 | 12 | 53044109 | 53187036 | 1.135043234 | 5 | amp |

| | | | | | | |
|---|---|---|---|---|---|---|
| TCGA-04-1336 | 12 | 53187884 | 53697017 | 0.992122656 | 4 | amp |
| TCGA-04-1336 | 12 | 53708493 | 53994820 | 0.920353181 | 4 | amp |
| TCGA-04-1336 | 12 | 54059094 | 54119068 | 0.677071775 | 3 | amp |
| TCGA-04-1336 | 12 | 54338701 | 54905930 | 0.9681917 | 4 | amp |
| TCGA-04-1336 | 12 | 54909990 | 55250780 | 0.756483869 | 3 | amp |
| TCGA-04-1336 | 12 | 55251941 | 56142808 | 0.952606065 | 4 | amp |
| TCGA-04-1336 | 12 | 56143235 | 56297277 | 0.779743447 | 3 | amp |
| TCGA-04-1336 | 12 | 56330229 | 56380945 | 0.990375626 | 4 | amp |
| TCGA-04-1336 | 12 | 56383686 | 57571045 | 0.832133422 | 3 | amp |
| TCGA-04-1336 | 12 | 57571141 | 57638422 | 1.000000537 | 4 | amp |
| TCGA-04-1336 | 12 | 57638633 | 57677722 | 0.676982767 | 3 | amp |
| TCGA-04-1336 | 12 | 57828615 | 57829005 | 0.900557408 | 4 | amp |
| TCGA-04-1336 | 12 | 57842982 | 57926614 | 1.220675593 | 5 | amp |
| TCGA-04-1336 | 12 | 57926725 | 57944238 | 1.000654546 | 4 | amp |
| TCGA-04-1336 | 12 | 57957205 | 57976458 | 0.763905193 | 3 | amp |
| TCGA-04-1336 | 12 | 57976862 | 104067894 | 0.944381999 | 4 | amp |
| TCGA-04-1336 | 12 | 104069662 | 104208926 | 0.800166226 | 3 | amp |
| TCGA-04-1336 | 12 | 104325289 | 110403627 | 0.922908266 | 4 | amp |
| TCGA-04-1336 | 12 | 110405061 | 111701663 | 0.824959572 | 3 | amp |
| TCGA-04-1336 | 12 | 111729168 | 120598062 | 0.906189836 | 4 | amp |
| TCGA-04-1336 | 12 | 120599280 | 120628211 | 0.73493705 | 3 | amp |
| TCGA-04-1336 | 12 | 120634530 | 122284863 | 0.917473302 | 4 | amp |
| TCGA-04-1336 | 12 | 122284918 | 122481995 | 0.796981174 | 3 | amp |
| TCGA-04-1336 | 12 | 122492652 | 123664564 | 0.911368625 | 4 | amp |
| TCGA-04-1336 | 12 | 123665673 | 123835042 | 0.780621852 | 3 | amp |
| TCGA-04-1336 | 12 | 123875104 | 132490871 | 0.994845714 | 4 | amp |
| TCGA-04-1336 | 12 | 132491213 | 133236107 | 1.119775862 | 5 | amp |
| TCGA-04-1336 | 12 | 133237502 | 133779395 | 1.00721735 | 4 | amp |
| TCGA-04-1336 | 13 | 107164838 | 109617299 | 0.983910333 | 4 | amp |
| TCGA-04-1336 | 13 | 109644699 | 110830614 | 1.197318427 | 5 | amp |
| TCGA-04-1336 | 13 | 110831266 | 110862255 | 1.018686047 | 4 | amp |
| TCGA-04-1336 | 13 | 110894961 | 113739505 | 1.15518888 | 5 | amp |
| TCGA-04-1336 | 13 | 113740350 | 113964187 | 0.999408762 | 4 | amp |
| TCGA-04-1336 | 13 | 113964982 | 114469279 | 1.227008659 | 5 | amp |
| TCGA-04-1336 | 13 | 114472042 | 115091796 | 0.948561759 | 4 | amp |
| TCGA-04-1336 | 14 | 19377543 | 102486431 | 0.912888996 | 4 | amp |
| TCGA-04-1336 | 14 | 102504749 | 102552754 | 1.095637519 | 5 | amp |
| TCGA-04-1336 | 14 | 102568166 | 105518484 | 0.947524035 | 4 | amp |
| TCGA-04-1336 | 14 | 105521680 | 106406091 | 1.26912127 | 5 | amp |
| TCGA-04-1336 | 14 | 106452641 | 107283263 | 0.959361204 | 4 | amp |
| TCGA-04-1336 | 15 | 43762039 | 69728658 | 0.899323675 | 4 | amp |
| TCGA-04-1336 | 15 | 69728861 | 90143925 | 1.359630261 | 5 | amp |
| TCGA-04-1336 | 15 | 90144515 | 91835810 | 2.093819464 | 6 | amp |
| TCGA-04-1336 | 15 | 92638099 | 102516522 | 1.348633144 | 5 | amp |
| TCGA-04-1336 | 16 | 5040711 | 8862875 | 0.754111214 | 3 | amp |
| TCGA-04-1336 | 16 | 8866555 | 14743863 | 0.891895889 | 4 | amp |
| TCGA-04-1336 | 16 | 14748844 | 15103636 | 0.752436642 | 3 | amp |
| TCGA-04-1336 | 16 | 15109967 | 15128421 | 1.018886389 | 4 | amp |
| TCGA-04-1336 | 16 | 15129822 | 15135011 | 1.358543049 | 5 | amp |
| TCGA-04-1336 | 16 | 15135416 | 15609336 | 0.773832883 | 3 | amp |

| | | | | | | |
|---|---|---|---|---|---|---|
| TCGA-04-1336 | 16 | 15661816 | 15815504 | 0.942066555 | 4 | amp |
| TCGA-04-1336 | 16 | 15817961 | 18535273 | 0.746994391 | 3 | amp |
| TCGA-04-1336 | 16 | 18535782 | 18806964 | 0.920063361 | 4 | amp |
| TCGA-04-1336 | 16 | 18809156 | 18847574 | 1.162761149 | 5 | amp |
| TCGA-04-1336 | 16 | 18847647 | 20844504 | 0.88696754 | 4 | amp |
| TCGA-04-1336 | 16 | 20851038 | 21049312 | 0.8027174 | 3 | amp |
| TCGA-04-1336 | 16 | 21051129 | 21063209 | 1.436988748 | 5 | amp |
| TCGA-04-1336 | 16 | 21065737 | 21209188 | 0.75542853 | 3 | amp |
| TCGA-04-1336 | 16 | 21210081 | 23464350 | 0.919029709 | 4 | amp |
| TCGA-04-1336 | 16 | 23478905 | 23691608 | 0.772682509 | 3 | amp |
| TCGA-04-1336 | 16 | 23692156 | 27752281 | 0.91357063 | 4 | amp |
| TCGA-04-1336 | 16 | 27760815 | 27788359 | 1.242288084 | 5 | amp |
| TCGA-04-1336 | 16 | 27788928 | 33781527 | 0.919592515 | 4 | amp |
| TCGA-04-1336 | 16 | 33783360 | 46633928 | 0.724490445 | 3 | amp |
| TCGA-04-1336 | 17 | 34493274 | 34851277 | 0.728893377 | 3 | amp |
| TCGA-04-1336 | 17 | 36285520 | 36347105 | 0.798360407 | 3 | amp |
| TCGA-04-1336 | 17 | 47075044 | 56440983 | 0.891121364 | 4 | amp |
| TCGA-04-1336 | 17 | 56448182 | 57279017 | 0.794217572 | 3 | amp |
| TCGA-04-1336 | 17 | 57287361 | 60505233 | 0.86497091 | 4 | amp |
| TCGA-04-1336 | 17 | 60512562 | 61884074 | 0.821426707 | 3 | amp |
| TCGA-04-1336 | 17 | 61885051 | 73259624 | 0.880963091 | 4 | amp |
| TCGA-04-1336 | 17 | 73261773 | 74063414 | 0.821450116 | 3 | amp |
| TCGA-04-1336 | 17 | 74066431 | 81188237 | 0.873271241 | 4 | amp |
| TCGA-04-1336 | 18 | 47273 | 39542645 | 0.933568133 | 4 | amp |
| TCGA-04-1336 | 19 | 5776098 | 6467057 | 0.922743681 | 4 | amp |
| TCGA-04-1336 | 19 | 6467461 | 6828928 | 1.087671809 | 5 | amp |
| TCGA-04-1336 | 19 | 6829712 | 7570517 | 0.857043583 | 4 | amp |
| TCGA-04-1336 | 19 | 7570800 | 7742074 | 1.117010222 | 5 | amp |
| TCGA-04-1336 | 19 | 7742477 | 8539149 | 1.006608066 | 4 | amp |
| TCGA-04-1336 | 19 | 8548008 | 8604931 | 1.144017242 | 5 | amp |
| TCGA-04-1336 | 19 | 8610486 | 8620731 | 0.745512803 | 3 | amp |
| TCGA-04-1336 | 19 | 8645741 | 8933379 | 1.149172244 | 5 | amp |
| TCGA-04-1336 | 19 | 8953316 | 9028429 | 0.682973127 | 3 | amp |
| TCGA-04-1336 | 19 | 9033204 | 10169711 | 0.880974671 | 4 | amp |
| TCGA-04-1336 | 19 | 10197546 | 10266634 | 1.16260872 | 5 | amp |
| TCGA-04-1336 | 19 | 10334480 | 10685190 | 1.108773238 | 5 | amp |
| TCGA-04-1336 | 19 | 10685522 | 10782242 | 0.918686298 | 4 | amp |
| TCGA-04-1336 | 19 | 10787762 | 11221505 | 1.076134664 | 5 | amp |
| TCGA-04-1336 | 19 | 11238632 | 12577720 | 0.94347139 | 4 | amp |
| TCGA-04-1336 | 19 | 12595379 | 12693753 | 0.744647232 | 3 | amp |
| TCGA-04-1336 | 19 | 12694186 | 14694267 | 0.89072153 | 4 | amp |
| TCGA-04-1336 | 19 | 14698404 | 14992167 | 0.724115396 | 3 | amp |
| TCGA-04-1336 | 19 | 15052210 | 15784558 | 0.912091443 | 4 | amp |
| TCGA-04-1336 | 19 | 15789011 | 15807340 | 0.746290783 | 3 | amp |
| TCGA-04-1336 | 19 | 15807656 | 33430836 | 0.874512512 | 4 | amp |
| TCGA-04-1336 | 19 | 33439162 | 33655196 | 0.716675772 | 3 | amp |
| TCGA-04-1336 | 19 | 33663239 | 39735155 | 0.906341095 | 4 | amp |
| TCGA-04-1336 | 19 | 39735397 | 39789213 | 1.142875607 | 5 | amp |
| TCGA-04-1336 | 19 | 39860289 | 39936627 | 1.085652834 | 5 | amp |
| TCGA-04-1336 | 19 | 39957074 | 39967087 | 1.203368245 | 5 | amp |

| | | | | | | |
|---|---|---|---|---|---|---|
| TCGA-04-1336 | 19 | 39971122 | 41727992 | 0.934140858 | 4 | amp |
| TCGA-04-1336 | 19 | 41765423 | 42947004 | 0.926068795 | 4 | amp |
| TCGA-04-1336 | 19 | 42989178 | 43026395 | 0.690380132 | 3 | amp |
| TCGA-04-1336 | 19 | 43031132 | 44936588 | 0.891049383 | 4 | amp |
| TCGA-04-1336 | 19 | 44946715 | 44983682 | 1.343812841 | 5 | amp |
| TCGA-04-1336 | 19 | 44988518 | 48389566 | 0.892392666 | 4 | amp |
| TCGA-04-1336 | 19 | 48609722 | 48893790 | 0.851198458 | 4 | amp |
| TCGA-04-1336 | 19 | 48907910 | 49245242 | 1.104988196 | 5 | amp |
| TCGA-04-1336 | 19 | 49253359 | 49363721 | 1.085044225 | 5 | amp |
| TCGA-04-1336 | 19 | 49364647 | 49422071 | 0.906383315 | 4 | amp |
| TCGA-04-1336 | 19 | 49422242 | 49477993 | 1.149947572 | 5 | amp |
| TCGA-04-1336 | 19 | 49490426 | 49714842 | 1.098617757 | 5 | amp |
| TCGA-04-1336 | 19 | 49838741 | 49940111 | 1.098974826 | 5 | amp |
| TCGA-04-1336 | 19 | 49949569 | 49954846 | 0.698946104 | 3 | amp |
| TCGA-04-1336 | 19 | 49962129 | 50212137 | 1.101616452 | 5 | amp |
| TCGA-04-1336 | 19 | 50213538 | 50216889 | 0.692407479 | 3 | amp |
| TCGA-04-1336 | 19 | 50242960 | 50504138 | 1.102826623 | 5 | amp |
| TCGA-04-1336 | 19 | 50510744 | 50578467 | 0.762880251 | 3 | amp |
| TCGA-04-1336 | 19 | 50655796 | 51331114 | 1.093967351 | 5 | amp |
| TCGA-04-1336 | 19 | 51376692 | 53747193 | 0.935935558 | 4 | amp |
| TCGA-04-1336 | 19 | 53754735 | 53819232 | 1.245181648 | 5 | amp |
| TCGA-04-1336 | 19 | 53848821 | 54396690 | 0.756941809 | 3 | amp |
| TCGA-04-1336 | 19 | 54401075 | 54410174 | 1.201614218 | 5 | amp |
| TCGA-04-1336 | 19 | 54416033 | 54567074 | 0.821787963 | 3 | amp |
| TCGA-04-1336 | 2 | 41527 | 25861982 | 1.159126525 | 5 | amp |
| TCGA-04-1336 | 2 | 25875435 | 26717995 | 1.010296515 | 4 | amp |
| TCGA-04-1336 | 2 | 26724588 | 27549770 | 1.142790506 | 5 | amp |
| TCGA-04-1336 | 2 | 27550037 | 38960728 | 1.050486358 | 4 | amp |
| TCGA-04-1336 | 2 | 38972211 | 38978444 | 1.531207724 | 6 | amp |
| TCGA-04-1336 | 2 | 39006076 | 46583485 | 1.052494673 | 4 | amp |
| TCGA-04-1336 | 2 | 46583814 | 61304353 | 1.138057043 | 5 | amp |
| TCGA-04-1336 | 2 | 61308555 | 62100439 | 1.021084095 | 4 | amp |
| TCGA-04-1336 | 2 | 62103179 | 64332019 | 1.19485103 | 5 | amp |
| TCGA-04-1336 | 2 | 64335348 | 68274493 | 1.499312789 | 6 | amp |
| TCGA-04-1336 | 2 | 68352426 | 73315887 | 1.198746176 | 5 | amp |
| TCGA-04-1336 | 2 | 73315954 | 73498056 | 1.091561041 | 4 | amp |
| TCGA-04-1336 | 2 | 73635692 | 97853009 | 0.874564526 | 3 | amp |
| TCGA-04-1336 | 2 | 97853042 | 98429216 | 1.0078094 | 4 | amp |
| TCGA-04-1336 | 2 | 98430398 | 108627362 | 0.88718829 | 3 | amp |
| TCGA-04-1336 | 2 | 112630187 | 176044925 | 0.863284902 | 3 | amp |
| TCGA-04-1336 | 20 | 68319 | 1301093 | 0.972049943 | 4 | amp |
| TCGA-04-1336 | 20 | 1352699 | 2560708 | 0.746815013 | 3 | amp |
| TCGA-04-1336 | 20 | 21689116 | 23335129 | 1.071249324 | 4 | amp |
| TCGA-04-1336 | 20 | 23344984 | 23358127 | 1.225118915 | 5 | amp |
| TCGA-04-1336 | 20 | 23360046 | 23546727 | 0.721847572 | 3 | amp |
| TCGA-04-1336 | 20 | 23548787 | 29847468 | 0.97936201 | 4 | amp |
| TCGA-04-1336 | 20 | 29890955 | 29993986 | 1.270395115 | 5 | amp |
| TCGA-04-1336 | 20 | 30028440 | 30253940 | 0.996152061 | 4 | amp |
| TCGA-04-1336 | 20 | 30309409 | 30385322 | 1.49434135 | 6 | amp |
| TCGA-04-1336 | 20 | 30386111 | 30459032 | 1.114128177 | 5 | amp |

| | | | | | | |
|---|---|---|---|---|---|---|
| TCGA-04-1336 | 20 | 30460756 | 30790031 | 0.911156425 | 4 | amp |
| TCGA-04-1336 | 20 | 30797814 | 30956931 | 0.685522927 | 3 | amp |
| TCGA-04-1336 | 20 | 31015870 | 31044239 | 1.158046435 | 5 | amp |
| TCGA-04-1336 | 20 | 31368077 | 32878460 | 0.919356671 | 4 | amp |
| TCGA-04-1336 | 20 | 44528232 | 45607332 | 0.687709146 | 3 | amp |
| TCGA-04-1336 | 20 | 57561115 | 61524357 | 0.955097302 | 4 | amp |
| TCGA-04-1336 | 20 | 61524981 | 61828132 | 1.124143822 | 5 | amp |
| TCGA-04-1336 | 20 | 61833588 | 62926333 | 0.776587539 | 3 | amp |
| TCGA-04-1336 | 21 | 9483321 | 15347588 | 0.837833965 | 3 | amp |
| TCGA-04-1336 | 21 | 15347838 | 47783861 | 0.977656962 | 4 | amp |
| TCGA-04-1336 | 21 | 47786446 | 48111215 | 1.073341859 | 5 | amp |
| TCGA-04-1336 | 22 | 16100468 | 16957524 | 0.715646739 | 3 | amp |
| TCGA-04-1336 | 22 | 29924026 | 29945148 | 1.032471987 | 4 | amp |
| TCGA-04-1336 | 22 | 30032652 | 30500467 | 0.944953331 | 4 | amp |
| TCGA-04-1336 | 22 | 30507734 | 30695546 | 0.694262714 | 3 | amp |
| TCGA-04-1336 | 3 | 47859469 | 48511244 | 0.97370933 | 4 | amp |
| TCGA-04-1336 | 3 | 62216881 | 98544244 | 0.800883669 | 4 | amp |
| TCGA-04-1336 | 3 | 98568253 | 126160818 | 1.205255949 | 5 | amp |
| TCGA-04-1336 | 3 | 126178447 | 128859360 | 0.958585269 | 4 | amp |
| TCGA-04-1336 | 3 | 128864569 | 165548842 | 1.2248513 | 5 | amp |
| TCGA-04-1336 | 3 | 166958523 | 183909096 | 1.643896588 | 6 | amp |
| TCGA-04-1336 | 3 | 183910372 | 184299415 | 1.417096593 | 5 | amp |
| TCGA-04-1336 | 3 | 184428553 | 197955154 | 1.88929864 | 6 | amp |
| TCGA-04-1336 | 4 | 53323 | 8621346 | 0.950064077 | 4 | amp |
| TCGA-04-1336 | 4 | 9024980 | 9370636 | 0.718473112 | 3 | amp |
| TCGA-04-1336 | 4 | 9385735 | 39184240 | 0.962987729 | 4 | amp |
| TCGA-04-1336 | 4 | 39188131 | 39478768 | 1.24671532 | 5 | amp |
| TCGA-04-1336 | 4 | 39501757 | 74608233 | 0.949919511 | 4 | amp |
| TCGA-04-1336 | 4 | 74702626 | 75041200 | 1.252952738 | 5 | amp |
| TCGA-04-1336 | 4 | 75065507 | 83778939 | 0.940646688 | 4 | amp |
| TCGA-04-1336 | 5 | 143116 | 256551 | 0.996646302 | 4 | amp |
| TCGA-04-1336 | 5 | 272796 | 423028 | 0.734977753 | 3 | amp |
| TCGA-04-1336 | 5 | 427706 | 31313612 | 0.9292338 | 4 | amp |
| TCGA-04-1336 | 5 | 31316255 | 41149643 | 0.880992106 | 3 | amp |
| TCGA-04-1336 | 5 | 41150021 | 52240878 | 0.948668258 | 4 | amp |
| TCGA-04-1336 | 5 | 86708432 | 89948391 | 0.930868278 | 4 | amp |
| TCGA-04-1336 | 5 | 89948977 | 90674611 | 1.105262496 | 5 | amp |
| TCGA-04-1336 | 5 | 90678589 | 122950152 | 0.988813231 | 4 | amp |
| TCGA-04-1336 | 5 | 123966326 | 131796419 | 0.859011304 | 3 | amp |
| TCGA-04-1336 | 5 | 131819554 | 132070213 | 0.949217716 | 4 | amp |
| TCGA-04-1336 | 5 | 132083952 | 139851934 | 0.830651188 | 3 | amp |
| TCGA-04-1336 | 5 | 139862148 | 140051346 | 0.955593404 | 4 | amp |
| TCGA-04-1336 | 5 | 140052764 | 140059480 | 1.350109794 | 5 | amp |
| TCGA-04-1336 | 5 | 140062624 | 142152440 | 0.99658139 | 4 | amp |
| TCGA-04-1336 | 5 | 142252952 | 145252543 | 0.856184102 | 3 | amp |
| TCGA-04-1336 | 5 | 145317410 | 145613169 | 1.02092233 | 4 | amp |
| TCGA-04-1336 | 5 | 145613229 | 149925080 | 0.866494889 | 3 | amp |
| TCGA-04-1336 | 6 | 105907 | 4119524 | 1.041181479 | 4 | amp |
| TCGA-04-1336 | 6 | 4122171 | 4735096 | 0.768413796 | 3 | amp |
| TCGA-04-1336 | 6 | 4891919 | 6197567 | 0.982562036 | 4 | amp |

| | | | | | | |
|---|---|---|---|---|---|---|
| TCGA-04-1336 | 6 | 6222214 | 7187771 | 0.825811847 | 3 | amp |
| TCGA-04-1336 | 6 | 7189323 | 7572307 | 0.994337562 | 4 | amp |
| TCGA-04-1336 | 6 | 7574281 | 7581854 | 0.755176762 | 3 | amp |
| TCGA-04-1336 | 6 | 7582859 | 10775746 | 1.010507984 | 4 | amp |
| TCGA-04-1336 | 6 | 10818023 | 17463325 | 0.984749125 | 4 | amp |
| TCGA-04-1336 | 6 | 17507321 | 17676056 | 0.845024807 | 3 | amp |
| TCGA-04-1336 | 6 | 17688617 | 18264251 | 0.963521161 | 4 | amp |
| TCGA-04-1336 | 6 | 18399727 | 20124854 | 0.817693091 | 3 | amp |
| TCGA-04-1336 | 6 | 20126689 | 24437548 | 1.011587336 | 4 | amp |
| TCGA-04-1336 | 6 | 24449981 | 25762295 | 0.968667732 | 4 | amp |
| TCGA-04-1336 | 6 | 25769195 | 25845796 | 0.816632854 | 3 | amp |
| TCGA-04-1336 | 6 | 25849586 | 26273633 | 1.033425247 | 4 | amp |
| TCGA-04-1336 | 6 | 26285361 | 26450407 | 0.790367175 | 3 | amp |
| TCGA-04-1336 | 6 | 26451868 | 28297509 | 1.027631923 | 4 | amp |
| TCGA-04-1336 | 6 | 28327294 | 28483536 | 1.230602472 | 5 | amp |
| TCGA-04-1336 | 6 | 28493743 | 28539785 | 0.71309899 | 3 | amp |
| TCGA-04-1336 | 6 | 28539812 | 29693125 | 0.991866717 | 4 | amp |
| TCGA-04-1336 | 6 | 29693161 | 30529935 | 1.152974212 | 5 | amp |
| TCGA-04-1336 | 6 | 30569320 | 30613924 | 1.14934591 | 5 | amp |
| TCGA-04-1336 | 6 | 30617265 | 30621202 | 0.886607625 | 4 | amp |
| TCGA-04-1336 | 6 | 30627204 | 30668445 | 1.095283918 | 4 | amp |
| TCGA-04-1336 | 6 | 30670324 | 30682009 | 0.799119233 | 3 | amp |
| TCGA-04-1336 | 6 | 30682764 | 31499021 | 1.11932014 | 5 | amp |
| TCGA-04-1336 | 6 | 31499067 | 31560536 | 1.054265752 | 4 | amp |
| TCGA-04-1336 | 6 | 31590532 | 31617465 | 1.181601754 | 5 | amp |
| TCGA-04-1336 | 6 | 31619396 | 31683459 | 0.994674778 | 4 | amp |
| TCGA-04-1336 | 6 | 31685319 | 31702045 | 1.274514957 | 5 | amp |
| TCGA-04-1336 | 6 | 31703967 | 31729985 | 0.788505323 | 3 | amp |
| TCGA-04-1336 | 6 | 31730162 | 31838826 | 1.048277285 | 4 | amp |
| TCGA-04-1336 | 6 | 31839042 | 31857466 | 1.133510287 | 5 | amp |
| TCGA-04-1336 | 6 | 31860141 | 31907112 | 0.916173686 | 4 | amp |
| TCGA-04-1336 | 6 | 31921466 | 31948624 | 1.056142495 | 4 | amp |
| TCGA-04-1336 | 6 | 31948742 | 31959393 | 0.766065358 | 3 | amp |
| TCGA-04-1336 | 6 | 31959479 | 31981601 | 1.071766235 | 4 | amp |
| TCGA-04-1336 | 6 | 31992217 | 32084358 | 1.045377074 | 4 | amp |
| TCGA-04-1336 | 6 | 32093841 | 32147386 | 1.02938588 | 4 | amp |
| TCGA-04-1336 | 6 | 32147393 | 32151770 | 0.791842601 | 3 | amp |
| TCGA-04-1336 | 6 | 32151883 | 32172219 | 1.063635374 | 4 | amp |
| TCGA-04-1336 | 6 | 32178500 | 32427910 | 0.821776701 | 3 | amp |
| TCGA-04-1336 | 6 | 32487077 | 33036623 | 0.987708667 | 4 | amp |
| TCGA-04-1336 | 6 | 33036726 | 33144855 | 0.804861164 | 3 | amp |
| TCGA-04-1336 | 6 | 33151891 | 33167813 | 0.983127186 | 4 | amp |
| TCGA-04-1336 | 6 | 33171273 | 33177602 | 0.948490388 | 4 | amp |
| TCGA-04-1336 | 6 | 33177679 | 33219752 | 1.314317057 | 5 | amp |
| TCGA-04-1336 | 6 | 33243526 | 33248787 | 1.317708926 | 5 | amp |
| TCGA-04-1336 | 6 | 33254531 | 33381620 | 0.992175075 | 4 | amp |
| TCGA-04-1336 | 6 | 33385696 | 34725384 | 1.020174952 | 4 | amp |
| TCGA-04-1336 | 6 | 34725649 | 34835437 | 0.762149847 | 3 | amp |
| TCGA-04-1336 | 6 | 34838593 | 35196573 | 0.983261241 | 4 | amp |
| TCGA-04-1336 | 6 | 35210327 | 35912136 | 1.020836443 | 4 | amp |

| | | | | | | |
|---|---|---|---|---|---|---|
| TCGA-04-1336 | 6 | 35928762 | 36359658 | 0.955734823 | 4 | amp |
| TCGA-04-1336 | 6 | 36368195 | 36467828 | 0.806384616 | 3 | amp |
| TCGA-04-1336 | 6 | 36474300 | 38562091 | 0.980898862 | 4 | amp |
| TCGA-04-1336 | 6 | 38565657 | 38913423 | 0.866182503 | 3 | amp |
| TCGA-04-1336 | 6 | 38917136 | 42713830 | 0.974663034 | 4 | amp |
| TCGA-04-1336 | 6 | 42789720 | 42851390 | 0.724794622 | 3 | amp |
| TCGA-04-1336 | 6 | 42852299 | 42934202 | 0.978124894 | 4 | amp |
| TCGA-04-1336 | 6 | 42934208 | 42997017 | 0.810194006 | 3 | amp |
| TCGA-04-1336 | 6 | 43005407 | 43221125 | 0.985216551 | 4 | amp |
| TCGA-04-1336 | 6 | 43221225 | 43276568 | 1.096865888 | 5 | amp |
| TCGA-04-1336 | 6 | 43316032 | 44108859 | 0.983477095 | 4 | amp |
| TCGA-04-1336 | 6 | 44117588 | 44148426 | 0.972229069 | 4 | amp |
| TCGA-04-1336 | 6 | 44151410 | 44227048 | 1.140494785 | 5 | amp |
| TCGA-04-1336 | 6 | 44227719 | 47654808 | 0.981891465 | 4 | amp |
| TCGA-04-1336 | 6 | 47658229 | 47779498 | 0.799500253 | 3 | amp |
| TCGA-04-1336 | 6 | 47846015 | 52705627 | 0.99389848 | 4 | amp |
| TCGA-04-1336 | 6 | 52761544 | 52852218 | 0.788535405 | 3 | amp |
| TCGA-04-1336 | 6 | 52858937 | 74125952 | 0.995654577 | 4 | amp |
| TCGA-04-1336 | 6 | 74134945 | 74493608 | 0.833517918 | 3 | amp |
| TCGA-04-1336 | 6 | 74495083 | 76386972 | 1.018179423 | 4 | amp |
| TCGA-04-1336 | 6 | 76527202 | 83906012 | 0.987616825 | 4 | amp |
| TCGA-04-1336 | 6 | 83921607 | 84270684 | 0.836204632 | 3 | amp |
| TCGA-04-1336 | 6 | 84284724 | 86217791 | 0.980967297 | 4 | amp |
| TCGA-04-1336 | 6 | 86235809 | 88136423 | 0.986114856 | 4 | amp |
| TCGA-04-1336 | 6 | 88138322 | 88366720 | 0.868417451 | 3 | amp |
| TCGA-04-1336 | 6 | 88367597 | 89888815 | 0.973160898 | 4 | amp |
| TCGA-04-1336 | 6 | 89889958 | 90042984 | 0.849014475 | 3 | amp |
| TCGA-04-1336 | 6 | 90044965 | 90368662 | 1.018976834 | 4 | amp |
| TCGA-04-1336 | 6 | 90371103 | 90460281 | 0.873563663 | 3 | amp |
| TCGA-04-1336 | 6 | 90461105 | 105233206 | 0.993293116 | 4 | amp |
| TCGA-04-1336 | 6 | 105239333 | 105816958 | 0.851921616 | 3 | amp |
| TCGA-04-1336 | 6 | 105821228 | 106969366 | 1.009756756 | 4 | amp |
| TCGA-04-1336 | 6 | 106972971 | 107515101 | 0.874139541 | 3 | amp |
| TCGA-04-1336 | 6 | 107531558 | 109714144 | 0.957624325 | 4 | amp |
| TCGA-04-1336 | 6 | 109721185 | 109757414 | 0.714764451 | 3 | amp |
| TCGA-04-1336 | 6 | 109762259 | 111737700 | 0.986845391 | 4 | amp |
| TCGA-04-1336 | 6 | 111880530 | 112375617 | 0.809200889 | 3 | amp |
| TCGA-04-1336 | 6 | 112381186 | 117240499 | 0.992506121 | 4 | amp |
| TCGA-04-1336 | 6 | 117241424 | 117622338 | 0.767235805 | 3 | amp |
| TCGA-04-1336 | 6 | 117629903 | 118015353 | 1.028429932 | 4 | amp |
| TCGA-04-1336 | 6 | 118024757 | 118791813 | 0.799541795 | 3 | amp |
| TCGA-04-1336 | 6 | 118800998 | 129807812 | 0.977832828 | 4 | amp |
| TCGA-04-1336 | 6 | 129812983 | 131191284 | 0.878375866 | 3 | amp |
| TCGA-04-1336 | 6 | 131199196 | 131230041 | 1.043349261 | 4 | amp |
| TCGA-04-1336 | 6 | 131247706 | 131897898 | 0.823080225 | 3 | amp |
| TCGA-04-1336 | 6 | 131900217 | 131979571 | 0.983574861 | 4 | amp |
| TCGA-04-1336 | 6 | 131992367 | 132022554 | 0.829978287 | 3 | amp |
| TCGA-04-1336 | 6 | 132029910 | 135290485 | 0.973515115 | 4 | amp |
| TCGA-04-1336 | 6 | 135299780 | 135811942 | 1.204369169 | 5 | amp |
| TCGA-04-1336 | 6 | 136173056 | 136878985 | 1.031759236 | 4 | amp |

| | | | | | | |
|---|---|---|---|---|---|---|
| TCGA-04-1336 | 6 | 136879915 | 137041777 | 0.853377385 | 3 | amp |
| TCGA-04-1336 | 6 | 137112801 | 137219389 | 1.061052321 | 4 | amp |
| TCGA-04-1336 | 6 | 137234569 | 137528216 | 0.83494971 | 3 | amp |
| TCGA-04-1336 | 6 | 137814427 | 139113972 | 1.048812493 | 4 | amp |
| TCGA-04-1336 | 6 | 139134384 | 139202320 | 0.720202975 | 3 | amp |
| TCGA-04-1336 | 6 | 139203814 | 149838597 | 0.992545092 | 4 | amp |
| TCGA-04-1336 | 6 | 149842191 | 150322732 | 0.865699816 | 3 | amp |
| TCGA-04-1336 | 6 | 150341260 | 150569978 | 1.043363796 | 4 | amp |
| TCGA-04-1336 | 6 | 150690106 | 151144860 | 0.816695841 | 3 | amp |
| TCGA-04-1336 | 6 | 151151689 | 152708541 | 0.963741569 | 4 | amp |
| TCGA-04-1336 | 6 | 152711380 | 152749009 | 0.825441804 | 3 | amp |
| TCGA-04-1336 | 6 | 152749283 | 160450694 | 1.009140878 | 4 | amp |
| TCGA-04-1336 | 6 | 160467453 | 160864807 | 1.021017297 | 4 | amp |
| TCGA-04-1336 | 6 | 160868740 | 161455505 | 0.878237569 | 3 | amp |
| TCGA-04-1336 | 6 | 161469239 | 171055029 | 1.009103408 | 4 | amp |
| TCGA-04-1336 | 7 | 8043493 | 8198339 | 0.860590188 | 3 | amp |
| TCGA-04-1336 | 7 | 8257913 | 24757052 | 0.950028962 | 4 | amp |
| TCGA-04-1336 | 7 | 24758631 | 24910467 | 0.754763966 | 3 | amp |
| TCGA-04-1336 | 7 | 24911327 | 31014133 | 0.986754753 | 4 | amp |
| TCGA-04-1336 | 7 | 31014560 | 31125110 | 0.743325405 | 3 | amp |
| TCGA-04-1336 | 7 | 31125954 | 37954074 | 0.953265388 | 4 | amp |
| TCGA-04-1336 | 7 | 37988425 | 38398618 | 0.83095669 | 3 | amp |
| TCGA-04-1336 | 7 | 38402439 | 39612330 | 0.974507257 | 4 | amp |
| TCGA-04-1336 | 7 | 39726233 | 41730187 | 0.82756811 | 3 | amp |
| TCGA-04-1336 | 7 | 41739538 | 44612377 | 0.967874003 | 4 | amp |
| TCGA-04-1336 | 7 | 44612418 | 44715778 | 0.727859662 | 3 | amp |
| TCGA-04-1336 | 7 | 44721262 | 50672100 | 0.969201729 | 4 | amp |
| TCGA-04-1336 | 7 | 50672894 | 53855041 | 0.888252122 | 3 | amp |
| TCGA-04-1336 | 7 | 53930032 | 55236278 | 1.085131025 | 4 | amp |
| TCGA-04-1336 | 7 | 55237908 | 56088955 | 0.836859062 | 3 | amp |
| TCGA-04-1336 | 7 | 56120086 | 56358918 | 0.964166877 | 4 | amp |
| TCGA-04-1336 | 7 | 56428141 | 66563736 | 0.870568541 | 3 | amp |
| TCGA-04-1336 | 7 | 66582417 | 70255575 | 0.970621688 | 4 | amp |
| TCGA-04-1336 | 7 | 70800477 | 72286069 | 0.794493839 | 3 | amp |
| TCGA-04-1336 | 7 | 72296743 | 72442156 | 1.039861145 | 4 | amp |
| TCGA-04-1336 | 7 | 72468689 | 72609768 | 0.845703912 | 3 | amp |
| TCGA-04-1336 | 7 | 72609890 | 72649883 | 0.99023448 | 4 | amp |
| TCGA-04-1336 | 7 | 72707482 | 74004278 | 0.955451678 | 4 | amp |
| TCGA-04-1336 | 7 | 74005099 | 74301318 | 0.828422792 | 3 | amp |
| TCGA-04-1336 | 7 | 74303765 | 74474610 | 0.949981978 | 4 | amp |
| TCGA-04-1336 | 7 | 74573664 | 75684351 | 0.868260741 | 3 | amp |
| TCGA-04-1336 | 7 | 75686679 | 76028135 | 1.004764238 | 4 | amp |
| TCGA-04-1336 | 7 | 76029545 | 77423729 | 0.873421774 | 3 | amp |
| TCGA-04-1336 | 7 | 77469534 | 99008822 | 0.959549018 | 4 | amp |
| TCGA-04-1336 | 7 | 99013701 | 99270312 | 0.850558444 | 3 | amp |
| TCGA-04-1336 | 7 | 99272091 | 99315265 | 1.042493727 | 4 | amp |
| TCGA-04-1336 | 7 | 99317925 | 99675016 | 0.856350429 | 3 | amp |
| TCGA-04-1336 | 7 | 99686825 | 99707919 | 0.953509984 | 4 | amp |
| TCGA-04-1336 | 7 | 99708702 | 100193383 | 0.838292555 | 3 | amp |
| TCGA-04-1336 | 7 | 100197618 | 100275941 | 0.989573433 | 4 | amp |

| | | | | | | |
|---|---|---|---|---|---|---|
| TCGA-04-1336 | 7 | 100276008 | 100452428 | 0.812441713 | 3 | amp |
| TCGA-04-1336 | 7 | 100453243 | 100485773 | 1.025737586 | 4 | amp |
| TCGA-04-1336 | 7 | 100485790 | 100777194 | 0.815562842 | 3 | amp |
| TCGA-04-1336 | 7 | 100778734 | 101267557 | 0.971068257 | 4 | amp |
| TCGA-04-1336 | 7 | 101559359 | 102769256 | 0.880227099 | 3 | amp |
| TCGA-04-1336 | 7 | 102815782 | 102953590 | 1.080918812 | 4 | amp |
| TCGA-04-1336 | 7 | 102956204 | 103062005 | 0.864757029 | 3 | amp |
| TCGA-04-1336 | 7 | 103113246 | 104704032 | 0.97834561 | 4 | amp |
| TCGA-04-1336 | 7 | 104707153 | 107338581 | 0.865046722 | 3 | amp |
| TCGA-04-1336 | 7 | 107340483 | 107557932 | 0.998543518 | 4 | amp |
| TCGA-04-1336 | 7 | 107558317 | 107626833 | 0.810469766 | 3 | amp |
| TCGA-04-1336 | 7 | 107635278 | 128120785 | 0.966580574 | 4 | amp |
| TCGA-04-1336 | 7 | 128128124 | 128415848 | 0.811710694 | 3 | amp |
| TCGA-04-1336 | 7 | 128432413 | 128607449 | 0.970906665 | 4 | amp |
| TCGA-04-1336 | 7 | 128610165 | 129769466 | 0.856983807 | 3 | amp |
| TCGA-04-1336 | 7 | 129770361 | 130142582 | 0.930814071 | 4 | amp |
| TCGA-04-1336 | 7 | 130143745 | 132755005 | 0.870863314 | 3 | amp |
| TCGA-04-1336 | 7 | 132854110 | 133863367 | 1.009371414 | 4 | amp |
| TCGA-04-1336 | 7 | 133868425 | 134262556 | 0.79456331 | 3 | amp |
| TCGA-04-1336 | 7 | 134264219 | 137266694 | 0.958641397 | 4 | amp |
| TCGA-04-1336 | 7 | 137269903 | 138978740 | 0.869513985 | 3 | amp |
| TCGA-04-1336 | 7 | 138982497 | 139791920 | 0.967513737 | 4 | amp |
| TCGA-04-1336 | 7 | 139793836 | 140379138 | 0.83398158 | 3 | amp |
| TCGA-04-1336 | 7 | 140380799 | 141366275 | 0.962454007 | 4 | amp |
| TCGA-04-1336 | 7 | 141385236 | 142627552 | 0.856001405 | 3 | amp |
| TCGA-04-1336 | 7 | 142630342 | 142643408 | 0.995581273 | 4 | amp |
| TCGA-04-1336 | 7 | 142723272 | 142920197 | 0.836860023 | 3 | amp |
| TCGA-04-1336 | 7 | 142960552 | 143002218 | 1.022568047 | 4 | amp |
| TCGA-04-1336 | 7 | 143013245 | 143054564 | 0.77702785 | 3 | amp |
| TCGA-04-1336 | 7 | 143055898 | 143271277 | 0.992050771 | 4 | amp |
| TCGA-04-1336 | 7 | 143295091 | 143827151 | 0.868148197 | 3 | amp |
| TCGA-04-1336 | 7 | 143880563 | 143966376 | 1.533340391 | 6 | amp |
| TCGA-04-1336 | 7 | 143969513 | 148506501 | 0.945133363 | 4 | amp |
| TCGA-04-1336 | 7 | 148507405 | 149509221 | 0.880887483 | 3 | amp |
| TCGA-04-1336 | 7 | 149509628 | 158935247 | 0.977701328 | 4 | amp |
| TCGA-04-1336 | 8 | 116074 | 90802655 | 0.956666965 | 4 | amp |
| TCGA-04-1336 | 8 | 90921855 | 100026209 | 1.294244087 | 5 | amp |
| TCGA-04-1336 | 8 | 100050612 | 103283401 | 1.651023796 | 6 | amp |
| TCGA-04-1336 | 8 | 103284698 | 103300498 | 1.162692186 | 5 | amp |
| TCGA-04-1336 | 8 | 103301622 | 136619298 | 1.716584636 | 6 | amp |
| TCGA-04-1336 | 8 | 136657270 | 139856423 | 1.425608424 | 5 | amp |
| TCGA-04-1336 | 8 | 140630441 | 146279593 | 1.82322171 | 6 | amp |
| TCGA-04-1336 | 9 | 17322 | 334424 | 0.934639037 | 4 | amp |
| TCGA-04-1336 | 9 | 336529 | 422151 | 0.676373402 | 3 | amp |
| TCGA-04-1336 | 9 | 426872 | 33948598 | 0.879140647 | 4 | amp |
| TCGA-04-1336 | 9 | 33953227 | 34343459 | 0.750153369 | 3 | amp |
| TCGA-04-1336 | 9 | 34370788 | 35381214 | 0.925897316 | 4 | amp |
| TCGA-04-1336 | 9 | 35381514 | 35556494 | 0.69807447 | 3 | amp |
| TCGA-04-1336 | 9 | 35557888 | 35607424 | 1.157143326 | 5 | amp |
| TCGA-04-1336 | 9 | 35607533 | 35720245 | 0.941607624 | 4 | amp |

| | | | | | | |
|---|---|---|---|---|---|---|
| TCGA-04-1336 | 9 | 35720377 | 35741897 | 0.738676632 | 3 | amp |
| TCGA-04-1336 | 9 | 35744291 | 35805707 | 0.905925263 | 4 | amp |
| TCGA-04-1336 | 9 | 35805754 | 35811468 | 0.752247153 | 3 | amp |
| TCGA-04-1336 | 9 | 35811609 | 35971578 | 1.135817506 | 5 | amp |
| TCGA-04-1336 | 9 | 35971750 | 39891109 | 0.901543876 | 4 | amp |
| TCGA-04-1336 | 9 | 134103512 | 134396880 | 0.797003527 | 3 | amp |
| TCGA-04-1336 | 9 | 134397372 | 135152552 | 0.863335841 | 4 | amp |
| TCGA-04-1336 | 9 | 135153430 | 135797402 | 0.793780259 | 3 | amp |
| TCGA-04-1336 | 9 | 135798686 | 137630682 | 0.855469399 | 4 | amp |
| TCGA-04-1336 | 9 | 137648577 | 141071671 | 0.718212694 | 3 | amp |
| TCGA-04-1336 | X | 49189195 | 49242970 | 0.803255756 | 3 | amp |
| TCGA-04-1336 | X | 62519278 | 67943994 | 0.879469714 | 4 | amp |
| TCGA-04-1336 | X | 68004854 | 68749041 | 1.173759054 | 5 | amp |
| TCGA-04-1336 | X | 68749401 | 69455679 | 0.730133047 | 3 | amp |
| TCGA-04-1336 | X | 69455893 | 70517796 | 0.883509307 | 4 | amp |
| TCGA-04-1336 | X | 70518278 | 70618624 | 0.756654465 | 3 | amp |
| TCGA-04-1336 | X | 70621363 | 77227318 | 0.926964854 | 4 | amp |
| TCGA-04-1336 | X | 77243682 | 77289330 | 0.764354471 | 3 | amp |
| TCGA-04-1336 | X | 77294286 | 83357245 | 0.946564433 | 4 | amp |
| TCGA-04-1336 | X | 83359465 | 83375083 | 0.717756687 | 3 | amp |
| TCGA-04-1336 | X | 120064084 | 120114215 | 0.779899199 | 3 | amp |
| TCGA-04-1343 | 1 | 16834 | 881973 | 1.62940566 | 4 | amp |
| TCGA-04-1343 | 1 | 891414 | 1686179 | 2.689248148 | 5 | amp |
| TCGA-04-1343 | 1 | 1686773 | 6142354 | 1.645282598 | 4 | amp |
| TCGA-04-1343 | 1 | 6145225 | 6295049 | 2.909443302 | 5 | amp |
| TCGA-04-1343 | 1 | 6304375 | 6527687 | 1.73473462 | 4 | amp |
| TCGA-04-1343 | 1 | 6529209 | 6727879 | 3.357690037 | 5 | amp |
| TCGA-04-1343 | 1 | 6738400 | 10093800 | 1.490901593 | 4 | amp |
| TCGA-04-1343 | 1 | 10132058 | 10468222 | 0.963569366 | 3 | amp |
| TCGA-04-1343 | 1 | 10471391 | 10527437 | 2.318009253 | 5 | amp |
| TCGA-04-1343 | 1 | 10529194 | 11303383 | 1.553340973 | 4 | amp |
| TCGA-04-1343 | 1 | 11307675 | 11579605 | 2.815901246 | 5 | amp |
| TCGA-04-1343 | 1 | 11579706 | 11918578 | 1.738715335 | 4 | amp |
| TCGA-04-1343 | 1 | 11918674 | 12067327 | 2.344056005 | 5 | amp |
| TCGA-04-1343 | 1 | 12069565 | 12294431 | 1.54659267 | 4 | amp |
| TCGA-04-1343 | 1 | 12302518 | 13331003 | 1.068376392 | 3 | amp |
| TCGA-04-1343 | 1 | 13331341 | 16343755 | 1.438151888 | 4 | amp |
| TCGA-04-1343 | 1 | 16350238 | 16456937 | 2.362525857 | 5 | amp |
| TCGA-04-1343 | 1 | 16458170 | 17087643 | 1.535822863 | 4 | amp |
| TCGA-04-1343 | 1 | 17249075 | 17318683 | 4.034387259 | 5 | amp |
| TCGA-04-1343 | 1 | 17318697 | 17380538 | 1.646056277 | 4 | amp |
| TCGA-04-1343 | 1 | 17395505 | 19514044 | 1.061966107 | 3 | amp |
| TCGA-04-1343 | 1 | 19518626 | 19582553 | 1.61435668 | 4 | amp |
| TCGA-04-1343 | 1 | 19583508 | 19632696 | 3.452995266 | 5 | amp |
| TCGA-04-1343 | 1 | 19633455 | 22084321 | 1.332855788 | 4 | amp |
| TCGA-04-1343 | 1 | 22138896 | 22217192 | 2.859892595 | 5 | amp |
| TCGA-04-1343 | 1 | 22222338 | 23660127 | 1.076690871 | 3 | amp |
| TCGA-04-1343 | 1 | 23664240 | 24122757 | 1.81900364 | 4 | amp |
| TCGA-04-1343 | 1 | 24122960 | 24413345 | 2.865394833 | 5 | amp |
| TCGA-04-1343 | 1 | 24416521 | 24666264 | 1.455827645 | 4 | amp |

| | | | | | | |
|---|---|---|---|---|---|---|
| TCGA-04-1343 | 1 | 24668533 | 25824975 | 1.105942847 | 3 | amp |
| TCGA-04-1343 | 1 | 25880363 | 26135679 | 1.579788817 | 4 | amp |
| TCGA-04-1343 | 1 | 26136092 | 26231220 | 2.305014932 | 5 | amp |
| TCGA-04-1343 | 1 | 26288436 | 26510801 | 1.501018004 | 4 | amp |
| TCGA-04-1343 | 1 | 26513635 | 26520388 | 2.552076342 | 5 | amp |
| TCGA-04-1343 | 1 | 26524165 | 27190496 | 1.534905839 | 4 | amp |
| TCGA-04-1343 | 1 | 27206143 | 27272706 | 2.718133011 | 5 | amp |
| TCGA-04-1343 | 1 | 27276540 | 27658636 | 1.628747381 | 4 | amp |
| TCGA-04-1343 | 1 | 27660406 | 27682613 | 2.601988098 | 5 | amp |
| TCGA-04-1343 | 1 | 27682874 | 27689546 | 1.644190655 | 4 | amp |
| TCGA-04-1343 | 1 | 27690639 | 27721338 | 3.20798064 | 5 | amp |
| TCGA-04-1343 | 1 | 27734641 | 27878513 | 1.486735716 | 4 | amp |
| TCGA-04-1343 | 1 | 27939378 | 27950434 | 2.99446185 | 5 | amp |
| TCGA-04-1343 | 1 | 27992878 | 28206599 | 1.79072946 | 4 | amp |
| TCGA-04-1343 | 1 | 28208477 | 28835391 | 1.037078803 | 3 | amp |
| TCGA-04-1343 | 1 | 28856324 | 28863454 | 3.249679582 | 5 | amp |
| TCGA-04-1343 | 1 | 28880140 | 32148628 | 1.322782545 | 4 | amp |
| TCGA-04-1343 | 1 | 32148731 | 32224996 | 2.451258874 | 5 | amp |
| TCGA-04-1343 | 1 | 32256187 | 32694432 | 1.625901812 | 4 | amp |
| TCGA-04-1343 | 1 | 32694682 | 32768337 | 2.519385843 | 5 | amp |
| TCGA-04-1343 | 1 | 32782174 | 33613352 | 1.778851476 | 4 | amp |
| TCGA-04-1343 | 1 | 33623851 | 33841213 | 2.613418836 | 5 | amp |
| TCGA-04-1343 | 1 | 33944827 | 35657155 | 1.340160719 | 4 | amp |
| TCGA-04-1343 | 1 | 35790923 | 35972774 | 0.881279445 | 3 | amp |
| TCGA-04-1343 | 1 | 36019901 | 36557442 | 1.386086546 | 4 | amp |
| TCGA-04-1343 | 1 | 36557482 | 36645913 | 2.939973979 | 5 | amp |
| TCGA-04-1343 | 1 | 36748112 | 38447501 | 1.645421739 | 4 | amp |
| TCGA-04-1343 | 1 | 38449826 | 38482151 | 3.043713166 | 5 | amp |
| TCGA-04-1343 | 1 | 38483330 | 39715790 | 1.52432418 | 4 | amp |
| TCGA-04-1343 | 1 | 39719948 | 39930835 | 0.982353046 | 3 | amp |
| TCGA-04-1343 | 1 | 39934225 | 40323138 | 1.483676885 | 4 | amp |
| TCGA-04-1343 | 1 | 40362952 | 40434448 | 2.927475882 | 5 | amp |
| TCGA-04-1343 | 1 | 40435118 | 40773461 | 1.243410415 | 4 | amp |
| TCGA-04-1343 | 1 | 40773800 | 40780101 | 2.504065263 | 5 | amp |
| TCGA-04-1343 | 1 | 40872354 | 43204001 | 1.368756605 | 4 | amp |
| TCGA-04-1343 | 1 | 43212502 | 43273187 | 4.543337896 | 5 | amp |
| TCGA-04-1343 | 1 | 43282098 | 43675755 | 1.453638098 | 4 | amp |
| TCGA-04-1343 | 1 | 43738363 | 43788417 | 2.68644059 | 5 | amp |
| TCGA-04-1343 | 1 | 43803468 | 43818495 | 1.788989764 | 4 | amp |
| TCGA-04-1343 | 1 | 43824826 | 43851952 | 3.26206544 | 5 | amp |
| TCGA-04-1343 | 1 | 43852240 | 43917992 | 1.412418268 | 4 | amp |
| TCGA-04-1343 | 1 | 44019085 | 44064607 | 2.620932389 | 5 | amp |
| TCGA-04-1343 | 1 | 44067694 | 44386268 | 1.513789721 | 4 | amp |
| TCGA-04-1343 | 1 | 44386406 | 44456164 | 2.970534438 | 5 | amp |
| TCGA-04-1343 | 1 | 44457821 | 44805011 | 1.760060901 | 4 | amp |
| TCGA-04-1343 | 1 | 44818499 | 45481209 | 2.376649477 | 5 | amp |
| TCGA-04-1343 | 1 | 45484076 | 45965302 | 1.69227614 | 4 | amp |
| TCGA-04-1343 | 1 | 45965954 | 46034694 | 2.391662035 | 5 | amp |
| TCGA-04-1343 | 1 | 46034767 | 47279335 | 1.587372399 | 4 | amp |
| TCGA-04-1343 | 1 | 47279507 | 52291066 | 1.059409041 | 3 | amp |

| | | | | | | |
|---|---|---|---|---|---|---|
| TCGA-04-1343 | 1 | 52293444 | 52850427 | 1.436317223 | 4 | amp |
| TCGA-04-1343 | 1 | 52850823 | 53516418 | 1.068298592 | 3 | amp |
| TCGA-04-1343 | 1 | 53540219 | 53932361 | 2.500994353 | 5 | amp |
| TCGA-04-1343 | 1 | 53995394 | 54682035 | 1.309181888 | 4 | amp |
| TCGA-04-1343 | 1 | 54683823 | 54709022 | 2.832766435 | 5 | amp |
| TCGA-04-1343 | 1 | 54867515 | 55537601 | 1.547536452 | 4 | amp |
| TCGA-04-1343 | 1 | 55537972 | 109566175 | 0.966510909 | 3 | amp |
| TCGA-04-1343 | 1 | 109607109 | 109815688 | 2.3855607 | 5 | amp |
| TCGA-04-1343 | 1 | 109815722 | 109968997 | 1.169222279 | 4 | amp |
| TCGA-04-1343 | 1 | 110018114 | 110051573 | 2.769536275 | 5 | amp |
| TCGA-04-1343 | 1 | 110085572 | 110199089 | 1.55597736 | 4 | amp |
| TCGA-04-1343 | 1 | 110199281 | 110203891 | 2.63494725 | 5 | amp |
| TCGA-04-1343 | 1 | 110207527 | 111825275 | 1.560293086 | 4 | amp |
| TCGA-04-1343 | 1 | 111825969 | 112251873 | 1.048804844 | 3 | amp |
| TCGA-04-1343 | 1 | 112269527 | 113202421 | 1.496132597 | 4 | amp |
| TCGA-04-1343 | 1 | 113209671 | 113269336 | 2.319498624 | 5 | amp |
| TCGA-04-1343 | 1 | 113456469 | 116667012 | 1.007808548 | 3 | amp |
| TCGA-04-1343 | 1 | 116670055 | 117131760 | 1.591223412 | 4 | amp |
| TCGA-04-1343 | 1 | 117142524 | 120572629 | 1.044382959 | 3 | amp |
| TCGA-04-1343 | 1 | 120839789 | 145539799 | 1.434716126 | 4 | amp |
| TCGA-04-1343 | 1 | 145541398 | 145609382 | 2.417012215 | 5 | amp |
| TCGA-04-1343 | 1 | 145646083 | 149876777 | 1.443753776 | 4 | amp |
| TCGA-04-1343 | 1 | 149877361 | 149885441 | 2.550409515 | 5 | amp |
| TCGA-04-1343 | 1 | 149895699 | 150199188 | 1.425057104 | 4 | amp |
| TCGA-04-1343 | 1 | 150201367 | 150239658 | 2.814062326 | 5 | amp |
| TCGA-04-1343 | 1 | 150239666 | 150464985 | 1.355592397 | 4 | amp |
| TCGA-04-1343 | 1 | 150468910 | 150550023 | 2.502662774 | 5 | amp |
| TCGA-04-1343 | 1 | 150550663 | 150933712 | 1.351837899 | 4 | amp |
| TCGA-04-1343 | 1 | 150934492 | 151112568 | 2.780502081 | 5 | amp |
| TCGA-04-1343 | 1 | 151131150 | 151735663 | 1.596777731 | 4 | amp |
| TCGA-04-1343 | 1 | 151739292 | 151820395 | 2.484605601 | 5 | amp |
| TCGA-04-1343 | 1 | 151820622 | 153431538 | 1.25406104 | 4 | amp |
| TCGA-04-1343 | 1 | 153507088 | 153661845 | 2.604242245 | 5 | amp |
| TCGA-04-1343 | 1 | 153661902 | 153747984 | 1.758814488 | 4 | amp |
| TCGA-04-1343 | 1 | 153748948 | 153782839 | 4.967261917 | 5 | amp |
| TCGA-04-1343 | 1 | 153784115 | 153800835 | 1.284917419 | 4 | amp |
| TCGA-04-1343 | 1 | 153902725 | 153954953 | 3.365822644 | 5 | amp |
| TCGA-04-1343 | 1 | 153954983 | 154248194 | 1.359406213 | 4 | amp |
| TCGA-04-1343 | 1 | 154284899 | 154313553 | 3.329705386 | 5 | amp |
| TCGA-04-1343 | 1 | 154314917 | 154437912 | 1.670248434 | 4 | amp |
| TCGA-04-1343 | 1 | 154456567 | 154574958 | 2.715189116 | 5 | amp |
| TCGA-04-1343 | 1 | 154575010 | 154920212 | 1.719343081 | 4 | amp |
| TCGA-04-1343 | 1 | 154920379 | 155182023 | 3.052174904 | 5 | amp |
| TCGA-04-1343 | 1 | 155182146 | 155221402 | 1.742393078 | 4 | amp |
| TCGA-04-1343 | 1 | 155223409 | 155282235 | 2.540690848 | 5 | amp |
| TCGA-04-1343 | 1 | 155287711 | 155921803 | 1.628480619 | 4 | amp |
| TCGA-04-1343 | 1 | 155921846 | 155939129 | 3.437008815 | 5 | amp |
| TCGA-04-1343 | 1 | 155979325 | 155990000 | 1.176327222 | 4 | amp |
| TCGA-04-1343 | 1 | 156006694 | 156438117 | 2.492832971 | 5 | amp |
| TCGA-04-1343 | 1 | 156438469 | 156542362 | 1.529793298 | 4 | amp |

| | | | | | | |
|---|---|---|---|---|---|---|
| TCGA-04-1343 | 1 | 156552131 | 156707934 | 2.570186486 | 5 | amp |
| TCGA-04-1343 | 1 | 156708092 | 156822011 | 1.767090975 | 4 | amp |
| TCGA-04-1343 | 1 | 156823489 | 156879908 | 2.871453506 | 5 | amp |
| TCGA-04-1343 | 1 | 156879948 | 156918320 | 1.900964112 | 4 | amp |
| TCGA-04-1343 | 1 | 156921307 | 159785508 | 1.04348503 | 3 | amp |
| TCGA-04-1343 | 1 | 159796618 | 159863152 | 1.492313825 | 4 | amp |
| TCGA-04-1343 | 1 | 159869826 | 159923964 | 2.550666234 | 5 | amp |
| TCGA-04-1343 | 1 | 159943661 | 160208586 | 1.41273023 | 4 | amp |
| TCGA-04-1343 | 1 | 160209408 | 160313000 | 0.965174029 | 3 | amp |
| TCGA-04-1343 | 1 | 160313168 | 161019526 | 1.356909512 | 4 | amp |
| TCGA-04-1343 | 1 | 161020957 | 161161380 | 2.607381727 | 5 | amp |
| TCGA-04-1343 | 1 | 161161792 | 161719943 | 1.415488299 | 4 | amp |
| TCGA-04-1343 | 1 | 161721424 | 166040038 | 1.026046804 | 3 | amp |
| TCGA-04-1343 | 1 | 166123877 | 166991118 | 1.74453834 | 4 | amp |
| TCGA-04-1343 | 1 | 167023516 | 167974038 | 1.092514018 | 3 | amp |
| TCGA-04-1343 | 1 | 167992195 | 168211818 | 1.699830808 | 4 | amp |
| TCGA-04-1343 | 1 | 168250309 | 180849442 | 1.014253942 | 3 | amp |
| TCGA-04-1343 | 1 | 180853111 | 182572478 | 1.392426514 | 4 | amp |
| TCGA-04-1343 | 1 | 182615810 | 200827214 | 0.9146058 | 3 | amp |
| TCGA-04-1343 | 1 | 200867388 | 205239024 | 1.456208385 | 4 | amp |
| TCGA-04-1343 | 1 | 205240195 | 205312784 | 2.388853423 | 5 | amp |
| TCGA-04-1343 | 1 | 205350773 | 207095240 | 1.550789621 | 4 | amp |
| TCGA-04-1343 | 1 | 207103650 | 225755118 | 1.019584462 | 3 | amp |
| TCGA-04-1343 | 1 | 225965618 | 227182722 | 1.360061838 | 4 | amp |
| TCGA-04-1343 | 1 | 227192603 | 227843562 | 0.867750772 | 3 | amp |
| TCGA-04-1343 | 1 | 227922314 | 228873508 | 1.748765105 | 4 | amp |
| TCGA-04-1343 | 1 | 228878989 | 249108848 | 1.007327934 | 3 | amp |
| TCGA-04-1343 | 1 | 249110646 | 249231325 | 2.303590295 | 5 | amp |
| TCGA-04-1343 | 10 | 92880 | 298468 | 1.622784617 | 4 | amp |
| TCGA-04-1343 | 10 | 323180 | 866822 | 2.376017387 | 5 | amp |
| TCGA-04-1343 | 10 | 870967 | 1051862 | 1.747289032 | 4 | amp |
| TCGA-04-1343 | 10 | 1052952 | 5011947 | 2.408696151 | 5 | amp |
| TCGA-04-1343 | 10 | 5014387 | 5838835 | 1.665653241 | 4 | amp |
| TCGA-04-1343 | 10 | 5842554 | 5957569 | 2.273314119 | 5 | amp |
| TCGA-04-1343 | 10 | 5958187 | 5995173 | 1.684025157 | 4 | amp |
| TCGA-04-1343 | 10 | 5998319 | 6274941 | 2.915565775 | 5 | amp |
| TCGA-04-1343 | 10 | 6470096 | 6557158 | 1.310230186 | 4 | amp |
| TCGA-04-1343 | 10 | 7205681 | 7247893 | 2.36336404 | 5 | amp |
| TCGA-04-1343 | 10 | 7262285 | 7409910 | 1.680599727 | 4 | amp |
| TCGA-04-1343 | 10 | 7412229 | 7659280 | 2.578374374 | 5 | amp |
| TCGA-04-1343 | 10 | 7679105 | 7811363 | 1.560011376 | 4 | amp |
| TCGA-04-1343 | 10 | 7816733 | 7866581 | 2.289889887 | 5 | amp |
| TCGA-04-1343 | 10 | 8005848 | 8019304 | 1.535431515 | 4 | amp |
| TCGA-04-1343 | 10 | 8050984 | 11356281 | 2.468469831 | 5 | amp |
| TCGA-04-1343 | 10 | 11363130 | 11574263 | 1.649368218 | 4 | amp |
| TCGA-04-1343 | 10 | 11789288 | 11963120 | 2.311296289 | 5 | amp |
| TCGA-04-1343 | 10 | 11963211 | 12143230 | 1.437383612 | 4 | amp |
| TCGA-04-1343 | 10 | 12148199 | 12185214 | 2.526534796 | 5 | amp |
| TCGA-04-1343 | 10 | 12191539 | 12867754 | 1.769996395 | 4 | amp |
| TCGA-04-1343 | 10 | 12870706 | 13154663 | 2.643691848 | 5 | amp |

| | | | | | | |
|---|---|---|---|---|---|---|
| TCGA-04-1343 | 10 | 13158243 | 13175601 | 1.641845123 | 4 | amp |
| TCGA-04-1343 | 10 | 13178685 | 13217715 | 2.387328144 | 5 | amp |
| TCGA-04-1343 | 10 | 13222404 | 13251328 | 1.800135209 | 4 | amp |
| TCGA-04-1343 | 10 | 13264091 | 13494718 | 2.520923574 | 5 | amp |
| TCGA-04-1343 | 10 | 13522891 | 13672359 | 1.775063129 | 4 | amp |
| TCGA-04-1343 | 10 | 13693879 | 13779946 | 2.382041619 | 5 | amp |
| TCGA-04-1343 | 10 | 13782132 | 14816696 | 1.593753396 | 4 | amp |
| TCGA-04-1343 | 10 | 14861917 | 14897955 | 2.342327557 | 5 | amp |
| TCGA-04-1343 | 10 | 14909037 | 17216664 | 1.565981618 | 4 | amp |
| TCGA-04-1343 | 10 | 17272588 | 17279309 | 3.179036386 | 5 | amp |
| TCGA-04-1343 | 10 | 17362870 | 22608957 | 1.523901077 | 4 | amp |
| TCGA-04-1343 | 10 | 22615374 | 22618486 | 2.30540195 | 5 | amp |
| TCGA-04-1343 | 10 | 22653744 | 35855151 | 1.543197155 | 4 | amp |
| TCGA-04-1343 | 10 | 35857889 | 39011280 | 2.253434792 | 5 | amp |
| TCGA-04-1343 | 10 | 42680923 | 47901471 | 0.993176774 | 3 | amp |
| TCGA-04-1343 | 10 | 70700762 | 74100955 | 1.083230769 | 3 | amp |
| TCGA-04-1343 | 10 | 75391653 | 75485818 | 1.052556128 | 3 | amp |
| TCGA-04-1343 | 10 | 75487116 | 75520658 | 1.789132113 | 4 | amp |
| TCGA-04-1343 | 10 | 75523243 | 75530571 | 2.288608584 | 5 | amp |
| TCGA-04-1343 | 10 | 75530662 | 75561295 | 1.788484666 | 4 | amp |
| TCGA-04-1343 | 10 | 75562121 | 75608881 | 2.375412299 | 5 | amp |
| TCGA-04-1343 | 10 | 75608994 | 75676354 | 1.426786934 | 4 | amp |
| TCGA-04-1343 | 10 | 75802815 | 89272989 | 1.011525859 | 3 | amp |
| TCGA-04-1343 | 10 | 98469261 | 102035294 | 1.056179771 | 3 | amp |
| TCGA-04-1343 | 10 | 102039842 | 102741015 | 1.363383452 | 4 | amp |
| TCGA-04-1343 | 10 | 102741169 | 102824689 | 2.419011132 | 5 | amp |
| TCGA-04-1343 | 10 | 102849357 | 104140472 | 1.110041595 | 3 | amp |
| TCGA-04-1343 | 10 | 104140797 | 104240730 | 1.721062147 | 4 | amp |
| TCGA-04-1343 | 10 | 104240848 | 105484133 | 1.008728702 | 3 | amp |
| TCGA-04-1343 | 10 | 118353780 | 121652495 | 1.111762442 | 3 | amp |
| TCGA-04-1343 | 10 | 124591759 | 133787491 | 0.998867657 | 3 | amp |
| TCGA-04-1343 | 10 | 133930575 | 135516111 | 1.453411598 | 4 | amp |
| TCGA-04-1343 | 11 | 86637 | 7060141 | 1.510800584 | 4 | amp |
| TCGA-04-1343 | 11 | 7060890 | 34489948 | 1.041540289 | 3 | amp |
| TCGA-04-1343 | 11 | 34492456 | 134212866 | 1.71384246 | 4 | amp |
| TCGA-04-1343 | 11 | 134214224 | 134257557 | 4.237973715 | 5 | amp |
| TCGA-04-1343 | 12 | 73256 | 176637 | 2.807232157 | 5 | amp |
| TCGA-04-1343 | 12 | 250250 | 9578132 | 1.473460838 | 4 | amp |
| TCGA-04-1343 | 12 | 9578163 | 9581827 | 4.009944531 | 5 | amp |
| TCGA-04-1343 | 12 | 9582119 | 9698388 | 1.303148712 | 4 | amp |
| TCGA-04-1343 | 12 | 9704014 | 52938541 | 1.141448513 | 3 | amp |
| TCGA-04-1343 | 12 | 52939325 | 133733601 | 1.295158529 | 4 | amp |
| TCGA-04-1343 | 12 | 133758539 | 133779395 | 0.740719066 | 3 | amp |
| TCGA-04-1343 | 13 | 19240876 | 19414327 | 0.888153811 | 3 | amp |
| TCGA-04-1343 | 13 | 20656152 | 21013932 | 1.194458484 | 3 | amp |
| TCGA-04-1343 | 13 | 115089276 | 115091796 | 0.894919566 | 4 | amp |
| TCGA-04-1343 | 14 | 19377543 | 100126799 | 0.936984855 | 3 | amp |
| TCGA-04-1343 | 14 | 100129135 | 100158217 | 3.647504798 | 5 | amp |
| TCGA-04-1343 | 14 | 100165749 | 107283263 | 0.99944052 | 3 | amp |
| TCGA-04-1343 | 15 | 40556938 | 42140879 | 0.886194078 | 3 | amp |

| | | | | | | |
|---|---|---|---|---|---|---|
| TCGA-04-1343 | 15 | 43875044 | 44089150 | 0.973391718 | 3 | amp |
| TCGA-04-1343 | 15 | 45386258 | 64973587 | 0.889288756 | 3 | amp |
| TCGA-04-1343 | 15 | 64980847 | 65703712 | 1.519874818 | 4 | amp |
| TCGA-04-1343 | 15 | 65739162 | 68497768 | 0.907253181 | 3 | amp |
| TCGA-04-1343 | 15 | 68500432 | 70368525 | 1.501409409 | 4 | amp |
| TCGA-04-1343 | 15 | 70949370 | 72432643 | 0.893981791 | 3 | amp |
| TCGA-04-1343 | 15 | 72454261 | 72597170 | 1.791749413 | 4 | amp |
| TCGA-04-1343 | 15 | 72636359 | 73889716 | 0.936895535 | 3 | amp |
| TCGA-04-1343 | 15 | 73991929 | 75134810 | 1.389709659 | 4 | amp |
| TCGA-04-1343 | 15 | 75137723 | 75304243 | 2.517768527 | 5 | amp |
| TCGA-04-1343 | 15 | 75304961 | 76152330 | 1.34732703 | 4 | amp |
| TCGA-04-1343 | 15 | 76161196 | 77771708 | 1.039163678 | 3 | amp |
| TCGA-04-1343 | 15 | 77906473 | 79277550 | 1.560208746 | 4 | amp |
| TCGA-04-1343 | 15 | 79282512 | 82551522 | 1.055890168 | 3 | amp |
| TCGA-04-1343 | 15 | 82554013 | 83360546 | 1.507360023 | 4 | amp |
| TCGA-04-1343 | 15 | 83424639 | 89195547 | 1.001543723 | 3 | amp |
| TCGA-04-1343 | 15 | 89198553 | 89402583 | 1.326414594 | 4 | amp |
| TCGA-04-1343 | 15 | 89415214 | 89453208 | 3.00021891 | 5 | amp |
| TCGA-04-1343 | 15 | 89659505 | 90934115 | 1.547090563 | 4 | amp |
| TCGA-04-1343 | 15 | 90969299 | 91352547 | 0.897319313 | 3 | amp |
| TCGA-04-1343 | 15 | 91354385 | 91420462 | 1.606035174 | 4 | amp |
| TCGA-04-1343 | 15 | 91422633 | 91453540 | 4.039104969 | 5 | amp |
| TCGA-04-1343 | 15 | 91454374 | 91479722 | 1.807004545 | 4 | amp |
| TCGA-04-1343 | 15 | 91482916 | 91517480 | 3.544219866 | 5 | amp |
| TCGA-04-1343 | 15 | 91517767 | 91561134 | 1.948138356 | 4 | amp |
| TCGA-04-1343 | 15 | 91769478 | 101566375 | 1.001299251 | 3 | amp |
| TCGA-04-1343 | 15 | 101567405 | 102500889 | 1.493494281 | 4 | amp |
| TCGA-04-1343 | 16 | 66517 | 3799715 | 1.513070649 | 4 | amp |
| TCGA-04-1343 | 16 | 3801676 | 3901013 | 0.63627945 | 3 | amp |
| TCGA-04-1343 | 16 | 4015771 | 7102145 | 1.44060501 | 4 | amp |
| TCGA-04-1343 | 16 | 7382955 | 8990965 | 1.017756063 | 3 | amp |
| TCGA-04-1343 | 16 | 10626705 | 18820989 | 0.96043642 | 3 | amp |
| TCGA-04-1343 | 16 | 18908076 | 20381032 | 0.915995377 | 3 | amp |
| TCGA-04-1343 | 16 | 21495654 | 21716671 | 1.220742895 | 3 | amp |
| TCGA-04-1343 | 16 | 23197540 | 24741714 | 1.054749542 | 3 | amp |
| TCGA-04-1343 | 16 | 24988527 | 28603793 | 0.98592563 | 3 | amp |
| TCGA-04-1343 | 16 | 28604559 | 29372873 | 1.638469725 | 4 | amp |
| TCGA-04-1343 | 16 | 29376044 | 29690584 | 0.958496641 | 3 | amp |
| TCGA-04-1343 | 16 | 29705922 | 30214339 | 1.824781509 | 4 | amp |
| TCGA-04-1343 | 16 | 30214795 | 30317312 | 0.81819775 | 3 | amp |
| TCGA-04-1343 | 16 | 30354558 | 30393516 | 2.591357969 | 5 | amp |
| TCGA-04-1343 | 16 | 30393550 | 30725055 | 1.675223634 | 4 | amp |
| TCGA-04-1343 | 16 | 30727304 | 30745140 | 0.745311142 | 3 | amp |
| TCGA-04-1343 | 16 | 30745181 | 31271459 | 1.88132142 | 4 | amp |
| TCGA-04-1343 | 16 | 31273005 | 31343052 | 0.868746692 | 3 | amp |
| TCGA-04-1343 | 16 | 31366533 | 31885348 | 1.828678349 | 4 | amp |
| TCGA-04-1343 | 16 | 31895761 | 33123189 | 1.036571281 | 3 | amp |
| TCGA-04-1343 | 16 | 33205555 | 33793057 | 1.422696208 | 4 | amp |
| TCGA-04-1343 | 16 | 66431826 | 67917672 | 1.017419631 | 3 | amp |
| TCGA-04-1343 | 16 | 69963301 | 70516737 | 1.025168118 | 3 | amp |

| | | | | | | |
|---|---|---|---|---|---|---|
| TCGA-04-1343 | 16 | 85141580 | 90244214 | 1.066602124 | 3 | amp |
| TCGA-04-1343 | 17 | 1368942 | 1424976 | 1.658575052 | 4 | amp |
| TCGA-04-1343 | 17 | 2568647 | 3424359 | 1.711038359 | 4 | amp |
| TCGA-04-1343 | 17 | 3427424 | 3470314 | 2.605332163 | 5 | amp |
| TCGA-04-1343 | 17 | 3474815 | 3527553 | 1.760082782 | 4 | amp |
| TCGA-04-1343 | 17 | 3533449 | 3665302 | 3.416351126 | 5 | amp |
| TCGA-04-1343 | 17 | 3667118 | 3725334 | 1.554841182 | 4 | amp |
| TCGA-04-1343 | 17 | 3769161 | 3853907 | 3.115725178 | 5 | amp |
| TCGA-04-1343 | 17 | 3853936 | 3945912 | 1.66034426 | 4 | amp |
| TCGA-04-1343 | 17 | 3947472 | 3999318 | 0.920654011 | 3 | amp |
| TCGA-04-1343 | 17 | 3999858 | 4434440 | 1.658760291 | 4 | amp |
| TCGA-04-1343 | 17 | 4435801 | 4544998 | 2.83045014 | 5 | amp |
| TCGA-04-1343 | 17 | 4574719 | 4586292 | 1.762255505 | 4 | amp |
| TCGA-04-1343 | 17 | 4594175 | 5013165 | 3.634150036 | 5 | amp |
| TCGA-04-1343 | 17 | 5015045 | 5042999 | 2.08563041 | 4 | amp |
| TCGA-04-1343 | 17 | 5044696 | 5050493 | 2.619709232 | 5 | amp |
| TCGA-04-1343 | 17 | 5051836 | 5323630 | 1.565472335 | 4 | amp |
| TCGA-04-1343 | 17 | 5324580 | 5378116 | 3.268240403 | 5 | amp |
| TCGA-04-1343 | 17 | 5383358 | 6331851 | 1.699715955 | 4 | amp |
| TCGA-04-1343 | 17 | 6337208 | 6368152 | 2.641390145 | 5 | amp |
| TCGA-04-1343 | 17 | 6371452 | 6441414 | 1.620417831 | 4 | amp |
| TCGA-04-1343 | 17 | 6482975 | 6511845 | 0.785975315 | 3 | amp |
| TCGA-04-1343 | 17 | 6513246 | 6901928 | 1.566307304 | 4 | amp |
| TCGA-04-1343 | 17 | 6901956 | 6908678 | 3.938195123 | 5 | amp |
| TCGA-04-1343 | 17 | 6909111 | 6919203 | 2.041299488 | 4 | amp |
| TCGA-04-1343 | 17 | 6919758 | 6942235 | 3.049709009 | 5 | amp |
| TCGA-04-1343 | 17 | 6943031 | 7010652 | 1.451435853 | 4 | amp |
| TCGA-04-1343 | 17 | 7011137 | 7386353 | 3.28685041 | 5 | amp |
| TCGA-04-1343 | 17 | 7399205 | 7415351 | 1.440811823 | 4 | amp |
| TCGA-04-1343 | 17 | 7415449 | 7417507 | 0.752769399 | 3 | amp |
| TCGA-04-1343 | 17 | 7453384 | 7573027 | 3.375848945 | 5 | amp |
| TCGA-04-1343 | 17 | 7573919 | 7593023 | 1.695938414 | 4 | amp |
| TCGA-04-1343 | 17 | 7604012 | 7638086 | 3.035284505 | 5 | amp |
| TCGA-04-1343 | 17 | 7640360 | 7728069 | 1.597747098 | 4 | amp |
| TCGA-04-1343 | 17 | 7733593 | 7736268 | 3.789019186 | 5 | amp |
| TCGA-04-1343 | 17 | 7736313 | 7754561 | 1.983261172 | 4 | amp |
| TCGA-04-1343 | 17 | 7760014 | 7840196 | 3.320484616 | 5 | amp |
| TCGA-04-1343 | 17 | 7840440 | 7907501 | 1.899945255 | 4 | amp |
| TCGA-04-1343 | 17 | 7910359 | 8065124 | 2.889554344 | 5 | amp |
| TCGA-04-1343 | 17 | 8065476 | 8194846 | 1.878790225 | 4 | amp |
| TCGA-04-1343 | 17 | 8195783 | 8285664 | 4.140485292 | 5 | amp |
| TCGA-04-1343 | 17 | 8347572 | 8526571 | 1.416477479 | 4 | amp |
| TCGA-04-1343 | 17 | 8638372 | 8738801 | 2.731539251 | 5 | amp |
| TCGA-04-1343 | 17 | 8739838 | 9124581 | 1.565347018 | 4 | amp |
| TCGA-04-1343 | 17 | 12852946 | 12920472 | 1.528045439 | 4 | amp |
| TCGA-04-1343 | 17 | 17394550 | 17723065 | 1.743271548 | 4 | amp |
| TCGA-04-1343 | 17 | 17750872 | 18008025 | 0.990926879 | 3 | amp |
| TCGA-04-1343 | 17 | 18009683 | 18346096 | 1.603930014 | 4 | amp |
| TCGA-04-1343 | 17 | 18880839 | 19651777 | 1.336675653 | 4 | amp |
| TCGA-04-1343 | 17 | 21201615 | 21408126 | 1.394950622 | 4 | amp |

| | | | | | | |
|---|---|---|---|---|---|---|
| TCGA-04-1343 | 17 | 21544385 | 25919658 | 1.09163854 | 3 | amp |
| TCGA-04-1343 | 17 | 25924257 | 25950438 | 2.042584727 | 4 | amp |
| TCGA-04-1343 | 17 | 25958220 | 26094916 | 0.826611411 | 3 | amp |
| TCGA-04-1343 | 17 | 26095969 | 26125840 | 1.476750729 | 4 | amp |
| TCGA-04-1343 | 17 | 26207285 | 26604485 | 0.793688995 | 3 | amp |
| TCGA-04-1343 | 17 | 26652480 | 26692016 | 2.918460607 | 5 | amp |
| TCGA-04-1343 | 17 | 26695497 | 26816415 | 1.934837754 | 4 | amp |
| TCGA-04-1343 | 17 | 26817389 | 26824321 | 0.873954538 | 3 | amp |
| TCGA-04-1343 | 17 | 26851482 | 26864474 | 1.496062695 | 4 | amp |
| TCGA-04-1343 | 17 | 26874310 | 26888663 | 2.75664687 | 5 | amp |
| TCGA-04-1343 | 17 | 26890404 | 26907142 | 1.484254216 | 4 | amp |
| TCGA-04-1343 | 17 | 26910477 | 26913539 | 2.618761848 | 5 | amp |
| TCGA-04-1343 | 17 | 26918652 | 26951449 | 1.568863364 | 4 | amp |
| TCGA-04-1343 | 17 | 26955249 | 26961128 | 2.584136515 | 5 | amp |
| TCGA-04-1343 | 17 | 26961450 | 27448981 | 1.693684218 | 4 | amp |
| TCGA-04-1343 | 17 | 27449167 | 27849597 | 0.852853402 | 3 | amp |
| TCGA-04-1343 | 17 | 27857380 | 27963822 | 1.78659701 | 4 | amp |
| TCGA-04-1343 | 17 | 29253800 | 29344027 | 1.521276877 | 4 | amp |
| TCGA-04-1343 | 17 | 33427953 | 33592918 | 1.665668612 | 4 | amp |
| TCGA-04-1343 | 17 | 34842735 | 35311258 | 1.586684698 | 4 | amp |
| TCGA-04-1343 | 17 | 36476426 | 36926043 | 1.759181834 | 4 | amp |
| TCGA-04-1343 | 17 | 37334224 | 37371486 | 1.918522039 | 4 | amp |
| TCGA-04-1343 | 17 | 37762158 | 37871803 | 1.603332916 | 4 | amp |
| TCGA-04-1343 | 17 | 37871942 | 37879971 | 2.816089708 | 5 | amp |
| TCGA-04-1343 | 17 | 37880123 | 37903196 | 1.930836679 | 4 | amp |
| TCGA-04-1343 | 17 | 37922034 | 38029397 | 0.762428684 | 3 | amp |
| TCGA-04-1343 | 17 | 38031476 | 38151788 | 1.57440517 | 4 | amp |
| TCGA-04-1343 | 17 | 38152394 | 38173249 | 2.906924905 | 5 | amp |
| TCGA-04-1343 | 17 | 38175689 | 38192447 | 0.984466487 | 3 | amp |
| TCGA-04-1343 | 17 | 38209531 | 38350097 | 1.812478726 | 4 | amp |
| TCGA-04-1343 | 17 | 38412652 | 38458298 | 0.637921013 | 3 | amp |
| TCGA-04-1343 | 17 | 38487409 | 38508790 | 2.660420903 | 5 | amp |
| TCGA-04-1343 | 17 | 38510535 | 38634030 | 1.534462289 | 4 | amp |
| TCGA-04-1343 | 17 | 39672049 | 39969590 | 1.688028249 | 4 | amp |
| TCGA-04-1343 | 17 | 39974315 | 39981961 | 2.587707593 | 5 | amp |
| TCGA-04-1343 | 17 | 39983627 | 40263946 | 1.699124696 | 4 | amp |
| TCGA-04-1343 | 17 | 40265613 | 40270427 | 2.239506225 | 5 | amp |
| TCGA-04-1343 | 17 | 40271208 | 40475676 | 1.869570302 | 4 | amp |
| TCGA-04-1343 | 17 | 40689369 | 40865436 | 1.677553517 | 4 | amp |
| TCGA-04-1343 | 17 | 40869979 | 40881020 | 0.872805884 | 3 | amp |
| TCGA-04-1343 | 17 | 40914336 | 40950191 | 1.770703441 | 4 | amp |
| TCGA-04-1343 | 17 | 41122242 | 41180752 | 1.46273958 | 4 | amp |
| TCGA-04-1343 | 17 | 41609994 | 41852312 | 1.953591651 | 4 | amp |
| TCGA-04-1343 | 17 | 41879034 | 41891766 | 0.729597089 | 3 | amp |
| TCGA-04-1343 | 17 | 41898177 | 42189071 | 1.753708076 | 4 | amp |
| TCGA-04-1343 | 17 | 42194782 | 42272880 | 3.114947408 | 5 | amp |
| TCGA-04-1343 | 17 | 42273364 | 42330792 | 1.818241801 | 4 | amp |
| TCGA-04-1343 | 17 | 42331826 | 42430217 | 1.029411923 | 3 | amp |
| TCGA-04-1343 | 17 | 42449671 | 42455171 | 3.96434064 | 5 | amp |
| TCGA-04-1343 | 17 | 42456969 | 42466867 | 1.794532428 | 4 | amp |

| | | | | | | |
|---|---|---|---|---|---|---|
| TCGA-04-1343 | 17 | 42474898 | 42828651 | 0.903989237 | 3 | amp |
| TCGA-04-1343 | 17 | 42847063 | 42857174 | 1.614023892 | 4 | amp |
| TCGA-04-1343 | 17 | 42881989 | 42979073 | 0.896722023 | 3 | amp |
| TCGA-04-1343 | 17 | 42979688 | 43333822 | 1.406361098 | 4 | amp |
| TCGA-04-1343 | 17 | 43342519 | 43480191 | 1.000906527 | 3 | amp |
| TCGA-04-1343 | 17 | 43480939 | 43482066 | 30.3889136 | 5 | amp |
| TCGA-04-1343 | 17 | 43664066 | 44091725 | 1.616916655 | 4 | amp |
| TCGA-04-1343 | 17 | 44095949 | 44249524 | 0.874985759 | 3 | amp |
| TCGA-04-1343 | 17 | 44336902 | 44415124 | 1.689113883 | 4 | amp |
| TCGA-04-1343 | 17 | 44416822 | 44592703 | 0.727335546 | 3 | amp |
| TCGA-04-1343 | 17 | 44594420 | 44635038 | 1.697531962 | 4 | amp |
| TCGA-04-1343 | 17 | 45755666 | 46148988 | 1.416953808 | 4 | amp |
| TCGA-04-1343 | 17 | 46152324 | 46474154 | 0.781823816 | 3 | amp |
| TCGA-04-1343 | 17 | 46606892 | 46657314 | 1.87241699 | 4 | amp |
| TCGA-04-1343 | 17 | 46669514 | 46700499 | 2.927984506 | 5 | amp |
| TCGA-04-1343 | 17 | 46799626 | 46973133 | 0.996543423 | 3 | amp |
| TCGA-04-1343 | 17 | 46988145 | 47304090 | 1.562654087 | 4 | amp |
| TCGA-04-1343 | 17 | 47375722 | 47486581 | 0.752088167 | 3 | amp |
| TCGA-04-1343 | 17 | 47486613 | 47579578 | 1.801474126 | 4 | amp |
| TCGA-04-1343 | 17 | 47587689 | 47700193 | 0.855893477 | 3 | amp |
| TCGA-04-1343 | 17 | 47778785 | 47869441 | 1.264668285 | 4 | amp |
| TCGA-04-1343 | 17 | 47874079 | 47904873 | 0.823183793 | 3 | amp |
| TCGA-04-1343 | 17 | 47915959 | 48072379 | 1.620669194 | 4 | amp |
| TCGA-04-1343 | 17 | 48145334 | 48202361 | 3.689133512 | 5 | amp |
| TCGA-04-1343 | 17 | 48212662 | 48628293 | 1.588040771 | 4 | amp |
| TCGA-04-1343 | 17 | 48631591 | 48649438 | 2.999429924 | 5 | amp |
| TCGA-04-1343 | 17 | 48655496 | 48699207 | 1.534433085 | 4 | amp |
| TCGA-04-1343 | 17 | 55729400 | 56060766 | 1.745516587 | 4 | amp |
| TCGA-04-1343 | 17 | 56082743 | 56327998 | 0.956660495 | 3 | amp |
| TCGA-04-1343 | 17 | 56329195 | 56440821 | 1.914934575 | 4 | amp |
| TCGA-04-1343 | 17 | 56440833 | 56544352 | 0.794190886 | 3 | amp |
| TCGA-04-1343 | 17 | 56557200 | 56573711 | 1.919858975 | 4 | amp |
| TCGA-04-1343 | 17 | 56581031 | 56604563 | 2.58952213 | 5 | amp |
| TCGA-04-1343 | 17 | 58147020 | 58235845 | 2.315207196 | 5 | amp |
| TCGA-04-1343 | 17 | 60741839 | 60782980 | 2.386027886 | 5 | amp |
| TCGA-04-1343 | 17 | 60788558 | 60821932 | 1.292148654 | 3 | amp |
| TCGA-04-1343 | 17 | 61495657 | 61843583 | 1.528762433 | 4 | amp |
| TCGA-04-1343 | 17 | 61905473 | 62050244 | 1.550716654 | 4 | amp |
| TCGA-04-1343 | 17 | 63526006 | 63554750 | 1.688608354 | 4 | amp |
| TCGA-04-1343 | 17 | 65978325 | 66303863 | 1.690120537 | 4 | amp |
| TCGA-04-1343 | 17 | 71084759 | 72206015 | 1.282728044 | 4 | amp |
| TCGA-04-1343 | 17 | 72218589 | 72287275 | 0.894482138 | 3 | amp |
| TCGA-04-1343 | 17 | 72295776 | 72443197 | 1.656424216 | 4 | amp |
| TCGA-04-1343 | 17 | 72469633 | 72700965 | 0.835777195 | 3 | amp |
| TCGA-04-1343 | 17 | 72708893 | 72863011 | 1.453281919 | 4 | amp |
| TCGA-04-1343 | 17 | 72921598 | 72956063 | 3.291459365 | 5 | amp |
| TCGA-04-1343 | 17 | 72957905 | 73045428 | 1.84699504 | 4 | amp |
| TCGA-04-1343 | 17 | 73049064 | 73106749 | 2.681424809 | 5 | amp |
| TCGA-04-1343 | 17 | 73126844 | 73201932 | 1.623812967 | 4 | amp |
| TCGA-04-1343 | 17 | 73204578 | 73231305 | 0.958089745 | 3 | amp |

| | | | | | | |
|---|---|---|---|---|---|---|
| TCGA-04-1343 | 17 | 73231663 | 73659117 | 1.371810919 | 4 | amp |
| TCGA-04-1343 | 17 | 73661041 | 73723375 | 0.912832856 | 3 | amp |
| TCGA-04-1343 | 17 | 73723713 | 74750208 | 1.552195568 | 4 | amp |
| TCGA-04-1343 | 17 | 74763425 | 74774471 | 0.565693028 | 3 | amp |
| TCGA-04-1343 | 17 | 74868898 | 74944940 | 1.569860272 | 4 | amp |
| TCGA-04-1343 | 17 | 75085331 | 76064040 | 0.878641753 | 3 | amp |
| TCGA-04-1343 | 17 | 76067145 | 76688596 | 1.419963344 | 4 | amp |
| TCGA-04-1343 | 17 | 76692029 | 76795107 | 0.740948111 | 3 | amp |
| TCGA-04-1343 | 17 | 76795739 | 77044194 | 1.267359148 | 4 | amp |
| TCGA-04-1343 | 17 | 77073485 | 77707498 | 2.614566487 | 5 | amp |
| TCGA-04-1343 | 17 | 77708794 | 78164755 | 1.864764853 | 4 | amp |
| TCGA-04-1343 | 17 | 78165046 | 78180886 | 2.686179986 | 5 | amp |
| TCGA-04-1343 | 17 | 78196399 | 78283014 | 1.252887159 | 4 | amp |
| TCGA-04-1343 | 17 | 78406896 | 79478163 | 1.314953874 | 4 | amp |
| TCGA-04-1343 | 17 | 79478192 | 79575860 | 0.93121164 | 3 | amp |
| TCGA-04-1343 | 17 | 79580311 | 79684534 | 1.462016669 | 4 | amp |
| TCGA-04-1343 | 17 | 79686798 | 79813515 | 6.266908038 | 5 | amp |
| TCGA-04-1343 | 17 | 79817039 | 81188237 | 1.730907704 | 4 | amp |
| TCGA-04-1343 | 18 | 47273 | 3273108 | 0.917386525 | 3 | amp |
| TCGA-04-1343 | 18 | 10485439 | 10681763 | 1.32123063 | 4 | amp |
| TCGA-04-1343 | 18 | 10689606 | 12429435 | 2.550805146 | 5 | amp |
| TCGA-04-1343 | 18 | 12449585 | 12535657 | 1.631085422 | 4 | amp |
| TCGA-04-1343 | 18 | 12546626 | 12724677 | 2.246781394 | 5 | amp |
| TCGA-04-1343 | 18 | 12725393 | 12951938 | 1.601246687 | 4 | amp |
| TCGA-04-1343 | 18 | 12955414 | 12987066 | 2.940786229 | 5 | amp |
| TCGA-04-1343 | 18 | 13030536 | 14888919 | 1.718939084 | 4 | amp |
| TCGA-04-1343 | 18 | 18531256 | 19444676 | 0.732028254 | 3 | amp |
| TCGA-04-1343 | 18 | 19761356 | 21046288 | 1.593442113 | 4 | amp |
| TCGA-04-1343 | 18 | 21047306 | 21096395 | 2.332880727 | 5 | amp |
| TCGA-04-1343 | 18 | 21098838 | 21123533 | 1.676613716 | 4 | amp |
| TCGA-04-1343 | 18 | 21124278 | 21343547 | 2.48101826 | 5 | amp |
| TCGA-04-1343 | 18 | 21353445 | 21427649 | 1.551810909 | 4 | amp |
| TCGA-04-1343 | 18 | 21437759 | 21474346 | 2.291483425 | 5 | amp |
| TCGA-04-1343 | 18 | 21474796 | 25727752 | 1.703707523 | 4 | amp |
| TCGA-04-1343 | 18 | 27878840 | 29046724 | 0.939048554 | 3 | amp |
| TCGA-04-1343 | 18 | 40857169 | 43620022 | 1.270324158 | 4 | amp |
| TCGA-04-1343 | 18 | 43664228 | 43685366 | 2.254819535 | 5 | amp |
| TCGA-04-1343 | 18 | 43698099 | 44336478 | 1.57741728 | 4 | amp |
| TCGA-04-1343 | 18 | 44392365 | 46385942 | 1.063968862 | 3 | amp |
| TCGA-04-1343 | 18 | 47008657 | 47340842 | 1.245772103 | 3 | amp |
| TCGA-04-1343 | 18 | 77089101 | 77170155 | 0.941217371 | 3 | amp |
| TCGA-04-1343 | 18 | 77210889 | 77960823 | 1.995301819 | 4 | amp |
| TCGA-04-1343 | 19 | 71882 | 805590 | 1.586955764 | 4 | amp |
| TCGA-04-1343 | 19 | 807825 | 1369679 | 3.386699151 | 5 | amp |
| TCGA-04-1343 | 19 | 1373054 | 2515202 | 1.743251516 | 4 | amp |
| TCGA-04-1343 | 19 | 2759213 | 2873676 | 3.043917304 | 5 | amp |
| TCGA-04-1343 | 19 | 2876032 | 2994114 | 1.550798876 | 4 | amp |
| TCGA-04-1343 | 19 | 2997839 | 3013861 | 2.635177843 | 5 | amp |
| TCGA-04-1343 | 19 | 3017787 | 3905662 | 1.670011544 | 4 | amp |
| TCGA-04-1343 | 19 | 3907704 | 3924645 | 2.971335018 | 5 | amp |

| | | | | | | |
|---|---|---|---|---|---|---|
| TCGA-04-1343 | 19 | 3937201 | 4219791 | 1.792426836 | 4 | amp |
| TCGA-04-1343 | 19 | 4222609 | 4317287 | 3.229747436 | 5 | amp |
| TCGA-04-1343 | 19 | 4318302 | 4475369 | 1.5238057 | 4 | amp |
| TCGA-04-1343 | 19 | 4475390 | 4660781 | 2.571166244 | 5 | amp |
| TCGA-04-1343 | 19 | 4683444 | 5210872 | 1.747523007 | 4 | amp |
| TCGA-04-1343 | 19 | 5218396 | 5260884 | 2.636600074 | 5 | amp |
| TCGA-04-1343 | 19 | 5262918 | 6454768 | 1.438689377 | 4 | amp |
| TCGA-04-1343 | 19 | 6456426 | 6477171 | 2.408197687 | 5 | amp |
| TCGA-04-1343 | 19 | 6478947 | 6696696 | 1.684706849 | 4 | amp |
| TCGA-04-1343 | 19 | 6697340 | 6711222 | 2.494663203 | 5 | amp |
| TCGA-04-1343 | 19 | 6712192 | 6736003 | 1.836143757 | 4 | amp |
| TCGA-04-1343 | 19 | 6736579 | 6759714 | 3.087062681 | 5 | amp |
| TCGA-04-1343 | 19 | 6760643 | 6836630 | 1.323808522 | 4 | amp |
| TCGA-04-1343 | 19 | 6836938 | 7506987 | 0.97924412 | 3 | amp |
| TCGA-04-1343 | 19 | 7509004 | 7688235 | 1.674060401 | 4 | amp |
| TCGA-04-1343 | 19 | 7689138 | 7705897 | 4.210697546 | 5 | amp |
| TCGA-04-1343 | 19 | 7706871 | 8028708 | 1.473797068 | 4 | amp |
| TCGA-04-1343 | 19 | 8032441 | 8122865 | 2.433966114 | 5 | amp |
| TCGA-04-1343 | 19 | 8127031 | 8326769 | 1.234788858 | 4 | amp |
| TCGA-04-1343 | 19 | 8326783 | 8467468 | 2.911507738 | 5 | amp |
| TCGA-04-1343 | 19 | 8468219 | 8666040 | 1.566417374 | 4 | amp |
| TCGA-04-1343 | 19 | 8668539 | 9874140 | 0.994761937 | 3 | amp |
| TCGA-04-1343 | 19 | 9875558 | 9875678 | 1.190336383 | 4 | amp |
| TCGA-04-1343 | 19 | 9921562 | 9958820 | 2.970993479 | 5 | amp |
| TCGA-04-1343 | 19 | 9960166 | 10080631 | 1.421498302 | 4 | amp |
| TCGA-04-1343 | 19 | 10081587 | 10288065 | 1.15739123 | 3 | amp |
| TCGA-04-1343 | 19 | 10291015 | 10694787 | 1.817203034 | 4 | amp |
| TCGA-04-1343 | 19 | 10697826 | 10754067 | 2.804128503 | 5 | amp |
| TCGA-04-1343 | 19 | 10781222 | 11242034 | 1.455241506 | 4 | amp |
| TCGA-04-1343 | 19 | 11258471 | 11328106 | 2.79030532 | 5 | amp |
| TCGA-04-1343 | 19 | 11332491 | 12721555 | 1.576174536 | 4 | amp |
| TCGA-04-1343 | 19 | 12721804 | 12776627 | 2.616274479 | 5 | amp |
| TCGA-04-1343 | 19 | 12779145 | 12800126 | 1.28868296 | 4 | amp |
| TCGA-04-1343 | 19 | 12800128 | 12865887 | 2.779643825 | 5 | amp |
| TCGA-04-1343 | 19 | 12866141 | 12874437 | 1.713002771 | 4 | amp |
| TCGA-04-1343 | 19 | 12874446 | 13002223 | 2.550521474 | 5 | amp |
| TCGA-04-1343 | 19 | 13002228 | 13060275 | 1.578815849 | 4 | amp |
| TCGA-04-1343 | 19 | 13063464 | 13136400 | 2.624686312 | 5 | amp |
| TCGA-04-1343 | 19 | 13183831 | 14001297 | 1.482966801 | 4 | amp |
| TCGA-04-1343 | 19 | 14003590 | 14039028 | 2.7440459 | 5 | amp |
| TCGA-04-1343 | 19 | 14065110 | 14266374 | 1.806729174 | 4 | amp |
| TCGA-04-1343 | 19 | 14268047 | 14288569 | 2.468543672 | 5 | amp |
| TCGA-04-1343 | 19 | 14499226 | 14674103 | 1.597032519 | 4 | amp |
| TCGA-04-1343 | 19 | 14674401 | 15227335 | 1.033609803 | 3 | amp |
| TCGA-04-1343 | 19 | 15228608 | 15734229 | 1.43144011 | 4 | amp |
| TCGA-04-1343 | 19 | 15734481 | 17273277 | 1.071502643 | 3 | amp |
| TCGA-04-1343 | 19 | 17273744 | 17895753 | 1.727344032 | 4 | amp |
| TCGA-04-1343 | 19 | 17918569 | 17949255 | 2.323985614 | 5 | amp |
| TCGA-04-1343 | 19 | 17951034 | 17991777 | 1.680565619 | 4 | amp |
| TCGA-04-1343 | 19 | 17992764 | 18004723 | 2.527208669 | 5 | amp |

| | | | | | | |
|---|---|---|---|---|---|---|
| TCGA-04-1343 | 19 | 18085888 | 18272878 | 1.581430946 | 4 | amp |
| TCGA-04-1343 | 19 | 18273746 | 18280063 | 3.871762566 | 5 | amp |
| TCGA-04-1343 | 19 | 18304638 | 18672976 | 1.653341263 | 4 | amp |
| TCGA-04-1343 | 19 | 18675604 | 18685973 | 2.413849252 | 5 | amp |
| TCGA-04-1343 | 19 | 18709176 | 19312552 | 1.480339588 | 4 | amp |
| TCGA-04-1343 | 19 | 19312671 | 19330164 | 2.424243623 | 5 | amp |
| TCGA-04-1343 | 19 | 19334796 | 19453689 | 1.791001208 | 4 | amp |
| TCGA-04-1343 | 19 | 19454607 | 19627149 | 0.776702465 | 3 | amp |
| TCGA-04-1343 | 19 | 19645807 | 19791059 | 1.683433985 | 4 | amp |
| TCGA-04-1343 | 19 | 19822129 | 23159929 | 0.815485767 | 3 | amp |
| TCGA-04-1343 | 19 | 23542308 | 30311801 | 1.377696865 | 4 | amp |
| TCGA-04-1343 | 19 | 30312616 | 30314742 | 2.967961861 | 5 | amp |
| TCGA-04-1343 | 19 | 30462056 | 30462176 | 1.449150263 | 4 | amp |
| TCGA-04-1343 | 19 | 30476078 | 32973185 | 1.069729279 | 3 | amp |
| TCGA-04-1343 | 19 | 33075826 | 33616119 | 1.585787367 | 4 | amp |
| TCGA-04-1343 | 19 | 33617446 | 33878438 | 2.429865255 | 5 | amp |
| TCGA-04-1343 | 19 | 33953863 | 34833383 | 1.488584484 | 4 | amp |
| TCGA-04-1343 | 19 | 34838744 | 34872457 | 3.341941777 | 5 | amp |
| TCGA-04-1343 | 19 | 34884122 | 34890558 | 1.343454434 | 4 | amp |
| TCGA-04-1343 | 19 | 34890596 | 34922860 | 2.595655499 | 5 | amp |
| TCGA-04-1343 | 19 | 34924224 | 35435774 | 1.632826008 | 4 | amp |
| TCGA-04-1343 | 19 | 35448827 | 35651717 | 2.861933383 | 5 | amp |
| TCGA-04-1343 | 19 | 35655043 | 35719642 | 1.881398703 | 4 | amp |
| TCGA-04-1343 | 19 | 35741179 | 35804394 | 3.064180793 | 5 | amp |
| TCGA-04-1343 | 19 | 35822870 | 35993121 | 1.969635681 | 4 | amp |
| TCGA-04-1343 | 19 | 35993669 | 36015853 | 2.387368712 | 5 | amp |
| TCGA-04-1343 | 19 | 36017512 | 36019235 | 1.50357196 | 4 | amp |
| TCGA-04-1343 | 19 | 36024372 | 36036739 | 3.54705179 | 5 | amp |
| TCGA-04-1343 | 19 | 36037397 | 36111282 | 1.793660359 | 4 | amp |
| TCGA-04-1343 | 19 | 36113418 | 36272017 | 2.968582722 | 5 | amp |
| TCGA-04-1343 | 19 | 36272042 | 36339737 | 2.026369774 | 4 | amp |
| TCGA-04-1343 | 19 | 36339813 | 36640787 | 3.02060268 | 5 | amp |
| TCGA-04-1343 | 19 | 36641849 | 37203785 | 1.656152881 | 4 | amp |
| TCGA-04-1343 | 19 | 37209779 | 37838786 | 1.064255591 | 3 | amp |
| TCGA-04-1343 | 19 | 37853003 | 38601065 | 1.598617126 | 4 | amp |
| TCGA-04-1343 | 19 | 38609933 | 38903944 | 2.999483822 | 5 | amp |
| TCGA-04-1343 | 19 | 38910456 | 38954174 | 1.823640074 | 4 | amp |
| TCGA-04-1343 | 19 | 38954348 | 39003183 | 3.746853335 | 5 | amp |
| TCGA-04-1343 | 19 | 39005646 | 39037185 | 1.679325686 | 4 | amp |
| TCGA-04-1343 | 19 | 39038846 | 39103436 | 3.131473998 | 5 | amp |
| TCGA-04-1343 | 19 | 39104495 | 39114896 | 1.729338578 | 4 | amp |
| TCGA-04-1343 | 19 | 39116614 | 39760509 | 3.092503538 | 5 | amp |
| TCGA-04-1343 | 19 | 39786996 | 39789213 | 1.661401247 | 4 | amp |
| TCGA-04-1343 | 19 | 39819062 | 39860769 | 4.617986946 | 5 | amp |
| TCGA-04-1343 | 19 | 39866229 | 39874212 | 1.9875962 | 4 | amp |
| TCGA-04-1343 | 19 | 39876596 | 39926357 | 2.600349342 | 5 | amp |
| TCGA-04-1343 | 19 | 39926421 | 39965376 | 1.764887984 | 4 | amp |
| TCGA-04-1343 | 19 | 39966678 | 40009809 | 3.360241466 | 5 | amp |
| TCGA-04-1343 | 19 | 40022869 | 40319259 | 1.333717576 | 4 | amp |
| TCGA-04-1343 | 19 | 40320443 | 40477187 | 2.886592048 | 5 | amp |

| | | | | | | |
|---|---|---|---|---|---|---|
| TCGA-04-1343 | 19 | 40478011 | 40589094 | 1.318126415 | 4 | amp |
| TCGA-04-1343 | 19 | 40704221 | 40741273 | 2.847330928 | 5 | amp |
| TCGA-04-1343 | 19 | 40741753 | 40887132 | 1.796408513 | 4 | amp |
| TCGA-04-1343 | 19 | 40889627 | 41099139 | 2.982169345 | 5 | amp |
| TCGA-04-1343 | 19 | 41105071 | 41120406 | 1.536583671 | 4 | amp |
| TCGA-04-1343 | 19 | 41122740 | 41257409 | 2.909711839 | 5 | amp |
| TCGA-04-1343 | 19 | 41263172 | 41332596 | 1.961352245 | 4 | amp |
| TCGA-04-1343 | 19 | 41349671 | 41388140 | 0.814182519 | 3 | amp |
| TCGA-04-1343 | 19 | 41441971 | 41601084 | 1.373612182 | 4 | amp |
| TCGA-04-1343 | 19 | 41601633 | 41774308 | 2.760214532 | 5 | amp |
| TCGA-04-1343 | 19 | 41777913 | 41812531 | 1.872608765 | 4 | amp |
| TCGA-04-1343 | 19 | 41822246 | 41897926 | 2.714500997 | 5 | amp |
| TCGA-04-1343 | 19 | 41898768 | 42315302 | 1.427504814 | 4 | amp |
| TCGA-04-1343 | 19 | 42341167 | 42482973 | 2.713555817 | 5 | amp |
| TCGA-04-1343 | 19 | 42485570 | 42600350 | 1.73363065 | 4 | amp |
| TCGA-04-1343 | 19 | 42621309 | 42754096 | 3.087869514 | 5 | amp |
| TCGA-04-1343 | 19 | 42754449 | 42795661 | 1.812820557 | 4 | amp |
| TCGA-04-1343 | 19 | 42795683 | 42911970 | 3.307959844 | 5 | amp |
| TCGA-04-1343 | 19 | 42912170 | 43026395 | 1.608039047 | 4 | amp |
| TCGA-04-1343 | 19 | 43031132 | 43859993 | 1.08186399 | 3 | amp |
| TCGA-04-1343 | 19 | 43860141 | 43883334 | 1.71315423 | 4 | amp |
| TCGA-04-1343 | 19 | 43919993 | 43990505 | 2.45632934 | 5 | amp |
| TCGA-04-1343 | 19 | 43990744 | 44011087 | 1.713780811 | 4 | amp |
| TCGA-04-1343 | 19 | 44012093 | 44051142 | 2.6523788 | 5 | amp |
| TCGA-04-1343 | 19 | 44055630 | 44101362 | 1.98105907 | 4 | amp |
| TCGA-04-1343 | 19 | 44102926 | 44131988 | 2.976202827 | 5 | amp |
| TCGA-04-1343 | 19 | 44152988 | 44174233 | 1.453721685 | 4 | amp |
| TCGA-04-1343 | 19 | 44222656 | 44280800 | 2.266694997 | 5 | amp |
| TCGA-04-1343 | 19 | 44284838 | 44303975 | 1.567263446 | 4 | amp |
| TCGA-04-1343 | 19 | 44335449 | 44982390 | 0.971371088 | 3 | amp |
| TCGA-04-1343 | 19 | 44983442 | 45284354 | 1.543903216 | 4 | amp |
| TCGA-04-1343 | 19 | 45284420 | 45322188 | 2.417327678 | 5 | amp |
| TCGA-04-1343 | 19 | 45322230 | 45377780 | 1.652487075 | 4 | amp |
| TCGA-04-1343 | 19 | 45381776 | 46327138 | 3.292465155 | 5 | amp |
| TCGA-04-1343 | 19 | 46328358 | 46808653 | 1.510858654 | 4 | amp |
| TCGA-04-1343 | 19 | 46811429 | 46825247 | 2.663126422 | 5 | amp |
| TCGA-04-1343 | 19 | 46828753 | 47178392 | 1.889661827 | 4 | amp |
| TCGA-04-1343 | 19 | 47181606 | 47204390 | 3.090540976 | 5 | amp |
| TCGA-04-1343 | 19 | 47207386 | 47242153 | 1.884786141 | 4 | amp |
| TCGA-04-1343 | 19 | 47278675 | 47341814 | 2.341234505 | 5 | amp |
| TCGA-04-1343 | 19 | 47341966 | 47535632 | 1.535807859 | 4 | amp |
| TCGA-04-1343 | 19 | 47535855 | 47597341 | 2.477890885 | 5 | amp |
| TCGA-04-1343 | 19 | 47597635 | 47712521 | 1.464421385 | 4 | amp |
| TCGA-04-1343 | 19 | 47761568 | 47774945 | 2.495281471 | 5 | amp |
| TCGA-04-1343 | 19 | 47813063 | 47856999 | 1.714093095 | 4 | amp |
| TCGA-04-1343 | 19 | 47858241 | 47986653 | 3.367122328 | 5 | amp |
| TCGA-04-1343 | 19 | 47995263 | 48202084 | 1.983950213 | 4 | amp |
| TCGA-04-1343 | 19 | 48219831 | 48284646 | 2.648720322 | 5 | amp |
| TCGA-04-1343 | 19 | 48304940 | 48608032 | 1.540335592 | 4 | amp |
| TCGA-04-1343 | 19 | 48608585 | 48621086 | 2.442799527 | 5 | amp |

| | | | | | | |
|---|---|---|---|---|---|---|
| TCGA-04-1343 | 19 | 48624352 | 48737757 | 1.863545878 | 4 | amp |
| TCGA-04-1343 | 19 | 48782970 | 48830200 | 2.363184497 | 5 | amp |
| TCGA-04-1343 | 19 | 48830768 | 48869205 | 1.553144613 | 4 | amp |
| TCGA-04-1343 | 19 | 48876712 | 48893008 | 2.892053814 | 5 | amp |
| TCGA-04-1343 | 19 | 48893670 | 48945239 | 1.353557185 | 4 | amp |
| TCGA-04-1343 | 19 | 48945372 | 48982483 | 2.971499715 | 5 | amp |
| TCGA-04-1343 | 19 | 49000636 | 49119228 | 1.676300732 | 4 | amp |
| TCGA-04-1343 | 19 | 49119247 | 49249118 | 2.906220737 | 5 | amp |
| TCGA-04-1343 | 19 | 49253359 | 49443011 | 1.665183617 | 4 | amp |
| TCGA-04-1343 | 19 | 49445652 | 49655660 | 2.694841411 | 5 | amp |
| TCGA-04-1343 | 19 | 49656462 | 49684721 | 1.8657767 | 4 | amp |
| TCGA-04-1343 | 19 | 49685826 | 49714533 | 2.413220796 | 5 | amp |
| TCGA-04-1343 | 19 | 49714692 | 49814636 | 1.615281865 | 4 | amp |
| TCGA-04-1343 | 19 | 49838741 | 49966039 | 3.021702594 | 5 | amp |
| TCGA-04-1343 | 19 | 49967068 | 50017785 | 1.89110097 | 4 | amp |
| TCGA-04-1343 | 19 | 50027688 | 50140403 | 2.768861374 | 5 | amp |
| TCGA-04-1343 | 19 | 50148277 | 50157706 | 1.546499099 | 4 | amp |
| TCGA-04-1343 | 19 | 50157867 | 50204684 | 3.410055595 | 5 | amp |
| TCGA-04-1343 | 19 | 50207945 | 50213778 | 1.597074418 | 4 | amp |
| TCGA-04-1343 | 19 | 50213926 | 50266539 | 2.857054379 | 5 | amp |
| TCGA-04-1343 | 19 | 50284987 | 50295376 | 1.669022708 | 4 | amp |
| TCGA-04-1343 | 19 | 50302547 | 50463113 | 3.129791984 | 5 | amp |
| TCGA-04-1343 | 19 | 50463355 | 50727482 | 1.61847544 | 4 | amp |
| TCGA-04-1343 | 19 | 50730131 | 51322647 | 2.73229354 | 5 | amp |
| TCGA-04-1343 | 19 | 51323072 | 51485678 | 1.551770756 | 4 | amp |
| TCGA-04-1343 | 19 | 51499242 | 51518251 | 2.33474064 | 5 | amp |
| TCGA-04-1343 | 19 | 51518589 | 51771897 | 1.519398483 | 4 | amp |
| TCGA-04-1343 | 19 | 51825296 | 51914648 | 2.393589443 | 5 | amp |
| TCGA-04-1343 | 19 | 51916931 | 51920876 | 1.248331029 | 4 | amp |
| TCGA-04-1343 | 19 | 51955581 | 51981958 | 2.354340937 | 5 | amp |
| TCGA-04-1343 | 19 | 51983580 | 54264491 | 1.465165361 | 4 | amp |
| TCGA-04-1343 | 19 | 54265580 | 54304685 | 2.414431672 | 5 | amp |
| TCGA-04-1343 | 19 | 54307170 | 54327463 | 1.621964019 | 4 | amp |
| TCGA-04-1343 | 19 | 54376727 | 54403592 | 2.706794274 | 5 | amp |
| TCGA-04-1343 | 19 | 54403613 | 54610536 | 1.628923391 | 4 | amp |
| TCGA-04-1343 | 19 | 54611291 | 54723029 | 2.918160478 | 5 | amp |
| TCGA-04-1343 | 19 | 54724368 | 54876454 | 1.555501118 | 4 | amp |
| TCGA-04-1343 | 19 | 54932446 | 54969716 | 3.942286549 | 5 | amp |
| TCGA-04-1343 | 19 | 54973179 | 55021817 | 1.562371349 | 4 | amp |
| TCGA-04-1343 | 19 | 55043941 | 55367400 | 1.057831985 | 3 | amp |
| TCGA-04-1343 | 19 | 55370498 | 55418202 | 1.583725078 | 4 | amp |
| TCGA-04-1343 | 19 | 55420532 | 55568158 | 2.656140195 | 5 | amp |
| TCGA-04-1343 | 19 | 55590336 | 55671383 | 2.089057105 | 4 | amp |
| TCGA-04-1343 | 19 | 55671925 | 55703159 | 2.621189285 | 5 | amp |
| TCGA-04-1343 | 19 | 55707842 | 55753650 | 1.458069014 | 4 | amp |
| TCGA-04-1343 | 19 | 55753739 | 56220463 | 3.149942279 | 5 | amp |
| TCGA-04-1343 | 19 | 56228058 | 57029958 | 1.472714765 | 4 | amp |
| TCGA-04-1343 | 19 | 57035664 | 57184296 | 2.852689013 | 5 | amp |
| TCGA-04-1343 | 19 | 57285966 | 57988080 | 1.628824017 | 4 | amp |
| TCGA-04-1343 | 19 | 57988570 | 58016778 | 2.448533135 | 5 | amp |

| | | | | | | |
|---|---|---|---|---|---|---|
| TCGA-04-1343 | 19 | 58017688 | 58067781 | 1.638908048 | 4 | amp |
| TCGA-04-1343 | 19 | 58083437 | 58145289 | 2.930895156 | 5 | amp |
| TCGA-04-1343 | 19 | 58145319 | 58445247 | 1.475188658 | 4 | amp |
| TCGA-04-1343 | 19 | 58452253 | 58483911 | 2.396972351 | 5 | amp |
| TCGA-04-1343 | 19 | 58485458 | 58500145 | 1.380733028 | 4 | amp |
| TCGA-04-1343 | 19 | 58511129 | 58723108 | 2.895669019 | 5 | amp |
| TCGA-04-1343 | 19 | 58723477 | 58948617 | 1.83415093 | 4 | amp |
| TCGA-04-1343 | 19 | 58956769 | 59082798 | 2.55575998 | 5 | amp |
| TCGA-04-1343 | 19 | 59110398 | 59110878 | 1.223012283 | 4 | amp |
| TCGA-04-1343 | 2 | 41527 | 224951 | 1.664194372 | 4 | amp |
| TCGA-04-1343 | 2 | 229914 | 242663265 | 1.057833251 | 3 | amp |
| TCGA-04-1343 | 2 | 242684087 | 243160772 | 1.417981534 | 4 | amp |
| TCGA-04-1343 | 20 | 68319 | 368979 | 1.694638318 | 4 | amp |
| TCGA-04-1343 | 20 | 371866 | 425793 | 2.538002328 | 5 | amp |
| TCGA-04-1343 | 20 | 428505 | 2634041 | 1.344667103 | 4 | amp |
| TCGA-04-1343 | 20 | 2634804 | 2847267 | 2.437868921 | 5 | amp |
| TCGA-04-1343 | 20 | 2944899 | 3003522 | 1.748205585 | 4 | amp |
| TCGA-04-1343 | 20 | 3005072 | 3236874 | 2.804240064 | 5 | amp |
| TCGA-04-1343 | 20 | 3240102 | 3624917 | 0.960432409 | 3 | amp |
| TCGA-04-1343 | 20 | 3627326 | 3842184 | 3.2647976 | 5 | amp |
| TCGA-04-1343 | 20 | 3842830 | 5975050 | 1.252226438 | 4 | amp |
| TCGA-04-1343 | 20 | 5990369 | 6058026 | 2.523234693 | 5 | amp |
| TCGA-04-1343 | 20 | 6060003 | 9353782 | 1.295263744 | 4 | amp |
| TCGA-04-1343 | 20 | 9360694 | 9382246 | 2.491203011 | 5 | amp |
| TCGA-04-1343 | 20 | 9385917 | 17492726 | 1.317992947 | 4 | amp |
| TCGA-04-1343 | 20 | 17495323 | 17587796 | 2.452257267 | 5 | amp |
| TCGA-04-1343 | 20 | 17594755 | 19937474 | 1.51636528 | 4 | amp |
| TCGA-04-1343 | 20 | 19941339 | 20003171 | 2.377719839 | 5 | amp |
| TCGA-04-1343 | 20 | 20006275 | 25194090 | 1.408491668 | 4 | amp |
| TCGA-04-1343 | 20 | 25195480 | 25206243 | 2.724569063 | 5 | amp |
| TCGA-04-1343 | 20 | 25239833 | 25263922 | 1.801137129 | 4 | amp |
| TCGA-04-1343 | 20 | 25264663 | 25301014 | 2.505485049 | 5 | amp |
| TCGA-04-1343 | 20 | 25303953 | 30028590 | 1.438930948 | 4 | amp |
| TCGA-04-1343 | 20 | 30037784 | 30310039 | 3.201511622 | 5 | amp |
| TCGA-04-1343 | 20 | 30345272 | 30388887 | 1.306996032 | 4 | amp |
| TCGA-04-1343 | 20 | 30411242 | 30460876 | 3.296236415 | 5 | amp |
| TCGA-04-1343 | 20 | 30474960 | 30522712 | 1.455229106 | 4 | amp |
| TCGA-04-1343 | 20 | 30525165 | 30610650 | 2.533689121 | 5 | amp |
| TCGA-04-1343 | 20 | 30616742 | 30790031 | 1.743413218 | 4 | amp |
| TCGA-04-1343 | 20 | 30797814 | 30816281 | 2.636487357 | 5 | amp |
| TCGA-04-1343 | 20 | 30818532 | 30904737 | 1.78992348 | 4 | amp |
| TCGA-04-1343 | 20 | 30914570 | 31294593 | 2.433124889 | 5 | amp |
| TCGA-04-1343 | 20 | 31315690 | 31680483 | 1.746045046 | 4 | amp |
| TCGA-04-1343 | 20 | 31682872 | 31768405 | 2.951705086 | 5 | amp |
| TCGA-04-1343 | 20 | 31805285 | 31893899 | 1.628625863 | 4 | amp |
| TCGA-04-1343 | 20 | 31894664 | 31984898 | 2.814051574 | 5 | amp |
| TCGA-04-1343 | 20 | 31996298 | 32224539 | 1.493280654 | 4 | amp |
| TCGA-04-1343 | 20 | 32228121 | 32308016 | 3.334449502 | 5 | amp |
| TCGA-04-1343 | 20 | 32328702 | 32666392 | 2.113366876 | 4 | amp |
| TCGA-04-1343 | 20 | 32677503 | 32869025 | 3.135285013 | 5 | amp |

| | | | | | | |
|---|---|---|---|---|---|---|
| TCGA-04-1343 | 20 | 32873192 | 33440386 | 1.651127061 | 4 | amp |
| TCGA-04-1343 | 20 | 33442287 | 33507378 | 3.076357475 | 5 | amp |
| TCGA-04-1343 | 20 | 33508306 | 33567305 | 1.863231914 | 4 | amp |
| TCGA-04-1343 | 20 | 33567415 | 33586739 | 3.070568718 | 5 | amp |
| TCGA-04-1343 | 20 | 33588023 | 33732861 | 1.494201702 | 4 | amp |
| TCGA-04-1343 | 20 | 33734622 | 33876797 | 3.470153671 | 5 | amp |
| TCGA-04-1343 | 20 | 33891654 | 34257635 | 1.805954099 | 4 | amp |
| TCGA-04-1343 | 20 | 34260631 | 34284400 | 2.709828727 | 5 | amp |
| TCGA-04-1343 | 20 | 34285543 | 34568582 | 1.597547618 | 4 | amp |
| TCGA-04-1343 | 20 | 34571861 | 34611734 | 2.509165945 | 5 | amp |
| TCGA-04-1343 | 20 | 34618244 | 35176635 | 1.9343662 | 4 | amp |
| TCGA-04-1343 | 20 | 35177445 | 35240639 | 3.306753209 | 5 | amp |
| TCGA-04-1343 | 20 | 35242178 | 35350169 | 1.895768273 | 4 | amp |
| TCGA-04-1343 | 20 | 35381185 | 35433341 | 2.783092445 | 5 | amp |
| TCGA-04-1343 | 20 | 35434247 | 35569537 | 1.621243138 | 4 | amp |
| TCGA-04-1343 | 20 | 35575083 | 35651260 | 2.806847275 | 5 | amp |
| TCGA-04-1343 | 20 | 35661053 | 35675644 | 1.876039223 | 4 | amp |
| TCGA-04-1343 | 20 | 35683947 | 35800507 | 3.18140842 | 5 | amp |
| TCGA-04-1343 | 20 | 35802338 | 36500430 | 1.581422622 | 4 | amp |
| TCGA-04-1343 | 20 | 36560001 | 36669021 | 2.594938602 | 5 | amp |
| TCGA-04-1343 | 20 | 36676696 | 36718322 | 1.375118879 | 4 | amp |
| TCGA-04-1343 | 20 | 36758575 | 36846826 | 2.603070548 | 5 | amp |
| TCGA-04-1343 | 20 | 36847950 | 36965626 | 1.881336898 | 4 | amp |
| TCGA-04-1343 | 20 | 36974831 | 37145043 | 3.46440793 | 5 | amp |
| TCGA-04-1343 | 20 | 37146102 | 37210075 | 1.389237369 | 4 | amp |
| TCGA-04-1343 | 20 | 37214670 | 37395070 | 2.598512289 | 5 | amp |
| TCGA-04-1343 | 20 | 37396052 | 37518332 | 1.605080395 | 4 | amp |
| TCGA-04-1343 | 20 | 37524160 | 37597910 | 2.794443887 | 5 | amp |
| TCGA-04-1343 | 20 | 37601187 | 39976299 | 1.564638654 | 4 | amp |
| TCGA-04-1343 | 20 | 39977212 | 39993855 | 2.728467231 | 5 | amp |
| TCGA-04-1343 | 20 | 40033200 | 42089256 | 1.509401339 | 4 | amp |
| TCGA-04-1343 | 20 | 42089282 | 42159543 | 2.738637527 | 5 | amp |
| TCGA-04-1343 | 20 | 42161368 | 42195183 | 1.680169662 | 4 | amp |
| TCGA-04-1343 | 20 | 42195672 | 42247682 | 2.689625931 | 5 | amp |
| TCGA-04-1343 | 20 | 42249470 | 42252727 | 1.449695211 | 4 | amp |
| TCGA-04-1343 | 20 | 42264549 | 42320997 | 3.762483163 | 5 | amp |
| TCGA-04-1343 | 20 | 42328360 | 42697367 | 1.7340645 | 4 | amp |
| TCGA-04-1343 | 20 | 42747053 | 43129125 | 3.30971451 | 5 | amp |
| TCGA-04-1343 | 20 | 43129707 | 43253026 | 1.71990017 | 4 | amp |
| TCGA-04-1343 | 20 | 43255048 | 43585136 | 2.857575293 | 5 | amp |
| TCGA-04-1343 | 20 | 43600698 | 43681828 | 1.217929639 | 4 | amp |
| TCGA-04-1343 | 20 | 43703587 | 43743741 | 3.199600451 | 5 | amp |
| TCGA-04-1343 | 20 | 43752392 | 43883201 | 1.544281428 | 4 | amp |
| TCGA-04-1343 | 20 | 43922511 | 43964610 | 3.416792468 | 5 | amp |
| TCGA-04-1343 | 20 | 43994232 | 44006036 | 1.611450532 | 4 | amp |
| TCGA-04-1343 | 20 | 44006155 | 44108777 | 2.609576322 | 5 | amp |
| TCGA-04-1343 | 20 | 44163034 | 44238834 | 1.730830557 | 4 | amp |
| TCGA-04-1343 | 20 | 44258437 | 44279249 | 2.778705705 | 5 | amp |
| TCGA-04-1343 | 20 | 44313413 | 44333653 | 1.815045095 | 4 | amp |
| TCGA-04-1343 | 20 | 44334462 | 44473100 | 3.343236237 | 5 | amp |

| | | | | | | |
|---|---|---|---|---|---|---|
| TCGA-04-1343 | 20 | 44477051 | 44512724 | 1.803668264 | 4 | amp |
| TCGA-04-1343 | 20 | 44515079 | 44747067 | 3.676145786 | 5 | amp |
| TCGA-04-1343 | 20 | 44750407 | 44751901 | 1.325231938 | 4 | amp |
| TCGA-04-1343 | 20 | 44755249 | 45023142 | 2.906183809 | 5 | amp |
| TCGA-04-1343 | 20 | 45129875 | 45771802 | 1.660941852 | 4 | amp |
| TCGA-04-1343 | 20 | 45797770 | 45923526 | 2.526801717 | 5 | amp |
| TCGA-04-1343 | 20 | 45927461 | 46279813 | 1.862017409 | 4 | amp |
| TCGA-04-1343 | 20 | 46279901 | 47305388 | 3.692864281 | 5 | amp |
| TCGA-04-1343 | 20 | 47307439 | 47361742 | 1.992900226 | 4 | amp |
| TCGA-04-1343 | 20 | 47364321 | 47580460 | 2.750294798 | 5 | amp |
| TCGA-04-1343 | 20 | 47582364 | 47635537 | 1.849091316 | 4 | amp |
| TCGA-04-1343 | 20 | 47639538 | 47679929 | 3.279287833 | 5 | amp |
| TCGA-04-1343 | 20 | 47682719 | 47752478 | 1.735874303 | 4 | amp |
| TCGA-04-1343 | 20 | 47768081 | 48127760 | 3.065301129 | 5 | amp |
| TCGA-04-1343 | 20 | 48129587 | 48130967 | 1.304690538 | 4 | amp |
| TCGA-04-1343 | 20 | 48140564 | 48253988 | 2.721555167 | 5 | amp |
| TCGA-04-1343 | 20 | 48256130 | 48481388 | 2.053372328 | 4 | amp |
| TCGA-04-1343 | 20 | 48491149 | 50226731 | 3.610322755 | 5 | amp |
| TCGA-04-1343 | 20 | 50230173 | 50342474 | 1.912391553 | 4 | amp |
| TCGA-04-1343 | 20 | 50346324 | 50803670 | 2.951960708 | 5 | amp |
| TCGA-04-1343 | 20 | 51869925 | 51873162 | 1.341929613 | 4 | amp |
| TCGA-04-1343 | 20 | 52185608 | 52558089 | 2.480503699 | 5 | amp |
| TCGA-04-1343 | 20 | 52561407 | 52779448 | 1.944078754 | 4 | amp |
| TCGA-04-1343 | 20 | 52780986 | 53092578 | 3.804546946 | 5 | amp |
| TCGA-04-1343 | 20 | 53171465 | 53967929 | 1.618919221 | 4 | amp |
| TCGA-04-1343 | 20 | 54573591 | 54948657 | 2.952040951 | 5 | amp |
| TCGA-04-1343 | 20 | 54956437 | 54974457 | 1.377572577 | 4 | amp |
| TCGA-04-1343 | 20 | 54978443 | 55088487 | 2.969936113 | 5 | amp |
| TCGA-04-1343 | 20 | 55091927 | 55108701 | 1.665678013 | 4 | amp |
| TCGA-04-1343 | 20 | 55111180 | 55953171 | 3.277735512 | 5 | amp |
| TCGA-04-1343 | 20 | 56063329 | 56078691 | 1.9543421 | 4 | amp |
| TCGA-04-1343 | 20 | 56083632 | 56138297 | 3.440295546 | 5 | amp |
| TCGA-04-1343 | 20 | 56138581 | 56140872 | 1.552101395 | 4 | amp |
| TCGA-04-1343 | 20 | 56179576 | 56886198 | 3.65688437 | 5 | amp |
| TCGA-04-1343 | 20 | 56918724 | 56934770 | 1.653934178 | 4 | amp |
| TCGA-04-1343 | 20 | 56993222 | 57876800 | 3.205978898 | 5 | amp |
| TCGA-04-1343 | 20 | 57896039 | 58494659 | 1.502443725 | 4 | amp |
| TCGA-04-1343 | 20 | 58495357 | 58533809 | 2.642447472 | 5 | amp |
| TCGA-04-1343 | 20 | 58543990 | 58587803 | 1.777888845 | 4 | amp |
| TCGA-04-1343 | 20 | 58645003 | 60485690 | 2.386062263 | 5 | amp |
| TCGA-04-1343 | 20 | 60498425 | 60504903 | 1.802973024 | 4 | amp |
| TCGA-04-1343 | 20 | 60572545 | 62703318 | 3.780447389 | 5 | amp |
| TCGA-04-1343 | 20 | 62703468 | 62708058 | 1.635570522 | 4 | amp |
| TCGA-04-1343 | 20 | 62830181 | 62926333 | 4.289154995 | 5 | amp |
| TCGA-04-1343 | 21 | 9483321 | 11029657 | 0.999287762 | 3 | amp |
| TCGA-04-1343 | 21 | 11097535 | 11097655 | 1.370302975 | 3 | amp |
| TCGA-04-1343 | 21 | 11098669 | 47987588 | 1.743367287 | 4 | amp |
| TCGA-04-1343 | 21 | 48019225 | 48111215 | 2.675389095 | 5 | amp |
| TCGA-04-1343 | 22 | 16084594 | 51237627 | 1.543012055 | 4 | amp |
| TCGA-04-1343 | 3 | 361444 | 9547948 | 1.008863681 | 3 | amp |

| | | | | | | |
|---|---|---|---|---|---|---|
| TCGA-04-1343 | 3 | 9695258 | 9972662 | 2.60117058 | 5 | amp |
| TCGA-04-1343 | 3 | 9974249 | 11076420 | 1.653496102 | 4 | amp |
| TCGA-04-1343 | 3 | 11078509 | 46723611 | 1.022540735 | 3 | amp |
| TCGA-04-1343 | 3 | 46724597 | 50617889 | 1.659655077 | 4 | amp |
| TCGA-04-1343 | 3 | 50677766 | 51697478 | 0.946344977 | 3 | amp |
| TCGA-04-1343 | 3 | 51708269 | 52822364 | 1.735559256 | 4 | amp |
| TCGA-04-1343 | 3 | 52823649 | 112714166 | 1.096133146 | 3 | amp |
| TCGA-04-1343 | 3 | 112715728 | 127318395 | 1.421528509 | 4 | amp |
| TCGA-04-1343 | 3 | 127323688 | 127340620 | 3.869099031 | 5 | amp |
| TCGA-04-1343 | 3 | 127358046 | 127774451 | 1.746621338 | 4 | amp |
| TCGA-04-1343 | 3 | 127774494 | 127786923 | 3.891542066 | 5 | amp |
| TCGA-04-1343 | 3 | 127788261 | 129818210 | 1.903317072 | 4 | amp |
| TCGA-04-1343 | 3 | 129822656 | 132423248 | 1.140942729 | 3 | amp |
| TCGA-04-1343 | 3 | 132424560 | 182858414 | 1.429726208 | 4 | amp |
| TCGA-04-1343 | 3 | 182870106 | 182910895 | 3.818895586 | 5 | amp |
| TCGA-04-1343 | 3 | 182912966 | 183854581 | 2.094271146 | 4 | amp |
| TCGA-04-1343 | 3 | 183855380 | 184428703 | 5.511851809 | 5 | amp |
| TCGA-04-1343 | 3 | 184428763 | 186501473 | 2.044788643 | 4 | amp |
| TCGA-04-1343 | 3 | 186502183 | 187451485 | 2.970650304 | 5 | amp |
| TCGA-04-1343 | 3 | 188123854 | 195475973 | 1.79668482 | 4 | amp |
| TCGA-04-1343 | 3 | 195477721 | 196753640 | 3.224529975 | 5 | amp |
| TCGA-04-1343 | 3 | 196771486 | 197955154 | 1.870862091 | 4 | amp |
| TCGA-04-1343 | 4 | 53323 | 4285528 | 1.260643574 | 4 | amp |
| TCGA-04-1343 | 4 | 189063370 | 189063640 | 1.116113192 | 4 | amp |
| TCGA-04-1343 | 4 | 189064912 | 190878664 | 1.107001168 | 3 | amp |
| TCGA-04-1343 | 4 | 190881888 | 190948390 | 1.270403354 | 4 | amp |
| TCGA-04-1343 | 5 | 151610 | 180899507 | 1.257237216 | 4 | amp |
| TCGA-04-1343 | 6 | 105907 | 2749568 | 0.926138625 | 3 | amp |
| TCGA-04-1343 | 6 | 2768900 | 4735096 | 1.507309581 | 4 | amp |
| TCGA-04-1343 | 6 | 4891919 | 29455710 | 1.004271459 | 3 | amp |
| TCGA-04-1343 | 6 | 29523651 | 30297560 | 1.523258613 | 4 | amp |
| TCGA-04-1343 | 6 | 30298545 | 30546203 | 2.325439339 | 5 | amp |
| TCGA-04-1343 | 6 | 30546240 | 30613924 | 1.550361152 | 4 | amp |
| TCGA-04-1343 | 6 | 30617265 | 30630521 | 2.368153379 | 5 | amp |
| TCGA-04-1343 | 6 | 30630682 | 31504488 | 1.637190243 | 4 | amp |
| TCGA-04-1343 | 6 | 31506525 | 31516257 | 2.571027049 | 5 | amp |
| TCGA-04-1343 | 6 | 31525365 | 31560536 | 1.873277346 | 4 | amp |
| TCGA-04-1343 | 6 | 31583028 | 31591717 | 2.902517896 | 5 | amp |
| TCGA-04-1343 | 6 | 31591997 | 31805218 | 1.556605789 | 4 | amp |
| TCGA-04-1343 | 6 | 31807282 | 31839354 | 2.741041123 | 5 | amp |
| TCGA-04-1343 | 6 | 31842216 | 31869108 | 1.228964507 | 4 | amp |
| TCGA-04-1343 | 6 | 31895462 | 31951962 | 3.258178867 | 5 | amp |
| TCGA-04-1343 | 6 | 31951998 | 31963593 | 1.829469563 | 4 | amp |
| TCGA-04-1343 | 6 | 31963689 | 32002392 | 2.513636991 | 5 | amp |
| TCGA-04-1343 | 6 | 32002582 | 32138900 | 1.796050226 | 4 | amp |
| TCGA-04-1343 | 6 | 32139018 | 32156337 | 3.027466555 | 5 | amp |
| TCGA-04-1343 | 6 | 32156446 | 32181090 | 1.588950396 | 4 | amp |
| TCGA-04-1343 | 6 | 32181390 | 32803153 | 1.025793903 | 3 | amp |
| TCGA-04-1343 | 6 | 32803364 | 33371945 | 1.787648315 | 4 | amp |
| TCGA-04-1343 | 6 | 33372568 | 33419729 | 2.890873293 | 5 | amp |

| | | | | | | |
|---|---|---|---|---|---|---|
| TCGA-04-1343 | 6 | 33422800 | 34499590 | 1.584622797 | 4 | amp |
| TCGA-04-1343 | 6 | 34506019 | 35195537 | 0.914497239 | 3 | amp |
| TCGA-04-1343 | 6 | 35196303 | 35393912 | 1.294587188 | 4 | amp |
| TCGA-04-1343 | 6 | 35423436 | 35447147 | 2.776165493 | 5 | amp |
| TCGA-04-1343 | 6 | 35448059 | 35787278 | 1.413830167 | 4 | amp |
| TCGA-04-1343 | 6 | 35803052 | 41556485 | 1.047843056 | 3 | amp |
| TCGA-04-1343 | 6 | 41557460 | 42237353 | 1.609786024 | 4 | amp |
| TCGA-04-1343 | 6 | 42541391 | 42638510 | 0.789585301 | 3 | amp |
| TCGA-04-1343 | 6 | 42641516 | 42847742 | 1.314062969 | 4 | amp |
| TCGA-04-1343 | 6 | 42848591 | 43097575 | 2.474376816 | 5 | amp |
| TCGA-04-1343 | 6 | 43097912 | 43190150 | 1.37100138 | 4 | amp |
| TCGA-04-1343 | 6 | 43190263 | 43227011 | 2.917628749 | 5 | amp |
| TCGA-04-1343 | 6 | 43250447 | 43413077 | 1.50401608 | 4 | amp |
| TCGA-04-1343 | 6 | 43413298 | 43487184 | 3.681555269 | 5 | amp |
| TCGA-04-1343 | 6 | 43487389 | 43742163 | 1.771676621 | 4 | amp |
| TCGA-04-1343 | 6 | 43745183 | 44119783 | 2.911944861 | 5 | amp |
| TCGA-04-1343 | 6 | 44120292 | 44151715 | 1.263510448 | 4 | amp |
| TCGA-04-1343 | 6 | 44194974 | 44278934 | 2.98106756 | 5 | amp |
| TCGA-04-1343 | 6 | 44279049 | 44361366 | 1.541070754 | 4 | amp |
| TCGA-04-1343 | 6 | 44364067 | 109484182 | 0.87921253 | 3 | amp |
| TCGA-04-1343 | 6 | 109689986 | 109762852 | 1.683189952 | 4 | amp |
| TCGA-04-1343 | 6 | 109763133 | 109803233 | 2.850758747 | 5 | amp |
| TCGA-04-1343 | 6 | 109814552 | 137519831 | 1.033167389 | 3 | amp |
| TCGA-04-1343 | 6 | 137521961 | 138734235 | 1.498801559 | 4 | amp |
| TCGA-04-1343 | 6 | 139094775 | 144263840 | 0.978414925 | 3 | amp |
| TCGA-04-1343 | 6 | 144269077 | 144835090 | 1.807754255 | 4 | amp |
| TCGA-04-1343 | 6 | 144835155 | 150016403 | 0.980620148 | 3 | amp |
| TCGA-04-1343 | 6 | 150018232 | 150212054 | 1.952545097 | 4 | amp |
| TCGA-04-1343 | 6 | 150239294 | 150387346 | 2.330319841 | 5 | amp |
| TCGA-04-1343 | 6 | 150535831 | 151785790 | 1.263481176 | 4 | amp |
| TCGA-04-1343 | 6 | 151789441 | 159636199 | 1.041418954 | 3 | amp |
| TCGA-04-1343 | 6 | 159642618 | 160189721 | 1.50443444 | 4 | amp |
| TCGA-04-1343 | 6 | 160196153 | 160238221 | 2.518705055 | 5 | amp |
| TCGA-04-1343 | 6 | 160239576 | 165989977 | 1.015900641 | 3 | amp |
| TCGA-04-1343 | 6 | 166571759 | 167787948 | 1.438967397 | 4 | amp |
| TCGA-04-1343 | 6 | 167789449 | 171055029 | 0.938453987 | 3 | amp |
| TCGA-04-1343 | 7 | 540695 | 2406701 | 2.337384895 | 5 | amp |
| TCGA-04-1343 | 7 | 2409061 | 6634268 | 1.519378103 | 4 | amp |
| TCGA-04-1343 | 7 | 6639423 | 6785829 | 2.331171576 | 5 | amp |
| TCGA-04-1343 | 7 | 6786631 | 6863822 | 1.535942024 | 4 | amp |
| TCGA-04-1343 | 7 | 6864072 | 23213955 | 0.866959536 | 3 | amp |
| TCGA-04-1343 | 7 | 23224652 | 23352470 | 1.594455652 | 4 | amp |
| TCGA-04-1343 | 7 | 23353086 | 26894516 | 1.033026779 | 3 | amp |
| TCGA-04-1343 | 7 | 26903924 | 27203520 | 2.420289799 | 5 | amp |
| TCGA-04-1343 | 7 | 27211444 | 27566031 | 1.52440588 | 4 | amp |
| TCGA-04-1343 | 7 | 27570768 | 30469091 | 1.010424733 | 3 | amp |
| TCGA-04-1343 | 7 | 30472693 | 31120297 | 1.32996283 | 4 | amp |
| TCGA-04-1343 | 7 | 31121276 | 43846884 | 0.921347585 | 3 | amp |
| TCGA-04-1343 | 7 | 43906259 | 44611392 | 1.620632898 | 4 | amp |
| TCGA-04-1343 | 7 | 44611930 | 44621431 | 2.380135873 | 5 | amp |

| | | | | | | |
|---|---|---|---|---|---|---|
| TCGA-04-1343 | 7 | 44663903 | 44747657 | 0.923255403 | 3 | amp |
| TCGA-04-1343 | 7 | 44795813 | 45109612 | 1.665248837 | 4 | amp |
| TCGA-04-1343 | 7 | 45112293 | 47927787 | 0.879907127 | 3 | amp |
| TCGA-04-1343 | 7 | 47930100 | 48028490 | 1.617310228 | 4 | amp |
| TCGA-04-1343 | 7 | 48033845 | 56007726 | 0.915875198 | 3 | amp |
| TCGA-04-1343 | 7 | 56020799 | 66237095 | 1.336908587 | 4 | amp |
| TCGA-04-1343 | 7 | 66240176 | 72277984 | 1.09712905 | 3 | amp |
| TCGA-04-1343 | 7 | 72281034 | 72397540 | 1.39399832 | 4 | amp |
| TCGA-04-1343 | 7 | 72398872 | 72471062 | 2.763831154 | 5 | amp |
| TCGA-04-1343 | 7 | 72473505 | 72685718 | 1.349420773 | 4 | amp |
| TCGA-04-1343 | 7 | 72693953 | 72733056 | 2.264632477 | 5 | amp |
| TCGA-04-1343 | 7 | 72734133 | 74251511 | 1.623732908 | 4 | amp |
| TCGA-04-1343 | 7 | 74298949 | 74301318 | 2.813329002 | 5 | amp |
| TCGA-04-1343 | 7 | 74303765 | 74910447 | 1.473562577 | 4 | amp |
| TCGA-04-1343 | 7 | 74911430 | 74991396 | 0.940706063 | 3 | amp |
| TCGA-04-1343 | 7 | 74993843 | 75045079 | 2.86219512 | 5 | amp |
| TCGA-04-1343 | 7 | 75045503 | 75511590 | 1.66974136 | 4 | amp |
| TCGA-04-1343 | 7 | 75512940 | 75633184 | 2.621912201 | 5 | amp |
| TCGA-04-1343 | 7 | 75634550 | 76641386 | 1.634426606 | 4 | amp |
| TCGA-04-1343 | 7 | 76643237 | 95024050 | 0.862856954 | 3 | amp |
| TCGA-04-1343 | 7 | 95025535 | 95439820 | 1.822821745 | 4 | amp |
| TCGA-04-1343 | 7 | 95442458 | 96339097 | 0.891407528 | 3 | amp |
| TCGA-04-1343 | 7 | 96639053 | 98985892 | 1.378526628 | 4 | amp |
| TCGA-04-1343 | 7 | 98987491 | 99077459 | 2.728422453 | 5 | amp |
| TCGA-04-1343 | 7 | 99079744 | 99274234 | 1.411871251 | 4 | amp |
| TCGA-04-1343 | 7 | 99277423 | 99474710 | 0.880791835 | 3 | amp |
| TCGA-04-1343 | 7 | 99489777 | 99675016 | 1.635458606 | 4 | amp |
| TCGA-04-1343 | 7 | 99686825 | 99764775 | 2.593062142 | 5 | amp |
| TCGA-04-1343 | 7 | 99769654 | 100031210 | 1.369882381 | 4 | amp |
| TCGA-04-1343 | 7 | 100032919 | 100491859 | 2.621496227 | 5 | amp |
| TCGA-04-1343 | 7 | 100549479 | 100780764 | 1.373350006 | 4 | amp |
| TCGA-04-1343 | 7 | 100799843 | 100962334 | 2.948872695 | 5 | amp |
| TCGA-04-1343 | 7 | 101063198 | 102079635 | 1.799699724 | 4 | amp |
| TCGA-04-1343 | 7 | 102086899 | 102141728 | 2.371080066 | 5 | amp |
| TCGA-04-1343 | 7 | 102194714 | 102201652 | 1.058236751 | 4 | amp |
| TCGA-04-1343 | 7 | 102208423 | 102235870 | 2.537736085 | 5 | amp |
| TCGA-04-1343 | 7 | 102293806 | 102300742 | 1.032788144 | 3 | amp |
| TCGA-04-1343 | 7 | 102307503 | 102330919 | 2.808842745 | 5 | amp |
| TCGA-04-1343 | 7 | 102343839 | 105429161 | 0.88037532 | 3 | amp |
| TCGA-04-1343 | 7 | 105516231 | 105733608 | 1.560913517 | 4 | amp |
| TCGA-04-1343 | 7 | 105738119 | 127638092 | 0.951635954 | 3 | amp |
| TCGA-04-1343 | 7 | 127668682 | 128694884 | 1.539394763 | 4 | amp |
| TCGA-04-1343 | 7 | 128801458 | 128849243 | 2.258708476 | 5 | amp |
| TCGA-04-1343 | 7 | 128850146 | 129311385 | 1.664493142 | 4 | amp |
| TCGA-04-1343 | 7 | 129317414 | 129944428 | 1.054601204 | 3 | amp |
| TCGA-04-1343 | 7 | 129945647 | 130370096 | 1.428325499 | 4 | amp |
| TCGA-04-1343 | 7 | 130561477 | 134650156 | 1.03115456 | 3 | amp |
| TCGA-04-1343 | 7 | 134653003 | 134939981 | 1.904614392 | 4 | amp |
| TCGA-04-1343 | 7 | 135047577 | 139107117 | 0.918449378 | 3 | amp |
| TCGA-04-1343 | 7 | 139138272 | 140404756 | 1.484632114 | 4 | amp |

| | | | | | | |
|---|---|---|---|---|---|---|
| TCGA-04-1343 | 7 | 140434333 | 142499157 | 0.979457174 | 3 | amp |
| TCGA-04-1343 | 7 | 142499576 | 143573751 | 1.462755574 | 4 | amp |
| TCGA-04-1343 | 7 | 143632322 | 148544451 | 0.959039337 | 3 | amp |
| TCGA-04-1343 | 7 | 148700823 | 150649966 | 1.568954629 | 4 | amp |
| TCGA-04-1343 | 7 | 150656621 | 150918774 | 2.670661945 | 5 | amp |
| TCGA-04-1343 | 7 | 150919564 | 151135385 | 1.86725962 | 4 | amp |
| TCGA-04-1343 | 7 | 151164160 | 154785517 | 0.940709357 | 3 | amp |
| TCGA-04-1343 | 7 | 154790306 | 156451269 | 1.464890365 | 4 | amp |
| TCGA-04-1343 | 7 | 156468323 | 158935247 | 0.990421935 | 3 | amp |
| TCGA-04-1343 | 8 | 116074 | 2088847 | 1.44035837 | 4 | amp |
| TCGA-04-1343 | 8 | 2089021 | 7056187 | 1.054068137 | 3 | amp |
| TCGA-04-1343 | 8 | 7116415 | 12973190 | 1.45770869 | 4 | amp |
| TCGA-04-1343 | 8 | 13072058 | 19363388 | 1.00741322 | 3 | amp |
| TCGA-04-1343 | 8 | 19675759 | 21892097 | 1.426359427 | 4 | amp |
| TCGA-04-1343 | 8 | 21903593 | 22081917 | 2.646258326 | 5 | amp |
| TCGA-04-1343 | 8 | 22084330 | 22426843 | 1.604743553 | 4 | amp |
| TCGA-04-1343 | 8 | 22429222 | 22473822 | 2.525792799 | 5 | amp |
| TCGA-04-1343 | 8 | 22474854 | 23069736 | 1.436495339 | 4 | amp |
| TCGA-04-1343 | 8 | 23106688 | 23159727 | 2.427209735 | 5 | amp |
| TCGA-04-1343 | 8 | 23160762 | 23539182 | 1.528015426 | 4 | amp |
| TCGA-04-1343 | 8 | 23702237 | 26223902 | 1.053507357 | 3 | amp |
| TCGA-04-1343 | 8 | 26227609 | 27534103 | 1.400609955 | 4 | amp |
| TCGA-04-1343 | 8 | 27593687 | 33345980 | 1.036220913 | 3 | amp |
| TCGA-04-1343 | 8 | 33346002 | 37735087 | 1.671057707 | 4 | amp |
| TCGA-04-1343 | 8 | 37791787 | 37821806 | 2.352027683 | 5 | amp |
| TCGA-04-1343 | 8 | 37914568 | 38834275 | 1.493249891 | 4 | amp |
| TCGA-04-1343 | 8 | 38835448 | 38851181 | 2.651224597 | 5 | amp |
| TCGA-04-1343 | 8 | 38852809 | 40554961 | 1.032702897 | 3 | amp |
| TCGA-04-1343 | 8 | 40625144 | 41467503 | 1.750784309 | 4 | amp |
| TCGA-04-1343 | 8 | 41468231 | 41563769 | 2.788561386 | 5 | amp |
| TCGA-04-1343 | 8 | 41566244 | 43157304 | 1.55803961 | 4 | amp |
| TCGA-04-1343 | 8 | 43159846 | 48852303 | 1.02609487 | 3 | amp |
| TCGA-04-1343 | 8 | 48855727 | 49833844 | 1.785060504 | 4 | amp |
| TCGA-04-1343 | 8 | 49985356 | 54963779 | 1.069156698 | 3 | amp |
| TCGA-04-1343 | 8 | 54965205 | 59345820 | 1.45742382 | 4 | amp |
| TCGA-04-1343 | 8 | 59346918 | 63936751 | 0.980533453 | 3 | amp |
| TCGA-04-1343 | 8 | 63938665 | 67380605 | 1.403637175 | 4 | amp |
| TCGA-04-1343 | 8 | 67404492 | 67425883 | 2.721088742 | 5 | amp |
| TCGA-04-1343 | 8 | 67428069 | 68004137 | 1.41383294 | 4 | amp |
| TCGA-04-1343 | 8 | 68005766 | 70517222 | 1.051344129 | 3 | amp |
| TCGA-04-1343 | 8 | 70533257 | 71012870 | 1.589751705 | 4 | amp |
| TCGA-04-1343 | 8 | 71015769 | 86129731 | 1.182713824 | 3 | amp |
| TCGA-04-1343 | 8 | 86157985 | 87568627 | 1.408684735 | 4 | amp |
| TCGA-04-1343 | 8 | 87570456 | 95885713 | 1.112138106 | 3 | amp |
| TCGA-04-1343 | 8 | 95886744 | 97285662 | 1.522569646 | 4 | amp |
| TCGA-04-1343 | 8 | 97296268 | 124142625 | 1.025332768 | 3 | amp |
| TCGA-04-1343 | 8 | 124146309 | 124195636 | 1.497775217 | 4 | amp |
| TCGA-04-1343 | 8 | 124203976 | 124250229 | 2.508517775 | 5 | amp |
| TCGA-04-1343 | 8 | 124251276 | 124351723 | 1.403869328 | 4 | amp |
| TCGA-04-1343 | 8 | 124357096 | 134203481 | 1.077298901 | 3 | amp |

| | | | | | | |
|---|---|---|---|---|---|---|
| TCGA-04-1343 | 8 | 134225066 | 135522046 | 1.721330753 | 4 | amp |
| TCGA-04-1343 | 8 | 135524660 | 139890575 | 1.10998686 | 3 | amp |
| TCGA-04-1343 | 8 | 140630441 | 141449343 | 1.475983118 | 4 | amp |
| TCGA-04-1343 | 8 | 141460855 | 141554416 | 3.300284367 | 5 | amp |
| TCGA-04-1343 | 8 | 141557496 | 141675115 | 1.611880802 | 4 | amp |
| TCGA-04-1343 | 8 | 141678295 | 142170928 | 0.973923819 | 3 | amp |
| TCGA-04-1343 | 8 | 142173389 | 144408572 | 1.818986647 | 4 | amp |
| TCGA-04-1343 | 8 | 144411430 | 145268080 | 2.805953197 | 5 | amp |
| TCGA-04-1343 | 8 | 145293652 | 146279593 | 1.831323468 | 4 | amp |
| TCGA-04-1343 | 9 | 33026428 | 33038911 | 1.794162421 | 4 | amp |
| TCGA-04-1343 | 9 | 33047279 | 34339220 | 0.982751209 | 3 | amp |
| TCGA-04-1343 | 9 | 34343069 | 35108304 | 1.839443573 | 4 | amp |
| TCGA-04-1343 | 9 | 35147806 | 35558571 | 0.850192598 | 3 | amp |
| TCGA-04-1343 | 9 | 35559185 | 35675609 | 1.920756158 | 4 | amp |
| TCGA-04-1343 | 9 | 35676011 | 35685360 | 3.754029801 | 5 | amp |
| TCGA-04-1343 | 9 | 35685369 | 35826219 | 2.097508981 | 4 | amp |
| TCGA-04-1343 | 9 | 35842364 | 36117197 | 0.929946165 | 3 | amp |
| TCGA-04-1343 | 9 | 36118738 | 36204180 | 1.619883466 | 4 | amp |
| TCGA-04-1343 | 9 | 36209230 | 39461743 | 0.926991726 | 3 | amp |
| TCGA-04-1343 | 9 | 88844395 | 93650220 | 0.946374268 | 3 | amp |
| TCGA-04-1343 | 9 | 95778004 | 100845352 | 0.971878972 | 3 | amp |
| TCGA-04-1343 | 9 | 100849503 | 101061690 | 1.506379706 | 4 | amp |
| TCGA-04-1343 | 9 | 101065493 | 101801042 | 0.987518474 | 3 | amp |
| TCGA-04-1343 | 9 | 115818752 | 116085482 | 0.88673577 | 3 | amp |
| TCGA-04-1343 | 9 | 116091137 | 116188026 | 1.451551467 | 4 | amp |
| TCGA-04-1343 | 9 | 116190374 | 117401034 | 1.080379332 | 3 | amp |
| TCGA-04-1343 | 9 | 123632678 | 123676050 | 2.044488125 | 4 | amp |
| TCGA-04-1343 | 9 | 123676455 | 123751421 | 0.950131873 | 3 | amp |
| TCGA-04-1343 | 9 | 123949173 | 125622422 | 1.076963488 | 3 | amp |
| TCGA-04-1343 | 9 | 126776220 | 130469510 | 0.955960539 | 3 | amp |
| TCGA-04-1343 | 9 | 130471626 | 131250356 | 1.556307565 | 4 | amp |
| TCGA-04-1343 | 9 | 131254669 | 131454327 | 0.970202966 | 3 | amp |
| TCGA-04-1343 | 9 | 131454932 | 131721186 | 1.636895411 | 4 | amp |
| TCGA-04-1343 | 9 | 131721251 | 131768988 | 0.91596114 | 3 | amp |
| TCGA-04-1343 | 9 | 131770956 | 132636060 | 1.573280346 | 4 | amp |
| TCGA-04-1343 | 9 | 132636824 | 132891099 | 0.875042864 | 3 | amp |
| TCGA-04-1343 | 9 | 132897291 | 133589959 | 1.344322377 | 4 | amp |
| TCGA-04-1343 | 9 | 133729417 | 134361580 | 1.008422289 | 3 | amp |
| TCGA-04-1343 | 9 | 134362497 | 134406072 | 1.794526395 | 4 | amp |
| TCGA-04-1343 | 9 | 134454838 | 135933426 | 0.954492664 | 3 | amp |
| TCGA-04-1343 | 9 | 135939715 | 137717753 | 1.577219603 | 4 | amp |
| TCGA-04-1343 | 9 | 137721795 | 138742090 | 1.097629357 | 3 | amp |
| TCGA-04-1343 | 9 | 138742164 | 140693375 | 1.693510072 | 4 | amp |
| TCGA-04-1343 | 9 | 140705839 | 141071671 | 1.078710181 | 3 | amp |
| TCGA-04-1343 | X | 200797 | 1547060 | 1.232782678 | 3 | amp |
| TCGA-04-1343 | X | 1553885 | 1761921 | 1.712187749 | 4 | amp |
| TCGA-04-1343 | X | 2139055 | 13769547 | 0.975770403 | 3 | amp |
| TCGA-04-1343 | X | 18842002 | 20156788 | 0.89000561 | 3 | amp |
| TCGA-04-1343 | X | 38134323 | 40526157 | 1.024298689 | 3 | amp |
| TCGA-04-1343 | X | 46531963 | 46940744 | 0.90365605 | 3 | amp |

| | | | | | | |
|---|---|---|---|---|---|---|
| TCGA-04-1343 | X | 46949162 | 47518415 | 1.602450455 | 4 | amp |
| TCGA-04-1343 | X | 47528353 | 47867392 | 0.863959334 | 3 | amp |
| TCGA-04-1343 | X | 47867732 | 48025262 | 1.557707528 | 4 | amp |
| TCGA-04-1343 | X | 48046218 | 48370361 | 0.988713981 | 3 | amp |
| TCGA-04-1343 | X | 48371171 | 48386853 | 3.233150283 | 5 | amp |
| TCGA-04-1343 | X | 48399715 | 49072022 | 1.690475284 | 4 | amp |
| TCGA-04-1343 | X | 49072794 | 49077576 | 2.842652419 | 5 | amp |
| TCGA-04-1343 | X | 49078961 | 49370666 | 1.858380255 | 4 | amp |
| TCGA-04-1343 | X | 49452094 | 52977835 | 0.882641106 | 3 | amp |
| TCGA-04-1343 | X | 53113705 | 53278103 | 1.489921727 | 4 | amp |
| TCGA-04-1343 | X | 53279429 | 53432915 | 0.931412535 | 3 | amp |
| TCGA-04-1343 | X | 53435976 | 53458859 | 1.419303156 | 4 | amp |
| TCGA-04-1343 | X | 53458879 | 54823571 | 0.966784079 | 3 | amp |
| TCGA-04-1343 | X | 64738056 | 69496612 | 0.923688806 | 3 | amp |
| TCGA-04-1343 | X | 69497191 | 69674174 | 1.385284218 | 4 | amp |
| TCGA-04-1343 | X | 69698996 | 70342775 | 0.957072009 | 3 | amp |
| TCGA-04-1343 | X | 70342890 | 70470608 | 1.447441046 | 4 | amp |
| TCGA-04-1343 | X | 70471000 | 73751397 | 0.921868456 | 3 | amp |
| TCGA-04-1343 | X | 101159189 | 102193144 | 1.00613128 | 3 | amp |
| TCGA-04-1343 | X | 102317743 | 102347997 | 1.759498607 | 4 | amp |
| TCGA-04-1343 | X | 119673084 | 120117811 | 1.09322663 | 3 | amp |
| TCGA-04-1343 | X | 149930969 | 152097221 | 1.000218006 | 3 | amp |
| TCGA-04-1343 | X | 152100207 | 152773924 | 1.358469834 | 4 | amp |
| TCGA-04-1343 | X | 152806765 | 152937523 | 1.004036233 | 3 | amp |
| TCGA-04-1343 | X | 152937527 | 153228899 | 1.741014958 | 4 | amp |
| TCGA-04-1343 | X | 153229534 | 153533834 | 1.076984654 | 3 | amp |
| TCGA-04-1343 | X | 153537655 | 153880953 | 1.61365955 | 4 | amp |
| TCGA-04-1343 | X | 153906370 | 153944632 | 0.5550258 | 3 | amp |
| TCGA-04-1343 | X | 153993147 | 154020608 | 1.646384423 | 4 | amp |
| TCGA-04-1343 | X | 154065799 | 155240134 | 0.889075197 | 3 | amp |
| TCGA-04-1347 | 1 | 14642 | 19712278 | 1.107356542 | 4 | amp |
| TCGA-04-1347 | 1 | 19746139 | 34330372 | 1.328954658 | 5 | amp |
| TCGA-04-1347 | 1 | 34383648 | 35900708 | 1.970455382 | 6 | amp |
| TCGA-04-1347 | 1 | 35906528 | 48708328 | 1.431428212 | 5 | amp |
| TCGA-04-1347 | 1 | 48712960 | 51702609 | 2.132838819 | 6 | amp |
| TCGA-04-1347 | 1 | 51735592 | 52135204 | 1.638876055 | 5 | amp |
| TCGA-04-1347 | 1 | 52179622 | 52289516 | 1.983258553 | 6 | amp |
| TCGA-04-1347 | 1 | 52290946 | 55512377 | 1.466193741 | 5 | amp |
| TCGA-04-1347 | 1 | 55522945 | 89637643 | 2.131269202 | 6 | amp |
| TCGA-04-1347 | 1 | 89650894 | 90058645 | 1.438121838 | 5 | amp |
| TCGA-04-1347 | 1 | 90151981 | 109618615 | 2.545814385 | 6 | amp |
| TCGA-04-1347 | 1 | 109635457 | 109780050 | 1.354464646 | 5 | amp |
| TCGA-04-1347 | 1 | 109780311 | 109813254 | 2.435263297 | 6 | amp |
| TCGA-04-1347 | 1 | 109813514 | 109839861 | 1.193855355 | 5 | amp |
| TCGA-04-1347 | 1 | 109839991 | 110022240 | 2.206019707 | 6 | amp |
| TCGA-04-1347 | 1 | 110028621 | 110035274 | 1.144287584 | 5 | amp |
| TCGA-04-1347 | 1 | 110037723 | 110210815 | 2.059428839 | 6 | amp |
| TCGA-04-1347 | 1 | 110210994 | 110889012 | 1.518206584 | 5 | amp |
| TCGA-04-1347 | 1 | 110906301 | 113197309 | 2.125767669 | 6 | amp |
| TCGA-04-1347 | 1 | 113201593 | 113456829 | 1.354667141 | 5 | amp |

| | | | | | | |
|---|---|---|---|---|---|---|
| TCGA-04-1347 | 1 | 113459722 | 145416984 | 2.377525523 | 6 | amp |
| TCGA-04-1347 | 1 | 145438717 | 145539464 | 1.420381426 | 5 | amp |
| TCGA-04-1347 | 1 | 145539679 | 145580617 | 2.122776723 | 6 | amp |
| TCGA-04-1347 | 1 | 145581128 | 145592790 | 0.947919528 | 4 | amp |
| TCGA-04-1347 | 1 | 145593993 | 150230610 | 2.33441333 | 6 | amp |
| TCGA-04-1347 | 1 | 150232495 | 150240561 | 0.756557857 | 3 | amp |
| TCGA-04-1347 | 1 | 150241093 | 150919544 | 2.014078435 | 6 | amp |
| TCGA-04-1347 | 1 | 150921525 | 150972035 | 1.116552214 | 5 | amp |
| TCGA-04-1347 | 1 | 150972328 | 151060779 | 2.003368117 | 6 | amp |
| TCGA-04-1347 | 1 | 151062869 | 151146152 | 1.44637709 | 5 | amp |
| TCGA-04-1347 | 1 | 151146794 | 151271596 | 2.231308041 | 6 | amp |
| TCGA-04-1347 | 1 | 151274300 | 151340854 | 1.424314837 | 5 | amp |
| TCGA-04-1347 | 1 | 151341422 | 151414738 | 2.540093051 | 6 | amp |
| TCGA-04-1347 | 1 | 151490951 | 151508388 | 1.455597405 | 5 | amp |
| TCGA-04-1347 | 1 | 151508676 | 153431538 | 2.047078945 | 6 | amp |
| TCGA-04-1347 | 1 | 153507088 | 153964156 | 1.667220908 | 5 | amp |
| TCGA-04-1347 | 1 | 153964464 | 154245285 | 2.267084402 | 6 | amp |
| TCGA-04-1347 | 1 | 154245732 | 154316046 | 1.413533039 | 5 | amp |
| TCGA-04-1347 | 1 | 154316312 | 154514610 | 2.013348893 | 6 | amp |
| TCGA-04-1347 | 1 | 154515161 | 155025990 | 1.577953408 | 5 | amp |
| TCGA-04-1347 | 1 | 155026334 | 155149778 | 1.962445744 | 6 | amp |
| TCGA-04-1347 | 1 | 155150394 | 155188286 | 1.324012278 | 5 | amp |
| TCGA-04-1347 | 1 | 155202499 | 155933028 | 1.952179498 | 6 | amp |
| TCGA-04-1347 | 1 | 155934728 | 156377027 | 1.584148896 | 5 | amp |
| TCGA-04-1347 | 1 | 156384435 | 156901821 | 2.046995048 | 6 | amp |
| TCGA-04-1347 | 1 | 156902208 | 156928945 | 1.480969237 | 5 | amp |
| TCGA-04-1347 | 1 | 156930170 | 159784106 | 2.574935581 | 6 | amp |
| TCGA-04-1347 | 1 | 159785143 | 159828732 | 1.151056191 | 5 | amp |
| TCGA-04-1347 | 1 | 159832196 | 160094272 | 2.316020903 | 6 | amp |
| TCGA-04-1347 | 1 | 160094864 | 160125951 | 1.247701342 | 5 | amp |
| TCGA-04-1347 | 1 | 160128738 | 160160869 | 2.224894593 | 6 | amp |
| TCGA-04-1347 | 1 | 160162543 | 160168553 | 0.706607346 | 3 | amp |
| TCGA-04-1347 | 1 | 160168732 | 160254939 | 2.439876069 | 6 | amp |
| TCGA-04-1347 | 1 | 160259886 | 160265954 | 1.324864382 | 5 | amp |
| TCGA-04-1347 | 1 | 160267090 | 161089410 | 2.261755107 | 6 | amp |
| TCGA-04-1347 | 1 | 161089555 | 161094269 | 0.947189965 | 4 | amp |
| TCGA-04-1347 | 1 | 161123728 | 161134056 | 2.181646406 | 6 | amp |
| TCGA-04-1347 | 1 | 161134302 | 161198156 | 1.60256823 | 5 | amp |
| TCGA-04-1347 | 1 | 161198195 | 161882187 | 2.318515599 | 6 | amp |
| TCGA-04-1347 | 1 | 161928219 | 162124290 | 1.18315449 | 5 | amp |
| TCGA-04-1347 | 1 | 162257119 | 178885539 | 2.426229519 | 6 | amp |
| TCGA-04-1347 | 1 | 179012930 | 179087930 | 1.462104722 | 5 | amp |
| TCGA-04-1347 | 1 | 179089276 | 200825255 | 2.636364505 | 6 | amp |
| TCGA-04-1347 | 1 | 200826372 | 201465435 | 1.44349315 | 5 | amp |
| TCGA-04-1347 | 1 | 201681905 | 201759753 | 2.462734125 | 6 | amp |
| TCGA-04-1347 | 1 | 201759795 | 202284029 | 1.465108143 | 5 | amp |
| TCGA-04-1347 | 1 | 202287017 | 202742450 | 1.914915618 | 6 | amp |
| TCGA-04-1347 | 1 | 202743681 | 205042898 | 1.547654055 | 5 | amp |
| TCGA-04-1347 | 1 | 205052644 | 205085058 | 2.559954938 | 6 | amp |
| TCGA-04-1347 | 1 | 205090901 | 206760343 | 1.568993941 | 5 | amp |

| | | | | | | |
|---|---|---|---|---|---|---|
| TCGA-04-1347 | 1 | 206765081 | 206905313 | 2.262194595 | 6 | amp |
| TCGA-04-1347 | 1 | 206905319 | 207133172 | 1.564990061 | 5 | amp |
| TCGA-04-1347 | 1 | 207135546 | 208050384 | 2.152685658 | 6 | amp |
| TCGA-04-1347 | 1 | 208061055 | 208225859 | 1.46850232 | 5 | amp |
| TCGA-04-1347 | 1 | 208227704 | 209792020 | 1.98472787 | 6 | amp |
| TCGA-04-1347 | 1 | 209795802 | 209824335 | 1.233275677 | 5 | amp |
| TCGA-04-1347 | 1 | 209878271 | 223945136 | 2.253260827 | 6 | amp |
| TCGA-04-1347 | 1 | 223946957 | 223990634 | 1.502390063 | 5 | amp |
| TCGA-04-1347 | 1 | 223990913 | 226016641 | 1.960235957 | 6 | amp |
| TCGA-04-1347 | 1 | 226019449 | 227204809 | 1.550490852 | 5 | amp |
| TCGA-04-1347 | 1 | 227210945 | 227843562 | 2.156115892 | 6 | amp |
| TCGA-04-1347 | 1 | 227921038 | 228646260 | 0.988905461 | 4 | amp |
| TCGA-04-1347 | 1 | 228651984 | 247163414 | 2.284397697 | 6 | amp |
| TCGA-04-1347 | 1 | 247200699 | 247593091 | 1.141772307 | 5 | amp |
| TCGA-04-1347 | 1 | 247597339 | 248738122 | 2.166764457 | 6 | amp |
| TCGA-04-1347 | 1 | 248756119 | 249231325 | 1.467548035 | 5 | amp |
| TCGA-04-1347 | 10 | 92880 | 5043909 | 1.319336479 | 5 | amp |
| TCGA-04-1347 | 10 | 5045563 | 5837034 | 1.817013757 | 6 | amp |
| TCGA-04-1347 | 10 | 5838685 | 6148212 | 1.432593231 | 5 | amp |
| TCGA-04-1347 | 10 | 6150586 | 6157527 | 2.304875806 | 6 | amp |
| TCGA-04-1347 | 10 | 6255528 | 6527292 | 1.307492825 | 5 | amp |
| TCGA-04-1347 | 10 | 6527961 | 7618995 | 1.856587301 | 6 | amp |
| TCGA-04-1347 | 10 | 7621692 | 11791602 | 1.393760874 | 5 | amp |
| TCGA-04-1347 | 10 | 11797386 | 12077479 | 1.938937295 | 6 | amp |
| TCGA-04-1347 | 10 | 12123428 | 14870327 | 1.454726541 | 5 | amp |
| TCGA-04-1347 | 10 | 14881883 | 15103911 | 1.896739229 | 6 | amp |
| TCGA-04-1347 | 10 | 15106391 | 15151889 | 0.98516041 | 4 | amp |
| TCGA-04-1347 | 10 | 15154728 | 22690267 | 2.025365735 | 6 | amp |
| TCGA-04-1347 | 10 | 22699912 | 24822202 | 1.502436824 | 5 | amp |
| TCGA-04-1347 | 10 | 24825642 | 39006459 | 1.878274669 | 6 | amp |
| TCGA-04-1347 | 10 | 39011160 | 43692605 | 1.405698517 | 5 | amp |
| TCGA-04-1347 | 10 | 43693427 | 51947289 | 1.011233886 | 4 | amp |
| TCGA-04-1347 | 10 | 51949242 | 70229948 | 1.313883792 | 5 | amp |
| TCGA-04-1347 | 10 | 70243146 | 70967742 | 1.133259032 | 4 | amp |
| TCGA-04-1347 | 10 | 70968333 | 73765790 | 0.890564013 | 3 | amp |
| TCGA-04-1347 | 10 | 73822407 | 75632853 | 1.033405691 | 4 | amp |
| TCGA-04-1347 | 10 | 75671752 | 92509391 | 1.356623711 | 5 | amp |
| TCGA-04-1347 | 10 | 92616858 | 93744179 | 1.824920905 | 6 | amp |
| TCGA-04-1347 | 10 | 93748883 | 94397342 | 1.470892066 | 5 | amp |
| TCGA-04-1347 | 10 | 94399513 | 94837070 | 1.809370942 | 6 | amp |
| TCGA-04-1347 | 10 | 95066648 | 95400320 | 1.375041377 | 5 | amp |
| TCGA-04-1347 | 10 | 95400671 | 95537398 | 1.958542976 | 6 | amp |
| TCGA-04-1347 | 10 | 95549831 | 98405444 | 1.505710131 | 5 | amp |
| TCGA-04-1347 | 10 | 98408393 | 98744939 | 1.053168859 | 4 | amp |
| TCGA-04-1347 | 10 | 98761900 | 99664618 | 0.894031516 | 3 | amp |
| TCGA-04-1347 | 10 | 99667686 | 105495617 | 1.154869042 | 4 | amp |
| TCGA-04-1347 | 10 | 105526829 | 106581917 | 1.373262302 | 5 | amp |
| TCGA-04-1347 | 10 | 106602523 | 106918760 | 2.09165168 | 6 | amp |
| TCGA-04-1347 | 10 | 106924038 | 116595407 | 1.420498411 | 5 | amp |
| TCGA-04-1347 | 10 | 116595823 | 118404636 | 2.04156155 | 6 | amp |

| | | | | | | |
|---|---|---|---|---|---|---|
| TCGA-04-1347 | 10 | 118423592 | 129207491 | 1.379558219 | 5 | amp |
| TCGA-04-1347 | 10 | 129207555 | 133930935 | 1.139757902 | 4 | amp |
| TCGA-04-1347 | 10 | 133946803 | 135516111 | 0.749099216 | 3 | amp |
| TCGA-04-1347 | 11 | 3848780 | 20398314 | 0.840864716 | 3 | amp |
| TCGA-04-1347 | 11 | 20403694 | 45937128 | 1.22926354 | 5 | amp |
| TCGA-04-1347 | 11 | 45937170 | 46569982 | 0.956978366 | 4 | amp |
| TCGA-04-1347 | 11 | 46624999 | 46750409 | 0.729562669 | 3 | amp |
| TCGA-04-1347 | 11 | 46750900 | 46897555 | 1.171664867 | 5 | amp |
| TCGA-04-1347 | 11 | 46897952 | 46903473 | 0.92108933 | 4 | amp |
| TCGA-04-1347 | 11 | 46905414 | 47652196 | 0.833457134 | 3 | amp |
| TCGA-04-1347 | 11 | 47652523 | 47800761 | 0.962109035 | 4 | amp |
| TCGA-04-1347 | 11 | 47801068 | 57094389 | 1.200628827 | 5 | amp |
| TCGA-04-1347 | 11 | 57094853 | 57185389 | 0.654386289 | 3 | amp |
| TCGA-04-1347 | 11 | 57188403 | 60541426 | 1.102486121 | 5 | amp |
| TCGA-04-1347 | 11 | 60543042 | 60777374 | 0.938811246 | 3 | amp |
| TCGA-04-1347 | 11 | 61318835 | 62339401 | 0.898174533 | 3 | amp |
| TCGA-04-1347 | 11 | 62677130 | 62749517 | 0.782833243 | 3 | amp |
| TCGA-04-1347 | 11 | 62750965 | 63175697 | 1.171721645 | 5 | amp |
| TCGA-04-1347 | 11 | 63176099 | 64129518 | 0.845126188 | 3 | amp |
| TCGA-04-1347 | 11 | 71201806 | 73366959 | 0.89129457 | 3 | amp |
| TCGA-04-1347 | 11 | 73372458 | 74705779 | 1.193360755 | 5 | amp |
| TCGA-04-1347 | 11 | 74716457 | 75509492 | 0.832732456 | 4 | amp |
| TCGA-04-1347 | 11 | 75511355 | 116824851 | 1.386960705 | 5 | amp |
| TCGA-04-1347 | 11 | 116827571 | 118047171 | 0.870331177 | 3 | amp |
| TCGA-04-1347 | 11 | 118065025 | 118370648 | 1.205787524 | 5 | amp |
| TCGA-04-1347 | 11 | 118371661 | 120188125 | 0.870873663 | 3 | amp |
| TCGA-04-1347 | 11 | 120189021 | 126104999 | 1.095362625 | 5 | amp |
| TCGA-04-1347 | 11 | 126110602 | 126315056 | 0.724571524 | 3 | amp |
| TCGA-04-1347 | 11 | 126318857 | 134257557 | 1.09014422 | 5 | amp |
| TCGA-04-1347 | 12 | 73256 | 369237 | 1.40684722 | 5 | amp |
| TCGA-04-1347 | 12 | 394604 | 1882187 | 1.988872545 | 6 | amp |
| TCGA-04-1347 | 12 | 1886985 | 4554743 | 1.401792369 | 5 | amp |
| TCGA-04-1347 | 12 | 4598958 | 4700515 | 2.438691043 | 6 | amp |
| TCGA-04-1347 | 12 | 4702129 | 4719505 | 1.031727225 | 4 | amp |
| TCGA-04-1347 | 12 | 4721646 | 6061740 | 1.846146905 | 6 | amp |
| TCGA-04-1347 | 12 | 6062650 | 7025098 | 1.450957686 | 5 | amp |
| TCGA-04-1347 | 12 | 7025568 | 7046752 | 2.093489861 | 6 | amp |
| TCGA-04-1347 | 12 | 7047631 | 7475195 | 1.491649777 | 5 | amp |
| TCGA-04-1347 | 12 | 7475809 | 7656323 | 2.10282553 | 6 | amp |
| TCGA-04-1347 | 12 | 7802097 | 8051410 | 1.428635716 | 5 | amp |
| TCGA-04-1347 | 12 | 8073967 | 9353992 | 1.858632213 | 6 | amp |
| TCGA-04-1347 | 12 | 9354850 | 9685270 | 1.442269469 | 5 | amp |
| TCGA-04-1347 | 12 | 9688794 | 13215963 | 2.05357851 | 6 | amp |
| TCGA-04-1347 | 12 | 13219527 | 13717604 | 1.407238646 | 5 | amp |
| TCGA-04-1347 | 12 | 13719867 | 30894056 | 2.12617652 | 6 | amp |
| TCGA-04-1347 | 12 | 30903975 | 31249978 | 1.2647334 | 5 | amp |
| TCGA-04-1347 | 12 | 31250784 | 32520701 | 1.83444061 | 6 | amp |
| TCGA-04-1347 | 12 | 32530457 | 32778015 | 1.216752739 | 5 | amp |
| TCGA-04-1347 | 12 | 32778530 | 48096666 | 2.031942712 | 6 | amp |
| TCGA-04-1347 | 12 | 48104523 | 49495353 | 1.281227896 | 5 | amp |

| | | | | | | |
|---|---|---|---|---|---|---|
| TCGA-04-1347 | 12 | 49691690 | 50528526 | 1.229494353 | 5 | amp |
| TCGA-04-1347 | 12 | 50529477 | 51102347 | 1.756238238 | 6 | amp |
| TCGA-04-1347 | 12 | 51108158 | 53045871 | 1.404677191 | 5 | amp |
| TCGA-04-1347 | 12 | 53068891 | 53187036 | 2.045495421 | 6 | amp |
| TCGA-04-1347 | 12 | 53187884 | 53694049 | 1.250785335 | 5 | amp |
| TCGA-04-1347 | 12 | 53708003 | 55039500 | 1.252123478 | 5 | amp |
| TCGA-04-1347 | 12 | 55040863 | 56077894 | 2.049331289 | 6 | amp |
| TCGA-04-1347 | 12 | 56078806 | 57850673 | 1.308489491 | 5 | amp |
| TCGA-04-1347 | 12 | 57857434 | 57863527 | 2.563564838 | 6 | amp |
| TCGA-04-1347 | 12 | 57864041 | 58002957 | 1.407244291 | 5 | amp |
| TCGA-04-1347 | 12 | 58006628 | 58025184 | 2.202836467 | 6 | amp |
| TCGA-04-1347 | 12 | 58087905 | 58166915 | 1.229098681 | 5 | amp |
| TCGA-04-1347 | 12 | 58168409 | 77243262 | 1.952982384 | 6 | amp |
| TCGA-04-1347 | 12 | 77244575 | 77444559 | 1.284892199 | 5 | amp |
| TCGA-04-1347 | 12 | 77449592 | 94649080 | 2.119983838 | 6 | amp |
| TCGA-04-1347 | 12 | 94653071 | 94697849 | 1.117864762 | 5 | amp |
| TCGA-04-1347 | 12 | 94698918 | 96300256 | 1.821309099 | 6 | amp |
| TCGA-04-1347 | 12 | 96310869 | 96370489 | 1.222196684 | 5 | amp |
| TCGA-04-1347 | 12 | 96371669 | 103872225 | 1.903953985 | 6 | amp |
| TCGA-04-1347 | 12 | 103981234 | 104093059 | 1.452613335 | 5 | amp |
| TCGA-04-1347 | 12 | 104096869 | 104333464 | 1.837818655 | 6 | amp |
| TCGA-04-1347 | 12 | 104335106 | 104374765 | 1.074446379 | 5 | amp |
| TCGA-04-1347 | 12 | 104376554 | 105583970 | 1.822955008 | 6 | amp |
| TCGA-04-1347 | 12 | 105589022 | 115115482 | 1.376802106 | 5 | amp |
| TCGA-04-1347 | 12 | 115117262 | 117155716 | 1.916274993 | 6 | amp |
| TCGA-04-1347 | 12 | 117157564 | 120198920 | 1.573906142 | 5 | amp |
| TCGA-04-1347 | 12 | 120204878 | 120307054 | 2.251212853 | 6 | amp |
| TCGA-04-1347 | 12 | 120313864 | 121779898 | 1.400704172 | 5 | amp |
| TCGA-04-1347 | 12 | 121783617 | 121868002 | 2.147804416 | 6 | amp |
| TCGA-04-1347 | 12 | 121868031 | 123782741 | 1.384449575 | 5 | amp |
| TCGA-04-1347 | 12 | 123787348 | 123821013 | 1.88406826 | 6 | amp |
| TCGA-04-1347 | 12 | 123825494 | 124274690 | 1.390801787 | 5 | amp |
| TCGA-04-1347 | 12 | 124281170 | 124326100 | 1.970010114 | 6 | amp |
| TCGA-04-1347 | 12 | 124330101 | 133779395 | 1.328287479 | 5 | amp |
| TCGA-04-1347 | 13 | 19240876 | 24449076 | 0.79770627 | 3 | amp |
| TCGA-04-1347 | 13 | 25072250 | 31531200 | 0.845303329 | 3 | amp |
| TCGA-04-1347 | 13 | 31540326 | 32698838 | 0.973362167 | 4 | amp |
| TCGA-04-1347 | 13 | 32698911 | 33096409 | 0.741394907 | 3 | amp |
| TCGA-04-1347 | 13 | 33101520 | 36026300 | 0.958951726 | 4 | amp |
| TCGA-04-1347 | 13 | 36049135 | 41705673 | 0.841900522 | 3 | amp |
| TCGA-04-1347 | 13 | 41705700 | 45009070 | 0.924386772 | 4 | amp |
| TCGA-04-1347 | 13 | 45010551 | 50280329 | 0.850759078 | 3 | amp |
| TCGA-04-1347 | 13 | 50280395 | 50299654 | 0.999980021 | 4 | amp |
| TCGA-04-1347 | 13 | 50306494 | 52329604 | 1.250097113 | 5 | amp |
| TCGA-04-1347 | 13 | 52330382 | 88330017 | 0.956073556 | 4 | amp |
| TCGA-04-1347 | 13 | 88330092 | 95232237 | 1.219651363 | 5 | amp |
| TCGA-04-1347 | 13 | 95233323 | 95768275 | 0.922853788 | 4 | amp |
| TCGA-04-1347 | 13 | 95813395 | 96665767 | 1.20505991 | 5 | amp |
| TCGA-04-1347 | 13 | 96675259 | 99508338 | 0.944394735 | 4 | amp |
| TCGA-04-1347 | 13 | 99512657 | 100193067 | 1.204616839 | 5 | amp |

| | | | | | | |
|---|---|---|---|---|---|---|
| TCGA-04-1347 | 13 | 100193806 | 101721232 | 1.008379967 | 4 | amp |
| TCGA-04-1347 | 13 | 101725892 | 109779943 | 1.187011349 | 5 | amp |
| TCGA-04-1347 | 13 | 109817207 | 110844655 | 0.932749632 | 4 | amp |
| TCGA-04-1347 | 13 | 110845158 | 111926349 | 0.819426743 | 3 | amp |
| TCGA-04-1347 | 14 | 19377543 | 20265456 | 1.683617089 | 5 | amp |
| TCGA-04-1347 | 14 | 20295589 | 20586616 | 2.047722706 | 6 | amp |
| TCGA-04-1347 | 14 | 20611877 | 20837945 | 1.330623757 | 5 | amp |
| TCGA-04-1347 | 14 | 20839364 | 20863817 | 0.847164722 | 4 | amp |
| TCGA-04-1347 | 14 | 20864002 | 22265673 | 1.452296011 | 5 | amp |
| TCGA-04-1347 | 14 | 22265730 | 22521350 | 2.266561589 | 6 | amp |
| TCGA-04-1347 | 14 | 22538918 | 23862758 | 1.432552694 | 5 | amp |
| TCGA-04-1347 | 14 | 23862815 | 24586658 | 1.12725905 | 4 | amp |
| TCGA-04-1347 | 14 | 24610264 | 24647482 | 1.367037684 | 5 | amp |
| TCGA-04-1347 | 14 | 24647754 | 24653319 | 1.019264613 | 4 | amp |
| TCGA-04-1347 | 14 | 24659490 | 24676773 | 2.653382242 | 6 | amp |
| TCGA-04-1347 | 14 | 24677140 | 24709562 | 1.195622186 | 5 | amp |
| TCGA-04-1347 | 14 | 24709580 | 24711562 | 3.0928415 | 6 | amp |
| TCGA-04-1347 | 14 | 24718482 | 25101717 | 1.213318547 | 5 | amp |
| TCGA-04-1347 | 14 | 25102074 | 25326417 | 2.375046328 | 6 | amp |
| TCGA-04-1347 | 14 | 25443947 | 36140778 | 1.469635251 | 5 | amp |
| TCGA-04-1347 | 14 | 36142030 | 36841102 | 2.141108302 | 6 | amp |
| TCGA-04-1347 | 14 | 36943014 | 39819504 | 1.249722135 | 5 | amp |
| TCGA-04-1347 | 14 | 39868675 | 56086084 | 1.068127561 | 4 | amp |
| TCGA-04-1347 | 14 | 56094568 | 60951831 | 1.252111514 | 5 | amp |
| TCGA-04-1347 | 14 | 60976072 | 64532378 | 1.101794456 | 4 | amp |
| TCGA-04-1347 | 14 | 64537315 | 75618850 | 0.922212772 | 3 | amp |
| TCGA-04-1347 | 14 | 75746525 | 76662327 | 1.324908506 | 5 | amp |
| TCGA-04-1347 | 14 | 76667963 | 77723088 | 0.778446145 | 3 | amp |
| TCGA-04-1347 | 14 | 77732536 | 77949051 | 1.079486756 | 4 | amp |
| TCGA-04-1347 | 14 | 77950576 | 90485770 | 1.438206348 | 5 | amp |
| TCGA-04-1347 | 14 | 90489338 | 92071074 | 1.090925595 | 4 | amp |
| TCGA-04-1347 | 14 | 92074602 | 92627642 | 1.296610097 | 5 | amp |
| TCGA-04-1347 | 14 | 92627981 | 93718023 | 1.043499868 | 4 | amp |
| TCGA-04-1347 | 14 | 93719948 | 99969368 | 1.3152449 | 5 | amp |
| TCGA-04-1347 | 14 | 99973388 | 101351139 | 1.048034052 | 4 | amp |
| TCGA-04-1347 | 14 | 101364234 | 101459669 | 2.367187552 | 6 | amp |
| TCGA-04-1347 | 14 | 101500072 | 106068089 | 1.055331712 | 4 | amp |
| TCGA-04-1347 | 14 | 106090793 | 106110482 | 2.945763582 | 6 | amp |
| TCGA-04-1347 | 14 | 106110799 | 107283263 | 1.214280324 | 5 | amp |
| TCGA-04-1347 | 15 | 20169886 | 20832424 | 0.720343923 | 3 | amp |
| TCGA-04-1347 | 15 | 20833516 | 22466533 | 1.199906099 | 4 | amp |
| TCGA-04-1347 | 15 | 22472889 | 35084506 | 0.747472721 | 3 | amp |
| TCGA-04-1347 | 15 | 35084539 | 40512982 | 1.064408488 | 4 | amp |
| TCGA-04-1347 | 15 | 40556938 | 41870518 | 0.874025376 | 3 | amp |
| TCGA-04-1347 | 15 | 41961084 | 42503986 | 1.000570676 | 4 | amp |
| TCGA-04-1347 | 15 | 42509955 | 43016737 | 1.22971238 | 5 | amp |
| TCGA-04-1347 | 15 | 43067296 | 43447069 | 1.293124624 | 5 | amp |
| TCGA-04-1347 | 15 | 43452875 | 43678542 | 0.999182496 | 4 | amp |
| TCGA-04-1347 | 15 | 43687269 | 43701314 | 1.835841848 | 6 | amp |
| TCGA-04-1347 | 15 | 43701831 | 43874864 | 1.224262795 | 5 | amp |

| | | | | | | |
|---|---|---|---|---|---|---|
| TCGA-04-1347 | 15 | 43875044 | 44705658 | 1.073271463 | 4 | amp |
| TCGA-04-1347 | 15 | 44751155 | 45365765 | 1.269965412 | 5 | amp |
| TCGA-04-1347 | 15 | 45396330 | 45983259 | 1.257827893 | 5 | amp |
| TCGA-04-1347 | 15 | 48051989 | 48500366 | 1.841603655 | 6 | amp |
| TCGA-04-1347 | 15 | 48512776 | 51207826 | 1.533548518 | 5 | amp |
| TCGA-04-1347 | 15 | 51216104 | 51514753 | 1.753984888 | 6 | amp |
| TCGA-04-1347 | 15 | 51519932 | 56388853 | 1.424370666 | 5 | amp |
| TCGA-04-1347 | 15 | 56390246 | 57543644 | 1.755859487 | 6 | amp |
| TCGA-04-1347 | 15 | 57544594 | 58303031 | 1.301886866 | 5 | amp |
| TCGA-04-1347 | 15 | 58306005 | 60970930 | 1.046307027 | 4 | amp |
| TCGA-04-1347 | 15 | 62146646 | 62336492 | 1.284229007 | 5 | amp |
| TCGA-04-1347 | 15 | 62939457 | 64048971 | 1.031267952 | 4 | amp |
| TCGA-04-1347 | 15 | 64050350 | 65682704 | 0.793546504 | 3 | amp |
| TCGA-04-1347 | 15 | 65684178 | 68480206 | 1.007352404 | 4 | amp |
| TCGA-04-1347 | 15 | 68486341 | 69329578 | 0.707608914 | 3 | amp |
| TCGA-04-1347 | 15 | 69331142 | 69692503 | 1.03732985 | 4 | amp |
| TCGA-04-1347 | 15 | 69695891 | 72454725 | 1.325300981 | 5 | amp |
| TCGA-04-1347 | 15 | 72455591 | 72647992 | 0.974332474 | 4 | amp |
| TCGA-04-1347 | 15 | 72648851 | 72947180 | 1.380916677 | 5 | amp |
| TCGA-04-1347 | 15 | 72987483 | 74335524 | 1.346088812 | 5 | amp |
| TCGA-04-1347 | 15 | 74374748 | 74885587 | 1.008514285 | 4 | amp |
| TCGA-04-1347 | 15 | 74887893 | 75766128 | 0.789445602 | 3 | amp |
| TCGA-04-1347 | 15 | 75772161 | 76077945 | 1.006954377 | 4 | amp |
| TCGA-04-1347 | 15 | 76146717 | 78191180 | 1.386776821 | 5 | amp |
| TCGA-04-1347 | 15 | 78233764 | 80181745 | 1.117976065 | 4 | amp |
| TCGA-04-1347 | 15 | 80191249 | 81615299 | 1.258543226 | 5 | amp |
| TCGA-04-1347 | 15 | 81624701 | 82530874 | 1.777890635 | 6 | amp |
| TCGA-04-1347 | 15 | 82532827 | 83213456 | 1.229195752 | 5 | amp |
| TCGA-04-1347 | 15 | 83333580 | 83788441 | 1.289613153 | 5 | amp |
| TCGA-04-1347 | 15 | 83790653 | 84781774 | 1.834335175 | 6 | amp |
| TCGA-04-1347 | 15 | 84784786 | 85476560 | 1.225756014 | 5 | amp |
| TCGA-04-1347 | 15 | 85478220 | 85661090 | 1.785762624 | 6 | amp |
| TCGA-04-1347 | 15 | 85663986 | 85787286 | 0.941040986 | 4 | amp |
| TCGA-04-1347 | 15 | 85787638 | 87572123 | 1.7867012 | 6 | amp |
| TCGA-04-1347 | 15 | 88420138 | 89862365 | 1.310020104 | 5 | amp |
| TCGA-04-1347 | 15 | 89868588 | 91474839 | 1.308370295 | 5 | amp |
| TCGA-04-1347 | 15 | 91491803 | 102516522 | 1.357164976 | 5 | amp |
| TCGA-04-1347 | 16 | 66517 | 240595 | 1.081554764 | 4 | amp |
| TCGA-04-1347 | 16 | 242909 | 2256270 | 0.687344939 | 3 | amp |
| TCGA-04-1347 | 16 | 2256458 | 3721817 | 0.941896399 | 4 | amp |
| TCGA-04-1347 | 16 | 3724297 | 4312739 | 1.277429478 | 5 | amp |
| TCGA-04-1347 | 16 | 4384739 | 4836163 | 0.925359195 | 4 | amp |
| TCGA-04-1347 | 16 | 4837443 | 8839966 | 1.267287189 | 5 | amp |
| TCGA-04-1347 | 16 | 8841919 | 8906965 | 0.887894467 | 4 | amp |
| TCGA-04-1347 | 16 | 8941541 | 15177464 | 1.320137751 | 5 | amp |
| TCGA-04-1347 | 16 | 15178470 | 15675190 | 0.936101506 | 4 | amp |
| TCGA-04-1347 | 16 | 15676993 | 16173391 | 1.392343625 | 5 | amp |
| TCGA-04-1347 | 16 | 16177190 | 16355566 | 0.95791211 | 4 | amp |
| TCGA-04-1347 | 16 | 16356881 | 16371343 | 1.645650913 | 5 | amp |
| TCGA-04-1347 | 16 | 16372462 | 18532316 | 0.94943152 | 4 | amp |

| | | | | | | |
|---|---|---|---|---|---|---|
| TCGA-04-1347 | 16 | 18535153 | 19034535 | 1.355823548 | 5 | amp |
| TCGA-04-1347 | 16 | 19041498 | 19516428 | 1.032301989 | 4 | amp |
| TCGA-04-1347 | 16 | 19518994 | 22130405 | 1.306376014 | 5 | amp |
| TCGA-04-1347 | 16 | 22132236 | 22320849 | 0.998085392 | 4 | amp |
| TCGA-04-1347 | 16 | 22324904 | 22547602 | 1.403778897 | 5 | amp |
| TCGA-04-1347 | 16 | 22548791 | 24366343 | 1.043637304 | 4 | amp |
| TCGA-04-1347 | 16 | 24372628 | 24817075 | 1.337889629 | 5 | amp |
| TCGA-04-1347 | 16 | 24817505 | 28914003 | 0.977558646 | 4 | amp |
| TCGA-04-1347 | 16 | 28914289 | 28967684 | 1.432501793 | 5 | amp |
| TCGA-04-1347 | 16 | 28969989 | 30237171 | 0.979635541 | 4 | amp |
| TCGA-04-1347 | 16 | 30238338 | 30365693 | 1.263820621 | 5 | amp |
| TCGA-04-1347 | 16 | 30365873 | 30983068 | 0.883874777 | 4 | amp |
| TCGA-04-1347 | 16 | 30991741 | 31141527 | 1.258857995 | 5 | amp |
| TCGA-04-1347 | 16 | 31141731 | 32177057 | 0.948250407 | 4 | amp |
| TCGA-04-1347 | 16 | 32177931 | 32772334 | 1.477963304 | 5 | amp |
| TCGA-04-1347 | 16 | 32773208 | 47189719 | 0.992348778 | 4 | amp |
| TCGA-04-1347 | 16 | 47192722 | 48139268 | 1.421715165 | 5 | amp |
| TCGA-04-1347 | 16 | 48141194 | 48204132 | 0.829474972 | 4 | amp |
| TCGA-04-1347 | 16 | 48204748 | 48596409 | 1.348526171 | 5 | amp |
| TCGA-04-1347 | 16 | 49313293 | 56867342 | 0.760002386 | 3 | amp |
| TCGA-04-1347 | 16 | 70843677 | 70902701 | 0.747867132 | 3 | amp |
| TCGA-04-1347 | 16 | 70905890 | 71186743 | 1.039066474 | 4 | amp |
| TCGA-04-1347 | 16 | 71196413 | 71220850 | 0.761310578 | 3 | amp |
| TCGA-04-1347 | 16 | 81641114 | 87393996 | 0.724469749 | 3 | amp |
| TCGA-04-1347 | 17 | 69410 | 1746181 | 0.852719723 | 3 | amp |
| TCGA-04-1347 | 17 | 1747192 | 6980318 | 1.07480521 | 4 | amp |
| TCGA-04-1347 | 17 | 6981223 | 7259844 | 0.787563618 | 3 | amp |
| TCGA-04-1347 | 17 | 7259874 | 7464378 | 1.147032972 | 4 | amp |
| TCGA-04-1347 | 17 | 7466385 | 7481460 | 1.963742491 | 6 | amp |
| TCGA-04-1347 | 17 | 7489992 | 7496520 | 3.322891555 | 6 | amp |
| TCGA-04-1347 | 17 | 7496711 | 7577613 | 1.077870639 | 4 | amp |
| TCGA-04-1347 | 17 | 7578172 | 7808510 | 0.831406867 | 3 | amp |
| TCGA-04-1347 | 17 | 7808891 | 10250115 | 1.105651146 | 4 | amp |
| TCGA-04-1347 | 17 | 10253821 | 12608537 | 1.330792823 | 5 | amp |
| TCGA-04-1347 | 17 | 12618803 | 17062330 | 1.183895003 | 4 | amp |
| TCGA-04-1347 | 17 | 17074985 | 18302753 | 0.85406314 | 3 | amp |
| TCGA-04-1347 | 17 | 18304198 | 27047095 | 1.10880194 | 4 | amp |
| TCGA-04-1347 | 17 | 27047510 | 27902516 | 0.819897072 | 3 | amp |
| TCGA-04-1347 | 17 | 27902594 | 28378245 | 1.01510962 | 4 | amp |
| TCGA-04-1347 | 17 | 28380239 | 33310597 | 1.368711778 | 5 | amp |
| TCGA-04-1347 | 17 | 33312958 | 38721716 | 1.041205769 | 4 | amp |
| TCGA-04-1347 | 17 | 38785020 | 39643431 | 1.391205461 | 5 | amp |
| TCGA-04-1347 | 17 | 39643515 | 39643785 | 1.251870328 | 4 | amp |
| TCGA-04-1347 | 17 | 39643788 | 40134465 | 0.870214454 | 3 | amp |
| TCGA-04-1347 | 17 | 40135557 | 42392207 | 1.029275818 | 4 | amp |
| TCGA-04-1347 | 17 | 42392276 | 43595676 | 0.874066102 | 3 | amp |
| TCGA-04-1347 | 17 | 43595796 | 43910920 | 0.966874534 | 4 | amp |
| TCGA-04-1347 | 17 | 43911027 | 44717533 | 1.673117765 | 5 | amp |
| TCGA-04-1347 | 17 | 44717986 | 44833238 | 1.955066016 | 6 | amp |
| TCGA-04-1347 | 17 | 44845625 | 45127752 | 1.493812879 | 5 | amp |

| | | | | | | |
|---|---|---|---|---|---|---|
| TCGA-04-1347 | 17 | 45137315 | 45259025 | 1.855790254 | 6 | amp |
| TCGA-04-1347 | 17 | 45286679 | 45367655 | 1.212644451 | 5 | amp |
| TCGA-04-1347 | 17 | 45368266 | 45896468 | 1.872707959 | 6 | amp |
| TCGA-04-1347 | 17 | 45897031 | 45909578 | 0.890008542 | 3 | amp |
| TCGA-04-1347 | 17 | 45911754 | 46023410 | 1.947330209 | 6 | amp |
| TCGA-04-1347 | 17 | 46023663 | 46114629 | 1.319974045 | 5 | amp |
| TCGA-04-1347 | 17 | 46128435 | 46674084 | 1.86925566 | 6 | amp |
| TCGA-04-1347 | 17 | 46685221 | 46865386 | 1.197367611 | 5 | amp |
| TCGA-04-1347 | 17 | 46867260 | 46940403 | 2.099400075 | 6 | amp |
| TCGA-04-1347 | 17 | 46970738 | 47247107 | 1.339257323 | 5 | amp |
| TCGA-04-1347 | 17 | 47284031 | 47700193 | 2.021641692 | 6 | amp |
| TCGA-04-1347 | 17 | 47778785 | 47797802 | 1.219808571 | 5 | amp |
| TCGA-04-1347 | 17 | 47799848 | 47918989 | 2.133259209 | 6 | amp |
| TCGA-04-1347 | 17 | 47921382 | 48156979 | 1.376634634 | 5 | amp |
| TCGA-04-1347 | 17 | 48157592 | 48245139 | 1.881560838 | 6 | amp |
| TCGA-04-1347 | 17 | 48245253 | 48269422 | 1.449765352 | 5 | amp |
| TCGA-04-1347 | 17 | 48269802 | 48445673 | 2.024178653 | 6 | amp |
| TCGA-04-1347 | 17 | 48447366 | 48821178 | 1.369746501 | 5 | amp |
| TCGA-04-1347 | 17 | 48821973 | 54543944 | 2.058057956 | 6 | amp |
| TCGA-04-1347 | 17 | 54554838 | 54926255 | 1.341583327 | 5 | amp |
| TCGA-04-1347 | 17 | 54926507 | 54973359 | 2.083186225 | 6 | amp |
| TCGA-04-1347 | 17 | 54976418 | 56634449 | 1.351217335 | 5 | amp |
| TCGA-04-1347 | 17 | 56635125 | 56688730 | 1.917624317 | 6 | amp |
| TCGA-04-1347 | 17 | 56690693 | 56738988 | 1.324515987 | 5 | amp |
| TCGA-04-1347 | 17 | 56769956 | 57189731 | 1.809071573 | 6 | amp |
| TCGA-04-1347 | 17 | 57196647 | 57430948 | 1.412916111 | 5 | amp |
| TCGA-04-1347 | 17 | 57465649 | 57915766 | 2.331755137 | 6 | amp |
| TCGA-04-1347 | 17 | 57917080 | 57963637 | 1.292345766 | 5 | amp |
| TCGA-04-1347 | 17 | 57968144 | 58090487 | 1.952537268 | 6 | amp |
| TCGA-04-1347 | 17 | 58092094 | 58234118 | 1.405745644 | 5 | amp |
| TCGA-04-1347 | 17 | 58234740 | 59161936 | 1.975349863 | 6 | amp |
| TCGA-04-1347 | 17 | 59445651 | 59557345 | 1.268857508 | 5 | amp |
| TCGA-04-1347 | 17 | 59557445 | 60674050 | 1.957057805 | 6 | amp |
| TCGA-04-1347 | 17 | 60677976 | 61805771 | 1.434234551 | 5 | amp |
| TCGA-04-1347 | 17 | 61824220 | 61899555 | 1.986435372 | 6 | amp |
| TCGA-04-1347 | 17 | 61901094 | 62463767 | 1.280536499 | 5 | amp |
| TCGA-04-1347 | 17 | 62473902 | 62542478 | 1.818270136 | 6 | amp |
| TCGA-04-1347 | 17 | 62543698 | 64059259 | 1.508268222 | 5 | amp |
| TCGA-04-1347 | 17 | 64062885 | 64302289 | 1.985135718 | 6 | amp |
| TCGA-04-1347 | 17 | 64492299 | 65103842 | 1.241430456 | 5 | amp |
| TCGA-04-1347 | 17 | 65104574 | 65944429 | 2.080208329 | 6 | amp |
| TCGA-04-1347 | 17 | 65955601 | 66432631 | 1.520358589 | 5 | amp |
| TCGA-04-1347 | 17 | 66440621 | 71166613 | 2.396733031 | 6 | amp |
| TCGA-04-1347 | 17 | 71192557 | 71375433 | 1.655279568 | 5 | amp |
| TCGA-04-1347 | 17 | 71375577 | 79667904 | 1.070165488 | 4 | amp |
| TCGA-04-1347 | 17 | 79668027 | 80446092 | 0.688276191 | 3 | amp |
| TCGA-04-1347 | 17 | 80521176 | 80676977 | 1.173521241 | 4 | amp |
| TCGA-04-1347 | 17 | 80678133 | 80890632 | 1.848344123 | 6 | amp |
| TCGA-04-1347 | 17 | 80895131 | 81006722 | 1.198565228 | 5 | amp |
| TCGA-04-1347 | 18 | 47273 | 641553 | 2.076791228 | 6 | amp |

| | | | | | | |
|---|---|---|---|---|---|---|
| TCGA-04-1347 | 18 | 644883 | 706583 | 1.408211957 | 5 | amp |
| TCGA-04-1347 | 18 | 724407 | 2796521 | 2.39148417 | 6 | amp |
| TCGA-04-1347 | 18 | 2884899 | 2925413 | 1.185928137 | 5 | amp |
| TCGA-04-1347 | 18 | 2926671 | 10681763 | 1.974767741 | 6 | amp |
| TCGA-04-1347 | 18 | 10689606 | 14198533 | 1.506344996 | 5 | amp |
| TCGA-04-1347 | 18 | 14214186 | 20817217 | 2.002770792 | 6 | amp |
| TCGA-04-1347 | 18 | 20832916 | 21892128 | 1.516308846 | 5 | amp |
| TCGA-04-1347 | 18 | 21894171 | 23772388 | 1.946116175 | 6 | amp |
| TCGA-04-1347 | 18 | 23845056 | 28588172 | 1.419400624 | 5 | amp |
| TCGA-04-1347 | 18 | 28588182 | 77960823 | 0.809493754 | 3 | amp |
| TCGA-04-1347 | 19 | 71882 | 6307352 | 0.74780075 | 3 | amp |
| TCGA-04-1347 | 19 | 6309626 | 9006823 | 0.900075581 | 4 | amp |
| TCGA-04-1347 | 19 | 9007459 | 9091816 | 1.367684274 | 5 | amp |
| TCGA-04-1347 | 19 | 9203911 | 12858972 | 0.891220332 | 4 | amp |
| TCGA-04-1347 | 19 | 12863362 | 12867151 | 2.339777507 | 6 | amp |
| TCGA-04-1347 | 19 | 12874020 | 12907997 | 0.970722986 | 4 | amp |
| TCGA-04-1347 | 19 | 12910587 | 16000547 | 0.761541323 | 3 | amp |
| TCGA-04-1347 | 19 | 19653636 | 21326459 | 0.953461024 | 4 | amp |
| TCGA-04-1347 | 19 | 21349080 | 34857354 | 1.277584161 | 5 | amp |
| TCGA-04-1347 | 19 | 34859427 | 34904756 | 0.712954518 | 4 | amp |
| TCGA-04-1347 | 19 | 34912347 | 35649334 | 1.386191729 | 5 | amp |
| TCGA-04-1347 | 19 | 35651567 | 35786939 | 0.682802226 | 3 | amp |
| TCGA-04-1347 | 19 | 35793270 | 36054585 | 1.385376356 | 5 | amp |
| TCGA-04-1347 | 19 | 36104572 | 36341366 | 0.987126521 | 4 | amp |
| TCGA-04-1347 | 19 | 36341806 | 38743633 | 1.245977507 | 5 | amp |
| TCGA-04-1347 | 19 | 38774231 | 38817670 | 0.877018491 | 4 | amp |
| TCGA-04-1347 | 19 | 38817781 | 38855788 | 1.854228884 | 6 | amp |
| TCGA-04-1347 | 19 | 38857753 | 38942584 | 1.039445811 | 4 | amp |
| TCGA-04-1347 | 19 | 38945822 | 39327450 | 1.22292581 | 5 | amp |
| TCGA-04-1347 | 19 | 39328011 | 39396188 | 0.845028265 | 4 | amp |
| TCGA-04-1347 | 19 | 39397814 | 39874212 | 1.334364983 | 5 | amp |
| TCGA-04-1347 | 19 | 39876596 | 39957224 | 0.817847527 | 4 | amp |
| TCGA-04-1347 | 19 | 39957259 | 41727992 | 1.29375473 | 5 | amp |
| TCGA-04-1347 | 19 | 41758241 | 41826430 | 1.560010397 | 5 | amp |
| TCGA-04-1347 | 19 | 41828409 | 41925240 | 0.959407516 | 4 | amp |
| TCGA-04-1347 | 19 | 41928051 | 42818701 | 1.334995913 | 5 | amp |
| TCGA-04-1347 | 19 | 42818717 | 42820732 | 3.174916267 | 6 | amp |
| TCGA-04-1347 | 19 | 42820762 | 45395743 | 1.427206104 | 5 | amp |
| TCGA-04-1347 | 19 | 45396079 | 45573380 | 1.020732889 | 4 | amp |
| TCGA-04-1347 | 19 | 45573716 | 46124937 | 1.342356001 | 5 | amp |
| TCGA-04-1347 | 19 | 46127936 | 46192551 | 0.980398762 | 4 | amp |
| TCGA-04-1347 | 19 | 46195055 | 49337596 | 1.21475948 | 5 | amp |
| TCGA-04-1347 | 19 | 49339006 | 49619744 | 0.951557112 | 4 | amp |
| TCGA-04-1347 | 19 | 49621043 | 51358294 | 1.334533041 | 5 | amp |
| TCGA-04-1347 | 19 | 51359425 | 55605823 | 0.892755806 | 4 | amp |
| TCGA-04-1347 | 19 | 55605977 | 55687552 | 1.329980017 | 5 | amp |
| TCGA-04-1347 | 19 | 55689529 | 59110878 | 0.891762665 | 4 | amp |
| TCGA-04-1347 | 2 | 41527 | 1488673 | 2.134193861 | 6 | amp |
| TCGA-04-1347 | 2 | 1491557 | 1684169 | 1.336917829 | 5 | amp |
| TCGA-04-1347 | 2 | 1695675 | 10581861 | 2.079802302 | 6 | amp |

| | | | | | | |
|---|---|---|---|---|---|---|
| TCGA-04-1347 | 2 | 10585047 | 26683654 | 2.321024618 | 6 | amp |
| TCGA-04-1347 | 2 | 26683688 | 26724708 | 1.121368599 | 5 | amp |
| TCGA-04-1347 | 2 | 26726586 | 27169880 | 2.237729932 | 6 | amp |
| TCGA-04-1347 | 2 | 27244996 | 27551099 | 1.268924242 | 5 | amp |
| TCGA-04-1347 | 2 | 27551278 | 27607076 | 1.958274337 | 6 | amp |
| TCGA-04-1347 | 2 | 27607477 | 27660237 | 0.846001636 | 3 | amp |
| TCGA-04-1347 | 2 | 27662552 | 27679568 | 2.111369534 | 6 | amp |
| TCGA-04-1347 | 2 | 27680455 | 27702483 | 1.317459516 | 5 | amp |
| TCGA-04-1347 | 2 | 27702834 | 27721687 | 2.276236059 | 6 | amp |
| TCGA-04-1347 | 2 | 27721981 | 27825438 | 1.304398469 | 5 | amp |
| TCGA-04-1347 | 2 | 27825940 | 28775886 | 1.968409729 | 6 | amp |
| TCGA-04-1347 | 2 | 28785846 | 28841296 | 1.177898803 | 5 | amp |
| TCGA-04-1347 | 2 | 28843105 | 29420577 | 2.059645371 | 6 | amp |
| TCGA-04-1347 | 2 | 29429997 | 29543802 | 1.372737596 | 5 | amp |
| TCGA-04-1347 | 2 | 29551161 | 30977252 | 1.912134795 | 6 | amp |
| TCGA-04-1347 | 2 | 30980897 | 31565165 | 1.285261169 | 5 | amp |
| TCGA-04-1347 | 2 | 31567485 | 44050124 | 2.026217874 | 6 | amp |
| TCGA-04-1347 | 2 | 44051034 | 44102598 | 1.261231542 | 5 | amp |
| TCGA-04-1347 | 2 | 44104643 | 61710278 | 2.069157127 | 6 | amp |
| TCGA-04-1347 | 2 | 61711035 | 61724197 | 1.077659356 | 5 | amp |
| TCGA-04-1347 | 2 | 61725747 | 70502841 | 2.044802955 | 6 | amp |
| TCGA-04-1347 | 2 | 70503834 | 71365810 | 1.332356521 | 5 | amp |
| TCGA-04-1347 | 2 | 71366898 | 71655788 | 1.886093633 | 6 | amp |
| TCGA-04-1347 | 2 | 71658417 | 71913662 | 1.484972275 | 5 | amp |
| TCGA-04-1347 | 2 | 72359311 | 74590607 | 1.928943886 | 6 | amp |
| TCGA-04-1347 | 2 | 74590687 | 74598324 | 1.118319038 | 5 | amp |
| TCGA-04-1347 | 2 | 74598639 | 74718823 | 1.776596086 | 6 | amp |
| TCGA-04-1347 | 2 | 74719096 | 74754973 | 1.040394819 | 5 | amp |
| TCGA-04-1347 | 2 | 74755009 | 74867462 | 2.206257601 | 6 | amp |
| TCGA-04-1347 | 2 | 74883616 | 75081620 | 1.047195561 | 5 | amp |
| TCGA-04-1347 | 2 | 75094686 | 85280581 | 1.952821676 | 6 | amp |
| TCGA-04-1347 | 2 | 85510599 | 86346225 | 1.343497382 | 5 | amp |
| TCGA-04-1347 | 2 | 86348599 | 89197332 | 2.011508318 | 6 | amp |
| TCGA-04-1347 | 2 | 89246786 | 89399859 | 1.34035776 | 5 | amp |
| TCGA-04-1347 | 2 | 89416800 | 89476650 | 2.298799479 | 6 | amp |
| TCGA-04-1347 | 2 | 89512875 | 90259861 | 1.431770488 | 5 | amp |
| TCGA-04-1347 | 2 | 90259920 | 95715508 | 1.832228194 | 6 | amp |
| TCGA-04-1347 | 2 | 95719072 | 96078733 | 1.37126614 | 5 | amp |
| TCGA-04-1347 | 2 | 96079136 | 96698135 | 1.914416839 | 6 | amp |
| TCGA-04-1347 | 2 | 96789559 | 96953713 | 1.192024806 | 5 | amp |
| TCGA-04-1347 | 2 | 96954382 | 96967477 | 2.154059855 | 6 | amp |
| TCGA-04-1347 | 2 | 96968861 | 97757500 | 1.170930072 | 5 | amp |
| TCGA-04-1347 | 2 | 97784050 | 98737961 | 1.991455563 | 6 | amp |
| TCGA-04-1347 | 2 | 98744636 | 99180133 | 1.49373776 | 5 | amp |
| TCGA-04-1347 | 2 | 99181044 | 100052409 | 1.945004117 | 6 | amp |
| TCGA-04-1347 | 2 | 100055057 | 101706830 | 1.548519018 | 5 | amp |
| TCGA-04-1347 | 2 | 101869603 | 105889511 | 2.014936124 | 6 | amp |
| TCGA-04-1347 | 2 | 105889953 | 108475780 | 1.519592635 | 5 | amp |
| TCGA-04-1347 | 2 | 108475802 | 108881810 | 1.802540575 | 6 | amp |
| TCGA-04-1347 | 2 | 108910049 | 109345681 | 1.490370161 | 5 | amp |

| | | | | | | |
|---|---|---|---|---|---|---|
| TCGA-04-1347 | 2 | 109347225 | 109429389 | 1.801803953 | 6 | amp |
| TCGA-04-1347 | 2 | 109432358 | 131223405 | 1.426884718 | 5 | amp |
| TCGA-04-1347 | 2 | 131231913 | 131254220 | 2.304553321 | 6 | amp |
| TCGA-04-1347 | 2 | 131256605 | 131379227 | 1.016363731 | 4 | amp |
| TCGA-04-1347 | 2 | 131381611 | 131403920 | 2.352253525 | 6 | amp |
| TCGA-04-1347 | 2 | 131412433 | 136630459 | 1.430832455 | 5 | amp |
| TCGA-04-1347 | 2 | 136664844 | 149864588 | 2.010063152 | 6 | amp |
| TCGA-04-1347 | 2 | 149868076 | 152293869 | 1.517593799 | 5 | amp |
| TCGA-04-1347 | 2 | 152295168 | 152422341 | 1.75741803 | 6 | amp |
| TCGA-04-1347 | 2 | 152423657 | 153494197 | 1.578560377 | 5 | amp |
| TCGA-04-1347 | 2 | 153496460 | 159481964 | 1.79321798 | 6 | amp |
| TCGA-04-1347 | 2 | 159488208 | 162081259 | 1.477935401 | 5 | amp |
| TCGA-04-1347 | 2 | 162087471 | 163057162 | 1.869582905 | 6 | amp |
| TCGA-04-1347 | 2 | 163059356 | 163124811 | 1.258290517 | 5 | amp |
| TCGA-04-1347 | 2 | 163128696 | 169551574 | 1.832994569 | 6 | amp |
| TCGA-04-1347 | 2 | 169571454 | 169792961 | 1.361643588 | 5 | amp |
| TCGA-04-1347 | 2 | 169801075 | 169994010 | 1.886420084 | 6 | amp |
| TCGA-04-1347 | 2 | 169995031 | 175978838 | 1.393116981 | 5 | amp |
| TCGA-04-1347 | 2 | 175979386 | 178402982 | 1.75901484 | 6 | amp |
| TCGA-04-1347 | 2 | 178415472 | 179462585 | 1.441399253 | 5 | amp |
| TCGA-04-1347 | 2 | 179462589 | 179517092 | 1.848214452 | 6 | amp |
| TCGA-04-1347 | 2 | 179517166 | 179981575 | 1.421606176 | 5 | amp |
| TCGA-04-1347 | 2 | 179982239 | 182468846 | 1.799751462 | 6 | amp |
| TCGA-04-1347 | 2 | 182542427 | 183597307 | 1.466751327 | 5 | amp |
| TCGA-04-1347 | 2 | 183600924 | 192227105 | 1.809000349 | 6 | amp |
| TCGA-04-1347 | 2 | 192228407 | 192543904 | 1.381069537 | 5 | amp |
| TCGA-04-1347 | 2 | 192546647 | 197975588 | 1.842315783 | 6 | amp |
| TCGA-04-1347 | 2 | 197986041 | 198607893 | 1.550628512 | 5 | amp |
| TCGA-04-1347 | 2 | 198621095 | 201726624 | 1.849138755 | 6 | amp |
| TCGA-04-1347 | 2 | 201736060 | 203764366 | 1.54747414 | 5 | amp |
| TCGA-04-1347 | 2 | 203765734 | 204003081 | 1.942615494 | 6 | amp |
| TCGA-04-1347 | 2 | 204003286 | 207406876 | 1.506109969 | 5 | amp |
| TCGA-04-1347 | 2 | 207407948 | 208605464 | 1.892951603 | 6 | amp |
| TCGA-04-1347 | 2 | 208606892 | 209110155 | 1.48969628 | 5 | amp |
| TCGA-04-1347 | 2 | 209113058 | 216002956 | 2.022105888 | 6 | amp |
| TCGA-04-1347 | 2 | 216177163 | 217543807 | 1.446361047 | 5 | amp |
| TCGA-04-1347 | 2 | 217724313 | 219611941 | 1.072742024 | 4 | amp |
| TCGA-04-1347 | 2 | 219611993 | 220506471 | 0.902175934 | 3 | amp |
| TCGA-04-1347 | 2 | 222290714 | 222429142 | 1.452560079 | 5 | amp |
| TCGA-04-1347 | 2 | 222433411 | 227732087 | 1.753911582 | 6 | amp |
| TCGA-04-1347 | 2 | 227771462 | 228121326 | 1.563503222 | 5 | amp |
| TCGA-04-1347 | 2 | 228122279 | 232121375 | 2.0292043 | 6 | amp |
| TCGA-04-1347 | 2 | 232123669 | 233405172 | 1.465678118 | 5 | amp |
| TCGA-04-1347 | 2 | 233405276 | 234978674 | 1.901144606 | 6 | amp |
| TCGA-04-1347 | 2 | 235404404 | 240048420 | 1.359580952 | 5 | amp |
| TCGA-04-1347 | 2 | 240055919 | 243160772 | 1.005421942 | 4 | amp |
| TCGA-04-1347 | 20 | 68319 | 170299 | 2.254703535 | 6 | amp |
| TCGA-04-1347 | 20 | 207898 | 3363173 | 1.542063355 | 5 | amp |
| TCGA-04-1347 | 20 | 3515881 | 3627551 | 2.278616758 | 6 | amp |
| TCGA-04-1347 | 20 | 3643963 | 5154356 | 1.408553591 | 5 | amp |

| | | | | | | |
|---|---|---|---|---|---|---|
| TCGA-04-1347 | 20 | 5155816 | 23383729 | 2.327171939 | 6 | amp |
| TCGA-04-1347 | 20 | 23420863 | 25304073 | 1.59982965 | 5 | amp |
| TCGA-04-1347 | 20 | 25319804 | 32224539 | 2.064841858 | 6 | amp |
| TCGA-04-1347 | 20 | 32228121 | 32661697 | 1.460867343 | 5 | amp |
| TCGA-04-1347 | 20 | 32663612 | 33567628 | 2.430396176 | 6 | amp |
| TCGA-04-1347 | 20 | 33568327 | 33586739 | 1.038545112 | 5 | amp |
| TCGA-04-1347 | 20 | 33587285 | 35869771 | 2.163847955 | 6 | amp |
| TCGA-04-1347 | 20 | 35879564 | 36031301 | 1.326747579 | 5 | amp |
| TCGA-04-1347 | 20 | 36031557 | 36759682 | 2.671967283 | 6 | amp |
| TCGA-04-1347 | 20 | 36760671 | 37076913 | 1.607846789 | 5 | amp |
| TCGA-04-1347 | 20 | 37077929 | 42164955 | 2.628447537 | 6 | amp |
| TCGA-04-1347 | 20 | 42165013 | 42213690 | 1.256856305 | 5 | amp |
| TCGA-04-1347 | 20 | 42223247 | 42966107 | 2.252073513 | 6 | amp |
| TCGA-04-1347 | 20 | 42969744 | 43118222 | 1.41853858 | 5 | amp |
| TCGA-04-1347 | 20 | 43129707 | 43995814 | 2.392126275 | 6 | amp |
| TCGA-04-1347 | 20 | 44037390 | 44573707 | 2.13439268 | 6 | amp |
| TCGA-04-1347 | 20 | 44574285 | 44589046 | 1.318715583 | 5 | amp |
| TCGA-04-1347 | 20 | 44589065 | 46287180 | 2.403581729 | 6 | amp |
| TCGA-04-1347 | 20 | 46288109 | 46319074 | 0.989532146 | 4 | amp |
| TCGA-04-1347 | 20 | 46331229 | 55940544 | 2.201751657 | 6 | amp |
| TCGA-04-1347 | 20 | 55941845 | 56191531 | 1.597209839 | 5 | amp |
| TCGA-04-1347 | 20 | 56195274 | 60574221 | 2.916837632 | 6 | amp |
| TCGA-04-1347 | 20 | 60575124 | 62837209 | 1.223008254 | 5 | amp |
| TCGA-04-1347 | 20 | 62838892 | 62848646 | 4.346545251 | 6 | amp |
| TCGA-04-1347 | 20 | 62850175 | 62926333 | 1.309990561 | 5 | amp |
| TCGA-04-1347 | 21 | 9483321 | 34128725 | 1.363174078 | 5 | amp |
| TCGA-04-1347 | 21 | 34131365 | 34900676 | 1.008937947 | 4 | amp |
| TCGA-04-1347 | 21 | 34900796 | 34968149 | 1.389379354 | 5 | amp |
| TCGA-04-1347 | 21 | 34969526 | 38460691 | 0.974423574 | 4 | amp |
| TCGA-04-1347 | 21 | 38461082 | 41459275 | 1.284666158 | 5 | amp |
| TCGA-04-1347 | 21 | 41465574 | 44492318 | 0.995075388 | 4 | amp |
| TCGA-04-1347 | 21 | 44513115 | 45096824 | 1.44798826 | 5 | amp |
| TCGA-04-1347 | 21 | 45103146 | 45511986 | 1.022796104 | 4 | amp |
| TCGA-04-1347 | 21 | 45513908 | 45951030 | 0.706318 | 3 | amp |
| TCGA-04-1347 | 21 | 45959106 | 46191407 | 1.390067963 | 5 | amp |
| TCGA-04-1347 | 21 | 46193381 | 48111215 | 0.963205188 | 4 | amp |
| TCGA-04-1347 | 22 | 16100468 | 17062226 | 1.37134916 | 5 | amp |
| TCGA-04-1347 | 22 | 17071763 | 18138657 | 0.875954808 | 4 | amp |
| TCGA-04-1347 | 22 | 18165973 | 18218411 | 1.469992263 | 5 | amp |
| TCGA-04-1347 | 22 | 18220738 | 18382323 | 0.881896504 | 4 | amp |
| TCGA-04-1347 | 22 | 18383565 | 21088881 | 0.751998665 | 3 | amp |
| TCGA-04-1347 | 22 | 21096429 | 22057772 | 1.015477856 | 4 | amp |
| TCGA-04-1347 | 22 | 22064854 | 23657724 | 0.801900822 | 3 | amp |
| TCGA-04-1347 | 22 | 23915402 | 24432026 | 0.964482263 | 4 | amp |
| TCGA-04-1347 | 22 | 24432492 | 24580307 | 0.58523128 | 3 | amp |
| TCGA-04-1347 | 22 | 24580670 | 25130146 | 0.928110159 | 4 | amp |
| TCGA-04-1347 | 22 | 25131646 | 25151931 | 1.602214315 | 5 | amp |
| TCGA-04-1347 | 22 | 25152444 | 25853296 | 0.875586992 | 4 | amp |
| TCGA-04-1347 | 22 | 26000321 | 26099569 | 1.347515815 | 5 | amp |
| TCGA-04-1347 | 22 | 26100035 | 29621223 | 1.01637693 | 4 | amp |

| | | | | | | |
|---|---|---|---|---|---|---|
| TCGA-04-1347 | 22 | 29622449 | 30218508 | 0.812087936 | 3 | amp |
| TCGA-04-1347 | 22 | 30221010 | 30500467 | 1.171872295 | 4 | amp |
| TCGA-04-1347 | 22 | 30507734 | 31658232 | 0.716363853 | 3 | amp |
| TCGA-04-1347 | 22 | 31658528 | 35481746 | 1.011693655 | 4 | amp |
| TCGA-04-1347 | 22 | 35658308 | 35819390 | 0.67848673 | 3 | amp |
| TCGA-04-1347 | 22 | 35942811 | 36682947 | 1.034480799 | 4 | amp |
| TCGA-04-1347 | 22 | 36684228 | 38536202 | 0.769848898 | 3 | amp |
| TCGA-04-1347 | 22 | 38539053 | 39122473 | 1.045083125 | 4 | amp |
| TCGA-04-1347 | 22 | 39123135 | 40080524 | 0.736082873 | 3 | amp |
| TCGA-04-1347 | 22 | 40139625 | 41569827 | 0.915607777 | 4 | amp |
| TCGA-04-1347 | 3 | 361444 | 9798349 | 1.131492924 | 5 | amp |
| TCGA-04-1347 | 3 | 9798398 | 15132095 | 0.894259563 | 4 | amp |
| TCGA-04-1347 | 3 | 15137433 | 27333085 | 1.151561012 | 5 | amp |
| TCGA-04-1347 | 3 | 27335050 | 27418350 | 1.929772803 | 6 | amp |
| TCGA-04-1347 | 3 | 27424632 | 33602501 | 1.417391694 | 5 | amp |
| TCGA-04-1347 | 3 | 33614543 | 33686441 | 1.893939815 | 6 | amp |
| TCGA-04-1347 | 3 | 33686730 | 53353526 | 1.126706905 | 5 | amp |
| TCGA-04-1347 | 3 | 53376114 | 89176450 | 0.764948685 | 4 | amp |
| TCGA-04-1347 | 3 | 89258979 | 93813180 | 1.491015874 | 5 | amp |
| TCGA-04-1347 | 3 | 93813817 | 96945305 | 2.212854041 | 6 | amp |
| TCGA-04-1347 | 3 | 96962783 | 97439299 | 1.622110798 | 5 | amp |
| TCGA-04-1347 | 3 | 97454745 | 97666308 | 2.238298718 | 6 | amp |
| TCGA-04-1347 | 3 | 97668623 | 97673347 | 0.873972662 | 3 | amp |
| TCGA-04-1347 | 3 | 97677838 | 98600617 | 2.189414446 | 6 | amp |
| TCGA-04-1347 | 3 | 99509510 | 100039837 | 1.698430148 | 5 | amp |
| TCGA-04-1347 | 3 | 100042408 | 101232014 | 1.995628366 | 6 | amp |
| TCGA-04-1347 | 3 | 101283575 | 102181233 | 1.584405408 | 5 | amp |
| TCGA-04-1347 | 3 | 102182994 | 107492518 | 2.187260409 | 6 | amp |
| TCGA-04-1347 | 3 | 107493514 | 107770819 | 1.484581343 | 5 | amp |
| TCGA-04-1347 | 3 | 107776309 | 108100458 | 2.18472103 | 6 | amp |
| TCGA-04-1347 | 3 | 108102388 | 108124302 | 1.34404126 | 5 | amp |
| TCGA-04-1347 | 3 | 108127068 | 108407002 | 2.20015858 | 6 | amp |
| TCGA-04-1347 | 3 | 108407391 | 108541837 | 1.269444289 | 5 | amp |
| TCGA-04-1347 | 3 | 108549511 | 108833317 | 2.385812559 | 6 | amp |
| TCGA-04-1347 | 3 | 109019188 | 110401318 | 1.264352049 | 5 | amp |
| TCGA-04-1347 | 3 | 110830867 | 111296432 | 2.010758364 | 6 | amp |
| TCGA-04-1347 | 3 | 111297858 | 111632597 | 1.674274559 | 5 | amp |
| TCGA-04-1347 | 3 | 111637840 | 111718479 | 2.094076629 | 6 | amp |
| TCGA-04-1347 | 3 | 111719555 | 111888229 | 1.656365069 | 5 | amp |
| TCGA-04-1347 | 3 | 111898342 | 112644045 | 2.148171639 | 6 | amp |
| TCGA-04-1347 | 3 | 112647606 | 113004435 | 1.450478484 | 5 | amp |
| TCGA-04-1347 | 3 | 113005465 | 113146229 | 2.326079991 | 6 | amp |
| TCGA-04-1347 | 3 | 113152401 | 113188118 | 1.529258936 | 5 | amp |
| TCGA-04-1347 | 3 | 113207730 | 113649739 | 1.936292732 | 6 | amp |
| TCGA-04-1347 | 3 | 113652308 | 113684223 | 1.384496752 | 5 | amp |
| TCGA-04-1347 | 3 | 113696985 | 113850285 | 2.352401023 | 6 | amp |
| TCGA-04-1347 | 3 | 113858294 | 113955930 | 1.020386669 | 4 | amp |
| TCGA-04-1347 | 3 | 114014346 | 118945891 | 2.0994269 | 6 | amp |
| TCGA-04-1347 | 3 | 118948639 | 119121256 | 1.41667639 | 5 | amp |
| TCGA-04-1347 | 3 | 119128272 | 119156955 | 2.679633444 | 6 | amp |

| | | | | | | |
|---|---|---|---|---|---|---|
| TCGA-04-1347 | 3 | 119165821 | 119217797 | 1.566922467 | 5 | amp |
| TCGA-04-1347 | 3 | 119219504 | 119263735 | 2.302052817 | 6 | amp |
| TCGA-04-1347 | 3 | 119276435 | 119428784 | 1.507693909 | 5 | amp |
| TCGA-04-1347 | 3 | 119434379 | 119456390 | 2.710899945 | 6 | amp |
| TCGA-04-1347 | 3 | 119458047 | 119582478 | 1.519641315 | 5 | amp |
| TCGA-04-1347 | 3 | 119585365 | 119721109 | 3.020629377 | 6 | amp |
| TCGA-04-1347 | 3 | 119892087 | 121345769 | 2.366024835 | 6 | amp |
| TCGA-04-1347 | 3 | 121350640 | 121396334 | 1.453275032 | 5 | amp |
| TCGA-04-1347 | 3 | 121400503 | 121630526 | 2.319595046 | 6 | amp |
| TCGA-04-1347 | 3 | 121631827 | 121634561 | 1.020593133 | 4 | amp |
| TCGA-04-1347 | 3 | 121641047 | 122420499 | 2.124119927 | 6 | amp |
| TCGA-04-1347 | 3 | 122422586 | 124129149 | 1.432228142 | 5 | amp |
| TCGA-04-1347 | 3 | 124132294 | 124196196 | 2.540595801 | 6 | amp |
| TCGA-04-1347 | 3 | 124201640 | 124385508 | 1.436877761 | 5 | amp |
| TCGA-04-1347 | 3 | 124385824 | 124432001 | 2.25293097 | 6 | amp |
| TCGA-04-1347 | 3 | 124436042 | 124952823 | 1.58851229 | 5 | amp |
| TCGA-04-1347 | 3 | 124953023 | 125279412 | 1.998477736 | 6 | amp |
| TCGA-04-1347 | 3 | 125282494 | 125855786 | 1.174049117 | 5 | amp |
| TCGA-04-1347 | 3 | 127641899 | 130368459 | 1.309631996 | 5 | amp |
| TCGA-04-1347 | 3 | 130377507 | 130733245 | 2.274948432 | 6 | amp |
| TCGA-04-1347 | 3 | 130734965 | 130748757 | 1.248632862 | 5 | amp |
| TCGA-04-1347 | 3 | 130799229 | 132192089 | 2.151960675 | 6 | amp |
| TCGA-04-1347 | 3 | 132193732 | 132203533 | 1.49935881 | 5 | amp |
| TCGA-04-1347 | 3 | 132207098 | 133293980 | 1.995685285 | 6 | amp |
| TCGA-04-1347 | 3 | 133302769 | 133306934 | 0.856209206 | 3 | amp |
| TCGA-04-1347 | 3 | 133320045 | 133376752 | 2.096885633 | 6 | amp |
| TCGA-04-1347 | 3 | 133377806 | 133478215 | 1.229763178 | 5 | amp |
| TCGA-04-1347 | 3 | 133483012 | 133489443 | 2.230326058 | 6 | amp |
| TCGA-04-1347 | 3 | 133494248 | 134226159 | 1.382690077 | 5 | amp |
| TCGA-04-1347 | 3 | 134250644 | 134280417 | 2.567244923 | 6 | amp |
| TCGA-04-1347 | 3 | 134322388 | 136082318 | 1.543430666 | 5 | amp |
| TCGA-04-1347 | 3 | 136085734 | 136699490 | 2.071538729 | 6 | amp |
| TCGA-04-1347 | 3 | 136700946 | 137958399 | 1.606085239 | 5 | amp |
| TCGA-04-1347 | 3 | 137960604 | 138003380 | 2.138573987 | 6 | amp |
| TCGA-04-1347 | 3 | 138007865 | 138038431 | 1.446981619 | 5 | amp |
| TCGA-04-1347 | 3 | 138043666 | 138119475 | 2.583987549 | 6 | amp |
| TCGA-04-1347 | 3 | 138121001 | 138193224 | 1.081272948 | 5 | amp |
| TCGA-04-1347 | 3 | 138195027 | 139102328 | 2.501068348 | 6 | amp |
| TCGA-04-1347 | 3 | 139171892 | 139237373 | 1.367252923 | 5 | amp |
| TCGA-04-1347 | 3 | 139257561 | 139346571 | 2.263541715 | 6 | amp |
| TCGA-04-1347 | 3 | 139894733 | 140419832 | 1.62231119 | 5 | amp |
| TCGA-04-1347 | 3 | 140675330 | 141632705 | 2.012798419 | 6 | amp |
| TCGA-04-1347 | 3 | 141634776 | 141671865 | 1.250375307 | 5 | amp |
| TCGA-04-1347 | 3 | 141678448 | 141724343 | 2.268449411 | 6 | amp |
| TCGA-04-1347 | 3 | 141812741 | 141901947 | 1.428999659 | 5 | amp |
| TCGA-04-1347 | 3 | 141904530 | 150262324 | 2.13594043 | 6 | amp |
| TCGA-04-1347 | 3 | 150280327 | 150344978 | 1.516160173 | 5 | amp |
| TCGA-04-1347 | 3 | 150377630 | 155762039 | 2.324460169 | 6 | amp |
| TCGA-04-1347 | 3 | 155762149 | 156009926 | 1.136117218 | 5 | amp |
| TCGA-04-1347 | 3 | 156139366 | 159614597 | 2.357521934 | 6 | amp |

| | | | | | | |
|---|---|---|---|---|---|---|
| TCGA-04-1347 | 3 | 159711198 | 183210045 | 3.253325114 | 6 | amp |
| TCGA-04-1347 | 3 | 183210268 | 183217646 | 1.016975917 | 4 | amp |
| TCGA-04-1347 | 3 | 183225831 | 183683346 | 2.477848345 | 6 | amp |
| TCGA-04-1347 | 3 | 183685428 | 183696484 | 1.044903612 | 5 | amp |
| TCGA-04-1347 | 3 | 183699468 | 183855620 | 2.321529544 | 6 | amp |
| TCGA-04-1347 | 3 | 183855624 | 183858541 | 1.168554826 | 5 | amp |
| TCGA-04-1347 | 3 | 183859665 | 183888021 | 2.271078074 | 6 | amp |
| TCGA-04-1347 | 3 | 183894758 | 184022195 | 1.277373651 | 5 | amp |
| TCGA-04-1347 | 3 | 184023516 | 184024640 | 2.240497432 | 6 | amp |
| TCGA-04-1347 | 3 | 184025108 | 184299415 | 1.323898888 | 5 | amp |
| TCGA-04-1347 | 3 | 184428763 | 186947736 | 2.642741463 | 6 | amp |
| TCGA-04-1347 | 3 | 186953433 | 186965206 | 1.218413069 | 5 | amp |
| TCGA-04-1347 | 3 | 186967996 | 195408471 | 3.206959921 | 6 | amp |
| TCGA-04-1347 | 3 | 195426162 | 195613969 | 1.427738963 | 5 | amp |
| TCGA-04-1347 | 3 | 195692296 | 196666332 | 2.346562138 | 6 | amp |
| TCGA-04-1347 | 3 | 196674011 | 196753640 | 1.475013845 | 5 | amp |
| TCGA-04-1347 | 3 | 196771486 | 197585800 | 2.911865859 | 6 | amp |
| TCGA-04-1347 | 3 | 197592257 | 197593096 | 0.687754983 | 3 | amp |
| TCGA-04-1347 | 3 | 197596969 | 197765547 | 2.536248106 | 6 | amp |
| TCGA-04-1347 | 3 | 197846558 | 197955154 | 1.497146279 | 5 | amp |
| TCGA-04-1347 | 4 | 53323 | 1993476 | 0.871584157 | 3 | amp |
| TCGA-04-1347 | 4 | 2074635 | 9157341 | 1.043697212 | 5 | amp |
| TCGA-04-1347 | 4 | 9212938 | 9370636 | 0.766029571 | 3 | amp |
| TCGA-04-1347 | 4 | 9385735 | 25420010 | 1.547075231 | 5 | amp |
| TCGA-04-1347 | 4 | 25664088 | 25676308 | 0.738054537 | 3 | amp |
| TCGA-04-1347 | 4 | 25677710 | 36216132 | 1.806340852 | 6 | amp |
| TCGA-04-1347 | 4 | 36230175 | 48487173 | 1.56406724 | 5 | amp |
| TCGA-04-1347 | 4 | 48504793 | 69978494 | 1.427304582 | 5 | amp |
| TCGA-04-1347 | 4 | 70066117 | 70355342 | 1.794336372 | 6 | amp |
| TCGA-04-1347 | 4 | 70359364 | 89680098 | 1.623754485 | 5 | amp |
| TCGA-04-1347 | 4 | 89688634 | 100480522 | 1.797350073 | 6 | amp |
| TCGA-04-1347 | 4 | 100496036 | 103504122 | 1.150991542 | 5 | amp |
| TCGA-04-1347 | 4 | 103505787 | 191013488 | 0.853067261 | 3 | amp |
| TCGA-04-1347 | 5 | 143116 | 5235332 | 0.786421164 | 3 | amp |
| TCGA-04-1347 | 5 | 5237026 | 43667165 | 1.436551616 | 5 | amp |
| TCGA-04-1347 | 5 | 43675572 | 52160700 | 1.932079061 | 6 | amp |
| TCGA-04-1347 | 5 | 52160800 | 66492484 | 1.291576269 | 5 | amp |
| TCGA-04-1347 | 5 | 67522490 | 68903098 | 1.056044746 | 4 | amp |
| TCGA-04-1347 | 5 | 68914137 | 70307211 | 1.369449517 | 5 | amp |
| TCGA-04-1347 | 5 | 70308158 | 72189402 | 1.080842231 | 4 | amp |
| TCGA-04-1347 | 5 | 72189426 | 126862497 | 1.319567167 | 5 | amp |
| TCGA-04-1347 | 5 | 126865976 | 131298419 | 1.855777996 | 6 | amp |
| TCGA-04-1347 | 5 | 131302046 | 131796419 | 1.198013972 | 5 | amp |
| TCGA-04-1347 | 5 | 131819554 | 132094574 | 2.055598588 | 6 | amp |
| TCGA-04-1347 | 5 | 132096438 | 140073877 | 1.376989383 | 5 | amp |
| TCGA-04-1347 | 5 | 140075035 | 140230636 | 1.767400976 | 6 | amp |
| TCGA-04-1347 | 5 | 140235562 | 140909276 | 1.343227565 | 5 | amp |
| TCGA-04-1347 | 5 | 140913890 | 141001129 | 1.823445828 | 6 | amp |
| TCGA-04-1347 | 5 | 141004703 | 141024770 | 0.878515921 | 4 | amp |
| TCGA-04-1347 | 5 | 141025298 | 141039068 | 1.312310284 | 5 | amp |

| | | | | | | |
|---|---|---|---|---|---|---|
| TCGA-04-1347 | 5 | 141041243 | 141052231 | 2.281156198 | 6 | amp |
| TCGA-04-1347 | 5 | 141052279 | 141316962 | 0.797790604 | 4 | amp |
| TCGA-04-1347 | 5 | 141318024 | 145647391 | 1.629354865 | 5 | amp |
| TCGA-04-1347 | 5 | 145648746 | 147813308 | 1.912242452 | 6 | amp |
| TCGA-04-1347 | 5 | 147817857 | 149505193 | 1.305702871 | 5 | amp |
| TCGA-04-1347 | 5 | 149506039 | 150072884 | 0.980193774 | 4 | amp |
| TCGA-04-1347 | 5 | 150073599 | 151166288 | 1.279656136 | 5 | amp |
| TCGA-04-1347 | 5 | 151169864 | 153433296 | 1.767278283 | 6 | amp |
| TCGA-04-1347 | 5 | 153674366 | 157162957 | 1.315017293 | 5 | amp |
| TCGA-04-1347 | 5 | 157164888 | 159521129 | 1.769875603 | 6 | amp |
| TCGA-04-1347 | 5 | 159626048 | 159832149 | 1.157165344 | 5 | amp |
| TCGA-04-1347 | 5 | 159833435 | 162869595 | 1.780692149 | 6 | amp |
| TCGA-04-1347 | 5 | 162880954 | 171341437 | 1.282360975 | 5 | amp |
| TCGA-04-1347 | 5 | 171384561 | 176715932 | 1.002411806 | 4 | amp |
| TCGA-04-1347 | 5 | 176718906 | 180625260 | 0.907563479 | 3 | amp |
| TCGA-04-1347 | 5 | 180625657 | 180899507 | 1.27688717 | 5 | amp |
| TCGA-04-1347 | 6 | 105907 | 24786066 | 1.869644752 | 6 | amp |
| TCGA-04-1347 | 6 | 24843075 | 24851007 | 1.622783875 | 5 | amp |
| TCGA-04-1347 | 6 | 24852764 | 24873207 | 2.919709756 | 6 | amp |
| TCGA-04-1347 | 6 | 24873829 | 24977261 | 1.726870242 | 5 | amp |
| TCGA-04-1347 | 6 | 25140443 | 25482587 | 3.126310971 | 6 | amp |
| TCGA-04-1347 | 6 | 25488701 | 25492273 | 1.649545532 | 5 | amp |
| TCGA-04-1347 | 6 | 25495330 | 25689482 | 2.583244249 | 6 | amp |
| TCGA-04-1347 | 6 | 25689670 | 25727568 | 1.728137631 | 5 | amp |
| TCGA-04-1347 | 6 | 25762145 | 25773941 | 2.520327173 | 6 | amp |
| TCGA-04-1347 | 6 | 25776768 | 25779468 | 1.743053795 | 5 | amp |
| TCGA-04-1347 | 6 | 25798959 | 29365418 | 1.928392555 | 6 | amp |
| TCGA-04-1347 | 6 | 29394434 | 29635750 | 1.610999328 | 5 | amp |
| TCGA-04-1347 | 6 | 29637955 | 29639272 | 3.128801092 | 6 | amp |
| TCGA-04-1347 | 6 | 29640245 | 29644825 | 1.307285158 | 5 | amp |
| TCGA-04-1347 | 6 | 29691907 | 29695928 | 4.175441787 | 6 | amp |
| TCGA-04-1347 | 6 | 29794758 | 29913289 | 0.952115693 | 4 | amp |
| TCGA-04-1347 | 6 | 30029223 | 30043582 | 2.833445346 | 6 | amp |
| TCGA-04-1347 | 6 | 30071229 | 30080613 | 1.587541522 | 5 | amp |
| TCGA-04-1347 | 6 | 30104805 | 30164527 | 2.441563502 | 6 | amp |
| TCGA-04-1347 | 6 | 30166163 | 30308399 | 1.687662896 | 5 | amp |
| TCGA-04-1347 | 6 | 30309462 | 30458334 | 2.95340853 | 6 | amp |
| TCGA-04-1347 | 6 | 30458871 | 30708610 | 1.413534177 | 5 | amp |
| TCGA-04-1347 | 6 | 30708888 | 30862485 | 2.55825333 | 6 | amp |
| TCGA-04-1347 | 6 | 30863145 | 30879050 | 1.045305544 | 5 | amp |
| TCGA-04-1347 | 6 | 30879181 | 30883033 | 2.810774395 | 6 | amp |
| TCGA-04-1347 | 6 | 30883091 | 31687099 | 1.556615648 | 5 | amp |
| TCGA-04-1347 | 6 | 31687834 | 31702045 | 3.64232258 | 6 | amp |
| TCGA-04-1347 | 6 | 31703967 | 31737893 | 1.291326837 | 5 | amp |
| TCGA-04-1347 | 6 | 31740695 | 31749736 | 3.105069596 | 6 | amp |
| TCGA-04-1347 | 6 | 31750268 | 32160329 | 1.256529156 | 5 | amp |
| TCGA-04-1347 | 6 | 32164655 | 32291439 | 2.31762231 | 6 | amp |
| TCGA-04-1347 | 6 | 32298291 | 33170904 | 1.537596811 | 5 | amp |
| TCGA-04-1347 | 6 | 33171273 | 33174482 | 3.551165373 | 6 | amp |
| TCGA-04-1347 | 6 | 33177362 | 33238077 | 1.592369634 | 5 | amp |

| | | | | | | |
|---|---|---|---|---|---|---|
| TCGA-04-1347 | 6 | 33240363 | 33254681 | 2.817126523 | 6 | amp |
| TCGA-04-1347 | 6 | 33254815 | 33409566 | 1.440507583 | 5 | amp |
| TCGA-04-1347 | 6 | 33410158 | 33414577 | 3.055067041 | 6 | amp |
| TCGA-04-1347 | 6 | 33419459 | 33654049 | 1.682301454 | 5 | amp |
| TCGA-04-1347 | 6 | 33654180 | 33657220 | 2.979696224 | 6 | amp |
| TCGA-04-1347 | 6 | 33657772 | 38029579 | 1.637883013 | 5 | amp |
| TCGA-04-1347 | 6 | 38050140 | 38980438 | 2.445335365 | 6 | amp |
| TCGA-04-1347 | 6 | 38994262 | 42613385 | 1.519094283 | 5 | amp |
| TCGA-04-1347 | 6 | 42615821 | 42634003 | 2.733653726 | 6 | amp |
| TCGA-04-1347 | 6 | 42637783 | 43013836 | 1.550948773 | 5 | amp |
| TCGA-04-1347 | 6 | 43013939 | 43024233 | 2.970950489 | 6 | amp |
| TCGA-04-1347 | 6 | 43025766 | 44216559 | 1.304432658 | 5 | amp |
| TCGA-04-1347 | 6 | 44217096 | 44218987 | 2.736629464 | 6 | amp |
| TCGA-04-1347 | 6 | 44219129 | 44392357 | 1.57928317 | 5 | amp |
| TCGA-04-1347 | 6 | 44393819 | 46129516 | 2.395848048 | 6 | amp |
| TCGA-04-1347 | 6 | 46133091 | 46214690 | 1.62333091 | 5 | amp |
| TCGA-04-1347 | 6 | 46216396 | 49448848 | 2.468338022 | 6 | amp |
| TCGA-04-1347 | 6 | 49456004 | 54025415 | 1.735199484 | 5 | amp |
| TCGA-04-1347 | 6 | 54025516 | 54245406 | 2.550725517 | 6 | amp |
| TCGA-04-1347 | 6 | 54254535 | 54806854 | 1.536544051 | 5 | amp |
| TCGA-04-1347 | 6 | 55039376 | 55264292 | 2.546770419 | 6 | amp |
| TCGA-04-1347 | 6 | 55266529 | 55304414 | 1.507732956 | 5 | amp |
| TCGA-04-1347 | 6 | 55360190 | 56600116 | 3.496969813 | 6 | amp |
| TCGA-04-1347 | 6 | 56716178 | 56716418 | 0.992988242 | 4 | amp |
| TCGA-04-1347 | 6 | 56765198 | 62604758 | 3.927436965 | 6 | amp |
| TCGA-04-1347 | 6 | 62611062 | 84375348 | 1.699310407 | 5 | amp |
| TCGA-04-1347 | 6 | 84566884 | 89554277 | 2.826849866 | 6 | amp |
| TCGA-04-1347 | 6 | 89559449 | 90077970 | 1.738900536 | 5 | amp |
| TCGA-04-1347 | 6 | 90082199 | 90574107 | 2.062339903 | 6 | amp |
| TCGA-04-1347 | 6 | 90575616 | 90718576 | 1.697755866 | 5 | amp |
| TCGA-04-1347 | 6 | 91226189 | 100897580 | 2.938834357 | 6 | amp |
| TCGA-04-1347 | 6 | 100898097 | 100957455 | 1.683911762 | 5 | amp |
| TCGA-04-1347 | 6 | 100957783 | 105581459 | 4.191936337 | 6 | amp |
| TCGA-04-1347 | 6 | 105606305 | 105609841 | 1.654578173 | 5 | amp |
| TCGA-04-1347 | 6 | 105725955 | 106553853 | 3.511440612 | 6 | amp |
| TCGA-04-1347 | 6 | 106554159 | 106555373 | 1.550374079 | 5 | amp |
| TCGA-04-1347 | 6 | 106634362 | 108385505 | 4.010628433 | 6 | amp |
| TCGA-04-1347 | 6 | 108492611 | 108499491 | 1.718419811 | 5 | amp |
| TCGA-04-1347 | 6 | 108501514 | 109763517 | 4.178648584 | 6 | amp |
| TCGA-04-1347 | 6 | 109763726 | 109772912 | 1.576970498 | 5 | amp |
| TCGA-04-1347 | 6 | 109773406 | 111281856 | 4.368783912 | 6 | amp |
| TCGA-04-1347 | 6 | 111283591 | 111289032 | 1.578097489 | 5 | amp |
| TCGA-04-1347 | 6 | 111303260 | 111621408 | 3.340320905 | 6 | amp |
| TCGA-04-1347 | 6 | 111628548 | 111632486 | 1.750141173 | 5 | amp |
| TCGA-04-1347 | 6 | 111634503 | 111995887 | 3.621994798 | 6 | amp |
| TCGA-04-1347 | 6 | 112015534 | 112015928 | 1.335468525 | 5 | amp |
| TCGA-04-1347 | 6 | 112017419 | 112537679 | 3.563542345 | 6 | amp |
| TCGA-04-1347 | 6 | 112574900 | 112671726 | 1.121056065 | 5 | amp |
| TCGA-04-1347 | 6 | 112677493 | 114281185 | 2.71784937 | 6 | amp |
| TCGA-04-1347 | 6 | 114378407 | 171055029 | 1.265796252 | 5 | amp |

| | | | | | | |
|---|---|---|---|---|---|---|
| TCGA-04-1347 | 7 | 195522 | 6862013 | 0.867427728 | 3 | amp |
| TCGA-04-1347 | 7 | 6862907 | 30402111 | 1.222255823 | 5 | amp |
| TCGA-04-1347 | 7 | 30465199 | 31146323 | 0.722735295 | 3 | amp |
| TCGA-04-1347 | 7 | 31377848 | 40723787 | 1.237670474 | 5 | amp |
| TCGA-04-1347 | 7 | 40789004 | 57188862 | 0.811437761 | 3 | amp |
| TCGA-04-1347 | 7 | 57193712 | 66490067 | 1.57656635 | 5 | amp |
| TCGA-04-1347 | 7 | 66514874 | 66749002 | 2.265499052 | 6 | amp |
| TCGA-04-1347 | 7 | 66751009 | 74710964 | 1.499212743 | 5 | amp |
| TCGA-04-1347 | 7 | 74712243 | 74991396 | 2.056105147 | 6 | amp |
| TCGA-04-1347 | 7 | 74993843 | 76654486 | 1.414835903 | 5 | amp |
| TCGA-04-1347 | 7 | 76656128 | 97820234 | 2.561019853 | 6 | amp |
| TCGA-04-1347 | 7 | 97820874 | 98453842 | 1.376581216 | 5 | amp |
| TCGA-04-1347 | 7 | 98457731 | 98569614 | 1.940085534 | 6 | amp |
| TCGA-04-1347 | 7 | 98573711 | 99227648 | 1.457893828 | 5 | amp |
| TCGA-04-1347 | 7 | 99235826 | 99377721 | 2.324241256 | 6 | amp |
| TCGA-04-1347 | 7 | 99381578 | 99831321 | 1.371804756 | 5 | amp |
| TCGA-04-1347 | 7 | 99906585 | 100007228 | 2.099368916 | 6 | amp |
| TCGA-04-1347 | 7 | 100013537 | 100193383 | 1.413682209 | 5 | amp |
| TCGA-04-1347 | 7 | 100197618 | 100225947 | 3.148432575 | 6 | amp |
| TCGA-04-1347 | 7 | 100226785 | 100479887 | 1.235839115 | 5 | amp |
| TCGA-04-1347 | 7 | 100481625 | 100687110 | 2.3790592 | 6 | amp |
| TCGA-04-1347 | 7 | 100691180 | 102065569 | 1.371623233 | 5 | amp |
| TCGA-04-1347 | 7 | 102079365 | 102329542 | 0.790654566 | 3 | amp |
| TCGA-04-1347 | 7 | 102330799 | 104808558 | 2.110841297 | 6 | amp |
| TCGA-04-1347 | 7 | 104809597 | 105665037 | 1.485466205 | 5 | amp |
| TCGA-04-1347 | 7 | 105667074 | 127026221 | 2.203319746 | 6 | amp |
| TCGA-04-1347 | 7 | 127031490 | 128481624 | 1.55074647 | 5 | amp |
| TCGA-04-1347 | 7 | 128483758 | 128802770 | 1.991636168 | 6 | amp |
| TCGA-04-1347 | 7 | 128804302 | 129120737 | 1.463731688 | 5 | amp |
| TCGA-04-1347 | 7 | 129122634 | 129520832 | 2.178566653 | 6 | amp |
| TCGA-04-1347 | 7 | 129658447 | 131859727 | 1.582231867 | 5 | amp |
| TCGA-04-1347 | 7 | 131864442 | 134133251 | 2.234642658 | 6 | amp |
| TCGA-04-1347 | 7 | 134133689 | 134346919 | 1.452108722 | 5 | amp |
| TCGA-04-1347 | 7 | 134363573 | 142460914 | 2.117962085 | 6 | amp |
| TCGA-04-1347 | 7 | 142471717 | 142836792 | 1.372121757 | 5 | amp |
| TCGA-04-1347 | 7 | 142880479 | 143002218 | 2.157146119 | 6 | amp |
| TCGA-04-1347 | 7 | 143013245 | 143658020 | 1.602166422 | 5 | amp |
| TCGA-04-1347 | 7 | 143701077 | 148716306 | 2.092211906 | 6 | amp |
| TCGA-04-1347 | 7 | 148718045 | 150936890 | 1.30585538 | 5 | amp |
| TCGA-04-1347 | 7 | 150937145 | 157341736 | 2.164612122 | 6 | amp |
| TCGA-04-1347 | 7 | 157361565 | 158468378 | 1.5077096 | 5 | amp |
| TCGA-04-1347 | 7 | 158472594 | 158531822 | 2.206596488 | 6 | amp |
| TCGA-04-1347 | 7 | 158534160 | 158935247 | 1.66036939 | 5 | amp |
| TCGA-04-1347 | 8 | 116074 | 2820950 | 1.411129757 | 5 | amp |
| TCGA-04-1347 | 8 | 2824059 | 6293685 | 2.148939625 | 6 | amp |
| TCGA-04-1347 | 8 | 6296425 | 6479273 | 1.570288576 | 5 | amp |
| TCGA-04-1347 | 8 | 6500482 | 7154765 | 2.011335487 | 6 | amp |
| TCGA-04-1347 | 8 | 7189865 | 7287683 | 1.349575879 | 5 | amp |
| TCGA-04-1347 | 8 | 7305493 | 7721100 | 1.983535045 | 6 | amp |
| TCGA-04-1347 | 8 | 7738912 | 8239272 | 1.582001306 | 5 | amp |

| | | | | | | |
|---|---|---|---|---|---|---|
| TCGA-04-1347 | 8 | 8643488 | 9623314 | 2.161005762 | 6 | amp |
| TCGA-04-1347 | 8 | 9623721 | 10557982 | 1.494565934 | 5 | amp |
| TCGA-04-1347 | 8 | 10622868 | 11167236 | 2.32891266 | 6 | amp |
| TCGA-04-1347 | 8 | 11172382 | 11629145 | 1.426957978 | 5 | amp |
| TCGA-04-1347 | 8 | 11637072 | 11689222 | 2.637108603 | 6 | amp |
| TCGA-04-1347 | 8 | 11695887 | 12291651 | 1.674763123 | 5 | amp |
| TCGA-04-1347 | 8 | 12435418 | 18666340 | 2.254664348 | 6 | amp |
| TCGA-04-1347 | 8 | 18725141 | 20037053 | 1.595892042 | 5 | amp |
| TCGA-04-1347 | 8 | 20038263 | 20075848 | 2.179999005 | 6 | amp |
| TCGA-04-1347 | 8 | 20077723 | 24167774 | 1.200142333 | 5 | amp |
| TCGA-04-1347 | 8 | 24168851 | 24811876 | 2.224092549 | 6 | amp |
| TCGA-04-1347 | 8 | 25101171 | 25277015 | 1.611525679 | 5 | amp |
| TCGA-04-1347 | 8 | 25279076 | 25365299 | 1.988935322 | 6 | amp |
| TCGA-04-1347 | 8 | 25702080 | 25899808 | 1.523415766 | 5 | amp |
| TCGA-04-1347 | 8 | 25902189 | 26223902 | 2.332057397 | 6 | amp |
| TCGA-04-1347 | 8 | 26227609 | 28828097 | 1.445472363 | 5 | amp |
| TCGA-04-1347 | 8 | 28837570 | 28956763 | 2.360155268 | 6 | amp |
| TCGA-04-1347 | 8 | 28965622 | 30560803 | 1.598304975 | 5 | amp |
| TCGA-04-1347 | 8 | 30565563 | 32614103 | 2.140410508 | 6 | amp |
| TCGA-04-1347 | 8 | 32616768 | 33827152 | 1.377705376 | 5 | amp |
| TCGA-04-1347 | 8 | 35383169 | 36793474 | 2.118168194 | 6 | amp |
| TCGA-04-1347 | 8 | 37595433 | 38853049 | 1.411772402 | 5 | amp |
| TCGA-04-1347 | 8 | 38865393 | 39682436 | 2.354886182 | 6 | amp |
| TCGA-04-1347 | 8 | 39691430 | 41585534 | 1.358926958 | 5 | amp |
| TCGA-04-1347 | 8 | 41591477 | 41839474 | 2.166224365 | 6 | amp |
| TCGA-04-1347 | 8 | 41844966 | 43054741 | 1.449740209 | 5 | amp |
| TCGA-04-1347 | 8 | 43147566 | 47886263 | 2.366381881 | 6 | amp |
| TCGA-04-1347 | 8 | 48114032 | 56859089 | 1.379867149 | 5 | amp |
| TCGA-04-1347 | 8 | 56860139 | 57214145 | 2.286920135 | 6 | amp |
| TCGA-04-1347 | 8 | 57218098 | 57878950 | 1.290851857 | 5 | amp |
| TCGA-04-1347 | 8 | 57890596 | 59477677 | 2.12150821 | 6 | amp |
| TCGA-04-1347 | 8 | 59483424 | 59512614 | 1.583283258 | 5 | amp |
| TCGA-04-1347 | 8 | 59513815 | 65509522 | 2.763043218 | 6 | amp |
| TCGA-04-1347 | 8 | 65517206 | 66619547 | 1.317573368 | 5 | amp |
| TCGA-04-1347 | 8 | 66620041 | 67786851 | 2.749616382 | 6 | amp |
| TCGA-04-1347 | 8 | 67789561 | 67809224 | 1.22221211 | 5 | amp |
| TCGA-04-1347 | 8 | 67813393 | 71495598 | 2.56845825 | 6 | amp |
| TCGA-04-1347 | 8 | 71495836 | 71496076 | 0.802887876 | 3 | amp |
| TCGA-04-1347 | 8 | 71499091 | 121353641 | 1.005881846 | 4 | amp |
| TCGA-04-1347 | 8 | 121354591 | 134026046 | 1.331091399 | 5 | amp |
| TCGA-04-1347 | 8 | 134030012 | 142151482 | 1.128613701 | 4 | amp |
| TCGA-04-1347 | 8 | 142154242 | 146279593 | 0.585340131 | 3 | amp |
| TCGA-04-1347 | 9 | 17322 | 289605 | 1.327169768 | 5 | amp |
| TCGA-04-1347 | 9 | 304522 | 1057284 | 0.926197601 | 4 | amp |
| TCGA-04-1347 | 9 | 2029014 | 14740249 | 1.193595349 | 5 | amp |
| TCGA-04-1347 | 9 | 14746348 | 15459910 | 0.940165585 | 4 | amp |
| TCGA-04-1347 | 9 | 15465487 | 32984861 | 1.221249907 | 5 | amp |
| TCGA-04-1347 | 9 | 32985879 | 34660610 | 0.873927303 | 4 | amp |
| TCGA-04-1347 | 9 | 34660801 | 35101609 | 0.661402351 | 3 | amp |
| TCGA-04-1347 | 9 | 35101667 | 35698530 | 0.90431704 | 4 | amp |

| | | | | | | |
|---|---|---|---|---|---|---|
| TCGA-04-1347 | 9 | 35698554 | 35811468 | 0.645318829 | 3 | amp |
| TCGA-04-1347 | 9 | 35811609 | 40337489 | 0.94973684 | 4 | amp |
| TCGA-04-1347 | 9 | 40491573 | 65649139 | 1.126902413 | 5 | amp |
| TCGA-04-1347 | 9 | 65649990 | 67952034 | 0.79279888 | 4 | amp |
| TCGA-04-1347 | 9 | 67954675 | 90344688 | 1.194339329 | 5 | amp |
| TCGA-04-1347 | 9 | 90345204 | 101829376 | 0.911960121 | 4 | amp |
| TCGA-04-1347 | 9 | 101830834 | 115593203 | 1.220160509 | 5 | amp |
| TCGA-04-1347 | 9 | 115598441 | 116085482 | 0.927790587 | 4 | amp |
| TCGA-04-1347 | 9 | 116091137 | 117165244 | 0.644568524 | 3 | amp |
| TCGA-04-1347 | 9 | 117165407 | 123857350 | 0.995561068 | 4 | amp |
| TCGA-04-1347 | 9 | 123858650 | 124545879 | 1.157215484 | 5 | amp |
| TCGA-04-1347 | 9 | 124751362 | 125682300 | 0.899857003 | 4 | amp |
| TCGA-04-1347 | 9 | 125719293 | 126531877 | 1.189928894 | 5 | amp |
| TCGA-04-1347 | 9 | 126554827 | 130106625 | 0.915013295 | 4 | amp |
| TCGA-04-1347 | 9 | 130107658 | 131002329 | 0.699469998 | 3 | amp |
| TCGA-04-1347 | 9 | 131004507 | 131073903 | 1.076733774 | 4 | amp |
| TCGA-04-1347 | 9 | 131076037 | 133987076 | 0.719422632 | 3 | amp |
| TCGA-04-1347 | 9 | 133989924 | 135704132 | 0.904720134 | 4 | amp |
| TCGA-04-1347 | 9 | 135730217 | 136084042 | 0.744666198 | 3 | amp |
| TCGA-04-1347 | X | 44109385 | 44950158 | 1.191051779 | 5 | amp |
| TCGA-04-1347 | X | 44966597 | 47032621 | 1.060515399 | 4 | amp |
| TCGA-04-1347 | X | 47068993 | 47430908 | 0.759841728 | 3 | amp |
| TCGA-04-1347 | X | 47435707 | 48211619 | 0.899158105 | 4 | amp |
| TCGA-04-1347 | X | 48213421 | 48834874 | 0.704321904 | 3 | amp |
| TCGA-04-1347 | X | 48837616 | 48933624 | 0.928905629 | 4 | amp |
| TCGA-04-1347 | X | 49161822 | 49365476 | 1.395852442 | 5 | amp |
| TCGA-04-1347 | X | 49368213 | 49846520 | 0.946114527 | 4 | amp |
| TCGA-04-1347 | X | 49850616 | 49856890 | 1.486101683 | 5 | amp |
| TCGA-04-1347 | X | 49920792 | 73814242 | 0.659028984 | 3 | amp |
| TCGA-04-1347 | X | 73815607 | 76938246 | 0.870166389 | 4 | amp |
| TCGA-04-1347 | X | 76938316 | 79932900 | 0.720937291 | 3 | amp |
| TCGA-04-1347 | X | 79936798 | 85214043 | 0.881162799 | 4 | amp |
| TCGA-04-1347 | X | 85218623 | 101409249 | 0.723816975 | 3 | amp |
| TCGA-04-1347 | X | 101768943 | 102318253 | 1.102002091 | 5 | amp |
| TCGA-04-1347 | X | 102343408 | 103216747 | 0.697109148 | 3 | amp |
| TCGA-04-1347 | X | 103219085 | 106117237 | 0.910563937 | 4 | amp |
| TCGA-04-1347 | X | 106144022 | 107435816 | 0.703750706 | 3 | amp |
| TCGA-04-1347 | X | 107436802 | 110463690 | 0.908878087 | 4 | amp |
| TCGA-04-1347 | X | 110489775 | 111003274 | 0.740973426 | 3 | amp |
| TCGA-04-1347 | Y | 3447228 | 13488232 | 0.631137208 | 3 | amp |
| TCGA-04-1347 | Y | 13496242 | 14619201 | 0.911468462 | 4 | amp |
| TCGA-04-1348 | 1 | 7807745 | 9097866 | 0.780407905 | 3 | amp |
| TCGA-04-1348 | 1 | 10093680 | 10421936 | 1.31859134 | 5 | amp |
| TCGA-04-1348 | 1 | 10423311 | 11137742 | 0.609684038 | 3 | amp |
| TCGA-04-1348 | 1 | 11139733 | 11199760 | 0.869451608 | 4 | amp |
| TCGA-04-1348 | 1 | 11204698 | 11300661 | 1.504035739 | 5 | amp |
| TCGA-04-1348 | 1 | 12262005 | 12262245 | 1.571500412 | 5 | amp |
| TCGA-04-1348 | 1 | 12266756 | 12416637 | 1.878525817 | 6 | amp |
| TCGA-04-1348 | 1 | 12418465 | 12943249 | 1.30289457 | 5 | amp |
| TCGA-04-1348 | 1 | 12952725 | 14109451 | 0.940292485 | 4 | amp |

| | | | | | | |
|---|---|---|---|---|---|---|
| TCGA-04-1348 | 1 | 14109634 | 16262457 | 0.723509754 | 3 | amp |
| TCGA-04-1348 | 1 | 16890379 | 16918548 | 1.307908837 | 5 | amp |
| TCGA-04-1348 | 1 | 19438979 | 19496037 | 1.330551692 | 5 | amp |
| TCGA-04-1348 | 1 | 19497196 | 19514044 | 1.963983979 | 6 | amp |
| TCGA-04-1348 | 1 | 19518626 | 19549342 | 0.96387083 | 4 | amp |
| TCGA-04-1348 | 1 | 19549826 | 19567692 | 0.688776703 | 3 | amp |
| TCGA-04-1348 | 1 | 21151581 | 21546655 | 1.485593543 | 5 | amp |
| TCGA-04-1348 | 1 | 21751054 | 21809937 | 1.314292096 | 5 | amp |
| TCGA-04-1348 | 1 | 21961203 | 22084321 | 1.246274625 | 5 | amp |
| TCGA-04-1348 | 1 | 22816399 | 22848972 | 1.367843129 | 5 | amp |
| TCGA-04-1348 | 1 | 23381517 | 23667542 | 1.249851669 | 5 | amp |
| TCGA-04-1348 | 1 | 24668533 | 24780005 | 0.742388425 | 3 | amp |
| TCGA-04-1348 | 1 | 24782545 | 25747323 | 0.989089986 | 4 | amp |
| TCGA-04-1348 | 1 | 25773323 | 25824975 | 1.377480255 | 5 | amp |
| TCGA-04-1348 | 1 | 28071096 | 28862572 | 0.984905489 | 4 | amp |
| TCGA-04-1348 | 1 | 28862683 | 31347469 | 0.645820007 | 3 | amp |
| TCGA-04-1348 | 1 | 31349345 | 31821868 | 1.077053022 | 4 | amp |
| TCGA-04-1348 | 1 | 31836871 | 32090778 | 0.636558596 | 3 | amp |
| TCGA-04-1348 | 1 | 32849419 | 34112461 | 0.714167787 | 3 | amp |
| TCGA-04-1348 | 1 | 34117841 | 35454612 | 0.890047047 | 4 | amp |
| TCGA-04-1348 | 1 | 35457791 | 35827424 | 1.197511351 | 5 | amp |
| TCGA-04-1348 | 1 | 35835621 | 35900708 | 1.956350012 | 6 | amp |
| TCGA-04-1348 | 1 | 35906528 | 36282685 | 0.981127771 | 4 | amp |
| TCGA-04-1348 | 1 | 36288402 | 36319211 | 2.004854794 | 6 | amp |
| TCGA-04-1348 | 1 | 36348974 | 36551659 | 1.01863015 | 4 | amp |
| TCGA-04-1348 | 1 | 36552226 | 39715790 | 0.705963103 | 3 | amp |
| TCGA-04-1348 | 1 | 39719948 | 39758536 | 0.989900394 | 4 | amp |
| TCGA-04-1348 | 1 | 39759060 | 39930835 | 1.309324309 | 5 | amp |
| TCGA-04-1348 | 1 | 40205825 | 40434448 | 0.755986654 | 3 | amp |
| TCGA-04-1348 | 1 | 40435118 | 43162951 | 0.943204073 | 4 | amp |
| TCGA-04-1348 | 1 | 43166448 | 43675755 | 0.687044595 | 3 | amp |
| TCGA-04-1348 | 1 | 44084675 | 44422684 | 0.875968132 | 3 | amp |
| TCGA-04-1348 | 1 | 45811457 | 47080755 | 0.711514103 | 3 | amp |
| TCGA-04-1348 | 1 | 47101424 | 47284514 | 1.016857264 | 4 | amp |
| TCGA-04-1348 | 1 | 47310185 | 51439993 | 1.315644362 | 5 | amp |
| TCGA-04-1348 | 1 | 51702339 | 51868227 | 0.883730793 | 4 | amp |
| TCGA-04-1348 | 1 | 51869087 | 52301903 | 1.676578656 | 6 | amp |
| TCGA-04-1348 | 1 | 52301985 | 52811932 | 0.940379041 | 4 | amp |
| TCGA-04-1348 | 1 | 52849016 | 52876889 | 1.323262102 | 5 | amp |
| TCGA-04-1348 | 1 | 52878180 | 52992013 | 1.646278388 | 6 | amp |
| TCGA-04-1348 | 1 | 53072306 | 53458999 | 1.056288312 | 4 | amp |
| TCGA-04-1348 | 1 | 53480518 | 53701337 | 0.542416226 | 3 | amp |
| TCGA-04-1348 | 1 | 53704016 | 54562171 | 0.95447201 | 4 | amp |
| TCGA-04-1348 | 1 | 54605155 | 55538556 | 0.599592514 | 3 | amp |
| TCGA-04-1348 | 1 | 55539479 | 55603394 | 1.309767616 | 5 | amp |
| TCGA-04-1348 | 1 | 55603467 | 60370756 | 1.657527357 | 6 | amp |
| TCGA-04-1348 | 1 | 60373408 | 62960161 | 1.238283777 | 5 | amp |
| TCGA-04-1348 | 1 | 62961241 | 64624889 | 1.900735095 | 6 | amp |
| TCGA-04-1348 | 1 | 64643104 | 65524213 | 1.230304236 | 5 | amp |
| TCGA-04-1348 | 1 | 65656332 | 67145457 | 1.674381904 | 6 | amp |

| | | | | | | |
|---|---|---|---|---|---|---|
| TCGA-04-1348 | 1 | 67147501 | 68955332 | 1.293468467 | 5 | amp |
| TCGA-04-1348 | 1 | 68960065 | 70653044 | 1.696018042 | 6 | amp |
| TCGA-04-1348 | 1 | 70654745 | 72163876 | 1.377344971 | 5 | amp |
| TCGA-04-1348 | 1 | 72241797 | 75180348 | 1.758638486 | 6 | amp |
| TCGA-04-1348 | 1 | 75184854 | 76259963 | 1.332153436 | 5 | amp |
| TCGA-04-1348 | 1 | 76260144 | 76773649 | 1.783410425 | 6 | amp |
| TCGA-04-1348 | 1 | 76775603 | 78959247 | 1.393966937 | 5 | amp |
| TCGA-04-1348 | 1 | 78963533 | 84680037 | 1.777447887 | 6 | amp |
| TCGA-04-1348 | 1 | 84700805 | 86143017 | 1.334804071 | 5 | amp |
| TCGA-04-1348 | 1 | 86144363 | 87108313 | 1.654530877 | 6 | amp |
| TCGA-04-1348 | 1 | 87170558 | 89271756 | 1.341298565 | 5 | amp |
| TCGA-04-1348 | 1 | 89272940 | 89520581 | 1.701849661 | 6 | amp |
| TCGA-04-1348 | 1 | 89520786 | 90180544 | 1.202066741 | 5 | amp |
| TCGA-04-1348 | 1 | 90398595 | 91861965 | 1.810843842 | 6 | amp |
| TCGA-04-1348 | 1 | 91866543 | 94363484 | 1.40053613 | 5 | amp |
| TCGA-04-1348 | 1 | 94367117 | 94510354 | 0.807670678 | 4 | amp |
| TCGA-04-1348 | 1 | 94512441 | 94668770 | 1.322153494 | 5 | amp |
| TCGA-04-1348 | 1 | 94669427 | 100207841 | 1.699614181 | 6 | amp |
| TCGA-04-1348 | 1 | 100212807 | 100819422 | 1.302720506 | 5 | amp |
| TCGA-04-1348 | 1 | 100843079 | 101343485 | 1.788106979 | 6 | amp |
| TCGA-04-1348 | 1 | 101354285 | 101383717 | 0.704823355 | 4 | amp |
| TCGA-04-1348 | 1 | 101387209 | 108328158 | 1.748234628 | 6 | amp |
| TCGA-04-1348 | 1 | 108417460 | 109608865 | 1.224234555 | 5 | amp |
| TCGA-04-1348 | 1 | 109867495 | 110087042 | 0.820675031 | 3 | amp |
| TCGA-04-1348 | 1 | 110116319 | 110149087 | 1.485690651 | 5 | amp |
| TCGA-04-1348 | 1 | 110882018 | 111668942 | 1.260874342 | 5 | amp |
| TCGA-04-1348 | 1 | 111673990 | 113068806 | 0.985415323 | 4 | amp |
| TCGA-04-1348 | 1 | 113084502 | 113197309 | 1.244079754 | 5 | amp |
| TCGA-04-1348 | 1 | 113471701 | 114367838 | 1.386328041 | 5 | amp |
| TCGA-04-1348 | 1 | 114372211 | 114964277 | 0.990241976 | 4 | amp |
| TCGA-04-1348 | 1 | 114967174 | 115168622 | 1.722918753 | 6 | amp |
| TCGA-04-1348 | 1 | 115215693 | 115269717 | 0.876961916 | 4 | amp |
| TCGA-04-1348 | 1 | 115272840 | 117127710 | 1.315263905 | 5 | amp |
| TCGA-04-1348 | 1 | 117131280 | 117661442 | 0.906295328 | 4 | amp |
| TCGA-04-1348 | 1 | 117663279 | 118694041 | 1.754168979 | 6 | amp |
| TCGA-04-1348 | 1 | 118727646 | 145368622 | 1.27844367 | 5 | amp |
| TCGA-04-1348 | 1 | 145681998 | 147085019 | 1.307243537 | 5 | amp |
| TCGA-04-1348 | 1 | 147086200 | 147566175 | 0.852838058 | 4 | amp |
| TCGA-04-1348 | 1 | 147575887 | 148338478 | 1.330754342 | 5 | amp |
| TCGA-04-1348 | 1 | 148340204 | 148933389 | 1.075527773 | 4 | amp |
| TCGA-04-1348 | 1 | 149038809 | 150259403 | 0.641532613 | 3 | amp |
| TCGA-04-1348 | 1 | 150266737 | 150598318 | 0.889978253 | 4 | amp |
| TCGA-04-1348 | 1 | 150598908 | 150680897 | 2.162498643 | 6 | amp |
| TCGA-04-1348 | 1 | 150681343 | 150812143 | 1.325739446 | 5 | amp |
| TCGA-04-1348 | 1 | 150814861 | 150917608 | 0.907357678 | 4 | amp |
| TCGA-04-1348 | 1 | 150919304 | 152884086 | 0.670555772 | 3 | amp |
| TCGA-04-1348 | 1 | 152944224 | 153318682 | 1.159605473 | 4 | amp |
| TCGA-04-1348 | 1 | 153991356 | 154067641 | 1.334078011 | 5 | amp |
| TCGA-04-1348 | 1 | 154072426 | 154207788 | 0.880177028 | 4 | amp |
| TCGA-04-1348 | 1 | 154209004 | 154241436 | 1.29859998 | 5 | amp |

| | | | | | | |
|---|---|---|---|---|---|---|
| TCGA-04-1348 | 1 | 155307406 | 155385744 | 1.318894319 | 5 | amp |
| TCGA-04-1348 | 1 | 155403054 | 155912662 | 0.809670872 | 3 | amp |
| TCGA-04-1348 | 1 | 156930170 | 157494387 | 0.83542403 | 3 | amp |
| TCGA-04-1348 | 1 | 157497365 | 157718435 | 1.388039283 | 5 | amp |
| TCGA-04-1348 | 1 | 157718647 | 157773947 | 1.836727649 | 6 | amp |
| TCGA-04-1348 | 1 | 157776841 | 158226894 | 0.712773196 | 4 | amp |
| TCGA-04-1348 | 1 | 158227195 | 159161915 | 1.380271667 | 5 | amp |
| TCGA-04-1348 | 1 | 159162323 | 159860460 | 0.99658419 | 4 | amp |
| TCGA-04-1348 | 1 | 159862882 | 160105427 | 0.552405547 | 3 | amp |
| TCGA-04-1348 | 1 | 160105555 | 160395217 | 0.994627202 | 4 | amp |
| TCGA-04-1348 | 1 | 160456460 | 160832496 | 1.391886392 | 5 | amp |
| TCGA-04-1348 | 1 | 161172162 | 161519680 | 0.721794719 | 3 | amp |
| TCGA-04-1348 | 1 | 161551223 | 162725566 | 0.939509243 | 4 | amp |
| TCGA-04-1348 | 1 | 162729557 | 165173316 | 1.283145386 | 5 | amp |
| TCGA-04-1348 | 1 | 165175035 | 167803321 | 0.852740655 | 4 | amp |
| TCGA-04-1348 | 1 | 167805542 | 179033655 | 1.321275238 | 5 | amp |
| TCGA-04-1348 | 1 | 179035904 | 179102505 | 0.693098684 | 4 | amp |
| TCGA-04-1348 | 1 | 179112063 | 180053352 | 1.268390071 | 5 | amp |
| TCGA-04-1348 | 1 | 180056782 | 180257694 | 0.730862339 | 4 | amp |
| TCGA-04-1348 | 1 | 180283781 | 180794510 | 1.747611256 | 6 | amp |
| TCGA-04-1348 | 1 | 180803975 | 182763600 | 0.868395365 | 4 | amp |
| TCGA-04-1348 | 1 | 182772809 | 183387419 | 1.323039312 | 5 | amp |
| TCGA-04-1348 | 1 | 183481927 | 183521069 | 2.073737567 | 6 | amp |
| TCGA-04-1348 | 1 | 183525248 | 183596757 | 0.908804858 | 4 | amp |
| TCGA-04-1348 | 1 | 183599563 | 186266105 | 1.653294132 | 6 | amp |
| TCGA-04-1348 | 1 | 186269163 | 186329149 | 1.356051933 | 5 | amp |
| TCGA-04-1348 | 1 | 186329360 | 186648631 | 0.956354284 | 4 | amp |
| TCGA-04-1348 | 1 | 186823442 | 197101512 | 1.653209944 | 6 | amp |
| TCGA-04-1348 | 1 | 197102422 | 197404768 | 1.179843274 | 5 | amp |
| TCGA-04-1348 | 1 | 197407620 | 197704801 | 1.921796083 | 6 | amp |
| TCGA-04-1348 | 1 | 197874916 | 197898431 | 0.65781713 | 4 | amp |
| TCGA-04-1348 | 1 | 198201678 | 198828287 | 1.79672553 | 6 | amp |
| TCGA-04-1348 | 1 | 199996947 | 200827214 | 1.372046495 | 5 | amp |
| TCGA-04-1348 | 1 | 201749467 | 202318304 | 0.667912694 | 3 | amp |
| TCGA-04-1348 | 1 | 202385898 | 202746243 | 0.978082176 | 4 | amp |
| TCGA-04-1348 | 1 | 202850048 | 202931860 | 0.688093312 | 3 | amp |
| TCGA-04-1348 | 1 | 203667263 | 203803009 | 1.118043754 | 5 | amp |
| TCGA-04-1348 | 1 | 205052644 | 205138985 | 1.377869531 | 5 | amp |
| TCGA-04-1348 | 1 | 205566882 | 207235480 | 0.726222245 | 3 | amp |
| TCGA-04-1348 | 1 | 207235922 | 207803973 | 1.387870307 | 5 | amp |
| TCGA-04-1348 | 1 | 207812687 | 207959055 | 0.946300071 | 4 | amp |
| TCGA-04-1348 | 1 | 207963563 | 209878391 | 0.631739901 | 3 | amp |
| TCGA-04-1348 | 1 | 209879070 | 210001521 | 0.899121703 | 4 | amp |
| TCGA-04-1348 | 1 | 210003412 | 212220818 | 1.207797691 | 5 | amp |
| TCGA-04-1348 | 1 | 212225105 | 212538757 | 1.745644976 | 6 | amp |
| TCGA-04-1348 | 1 | 212548528 | 213009532 | 1.126142216 | 5 | amp |
| TCGA-04-1348 | 1 | 213037018 | 217787573 | 1.603413261 | 6 | amp |
| TCGA-04-1348 | 1 | 217793066 | 220835510 | 1.396203835 | 5 | amp |
| TCGA-04-1348 | 1 | 220869893 | 222721405 | 0.772104193 | 4 | amp |
| TCGA-04-1348 | 1 | 222731997 | 225742816 | 1.23602367 | 5 | amp |

| | | | | | | |
|---|---|---|---|---|---|---|
| TCGA-04-1348 | 1 | 226128505 | 226556080 | 0.943910024 | 3 | amp |
| TCGA-04-1348 | 1 | 227203719 | 227935963 | 1.414107018 | 5 | amp |
| TCGA-04-1348 | 1 | 229178170 | 229623406 | 1.346211862 | 5 | amp |
| TCGA-04-1348 | 1 | 229625655 | 230513331 | 0.816988588 | 4 | amp |
| TCGA-04-1348 | 1 | 230795170 | 230810926 | 2.049115607 | 6 | amp |
| TCGA-04-1348 | 1 | 230814578 | 233336014 | 1.00424891 | 4 | amp |
| TCGA-04-1348 | 1 | 233344229 | 235747148 | 1.197831024 | 5 | amp |
| TCGA-04-1348 | 1 | 235826158 | 235970097 | 1.664105015 | 6 | amp |
| TCGA-04-1348 | 1 | 235971714 | 237843936 | 1.35123317 | 5 | amp |
| TCGA-04-1348 | 1 | 237850719 | 237972387 | 1.675846137 | 6 | amp |
| TCGA-04-1348 | 1 | 237982293 | 244716126 | 1.262422153 | 5 | amp |
| TCGA-04-1348 | 1 | 244723908 | 246921712 | 0.907130925 | 4 | amp |
| TCGA-04-1348 | 1 | 246922274 | 247162755 | 1.335353566 | 5 | amp |
| TCGA-04-1348 | 1 | 247163174 | 247729306 | 0.752011971 | 4 | amp |
| TCGA-04-1348 | 1 | 247737384 | 249106573 | 1.318977077 | 5 | amp |
| TCGA-04-1348 | 10 | 92880 | 298468 | 1.447384587 | 5 | amp |
| TCGA-04-1348 | 10 | 323180 | 863866 | 0.729847365 | 3 | amp |
| TCGA-04-1348 | 10 | 866702 | 1043257 | 1.494794808 | 5 | amp |
| TCGA-04-1348 | 10 | 1044928 | 4889760 | 0.652896557 | 3 | amp |
| TCGA-04-1348 | 10 | 5005618 | 5203996 | 1.198358773 | 5 | amp |
| TCGA-04-1348 | 10 | 5204749 | 5255159 | 1.945430881 | 6 | amp |
| TCGA-04-1348 | 10 | 5258654 | 5777567 | 1.334151416 | 5 | amp |
| TCGA-04-1348 | 10 | 5781540 | 13749134 | 0.905770154 | 4 | amp |
| TCGA-04-1348 | 10 | 13779826 | 15713683 | 1.265078036 | 5 | amp |
| TCGA-04-1348 | 10 | 15714565 | 16528663 | 2.126624265 | 6 | amp |
| TCGA-04-1348 | 10 | 16546968 | 18629940 | 1.427832545 | 5 | amp |
| TCGA-04-1348 | 10 | 18689934 | 21435458 | 1.95180499 | 6 | amp |
| TCGA-04-1348 | 10 | 21461268 | 21901388 | 1.05896963 | 5 | amp |
| TCGA-04-1348 | 10 | 21903716 | 22045709 | 1.983581677 | 6 | amp |
| TCGA-04-1348 | 10 | 22048052 | 22856858 | 1.188312849 | 5 | amp |
| TCGA-04-1348 | 10 | 22862192 | 23321990 | 1.886470703 | 6 | amp |
| TCGA-04-1348 | 10 | 23326153 | 25314408 | 1.412745336 | 5 | amp |
| TCGA-04-1348 | 10 | 25509943 | 26434478 | 1.725930467 | 6 | amp |
| TCGA-04-1348 | 10 | 26436340 | 26581935 | 1.319389528 | 5 | amp |
| TCGA-04-1348 | 10 | 26589653 | 26830655 | 1.845351573 | 6 | amp |
| TCGA-04-1348 | 10 | 26849022 | 27013057 | 1.138412234 | 5 | amp |
| TCGA-04-1348 | 10 | 27024148 | 27342344 | 1.783649297 | 6 | amp |
| TCGA-04-1348 | 10 | 27349249 | 28196708 | 1.184367045 | 5 | amp |
| TCGA-04-1348 | 10 | 28223879 | 28899808 | 1.650234488 | 6 | amp |
| TCGA-04-1348 | 10 | 28900656 | 29776279 | 0.667483099 | 3 | amp |
| TCGA-04-1348 | 10 | 29777487 | 31139337 | 0.989487291 | 4 | amp |
| TCGA-04-1348 | 10 | 31165884 | 31815924 | 1.421088269 | 5 | amp |
| TCGA-04-1348 | 10 | 31816046 | 32143183 | 1.782548661 | 6 | amp |
| TCGA-04-1348 | 10 | 32150274 | 32863419 | 1.368767802 | 5 | amp |
| TCGA-04-1348 | 10 | 32873150 | 33224512 | 1.714669785 | 6 | amp |
| TCGA-04-1348 | 10 | 33468968 | 38127095 | 1.29769156 | 5 | amp |
| TCGA-04-1348 | 10 | 38145140 | 43297685 | 0.988893636 | 4 | amp |
| TCGA-04-1348 | 10 | 43312023 | 46552559 | 0.652569266 | 3 | amp |
| TCGA-04-1348 | 10 | 46564901 | 46673730 | 1.414833771 | 5 | amp |
| TCGA-04-1348 | 10 | 46675443 | 48739478 | 0.639026436 | 3 | amp |

| | | | | | | |
|---|---|---|---|---|---|---|
| TCGA-04-1348 | 10 | 48751794 | 48860629 | 1.42476608 | 5 | amp |
| TCGA-04-1348 | 10 | 48862343 | 49265529 | 0.620642471 | 3 | amp |
| TCGA-04-1348 | 10 | 49272333 | 49661527 | 1.252853764 | 5 | amp |
| TCGA-04-1348 | 10 | 49662077 | 51829508 | 0.709561082 | 3 | amp |
| TCGA-04-1348 | 10 | 51837838 | 61444071 | 0.943183538 | 4 | amp |
| TCGA-04-1348 | 10 | 61552291 | 61802542 | 1.344823963 | 5 | amp |
| TCGA-04-1348 | 10 | 61815364 | 61932173 | 1.824389216 | 6 | amp |
| TCGA-04-1348 | 10 | 61932616 | 62023805 | 1.476563308 | 5 | amp |
| TCGA-04-1348 | 10 | 62029847 | 70225616 | 0.951744616 | 4 | amp |
| TCGA-04-1348 | 10 | 70227851 | 70922274 | 0.704066077 | 3 | amp |
| TCGA-04-1348 | 10 | 73856999 | 74104916 | 0.726993163 | 3 | amp |
| TCGA-04-1348 | 10 | 74114468 | 75239294 | 0.983962759 | 4 | amp |
| TCGA-04-1348 | 10 | 75258336 | 75335462 | 0.793835711 | 3 | amp |
| TCGA-04-1348 | 10 | 75802815 | 75960618 | 0.772784367 | 3 | amp |
| TCGA-04-1348 | 10 | 75984262 | 76780575 | 1.338292851 | 5 | amp |
| TCGA-04-1348 | 10 | 76780843 | 79493777 | 0.834451155 | 3 | amp |
| TCGA-04-1348 | 10 | 89123945 | 89272989 | 0.76035511 | 3 | amp |
| TCGA-04-1348 | 10 | 89280859 | 92655285 | 1.009705507 | 4 | amp |
| TCGA-04-1348 | 10 | 92655595 | 93221121 | 1.456276363 | 5 | amp |
| TCGA-04-1348 | 10 | 93221848 | 94835769 | 1.01316645 | 4 | amp |
| TCGA-04-1348 | 10 | 94836256 | 95381878 | 0.687565976 | 3 | amp |
| TCGA-04-1348 | 10 | 95385278 | 98082509 | 0.929781329 | 4 | amp |
| TCGA-04-1348 | 10 | 98083964 | 98392850 | 1.316937732 | 5 | amp |
| TCGA-04-1348 | 10 | 98405204 | 99025929 | 0.670437687 | 3 | amp |
| TCGA-04-1348 | 10 | 100242315 | 101579055 | 1.256508058 | 5 | amp |
| TCGA-04-1348 | 10 | 101590006 | 102719278 | 0.736149657 | 3 | amp |
| TCGA-04-1348 | 10 | 103427599 | 103609710 | 0.731540944 | 3 | amp |
| TCGA-04-1348 | 10 | 103649117 | 103753387 | 1.512107603 | 5 | amp |
| TCGA-04-1348 | 10 | 103754992 | 104130660 | 0.690201272 | 3 | amp |
| TCGA-04-1348 | 10 | 104416024 | 104850782 | 0.726848314 | 3 | amp |
| TCGA-04-1348 | 10 | 104851286 | 105093755 | 1.527780931 | 5 | amp |
| TCGA-04-1348 | 10 | 105104744 | 105648925 | 0.682651825 | 3 | amp |
| TCGA-04-1348 | 10 | 105651828 | 105792080 | 1.30428172 | 5 | amp |
| TCGA-04-1348 | 10 | 105792381 | 105824394 | 0.674804015 | 3 | amp |
| TCGA-04-1348 | 10 | 105830153 | 105891285 | 1.168688115 | 5 | amp |
| TCGA-04-1348 | 10 | 105891853 | 105967622 | 1.923000234 | 6 | amp |
| TCGA-04-1348 | 10 | 105971719 | 106124688 | 0.969240522 | 4 | amp |
| TCGA-04-1348 | 10 | 106125518 | 106918760 | 1.993146494 | 6 | amp |
| TCGA-04-1348 | 10 | 106924038 | 112054085 | 1.395100505 | 5 | amp |
| TCGA-04-1348 | 10 | 112054942 | 112342419 | 0.787052216 | 3 | amp |
| TCGA-04-1348 | 10 | 112343103 | 112356351 | 1.315159286 | 5 | amp |
| TCGA-04-1348 | 10 | 112357852 | 112364084 | 1.890276874 | 6 | amp |
| TCGA-04-1348 | 10 | 112635723 | 114154887 | 1.323143484 | 5 | amp |
| TCGA-04-1348 | 10 | 114158622 | 115618482 | 1.008942371 | 4 | amp |
| TCGA-04-1348 | 10 | 115636271 | 115887444 | 1.466603736 | 5 | amp |
| TCGA-04-1348 | 10 | 115889629 | 115923071 | 1.902142102 | 6 | amp |
| TCGA-04-1348 | 10 | 115947542 | 115970732 | 1.308789801 | 5 | amp |
| TCGA-04-1348 | 10 | 115971589 | 115987064 | 1.981459926 | 6 | amp |
| TCGA-04-1348 | 10 | 115987631 | 116021059 | 1.254118656 | 5 | amp |
| TCGA-04-1348 | 10 | 116032445 | 116201572 | 0.678786149 | 3 | amp |

| | | | | | | |
|---|---|---|---|---|---|---|
| TCGA-04-1348 | 10 | 116203750 | 116603717 | 1.277274978 | 5 | amp |
| TCGA-04-1348 | 10 | 116605092 | 118204079 | 2.016444882 | 6 | amp |
| TCGA-04-1348 | 10 | 118215197 | 118357738 | 1.520414176 | 5 | amp |
| TCGA-04-1348 | 10 | 118359467 | 118464845 | 0.554811942 | 3 | amp |
| TCGA-04-1348 | 10 | 118466673 | 120833473 | 0.960539491 | 4 | amp |
| TCGA-04-1348 | 10 | 120867457 | 121587317 | 0.751021621 | 3 | amp |
| TCGA-04-1348 | 10 | 121590898 | 122661861 | 1.395584651 | 5 | amp |
| TCGA-04-1348 | 10 | 122662535 | 129881180 | 0.932513649 | 4 | amp |
| TCGA-04-1348 | 10 | 129897426 | 129914331 | 1.644619462 | 6 | amp |
| TCGA-04-1348 | 10 | 129914717 | 133754201 | 0.743769877 | 3 | amp |
| TCGA-04-1348 | 11 | 2970431 | 3707433 | 0.685949781 | 3 | amp |
| TCGA-04-1348 | 11 | 3712527 | 5618195 | 1.285533758 | 5 | amp |
| TCGA-04-1348 | 11 | 5624390 | 5730880 | 0.731431835 | 4 | amp |
| TCGA-04-1348 | 11 | 5757663 | 6221474 | 1.345720517 | 5 | amp |
| TCGA-04-1348 | 11 | 6789232 | 7531443 | 1.233305707 | 4 | amp |
| TCGA-04-1348 | 11 | 7570678 | 7713239 | 0.619864715 | 3 | amp |
| TCGA-04-1348 | 11 | 7716353 | 7723909 | 1.243554054 | 4 | amp |
| TCGA-04-1348 | 11 | 7725230 | 7950216 | 1.951116652 | 6 | amp |
| TCGA-04-1348 | 11 | 7960114 | 8462322 | 0.660452848 | 3 | amp |
| TCGA-04-1348 | 11 | 8474350 | 8496459 | 1.89495455 | 6 | amp |
| TCGA-04-1348 | 11 | 8704271 | 8977850 | 0.631405611 | 3 | amp |
| TCGA-04-1348 | 11 | 8983624 | 9009195 | 1.016802905 | 4 | amp |
| TCGA-04-1348 | 11 | 9009626 | 9438710 | 1.384936556 | 5 | amp |
| TCGA-04-1348 | 11 | 9441944 | 9530399 | 2.068369833 | 6 | amp |
| TCGA-04-1348 | 11 | 9533948 | 9838598 | 1.33022467 | 5 | amp |
| TCGA-04-1348 | 11 | 9850822 | 10215551 | 1.902141983 | 6 | amp |
| TCGA-04-1348 | 11 | 10327206 | 11354429 | 0.798843429 | 3 | amp |
| TCGA-04-1348 | 11 | 11362308 | 11643142 | 1.18093211 | 4 | amp |
| TCGA-04-1348 | 11 | 11906010 | 11977754 | 1.745975151 | 6 | amp |
| TCGA-04-1348 | 11 | 11985971 | 12284106 | 0.734366986 | 3 | amp |
| TCGA-04-1348 | 11 | 12313582 | 12946648 | 1.06665903 | 4 | amp |
| TCGA-04-1348 | 11 | 12951676 | 13743461 | 1.647688372 | 6 | amp |
| TCGA-04-1348 | 11 | 13749046 | 16810858 | 1.22851099 | 5 | amp |
| TCGA-04-1348 | 11 | 16811313 | 17167509 | 0.943363549 | 4 | amp |
| TCGA-04-1348 | 11 | 17169059 | 17333680 | 1.71962153 | 6 | amp |
| TCGA-04-1348 | 11 | 17899712 | 18634067 | 1.01366372 | 4 | amp |
| TCGA-04-1348 | 11 | 18636067 | 19167908 | 0.637566704 | 3 | amp |
| TCGA-04-1348 | 11 | 19169121 | 19251891 | 1.570888981 | 5 | amp |
| TCGA-04-1348 | 11 | 19252159 | 20005851 | 0.67365826 | 3 | amp |
| TCGA-04-1348 | 11 | 20057423 | 20424557 | 0.979243609 | 4 | amp |
| TCGA-04-1348 | 11 | 20429397 | 20515816 | 2.135755499 | 6 | amp |
| TCGA-04-1348 | 11 | 20529844 | 20869333 | 0.995743105 | 4 | amp |
| TCGA-04-1348 | 11 | 20906947 | 22277112 | 1.706643049 | 6 | amp |
| TCGA-04-1348 | 11 | 22279202 | 26663629 | 1.390802277 | 5 | amp |
| TCGA-04-1348 | 11 | 26664684 | 28119203 | 1.650613164 | 6 | amp |
| TCGA-04-1348 | 11 | 28119282 | 30902899 | 1.129831108 | 5 | amp |
| TCGA-04-1348 | 11 | 30914334 | 31815103 | 1.785556722 | 6 | amp |
| TCGA-04-1348 | 11 | 31815154 | 32622426 | 0.71958933 | 3 | amp |
| TCGA-04-1348 | 11 | 32623277 | 33182917 | 1.205725894 | 5 | amp |
| TCGA-04-1348 | 11 | 33308028 | 33369019 | 2.161962281 | 6 | amp |

| | | | | | | |
|---|---|---|---|---|---|---|
| TCGA-04-1348 | 11 | 33369116 | 33640263 | 1.35858515 | 5 | amp |
| TCGA-04-1348 | 11 | 33667226 | 34149197 | 1.031124534 | 4 | amp |
| TCGA-04-1348 | 11 | 34152271 | 34194897 | 0.60919138 | 3 | amp |
| TCGA-04-1348 | 11 | 34218828 | 34533163 | 0.944920407 | 4 | amp |
| TCGA-04-1348 | 11 | 34664135 | 43861641 | 1.359088462 | 5 | amp |
| TCGA-04-1348 | 11 | 43876189 | 44129830 | 0.943255497 | 4 | amp |
| TCGA-04-1348 | 11 | 44130698 | 44265887 | 1.312817339 | 5 | amp |
| TCGA-04-1348 | 11 | 45970376 | 45992972 | 1.198412093 | 4 | amp |
| TCGA-04-1348 | 11 | 46001214 | 46105803 | 1.855176978 | 6 | amp |
| TCGA-04-1348 | 11 | 46454980 | 46686513 | 1.202222946 | 4 | amp |
| TCGA-04-1348 | 11 | 46686880 | 46766171 | 0.539585215 | 3 | amp |
| TCGA-04-1348 | 11 | 46771783 | 46829697 | 1.748919601 | 6 | amp |
| TCGA-04-1348 | 11 | 46830939 | 46903473 | 1.095054431 | 5 | amp |
| TCGA-04-1348 | 11 | 47493695 | 47820027 | 0.938072896 | 4 | amp |
| TCGA-04-1348 | 11 | 47823302 | 48166733 | 1.382388797 | 5 | amp |
| TCGA-04-1348 | 11 | 48168411 | 48188954 | 1.952584428 | 6 | amp |
| TCGA-04-1348 | 11 | 48238346 | 50252738 | 1.390081227 | 5 | amp |
| TCGA-04-1348 | 11 | 51411441 | 55607260 | 1.751543999 | 6 | amp |
| TCGA-04-1348 | 11 | 55648292 | 56786270 | 1.446078316 | 5 | amp |
| TCGA-04-1348 | 11 | 56949312 | 57561577 | 0.608688156 | 3 | amp |
| TCGA-04-1348 | 11 | 57563004 | 58352402 | 1.305315269 | 5 | amp |
| TCGA-04-1348 | 11 | 58377287 | 58384298 | 2.319812262 | 6 | amp |
| TCGA-04-1348 | 11 | 58384641 | 58723531 | 1.272577081 | 5 | amp |
| TCGA-04-1348 | 11 | 58877078 | 59578163 | 0.966437573 | 4 | amp |
| TCGA-04-1348 | 11 | 59596927 | 60541000 | 1.307140374 | 5 | amp |
| TCGA-04-1348 | 11 | 61959501 | 62012216 | 1.579094873 | 5 | amp |
| TCGA-04-1348 | 11 | 62931991 | 63177393 | 1.796676089 | 6 | amp |
| TCGA-04-1348 | 11 | 63230930 | 63330851 | 0.63183956 | 3 | amp |
| TCGA-04-1348 | 11 | 63342377 | 63398967 | 1.020543677 | 4 | amp |
| TCGA-04-1348 | 11 | 63400473 | 63521216 | 1.261643448 | 5 | amp |
| TCGA-04-1348 | 11 | 67817892 | 68030409 | 1.161752905 | 4 | amp |
| TCGA-04-1348 | 11 | 68115242 | 68525214 | 0.780217824 | 3 | amp |
| TCGA-04-1348 | 11 | 71316394 | 73718144 | 0.662170552 | 3 | amp |
| TCGA-04-1348 | 11 | 73745587 | 73834226 | 1.935028283 | 6 | amp |
| TCGA-04-1348 | 11 | 73843832 | 74351829 | 1.282318922 | 5 | amp |
| TCGA-04-1348 | 11 | 74407526 | 74862494 | 0.968533949 | 4 | amp |
| TCGA-04-1348 | 11 | 74873614 | 75495846 | 0.59698953 | 3 | amp |
| TCGA-04-1348 | 11 | 75501196 | 76207565 | 1.138594062 | 4 | amp |
| TCGA-04-1348 | 11 | 76224384 | 76261212 | 1.826104157 | 6 | amp |
| TCGA-04-1348 | 11 | 76370639 | 76731404 | 0.944704185 | 3 | amp |
| TCGA-04-1348 | 11 | 76928286 | 77934769 | 1.128006984 | 4 | amp |
| TCGA-04-1348 | 11 | 77936110 | 83183854 | 1.327882971 | 5 | amp |
| TCGA-04-1348 | 11 | 83191622 | 85309780 | 1.801701648 | 6 | amp |
| TCGA-04-1348 | 11 | 85342735 | 87006904 | 1.271488362 | 5 | amp |
| TCGA-04-1348 | 11 | 87013290 | 89378263 | 1.647763898 | 6 | amp |
| TCGA-04-1348 | 11 | 89385654 | 95551128 | 1.203931348 | 5 | amp |
| TCGA-04-1348 | 11 | 95551939 | 95595542 | 1.86346851 | 6 | amp |
| TCGA-04-1348 | 11 | 95598712 | 102713704 | 1.373470636 | 5 | amp |
| TCGA-04-1348 | 11 | 102714134 | 107422645 | 1.67209373 | 6 | amp |
| TCGA-04-1348 | 11 | 107423821 | 107682576 | 1.200304964 | 5 | amp |

| | | | | | | |
|---|---|---|---|---|---|---|
| TCGA-04-1348 | 11 | 107686483 | 108218108 | 1.015667389 | 4 | amp |
| TCGA-04-1348 | 11 | 108224489 | 116827841 | 0.63646522 | 3 | amp |
| TCGA-04-1348 | 11 | 118067402 | 118267211 | 0.810338744 | 3 | amp |
| TCGA-04-1348 | 11 | 120201109 | 124564390 | 0.754229584 | 3 | amp |
| TCGA-04-1348 | 12 | 347073 | 551132 | 1.594314115 | 6 | amp |
| TCGA-04-1348 | 12 | 644293 | 923013 | 0.641181296 | 3 | amp |
| TCGA-04-1348 | 12 | 936167 | 1895293 | 1.145618669 | 4 | amp |
| TCGA-04-1348 | 12 | 1902812 | 4553410 | 0.643954601 | 3 | amp |
| TCGA-04-1348 | 12 | 4554353 | 4855426 | 1.304596957 | 5 | amp |
| TCGA-04-1348 | 12 | 4870066 | 5848583 | 1.04903115 | 4 | amp |
| TCGA-04-1348 | 12 | 5853282 | 6220142 | 0.76791532 | 3 | amp |
| TCGA-04-1348 | 12 | 7086215 | 7355313 | 0.753940733 | 3 | amp |
| TCGA-04-1348 | 12 | 7356039 | 7596767 | 1.339653205 | 5 | amp |
| TCGA-04-1348 | 12 | 7632408 | 7656323 | 1.913594568 | 6 | amp |
| TCGA-04-1348 | 12 | 7802097 | 8391364 | 0.732094012 | 3 | amp |
| TCGA-04-1348 | 12 | 8519384 | 9353992 | 1.046074011 | 4 | amp |
| TCGA-04-1348 | 12 | 9354850 | 9659785 | 0.554302858 | 3 | amp |
| TCGA-04-1348 | 12 | 9660025 | 11286858 | 1.323324917 | 5 | amp |
| TCGA-04-1348 | 12 | 11338590 | 11546904 | 1.979975088 | 6 | amp |
| TCGA-04-1348 | 12 | 11547386 | 18658430 | 1.337193279 | 5 | amp |
| TCGA-04-1348 | 12 | 18691047 | 19422821 | 1.722770345 | 6 | amp |
| TCGA-04-1348 | 12 | 19427443 | 20903787 | 1.359331234 | 5 | amp |
| TCGA-04-1348 | 12 | 20905200 | 21644701 | 1.62152608 | 6 | amp |
| TCGA-04-1348 | 12 | 21652436 | 21967676 | 1.199027974 | 5 | amp |
| TCGA-04-1348 | 12 | 21968641 | 26276148 | 1.615260538 | 6 | amp |
| TCGA-04-1348 | 12 | 26383586 | 27117717 | 1.610045357 | 6 | amp |
| TCGA-04-1348 | 12 | 27126818 | 27529359 | 1.187945791 | 5 | amp |
| TCGA-04-1348 | 12 | 27533108 | 29630560 | 1.673615838 | 6 | amp |
| TCGA-04-1348 | 12 | 29631744 | 30894056 | 1.376644795 | 5 | amp |
| TCGA-04-1348 | 12 | 30903975 | 31286136 | 0.549760627 | 3 | amp |
| TCGA-04-1348 | 12 | 31286811 | 31291244 | 0.960322687 | 4 | amp |
| TCGA-04-1348 | 12 | 31291971 | 38715028 | 1.268894259 | 5 | amp |
| TCGA-04-1348 | 12 | 39047627 | 41961762 | 1.618357086 | 6 | amp |
| TCGA-04-1348 | 12 | 41966148 | 45568178 | 1.285830686 | 5 | amp |
| TCGA-04-1348 | 12 | 45610079 | 48081841 | 1.735568024 | 6 | amp |
| TCGA-04-1348 | 12 | 48393684 | 48537626 | 1.163907137 | 4 | amp |
| TCGA-04-1348 | 12 | 48537794 | 49168362 | 0.834134897 | 3 | amp |
| TCGA-04-1348 | 12 | 50027651 | 50503313 | 0.704151846 | 3 | amp |
| TCGA-04-1348 | 12 | 50513793 | 51069257 | 1.39157375 | 5 | amp |
| TCGA-04-1348 | 12 | 51072440 | 51098022 | 1.950832052 | 6 | amp |
| TCGA-04-1348 | 12 | 51100264 | 51445992 | 1.354409635 | 5 | amp |
| TCGA-04-1348 | 12 | 51447541 | 51566264 | 1.009521832 | 4 | amp |
| TCGA-04-1348 | 12 | 51584022 | 51852448 | 0.687025008 | 3 | amp |
| TCGA-04-1348 | 12 | 51853693 | 52188469 | 1.178322285 | 4 | amp |
| TCGA-04-1348 | 12 | 54812911 | 54932772 | 1.638097846 | 6 | amp |
| TCGA-04-1348 | 12 | 54936323 | 54976607 | 0.678336036 | 3 | amp |
| TCGA-04-1348 | 12 | 54976953 | 55026214 | 1.053971345 | 4 | amp |
| TCGA-04-1348 | 12 | 55026900 | 55040983 | 1.236638133 | 5 | amp |
| TCGA-04-1348 | 12 | 55041998 | 55759843 | 1.599571682 | 6 | amp |
| TCGA-04-1348 | 12 | 56744867 | 57490943 | 0.778812013 | 3 | amp |

| | | | | | | |
|---|---|---|---|---|---|---|
| TCGA-04-1348 | 12 | 58189922 | 58340878 | 0.811284914 | 3 | amp |
| TCGA-04-1348 | 12 | 58345489 | 60098810 | 1.23733688 | 5 | amp |
| TCGA-04-1348 | 12 | 60164951 | 64895195 | 1.558698526 | 6 | amp |
| TCGA-04-1348 | 12 | 65016179 | 78531170 | 1.347297512 | 5 | amp |
| TCGA-04-1348 | 12 | 78534027 | 80771002 | 1.722756079 | 6 | amp |
| TCGA-04-1348 | 12 | 80771607 | 81738517 | 1.292849226 | 5 | amp |
| TCGA-04-1348 | 12 | 81741310 | 85446051 | 1.592529696 | 6 | amp |
| TCGA-04-1348 | 12 | 85449296 | 88547296 | 1.372734859 | 5 | amp |
| TCGA-04-1348 | 12 | 88548054 | 93210195 | 1.566182406 | 6 | amp |
| TCGA-04-1348 | 12 | 93213065 | 96292262 | 1.321886545 | 5 | amp |
| TCGA-04-1348 | 12 | 96292307 | 96394919 | 0.773046531 | 4 | amp |
| TCGA-04-1348 | 12 | 96396729 | 101761815 | 1.261361554 | 5 | amp |
| TCGA-04-1348 | 12 | 101763463 | 102074333 | 0.964809352 | 4 | amp |
| TCGA-04-1348 | 12 | 102076320 | 104014348 | 1.371138872 | 5 | amp |
| TCGA-04-1348 | 12 | 104015794 | 104111643 | 0.908501789 | 4 | amp |
| TCGA-04-1348 | 12 | 104118662 | 104374765 | 1.178205021 | 5 | amp |
| TCGA-04-1348 | 12 | 104376554 | 104520076 | 1.799227759 | 6 | amp |
| TCGA-04-1348 | 12 | 104522188 | 108004169 | 1.255119358 | 5 | amp |
| TCGA-04-1348 | 12 | 108006541 | 108590040 | 1.078236861 | 4 | amp |
| TCGA-04-1348 | 12 | 108600062 | 110376398 | 0.684955777 | 3 | amp |
| TCGA-04-1348 | 12 | 110376920 | 110778811 | 1.111957931 | 4 | amp |
| TCGA-04-1348 | 12 | 110780022 | 112247422 | 0.764195704 | 3 | amp |
| TCGA-04-1348 | 12 | 112303030 | 113357375 | 1.095962141 | 4 | amp |
| TCGA-04-1348 | 12 | 113376303 | 113448293 | 0.795045482 | 3 | amp |
| TCGA-04-1348 | 12 | 116401177 | 116586396 | 1.508489916 | 5 | amp |
| TCGA-04-1348 | 12 | 116675211 | 120307054 | 0.870945494 | 3 | amp |
| TCGA-04-1348 | 12 | 122864866 | 123055510 | 1.568982876 | 6 | amp |
| TCGA-04-1348 | 12 | 123055562 | 123099645 | 1.197468382 | 5 | amp |
| TCGA-04-1348 | 12 | 123102856 | 123519158 | 0.608976318 | 3 | amp |
| TCGA-04-1348 | 12 | 123641314 | 123835042 | 1.173899678 | 4 | amp |
| TCGA-04-1348 | 12 | 123875104 | 124363917 | 0.769871593 | 3 | amp |
| TCGA-04-1348 | 12 | 125811135 | 130892382 | 0.829767106 | 3 | amp |
| TCGA-04-1348 | 12 | 133381274 | 133779395 | 0.847857973 | 3 | amp |
| TCGA-04-1348 | 13 | 19240876 | 19982127 | 0.922250545 | 3 | amp |
| TCGA-04-1348 | 13 | 19997192 | 20048217 | 1.663648051 | 6 | amp |
| TCGA-04-1348 | 13 | 20049677 | 20426363 | 0.935307055 | 4 | amp |
| TCGA-04-1348 | 13 | 20567155 | 21442847 | 1.256543909 | 5 | amp |
| TCGA-04-1348 | 13 | 21535765 | 24449076 | 1.089743887 | 4 | amp |
| TCGA-04-1348 | 13 | 24453359 | 25341516 | 0.780707725 | 3 | amp |
| TCGA-04-1348 | 13 | 25348906 | 25439193 | 1.54260824 | 5 | amp |
| TCGA-04-1348 | 13 | 25440251 | 25672274 | 0.785535371 | 4 | amp |
| TCGA-04-1348 | 13 | 25743700 | 27241850 | 1.378938335 | 5 | amp |
| TCGA-04-1348 | 13 | 27245947 | 28610185 | 0.982087181 | 4 | amp |
| TCGA-04-1348 | 13 | 28611313 | 28647587 | 1.330686567 | 5 | amp |
| TCGA-04-1348 | 13 | 28748413 | 28855543 | 1.840017127 | 6 | amp |
| TCGA-04-1348 | 13 | 28862039 | 28880921 | 1.150434593 | 5 | amp |
| TCGA-04-1348 | 13 | 28882961 | 30784635 | 1.016043837 | 4 | amp |
| TCGA-04-1348 | 13 | 30801465 | 32057794 | 1.212954118 | 5 | amp |
| TCGA-04-1348 | 13 | 32313736 | 32699031 | 1.74959604 | 6 | amp |
| TCGA-04-1348 | 13 | 32705742 | 32900322 | 1.211979269 | 5 | amp |

| | | | | | | |
|---|---|---|---|---|---|---|
| TCGA-04-1348 | 13 | 32900338 | 33692420 | 1.738604348 | 6 | amp |
| TCGA-04-1348 | 13 | 33700147 | 34395457 | 1.082416482 | 5 | amp |
| TCGA-04-1348 | 13 | 34398027 | 36006587 | 2.003877474 | 6 | amp |
| TCGA-04-1348 | 13 | 36026180 | 41900030 | 1.25360743 | 5 | amp |
| TCGA-04-1348 | 13 | 41902799 | 41949759 | 2.218510211 | 6 | amp |
| TCGA-04-1348 | 13 | 41959074 | 42761294 | 1.385915038 | 5 | amp |
| TCGA-04-1348 | 13 | 42763128 | 43474542 | 1.790566533 | 6 | amp |
| TCGA-04-1348 | 13 | 43491628 | 43900667 | 1.329676097 | 5 | amp |
| TCGA-04-1348 | 13 | 43918659 | 45857756 | 1.059708656 | 4 | amp |
| TCGA-04-1348 | 13 | 45911461 | 46358048 | 0.87869751 | 3 | amp |
| TCGA-04-1348 | 13 | 46358121 | 46425816 | 0.934544909 | 4 | amp |
| TCGA-04-1348 | 13 | 46537916 | 46549542 | 2.085214944 | 6 | amp |
| TCGA-04-1348 | 13 | 46549857 | 48922039 | 1.356933287 | 5 | amp |
| TCGA-04-1348 | 13 | 48923065 | 48986610 | 2.438094256 | 6 | amp |
| TCGA-04-1348 | 13 | 49027067 | 51921385 | 1.243242637 | 5 | amp |
| TCGA-04-1348 | 13 | 51922327 | 52004534 | 1.899362916 | 6 | amp |
| TCGA-04-1348 | 13 | 52025115 | 52249402 | 1.20265988 | 5 | amp |
| TCGA-04-1348 | 13 | 52277698 | 53419121 | 0.843401972 | 3 | amp |
| TCGA-04-1348 | 13 | 53602911 | 57747822 | 1.103944494 | 4 | amp |
| TCGA-04-1348 | 13 | 58206673 | 58207868 | 1.346015346 | 5 | amp |
| TCGA-04-1348 | 13 | 58240570 | 60584854 | 1.690714771 | 6 | amp |
| TCGA-04-1348 | 13 | 60590021 | 72049366 | 1.43206816 | 5 | amp |
| TCGA-04-1348 | 13 | 72049789 | 73573166 | 1.614241899 | 6 | amp |
| TCGA-04-1348 | 13 | 73589971 | 76141465 | 1.249968744 | 5 | amp |
| TCGA-04-1348 | 13 | 76143529 | 77720384 | 1.679422159 | 6 | amp |
| TCGA-04-1348 | 13 | 77724856 | 77743858 | 1.088017928 | 5 | amp |
| TCGA-04-1348 | 13 | 77745547 | 78192226 | 1.683963575 | 6 | amp |
| TCGA-04-1348 | 13 | 78202052 | 79233279 | 1.378782563 | 5 | amp |
| TCGA-04-1348 | 13 | 79766127 | 80125263 | 1.699958242 | 6 | amp |
| TCGA-04-1348 | 13 | 80910886 | 96410093 | 1.309508453 | 5 | amp |
| TCGA-04-1348 | 13 | 96412234 | 96519694 | 1.802876785 | 6 | amp |
| TCGA-04-1348 | 13 | 96529485 | 96624939 | 1.314672306 | 5 | amp |
| TCGA-04-1348 | 13 | 96635775 | 96651572 | 2.169686364 | 6 | amp |
| TCGA-04-1348 | 13 | 96665527 | 98829492 | 1.265872237 | 5 | amp |
| TCGA-04-1348 | 13 | 98865431 | 99337193 | 0.803256821 | 3 | amp |
| TCGA-04-1348 | 13 | 99338407 | 99537405 | 1.121997503 | 4 | amp |
| TCGA-04-1348 | 13 | 99537903 | 102105293 | 1.218205406 | 5 | amp |
| TCGA-04-1348 | 13 | 102220002 | 102250695 | 2.241172778 | 6 | amp |
| TCGA-04-1348 | 13 | 102344872 | 109717994 | 1.200908971 | 5 | amp |
| TCGA-04-1348 | 13 | 109753123 | 110862577 | 0.836780296 | 3 | amp |
| TCGA-04-1348 | 13 | 115010314 | 115091796 | 1.070042261 | 4 | amp |
| TCGA-04-1348 | 14 | 19377543 | 20768991 | 1.090295 | 4 | amp |
| TCGA-04-1348 | 14 | 20769937 | 20839839 | 0.611095347 | 3 | amp |
| TCGA-04-1348 | 14 | 21024529 | 21561807 | 0.687108007 | 3 | amp |
| TCGA-04-1348 | 14 | 21623039 | 21813373 | 1.048201527 | 4 | amp |
| TCGA-04-1348 | 14 | 21816275 | 21894403 | 1.387597915 | 5 | amp |
| TCGA-04-1348 | 14 | 21895980 | 22038884 | 0.904178048 | 4 | amp |
| TCGA-04-1348 | 14 | 22070029 | 22280183 | 1.261585914 | 5 | amp |
| TCGA-04-1348 | 14 | 22293637 | 22322714 | 1.90269863 | 6 | amp |
| TCGA-04-1348 | 14 | 22337006 | 22918170 | 1.397676483 | 5 | amp |

| | | | | | | |
|---|---|---|---|---|---|---|
| TCGA-04-1348 | 14 | 22919045 | 22971305 | 0.92423702 | 4 | amp |
| TCGA-04-1348 | 14 | 22972705 | 23282657 | 0.806776408 | 3 | amp |
| TCGA-04-1348 | 14 | 23355216 | 23415945 | 0.937273379 | 3 | amp |
| TCGA-04-1348 | 14 | 23898932 | 23947285 | 0.96919729 | 3 | amp |
| TCGA-04-1348 | 14 | 25075730 | 25101717 | 1.11468933 | 5 | amp |
| TCGA-04-1348 | 14 | 25102074 | 26941621 | 1.847776711 | 6 | amp |
| TCGA-04-1348 | 14 | 26949145 | 30068341 | 1.249324413 | 5 | amp |
| TCGA-04-1348 | 14 | 30068822 | 30100267 | 2.047353082 | 6 | amp |
| TCGA-04-1348 | 14 | 30102053 | 31107511 | 1.366138211 | 5 | amp |
| TCGA-04-1348 | 14 | 31108966 | 31144290 | 1.812399843 | 6 | amp |
| TCGA-04-1348 | 14 | 31163966 | 31374813 | 1.106361357 | 5 | amp |
| TCGA-04-1348 | 14 | 31376046 | 31405885 | 1.976635927 | 6 | amp |
| TCGA-04-1348 | 14 | 31416262 | 31572316 | 1.355300219 | 5 | amp |
| TCGA-04-1348 | 14 | 31574575 | 31771732 | 1.682057225 | 6 | amp |
| TCGA-04-1348 | 14 | 31774066 | 34243759 | 1.484693629 | 5 | amp |
| TCGA-04-1348 | 14 | 34247664 | 35182940 | 0.85457098 | 4 | amp |
| TCGA-04-1348 | 14 | 35222691 | 36125126 | 1.262214948 | 5 | amp |
| TCGA-04-1348 | 14 | 36128312 | 36244955 | 1.756116063 | 6 | amp |
| TCGA-04-1348 | 14 | 36289311 | 39562489 | 1.412922875 | 5 | amp |
| TCGA-04-1348 | 14 | 39565091 | 39763299 | 1.038949662 | 4 | amp |
| TCGA-04-1348 | 14 | 39764090 | 39819504 | 2.159798105 | 6 | amp |
| TCGA-04-1348 | 14 | 39868675 | 44589498 | 1.394086303 | 5 | amp |
| TCGA-04-1348 | 14 | 44973691 | 47530796 | 0.934545144 | 4 | amp |
| TCGA-04-1348 | 14 | 47566011 | 50596759 | 0.761993653 | 3 | amp |
| TCGA-04-1348 | 14 | 50597169 | 51225379 | 0.98549207 | 4 | amp |
| TCGA-04-1348 | 14 | 51226495 | 52937433 | 0.719447681 | 3 | amp |
| TCGA-04-1348 | 14 | 52948887 | 64619477 | 0.942949811 | 4 | amp |
| TCGA-04-1348 | 14 | 64625307 | 65067059 | 0.642881484 | 3 | amp |
| TCGA-04-1348 | 14 | 65470938 | 71445411 | 0.695548644 | 3 | amp |
| TCGA-04-1348 | 14 | 71455229 | 72176399 | 1.104638577 | 4 | amp |
| TCGA-04-1348 | 14 | 72190315 | 73007881 | 0.712076462 | 3 | amp |
| TCGA-04-1348 | 14 | 74347891 | 74726514 | 0.693638793 | 3 | amp |
| TCGA-04-1348 | 14 | 75136298 | 76091144 | 0.684932423 | 3 | amp |
| TCGA-04-1348 | 14 | 76099945 | 76249921 | 1.029775026 | 4 | amp |
| TCGA-04-1348 | 14 | 76259200 | 76668233 | 0.746561441 | 3 | amp |
| TCGA-04-1348 | 14 | 77808102 | 78748544 | 0.692469732 | 3 | amp |
| TCGA-04-1348 | 14 | 78993050 | 90458379 | 0.977104184 | 4 | amp |
| TCGA-04-1348 | 14 | 90459713 | 92071074 | 0.675351583 | 3 | amp |
| TCGA-04-1348 | 14 | 92074602 | 92909840 | 0.945191948 | 4 | amp |
| TCGA-04-1348 | 14 | 92911613 | 93814479 | 0.602890282 | 3 | amp |
| TCGA-04-1348 | 14 | 93943970 | 94171051 | 0.987937563 | 4 | amp |
| TCGA-04-1348 | 14 | 94172999 | 96769614 | 0.675649455 | 3 | amp |
| TCGA-04-1348 | 14 | 96770825 | 99876591 | 1.02417597 | 4 | amp |
| TCGA-04-1348 | 14 | 99879254 | 99932150 | 1.721968239 | 6 | amp |
| TCGA-04-1348 | 14 | 101391132 | 101456521 | 2.070257557 | 6 | amp |
| TCGA-04-1348 | 14 | 101458230 | 103188758 | 0.633160932 | 3 | amp |
| TCGA-04-1348 | 14 | 104219314 | 104462175 | 1.004363461 | 5 | amp |
| TCGA-04-1348 | 14 | 104464952 | 104492488 | 1.745763298 | 6 | amp |
| TCGA-04-1348 | 14 | 106518370 | 106573717 | 1.275005351 | 5 | amp |
| TCGA-04-1348 | 14 | 106578972 | 107283263 | 0.869665975 | 4 | amp |

| | | | | | | |
|---|---|---|---|---|---|---|
| TCGA-04-1348 | 15 | 20867277 | 22414056 | 1.223372949 | 5 | amp |
| TCGA-04-1348 | 15 | 23810911 | 25351768 | 1.196112054 | 5 | amp |
| TCGA-04-1348 | 15 | 25514907 | 28171456 | 0.92425697 | 4 | amp |
| TCGA-04-1348 | 15 | 28857202 | 32915800 | 0.823020845 | 4 | amp |
| TCGA-04-1348 | 15 | 32916320 | 33945121 | 1.453837906 | 5 | amp |
| TCGA-04-1348 | 15 | 33951903 | 34628925 | 1.010509533 | 4 | amp |
| TCGA-04-1348 | 15 | 35085427 | 40318293 | 1.211212604 | 5 | amp |
| TCGA-04-1348 | 15 | 40321575 | 40512982 | 0.941994065 | 4 | amp |
| TCGA-04-1348 | 15 | 40863770 | 40949675 | 1.286062335 | 5 | amp |
| TCGA-04-1348 | 15 | 41289702 | 41657813 | 1.233351999 | 5 | amp |
| TCGA-04-1348 | 15 | 41961084 | 42059499 | 1.321117187 | 5 | amp |
| TCGA-04-1348 | 15 | 42455784 | 42823703 | 1.260418707 | 5 | amp |
| TCGA-04-1348 | 15 | 43027425 | 43452995 | 1.249716998 | 5 | amp |
| TCGA-04-1348 | 15 | 43668641 | 43696058 | 1.163711375 | 5 | amp |
| TCGA-04-1348 | 15 | 43696530 | 43701314 | 2.565486484 | 6 | amp |
| TCGA-04-1348 | 15 | 43701831 | 43974699 | 0.860934336 | 4 | amp |
| TCGA-04-1348 | 15 | 44097232 | 44198207 | 0.892287172 | 4 | amp |
| TCGA-04-1348 | 15 | 44202068 | 45365765 | 1.223268729 | 5 | amp |
| TCGA-04-1348 | 15 | 45456887 | 45779837 | 0.863483492 | 4 | amp |
| TCGA-04-1348 | 15 | 45781019 | 45983259 | 1.32270149 | 5 | amp |
| TCGA-04-1348 | 15 | 48051989 | 48056275 | 2.30768787 | 6 | amp |
| TCGA-04-1348 | 15 | 48056316 | 48413421 | 1.436952475 | 5 | amp |
| TCGA-04-1348 | 15 | 48413993 | 48444515 | 1.964916561 | 6 | amp |
| TCGA-04-1348 | 15 | 48445949 | 50731382 | 1.471389067 | 5 | amp |
| TCGA-04-1348 | 15 | 50733497 | 50822179 | 2.185226845 | 6 | amp |
| TCGA-04-1348 | 15 | 50830976 | 50950056 | 1.535004033 | 5 | amp |
| TCGA-04-1348 | 15 | 50955143 | 51535156 | 2.225463183 | 6 | amp |
| TCGA-04-1348 | 15 | 51669611 | 51749713 | 1.388190059 | 5 | amp |
| TCGA-04-1348 | 15 | 51750643 | 51991647 | 1.988441184 | 6 | amp |
| TCGA-04-1348 | 15 | 51993222 | 52575093 | 1.347509815 | 5 | amp |
| TCGA-04-1348 | 15 | 52605848 | 52725486 | 2.169256263 | 6 | amp |
| TCGA-04-1348 | 15 | 52797724 | 55489107 | 1.416861042 | 5 | amp |
| TCGA-04-1348 | 15 | 55497683 | 55930930 | 0.903154863 | 4 | amp |
| TCGA-04-1348 | 15 | 55931812 | 56676249 | 1.156119546 | 5 | amp |
| TCGA-04-1348 | 15 | 56680633 | 56993428 | 1.995022311 | 6 | amp |
| TCGA-04-1348 | 15 | 56996271 | 62146766 | 1.261529812 | 5 | amp |
| TCGA-04-1348 | 15 | 62147051 | 62176517 | 2.177604635 | 6 | amp |
| TCGA-04-1348 | 15 | 62182361 | 62228992 | 1.228012801 | 5 | amp |
| TCGA-04-1348 | 15 | 62232799 | 62336492 | 2.040791488 | 6 | amp |
| TCGA-04-1348 | 15 | 62939457 | 63354009 | 0.729039904 | 4 | amp |
| TCGA-04-1348 | 15 | 63354384 | 63908162 | 1.238576702 | 5 | amp |
| TCGA-04-1348 | 15 | 63908574 | 64047535 | 2.26784045 | 6 | amp |
| TCGA-04-1348 | 15 | 64048611 | 64915258 | 1.234161265 | 5 | amp |
| TCGA-04-1348 | 15 | 65255868 | 65472581 | 1.156464135 | 5 | amp |
| TCGA-04-1348 | 15 | 65702436 | 66845638 | 1.202501461 | 5 | amp |
| TCGA-04-1348 | 15 | 66850020 | 67501900 | 0.674626701 | 4 | amp |
| TCGA-04-1348 | 15 | 67524133 | 68473740 | 1.42188735 | 5 | amp |
| TCGA-04-1348 | 15 | 70366848 | 70371818 | 1.216926597 | 5 | amp |
| TCGA-04-1348 | 15 | 70949370 | 71125462 | 3.421450235 | 6 | amp |
| TCGA-04-1348 | 15 | 71128618 | 71203963 | 1.016600369 | 5 | amp |

| | | | | | | |
|---|---|---|---|---|---|---|
| TCGA-04-1348 | 15 | 71211340 | 71341969 | 2.442091579 | 6 | amp |
| TCGA-04-1348 | 15 | 71403447 | 72120415 | 1.011614195 | 5 | amp |
| TCGA-04-1348 | 15 | 72122430 | 72196384 | 2.072499389 | 6 | amp |
| TCGA-04-1348 | 15 | 72197246 | 72324941 | 1.247084931 | 5 | amp |
| TCGA-04-1348 | 15 | 72338004 | 73067445 | 0.843756966 | 4 | amp |
| TCGA-04-1348 | 15 | 73408859 | 73884512 | 1.345440651 | 5 | amp |
| TCGA-04-1348 | 15 | 75667989 | 76077945 | 0.781162934 | 4 | amp |
| TCGA-04-1348 | 15 | 76146717 | 76303626 | 1.401965435 | 5 | amp |
| TCGA-04-1348 | 15 | 76426533 | 76634237 | 0.700881901 | 4 | amp |
| TCGA-04-1348 | 15 | 76643543 | 77750853 | 1.329503589 | 5 | amp |
| TCGA-04-1348 | 15 | 78572376 | 81631849 | 0.798070816 | 4 | amp |
| TCGA-04-1348 | 15 | 81633674 | 82456379 | 1.165291576 | 5 | amp |
| TCGA-04-1348 | 15 | 82507006 | 82530874 | 2.10321807 | 6 | amp |
| TCGA-04-1348 | 15 | 82532827 | 82575411 | 0.884211095 | 4 | amp |
| TCGA-04-1348 | 15 | 83360396 | 83781660 | 0.882850706 | 4 | amp |
| TCGA-04-1348 | 15 | 83784585 | 84795403 | 1.531985705 | 5 | amp |
| TCGA-04-1348 | 15 | 85607568 | 88672013 | 1.219182015 | 5 | amp |
| TCGA-04-1348 | 15 | 89790830 | 89828488 | 1.432325279 | 5 | amp |
| TCGA-04-1348 | 15 | 89833417 | 90321610 | 0.770291611 | 4 | amp |
| TCGA-04-1348 | 15 | 90969299 | 91347612 | 1.278212442 | 5 | amp |
| TCGA-04-1348 | 15 | 91522335 | 93015775 | 0.87003146 | 4 | amp |
| TCGA-04-1348 | 15 | 93016122 | 95013676 | 1.326063484 | 5 | amp |
| TCGA-04-1348 | 15 | 95019913 | 101445929 | 0.811970164 | 4 | amp |
| TCGA-04-1348 | 16 | 7102025 | 8998476 | 0.972014038 | 4 | amp |
| TCGA-04-1348 | 16 | 8998995 | 9250611 | 1.266313555 | 5 | amp |
| TCGA-04-1348 | 16 | 9856945 | 10576139 | 2.738942977 | 6 | amp |
| TCGA-04-1348 | 16 | 10626705 | 10861907 | 1.477127017 | 5 | amp |
| TCGA-04-1348 | 16 | 10862842 | 10869000 | 2.392261097 | 6 | amp |
| TCGA-04-1348 | 16 | 10934307 | 11017182 | 1.220060733 | 5 | amp |
| TCGA-04-1348 | 16 | 11051582 | 11114235 | 2.727960428 | 6 | amp |
| TCGA-04-1348 | 16 | 11118667 | 11850279 | 1.221753908 | 5 | amp |
| TCGA-04-1348 | 16 | 11852280 | 11862978 | 2.313416276 | 6 | amp |
| TCGA-04-1348 | 16 | 11864620 | 14693848 | 1.541524126 | 5 | amp |
| TCGA-04-1348 | 16 | 14697982 | 14951557 | 0.921365146 | 4 | amp |
| TCGA-04-1348 | 16 | 14956836 | 14978344 | 1.527240162 | 5 | amp |
| TCGA-04-1348 | 16 | 14980608 | 15696073 | 0.82092184 | 3 | amp |
| TCGA-04-1348 | 16 | 15697989 | 15781419 | 1.410877815 | 5 | amp |
| TCGA-04-1348 | 16 | 15784970 | 16337128 | 0.75580113 | 3 | amp |
| TCGA-04-1348 | 16 | 16338917 | 16375197 | 1.162817432 | 4 | amp |
| TCGA-04-1348 | 16 | 16376867 | 18823479 | 0.826095413 | 3 | amp |
| TCGA-04-1348 | 16 | 18826489 | 18872103 | 2.404718635 | 6 | amp |
| TCGA-04-1348 | 16 | 18874952 | 18908316 | 1.4283929 | 5 | amp |
| TCGA-04-1348 | 16 | 19020405 | 19745204 | 1.008278605 | 4 | amp |
| TCGA-04-1348 | 16 | 19746663 | 20322633 | 1.253663414 | 5 | amp |
| TCGA-04-1348 | 16 | 20325940 | 20331135 | 2.463726418 | 6 | amp |
| TCGA-04-1348 | 16 | 20331569 | 20837272 | 1.36548233 | 5 | amp |
| TCGA-04-1348 | 16 | 20838328 | 20851188 | 2.352095056 | 6 | amp |
| TCGA-04-1348 | 16 | 20851601 | 21273538 | 1.3253412 | 5 | amp |
| TCGA-04-1348 | 16 | 21278846 | 21426462 | 0.886606359 | 4 | amp |
| TCGA-04-1348 | 16 | 21436299 | 21495030 | 1.462482622 | 5 | amp |

| | | | | | | |
|---|---|---|---|---|---|---|
| TCGA-04-1348 | 16 | 21495654 | 21858918 | 1.02403833 | 4 | amp |
| TCGA-04-1348 | 16 | 21868579 | 22126850 | 1.396928477 | 5 | amp |
| TCGA-04-1348 | 16 | 22128073 | 22466968 | 0.581612901 | 3 | amp |
| TCGA-04-1348 | 16 | 22470744 | 23119555 | 1.197755287 | 4 | amp |
| TCGA-04-1348 | 16 | 23197540 | 24046923 | 0.55773719 | 3 | amp |
| TCGA-04-1348 | 16 | 24104069 | 24990366 | 1.023766757 | 4 | amp |
| TCGA-04-1348 | 16 | 25066103 | 28474412 | 0.612082876 | 3 | amp |
| TCGA-04-1348 | 16 | 29105545 | 29391676 | 0.629097072 | 3 | amp |
| TCGA-04-1348 | 16 | 29395343 | 29458354 | 1.192707738 | 4 | amp |
| TCGA-04-1348 | 16 | 29496773 | 29516913 | 0.952958094 | 4 | amp |
| TCGA-04-1348 | 16 | 29538947 | 29577608 | 1.237964287 | 5 | amp |
| TCGA-04-1348 | 16 | 30215124 | 30256894 | 0.88062436 | 4 | amp |
| TCGA-04-1348 | 16 | 30278932 | 30317312 | 1.529223813 | 5 | amp |
| TCGA-04-1348 | 16 | 31895761 | 50809140 | 0.646992993 | 3 | amp |
| TCGA-04-1348 | 16 | 50810078 | 53513161 | 1.058877085 | 4 | amp |
| TCGA-04-1348 | 16 | 53513790 | 56540143 | 0.704501306 | 3 | amp |
| TCGA-04-1348 | 16 | 56782128 | 56892531 | 0.945242364 | 3 | amp |
| TCGA-04-1348 | 16 | 58552600 | 65397177 | 0.728478547 | 3 | amp |
| TCGA-04-1348 | 16 | 69381644 | 70863746 | 0.651523536 | 3 | amp |
| TCGA-04-1348 | 16 | 70866740 | 70998834 | 1.129975662 | 4 | amp |
| TCGA-04-1348 | 16 | 71004362 | 71220850 | 1.514833536 | 5 | amp |
| TCGA-04-1348 | 16 | 71317466 | 71689359 | 0.627066066 | 3 | amp |
| TCGA-04-1348 | 16 | 71690468 | 72058130 | 1.127265399 | 4 | amp |
| TCGA-04-1348 | 16 | 72088493 | 72130934 | 1.610097526 | 6 | amp |
| TCGA-04-1348 | 16 | 72131567 | 72157556 | 0.65061024 | 3 | amp |
| TCGA-04-1348 | 16 | 72158599 | 72184737 | 1.294241646 | 5 | amp |
| TCGA-04-1348 | 16 | 72188053 | 76311662 | 0.719281858 | 3 | amp |
| TCGA-04-1348 | 16 | 76350304 | 76573802 | 1.568045618 | 5 | amp |
| TCGA-04-1348 | 16 | 76587168 | 81097575 | 0.671754695 | 3 | amp |
| TCGA-04-1348 | 16 | 81946111 | 83940707 | 0.748888036 | 3 | amp |
| TCGA-04-1348 | 17 | 2995340 | 3424359 | 0.758782892 | 3 | amp |
| TCGA-04-1348 | 17 | 3925912 | 4200120 | 0.724198945 | 3 | amp |
| TCGA-04-1348 | 17 | 5051836 | 5314125 | 0.738760193 | 3 | amp |
| TCGA-04-1348 | 17 | 6406780 | 6532108 | 0.876946999 | 3 | amp |
| TCGA-04-1348 | 17 | 8347572 | 10231490 | 0.645205612 | 3 | amp |
| TCGA-04-1348 | 17 | 10233651 | 10267865 | 1.151477623 | 4 | amp |
| TCGA-04-1348 | 17 | 10293725 | 10370080 | 0.750556568 | 3 | amp |
| TCGA-04-1348 | 17 | 10395688 | 10451255 | 0.989506553 | 4 | amp |
| TCGA-04-1348 | 17 | 10531892 | 12844466 | 0.770415675 | 3 | amp |
| TCGA-04-1348 | 17 | 15884345 | 16120777 | 0.727453373 | 3 | amp |
| TCGA-04-1348 | 17 | 16537163 | 16705659 | 0.807074929 | 3 | amp |
| TCGA-04-1348 | 17 | 18692646 | 18827341 | 0.818234422 | 3 | amp |
| TCGA-04-1348 | 17 | 19685170 | 20334237 | 0.77362879 | 3 | amp |
| TCGA-04-1348 | 17 | 27977624 | 28644290 | 0.765757954 | 3 | amp |
| TCGA-04-1348 | 17 | 28645819 | 28778916 | 1.118552836 | 4 | amp |
| TCGA-04-1348 | 17 | 28782302 | 29369337 | 0.654203131 | 3 | amp |
| TCGA-04-1348 | 17 | 29372203 | 29647507 | 1.117053585 | 4 | amp |
| TCGA-04-1348 | 17 | 29652813 | 32965263 | 0.725267023 | 3 | amp |
| TCGA-04-1348 | 17 | 33255041 | 33281630 | 1.278394146 | 5 | amp |
| TCGA-04-1348 | 17 | 35343912 | 35512713 | 0.71730007 | 3 | amp |

| | | | | | | |
|---|---|---|---|---|---|---|
| TCGA-04-1348 | 17 | 35518649 | 35646486 | 0.981311758 | 4 | amp |
| TCGA-04-1348 | 17 | 35656170 | 36002357 | 0.703968913 | 3 | amp |
| TCGA-04-1348 | 17 | 36916652 | 36977359 | 1.005294961 | 3 | amp |
| TCGA-04-1348 | 17 | 38445611 | 38818351 | 0.623448173 | 3 | amp |
| TCGA-04-1348 | 17 | 38821243 | 38926413 | 1.091192421 | 4 | amp |
| TCGA-04-1348 | 17 | 41197636 | 41599624 | 0.782204344 | 3 | amp |
| TCGA-04-1348 | 17 | 42931590 | 42937462 | 0.799242796 | 3 | amp |
| TCGA-04-1348 | 17 | 42937795 | 42964070 | 1.099516301 | 4 | amp |
| TCGA-04-1348 | 17 | 44116336 | 44714944 | 0.767216792 | 3 | amp |
| TCGA-04-1348 | 17 | 44717413 | 44833238 | 1.189542871 | 4 | amp |
| TCGA-04-1348 | 17 | 44845625 | 45137555 | 0.759674675 | 3 | amp |
| TCGA-04-1348 | 17 | 45198251 | 45209801 | 1.851087528 | 6 | amp |
| TCGA-04-1348 | 17 | 45214501 | 45745783 | 0.830472239 | 3 | amp |
| TCGA-04-1348 | 17 | 45747019 | 45758074 | 1.266057402 | 5 | amp |
| TCGA-04-1348 | 17 | 46148748 | 46239940 | 0.789554895 | 3 | amp |
| TCGA-04-1348 | 17 | 46247935 | 46474154 | 1.715459336 | 6 | amp |
| TCGA-04-1348 | 17 | 46925600 | 46940403 | 1.500217543 | 5 | amp |
| TCGA-04-1348 | 17 | 47388625 | 47677931 | 0.801212554 | 3 | amp |
| TCGA-04-1348 | 17 | 47679177 | 47700193 | 1.933320871 | 6 | amp |
| TCGA-04-1348 | 17 | 47778785 | 47875980 | 0.620286468 | 3 | amp |
| TCGA-04-1348 | 17 | 47882698 | 47916079 | 1.290734932 | 5 | amp |
| TCGA-04-1348 | 17 | 48777882 | 48828061 | 1.340713957 | 5 | amp |
| TCGA-04-1348 | 17 | 48913230 | 49052339 | 0.599446969 | 3 | amp |
| TCGA-04-1348 | 17 | 49054465 | 49079260 | 1.088370204 | 4 | amp |
| TCGA-04-1348 | 17 | 49082538 | 49157124 | 1.815991025 | 6 | amp |
| TCGA-04-1348 | 17 | 49231701 | 49249026 | 0.525212571 | 3 | amp |
| TCGA-04-1348 | 17 | 49270062 | 49281338 | 1.97410186 | 6 | amp |
| TCGA-04-1348 | 17 | 49284244 | 52990221 | 1.2985071 | 5 | amp |
| TCGA-04-1348 | 17 | 52991051 | 53024707 | 1.923037271 | 6 | amp |
| TCGA-04-1348 | 17 | 53026871 | 53046064 | 1.043100597 | 5 | amp |
| TCGA-04-1348 | 17 | 53063543 | 53218759 | 1.765642394 | 6 | amp |
| TCGA-04-1348 | 17 | 53237154 | 54558223 | 1.347203906 | 5 | amp |
| TCGA-04-1348 | 17 | 54559662 | 55083624 | 0.905104586 | 4 | amp |
| TCGA-04-1348 | 17 | 56634329 | 56642339 | 1.679344907 | 6 | amp |
| TCGA-04-1348 | 17 | 56643056 | 56659151 | 0.595971652 | 3 | amp |
| TCGA-04-1348 | 17 | 56661850 | 56707917 | 1.325211436 | 5 | amp |
| TCGA-04-1348 | 17 | 56729174 | 57725072 | 0.961322557 | 4 | amp |
| TCGA-04-1348 | 17 | 57725561 | 57768094 | 2.017630654 | 6 | amp |
| TCGA-04-1348 | 17 | 57771030 | 57895163 | 1.359512937 | 5 | amp |
| TCGA-04-1348 | 17 | 57915616 | 57992089 | 0.878639569 | 4 | amp |
| TCGA-04-1348 | 17 | 58003749 | 58018310 | 1.795530733 | 6 | amp |
| TCGA-04-1348 | 17 | 58022762 | 58073204 | 1.206668149 | 5 | amp |
| TCGA-04-1348 | 17 | 58073297 | 58236807 | 0.545428308 | 3 | amp |
| TCGA-04-1348 | 17 | 58256582 | 59161936 | 1.233496396 | 5 | amp |
| TCGA-04-1348 | 17 | 59667857 | 59934625 | 1.881583442 | 6 | amp |
| TCGA-04-1348 | 17 | 59937152 | 59989485 | 1.493541251 | 5 | amp |
| TCGA-04-1348 | 17 | 59996757 | 60130101 | 1.044016623 | 4 | amp |
| TCGA-04-1348 | 17 | 60140424 | 60464798 | 0.712989619 | 3 | amp |
| TCGA-04-1348 | 17 | 60469134 | 60683606 | 1.237691875 | 5 | amp |
| TCGA-04-1348 | 17 | 60802231 | 60866020 | 0.806566133 | 3 | amp |

| | | | | | | |
|---|---|---|---|---|---|---|
| TCGA-04-1348 | 17 | 60878991 | 61495777 | 1.308028655 | 5 | amp |
| TCGA-04-1348 | 17 | 61655789 | 61824340 | 0.749311518 | 3 | amp |
| TCGA-04-1348 | 17 | 61829263 | 61897377 | 1.303813191 | 5 | amp |
| TCGA-04-1348 | 17 | 61897425 | 61901790 | 0.895256179 | 4 | amp |
| TCGA-04-1348 | 17 | 62132082 | 62506404 | 0.642872713 | 3 | amp |
| TCGA-04-1348 | 17 | 62512833 | 62594613 | 1.207105258 | 5 | amp |
| TCGA-04-1348 | 17 | 62602678 | 62758099 | 1.79480285 | 6 | amp |
| TCGA-04-1348 | 17 | 62758470 | 63632150 | 0.736395579 | 3 | amp |
| TCGA-04-1348 | 17 | 63633213 | 64023708 | 1.117390129 | 4 | amp |
| TCGA-04-1348 | 17 | 64024416 | 64092752 | 1.758269 | 6 | amp |
| TCGA-04-1348 | 17 | 64125800 | 64637588 | 1.221450021 | 5 | amp |
| TCGA-04-1348 | 17 | 64641414 | 65083250 | 0.560798801 | 3 | amp |
| TCGA-04-1348 | 17 | 65103242 | 65337183 | 1.476291594 | 5 | amp |
| TCGA-04-1348 | 17 | 65338280 | 65972100 | 1.081006256 | 4 | amp |
| TCGA-04-1348 | 17 | 65978325 | 66449181 | 0.699439859 | 3 | amp |
| TCGA-04-1348 | 17 | 66511508 | 66872866 | 1.030195211 | 4 | amp |
| TCGA-04-1348 | 17 | 66873619 | 66925402 | 1.28728759 | 5 | amp |
| TCGA-04-1348 | 17 | 66925622 | 66987157 | 1.859638218 | 6 | amp |
| TCGA-04-1348 | 17 | 66988301 | 67012582 | 1.153753017 | 5 | amp |
| TCGA-04-1348 | 17 | 67013743 | 67179002 | 1.865789801 | 6 | amp |
| TCGA-04-1348 | 17 | 67181550 | 67221542 | 1.050920782 | 5 | amp |
| TCGA-04-1348 | 17 | 67243671 | 67248067 | 2.231492714 | 6 | amp |
| TCGA-04-1348 | 17 | 67249670 | 67252427 | 1.245673872 | 5 | amp |
| TCGA-04-1348 | 17 | 67255832 | 67309454 | 1.8722121 | 6 | amp |
| TCGA-04-1348 | 17 | 67310445 | 68129516 | 1.383395436 | 5 | amp |
| TCGA-04-1348 | 17 | 73647215 | 73667979 | 1.188101391 | 5 | amp |
| TCGA-04-1348 | 17 | 73944314 | 74060274 | 0.704218902 | 3 | amp |
| TCGA-04-1348 | 18 | 47273 | 196821 | 0.923067723 | 4 | amp |
| TCGA-04-1348 | 18 | 197595 | 224191 | 1.933629345 | 6 | amp |
| TCGA-04-1348 | 18 | 224898 | 2590208 | 1.130903047 | 5 | amp |
| TCGA-04-1348 | 18 | 2595367 | 2796145 | 1.613393036 | 6 | amp |
| TCGA-04-1348 | 18 | 2796401 | 6837440 | 1.123132502 | 5 | amp |
| TCGA-04-1348 | 18 | 6851018 | 7044899 | 0.989297609 | 3 | amp |
| TCGA-04-1348 | 18 | 7046261 | 9204567 | 1.17372789 | 5 | amp |
| TCGA-04-1348 | 18 | 9208607 | 9275713 | 1.981263866 | 6 | amp |
| TCGA-04-1348 | 18 | 9279534 | 10546421 | 1.111681664 | 5 | amp |
| TCGA-04-1348 | 18 | 10548275 | 10681763 | 1.788899396 | 6 | amp |
| TCGA-04-1348 | 18 | 10689606 | 11609833 | 1.201000533 | 5 | amp |
| TCGA-04-1348 | 18 | 11610163 | 14392975 | 0.954027866 | 3 | amp |
| TCGA-04-1348 | 18 | 14511753 | 18533762 | 1.366114288 | 5 | amp |
| TCGA-04-1348 | 18 | 18534694 | 19146196 | 1.805022733 | 6 | amp |
| TCGA-04-1348 | 18 | 19147937 | 19244194 | 1.125067686 | 5 | amp |
| TCGA-04-1348 | 18 | 19263454 | 20581740 | 1.587334261 | 6 | amp |
| TCGA-04-1348 | 18 | 20586300 | 21860918 | 1.155006935 | 5 | amp |
| TCGA-04-1348 | 18 | 21883581 | 21898646 | 1.882332669 | 6 | amp |
| TCGA-04-1348 | 18 | 21898698 | 22932159 | 1.192832943 | 5 | amp |
| TCGA-04-1348 | 18 | 23598200 | 23970012 | 1.61261181 | 6 | amp |
| TCGA-04-1348 | 18 | 24035665 | 24445697 | 0.863591341 | 4 | amp |
| TCGA-04-1348 | 18 | 24496138 | 28667823 | 1.580599462 | 6 | amp |
| TCGA-04-1348 | 18 | 28669359 | 28742585 | 1.201524342 | 5 | amp |

| | | | | | | |
|---|---|---|---|---|---|---|
| TCGA-04-1348 | 18 | 28898227 | 28970821 | 1.563278357 | 6 | amp |
| TCGA-04-1348 | 18 | 28970987 | 32954287 | 1.229451209 | 5 | amp |
| TCGA-04-1348 | 18 | 33048520 | 33692563 | 1.807814646 | 6 | amp |
| TCGA-04-1348 | 18 | 33694035 | 33716394 | 1.11308316 | 5 | amp |
| TCGA-04-1348 | 18 | 33718190 | 33747185 | 2.016348947 | 6 | amp |
| TCGA-04-1348 | 18 | 33749972 | 39576707 | 1.176803552 | 5 | amp |
| TCGA-04-1348 | 18 | 39584292 | 39638050 | 1.646815935 | 6 | amp |
| TCGA-04-1348 | 18 | 39644688 | 44471019 | 1.119370178 | 5 | amp |
| TCGA-04-1348 | 18 | 44559614 | 45556328 | 1.305473542 | 5 | amp |
| TCGA-04-1348 | 18 | 46476150 | 46956814 | 1.44412441 | 5 | amp |
| TCGA-04-1348 | 18 | 47008657 | 47431239 | 0.811708727 | 3 | amp |
| TCGA-04-1348 | 18 | 47432774 | 47769497 | 1.249983045 | 5 | amp |
| TCGA-04-1348 | 18 | 47917458 | 48335780 | 1.015333423 | 5 | amp |
| TCGA-04-1348 | 18 | 48422184 | 50994441 | 1.767791927 | 6 | amp |
| TCGA-04-1348 | 18 | 51013127 | 52896312 | 1.200606717 | 5 | amp |
| TCGA-04-1348 | 18 | 52899700 | 54547405 | 1.628646669 | 6 | amp |
| TCGA-04-1348 | 18 | 54591125 | 57016526 | 1.155119404 | 5 | amp |
| TCGA-04-1348 | 18 | 57020423 | 72103964 | 0.99500004 | 3 | amp |
| TCGA-04-1348 | 19 | 71882 | 282341 | 0.900767492 | 3 | amp |
| TCGA-04-1348 | 19 | 6887574 | 6908801 | 1.399359593 | 5 | amp |
| TCGA-04-1348 | 19 | 6913632 | 6937435 | 0.80069828 | 3 | amp |
| TCGA-04-1348 | 19 | 8520253 | 8539149 | 0.977024538 | 3 | amp |
| TCGA-04-1348 | 19 | 8841352 | 9001923 | 0.992373695 | 4 | amp |
| TCGA-04-1348 | 19 | 9002110 | 9091816 | 1.595623031 | 5 | amp |
| TCGA-04-1348 | 19 | 9203911 | 9526433 | 0.930287756 | 4 | amp |
| TCGA-04-1348 | 19 | 9528453 | 9581250 | 1.539488317 | 5 | amp |
| TCGA-04-1348 | 19 | 9581952 | 9870636 | 0.809109038 | 3 | amp |
| TCGA-04-1348 | 19 | 13010116 | 13356097 | 0.595968217 | 3 | amp |
| TCGA-04-1348 | 19 | 13363799 | 13482607 | 1.842774038 | 6 | amp |
| TCGA-04-1348 | 19 | 13563639 | 14676117 | 0.752314401 | 3 | amp |
| TCGA-04-1348 | 19 | 14693933 | 14857800 | 2.037810777 | 6 | amp |
| TCGA-04-1348 | 19 | 14862245 | 14887612 | 1.33769067 | 5 | amp |
| TCGA-04-1348 | 19 | 14909926 | 15053320 | 2.24898431 | 6 | amp |
| TCGA-04-1348 | 19 | 15060984 | 15133986 | 0.626410666 | 3 | amp |
| TCGA-04-1348 | 19 | 15163015 | 15198821 | 2.855779233 | 6 | amp |
| TCGA-04-1348 | 19 | 15350686 | 15587423 | 0.674632842 | 3 | amp |
| TCGA-04-1348 | 19 | 15590061 | 15756720 | 1.021041268 | 4 | amp |
| TCGA-04-1348 | 19 | 15757828 | 16001302 | 1.448833767 | 5 | amp |
| TCGA-04-1348 | 19 | 16003066 | 16025758 | 0.650224344 | 3 | amp |
| TCGA-04-1348 | 19 | 16032791 | 16212174 | 1.543118043 | 5 | amp |
| TCGA-04-1348 | 19 | 16242977 | 16284049 | 1.412385563 | 5 | amp |
| TCGA-04-1348 | 19 | 16284200 | 16861245 | 0.885780132 | 4 | amp |
| TCGA-04-1348 | 19 | 16869987 | 16926136 | 1.40081461 | 5 | amp |
| TCGA-04-1348 | 19 | 16940594 | 17081891 | 0.767301001 | 3 | amp |
| TCGA-04-1348 | 19 | 19789474 | 20579340 | 0.80953488 | 3 | amp |
| TCGA-04-1348 | 19 | 20807055 | 21205700 | 1.011688922 | 4 | amp |
| TCGA-04-1348 | 19 | 21216204 | 21588670 | 1.291437766 | 5 | amp |
| TCGA-04-1348 | 19 | 21605670 | 22153573 | 0.894827651 | 4 | amp |
| TCGA-04-1348 | 19 | 22153966 | 22171767 | 1.902170203 | 6 | amp |
| TCGA-04-1348 | 19 | 22193534 | 30199365 | 0.980202786 | 4 | amp |

| | | | | | | |
|---|---|---|---|---|---|---|
| TCGA-04-1348 | 19 | 30303819 | 30314742 | 1.529377567 | 5 | amp |
| TCGA-04-1348 | 19 | 30462056 | 30528881 | 2.9428096 | 6 | amp |
| TCGA-04-1348 | 19 | 30934384 | 32927532 | 1.277769862 | 5 | amp |
| TCGA-04-1348 | 19 | 32928052 | 32955721 | 2.228266538 | 6 | amp |
| TCGA-04-1348 | 19 | 32959617 | 33467642 | 0.658195446 | 3 | amp |
| TCGA-04-1348 | 19 | 33470851 | 33655196 | 1.024958279 | 4 | amp |
| TCGA-04-1348 | 19 | 34685344 | 34712647 | 1.54474722 | 5 | amp |
| TCGA-04-1348 | 19 | 34718221 | 34925882 | 0.612316049 | 3 | amp |
| TCGA-04-1348 | 19 | 34929490 | 34949871 | 1.708889954 | 6 | amp |
| TCGA-04-1348 | 19 | 34951332 | 34991223 | 0.743291034 | 4 | amp |
| TCGA-04-1348 | 19 | 35033441 | 35435774 | 1.224505736 | 5 | amp |
| TCGA-04-1348 | 19 | 35639593 | 35990955 | 0.619215226 | 3 | amp |
| TCGA-04-1348 | 19 | 36303940 | 36326694 | 1.588261922 | 5 | amp |
| TCGA-04-1348 | 19 | 36673341 | 36940930 | 1.014321665 | 4 | amp |
| TCGA-04-1348 | 19 | 36963770 | 38014408 | 1.309390623 | 5 | amp |
| TCGA-04-1348 | 19 | 38023320 | 38231143 | 1.041844189 | 4 | amp |
| TCGA-04-1348 | 19 | 40097800 | 40228644 | 2.080989839 | 6 | amp |
| TCGA-04-1348 | 19 | 40540258 | 40589094 | 1.587258055 | 5 | amp |
| TCGA-04-1348 | 19 | 41384632 | 41518769 | 1.46081608 | 5 | amp |
| TCGA-04-1348 | 19 | 41522491 | 41800646 | 0.592972028 | 3 | amp |
| TCGA-04-1348 | 19 | 41942246 | 42375491 | 0.785386845 | 3 | amp |
| TCGA-04-1348 | 19 | 42932994 | 43087503 | 0.96481561 | 4 | amp |
| TCGA-04-1348 | 19 | 43092882 | 43234248 | 1.448735855 | 5 | amp |
| TCGA-04-1348 | 19 | 43236894 | 43349246 | 0.875586262 | 4 | amp |
| TCGA-04-1348 | 19 | 43352133 | 43376237 | 1.539390127 | 5 | amp |
| TCGA-04-1348 | 19 | 43382007 | 43448306 | 0.886482467 | 4 | amp |
| TCGA-04-1348 | 19 | 43514111 | 43679673 | 1.688376956 | 6 | amp |
| TCGA-04-1348 | 19 | 43679980 | 43858194 | 0.797012283 | 3 | amp |
| TCGA-04-1348 | 19 | 44065056 | 44339760 | 0.674435421 | 3 | amp |
| TCGA-04-1348 | 19 | 44341147 | 44847472 | 1.519027555 | 5 | amp |
| TCGA-04-1348 | 19 | 44890632 | 45010299 | 0.961098986 | 4 | amp |
| TCGA-04-1348 | 19 | 45015030 | 45021325 | 1.563736843 | 5 | amp |
| TCGA-04-1348 | 19 | 45024466 | 45465335 | 0.683176355 | 3 | amp |
| TCGA-04-1348 | 19 | 46543111 | 46733103 | 1.196225169 | 5 | amp |
| TCGA-04-1348 | 19 | 52249150 | 52663900 | 0.71736038 | 3 | amp |
| TCGA-04-1348 | 19 | 54183145 | 54200888 | 1.287116757 | 5 | amp |
| TCGA-04-1348 | 19 | 54201621 | 54260099 | 0.707274681 | 3 | amp |
| TCGA-04-1348 | 19 | 55174917 | 55317732 | 0.721755029 | 3 | amp |
| TCGA-04-1348 | 19 | 56312901 | 56490964 | 0.848243622 | 3 | amp |
| TCGA-04-1348 | 2 | 41527 | 1418287 | 0.79418339 | 3 | amp |
| TCGA-04-1348 | 2 | 3749043 | 8911000 | 1.172638437 | 5 | amp |
| TCGA-04-1348 | 2 | 8916829 | 8953475 | 1.84668355 | 6 | amp |
| TCGA-04-1348 | 2 | 8957736 | 9645514 | 0.964832195 | 3 | amp |
| TCGA-04-1348 | 2 | 9650063 | 9668118 | 1.92911519 | 6 | amp |
| TCGA-04-1348 | 2 | 9675938 | 11317968 | 0.819255097 | 3 | amp |
| TCGA-04-1348 | 2 | 11323504 | 11593935 | 1.373866237 | 5 | amp |
| TCGA-04-1348 | 2 | 11932025 | 12864972 | 0.928796452 | 3 | amp |
| TCGA-04-1348 | 2 | 12880415 | 15691698 | 1.381815972 | 5 | amp |
| TCGA-04-1348 | 2 | 15693513 | 17836682 | 0.925870066 | 3 | amp |
| TCGA-04-1348 | 2 | 17846762 | 17906634 | 1.963593083 | 6 | amp |

| | | | | | | |
|---|---|---|---|---|---|---|
| TCGA-04-1348 | 2 | 17907635 | 21265350 | 1.199299922 | 5 | amp |
| TCGA-04-1348 | 2 | 23974876 | 24118884 | 1.672361858 | 6 | amp |
| TCGA-04-1348 | 2 | 24181144 | 24443994 | 0.669356275 | 3 | amp |
| TCGA-04-1348 | 2 | 24468973 | 24523088 | 1.669980307 | 6 | amp |
| TCGA-04-1348 | 2 | 24524005 | 26673564 | 0.874661316 | 3 | amp |
| TCGA-04-1348 | 2 | 27693758 | 27706546 | 1.572391625 | 6 | amp |
| TCGA-04-1348 | 2 | 27706711 | 27826210 | 0.855708185 | 3 | amp |
| TCGA-04-1348 | 2 | 27830658 | 28762065 | 1.249536716 | 5 | amp |
| TCGA-04-1348 | 2 | 28763180 | 28852066 | 0.758888114 | 3 | amp |
| TCGA-04-1348 | 2 | 28853401 | 29354313 | 1.151797983 | 5 | amp |
| TCGA-04-1348 | 2 | 29355004 | 29420577 | 1.996963747 | 6 | amp |
| TCGA-04-1348 | 2 | 29429997 | 31609470 | 0.870861893 | 3 | amp |
| TCGA-04-1348 | 2 | 31610659 | 43947907 | 1.332685642 | 5 | amp |
| TCGA-04-1348 | 2 | 43953380 | 44050124 | 2.0319084 | 6 | amp |
| TCGA-04-1348 | 2 | 44051034 | 44101145 | 1.007889613 | 5 | amp |
| TCGA-04-1348 | 2 | 44101493 | 44445707 | 1.996620674 | 6 | amp |
| TCGA-04-1348 | 2 | 44445966 | 45829251 | 1.447394741 | 5 | amp |
| TCGA-04-1348 | 2 | 45832480 | 47657100 | 0.909692467 | 3 | amp |
| TCGA-04-1348 | 2 | 47672681 | 61430456 | 1.307506933 | 5 | amp |
| TCGA-04-1348 | 2 | 61431302 | 61597733 | 1.728737113 | 6 | amp |
| TCGA-04-1348 | 2 | 61605468 | 65467100 | 1.373777868 | 5 | amp |
| TCGA-04-1348 | 2 | 65469129 | 68805172 | 2.022945991 | 6 | amp |
| TCGA-04-1348 | 2 | 68872895 | 69015149 | 0.831425756 | 3 | amp |
| TCGA-04-1348 | 2 | 69034359 | 69601251 | 1.980686689 | 6 | amp |
| TCGA-04-1348 | 2 | 69623339 | 70070396 | 1.417892989 | 5 | amp |
| TCGA-04-1348 | 2 | 70071259 | 70131472 | 2.058627731 | 6 | amp |
| TCGA-04-1348 | 2 | 70143212 | 71661964 | 0.793094469 | 3 | amp |
| TCGA-04-1348 | 2 | 72406406 | 72968635 | 1.694417671 | 6 | amp |
| TCGA-04-1348 | 2 | 73052922 | 74362836 | 0.829816332 | 3 | amp |
| TCGA-04-1348 | 2 | 75196482 | 85270797 | 1.152453938 | 5 | amp |
| TCGA-04-1348 | 2 | 85273193 | 85768558 | 0.70310661 | 3 | amp |
| TCGA-04-1348 | 2 | 86310157 | 86683746 | 1.190901644 | 5 | amp |
| TCGA-04-1348 | 2 | 86683903 | 86718450 | 2.232619883 | 6 | amp |
| TCGA-04-1348 | 2 | 86719111 | 95484711 | 1.256543069 | 5 | amp |
| TCGA-04-1348 | 2 | 95488723 | 95504633 | 2.122703759 | 6 | amp |
| TCGA-04-1348 | 2 | 95511075 | 96519652 | 0.780493537 | 3 | amp |
| TCGA-04-1348 | 2 | 96521110 | 96595060 | 1.833080951 | 6 | amp |
| TCGA-04-1348 | 2 | 96652462 | 97399356 | 0.77319055 | 3 | amp |
| TCGA-04-1348 | 2 | 97784050 | 98201610 | 1.709377093 | 6 | amp |
| TCGA-04-1348 | 2 | 98201744 | 99233005 | 0.801974804 | 3 | amp |
| TCGA-04-1348 | 2 | 99234668 | 100628055 | 1.241999108 | 5 | amp |
| TCGA-04-1348 | 2 | 100903330 | 102315027 | 0.805149171 | 3 | amp |
| TCGA-04-1348 | 2 | 102407149 | 113765634 | 1.232115242 | 5 | amp |
| TCGA-04-1348 | 2 | 113780242 | 113789339 | 2.18747458 | 6 | amp |
| TCGA-04-1348 | 2 | 113816939 | 114476927 | 0.979320612 | 3 | amp |
| TCGA-04-1348 | 2 | 114480651 | 116540041 | 1.379874738 | 5 | amp |
| TCGA-04-1348 | 2 | 116548645 | 120439377 | 0.926999531 | 3 | amp |
| TCGA-04-1348 | 2 | 120567378 | 120776870 | 1.436888952 | 5 | amp |
| TCGA-04-1348 | 2 | 120799573 | 120900762 | 1.811349224 | 6 | amp |
| TCGA-04-1348 | 2 | 120903772 | 122125465 | 0.709934625 | 3 | amp |

| | | | | | | |
|---|---|---|---|---|---|---|
| TCGA-04-1348 | 2 | 122135029 | 127315655 | 1.223325214 | 5 | amp |
| TCGA-04-1348 | 2 | 127447768 | 128253768 | 0.784796153 | 3 | amp |
| TCGA-04-1348 | 2 | 128703021 | 128939896 | 1.46432451 | 5 | amp |
| TCGA-04-1348 | 2 | 128941245 | 130910799 | 0.942135506 | 3 | amp |
| TCGA-04-1348 | 2 | 131231913 | 131254220 | 1.795771878 | 6 | amp |
| TCGA-04-1348 | 2 | 131256605 | 131379227 | 0.599633826 | 3 | amp |
| TCGA-04-1348 | 2 | 131381611 | 131403920 | 1.913559481 | 6 | amp |
| TCGA-04-1348 | 2 | 131412433 | 132010709 | 0.730421597 | 3 | amp |
| TCGA-04-1348 | 2 | 133066829 | 138169490 | 1.153018309 | 5 | amp |
| TCGA-04-1348 | 2 | 138208383 | 149241021 | 1.813925251 | 6 | amp |
| TCGA-04-1348 | 2 | 149243294 | 152420236 | 1.29503696 | 5 | amp |
| TCGA-04-1348 | 2 | 152420330 | 152501095 | 1.778386949 | 6 | amp |
| TCGA-04-1348 | 2 | 152502635 | 157427844 | 1.274817674 | 5 | amp |
| TCGA-04-1348 | 2 | 157435396 | 159107484 | 1.680330741 | 6 | amp |
| TCGA-04-1348 | 2 | 159165927 | 160181513 | 1.169208731 | 5 | amp |
| TCGA-04-1348 | 2 | 160182116 | 160585729 | 1.724259659 | 6 | amp |
| TCGA-04-1348 | 2 | 160599547 | 160672119 | 1.334377998 | 5 | amp |
| TCGA-04-1348 | 2 | 160673309 | 160840631 | 1.700008629 | 6 | amp |
| TCGA-04-1348 | 2 | 160843647 | 166237714 | 1.37862872 | 5 | amp |
| TCGA-04-1348 | 2 | 166243210 | 167992615 | 1.660966325 | 6 | amp |
| TCGA-04-1348 | 2 | 168067174 | 168921919 | 1.318348875 | 5 | amp |
| TCGA-04-1348 | 2 | 168931446 | 169417886 | 1.915789142 | 6 | amp |
| TCGA-04-1348 | 2 | 169487463 | 171192017 | 0.870017347 | 3 | amp |
| TCGA-04-1348 | 2 | 171225689 | 176044925 | 1.282398331 | 5 | amp |
| TCGA-04-1348 | 2 | 176794690 | 176947221 | 1.848524628 | 6 | amp |
| TCGA-04-1348 | 2 | 176948050 | 177135202 | 0.795081455 | 3 | amp |
| TCGA-04-1348 | 2 | 177161551 | 179301078 | 2.33703509 | 6 | amp |
| TCGA-04-1348 | 2 | 179306323 | 179343265 | 1.201965422 | 5 | amp |
| TCGA-04-1348 | 2 | 179350305 | 179393960 | 2.235733893 | 6 | amp |
| TCGA-04-1348 | 2 | 179394644 | 179462829 | 1.471479676 | 5 | amp |
| TCGA-04-1348 | 2 | 179463186 | 179516712 | 1.743792859 | 6 | amp |
| TCGA-04-1348 | 2 | 179516804 | 179530205 | 1.404455998 | 5 | amp |
| TCGA-04-1348 | 2 | 179531529 | 179537230 | 2.299863385 | 6 | amp |
| TCGA-04-1348 | 2 | 179537335 | 179710530 | 1.33873463 | 5 | amp |
| TCGA-04-1348 | 2 | 179714771 | 182438665 | 1.70873692 | 6 | amp |
| TCGA-04-1348 | 2 | 182468415 | 183597307 | 1.244418148 | 5 | amp |
| TCGA-04-1348 | 2 | 183600924 | 185803798 | 1.777871571 | 6 | amp |
| TCGA-04-1348 | 2 | 186411813 | 189904323 | 1.383114631 | 5 | amp |
| TCGA-04-1348 | 2 | 189906275 | 189945802 | 1.758321745 | 6 | amp |
| TCGA-04-1348 | 2 | 189948646 | 190927375 | 1.345291578 | 5 | amp |
| TCGA-04-1348 | 2 | 191064520 | 191227442 | 2.064158633 | 6 | amp |
| TCGA-04-1348 | 2 | 191231370 | 201477543 | 1.37367237 | 5 | amp |
| TCGA-04-1348 | 2 | 201478457 | 201521672 | 1.925850549 | 6 | amp |
| TCGA-04-1348 | 2 | 201523831 | 207310259 | 1.256531943 | 5 | amp |
| TCGA-04-1348 | 2 | 207345903 | 207825680 | 1.693185357 | 6 | amp |
| TCGA-04-1348 | 2 | 207827086 | 216300571 | 1.415968061 | 5 | amp |
| TCGA-04-1348 | 2 | 216809532 | 216931294 | 2.399044228 | 6 | amp |
| TCGA-04-1348 | 2 | 216946252 | 216983946 | 1.06396829 | 5 | amp |
| TCGA-04-1348 | 2 | 216986803 | 217069977 | 2.004313718 | 6 | amp |
| TCGA-04-1348 | 2 | 217123948 | 219134868 | 0.950287098 | 3 | amp |

| | | | | | | |
|---|---|---|---|---|---|---|
| TCGA-04-1348 | 2 | 219313912 | 219457444 | 1.679560236 | 6 | amp |
| TCGA-04-1348 | 2 | 219458764 | 219533424 | 0.803737546 | 3 | amp |
| TCGA-04-1348 | 2 | 219536582 | 219677493 | 1.257712257 | 5 | amp |
| TCGA-04-1348 | 2 | 222290714 | 228205111 | 1.423546476 | 5 | amp |
| TCGA-04-1348 | 2 | 228207437 | 231157551 | 1.97510322 | 6 | amp |
| TCGA-04-1348 | 2 | 231158949 | 231314988 | 1.272582871 | 5 | amp |
| TCGA-04-1348 | 2 | 231325902 | 231624833 | 2.294292555 | 6 | amp |
| TCGA-04-1348 | 2 | 231655518 | 231934884 | 1.228255225 | 5 | amp |
| TCGA-04-1348 | 2 | 231936895 | 232081548 | 1.961704528 | 6 | amp |
| TCGA-04-1348 | 2 | 232087396 | 232879723 | 0.96401664 | 3 | amp |
| TCGA-04-1348 | 2 | 232880182 | 233075135 | 1.888689742 | 6 | amp |
| TCGA-04-1348 | 2 | 233103208 | 233199263 | 1.014901128 | 3 | amp |
| TCGA-04-1348 | 2 | 233546211 | 233709301 | 2.01238417 | 6 | amp |
| TCGA-04-1348 | 2 | 233710362 | 234173843 | 0.788421355 | 3 | amp |
| TCGA-04-1348 | 2 | 234178590 | 234217932 | 1.834466649 | 6 | amp |
| TCGA-04-1348 | 2 | 234224690 | 234396977 | 0.803547846 | 3 | amp |
| TCGA-04-1348 | 2 | 234399799 | 234602560 | 2.082627775 | 6 | amp |
| TCGA-04-1348 | 2 | 234621590 | 234916790 | 1.16625049 | 5 | amp |
| TCGA-04-1348 | 2 | 234923170 | 234975952 | 2.1552489 | 6 | amp |
| TCGA-04-1348 | 2 | 234978554 | 238672051 | 0.895437693 | 3 | amp |
| TCGA-04-1348 | 2 | 238672095 | 238743102 | 1.624548569 | 6 | amp |
| TCGA-04-1348 | 2 | 238785784 | 239155210 | 0.728684708 | 3 | amp |
| TCGA-04-1348 | 20 | 68319 | 210465 | 1.484263714 | 5 | amp |
| TCGA-04-1348 | 20 | 3285048 | 3624917 | 1.201433938 | 5 | amp |
| TCGA-04-1348 | 20 | 3888568 | 3928964 | 1.49763213 | 5 | amp |
| TCGA-04-1348 | 20 | 3944472 | 6031646 | 0.915486009 | 4 | amp |
| TCGA-04-1348 | 20 | 6057048 | 10541527 | 1.400944685 | 5 | amp |
| TCGA-04-1348 | 20 | 10579301 | 10653681 | 0.809555138 | 4 | amp |
| TCGA-04-1348 | 20 | 11898876 | 16387119 | 1.346786824 | 5 | amp |
| TCGA-04-1348 | 20 | 16407667 | 16478350 | 2.466631102 | 6 | amp |
| TCGA-04-1348 | 20 | 16484984 | 17339088 | 1.239843797 | 5 | amp |
| TCGA-04-1348 | 20 | 17341170 | 17641187 | 0.530693099 | 3 | amp |
| TCGA-04-1348 | 20 | 17705613 | 20056315 | 0.943986249 | 4 | amp |
| TCGA-04-1348 | 20 | 20066082 | 20565613 | 1.243574077 | 5 | amp |
| TCGA-04-1348 | 20 | 20569900 | 20600109 | 2.034079243 | 6 | amp |
| TCGA-04-1348 | 20 | 20601070 | 21349284 | 1.445846096 | 5 | amp |
| TCGA-04-1348 | 20 | 21362603 | 23473711 | 0.848645936 | 3 | amp |
| TCGA-04-1348 | 20 | 25733291 | 29978315 | 1.36425031 | 5 | amp |
| TCGA-04-1348 | 20 | 29992681 | 30310039 | 0.633098421 | 3 | amp |
| TCGA-04-1348 | 20 | 30345272 | 30388887 | 1.707446732 | 6 | amp |
| TCGA-04-1348 | 20 | 30407868 | 30616939 | 0.603781008 | 3 | amp |
| TCGA-04-1348 | 20 | 30617493 | 30818922 | 0.892853899 | 4 | amp |
| TCGA-04-1348 | 20 | 30822249 | 30956931 | 1.569566629 | 5 | amp |
| TCGA-04-1348 | 20 | 31015870 | 31413913 | 0.705767746 | 3 | amp |
| TCGA-04-1348 | 20 | 31421475 | 31436565 | 2.435337067 | 6 | amp |
| TCGA-04-1348 | 20 | 31695527 | 31815443 | 1.308395992 | 5 | amp |
| TCGA-04-1348 | 20 | 31825477 | 31830372 | 2.166188955 | 6 | amp |
| TCGA-04-1348 | 20 | 31873876 | 33517418 | 0.902172524 | 4 | amp |
| TCGA-04-1348 | 20 | 33519089 | 33902589 | 0.656222742 | 3 | amp |
| TCGA-04-1348 | 20 | 33934960 | 34292512 | 0.846185497 | 4 | amp |

| | | | | | | |
|---|---|---|---|---|---|---|
| TCGA-04-1348 | 20 | 34292538 | 34556769 | 1.26847917 | 5 | amp |
| TCGA-04-1348 | 20 | 35515804 | 35563631 | 1.39118634 | 5 | amp |
| TCGA-04-1348 | 20 | 35569387 | 35812799 | 0.820019304 | 4 | amp |
| TCGA-04-1348 | 20 | 35826787 | 35858478 | 1.217214332 | 5 | amp |
| TCGA-04-1348 | 20 | 35860686 | 35879684 | 2.010525348 | 6 | amp |
| TCGA-04-1348 | 20 | 37117048 | 37198644 | 1.279746807 | 5 | amp |
| TCGA-04-1348 | 20 | 37199360 | 37597910 | 0.673940039 | 4 | amp |
| TCGA-04-1348 | 20 | 37601187 | 39751945 | 1.342564917 | 5 | amp |
| TCGA-04-1348 | 20 | 40043811 | 41514629 | 1.343904208 | 5 | amp |
| TCGA-04-1348 | 20 | 43048288 | 43600848 | 0.830505782 | 3 | amp |
| TCGA-04-1348 | 20 | 43607027 | 43629216 | 2.290449304 | 6 | amp |
| TCGA-04-1348 | 20 | 43629780 | 43883201 | 1.207714979 | 5 | amp |
| TCGA-04-1348 | 20 | 43922375 | 44108777 | 0.642981024 | 3 | amp |
| TCGA-04-1348 | 20 | 44163034 | 44184543 | 2.295587162 | 6 | amp |
| TCGA-04-1348 | 20 | 44187440 | 44405900 | 1.214568607 | 5 | amp |
| TCGA-04-1348 | 20 | 44671726 | 44996218 | 0.787786022 | 3 | amp |
| TCGA-04-1348 | 20 | 44997478 | 45022767 | 2.151415721 | 6 | amp |
| TCGA-04-1348 | 20 | 45023022 | 45891221 | 1.242892772 | 5 | amp |
| TCGA-04-1348 | 20 | 45904968 | 46279813 | 2.050101395 | 6 | amp |
| TCGA-04-1348 | 20 | 46279901 | 46288229 | 1.148331465 | 5 | amp |
| TCGA-04-1348 | 20 | 47307439 | 47602167 | 1.110702063 | 5 | amp |
| TCGA-04-1348 | 20 | 47604822 | 47612429 | 2.197923934 | 6 | amp |
| TCGA-04-1348 | 20 | 47614696 | 47679929 | 1.242909493 | 5 | amp |
| TCGA-04-1348 | 20 | 47682719 | 47706298 | 2.206798499 | 6 | amp |
| TCGA-04-1348 | 20 | 47707237 | 47782755 | 1.479597419 | 5 | amp |
| TCGA-04-1348 | 20 | 47835835 | 48253988 | 0.917971671 | 4 | amp |
| TCGA-04-1348 | 20 | 48256130 | 48500612 | 1.534290845 | 5 | amp |
| TCGA-04-1348 | 20 | 48503211 | 52558089 | 0.829989115 | 4 | amp |
| TCGA-04-1348 | 20 | 52561407 | 52779448 | 2.104102887 | 6 | amp |
| TCGA-04-1348 | 20 | 52780986 | 52832028 | 1.098223408 | 5 | amp |
| TCGA-04-1348 | 20 | 52835623 | 53260176 | 2.123186587 | 6 | amp |
| TCGA-04-1348 | 20 | 53266925 | 54945747 | 1.340298932 | 5 | amp |
| TCGA-04-1348 | 20 | 54948387 | 54987592 | 2.054143722 | 6 | amp |
| TCGA-04-1348 | 20 | 55012160 | 55059265 | 1.540529049 | 5 | amp |
| TCGA-04-1348 | 20 | 55091927 | 56886198 | 0.840752867 | 4 | amp |
| TCGA-04-1348 | 20 | 56918724 | 56934770 | 3.233156962 | 6 | amp |
| TCGA-04-1348 | 20 | 56993222 | 57243232 | 1.095060905 | 5 | amp |
| TCGA-04-1348 | 20 | 57244307 | 58425556 | 0.647648587 | 3 | amp |
| TCGA-04-1348 | 20 | 58439344 | 58482508 | 2.110685015 | 6 | amp |
| TCGA-04-1348 | 20 | 58486792 | 58559868 | 1.374784704 | 5 | amp |
| TCGA-04-1348 | 21 | 9483321 | 17138507 | 1.389605626 | 5 | amp |
| TCGA-04-1348 | 21 | 17150164 | 17912249 | 1.876275449 | 6 | amp |
| TCGA-04-1348 | 21 | 17962540 | 19756169 | 1.198790937 | 5 | amp |
| TCGA-04-1348 | 21 | 19770169 | 26946383 | 1.811433911 | 6 | amp |
| TCGA-04-1348 | 21 | 26958010 | 30407269 | 1.240396457 | 5 | amp |
| TCGA-04-1348 | 21 | 30408583 | 30433912 | 2.195671268 | 6 | amp |
| TCGA-04-1348 | 21 | 30434446 | 31971218 | 1.427636916 | 5 | amp |
| TCGA-04-1348 | 21 | 31973392 | 32435274 | 1.96564614 | 6 | amp |
| TCGA-04-1348 | 21 | 32492680 | 32598273 | 1.241050555 | 5 | amp |
| TCGA-04-1348 | 21 | 32617739 | 33980052 | 0.768503535 | 3 | amp |

| | | | | | | |
|---|---|---|---|---|---|---|
| TCGA-04-1348 | 21 | 33982111 | 35514923 | 1.201544532 | 5 | amp |
| TCGA-04-1348 | 21 | 35742693 | 37620891 | 0.740164515 | 3 | amp |
| TCGA-04-1348 | 21 | 37623400 | 38462619 | 1.12035867 | 5 | amp |
| TCGA-04-1348 | 21 | 38463532 | 39580574 | 1.953637986 | 6 | amp |
| TCGA-04-1348 | 21 | 39671117 | 42551578 | 1.308315019 | 5 | amp |
| TCGA-04-1348 | 21 | 42598146 | 42719043 | 0.712605549 | 3 | amp |
| TCGA-04-1348 | 22 | 16084594 | 17119669 | 0.84017331 | 3 | amp |
| TCGA-04-1348 | 22 | 17280607 | 17414938 | 1.181653771 | 4 | amp |
| TCGA-04-1348 | 22 | 18080916 | 18210351 | 0.761111519 | 3 | amp |
| TCGA-04-1348 | 22 | 21107132 | 21288539 | 0.639294224 | 3 | amp |
| TCGA-04-1348 | 22 | 24698155 | 24743158 | 0.832237875 | 3 | amp |
| TCGA-04-1348 | 22 | 25130026 | 25156015 | 0.82308017 | 3 | amp |
| TCGA-04-1348 | 22 | 26000321 | 26114399 | 0.692121607 | 3 | amp |
| TCGA-04-1348 | 22 | 26272107 | 26778061 | 0.630707851 | 3 | amp |
| TCGA-04-1348 | 22 | 28254315 | 29121412 | 0.738616572 | 3 | amp |
| TCGA-04-1348 | 22 | 29914961 | 30204196 | 0.680637394 | 3 | amp |
| TCGA-04-1348 | 22 | 30209384 | 30409557 | 1.443002975 | 5 | amp |
| TCGA-04-1348 | 22 | 30412481 | 30421824 | 0.642301307 | 3 | amp |
| TCGA-04-1348 | 22 | 30489906 | 30572163 | 1.428836288 | 5 | amp |
| TCGA-04-1348 | 22 | 31330687 | 31338298 | 1.446194989 | 5 | amp |
| TCGA-04-1348 | 22 | 31798982 | 31927139 | 1.417312808 | 5 | amp |
| TCGA-04-1348 | 22 | 31942841 | 31981146 | 1.076686233 | 4 | amp |
| TCGA-04-1348 | 22 | 32044054 | 35683452 | 0.669783773 | 3 | amp |
| TCGA-04-1348 | 22 | 36006928 | 36651073 | 0.697313308 | 3 | amp |
| TCGA-04-1348 | 22 | 38883791 | 38951447 | 0.752586412 | 3 | amp |
| TCGA-04-1348 | 22 | 40139625 | 40681831 | 0.693783149 | 3 | amp |
| TCGA-04-1348 | 22 | 41264940 | 41575008 | 0.594876331 | 3 | amp |
| TCGA-04-1348 | 22 | 43213671 | 43243698 | 0.778969854 | 3 | amp |
| TCGA-04-1348 | 22 | 43972152 | 44031098 | 1.140415972 | 5 | amp |
| TCGA-04-1348 | 22 | 44062947 | 44112909 | 1.686692036 | 6 | amp |
| TCGA-04-1348 | 22 | 44127574 | 44178248 | 0.861979444 | 3 | amp |
| TCGA-04-1348 | 22 | 44359130 | 44395570 | 0.672300926 | 3 | amp |
| TCGA-04-1348 | 22 | 45740397 | 45782956 | 1.453855278 | 5 | amp |
| TCGA-04-1348 | 22 | 45785564 | 45802562 | 1.074255617 | 4 | amp |
| TCGA-04-1348 | 22 | 46098527 | 46202930 | 1.618572922 | 6 | amp |
| TCGA-04-1348 | 3 | 361444 | 9719764 | 1.412386592 | 5 | amp |
| TCGA-04-1348 | 3 | 9724804 | 9768437 | 0.817484415 | 3 | amp |
| TCGA-04-1348 | 3 | 9990369 | 10167410 | 1.352402938 | 5 | amp |
| TCGA-04-1348 | 3 | 10167905 | 11302235 | 0.688348314 | 3 | amp |
| TCGA-04-1348 | 3 | 11340129 | 12779710 | 1.521424885 | 5 | amp |
| TCGA-04-1348 | 3 | 12779979 | 12955110 | 0.882113383 | 3 | amp |
| TCGA-04-1348 | 3 | 14696995 | 14974715 | 0.870604599 | 3 | amp |
| TCGA-04-1348 | 3 | 15045397 | 25640002 | 1.466066289 | 5 | amp |
| TCGA-04-1348 | 3 | 25640912 | 27498217 | 2.520651872 | 6 | amp |
| TCGA-04-1348 | 3 | 27758511 | 37560857 | 1.479981408 | 5 | amp |
| TCGA-04-1348 | 3 | 37564996 | 38740106 | 0.785738412 | 3 | amp |
| TCGA-04-1348 | 3 | 38743254 | 45972787 | 1.102036328 | 5 | amp |
| TCGA-04-1348 | 3 | 45977927 | 46621555 | 0.880380642 | 3 | amp |
| TCGA-04-1348 | 3 | 47059118 | 48265221 | 0.935118376 | 3 | amp |
| TCGA-04-1348 | 3 | 50685250 | 50816207 | 0.696484848 | 4 | amp |

| | | | | | | |
|---|---|---|---|---|---|---|
| TCGA-04-1348 | 3 | 50879065 | 51718659 | 1.303275684 | 5 | amp |
| TCGA-04-1348 | 3 | 54596772 | 54880516 | 0.839396313 | 3 | amp |
| TCGA-04-1348 | 3 | 54905547 | 57743597 | 1.404627425 | 5 | amp |
| TCGA-04-1348 | 3 | 57817063 | 57913128 | 2.461657734 | 6 | amp |
| TCGA-04-1348 | 3 | 57994257 | 111821868 | 1.287861325 | 5 | amp |
| TCGA-04-1348 | 3 | 111828364 | 112650045 | 2.23587032 | 6 | amp |
| TCGA-04-1348 | 3 | 112666646 | 122433312 | 1.203518045 | 5 | amp |
| TCGA-04-1348 | 3 | 122436900 | 123419899 | 0.650225075 | 3 | amp |
| TCGA-04-1348 | 3 | 123420230 | 125509489 | 1.101266701 | 5 | amp |
| TCGA-04-1348 | 3 | 130159007 | 130361753 | 0.893072962 | 3 | amp |
| TCGA-04-1348 | 3 | 130361788 | 133379989 | 1.356413409 | 5 | amp |
| TCGA-04-1348 | 3 | 133467221 | 133672615 | 0.509060653 | 3 | amp |
| TCGA-04-1348 | 3 | 133673773 | 134214226 | 1.231514247 | 5 | amp |
| TCGA-04-1348 | 3 | 134225919 | 136667317 | 1.939458194 | 6 | amp |
| TCGA-04-1348 | 3 | 136676939 | 138384081 | 1.290148754 | 5 | amp |
| TCGA-04-1348 | 3 | 138400787 | 139098324 | 2.192585042 | 6 | amp |
| TCGA-04-1348 | 3 | 139102088 | 141164824 | 1.53754481 | 5 | amp |
| TCGA-04-1348 | 3 | 141230943 | 142119397 | 2.006073811 | 6 | amp |
| TCGA-04-1348 | 3 | 142122474 | 142184130 | 1.542446959 | 5 | amp |
| TCGA-04-1348 | 3 | 142185150 | 146323052 | 1.886040987 | 6 | amp |
| TCGA-04-1348 | 3 | 147106555 | 147131359 | 0.977492169 | 4 | amp |
| TCGA-04-1348 | 3 | 148458762 | 159614597 | 2.428084781 | 6 | amp |
| TCGA-04-1348 | 3 | 159713118 | 169574696 | 2.828843589 | 6 | amp |
| TCGA-04-1348 | 3 | 169578315 | 169656319 | 1.312722673 | 5 | amp |
| TCGA-04-1348 | 3 | 169693389 | 170013828 | 2.228216417 | 6 | amp |
| TCGA-04-1348 | 3 | 170015046 | 170219195 | 1.259923377 | 5 | amp |
| TCGA-04-1348 | 3 | 170244393 | 170825043 | 2.246458112 | 6 | amp |
| TCGA-04-1348 | 3 | 170825842 | 170895201 | 1.391149212 | 5 | amp |
| TCGA-04-1348 | 3 | 170906435 | 178976796 | 2.38684275 | 6 | amp |
| TCGA-04-1348 | 3 | 178977269 | 179085404 | 1.442461553 | 5 | amp |
| TCGA-04-1348 | 3 | 179085797 | 179399848 | 2.388231382 | 6 | amp |
| TCGA-04-1348 | 3 | 179407998 | 180337798 | 1.390839608 | 5 | amp |
| TCGA-04-1348 | 3 | 180349198 | 183059462 | 2.415250045 | 6 | amp |
| TCGA-04-1348 | 3 | 183097081 | 183273474 | 1.341899169 | 5 | amp |
| TCGA-04-1348 | 3 | 183368124 | 183493953 | 2.146758298 | 6 | amp |
| TCGA-04-1348 | 3 | 183495288 | 183778158 | 1.552190556 | 5 | amp |
| TCGA-04-1348 | 3 | 183801647 | 183824486 | 0.885890639 | 3 | amp |
| TCGA-04-1348 | 3 | 184428553 | 184542616 | 0.861859049 | 4 | amp |
| TCGA-04-1348 | 3 | 184543924 | 185198348 | 2.698355549 | 6 | amp |
| TCGA-04-1348 | 3 | 185200133 | 186384031 | 1.542917277 | 5 | amp |
| TCGA-04-1348 | 3 | 186386661 | 186395677 | 3.491440507 | 6 | amp |
| TCGA-04-1348 | 3 | 186435308 | 187451485 | 1.232044406 | 5 | amp |
| TCGA-04-1348 | 3 | 188123854 | 193132573 | 2.488351004 | 6 | amp |
| TCGA-04-1348 | 3 | 193151609 | 193158446 | 0.710935618 | 3 | amp |
| TCGA-04-1348 | 3 | 193159195 | 193409943 | 2.417925401 | 6 | amp |
| TCGA-04-1348 | 3 | 193854163 | 194126866 | 1.024376458 | 5 | amp |
| TCGA-04-1348 | 3 | 194134467 | 194182983 | 2.437688584 | 6 | amp |
| TCGA-04-1348 | 3 | 194309252 | 195000166 | 1.171693308 | 5 | amp |
| TCGA-04-1348 | 3 | 195006495 | 195306390 | 2.254934937 | 6 | amp |
| TCGA-04-1348 | 3 | 195345440 | 195779113 | 0.515333696 | 3 | amp |

| | | | | | | |
|---|---|---|---|---|---|---|
| TCGA-04-1348 | 3 | 195780238 | 197541970 | 1.352086677 | 5 | amp |
| TCGA-04-1348 | 3 | 197543989 | 197597209 | 2.477459743 | 6 | amp |
| TCGA-04-1348 | 3 | 197598148 | 197955154 | 1.560153055 | 5 | amp |
| TCGA-04-1348 | 4 | 2160849 | 2231011 | 1.533803933 | 6 | amp |
| TCGA-04-1348 | 4 | 2233650 | 3182429 | 0.820957159 | 4 | amp |
| TCGA-04-1348 | 4 | 10100569 | 25335122 | 0.927186836 | 4 | amp |
| TCGA-04-1348 | 4 | 25335509 | 25408906 | 1.327112999 | 5 | amp |
| TCGA-04-1348 | 4 | 25411306 | 26616112 | 0.834882876 | 4 | amp |
| TCGA-04-1348 | 4 | 26622212 | 31144522 | 1.111838944 | 5 | amp |
| TCGA-04-1348 | 4 | 36069474 | 36231135 | 1.727585758 | 6 | amp |
| TCGA-04-1348 | 4 | 37590415 | 38972783 | 1.135690768 | 5 | amp |
| TCGA-04-1348 | 4 | 38987907 | 39325058 | 1.571147155 | 6 | amp |
| TCGA-04-1348 | 4 | 39328131 | 44724282 | 1.253804008 | 5 | amp |
| TCGA-04-1348 | 4 | 46042927 | 46388208 | 1.662678154 | 6 | amp |
| TCGA-04-1348 | 4 | 46390597 | 47597948 | 1.222832052 | 5 | amp |
| TCGA-04-1348 | 4 | 47602177 | 48230682 | 1.628862539 | 6 | amp |
| TCGA-04-1348 | 4 | 48371819 | 48503754 | 1.197169059 | 5 | amp |
| TCGA-04-1348 | 4 | 48504793 | 48862820 | 1.640869264 | 6 | amp |
| TCGA-04-1348 | 4 | 48887451 | 49000580 | 1.168920448 | 5 | amp |
| TCGA-04-1348 | 4 | 49005670 | 52890344 | 1.62813739 | 6 | amp |
| TCGA-04-1348 | 4 | 52894084 | 54266011 | 1.181498992 | 5 | amp |
| TCGA-04-1348 | 4 | 54280775 | 54344940 | 1.643013486 | 6 | amp |
| TCGA-04-1348 | 4 | 54347833 | 56230459 | 1.218369849 | 5 | amp |
| TCGA-04-1348 | 4 | 56233701 | 56349009 | 1.703515611 | 6 | amp |
| TCGA-04-1348 | 4 | 56422680 | 57248822 | 1.22639792 | 5 | amp |
| TCGA-04-1348 | 4 | 57250230 | 57857108 | 0.939381565 | 4 | amp |
| TCGA-04-1348 | 4 | 57860535 | 57873194 | 1.329970154 | 5 | amp |
| TCGA-04-1348 | 4 | 57876478 | 66509172 | 1.637713612 | 6 | amp |
| TCGA-04-1348 | 4 | 67142418 | 69101986 | 1.33403758 | 5 | amp |
| TCGA-04-1348 | 4 | 69107404 | 70355342 | 1.661111793 | 6 | amp |
| TCGA-04-1348 | 4 | 70359364 | 70620981 | 1.232083725 | 5 | amp |
| TCGA-04-1348 | 4 | 70707707 | 71500334 | 1.702315326 | 6 | amp |
| TCGA-04-1348 | 4 | 71501519 | 72319418 | 1.353397118 | 5 | amp |
| TCGA-04-1348 | 4 | 72332107 | 73280729 | 1.612594705 | 6 | amp |
| TCGA-04-1348 | 4 | 73414157 | 73968349 | 1.167197064 | 5 | amp |
| TCGA-04-1348 | 4 | 73979474 | 74008521 | 1.726514614 | 6 | amp |
| TCGA-04-1348 | 4 | 74010445 | 74702866 | 1.265853261 | 5 | amp |
| TCGA-04-1348 | 4 | 74702871 | 76570929 | 0.965064578 | 4 | amp |
| TCGA-04-1348 | 4 | 76571460 | 76704041 | 1.38935708 | 5 | amp |
| TCGA-04-1348 | 4 | 76708164 | 76726494 | 1.921411053 | 6 | amp |
| TCGA-04-1348 | 4 | 76730094 | 76857444 | 0.73304123 | 4 | amp |
| TCGA-04-1348 | 4 | 76861085 | 76903197 | 1.636260436 | 6 | amp |
| TCGA-04-1348 | 4 | 76911859 | 78979261 | 1.252014437 | 5 | amp |
| TCGA-04-1348 | 4 | 78987094 | 113377863 | 0.923366167 | 4 | amp |
| TCGA-04-1348 | 4 | 115898296 | 142153857 | 0.875405865 | 4 | amp |
| TCGA-04-1348 | 4 | 142154863 | 144133583 | 1.185863501 | 5 | amp |
| TCGA-04-1348 | 4 | 144134664 | 144471263 | 2.020786309 | 6 | amp |
| TCGA-04-1348 | 4 | 144474244 | 146576622 | 1.278164629 | 5 | amp |
| TCGA-04-1348 | 4 | 146601351 | 166243257 | 0.929296115 | 4 | amp |
| TCGA-04-1348 | 4 | 166254469 | 169223628 | 1.198944162 | 5 | amp |

| | | | | | | |
|---|---|---|---|---|---|---|
| TCGA-04-1348 | 4 | 169227520 | 186296883 | 0.872516881 | 4 | amp |
| TCGA-04-1348 | 5 | 5200313 | 7414904 | 0.839959171 | 3 | amp |
| TCGA-04-1348 | 5 | 7520811 | 7875549 | 1.091361813 | 4 | amp |
| TCGA-04-1348 | 5 | 7878005 | 11199786 | 1.204911023 | 5 | amp |
| TCGA-04-1348 | 5 | 11236718 | 14336860 | 1.851883536 | 6 | amp |
| TCGA-04-1348 | 5 | 14358221 | 14749467 | 0.8663636 | 3 | amp |
| TCGA-04-1348 | 5 | 14751142 | 16877826 | 1.042099466 | 4 | amp |
| TCGA-04-1348 | 5 | 16902636 | 17354033 | 1.361749429 | 5 | amp |
| TCGA-04-1348 | 5 | 18049639 | 23509236 | 1.815161626 | 6 | amp |
| TCGA-04-1348 | 5 | 23509520 | 23527891 | 0.815669109 | 3 | amp |
| TCGA-04-1348 | 5 | 24487716 | 31451753 | 1.70068975 | 6 | amp |
| TCGA-04-1348 | 5 | 31464336 | 32775001 | 1.310524984 | 5 | amp |
| TCGA-04-1348 | 5 | 32780814 | 33684181 | 1.699122907 | 6 | amp |
| TCGA-04-1348 | 5 | 33751460 | 36066904 | 1.272181285 | 5 | amp |
| TCGA-04-1348 | 5 | 36104117 | 36301532 | 2.11038979 | 6 | amp |
| TCGA-04-1348 | 5 | 36608495 | 38482368 | 1.296765673 | 5 | amp |
| TCGA-04-1348 | 5 | 38482649 | 39393545 | 1.981781256 | 6 | amp |
| TCGA-04-1348 | 5 | 39394316 | 40777737 | 1.173327731 | 5 | amp |
| TCGA-04-1348 | 5 | 40798107 | 41203380 | 1.596279742 | 6 | amp |
| TCGA-04-1348 | 5 | 41313675 | 52227981 | 1.314564156 | 5 | amp |
| TCGA-04-1348 | 5 | 52229714 | 52374712 | 1.774344207 | 6 | amp |
| TCGA-04-1348 | 5 | 52376335 | 54275271 | 1.403147617 | 5 | amp |
| TCGA-04-1348 | 5 | 54277779 | 55186000 | 1.665880275 | 6 | amp |
| TCGA-04-1348 | 5 | 55195701 | 55213009 | 0.943203254 | 4 | amp |
| TCGA-04-1348 | 5 | 55236858 | 56207419 | 1.728548345 | 6 | amp |
| TCGA-04-1348 | 5 | 56208779 | 57913403 | 1.179756061 | 5 | amp |
| TCGA-04-1348 | 5 | 57913463 | 60083252 | 1.731622122 | 6 | amp |
| TCGA-04-1348 | 5 | 60170415 | 64024125 | 1.38691136 | 5 | amp |
| TCGA-04-1348 | 5 | 64036835 | 64905297 | 1.691349749 | 6 | amp |
| TCGA-04-1348 | 5 | 64906658 | 65029502 | 1.338906078 | 5 | amp |
| TCGA-04-1348 | 5 | 65054343 | 65321403 | 1.77275154 | 6 | amp |
| TCGA-04-1348 | 5 | 65321710 | 82818147 | 1.245487159 | 5 | amp |
| TCGA-04-1348 | 5 | 82832781 | 86645225 | 1.706760429 | 6 | amp |
| TCGA-04-1348 | 5 | 86648952 | 89986915 | 1.402699807 | 5 | amp |
| TCGA-04-1348 | 5 | 89988392 | 90098747 | 1.749678621 | 6 | amp |
| TCGA-04-1348 | 5 | 90101067 | 96058432 | 1.202436546 | 5 | amp |
| TCGA-04-1348 | 5 | 96062470 | 96101862 | 1.7375931 | 6 | amp |
| TCGA-04-1348 | 5 | 96103103 | 98195783 | 1.440328379 | 5 | amp |
| TCGA-04-1348 | 5 | 98199080 | 101813592 | 1.62765526 | 6 | amp |
| TCGA-04-1348 | 5 | 101815829 | 112884744 | 1.425959713 | 5 | amp |
| TCGA-04-1348 | 5 | 112888966 | 113740583 | 1.834062732 | 6 | amp |
| TCGA-04-1348 | 5 | 113798696 | 116052262 | 1.150484572 | 5 | amp |
| TCGA-04-1348 | 5 | 118176576 | 118965558 | 2.011213749 | 6 | amp |
| TCGA-04-1348 | 5 | 118968392 | 127497541 | 1.279227381 | 5 | amp |
| TCGA-04-1348 | 5 | 127503445 | 127522375 | 2.001355083 | 6 | amp |
| TCGA-04-1348 | 5 | 127595063 | 131305916 | 1.291125173 | 5 | amp |
| TCGA-04-1348 | 5 | 131822451 | 132056413 | 1.405174432 | 5 | amp |
| TCGA-04-1348 | 5 | 132224727 | 132433035 | 1.219824931 | 4 | amp |
| TCGA-04-1348 | 5 | 132435230 | 137235435 | 0.904068544 | 3 | amp |
| TCGA-04-1348 | 5 | 137241888 | 137528137 | 1.2998676 | 5 | amp |

| | | | | | | |
|---|---|---|---|---|---|---|
| TCGA-04-1348 | 5 | 137528149 | 138261140 | 1.037952386 | 4 | amp |
| TCGA-04-1348 | 5 | 139752296 | 139828932 | 1.244223948 | 5 | amp |
| TCGA-04-1348 | 5 | 139838148 | 139887596 | 1.889455261 | 6 | amp |
| TCGA-04-1348 | 5 | 139889189 | 139917878 | 1.228189285 | 5 | amp |
| TCGA-04-1348 | 5 | 140903662 | 140963236 | 1.414078326 | 5 | amp |
| TCGA-04-1348 | 5 | 141511326 | 142421477 | 1.443950097 | 5 | amp |
| TCGA-04-1348 | 5 | 142433957 | 142680368 | 1.90656911 | 6 | amp |
| TCGA-04-1348 | 5 | 142689599 | 145519855 | 1.242111123 | 5 | amp |
| TCGA-04-1348 | 5 | 145522420 | 145533559 | 2.044318378 | 6 | amp |
| TCGA-04-1348 | 5 | 145536958 | 145843414 | 0.860923495 | 3 | amp |
| TCGA-04-1348 | 5 | 145847874 | 148980852 | 1.307421672 | 5 | amp |
| TCGA-04-1348 | 5 | 149226980 | 149361395 | 1.293985959 | 5 | amp |
| TCGA-04-1348 | 5 | 150097801 | 150282769 | 1.52771861 | 5 | amp |
| TCGA-04-1348 | 5 | 150483071 | 151054263 | 1.271785537 | 5 | amp |
| TCGA-04-1348 | 5 | 151055660 | 151183668 | 1.895594784 | 6 | amp |
| TCGA-04-1348 | 5 | 151202168 | 153182102 | 1.358640841 | 5 | amp |
| TCGA-04-1348 | 5 | 153190534 | 153409154 | 1.900158082 | 6 | amp |
| TCGA-04-1348 | 5 | 153413336 | 154244968 | 0.823215435 | 3 | amp |
| TCGA-04-1348 | 5 | 154250181 | 156668752 | 1.138280899 | 4 | amp |
| TCGA-04-1348 | 5 | 156670568 | 157178587 | 0.865230436 | 3 | amp |
| TCGA-04-1348 | 5 | 157180993 | 161300352 | 1.218323598 | 5 | amp |
| TCGA-04-1348 | 5 | 161302546 | 161580416 | 1.771694925 | 6 | amp |
| TCGA-04-1348 | 5 | 162866184 | 168222607 | 0.921943229 | 3 | amp |
| TCGA-04-1348 | 5 | 168233371 | 169021506 | 1.089148857 | 4 | amp |
| TCGA-04-1348 | 5 | 169021539 | 169111396 | 1.153601204 | 5 | amp |
| TCGA-04-1348 | 5 | 169116236 | 169454969 | 1.713568085 | 6 | amp |
| TCGA-04-1348 | 5 | 169461330 | 170338175 | 1.097391342 | 5 | amp |
| TCGA-04-1348 | 5 | 170341147 | 170669874 | 1.720164357 | 6 | amp |
| TCGA-04-1348 | 5 | 170692617 | 175779807 | 0.892990903 | 3 | amp |
| TCGA-04-1348 | 5 | 176618802 | 176722486 | 1.322794676 | 5 | amp |
| TCGA-04-1348 | 5 | 177054379 | 177469301 | 1.049841959 | 4 | amp |
| TCGA-04-1348 | 6 | 105907 | 2834283 | 1.075144662 | 4 | amp |
| TCGA-04-1348 | 6 | 2836053 | 3737491 | 0.73779191 | 3 | amp |
| TCGA-04-1348 | 6 | 3738276 | 7563068 | 1.377892486 | 5 | amp |
| TCGA-04-1348 | 6 | 7563933 | 7574521 | 1.941786124 | 6 | amp |
| TCGA-04-1348 | 6 | 7574838 | 8097719 | 1.294124079 | 5 | amp |
| TCGA-04-1348 | 6 | 8413736 | 9939587 | 1.738810337 | 6 | amp |
| TCGA-04-1348 | 6 | 10398613 | 10770523 | 0.879023066 | 3 | amp |
| TCGA-04-1348 | 6 | 10775476 | 10903012 | 1.228672201 | 5 | amp |
| TCGA-04-1348 | 6 | 10903091 | 10961821 | 2.106982204 | 6 | amp |
| TCGA-04-1348 | 6 | 10963936 | 11233745 | 1.183529535 | 5 | amp |
| TCGA-04-1348 | 6 | 11306170 | 12165422 | 1.84697307 | 6 | amp |
| TCGA-04-1348 | 6 | 12290834 | 13228231 | 1.405070103 | 5 | amp |
| TCGA-04-1348 | 6 | 13230226 | 13620738 | 0.873247954 | 3 | amp |
| TCGA-04-1348 | 6 | 13620962 | 13791128 | 2.249729311 | 6 | amp |
| TCGA-04-1348 | 6 | 13792106 | 17616429 | 1.215755259 | 5 | amp |
| TCGA-04-1348 | 6 | 17616721 | 17675298 | 1.857195209 | 6 | amp |
| TCGA-04-1348 | 6 | 17675409 | 17785928 | 1.391080196 | 5 | amp |
| TCGA-04-1348 | 6 | 17787996 | 17898464 | 1.796857091 | 6 | amp |
| TCGA-04-1348 | 6 | 17987269 | 18399967 | 1.474861912 | 5 | amp |

| | | | | | | |
|---|---|---|---|---|---|---|
| TCGA-04-1348 | 6 | 18406264 | 18465347 | 2.013230972 | 6 | amp |
| TCGA-04-1348 | 6 | 18571905 | 20548939 | 1.495183797 | 5 | amp |
| TCGA-04-1348 | 6 | 20649505 | 21108722 | 2.076339633 | 6 | amp |
| TCGA-04-1348 | 6 | 21198233 | 24178888 | 1.193477942 | 5 | amp |
| TCGA-04-1348 | 6 | 24205219 | 24302293 | 2.105533503 | 6 | amp |
| TCGA-04-1348 | 6 | 24353763 | 24520796 | 1.046633109 | 5 | amp |
| TCGA-04-1348 | 6 | 24522953 | 24589036 | 1.722281548 | 6 | amp |
| TCGA-04-1348 | 6 | 24596023 | 25285148 | 1.311720122 | 5 | amp |
| TCGA-04-1348 | 6 | 25420306 | 25581688 | 2.061105549 | 6 | amp |
| TCGA-04-1348 | 6 | 25594638 | 26017996 | 1.241968077 | 5 | amp |
| TCGA-04-1348 | 6 | 26020682 | 29571520 | 0.73869629 | 3 | amp |
| TCGA-04-1348 | 6 | 32260716 | 32307467 | 2.150459067 | 6 | amp |
| TCGA-04-1348 | 6 | 32317501 | 32906781 | 0.810483735 | 3 | amp |
| TCGA-04-1348 | 6 | 34511774 | 35199688 | 0.85163018 | 3 | amp |
| TCGA-04-1348 | 6 | 35480343 | 35480671 | 0.684352169 | 3 | amp |
| TCGA-04-1348 | 6 | 35543605 | 35996052 | 1.040261761 | 4 | amp |
| TCGA-04-1348 | 6 | 36020420 | 36063863 | 1.966323948 | 6 | amp |
| TCGA-04-1348 | 6 | 36067944 | 36099232 | 1.299212531 | 5 | amp |
| TCGA-04-1348 | 6 | 36100366 | 37631932 | 0.682294342 | 3 | amp |
| TCGA-04-1348 | 6 | 37897642 | 38645174 | 1.365184415 | 5 | amp |
| TCGA-04-1348 | 6 | 38649772 | 38854793 | 1.896525445 | 6 | amp |
| TCGA-04-1348 | 6 | 38858422 | 38942347 | 1.464858454 | 5 | amp |
| TCGA-04-1348 | 6 | 38950059 | 38980438 | 1.973578113 | 6 | amp |
| TCGA-04-1348 | 6 | 38994262 | 39353482 | 0.57767197 | 3 | amp |
| TCGA-04-1348 | 6 | 39387661 | 39693154 | 1.499462791 | 5 | amp |
| TCGA-04-1348 | 6 | 39828688 | 41065182 | 0.663391493 | 3 | amp |
| TCGA-04-1348 | 6 | 42541391 | 42585086 | 1.683692477 | 6 | amp |
| TCGA-04-1348 | 6 | 42600251 | 42620396 | 1.255000984 | 5 | amp |
| TCGA-04-1348 | 6 | 42623349 | 42638510 | 1.73376707 | 6 | amp |
| TCGA-04-1348 | 6 | 42641516 | 42830358 | 0.861077207 | 3 | amp |
| TCGA-04-1348 | 6 | 43492517 | 43592831 | 0.871485247 | 3 | amp |
| TCGA-04-1348 | 6 | 44310781 | 44336295 | 1.665879624 | 6 | amp |
| TCGA-04-1348 | 6 | 44337758 | 46678398 | 1.381408356 | 5 | amp |
| TCGA-04-1348 | 6 | 46679144 | 46794285 | 1.778870835 | 6 | amp |
| TCGA-04-1348 | 6 | 46797050 | 46852039 | 1.25493103 | 5 | amp |
| TCGA-04-1348 | 6 | 46856036 | 46993784 | 1.783999801 | 6 | amp |
| TCGA-04-1348 | 6 | 46995425 | 47471215 | 1.297870614 | 5 | amp |
| TCGA-04-1348 | 6 | 47501294 | 47592002 | 1.966039873 | 6 | amp |
| TCGA-04-1348 | 6 | 47641131 | 50811134 | 1.352579867 | 5 | amp |
| TCGA-04-1348 | 6 | 51274850 | 51701294 | 1.812606688 | 6 | amp |
| TCGA-04-1348 | 6 | 51712550 | 51923439 | 1.375474441 | 5 | amp |
| TCGA-04-1348 | 6 | 51924722 | 52052621 | 1.900407395 | 6 | amp |
| TCGA-04-1348 | 6 | 52053761 | 53883917 | 1.243320994 | 5 | amp |
| TCGA-04-1348 | 6 | 53986202 | 56047432 | 1.864407563 | 6 | amp |
| TCGA-04-1348 | 6 | 56323753 | 64413613 | 1.370466365 | 5 | amp |
| TCGA-04-1348 | 6 | 64415866 | 73951988 | 1.129181714 | 4 | amp |
| TCGA-04-1348 | 6 | 73952124 | 74472654 | 0.543140124 | 3 | amp |
| TCGA-04-1348 | 6 | 74473293 | 86259638 | 1.006567559 | 4 | amp |
| TCGA-04-1348 | 6 | 86267675 | 90415964 | 0.764773634 | 3 | amp |
| TCGA-04-1348 | 6 | 90417077 | 97720954 | 0.936095824 | 4 | amp |

| | | | | | | |
|---|---|---|---|---|---|---|
| TCGA-04-1348 | 6 | 97726659 | 100911376 | 0.756354939 | 3 | amp |
| TCGA-04-1348 | 6 | 100957185 | 105297128 | 1.078939183 | 4 | amp |
| TCGA-04-1348 | 6 | 105298736 | 109748387 | 0.774125155 | 3 | amp |
| TCGA-04-1348 | 6 | 109788821 | 111631358 | 0.827806911 | 3 | amp |
| TCGA-04-1348 | 6 | 111632216 | 137519831 | 0.991954246 | 4 | amp |
| TCGA-04-1348 | 6 | 137521961 | 139610064 | 0.701808156 | 3 | amp |
| TCGA-04-1348 | 6 | 139694225 | 147705930 | 1.032167148 | 4 | amp |
| TCGA-04-1348 | 6 | 147728408 | 149795706 | 0.682813152 | 3 | amp |
| TCGA-04-1348 | 6 | 149826534 | 149856922 | 1.854583447 | 6 | amp |
| TCGA-04-1348 | 6 | 149862016 | 149903777 | 1.236429112 | 5 | amp |
| TCGA-04-1348 | 6 | 149911828 | 152688554 | 0.755400316 | 3 | amp |
| TCGA-04-1348 | 6 | 152690034 | 153078313 | 1.001284075 | 4 | amp |
| TCGA-04-1348 | 6 | 153292243 | 160819122 | 0.690595686 | 3 | amp |
| TCGA-04-1348 | 6 | 160828029 | 161494666 | 1.036454593 | 4 | amp |
| TCGA-04-1348 | 6 | 161501871 | 168440940 | 0.718001634 | 3 | amp |
| TCGA-04-1348 | 7 | 5567330 | 6864379 | 0.6291473 | 3 | amp |
| TCGA-04-1348 | 7 | 6897253 | 21510730 | 1.288746661 | 5 | amp |
| TCGA-04-1348 | 7 | 21516690 | 23207675 | 1.839567009 | 6 | amp |
| TCGA-04-1348 | 7 | 23212555 | 25208115 | 1.189989867 | 5 | amp |
| TCGA-04-1348 | 7 | 25218740 | 27566031 | 0.866997681 | 4 | amp |
| TCGA-04-1348 | 7 | 27570768 | 30469091 | 1.165541143 | 5 | amp |
| TCGA-04-1348 | 7 | 30472693 | 31146323 | 0.581506952 | 3 | amp |
| TCGA-04-1348 | 7 | 31377848 | 37262318 | 1.390320871 | 5 | amp |
| TCGA-04-1348 | 7 | 37264409 | 37936702 | 1.97438398 | 6 | amp |
| TCGA-04-1348 | 7 | 37947053 | 43664458 | 1.338588755 | 5 | amp |
| TCGA-04-1348 | 7 | 43679140 | 43846884 | 1.088455383 | 4 | amp |
| TCGA-04-1348 | 7 | 43906259 | 43988343 | 0.707305217 | 3 | amp |
| TCGA-04-1348 | 7 | 45112293 | 47336865 | 0.60626083 | 3 | amp |
| TCGA-04-1348 | 7 | 47341766 | 47894867 | 0.887946945 | 4 | amp |
| TCGA-04-1348 | 7 | 47897196 | 48260951 | 1.235427913 | 5 | amp |
| TCGA-04-1348 | 7 | 48266820 | 50070916 | 1.60024015 | 6 | amp |
| TCGA-04-1348 | 7 | 50097540 | 66490067 | 0.810237557 | 3 | amp |
| TCGA-04-1348 | 7 | 66514874 | 66703525 | 1.807079473 | 6 | amp |
| TCGA-04-1348 | 7 | 66744713 | 72685718 | 0.699424867 | 3 | amp |
| TCGA-04-1348 | 7 | 74103421 | 74994083 | 0.711323239 | 3 | amp |
| TCGA-04-1348 | 7 | 76165471 | 76904003 | 0.85313777 | 3 | amp |
| TCGA-04-1348 | 7 | 76908052 | 87810929 | 1.628416456 | 6 | amp |
| TCGA-04-1348 | 7 | 87811247 | 92735425 | 1.319685392 | 5 | amp |
| TCGA-04-1348 | 7 | 92760476 | 93116327 | 1.95067038 | 6 | amp |
| TCGA-04-1348 | 7 | 93516109 | 94044619 | 1.058587704 | 5 | amp |
| TCGA-04-1348 | 7 | 94047029 | 94259211 | 1.885337161 | 6 | amp |
| TCGA-04-1348 | 7 | 94285295 | 96339097 | 1.301312567 | 5 | amp |
| TCGA-04-1348 | 7 | 96636926 | 97816369 | 0.727445413 | 3 | amp |
| TCGA-04-1348 | 7 | 99110024 | 99277543 | 0.736991816 | 3 | amp |
| TCGA-04-1348 | 7 | 99282793 | 99441875 | 1.971880808 | 6 | amp |
| TCGA-04-1348 | 7 | 99445077 | 99669840 | 0.840461835 | 3 | amp |
| TCGA-04-1348 | 7 | 99780253 | 99987739 | 0.719167338 | 3 | amp |
| TCGA-04-1348 | 7 | 99995428 | 100007228 | 1.776706378 | 6 | amp |
| TCGA-04-1348 | 7 | 102323665 | 102330919 | 0.834501177 | 3 | amp |
| TCGA-04-1348 | 7 | 102343839 | 102524769 | 1.791435521 | 6 | amp |

| | | | | | | |
|---|---|---|---|---|---|---|
| TCGA-04-1348 | 7 | 102553447 | 103032185 | 1.196398924 | 5 | amp |
| TCGA-04-1348 | 7 | 103033319 | 104909331 | 1.623253956 | 6 | amp |
| TCGA-04-1348 | 7 | 105098184 | 105667194 | 0.867060648 | 4 | amp |
| TCGA-04-1348 | 7 | 105668952 | 114582510 | 1.304776538 | 5 | amp |
| TCGA-04-1348 | 7 | 114619518 | 117307181 | 1.600622299 | 6 | amp |
| TCGA-04-1348 | 7 | 117351533 | 121653968 | 1.380985835 | 5 | amp |
| TCGA-04-1348 | 7 | 121659129 | 121769622 | 1.677243093 | 6 | amp |
| TCGA-04-1348 | 7 | 121773525 | 127031640 | 1.465074109 | 5 | amp |
| TCGA-04-1348 | 7 | 127222055 | 128457922 | 0.987736054 | 4 | amp |
| TCGA-04-1348 | 7 | 128470661 | 128607449 | 0.587003377 | 3 | amp |
| TCGA-04-1348 | 7 | 128610165 | 128658230 | 1.89003463 | 6 | amp |
| TCGA-04-1348 | 7 | 128694614 | 129122904 | 0.838747945 | 4 | amp |
| TCGA-04-1348 | 7 | 129125334 | 129906819 | 1.40114742 | 5 | amp |
| TCGA-04-1348 | 7 | 129908749 | 131012767 | 0.961740377 | 4 | amp |
| TCGA-04-1348 | 7 | 131060157 | 131163518 | 1.771917992 | 6 | amp |
| TCGA-04-1348 | 7 | 131172306 | 132470499 | 0.912253243 | 4 | amp |
| TCGA-04-1348 | 7 | 132481120 | 134650156 | 1.28307157 | 5 | amp |
| TCGA-04-1348 | 7 | 134653003 | 135048831 | 0.708299882 | 4 | amp |
| TCGA-04-1348 | 7 | 135069394 | 135277957 | 1.760250129 | 6 | amp |
| TCGA-04-1348 | 7 | 135279235 | 138305895 | 1.238520871 | 5 | amp |
| TCGA-04-1348 | 7 | 138310694 | 138774557 | 0.946428559 | 4 | amp |
| TCGA-04-1348 | 7 | 138818514 | 138954324 | 1.405507346 | 5 | amp |
| TCGA-04-1348 | 7 | 138957006 | 138982617 | 2.176786124 | 6 | amp |
| TCGA-04-1348 | 7 | 139026049 | 140487425 | 0.93190055 | 4 | amp |
| TCGA-04-1348 | 7 | 140494067 | 142224148 | 1.351348638 | 5 | amp |
| TCGA-04-1348 | 7 | 142224183 | 142448196 | 1.723501227 | 6 | amp |
| TCGA-04-1348 | 7 | 142448410 | 143573751 | 0.753509914 | 3 | amp |
| TCGA-04-1348 | 7 | 143632322 | 148484266 | 1.068727142 | 4 | amp |
| TCGA-04-1348 | 7 | 148485587 | 148975673 | 0.756020864 | 3 | amp |
| TCGA-04-1348 | 7 | 150938538 | 151164310 | 0.744265209 | 3 | amp |
| TCGA-04-1348 | 7 | 151167637 | 151875099 | 0.979567667 | 4 | amp |
| TCGA-04-1348 | 7 | 151876884 | 152517575 | 1.469766125 | 5 | amp |
| TCGA-04-1348 | 7 | 152520393 | 157049792 | 1.019575028 | 4 | amp |
| TCGA-04-1348 | 7 | 157060243 | 158935247 | 0.683106754 | 3 | amp |
| TCGA-04-1348 | 8 | 2112069 | 11282193 | 0.787739626 | 3 | amp |
| TCGA-04-1348 | 8 | 12293744 | 13251251 | 0.792120989 | 3 | amp |
| TCGA-04-1348 | 8 | 13258933 | 15480758 | 1.049738035 | 4 | amp |
| TCGA-04-1348 | 8 | 15508144 | 15531360 | 1.293570295 | 5 | amp |
| TCGA-04-1348 | 8 | 15588146 | 16021786 | 1.97547267 | 6 | amp |
| TCGA-04-1348 | 8 | 16025932 | 17137977 | 1.430923174 | 5 | amp |
| TCGA-04-1348 | 8 | 17141933 | 18666340 | 1.056388362 | 4 | amp |
| TCGA-04-1348 | 8 | 18725141 | 21843209 | 0.85084035 | 3 | amp |
| TCGA-04-1348 | 8 | 23428807 | 26252841 | 0.896316715 | 3 | amp |
| TCGA-04-1348 | 8 | 27593687 | 33311060 | 0.879421129 | 3 | amp |
| TCGA-04-1348 | 8 | 35383169 | 36793474 | 1.970105785 | 6 | amp |
| TCGA-04-1348 | 8 | 37731986 | 38865513 | 0.796245967 | 3 | amp |
| TCGA-04-1348 | 8 | 38869145 | 38899670 | 0.998858157 | 4 | amp |
| TCGA-04-1348 | 8 | 38911985 | 38959475 | 1.195899669 | 5 | amp |
| TCGA-04-1348 | 8 | 38961112 | 39564425 | 1.758973887 | 6 | amp |
| TCGA-04-1348 | 8 | 39581226 | 39679202 | 1.470168893 | 5 | amp |

| | | | | | | |
|---|---|---|---|---|---|---|
| TCGA-04-1348 | 8 | 39682316 | 40625264 | 0.987799765 | 4 | amp |
| TCGA-04-1348 | 8 | 41122666 | 41472026 | 0.798323426 | 3 | amp |
| TCGA-04-1348 | 8 | 41753815 | 49647741 | 0.837832538 | 3 | amp |
| TCGA-04-1348 | 8 | 49831336 | 53126849 | 1.385695248 | 5 | amp |
| TCGA-04-1348 | 8 | 53450974 | 53598053 | 1.987476487 | 6 | amp |
| TCGA-04-1348 | 8 | 54141822 | 54934713 | 1.286749584 | 5 | amp |
| TCGA-04-1348 | 8 | 54960591 | 59520423 | 1.000576899 | 4 | amp |
| TCGA-04-1348 | 8 | 59522107 | 62538895 | 1.342439092 | 5 | amp |
| TCGA-04-1348 | 8 | 62545164 | 63502372 | 1.825008639 | 6 | amp |
| TCGA-04-1348 | 8 | 63659469 | 68131880 | 1.269102252 | 5 | amp |
| TCGA-04-1348 | 8 | 68137082 | 69351930 | 1.755389885 | 6 | amp |
| TCGA-04-1348 | 8 | 69358479 | 80992738 | 1.304792602 | 5 | amp |
| TCGA-04-1348 | 8 | 81399804 | 88886205 | 1.091108525 | 4 | amp |
| TCGA-04-1348 | 8 | 89053645 | 95683909 | 1.341854942 | 5 | amp |
| TCGA-04-1348 | 8 | 95686514 | 97343348 | 1.045046234 | 4 | amp |
| TCGA-04-1348 | 8 | 97345649 | 103842166 | 1.31837327 | 5 | amp |
| TCGA-04-1348 | 8 | 103845214 | 110442273 | 1.653117518 | 6 | amp |
| TCGA-04-1348 | 8 | 110445329 | 110598399 | 1.379033898 | 5 | amp |
| TCGA-04-1348 | 8 | 110631049 | 118174171 | 1.78703503 | 6 | amp |
| TCGA-04-1348 | 8 | 118175656 | 120613759 | 1.369340334 | 5 | amp |
| TCGA-04-1348 | 8 | 120620129 | 122629455 | 2.028874679 | 6 | amp |
| TCGA-04-1348 | 8 | 122640906 | 124787582 | 1.274436115 | 5 | amp |
| TCGA-04-1348 | 8 | 124789473 | 124982365 | 1.8296591 | 6 | amp |
| TCGA-04-1348 | 8 | 124985652 | 126021619 | 1.280406287 | 5 | amp |
| TCGA-04-1348 | 8 | 126023766 | 126087356 | 1.730015525 | 6 | amp |
| TCGA-04-1348 | 8 | 126088545 | 131793179 | 1.169968493 | 5 | amp |
| TCGA-04-1348 | 8 | 131795933 | 133023149 | 1.752562605 | 6 | amp |
| TCGA-04-1348 | 8 | 133036695 | 133882082 | 1.389570528 | 5 | amp |
| TCGA-04-1348 | 8 | 133883574 | 141711175 | 0.99062743 | 4 | amp |
| TCGA-04-1348 | 8 | 141712618 | 141754868 | 1.456631981 | 5 | amp |
| TCGA-04-1348 | 8 | 141756818 | 141902766 | 1.785382191 | 6 | amp |
| TCGA-04-1348 | 9 | 16945 | 152116 | 0.879884506 | 4 | amp |
| TCGA-04-1348 | 9 | 154691 | 418257 | 1.423905255 | 5 | amp |
| TCGA-04-1348 | 9 | 420371 | 2104205 | 0.877743465 | 4 | amp |
| TCGA-04-1348 | 9 | 2110215 | 5557804 | 1.297031827 | 5 | amp |
| TCGA-04-1348 | 9 | 5563126 | 5805817 | 1.702700743 | 6 | amp |
| TCGA-04-1348 | 9 | 5810000 | 8523578 | 1.388967211 | 5 | amp |
| TCGA-04-1348 | 9 | 8524724 | 14120650 | 1.696336642 | 6 | amp |
| TCGA-04-1348 | 9 | 14125547 | 15695343 | 1.419483983 | 5 | amp |
| TCGA-04-1348 | 9 | 15721729 | 17795761 | 1.736627268 | 6 | amp |
| TCGA-04-1348 | 9 | 18474201 | 19550320 | 1.268690096 | 5 | amp |
| TCGA-04-1348 | 9 | 19573287 | 21202179 | 1.666092197 | 6 | amp |
| TCGA-04-1348 | 9 | 21206511 | 32988163 | 1.267613353 | 5 | amp |
| TCGA-04-1348 | 9 | 32989553 | 33369999 | 0.879761626 | 4 | amp |
| TCGA-04-1348 | 9 | 33384935 | 34513209 | 0.745739184 | 3 | amp |
| TCGA-04-1348 | 9 | 35227966 | 35381754 | 1.316694051 | 5 | amp |
| TCGA-04-1348 | 9 | 35382278 | 35555737 | 1.103193923 | 4 | amp |
| TCGA-04-1348 | 9 | 35971368 | 36677365 | 1.189352695 | 5 | amp |
| TCGA-04-1348 | 9 | 36840534 | 67945281 | 0.949348196 | 4 | amp |
| TCGA-04-1348 | 9 | 67947896 | 69440348 | 1.351731748 | 5 | amp |

| | | | | | | |
|---|---|---|---|---|---|---|
| TCGA-04-1348 | 9 | 69446639 | 70919342 | 0.929557255 | 4 | amp |
| TCGA-04-1348 | 9 | 70993075 | 75450984 | 1.332253541 | 5 | amp |
| TCGA-04-1348 | 9 | 75516099 | 77282821 | 1.783656351 | 6 | amp |
| TCGA-04-1348 | 9 | 77286700 | 78722276 | 1.380292396 | 5 | amp |
| TCGA-04-1348 | 9 | 78748986 | 79981835 | 1.807082095 | 6 | amp |
| TCGA-04-1348 | 9 | 79982951 | 88204639 | 1.285782839 | 5 | amp |
| TCGA-04-1348 | 9 | 88207426 | 88642829 | 1.662152785 | 6 | amp |
| TCGA-04-1348 | 9 | 88648193 | 94809561 | 0.874761666 | 4 | amp |
| TCGA-04-1348 | 9 | 94809882 | 95362827 | 1.345754382 | 5 | amp |
| TCGA-04-1348 | 9 | 95373575 | 98643602 | 0.65290525 | 3 | amp |
| TCGA-04-1348 | 9 | 98660071 | 98740539 | 1.845960206 | 6 | amp |
| TCGA-04-1348 | 9 | 98774455 | 100449580 | 0.963208455 | 4 | amp |
| TCGA-04-1348 | 9 | 100451808 | 101829376 | 0.700702625 | 3 | amp |
| TCGA-04-1348 | 9 | 101830834 | 101900375 | 1.805795723 | 6 | amp |
| TCGA-04-1348 | 9 | 101904781 | 111653703 | 1.32278023 | 5 | amp |
| TCGA-04-1348 | 9 | 111655224 | 111665955 | 2.141182981 | 6 | amp |
| TCGA-04-1348 | 9 | 111668553 | 113066839 | 1.310618018 | 5 | amp |
| TCGA-04-1348 | 9 | 113088397 | 114204704 | 1.733505833 | 6 | amp |
| TCGA-04-1348 | 9 | 114206655 | 114449159 | 1.129459673 | 5 | amp |
| TCGA-04-1348 | 9 | 114453770 | 115204074 | 1.83312472 | 6 | amp |
| TCGA-04-1348 | 9 | 115216247 | 115600997 | 1.375903324 | 5 | amp |
| TCGA-04-1348 | 9 | 115613121 | 116048600 | 0.966293783 | 4 | amp |
| TCGA-04-1348 | 9 | 116048926 | 116241855 | 0.666818518 | 3 | amp |
| TCGA-04-1348 | 9 | 117399219 | 119382775 | 1.315228365 | 5 | amp |
| TCGA-04-1348 | 9 | 119413759 | 123594226 | 1.028683182 | 4 | amp |
| TCGA-04-1348 | 9 | 123595511 | 123688403 | 0.456787588 | 3 | amp |
| TCGA-04-1348 | 9 | 123714931 | 123739200 | 1.972453814 | 6 | amp |
| TCGA-04-1348 | 9 | 123742326 | 123906390 | 1.346257261 | 5 | amp |
| TCGA-04-1348 | 9 | 123907028 | 123953040 | 1.702485601 | 6 | amp |
| TCGA-04-1348 | 9 | 123954415 | 125639865 | 0.683142734 | 3 | amp |
| TCGA-04-1348 | 9 | 125641972 | 125682300 | 0.949697031 | 4 | amp |
| TCGA-04-1348 | 9 | 125719293 | 126125485 | 1.329616872 | 5 | amp |
| TCGA-04-1348 | 9 | 126129393 | 126641324 | 0.871603908 | 4 | amp |
| TCGA-04-1348 | 9 | 126776220 | 130149660 | 0.712241033 | 3 | amp |
| TCGA-04-1348 | 9 | 131246977 | 131387479 | 0.762827472 | 3 | amp |
| TCGA-04-1348 | 9 | 131711434 | 131760528 | 0.89225632 | 3 | amp |
| TCGA-04-1348 | 9 | 134002858 | 134098360 | 1.043730655 | 3 | amp |
| TCGA-04-1348 | 9 | 134526146 | 135862175 | 0.763531279 | 3 | amp |
| TCGA-04-1348 | X | 1748652 | 12633008 | 0.814486929 | 3 | amp |
| TCGA-04-1348 | X | 12692944 | 12735259 | 1.76932024 | 6 | amp |
| TCGA-04-1348 | X | 12735574 | 15262809 | 1.253383672 | 5 | amp |
| TCGA-04-1348 | X | 15266796 | 15365503 | 0.938411813 | 4 | amp |
| TCGA-04-1348 | X | 15371188 | 15607582 | 1.560860464 | 6 | amp |
| TCGA-04-1348 | X | 15609757 | 18842242 | 1.283690397 | 5 | amp |
| TCGA-04-1348 | X | 18843850 | 19042106 | 0.809663701 | 3 | amp |
| TCGA-04-1348 | X | 19045274 | 38546946 | 1.334805971 | 5 | amp |
| TCGA-04-1348 | X | 38664175 | 40540205 | 0.875112962 | 3 | amp |
| TCGA-04-1348 | X | 40541089 | 46914034 | 1.294398547 | 5 | amp |
| TCGA-04-1348 | X | 46915422 | 46951262 | 0.947411895 | 3 | amp |
| TCGA-04-1348 | X | 47705633 | 48076938 | 0.847121415 | 3 | amp |

| | | | | | | |
|---|---|---|---|---|---|---|
| TCGA-04-1348 | X | 48116650 | 48244901 | 1.403201709 | 5 | amp |
| TCGA-04-1348 | X | 49369631 | 49777986 | 0.941212515 | 3 | amp |
| TCGA-04-1348 | X | 49779188 | 50370704 | 1.197852765 | 5 | amp |
| TCGA-04-1348 | X | 50376162 | 53571741 | 0.721287954 | 3 | amp |
| TCGA-04-1348 | X | 53571935 | 54467227 | 1.186662586 | 5 | amp |
| TCGA-04-1348 | X | 54469818 | 54985380 | 0.721395053 | 3 | amp |
| TCGA-04-1348 | X | 54986232 | 67885417 | 1.210218546 | 5 | amp |
| TCGA-04-1348 | X | 67932731 | 69424998 | 0.892328505 | 3 | amp |
| TCGA-04-1348 | X | 69454523 | 69460182 | 1.969005563 | 6 | amp |
| TCGA-04-1348 | X | 69478325 | 69510657 | 0.614606434 | 3 | amp |
| TCGA-04-1348 | X | 69516822 | 69626216 | 1.303580043 | 5 | amp |
| TCGA-04-1348 | X | 69626706 | 69715360 | 0.860792059 | 3 | amp |
| TCGA-04-1348 | X | 69716990 | 70149902 | 1.427459037 | 5 | amp |
| TCGA-04-1348 | X | 70183070 | 70471489 | 0.626284371 | 3 | amp |
| TCGA-04-1348 | X | 70472411 | 71925130 | 1.259729478 | 5 | amp |
| TCGA-04-1348 | X | 71932579 | 72783429 | 0.714077338 | 3 | amp |
| TCGA-04-1348 | X | 72797209 | 100534062 | 1.314600181 | 5 | amp |
| TCGA-04-1348 | X | 100536645 | 102977269 | 0.844862016 | 3 | amp |
| TCGA-04-1348 | X | 102978771 | 103499549 | 1.087365993 | 5 | amp |
| TCGA-04-1348 | X | 103903545 | 106117237 | 1.54587491 | 6 | amp |
| TCGA-04-1348 | X | 106144022 | 117786043 | 1.413079663 | 5 | amp |
| TCGA-04-1348 | X | 117788522 | 118750760 | 0.995362907 | 4 | amp |
| TCGA-04-1348 | X | 118752646 | 118979269 | 1.445622713 | 5 | amp |
| TCGA-04-1348 | X | 118985450 | 119513528 | 0.764101339 | 3 | amp |
| TCGA-04-1348 | X | 119562289 | 119691896 | 1.471534081 | 5 | amp |
| TCGA-04-1348 | X | 119693905 | 122319882 | 0.592527393 | 3 | amp |
| TCGA-04-1348 | X | 122387093 | 128645990 | 1.58627909 | 6 | amp |
| TCGA-04-1348 | X | 128649623 | 129306345 | 0.782688872 | 3 | amp |
| TCGA-04-1348 | X | 129318246 | 129370647 | 1.869374687 | 6 | amp |
| TCGA-04-1348 | X | 129373521 | 129843300 | 1.097642807 | 5 | amp |
| TCGA-04-1348 | X | 130215555 | 130438137 | 0.610771801 | 3 | amp |
| TCGA-04-1348 | X | 130678030 | 134715081 | 1.291261919 | 5 | amp |
| TCGA-04-1348 | X | 134715446 | 134992763 | 0.694657112 | 3 | amp |
| TCGA-04-1348 | X | 134993300 | 148564752 | 1.260681126 | 5 | amp |
| TCGA-04-1348 | X | 148568422 | 149678311 | 0.725859825 | 3 | amp |
| TCGA-04-1348 | X | 149761047 | 149818401 | 1.781727843 | 6 | amp |
| TCGA-04-1348 | X | 149826276 | 149919318 | 1.192220633 | 5 | amp |
| TCGA-04-1348 | X | 149919466 | 150773221 | 0.710085387 | 3 | amp |
| TCGA-04-1348 | X | 150780120 | 150844661 | 1.672624856 | 6 | amp |
| TCGA-04-1348 | X | 153880351 | 155254972 | 0.971446173 | 3 | amp |
| TCGA-04-1348 | Y | 13541157 | 28704298 | 1.088688153 | 3 | amp |
| TCGA-04-1349 | 1 | 16834 | 881085 | 1.026401019 | 3 | amp |
| TCGA-04-1349 | 1 | 1309171 | 1342403 | 1.424210927 | 4 | amp |
| TCGA-04-1349 | 1 | 1387719 | 10163197 | 0.936995304 | 3 | amp |
| TCGA-04-1349 | 1 | 10459663 | 12418705 | 0.938307111 | 3 | amp |
| TCGA-04-1349 | 1 | 12921054 | 15871105 | 0.910192859 | 3 | amp |
| TCGA-04-1349 | 1 | 15900092 | 16356574 | 0.901436364 | 3 | amp |
| TCGA-04-1349 | 1 | 16356912 | 16359767 | 1.688194952 | 4 | amp |
| TCGA-04-1349 | 1 | 16362731 | 17315012 | 0.928716864 | 3 | amp |
| TCGA-04-1349 | 1 | 17316165 | 17318683 | 2.237033543 | 5 | amp |

| | | | | | | |
|---|---|---|---|---|---|---|
| TCGA-04-1349 | 1 | 17318697 | 19428194 | 0.924677924 | 3 | amp |
| TCGA-04-1349 | 1 | 19559042 | 19582553 | 1.010360321 | 3 | amp |
| TCGA-04-1349 | 1 | 19583508 | 19593994 | 0.573693495 | 1 | loss |
| TCGA-04-1349 | 1 | 19595010 | 19612493 | 1.57621035 | 4 | amp |
| TCGA-04-1349 | 1 | 19612652 | 21042178 | 0.895684099 | 3 | amp |
| TCGA-04-1349 | 1 | 21099903 | 22307717 | 0.915000169 | 3 | amp |
| TCGA-04-1349 | 1 | 23735127 | 25570142 | 0.951766893 | 3 | amp |
| TCGA-04-1349 | 1 | 26131549 | 26515473 | 0.915107452 | 3 | amp |
| TCGA-04-1349 | 1 | 26528946 | 26898437 | 0.938759878 | 3 | amp |
| TCGA-04-1349 | 1 | 27100785 | 27721338 | 0.971836342 | 3 | amp |
| TCGA-04-1349 | 1 | 27873772 | 28598403 | 0.93916141 | 3 | amp |
| TCGA-04-1349 | 1 | 31762063 | 32267375 | 0.966106261 | 3 | amp |
| TCGA-04-1349 | 1 | 32669412 | 32682965 | 1.261096077 | 4 | amp |
| TCGA-04-1349 | 1 | 32686674 | 34258135 | 0.914751126 | 3 | amp |
| TCGA-04-1349 | 1 | 35350561 | 35827424 | 1.009982935 | 3 | amp |
| TCGA-04-1349 | 1 | 36215039 | 37962382 | 0.909866193 | 3 | amp |
| TCGA-04-1349 | 1 | 37967350 | 38018356 | 1.27985467 | 4 | amp |
| TCGA-04-1349 | 1 | 38022452 | 39748082 | 0.939818824 | 3 | amp |
| TCGA-04-1349 | 1 | 39909061 | 39920794 | 0.491988823 | 1 | loss |
| TCGA-04-1349 | 1 | 39950217 | 46812789 | 0.913416593 | 3 | amp |
| TCGA-04-1349 | 1 | 47014841 | 47279335 | 1.010552158 | 3 | amp |
| TCGA-04-1349 | 1 | 48771444 | 52850427 | 0.908435366 | 3 | amp |
| TCGA-04-1349 | 1 | 52933805 | 65124591 | 0.914114601 | 3 | amp |
| TCGA-04-1349 | 1 | 65684402 | 75609641 | 0.925045809 | 3 | amp |
| TCGA-04-1349 | 1 | 75614259 | 75684485 | 1.214662294 | 4 | amp |
| TCGA-04-1349 | 1 | 75684925 | 78390953 | 0.928380895 | 3 | amp |
| TCGA-04-1349 | 1 | 78591545 | 92163726 | 0.937871001 | 3 | amp |
| TCGA-04-1349 | 1 | 92430132 | 100591527 | 0.89290512 | 3 | amp |
| TCGA-04-1349 | 1 | 100633955 | 109265255 | 0.944162619 | 3 | amp |
| TCGA-04-1349 | 1 | 109607109 | 109836925 | 0.902360996 | 3 | amp |
| TCGA-04-1349 | 1 | 109909976 | 113636231 | 0.939277949 | 3 | amp |
| TCGA-04-1349 | 1 | 114281326 | 114499460 | 0.99187181 | 3 | amp |
| TCGA-04-1349 | 1 | 114964007 | 117492243 | 0.904862739 | 3 | amp |
| TCGA-04-1349 | 1 | 117602892 | 145706781 | 0.982375028 | 3 | amp |
| TCGA-04-1349 | 1 | 145939808 | 146215375 | 1.046358216 | 3 | amp |
| TCGA-04-1349 | 1 | 146215842 | 146232708 | 1.191849526 | 4 | amp |
| TCGA-04-1349 | 1 | 146233255 | 146420225 | 0.949564652 | 3 | amp |
| TCGA-04-1349 | 1 | 146420876 | 146516439 | 1.139354003 | 4 | amp |
| TCGA-04-1349 | 1 | 146528057 | 146661851 | 0.982009418 | 3 | amp |
| TCGA-04-1349 | 1 | 147087562 | 147120250 | 0.475850755 | 1 | loss |
| TCGA-04-1349 | 1 | 147131705 | 147908612 | 0.97178673 | 3 | amp |
| TCGA-04-1349 | 1 | 147954796 | 148890319 | 1.105816061 | 4 | amp |
| TCGA-04-1349 | 1 | 148891515 | 149900835 | 1.001182491 | 3 | amp |
| TCGA-04-1349 | 1 | 150039870 | 150234067 | 0.935484798 | 3 | amp |
| TCGA-04-1349 | 1 | 150234477 | 150246591 | 1.279677557 | 4 | amp |
| TCGA-04-1349 | 1 | 150248118 | 150318672 | 0.958616235 | 3 | amp |
| TCGA-04-1349 | 1 | 150525859 | 151090762 | 0.914850642 | 3 | amp |
| TCGA-04-1349 | 1 | 151106838 | 151112205 | 1.223095889 | 4 | amp |
| TCGA-04-1349 | 1 | 151112418 | 151298711 | 0.875129795 | 3 | amp |
| TCGA-04-1349 | 1 | 151397423 | 152800292 | 0.986082463 | 3 | amp |

| | | | | | | |
|---|---|---|---|---|---|---|
| TCGA-04-1349 | 1 | 153177121 | 153633786 | 0.946353895 | 3 | amp |
| TCGA-04-1349 | 1 | 153634741 | 153641040 | 1.17714972 | 4 | amp |
| TCGA-04-1349 | 1 | 153642242 | 154116107 | 0.927803218 | 3 | amp |
| TCGA-04-1349 | 1 | 154284899 | 155786087 | 0.933590037 | 3 | amp |
| TCGA-04-1349 | 1 | 156011248 | 156699011 | 0.943688365 | 3 | amp |
| TCGA-04-1349 | 1 | 156701724 | 156707377 | 1.603986978 | 4 | amp |
| TCGA-04-1349 | 1 | 156707382 | 156924758 | 0.935764819 | 3 | amp |
| TCGA-04-1349 | 1 | 156946720 | 159772289 | 0.942991013 | 3 | amp |
| TCGA-04-1349 | 1 | 159804921 | 160090837 | 0.92794458 | 3 | amp |
| TCGA-04-1349 | 1 | 160156384 | 160188818 | 0.97368508 | 3 | amp |
| TCGA-04-1349 | 1 | 160302232 | 160794064 | 0.968873668 | 3 | amp |
| TCGA-04-1349 | 1 | 160922406 | 160971168 | 0.95322829 | 3 | amp |
| TCGA-04-1349 | 1 | 160990771 | 161126833 | 1.251763033 | 4 | amp |
| TCGA-04-1349 | 1 | 161127015 | 161184024 | 1.03700001 | 3 | amp |
| TCGA-04-1349 | 1 | 161487724 | 176833718 | 0.913836678 | 3 | amp |
| TCGA-04-1349 | 1 | 176927429 | 182848584 | 0.906245971 | 3 | amp |
| TCGA-04-1349 | 1 | 183599563 | 197020037 | 0.9401587 | 3 | amp |
| TCGA-04-1349 | 1 | 197021713 | 197061231 | 1.158998699 | 4 | amp |
| TCGA-04-1349 | 1 | 197062166 | 198691621 | 0.955077164 | 3 | amp |
| TCGA-04-1349 | 1 | 198697432 | 198723585 | 1.168506417 | 4 | amp |
| TCGA-04-1349 | 1 | 198724962 | 201687940 | 0.902137668 | 3 | amp |
| TCGA-04-1349 | 1 | 201749467 | 201758953 | 0.40421562 | 1 | loss |
| TCGA-04-1349 | 1 | 201782206 | 202509378 | 0.891626564 | 3 | amp |
| TCGA-04-1349 | 1 | 202527997 | 202536992 | 0.418228889 | 1 | loss |
| TCGA-04-1349 | 1 | 202538239 | 207533001 | 0.899294619 | 3 | amp |
| TCGA-04-1349 | 1 | 207639823 | 207648604 | 1.368575023 | 4 | amp |
| TCGA-04-1349 | 1 | 207649551 | 211307307 | 0.897805938 | 3 | amp |
| TCGA-04-1349 | 1 | 211529699 | 215753356 | 0.924786762 | 3 | amp |
| TCGA-04-1349 | 1 | 215813837 | 220970185 | 0.912610872 | 3 | amp |
| TCGA-04-1349 | 1 | 222860264 | 224424310 | 0.885751398 | 3 | amp |
| TCGA-04-1349 | 1 | 226026318 | 228598881 | 0.895313846 | 3 | amp |
| TCGA-04-1349 | 1 | 229567167 | 230898585 | 0.905356575 | 3 | amp |
| TCGA-04-1349 | 1 | 231361182 | 236332090 | 0.893951121 | 3 | amp |
| TCGA-04-1349 | 1 | 236749030 | 245247016 | 0.922841563 | 3 | amp |
| TCGA-04-1349 | 1 | 246711817 | 249231325 | 0.928671021 | 3 | amp |
| TCGA-04-1349 | 10 | 92880 | 298468 | 1.060219776 | 3 | amp |
| TCGA-04-1349 | 10 | 323180 | 6274941 | 1.190590103 | 4 | amp |
| TCGA-04-1349 | 10 | 6470096 | 7786263 | 1.114622549 | 3 | amp |
| TCGA-04-1349 | 10 | 7786719 | 11963120 | 1.196083236 | 4 | amp |
| TCGA-04-1349 | 10 | 11963211 | 12126764 | 0.958574951 | 3 | amp |
| TCGA-04-1349 | 10 | 12148199 | 12197378 | 1.106314778 | 3 | amp |
| TCGA-04-1349 | 10 | 12208672 | 13240867 | 1.104153357 | 3 | amp |
| TCGA-04-1349 | 10 | 13243417 | 13779946 | 1.182517575 | 4 | amp |
| TCGA-04-1349 | 10 | 13782132 | 14816696 | 0.997945015 | 3 | amp |
| TCGA-04-1349 | 10 | 14861917 | 15161546 | 1.191846952 | 4 | amp |
| TCGA-04-1349 | 10 | 15183342 | 15858933 | 1.2541581 | 4 | amp |
| TCGA-04-1349 | 10 | 15863599 | 17838511 | 1.089664926 | 3 | amp |
| TCGA-04-1349 | 10 | 17840563 | 18629940 | 1.200078758 | 4 | amp |
| TCGA-04-1349 | 10 | 18689934 | 18835087 | 0.993774316 | 3 | amp |
| TCGA-04-1349 | 10 | 18836983 | 22657635 | 1.197938836 | 4 | amp |

| | | | | | | |
|---|---|---|---|---|---|---|
| TCGA-04-1349 | 10 | 22675665 | 23295921 | 1.045115888 | 3 | amp |
| TCGA-04-1349 | 10 | 23297193 | 24822202 | 1.239475906 | 4 | amp |
| TCGA-04-1349 | 10 | 24825642 | 24924045 | 1.046672589 | 3 | amp |
| TCGA-04-1349 | 10 | 24955865 | 28276472 | 1.214590689 | 4 | amp |
| TCGA-04-1349 | 10 | 28283839 | 32197801 | 1.051884668 | 3 | amp |
| TCGA-04-1349 | 10 | 32310094 | 33000662 | 1.050439548 | 3 | amp |
| TCGA-04-1349 | 10 | 33015650 | 33197498 | 1.268234445 | 4 | amp |
| TCGA-04-1349 | 10 | 33199146 | 35819231 | 1.08481242 | 3 | amp |
| TCGA-04-1349 | 10 | 35841909 | 46240359 | 1.218805494 | 4 | amp |
| TCGA-04-1349 | 10 | 46242011 | 46286012 | 0.961264801 | 3 | amp |
| TCGA-04-1349 | 10 | 46287658 | 47900057 | 1.224460876 | 4 | amp |
| TCGA-04-1349 | 10 | 47901351 | 47947017 | 1.044181301 | 3 | amp |
| TCGA-04-1349 | 10 | 47948663 | 49431359 | 1.222986744 | 4 | amp |
| TCGA-04-1349 | 10 | 49440124 | 50701350 | 1.10012202 | 3 | amp |
| TCGA-04-1349 | 10 | 50708542 | 51767870 | 1.200966916 | 4 | amp |
| TCGA-04-1349 | 10 | 51768428 | 51892755 | 1.007871363 | 3 | amp |
| TCGA-04-1349 | 10 | 51947049 | 60588705 | 1.230924718 | 4 | amp |
| TCGA-04-1349 | 10 | 60896487 | 61973278 | 1.097994282 | 3 | amp |
| TCGA-04-1349 | 10 | 61994434 | 64928380 | 1.191920969 | 4 | amp |
| TCGA-04-1349 | 10 | 64950640 | 65357843 | 1.032692608 | 3 | amp |
| TCGA-04-1349 | 10 | 65358926 | 69556980 | 1.216151119 | 4 | amp |
| TCGA-04-1349 | 10 | 69565292 | 70276613 | 1.116370428 | 3 | amp |
| TCGA-04-1349 | 10 | 70426787 | 70548112 | 1.019610584 | 3 | amp |
| TCGA-04-1349 | 10 | 70549449 | 70955042 | 1.217305474 | 4 | amp |
| TCGA-04-1349 | 10 | 70956733 | 71871476 | 1.091906123 | 3 | amp |
| TCGA-04-1349 | 10 | 71873859 | 74103316 | 1.16635363 | 4 | amp |
| TCGA-04-1349 | 10 | 74104646 | 75290330 | 1.082851869 | 3 | amp |
| TCGA-04-1349 | 10 | 75305212 | 75397710 | 0.975537627 | 3 | amp |
| TCGA-04-1349 | 10 | 75399678 | 75551289 | 1.267233805 | 4 | amp |
| TCGA-04-1349 | 10 | 75551573 | 75562844 | 1.02670252 | 3 | amp |
| TCGA-04-1349 | 10 | 75562881 | 75620677 | 1.238402692 | 4 | amp |
| TCGA-04-1349 | 10 | 75632699 | 76936037 | 1.041452263 | 3 | amp |
| TCGA-04-1349 | 10 | 76971961 | 77795948 | 1.344367869 | 4 | amp |
| TCGA-04-1349 | 10 | 77806871 | 79493777 | 1.030766499 | 3 | amp |
| TCGA-04-1349 | 10 | 79552159 | 79628980 | 1.214565223 | 4 | amp |
| TCGA-04-1349 | 10 | 79737189 | 79773597 | 1.004598623 | 3 | amp |
| TCGA-04-1349 | 10 | 79777283 | 81272877 | 1.199373271 | 4 | amp |
| TCGA-04-1349 | 10 | 81316912 | 81702696 | 1.02306749 | 3 | amp |
| TCGA-04-1349 | 10 | 81706195 | 86001254 | 1.165126015 | 4 | amp |
| TCGA-04-1349 | 10 | 90342768 | 90497584 | 1.05587906 | 3 | amp |
| TCGA-04-1349 | 10 | 90499728 | 92672737 | 1.227479465 | 4 | amp |
| TCGA-04-1349 | 10 | 92675288 | 94274857 | 1.099996081 | 3 | amp |
| TCGA-04-1349 | 10 | 94291469 | 95141184 | 1.189671834 | 4 | amp |
| TCGA-04-1349 | 10 | 95161956 | 95241967 | 1.064755897 | 3 | amp |
| TCGA-04-1349 | 10 | 95259779 | 96829195 | 1.22014549 | 4 | amp |
| TCGA-04-1349 | 10 | 96966979 | 97769696 | 1.068426416 | 3 | amp |
| TCGA-04-1349 | 10 | 97772267 | 98136632 | 1.296553608 | 4 | amp |
| TCGA-04-1349 | 10 | 98138636 | 98408633 | 1.051489189 | 3 | amp |
| TCGA-04-1349 | 10 | 98410950 | 100022817 | 1.203145921 | 4 | amp |
| TCGA-04-1349 | 10 | 100143499 | 100155079 | 0.936804477 | 3 | amp |

| | | | | | | |
|---|---|---|---|---|---|---|
| TCGA-04-1349 | 10 | 100155088 | 103904095 | 1.214464394 | 4 | amp |
| TCGA-04-1349 | 10 | 103908860 | 104140472 | 1.124781387 | 3 | amp |
| TCGA-04-1349 | 10 | 104140797 | 104486948 | 1.233264593 | 4 | amp |
| TCGA-04-1349 | 10 | 104488146 | 104595199 | 1.049669301 | 3 | amp |
| TCGA-04-1349 | 10 | 104596759 | 105377132 | 1.209457699 | 4 | amp |
| TCGA-04-1349 | 10 | 105386842 | 105798904 | 1.07036407 | 3 | amp |
| TCGA-04-1349 | 10 | 105799179 | 105912544 | 1.289791488 | 4 | amp |
| TCGA-04-1349 | 10 | 105920737 | 105932350 | 1.046371521 | 3 | amp |
| TCGA-04-1349 | 10 | 105951948 | 111629808 | 1.313700808 | 4 | amp |
| TCGA-04-1349 | 10 | 111630425 | 111646111 | 0.938176923 | 3 | amp |
| TCGA-04-1349 | 10 | 111647758 | 114575155 | 1.203562754 | 4 | amp |
| TCGA-04-1349 | 10 | 114724288 | 115417340 | 1.085773176 | 3 | amp |
| TCGA-04-1349 | 10 | 115422421 | 116100645 | 1.227254785 | 4 | amp |
| TCGA-04-1349 | 10 | 116196015 | 116444140 | 1.02836024 | 3 | amp |
| TCGA-04-1349 | 10 | 116590589 | 118427855 | 1.21717551 | 4 | amp |
| TCGA-04-1349 | 10 | 118434250 | 119100671 | 1.061034836 | 3 | amp |
| TCGA-04-1349 | 10 | 119302957 | 120796845 | 1.263811937 | 4 | amp |
| TCGA-04-1349 | 10 | 120797851 | 120907389 | 1.05791645 | 3 | amp |
| TCGA-04-1349 | 10 | 120914571 | 122665580 | 1.242567475 | 4 | amp |
| TCGA-04-1349 | 10 | 122666267 | 124380914 | 1.084132324 | 3 | amp |
| TCGA-04-1349 | 10 | 124384745 | 125447680 | 1.22086089 | 4 | amp |
| TCGA-04-1349 | 10 | 125506226 | 125622244 | 0.985473864 | 3 | amp |
| TCGA-04-1349 | 10 | 125639685 | 127519249 | 1.225807935 | 4 | amp |
| TCGA-04-1349 | 10 | 127519962 | 127555775 | 1.026490341 | 3 | amp |
| TCGA-04-1349 | 10 | 127569072 | 135516111 | 1.236131817 | 4 | amp |
| TCGA-04-1349 | 11 | 26463411 | 134257557 | 0.976364228 | 3 | amp |
| TCGA-04-1349 | 12 | 73256 | 6680236 | 0.913377216 | 3 | amp |
| TCGA-04-1349 | 12 | 6704457 | 8673913 | 0.911394118 | 3 | amp |
| TCGA-04-1349 | 12 | 9087716 | 9304293 | 0.962388455 | 3 | amp |
| TCGA-04-1349 | 12 | 9446201 | 10602599 | 0.922557633 | 3 | amp |
| TCGA-04-1349 | 12 | 15835795 | 21712674 | 0.906675276 | 3 | amp |
| TCGA-04-1349 | 12 | 26839369 | 27175593 | 0.940442347 | 3 | amp |
| TCGA-04-1349 | 12 | 27844651 | 34179863 | 0.898710412 | 3 | amp |
| TCGA-04-1349 | 12 | 38710630 | 49960569 | 1.208773055 | 4 | amp |
| TCGA-04-1349 | 12 | 49981311 | 50060009 | 0.958840925 | 3 | amp |
| TCGA-04-1349 | 12 | 50062205 | 50535924 | 1.140672891 | 4 | amp |
| TCGA-04-1349 | 12 | 50536771 | 50822915 | 0.92672261 | 3 | amp |
| TCGA-04-1349 | 12 | 50824225 | 53097246 | 1.146689245 | 4 | amp |
| TCGA-04-1349 | 12 | 53162422 | 53240088 | 1.063979839 | 3 | amp |
| TCGA-04-1349 | 12 | 53240517 | 53670703 | 1.212993446 | 4 | amp |
| TCGA-04-1349 | 12 | 53670818 | 53865626 | 0.981072263 | 3 | amp |
| TCGA-04-1349 | 12 | 53873147 | 54678129 | 1.167851626 | 4 | amp |
| TCGA-04-1349 | 12 | 54756409 | 56740004 | 1.205012485 | 4 | amp |
| TCGA-04-1349 | 12 | 56740221 | 56750112 | 0.959279566 | 3 | amp |
| TCGA-04-1349 | 12 | 56750139 | 56963788 | 1.333755057 | 4 | amp |
| TCGA-04-1349 | 12 | 56965440 | 56999163 | 0.987634771 | 3 | amp |
| TCGA-04-1349 | 12 | 56999560 | 57409584 | 1.222069118 | 4 | amp |
| TCGA-04-1349 | 12 | 57422481 | 57592470 | 1.01881749 | 3 | amp |
| TCGA-04-1349 | 12 | 57592938 | 57944238 | 1.296276514 | 4 | amp |
| TCGA-04-1349 | 12 | 57957205 | 57972132 | 0.860770624 | 3 | amp |

| | | | | | | |
|---|---|---|---|---|---|---|
| TCGA-04-1349 | 12 | 57974696 | 58165968 | 1.337554892 | 4 | amp |
| TCGA-04-1349 | 12 | 58166445 | 64890226 | 0.965963961 | 3 | amp |
| TCGA-04-1349 | 12 | 66221763 | 93221882 | 0.946812299 | 3 | amp |
| TCGA-04-1349 | 12 | 96266015 | 101684708 | 0.972371666 | 3 | amp |
| TCGA-04-1349 | 12 | 101746805 | 104333464 | 0.921397642 | 3 | amp |
| TCGA-04-1349 | 12 | 104335106 | 104341319 | 1.384230378 | 4 | amp |
| TCGA-04-1349 | 12 | 104341365 | 110734592 | 0.922706309 | 3 | amp |
| TCGA-04-1349 | 12 | 110815189 | 112306670 | 0.942866889 | 3 | amp |
| TCGA-04-1349 | 12 | 112939943 | 118469094 | 0.937617942 | 3 | amp |
| TCGA-04-1349 | 12 | 118671434 | 120168487 | 0.943055423 | 3 | amp |
| TCGA-04-1349 | 12 | 120616374 | 121097755 | 0.940581093 | 3 | amp |
| TCGA-04-1349 | 12 | 121097950 | 121165030 | 1.264808724 | 4 | amp |
| TCGA-04-1349 | 12 | 121175154 | 133779395 | 0.947323772 | 3 | amp |
| TCGA-04-1349 | 13 | 114549471 | 114774961 | 4.311577867 | 5 | amp |
| TCGA-04-1349 | 14 | 19377543 | 107283263 | 0.930697828 | 3 | amp |
| TCGA-04-1349 | 15 | 20169886 | 20641293 | 1.166379007 | 4 | amp |
| TCGA-04-1349 | 15 | 20642495 | 20776199 | 1.067475248 | 3 | amp |
| TCGA-04-1349 | 15 | 20777258 | 22567092 | 1.333476714 | 4 | amp |
| TCGA-04-1349 | 15 | 22691011 | 34655069 | 1.058047037 | 3 | amp |
| TCGA-04-1349 | 15 | 34655549 | 34823777 | 1.342934923 | 4 | amp |
| TCGA-04-1349 | 15 | 34823812 | 41769744 | 1.052084874 | 3 | amp |
| TCGA-04-1349 | 15 | 41770694 | 41815557 | 1.320601962 | 4 | amp |
| TCGA-04-1349 | 15 | 41815949 | 42139069 | 1.026253369 | 3 | amp |
| TCGA-04-1349 | 15 | 42139477 | 42145670 | 1.301325919 | 4 | amp |
| TCGA-04-1349 | 15 | 42146956 | 42694043 | 1.015016225 | 3 | amp |
| TCGA-04-1349 | 15 | 42730777 | 42807613 | 1.032032195 | 3 | amp |
| TCGA-04-1349 | 15 | 42820418 | 43045468 | 1.290748615 | 4 | amp |
| TCGA-04-1349 | 15 | 43067296 | 43170846 | 0.918845784 | 3 | amp |
| TCGA-04-1349 | 15 | 43237477 | 43398239 | 1.330441076 | 4 | amp |
| TCGA-04-1349 | 15 | 43426450 | 43502644 | 1.821380973 | 5 | amp |
| TCGA-04-1349 | 15 | 43503552 | 43873625 | 0.977930165 | 3 | amp |
| TCGA-04-1349 | 15 | 43874041 | 43940306 | 1.259732457 | 4 | amp |
| TCGA-04-1349 | 15 | 43955822 | 43970898 | 0.933440097 | 3 | amp |
| TCGA-04-1349 | 15 | 43972975 | 44067002 | 1.256713556 | 4 | amp |
| TCGA-04-1349 | 15 | 44067451 | 45962212 | 1.04498794 | 3 | amp |
| TCGA-04-1349 | 15 | 45965751 | 48063997 | 1.264321696 | 4 | amp |
| TCGA-04-1349 | 15 | 48413181 | 52342381 | 1.016399788 | 3 | amp |
| TCGA-04-1349 | 15 | 52350791 | 53957986 | 1.275236652 | 4 | amp |
| TCGA-04-1349 | 15 | 53991924 | 78783151 | 1.011378576 | 3 | amp |
| TCGA-04-1349 | 15 | 78786204 | 78841328 | 1.343260596 | 4 | amp |
| TCGA-04-1349 | 15 | 78873108 | 79265823 | 1.035364544 | 3 | amp |
| TCGA-04-1349 | 16 | 46702782 | 90244214 | 0.44567534 | 1 | loss |
| TCGA-04-1349 | 17 | 1248706 | 1268372 | 0.340283731 | 1 | loss |
| TCGA-04-1349 | 17 | 2224509 | 2236456 | 1.052041933 | 3 | amp |
| TCGA-04-1349 | 17 | 3917604 | 3999318 | 0.491388939 | 1 | loss |
| TCGA-04-1349 | 17 | 4463596 | 4535104 | 0.376250182 | 1 | loss |
| TCGA-04-1349 | 17 | 5331340 | 5353744 | 0.973214315 | 3 | amp |
| TCGA-04-1349 | 17 | 6978336 | 6982157 | 0.360432596 | 1 | loss |
| TCGA-04-1349 | 17 | 7106173 | 7195421 | 0.965921574 | 3 | amp |
| TCGA-04-1349 | 17 | 7801204 | 7806436 | 0.944659589 | 3 | amp |

| | | | | | | |
|---|---|---|---|---|---|---|
| TCGA-04-1349 | 17 | 8404095 | 8424626 | 0.44768407 | 1 | loss |
| TCGA-04-1349 | 17 | 10531892 | 10543975 | 0.474377338 | 1 | loss |
| TCGA-04-1349 | 17 | 18634345 | 18670221 | 0.466129276 | 1 | loss |
| TCGA-04-1349 | 17 | 18785848 | 18834051 | 0.383248502 | 1 | loss |
| TCGA-04-1349 | 17 | 19643569 | 19689444 | 0.898199481 | 3 | amp |
| TCGA-04-1349 | 17 | 26881165 | 26925998 | 0.975338644 | 3 | amp |
| TCGA-04-1349 | 17 | 27022368 | 27030894 | 0.467018112 | 1 | loss |
| TCGA-04-1349 | 17 | 27030971 | 27086450 | 0.96610787 | 3 | amp |
| TCGA-04-1349 | 17 | 34146993 | 34266410 | 0.472571071 | 1 | loss |
| TCGA-04-1349 | 17 | 35580387 | 35614826 | 0.490525873 | 1 | loss |
| TCGA-04-1349 | 17 | 37864530 | 37876123 | 0.948310608 | 3 | amp |
| TCGA-04-1349 | 17 | 38412652 | 38450314 | 0.461661112 | 1 | loss |
| TCGA-04-1349 | 17 | 38569435 | 38637045 | 0.489002849 | 1 | loss |
| TCGA-04-1349 | 17 | 38854480 | 38859997 | 0.371716799 | 1 | loss |
| TCGA-04-1349 | 17 | 39382868 | 39412202 | 0.289046055 | 1 | loss |
| TCGA-04-1349 | 17 | 39658585 | 39675127 | 0.419391624 | 1 | loss |
| TCGA-04-1349 | 17 | 42929703 | 42941206 | 0.474763336 | 1 | loss |
| TCGA-04-1349 | 17 | 43587590 | 43627717 | 0.379823536 | 1 | loss |
| TCGA-04-1349 | 17 | 44382850 | 44435809 | 0.951572157 | 3 | amp |
| TCGA-04-1349 | 17 | 44600425 | 44653734 | 0.935506061 | 3 | amp |
| TCGA-04-1349 | 17 | 73490678 | 73554336 | 1.012994344 | 3 | amp |
| TCGA-04-1349 | 17 | 74763425 | 74774471 | 0.44849507 | 1 | loss |
| TCGA-04-1349 | 18 | 47273 | 74536397 | 1.043674911 | 3 | amp |
| TCGA-04-1349 | 19 | 9577229 | 10166472 | 1.19252828 | 4 | amp |
| TCGA-04-1349 | 19 | 10169198 | 10257233 | 2.6974055 | 5 | amp |
| TCGA-04-1349 | 19 | 10259506 | 10271136 | 1.708009725 | 4 | amp |
| TCGA-04-1349 | 19 | 10273323 | 10870510 | 3.003858511 | 5 | amp |
| TCGA-04-1349 | 19 | 10883079 | 12867151 | 1.264286513 | 4 | amp |
| TCGA-04-1349 | 19 | 12874020 | 13566033 | 2.771437524 | 5 | amp |
| TCGA-04-1349 | 19 | 13862563 | 15508443 | 1.655281127 | 4 | amp |
| TCGA-04-1349 | 19 | 15508475 | 15538368 | 2.701978093 | 5 | amp |
| TCGA-04-1349 | 19 | 15558865 | 15763817 | 1.457915389 | 4 | amp |
| TCGA-04-1349 | 19 | 15768990 | 18727920 | 2.585210273 | 5 | amp |
| TCGA-04-1349 | 19 | 18728873 | 30500307 | 1.478784954 | 4 | amp |
| TCGA-04-1349 | 2 | 41527 | 3749283 | 1.189948143 | 4 | amp |
| TCGA-04-1349 | 2 | 4135920 | 9499071 | 1.02319705 | 3 | amp |
| TCGA-04-1349 | 2 | 9508540 | 24440888 | 1.151016805 | 4 | amp |
| TCGA-04-1349 | 2 | 24443724 | 26676254 | 1.069613054 | 3 | amp |
| TCGA-04-1349 | 2 | 26676334 | 27375765 | 1.201973408 | 4 | amp |
| TCGA-04-1349 | 2 | 27423225 | 27587794 | 1.004235861 | 3 | amp |
| TCGA-04-1349 | 2 | 27589594 | 27670793 | 1.369597672 | 4 | amp |
| TCGA-04-1349 | 2 | 27671689 | 48941218 | 1.064976605 | 3 | amp |
| TCGA-04-1349 | 2 | 48950573 | 54102947 | 1.205489495 | 4 | amp |
| TCGA-04-1349 | 2 | 54112809 | 71299885 | 1.094861975 | 3 | amp |
| TCGA-04-1349 | 2 | 71300577 | 73652033 | 1.180086337 | 4 | amp |
| TCGA-04-1349 | 2 | 73653540 | 74595297 | 1.068763701 | 3 | amp |
| TCGA-04-1349 | 2 | 74595833 | 85858076 | 1.203550214 | 4 | amp |
| TCGA-04-1349 | 2 | 85863154 | 88111730 | 1.078222402 | 3 | amp |
| TCGA-04-1349 | 2 | 88115744 | 97019149 | 1.197629834 | 4 | amp |
| TCGA-04-1349 | 2 | 97019873 | 97506706 | 1.025048893 | 3 | amp |

| | | | | | | |
|---|---|---|---|---|---|---|
| TCGA-04-1349 | 2 | 97507739 | 98779496 | 1.17995603 | 4 | amp |
| TCGA-04-1349 | 2 | 98797456 | 101023147 | 0.981798846 | 3 | amp |
| TCGA-04-1349 | 2 | 233390861 | 233393408 | 1.569208894 | 4 | amp |
| TCGA-04-1349 | 20 | 68319 | 264782 | 1.224257518 | 4 | amp |
| TCGA-04-1349 | 20 | 270138 | 896882 | 1.071385451 | 3 | amp |
| TCGA-04-1349 | 20 | 944520 | 1438941 | 1.200928521 | 4 | amp |
| TCGA-04-1349 | 20 | 1444932 | 2644414 | 1.07628722 | 3 | amp |
| TCGA-04-1349 | 20 | 2644520 | 3215583 | 1.229506702 | 4 | amp |
| TCGA-04-1349 | 20 | 3218137 | 3654806 | 1.031761445 | 3 | amp |
| TCGA-04-1349 | 20 | 3654808 | 3784237 | 1.429302107 | 4 | amp |
| TCGA-04-1349 | 20 | 3785181 | 5566937 | 1.03366855 | 3 | amp |
| TCGA-04-1349 | 20 | 5573954 | 10394209 | 1.21613271 | 4 | amp |
| TCGA-04-1349 | 20 | 10438805 | 10632958 | 0.979575688 | 3 | amp |
| TCGA-04-1349 | 20 | 10633060 | 13789577 | 1.201745971 | 4 | amp |
| TCGA-04-1349 | 20 | 13797090 | 13868677 | 0.908993471 | 3 | amp |
| TCGA-04-1349 | 20 | 13869071 | 18492957 | 1.200838855 | 4 | amp |
| TCGA-04-1349 | 20 | 18496276 | 19634802 | 1.01449544 | 3 | amp |
| TCGA-04-1349 | 20 | 19646476 | 20392851 | 1.200565628 | 4 | amp |
| TCGA-04-1349 | 20 | 20453416 | 20661449 | 1.06462955 | 3 | amp |
| TCGA-04-1349 | 20 | 21116991 | 23383729 | 1.249613965 | 4 | amp |
| TCGA-04-1349 | 20 | 23420863 | 23860317 | 1.040916352 | 3 | amp |
| TCGA-04-1349 | 20 | 23965805 | 31374490 | 1.193216734 | 4 | amp |
| TCGA-04-1349 | 20 | 31374996 | 31571797 | 0.901772048 | 3 | amp |
| TCGA-04-1349 | 20 | 31572864 | 32881984 | 1.262471319 | 4 | amp |
| TCGA-04-1349 | 20 | 32883145 | 33045296 | 0.885534873 | 3 | amp |
| TCGA-04-1349 | 20 | 33049811 | 33320440 | 1.224028954 | 4 | amp |
| TCGA-04-1349 | 20 | 33433092 | 33442437 | 0.514874342 | 1 | loss |
| TCGA-04-1349 | 20 | 33442512 | 33502242 | 1.635074846 | 4 | amp |
| TCGA-04-1349 | 20 | 33502979 | 33519958 | 0.837809637 | 3 | amp |
| TCGA-04-1349 | 20 | 33523321 | 34313092 | 1.182010875 | 4 | amp |
| TCGA-04-1349 | 20 | 34317200 | 35071214 | 1.034353817 | 3 | amp |
| TCGA-04-1349 | 20 | 35075009 | 35812799 | 1.197756974 | 4 | amp |
| TCGA-04-1349 | 20 | 35826787 | 36982881 | 1.077851478 | 3 | amp |
| TCGA-04-1349 | 20 | 36983687 | 37159901 | 1.373584116 | 4 | amp |
| TCGA-04-1349 | 20 | 37161317 | 39833566 | 1.042077419 | 3 | amp |
| TCGA-04-1349 | 20 | 39974419 | 43052959 | 1.196146255 | 4 | amp |
| TCGA-04-1349 | 20 | 43056930 | 43247025 | 0.92480103 | 3 | amp |
| TCGA-04-1349 | 20 | 43248391 | 43610586 | 1.299923016 | 4 | amp |
| TCGA-04-1349 | 20 | 43615734 | 43739465 | 0.970525845 | 3 | amp |
| TCGA-04-1349 | 20 | 43743621 | 45140790 | 1.25927605 | 4 | amp |
| TCGA-04-1349 | 20 | 45188628 | 46277860 | 1.07552097 | 3 | amp |
| TCGA-04-1349 | 20 | 46279663 | 47268131 | 1.214746428 | 4 | amp |
| TCGA-04-1349 | 20 | 47269063 | 49185025 | 1.050590864 | 3 | amp |
| TCGA-04-1349 | 20 | 49190972 | 50273702 | 1.224849027 | 4 | amp |
| TCGA-04-1349 | 20 | 50400773 | 57470762 | 1.213795767 | 4 | amp |
| TCGA-04-1349 | 20 | 57473927 | 57485930 | 0.9006213 | 3 | amp |
| TCGA-04-1349 | 20 | 57561115 | 62926333 | 1.251443241 | 4 | amp |
| TCGA-04-1349 | 21 | 9483321 | 34717668 | 1.030009146 | 3 | amp |
| TCGA-04-1349 | 21 | 34889251 | 38517004 | 0.996097035 | 3 | amp |
| TCGA-04-1349 | 21 | 38593378 | 40610511 | 1.064195459 | 3 | amp |

| | | | | | | |
|---|---|---|---|---|---|---|
| TCGA-04-1349 | 21 | 41452022 | 43508616 | 1.056807195 | 3 | amp |
| TCGA-04-1349 | 21 | 43510357 | 43536098 | 1.610723191 | 4 | amp |
| TCGA-04-1349 | 21 | 43539177 | 48111215 | 1.044519171 | 3 | amp |
| TCGA-04-1349 | 22 | 16084594 | 17055560 | 1.027124545 | 3 | amp |
| TCGA-04-1349 | 22 | 17669222 | 18189631 | 0.513648881 | 1 | loss |
| TCGA-04-1349 | 22 | 19443186 | 19459390 | 1.107816772 | 3 | amp |
| TCGA-04-1349 | 22 | 21098901 | 21242165 | 0.48828542 | 1 | loss |
| TCGA-04-1349 | 22 | 23494590 | 23610708 | 0.37605434 | 1 | loss |
| TCGA-04-1349 | 22 | 30781845 | 30781995 | 2.218584346 | 5 | amp |
| TCGA-04-1349 | 22 | 31829817 | 31998801 | 0.487343531 | 1 | loss |
| TCGA-04-1349 | 22 | 36900069 | 36922226 | 0.904833127 | 3 | amp |
| TCGA-04-1349 | 22 | 40745817 | 40762542 | 0.960497882 | 3 | amp |
| TCGA-04-1349 | 22 | 41542709 | 41616924 | 0.485622889 | 1 | loss |
| TCGA-04-1349 | 22 | 45564002 | 45579461 | 1.072841886 | 3 | amp |
| TCGA-04-1349 | 3 | 361444 | 39144390 | 1.153146256 | 3 | amp |
| TCGA-04-1349 | 3 | 39144913 | 39554953 | 1.34453533 | 4 | amp |
| TCGA-04-1349 | 3 | 39942272 | 44490288 | 1.15360571 | 3 | amp |
| TCGA-04-1349 | 3 | 44490969 | 44751813 | 1.49271336 | 4 | amp |
| TCGA-04-1349 | 3 | 44761690 | 48436134 | 1.175645328 | 3 | amp |
| TCGA-04-1349 | 3 | 48445854 | 48625039 | 1.386641372 | 4 | amp |
| TCGA-04-1349 | 3 | 48625148 | 49012334 | 1.176859351 | 3 | amp |
| TCGA-04-1349 | 3 | 49012341 | 49450575 | 1.29442597 | 4 | amp |
| TCGA-04-1349 | 3 | 49452213 | 49662851 | 1.099187416 | 3 | amp |
| TCGA-04-1349 | 3 | 49679662 | 50386492 | 1.280296825 | 4 | amp |
| TCGA-04-1349 | 3 | 50387029 | 125295240 | 1.107335644 | 3 | amp |
| TCGA-04-1349 | 3 | 125297776 | 134251800 | 1.555099233 | 4 | amp |
| TCGA-04-1349 | 3 | 134255963 | 134280417 | 1.926606298 | 5 | amp |
| TCGA-04-1349 | 3 | 134322388 | 154034009 | 1.551275167 | 4 | amp |
| TCGA-04-1349 | 3 | 154041903 | 155218597 | 1.888167098 | 5 | amp |
| TCGA-04-1349 | 3 | 155222319 | 156763573 | 1.615494068 | 4 | amp |
| TCGA-04-1349 | 3 | 156866023 | 156983502 | 2.048546458 | 5 | amp |
| TCGA-04-1349 | 3 | 157004254 | 161221735 | 1.632754542 | 4 | amp |
| TCGA-04-1349 | 3 | 164697123 | 167078546 | 1.968706456 | 5 | amp |
| TCGA-04-1349 | 3 | 167083658 | 169486158 | 1.572634465 | 4 | amp |
| TCGA-04-1349 | 3 | 169492036 | 169540759 | 2.134583549 | 5 | amp |
| TCGA-04-1349 | 3 | 169546513 | 183856053 | 1.540550209 | 4 | amp |
| TCGA-04-1349 | 3 | 183857816 | 183873623 | 2.262653892 | 5 | amp |
| TCGA-04-1349 | 3 | 183881389 | 183888021 | 1.441102436 | 4 | amp |
| TCGA-04-1349 | 3 | 183896597 | 184070937 | 2.133148918 | 5 | amp |
| TCGA-04-1349 | 3 | 184070973 | 186395139 | 1.597306386 | 4 | amp |
| TCGA-04-1349 | 3 | 186395321 | 186519004 | 1.856497813 | 5 | amp |
| TCGA-04-1349 | 3 | 186522316 | 193044875 | 1.574627078 | 4 | amp |
| TCGA-04-1349 | 3 | 193048885 | 193130183 | 1.928080241 | 5 | amp |
| TCGA-04-1349 | 3 | 193132333 | 197955154 | 1.505163137 | 4 | amp |
| TCGA-04-1349 | 4 | 53323 | 4214782 | 1.109383273 | 3 | amp |
| TCGA-04-1349 | 4 | 4239552 | 4304866 | 1.3674743 | 4 | amp |
| TCGA-04-1349 | 4 | 4307827 | 6349774 | 1.107630444 | 3 | amp |
| TCGA-04-1349 | 4 | 6374188 | 23891613 | 1.232106572 | 4 | amp |
| TCGA-04-1349 | 4 | 24521773 | 25014158 | 0.943779538 | 3 | amp |
| TCGA-04-1349 | 4 | 25019575 | 25667941 | 1.289798873 | 4 | amp |

| | | | | | | |
|---|---|---|---|---|---|---|
| TCGA-04-1349 | 4 | 25669497 | 25759420 | 0.945604116 | 3 | amp |
| TCGA-04-1349 | 4 | 47746389 | 47853198 | 1.103385816 | 3 | amp |
| TCGA-04-1349 | 4 | 47853855 | 48553612 | 1.214821909 | 4 | amp |
| TCGA-04-1349 | 4 | 48555206 | 48862820 | 1.054177332 | 3 | amp |
| TCGA-04-1349 | 4 | 48887451 | 49052908 | 1.312402414 | 4 | amp |
| TCGA-04-1349 | 4 | 49063827 | 55592239 | 1.099721464 | 3 | amp |
| TCGA-04-1349 | 4 | 55593376 | 57852480 | 1.205700239 | 4 | amp |
| TCGA-04-1349 | 4 | 57852495 | 62363098 | 1.001995531 | 3 | amp |
| TCGA-04-1349 | 4 | 62445256 | 71501639 | 1.269905447 | 4 | amp |
| TCGA-04-1349 | 4 | 71503473 | 72205273 | 1.059977266 | 3 | amp |
| TCGA-04-1349 | 4 | 72215588 | 73951184 | 1.298185051 | 4 | amp |
| TCGA-04-1349 | 4 | 73956360 | 74349741 | 1.042526053 | 3 | amp |
| TCGA-04-1349 | 4 | 74349920 | 76817510 | 1.202710483 | 4 | amp |
| TCGA-04-1349 | 4 | 76836007 | 79440679 | 1.126283934 | 3 | amp |
| TCGA-04-1349 | 4 | 79442670 | 83742285 | 1.227520946 | 4 | amp |
| TCGA-04-1349 | 4 | 83745657 | 84379600 | 1.104949742 | 3 | amp |
| TCGA-04-1349 | 4 | 84380861 | 84519896 | 1.32394739 | 4 | amp |
| TCGA-04-1349 | 4 | 84525780 | 89345128 | 1.10474338 | 3 | amp |
| TCGA-04-1349 | 4 | 89345740 | 94159701 | 1.18367556 | 4 | amp |
| TCGA-04-1349 | 4 | 94316748 | 100232865 | 1.099473319 | 3 | amp |
| TCGA-04-1349 | 4 | 100234927 | 101439099 | 1.196875459 | 4 | amp |
| TCGA-04-1349 | 4 | 101947006 | 104072574 | 1.107489679 | 3 | amp |
| TCGA-04-1349 | 4 | 104074185 | 110754509 | 1.200705667 | 4 | amp |
| TCGA-04-1349 | 4 | 110756511 | 111464258 | 1.004780655 | 3 | amp |
| TCGA-04-1349 | 4 | 111469406 | 113970986 | 1.201964911 | 4 | amp |
| TCGA-04-1349 | 4 | 114028004 | 120987912 | 1.139342366 | 3 | amp |
| TCGA-04-1349 | 4 | 121616227 | 123145862 | 1.249614723 | 4 | amp |
| TCGA-04-1349 | 4 | 123147805 | 123192885 | 0.989368493 | 3 | amp |
| TCGA-04-1349 | 4 | 123193227 | 140297632 | 1.201311784 | 4 | amp |
| TCGA-04-1349 | 4 | 140299898 | 140812172 | 0.983461191 | 3 | amp |
| TCGA-04-1349 | 4 | 141073973 | 146042573 | 1.240767584 | 4 | amp |
| TCGA-04-1349 | 4 | 146044167 | 154548857 | 1.140225241 | 3 | amp |
| TCGA-04-1349 | 4 | 154553779 | 159756652 | 1.191996425 | 4 | amp |
| TCGA-04-1349 | 4 | 159772417 | 160279338 | 1.009773619 | 3 | amp |
| TCGA-04-1349 | 4 | 162306889 | 165118891 | 1.228225445 | 4 | amp |
| TCGA-04-1349 | 4 | 165836680 | 166915728 | 1.075995368 | 3 | amp |
| TCGA-04-1349 | 4 | 166916121 | 169183940 | 1.25344602 | 4 | amp |
| TCGA-04-1349 | 4 | 169188727 | 175649857 | 1.153861157 | 3 | amp |
| TCGA-04-1349 | 4 | 175688087 | 183836730 | 1.270581008 | 4 | amp |
| TCGA-04-1349 | 4 | 184114746 | 186299347 | 1.105594047 | 3 | amp |
| TCGA-04-1349 | 4 | 186318000 | 187209027 | 1.190262016 | 4 | amp |
| TCGA-04-1349 | 4 | 187209567 | 188924907 | 1.059635549 | 3 | amp |
| TCGA-04-1349 | 4 | 189012510 | 190948390 | 1.249339866 | 4 | amp |
| TCGA-04-1349 | 5 | 151610 | 17354033 | 1.083368678 | 3 | amp |
| TCGA-04-1349 | 5 | 18049639 | 31267814 | 1.216518868 | 4 | amp |
| TCGA-04-1349 | 5 | 31294035 | 34998937 | 1.048202662 | 3 | amp |
| TCGA-04-1349 | 5 | 35003826 | 37314442 | 1.162551553 | 4 | amp |
| TCGA-04-1349 | 5 | 37318048 | 39721291 | 1.093054333 | 3 | amp |
| TCGA-04-1349 | 5 | 40681018 | 49963034 | 1.208132107 | 4 | amp |
| TCGA-04-1349 | 5 | 49963871 | 52285390 | 1.023086932 | 3 | amp |

| | | | | | | |
|---|---|---|---|---|---|---|
| TCGA-04-1349 | 5 | 98193868 | 100238662 | 0.99467385 | 3 | amp |
| TCGA-04-1349 | 5 | 101572521 | 102504007 | 1.191406978 | 4 | amp |
| TCGA-04-1349 | 5 | 102508802 | 109065248 | 1.061091592 | 3 | amp |
| TCGA-04-1349 | 5 | 109090973 | 111504536 | 1.192585999 | 4 | amp |
| TCGA-04-1349 | 5 | 111504686 | 118525611 | 1.082944228 | 3 | amp |
| TCGA-04-1349 | 5 | 118529460 | 118877704 | 1.263347331 | 4 | amp |
| TCGA-04-1349 | 5 | 118965438 | 139917878 | 1.083424008 | 3 | amp |
| TCGA-04-1349 | 5 | 139918483 | 140812767 | 1.220261606 | 4 | amp |
| TCGA-04-1349 | 5 | 140855608 | 141053393 | 1.117019494 | 3 | amp |
| TCGA-04-1349 | 5 | 141059077 | 141511476 | 1.272751782 | 4 | amp |
| TCGA-04-1349 | 5 | 141511721 | 176829744 | 1.067332507 | 3 | amp |
| TCGA-04-1349 | 5 | 176832000 | 176943216 | 1.482068346 | 4 | amp |
| TCGA-04-1349 | 5 | 176943218 | 180899507 | 1.067057212 | 3 | amp |
| TCGA-04-1349 | 6 | 105907 | 30645115 | 1.417127071 | 4 | amp |
| TCGA-04-1349 | 6 | 30646910 | 31498278 | 1.061361406 | 3 | amp |
| TCGA-04-1349 | 6 | 31498509 | 31713114 | 1.137143324 | 4 | amp |
| TCGA-04-1349 | 6 | 31715126 | 31948624 | 1.024644301 | 3 | amp |
| TCGA-04-1349 | 6 | 31948742 | 32024704 | 1.225381651 | 4 | amp |
| TCGA-04-1349 | 6 | 32029152 | 33037720 | 0.996063839 | 3 | amp |
| TCGA-04-1349 | 6 | 33041207 | 33054065 | 1.755689281 | 4 | amp |
| TCGA-04-1349 | 6 | 33095674 | 33231353 | 0.968091707 | 3 | amp |
| TCGA-04-1349 | 6 | 33231512 | 33238077 | 1.461480402 | 4 | amp |
| TCGA-04-1349 | 6 | 33239837 | 38691020 | 0.993108373 | 3 | amp |
| TCGA-04-1349 | 6 | 38691059 | 38980186 | 1.221564514 | 4 | amp |
| TCGA-04-1349 | 6 | 38980198 | 42830358 | 0.994871092 | 3 | amp |
| TCGA-04-1349 | 6 | 42832370 | 43154885 | 1.318439501 | 4 | amp |
| TCGA-04-1349 | 6 | 43154930 | 43189097 | 0.928529887 | 3 | amp |
| TCGA-04-1349 | 6 | 43189409 | 64356710 | 1.266362216 | 4 | amp |
| TCGA-04-1349 | 6 | 64389861 | 65525180 | 1.049242896 | 3 | amp |
| TCGA-04-1349 | 6 | 109748267 | 109764291 | 0.901889871 | 3 | amp |
| TCGA-04-1349 | 6 | 109764396 | 109769181 | 1.534916258 | 4 | amp |
| TCGA-04-1349 | 6 | 159647498 | 160241577 | 1.017215603 | 3 | amp |
| TCGA-04-1349 | 6 | 168355096 | 171055029 | 0.934087228 | 3 | amp |
| TCGA-04-1349 | 7 | 48356703 | 93116327 | 1.258433669 | 4 | amp |
| TCGA-04-1349 | 7 | 93516109 | 100086644 | 1.11891462 | 3 | amp |
| TCGA-04-1349 | 7 | 100146431 | 100201166 | 1.242437686 | 4 | amp |
| TCGA-04-1349 | 7 | 100201500 | 100205762 | 2.164858805 | 5 | amp |
| TCGA-04-1349 | 7 | 100210524 | 100244936 | 1.234768737 | 4 | amp |
| TCGA-04-1349 | 7 | 100246308 | 100274266 | 2.166337412 | 5 | amp |
| TCGA-04-1349 | 7 | 100274916 | 102210386 | 1.194647481 | 4 | amp |
| TCGA-04-1349 | 7 | 102212883 | 155094566 | 1.08042836 | 3 | amp |
| TCGA-04-1349 | 7 | 155095509 | 158935247 | 1.403353556 | 4 | amp |
| TCGA-04-1349 | 8 | 116074 | 33342791 | 0.45886757 | 1 | loss |
| TCGA-04-1349 | 8 | 33345830 | 35383289 | 0.842783807 | 3 | amp |
| TCGA-04-1349 | 8 | 35401919 | 146279593 | 2.44319235 | 5 | amp |
| TCGA-04-1349 | 9 | 16945 | 123309 | 0.867546885 | 3 | amp |
| TCGA-04-1349 | 9 | 26905491 | 29999683 | 1.013335893 | 3 | amp |
| TCGA-04-1349 | 9 | 131196294 | 131222951 | 1.122799139 | 3 | amp |
| TCGA-04-1349 | X | 2832619 | 2867796 | 1.032103166 | 3 | amp |
| TCGA-04-1349 | X | 12692944 | 12728706 | 1.03003687 | 3 | amp |

| | | | | | | |
|---|---|---|---|---|---|---|
| TCGA-04-1349 | X | 13778176 | 13795567 | 0.447118498 | 1 | loss |
| TCGA-04-1349 | X | 15272822 | 15320949 | 0.496347569 | 1 | loss |
| TCGA-04-1349 | X | 16707498 | 16717250 | 0.44800051 | 1 | loss |
| TCGA-04-1349 | X | 24197243 | 24229501 | 0.437507301 | 1 | loss |
| TCGA-04-1349 | X | 48243468 | 48300840 | 0.491498008 | 1 | loss |
| TCGA-04-1349 | X | 49137836 | 49242970 | 1.025624708 | 3 | amp |
| TCGA-04-1349 | X | 49294422 | 49368453 | 1.114949187 | 4 | amp |
| TCGA-04-1349 | X | 70608037 | 70621603 | 0.445839637 | 1 | loss |
| TCGA-04-1349 | X | 99941666 | 99956003 | 0.485134746 | 1 | loss |
| TCGA-04-1349 | X | 101091107 | 101093840 | 0.464861193 | 1 | loss |
| TCGA-04-1349 | X | 102962206 | 102977269 | 0.354870277 | 1 | loss |
| TCGA-04-1349 | X | 106884090 | 106893275 | 0.506485569 | 1 | loss |
| TCGA-04-1349 | X | 107807073 | 107816903 | 0.437952926 | 1 | loss |
| TCGA-04-1349 | X | 118673660 | 118699376 | 0.443441967 | 1 | loss |
| TCGA-04-1349 | X | 119063933 | 119292756 | 0.907902872 | 3 | amp |
| TCGA-04-1349 | X | 120068945 | 120119428 | 1.353040447 | 4 | amp |
| TCGA-04-1349 | X | 128887089 | 128895259 | 0.594618554 | 1 | loss |
| TCGA-04-1349 | X | 129158885 | 129173304 | 0.439674735 | 1 | loss |
| TCGA-04-1349 | X | 135084259 | 135292226 | 0.505022581 | 1 | loss |
| TCGA-04-1349 | X | 135757150 | 135763082 | 0.444086661 | 1 | loss |
| TCGA-04-1349 | X | 140008300 | 140097831 | 0.44296482 | 1 | loss |
| TCGA-04-1349 | X | 149630984 | 149642132 | 0.409809662 | 1 | loss |
| TCGA-04-1349 | X | 151928333 | 151998344 | 0.91455727 | 3 | amp |
| TCGA-04-1349 | X | 153537655 | 153553786 | 0.42362566 | 1 | loss |
| TCGA-04-1349 | X | 153590564 | 153608400 | 1.118245037 | 3 | amp |
| TCGA-04-1361 | 1 | 6727759 | 10166687 | 0.864093455 | 4 | amp |
| TCGA-04-1361 | 1 | 10177467 | 10425350 | 1.332231598 | 5 | amp |
| TCGA-04-1361 | 1 | 10425376 | 11303383 | 0.792505274 | 4 | amp |
| TCGA-04-1361 | 1 | 12064527 | 14113075 | 0.836373233 | 4 | amp |
| TCGA-04-1361 | 1 | 14142861 | 15783328 | 0.801197772 | 3 | amp |
| TCGA-04-1361 | 1 | 19438979 | 19553995 | 0.955429253 | 4 | amp |
| TCGA-04-1361 | 1 | 28282134 | 28907618 | 0.823179162 | 4 | amp |
| TCGA-04-1361 | 1 | 28930008 | 29487077 | 1.330309211 | 5 | amp |
| TCGA-04-1361 | 1 | 29520462 | 31351648 | 0.834719914 | 4 | amp |
| TCGA-04-1361 | 1 | 31406003 | 31409705 | 1.455298109 | 5 | amp |
| TCGA-04-1361 | 1 | 31413938 | 31836991 | 2.058322843 | 6 | amp |
| TCGA-04-1361 | 1 | 32374446 | 32505204 | 1.888643332 | 6 | amp |
| TCGA-04-1361 | 1 | 32508116 | 32658091 | 1.175749351 | 5 | amp |
| TCGA-04-1361 | 1 | 32658204 | 32742133 | 0.599185878 | 3 | amp |
| TCGA-04-1361 | 1 | 32742147 | 35453182 | 1.30251718 | 5 | amp |
| TCGA-04-1361 | 1 | 35454492 | 36020141 | 2.060469497 | 6 | amp |
| TCGA-04-1361 | 1 | 36023747 | 36215159 | 1.378106454 | 5 | amp |
| TCGA-04-1361 | 1 | 36215202 | 36319211 | 2.199370315 | 6 | amp |
| TCGA-04-1361 | 1 | 36353999 | 36411498 | 1.081621746 | 5 | amp |
| TCGA-04-1361 | 1 | 36432495 | 36550946 | 2.223879033 | 6 | amp |
| TCGA-04-1361 | 1 | 36551420 | 38270164 | 1.044690144 | 5 | amp |
| TCGA-04-1361 | 1 | 38272438 | 38357137 | 2.159474471 | 6 | amp |
| TCGA-04-1361 | 1 | 38406354 | 39500175 | 1.503619308 | 5 | amp |
| TCGA-04-1361 | 1 | 39549880 | 39927751 | 2.067255778 | 6 | amp |
| TCGA-04-1361 | 1 | 39929230 | 40435268 | 1.357195688 | 5 | amp |

| | | | | | | |
|---|---|---|---|---|---|---|
| TCGA-04-1361 | 1 | 40524984 | 40735842 | 1.817226003 | 6 | amp |
| TCGA-04-1361 | 1 | 40737516 | 41298795 | 1.375941771 | 5 | amp |
| TCGA-04-1361 | 1 | 41300584 | 41608786 | 1.812127652 | 6 | amp |
| TCGA-04-1361 | 1 | 41617230 | 42647701 | 1.220653961 | 5 | amp |
| TCGA-04-1361 | 1 | 42654383 | 42914343 | 2.260977283 | 6 | amp |
| TCGA-04-1361 | 1 | 42915539 | 43675755 | 1.241981086 | 5 | amp |
| TCGA-04-1361 | 1 | 43748436 | 43826081 | 0.879221853 | 4 | amp |
| TCGA-04-1361 | 1 | 44128541 | 45851667 | 0.98015105 | 4 | amp |
| TCGA-04-1361 | 1 | 45887337 | 46491537 | 1.277677836 | 5 | amp |
| TCGA-04-1361 | 1 | 46493348 | 47080755 | 0.891860646 | 4 | amp |
| TCGA-04-1361 | 1 | 47101424 | 48869573 | 1.339857157 | 5 | amp |
| TCGA-04-1361 | 1 | 48877077 | 52302135 | 1.854837831 | 6 | amp |
| TCGA-04-1361 | 1 | 52303105 | 52891219 | 1.280707086 | 5 | amp |
| TCGA-04-1361 | 1 | 52896668 | 53267847 | 1.834821546 | 6 | amp |
| TCGA-04-1361 | 1 | 53279203 | 54607118 | 1.26532744 | 5 | amp |
| TCGA-04-1361 | 1 | 54610102 | 55465067 | 0.908427724 | 4 | amp |
| TCGA-04-1361 | 1 | 55470651 | 55598415 | 1.471280235 | 5 | amp |
| TCGA-04-1361 | 1 | 55599729 | 62672712 | 1.891798986 | 6 | amp |
| TCGA-04-1361 | 1 | 62675433 | 62958566 | 1.399868427 | 5 | amp |
| TCGA-04-1361 | 1 | 62959921 | 65304311 | 2.047979367 | 6 | amp |
| TCGA-04-1361 | 1 | 65305261 | 65656572 | 1.130135153 | 5 | amp |
| TCGA-04-1361 | 1 | 65684402 | 66458827 | 2.017657128 | 6 | amp |
| TCGA-04-1361 | 1 | 66713089 | 94674483 | 1.381331429 | 5 | amp |
| TCGA-04-1361 | 1 | 94674797 | 109177810 | 2.067978982 | 6 | amp |
| TCGA-04-1361 | 1 | 109191160 | 109284470 | 1.33338217 | 5 | amp |
| TCGA-04-1361 | 1 | 109294843 | 109359854 | 1.977124019 | 6 | amp |
| TCGA-04-1361 | 1 | 109363117 | 109607349 | 1.5169461 | 5 | amp |
| TCGA-04-1361 | 1 | 109608745 | 110775623 | 0.909855976 | 4 | amp |
| TCGA-04-1361 | 1 | 110882018 | 113232770 | 1.328570611 | 5 | amp |
| TCGA-04-1361 | 1 | 113234203 | 113634032 | 0.701058744 | 4 | amp |
| TCGA-04-1361 | 1 | 113635774 | 114226677 | 1.58188358 | 5 | amp |
| TCGA-04-1361 | 1 | 114240276 | 114340718 | 1.848329998 | 6 | amp |
| TCGA-04-1361 | 1 | 114354322 | 114949762 | 1.304712995 | 5 | amp |
| TCGA-04-1361 | 1 | 114951270 | 115168622 | 2.007557073 | 6 | amp |
| TCGA-04-1361 | 1 | 115215693 | 115401399 | 1.323090426 | 5 | amp |
| TCGA-04-1361 | 1 | 115403303 | 116655320 | 1.805206995 | 6 | amp |
| TCGA-04-1361 | 1 | 116663472 | 145368622 | 1.319251876 | 5 | amp |
| TCGA-04-1361 | 1 | 145414739 | 145663382 | 0.640266417 | 3 | amp |
| TCGA-04-1361 | 1 | 145681998 | 146414253 | 1.397858799 | 5 | amp |
| TCGA-04-1361 | 1 | 146418099 | 146457336 | 1.753250693 | 6 | amp |
| TCGA-04-1361 | 1 | 146457864 | 149760212 | 1.383935032 | 5 | amp |
| TCGA-04-1361 | 1 | 150252011 | 153318682 | 0.802839113 | 4 | amp |
| TCGA-04-1361 | 1 | 153997984 | 154245285 | 1.048561295 | 4 | amp |
| TCGA-04-1361 | 1 | 155269829 | 155924808 | 0.922550499 | 4 | amp |
| TCGA-04-1361 | 1 | 156933288 | 157491124 | 0.85900809 | 4 | amp |
| TCGA-04-1361 | 1 | 157493997 | 157773947 | 1.237972499 | 5 | amp |
| TCGA-04-1361 | 1 | 157776841 | 158227551 | 0.747618176 | 4 | amp |
| TCGA-04-1361 | 1 | 158259824 | 159785508 | 1.217922457 | 5 | amp |
| TCGA-04-1361 | 1 | 159796618 | 160395217 | 0.873510029 | 4 | amp |
| TCGA-04-1361 | 1 | 160456460 | 160849212 | 1.31366772 | 5 | amp |

| | | | | | | |
|---|---|---|---|---|---|---|
| TCGA-04-1361 | 1 | 160850287 | 162381859 | 0.808435584 | 4 | amp |
| TCGA-04-1361 | 1 | 162467714 | 165649941 | 1.362214384 | 5 | amp |
| TCGA-04-1361 | 1 | 165651290 | 167802432 | 0.944857949 | 4 | amp |
| TCGA-04-1361 | 1 | 167803201 | 180010986 | 1.26170365 | 5 | amp |
| TCGA-04-1361 | 1 | 180012138 | 182545577 | 0.909410721 | 4 | amp |
| TCGA-04-1361 | 1 | 182550305 | 196274479 | 1.414967661 | 5 | amp |
| TCGA-04-1361 | 1 | 196284938 | 197094368 | 1.706514792 | 6 | amp |
| TCGA-04-1361 | 1 | 197097587 | 200827214 | 1.388048564 | 5 | amp |
| TCGA-04-1361 | 1 | 201782206 | 202915772 | 0.861907478 | 4 | amp |
| TCGA-04-1361 | 1 | 205052644 | 207243839 | 0.844001585 | 4 | amp |
| TCGA-04-1361 | 1 | 207244503 | 207963683 | 1.352870939 | 5 | amp |
| TCGA-04-1361 | 1 | 207966811 | 209849365 | 0.748503509 | 4 | amp |
| TCGA-04-1361 | 1 | 209878271 | 225692783 | 1.317889439 | 5 | amp |
| TCGA-04-1361 | 1 | 225695631 | 227257575 | 0.809292034 | 4 | amp |
| TCGA-04-1361 | 1 | 227259864 | 227843562 | 1.446119649 | 5 | amp |
| TCGA-04-1361 | 1 | 228553770 | 231386917 | 1.038562963 | 4 | amp |
| TCGA-04-1361 | 1 | 231396220 | 249231325 | 1.243367451 | 5 | amp |
| TCGA-04-1361 | 10 | 3209120 | 13789837 | 0.896080211 | 3 | amp |
| TCGA-04-1361 | 10 | 13803597 | 18331817 | 1.318008283 | 5 | amp |
| TCGA-04-1361 | 10 | 18439809 | 22030949 | 1.851890828 | 6 | amp |
| TCGA-04-1361 | 10 | 22045589 | 24822202 | 1.395344489 | 5 | amp |
| TCGA-04-1361 | 10 | 24825642 | 28903674 | 1.907553503 | 6 | amp |
| TCGA-04-1361 | 10 | 28905076 | 30336780 | 0.897889497 | 3 | amp |
| TCGA-04-1361 | 10 | 30602478 | 38894594 | 1.293220537 | 5 | amp |
| TCGA-04-1361 | 10 | 38934089 | 43289489 | 1.033592082 | 4 | amp |
| TCGA-04-1361 | 10 | 43291880 | 43319264 | 0.847128454 | 3 | amp |
| TCGA-04-1361 | 10 | 46210533 | 46664255 | 0.85160118 | 3 | amp |
| TCGA-04-1361 | 10 | 47893975 | 47922420 | 0.935983661 | 3 | amp |
| TCGA-04-1361 | 10 | 47925971 | 48050564 | 1.408445602 | 5 | amp |
| TCGA-04-1361 | 10 | 48187381 | 51769988 | 0.760577581 | 3 | amp |
| TCGA-04-1361 | 10 | 51807906 | 51956725 | 1.314218649 | 5 | amp |
| TCGA-04-1361 | 10 | 51958798 | 52071218 | 1.704685103 | 6 | amp |
| TCGA-04-1361 | 10 | 52086873 | 52505081 | 0.609316382 | 3 | amp |
| TCGA-04-1361 | 10 | 52509072 | 55571404 | 1.726134661 | 6 | amp |
| TCGA-04-1361 | 10 | 55581562 | 65930392 | 1.47712651 | 5 | amp |
| TCGA-04-1361 | 10 | 67680051 | 69961788 | 1.678091276 | 6 | amp |
| TCGA-04-1361 | 10 | 69966443 | 71000517 | 1.289261818 | 5 | amp |
| TCGA-04-1361 | 10 | 71002909 | 72643815 | 0.771234745 | 3 | amp |
| TCGA-04-1361 | 10 | 73856999 | 73956777 | 0.893546302 | 3 | amp |
| TCGA-04-1361 | 10 | 73963173 | 74167800 | 1.065053475 | 4 | amp |
| TCGA-04-1361 | 10 | 74182940 | 75335462 | 1.285002206 | 5 | amp |
| TCGA-04-1361 | 10 | 75391653 | 75802965 | 0.723072599 | 3 | amp |
| TCGA-04-1361 | 10 | 75830382 | 75871888 | 1.06900724 | 4 | amp |
| TCGA-04-1361 | 10 | 75873923 | 79493777 | 1.316905189 | 5 | amp |
| TCGA-04-1361 | 10 | 79552159 | 89268311 | 0.828318433 | 3 | amp |
| TCGA-04-1361 | 10 | 89272869 | 92645673 | 1.362253452 | 5 | amp |
| TCGA-04-1361 | 10 | 92654469 | 93221968 | 1.889018968 | 6 | amp |
| TCGA-04-1361 | 10 | 93237929 | 94594598 | 1.373540172 | 5 | amp |
| TCGA-04-1361 | 10 | 94653041 | 94824335 | 1.680618953 | 6 | amp |
| TCGA-04-1361 | 10 | 94836256 | 95116627 | 0.760043033 | 3 | amp |

| | | | | | | |
|---|---|---|---|---|---|---|
| TCGA-04-1361 | 10 | 95119542 | 95148968 | 1.102613918 | 4 | amp |
| TCGA-04-1361 | 10 | 95152632 | 98355422 | 1.289770884 | 5 | amp |
| TCGA-04-1361 | 10 | 98361975 | 98408633 | 1.104370369 | 4 | amp |
| TCGA-04-1361 | 10 | 98410950 | 98761167 | 0.877037737 | 3 | amp |
| TCGA-04-1361 | 10 | 100242315 | 101579055 | 1.204065081 | 4 | amp |
| TCGA-04-1361 | 10 | 101590006 | 102733394 | 0.879387224 | 3 | amp |
| TCGA-04-1361 | 10 | 102896408 | 104130660 | 0.862817264 | 3 | amp |
| TCGA-04-1361 | 10 | 104445545 | 105822111 | 0.778222437 | 3 | amp |
| TCGA-04-1361 | 10 | 105823480 | 105893579 | 1.092547417 | 4 | amp |
| TCGA-04-1361 | 10 | 105900589 | 115657987 | 1.252451583 | 5 | amp |
| TCGA-04-1361 | 10 | 115661390 | 115987751 | 1.764388846 | 6 | amp |
| TCGA-04-1361 | 10 | 115987780 | 116199904 | 0.672903961 | 3 | amp |
| TCGA-04-1361 | 10 | 116200745 | 116605242 | 1.254665145 | 5 | amp |
| TCGA-04-1361 | 10 | 116605750 | 117607504 | 1.665385088 | 6 | amp |
| TCGA-04-1361 | 10 | 117704109 | 118891826 | 1.35802886 | 5 | amp |
| TCGA-04-1361 | 10 | 118891909 | 120832603 | 1.083163966 | 4 | amp |
| TCGA-04-1361 | 10 | 120832900 | 121296038 | 0.861779457 | 3 | amp |
| TCGA-04-1361 | 10 | 121335119 | 123722857 | 1.325344128 | 5 | amp |
| TCGA-04-1361 | 10 | 123724790 | 124189476 | 1.023163596 | 4 | amp |
| TCGA-04-1361 | 10 | 124214197 | 126395303 | 0.942455188 | 3 | amp |
| TCGA-04-1361 | 10 | 126449011 | 127442458 | 1.16051561 | 4 | amp |
| TCGA-04-1361 | 10 | 127451835 | 134038848 | 0.876846623 | 3 | amp |
| TCGA-04-1361 | 10 | 135215003 | 135516111 | 0.876915894 | 3 | amp |
| TCGA-04-1361 | 11 | 3435159 | 6191610 | 0.861454673 | 4 | amp |
| TCGA-04-1361 | 11 | 6806263 | 30601976 | 0.878796847 | 4 | amp |
| TCGA-04-1361 | 11 | 30900074 | 33120757 | 1.38183767 | 5 | amp |
| TCGA-04-1361 | 11 | 33121148 | 33808690 | 1.809656218 | 6 | amp |
| TCGA-04-1361 | 11 | 33880891 | 43904746 | 1.46336855 | 5 | amp |
| TCGA-04-1361 | 11 | 43905489 | 46751140 | 0.890879107 | 4 | amp |
| TCGA-04-1361 | 11 | 46760572 | 46893189 | 1.511912301 | 5 | amp |
| TCGA-04-1361 | 11 | 46894567 | 47074089 | 0.775585508 | 4 | amp |
| TCGA-04-1361 | 11 | 47470270 | 56954956 | 1.362258607 | 5 | amp |
| TCGA-04-1361 | 11 | 57003276 | 57561577 | 0.843310566 | 4 | amp |
| TCGA-04-1361 | 11 | 57563004 | 60184504 | 1.365941397 | 5 | amp |
| TCGA-04-1361 | 11 | 60197103 | 60714328 | 0.827392504 | 4 | amp |
| TCGA-04-1361 | 11 | 61719234 | 62782443 | 0.791577539 | 4 | amp |
| TCGA-04-1361 | 11 | 62931210 | 63233892 | 1.682431431 | 6 | amp |
| TCGA-04-1361 | 11 | 63235792 | 63521216 | 0.871641935 | 4 | amp |
| TCGA-04-1361 | 11 | 67829400 | 67957583 | 1.625025922 | 6 | amp |
| TCGA-04-1361 | 11 | 68029089 | 68514874 | 0.945013889 | 4 | amp |
| TCGA-04-1361 | 11 | 69954424 | 72296677 | 0.837665644 | 4 | amp |
| TCGA-04-1361 | 11 | 72551902 | 73718144 | 1.327002898 | 5 | amp |
| TCGA-04-1361 | 11 | 73745587 | 74351829 | 2.08106001 | 6 | amp |
| TCGA-04-1361 | 11 | 74407526 | 74862494 | 1.613627657 | 5 | amp |
| TCGA-04-1361 | 11 | 74873614 | 75279884 | 0.676309685 | 4 | amp |
| TCGA-04-1361 | 11 | 75279979 | 76207565 | 1.400877098 | 5 | amp |
| TCGA-04-1361 | 11 | 76224384 | 76731404 | 2.178366934 | 6 | amp |
| TCGA-04-1361 | 11 | 76928286 | 77838502 | 2.432681629 | 6 | amp |
| TCGA-04-1361 | 11 | 77850526 | 77938103 | 1.249171182 | 5 | amp |
| TCGA-04-1361 | 11 | 77961114 | 107949034 | 2.06581031 | 6 | amp |

| | | | | | | |
|---|---|---|---|---|---|---|
| TCGA-04-1361 | 11 | 107959199 | 108225629 | 1.200143551 | 5 | amp |
| TCGA-04-1361 | 11 | 108235756 | 113577004 | 0.753323035 | 4 | amp |
| TCGA-04-1361 | 11 | 113604355 | 116633985 | 1.215552329 | 5 | amp |
| TCGA-04-1361 | 11 | 116636059 | 117034666 | 0.884681928 | 4 | amp |
| TCGA-04-1361 | 11 | 117860125 | 118047171 | 0.916730275 | 4 | amp |
| TCGA-04-1361 | 11 | 118065025 | 118263651 | 1.345614958 | 5 | amp |
| TCGA-04-1361 | 11 | 118266971 | 118471444 | 0.927361728 | 4 | amp |
| TCGA-04-1361 | 11 | 120276778 | 120331483 | 1.823230412 | 6 | amp |
| TCGA-04-1361 | 11 | 120335858 | 124440994 | 1.168115674 | 5 | amp |
| TCGA-04-1361 | 11 | 124481422 | 124564390 | 1.022275436 | 4 | amp |
| TCGA-04-1361 | 11 | 125447372 | 125893393 | 1.21479016 | 5 | amp |
| TCGA-04-1361 | 11 | 126073252 | 129753118 | 0.801370395 | 4 | amp |
| TCGA-04-1361 | 12 | 73256 | 347193 | 0.647968343 | 3 | amp |
| TCGA-04-1361 | 12 | 351729 | 443617 | 1.696125808 | 6 | amp |
| TCGA-04-1361 | 12 | 459745 | 645482 | 1.221510827 | 5 | amp |
| TCGA-04-1361 | 12 | 653492 | 923013 | 0.668059329 | 3 | amp |
| TCGA-04-1361 | 12 | 936167 | 1372385 | 1.314538064 | 5 | amp |
| TCGA-04-1361 | 12 | 1398947 | 2706485 | 0.949549564 | 4 | amp |
| TCGA-04-1361 | 12 | 2706572 | 4554743 | 0.732921506 | 3 | amp |
| TCGA-04-1361 | 12 | 4598958 | 4855426 | 1.301770033 | 5 | amp |
| TCGA-04-1361 | 12 | 4870066 | 6059060 | 0.929032743 | 4 | amp |
| TCGA-04-1361 | 12 | 6060969 | 6697645 | 0.69853425 | 3 | amp |
| TCGA-04-1361 | 12 | 6700570 | 6711143 | 1.16756364 | 5 | amp |
| TCGA-04-1361 | 12 | 6979648 | 7303332 | 0.72209823 | 3 | amp |
| TCGA-04-1361 | 12 | 7303441 | 7470819 | 1.12463123 | 5 | amp |
| TCGA-04-1361 | 12 | 7473300 | 7640291 | 1.702580388 | 6 | amp |
| TCGA-04-1361 | 12 | 7640315 | 7656323 | 1.478897778 | 5 | amp |
| TCGA-04-1361 | 12 | 7802097 | 9360955 | 1.066412951 | 4 | amp |
| TCGA-04-1361 | 12 | 9392014 | 9596322 | 0.678909745 | 3 | amp |
| TCGA-04-1361 | 12 | 9633110 | 10275992 | 1.365454306 | 5 | amp |
| TCGA-04-1361 | 12 | 10277851 | 16055962 | 0.935840726 | 4 | amp |
| TCGA-04-1361 | 12 | 16109647 | 19665425 | 0.623244584 | 3 | amp |
| TCGA-04-1361 | 12 | 19667601 | 27554651 | 1.039947564 | 4 | amp |
| TCGA-04-1361 | 12 | 27555490 | 29725154 | 1.700547721 | 6 | amp |
| TCGA-04-1361 | 12 | 29736273 | 31116976 | 1.280623585 | 5 | amp |
| TCGA-04-1361 | 12 | 31131472 | 31270182 | 0.574447442 | 3 | amp |
| TCGA-04-1361 | 12 | 31278213 | 31819205 | 1.338752435 | 5 | amp |
| TCGA-04-1361 | 12 | 31820572 | 40703073 | 1.796053728 | 6 | amp |
| TCGA-04-1361 | 12 | 40704191 | 45784302 | 1.360968266 | 5 | amp |
| TCGA-04-1361 | 12 | 45795570 | 46623444 | 1.632332205 | 6 | amp |
| TCGA-04-1361 | 12 | 46633414 | 48091560 | 1.363116138 | 5 | amp |
| TCGA-04-1361 | 12 | 48439075 | 49110497 | 1.071286604 | 4 | amp |
| TCGA-04-1361 | 12 | 50055700 | 50503313 | 0.697310439 | 3 | amp |
| TCGA-04-1361 | 12 | 50513793 | 51128944 | 1.311352864 | 5 | amp |
| TCGA-04-1361 | 12 | 51130765 | 51681991 | 1.020440737 | 4 | amp |
| TCGA-04-1361 | 12 | 51685337 | 51773619 | 0.670055903 | 3 | amp |
| TCGA-04-1361 | 12 | 51834472 | 52174613 | 1.193698093 | 5 | amp |
| TCGA-04-1361 | 12 | 53007433 | 53400333 | 0.6442496 | 3 | amp |
| TCGA-04-1361 | 12 | 53410205 | 53428504 | 1.268605978 | 5 | amp |
| TCGA-04-1361 | 12 | 53879815 | 54891684 | 0.708648323 | 3 | amp |

| | | | | | | |
|---|---|---|---|---|---|---|
| TCGA-04-1361 | 12 | 54893133 | 54936473 | 1.299183153 | 5 | amp |
| TCGA-04-1361 | 12 | 54943622 | 54978133 | 0.527739575 | 3 | amp |
| TCGA-04-1361 | 12 | 55024646 | 56031626 | 1.243468787 | 5 | amp |
| TCGA-04-1361 | 12 | 56666832 | 57409584 | 0.738584163 | 3 | amp |
| TCGA-04-1361 | 12 | 57422481 | 57458535 | 1.222260642 | 5 | amp |
| TCGA-04-1361 | 12 | 58158743 | 58191781 | 0.73118355 | 3 | amp |
| TCGA-04-1361 | 12 | 58193520 | 59274702 | 0.950323835 | 4 | amp |
| TCGA-04-1361 | 12 | 59276612 | 65640143 | 1.757680435 | 6 | amp |
| TCGA-04-1361 | 12 | 65702337 | 66859248 | 1.271481973 | 5 | amp |
| TCGA-04-1361 | 12 | 66909321 | 68715416 | 1.607222817 | 6 | amp |
| TCGA-04-1361 | 12 | 68716816 | 71519200 | 1.339816958 | 5 | amp |
| TCGA-04-1361 | 12 | 71523092 | 75676138 | 1.6377597 | 6 | amp |
| TCGA-04-1361 | 12 | 75678680 | 76462791 | 1.310846103 | 5 | amp |
| TCGA-04-1361 | 12 | 76467889 | 103233034 | 0.990057938 | 4 | amp |
| TCGA-04-1361 | 12 | 103234175 | 104373909 | 0.738349983 | 3 | amp |
| TCGA-04-1361 | 12 | 104374615 | 108169554 | 0.944752045 | 4 | amp |
| TCGA-04-1361 | 12 | 108589530 | 109528032 | 0.637350328 | 3 | amp |
| TCGA-04-1361 | 12 | 110350776 | 113448293 | 0.749285773 | 3 | amp |
| TCGA-04-1361 | 12 | 116403817 | 120295534 | 0.694490032 | 3 | amp |
| TCGA-04-1361 | 12 | 122812481 | 122962506 | 0.692552767 | 3 | amp |
| TCGA-04-1361 | 12 | 122962735 | 123107201 | 0.992453091 | 4 | amp |
| TCGA-04-1361 | 12 | 123109129 | 124320133 | 0.644831905 | 3 | amp |
| TCGA-04-1361 | 13 | 19255757 | 19972080 | 0.73243582 | 3 | amp |
| TCGA-04-1361 | 13 | 19981887 | 22067499 | 0.88623751 | 4 | amp |
| TCGA-04-1361 | 13 | 22069258 | 23411349 | 0.688373142 | 3 | amp |
| TCGA-04-1361 | 13 | 23777810 | 23930181 | 1.614966959 | 5 | amp |
| TCGA-04-1361 | 13 | 23932426 | 24384085 | 0.876099813 | 4 | amp |
| TCGA-04-1361 | 13 | 24410343 | 25341516 | 0.666828471 | 3 | amp |
| TCGA-04-1361 | 13 | 25348906 | 25440371 | 1.058142614 | 4 | amp |
| TCGA-04-1361 | 13 | 25442675 | 25672274 | 0.613455773 | 3 | amp |
| TCGA-04-1361 | 13 | 25743700 | 27246187 | 0.93975133 | 4 | amp |
| TCGA-04-1361 | 13 | 27250653 | 28599098 | 0.70442989 | 3 | amp |
| TCGA-04-1361 | 13 | 28601181 | 29287731 | 0.918895503 | 4 | amp |
| TCGA-04-1361 | 13 | 29291920 | 30075342 | 0.575384453 | 3 | amp |
| TCGA-04-1361 | 13 | 30077116 | 41333282 | 0.946122596 | 4 | amp |
| TCGA-04-1361 | 13 | 41340845 | 41429089 | 0.434089655 | 3 | amp |
| TCGA-04-1361 | 13 | 41507502 | 52333950 | 0.939201866 | 4 | amp |
| TCGA-04-1361 | 13 | 52345480 | 53236870 | 0.65921318 | 3 | amp |
| TCGA-04-1361 | 13 | 53237178 | 60241028 | 0.825251987 | 4 | amp |
| TCGA-04-1361 | 13 | 60348234 | 73573166 | 1.106944823 | 5 | amp |
| TCGA-04-1361 | 13 | 73589971 | 77785460 | 1.008260055 | 4 | amp |
| TCGA-04-1361 | 13 | 77786072 | 78208609 | 1.148652094 | 5 | amp |
| TCGA-04-1361 | 13 | 78211231 | 96676067 | 0.997677917 | 4 | amp |
| TCGA-04-1361 | 13 | 96684123 | 99515389 | 0.717734192 | 3 | amp |
| TCGA-04-1361 | 13 | 99515592 | 101020836 | 0.941655232 | 4 | amp |
| TCGA-04-1361 | 13 | 101077875 | 101726042 | 0.640001138 | 3 | amp |
| TCGA-04-1361 | 13 | 101726818 | 109699361 | 0.885379439 | 4 | amp |
| TCGA-04-1361 | 13 | 109700633 | 109707960 | 0.709938195 | 3 | amp |
| TCGA-04-1361 | 14 | 19377543 | 20764059 | 1.211660396 | 5 | amp |
| TCGA-04-1361 | 14 | 20764486 | 20779939 | 1.070067853 | 4 | amp |

| | | | | | | |
|---|---|---|---|---|---|---|
| TCGA-04-1361 | 14 | 20781613 | 20979286 | 0.660611925 | 3 | amp |
| TCGA-04-1361 | 14 | 21024529 | 21557069 | 0.878024464 | 4 | amp |
| TCGA-04-1361 | 14 | 21623039 | 22945629 | 1.286234657 | 5 | amp |
| TCGA-04-1361 | 14 | 22946666 | 23243757 | 0.997311636 | 4 | amp |
| TCGA-04-1361 | 14 | 23244610 | 23900218 | 0.640112309 | 3 | amp |
| TCGA-04-1361 | 14 | 23900568 | 23947285 | 1.117964335 | 4 | amp |
| TCGA-04-1361 | 14 | 24975622 | 25102314 | 0.742104173 | 3 | amp |
| TCGA-04-1361 | 14 | 25281836 | 34396262 | 1.448780406 | 5 | amp |
| TCGA-04-1361 | 14 | 34398229 | 36017800 | 0.993013195 | 4 | amp |
| TCGA-04-1361 | 14 | 36018257 | 51223273 | 1.188579203 | 5 | amp |
| TCGA-04-1361 | 14 | 51223729 | 52922230 | 0.982058682 | 4 | amp |
| TCGA-04-1361 | 14 | 52923789 | 55480318 | 1.193257627 | 5 | amp |
| TCGA-04-1361 | 14 | 55493406 | 55907308 | 0.958961753 | 4 | amp |
| TCGA-04-1361 | 14 | 56078720 | 64484513 | 1.215687409 | 5 | amp |
| TCGA-04-1361 | 14 | 64486700 | 65067059 | 0.922944813 | 4 | amp |
| TCGA-04-1361 | 14 | 65390656 | 66191072 | 0.770558297 | 3 | amp |
| TCGA-04-1361 | 14 | 66199903 | 67805528 | 1.028006373 | 4 | amp |
| TCGA-04-1361 | 14 | 67807135 | 68276021 | 0.736400673 | 3 | amp |
| TCGA-04-1361 | 14 | 68280681 | 71455469 | 0.933314872 | 4 | amp |
| TCGA-04-1361 | 14 | 71462429 | 72128225 | 1.406189116 | 5 | amp |
| TCGA-04-1361 | 14 | 72137792 | 73007027 | 1.065883216 | 4 | amp |
| TCGA-04-1361 | 14 | 73007247 | 74327420 | 0.730757846 | 3 | amp |
| TCGA-04-1361 | 14 | 74340701 | 74535743 | 0.953512422 | 4 | amp |
| TCGA-04-1361 | 14 | 74537838 | 75070432 | 0.66460299 | 3 | amp |
| TCGA-04-1361 | 14 | 75130383 | 76165631 | 0.965338826 | 4 | amp |
| TCGA-04-1361 | 14 | 76173335 | 76286546 | 1.384615746 | 5 | amp |
| TCGA-04-1361 | 14 | 76329957 | 77808342 | 0.776500841 | 3 | amp |
| TCGA-04-1361 | 14 | 77809493 | 77917722 | 1.101049445 | 4 | amp |
| TCGA-04-1361 | 14 | 77919602 | 77951320 | 0.541790223 | 3 | amp |
| TCGA-04-1361 | 14 | 77978566 | 78189631 | 1.083619921 | 4 | amp |
| TCGA-04-1361 | 14 | 78197281 | 89747385 | 1.308015495 | 5 | amp |
| TCGA-04-1361 | 14 | 89816963 | 91633765 | 0.973247219 | 4 | amp |
| TCGA-04-1361 | 14 | 91636295 | 91810031 | 0.590908492 | 3 | amp |
| TCGA-04-1361 | 14 | 91825960 | 92604693 | 1.087216738 | 4 | amp |
| TCGA-04-1361 | 14 | 92608481 | 92628101 | 1.667108211 | 6 | amp |
| TCGA-04-1361 | 14 | 92792206 | 93715030 | 0.719822736 | 3 | amp |
| TCGA-04-1361 | 14 | 93717783 | 94160779 | 1.200136004 | 4 | amp |
| TCGA-04-1361 | 14 | 94170931 | 94697675 | 0.619649338 | 3 | amp |
| TCGA-04-1361 | 14 | 94699982 | 94733390 | 1.410695965 | 5 | amp |
| TCGA-04-1361 | 14 | 94741664 | 95111388 | 0.766710328 | 3 | amp |
| TCGA-04-1361 | 14 | 95192939 | 95679770 | 1.09055784 | 4 | amp |
| TCGA-04-1361 | 14 | 95681963 | 96103158 | 0.602176394 | 3 | amp |
| TCGA-04-1361 | 14 | 96128832 | 96783064 | 1.031234737 | 4 | amp |
| TCGA-04-1361 | 14 | 96783417 | 99929928 | 1.371872281 | 5 | amp |
| TCGA-04-1361 | 14 | 99932030 | 101393773 | 0.607245188 | 3 | amp |
| TCGA-04-1361 | 14 | 101396239 | 101459669 | 1.610285161 | 6 | amp |
| TCGA-04-1361 | 14 | 102372652 | 104224097 | 0.696988672 | 3 | amp |
| TCGA-04-1361 | 14 | 104245059 | 104493325 | 1.087840773 | 4 | amp |
| TCGA-04-1361 | 14 | 106382639 | 106963403 | 0.763124926 | 3 | amp |
| TCGA-04-1361 | 14 | 106967019 | 107049157 | 1.301580052 | 5 | amp |

| | | | | | | |
|---|---|---|---|---|---|---|
| TCGA-04-1361 | 14 | 107062097 | 107283263 | 0.709804199 | 3 | amp |
| TCGA-04-1361 | 15 | 20169886 | 20778092 | 0.693470374 | 3 | amp |
| TCGA-04-1361 | 15 | 20832064 | 22414056 | 1.350360167 | 5 | amp |
| TCGA-04-1361 | 15 | 22466023 | 24924489 | 0.730720505 | 3 | amp |
| TCGA-04-1361 | 15 | 25154454 | 25422079 | 1.209792486 | 4 | amp |
| TCGA-04-1361 | 15 | 25495986 | 25616995 | 1.458087083 | 5 | amp |
| TCGA-04-1361 | 15 | 25620580 | 30923000 | 0.761682946 | 3 | amp |
| TCGA-04-1361 | 15 | 30925680 | 31320726 | 1.140140384 | 4 | amp |
| TCGA-04-1361 | 15 | 31321498 | 32892455 | 0.73012336 | 3 | amp |
| TCGA-04-1361 | 15 | 32894706 | 34655069 | 1.129722324 | 4 | amp |
| TCGA-04-1361 | 15 | 35148969 | 39544912 | 1.266152643 | 5 | amp |
| TCGA-04-1361 | 15 | 39874001 | 40512982 | 1.07470174 | 4 | amp |
| TCGA-04-1361 | 15 | 40898560 | 40947200 | 1.341158378 | 5 | amp |
| TCGA-04-1361 | 15 | 40949317 | 41279399 | 0.62847059 | 3 | amp |
| TCGA-04-1361 | 15 | 41279942 | 41650461 | 1.245138413 | 4 | amp |
| TCGA-04-1361 | 15 | 41657573 | 41866064 | 0.583151938 | 3 | amp |
| TCGA-04-1361 | 15 | 41870008 | 42092126 | 1.234902406 | 4 | amp |
| TCGA-04-1361 | 15 | 42447660 | 42644342 | 1.31888287 | 5 | amp |
| TCGA-04-1361 | 15 | 42646535 | 43016737 | 1.132705625 | 4 | amp |
| TCGA-04-1361 | 15 | 43037983 | 43473557 | 1.226491709 | 5 | amp |
| TCGA-04-1361 | 15 | 43476440 | 44062547 | 0.798930654 | 3 | amp |
| TCGA-04-1361 | 15 | 44105144 | 45270876 | 1.244295315 | 5 | amp |
| TCGA-04-1361 | 15 | 45332562 | 45394208 | 0.727374016 | 3 | amp |
| TCGA-04-1361 | 15 | 45467399 | 51795230 | 1.402418837 | 5 | amp |
| TCGA-04-1361 | 15 | 51799270 | 51984564 | 1.811892813 | 6 | amp |
| TCGA-04-1361 | 15 | 51988009 | 56471544 | 1.301271391 | 5 | amp |
| TCGA-04-1361 | 15 | 56657571 | 57565463 | 1.795956307 | 6 | amp |
| TCGA-04-1361 | 15 | 57574593 | 59383365 | 1.264493239 | 5 | amp |
| TCGA-04-1361 | 15 | 59384727 | 60648217 | 0.932904021 | 4 | amp |
| TCGA-04-1361 | 15 | 60649329 | 62234293 | 1.4064792 | 5 | amp |
| TCGA-04-1361 | 15 | 62237847 | 62284003 | 2.013630162 | 6 | amp |
| TCGA-04-1361 | 15 | 62292733 | 62966117 | 1.471322104 | 5 | amp |
| TCGA-04-1361 | 15 | 62967407 | 63131232 | 0.864722149 | 3 | amp |
| TCGA-04-1361 | 15 | 63132594 | 64050590 | 1.914717918 | 6 | amp |
| TCGA-04-1361 | 15 | 64056282 | 65236956 | 1.214157488 | 5 | amp |
| TCGA-04-1361 | 15 | 65242007 | 65459165 | 2.204610392 | 6 | amp |
| TCGA-04-1361 | 15 | 65471184 | 65563461 | 1.266682309 | 5 | amp |
| TCGA-04-1361 | 15 | 65621156 | 65693319 | 0.552384054 | 3 | amp |
| TCGA-04-1361 | 15 | 65702436 | 66191288 | 2.246891936 | 6 | amp |
| TCGA-04-1361 | 15 | 66198390 | 67636584 | 1.242595583 | 5 | amp |
| TCGA-04-1361 | 15 | 67649660 | 68099265 | 1.945937828 | 6 | amp |
| TCGA-04-1361 | 15 | 68123101 | 68126186 | 0.766292767 | 3 | amp |
| TCGA-04-1361 | 15 | 68378625 | 68466269 | 2.443914534 | 6 | amp |
| TCGA-04-1361 | 15 | 68467919 | 69347876 | 0.707288377 | 3 | amp |
| TCGA-04-1361 | 15 | 69348850 | 70368525 | 1.047284574 | 4 | amp |
| TCGA-04-1361 | 15 | 70949370 | 70957196 | 1.398705079 | 5 | amp |
| TCGA-04-1361 | 15 | 70959051 | 70994294 | 2.088492156 | 6 | amp |
| TCGA-04-1361 | 15 | 71124352 | 72122670 | 1.423265617 | 5 | amp |
| TCGA-04-1361 | 15 | 72141127 | 72324941 | 2.019341609 | 6 | amp |
| TCGA-04-1361 | 15 | 72338004 | 72690767 | 1.245320263 | 5 | amp |

| | | | | | | |
|---|---|---|---|---|---|---|
| TCGA-04-1361 | 15 | 72690941 | 72879665 | 1.97519029 | 6 | amp |
| TCGA-04-1361 | 15 | 72931429 | 72987603 | 0.676844777 | 3 | amp |
| TCGA-04-1361 | 15 | 73001990 | 73562845 | 1.930621648 | 6 | amp |
| TCGA-04-1361 | 15 | 73564789 | 74003553 | 1.39872591 | 5 | amp |
| TCGA-04-1361 | 15 | 75651918 | 75913433 | 1.046274482 | 4 | amp |
| TCGA-04-1361 | 15 | 75931931 | 76077945 | 0.808465129 | 3 | amp |
| TCGA-04-1361 | 15 | 76146717 | 76303626 | 1.886710426 | 6 | amp |
| TCGA-04-1361 | 15 | 76426533 | 76914197 | 1.057341863 | 5 | amp |
| TCGA-04-1361 | 15 | 76957941 | 77287992 | 1.96185145 | 6 | amp |
| TCGA-04-1361 | 15 | 77317581 | 78207589 | 1.383961238 | 5 | amp |
| TCGA-04-1361 | 15 | 78207696 | 78732239 | 0.834186608 | 3 | amp |
| TCGA-04-1361 | 15 | 78755226 | 79187263 | 1.446918825 | 5 | amp |
| TCGA-04-1361 | 15 | 79189306 | 79356874 | 0.69157394 | 3 | amp |
| TCGA-04-1361 | 15 | 79382522 | 81199211 | 1.108410036 | 4 | amp |
| TCGA-04-1361 | 15 | 81201422 | 81660691 | 0.837081575 | 3 | amp |
| TCGA-04-1361 | 15 | 81664884 | 82533797 | 1.98736994 | 6 | amp |
| TCGA-04-1361 | 15 | 82545013 | 83360546 | 0.74507849 | 3 | amp |
| TCGA-04-1361 | 15 | 83424639 | 83679171 | 1.088747192 | 4 | amp |
| TCGA-04-1361 | 15 | 83686747 | 83788441 | 1.357697261 | 5 | amp |
| TCGA-04-1361 | 15 | 83790653 | 84795403 | 1.890752795 | 6 | amp |
| TCGA-04-1361 | 15 | 84859539 | 85488453 | 0.736233855 | 3 | amp |
| TCGA-04-1361 | 15 | 85607568 | 85661090 | 2.240296643 | 6 | amp |
| TCGA-04-1361 | 15 | 85663986 | 86313827 | 1.452655348 | 5 | amp |
| TCGA-04-1361 | 15 | 86686894 | 87217705 | 1.918073384 | 6 | amp |
| TCGA-04-1361 | 15 | 87531208 | 89011040 | 1.342758282 | 5 | amp |
| TCGA-04-1361 | 15 | 89011076 | 89762292 | 0.696697528 | 3 | amp |
| TCGA-04-1361 | 15 | 89790830 | 89825090 | 1.900945971 | 6 | amp |
| TCGA-04-1361 | 15 | 89826363 | 89848695 | 1.434553648 | 5 | amp |
| TCGA-04-1361 | 15 | 89848792 | 90281489 | 1.111403026 | 4 | amp |
| TCGA-04-1361 | 15 | 90286482 | 90934115 | 0.710317355 | 3 | amp |
| TCGA-04-1361 | 15 | 90969299 | 90999585 | 1.892182415 | 6 | amp |
| TCGA-04-1361 | 15 | 91009217 | 91420309 | 1.421445408 | 5 | amp |
| TCGA-04-1361 | 15 | 91517767 | 93480891 | 1.197589095 | 5 | amp |
| TCGA-04-1361 | 15 | 93482727 | 95013676 | 1.805597969 | 6 | amp |
| TCGA-04-1361 | 15 | 95019913 | 100230659 | 1.211840286 | 5 | amp |
| TCGA-04-1361 | 15 | 100230717 | 100739635 | 0.7504321 | 3 | amp |
| TCGA-04-1361 | 15 | 100794302 | 101121087 | 1.740054388 | 6 | amp |
| TCGA-04-1361 | 15 | 101152406 | 102190405 | 0.765337812 | 3 | amp |
| TCGA-04-1361 | 15 | 102191877 | 102513951 | 1.206925127 | 4 | amp |
| TCGA-04-1361 | 16 | 3293117 | 3594347 | 0.727473499 | 3 | amp |
| TCGA-04-1361 | 16 | 4774701 | 5145543 | 0.605444391 | 3 | amp |
| TCGA-04-1361 | 16 | 7102025 | 8844457 | 1.183007981 | 5 | amp |
| TCGA-04-1361 | 16 | 8851578 | 8996348 | 0.658861525 | 3 | amp |
| TCGA-04-1361 | 16 | 8997041 | 9004702 | 0.991617286 | 4 | amp |
| TCGA-04-1361 | 16 | 9009095 | 10576139 | 1.279399925 | 5 | amp |
| TCGA-04-1361 | 16 | 10626705 | 11824638 | 0.759744833 | 3 | amp |
| TCGA-04-1361 | 16 | 11827826 | 11850279 | 0.934187691 | 4 | amp |
| TCGA-04-1361 | 16 | 11852280 | 14802920 | 1.18143793 | 5 | amp |
| TCGA-04-1361 | 16 | 14809868 | 14978344 | 0.969643012 | 4 | amp |
| TCGA-04-1361 | 16 | 14980608 | 15696073 | 0.774334701 | 3 | amp |

| | | | | | | |
|---|---|---|---|---|---|---|
| TCGA-04-1361 | 16 | 15697989 | 15705639 | 0.881118304 | 4 | amp |
| TCGA-04-1361 | 16 | 15706373 | 15728820 | 1.376324119 | 5 | amp |
| TCGA-04-1361 | 16 | 15729495 | 18518310 | 0.768360194 | 3 | amp |
| TCGA-04-1361 | 16 | 18520477 | 18820989 | 0.910730441 | 4 | amp |
| TCGA-04-1361 | 16 | 18823075 | 21093062 | 1.153096363 | 5 | amp |
| TCGA-04-1361 | 16 | 21098155 | 21152063 | 1.53037302 | 6 | amp |
| TCGA-04-1361 | 16 | 21152596 | 22130405 | 1.116149008 | 5 | amp |
| TCGA-04-1361 | 16 | 22132236 | 22444160 | 0.716113616 | 3 | amp |
| TCGA-04-1361 | 16 | 22445919 | 23085241 | 0.975016388 | 4 | amp |
| TCGA-04-1361 | 16 | 23091259 | 23197930 | 1.459021566 | 5 | amp |
| TCGA-04-1361 | 16 | 23200631 | 23721588 | 0.727648284 | 3 | amp |
| TCGA-04-1361 | 16 | 23721763 | 24046923 | 0.905326063 | 4 | amp |
| TCGA-04-1361 | 16 | 24104069 | 24583564 | 1.132346496 | 5 | amp |
| TCGA-04-1361 | 16 | 24761936 | 24818119 | 1.557982778 | 6 | amp |
| TCGA-04-1361 | 16 | 24820616 | 25186366 | 1.170242034 | 5 | amp |
| TCGA-04-1361 | 16 | 25189272 | 26147610 | 0.727417377 | 3 | amp |
| TCGA-04-1361 | 16 | 27518112 | 28847843 | 0.691890894 | 3 | amp |
| TCGA-04-1361 | 16 | 28947437 | 29395657 | 0.680835388 | 3 | amp |
| TCGA-04-1361 | 16 | 29396824 | 29415123 | 0.932887698 | 4 | amp |
| TCGA-04-1361 | 16 | 29437179 | 29447035 | 1.898792723 | 6 | amp |
| TCGA-04-1361 | 16 | 29447144 | 29458354 | 1.25837744 | 5 | amp |
| TCGA-04-1361 | 16 | 29464943 | 29516913 | 0.650181418 | 3 | amp |
| TCGA-04-1361 | 16 | 29538947 | 29577608 | 1.115085054 | 4 | amp |
| TCGA-04-1361 | 16 | 29624392 | 29842416 | 0.616279427 | 3 | amp |
| TCGA-04-1361 | 16 | 30215124 | 30317312 | 0.983456326 | 3 | amp |
| TCGA-04-1361 | 16 | 31895761 | 33816189 | 0.810258947 | 3 | amp |
| TCGA-04-1361 | 16 | 34324862 | 47120253 | 0.979211018 | 4 | amp |
| TCGA-04-1361 | 16 | 47143387 | 48117728 | 1.496726263 | 5 | amp |
| TCGA-04-1361 | 16 | 48117781 | 48368317 | 0.959036069 | 4 | amp |
| TCGA-04-1361 | 16 | 48381401 | 50353989 | 0.744125759 | 3 | amp |
| TCGA-04-1361 | 16 | 50354080 | 50388424 | 1.366296272 | 5 | amp |
| TCGA-04-1361 | 16 | 50388618 | 50810198 | 0.71207446 | 4 | amp |
| TCGA-04-1361 | 16 | 50811673 | 53348965 | 1.555843957 | 6 | amp |
| TCGA-04-1361 | 16 | 53352067 | 53679965 | 1.137570436 | 5 | amp |
| TCGA-04-1361 | 16 | 53682837 | 53709041 | 1.704936215 | 6 | amp |
| TCGA-04-1361 | 16 | 53720296 | 54145870 | 1.265351167 | 5 | amp |
| TCGA-04-1361 | 16 | 54317564 | 55562517 | 0.738003636 | 4 | amp |
| TCGA-04-1361 | 16 | 55563742 | 56368824 | 1.152731969 | 5 | amp |
| TCGA-04-1361 | 16 | 56370587 | 56492521 | 0.698238819 | 3 | amp |
| TCGA-04-1361 | 16 | 56496435 | 56545253 | 1.299345286 | 5 | amp |
| TCGA-04-1361 | 16 | 56548358 | 56863074 | 0.683327548 | 3 | amp |
| TCGA-04-1361 | 16 | 56864398 | 56878525 | 1.297919633 | 5 | amp |
| TCGA-04-1361 | 16 | 56892381 | 57406575 | 0.614972558 | 3 | amp |
| TCGA-04-1361 | 16 | 58147871 | 58555273 | 0.772412349 | 3 | amp |
| TCGA-04-1361 | 16 | 58557219 | 58592597 | 1.606209643 | 6 | amp |
| TCGA-04-1361 | 16 | 58593647 | 58610546 | 1.197714251 | 5 | amp |
| TCGA-04-1361 | 16 | 58612542 | 58622816 | 1.009582488 | 4 | amp |
| TCGA-04-1361 | 16 | 58633130 | 65397177 | 0.844647693 | 3 | amp |
| TCGA-04-1361 | 16 | 70560534 | 72124702 | 0.715412505 | 3 | amp |
| TCGA-04-1361 | 16 | 72164376 | 81116579 | 0.661966804 | 3 | amp |

| | | | | | | |
|---|---|---|---|---|---|---|
| TCGA-04-1361 | 17 | 63618 | 680287 | 0.682336588 | 3 | amp |
| TCGA-04-1361 | 17 | 1559622 | 1581043 | 0.843779526 | 3 | amp |
| TCGA-04-1361 | 17 | 1731101 | 1798405 | 0.848474369 | 3 | amp |
| TCGA-04-1361 | 17 | 1985026 | 2583655 | 0.689229431 | 3 | amp |
| TCGA-04-1361 | 17 | 2965951 | 3343656 | 1.513325381 | 5 | amp |
| TCGA-04-1361 | 17 | 3346456 | 3424359 | 0.972766297 | 4 | amp |
| TCGA-04-1361 | 17 | 3716305 | 3725334 | 1.298570435 | 5 | amp |
| TCGA-04-1361 | 17 | 3922952 | 3981376 | 0.738521168 | 3 | amp |
| TCGA-04-1361 | 17 | 3984636 | 4027392 | 1.141255623 | 4 | amp |
| TCGA-04-1361 | 17 | 4098641 | 4210426 | 0.857758373 | 3 | amp |
| TCGA-04-1361 | 17 | 4856284 | 4873852 | 0.756169682 | 3 | amp |
| TCGA-04-1361 | 17 | 5039042 | 5045487 | 0.732141768 | 3 | amp |
| TCGA-04-1361 | 17 | 5045684 | 5314125 | 1.026393881 | 4 | amp |
| TCGA-04-1361 | 17 | 5317271 | 6329164 | 0.574401774 | 3 | amp |
| TCGA-04-1361 | 17 | 6428669 | 6538436 | 1.201310449 | 5 | amp |
| TCGA-04-1361 | 17 | 6545546 | 6684238 | 0.667259326 | 3 | amp |
| TCGA-04-1361 | 17 | 6690066 | 6719336 | 1.582495309 | 5 | amp |
| TCGA-04-1361 | 17 | 8272417 | 8726818 | 0.755164682 | 3 | amp |
| TCGA-04-1361 | 17 | 9281859 | 10215430 | 0.762958837 | 3 | amp |
| TCGA-04-1361 | 17 | 10215862 | 10254061 | 0.989987802 | 4 | amp |
| TCGA-04-1361 | 17 | 10257988 | 10267865 | 1.583401358 | 5 | amp |
| TCGA-04-1361 | 17 | 10297547 | 10364421 | 1.358523134 | 5 | amp |
| TCGA-04-1361 | 17 | 10366173 | 10409432 | 0.908059732 | 4 | amp |
| TCGA-04-1361 | 17 | 10411147 | 10451255 | 1.382407237 | 5 | amp |
| TCGA-04-1361 | 17 | 10531892 | 11840873 | 1.023349033 | 4 | amp |
| TCGA-04-1361 | 17 | 11845560 | 12608537 | 1.272673199 | 5 | amp |
| TCGA-04-1361 | 17 | 12618803 | 12859300 | 1.077092884 | 4 | amp |
| TCGA-04-1361 | 17 | 12859957 | 15217595 | 0.64938493 | 3 | amp |
| TCGA-04-1361 | 17 | 15222368 | 15458697 | 1.220952131 | 4 | amp |
| TCGA-04-1361 | 17 | 15468837 | 15643497 | 0.640114173 | 3 | amp |
| TCGA-04-1361 | 17 | 15644345 | 16253353 | 1.037018187 | 4 | amp |
| TCGA-04-1361 | 17 | 16347103 | 16705659 | 1.014807943 | 4 | amp |
| TCGA-04-1361 | 17 | 16721444 | 17179533 | 0.585367154 | 3 | amp |
| TCGA-04-1361 | 17 | 18418986 | 18486156 | 0.757039727 | 3 | amp |
| TCGA-04-1361 | 17 | 18486626 | 18862559 | 0.974231865 | 4 | amp |
| TCGA-04-1361 | 17 | 19289600 | 19582228 | 0.773136827 | 3 | amp |
| TCGA-04-1361 | 17 | 19685170 | 19813324 | 1.296761842 | 5 | amp |
| TCGA-04-1361 | 17 | 19823329 | 20243632 | 0.890046866 | 4 | amp |
| TCGA-04-1361 | 17 | 20245618 | 20334237 | 1.249619047 | 5 | amp |
| TCGA-04-1361 | 17 | 20619869 | 20796800 | 0.906541139 | 3 | amp |
| TCGA-04-1361 | 17 | 26938532 | 27012040 | 0.602386767 | 3 | amp |
| TCGA-04-1361 | 17 | 27778512 | 28618547 | 0.728394751 | 3 | amp |
| TCGA-04-1361 | 17 | 28643646 | 28837972 | 1.017901695 | 4 | amp |
| TCGA-04-1361 | 17 | 28846937 | 29369337 | 0.676082322 | 3 | amp |
| TCGA-04-1361 | 17 | 29372203 | 29684162 | 1.042748442 | 4 | amp |
| TCGA-04-1361 | 17 | 29684276 | 31072325 | 0.662580784 | 3 | amp |
| TCGA-04-1361 | 17 | 31075898 | 31103084 | 1.299739999 | 5 | amp |
| TCGA-04-1361 | 17 | 31105454 | 34433721 | 0.646280194 | 3 | amp |
| TCGA-04-1361 | 17 | 35343912 | 35508405 | 0.710042737 | 3 | amp |
| TCGA-04-1361 | 17 | 35512593 | 35734919 | 0.997161837 | 4 | amp |

| | | | | | | |
|---|---|---|---|---|---|---|
| TCGA-04-1361 | 17 | 35736137 | 36047444 | 0.767292831 | 3 | amp |
| TCGA-04-1361 | 17 | 37420413 | 37676394 | 0.725373418 | 3 | amp |
| TCGA-04-1361 | 17 | 38445611 | 38569236 | 0.724151706 | 3 | amp |
| TCGA-04-1361 | 17 | 38715109 | 39412202 | 0.690028613 | 3 | amp |
| TCGA-04-1361 | 17 | 40483459 | 40659739 | 0.756037709 | 3 | amp |
| TCGA-04-1361 | 17 | 41201114 | 41258571 | 0.801913083 | 3 | amp |
| TCGA-04-1361 | 17 | 44115857 | 45776893 | 0.687493655 | 3 | amp |
| TCGA-04-1361 | 17 | 46189366 | 46474154 | 0.883585456 | 3 | amp |
| TCGA-04-1361 | 17 | 47388625 | 47904873 | 0.688506168 | 3 | amp |
| TCGA-04-1361 | 17 | 48814293 | 54558223 | 0.974343699 | 4 | amp |
| TCGA-04-1361 | 17 | 54559662 | 55193666 | 0.685349708 | 3 | amp |
| TCGA-04-1361 | 17 | 56634329 | 57648043 | 0.747379052 | 3 | amp |
| TCGA-04-1361 | 17 | 57650416 | 58078684 | 0.932642391 | 4 | amp |
| TCGA-04-1361 | 17 | 58078871 | 58232761 | 0.591219455 | 3 | amp |
| TCGA-04-1361 | 17 | 58233848 | 60088612 | 0.929265792 | 4 | amp |
| TCGA-04-1361 | 17 | 60106896 | 60742349 | 0.796116523 | 3 | amp |
| TCGA-04-1361 | 17 | 60813272 | 61493081 | 0.77500628 | 3 | amp |
| TCGA-04-1361 | 17 | 61621540 | 61899555 | 0.651114911 | 3 | amp |
| TCGA-04-1361 | 17 | 62125161 | 62506404 | 0.58707671 | 3 | amp |
| TCGA-04-1361 | 17 | 62512833 | 62758710 | 0.954468078 | 4 | amp |
| TCGA-04-1361 | 17 | 62788443 | 63632150 | 0.639715093 | 3 | amp |
| TCGA-04-1361 | 17 | 63633213 | 64173162 | 1.043057927 | 4 | amp |
| TCGA-04-1361 | 17 | 64179304 | 66881516 | 0.7881388 | 3 | amp |
| TCGA-04-1361 | 17 | 66883154 | 67101805 | 1.018111687 | 4 | amp |
| TCGA-04-1361 | 17 | 67102140 | 67179002 | 1.276389992 | 5 | amp |
| TCGA-04-1361 | 17 | 67181550 | 67261080 | 0.991522331 | 4 | amp |
| TCGA-04-1361 | 17 | 67264080 | 68129516 | 1.214264823 | 5 | amp |
| TCGA-04-1361 | 17 | 78023656 | 78115673 | 0.755371381 | 3 | amp |
| TCGA-04-1361 | 17 | 80363137 | 80382384 | 1.174896361 | 5 | amp |
| TCGA-04-1361 | 18 | 47273 | 320114 | 2.036128486 | 6 | amp |
| TCGA-04-1361 | 18 | 321584 | 580967 | 1.380058357 | 5 | amp |
| TCGA-04-1361 | 18 | 617993 | 633486 | 2.422094915 | 6 | amp |
| TCGA-04-1361 | 18 | 641283 | 678776 | 1.396616175 | 5 | amp |
| TCGA-04-1361 | 18 | 683192 | 2784653 | 2.153218299 | 6 | amp |
| TCGA-04-1361 | 18 | 2796401 | 2951383 | 0.997774456 | 5 | amp |
| TCGA-04-1361 | 18 | 2954489 | 3845316 | 2.084914814 | 6 | amp |
| TCGA-04-1361 | 18 | 5290809 | 9204567 | 1.384834676 | 5 | amp |
| TCGA-04-1361 | 18 | 9208607 | 9275713 | 2.393231621 | 6 | amp |
| TCGA-04-1361 | 18 | 9279534 | 11825025 | 1.426334707 | 5 | amp |
| TCGA-04-1361 | 18 | 11851510 | 12264498 | 0.785894766 | 3 | amp |
| TCGA-04-1361 | 18 | 12274032 | 12421643 | 1.248384697 | 5 | amp |
| TCGA-04-1361 | 18 | 12427014 | 12678441 | 1.867613336 | 6 | amp |
| TCGA-04-1361 | 18 | 12680623 | 14513842 | 1.512030369 | 5 | amp |
| TCGA-04-1361 | 18 | 14522216 | 14533248 | 2.717270535 | 6 | amp |
| TCGA-04-1361 | 18 | 14534892 | 18540191 | 1.373396053 | 5 | amp |
| TCGA-04-1361 | 18 | 18546837 | 18586765 | 1.944164573 | 6 | amp |
| TCGA-04-1361 | 18 | 18587967 | 23937763 | 1.253777368 | 5 | amp |
| TCGA-04-1361 | 18 | 23969772 | 39542645 | 1.06198598 | 4 | amp |
| TCGA-04-1361 | 18 | 39550235 | 39620732 | 1.818547535 | 6 | amp |
| TCGA-04-1361 | 18 | 39623678 | 45567524 | 1.263367513 | 5 | amp |

| | | | | | | |
|---|---|---|---|---|---|---|
| TCGA-04-1361 | 18 | 46190099 | 46623888 | 0.672230016 | 3 | amp |
| TCGA-04-1361 | 18 | 46645084 | 46918048 | 2.219305857 | 6 | amp |
| TCGA-04-1361 | 18 | 46956574 | 47796670 | 1.410154793 | 5 | amp |
| TCGA-04-1361 | 18 | 47797800 | 48252558 | 0.916540165 | 3 | amp |
| TCGA-04-1361 | 18 | 48325673 | 48703993 | 2.184791045 | 6 | amp |
| TCGA-04-1361 | 18 | 49867112 | 50994441 | 1.237214569 | 5 | amp |
| TCGA-04-1361 | 18 | 51013127 | 71930767 | 1.055541062 | 4 | amp |
| TCGA-04-1361 | 19 | 6887574 | 6937435 | 0.987359513 | 4 | amp |
| TCGA-04-1361 | 19 | 8841352 | 9000623 | 0.6574667 | 3 | amp |
| TCGA-04-1361 | 19 | 9001803 | 9025684 | 0.971953576 | 4 | amp |
| TCGA-04-1361 | 19 | 9026132 | 9870636 | 0.738859493 | 3 | amp |
| TCGA-04-1361 | 19 | 13342478 | 13865189 | 0.75876457 | 3 | amp |
| TCGA-04-1361 | 19 | 14698404 | 14854631 | 1.000286606 | 4 | amp |
| TCGA-04-1361 | 19 | 14856999 | 15163135 | 0.627473405 | 3 | amp |
| TCGA-04-1361 | 19 | 15164221 | 15198821 | 1.442578545 | 5 | amp |
| TCGA-04-1361 | 19 | 15757828 | 16229125 | 0.660510938 | 3 | amp |
| TCGA-04-1361 | 19 | 19788615 | 19825352 | 1.668776495 | 6 | amp |
| TCGA-04-1361 | 19 | 19843675 | 19976866 | 0.700440043 | 3 | amp |
| TCGA-04-1361 | 19 | 19989232 | 21133168 | 1.142044401 | 4 | amp |
| TCGA-04-1361 | 19 | 21203547 | 30099649 | 1.32654884 | 5 | amp |
| TCGA-04-1361 | 19 | 30102717 | 30462176 | 0.571114352 | 3 | amp |
| TCGA-04-1361 | 19 | 30476078 | 30503494 | 1.413924037 | 5 | amp |
| TCGA-04-1361 | 19 | 30505764 | 32845879 | 0.718873095 | 3 | amp |
| TCGA-04-1361 | 19 | 32847460 | 32955721 | 1.320115238 | 5 | amp |
| TCGA-04-1361 | 19 | 32959617 | 33655196 | 0.733514103 | 3 | amp |
| TCGA-04-1361 | 19 | 34302641 | 34811036 | 1.108033008 | 4 | amp |
| TCGA-04-1361 | 19 | 34895774 | 35500247 | 0.801739253 | 3 | amp |
| TCGA-04-1361 | 19 | 36641849 | 38579545 | 1.02742956 | 4 | amp |
| TCGA-04-1361 | 19 | 39926207 | 40093275 | 0.575620107 | 3 | amp |
| TCGA-04-1361 | 19 | 40095219 | 40228644 | 1.247758565 | 5 | amp |
| TCGA-04-1361 | 19 | 40485954 | 40512105 | 0.736051947 | 3 | amp |
| TCGA-04-1361 | 19 | 40513123 | 40582169 | 1.246866706 | 5 | amp |
| TCGA-04-1361 | 19 | 41447168 | 41518769 | 1.249518093 | 5 | amp |
| TCGA-04-1361 | 19 | 42027347 | 42353380 | 0.731408303 | 3 | amp |
| TCGA-04-1361 | 19 | 42937907 | 43093928 | 1.0347171 | 4 | amp |
| TCGA-04-1361 | 19 | 43097632 | 43430908 | 0.732258916 | 3 | amp |
| TCGA-04-1361 | 19 | 43433552 | 43520237 | 1.793382665 | 6 | amp |
| TCGA-04-1361 | 19 | 43522880 | 43576820 | 1.424467391 | 5 | amp |
| TCGA-04-1361 | 19 | 43579464 | 43780585 | 0.722312488 | 3 | amp |
| TCGA-04-1361 | 19 | 44341147 | 44901430 | 1.09098805 | 4 | amp |
| TCGA-04-1361 | 19 | 44932427 | 45033566 | 0.759495664 | 3 | amp |
| TCGA-04-1361 | 19 | 47585406 | 47658480 | 0.96104131 | 3 | amp |
| TCGA-04-1361 | 19 | 48374707 | 48544053 | 1.111713256 | 4 | amp |
| TCGA-04-1361 | 19 | 48544784 | 48593707 | 0.730473687 | 3 | amp |
| TCGA-04-1361 | 19 | 48653285 | 48735111 | 0.901788288 | 3 | amp |
| TCGA-04-1361 | 19 | 52023302 | 52222617 | 0.594132926 | 3 | amp |
| TCGA-04-1361 | 19 | 52249150 | 52627358 | 1.035806408 | 4 | amp |
| TCGA-04-1361 | 19 | 52633699 | 52785157 | 0.729664081 | 3 | amp |
| TCGA-04-1361 | 19 | 52785306 | 53432702 | 1.026933831 | 4 | amp |
| TCGA-04-1361 | 19 | 53453054 | 54189825 | 0.774492203 | 3 | amp |

| | | | | | | |
|---|---|---|---|---|---|---|
| TCGA-04-1361 | 19 | 54191717 | 54206091 | 1.563892555 | 5 | amp |
| TCGA-04-1361 | 19 | 54209457 | 54265700 | 1.009204803 | 4 | amp |
| TCGA-04-1361 | 19 | 55235958 | 55445141 | 0.722478595 | 3 | amp |
| TCGA-04-1361 | 19 | 56235307 | 56307650 | 0.683430801 | 3 | amp |
| TCGA-04-1361 | 19 | 56312901 | 56487703 | 1.233778665 | 5 | amp |
| TCGA-04-1361 | 19 | 56490694 | 58500145 | 0.710547645 | 3 | amp |
| TCGA-04-1361 | 2 | 3517567 | 8874893 | 1.167910982 | 5 | amp |
| TCGA-04-1361 | 2 | 8876913 | 9098818 | 1.994736414 | 6 | amp |
| TCGA-04-1361 | 2 | 9419421 | 11702768 | 1.283899592 | 5 | amp |
| TCGA-04-1361 | 2 | 15415594 | 17847795 | 1.35367708 | 5 | amp |
| TCGA-04-1361 | 2 | 17851645 | 17927273 | 1.92581908 | 6 | amp |
| TCGA-04-1361 | 2 | 17941170 | 24991294 | 1.185488857 | 5 | amp |
| TCGA-04-1361 | 2 | 25610142 | 26652684 | 1.13539689 | 5 | amp |
| TCGA-04-1361 | 2 | 27532754 | 27693998 | 0.887014216 | 3 | amp |
| TCGA-04-1361 | 2 | 27695112 | 29416798 | 1.207109978 | 5 | amp |
| TCGA-04-1361 | 2 | 29419590 | 29940623 | 1.006357862 | 4 | amp |
| TCGA-04-1361 | 2 | 30379467 | 31628861 | 0.816511997 | 3 | amp |
| TCGA-04-1361 | 2 | 31637421 | 33525698 | 1.636371427 | 6 | amp |
| TCGA-04-1361 | 2 | 33526529 | 45829251 | 1.296318976 | 5 | amp |
| TCGA-04-1361 | 2 | 45832480 | 46386937 | 0.997503852 | 4 | amp |
| TCGA-04-1361 | 2 | 46411796 | 47607044 | 0.776419782 | 3 | amp |
| TCGA-04-1361 | 2 | 47612266 | 51149072 | 1.339566782 | 5 | amp |
| TCGA-04-1361 | 2 | 51149750 | 54849650 | 2.277678513 | 6 | amp |
| TCGA-04-1361 | 2 | 54850554 | 55214850 | 1.389526845 | 5 | amp |
| TCGA-04-1361 | 2 | 55237179 | 70131472 | 2.075242988 | 6 | amp |
| TCGA-04-1361 | 2 | 70143212 | 70387957 | 0.973952691 | 4 | amp |
| TCGA-04-1361 | 2 | 70392183 | 70463318 | 1.991213723 | 6 | amp |
| TCGA-04-1361 | 2 | 70475459 | 71148441 | 1.143293142 | 5 | amp |
| TCGA-04-1361 | 2 | 71302668 | 71582939 | 1.322201419 | 5 | amp |
| TCGA-04-1361 | 2 | 71590201 | 71655788 | 2.018842882 | 6 | amp |
| TCGA-04-1361 | 2 | 71658417 | 71741068 | 1.499186418 | 5 | amp |
| TCGA-04-1361 | 2 | 71742696 | 72362590 | 0.834371192 | 3 | amp |
| TCGA-04-1361 | 2 | 72406406 | 72968635 | 1.841548303 | 6 | amp |
| TCGA-04-1361 | 2 | 73052922 | 73053042 | 1.097313033 | 5 | amp |
| TCGA-04-1361 | 2 | 73115407 | 73471210 | 0.976231756 | 4 | amp |
| TCGA-04-1361 | 2 | 73471637 | 73498056 | 0.675574336 | 3 | amp |
| TCGA-04-1361 | 2 | 73635692 | 73800569 | 2.11606658 | 6 | amp |
| TCGA-04-1361 | 2 | 73826467 | 74448670 | 1.378588966 | 5 | amp |
| TCGA-04-1361 | 2 | 74756183 | 75118115 | 0.972492098 | 3 | amp |
| TCGA-04-1361 | 2 | 75186449 | 79255428 | 2.041996107 | 6 | amp |
| TCGA-04-1361 | 2 | 79312285 | 79386553 | 1.193618828 | 5 | amp |
| TCGA-04-1361 | 2 | 79422904 | 84676884 | 2.093336697 | 6 | amp |
| TCGA-04-1361 | 2 | 84775454 | 86344289 | 1.099450441 | 5 | amp |
| TCGA-04-1361 | 2 | 86345985 | 87397792 | 1.860838396 | 6 | amp |
| TCGA-04-1361 | 2 | 87398251 | 87566187 | 1.145522344 | 5 | amp |
| TCGA-04-1361 | 2 | 88003844 | 88093753 | 1.882027251 | 6 | amp |
| TCGA-04-1361 | 2 | 88094577 | 88874993 | 1.19641362 | 5 | amp |
| TCGA-04-1361 | 2 | 88876026 | 89513414 | 1.844566062 | 6 | amp |
| TCGA-04-1361 | 2 | 89521153 | 95947171 | 1.200577274 | 5 | amp |
| TCGA-04-1361 | 2 | 96079136 | 96693353 | 1.368871593 | 5 | amp |

| | | | | | | |
|---|---|---|---|---|---|---|
| TCGA-04-1361 | 2 | 96696097 | 96920772 | 1.055897923 | 4 | amp |
| TCGA-04-1361 | 2 | 96932982 | 97365862 | 0.90159312 | 3 | amp |
| TCGA-04-1361 | 2 | 97751396 | 98167988 | 1.742953375 | 6 | amp |
| TCGA-04-1361 | 2 | 98169606 | 103318972 | 1.246529468 | 5 | amp |
| TCGA-04-1361 | 2 | 103320948 | 110601219 | 1.048786843 | 4 | amp |
| TCGA-04-1361 | 2 | 110602629 | 111851129 | 0.88677199 | 3 | amp |
| TCGA-04-1361 | 2 | 111907612 | 112631275 | 1.343473224 | 5 | amp |
| TCGA-04-1361 | 2 | 112636347 | 113243592 | 1.08306215 | 4 | amp |
| TCGA-04-1361 | 2 | 113251715 | 122206732 | 0.847132359 | 3 | amp |
| TCGA-04-1361 | 2 | 122208467 | 128263333 | 1.075615484 | 4 | amp |
| TCGA-04-1361 | 2 | 128699538 | 130910799 | 1.426140058 | 5 | amp |
| TCGA-04-1361 | 2 | 131231913 | 131248338 | 2.087469658 | 6 | amp |
| TCGA-04-1361 | 2 | 131254099 | 132010709 | 1.22250168 | 5 | amp |
| TCGA-04-1361 | 2 | 133430809 | 138378297 | 1.237060423 | 5 | amp |
| TCGA-04-1361 | 2 | 138400010 | 148733558 | 1.738011548 | 6 | amp |
| TCGA-04-1361 | 2 | 149216323 | 161143597 | 1.382465554 | 5 | amp |
| TCGA-04-1361 | 2 | 161157121 | 170150774 | 1.910928829 | 6 | amp |
| TCGA-04-1361 | 2 | 170151074 | 170374912 | 1.245287722 | 5 | amp |
| TCGA-04-1361 | 2 | 170377323 | 172666819 | 1.909300467 | 6 | amp |
| TCGA-04-1361 | 2 | 172669777 | 172951601 | 1.395858425 | 5 | amp |
| TCGA-04-1361 | 2 | 172952647 | 175216459 | 1.803967721 | 6 | amp |
| TCGA-04-1361 | 2 | 175234654 | 175939569 | 1.324523041 | 5 | amp |
| TCGA-04-1361 | 2 | 175945380 | 176857990 | 1.843708474 | 6 | amp |
| TCGA-04-1361 | 2 | 176860238 | 177162659 | 0.971707253 | 4 | amp |
| TCGA-04-1361 | 2 | 177188095 | 179529482 | 1.837733538 | 6 | amp |
| TCGA-04-1361 | 2 | 179529555 | 183606086 | 1.336419097 | 5 | amp |
| TCGA-04-1361 | 2 | 183608321 | 183998358 | 1.720642742 | 6 | amp |
| TCGA-04-1361 | 2 | 184016156 | 201791676 | 1.367055236 | 5 | amp |
| TCGA-04-1361 | 2 | 201796056 | 202065255 | 1.836479578 | 6 | amp |
| TCGA-04-1361 | 2 | 202068410 | 202514901 | 1.504851947 | 5 | amp |
| TCGA-04-1361 | 2 | 202519539 | 203329736 | 1.835627213 | 6 | amp |
| TCGA-04-1361 | 2 | 203332206 | 217124458 | 1.436544658 | 5 | amp |
| TCGA-04-1361 | 2 | 219313912 | 219457444 | 1.378338977 | 5 | amp |
| TCGA-04-1361 | 2 | 219458764 | 219497066 | 1.024597731 | 4 | amp |
| TCGA-04-1361 | 2 | 219498285 | 219618409 | 0.952373535 | 3 | amp |
| TCGA-04-1361 | 2 | 222298816 | 232320343 | 1.389805545 | 5 | amp |
| TCGA-04-1361 | 2 | 232320428 | 233195502 | 0.92590173 | 3 | amp |
| TCGA-04-1361 | 2 | 233421122 | 234235864 | 1.250601625 | 5 | amp |
| TCGA-04-1361 | 2 | 234394164 | 235405240 | 1.210019765 | 5 | amp |
| TCGA-04-1361 | 2 | 238659818 | 238978147 | 1.305932868 | 5 | amp |
| TCGA-04-1361 | 20 | 68319 | 3185287 | 0.642790415 | 3 | amp |
| TCGA-04-1361 | 20 | 3285048 | 3624917 | 1.348368572 | 5 | amp |
| TCGA-04-1361 | 20 | 3888568 | 3944712 | 1.430559914 | 5 | amp |
| TCGA-04-1361 | 20 | 4155656 | 5149333 | 0.811111401 | 3 | amp |
| TCGA-04-1361 | 20 | 5154116 | 5538790 | 1.68718953 | 6 | amp |
| TCGA-04-1361 | 20 | 5539277 | 6068536 | 1.042961628 | 4 | amp |
| TCGA-04-1361 | 20 | 6069540 | 13415805 | 1.294478663 | 5 | amp |
| TCGA-04-1361 | 20 | 13463868 | 13698207 | 1.894194256 | 6 | amp |
| TCGA-04-1361 | 20 | 13699532 | 13868677 | 1.285467173 | 5 | amp |
| TCGA-04-1361 | 20 | 13869071 | 16478350 | 1.657853048 | 6 | amp |

| | | | | | | |
|---|---|---|---|---|---|---|
| TCGA-04-1361 | 20 | 16484984 | 16509110 | 1.177927752 | 5 | amp |
| TCGA-04-1361 | 20 | 16712319 | 16731811 | 1.905489532 | 6 | amp |
| TCGA-04-1361 | 20 | 17207888 | 17390005 | 1.162068625 | 4 | amp |
| TCGA-04-1361 | 20 | 17410033 | 17641187 | 0.574882387 | 3 | amp |
| TCGA-04-1361 | 20 | 17705613 | 20006425 | 1.095064116 | 4 | amp |
| TCGA-04-1361 | 20 | 20007375 | 21362753 | 1.286353394 | 5 | amp |
| TCGA-04-1361 | 20 | 21367424 | 23545689 | 1.012292064 | 4 | amp |
| TCGA-04-1361 | 20 | 23546607 | 25667099 | 0.563339309 | 3 | amp |
| TCGA-04-1361 | 20 | 25733291 | 30038024 | 1.187807294 | 4 | amp |
| TCGA-04-1361 | 20 | 30053237 | 34292688 | 0.718361877 | 3 | amp |
| TCGA-04-1361 | 20 | 34293139 | 34459781 | 1.088054732 | 4 | amp |
| TCGA-04-1361 | 20 | 34487250 | 35526954 | 0.710235182 | 3 | amp |
| TCGA-04-1361 | 20 | 35532545 | 35689708 | 1.12065736 | 4 | amp |
| TCGA-04-1361 | 20 | 35690429 | 37078199 | 0.687142035 | 3 | amp |
| TCGA-04-1361 | 20 | 37117048 | 37195926 | 1.284476321 | 4 | amp |
| TCGA-04-1361 | 20 | 37198524 | 37581212 | 0.593513326 | 3 | amp |
| TCGA-04-1361 | 20 | 37597640 | 39727035 | 1.14780925 | 4 | amp |
| TCGA-04-1361 | 20 | 39728639 | 40050741 | 0.537667686 | 3 | amp |
| TCGA-04-1361 | 20 | 40052085 | 41076974 | 1.099544977 | 4 | amp |
| TCGA-04-1361 | 20 | 41100873 | 43883201 | 0.63078547 | 3 | amp |
| TCGA-04-1361 | 20 | 44163034 | 44405900 | 1.299123795 | 5 | amp |
| TCGA-04-1361 | 20 | 44671726 | 52570282 | 0.687621881 | 3 | amp |
| TCGA-04-1361 | 20 | 52573943 | 54974457 | 1.084649963 | 4 | amp |
| TCGA-04-1361 | 20 | 54978443 | 58416639 | 0.677721494 | 3 | amp |
| TCGA-04-1361 | 20 | 58420221 | 58497516 | 1.755811025 | 6 | amp |
| TCGA-04-1361 | 20 | 58518974 | 59829997 | 1.109300284 | 4 | amp |
| TCGA-04-1361 | 20 | 60419681 | 60713406 | 0.632940276 | 3 | amp |
| TCGA-04-1361 | 21 | 9483321 | 35153868 | 1.29841646 | 5 | amp |
| TCGA-04-1361 | 21 | 35154217 | 38439740 | 0.877440255 | 4 | amp |
| TCGA-04-1361 | 21 | 38444660 | 38498471 | 1.586999299 | 6 | amp |
| TCGA-04-1361 | 21 | 38501272 | 40552406 | 1.10946914 | 5 | amp |
| TCGA-04-1361 | 21 | 40553660 | 40648178 | 1.527940779 | 6 | amp |
| TCGA-04-1361 | 21 | 40649136 | 42064928 | 1.138524203 | 5 | amp |
| TCGA-04-1361 | 21 | 42080358 | 42780218 | 0.808103875 | 4 | amp |
| TCGA-04-1361 | 22 | 16084594 | 16256013 | 0.583846639 | 3 | amp |
| TCGA-04-1361 | 22 | 16256133 | 16282595 | 1.294332172 | 5 | amp |
| TCGA-04-1361 | 22 | 16287209 | 17062226 | 0.894930596 | 3 | amp |
| TCGA-04-1361 | 22 | 17280607 | 17385630 | 1.22063246 | 4 | amp |
| TCGA-04-1361 | 22 | 18082740 | 18210351 | 0.698595022 | 3 | amp |
| TCGA-04-1361 | 22 | 18374204 | 18389626 | 0.869021852 | 3 | amp |
| TCGA-04-1361 | 22 | 21105562 | 21328921 | 0.632091877 | 3 | amp |
| TCGA-04-1361 | 22 | 22123400 | 22240051 | 0.747661174 | 3 | amp |
| TCGA-04-1361 | 22 | 22657565 | 22973546 | 0.695593875 | 3 | amp |
| TCGA-04-1361 | 22 | 23958990 | 23974226 | 0.769638486 | 3 | amp |
| TCGA-04-1361 | 22 | 24431906 | 24452858 | 0.73815159 | 3 | amp |
| TCGA-04-1361 | 22 | 24698155 | 24765296 | 0.675306991 | 3 | amp |
| TCGA-04-1361 | 22 | 25119039 | 25154060 | 0.657629574 | 3 | amp |
| TCGA-04-1361 | 22 | 26000321 | 26778061 | 0.593815959 | 3 | amp |
| TCGA-04-1361 | 22 | 28250843 | 29414060 | 0.711593117 | 3 | amp |
| TCGA-04-1361 | 22 | 29755730 | 29938956 | 0.59614483 | 3 | amp |

| | | | | | | |
|---|---|---|---|---|---|---|
| TCGA-04-1361 | 22 | 29965168 | 30067995 | 0.638922385 | 3 | amp |
| TCGA-04-1361 | 22 | 30209384 | 30572163 | 0.774039483 | 3 | amp |
| TCGA-04-1361 | 22 | 31329992 | 31342441 | 0.667231212 | 3 | amp |
| TCGA-04-1361 | 22 | 31798982 | 31981146 | 0.902620708 | 3 | amp |
| TCGA-04-1361 | 22 | 32154515 | 35682081 | 0.688428102 | 3 | amp |
| TCGA-04-1361 | 22 | 36052407 | 36629263 | 0.713245551 | 3 | amp |
| TCGA-04-1361 | 22 | 38710034 | 39117928 | 0.75796904 | 3 | amp |
| TCGA-04-1361 | 22 | 40139625 | 40258059 | 1.290365563 | 5 | amp |
| TCGA-04-1361 | 22 | 40283397 | 40362197 | 0.599956713 | 3 | amp |
| TCGA-04-1361 | 22 | 41222485 | 41562696 | 0.596761407 | 3 | amp |
| TCGA-04-1361 | 22 | 42042842 | 42073140 | 0.676992864 | 3 | amp |
| TCGA-04-1361 | 22 | 42128419 | 42167007 | 0.713956332 | 3 | amp |
| TCGA-04-1361 | 22 | 43972152 | 44178248 | 0.69786649 | 3 | amp |
| TCGA-04-1361 | 22 | 45740397 | 45813837 | 0.862232017 | 3 | amp |
| TCGA-04-1361 | 22 | 46114242 | 46202930 | 0.87263621 | 3 | amp |
| TCGA-04-1361 | 3 | 361444 | 8661721 | 1.293278891 | 5 | amp |
| TCGA-04-1361 | 3 | 8667220 | 9719764 | 1.065253581 | 4 | amp |
| TCGA-04-1361 | 3 | 9986005 | 12791354 | 0.892914272 | 4 | amp |
| TCGA-04-1361 | 3 | 14695924 | 15516504 | 1.035206261 | 4 | amp |
| TCGA-04-1361 | 3 | 15516902 | 37081790 | 1.448219779 | 5 | amp |
| TCGA-04-1361 | 3 | 37083730 | 37292990 | 1.821713302 | 6 | amp |
| TCGA-04-1361 | 3 | 37323425 | 37536085 | 1.400731775 | 5 | amp |
| TCGA-04-1361 | 3 | 37544613 | 38006172 | 0.933825184 | 4 | amp |
| TCGA-04-1361 | 3 | 38181848 | 46580732 | 1.006392121 | 4 | amp |
| TCGA-04-1361 | 3 | 47084000 | 48446004 | 0.885747404 | 4 | amp |
| TCGA-04-1361 | 3 | 50615191 | 51680518 | 1.118582164 | 4 | amp |
| TCGA-04-1361 | 3 | 52558112 | 52800412 | 1.167356997 | 4 | amp |
| TCGA-04-1361 | 3 | 52867332 | 53215312 | 1.092835833 | 4 | amp |
| TCGA-04-1361 | 3 | 53891619 | 56605389 | 1.235720329 | 5 | amp |
| TCGA-04-1361 | 3 | 56626913 | 56695039 | 1.903778216 | 6 | amp |
| TCGA-04-1361 | 3 | 56763293 | 57563138 | 1.293607893 | 5 | amp |
| TCGA-04-1361 | 3 | 57569619 | 57913128 | 1.861084315 | 6 | amp |
| TCGA-04-1361 | 3 | 58062716 | 62142939 | 0.948822323 | 4 | amp |
| TCGA-04-1361 | 3 | 62153620 | 98304513 | 1.309063722 | 5 | amp |
| TCGA-04-1361 | 3 | 98307507 | 101232014 | 2.012876684 | 6 | amp |
| TCGA-04-1361 | 3 | 101283575 | 102176720 | 1.402553134 | 5 | amp |
| TCGA-04-1361 | 3 | 102181083 | 112335750 | 1.912604243 | 6 | amp |
| TCGA-04-1361 | 3 | 112337762 | 113082411 | 1.224920649 | 5 | amp |
| TCGA-04-1361 | 3 | 113084810 | 113850285 | 1.750900781 | 6 | amp |
| TCGA-04-1361 | 3 | 113858294 | 119545728 | 1.492284394 | 5 | amp |
| TCGA-04-1361 | 3 | 119562060 | 119812297 | 2.194717805 | 6 | amp |
| TCGA-04-1361 | 3 | 119885804 | 120321145 | 1.298297477 | 5 | amp |
| TCGA-04-1361 | 3 | 120347181 | 121209261 | 2.036738907 | 6 | amp |
| TCGA-04-1361 | 3 | 121212291 | 121411432 | 1.260056848 | 5 | amp |
| TCGA-04-1361 | 3 | 121412541 | 121976260 | 1.872516019 | 6 | amp |
| TCGA-04-1361 | 3 | 121980323 | 124952823 | 1.354808339 | 5 | amp |
| TCGA-04-1361 | 3 | 124953023 | 125301847 | 1.969178841 | 6 | amp |
| TCGA-04-1361 | 3 | 125313380 | 125702189 | 1.036164039 | 4 | amp |
| TCGA-04-1361 | 3 | 127454490 | 129179779 | 0.885675613 | 4 | amp |
| TCGA-04-1361 | 3 | 130159007 | 130313215 | 1.44467981 | 5 | amp |

| | | | | | | |
|---|---|---|---|---|---|---|
| TCGA-04-1361 | 3 | 130318567 | 133372398 | 1.901539592 | 6 | amp |
| TCGA-04-1361 | 3 | 133374111 | 134898882 | 1.177020637 | 5 | amp |
| TCGA-04-1361 | 3 | 134911361 | 136667317 | 1.856350456 | 6 | amp |
| TCGA-04-1361 | 3 | 136676939 | 138216972 | 1.352463532 | 5 | amp |
| TCGA-04-1361 | 3 | 138218918 | 169524737 | 1.905862904 | 6 | amp |
| TCGA-04-1361 | 3 | 169525185 | 169656319 | 1.232944861 | 5 | amp |
| TCGA-04-1361 | 3 | 169693389 | 183732235 | 1.947853294 | 6 | amp |
| TCGA-04-1361 | 3 | 183754137 | 183801767 | 1.299379812 | 5 | amp |
| TCGA-04-1361 | 3 | 183818145 | 183862761 | 0.970257321 | 4 | amp |
| TCGA-04-1361 | 3 | 184298736 | 184429622 | 1.310238438 | 5 | amp |
| TCGA-04-1361 | 3 | 184542376 | 186460135 | 1.828349485 | 6 | amp |
| TCGA-04-1361 | 3 | 186461443 | 187451485 | 1.253141644 | 5 | amp |
| TCGA-04-1361 | 3 | 188123854 | 189038631 | 2.036077685 | 6 | amp |
| TCGA-04-1361 | 3 | 189349275 | 194309372 | 1.363182377 | 5 | amp |
| TCGA-04-1361 | 3 | 194313695 | 195298247 | 0.968703446 | 4 | amp |
| TCGA-04-1361 | 4 | 53323 | 466440 | 0.872613813 | 3 | amp |
| TCGA-04-1361 | 4 | 2132928 | 2717850 | 0.897506515 | 3 | amp |
| TCGA-04-1361 | 4 | 2877589 | 2906842 | 1.546139 | 6 | amp |
| TCGA-04-1361 | 4 | 3088647 | 3201682 | 0.951144954 | 3 | amp |
| TCGA-04-1361 | 4 | 4190464 | 5642641 | 0.828670465 | 3 | amp |
| TCGA-04-1361 | 4 | 6843712 | 6873414 | 1.330274753 | 5 | amp |
| TCGA-04-1361 | 4 | 9024980 | 10529768 | 0.830583278 | 3 | amp |
| TCGA-04-1361 | 4 | 10533833 | 20543278 | 1.304049033 | 5 | amp |
| TCGA-04-1361 | 4 | 20544062 | 25664515 | 1.567156854 | 6 | amp |
| TCGA-04-1361 | 4 | 25749996 | 36231135 | 1.541845808 | 6 | amp |
| TCGA-04-1361 | 4 | 37590415 | 38995667 | 1.146463064 | 5 | amp |
| TCGA-04-1361 | 4 | 39000214 | 39226698 | 1.567751999 | 6 | amp |
| TCGA-04-1361 | 4 | 39229783 | 39291683 | 1.210465675 | 5 | amp |
| TCGA-04-1361 | 4 | 39293245 | 39318648 | 1.815463965 | 6 | amp |
| TCGA-04-1361 | 4 | 39321994 | 41621540 | 1.338083036 | 5 | amp |
| TCGA-04-1361 | 4 | 41640876 | 41665039 | 0.86891506 | 4 | amp |
| TCGA-04-1361 | 4 | 41668570 | 46995458 | 1.491367346 | 5 | amp |
| TCGA-04-1361 | 4 | 47033646 | 47455273 | 0.979960792 | 4 | amp |
| TCGA-04-1361 | 4 | 47458549 | 47560345 | 1.539520682 | 6 | amp |
| TCGA-04-1361 | 4 | 47560902 | 47584088 | 0.989007665 | 4 | amp |
| TCGA-04-1361 | 4 | 47589009 | 47907368 | 1.533337935 | 6 | amp |
| TCGA-04-1361 | 4 | 47912805 | 48116451 | 1.172233933 | 5 | amp |
| TCGA-04-1361 | 4 | 48136119 | 52894319 | 1.549776187 | 6 | amp |
| TCGA-04-1361 | 4 | 52894871 | 57164575 | 0.966161552 | 4 | amp |
| TCGA-04-1361 | 4 | 57173662 | 57840225 | 0.777440305 | 3 | amp |
| TCGA-04-1361 | 4 | 57842586 | 68797804 | 1.062971211 | 4 | amp |
| TCGA-04-1361 | 4 | 68810171 | 70080499 | 1.17597894 | 5 | amp |
| TCGA-04-1361 | 4 | 70146158 | 70359604 | 1.539353746 | 6 | amp |
| TCGA-04-1361 | 4 | 70360798 | 74303986 | 1.021868381 | 4 | amp |
| TCGA-04-1361 | 4 | 74306304 | 76878885 | 0.83980046 | 3 | amp |
| TCGA-04-1361 | 4 | 76878986 | 143081732 | 0.979506047 | 4 | amp |
| TCGA-04-1361 | 4 | 143094753 | 146572316 | 1.349004815 | 5 | amp |
| TCGA-04-1361 | 4 | 146575100 | 159814921 | 0.951148167 | 4 | amp |
| TCGA-04-1361 | 4 | 159816899 | 160260565 | 1.651580447 | 6 | amp |
| TCGA-04-1361 | 4 | 160262667 | 169167788 | 1.220475477 | 5 | amp |

| | | | | | | |
|---|---|---|---|---|---|---|
| TCGA-04-1361 | 4 | 169169330 | 186541320 | 1.000298956 | 4 | amp |
| TCGA-04-1361 | 4 | 186544039 | 187000209 | 0.883742326 | 3 | amp |
| TCGA-04-1361 | 4 | 190874191 | 190948390 | 0.926680064 | 3 | amp |
| TCGA-04-1361 | 5 | 5186114 | 5306848 | 0.748186097 | 3 | amp |
| TCGA-04-1361 | 5 | 5318200 | 10402589 | 1.182051552 | 5 | amp |
| TCGA-04-1361 | 5 | 10402621 | 10448553 | 1.648619809 | 6 | amp |
| TCGA-04-1361 | 5 | 10450024 | 11236988 | 0.886552017 | 4 | amp |
| TCGA-04-1361 | 5 | 11346401 | 52404532 | 1.33540128 | 5 | amp |
| TCGA-04-1361 | 5 | 52778655 | 96066583 | 0.96193639 | 4 | amp |
| TCGA-04-1361 | 5 | 96071819 | 96253327 | 1.271991268 | 5 | amp |
| TCGA-04-1361 | 5 | 96314781 | 110097492 | 1.610969736 | 6 | amp |
| TCGA-04-1361 | 5 | 110427941 | 131080439 | 1.381993389 | 5 | amp |
| TCGA-04-1361 | 5 | 131283282 | 131553552 | 0.817866714 | 3 | amp |
| TCGA-04-1361 | 5 | 131554218 | 131895077 | 1.065029369 | 5 | amp |
| TCGA-04-1361 | 5 | 131911424 | 131945091 | 1.738628487 | 6 | amp |
| TCGA-04-1361 | 5 | 131951608 | 132032409 | 1.007428974 | 5 | amp |
| TCGA-04-1361 | 5 | 132034804 | 132052726 | 1.737190178 | 6 | amp |
| TCGA-04-1361 | 5 | 132056293 | 132209831 | 0.725665305 | 3 | amp |
| TCGA-04-1361 | 5 | 132210029 | 132439757 | 1.314460833 | 5 | amp |
| TCGA-04-1361 | 5 | 132439876 | 137223107 | 1.034874454 | 4 | amp |
| TCGA-04-1361 | 5 | 137226160 | 137534018 | 1.211528893 | 5 | amp |
| TCGA-04-1361 | 5 | 137534103 | 138704514 | 1.026809597 | 4 | amp |
| TCGA-04-1361 | 5 | 138707638 | 139189443 | 0.803335694 | 3 | amp |
| TCGA-04-1361 | 5 | 139748147 | 139906705 | 1.333499439 | 5 | amp |
| TCGA-04-1361 | 5 | 139907412 | 140433543 | 0.726921794 | 3 | amp |
| TCGA-04-1361 | 5 | 140905552 | 140951058 | 1.865277492 | 6 | amp |
| TCGA-04-1361 | 5 | 140951459 | 141005668 | 1.011316121 | 3 | amp |
| TCGA-04-1361 | 5 | 141511326 | 145393646 | 1.426417417 | 5 | amp |
| TCGA-04-1361 | 5 | 145464971 | 145532823 | 1.560067182 | 6 | amp |
| TCGA-04-1361 | 5 | 145533274 | 148384489 | 1.332836209 | 5 | amp |
| TCGA-04-1361 | 5 | 148386391 | 148899961 | 1.03611092 | 4 | amp |
| TCGA-04-1361 | 5 | 148904550 | 149361395 | 0.811401461 | 3 | amp |
| TCGA-04-1361 | 5 | 150097801 | 162869159 | 0.777102837 | 3 | amp |
| TCGA-04-1361 | 5 | 169021356 | 169446157 | 0.893578292 | 3 | amp |
| TCGA-04-1361 | 5 | 176477724 | 176729016 | 0.967603238 | 3 | amp |
| TCGA-04-1361 | 5 | 177020593 | 177483199 | 0.944028254 | 3 | amp |
| TCGA-04-1361 | 5 | 179046218 | 180326394 | 0.77430145 | 3 | amp |
| TCGA-04-1361 | 6 | 105907 | 3177881 | 0.794943037 | 3 | amp |
| TCGA-04-1361 | 6 | 3179831 | 6002777 | 1.04918467 | 4 | amp |
| TCGA-04-1361 | 6 | 6006864 | 10709697 | 1.217603276 | 5 | amp |
| TCGA-04-1361 | 6 | 10724796 | 10882041 | 0.849055032 | 4 | amp |
| TCGA-04-1361 | 6 | 10887303 | 12290954 | 1.349343023 | 5 | amp |
| TCGA-04-1361 | 6 | 12292507 | 13620738 | 0.878668492 | 4 | amp |
| TCGA-04-1361 | 6 | 13620962 | 26046083 | 1.245466234 | 5 | amp |
| TCGA-04-1361 | 6 | 26055945 | 29571520 | 0.870453368 | 4 | amp |
| TCGA-04-1361 | 6 | 29572192 | 31106085 | 0.689649323 | 3 | amp |
| TCGA-04-1361 | 6 | 32191507 | 32307467 | 1.506174375 | 5 | amp |
| TCGA-04-1361 | 6 | 32317501 | 32796841 | 0.955381192 | 4 | amp |
| TCGA-04-1361 | 6 | 32797124 | 33095794 | 0.727407484 | 3 | amp |
| TCGA-04-1361 | 6 | 34214783 | 35090123 | 0.965606169 | 4 | amp |

| | | | | | | |
|---|---|---|---|---|---|---|
| TCGA-04-1361 | 6 | 35096778 | 35478908 | 0.551234516 | 3 | amp |
| TCGA-04-1361 | 6 | 35480343 | 36076250 | 1.142862791 | 4 | amp |
| TCGA-04-1361 | 6 | 36100366 | 37141908 | 0.733211515 | 3 | amp |
| TCGA-04-1361 | 6 | 37182937 | 38565897 | 1.000280611 | 4 | amp |
| TCGA-04-1361 | 6 | 38645024 | 38919314 | 1.638048383 | 6 | amp |
| TCGA-04-1361 | 6 | 38939344 | 39041585 | 1.208059372 | 5 | amp |
| TCGA-04-1361 | 6 | 39046067 | 42237353 | 0.661531732 | 3 | amp |
| TCGA-04-1361 | 6 | 42541391 | 42638510 | 1.453568646 | 5 | amp |
| TCGA-04-1361 | 6 | 42641516 | 42847742 | 0.982234044 | 4 | amp |
| TCGA-04-1361 | 6 | 43491338 | 43591857 | 0.976754049 | 4 | amp |
| TCGA-04-1361 | 6 | 44357966 | 52138746 | 1.480191629 | 5 | amp |
| TCGA-04-1361 | 6 | 52141049 | 52881084 | 0.987810793 | 4 | amp |
| TCGA-04-1361 | 6 | 52883063 | 73787667 | 1.390821804 | 5 | amp |
| TCGA-04-1361 | 6 | 73814974 | 75811801 | 0.932720967 | 4 | amp |
| TCGA-04-1361 | 6 | 75812300 | 86332422 | 1.53092418 | 6 | amp |
| TCGA-04-1361 | 6 | 86333659 | 109748243 | 1.2913306 | 5 | amp |
| TCGA-04-1361 | 6 | 109748267 | 109773646 | 0.539415195 | 3 | amp |
| TCGA-04-1361 | 6 | 109773705 | 109803233 | 0.911086563 | 4 | amp |
| TCGA-04-1361 | 6 | 109814552 | 112435972 | 1.272875307 | 5 | amp |
| TCGA-04-1361 | 6 | 112437010 | 132206256 | 1.566928562 | 6 | amp |
| TCGA-04-1361 | 6 | 132207662 | 132860491 | 1.279791352 | 5 | amp |
| TCGA-04-1361 | 6 | 132873805 | 149854771 | 0.979117389 | 4 | amp |
| TCGA-04-1361 | 6 | 149855801 | 151239818 | 0.687548715 | 3 | amp |
| TCGA-04-1361 | 6 | 151243299 | 155152274 | 0.923562281 | 4 | amp |
| TCGA-04-1361 | 6 | 155153020 | 160579640 | 0.652297943 | 3 | amp |
| TCGA-04-1361 | 6 | 160638406 | 161505710 | 0.986618047 | 4 | amp |
| TCGA-04-1361 | 6 | 161507400 | 171055029 | 0.645985302 | 3 | amp |
| TCGA-04-1361 | 7 | 881554 | 881794 | 9.963497376 | 6 | amp |
| TCGA-04-1361 | 7 | 6026348 | 6970595 | 0.719527371 | 3 | amp |
| TCGA-04-1361 | 7 | 7118571 | 21904333 | 1.235567185 | 5 | amp |
| TCGA-04-1361 | 7 | 21906064 | 27211834 | 0.993064183 | 4 | amp |
| TCGA-04-1361 | 7 | 27222410 | 30494959 | 1.253670034 | 5 | amp |
| TCGA-04-1361 | 7 | 30496316 | 30911949 | 0.784566878 | 3 | amp |
| TCGA-04-1361 | 7 | 31377848 | 42018392 | 1.465713538 | 5 | amp |
| TCGA-04-1361 | 7 | 42063016 | 43679290 | 1.086083919 | 4 | amp |
| TCGA-04-1361 | 7 | 43680119 | 43982651 | 0.753982148 | 3 | amp |
| TCGA-04-1361 | 7 | 45010143 | 47860788 | 0.746127272 | 3 | amp |
| TCGA-04-1361 | 7 | 47866856 | 48146694 | 1.003924863 | 4 | amp |
| TCGA-04-1361 | 7 | 48146910 | 50070916 | 1.383208655 | 5 | amp |
| TCGA-04-1361 | 7 | 50097540 | 50571783 | 1.051672787 | 4 | amp |
| TCGA-04-1361 | 7 | 50595810 | 55753066 | 0.788306395 | 3 | amp |
| TCGA-04-1361 | 7 | 55755424 | 56065160 | 1.228150972 | 4 | amp |
| TCGA-04-1361 | 7 | 56066672 | 57142378 | 0.658205251 | 3 | amp |
| TCGA-04-1361 | 7 | 57187542 | 72341127 | 1.062230265 | 4 | amp |
| TCGA-04-1361 | 7 | 72361109 | 73105353 | 0.779076178 | 3 | amp |
| TCGA-04-1361 | 7 | 74114548 | 76682184 | 0.725347844 | 3 | amp |
| TCGA-04-1361 | 7 | 76684535 | 76958738 | 1.19938772 | 5 | amp |
| TCGA-04-1361 | 7 | 76959499 | 87811367 | 1.54746575 | 6 | amp |
| TCGA-04-1361 | 7 | 87815896 | 97788753 | 1.383618515 | 5 | amp |
| TCGA-04-1361 | 7 | 99261534 | 99375716 | 1.644951871 | 6 | amp |

| | | | | | | |
|---|---|---|---|---|---|---|
| TCGA-04-1361 | 7 | 99377601 | 99461359 | 1.325249214 | 5 | amp |
| TCGA-04-1361 | 7 | 99463516 | 99521220 | 0.964289729 | 4 | amp |
| TCGA-04-1361 | 7 | 99526402 | 99972101 | 0.725083359 | 3 | amp |
| TCGA-04-1361 | 7 | 99986657 | 100018318 | 1.332669294 | 5 | amp |
| TCGA-04-1361 | 7 | 100027636 | 100151166 | 0.762586749 | 3 | amp |
| TCGA-04-1361 | 7 | 101967609 | 102343959 | 0.679587856 | 3 | amp |
| TCGA-04-1361 | 7 | 102453763 | 103132496 | 1.253396937 | 5 | amp |
| TCGA-04-1361 | 7 | 103136137 | 104748390 | 1.686499968 | 6 | amp |
| TCGA-04-1361 | 7 | 104749351 | 106800080 | 1.209213268 | 5 | amp |
| TCGA-04-1361 | 7 | 106820302 | 126542784 | 1.527332212 | 6 | amp |
| TCGA-04-1361 | 7 | 126543982 | 127230215 | 1.395237115 | 5 | amp |
| TCGA-04-1361 | 7 | 127230959 | 128415848 | 0.999641939 | 4 | amp |
| TCGA-04-1361 | 7 | 128441221 | 128477869 | 0.850429191 | 3 | amp |
| TCGA-04-1361 | 7 | 128588569 | 130044596 | 1.03673988 | 4 | amp |
| TCGA-04-1361 | 7 | 130050907 | 131149169 | 1.400800674 | 5 | amp |
| TCGA-04-1361 | 7 | 131150984 | 132193458 | 0.935213384 | 4 | amp |
| TCGA-04-1361 | 7 | 132470349 | 134363813 | 1.370012907 | 5 | amp |
| TCGA-04-1361 | 7 | 134552459 | 135048831 | 0.857785563 | 4 | amp |
| TCGA-04-1361 | 7 | 135069394 | 135323487 | 1.664168428 | 6 | amp |
| TCGA-04-1361 | 7 | 135327902 | 138394574 | 1.287361671 | 5 | amp |
| TCGA-04-1361 | 7 | 138400417 | 140390646 | 1.043741957 | 4 | amp |
| TCGA-04-1361 | 7 | 140394412 | 142471836 | 1.303403688 | 5 | amp |
| TCGA-04-1361 | 7 | 142479868 | 142510596 | 1.007143952 | 4 | amp |
| TCGA-04-1361 | 7 | 142510890 | 144097423 | 0.7265509 | 3 | amp |
| TCGA-04-1361 | 7 | 144098114 | 148769619 | 1.111560861 | 4 | amp |
| TCGA-04-1361 | 7 | 148771433 | 151135385 | 0.677252425 | 3 | amp |
| TCGA-04-1361 | 7 | 151164160 | 154264023 | 1.367651659 | 5 | amp |
| TCGA-04-1361 | 7 | 154379580 | 156468593 | 1.010429136 | 4 | amp |
| TCGA-04-1361 | 7 | 156469050 | 157000632 | 1.387217118 | 5 | amp |
| TCGA-04-1361 | 7 | 157009552 | 158486214 | 0.950391318 | 4 | amp |
| TCGA-04-1361 | 7 | 158494491 | 158935247 | 0.762518813 | 3 | amp |
| TCGA-04-1361 | 8 | 116074 | 15531360 | 0.777558176 | 3 | amp |
| TCGA-04-1361 | 8 | 15588146 | 18793657 | 1.119482263 | 5 | amp |
| TCGA-04-1361 | 8 | 19177028 | 30706566 | 0.678345151 | 3 | amp |
| TCGA-04-1361 | 8 | 30840897 | 37603163 | 1.183846065 | 5 | amp |
| TCGA-04-1361 | 8 | 37607053 | 38099955 | 0.784568677 | 3 | amp |
| TCGA-04-1361 | 8 | 38103213 | 38184430 | 1.194405036 | 5 | amp |
| TCGA-04-1361 | 8 | 38186853 | 38851181 | 0.703015257 | 3 | amp |
| TCGA-04-1361 | 8 | 38852809 | 40625264 | 1.52768334 | 5 | amp |
| TCGA-04-1361 | 8 | 41122666 | 42780795 | 0.732524277 | 3 | amp |
| TCGA-04-1361 | 8 | 42785229 | 95752065 | 1.388974398 | 5 | amp |
| TCGA-04-1361 | 8 | 95768219 | 95905241 | 2.26163161 | 6 | amp |
| TCGA-04-1361 | 8 | 95906055 | 95943011 | 0.864379608 | 4 | amp |
| TCGA-04-1361 | 8 | 95944264 | 97332583 | 2.5845465 | 6 | amp |
| TCGA-04-1361 | 8 | 97342414 | 97605823 | 1.477089146 | 5 | amp |
| TCGA-04-1361 | 8 | 97614569 | 98837393 | 2.812751084 | 6 | amp |
| TCGA-04-1361 | 8 | 98863603 | 99019887 | 1.604674751 | 5 | amp |
| TCGA-04-1361 | 8 | 99028708 | 99055057 | 2.777539855 | 6 | amp |
| TCGA-04-1361 | 8 | 99057123 | 99102260 | 0.696838401 | 3 | amp |
| TCGA-04-1361 | 8 | 99105414 | 99146913 | 2.246250862 | 6 | amp |

| | | | | | | |
|---|---|---|---|---|---|---|
| TCGA-04-1361 | 8 | 99148657 | 99149195 | 1.09280404 | 5 | amp |
| TCGA-04-1361 | 8 | 99152301 | 99779654 | 2.718514235 | 6 | amp |
| TCGA-04-1361 | 8 | 99786946 | 100026209 | 1.583298525 | 5 | amp |
| TCGA-04-1361 | 8 | 100050612 | 101206537 | 2.839831845 | 6 | amp |
| TCGA-04-1361 | 8 | 101226036 | 101232702 | 0.859893817 | 4 | amp |
| TCGA-04-1361 | 8 | 101237363 | 110334734 | 2.743583036 | 6 | amp |
| TCGA-04-1361 | 8 | 110346030 | 110348454 | 0.920253858 | 4 | amp |
| TCGA-04-1361 | 8 | 110351493 | 120850692 | 3.050666566 | 6 | amp |
| TCGA-04-1361 | 8 | 120853990 | 120862808 | 1.293264 | 5 | amp |
| TCGA-04-1361 | 8 | 120865250 | 122641626 | 3.504086585 | 6 | amp |
| TCGA-04-1361 | 8 | 123963747 | 124089543 | 1.614654174 | 5 | amp |
| TCGA-04-1361 | 8 | 124094939 | 124453660 | 2.392800434 | 6 | amp |
| TCGA-04-1361 | 8 | 124515567 | 124725015 | 1.459636887 | 5 | amp |
| TCGA-04-1361 | 8 | 124747935 | 125528319 | 2.761824821 | 6 | amp |
| TCGA-04-1361 | 8 | 125531030 | 125603493 | 1.515241419 | 5 | amp |
| TCGA-04-1361 | 8 | 125711743 | 131140374 | 2.938079324 | 6 | amp |
| TCGA-04-1361 | 8 | 131146505 | 131149300 | 1.092415566 | 5 | amp |
| TCGA-04-1361 | 8 | 131164926 | 133144528 | 3.412964131 | 6 | amp |
| TCGA-04-1361 | 8 | 133146495 | 133153596 | 1.378364235 | 5 | amp |
| TCGA-04-1361 | 8 | 133175637 | 134147098 | 3.071020027 | 6 | amp |
| TCGA-04-1361 | 8 | 134225066 | 135812866 | 1.522731881 | 5 | amp |
| TCGA-04-1361 | 8 | 135817063 | 139190975 | 3.011849984 | 6 | amp |
| TCGA-04-1361 | 8 | 139207465 | 139209955 | 0.874205072 | 4 | amp |
| TCGA-04-1361 | 8 | 139255127 | 139703150 | 2.554149317 | 6 | amp |
| TCGA-04-1361 | 8 | 139705855 | 139712430 | 1.191914903 | 5 | amp |
| TCGA-04-1361 | 8 | 139715509 | 141902766 | 2.376095866 | 6 | amp |
| TCGA-04-1361 | 8 | 142146697 | 145758729 | 0.811785655 | 3 | amp |
| TCGA-04-1361 | 8 | 145759459 | 146279593 | 1.291233048 | 5 | amp |
| TCGA-04-1361 | 9 | 14753 | 5549630 | 0.829633707 | 3 | amp |
| TCGA-04-1361 | 9 | 5557564 | 21512208 | 0.998815462 | 4 | amp |
| TCGA-04-1361 | 9 | 21815414 | 32541837 | 1.319650515 | 5 | amp |
| TCGA-04-1361 | 9 | 32541876 | 33364816 | 1.090251931 | 4 | amp |
| TCGA-04-1361 | 9 | 33366549 | 33933676 | 0.638898336 | 3 | amp |
| TCGA-04-1361 | 9 | 33935766 | 34343459 | 1.116848793 | 4 | amp |
| TCGA-04-1361 | 9 | 34379066 | 34513209 | 0.748451685 | 3 | amp |
| TCGA-04-1361 | 9 | 35106527 | 35108304 | 0.603583276 | 3 | amp |
| TCGA-04-1361 | 9 | 35147806 | 35396655 | 1.168835272 | 4 | amp |
| TCGA-04-1361 | 9 | 35396795 | 35555737 | 0.820925802 | 3 | amp |
| TCGA-04-1361 | 9 | 35971750 | 42376374 | 0.757461329 | 3 | amp |
| TCGA-04-1361 | 9 | 42377362 | 67930870 | 0.958181728 | 4 | amp |
| TCGA-04-1361 | 9 | 67934715 | 71395992 | 1.236661429 | 5 | amp |
| TCGA-04-1361 | 9 | 71437504 | 71550003 | 1.981376395 | 6 | amp |
| TCGA-04-1361 | 9 | 71555513 | 72082931 | 1.224222952 | 5 | amp |
| TCGA-04-1361 | 9 | 72086531 | 80022523 | 1.748898617 | 6 | amp |
| TCGA-04-1361 | 9 | 80030836 | 86243919 | 1.403718037 | 5 | amp |
| TCGA-04-1361 | 9 | 86256424 | 86514767 | 1.809351605 | 6 | amp |
| TCGA-04-1361 | 9 | 86517959 | 87359997 | 1.419707357 | 5 | amp |
| TCGA-04-1361 | 9 | 87366890 | 88642829 | 1.721243089 | 6 | amp |
| TCGA-04-1361 | 9 | 88648193 | 88968154 | 1.266371877 | 5 | amp |
| TCGA-04-1361 | 9 | 89763609 | 90256988 | 0.99481182 | 4 | amp |

| | | | | | | |
|---|---|---|---|---|---|---|
| TCGA-04-1361 | 9 | 90258259 | 95373695 | 0.78281768 | 3 | amp |
| TCGA-04-1361 | 9 | 98660071 | 100849653 | 0.721852088 | 3 | amp |
| TCGA-04-1361 | 9 | 101798409 | 101825399 | 0.681621025 | 3 | amp |
| TCGA-04-1361 | 9 | 101829136 | 115456521 | 0.966988808 | 4 | amp |
| TCGA-04-1361 | 9 | 115478692 | 116083884 | 0.784519935 | 3 | amp |
| TCGA-04-1361 | 9 | 117389142 | 127636066 | 0.761823656 | 3 | amp |
| TCGA-04-1361 | 9 | 127637191 | 130151410 | 1.01231695 | 4 | amp |
| TCGA-04-1361 | 9 | 130152832 | 130210062 | 0.688500947 | 3 | amp |
| TCGA-04-1361 | 9 | 131246977 | 131286177 | 0.679197218 | 3 | amp |
| TCGA-04-1361 | 9 | 131287436 | 131370601 | 1.20884693 | 4 | amp |
| TCGA-04-1361 | 9 | 131371114 | 131757815 | 0.63470668 | 3 | amp |
| TCGA-04-1361 | 9 | 132591442 | 132891099 | 0.861126781 | 3 | amp |
| TCGA-04-1361 | 9 | 134002858 | 134006247 | 0.789759806 | 3 | amp |
| TCGA-04-1361 | 9 | 134007954 | 134105250 | 1.129042174 | 4 | amp |
| TCGA-04-1361 | 9 | 134105966 | 135144892 | 0.572213502 | 3 | amp |
| TCGA-04-1361 | 9 | 135144955 | 135802699 | 0.95314431 | 4 | amp |
| TCGA-04-1361 | X | 2839884 | 10201638 | 0.841502377 | 3 | amp |
| TCGA-04-1361 | X | 10417371 | 15509392 | 1.079960719 | 4 | amp |
| TCGA-04-1361 | X | 15526425 | 15610458 | 1.475054724 | 5 | amp |
| TCGA-04-1361 | X | 15612896 | 19026289 | 1.050539379 | 4 | amp |
| TCGA-04-1361 | X | 19027701 | 19626169 | 1.362191659 | 5 | amp |
| TCGA-04-1361 | X | 19649938 | 20222240 | 1.710747764 | 6 | amp |
| TCGA-04-1361 | X | 20227343 | 21613537 | 1.469466464 | 5 | amp |
| TCGA-04-1361 | X | 21619373 | 30746913 | 1.078409466 | 4 | amp |
| TCGA-04-1361 | X | 30849497 | 39911687 | 1.325710821 | 5 | amp |
| TCGA-04-1361 | X | 39913096 | 43626898 | 1.105715198 | 4 | amp |
| TCGA-04-1361 | X | 43627871 | 46309962 | 1.289155241 | 5 | amp |
| TCGA-04-1361 | X | 46322125 | 47036001 | 1.86305799 | 6 | amp |
| TCGA-04-1361 | X | 47065309 | 47486755 | 1.069240251 | 4 | amp |
| TCGA-04-1361 | X | 47486834 | 47705753 | 1.319915066 | 5 | amp |
| TCGA-04-1361 | X | 47747340 | 48300840 | 2.177827349 | 6 | amp |
| TCGA-04-1361 | X | 48326074 | 48781026 | 0.879342653 | 3 | amp |
| TCGA-04-1361 | X | 48781108 | 48929717 | 1.112596904 | 4 | amp |
| TCGA-04-1361 | X | 48932456 | 49021185 | 0.766227292 | 3 | amp |
| TCGA-04-1361 | X | 49021194 | 49034842 | 2.811428655 | 6 | amp |
| TCGA-04-1361 | X | 49035548 | 49143583 | 1.183370288 | 5 | amp |
| TCGA-04-1361 | X | 49161318 | 50659661 | 1.818626122 | 6 | amp |
| TCGA-04-1361 | X | 51807113 | 53619592 | 0.779959441 | 3 | amp |
| TCGA-04-1361 | X | 53620262 | 54467227 | 1.100944057 | 4 | amp |
| TCGA-04-1361 | X | 54469818 | 55117048 | 0.760566234 | 3 | amp |
| TCGA-04-1361 | X | 55117707 | 67518950 | 1.151666014 | 4 | amp |
| TCGA-04-1361 | X | 67652665 | 70588081 | 0.788594576 | 3 | amp |
| TCGA-04-1361 | X | 70594956 | 76777923 | 1.06838627 | 4 | amp |
| TCGA-04-1361 | X | 76778684 | 100630321 | 1.314935233 | 5 | amp |
| TCGA-04-1361 | X | 100645972 | 100659005 | 1.024103451 | 4 | amp |
| TCGA-04-1361 | X | 100662644 | 102339846 | 0.856497508 | 3 | amp |
| TCGA-04-1361 | X | 102342201 | 103433033 | 1.065883979 | 4 | amp |
| TCGA-04-1361 | X | 103434223 | 104512267 | 1.379288136 | 5 | amp |
| TCGA-04-1361 | X | 104728281 | 106117237 | 1.818131043 | 6 | amp |
| TCGA-04-1361 | X | 106144022 | 107434812 | 1.393825274 | 5 | amp |

| | | | | | | |
|---|---|---|---|---|---|---|
| TCGA-04-1361 | X | 107435696 | 108641972 | 1.789469388 | 6 | amp |
| TCGA-04-1361 | X | 108647514 | 108921688 | 1.289530922 | 5 | amp |
| TCGA-04-1361 | X | 108924145 | 110463690 | 1.719351763 | 6 | amp |
| TCGA-04-1361 | X | 110489775 | 113966039 | 1.42616319 | 5 | amp |
| TCGA-04-1361 | X | 114082545 | 117752721 | 1.615683528 | 6 | amp |
| TCGA-04-1361 | X | 117758439 | 118148364 | 1.377169827 | 5 | amp |
| TCGA-04-1361 | X | 118151455 | 118986933 | 1.087234972 | 4 | amp |
| TCGA-04-1361 | X | 119004490 | 119496114 | 0.750647566 | 3 | amp |
| TCGA-04-1361 | X | 119500347 | 122319882 | 1.128451043 | 4 | amp |
| TCGA-04-1361 | X | 122387093 | 128652440 | 1.499991664 | 5 | amp |
| TCGA-04-1361 | X | 128678884 | 128876180 | 1.110584723 | 4 | amp |
| TCGA-04-1361 | X | 128877901 | 129270721 | 0.765461769 | 3 | amp |
| TCGA-04-1361 | X | 129271053 | 130215945 | 1.201512639 | 4 | amp |
| TCGA-04-1361 | X | 130217111 | 130420061 | 0.617148451 | 3 | amp |
| TCGA-04-1361 | X | 130420333 | 138668651 | 1.299331543 | 5 | amp |
| TCGA-04-1361 | X | 138669820 | 138724711 | 1.831437986 | 6 | amp |
| TCGA-04-1361 | X | 138727711 | 148044475 | 1.364730425 | 5 | amp |
| TCGA-04-1361 | X | 148048219 | 148628516 | 0.987904009 | 4 | amp |
| TCGA-04-1361 | X | 148663881 | 149783176 | 0.768188429 | 3 | amp |
| TCGA-04-1361 | X | 149787471 | 149818401 | 2.246371737 | 6 | amp |
| TCGA-04-1361 | X | 149826276 | 149905936 | 1.272179424 | 5 | amp |
| TCGA-04-1361 | X | 149912818 | 150867349 | 1.084829986 | 4 | amp |
| TCGA-04-1361 | X | 150868397 | 152087668 | 0.801902618 | 3 | amp |
| TCGA-04-1361 | X | 153743999 | 153869202 | 0.760727289 | 3 | amp |
| TCGA-04-1361 | X | 153869710 | 153994702 | 1.14204366 | 4 | amp |
| TCGA-04-1361 | X | 153995213 | 155252897 | 1.323958814 | 5 | amp |
| TCGA-04-1361 | Y | 4966220 | 14619201 | 0.82900632 | 4 | amp |
| TCGA-04-1362 | 1 | 16834 | 1191524 | 1.663592825 | 4 | amp |
| TCGA-04-1362 | 1 | 1198655 | 8585890 | 2.925771066 | 5 | amp |
| TCGA-04-1362 | 1 | 8601264 | 8716467 | 1.377414949 | 4 | amp |
| TCGA-04-1362 | 1 | 8921363 | 9992049 | 2.92400045 | 5 | amp |
| TCGA-04-1362 | 1 | 9992848 | 10425766 | 1.662888955 | 4 | amp |
| TCGA-04-1362 | 1 | 10428500 | 11199760 | 2.727293094 | 5 | amp |
| TCGA-04-1362 | 1 | 11204698 | 11318655 | 1.671430284 | 4 | amp |
| TCGA-04-1362 | 1 | 11319265 | 12294431 | 3.286839063 | 5 | amp |
| TCGA-04-1362 | 1 | 12302518 | 14113075 | 1.719460162 | 4 | amp |
| TCGA-04-1362 | 1 | 14142861 | 15894712 | 2.444010367 | 5 | amp |
| TCGA-04-1362 | 1 | 15900092 | 16875383 | 1.422839139 | 4 | amp |
| TCGA-04-1362 | 1 | 16945531 | 19420013 | 1.760804106 | 4 | amp |
| TCGA-04-1362 | 1 | 19420387 | 19569022 | 0.822775141 | 3 | amp |
| TCGA-04-1362 | 1 | 19570064 | 21071616 | 1.530944685 | 4 | amp |
| TCGA-04-1362 | 1 | 21072032 | 22083236 | 1.072775889 | 3 | amp |
| TCGA-04-1362 | 1 | 22084118 | 22448123 | 1.903985706 | 4 | amp |
| TCGA-04-1362 | 1 | 22816399 | 23743838 | 1.109199544 | 3 | amp |
| TCGA-04-1362 | 1 | 23744389 | 24400764 | 1.721368694 | 4 | amp |
| TCGA-04-1362 | 1 | 24401766 | 24664597 | 2.308223221 | 5 | amp |
| TCGA-04-1362 | 1 | 24666114 | 26360386 | 1.500425998 | 4 | amp |
| TCGA-04-1362 | 1 | 26361260 | 26566361 | 2.346417318 | 5 | amp |
| TCGA-04-1362 | 1 | 26570582 | 26611032 | 1.517313034 | 4 | amp |
| TCGA-04-1362 | 1 | 26620608 | 26691729 | 2.342691315 | 5 | amp |

| | | | | | | |
|---|---|---|---|---|---|---|
| TCGA-04-1362 | 1 | 26694945 | 32048855 | 1.695779078 | 4 | amp |
| TCGA-04-1362 | 1 | 32050190 | 32267375 | 2.594807053 | 5 | amp |
| TCGA-04-1362 | 1 | 32374446 | 32636521 | 1.679862235 | 4 | amp |
| TCGA-04-1362 | 1 | 32650111 | 32696811 | 2.291474072 | 5 | amp |
| TCGA-04-1362 | 1 | 32712942 | 35469647 | 1.494158886 | 4 | amp |
| TCGA-04-1362 | 1 | 35470682 | 36307397 | 1.046867158 | 3 | amp |
| TCGA-04-1362 | 1 | 36315748 | 36557442 | 1.409263754 | 4 | amp |
| TCGA-04-1362 | 1 | 36558662 | 36645913 | 2.888352063 | 5 | amp |
| TCGA-04-1362 | 1 | 36748112 | 36759553 | 1.339840689 | 4 | amp |
| TCGA-04-1362 | 1 | 36762127 | 37948147 | 2.695052444 | 5 | amp |
| TCGA-04-1362 | 1 | 37959613 | 37979141 | 1.501351618 | 4 | amp |
| TCGA-04-1362 | 1 | 38003300 | 38164672 | 2.258059074 | 5 | amp |
| TCGA-04-1362 | 1 | 38166090 | 38453541 | 1.854015022 | 4 | amp |
| TCGA-04-1362 | 1 | 38455529 | 38489318 | 2.713266523 | 5 | amp |
| TCGA-04-1362 | 1 | 39175270 | 40363702 | 1.461102343 | 4 | amp |
| TCGA-04-1362 | 1 | 40422705 | 40435268 | 2.699181766 | 5 | amp |
| TCGA-04-1362 | 1 | 40524984 | 43221323 | 1.515458516 | 4 | amp |
| TCGA-04-1362 | 1 | 43223403 | 43638622 | 2.395619537 | 5 | amp |
| TCGA-04-1362 | 1 | 43647182 | 43675755 | 1.050006083 | 3 | amp |
| TCGA-04-1362 | 1 | 43748436 | 44134990 | 2.525180382 | 5 | amp |
| TCGA-04-1362 | 1 | 44137120 | 44438268 | 1.727138065 | 4 | amp |
| TCGA-04-1362 | 1 | 44441405 | 45307734 | 2.464889924 | 5 | amp |
| TCGA-04-1362 | 1 | 45316558 | 46546427 | 1.814618997 | 4 | amp |
| TCGA-04-1362 | 1 | 46597324 | 46659333 | 2.582344317 | 5 | amp |
| TCGA-04-1362 | 1 | 46659501 | 46740419 | 1.757793834 | 4 | amp |
| TCGA-04-1362 | 1 | 46743420 | 47080755 | 2.457201078 | 5 | amp |
| TCGA-04-1362 | 1 | 47101424 | 47284514 | 1.317684213 | 4 | amp |
| TCGA-04-1362 | 1 | 47310185 | 52299885 | 1.15352728 | 3 | amp |
| TCGA-04-1362 | 1 | 52301783 | 54607118 | 1.52151056 | 4 | amp |
| TCGA-04-1362 | 1 | 54610102 | 55197385 | 2.463367387 | 5 | amp |
| TCGA-04-1362 | 1 | 55199282 | 55600103 | 1.589297541 | 4 | amp |
| TCGA-04-1362 | 1 | 55603154 | 60499350 | 1.088133448 | 3 | amp |
| TCGA-04-1362 | 1 | 60503661 | 62958566 | 1.509763064 | 4 | amp |
| TCGA-04-1362 | 1 | 62959921 | 65270790 | 0.979703185 | 3 | amp |
| TCGA-04-1362 | 1 | 65272842 | 65325986 | 1.951773412 | 4 | amp |
| TCGA-04-1362 | 1 | 65330442 | 67508024 | 1.057418678 | 3 | amp |
| TCGA-04-1362 | 1 | 67512938 | 68915642 | 1.447314139 | 4 | amp |
| TCGA-04-1362 | 1 | 68942533 | 84874220 | 1.108104851 | 3 | amp |
| TCGA-04-1362 | 1 | 84876481 | 85115684 | 1.729574369 | 4 | amp |
| TCGA-04-1362 | 1 | 85115977 | 93744086 | 1.128828072 | 3 | amp |
| TCGA-04-1362 | 1 | 93819396 | 94362394 | 1.728852257 | 4 | amp |
| TCGA-04-1362 | 1 | 94363364 | 94509059 | 2.455868708 | 5 | amp |
| TCGA-04-1362 | 1 | 94510114 | 94697184 | 1.629335172 | 4 | amp |
| TCGA-04-1362 | 1 | 94924120 | 108691014 | 1.041672771 | 3 | amp |
| TCGA-04-1362 | 1 | 108697560 | 109566175 | 1.585666955 | 4 | amp |
| TCGA-04-1362 | 1 | 109607109 | 109840894 | 2.575915255 | 5 | amp |
| TCGA-04-1362 | 1 | 109856581 | 109955783 | 1.62938464 | 4 | amp |
| TCGA-04-1362 | 1 | 109957801 | 110087042 | 2.417133455 | 5 | amp |
| TCGA-04-1362 | 1 | 110116319 | 110152852 | 1.323337962 | 4 | amp |
| TCGA-04-1362 | 1 | 110153047 | 110467849 | 3.073124242 | 5 | amp |

| | | | | | | |
|---|---|---|---|---|---|---|
| TCGA-04-1362 | 1 | 110551651 | 110564358 | 1.519544811 | 4 | amp |
| TCGA-04-1362 | 1 | 110580487 | 110884898 | 2.908474361 | 5 | amp |
| TCGA-04-1362 | 1 | 110888135 | 113212255 | 1.50921231 | 4 | amp |
| TCGA-04-1362 | 1 | 113212563 | 113636231 | 3.238276716 | 5 | amp |
| TCGA-04-1362 | 1 | 113636912 | 114505119 | 1.445341383 | 4 | amp |
| TCGA-04-1362 | 1 | 114505955 | 114682544 | 2.358503387 | 5 | amp |
| TCGA-04-1362 | 1 | 114940255 | 115168622 | 1.061244864 | 3 | amp |
| TCGA-04-1362 | 1 | 115215693 | 115227022 | 2.542278708 | 5 | amp |
| TCGA-04-1362 | 1 | 115229328 | 115262401 | 1.359557076 | 4 | amp |
| TCGA-04-1362 | 1 | 115263128 | 120057282 | 1.160612919 | 3 | amp |
| TCGA-04-1362 | 1 | 120168490 | 145368622 | 1.468742864 | 4 | amp |
| TCGA-04-1362 | 1 | 145414739 | 145609382 | 3.4391919 | 5 | amp |
| TCGA-04-1362 | 1 | 145646083 | 147092882 | 1.389858321 | 4 | amp |
| TCGA-04-1362 | 1 | 147094021 | 147416221 | 2.455612761 | 5 | amp |
| TCGA-04-1362 | 1 | 147425511 | 149755866 | 1.435812589 | 4 | amp |
| TCGA-04-1362 | 1 | 149759867 | 150601618 | 3.536189417 | 5 | amp |
| TCGA-04-1362 | 1 | 150620770 | 150672683 | 1.403489332 | 4 | amp |
| TCGA-04-1362 | 1 | 150675767 | 150776725 | 2.420199031 | 5 | amp |
| TCGA-04-1362 | 1 | 150778264 | 150808982 | 1.862931655 | 4 | amp |
| TCGA-04-1362 | 1 | 150811903 | 151171574 | 2.959016645 | 5 | amp |
| TCGA-04-1362 | 1 | 151196679 | 151215098 | 1.27717974 | 4 | amp |
| TCGA-04-1362 | 1 | 151219358 | 151374358 | 2.249866325 | 5 | amp |
| TCGA-04-1362 | 1 | 151377268 | 154002570 | 1.505732776 | 4 | amp |
| TCGA-04-1362 | 1 | 154018503 | 154303632 | 1.111502568 | 3 | amp |
| TCGA-04-1362 | 1 | 154303860 | 155167754 | 1.689816768 | 4 | amp |
| TCGA-04-1362 | 1 | 155167801 | 155228800 | 2.366939058 | 5 | amp |
| TCGA-04-1362 | 1 | 155230060 | 155921609 | 1.460955843 | 4 | amp |
| TCGA-04-1362 | 1 | 155921653 | 156146791 | 2.310016108 | 5 | amp |
| TCGA-04-1362 | 1 | 156169590 | 156347304 | 1.785038655 | 4 | amp |
| TCGA-04-1362 | 1 | 156347735 | 156509825 | 2.376282822 | 5 | amp |
| TCGA-04-1362 | 1 | 156510483 | 156621514 | 1.584589082 | 4 | amp |
| TCGA-04-1362 | 1 | 156621979 | 156931577 | 2.394123495 | 5 | amp |
| TCGA-04-1362 | 1 | 156932955 | 157494387 | 1.531804845 | 4 | amp |
| TCGA-04-1362 | 1 | 157497365 | 158326721 | 1.059194371 | 3 | amp |
| TCGA-04-1362 | 1 | 158906652 | 159019425 | 1.106622722 | 3 | amp |
| TCGA-04-1362 | 1 | 159021408 | 160395217 | 1.544406478 | 4 | amp |
| TCGA-04-1362 | 1 | 160456460 | 160811712 | 0.776055263 | 3 | amp |
| TCGA-04-1362 | 1 | 160832346 | 161043607 | 1.666065844 | 4 | amp |
| TCGA-04-1362 | 1 | 161043964 | 161182325 | 2.538086253 | 5 | amp |
| TCGA-04-1362 | 1 | 161183157 | 161519680 | 1.605576216 | 4 | amp |
| TCGA-04-1362 | 1 | 161551223 | 168344870 | 1.155841137 | 3 | amp |
| TCGA-04-1362 | 1 | 179604791 | 180974634 | 1.054184657 | 3 | amp |
| TCGA-04-1362 | 1 | 181003086 | 182775383 | 1.547273233 | 4 | amp |
| TCGA-04-1362 | 1 | 182781229 | 184859358 | 1.109944585 | 3 | amp |
| TCGA-04-1362 | 1 | 199996947 | 200822632 | 1.101984245 | 3 | amp |
| TCGA-04-1362 | 1 | 200823890 | 200827214 | 1.447437752 | 4 | amp |
| TCGA-04-1362 | 1 | 200867388 | 201789072 | 2.811737558 | 5 | amp |
| TCGA-04-1362 | 1 | 201816347 | 201958221 | 1.401179871 | 4 | amp |
| TCGA-04-1362 | 1 | 201958434 | 202304887 | 2.387558844 | 5 | amp |
| TCGA-04-1362 | 1 | 202385898 | 202990118 | 1.434090792 | 4 | amp |

| | | | | | | |
|---|---|---|---|---|---|---|
| TCGA-04-1362 | 1 | 202991955 | 203456041 | 2.919296542 | 5 | amp |
| TCGA-04-1362 | 1 | 203652279 | 206943266 | 1.421627409 | 4 | amp |
| TCGA-04-1362 | 1 | 206944177 | 207271579 | 1.103761071 | 3 | amp |
| TCGA-04-1362 | 1 | 207780578 | 207966961 | 1.088676451 | 3 | amp |
| TCGA-04-1362 | 1 | 207975767 | 209781300 | 1.958202748 | 4 | amp |
| TCGA-04-1362 | 1 | 209782320 | 209823505 | 2.275305917 | 5 | amp |
| TCGA-04-1362 | 1 | 209824185 | 210003532 | 1.523818828 | 4 | amp |
| TCGA-04-1362 | 1 | 210004128 | 214792577 | 1.098584131 | 3 | amp |
| TCGA-04-1362 | 1 | 220826388 | 225755118 | 1.09534983 | 3 | amp |
| TCGA-04-1362 | 1 | 225970975 | 227173057 | 1.776229187 | 4 | amp |
| TCGA-04-1362 | 1 | 227182482 | 227843562 | 0.883020883 | 3 | amp |
| TCGA-04-1362 | 1 | 227935333 | 228437983 | 2.022063047 | 4 | amp |
| TCGA-04-1362 | 1 | 228479550 | 228613060 | 2.967413173 | 5 | amp |
| TCGA-04-1362 | 1 | 228645750 | 229422362 | 1.507511155 | 4 | amp |
| TCGA-04-1362 | 1 | 229424472 | 229676545 | 0.997276306 | 3 | amp |
| TCGA-04-1362 | 1 | 229677930 | 235612090 | 1.396540544 | 4 | amp |
| TCGA-04-1362 | 1 | 235613467 | 247040651 | 1.055421899 | 3 | amp |
| TCGA-04-1362 | 10 | 14816216 | 15600256 | 1.413979491 | 4 | amp |
| TCGA-04-1362 | 10 | 15614168 | 22029182 | 1.041367268 | 3 | amp |
| TCGA-04-1362 | 10 | 22030829 | 23221009 | 1.587721177 | 4 | amp |
| TCGA-04-1362 | 10 | 23235011 | 24835255 | 1.056462517 | 3 | amp |
| TCGA-04-1362 | 10 | 28903404 | 30336780 | 1.439317229 | 4 | amp |
| TCGA-04-1362 | 10 | 30602478 | 31750185 | 1.097351511 | 3 | amp |
| TCGA-04-1362 | 10 | 34985236 | 37455637 | 1.506280228 | 4 | amp |
| TCGA-04-1362 | 10 | 37458411 | 43090183 | 1.109712574 | 3 | amp |
| TCGA-04-1362 | 10 | 43127334 | 43679023 | 1.649601106 | 4 | amp |
| TCGA-04-1362 | 10 | 43691613 | 45956888 | 2.392245785 | 5 | amp |
| TCGA-04-1362 | 10 | 45959640 | 46938387 | 1.468320396 | 4 | amp |
| TCGA-04-1362 | 10 | 46959933 | 47894662 | 2.482873749 | 5 | amp |
| TCGA-04-1362 | 10 | 47896622 | 48195879 | 1.359111637 | 4 | amp |
| TCGA-04-1362 | 10 | 48215827 | 48739478 | 2.582550298 | 5 | amp |
| TCGA-04-1362 | 10 | 48751794 | 50820425 | 1.334265989 | 4 | amp |
| TCGA-04-1362 | 10 | 50824058 | 50952806 | 2.391719663 | 5 | amp |
| TCGA-04-1362 | 10 | 50959799 | 51892755 | 1.524034693 | 4 | amp |
| TCGA-04-1362 | 10 | 51947049 | 70210014 | 1.050290711 | 3 | amp |
| TCGA-04-1362 | 10 | 70218806 | 71007352 | 1.459735275 | 4 | amp |
| TCGA-04-1362 | 10 | 71008121 | 71921702 | 2.601947975 | 5 | amp |
| TCGA-04-1362 | 10 | 71978486 | 72541794 | 2.478978248 | 5 | amp |
| TCGA-04-1362 | 10 | 72576532 | 72636477 | 1.63014097 | 4 | amp |
| TCGA-04-1362 | 10 | 72636871 | 73826888 | 3.079337681 | 5 | amp |
| TCGA-04-1362 | 10 | 73856999 | 74128152 | 1.757113418 | 4 | amp |
| TCGA-04-1362 | 10 | 74135525 | 74831982 | 1.00881006 | 3 | amp |
| TCGA-04-1362 | 10 | 74833476 | 75394546 | 1.339836956 | 4 | amp |
| TCGA-04-1362 | 10 | 75397440 | 75671872 | 2.51671554 | 5 | amp |
| TCGA-04-1362 | 10 | 75671936 | 75984382 | 1.52885448 | 4 | amp |
| TCGA-04-1362 | 10 | 76074403 | 76781083 | 0.840225893 | 3 | amp |
| TCGA-04-1362 | 10 | 76781610 | 79011043 | 1.416731307 | 4 | amp |
| TCGA-04-1362 | 10 | 79163550 | 79628980 | 2.299746841 | 5 | amp |
| TCGA-04-1362 | 10 | 79737189 | 79786039 | 1.429598286 | 4 | amp |
| TCGA-04-1362 | 10 | 79789053 | 82369364 | 2.268824388 | 5 | amp |

| | | | | | | |
|---|---|---|---|---|---|---|
| TCGA-04-1362 | 10 | 82403732 | 84745407 | 1.102170045 | 3 | amp |
| TCGA-04-1362 | 10 | 85901200 | 86008852 | 2.639600487 | 5 | amp |
| TCGA-04-1362 | 10 | 86012569 | 89268311 | 1.624261939 | 4 | amp |
| TCGA-04-1362 | 10 | 89272869 | 94774077 | 1.038170629 | 3 | amp |
| TCGA-04-1362 | 10 | 94816676 | 95141184 | 2.321353136 | 5 | amp |
| TCGA-04-1362 | 10 | 95147491 | 95263187 | 1.405442628 | 4 | amp |
| TCGA-04-1362 | 10 | 95266718 | 96979757 | 1.075990439 | 3 | amp |
| TCGA-04-1362 | 10 | 96997624 | 99118394 | 1.437005944 | 4 | amp |
| TCGA-04-1362 | 10 | 99120248 | 99219069 | 2.782855668 | 5 | amp |
| TCGA-04-1362 | 10 | 99219396 | 99238163 | 1.515269888 | 4 | amp |
| TCGA-04-1362 | 10 | 99240688 | 99771114 | 2.362181389 | 5 | amp |
| TCGA-04-1362 | 10 | 100008652 | 100203058 | 2.287693168 | 5 | amp |
| TCGA-04-1362 | 10 | 100219263 | 101421413 | 1.348506921 | 4 | amp |
| TCGA-04-1362 | 10 | 101439000 | 101579055 | 0.999681302 | 3 | amp |
| TCGA-04-1362 | 10 | 101590006 | 102719278 | 1.658207913 | 4 | amp |
| TCGA-04-1362 | 10 | 102721591 | 102824689 | 2.817425105 | 5 | amp |
| TCGA-04-1362 | 10 | 103174362 | 103773812 | 1.682825713 | 4 | amp |
| TCGA-04-1362 | 10 | 103783186 | 103919332 | 2.296972983 | 5 | amp |
| TCGA-04-1362 | 10 | 103919635 | 104130026 | 1.79974117 | 4 | amp |
| TCGA-04-1362 | 10 | 104130097 | 104493395 | 2.627426327 | 5 | amp |
| TCGA-04-1362 | 10 | 104495582 | 105183417 | 1.662777757 | 4 | amp |
| TCGA-04-1362 | 10 | 105184711 | 105642640 | 2.487366419 | 5 | amp |
| TCGA-04-1362 | 10 | 105648805 | 105785524 | 1.420255581 | 4 | amp |
| TCGA-04-1362 | 10 | 105791960 | 105820041 | 2.40774804 | 5 | amp |
| TCGA-04-1362 | 10 | 105821991 | 105990654 | 1.210805056 | 3 | amp |
| TCGA-04-1362 | 10 | 106019324 | 106059083 | 2.494310296 | 5 | amp |
| TCGA-04-1362 | 10 | 106074117 | 106118419 | 1.331706088 | 4 | amp |
| TCGA-04-1362 | 10 | 106121734 | 114169473 | 1.049921644 | 3 | amp |
| TCGA-04-1362 | 10 | 114170176 | 115335769 | 1.585936721 | 4 | amp |
| TCGA-04-1362 | 10 | 115336846 | 115357839 | 2.332455997 | 5 | amp |
| TCGA-04-1362 | 10 | 115364343 | 115374711 | 1.530883713 | 4 | amp |
| TCGA-04-1362 | 10 | 115375462 | 115987751 | 0.978658955 | 3 | amp |
| TCGA-04-1362 | 10 | 115987780 | 116021059 | 1.553767626 | 4 | amp |
| TCGA-04-1362 | 10 | 116032445 | 116100645 | 2.357702931 | 5 | amp |
| TCGA-04-1362 | 10 | 116196015 | 116444140 | 1.356145796 | 4 | amp |
| TCGA-04-1362 | 10 | 116590589 | 118204079 | 0.859529683 | 3 | amp |
| TCGA-04-1362 | 10 | 118215197 | 118423742 | 1.38414383 | 4 | amp |
| TCGA-04-1362 | 10 | 118424214 | 118667474 | 2.247572449 | 5 | amp |
| TCGA-04-1362 | 10 | 118671267 | 118738852 | 1.242030786 | 3 | amp |
| TCGA-04-1362 | 10 | 118891706 | 119017415 | 2.247572449 | 5 | amp |
| TCGA-04-1362 | 10 | 119026192 | 120920615 | 1.512032981 | 4 | amp |
| TCGA-04-1362 | 10 | 120921828 | 121212842 | 2.687412918 | 5 | amp |
| TCGA-04-1362 | 10 | 121259547 | 121583430 | 1.468506381 | 4 | amp |
| TCGA-04-1362 | 10 | 121586087 | 122660638 | 0.969152094 | 3 | amp |
| TCGA-04-1362 | 10 | 122661741 | 123730668 | 1.498304602 | 4 | amp |
| TCGA-04-1362 | 10 | 123733497 | 124152906 | 2.219382336 | 5 | amp |
| TCGA-04-1362 | 10 | 124214197 | 124459338 | 2.427920622 | 5 | amp |
| TCGA-04-1362 | 10 | 124591759 | 128936312 | 1.586003348 | 4 | amp |
| TCGA-04-1362 | 10 | 128944164 | 129897546 | 2.427029309 | 5 | amp |
| TCGA-04-1362 | 10 | 129899503 | 129914331 | 1.337777167 | 4 | amp |

| | | | | | | |
|---|---|---|---|---|---|---|
| TCGA-04-1362 | 10 | 129914717 | 135209778 | 2.647383556 | 5 | amp |
| TCGA-04-1362 | 10 | 135211918 | 135516111 | 1.897316164 | 4 | amp |
| TCGA-04-1362 | 11 | 86637 | 34152541 | 1.350324558 | 4 | amp |
| TCGA-04-1362 | 11 | 34152882 | 34192655 | 3.639473018 | 5 | amp |
| TCGA-04-1362 | 11 | 34470704 | 43876805 | 1.487040027 | 4 | amp |
| TCGA-04-1362 | 11 | 43904181 | 43941573 | 2.897924801 | 5 | amp |
| TCGA-04-1362 | 11 | 44069538 | 44265887 | 1.783088436 | 4 | amp |
| TCGA-04-1362 | 11 | 44288957 | 45959905 | 3.879393661 | 5 | amp |
| TCGA-04-1362 | 11 | 45967352 | 46693903 | 1.829339107 | 4 | amp |
| TCGA-04-1362 | 11 | 46700530 | 46744842 | 4.466997427 | 5 | amp |
| TCGA-04-1362 | 11 | 46750180 | 47074089 | 1.494688202 | 4 | amp |
| TCGA-04-1362 | 11 | 47159177 | 47295556 | 3.591815209 | 5 | amp |
| TCGA-04-1362 | 11 | 47296051 | 47350708 | 2.033801356 | 4 | amp |
| TCGA-04-1362 | 11 | 47353595 | 47464406 | 3.34224133 | 5 | amp |
| TCGA-04-1362 | 11 | 47493695 | 47593221 | 1.655514771 | 4 | amp |
| TCGA-04-1362 | 11 | 47594431 | 47606069 | 3.050610136 | 5 | amp |
| TCGA-04-1362 | 11 | 47640354 | 57100647 | 1.439528371 | 4 | amp |
| TCGA-04-1362 | 11 | 57100901 | 57135937 | 2.997894768 | 5 | amp |
| TCGA-04-1362 | 11 | 57136782 | 60558019 | 1.391195836 | 4 | amp |
| TCGA-04-1362 | 11 | 60558386 | 60567492 | 3.324683753 | 5 | amp |
| TCGA-04-1362 | 11 | 60615311 | 61109399 | 2.095330502 | 4 | amp |
| TCGA-04-1362 | 11 | 61109859 | 61133715 | 3.437004162 | 5 | amp |
| TCGA-04-1362 | 11 | 61135342 | 61254536 | 1.934060425 | 4 | amp |
| TCGA-04-1362 | 11 | 61254574 | 61909037 | 7.926507219 | 5 | amp |
| TCGA-04-1362 | 11 | 61919220 | 62065156 | 1.937950061 | 4 | amp |
| TCGA-04-1362 | 11 | 62066394 | 62782443 | 4.735537526 | 5 | amp |
| TCGA-04-1362 | 11 | 62931210 | 63257894 | 1.41145055 | 4 | amp |
| TCGA-04-1362 | 11 | 63273811 | 66488752 | 4.434814874 | 5 | amp |
| TCGA-04-1362 | 11 | 66555625 | 66595845 | 1.883310081 | 4 | amp |
| TCGA-04-1362 | 11 | 66605816 | 66838082 | 4.400927964 | 5 | amp |
| TCGA-04-1362 | 11 | 66888748 | 66983433 | 2.036988429 | 4 | amp |
| TCGA-04-1362 | 11 | 66986756 | 72040859 | 4.22098034 | 5 | amp |
| TCGA-04-1362 | 11 | 72069903 | 72084073 | 1.74676683 | 4 | amp |
| TCGA-04-1362 | 11 | 72091316 | 73718144 | 4.318667272 | 5 | amp |
| TCGA-04-1362 | 11 | 73745587 | 73950359 | 1.734575659 | 4 | amp |
| TCGA-04-1362 | 11 | 73958085 | 73967058 | 3.094401366 | 5 | amp |
| TCGA-04-1362 | 11 | 73978267 | 74082868 | 2.040913464 | 4 | amp |
| TCGA-04-1362 | 11 | 74085396 | 74322623 | 3.220068924 | 5 | amp |
| TCGA-04-1362 | 11 | 74323868 | 74351829 | 2.074173559 | 4 | amp |
| TCGA-04-1362 | 11 | 74407526 | 76207565 | 3.695148383 | 5 | amp |
| TCGA-04-1362 | 11 | 76224384 | 76261212 | 1.234112769 | 3 | amp |
| TCGA-04-1362 | 11 | 76370639 | 76696787 | 3.500579518 | 5 | amp |
| TCGA-04-1362 | 11 | 76701510 | 76731404 | 1.442030106 | 4 | amp |
| TCGA-04-1362 | 11 | 76825253 | 76940309 | 5.716777305 | 5 | amp |
| TCGA-04-1362 | 11 | 76944023 | 77583410 | 1.992059999 | 4 | amp |
| TCGA-04-1362 | 11 | 77589963 | 77938103 | 3.053716527 | 5 | amp |
| TCGA-04-1362 | 11 | 77961114 | 78279857 | 2.108658001 | 4 | amp |
| TCGA-04-1362 | 11 | 78282374 | 82874934 | 2.724090395 | 5 | amp |
| TCGA-04-1362 | 11 | 82875225 | 85309780 | 2.03761214 | 4 | amp |
| TCGA-04-1362 | 11 | 85342735 | 85445803 | 3.103580704 | 5 | amp |

| | | | | | | |
|---|---|---|---|---|---|---|
| TCGA-04-1362 | 11 | 85447539 | 86126364 | 1.967260717 | 4 | amp |
| TCGA-04-1362 | 11 | 86130882 | 86219931 | 3.291260922 | 5 | amp |
| TCGA-04-1362 | 11 | 86267519 | 113264529 | 1.287979287 | 4 | amp |
| TCGA-04-1362 | 11 | 113266045 | 113566203 | 3.462852452 | 5 | amp |
| TCGA-04-1362 | 11 | 113567952 | 117052858 | 1.603341395 | 4 | amp |
| TCGA-04-1362 | 11 | 117053225 | 117691651 | 2.731430124 | 5 | amp |
| TCGA-04-1362 | 11 | 117710470 | 117988682 | 2.05799145 | 4 | amp |
| TCGA-04-1362 | 11 | 118007728 | 118047171 | 2.970771794 | 5 | amp |
| TCGA-04-1362 | 11 | 118065025 | 118471444 | 1.502407011 | 4 | amp |
| TCGA-04-1362 | 11 | 118486690 | 118532451 | 4.306897501 | 5 | amp |
| TCGA-04-1362 | 11 | 118625388 | 118656963 | 1.486143515 | 4 | amp |
| TCGA-04-1362 | 11 | 118754615 | 119063965 | 3.157097971 | 5 | amp |
| TCGA-04-1362 | 11 | 119103071 | 119229574 | 2.054992592 | 4 | amp |
| TCGA-04-1362 | 11 | 119229728 | 120139995 | 4.006008765 | 5 | amp |
| TCGA-04-1362 | 11 | 120168886 | 122850186 | 1.660478319 | 4 | amp |
| TCGA-04-1362 | 11 | 122928414 | 124637965 | 2.516574042 | 5 | amp |
| TCGA-04-1362 | 11 | 124642859 | 124644724 | 1.716113502 | 4 | amp |
| TCGA-04-1362 | 11 | 124739903 | 124793945 | 5.147695971 | 5 | amp |
| TCGA-04-1362 | 11 | 124794584 | 124910666 | 2.037125424 | 4 | amp |
| TCGA-04-1362 | 11 | 124947082 | 125507451 | 4.073611901 | 5 | amp |
| TCGA-04-1362 | 11 | 125513680 | 125618681 | 1.82560751 | 4 | amp |
| TCGA-04-1362 | 11 | 125647224 | 125831935 | 3.124204659 | 5 | amp |
| TCGA-04-1362 | 11 | 125848178 | 126073612 | 1.495173667 | 4 | amp |
| TCGA-04-1362 | 11 | 126074078 | 126081558 | 3.390666083 | 5 | amp |
| TCGA-04-1362 | 11 | 126104879 | 126126784 | 1.959296161 | 4 | amp |
| TCGA-04-1362 | 11 | 126131289 | 126432828 | 4.250716497 | 5 | amp |
| TCGA-04-1362 | 11 | 128332243 | 129312831 | 2.130299905 | 4 | amp |
| TCGA-04-1362 | 11 | 129320953 | 130109808 | 3.984512092 | 5 | amp |
| TCGA-04-1362 | 11 | 130130719 | 130131799 | 1.635781794 | 4 | amp |
| TCGA-04-1362 | 11 | 130275407 | 130341025 | 4.031956139 | 5 | amp |
| TCGA-04-1362 | 11 | 130748232 | 130773334 | 2.081097498 | 4 | amp |
| TCGA-04-1362 | 11 | 130775817 | 134251934 | 2.719598198 | 5 | amp |
| TCGA-04-1362 | 12 | 73256 | 353031 | 3.880695114 | 5 | amp |
| TCGA-04-1362 | 12 | 368997 | 551132 | 1.578323013 | 4 | amp |
| TCGA-04-1362 | 12 | 645362 | 674603 | 4.023259452 | 5 | amp |
| TCGA-04-1362 | 12 | 675109 | 1895293 | 1.730054196 | 4 | amp |
| TCGA-04-1362 | 12 | 1910186 | 3951253 | 3.060894024 | 5 | amp |
| TCGA-04-1362 | 12 | 3951255 | 6062770 | 1.824406758 | 4 | amp |
| TCGA-04-1362 | 12 | 6076559 | 6604447 | 2.753711865 | 5 | amp |
| TCGA-04-1362 | 12 | 6618830 | 6637536 | 2.027177176 | 4 | amp |
| TCGA-04-1362 | 12 | 6637783 | 7355313 | 2.91301867 | 5 | amp |
| TCGA-04-1362 | 12 | 7356039 | 8693418 | 1.817239904 | 4 | amp |
| TCGA-04-1362 | 12 | 8756816 | 8814731 | 2.600252353 | 5 | amp |
| TCGA-04-1362 | 12 | 8866429 | 9360955 | 1.760395126 | 4 | amp |
| TCGA-04-1362 | 12 | 9392014 | 9698388 | 2.801002193 | 5 | amp |
| TCGA-04-1362 | 12 | 9704014 | 30894056 | 1.327367311 | 4 | amp |
| TCGA-04-1362 | 12 | 30903975 | 31285280 | 3.272375247 | 5 | amp |
| TCGA-04-1362 | 12 | 31286016 | 45568178 | 1.517924459 | 4 | amp |
| TCGA-04-1362 | 12 | 45695776 | 48081841 | 1.08672658 | 3 | amp |
| TCGA-04-1362 | 12 | 48082882 | 48110302 | 1.852477295 | 4 | amp |

| | | | | | | |
|---|---|---|---|---|---|---|
| TCGA-04-1362 | 12 | 48110636 | 48392265 | 3.87991326 | 5 | amp |
| TCGA-04-1362 | 12 | 48439075 | 49110497 | 1.530628181 | 4 | amp |
| TCGA-04-1362 | 12 | 49162316 | 50369470 | 3.132969861 | 5 | amp |
| TCGA-04-1362 | 12 | 50383998 | 50410515 | 1.693039404 | 4 | amp |
| TCGA-04-1362 | 12 | 50452490 | 50501919 | 2.812540186 | 5 | amp |
| TCGA-04-1362 | 12 | 50503193 | 51723662 | 1.548674164 | 4 | amp |
| TCGA-04-1362 | 12 | 51733566 | 51847522 | 2.868289887 | 5 | amp |
| TCGA-04-1362 | 12 | 51851108 | 52407577 | 1.734281502 | 4 | amp |
| TCGA-04-1362 | 12 | 52407603 | 53010176 | 3.120672984 | 5 | amp |
| TCGA-04-1362 | 12 | 53011844 | 53085798 | 1.977935483 | 4 | amp |
| TCGA-04-1362 | 12 | 53086164 | 53291490 | 2.726100809 | 5 | amp |
| TCGA-04-1362 | 12 | 53292452 | 53434026 | 1.963902108 | 4 | amp |
| TCGA-04-1362 | 12 | 53445602 | 53513092 | 3.058987087 | 5 | amp |
| TCGA-04-1362 | 12 | 53514558 | 53579275 | 1.889849722 | 4 | amp |
| TCGA-04-1362 | 12 | 53579679 | 53925723 | 3.27104679 | 5 | amp |
| TCGA-04-1362 | 12 | 53926933 | 54115444 | 1.891326366 | 4 | amp |
| TCGA-04-1362 | 12 | 54115737 | 54891684 | 3.06892648 | 5 | amp |
| TCGA-04-1362 | 12 | 54893133 | 54936473 | 1.10775406 | 3 | amp |
| TCGA-04-1362 | 12 | 54943622 | 54977073 | 2.845413612 | 5 | amp |
| TCGA-04-1362 | 12 | 55024646 | 56031626 | 1.344567625 | 4 | amp |
| TCGA-04-1362 | 12 | 56075652 | 56142808 | 3.026621454 | 5 | amp |
| TCGA-04-1362 | 12 | 56143235 | 56211527 | 1.685135928 | 4 | amp |
| TCGA-04-1362 | 12 | 56212750 | 56812150 | 3.310876082 | 5 | amp |
| TCGA-04-1362 | 12 | 56814326 | 56827965 | 1.977376228 | 4 | amp |
| TCGA-04-1362 | 12 | 56845036 | 57039165 | 2.863966212 | 5 | amp |
| TCGA-04-1362 | 12 | 57058212 | 57458535 | 1.96434231 | 4 | amp |
| TCGA-04-1362 | 12 | 57464549 | 57660623 | 3.912157661 | 5 | amp |
| TCGA-04-1362 | 12 | 57662042 | 57677722 | 1.3591226 | 4 | amp |
| TCGA-04-1362 | 12 | 57828615 | 58220216 | 3.368674426 | 5 | amp |
| TCGA-04-1362 | 12 | 58220739 | 88542306 | 1.373287477 | 4 | amp |
| TCGA-04-1362 | 12 | 88547056 | 93285510 | 1.041863055 | 3 | amp |
| TCGA-04-1362 | 12 | 93789226 | 96312763 | 1.482544201 | 4 | amp |
| TCGA-04-1362 | 12 | 96330208 | 96389744 | 2.50401758 | 5 | amp |
| TCGA-04-1362 | 12 | 96394705 | 104034087 | 1.552287803 | 4 | amp |
| TCGA-04-1362 | 12 | 104042414 | 104069812 | 2.712355004 | 5 | amp |
| TCGA-04-1362 | 12 | 104071159 | 109095116 | 1.615835296 | 4 | amp |
| TCGA-04-1362 | 12 | 109181731 | 109286844 | 2.677179687 | 5 | amp |
| TCGA-04-1362 | 12 | 109290732 | 109519228 | 1.688383978 | 4 | amp |
| TCGA-04-1362 | 12 | 109519720 | 109684298 | 2.572691147 | 5 | amp |
| TCGA-04-1362 | 12 | 109685369 | 109939377 | 2.077915985 | 4 | amp |
| TCGA-04-1362 | 12 | 109940761 | 110386699 | 3.191476047 | 5 | amp |
| TCGA-04-1362 | 12 | 110388926 | 111322063 | 1.7093505 | 4 | amp |
| TCGA-04-1362 | 12 | 111330856 | 111894066 | 3.10008457 | 5 | amp |
| TCGA-04-1362 | 12 | 111894940 | 112174841 | 1.540636545 | 4 | amp |
| TCGA-04-1362 | 12 | 112182406 | 112304057 | 2.892139714 | 5 | amp |
| TCGA-04-1362 | 12 | 112305364 | 113448293 | 1.508976801 | 4 | amp |
| TCGA-04-1362 | 12 | 113532552 | 114393035 | 2.948837581 | 5 | amp |
| TCGA-04-1362 | 12 | 114395540 | 120510543 | 1.691872876 | 4 | amp |
| TCGA-04-1362 | 12 | 120518635 | 120936064 | 2.700725886 | 5 | amp |
| TCGA-04-1362 | 12 | 120941577 | 121613295 | 2.024700576 | 4 | amp |

| | | | | | | |
|---|---|---|---|---|---|---|
| TCGA-04-1362 | 12 | 121614922 | 121756273 | 2.823600235 | 5 | amp |
| TCGA-04-1362 | 12 | 121756297 | 121986894 | 1.96122577 | 4 | amp |
| TCGA-04-1362 | 12 | 121987303 | 122734624 | 2.758640547 | 5 | amp |
| TCGA-04-1362 | 12 | 122735437 | 123835042 | 1.579417441 | 4 | amp |
| TCGA-04-1362 | 12 | 123875104 | 124109431 | 2.604163679 | 5 | amp |
| TCGA-04-1362 | 12 | 124110945 | 124330461 | 1.722082063 | 4 | amp |
| TCGA-04-1362 | 12 | 124330489 | 125811255 | 2.562896181 | 5 | amp |
| TCGA-04-1362 | 12 | 125833963 | 130892382 | 1.701551434 | 4 | amp |
| TCGA-04-1362 | 12 | 130897097 | 133428383 | 2.777664978 | 5 | amp |
| TCGA-04-1362 | 12 | 133432984 | 133779395 | 1.587795534 | 4 | amp |
| TCGA-04-1362 | 13 | 19255757 | 19982127 | 1.950226944 | 4 | amp |
| TCGA-04-1362 | 13 | 19997192 | 20067076 | 1.188025299 | 3 | amp |
| TCGA-04-1362 | 13 | 20067554 | 21364779 | 1.682044272 | 4 | amp |
| TCGA-04-1362 | 13 | 21370184 | 21442847 | 0.938934525 | 3 | amp |
| TCGA-04-1362 | 13 | 21535765 | 24449076 | 1.709299334 | 4 | amp |
| TCGA-04-1362 | 13 | 24453359 | 25341516 | 2.178626814 | 5 | amp |
| TCGA-04-1362 | 13 | 25348906 | 25376733 | 0.984115303 | 3 | amp |
| TCGA-04-1362 | 13 | 25378396 | 25887215 | 1.72250542 | 4 | amp |
| TCGA-04-1362 | 13 | 25887670 | 26104246 | 0.835768927 | 3 | amp |
| TCGA-04-1362 | 13 | 26104688 | 27828006 | 1.518124901 | 4 | amp |
| TCGA-04-1362 | 13 | 27828327 | 28014595 | 2.179754131 | 5 | amp |
| TCGA-04-1362 | 13 | 28122389 | 28155876 | 0.917174511 | 3 | amp |
| TCGA-04-1362 | 13 | 28196998 | 28647587 | 1.814210033 | 4 | amp |
| TCGA-04-1362 | 13 | 28862039 | 31549073 | 1.522633793 | 4 | amp |
| TCGA-04-1362 | 13 | 31711179 | 31724324 | 0.902747134 | 3 | amp |
| TCGA-04-1362 | 13 | 31725025 | 31903841 | 1.548987881 | 4 | amp |
| TCGA-04-1362 | 13 | 32057674 | 32760596 | 1.01158041 | 3 | amp |
| TCGA-04-1362 | 13 | 32761677 | 32921058 | 1.382473314 | 4 | amp |
| TCGA-04-1362 | 13 | 32928971 | 33020584 | 1.137823674 | 3 | amp |
| TCGA-04-1362 | 13 | 33627858 | 36909982 | 1.061529327 | 3 | amp |
| TCGA-04-1362 | 13 | 37007143 | 37583438 | 1.448990936 | 4 | amp |
| TCGA-04-1362 | 13 | 37583757 | 39546749 | 1.02403488 | 3 | amp |
| TCGA-04-1362 | 13 | 39550652 | 41910898 | 1.440815898 | 4 | amp |
| TCGA-04-1362 | 13 | 41929266 | 42763488 | 1.167427193 | 3 | amp |
| TCGA-04-1362 | 13 | 43155225 | 43493511 | 1.003223848 | 3 | amp |
| TCGA-04-1362 | 13 | 43500458 | 46425816 | 1.477971035 | 4 | amp |
| TCGA-04-1362 | 13 | 46537916 | 46594697 | 0.813654318 | 3 | amp |
| TCGA-04-1362 | 13 | 46616294 | 47224527 | 1.638519487 | 4 | amp |
| TCGA-04-1362 | 13 | 47243107 | 49027307 | 0.973596138 | 3 | amp |
| TCGA-04-1362 | 13 | 49030262 | 49086019 | 1.540828998 | 4 | amp |
| TCGA-04-1362 | 13 | 49086110 | 50059872 | 1.061843295 | 3 | amp |
| TCGA-04-1362 | 13 | 50059915 | 50505306 | 1.415201889 | 4 | amp |
| TCGA-04-1362 | 13 | 50586025 | 52373852 | 1.037123585 | 3 | amp |
| TCGA-04-1362 | 13 | 52439505 | 52524339 | 2.369777864 | 5 | amp |
| TCGA-04-1362 | 13 | 52524351 | 53236870 | 1.531560737 | 4 | amp |
| TCGA-04-1362 | 13 | 53237178 | 54615423 | 1.047363791 | 3 | amp |
| TCGA-04-1362 | 13 | 54886084 | 77629917 | 1.411777912 | 4 | amp |
| TCGA-04-1362 | 13 | 77631084 | 77673209 | 1.066884925 | 3 | amp |
| TCGA-04-1362 | 13 | 77692444 | 79233279 | 1.486295578 | 4 | amp |
| TCGA-04-1362 | 13 | 79766127 | 96665767 | 1.051980263 | 3 | amp |

| | | | | | | |
|---|---|---|---|---|---|---|
| TCGA-04-1362 | 13 | 96675259 | 99340837 | 1.581138888 | 4 | amp |
| TCGA-04-1362 | 13 | 99354698 | 99376345 | 0.922438689 | 3 | amp |
| TCGA-04-1362 | 13 | 99378327 | 100190174 | 1.448393318 | 4 | amp |
| TCGA-04-1362 | 13 | 100191665 | 101020836 | 1.002812322 | 3 | amp |
| TCGA-04-1362 | 13 | 101077875 | 101721232 | 1.569167314 | 4 | amp |
| TCGA-04-1362 | 13 | 101725892 | 103330681 | 0.942025865 | 3 | amp |
| TCGA-04-1362 | 13 | 103338337 | 103460156 | 1.527812863 | 4 | amp |
| TCGA-04-1362 | 13 | 103468720 | 109707960 | 1.188642556 | 3 | amp |
| TCGA-04-1362 | 13 | 109717874 | 114839373 | 2.154276278 | 5 | amp |
| TCGA-04-1362 | 13 | 115002086 | 115011559 | 1.662216663 | 4 | amp |
| TCGA-04-1362 | 13 | 115012382 | 115037968 | 0.787486931 | 3 | amp |
| TCGA-04-1362 | 13 | 115048304 | 115091796 | 2.010369612 | 4 | amp |
| TCGA-04-1362 | 14 | 19377543 | 20647490 | 1.143797916 | 3 | amp |
| TCGA-04-1362 | 14 | 20665466 | 20819303 | 1.572571505 | 4 | amp |
| TCGA-04-1362 | 14 | 20820364 | 20943435 | 2.532559021 | 5 | amp |
| TCGA-04-1362 | 14 | 20944501 | 22265673 | 1.558945349 | 4 | amp |
| TCGA-04-1362 | 14 | 22265730 | 22447392 | 0.924273222 | 3 | amp |
| TCGA-04-1362 | 14 | 22458697 | 23249317 | 1.771923793 | 4 | amp |
| TCGA-04-1362 | 14 | 23282057 | 23938990 | 2.704210359 | 5 | amp |
| TCGA-04-1362 | 14 | 23939190 | 23947285 | 1.150987159 | 3 | amp |
| TCGA-04-1362 | 14 | 24027826 | 24912027 | 2.963971049 | 5 | amp |
| TCGA-04-1362 | 14 | 24974643 | 25102314 | 1.493989105 | 4 | amp |
| TCGA-04-1362 | 14 | 25281836 | 35596861 | 0.99550389 | 3 | amp |
| TCGA-04-1362 | 14 | 64655226 | 65568336 | 1.621118445 | 4 | amp |
| TCGA-04-1362 | 14 | 66028262 | 67770337 | 0.960802703 | 3 | amp |
| TCGA-04-1362 | 14 | 67779215 | 68220513 | 1.53766555 | 4 | amp |
| TCGA-04-1362 | 14 | 68220715 | 72818912 | 1.095465179 | 3 | amp |
| TCGA-04-1362 | 14 | 72921200 | 74428275 | 1.470957379 | 4 | amp |
| TCGA-04-1362 | 14 | 74428372 | 74541748 | 0.943467711 | 3 | amp |
| TCGA-04-1362 | 14 | 74551620 | 75131643 | 1.45084759 | 4 | amp |
| TCGA-04-1362 | 14 | 75133990 | 76668233 | 1.007727975 | 3 | amp |
| TCGA-04-1362 | 14 | 76905624 | 77808342 | 1.500985221 | 4 | amp |
| TCGA-04-1362 | 14 | 77809493 | 99932150 | 1.026174034 | 3 | amp |
| TCGA-04-1362 | 14 | 99958992 | 101393773 | 1.602168777 | 4 | amp |
| TCGA-04-1362 | 14 | 101396239 | 101459669 | 0.529124696 | 1 | loss |
| TCGA-04-1362 | 14 | 101492021 | 106926521 | 1.431993733 | 4 | amp |
| TCGA-04-1362 | 14 | 106926561 | 107283263 | 1.117012781 | 3 | amp |
| TCGA-04-1362 | 15 | 20169886 | 22414056 | 1.628603503 | 4 | amp |
| TCGA-04-1362 | 15 | 22466023 | 22739835 | 2.223509497 | 5 | amp |
| TCGA-04-1362 | 15 | 22741176 | 22872508 | 1.685035693 | 4 | amp |
| TCGA-04-1362 | 15 | 22873164 | 23003077 | 2.427968949 | 5 | amp |
| TCGA-04-1362 | 15 | 23006177 | 23145934 | 1.267457385 | 4 | amp |
| TCGA-04-1362 | 15 | 23155342 | 24924489 | 2.157356894 | 5 | amp |
| TCGA-04-1362 | 15 | 25154454 | 25322303 | 1.578862325 | 4 | amp |
| TCGA-04-1362 | 15 | 25324190 | 25351768 | 0.972935008 | 3 | amp |
| TCGA-04-1362 | 15 | 25415850 | 25523582 | 3.375740978 | 5 | amp |
| TCGA-04-1362 | 15 | 25584345 | 25653840 | 1.29285857 | 4 | amp |
| TCGA-04-1362 | 15 | 25654184 | 25981305 | 2.27381838 | 5 | amp |
| TCGA-04-1362 | 15 | 26792930 | 29037174 | 1.491017911 | 4 | amp |
| TCGA-04-1362 | 15 | 29046577 | 40512982 | 1.105035807 | 3 | amp |

| | | | | | | |
|---|---|---|---|---|---|---|
| TCGA-04-1362 | 15 | 40556938 | 41280212 | 1.634001522 | 4 | amp |
| TCGA-04-1362 | 15 | 41455382 | 41870518 | 1.536208669 | 4 | amp |
| TCGA-04-1362 | 15 | 41961084 | 42103171 | 1.05368939 | 3 | amp |
| TCGA-04-1362 | 15 | 42103314 | 42159847 | 2.200501528 | 5 | amp |
| TCGA-04-1362 | 15 | 42201828 | 42462112 | 1.568653723 | 4 | amp |
| TCGA-04-1362 | 15 | 42465865 | 43452995 | 1.032097608 | 3 | amp |
| TCGA-04-1362 | 15 | 43461772 | 43668881 | 1.619013905 | 4 | amp |
| TCGA-04-1362 | 15 | 43669178 | 43724915 | 0.924498791 | 3 | amp |
| TCGA-04-1362 | 15 | 43730507 | 44128476 | 1.562378606 | 4 | amp |
| TCGA-04-1362 | 15 | 44131714 | 45388261 | 1.144082978 | 3 | amp |
| TCGA-04-1362 | 15 | 45389362 | 45545733 | 1.902626567 | 4 | amp |
| TCGA-04-1362 | 15 | 45554168 | 45777557 | 1.197960758 | 3 | amp |
| TCGA-04-1362 | 15 | 52179696 | 52652281 | 1.147513408 | 3 | amp |
| TCGA-04-1362 | 15 | 62336372 | 63667926 | 1.243037126 | 3 | amp |
| TCGA-04-1362 | 15 | 64056282 | 64962648 | 1.194262395 | 3 | amp |
| TCGA-04-1362 | 15 | 64966084 | 65234772 | 1.583844377 | 4 | amp |
| TCGA-04-1362 | 15 | 65235638 | 66845638 | 1.140291228 | 3 | amp |
| TCGA-04-1362 | 15 | 66850020 | 67555566 | 1.727006326 | 4 | amp |
| TCGA-04-1362 | 15 | 67571671 | 68469021 | 0.891647806 | 3 | amp |
| TCGA-04-1362 | 15 | 68473500 | 69349090 | 2.204569619 | 5 | amp |
| TCGA-04-1362 | 15 | 69548138 | 69672405 | 1.049447139 | 3 | amp |
| TCGA-04-1362 | 15 | 69676998 | 70368525 | 2.16151064 | 5 | amp |
| TCGA-04-1362 | 15 | 70949370 | 70994294 | 0.632501067 | 1 | loss |
| TCGA-04-1362 | 15 | 71124352 | 72109956 | 1.496119563 | 4 | amp |
| TCGA-04-1362 | 15 | 72118912 | 72432643 | 0.851986573 | 3 | amp |
| TCGA-04-1362 | 15 | 72454261 | 72501276 | 2.266741309 | 5 | amp |
| TCGA-04-1362 | 15 | 72501986 | 72691301 | 1.725853057 | 4 | amp |
| TCGA-04-1362 | 15 | 72698897 | 72875650 | 0.971230658 | 3 | amp |
| TCGA-04-1362 | 15 | 72879545 | 72958739 | 2.405196208 | 5 | amp |
| TCGA-04-1362 | 15 | 72987483 | 73067445 | 1.490230809 | 4 | amp |
| TCGA-04-1362 | 15 | 73408859 | 73567108 | 0.845370398 | 3 | amp |
| TCGA-04-1362 | 15 | 73570445 | 73889716 | 1.469710764 | 4 | amp |
| TCGA-04-1362 | 15 | 74001919 | 75664554 | 2.597951912 | 5 | amp |
| TCGA-04-1362 | 15 | 75667989 | 76077945 | 1.809998348 | 4 | amp |
| TCGA-04-1362 | 15 | 76146717 | 76303626 | 0.978895849 | 3 | amp |
| TCGA-04-1362 | 15 | 76426533 | 76508978 | 2.243257775 | 5 | amp |
| TCGA-04-1362 | 15 | 76518169 | 76673957 | 1.749376975 | 4 | amp |
| TCGA-04-1362 | 15 | 76696822 | 77771708 | 1.110803557 | 3 | amp |
| TCGA-04-1362 | 15 | 77906473 | 78732239 | 2.165484984 | 5 | amp |
| TCGA-04-1362 | 15 | 78755226 | 78894640 | 1.588293182 | 4 | amp |
| TCGA-04-1362 | 15 | 78910890 | 79172947 | 2.412160386 | 5 | amp |
| TCGA-04-1362 | 15 | 79177246 | 79189426 | 1.03473674 | 3 | amp |
| TCGA-04-1362 | 15 | 79217638 | 79760747 | 2.318205199 | 5 | amp |
| TCGA-04-1362 | 15 | 80137547 | 80430015 | 1.133956208 | 3 | amp |
| TCGA-04-1362 | 15 | 80445376 | 81654661 | 1.902061904 | 4 | amp |
| TCGA-04-1362 | 15 | 81660571 | 82575411 | 0.955170786 | 3 | amp |
| TCGA-04-1362 | 15 | 82635074 | 83784705 | 1.989245986 | 4 | amp |
| TCGA-04-1362 | 15 | 83788321 | 84795403 | 0.964258236 | 3 | amp |
| TCGA-04-1362 | 15 | 84859539 | 85488453 | 2.080629567 | 4 | amp |
| TCGA-04-1362 | 15 | 85607568 | 88524701 | 1.191949977 | 3 | amp |

| | | | | | | |
|---|---|---|---|---|---|---|
| TCGA-04-1362 | 15 | 88576061 | 89762292 | 2.248624819 | 5 | amp |
| TCGA-04-1362 | 15 | 89790830 | 89856258 | 1.306671083 | 4 | amp |
| TCGA-04-1362 | 15 | 89857830 | 90039803 | 2.1971917 | 5 | amp |
| TCGA-04-1362 | 15 | 90125876 | 90281489 | 1.412218336 | 4 | amp |
| TCGA-04-1362 | 15 | 90286482 | 90934115 | 2.408359205 | 5 | amp |
| TCGA-04-1362 | 15 | 90969299 | 91040577 | 1.076293825 | 3 | amp |
| TCGA-04-1362 | 15 | 91043193 | 91185387 | 2.26110407 | 5 | amp |
| TCGA-04-1362 | 15 | 91290611 | 91352547 | 1.165901199 | 3 | amp |
| TCGA-04-1362 | 15 | 91354385 | 91545465 | 2.196164879 | 5 | amp |
| TCGA-04-1362 | 15 | 91546247 | 93489132 | 1.749993132 | 4 | amp |
| TCGA-04-1362 | 15 | 93489236 | 95022288 | 1.064022534 | 3 | amp |
| TCGA-04-1362 | 15 | 96869499 | 99696547 | 2.151853021 | 5 | amp |
| TCGA-04-1362 | 15 | 99701888 | 100943002 | 1.871840467 | 4 | amp |
| TCGA-04-1362 | 15 | 100996054 | 101121087 | 0.934890919 | 3 | amp |
| TCGA-04-1362 | 15 | 101152406 | 102198083 | 2.239376761 | 5 | amp |
| TCGA-04-1362 | 15 | 102201906 | 102500889 | 1.518768312 | 4 | amp |
| TCGA-04-1362 | 16 | 66894 | 5145543 | 1.759329169 | 4 | amp |
| TCGA-04-1362 | 16 | 8719468 | 9024261 | 1.501504186 | 4 | amp |
| TCGA-04-1362 | 16 | 9196798 | 10576139 | 0.782704822 | 3 | amp |
| TCGA-04-1362 | 16 | 10626705 | 11846726 | 1.418487091 | 4 | amp |
| TCGA-04-1362 | 16 | 11850041 | 15116645 | 1.038527479 | 3 | amp |
| TCGA-04-1362 | 16 | 15120471 | 15702381 | 1.334604855 | 4 | amp |
| TCGA-04-1362 | 16 | 15703360 | 15771851 | 0.779750481 | 3 | amp |
| TCGA-04-1362 | 16 | 15781149 | 16330862 | 1.451367603 | 4 | amp |
| TCGA-04-1362 | 16 | 16333670 | 18839541 | 1.183971777 | 3 | amp |
| TCGA-04-1362 | 16 | 18903487 | 20638694 | 1.093805661 | 3 | amp |
| TCGA-04-1362 | 16 | 21286823 | 21964820 | 1.104244788 | 3 | amp |
| TCGA-04-1362 | 16 | 22122201 | 22143097 | 1.114082725 | 3 | amp |
| TCGA-04-1362 | 16 | 22144198 | 22345052 | 1.349337515 | 4 | amp |
| TCGA-04-1362 | 16 | 22358234 | 22525242 | 0.997693916 | 3 | amp |
| TCGA-04-1362 | 16 | 22535084 | 23085241 | 1.48391925 | 4 | amp |
| TCGA-04-1362 | 16 | 23197540 | 23721588 | 1.497746407 | 4 | amp |
| TCGA-04-1362 | 16 | 23721763 | 26147610 | 0.972721759 | 3 | amp |
| TCGA-04-1362 | 16 | 27078094 | 28842143 | 1.518920813 | 4 | amp |
| TCGA-04-1362 | 16 | 28842247 | 28913725 | 2.214277128 | 5 | amp |
| TCGA-04-1362 | 16 | 28914045 | 29405453 | 1.478710676 | 4 | amp |
| TCGA-04-1362 | 16 | 29449437 | 30256894 | 1.870040605 | 4 | amp |
| TCGA-04-1362 | 16 | 30278932 | 30316573 | 0.96769828 | 3 | amp |
| TCGA-04-1362 | 16 | 30317072 | 32177057 | 1.922081581 | 4 | amp |
| TCGA-04-1362 | 16 | 32177931 | 32772334 | 0.916197111 | 3 | amp |
| TCGA-04-1362 | 16 | 32773208 | 34339128 | 1.310581136 | 4 | amp |
| TCGA-04-1362 | 16 | 50809020 | 54145870 | 0.47418041 | 1 | loss |
| TCGA-04-1362 | 16 | 56623724 | 56782368 | 1.330777517 | 4 | amp |
| TCGA-04-1362 | 16 | 56792388 | 56878525 | 0.485729325 | 1 | loss |
| TCGA-04-1362 | 16 | 56899078 | 57181576 | 1.300118031 | 4 | amp |
| TCGA-04-1362 | 16 | 57197854 | 57265274 | 0.772801016 | 3 | amp |
| TCGA-04-1362 | 16 | 57269027 | 58074641 | 1.391381165 | 4 | amp |
| TCGA-04-1362 | 16 | 58147871 | 58150875 | 1.069555712 | 3 | amp |
| TCGA-04-1362 | 16 | 66413195 | 66855467 | 1.026659334 | 3 | amp |
| TCGA-04-1362 | 16 | 66857109 | 67035407 | 1.37206948 | 4 | amp |

| | | | | | | |
|---|---|---|---|---|---|---|
| TCGA-04-1362 | 16 | 67036964 | 67168373 | 0.899601598 | 3 | amp |
| TCGA-04-1362 | 16 | 67173833 | 67333490 | 1.37040644 | 4 | amp |
| TCGA-04-1362 | 16 | 67335609 | 67663488 | 1.060432688 | 3 | amp |
| TCGA-04-1362 | 16 | 67670553 | 68057133 | 1.36713274 | 4 | amp |
| TCGA-04-1362 | 16 | 68071869 | 68732318 | 1.045512241 | 3 | amp |
| TCGA-04-1362 | 16 | 69760248 | 70563118 | 1.032529569 | 3 | amp |
| TCGA-04-1362 | 16 | 72130037 | 74496102 | 0.994922782 | 3 | amp |
| TCGA-04-1362 | 16 | 81116459 | 81960794 | 1.033358909 | 3 | amp |
| TCGA-04-1362 | 16 | 83828571 | 84432219 | 1.032689691 | 3 | amp |
| TCGA-04-1362 | 16 | 84438671 | 90244214 | 1.370059985 | 4 | amp |
| TCGA-04-1362 | 17 | 69410 | 707103 | 1.447784878 | 4 | amp |
| TCGA-04-1362 | 17 | 725556 | 1444956 | 2.18829305 | 5 | amp |
| TCGA-04-1362 | 17 | 1446257 | 5045804 | 1.676691927 | 4 | amp |
| TCGA-04-1362 | 17 | 5047951 | 5314125 | 1.008672067 | 3 | amp |
| TCGA-04-1362 | 17 | 5317271 | 10243570 | 1.836748394 | 4 | amp |
| TCGA-04-1362 | 17 | 10243621 | 10309772 | 1.154021023 | 3 | amp |
| TCGA-04-1362 | 17 | 10531892 | 10596299 | 1.612436346 | 4 | amp |
| TCGA-04-1362 | 17 | 10599042 | 12847005 | 0.934858629 | 3 | amp |
| TCGA-04-1362 | 17 | 12847329 | 15907605 | 1.309470307 | 4 | amp |
| TCGA-04-1362 | 17 | 15909774 | 16090084 | 0.882125014 | 3 | amp |
| TCGA-04-1362 | 17 | 16097769 | 27620921 | 1.527107416 | 4 | amp |
| TCGA-04-1362 | 17 | 27778512 | 29326244 | 1.006874755 | 3 | amp |
| TCGA-04-1362 | 17 | 30321541 | 33328468 | 1.020585348 | 3 | amp |
| TCGA-04-1362 | 17 | 33328875 | 35310630 | 1.422524008 | 4 | amp |
| TCGA-04-1362 | 17 | 35311018 | 35470231 | 1.11911973 | 3 | amp |
| TCGA-04-1362 | 17 | 36047324 | 36552280 | 1.67752632 | 4 | amp |
| TCGA-04-1362 | 17 | 36704748 | 36912249 | 2.403928054 | 5 | amp |
| TCGA-04-1362 | 17 | 36916652 | 36977359 | 1.669425347 | 4 | amp |
| TCGA-04-1362 | 17 | 36991370 | 37371486 | 2.529667352 | 5 | amp |
| TCGA-04-1362 | 17 | 37417676 | 37681152 | 1.556489666 | 4 | amp |
| TCGA-04-1362 | 17 | 37682072 | 37902468 | 2.978756502 | 5 | amp |
| TCGA-04-1362 | 17 | 37922034 | 38073571 | 1.317118553 | 4 | amp |
| TCGA-04-1362 | 17 | 38078720 | 38548401 | 2.407460103 | 5 | amp |
| TCGA-04-1362 | 17 | 38548411 | 38569236 | 1.20143123 | 3 | amp |
| TCGA-04-1362 | 17 | 38569435 | 38715229 | 2.649601394 | 5 | amp |
| TCGA-04-1362 | 17 | 38721566 | 39156136 | 1.441007709 | 4 | amp |
| TCGA-04-1362 | 17 | 39164997 | 40476916 | 2.589685128 | 5 | amp |
| TCGA-04-1362 | 17 | 40476939 | 40659739 | 1.501014243 | 4 | amp |
| TCGA-04-1362 | 17 | 40660538 | 40861999 | 2.47198114 | 5 | amp |
| TCGA-04-1362 | 17 | 40864254 | 40876502 | 1.302075959 | 4 | amp |
| TCGA-04-1362 | 17 | 40879566 | 41197876 | 2.218177464 | 5 | amp |
| TCGA-04-1362 | 17 | 41199599 | 41258571 | 1.164080262 | 3 | amp |
| TCGA-04-1362 | 17 | 41267709 | 41569665 | 2.374859397 | 5 | amp |
| TCGA-04-1362 | 17 | 41570120 | 41599624 | 1.421678368 | 4 | amp |
| TCGA-04-1362 | 17 | 41600955 | 42932388 | 2.441833603 | 5 | amp |
| TCGA-04-1362 | 17 | 42934423 | 42964070 | 1.550474573 | 4 | amp |
| TCGA-04-1362 | 17 | 42971776 | 43580944 | 2.564812615 | 5 | amp |
| TCGA-04-1362 | 17 | 43587590 | 45774257 | 1.530675384 | 4 | amp |
| TCGA-04-1362 | 17 | 45776972 | 46154416 | 2.801142855 | 5 | amp |
| TCGA-04-1362 | 17 | 46189366 | 46474154 | 1.193012866 | 3 | amp |

| | | | | | | |
|---|---|---|---|---|---|---|
| TCGA-04-1362 | 17 | 46606892 | 46871641 | 2.64522653 | 5 | amp |
| TCGA-04-1362 | 17 | 46919039 | 46940403 | 1.293288886 | 4 | amp |
| TCGA-04-1362 | 17 | 46970738 | 47304090 | 2.202693374 | 5 | amp |
| TCGA-04-1362 | 17 | 47375722 | 47700193 | 1.384412803 | 4 | amp |
| TCGA-04-1362 | 17 | 47778785 | 47869441 | 2.382228066 | 5 | amp |
| TCGA-04-1362 | 17 | 47874079 | 47904873 | 1.061318468 | 3 | amp |
| TCGA-04-1362 | 17 | 47915959 | 48777219 | 2.846707965 | 5 | amp |
| TCGA-04-1362 | 17 | 48777882 | 54559932 | 1.0914698 | 3 | amp |
| TCGA-04-1362 | 17 | 54892189 | 54926255 | 1.907561538 | 4 | amp |
| TCGA-04-1362 | 17 | 54926507 | 56606565 | 2.228752608 | 5 | amp |
| TCGA-04-1362 | 17 | 56619101 | 57684583 | 1.457047515 | 4 | amp |
| TCGA-04-1362 | 17 | 57721590 | 57851300 | 0.76948053 | 3 | amp |
| TCGA-04-1362 | 17 | 57886136 | 58092214 | 1.426833064 | 4 | amp |
| TCGA-04-1362 | 17 | 58093174 | 58235845 | 2.230964513 | 5 | amp |
| TCGA-04-1362 | 17 | 58256582 | 59093306 | 1.00941267 | 3 | amp |
| TCGA-04-1362 | 17 | 59104188 | 59560928 | 1.817704223 | 4 | amp |
| TCGA-04-1362 | 17 | 59667857 | 59974946 | 0.91728964 | 3 | amp |
| TCGA-04-1362 | 17 | 59975004 | 60140664 | 1.461882914 | 4 | amp |
| TCGA-04-1362 | 17 | 60216946 | 60351500 | 2.280841786 | 5 | amp |
| TCGA-04-1362 | 17 | 60360048 | 61901648 | 1.432733498 | 4 | amp |
| TCGA-04-1362 | 17 | 61901670 | 62149521 | 2.393927228 | 5 | amp |
| TCGA-04-1362 | 17 | 62156997 | 63632150 | 1.547780451 | 4 | amp |
| TCGA-04-1362 | 17 | 63633213 | 64179424 | 0.842236997 | 3 | amp |
| TCGA-04-1362 | 17 | 64208218 | 64685176 | 1.290693358 | 4 | amp |
| TCGA-04-1362 | 17 | 64728724 | 65074714 | 2.253856084 | 5 | amp |
| TCGA-04-1362 | 17 | 65082890 | 65214935 | 0.780309531 | 3 | amp |
| TCGA-04-1362 | 17 | 65336943 | 66883681 | 1.543374113 | 4 | amp |
| TCGA-04-1362 | 17 | 66887593 | 68129516 | 0.882191603 | 3 | amp |
| TCGA-04-1362 | 17 | 68171162 | 70845977 | 1.691735268 | 4 | amp |
| TCGA-04-1362 | 17 | 70943802 | 81188237 | 2.581990274 | 5 | amp |
| TCGA-04-1362 | 18 | 47273 | 163500 | 1.338557247 | 4 | amp |
| TCGA-04-1362 | 18 | 166742 | 166862 | 1.103629439 | 3 | amp |
| TCGA-04-1362 | 18 | 218867 | 226910 | 1.103629439 | 3 | amp |
| TCGA-04-1362 | 18 | 246269 | 724647 | 1.522389381 | 4 | amp |
| TCGA-04-1362 | 18 | 732779 | 2585216 | 1.02805518 | 3 | amp |
| TCGA-04-1362 | 18 | 6959295 | 7232069 | 0.98221961 | 3 | amp |
| TCGA-04-1362 | 18 | 8252431 | 8252551 | 0.925590992 | 3 | amp |
| TCGA-04-1362 | 18 | 8253198 | 9119649 | 1.296097406 | 4 | amp |
| TCGA-04-1362 | 18 | 9122474 | 10697831 | 1.079715219 | 3 | amp |
| TCGA-04-1362 | 18 | 11609562 | 12464971 | 1.505155669 | 4 | amp |
| TCGA-04-1362 | 18 | 12479633 | 14248300 | 1.079912118 | 3 | amp |
| TCGA-04-1362 | 18 | 18595355 | 22025453 | 1.06417604 | 3 | amp |
| TCGA-04-1362 | 18 | 24035665 | 25573652 | 1.045040369 | 3 | amp |
| TCGA-04-1362 | 18 | 28713793 | 28742585 | 1.133293764 | 3 | amp |
| TCGA-04-1362 | 18 | 28983367 | 29890340 | 1.00656845 | 3 | amp |
| TCGA-04-1362 | 18 | 30903405 | 33058339 | 0.965489777 | 3 | amp |
| TCGA-04-1362 | 18 | 33689481 | 33716394 | 1.122176764 | 3 | amp |
| TCGA-04-1362 | 18 | 33751006 | 34359511 | 1.124208233 | 3 | amp |
| TCGA-04-1362 | 18 | 42618414 | 44580877 | 1.093112685 | 3 | amp |
| TCGA-04-1362 | 18 | 44639319 | 46190929 | 1.082700481 | 3 | amp |

| | | | | | | |
|---|---|---|---|---|---|---|
| TCGA-04-1362 | 18 | 46197017 | 46468947 | 2.256087419 | 5 | amp |
| TCGA-04-1362 | 18 | 46570349 | 46690165 | 1.162698203 | 3 | amp |
| TCGA-04-1362 | 18 | 47008657 | 47769497 | 1.076649217 | 3 | amp |
| TCGA-04-1362 | 18 | 47777116 | 48190901 | 1.705775064 | 4 | amp |
| TCGA-04-1362 | 18 | 48241400 | 48335780 | 0.998712111 | 3 | amp |
| TCGA-04-1362 | 18 | 54483258 | 56182328 | 1.12097768 | 3 | amp |
| TCGA-04-1362 | 18 | 56816739 | 59739991 | 1.021053126 | 3 | amp |
| TCGA-04-1362 | 18 | 60015351 | 61030116 | 1.025211637 | 3 | amp |
| TCGA-04-1362 | 18 | 61597229 | 63492093 | 1.096205835 | 3 | amp |
| TCGA-04-1362 | 18 | 67863660 | 70526364 | 0.964153187 | 3 | amp |
| TCGA-04-1362 | 18 | 70531961 | 72234687 | 1.540214474 | 4 | amp |
| TCGA-04-1362 | 18 | 72238372 | 74563932 | 1.192657014 | 3 | amp |
| TCGA-04-1362 | 18 | 74580579 | 76757346 | 1.548076861 | 4 | amp |
| TCGA-04-1362 | 18 | 76856442 | 76903890 | 0.61156405 | 1 | loss |
| TCGA-04-1362 | 18 | 76914469 | 77960823 | 1.550524396 | 4 | amp |
| TCGA-04-1362 | 19 | 71882 | 8977724 | 1.9287868 | 4 | amp |
| TCGA-04-1362 | 19 | 8979174 | 9801480 | 0.899506452 | 3 | amp |
| TCGA-04-1362 | 19 | 9868135 | 10292824 | 2.054440456 | 4 | amp |
| TCGA-04-1362 | 19 | 10334480 | 11725524 | 2.35168934 | 5 | amp |
| TCGA-04-1362 | 19 | 11727476 | 12061099 | 1.544119907 | 4 | amp |
| TCGA-04-1362 | 19 | 12087791 | 12385877 | 2.261782261 | 5 | amp |
| TCGA-04-1362 | 19 | 12386713 | 12694426 | 1.581008835 | 4 | amp |
| TCGA-04-1362 | 19 | 12721315 | 14694053 | 2.878037185 | 5 | amp |
| TCGA-04-1362 | 19 | 14694147 | 14785629 | 1.140371682 | 3 | amp |
| TCGA-04-1362 | 19 | 14804214 | 14826297 | 2.598784389 | 5 | amp |
| TCGA-04-1362 | 19 | 14826836 | 14857149 | 1.26351351 | 4 | amp |
| TCGA-04-1362 | 19 | 14857680 | 15132734 | 2.201971238 | 5 | amp |
| TCGA-04-1362 | 19 | 15163015 | 15222642 | 1.193184886 | 3 | amp |
| TCGA-04-1362 | 19 | 15224302 | 19770624 | 2.66134565 | 5 | amp |
| TCGA-04-1362 | 19 | 19788615 | 21205700 | 1.392087198 | 4 | amp |
| TCGA-04-1362 | 19 | 21216204 | 29699119 | 1.075848341 | 3 | amp |
| TCGA-04-1362 | 19 | 30099499 | 30314742 | 2.49264022 | 5 | amp |
| TCGA-04-1362 | 19 | 30934384 | 31770676 | 2.808058525 | 5 | amp |
| TCGA-04-1362 | 19 | 32843719 | 32959767 | 1.067680683 | 3 | amp |
| TCGA-04-1362 | 19 | 32968373 | 34890993 | 2.320174774 | 5 | amp |
| TCGA-04-1362 | 19 | 34900060 | 35451954 | 1.677527056 | 4 | amp |
| TCGA-04-1362 | 19 | 35500743 | 36900746 | 2.645383614 | 5 | amp |
| TCGA-04-1362 | 19 | 36939850 | 38231143 | 1.377569486 | 4 | amp |
| TCGA-04-1362 | 19 | 38283374 | 40023499 | 2.726301034 | 5 | amp |
| TCGA-04-1362 | 19 | 40029973 | 40228644 | 0.929299934 | 3 | amp |
| TCGA-04-1362 | 19 | 40316782 | 40480545 | 2.57433648 | 5 | amp |
| TCGA-04-1362 | 19 | 40485687 | 40589094 | 1.318568023 | 4 | amp |
| TCGA-04-1362 | 19 | 40710965 | 41386578 | 2.596942193 | 5 | amp |
| TCGA-04-1362 | 19 | 41387454 | 41595035 | 1.327758702 | 4 | amp |
| TCGA-04-1362 | 19 | 41596268 | 41938344 | 2.410985915 | 5 | amp |
| TCGA-04-1362 | 19 | 41939137 | 44339760 | 1.637755327 | 4 | amp |
| TCGA-04-1362 | 19 | 44341147 | 44947074 | 0.914253854 | 3 | amp |
| TCGA-04-1362 | 19 | 44968395 | 53905500 | 1.81978286 | 4 | amp |
| TCGA-04-1362 | 19 | 53910945 | 54264491 | 1.030864105 | 3 | amp |
| TCGA-04-1362 | 19 | 54265580 | 56297293 | 1.749694921 | 4 | amp |

| | | | | | | |
|---|---|---|---|---|---|---|
| TCGA-04-1362 | 19 | 56300137 | 56511182 | 0.89607419 | 3 | amp |
| TCGA-04-1362 | 19 | 56515031 | 59110878 | 1.56723941 | 4 | amp |
| TCGA-04-1362 | 2 | 286256 | 286376 | 0.819314335 | 3 | amp |
| TCGA-04-1362 | 2 | 669623 | 8877183 | 1.579698592 | 4 | amp |
| TCGA-04-1362 | 2 | 8887212 | 11706813 | 1.036526653 | 3 | amp |
| TCGA-04-1362 | 2 | 11716452 | 11932145 | 1.950635378 | 4 | amp |
| TCGA-04-1362 | 2 | 11935513 | 15307489 | 1.199202694 | 3 | amp |
| TCGA-04-1362 | 2 | 24111184 | 26663435 | 1.161415445 | 3 | amp |
| TCGA-04-1362 | 2 | 26667006 | 27015113 | 2.177371436 | 5 | amp |
| TCGA-04-1362 | 2 | 27015602 | 27448803 | 1.81429205 | 4 | amp |
| TCGA-04-1362 | 2 | 27448942 | 27501241 | 2.273047384 | 5 | amp |
| TCGA-04-1362 | 2 | 27502998 | 27681101 | 1.510882316 | 4 | amp |
| TCGA-04-1362 | 2 | 27681630 | 32409434 | 1.109069318 | 3 | amp |
| TCGA-04-1362 | 2 | 45832480 | 46372420 | 1.157798346 | 3 | amp |
| TCGA-04-1362 | 2 | 46378153 | 47249179 | 1.545639261 | 4 | amp |
| TCGA-04-1362 | 2 | 47250485 | 48023237 | 1.061927724 | 3 | amp |
| TCGA-04-1362 | 2 | 70121043 | 70131472 | 1.013571751 | 3 | amp |
| TCGA-04-1362 | 2 | 70143212 | 71743400 | 1.689152742 | 4 | amp |
| TCGA-04-1362 | 2 | 71744053 | 72371349 | 2.784321032 | 5 | amp |
| TCGA-04-1362 | 2 | 72406406 | 73053042 | 1.22673758 | 3 | amp |
| TCGA-04-1362 | 2 | 73115407 | 73498056 | 2.375824167 | 5 | amp |
| TCGA-04-1362 | 2 | 73635692 | 74448670 | 1.632576949 | 4 | amp |
| TCGA-04-1362 | 2 | 74449946 | 74789566 | 2.970301722 | 5 | amp |
| TCGA-04-1362 | 2 | 74802515 | 74842303 | 1.013397772 | 3 | amp |
| TCGA-04-1362 | 2 | 74867102 | 75187783 | 2.201781681 | 5 | amp |
| TCGA-04-1362 | 2 | 75196482 | 75907499 | 1.298260477 | 4 | amp |
| TCGA-04-1362 | 2 | 75914907 | 84780219 | 1.024416622 | 3 | amp |
| TCGA-04-1362 | 2 | 84784838 | 85510719 | 1.557027394 | 4 | amp |
| TCGA-04-1362 | 2 | 85529522 | 85590314 | 2.620217423 | 5 | amp |
| TCGA-04-1362 | 2 | 85595752 | 85788142 | 1.613996355 | 4 | amp |
| TCGA-04-1362 | 2 | 85808648 | 85991329 | 2.194121207 | 5 | amp |
| TCGA-04-1362 | 2 | 86067210 | 86090666 | 1.400950987 | 4 | amp |
| TCGA-04-1362 | 2 | 86094673 | 86305163 | 2.612725314 | 5 | amp |
| TCGA-04-1362 | 2 | 86305201 | 86509388 | 1.451898295 | 4 | amp |
| TCGA-04-1362 | 2 | 86676887 | 90260280 | 1.104921307 | 3 | amp |
| TCGA-04-1362 | 2 | 90273672 | 95847890 | 1.399191363 | 4 | amp |
| TCGA-04-1362 | 2 | 95947628 | 96116883 | 3.129516844 | 5 | amp |
| TCGA-04-1362 | 2 | 96142852 | 96459977 | 1.404196243 | 4 | amp |
| TCGA-04-1362 | 2 | 96464663 | 96595060 | 0.963002604 | 3 | amp |
| TCGA-04-1362 | 2 | 96652462 | 97279410 | 1.853799193 | 4 | amp |
| TCGA-04-1362 | 2 | 97285068 | 97695181 | 2.239674763 | 5 | amp |
| TCGA-04-1362 | 2 | 97696237 | 97784290 | 1.878684704 | 4 | amp |
| TCGA-04-1362 | 2 | 97845429 | 98195461 | 1.053890661 | 3 | amp |
| TCGA-04-1362 | 2 | 98201370 | 98264574 | 1.417864555 | 4 | amp |
| TCGA-04-1362 | 2 | 98273851 | 98383122 | 3.276003584 | 5 | amp |
| TCGA-04-1362 | 2 | 98388703 | 98887365 | 1.834513795 | 4 | amp |
| TCGA-04-1362 | 2 | 98906939 | 99272005 | 2.592117374 | 5 | amp |
| TCGA-04-1362 | 2 | 99272781 | 100454022 | 1.678434364 | 4 | amp |
| TCGA-04-1362 | 2 | 100623016 | 101097035 | 2.244022658 | 5 | amp |
| TCGA-04-1362 | 2 | 101097548 | 101549477 | 1.718907091 | 4 | amp |

| | | | | | | |
|---|---|---|---|---|---|---|
| TCGA-04-1362 | 2 | 101554199 | 101971831 | 2.732743572 | 5 | amp |
| TCGA-04-1362 | 2 | 101999959 | 103348884 | 1.561823258 | 4 | amp |
| TCGA-04-1362 | 2 | 103378742 | 106994560 | 1.149856294 | 3 | amp |
| TCGA-04-1362 | 2 | 111423765 | 135012274 | 1.049225223 | 3 | amp |
| TCGA-04-1362 | 2 | 152520037 | 157186746 | 1.079556127 | 3 | amp |
| TCGA-04-1362 | 2 | 159536884 | 159537244 | 0.860291249 | 3 | amp |
| TCGA-04-1362 | 2 | 159660777 | 160139621 | 1.824439665 | 4 | amp |
| TCGA-04-1362 | 2 | 160176732 | 162092060 | 1.120480307 | 3 | amp |
| TCGA-04-1362 | 2 | 162175300 | 163130499 | 1.368562202 | 4 | amp |
| TCGA-04-1362 | 2 | 163133174 | 169551574 | 1.138422341 | 3 | amp |
| TCGA-04-1362 | 2 | 169571454 | 171867983 | 1.40055837 | 4 | amp |
| TCGA-04-1362 | 2 | 171871349 | 172604402 | 1.080055024 | 3 | amp |
| TCGA-04-1362 | 2 | 172641764 | 175246534 | 1.603037733 | 4 | amp |
| TCGA-04-1362 | 2 | 175252399 | 175816970 | 2.304395993 | 5 | amp |
| TCGA-04-1362 | 2 | 175939329 | 176857172 | 1.378070594 | 4 | amp |
| TCGA-04-1362 | 2 | 176857870 | 177135202 | 3.093576064 | 5 | amp |
| TCGA-04-1362 | 2 | 177161551 | 179355583 | 1.465790687 | 4 | amp |
| TCGA-04-1362 | 2 | 179358527 | 179537455 | 1.060415846 | 3 | amp |
| TCGA-04-1362 | 2 | 179538338 | 179583740 | 1.641957519 | 4 | amp |
| TCGA-04-1362 | 2 | 179583854 | 182438665 | 1.102572402 | 3 | amp |
| TCGA-04-1362 | 2 | 182467768 | 183011881 | 1.68054104 | 4 | amp |
| TCGA-04-1362 | 2 | 183032935 | 186698004 | 1.116157433 | 3 | amp |
| TCGA-04-1362 | 2 | 187359920 | 198358987 | 1.436701367 | 4 | amp |
| TCGA-04-1362 | 2 | 198359368 | 198498761 | 2.243613904 | 5 | amp |
| TCGA-04-1362 | 2 | 198508848 | 201535436 | 1.471794215 | 4 | amp |
| TCGA-04-1362 | 2 | 201677912 | 201785880 | 2.254351162 | 5 | amp |
| TCGA-04-1362 | 2 | 201790546 | 203168114 | 1.658966208 | 4 | amp |
| TCGA-04-1362 | 2 | 203329504 | 203560804 | 2.27773155 | 5 | amp |
| TCGA-04-1362 | 2 | 203589595 | 203684642 | 1.60132673 | 4 | amp |
| TCGA-04-1362 | 2 | 203686022 | 203776238 | 2.328730688 | 5 | amp |
| TCGA-04-1362 | 2 | 203806604 | 206565514 | 1.393955136 | 4 | amp |
| TCGA-04-1362 | 2 | 206580857 | 206657043 | 3.221373638 | 5 | amp |
| TCGA-04-1362 | 2 | 206659419 | 207014655 | 1.856295446 | 4 | amp |
| TCGA-04-1362 | 2 | 207017128 | 207027395 | 2.804061426 | 5 | amp |
| TCGA-04-1362 | 2 | 207027409 | 207583053 | 1.507726327 | 4 | amp |
| TCGA-04-1362 | 2 | 207603160 | 208442423 | 0.940658421 | 3 | amp |
| TCGA-04-1362 | 2 | 208461589 | 209138820 | 1.636170884 | 4 | amp |
| TCGA-04-1362 | 2 | 209141374 | 217069977 | 1.06340592 | 3 | amp |
| TCGA-04-1362 | 2 | 217123948 | 217724807 | 1.813469184 | 4 | amp |
| TCGA-04-1362 | 2 | 218669103 | 219305634 | 2.678538775 | 5 | amp |
| TCGA-04-1362 | 2 | 219313912 | 219486377 | 1.366790023 | 4 | amp |
| TCGA-04-1362 | 2 | 219487365 | 219528162 | 2.526340294 | 5 | amp |
| TCGA-04-1362 | 2 | 219528638 | 219619164 | 1.486755788 | 4 | amp |
| TCGA-04-1362 | 2 | 219674274 | 220505356 | 2.882418088 | 5 | amp |
| TCGA-04-1362 | 2 | 220506351 | 231407665 | 1.121317579 | 3 | amp |
| TCGA-04-1362 | 2 | 231437830 | 233128153 | 1.624131793 | 4 | amp |
| TCGA-04-1362 | 2 | 233164729 | 233429068 | 2.69173945 | 5 | amp |
| TCGA-04-1362 | 2 | 233431494 | 234231681 | 1.49462739 | 4 | amp |
| TCGA-04-1362 | 2 | 234235744 | 234375910 | 2.450012721 | 5 | amp |
| TCGA-04-1362 | 2 | 234394164 | 234638685 | 1.0732501 | 3 | amp |

| | | | | | | |
|---|---|---|---|---|---|---|
| TCGA-04-1362 | 2 | 234652103 | 234978674 | 1.938564023 | 4 | amp |
| TCGA-04-1362 | 2 | 235404404 | 238688179 | 2.265284748 | 5 | amp |
| TCGA-04-1362 | 2 | 238722228 | 239141639 | 1.599917203 | 4 | amp |
| TCGA-04-1362 | 2 | 239157627 | 243160772 | 2.416060726 | 5 | amp |
| TCGA-04-1362 | 20 | 68319 | 208018 | 1.246318847 | 3 | amp |
| TCGA-04-1362 | 20 | 209865 | 464722 | 2.316106758 | 5 | amp |
| TCGA-04-1362 | 20 | 467002 | 489204 | 1.529125411 | 4 | amp |
| TCGA-04-1362 | 20 | 744064 | 1445082 | 2.369963451 | 5 | amp |
| TCGA-04-1362 | 20 | 1456776 | 1559401 | 1.619801348 | 4 | amp |
| TCGA-04-1362 | 20 | 1600462 | 2560708 | 2.397329585 | 5 | amp |
| TCGA-04-1362 | 20 | 2572944 | 2605099 | 1.345741621 | 4 | amp |
| TCGA-04-1362 | 20 | 2616520 | 3278868 | 2.790039655 | 5 | amp |
| TCGA-04-1362 | 20 | 3285048 | 3615056 | 1.730500851 | 4 | amp |
| TCGA-04-1362 | 20 | 3619468 | 3845468 | 3.748395968 | 5 | amp |
| TCGA-04-1362 | 20 | 3888568 | 3944712 | 1.561222968 | 4 | amp |
| TCGA-04-1362 | 20 | 4155656 | 5149333 | 2.889224794 | 5 | amp |
| TCGA-04-1362 | 20 | 5154116 | 5550898 | 1.499290616 | 4 | amp |
| TCGA-04-1362 | 20 | 5554497 | 5948277 | 2.227562688 | 5 | amp |
| TCGA-04-1362 | 20 | 5948453 | 10582482 | 1.577233475 | 4 | amp |
| TCGA-04-1362 | 20 | 10594023 | 10644696 | 2.586084981 | 5 | amp |
| TCGA-04-1362 | 20 | 11898876 | 20512444 | 1.267277003 | 4 | amp |
| TCGA-04-1362 | 20 | 20517243 | 23383729 | 0.943692637 | 3 | amp |
| TCGA-04-1362 | 20 | 23420863 | 23966046 | 1.410554254 | 4 | amp |
| TCGA-04-1362 | 20 | 23966277 | 25394517 | 2.4632913 | 5 | amp |
| TCGA-04-1362 | 20 | 25397698 | 25426652 | 0.999706257 | 3 | amp |
| TCGA-04-1362 | 20 | 25434062 | 25498496 | 2.487419738 | 5 | amp |
| TCGA-04-1362 | 20 | 25506983 | 30388887 | 1.490660553 | 4 | amp |
| TCGA-04-1362 | 20 | 30411242 | 30818922 | 2.163699561 | 5 | amp |
| TCGA-04-1362 | 20 | 30822249 | 30956931 | 1.113432629 | 3 | amp |
| TCGA-04-1362 | 20 | 31015870 | 31395758 | 2.554725179 | 5 | amp |
| TCGA-04-1362 | 20 | 31413673 | 31436565 | 1.001470323 | 3 | amp |
| TCGA-04-1362 | 20 | 31571527 | 31671726 | 2.878054491 | 5 | amp |
| TCGA-04-1362 | 20 | 31673748 | 31830372 | 1.617982163 | 4 | amp |
| TCGA-04-1362 | 20 | 31873876 | 31897603 | 2.430540359 | 5 | amp |
| TCGA-04-1362 | 20 | 31946798 | 32224539 | 1.519749901 | 4 | amp |
| TCGA-04-1362 | 20 | 32228121 | 32666392 | 2.289564017 | 5 | amp |
| TCGA-04-1362 | 20 | 32677503 | 33517418 | 1.560355851 | 4 | amp |
| TCGA-04-1362 | 20 | 33519089 | 33609195 | 2.361899725 | 5 | amp |
| TCGA-04-1362 | 20 | 33622898 | 33725865 | 1.128168443 | 3 | amp |
| TCGA-04-1362 | 20 | 33730138 | 33891924 | 2.534041648 | 5 | amp |
| TCGA-04-1362 | 20 | 33894465 | 34487610 | 1.631374808 | 4 | amp |
| TCGA-04-1362 | 20 | 34501024 | 35509165 | 2.225246458 | 5 | amp |
| TCGA-04-1362 | 20 | 35515804 | 36718322 | 1.485693831 | 4 | amp |
| TCGA-04-1362 | 20 | 36759412 | 36936085 | 2.478434405 | 5 | amp |
| TCGA-04-1362 | 20 | 36937275 | 40046033 | 1.484278038 | 4 | amp |
| TCGA-04-1362 | 20 | 40049094 | 40162248 | 0.519652637 | 1 | loss |
| TCGA-04-1362 | 20 | 40179899 | 44053237 | 1.683697285 | 4 | amp |
| TCGA-04-1362 | 20 | 44108264 | 44416659 | 0.786743655 | 3 | amp |
| TCGA-04-1362 | 20 | 44417513 | 44434034 | 1.548887586 | 4 | amp |
| TCGA-04-1362 | 20 | 44437728 | 44586580 | 2.26636975 | 5 | amp |

| | | | | | | |
|---|---|---|---|---|---|---|
| TCGA-04-1362 | 20 | 44589065 | 46282245 | 1.584176255 | 4 | amp |
| TCGA-04-1362 | 20 | 46287060 | 47557810 | 2.668939997 | 5 | amp |
| TCGA-04-1362 | 20 | 47558342 | 48491419 | 1.609622252 | 4 | amp |
| TCGA-04-1362 | 20 | 48494492 | 49493203 | 2.594311427 | 5 | amp |
| TCGA-04-1362 | 20 | 49507935 | 49558675 | 1.432829031 | 4 | amp |
| TCGA-04-1362 | 20 | 49562226 | 52195050 | 2.558870391 | 5 | amp |
| TCGA-04-1362 | 20 | 52197962 | 52675281 | 1.351329469 | 4 | amp |
| TCGA-04-1362 | 20 | 52773682 | 53171585 | 2.330755176 | 5 | amp |
| TCGA-04-1362 | 20 | 53205018 | 54959412 | 1.757898667 | 4 | amp |
| TCGA-04-1362 | 20 | 54961270 | 56886198 | 2.874943806 | 5 | amp |
| TCGA-04-1362 | 20 | 56918724 | 57019331 | 1.246822474 | 3 | amp |
| TCGA-04-1362 | 20 | 57035777 | 58425556 | 2.5941946 | 5 | amp |
| TCGA-04-1362 | 20 | 58440410 | 58482508 | 0.786752523 | 3 | amp |
| TCGA-04-1362 | 20 | 58486792 | 58559868 | 1.784434404 | 4 | amp |
| TCGA-04-1362 | 20 | 58559999 | 62926333 | 2.970028185 | 5 | amp |
| TCGA-04-1362 | 21 | 10951226 | 32435274 | 1.045513171 | 3 | amp |
| TCGA-04-1362 | 21 | 32492680 | 34030223 | 1.860293498 | 4 | amp |
| TCGA-04-1362 | 21 | 34037181 | 34633039 | 0.989234138 | 3 | amp |
| TCGA-04-1362 | 21 | 34635011 | 37537220 | 1.492395869 | 4 | amp |
| TCGA-04-1362 | 21 | 37571308 | 37626258 | 2.168821066 | 5 | amp |
| TCGA-04-1362 | 21 | 37632942 | 37788678 | 1.294588506 | 4 | amp |
| TCGA-04-1362 | 21 | 38084817 | 38137512 | 2.489708425 | 5 | amp |
| TCGA-04-1362 | 21 | 38139492 | 38461202 | 1.596947733 | 4 | amp |
| TCGA-04-1362 | 21 | 38462499 | 38525577 | 0.802340933 | 3 | amp |
| TCGA-04-1362 | 21 | 38528902 | 40553810 | 1.391478301 | 4 | amp |
| TCGA-04-1362 | 21 | 40665727 | 42710486 | 1.467478495 | 4 | amp |
| TCGA-04-1362 | 21 | 42716371 | 48111215 | 2.308241552 | 5 | amp |
| TCGA-04-1362 | 22 | 16100468 | 16989152 | 1.026738006 | 3 | amp |
| TCGA-04-1362 | 22 | 17052859 | 21161757 | 1.515393413 | 4 | amp |
| TCGA-04-1362 | 22 | 21165197 | 21304185 | 0.951755087 | 3 | amp |
| TCGA-04-1362 | 22 | 21331966 | 22123670 | 1.989178807 | 4 | amp |
| TCGA-04-1362 | 22 | 22127156 | 22162163 | 0.837804845 | 3 | amp |
| TCGA-04-1362 | 22 | 22239811 | 23917271 | 1.681239299 | 4 | amp |
| TCGA-04-1362 | 22 | 23955436 | 23974226 | 1.054443649 | 3 | amp |
| TCGA-04-1362 | 22 | 23982732 | 24325812 | 1.952318571 | 4 | amp |
| TCGA-04-1362 | 22 | 24431906 | 24451658 | 0.820592889 | 3 | amp |
| TCGA-04-1362 | 22 | 24452588 | 24622786 | 1.545326228 | 4 | amp |
| TCGA-04-1362 | 22 | 24698155 | 24765296 | 0.793725565 | 3 | amp |
| TCGA-04-1362 | 22 | 24807463 | 25084092 | 1.53816076 | 4 | amp |
| TCGA-04-1362 | 22 | 25115376 | 25150850 | 0.762217601 | 3 | amp |
| TCGA-04-1362 | 22 | 25151661 | 28250963 | 1.464173874 | 4 | amp |
| TCGA-04-1362 | 22 | 29099463 | 30228333 | 1.500338516 | 4 | amp |
| TCGA-04-1362 | 22 | 30408334 | 30421824 | 1.594025929 | 4 | amp |
| TCGA-04-1362 | 22 | 30489906 | 30572163 | 0.636586749 | 1 | loss |
| TCGA-04-1362 | 22 | 30639604 | 31329539 | 1.836349045 | 4 | amp |
| TCGA-04-1362 | 22 | 31329992 | 31345842 | 0.867128454 | 3 | amp |
| TCGA-04-1362 | 22 | 31346322 | 31795750 | 1.735362581 | 4 | amp |
| TCGA-04-1362 | 22 | 31796517 | 31979969 | 0.851455476 | 3 | amp |
| TCGA-04-1362 | 22 | 31981026 | 32174179 | 1.574652392 | 4 | amp |
| TCGA-04-1362 | 22 | 32179871 | 32555206 | 0.93576291 | 3 | amp |

| | | | | | | |
|---|---|---|---|---|---|---|
| TCGA-04-1362 | 22 | 32579498 | 32838747 | 1.461917795 | 4 | amp |
| TCGA-04-1362 | 22 | 32841540 | 32894563 | 0.799174857 | 3 | amp |
| TCGA-04-1362 | 22 | 32913965 | 32937747 | 2.041014985 | 4 | amp |
| TCGA-04-1362 | 22 | 32992630 | 35684428 | 1.037629907 | 3 | amp |
| TCGA-04-1362 | 22 | 35688925 | 36013316 | 1.977955801 | 4 | amp |
| TCGA-04-1362 | 22 | 36052407 | 36334979 | 0.898587203 | 3 | amp |
| TCGA-04-1362 | 22 | 36537166 | 38247511 | 1.828677735 | 4 | amp |
| TCGA-04-1362 | 22 | 38251521 | 38282871 | 1.019101087 | 3 | amp |
| TCGA-04-1362 | 22 | 38284391 | 38710184 | 2.040311803 | 4 | amp |
| TCGA-04-1362 | 22 | 38757447 | 38935431 | 0.887602132 | 3 | amp |
| TCGA-04-1362 | 22 | 38945860 | 40068318 | 2.054307478 | 4 | amp |
| TCGA-04-1362 | 22 | 40139625 | 40356238 | 0.86989183 | 3 | amp |
| TCGA-04-1362 | 22 | 40361957 | 41228683 | 1.556350191 | 4 | amp |
| TCGA-04-1362 | 22 | 41231528 | 41322475 | 0.954668228 | 3 | amp |
| TCGA-04-1362 | 22 | 41349537 | 43204954 | 1.728768909 | 4 | amp |
| TCGA-04-1362 | 22 | 43206764 | 43243698 | 0.986441502 | 3 | amp |
| TCGA-04-1362 | 22 | 43267358 | 43950982 | 1.934764029 | 4 | amp |
| TCGA-04-1362 | 22 | 43972152 | 44178248 | 0.952280801 | 3 | amp |
| TCGA-04-1362 | 22 | 44221876 | 45309855 | 1.860732305 | 4 | amp |
| TCGA-04-1362 | 22 | 45564002 | 45580554 | 1.02937575 | 3 | amp |
| TCGA-04-1362 | 22 | 45593629 | 45732310 | 1.808618323 | 4 | amp |
| TCGA-04-1362 | 22 | 45790545 | 45996376 | 1.701600821 | 4 | amp |
| TCGA-04-1362 | 22 | 46085567 | 46239084 | 0.97639147 | 3 | amp |
| TCGA-04-1362 | 22 | 46239489 | 51237627 | 1.846801082 | 4 | amp |
| TCGA-04-1362 | 3 | 361444 | 8579178 | 1.525643461 | 4 | amp |
| TCGA-04-1362 | 3 | 8590241 | 8673824 | 2.777569667 | 5 | amp |
| TCGA-04-1362 | 3 | 8775559 | 9506412 | 1.640396454 | 4 | amp |
| TCGA-04-1362 | 3 | 9512137 | 9754316 | 2.635386797 | 5 | amp |
| TCGA-04-1362 | 3 | 9754370 | 9757243 | 1.806357316 | 4 | amp |
| TCGA-04-1362 | 3 | 9757628 | 9990639 | 3.280904994 | 5 | amp |
| TCGA-04-1362 | 3 | 9990721 | 10140638 | 1.838514465 | 4 | amp |
| TCGA-04-1362 | 3 | 10142818 | 11340922 | 3.057599824 | 5 | amp |
| TCGA-04-1362 | 3 | 11348355 | 12779289 | 1.729964265 | 4 | amp |
| TCGA-04-1362 | 3 | 12779590 | 14526564 | 3.179851243 | 5 | amp |
| TCGA-04-1362 | 3 | 14536303 | 14768589 | 1.813316251 | 4 | amp |
| TCGA-04-1362 | 3 | 14769860 | 14967760 | 2.698803031 | 5 | amp |
| TCGA-04-1362 | 3 | 14974040 | 17209340 | 1.560348376 | 4 | amp |
| TCGA-04-1362 | 3 | 17226590 | 20216605 | 1.138111648 | 3 | amp |
| TCGA-04-1362 | 3 | 20218061 | 24169298 | 1.581750623 | 4 | amp |
| TCGA-04-1362 | 3 | 24174759 | 31677575 | 1.078703461 | 3 | amp |
| TCGA-04-1362 | 3 | 31703495 | 31744045 | 2.594928627 | 5 | amp |
| TCGA-04-1362 | 3 | 31774705 | 32188229 | 1.721209585 | 4 | amp |
| TCGA-04-1362 | 3 | 32200364 | 32493984 | 2.582077457 | 5 | amp |
| TCGA-04-1362 | 3 | 32525400 | 37061997 | 1.520341514 | 4 | amp |
| TCGA-04-1362 | 3 | 37067192 | 37170656 | 0.908264507 | 3 | amp |
| TCGA-04-1362 | 3 | 37190369 | 38043391 | 1.813390939 | 4 | amp |
| TCGA-04-1362 | 3 | 38043826 | 38182819 | 3.228167218 | 5 | amp |
| TCGA-04-1362 | 3 | 38315754 | 38671920 | 3.046735083 | 5 | amp |
| TCGA-04-1362 | 3 | 38738786 | 38805101 | 1.865053355 | 4 | amp |
| TCGA-04-1362 | 3 | 38812692 | 38967012 | 0.938913358 | 3 | amp |

| | | | | | | |
|---|---|---|---|---|---|---|
| TCGA-04-1362 | 3 | 38968207 | 39174756 | 1.964995331 | 4 | amp |
| TCGA-04-1362 | 3 | 39175673 | 39230990 | 2.622260268 | 5 | amp |
| TCGA-04-1362 | 3 | 39306896 | 40803262 | 1.904663929 | 4 | amp |
| TCGA-04-1362 | 3 | 41265505 | 41861016 | 0.942415618 | 3 | amp |
| TCGA-04-1362 | 3 | 41877319 | 42236484 | 1.816291929 | 4 | amp |
| TCGA-04-1362 | 3 | 42240646 | 42573839 | 2.66136222 | 5 | amp |
| TCGA-04-1362 | 3 | 42576381 | 42732543 | 1.567708184 | 4 | amp |
| TCGA-04-1362 | 3 | 42733369 | 42787526 | 2.97138874 | 5 | amp |
| TCGA-04-1362 | 3 | 42788656 | 44938314 | 1.713020204 | 4 | amp |
| TCGA-04-1362 | 3 | 44945337 | 45043124 | 2.548072312 | 5 | amp |
| TCGA-04-1362 | 3 | 45046724 | 46593138 | 1.627228259 | 4 | amp |
| TCGA-04-1362 | 3 | 46619416 | 47099005 | 2.631255817 | 5 | amp |
| TCGA-04-1362 | 3 | 47168085 | 47869021 | 1.795451176 | 4 | amp |
| TCGA-04-1362 | 3 | 47882337 | 47912806 | 2.450542337 | 5 | amp |
| TCGA-04-1362 | 3 | 47913348 | 48448525 | 1.512194783 | 4 | amp |
| TCGA-04-1362 | 3 | 48450690 | 48716909 | 3.33172089 | 5 | amp |
| TCGA-04-1362 | 3 | 48716956 | 49020727 | 1.811097621 | 4 | amp |
| TCGA-04-1362 | 3 | 49039890 | 49167481 | 2.814129622 | 5 | amp |
| TCGA-04-1362 | 3 | 49168082 | 49413064 | 2.071918572 | 4 | amp |
| TCGA-04-1362 | 3 | 49452213 | 49950835 | 2.627147527 | 5 | amp |
| TCGA-04-1362 | 3 | 50000006 | 50154867 | 1.730704066 | 4 | amp |
| TCGA-04-1362 | 3 | 50155702 | 50685520 | 3.006361436 | 5 | amp |
| TCGA-04-1362 | 3 | 50900801 | 51102072 | 1.541751181 | 4 | amp |
| TCGA-04-1362 | 3 | 51112768 | 51674015 | 1.08408772 | 3 | amp |
| TCGA-04-1362 | 3 | 51675763 | 51863764 | 1.98259299 | 4 | amp |
| TCGA-04-1362 | 3 | 51864377 | 52238073 | 2.671887915 | 5 | amp |
| TCGA-04-1362 | 3 | 52238699 | 52304836 | 1.934706126 | 4 | amp |
| TCGA-04-1362 | 3 | 52356444 | 52512648 | 3.022454408 | 5 | amp |
| TCGA-04-1362 | 3 | 52582044 | 52813648 | 1.5063323 | 4 | amp |
| TCGA-04-1362 | 3 | 52814281 | 52889495 | 3.44657118 | 5 | amp |
| TCGA-04-1362 | 3 | 52939101 | 53218999 | 1.5496416 | 4 | amp |
| TCGA-04-1362 | 3 | 53219577 | 53262393 | 3.319661685 | 5 | amp |
| TCGA-04-1362 | 3 | 53262937 | 53922524 | 2.129272013 | 4 | amp |
| TCGA-04-1362 | 3 | 53925745 | 56695039 | 0.965328482 | 3 | amp |
| TCGA-04-1362 | 3 | 56763293 | 57286377 | 1.599107585 | 4 | amp |
| TCGA-04-1362 | 3 | 57290941 | 58088118 | 0.974739346 | 3 | amp |
| TCGA-04-1362 | 3 | 58089628 | 58118663 | 1.825946631 | 4 | amp |
| TCGA-04-1362 | 3 | 58120300 | 58132753 | 2.552301189 | 5 | amp |
| TCGA-04-1362 | 3 | 58133891 | 58870472 | 1.65300558 | 4 | amp |
| TCGA-04-1362 | 3 | 58899391 | 65607824 | 1.038945482 | 3 | amp |
| TCGA-04-1362 | 3 | 66023616 | 66512957 | 1.690170999 | 4 | amp |
| TCGA-04-1362 | 3 | 67049420 | 69169267 | 0.985240135 | 3 | amp |
| TCGA-04-1362 | 3 | 69171210 | 69928589 | 1.62414826 | 4 | amp |
| TCGA-04-1362 | 3 | 69985829 | 87313669 | 1.044034418 | 3 | amp |
| TCGA-04-1362 | 3 | 87322472 | 97467547 | 1.488165406 | 4 | amp |
| TCGA-04-1362 | 3 | 97486892 | 101212832 | 1.097899335 | 3 | amp |
| TCGA-04-1362 | 3 | 101219878 | 105250937 | 1.865643456 | 4 | amp |
| TCGA-04-1362 | 3 | 105252430 | 108822789 | 1.132226833 | 3 | amp |
| TCGA-04-1362 | 3 | 108829552 | 112727327 | 1.761264983 | 4 | amp |
| TCGA-04-1362 | 3 | 112729430 | 113005733 | 3.033696679 | 5 | amp |

| | | | | | | |
|---|---|---|---|---|---|---|
| TCGA-04-1362 | 3 | 113081945 | 113152521 | 1.225127247 | 3 | amp |
| TCGA-04-1362 | 3 | 113164120 | 113184685 | 3.112667708 | 5 | amp |
| TCGA-04-1362 | 3 | 113186938 | 119084273 | 1.652797684 | 4 | amp |
| TCGA-04-1362 | 3 | 119087170 | 119128662 | 2.590958237 | 5 | amp |
| TCGA-04-1362 | 3 | 119132646 | 121228598 | 1.701310764 | 4 | amp |
| TCGA-04-1362 | 3 | 121228867 | 121265522 | 2.48742492 | 5 | amp |
| TCGA-04-1362 | 3 | 121289551 | 121305457 | 1.334635787 | 4 | amp |
| TCGA-04-1362 | 3 | 121339333 | 121363779 | 2.753922954 | 5 | amp |
| TCGA-04-1362 | 3 | 121366127 | 122634798 | 1.719081067 | 4 | amp |
| TCGA-04-1362 | 3 | 122642411 | 122928275 | 3.767793115 | 5 | amp |
| TCGA-04-1362 | 3 | 122942367 | 122990588 | 1.63538635 | 4 | amp |
| TCGA-04-1362 | 3 | 123003398 | 123221587 | 3.686406407 | 5 | amp |
| TCGA-04-1362 | 3 | 123247216 | 123339238 | 1.843597699 | 4 | amp |
| TCGA-04-1362 | 3 | 123345606 | 123426905 | 3.18681582 | 5 | amp |
| TCGA-04-1362 | 3 | 123427523 | 124356162 | 1.82279452 | 4 | amp |
| TCGA-04-1362 | 3 | 124369569 | 124398394 | 2.803330398 | 5 | amp |
| TCGA-04-1362 | 3 | 124412604 | 124420983 | 1.673605874 | 4 | amp |
| TCGA-04-1362 | 3 | 124431731 | 124536572 | 2.67033438 | 5 | amp |
| TCGA-04-1362 | 3 | 124538573 | 125639767 | 1.905240332 | 4 | amp |
| TCGA-04-1362 | 3 | 125648152 | 127540718 | 3.362827554 | 5 | amp |
| TCGA-04-1362 | 3 | 127641899 | 127646921 | 1.686227416 | 4 | amp |
| TCGA-04-1362 | 3 | 127648930 | 127819632 | 2.84432483 | 5 | amp |
| TCGA-04-1362 | 3 | 127820356 | 127831916 | 1.753023823 | 4 | amp |
| TCGA-04-1362 | 3 | 127838153 | 129290194 | 3.191522027 | 5 | amp |
| TCGA-04-1362 | 3 | 129370270 | 129547263 | 1.385921456 | 4 | amp |
| TCGA-04-1362 | 3 | 129695446 | 129818210 | 4.259478544 | 5 | amp |
| TCGA-04-1362 | 3 | 130159007 | 130189822 | 1.164090817 | 3 | amp |
| TCGA-04-1362 | 3 | 130202821 | 130329583 | 2.677344868 | 5 | amp |
| TCGA-04-1362 | 3 | 130340635 | 132203533 | 1.386936229 | 4 | amp |
| TCGA-04-1362 | 3 | 132207098 | 132338410 | 1.068594306 | 3 | amp |
| TCGA-04-1362 | 3 | 132345477 | 133376752 | 1.473844566 | 4 | amp |
| TCGA-04-1362 | 3 | 133377806 | 133698513 | 3.327238104 | 5 | amp |
| TCGA-04-1362 | 3 | 133876992 | 137742701 | 1.713146459 | 4 | amp |
| TCGA-04-1362 | 3 | 137743387 | 137928756 | 2.657956089 | 5 | amp |
| TCGA-04-1362 | 3 | 137940743 | 138003380 | 2.028432392 | 4 | amp |
| TCGA-04-1362 | 3 | 138007865 | 138216972 | 3.306104586 | 5 | amp |
| TCGA-04-1362 | 3 | 138218918 | 138227393 | 2.068570248 | 4 | amp |
| TCGA-04-1362 | 3 | 138244308 | 138256224 | 2.761249645 | 5 | amp |
| TCGA-04-1362 | 3 | 138289129 | 138291794 | 1.312447615 | 4 | amp |
| TCGA-04-1362 | 3 | 138329779 | 138384081 | 2.587147679 | 5 | amp |
| TCGA-04-1362 | 3 | 138400787 | 139181162 | 1.632865027 | 4 | amp |
| TCGA-04-1362 | 3 | 139195204 | 139346571 | 2.619158577 | 5 | amp |
| TCGA-04-1362 | 3 | 139894733 | 140283014 | 1.823103351 | 4 | amp |
| TCGA-04-1362 | 3 | 140284874 | 140678403 | 2.999410363 | 5 | amp |
| TCGA-04-1362 | 3 | 140681958 | 140695319 | 1.233659986 | 3 | amp |
| TCGA-04-1362 | 3 | 140865897 | 141164824 | 2.92706439 | 5 | amp |
| TCGA-04-1362 | 3 | 141230943 | 141299311 | 1.591557127 | 4 | amp |
| TCGA-04-1362 | 3 | 141299920 | 141535903 | 2.564461688 | 5 | amp |
| TCGA-04-1362 | 3 | 141622405 | 141812861 | 1.860141014 | 4 | amp |
| TCGA-04-1362 | 3 | 141884417 | 141906643 | 2.494255702 | 5 | amp |

| | | | | | | |
|---|---|---|---|---|---|---|
| TCGA-04-1362 | 3 | 141917589 | 142140002 | 1.620734216 | 4 | amp |
| TCGA-04-1362 | 3 | 142140291 | 142178266 | 2.504822306 | 5 | amp |
| TCGA-04-1362 | 3 | 142180736 | 146323052 | 1.715221635 | 4 | amp |
| TCGA-04-1362 | 3 | 147106555 | 148460047 | 3.58754097 | 5 | amp |
| TCGA-04-1362 | 3 | 148545585 | 148614520 | 1.6763002 | 4 | amp |
| TCGA-04-1362 | 3 | 148703573 | 148858337 | 2.817752967 | 5 | amp |
| TCGA-04-1362 | 3 | 148858769 | 150845811 | 1.897295622 | 4 | amp |
| TCGA-04-1362 | 3 | 150873912 | 150883820 | 2.626473198 | 5 | amp |
| TCGA-04-1362 | 3 | 150903062 | 154056073 | 1.779594903 | 4 | amp |
| TCGA-04-1362 | 3 | 154138811 | 154836627 | 2.522430155 | 5 | amp |
| TCGA-04-1362 | 3 | 154855837 | 154898258 | 1.264225836 | 4 | amp |
| TCGA-04-1362 | 3 | 155198688 | 155241799 | 2.750755599 | 5 | amp |
| TCGA-04-1362 | 3 | 155267541 | 156867389 | 1.936755134 | 4 | amp |
| TCGA-04-1362 | 3 | 156867545 | 156876769 | 2.713163761 | 5 | amp |
| TCGA-04-1362 | 3 | 156978860 | 158422767 | 1.948037259 | 4 | amp |
| TCGA-04-1362 | 3 | 158428504 | 158537555 | 2.636049544 | 5 | amp |
| TCGA-04-1362 | 3 | 158537999 | 160099521 | 1.841556984 | 4 | amp |
| TCGA-04-1362 | 3 | 160102279 | 160132349 | 2.559654294 | 5 | amp |
| TCGA-04-1362 | 3 | 160134000 | 160222852 | 2.002855835 | 4 | amp |
| TCGA-04-1362 | 3 | 160225855 | 160243669 | 2.604661312 | 5 | amp |
| TCGA-04-1362 | 3 | 160243690 | 161221735 | 1.490581119 | 4 | amp |
| TCGA-04-1362 | 3 | 164697123 | 164793814 | 1.048127384 | 3 | amp |
| TCGA-04-1362 | 3 | 164905645 | 167742407 | 1.64891727 | 4 | amp |
| TCGA-04-1362 | 3 | 167742679 | 167754822 | 2.888397505 | 5 | amp |
| TCGA-04-1362 | 3 | 167758525 | 169522054 | 1.922103624 | 4 | amp |
| TCGA-04-1362 | 3 | 169524617 | 169694846 | 3.002513717 | 5 | amp |
| TCGA-04-1362 | 3 | 169700431 | 169863319 | 1.356085978 | 4 | amp |
| TCGA-04-1362 | 3 | 169866832 | 170011353 | 2.660836496 | 5 | amp |
| TCGA-04-1362 | 3 | 170013678 | 170020931 | 1.966480135 | 4 | amp |
| TCGA-04-1362 | 3 | 170078038 | 170244753 | 2.629584553 | 5 | amp |
| TCGA-04-1362 | 3 | 170584120 | 170824560 | 1.679967324 | 4 | amp |
| TCGA-04-1362 | 3 | 170824923 | 170858302 | 2.665971408 | 5 | amp |
| TCGA-04-1362 | 3 | 170879031 | 176755982 | 1.92171332 | 4 | amp |
| TCGA-04-1362 | 3 | 176756041 | 176771753 | 2.836509567 | 5 | amp |
| TCGA-04-1362 | 3 | 178525165 | 178546183 | 1.727780406 | 4 | amp |
| TCGA-04-1362 | 3 | 178560402 | 178922393 | 2.787654958 | 5 | amp |
| TCGA-04-1362 | 3 | 178927375 | 178968917 | 1.732454765 | 4 | amp |
| TCGA-04-1362 | 3 | 178976676 | 179069875 | 2.815058917 | 5 | amp |
| TCGA-04-1362 | 3 | 179076588 | 179144019 | 2.149859397 | 4 | amp |
| TCGA-04-1362 | 3 | 179287589 | 180328353 | 2.962942266 | 5 | amp |
| TCGA-04-1362 | 3 | 180332666 | 182591821 | 2.019768456 | 4 | amp |
| TCGA-04-1362 | 3 | 182597240 | 182607386 | 2.61804547 | 5 | amp |
| TCGA-04-1362 | 3 | 182614460 | 182763384 | 1.907125874 | 4 | amp |
| TCGA-04-1362 | 3 | 182769927 | 182913086 | 2.79276135 | 5 | amp |
| TCGA-04-1362 | 3 | 182923642 | 182937737 | 1.923998625 | 4 | amp |
| TCGA-04-1362 | 3 | 182941119 | 183007017 | 2.766652903 | 5 | amp |
| TCGA-04-1362 | 3 | 183013056 | 183056767 | 2.14093587 | 4 | amp |
| TCGA-04-1362 | 3 | 183059312 | 183454621 | 2.966241718 | 5 | amp |
| TCGA-04-1362 | 3 | 183465421 | 183479408 | 2.052492932 | 4 | amp |
| TCGA-04-1362 | 3 | 183479825 | 183558454 | 3.424216002 | 5 | amp |

| | | | | | | |
|---|---|---|---|---|---|---|
| TCGA-04-1362 | 3 | 183560010 | 183585870 | 2.004522867 | 4 | amp |
| TCGA-04-1362 | 3 | 183639048 | 184557572 | 4.993037355 | 5 | amp |
| TCGA-04-1362 | 3 | 184560970 | 185161448 | 2.135256734 | 4 | amp |
| TCGA-04-1362 | 3 | 185165535 | 185766658 | 3.552205605 | 5 | amp |
| TCGA-04-1362 | 3 | 185769779 | 185783911 | 1.985190863 | 4 | amp |
| TCGA-04-1362 | 3 | 185797569 | 186358933 | 3.819406813 | 5 | amp |
| TCGA-04-1362 | 3 | 186360243 | 186395677 | 2.013051606 | 4 | amp |
| TCGA-04-1362 | 3 | 186440177 | 187451485 | 4.01490383 | 5 | amp |
| TCGA-04-1362 | 3 | 188123854 | 189608684 | 1.859494412 | 4 | amp |
| TCGA-04-1362 | 3 | 189611962 | 190030862 | 2.809310797 | 5 | amp |
| TCGA-04-1362 | 3 | 190105860 | 190127878 | 2.019078377 | 4 | amp |
| TCGA-04-1362 | 3 | 190147301 | 190282170 | 3.025683717 | 5 | amp |
| TCGA-04-1362 | 3 | 190321879 | 190345294 | 1.823099492 | 4 | amp |
| TCGA-04-1362 | 3 | 190347092 | 190999986 | 2.600353271 | 5 | amp |
| TCGA-04-1362 | 3 | 191074847 | 191179278 | 1.643470267 | 4 | amp |
| TCGA-04-1362 | 3 | 191861790 | 192874644 | 2.680827385 | 5 | amp |
| TCGA-04-1362 | 3 | 192973388 | 193125201 | 1.882758404 | 4 | amp |
| TCGA-04-1362 | 3 | 193128754 | 193172005 | 3.144620843 | 5 | amp |
| TCGA-04-1362 | 3 | 193174759 | 193220474 | 2.017916498 | 4 | amp |
| TCGA-04-1362 | 3 | 193232423 | 193343995 | 2.789371818 | 5 | amp |
| TCGA-04-1362 | 3 | 193349367 | 193385073 | 1.59058681 | 4 | amp |
| TCGA-04-1362 | 3 | 193409823 | 194126866 | 3.309425404 | 5 | amp |
| TCGA-04-1362 | 3 | 194134467 | 194146284 | 1.904358291 | 4 | amp |
| TCGA-04-1362 | 3 | 194147772 | 194162234 | 3.13077257 | 5 | amp |
| TCGA-04-1362 | 3 | 194165394 | 194181593 | 1.785861039 | 4 | amp |
| TCGA-04-1362 | 3 | 194182833 | 195012549 | 4.004326944 | 5 | amp |
| TCGA-04-1362 | 3 | 195012967 | 195246018 | 2.012074754 | 4 | amp |
| TCGA-04-1362 | 3 | 195250476 | 196555292 | 3.47704011 | 5 | amp |
| TCGA-04-1362 | 3 | 196612028 | 196650473 | 1.749675799 | 4 | amp |
| TCGA-04-1362 | 3 | 196654618 | 196802784 | 3.105934274 | 5 | amp |
| TCGA-04-1362 | 3 | 196803446 | 196876700 | 2.086086979 | 4 | amp |
| TCGA-04-1362 | 3 | 196888499 | 197541970 | 3.164974004 | 5 | amp |
| TCGA-04-1362 | 3 | 197543989 | 197585800 | 1.636502579 | 4 | amp |
| TCGA-04-1362 | 3 | 197592257 | 197701356 | 2.961438053 | 5 | amp |
| TCGA-04-1362 | 3 | 197701867 | 197762928 | 2.075047232 | 4 | amp |
| TCGA-04-1362 | 3 | 197765397 | 197955154 | 2.579752503 | 5 | amp |
| TCGA-04-1362 | 4 | 53323 | 466440 | 1.029609595 | 3 | amp |
| TCGA-04-1362 | 4 | 494167 | 3201682 | 1.437697518 | 4 | amp |
| TCGA-04-1362 | 4 | 3205684 | 3319797 | 2.227943201 | 5 | amp |
| TCGA-04-1362 | 4 | 3371931 | 6826415 | 1.627800178 | 4 | amp |
| TCGA-04-1362 | 4 | 6843712 | 6882723 | 1.069645123 | 3 | amp |
| TCGA-04-1362 | 4 | 6925109 | 8465824 | 2.242062535 | 5 | amp |
| TCGA-04-1362 | 4 | 8467076 | 10503077 | 0.991339947 | 3 | amp |
| TCGA-04-1362 | 4 | 39408561 | 40104866 | 1.009964613 | 3 | amp |
| TCGA-04-1362 | 4 | 40108461 | 41663541 | 1.4059674 | 4 | amp |
| TCGA-04-1362 | 4 | 41664799 | 46981141 | 1.065293625 | 3 | amp |
| TCGA-04-1362 | 4 | 46994813 | 47584088 | 1.454554286 | 4 | amp |
| TCGA-04-1362 | 4 | 47589009 | 52883831 | 0.992408815 | 3 | amp |
| TCGA-04-1362 | 4 | 52890074 | 54306821 | 1.385849644 | 4 | amp |
| TCGA-04-1362 | 4 | 54308786 | 56436076 | 1.014362141 | 3 | amp |

| | | | | | | |
|---|---|---|---|---|---|---|
| TCGA-04-1362 | 4 | 56446972 | 56734715 | 1.840611347 | 4 | amp |
| TCGA-04-1362 | 4 | 56736837 | 56770704 | 0.805970153 | 3 | amp |
| TCGA-04-1362 | 4 | 56818262 | 57877270 | 1.498340669 | 4 | amp |
| TCGA-04-1362 | 4 | 57881624 | 68396642 | 1.133687478 | 3 | amp |
| TCGA-04-1362 | 4 | 68406672 | 69095284 | 1.290200343 | 4 | amp |
| TCGA-04-1362 | 4 | 69096889 | 71503593 | 1.065267564 | 3 | amp |
| TCGA-04-1362 | 4 | 71507681 | 72215828 | 1.425438287 | 4 | amp |
| TCGA-04-1362 | 4 | 72222694 | 73673373 | 1.066403472 | 3 | amp |
| TCGA-04-1362 | 4 | 73923797 | 74964697 | 1.580929629 | 4 | amp |
| TCGA-04-1362 | 4 | 75245147 | 76530821 | 1.590224814 | 4 | amp |
| TCGA-04-1362 | 4 | 76532305 | 76782126 | 1.028035735 | 3 | amp |
| TCGA-04-1362 | 4 | 76785576 | 77082944 | 1.37623254 | 4 | amp |
| TCGA-04-1362 | 4 | 77084336 | 82065494 | 1.021664267 | 3 | amp |
| TCGA-04-1362 | 4 | 82069981 | 84243564 | 1.351525002 | 4 | amp |
| TCGA-04-1362 | 4 | 84328599 | 84525930 | 1.063396677 | 3 | amp |
| TCGA-04-1362 | 4 | 85165487 | 85676605 | 1.49786194 | 4 | amp |
| TCGA-04-1362 | 4 | 85678046 | 87725055 | 0.95870297 | 3 | amp |
| TCGA-04-1362 | 4 | 87726416 | 88356432 | 1.369651618 | 4 | amp |
| TCGA-04-1362 | 4 | 88359416 | 99812472 | 1.048974436 | 3 | amp |
| TCGA-04-1362 | 4 | 99823019 | 108831727 | 0.516944552 | 1 | loss |
| TCGA-04-1362 | 4 | 108866083 | 109684086 | 1.405231963 | 4 | amp |
| TCGA-04-1362 | 4 | 109735013 | 139153538 | 1.032399595 | 3 | amp |
| TCGA-04-1362 | 4 | 139157391 | 142654005 | 1.558905842 | 4 | amp |
| TCGA-04-1362 | 4 | 142949880 | 144109166 | 1.010088233 | 3 | amp |
| TCGA-04-1362 | 5 | 151610 | 5306848 | 3.104584874 | 5 | amp |
| TCGA-04-1362 | 5 | 5436499 | 5448041 | 1.517321368 | 4 | amp |
| TCGA-04-1362 | 5 | 5454647 | 7883448 | 2.560178545 | 5 | amp |
| TCGA-04-1362 | 5 | 7885770 | 9630149 | 1.899449449 | 4 | amp |
| TCGA-04-1362 | 5 | 10227505 | 10402270 | 2.554059294 | 5 | amp |
| TCGA-04-1362 | 5 | 10402469 | 10433810 | 1.543988283 | 4 | amp |
| TCGA-04-1362 | 5 | 10448313 | 11111182 | 2.685087419 | 5 | amp |
| TCGA-04-1362 | 5 | 11117522 | 13944578 | 1.726075354 | 4 | amp |
| TCGA-04-1362 | 5 | 14270910 | 14758767 | 3.240928674 | 5 | amp |
| TCGA-04-1362 | 5 | 14769041 | 32069775 | 1.664493564 | 4 | amp |
| TCGA-04-1362 | 5 | 32071416 | 32263403 | 2.603892593 | 5 | amp |
| TCGA-04-1362 | 5 | 32268793 | 38493917 | 1.43012447 | 4 | amp |
| TCGA-04-1362 | 5 | 38496470 | 53467805 | 1.099534257 | 3 | amp |
| TCGA-04-1362 | 5 | 68720385 | 70429147 | 1.040754163 | 3 | amp |
| TCGA-04-1362 | 5 | 70754333 | 74001154 | 1.52661612 | 4 | amp |
| TCGA-04-1362 | 5 | 74009275 | 131305916 | 0.946097479 | 3 | amp |
| TCGA-04-1362 | 5 | 131307230 | 131822853 | 1.87602582 | 4 | amp |
| TCGA-04-1362 | 5 | 131993845 | 137235435 | 1.392448297 | 4 | amp |
| TCGA-04-1362 | 5 | 137241888 | 137536981 | 1.00297366 | 3 | amp |
| TCGA-04-1362 | 5 | 137536994 | 139930759 | 1.496124495 | 4 | amp |
| TCGA-04-1362 | 5 | 139931517 | 140077709 | 2.274929943 | 5 | amp |
| TCGA-04-1362 | 5 | 140078047 | 141009711 | 1.754709699 | 4 | amp |
| TCGA-04-1362 | 5 | 141014328 | 141391658 | 2.390444011 | 5 | amp |
| TCGA-04-1362 | 5 | 141511326 | 148016624 | 1.020100517 | 3 | amp |
| TCGA-04-1362 | 5 | 148206325 | 149361395 | 1.735996602 | 4 | amp |
| TCGA-04-1362 | 5 | 149374258 | 150072642 | 2.771324926 | 5 | amp |

| | | | | | | |
|---|---|---|---|---|---|---|
| TCGA-04-1362 | 5 | 150072734 | 153766051 | 1.510566455 | 4 | amp |
| TCGA-04-1362 | 5 | 153783627 | 154217774 | 2.747096245 | 5 | amp |
| TCGA-04-1362 | 5 | 154242776 | 154287384 | 1.691364405 | 4 | amp |
| TCGA-04-1362 | 5 | 154291252 | 155756647 | 2.844835823 | 5 | amp |
| TCGA-04-1362 | 5 | 155771472 | 156390240 | 1.684044046 | 4 | amp |
| TCGA-04-1362 | 5 | 156456707 | 156485021 | 2.664037028 | 5 | amp |
| TCGA-04-1362 | 5 | 156514088 | 156679712 | 1.490675973 | 4 | amp |
| TCGA-04-1362 | 5 | 156712369 | 157182306 | 2.642218529 | 5 | amp |
| TCGA-04-1362 | 5 | 157214646 | 158634844 | 1.754711935 | 4 | amp |
| TCGA-04-1362 | 5 | 158695880 | 159912467 | 2.715121184 | 5 | amp |
| TCGA-04-1362 | 5 | 159992443 | 167631681 | 1.804666934 | 4 | amp |
| TCGA-04-1362 | 5 | 167642040 | 167915793 | 2.817092972 | 5 | amp |
| TCGA-04-1362 | 5 | 167919637 | 167996006 | 1.606996975 | 4 | amp |
| TCGA-04-1362 | 5 | 168043924 | 168310365 | 2.921123685 | 5 | amp |
| TCGA-04-1362 | 5 | 168620458 | 169504897 | 1.58459639 | 4 | amp |
| TCGA-04-1362 | 5 | 169505914 | 170235799 | 2.48257731 | 5 | amp |
| TCGA-04-1362 | 5 | 170236545 | 170692887 | 1.36625646 | 4 | amp |
| TCGA-04-1362 | 5 | 170720875 | 170883855 | 2.93441584 | 5 | amp |
| TCGA-04-1362 | 5 | 171295569 | 171337808 | 1.410656172 | 4 | amp |
| TCGA-04-1362 | 5 | 171341317 | 172421861 | 2.971397239 | 5 | amp |
| TCGA-04-1362 | 5 | 172447227 | 172539405 | 1.559662723 | 4 | amp |
| TCGA-04-1362 | 5 | 172549993 | 173317898 | 2.925301617 | 5 | amp |
| TCGA-04-1362 | 5 | 173337476 | 173380304 | 1.257008141 | 3 | amp |
| TCGA-04-1362 | 5 | 173382906 | 175394311 | 2.304662605 | 5 | amp |
| TCGA-04-1362 | 5 | 175511920 | 175530831 | 1.70764944 | 4 | amp |
| TCGA-04-1362 | 5 | 175533036 | 176318589 | 2.632942508 | 5 | amp |
| TCGA-04-1362 | 5 | 176323022 | 176491616 | 1.820983177 | 4 | amp |
| TCGA-04-1362 | 5 | 176517312 | 177156099 | 2.696201188 | 5 | amp |
| TCGA-04-1362 | 5 | 177156460 | 177180252 | 1.736653725 | 4 | amp |
| TCGA-04-1362 | 5 | 177303491 | 179036568 | 3.024988392 | 5 | amp |
| TCGA-04-1362 | 5 | 179042511 | 180432902 | 1.096178866 | 3 | amp |
| TCGA-04-1362 | 5 | 180477048 | 180899507 | 1.566785202 | 4 | amp |
| TCGA-04-1362 | 6 | 105907 | 2948972 | 1.736521954 | 4 | amp |
| TCGA-04-1362 | 6 | 2949105 | 5369469 | 2.888184584 | 5 | amp |
| TCGA-04-1362 | 6 | 5404734 | 9933210 | 1.94659157 | 4 | amp |
| TCGA-04-1362 | 6 | 9933447 | 10430547 | 2.716630427 | 5 | amp |
| TCGA-04-1362 | 6 | 10529126 | 15497421 | 1.688982778 | 4 | amp |
| TCGA-04-1362 | 6 | 15501088 | 15533668 | 3.159503777 | 5 | amp |
| TCGA-04-1362 | 6 | 15593240 | 24302293 | 1.614946956 | 4 | amp |
| TCGA-04-1362 | 6 | 24353763 | 24463050 | 2.60520628 | 5 | amp |
| TCGA-04-1362 | 6 | 24466857 | 30515337 | 1.59523365 | 4 | amp |
| TCGA-04-1362 | 6 | 30520220 | 31138434 | 2.550480811 | 5 | amp |
| TCGA-04-1362 | 6 | 31170268 | 31499021 | 1.666351468 | 4 | amp |
| TCGA-04-1362 | 6 | 31499067 | 31839354 | 2.928409115 | 5 | amp |
| TCGA-04-1362 | 6 | 31842410 | 31913164 | 1.848811976 | 4 | amp |
| TCGA-04-1362 | 6 | 31913970 | 32052517 | 2.778325081 | 5 | amp |
| TCGA-04-1362 | 6 | 32083480 | 32137838 | 1.91927599 | 4 | amp |
| TCGA-04-1362 | 6 | 32137899 | 32151770 | 3.062534754 | 5 | amp |
| TCGA-04-1362 | 6 | 32154144 | 32805430 | 1.344995097 | 4 | amp |
| TCGA-04-1362 | 6 | 32805463 | 32820281 | 2.511178172 | 5 | amp |

| | | | | | | |
|---|---|---|---|---|---|---|
| TCGA-04-1362 | 6 | 32823905 | 33095794 | 1.837231691 | 4 | amp |
| TCGA-04-1362 | 6 | 33131374 | 34214933 | 3.156767749 | 5 | amp |
| TCGA-04-1362 | 6 | 34256498 | 35090123 | 1.492106994 | 4 | amp |
| TCGA-04-1362 | 6 | 35096778 | 35478908 | 2.778495169 | 5 | amp |
| TCGA-04-1362 | 6 | 35543605 | 36275548 | 1.540241983 | 4 | amp |
| TCGA-04-1362 | 6 | 36285090 | 36368315 | 3.068362888 | 5 | amp |
| TCGA-04-1362 | 6 | 36437834 | 36714383 | 1.775597705 | 4 | amp |
| TCGA-04-1362 | 6 | 36715904 | 36995397 | 2.837291283 | 5 | amp |
| TCGA-04-1362 | 6 | 37138691 | 37439711 | 1.593968462 | 4 | amp |
| TCGA-04-1362 | 6 | 37440168 | 37631932 | 2.587502502 | 5 | amp |
| TCGA-04-1362 | 6 | 37897642 | 38697779 | 1.537947151 | 4 | amp |
| TCGA-04-1362 | 6 | 38702255 | 38957987 | 1.022249475 | 3 | amp |
| TCGA-04-1362 | 6 | 38976517 | 38998204 | 1.638451596 | 4 | amp |
| TCGA-04-1362 | 6 | 39024130 | 39325161 | 2.577942885 | 5 | amp |
| TCGA-04-1362 | 6 | 39328118 | 39843294 | 1.562496569 | 4 | amp |
| TCGA-04-1362 | 6 | 39845947 | 41011486 | 2.644596592 | 5 | amp |
| TCGA-04-1362 | 6 | 41011657 | 41309890 | 1.596635106 | 4 | amp |
| TCGA-04-1362 | 6 | 41318329 | 41752861 | 2.891282072 | 5 | amp |
| TCGA-04-1362 | 6 | 41753026 | 42828106 | 1.719867274 | 4 | amp |
| TCGA-04-1362 | 6 | 42830208 | 43275535 | 3.770001792 | 5 | amp |
| TCGA-04-1362 | 6 | 43304872 | 43325543 | 1.20584411 | 3 | amp |
| TCGA-04-1362 | 6 | 43332953 | 43491794 | 3.728995045 | 5 | amp |
| TCGA-04-1362 | 6 | 43492170 | 43582325 | 1.927552926 | 4 | amp |
| TCGA-04-1362 | 6 | 43587968 | 44280024 | 3.365526466 | 5 | amp |
| TCGA-04-1362 | 6 | 44310781 | 52357163 | 1.400343753 | 4 | amp |
| TCGA-04-1362 | 6 | 52367941 | 52541995 | 2.725470813 | 5 | amp |
| TCGA-04-1362 | 6 | 52546601 | 73902470 | 1.359713199 | 4 | amp |
| TCGA-04-1362 | 6 | 73904115 | 74407328 | 2.783965163 | 5 | amp |
| TCGA-04-1362 | 6 | 74432927 | 90097341 | 1.392595156 | 4 | amp |
| TCGA-04-1362 | 6 | 90276695 | 90356350 | 2.862057425 | 5 | amp |
| TCGA-04-1362 | 6 | 90357747 | 100966062 | 1.523903038 | 4 | amp |
| TCGA-04-1362 | 6 | 100988030 | 102516423 | 0.916399337 | 3 | amp |
| TCGA-04-1362 | 6 | 105177497 | 109484182 | 1.797471015 | 4 | amp |
| TCGA-04-1362 | 6 | 109689986 | 109827612 | 3.044391129 | 5 | amp |
| TCGA-04-1362 | 6 | 109850205 | 111346815 | 1.28929644 | 4 | amp |
| TCGA-04-1362 | 6 | 111493849 | 112537679 | 0.946618263 | 3 | amp |
| TCGA-04-1362 | 6 | 112574900 | 116901550 | 1.549068275 | 4 | amp |
| TCGA-04-1362 | 6 | 134327860 | 135265150 | 1.904503618 | 4 | amp |
| TCGA-04-1362 | 6 | 135271002 | 137528216 | 0.943440385 | 3 | amp |
| TCGA-04-1362 | 6 | 138192331 | 142468540 | 1.592816724 | 4 | amp |
| TCGA-04-1362 | 6 | 142487351 | 144814616 | 1.13717558 | 3 | amp |
| TCGA-04-1362 | 6 | 147728408 | 148761578 | 1.42046282 | 4 | amp |
| TCGA-04-1362 | 6 | 148789576 | 148869742 | 2.579674698 | 5 | amp |
| TCGA-04-1362 | 6 | 149208083 | 150211330 | 1.342971222 | 4 | amp |
| TCGA-04-1362 | 6 | 150211934 | 150386851 | 2.714870509 | 5 | amp |
| TCGA-04-1362 | 6 | 150386986 | 152690797 | 1.4405111 | 4 | amp |
| TCGA-04-1362 | 6 | 152694142 | 153078313 | 0.89103838 | 3 | amp |
| TCGA-04-1362 | 6 | 153292243 | 159005090 | 1.673777813 | 4 | amp |
| TCGA-04-1362 | 6 | 159006276 | 159204744 | 2.448150842 | 5 | amp |
| TCGA-04-1362 | 6 | 159205627 | 160495017 | 1.782123068 | 4 | amp |

| | | | | | | |
|---|---|---|---|---|---|---|
| TCGA-04-1362 | 6 | 160496833 | 160557368 | 2.595823784 | 5 | amp |
| TCGA-04-1362 | 6 | 160557568 | 160575945 | 1.712545154 | 4 | amp |
| TCGA-04-1362 | 6 | 161508672 | 165989977 | 1.404312628 | 4 | amp |
| TCGA-04-1362 | 6 | 166574269 | 167436129 | 2.352112908 | 5 | amp |
| TCGA-04-1362 | 6 | 167438235 | 168353002 | 1.497940373 | 4 | amp |
| TCGA-04-1362 | 6 | 168355096 | 170064441 | 2.635757755 | 5 | amp |
| TCGA-04-1362 | 6 | 170065556 | 171055029 | 1.757271317 | 4 | amp |
| TCGA-04-1362 | 7 | 540695 | 4308405 | 3.313515301 | 5 | amp |
| TCGA-04-1362 | 7 | 4490793 | 6819358 | 1.460650823 | 4 | amp |
| TCGA-04-1362 | 7 | 6820419 | 6970595 | 1.207642156 | 3 | amp |
| TCGA-04-1362 | 7 | 24931983 | 30395456 | 1.036178196 | 3 | amp |
| TCGA-04-1362 | 7 | 30401961 | 30831225 | 1.724751964 | 4 | amp |
| TCGA-04-1362 | 7 | 30868263 | 31102965 | 2.166814547 | 5 | amp |
| TCGA-04-1362 | 7 | 31104439 | 31146323 | 1.783672526 | 4 | amp |
| TCGA-04-1362 | 7 | 31377848 | 31609457 | 1.024661889 | 3 | amp |
| TCGA-04-1362 | 7 | 42116313 | 43846884 | 1.185758536 | 3 | amp |
| TCGA-04-1362 | 7 | 43906259 | 47886692 | 1.697067057 | 4 | amp |
| TCGA-04-1362 | 7 | 47892684 | 50544391 | 1.059615155 | 3 | amp |
| TCGA-04-1362 | 7 | 50547432 | 57142378 | 1.353868384 | 4 | amp |
| TCGA-04-1362 | 7 | 57187542 | 72397540 | 1.090600581 | 3 | amp |
| TCGA-04-1362 | 7 | 72398872 | 76671422 | 1.484398163 | 4 | amp |
| TCGA-04-1362 | 7 | 76671547 | 76891650 | 1.016139748 | 3 | amp |
| TCGA-04-1362 | 7 | 87822372 | 97788753 | 1.049118093 | 3 | amp |
| TCGA-04-1362 | 7 | 97800769 | 99235946 | 1.831967041 | 4 | amp |
| TCGA-04-1362 | 7 | 99245915 | 99274234 | 0.945711421 | 3 | amp |
| TCGA-04-1362 | 7 | 99463516 | 99514413 | 1.18251841 | 3 | amp |
| TCGA-04-1362 | 7 | 99521100 | 100161640 | 1.546580511 | 4 | amp |
| TCGA-04-1362 | 7 | 100162524 | 100549959 | 2.33341772 | 5 | amp |
| TCGA-04-1362 | 7 | 100550124 | 100775372 | 1.661686172 | 4 | amp |
| TCGA-04-1362 | 7 | 100776954 | 101961003 | 2.181792458 | 5 | amp |
| TCGA-04-1362 | 7 | 101967609 | 102309466 | 1.720441506 | 4 | amp |
| TCGA-04-1362 | 7 | 102343839 | 103033559 | 1.039226347 | 3 | amp |
| TCGA-04-1362 | 7 | 104846347 | 106523662 | 1.109382272 | 3 | amp |
| TCGA-04-1362 | 7 | 116140257 | 123197898 | 1.164266142 | 3 | amp |
| TCGA-04-1362 | 7 | 123254549 | 127225260 | 1.392455549 | 4 | amp |
| TCGA-04-1362 | 7 | 127229086 | 127255634 | 2.304520449 | 5 | amp |
| TCGA-04-1362 | 7 | 127326621 | 127638092 | 1.085748572 | 3 | amp |
| TCGA-04-1362 | 7 | 127668682 | 128220160 | 2.36850739 | 5 | amp |
| TCGA-04-1362 | 7 | 128248159 | 128478139 | 1.764127918 | 4 | amp |
| TCGA-04-1362 | 7 | 128481170 | 128597398 | 3.457369349 | 5 | amp |
| TCGA-04-1362 | 7 | 128607329 | 128658230 | 1.338700574 | 4 | amp |
| TCGA-04-1362 | 7 | 128694614 | 128851663 | 2.807042007 | 5 | amp |
| TCGA-04-1362 | 7 | 129008303 | 129053541 | 1.365328767 | 4 | amp |
| TCGA-04-1362 | 7 | 129062669 | 129311385 | 2.624234731 | 5 | amp |
| TCGA-04-1362 | 7 | 129317414 | 129825239 | 1.585183169 | 4 | amp |
| TCGA-04-1362 | 7 | 129832442 | 130027878 | 2.322906711 | 5 | amp |
| TCGA-04-1362 | 7 | 130038655 | 132193458 | 1.576023856 | 4 | amp |
| TCGA-04-1362 | 7 | 132470349 | 133948781 | 0.957376381 | 3 | amp |
| TCGA-04-1362 | 7 | 133979497 | 134813739 | 1.388170581 | 4 | amp |
| TCGA-04-1362 | 7 | 134849142 | 134928179 | 2.203913987 | 5 | amp |

| | | | | | | |
|---|---|---|---|---|---|---|
| TCGA-04-1362 | 7 | 134930007 | 135073655 | 1.577100342 | 4 | amp |
| TCGA-04-1362 | 7 | 135078588 | 135299023 | 0.811948941 | 3 | amp |
| TCGA-04-1362 | 7 | 135300618 | 140483008 | 1.521063934 | 4 | amp |
| TCGA-04-1362 | 7 | 140487275 | 141795558 | 1.064898689 | 3 | amp |
| TCGA-04-1362 | 7 | 141796112 | 142562050 | 1.396302955 | 4 | amp |
| TCGA-04-1362 | 7 | 142562055 | 142643408 | 2.35677813 | 5 | amp |
| TCGA-04-1362 | 7 | 142649510 | 148486939 | 1.751425796 | 4 | amp |
| TCGA-04-1362 | 7 | 148487347 | 151836937 | 2.353566551 | 5 | amp |
| TCGA-04-1362 | 7 | 151841761 | 151875099 | 1.732146228 | 4 | amp |
| TCGA-04-1362 | 7 | 151876884 | 152055775 | 0.920025021 | 3 | amp |
| TCGA-04-1362 | 7 | 152142528 | 154264023 | 1.63064138 | 4 | amp |
| TCGA-04-1362 | 7 | 154379580 | 156469410 | 2.307744102 | 5 | amp |
| TCGA-04-1362 | 7 | 156476724 | 156626526 | 1.133106582 | 3 | amp |
| TCGA-04-1362 | 7 | 156629482 | 158935247 | 2.196378941 | 5 | amp |
| TCGA-04-1362 | 8 | 116074 | 1876824 | 1.863577015 | 4 | amp |
| TCGA-04-1362 | 8 | 1877410 | 2077227 | 2.869696907 | 5 | amp |
| TCGA-04-1362 | 8 | 2088607 | 10387232 | 1.408913282 | 4 | amp |
| TCGA-04-1362 | 8 | 10388749 | 10623468 | 2.757967017 | 5 | amp |
| TCGA-04-1362 | 8 | 10677680 | 21890747 | 1.227990329 | 3 | amp |
| TCGA-04-1362 | 8 | 21891517 | 22105565 | 2.949395605 | 5 | amp |
| TCGA-04-1362 | 8 | 22105578 | 22582472 | 1.573565984 | 4 | amp |
| TCGA-04-1362 | 8 | 22584650 | 22885310 | 2.826498919 | 5 | amp |
| TCGA-04-1362 | 8 | 22885769 | 27278277 | 1.192834571 | 3 | amp |
| TCGA-04-1362 | 8 | 27287550 | 27300505 | 2.780898782 | 5 | amp |
| TCGA-04-1362 | 8 | 27301695 | 33356168 | 1.223503092 | 3 | amp |
| TCGA-04-1362 | 8 | 33356652 | 33455042 | 2.905948529 | 5 | amp |
| TCGA-04-1362 | 8 | 33827032 | 36793474 | 1.40417033 | 4 | amp |
| TCGA-04-1362 | 8 | 37595433 | 38050319 | 3.227762963 | 5 | amp |
| TCGA-04-1362 | 8 | 38064976 | 38178699 | 2.055403284 | 4 | amp |
| TCGA-04-1362 | 8 | 38184190 | 38196144 | 2.816494593 | 5 | amp |
| TCGA-04-1362 | 8 | 38204991 | 38205711 | 1.642277228 | 4 | amp |
| TCGA-04-1362 | 8 | 38250063 | 38865513 | 3.556232936 | 5 | amp |
| TCGA-04-1362 | 8 | 38869145 | 39695736 | 1.649992915 | 4 | amp |
| TCGA-04-1362 | 8 | 39775381 | 39782325 | 3.324138977 | 5 | amp |
| TCGA-04-1362 | 8 | 39782695 | 41394815 | 1.697910729 | 4 | amp |
| TCGA-04-1362 | 8 | 41397155 | 41580751 | 4.006193568 | 5 | amp |
| TCGA-04-1362 | 8 | 41581011 | 42037947 | 1.953620456 | 4 | amp |
| TCGA-04-1362 | 8 | 42037955 | 42196232 | 4.641484243 | 5 | amp |
| TCGA-04-1362 | 8 | 42196498 | 48877322 | 1.912317195 | 4 | amp |
| TCGA-04-1362 | 8 | 48879864 | 48889351 | 2.764862662 | 5 | amp |
| TCGA-04-1362 | 8 | 48955516 | 54147674 | 1.421540188 | 4 | amp |
| TCGA-04-1362 | 8 | 54625396 | 54684757 | 3.18808091 | 5 | amp |
| TCGA-04-1362 | 8 | 54708152 | 67356688 | 1.86191951 | 4 | amp |
| TCGA-04-1362 | 8 | 67356785 | 67380605 | 2.844120425 | 5 | amp |
| TCGA-04-1362 | 8 | 67404492 | 67930037 | 2.01356129 | 4 | amp |
| TCGA-04-1362 | 8 | 67955449 | 68004137 | 2.844713043 | 5 | amp |
| TCGA-04-1362 | 8 | 68005766 | 70674163 | 1.586844649 | 4 | amp |
| TCGA-04-1362 | 8 | 70743974 | 71037131 | 2.821628357 | 5 | amp |
| TCGA-04-1362 | 8 | 71039044 | 86022052 | 1.446057938 | 4 | amp |
| TCGA-04-1362 | 8 | 86022348 | 86042343 | 3.041027633 | 5 | amp |

| | | | | | | |
|---|---|---|---|---|---|---|
| TCGA-04-1362 | 8 | 86043975 | 94722110 | 1.701102167 | 4 | amp |
| TCGA-04-1362 | 8 | 94730899 | 94776179 | 3.672135326 | 5 | amp |
| TCGA-04-1362 | 8 | 94777597 | 101237603 | 1.884307588 | 4 | amp |
| TCGA-04-1362 | 8 | 101243329 | 101253304 | 3.315738504 | 5 | amp |
| TCGA-04-1362 | 8 | 101270768 | 113349985 | 1.679960979 | 4 | amp |
| TCGA-04-1362 | 8 | 113353698 | 113403074 | 2.8089805 | 5 | amp |
| TCGA-04-1362 | 8 | 113418734 | 114290936 | 1.984671055 | 4 | amp |
| TCGA-04-1362 | 8 | 114326790 | 119945594 | 2.589670822 | 5 | amp |
| TCGA-04-1362 | 8 | 120079474 | 120118474 | 1.945626128 | 4 | amp |
| TCGA-04-1362 | 8 | 120233791 | 121019138 | 2.94014433 | 5 | amp |
| TCGA-04-1362 | 8 | 121021259 | 124154718 | 2.029897106 | 4 | amp |
| TCGA-04-1362 | 8 | 124156875 | 124787582 | 3.109644018 | 5 | amp |
| TCGA-04-1362 | 8 | 124789473 | 125074302 | 2.144933325 | 4 | amp |
| TCGA-04-1362 | 8 | 125076550 | 125082893 | 2.889190352 | 5 | amp |
| TCGA-04-1362 | 8 | 125083689 | 125551336 | 2.097363358 | 4 | amp |
| TCGA-04-1362 | 8 | 125551387 | 125990124 | 4.24193169 | 5 | amp |
| TCGA-04-1362 | 8 | 126015363 | 126194571 | 2.001436321 | 4 | amp |
| TCGA-04-1362 | 8 | 126369450 | 126448720 | 3.762009163 | 5 | amp |
| TCGA-04-1362 | 8 | 128098803 | 128098923 | 1.634178791 | 4 | amp |
| TCGA-04-1362 | 8 | 128428998 | 133584760 | 2.77118756 | 5 | amp |
| TCGA-04-1362 | 8 | 133595893 | 133673909 | 2.071863519 | 4 | amp |
| TCGA-04-1362 | 8 | 133687644 | 133790175 | 3.879655321 | 5 | amp |
| TCGA-04-1362 | 8 | 133806621 | 133858216 | 2.067125896 | 4 | amp |
| TCGA-04-1362 | 8 | 133879218 | 135812866 | 4.97094413 | 5 | amp |
| TCGA-04-1362 | 8 | 135817063 | 139153636 | 2.14963258 | 4 | amp |
| TCGA-04-1362 | 8 | 139155243 | 139631784 | 3.227643508 | 5 | amp |
| TCGA-04-1362 | 8 | 139635911 | 139649071 | 2.14253444 | 4 | amp |
| TCGA-04-1362 | 8 | 139658838 | 141696821 | 4.575701706 | 5 | amp |
| TCGA-04-1362 | 8 | 141710935 | 141900858 | 2.100421042 | 4 | amp |
| TCGA-04-1362 | 8 | 141902646 | 146279593 | 4.797733362 | 5 | amp |
| TCGA-04-1362 | 9 | 30053 | 2838526 | 1.139865795 | 3 | amp |
| TCGA-04-1362 | 9 | 27284665 | 32988163 | 0.968567509 | 3 | amp |
| TCGA-04-1362 | 9 | 32989553 | 34622121 | 1.438556752 | 4 | amp |
| TCGA-04-1362 | 9 | 34622284 | 34976247 | 2.5125427 | 5 | amp |
| TCGA-04-1362 | 9 | 34977009 | 35090685 | 1.937677558 | 4 | amp |
| TCGA-04-1362 | 9 | 35091190 | 35103128 | 2.483368267 | 5 | amp |
| TCGA-04-1362 | 9 | 35105616 | 35662279 | 1.439786039 | 4 | amp |
| TCGA-04-1362 | 9 | 35662473 | 35705857 | 2.315955592 | 5 | amp |
| TCGA-04-1362 | 9 | 35705913 | 36052347 | 1.867591025 | 4 | amp |
| TCGA-04-1362 | 9 | 36058770 | 37357288 | 1.036877797 | 3 | amp |
| TCGA-04-1362 | 9 | 37425894 | 71555753 | 1.384399197 | 4 | amp |
| TCGA-04-1362 | 9 | 71555948 | 71869327 | 2.324589691 | 5 | amp |
| TCGA-04-1362 | 9 | 71951119 | 86530537 | 1.546201562 | 4 | amp |
| TCGA-04-1362 | 9 | 86554421 | 86588317 | 2.37301678 | 5 | amp |
| TCGA-04-1362 | 9 | 86588792 | 88650585 | 1.450836611 | 4 | amp |
| TCGA-04-1362 | 9 | 88651222 | 88934601 | 2.267658502 | 5 | amp |
| TCGA-04-1362 | 9 | 88937194 | 89771714 | 1.390178817 | 4 | amp |
| TCGA-04-1362 | 9 | 90062762 | 91692846 | 2.415533179 | 5 | amp |
| TCGA-04-1362 | 9 | 91727415 | 91965776 | 1.729912072 | 4 | amp |
| TCGA-04-1362 | 9 | 91972282 | 93983300 | 2.717242544 | 5 | amp |

| | | | | | | |
|---|---|---|---|---|---|---|
| TCGA-04-1362 | 9 | 94058270 | 95285182 | 1.692521196 | 4 | amp |
| TCGA-04-1362 | 9 | 95298155 | 97090957 | 2.704172118 | 5 | amp |
| TCGA-04-1362 | 9 | 97177427 | 97221748 | 1.461754462 | 4 | amp |
| TCGA-04-1362 | 9 | 97321196 | 98279106 | 2.819862624 | 5 | amp |
| TCGA-04-1362 | 9 | 98774455 | 100139214 | 2.384721023 | 5 | amp |
| TCGA-04-1362 | 9 | 100190730 | 100249653 | 1.24292081 | 3 | amp |
| TCGA-04-1362 | 9 | 100257904 | 100425342 | 2.181329819 | 5 | amp |
| TCGA-04-1362 | 9 | 100426613 | 100449580 | 1.409295687 | 4 | amp |
| TCGA-04-1362 | 9 | 100451808 | 101830954 | 2.925101743 | 5 | amp |
| TCGA-04-1362 | 9 | 101831911 | 101911644 | 1.110067113 | 3 | amp |
| TCGA-04-1362 | 9 | 101980186 | 102595944 | 2.547457006 | 5 | amp |
| TCGA-04-1362 | 9 | 102606910 | 112004135 | 1.585991864 | 4 | amp |
| TCGA-04-1362 | 9 | 112005869 | 112189435 | 2.715419738 | 5 | amp |
| TCGA-04-1362 | 9 | 112190872 | 116034807 | 1.500722322 | 4 | amp |
| TCGA-04-1362 | 9 | 116038794 | 117119255 | 2.803364096 | 5 | amp |
| TCGA-04-1362 | 9 | 117120113 | 117396162 | 1.914163692 | 4 | amp |
| TCGA-04-1362 | 9 | 117399219 | 127316907 | 0.976248747 | 3 | amp |
| TCGA-04-1362 | 9 | 127454685 | 130982644 | 1.469267345 | 4 | amp |
| TCGA-04-1362 | 9 | 130984426 | 131039069 | 2.329096673 | 5 | amp |
| TCGA-04-1362 | 9 | 131046699 | 131255091 | 1.841851696 | 4 | amp |
| TCGA-04-1362 | 9 | 131256749 | 131378134 | 0.912272209 | 3 | amp |
| TCGA-04-1362 | 9 | 131379828 | 132897411 | 1.586854158 | 4 | amp |
| TCGA-04-1362 | 9 | 132963158 | 133995725 | 2.848054085 | 5 | amp |
| TCGA-04-1362 | 9 | 134002858 | 134090796 | 1.452932494 | 4 | amp |
| TCGA-04-1362 | 9 | 134098120 | 134759525 | 3.054645477 | 5 | amp |
| TCGA-04-1362 | 9 | 134769265 | 135262014 | 1.780618613 | 4 | amp |
| TCGA-04-1362 | 9 | 135263480 | 135523688 | 2.205714093 | 5 | amp |
| TCGA-04-1362 | 9 | 135523758 | 135564359 | 1.606670852 | 4 | amp |
| TCGA-04-1362 | 9 | 135602787 | 135763870 | 2.528397151 | 5 | amp |
| TCGA-04-1362 | 9 | 135765259 | 135804266 | 1.463406143 | 4 | amp |
| TCGA-04-1362 | 9 | 135862617 | 141071671 | 3.039843848 | 5 | amp |
| TCGA-04-1362 | X | 200797 | 34962937 | 1.358153552 | 4 | amp |
| TCGA-04-1362 | X | 35820260 | 35821340 | 0.827928928 | 3 | amp |
| TCGA-04-1362 | X | 35937860 | 155240134 | 1.623027404 | 4 | amp |
| TCGA-04-1362 | Y | 3447228 | 13496362 | 1.528356237 | 5 | amp |
| TCGA-04-1542 | 1 | 16834 | 12294431 | 0.921027834 | 4 | amp |
| TCGA-04-1542 | 1 | 12302518 | 14109451 | 0.745883979 | 3 | amp |
| TCGA-04-1542 | 1 | 14109634 | 17355282 | 0.932667607 | 4 | amp |
| TCGA-04-1542 | 1 | 17359537 | 19423790 | 1.07753992 | 5 | amp |
| TCGA-04-1542 | 1 | 19426040 | 19559649 | 0.717665481 | 3 | amp |
| TCGA-04-1542 | 1 | 19561564 | 35470922 | 0.901699457 | 4 | amp |
| TCGA-04-1542 | 1 | 35472505 | 36307397 | 0.747569551 | 3 | amp |
| TCGA-04-1542 | 1 | 36315748 | 39305420 | 0.884120524 | 4 | amp |
| TCGA-04-1542 | 1 | 39311564 | 39934465 | 0.71483947 | 3 | amp |
| TCGA-04-1542 | 1 | 39945455 | 56962405 | 0.864652067 | 4 | amp |
| TCGA-04-1542 | 1 | 56977585 | 93680531 | 0.776557699 | 3 | amp |
| TCGA-04-1542 | 1 | 107866833 | 109198323 | 0.950936291 | 4 | amp |
| TCGA-04-1542 | 1 | 117503837 | 145311992 | 1.132770373 | 5 | amp |
| TCGA-04-1542 | 1 | 145312643 | 145363064 | 0.759400392 | 3 | amp |
| TCGA-04-1542 | 1 | 145368382 | 153737599 | 1.087955499 | 5 | amp |

| | | | | | | |
|---|---|---|---|---|---|---|
| TCGA-04-1542 | 1 | 153740106 | 153964614 | 0.887589459 | 4 | amp |
| TCGA-04-1542 | 1 | 153965270 | 154241436 | 1.190287386 | 5 | amp |
| TCGA-04-1542 | 1 | 154242633 | 154941150 | 0.833701023 | 4 | amp |
| TCGA-04-1542 | 1 | 154941188 | 155300397 | 0.774015201 | 3 | amp |
| TCGA-04-1542 | 1 | 155307406 | 155322692 | 0.92525831 | 4 | amp |
| TCGA-04-1542 | 1 | 155324246 | 155990000 | 1.103081888 | 5 | amp |
| TCGA-04-1542 | 1 | 156006694 | 156438117 | 0.870300396 | 4 | amp |
| TCGA-04-1542 | 1 | 156438469 | 156535917 | 1.185192088 | 5 | amp |
| TCGA-04-1542 | 1 | 156536109 | 156844808 | 0.831781956 | 4 | amp |
| TCGA-04-1542 | 1 | 156845234 | 190068305 | 1.113341052 | 5 | amp |
| TCGA-04-1542 | 1 | 190129788 | 196288709 | 0.849184784 | 4 | amp |
| TCGA-04-1542 | 1 | 196295812 | 201019700 | 1.138042888 | 5 | amp |
| TCGA-04-1542 | 1 | 201020041 | 201789072 | 1.839794085 | 6 | amp |
| TCGA-04-1542 | 1 | 201816347 | 202245661 | 1.374719629 | 5 | amp |
| TCGA-04-1542 | 1 | 202249854 | 249152102 | 1.710307744 | 6 | amp |
| TCGA-04-1542 | 1 | 249208009 | 249231325 | 0.72851993 | 3 | amp |
| TCGA-04-1542 | 10 | 92880 | 5815914 | 1.329176048 | 5 | amp |
| TCGA-04-1542 | 10 | 5827050 | 5922359 | 0.930479343 | 4 | amp |
| TCGA-04-1542 | 10 | 5924929 | 15831341 | 1.311533354 | 5 | amp |
| TCGA-04-1542 | 10 | 15838044 | 16547208 | 1.069275702 | 4 | amp |
| TCGA-04-1542 | 10 | 16553029 | 16919115 | 1.205376387 | 5 | amp |
| TCGA-04-1542 | 10 | 16930387 | 16962168 | 1.437134412 | 6 | amp |
| TCGA-04-1542 | 10 | 16967211 | 18098544 | 1.292461852 | 5 | amp |
| TCGA-04-1542 | 10 | 18112004 | 18150433 | 1.467204124 | 6 | amp |
| TCGA-04-1542 | 10 | 18152416 | 26851366 | 1.240319934 | 5 | amp |
| TCGA-04-1542 | 10 | 26993547 | 27024268 | 0.96876791 | 4 | amp |
| TCGA-04-1542 | 10 | 27024391 | 27401097 | 1.222551483 | 5 | amp |
| TCGA-04-1542 | 10 | 27403432 | 27420888 | 1.074844149 | 4 | amp |
| TCGA-04-1542 | 10 | 27422949 | 28897417 | 1.168177661 | 5 | amp |
| TCGA-04-1542 | 10 | 28899568 | 30306888 | 1.416224958 | 6 | amp |
| TCGA-04-1542 | 10 | 30314984 | 34649212 | 1.263981745 | 5 | amp |
| TCGA-04-1542 | 10 | 34661384 | 34759221 | 1.049083184 | 4 | amp |
| TCGA-04-1542 | 10 | 34805876 | 43090183 | 1.286311323 | 5 | amp |
| TCGA-04-1542 | 10 | 43127334 | 43289489 | 1.466274237 | 6 | amp |
| TCGA-04-1542 | 10 | 43291880 | 69921557 | 1.249089271 | 5 | amp |
| TCGA-04-1542 | 10 | 69925396 | 70052000 | 1.059141465 | 4 | amp |
| TCGA-04-1542 | 10 | 70056011 | 72289095 | 1.291800515 | 5 | amp |
| TCGA-04-1542 | 10 | 72289584 | 72543174 | 1.416512751 | 6 | amp |
| TCGA-04-1542 | 10 | 72576532 | 72630913 | 1.177738063 | 5 | amp |
| TCGA-04-1542 | 10 | 72631548 | 72637141 | 0.934835918 | 4 | amp |
| TCGA-04-1542 | 10 | 72643665 | 75156348 | 1.292616375 | 5 | amp |
| TCGA-04-1542 | 10 | 75156916 | 75184962 | 1.064907056 | 4 | amp |
| TCGA-04-1542 | 10 | 75185521 | 75258966 | 0.846967226 | 3 | amp |
| TCGA-04-1542 | 10 | 75260354 | 75608881 | 1.052929073 | 4 | amp |
| TCGA-04-1542 | 10 | 75608994 | 88123895 | 1.273871228 | 5 | amp |
| TCGA-04-1542 | 10 | 88197272 | 88211925 | 0.918513582 | 4 | amp |
| TCGA-04-1542 | 10 | 88212944 | 93242838 | 1.232062735 | 5 | amp |
| TCGA-04-1542 | 10 | 93244217 | 93272200 | 1.482353918 | 6 | amp |
| TCGA-04-1542 | 10 | 93389673 | 94376601 | 1.251524505 | 5 | amp |
| TCGA-04-1542 | 10 | 94381125 | 94399663 | 1.560305619 | 6 | amp |

| | | | | | | |
|---|---|---|---|---|---|---|
| TCGA-04-1542 | 10 | 94405062 | 94837070 | 1.187663742 | 5 | amp |
| TCGA-04-1542 | 10 | 95066648 | 95157238 | 1.552831718 | 6 | amp |
| TCGA-04-1542 | 10 | 95159140 | 95266838 | 1.25634354 | 5 | amp |
| TCGA-04-1542 | 10 | 95275116 | 95351910 | 0.993462737 | 4 | amp |
| TCGA-04-1542 | 10 | 95353565 | 96109160 | 1.245748148 | 5 | amp |
| TCGA-04-1542 | 10 | 96109837 | 96122650 | 1.517639196 | 6 | amp |
| TCGA-04-1542 | 10 | 96201594 | 96306314 | 1.288599419 | 5 | amp |
| TCGA-04-1542 | 10 | 96313823 | 96495230 | 1.065672564 | 4 | amp |
| TCGA-04-1542 | 10 | 96522396 | 98790609 | 1.26858286 | 5 | amp |
| TCGA-04-1542 | 10 | 98794173 | 98985943 | 1.430464543 | 6 | amp |
| TCGA-04-1542 | 10 | 98988882 | 99025929 | 1.029649273 | 4 | amp |
| TCGA-04-1542 | 10 | 99116841 | 99161038 | 1.418539473 | 6 | amp |
| TCGA-04-1542 | 10 | 99190092 | 101421413 | 1.314230134 | 5 | amp |
| TCGA-04-1542 | 10 | 101439000 | 101474529 | 1.082663758 | 4 | amp |
| TCGA-04-1542 | 10 | 101476071 | 103361146 | 1.214386937 | 5 | amp |
| TCGA-04-1542 | 10 | 103368582 | 103384594 | 1.657782826 | 6 | amp |
| TCGA-04-1542 | 10 | 103427599 | 104122446 | 1.147369498 | 5 | amp |
| TCGA-04-1542 | 10 | 104122932 | 104156285 | 1.40761189 | 6 | amp |
| TCGA-04-1542 | 10 | 104156470 | 104233792 | 1.086651858 | 4 | amp |
| TCGA-04-1542 | 10 | 104235399 | 104850573 | 1.284805421 | 5 | amp |
| TCGA-04-1542 | 10 | 104850662 | 105074045 | 1.053251683 | 4 | amp |
| TCGA-04-1542 | 10 | 105086279 | 105792754 | 1.292518298 | 5 | amp |
| TCGA-04-1542 | 10 | 105793627 | 105824394 | 1.411384822 | 6 | amp |
| TCGA-04-1542 | 10 | 105830153 | 105927556 | 1.236529383 | 5 | amp |
| TCGA-04-1542 | 10 | 105928493 | 106124688 | 1.431651924 | 6 | amp |
| TCGA-04-1542 | 10 | 106125518 | 106983060 | 1.2654233 | 5 | amp |
| TCGA-04-1542 | 10 | 107005241 | 107022288 | 1.657808136 | 6 | amp |
| TCGA-04-1542 | 10 | 107023039 | 114182260 | 1.22209316 | 5 | amp |
| TCGA-04-1542 | 10 | 114185047 | 114220371 | 1.468998131 | 6 | amp |
| TCGA-04-1542 | 10 | 114224300 | 114911683 | 1.209124706 | 5 | amp |
| TCGA-04-1542 | 10 | 114912055 | 115526483 | 1.41695271 | 6 | amp |
| TCGA-04-1542 | 10 | 115527050 | 115618482 | 1.262430878 | 5 | amp |
| TCGA-04-1542 | 10 | 115636271 | 115982523 | 1.077260114 | 4 | amp |
| TCGA-04-1542 | 10 | 115985771 | 116595407 | 1.291854367 | 5 | amp |
| TCGA-04-1542 | 10 | 116595823 | 116615147 | 1.011712509 | 4 | amp |
| TCGA-04-1542 | 10 | 116620457 | 121286996 | 1.282894851 | 5 | amp |
| TCGA-04-1542 | 10 | 121335119 | 121342165 | 1.063070529 | 4 | amp |
| TCGA-04-1542 | 10 | 121347651 | 121583430 | 1.307107056 | 5 | amp |
| TCGA-04-1542 | 10 | 121586087 | 121692717 | 1.090873253 | 4 | amp |
| TCGA-04-1542 | 10 | 121693148 | 123683872 | 1.376474918 | 5 | amp |
| TCGA-04-1542 | 10 | 123718797 | 123722857 | 0.95583339 | 4 | amp |
| TCGA-04-1542 | 10 | 123724790 | 123987557 | 1.251212128 | 5 | amp |
| TCGA-04-1542 | 10 | 123988810 | 124605378 | 1.439062156 | 6 | amp |
| TCGA-04-1542 | 10 | 124608738 | 126495534 | 1.281641088 | 5 | amp |
| TCGA-04-1542 | 10 | 126505102 | 126672262 | 1.102425993 | 4 | amp |
| TCGA-04-1542 | 10 | 126673301 | 127529610 | 1.284562387 | 5 | amp |
| TCGA-04-1542 | 10 | 127529815 | 129878011 | 1.453599449 | 6 | amp |
| TCGA-04-1542 | 10 | 129881060 | 129899983 | 1.233823321 | 5 | amp |
| TCGA-04-1542 | 10 | 129900784 | 129914331 | 0.996278496 | 4 | amp |
| TCGA-04-1542 | 10 | 129914717 | 135516111 | 1.169321688 | 5 | amp |

| | | | | | | |
|---|---|---|---|---|---|---|
| TCGA-04-1542 | 11 | 86637 | 44265887 | 0.769754468 | 3 | amp |
| TCGA-04-1542 | 11 | 44288957 | 46515773 | 0.920072359 | 4 | amp |
| TCGA-04-1542 | 11 | 46529710 | 49831723 | 0.719467577 | 3 | amp |
| TCGA-04-1542 | 11 | 61323567 | 61323687 | 0.667033441 | 3 | amp |
| TCGA-04-1542 | 11 | 61487584 | 62434555 | 0.956670906 | 4 | amp |
| TCGA-04-1542 | 11 | 62434956 | 65651971 | 1.153478785 | 5 | amp |
| TCGA-04-1542 | 11 | 65652023 | 65961060 | 0.746284864 | 3 | amp |
| TCGA-04-1542 | 11 | 65977817 | 66003449 | 1.305125515 | 5 | amp |
| TCGA-04-1542 | 11 | 66006235 | 67431242 | 0.812071736 | 3 | amp |
| TCGA-04-1542 | 11 | 67431799 | 68204471 | 1.117720522 | 5 | amp |
| TCGA-04-1542 | 11 | 68305125 | 68682510 | 1.467688516 | 6 | amp |
| TCGA-04-1542 | 11 | 68685157 | 69957900 | 1.275470655 | 5 | amp |
| TCGA-04-1542 | 11 | 69972113 | 71847202 | 1.464581509 | 6 | amp |
| TCGA-04-1542 | 11 | 71849979 | 72005548 | 1.120812801 | 5 | amp |
| TCGA-04-1542 | 11 | 72005640 | 72114112 | 1.589821356 | 6 | amp |
| TCGA-04-1542 | 11 | 72141321 | 73122629 | 1.148844813 | 5 | amp |
| TCGA-04-1542 | 11 | 73130831 | 75113502 | 1.463873971 | 6 | amp |
| TCGA-04-1542 | 11 | 75115037 | 75599969 | 1.275275897 | 5 | amp |
| TCGA-04-1542 | 11 | 75622980 | 76687412 | 1.440770757 | 6 | amp |
| TCGA-04-1542 | 11 | 76696667 | 76917254 | 1.241524286 | 5 | amp |
| TCGA-04-1542 | 11 | 76922860 | 77103590 | 1.509259993 | 6 | amp |
| TCGA-04-1542 | 11 | 77300986 | 77918682 | 1.20682509 | 5 | amp |
| TCGA-04-1542 | 11 | 77919865 | 90017101 | 1.659659942 | 6 | amp |
| TCGA-04-1542 | 11 | 90602269 | 95521754 | 0.969654817 | 4 | amp |
| TCGA-04-1542 | 11 | 95532353 | 96125408 | 1.48597921 | 6 | amp |
| TCGA-04-1542 | 11 | 99715281 | 118378343 | 0.923145584 | 4 | amp |
| TCGA-04-1542 | 11 | 118379785 | 119215768 | 0.749752804 | 3 | amp |
| TCGA-04-1542 | 11 | 119216086 | 134252931 | 0.919860677 | 4 | amp |
| TCGA-04-1542 | 12 | 73256 | 38710900 | 1.542678612 | 6 | amp |
| TCGA-04-1542 | 12 | 38712041 | 133779395 | 0.689957399 | 3 | amp |
| TCGA-04-1542 | 13 | 19240876 | 61141836 | 0.684509557 | 3 | amp |
| TCGA-04-1542 | 13 | 61985357 | 110802810 | 0.961426051 | 4 | amp |
| TCGA-04-1542 | 13 | 110804646 | 111176577 | 1.154856415 | 5 | amp |
| TCGA-04-1542 | 13 | 111274487 | 113487375 | 0.938567442 | 4 | amp |
| TCGA-04-1542 | 13 | 113488848 | 115091796 | 0.713479768 | 3 | amp |
| TCGA-04-1542 | 14 | 19377543 | 20813647 | 0.984190205 | 4 | amp |
| TCGA-04-1542 | 14 | 20814986 | 20876614 | 1.112544675 | 5 | amp |
| TCGA-04-1542 | 14 | 20915328 | 22539511 | 0.937061731 | 4 | amp |
| TCGA-04-1542 | 14 | 22554694 | 23012212 | 1.164654637 | 5 | amp |
| TCGA-04-1542 | 14 | 23012987 | 24664232 | 0.937263536 | 4 | amp |
| TCGA-04-1542 | 14 | 24675121 | 24710523 | 1.08630468 | 5 | amp |
| TCGA-04-1542 | 14 | 24710871 | 24766135 | 0.912632041 | 4 | amp |
| TCGA-04-1542 | 14 | 24768117 | 24977472 | 0.785630305 | 3 | amp |
| TCGA-04-1542 | 14 | 25042779 | 25901337 | 0.91980105 | 4 | amp |
| TCGA-04-1542 | 14 | 26917147 | 31354832 | 0.720153487 | 3 | amp |
| TCGA-04-1542 | 14 | 64655226 | 70530652 | 0.704478572 | 3 | amp |
| TCGA-04-1542 | 14 | 89192833 | 91925149 | 0.71526282 | 3 | amp |
| TCGA-04-1542 | 14 | 92792206 | 96770945 | 0.709854502 | 3 | amp |
| TCGA-04-1542 | 14 | 100126559 | 100576100 | 0.762920665 | 3 | amp |
| TCGA-04-1542 | 14 | 101488345 | 103352608 | 0.723204323 | 3 | amp |

| | | | | | | |
|---|---|---|---|---|---|---|
| TCGA-04-1542 | 14 | 106452641 | 107283263 | 0.740971389 | 3 | amp |
| TCGA-04-1542 | 15 | 20169886 | 34158931 | 0.734786149 | 3 | amp |
| TCGA-04-1542 | 15 | 62967407 | 66774274 | 0.927177554 | 4 | amp |
| TCGA-04-1542 | 15 | 66777278 | 66808042 | 1.167707257 | 5 | amp |
| TCGA-04-1542 | 15 | 66811196 | 72932485 | 0.944467947 | 4 | amp |
| TCGA-04-1542 | 15 | 72947060 | 72987603 | 1.189667472 | 5 | amp |
| TCGA-04-1542 | 15 | 73001990 | 74363661 | 0.953368225 | 4 | amp |
| TCGA-04-1542 | 15 | 74363699 | 74373136 | 1.232515846 | 5 | amp |
| TCGA-04-1542 | 15 | 74374748 | 75541748 | 0.975157782 | 4 | amp |
| TCGA-04-1542 | 15 | 75550921 | 75586367 | 1.25326543 | 5 | amp |
| TCGA-04-1542 | 15 | 75586405 | 79264301 | 0.931102624 | 4 | amp |
| TCGA-04-1542 | 15 | 79265583 | 79606402 | 1.140305079 | 5 | amp |
| TCGA-04-1542 | 15 | 79614257 | 80472576 | 0.988889374 | 4 | amp |
| TCGA-04-1542 | 15 | 80473322 | 80886049 | 1.135442136 | 5 | amp |
| TCGA-04-1542 | 15 | 80889507 | 81199211 | 0.96179676 | 4 | amp |
| TCGA-04-1542 | 15 | 81201422 | 81241321 | 1.161911068 | 5 | amp |
| TCGA-04-1542 | 15 | 81271478 | 102500889 | 0.956157435 | 4 | amp |
| TCGA-04-1542 | 16 | 79275 | 90161052 | 1.303877966 | 5 | amp |
| TCGA-04-1542 | 16 | 90161507 | 90244214 | 1.049019615 | 4 | amp |
| TCGA-04-1542 | 17 | 1616948 | 1798405 | 0.687852247 | 3 | amp |
| TCGA-04-1542 | 17 | 3945642 | 4100889 | 0.697642684 | 3 | amp |
| TCGA-04-1542 | 17 | 5074867 | 5317511 | 0.687430838 | 3 | amp |
| TCGA-04-1542 | 17 | 7400958 | 7417216 | 0.717808838 | 3 | amp |
| TCGA-04-1542 | 17 | 7636398 | 7691602 | 0.748165287 | 3 | amp |
| TCGA-04-1542 | 17 | 7695190 | 7705032 | 0.90145798 | 4 | amp |
| TCGA-04-1542 | 17 | 7705162 | 7728069 | 0.721953092 | 3 | amp |
| TCGA-04-1542 | 17 | 8110855 | 8159774 | 0.692709694 | 3 | amp |
| TCGA-04-1542 | 17 | 8363282 | 8383928 | 0.771577742 | 3 | amp |
| TCGA-04-1542 | 17 | 8387399 | 8413318 | 0.88910237 | 4 | amp |
| TCGA-04-1542 | 17 | 8415751 | 10216102 | 0.746458943 | 3 | amp |
| TCGA-04-1542 | 17 | 10216452 | 10227639 | 0.88599642 | 4 | amp |
| TCGA-04-1542 | 17 | 10231130 | 10243741 | 0.735089975 | 3 | amp |
| TCGA-04-1542 | 17 | 10293725 | 10305160 | 0.858443399 | 4 | amp |
| TCGA-04-1542 | 17 | 10307594 | 10395928 | 0.695134091 | 3 | amp |
| TCGA-04-1542 | 17 | 10397658 | 10409432 | 0.854873886 | 4 | amp |
| TCGA-04-1542 | 17 | 10411147 | 10418258 | 0.679511413 | 3 | amp |
| TCGA-04-1542 | 17 | 10451015 | 15516076 | 0.70480654 | 3 | amp |
| TCGA-04-1542 | 17 | 16097769 | 17118411 | 0.692089589 | 3 | amp |
| TCGA-04-1542 | 17 | 18304198 | 18638781 | 0.723573116 | 3 | amp |
| TCGA-04-1542 | 17 | 18785848 | 19451491 | 0.663972719 | 3 | amp |
| TCGA-04-1542 | 17 | 19568250 | 26633197 | 0.682204772 | 3 | amp |
| TCGA-04-1542 | 17 | 26883499 | 26913213 | 0.675053624 | 3 | amp |
| TCGA-04-1542 | 17 | 27296752 | 27591617 | 0.691301077 | 3 | amp |
| TCGA-04-1542 | 17 | 31094591 | 31439129 | 0.687726791 | 3 | amp |
| TCGA-04-1542 | 17 | 33900663 | 34799213 | 0.719321799 | 3 | amp |
| TCGA-04-1542 | 17 | 34799529 | 34803874 | 0.891014775 | 4 | amp |
| TCGA-04-1542 | 17 | 34804291 | 34871886 | 0.669789473 | 3 | amp |
| TCGA-04-1542 | 17 | 35388886 | 35627756 | 0.676456839 | 3 | amp |
| TCGA-04-1542 | 17 | 36099410 | 36499695 | 0.716732786 | 3 | amp |
| TCGA-04-1542 | 17 | 38416738 | 39919583 | 0.697863478 | 3 | amp |

| | | | | | | |
|---|---|---|---|---|---|---|
| TCGA-04-1542 | 17 | 40842708 | 40861999 | 0.724427692 | 3 | amp |
| TCGA-04-1542 | 17 | 40946598 | 40986436 | 0.702636094 | 3 | amp |
| TCGA-04-1542 | 17 | 40986502 | 40990822 | 0.879494896 | 4 | amp |
| TCGA-04-1542 | 17 | 40990905 | 41103923 | 0.733036425 | 3 | amp |
| TCGA-04-1542 | 17 | 41346321 | 41355810 | 0.645042176 | 3 | amp |
| TCGA-04-1542 | 17 | 41362006 | 41369879 | 0.860316301 | 4 | amp |
| TCGA-04-1542 | 17 | 41477013 | 41582222 | 0.720213408 | 3 | amp |
| TCGA-04-1542 | 17 | 41930240 | 42162576 | 0.681834742 | 3 | amp |
| TCGA-04-1542 | 17 | 42928599 | 42940297 | 0.723557891 | 3 | amp |
| TCGA-04-1542 | 17 | 43664066 | 44116606 | 0.696813341 | 3 | amp |
| TCGA-04-1542 | 17 | 44399663 | 44414984 | 0.793503309 | 3 | amp |
| TCGA-04-1542 | 17 | 44415004 | 44503090 | 0.931101024 | 4 | amp |
| TCGA-04-1542 | 17 | 44534052 | 44623800 | 0.761879591 | 3 | amp |
| TCGA-04-1542 | 17 | 44625663 | 44806320 | 0.861137149 | 4 | amp |
| TCGA-04-1542 | 17 | 45206672 | 45690016 | 0.689018814 | 3 | amp |
| TCGA-04-1542 | 17 | 45992625 | 46257551 | 0.667775889 | 3 | amp |
| TCGA-04-1542 | 17 | 47041636 | 47123738 | 0.729289909 | 3 | amp |
| TCGA-04-1542 | 17 | 48627260 | 48777219 | 0.69977667 | 3 | amp |
| TCGA-04-1542 | 17 | 53026871 | 53398252 | 0.716939366 | 3 | amp |
| TCGA-04-1542 | 17 | 54925234 | 55607120 | 0.711982313 | 3 | amp |
| TCGA-04-1542 | 17 | 56270155 | 56332371 | 0.709108192 | 3 | amp |
| TCGA-04-1542 | 17 | 56638836 | 56801490 | 0.696781805 | 3 | amp |
| TCGA-04-1542 | 17 | 57109233 | 57742328 | 0.685104228 | 3 | amp |
| TCGA-04-1542 | 17 | 58072964 | 58145123 | 0.67331654 | 3 | amp |
| TCGA-04-1542 | 17 | 58756803 | 59001874 | 0.736117792 | 3 | amp |
| TCGA-04-1542 | 17 | 60003687 | 60050288 | 0.729791499 | 3 | amp |
| TCGA-04-1542 | 17 | 60308824 | 60518167 | 0.698529302 | 3 | amp |
| TCGA-04-1542 | 17 | 61886909 | 62506404 | 0.686486044 | 3 | amp |
| TCGA-04-1542 | 17 | 62882908 | 65083250 | 0.680981636 | 3 | amp |
| TCGA-04-1542 | 17 | 65336943 | 65822490 | 0.707350485 | 3 | amp |
| TCGA-04-1542 | 17 | 66533539 | 67016747 | 0.676414314 | 3 | amp |
| TCGA-04-1542 | 17 | 70118806 | 72709043 | 0.703780622 | 3 | amp |
| TCGA-04-1542 | 17 | 73204578 | 73328889 | 0.687632347 | 3 | amp |
| TCGA-04-1542 | 17 | 73647215 | 73700924 | 0.740961906 | 3 | amp |
| TCGA-04-1542 | 17 | 75085331 | 75196780 | 0.777027729 | 3 | amp |
| TCGA-04-1542 | 17 | 76490668 | 76705832 | 0.689409312 | 3 | amp |
| TCGA-04-1542 | 17 | 78263426 | 78728017 | 0.727726405 | 3 | amp |
| TCGA-04-1542 | 18 | 47273 | 14392975 | 1.886686201 | 6 | amp |
| TCGA-04-1542 | 18 | 14511753 | 29770124 | 1.347845757 | 5 | amp |
| TCGA-04-1542 | 18 | 29772570 | 30518074 | 1.806614212 | 6 | amp |
| TCGA-04-1542 | 18 | 30554457 | 77960823 | 1.002320747 | 4 | amp |
| TCGA-04-1542 | 19 | 12511822 | 12822312 | 0.97176461 | 4 | amp |
| TCGA-04-1542 | 19 | 12822336 | 13004501 | 1.261327069 | 5 | amp |
| TCGA-04-1542 | 19 | 13006750 | 14037900 | 1.69963251 | 6 | amp |
| TCGA-04-1542 | 19 | 14037942 | 14720948 | 1.384220044 | 5 | amp |
| TCGA-04-1542 | 19 | 14730203 | 15075211 | 1.778362954 | 6 | amp |
| TCGA-04-1542 | 19 | 15079111 | 15587423 | 1.402890361 | 5 | amp |
| TCGA-04-1542 | 19 | 15590061 | 16614213 | 1.806729638 | 6 | amp |
| TCGA-04-1542 | 19 | 16617488 | 16764950 | 1.361917914 | 5 | amp |
| TCGA-04-1542 | 19 | 16770171 | 21205700 | 1.667909384 | 6 | amp |

| | | | | | | |
|---|---|---|---|---|---|---|
| TCGA-04-1542 | 19 | 21216204 | 24345291 | 1.065120049 | 4 | amp |
| TCGA-04-1542 | 19 | 29698399 | 38202574 | 1.722863887 | 6 | amp |
| TCGA-04-1542 | 19 | 54722609 | 59110878 | 0.886778894 | 4 | amp |
| TCGA-04-1542 | 2 | 41527 | 27258938 | 0.971079992 | 4 | amp |
| TCGA-04-1542 | 2 | 27259317 | 27805416 | 0.848184459 | 3 | amp |
| TCGA-04-1542 | 2 | 27805934 | 37580124 | 0.990961937 | 4 | amp |
| TCGA-04-1542 | 2 | 37586681 | 39250398 | 0.83292944 | 3 | amp |
| TCGA-04-1542 | 2 | 39251094 | 74653715 | 0.954291225 | 4 | amp |
| TCGA-04-1542 | 2 | 74654287 | 74809007 | 0.85141042 | 3 | amp |
| TCGA-04-1542 | 2 | 74834145 | 92215830 | 1.013601282 | 4 | amp |
| TCGA-04-1542 | 2 | 92222503 | 100906920 | 1.234614284 | 5 | amp |
| TCGA-04-1542 | 2 | 100910649 | 116538630 | 0.989858041 | 4 | amp |
| TCGA-04-1542 | 2 | 116539891 | 116599973 | 1.241127383 | 5 | amp |
| TCGA-04-1542 | 2 | 118572335 | 121732712 | 1.626639181 | 6 | amp |
| TCGA-04-1542 | 2 | 121735934 | 121989592 | 1.251481016 | 5 | amp |
| TCGA-04-1542 | 2 | 121991628 | 122135269 | 1.481505081 | 6 | amp |
| TCGA-04-1542 | 2 | 122144690 | 152296650 | 1.234238671 | 5 | amp |
| TCGA-04-1542 | 2 | 152298401 | 152303134 | 1.739001703 | 6 | amp |
| TCGA-04-1542 | 2 | 152308109 | 162865181 | 1.255448687 | 5 | amp |
| TCGA-04-1542 | 2 | 162865717 | 162881450 | 1.560295071 | 6 | amp |
| TCGA-04-1542 | 2 | 162890046 | 168115899 | 1.248348599 | 5 | amp |
| TCGA-04-1542 | 2 | 168571053 | 168726579 | 1.682189799 | 6 | amp |
| TCGA-04-1542 | 2 | 168811982 | 170092583 | 1.267201475 | 5 | amp |
| TCGA-04-1542 | 2 | 170093584 | 170104076 | 1.564816025 | 6 | amp |
| TCGA-04-1542 | 2 | 170112560 | 206628708 | 1.220071424 | 5 | amp |
| TCGA-04-1542 | 2 | 206630185 | 206657043 | 1.60342368 | 6 | amp |
| TCGA-04-1542 | 2 | 206659419 | 220041058 | 1.23775196 | 5 | amp |
| TCGA-04-1542 | 2 | 220043636 | 220421490 | 1.010046989 | 4 | amp |
| TCGA-04-1542 | 2 | 220423902 | 227860252 | 1.229062131 | 5 | amp |
| TCGA-04-1542 | 2 | 227871962 | 227892731 | 1.562785925 | 6 | amp |
| TCGA-04-1542 | 2 | 227895116 | 238263619 | 1.236261754 | 5 | amp |
| TCGA-04-1542 | 2 | 238265917 | 238290149 | 1.537735543 | 6 | amp |
| TCGA-04-1542 | 2 | 238296135 | 242432457 | 1.221927859 | 5 | amp |
| TCGA-04-1542 | 2 | 242432663 | 243160772 | 1.049918523 | 4 | amp |
| TCGA-04-1542 | 20 | 68319 | 469431 | 1.364146532 | 5 | amp |
| TCGA-04-1542 | 20 | 470414 | 2637240 | 1.519242948 | 6 | amp |
| TCGA-04-1542 | 20 | 2637244 | 3218377 | 1.309302176 | 5 | amp |
| TCGA-04-1542 | 20 | 3233213 | 3619588 | 1.618191448 | 6 | amp |
| TCGA-04-1542 | 20 | 3624797 | 3897755 | 1.290257094 | 5 | amp |
| TCGA-04-1542 | 20 | 3898089 | 30371735 | 1.5463106 | 6 | amp |
| TCGA-04-1542 | 20 | 30380525 | 31436565 | 1.33197759 | 5 | amp |
| TCGA-04-1542 | 20 | 31571527 | 31680483 | 1.544588256 | 6 | amp |
| TCGA-04-1542 | 20 | 31682872 | 34262372 | 1.233680538 | 5 | amp |
| TCGA-04-1542 | 20 | 34262396 | 56886198 | 0.951417028 | 4 | amp |
| TCGA-04-1542 | 20 | 56918724 | 57287644 | 0.859815553 | 3 | amp |
| TCGA-04-1542 | 20 | 57876410 | 62926333 | 0.942028833 | 4 | amp |
| TCGA-04-1542 | 21 | 9483321 | 11098789 | 0.784190758 | 3 | amp |
| TCGA-04-1542 | 21 | 15554069 | 15873072 | 0.821283258 | 3 | amp |
| TCGA-04-1542 | 21 | 17763895 | 22658780 | 0.768092229 | 3 | amp |
| TCGA-04-1542 | 21 | 22906797 | 27327013 | 0.820938898 | 3 | amp |

| | | | | | | |
|---|---|---|---|---|---|---|
| TCGA-04-1542 | 21 | 27327884 | 33038856 | 0.966369839 | 4 | amp |
| TCGA-04-1542 | 21 | 33039509 | 33368290 | 1.187867169 | 5 | amp |
| TCGA-04-1542 | 21 | 33370807 | 34116086 | 1.006711605 | 4 | amp |
| TCGA-04-1542 | 21 | 34117043 | 34652259 | 0.763873827 | 3 | amp |
| TCGA-04-1542 | 21 | 34655352 | 34929661 | 0.908927896 | 4 | amp |
| TCGA-04-1542 | 21 | 34931488 | 35140134 | 0.825464629 | 3 | amp |
| TCGA-04-1542 | 21 | 35144335 | 37633062 | 1.015945052 | 4 | amp |
| TCGA-04-1542 | 21 | 37635835 | 37713890 | 0.778816133 | 3 | amp |
| TCGA-04-1542 | 21 | 37716820 | 40553810 | 0.976822973 | 4 | amp |
| TCGA-04-1542 | 21 | 40558907 | 40604216 | 0.77921723 | 3 | amp |
| TCGA-04-1542 | 21 | 40604241 | 43291786 | 0.974271674 | 4 | amp |
| TCGA-04-1542 | 21 | 43309222 | 43864758 | 1.125245138 | 5 | amp |
| TCGA-04-1542 | 21 | 43867085 | 48111215 | 0.942415725 | 4 | amp |
| TCGA-04-1542 | 22 | 16100468 | 42524383 | 0.665478725 | 3 | amp |
| TCGA-04-1542 | 3 | 361444 | 52387702 | 0.777266257 | 3 | amp |
| TCGA-04-1542 | 3 | 68782238 | 69079181 | 0.739139638 | 3 | amp |
| TCGA-04-1542 | 3 | 69082656 | 116163920 | 0.915781731 | 4 | amp |
| TCGA-04-1542 | 3 | 116746302 | 126155250 | 1.192144906 | 5 | amp |
| TCGA-04-1542 | 3 | 126160548 | 128349009 | 0.981132417 | 4 | amp |
| TCGA-04-1542 | 3 | 128350745 | 169566111 | 1.171215551 | 5 | amp |
| TCGA-04-1542 | 3 | 169569370 | 169656319 | 1.634596169 | 6 | amp |
| TCGA-04-1542 | 3 | 169693389 | 170736425 | 1.190306975 | 5 | amp |
| TCGA-04-1542 | 3 | 170744391 | 183862069 | 1.454746686 | 6 | amp |
| TCGA-04-1542 | 3 | 183862349 | 184556577 | 1.080944109 | 5 | amp |
| TCGA-04-1542 | 3 | 184557452 | 195346892 | 1.485611045 | 6 | amp |
| TCGA-04-1542 | 3 | 195377234 | 196771606 | 1.15700591 | 5 | amp |
| TCGA-04-1542 | 3 | 196778433 | 197955154 | 1.457613862 | 6 | amp |
| TCGA-04-1542 | 4 | 2673793 | 36085090 | 0.684701862 | 3 | amp |
| TCGA-04-1542 | 4 | 36093452 | 47548939 | 0.935111389 | 4 | amp |
| TCGA-04-1542 | 4 | 47556716 | 47597948 | 1.173169981 | 5 | amp |
| TCGA-04-1542 | 4 | 47602177 | 53752093 | 0.937628862 | 4 | amp |
| TCGA-04-1542 | 4 | 53773570 | 54440219 | 1.156336084 | 5 | amp |
| TCGA-04-1542 | 4 | 54852745 | 185039102 | 0.692170147 | 3 | amp |
| TCGA-04-1542 | 4 | 185045309 | 185350234 | 0.967029883 | 4 | amp |
| TCGA-04-1542 | 4 | 185550390 | 186244997 | 1.079228871 | 5 | amp |
| TCGA-04-1542 | 4 | 186253710 | 186274817 | 1.666022306 | 6 | amp |
| TCGA-04-1542 | 4 | 186278797 | 186339952 | 1.094181625 | 5 | amp |
| TCGA-04-1542 | 4 | 186343617 | 186583364 | 1.407668226 | 6 | amp |
| TCGA-04-1542 | 4 | 186997753 | 187077368 | 1.205363994 | 5 | amp |
| TCGA-04-1542 | 4 | 187078675 | 187179390 | 1.41857044 | 6 | amp |
| TCGA-04-1542 | 4 | 187188257 | 187510419 | 1.181103061 | 5 | amp |
| TCGA-04-1542 | 4 | 187516791 | 187584789 | 1.454422862 | 6 | amp |
| TCGA-04-1542 | 4 | 187627668 | 190948390 | 1.191054686 | 5 | amp |
| TCGA-04-1542 | 5 | 151610 | 45645738 | 1.231770906 | 5 | amp |
| TCGA-04-1542 | 5 | 49695055 | 79443147 | 0.66263968 | 3 | amp |
| TCGA-04-1542 | 5 | 80756837 | 86627104 | 0.655736411 | 3 | amp |
| TCGA-04-1542 | 5 | 123966326 | 169021343 | 0.72080376 | 3 | amp |
| TCGA-04-1542 | 5 | 169021356 | 175751815 | 0.919826554 | 4 | amp |
| TCGA-04-1542 | 5 | 175763680 | 180899507 | 0.797675679 | 3 | amp |
| TCGA-04-1542 | 6 | 105907 | 407617 | 1.102074863 | 5 | amp |

| | | | | | | |
|---|---|---|---|---|---|---|
| TCGA-04-1542 | 6 | 486627 | 599272 | 1.554875859 | 6 | amp |
| TCGA-04-1542 | 6 | 610077 | 2749568 | 1.139224971 | 5 | amp |
| TCGA-04-1542 | 6 | 2768900 | 4037837 | 0.992250933 | 4 | amp |
| TCGA-04-1542 | 6 | 4040963 | 28097767 | 1.14442831 | 5 | amp |
| TCGA-04-1542 | 6 | 28116123 | 28121854 | 1.578491889 | 6 | amp |
| TCGA-04-1542 | 6 | 28194802 | 29589093 | 1.195736288 | 5 | amp |
| TCGA-04-1542 | 6 | 29589471 | 30073041 | 1.004988974 | 4 | amp |
| TCGA-04-1542 | 6 | 30075827 | 30113878 | 1.29243734 | 5 | amp |
| TCGA-04-1542 | 6 | 30114797 | 30126410 | 1.537199594 | 6 | amp |
| TCGA-04-1542 | 6 | 30126914 | 30570262 | 1.147401195 | 5 | amp |
| TCGA-04-1542 | 6 | 30570964 | 30572558 | 1.513913211 | 6 | amp |
| TCGA-04-1542 | 6 | 30572760 | 30624574 | 1.205176514 | 5 | amp |
| TCGA-04-1542 | 6 | 30624648 | 30899367 | 0.932006433 | 4 | amp |
| TCGA-04-1542 | 6 | 30899467 | 31644935 | 1.132301428 | 5 | amp |
| TCGA-04-1542 | 6 | 31646811 | 31656113 | 1.499980914 | 6 | amp |
| TCGA-04-1542 | 6 | 31656441 | 31747972 | 1.17205823 | 5 | amp |
| TCGA-04-1542 | 6 | 31748131 | 32125515 | 1.01501043 | 4 | amp |
| TCGA-04-1542 | 6 | 32125598 | 33147598 | 1.172124129 | 5 | amp |
| TCGA-04-1542 | 6 | 33147981 | 33424346 | 1.030429733 | 4 | amp |
| TCGA-04-1542 | 6 | 33541571 | 33740599 | 1.269277326 | 5 | amp |
| TCGA-04-1542 | 6 | 33744721 | 90276815 | 0.960869934 | 4 | amp |
| TCGA-04-1542 | 6 | 90305623 | 90406305 | 1.130974864 | 5 | amp |
| TCGA-04-1542 | 6 | 90408518 | 114265628 | 1.029239389 | 4 | amp |
| TCGA-04-1542 | 6 | 114266519 | 116906074 | 0.812255456 | 3 | amp |
| TCGA-04-1542 | 6 | 117681476 | 117737510 | 0.884750352 | 4 | amp |
| TCGA-04-1542 | 6 | 119228552 | 119301528 | 0.837248576 | 3 | amp |
| TCGA-04-1542 | 6 | 123100915 | 126080965 | 0.820763032 | 3 | amp |
| TCGA-04-1542 | 6 | 127794420 | 129775486 | 0.779881337 | 3 | amp |
| TCGA-04-1542 | 6 | 131921119 | 131949229 | 0.819775866 | 3 | amp |
| TCGA-04-1542 | 6 | 132006971 | 132039892 | 1.111787839 | 5 | amp |
| TCGA-04-1542 | 6 | 132041415 | 132649262 | 0.692481347 | 3 | amp |
| TCGA-04-1542 | 6 | 132649523 | 132796826 | 0.868257905 | 4 | amp |
| TCGA-04-1542 | 6 | 132824566 | 136880035 | 0.749878791 | 3 | amp |
| TCGA-04-1542 | 6 | 137482795 | 139095045 | 0.867058427 | 4 | amp |
| TCGA-04-1542 | 6 | 139207936 | 144768460 | 0.786944898 | 3 | amp |
| TCGA-04-1542 | 6 | 148792549 | 151665149 | 0.796541217 | 3 | amp |
| TCGA-04-1542 | 6 | 152415461 | 152473304 | 0.835586037 | 3 | amp |
| TCGA-04-1542 | 6 | 152554886 | 152694382 | 0.883784889 | 4 | amp |
| TCGA-04-1542 | 6 | 152751235 | 152787224 | 0.860948743 | 3 | amp |
| TCGA-04-1542 | 6 | 153078163 | 155099216 | 0.802375801 | 3 | amp |
| TCGA-04-1542 | 6 | 155450354 | 160199895 | 0.837561555 | 3 | amp |
| TCGA-04-1542 | 6 | 160206920 | 160885351 | 0.82212728 | 3 | amp |
| TCGA-04-1542 | 6 | 161507597 | 162622343 | 0.863153427 | 3 | amp |
| TCGA-04-1542 | 6 | 165695095 | 168264524 | 0.853243257 | 3 | amp |
| TCGA-04-1542 | 6 | 168314770 | 171055029 | 0.842620707 | 3 | amp |
| TCGA-04-1542 | 7 | 540695 | 2415212 | 1.167839407 | 5 | amp |
| TCGA-04-1542 | 7 | 2418272 | 2771399 | 0.984575792 | 4 | amp |
| TCGA-04-1542 | 7 | 2793390 | 5952589 | 1.228760136 | 5 | amp |
| TCGA-04-1542 | 7 | 5958454 | 5997718 | 1.452693965 | 6 | amp |
| TCGA-04-1542 | 7 | 5998606 | 6786871 | 1.149965509 | 5 | amp |

| | | | | | | |
|---|---|---|---|---|---|---|
| TCGA-04-1542 | 7 | 6790831 | 6841195 | 1.467031359 | 6 | amp |
| TCGA-04-1542 | 7 | 6844507 | 16899290 | 1.274984443 | 5 | amp |
| TCGA-04-1542 | 7 | 16900105 | 17382736 | 1.420862063 | 6 | amp |
| TCGA-04-1542 | 7 | 17833612 | 25268109 | 1.255424163 | 5 | amp |
| TCGA-04-1542 | 7 | 25989512 | 27582772 | 1.080510174 | 4 | amp |
| TCGA-04-1542 | 7 | 27668930 | 30639673 | 1.260615401 | 5 | amp |
| TCGA-04-1542 | 7 | 30640625 | 30962294 | 1.434110004 | 6 | amp |
| TCGA-04-1542 | 7 | 30963004 | 38768401 | 1.23843266 | 5 | amp |
| TCGA-04-1542 | 7 | 38781532 | 38813899 | 1.043025331 | 4 | amp |
| TCGA-04-1542 | 7 | 38816237 | 45699831 | 1.254814146 | 5 | amp |
| TCGA-04-1542 | 7 | 45701615 | 47924285 | 1.415255685 | 6 | amp |
| TCGA-04-1542 | 7 | 47925197 | 50537879 | 1.281522361 | 5 | amp |
| TCGA-04-1542 | 7 | 50544271 | 55273370 | 1.447368627 | 6 | amp |
| TCGA-04-1542 | 7 | 55459424 | 72337038 | 1.231464563 | 5 | amp |
| TCGA-04-1542 | 7 | 72338213 | 72473625 | 1.09103871 | 4 | amp |
| TCGA-04-1542 | 7 | 72479912 | 72877460 | 1.180941985 | 5 | amp |
| TCGA-04-1542 | 7 | 72879757 | 72892949 | 1.563209855 | 6 | amp |
| TCGA-04-1542 | 7 | 72903502 | 72922848 | 1.175682445 | 5 | amp |
| TCGA-04-1542 | 7 | 72924973 | 73151469 | 1.09449021 | 4 | amp |
| TCGA-04-1542 | 7 | 73151515 | 75055749 | 1.268616386 | 5 | amp |
| TCGA-04-1542 | 7 | 75066758 | 75178303 | 1.085153127 | 4 | amp |
| TCGA-04-1542 | 7 | 75182669 | 86804006 | 1.249272934 | 5 | amp |
| TCGA-04-1542 | 7 | 86808789 | 86827368 | 1.000465135 | 4 | amp |
| TCGA-04-1542 | 7 | 86828287 | 98956060 | 1.251765352 | 5 | amp |
| TCGA-04-1542 | 7 | 98957144 | 99077459 | 1.083757444 | 4 | amp |
| TCGA-04-1542 | 7 | 99079744 | 99277543 | 1.272311816 | 5 | amp |
| TCGA-04-1542 | 7 | 99282793 | 99381728 | 1.080132027 | 4 | amp |
| TCGA-04-1542 | 7 | 99425683 | 99662592 | 1.232661188 | 5 | amp |
| TCGA-04-1542 | 7 | 99668730 | 100304904 | 1.028135459 | 4 | amp |
| TCGA-04-1542 | 7 | 100319103 | 100361543 | 1.276117393 | 5 | amp |
| TCGA-04-1542 | 7 | 100361614 | 100395401 | 1.437426713 | 6 | amp |
| TCGA-04-1542 | 7 | 100401027 | 100410833 | 1.272005907 | 5 | amp |
| TCGA-04-1542 | 7 | 100411215 | 100692309 | 1.032232138 | 4 | amp |
| TCGA-04-1542 | 7 | 100692543 | 100780764 | 1.291472955 | 5 | amp |
| TCGA-04-1542 | 7 | 100799843 | 100880873 | 1.029760852 | 4 | amp |
| TCGA-04-1542 | 7 | 100883415 | 102964137 | 1.252143299 | 5 | amp |
| TCGA-04-1542 | 7 | 102964908 | 103030974 | 1.417233499 | 6 | amp |
| TCGA-04-1542 | 7 | 103032035 | 104681494 | 1.203537158 | 5 | amp |
| TCGA-04-1542 | 7 | 104702577 | 104753805 | 1.04511436 | 4 | amp |
| TCGA-04-1542 | 7 | 104758256 | 107324000 | 1.228460133 | 5 | amp |
| TCGA-04-1542 | 7 | 107329451 | 107688574 | 1.101536308 | 4 | amp |
| TCGA-04-1542 | 7 | 107689753 | 117423055 | 1.232390505 | 5 | amp |
| TCGA-04-1542 | 7 | 117424286 | 117877031 | 1.483616463 | 6 | amp |
| TCGA-04-1542 | 7 | 117879928 | 121549056 | 1.219621323 | 5 | amp |
| TCGA-04-1542 | 7 | 121568182 | 121624215 | 1.564447377 | 6 | amp |
| TCGA-04-1542 | 7 | 121636407 | 135292176 | 1.212074226 | 5 | amp |
| TCGA-04-1542 | 7 | 135298903 | 135323487 | 1.430562214 | 6 | amp |
| TCGA-04-1542 | 7 | 135327902 | 141311100 | 1.27293145 | 5 | amp |
| TCGA-04-1542 | 7 | 141313893 | 141352775 | 1.458259614 | 6 | amp |
| TCGA-04-1542 | 7 | 141362433 | 143573751 | 1.308015616 | 5 | amp |

| | | | | | | |
|---|---|---|---|---|---|---|
| TCGA-04-1542 | 7 | 143632322 | 143966376 | 1.433801556 | 6 | amp |
| TCGA-04-1542 | 7 | 143969513 | 148769619 | 1.346642584 | 5 | amp |
| TCGA-04-1542 | 7 | 148771433 | 149495244 | 1.058959905 | 4 | amp |
| TCGA-04-1542 | 7 | 149502465 | 150939987 | 0.861344183 | 3 | amp |
| TCGA-04-1542 | 7 | 150940705 | 151877244 | 1.025983218 | 4 | amp |
| TCGA-04-1542 | 7 | 151877788 | 151904568 | 0.843134834 | 3 | amp |
| TCGA-04-1542 | 7 | 151917593 | 154143415 | 0.992866578 | 4 | amp |
| TCGA-04-1542 | 7 | 154172012 | 154735926 | 1.238307257 | 5 | amp |
| TCGA-04-1542 | 7 | 154738044 | 154754219 | 1.46658223 | 6 | amp |
| TCGA-04-1542 | 7 | 154755368 | 157000632 | 1.241146512 | 5 | amp |
| TCGA-04-1542 | 7 | 157009552 | 158935247 | 1.422463007 | 6 | amp |
| TCGA-04-1542 | 8 | 116074 | 41364661 | 1.015448436 | 4 | amp |
| TCGA-04-1542 | 8 | 41387709 | 42868567 | 1.561126663 | 6 | amp |
| TCGA-04-1542 | 8 | 42873450 | 43152279 | 1.329278753 | 5 | amp |
| TCGA-04-1542 | 8 | 43152395 | 67988826 | 1.673019973 | 6 | amp |
| TCGA-04-1542 | 8 | 67998233 | 68208875 | 1.385361929 | 5 | amp |
| TCGA-04-1542 | 8 | 68211517 | 86114496 | 1.585221183 | 6 | amp |
| TCGA-04-1542 | 8 | 86115289 | 87424114 | 1.375947539 | 5 | amp |
| TCGA-04-1542 | 8 | 87437439 | 144351748 | 1.620953068 | 6 | amp |
| TCGA-04-1542 | 8 | 144391601 | 146279593 | 1.080528317 | 4 | amp |
| TCGA-04-1542 | 9 | 30053 | 3228927 | 0.707839492 | 3 | amp |
| TCGA-04-1542 | 9 | 3855976 | 4722685 | 0.695959623 | 3 | amp |
| TCGA-04-1542 | 9 | 5122942 | 5335603 | 0.746629266 | 3 | amp |
| TCGA-04-1542 | 9 | 6255947 | 6880096 | 0.69690631 | 3 | amp |
| TCGA-04-1542 | 9 | 12821301 | 15226022 | 0.674458202 | 3 | amp |
| TCGA-04-1542 | 9 | 15506530 | 17226291 | 0.687688366 | 3 | amp |
| TCGA-04-1542 | 9 | 18657632 | 18929091 | 0.703354702 | 3 | amp |
| TCGA-04-1542 | 9 | 21140975 | 27169670 | 0.690680284 | 3 | amp |
| TCGA-04-1542 | 9 | 27206564 | 32467964 | 0.692191117 | 3 | amp |
| TCGA-04-1542 | 9 | 32988043 | 34512438 | 0.691178138 | 3 | amp |
| TCGA-04-1542 | 9 | 37425894 | 37762141 | 0.676550812 | 3 | amp |
| TCGA-04-1542 | 9 | 39266887 | 40610153 | 0.721767077 | 3 | amp |
| TCGA-04-1542 | 9 | 40700293 | 40706439 | 0.9189224 | 4 | amp |
| TCGA-04-1542 | 9 | 40715839 | 43187316 | 0.783518048 | 3 | amp |
| TCGA-04-1542 | 9 | 43609409 | 43630727 | 0.930013785 | 4 | amp |
| TCGA-04-1542 | 9 | 43709645 | 44172279 | 0.694108034 | 3 | amp |
| TCGA-04-1542 | 9 | 44172891 | 45376921 | 0.954104381 | 4 | amp |
| TCGA-04-1542 | 9 | 46744168 | 66401226 | 0.842240845 | 3 | amp |
| TCGA-04-1542 | 9 | 66513704 | 67047362 | 0.867896601 | 4 | amp |
| TCGA-04-1542 | 9 | 67047650 | 69113804 | 0.715843613 | 3 | amp |
| TCGA-04-1542 | 9 | 69117791 | 69262530 | 0.894365955 | 4 | amp |
| TCGA-04-1542 | 9 | 69367613 | 69718865 | 0.749642613 | 3 | amp |
| TCGA-04-1542 | 9 | 69719673 | 70086499 | 0.87605879 | 4 | amp |
| TCGA-04-1542 | 9 | 70144088 | 70428931 | 0.765602108 | 3 | amp |
| TCGA-04-1542 | 9 | 70432421 | 70457175 | 0.912735947 | 4 | amp |
| TCGA-04-1542 | 9 | 70462992 | 70734062 | 0.755447496 | 3 | amp |
| TCGA-04-1542 | 9 | 70856904 | 70912760 | 0.895501176 | 4 | amp |
| TCGA-04-1542 | 9 | 70913824 | 70919342 | 0.754661282 | 3 | amp |
| TCGA-04-1542 | 9 | 71555948 | 72347281 | 0.697496475 | 3 | amp |
| TCGA-04-1542 | 9 | 73002656 | 74300770 | 0.718446587 | 3 | amp |

| | | | | | | |
|---|---|---|---|---|---|---|
| TCGA-04-1542 | 9 | 74825586 | 75369810 | 0.691942976 | 3 | amp |
| TCGA-04-1542 | 9 | 75543752 | 77401095 | 0.680770418 | 3 | amp |
| TCGA-04-1542 | 9 | 77697926 | 78722276 | 0.711530518 | 3 | amp |
| TCGA-04-1542 | 9 | 78842409 | 79827974 | 0.685452443 | 3 | amp |
| TCGA-04-1542 | 9 | 79946917 | 84531638 | 0.693952761 | 3 | amp |
| TCGA-04-1542 | 9 | 84562615 | 86243919 | 0.745498892 | 3 | amp |
| TCGA-04-1542 | 9 | 87325508 | 88270146 | 0.707654693 | 3 | amp |
| TCGA-04-1542 | 9 | 88618469 | 88934601 | 0.700109958 | 3 | amp |
| TCGA-04-1542 | 9 | 88967554 | 90747970 | 0.726988064 | 3 | amp |
| TCGA-04-1542 | 9 | 91266863 | 94797188 | 0.693480564 | 3 | amp |
| TCGA-04-1542 | 9 | 94841785 | 95108114 | 0.692929303 | 3 | amp |
| TCGA-04-1542 | 9 | 95256337 | 98242890 | 0.704964605 | 3 | amp |
| TCGA-04-1542 | 9 | 98740299 | 99122529 | 0.708191092 | 3 | amp |
| TCGA-04-1542 | 9 | 99614145 | 100139214 | 0.715153414 | 3 | amp |
| TCGA-04-1542 | 9 | 100257904 | 100700438 | 0.689266209 | 3 | amp |
| TCGA-04-1542 | 9 | 100890854 | 101829376 | 0.708950836 | 3 | amp |
| TCGA-04-1542 | 9 | 104356651 | 106878555 | 0.689881046 | 3 | amp |
| TCGA-04-1542 | 9 | 107484980 | 108098031 | 0.694575248 | 3 | amp |
| TCGA-04-1542 | 9 | 108110595 | 108127961 | 0.879367281 | 4 | amp |
| TCGA-04-1542 | 9 | 108128590 | 111698772 | 0.691553008 | 3 | amp |
| TCGA-04-1542 | 9 | 111936778 | 113563286 | 0.69857088 | 3 | amp |
| TCGA-04-1542 | 9 | 115919979 | 117386744 | 0.716227792 | 3 | amp |
| TCGA-04-1542 | 9 | 117821934 | 123374865 | 0.692623714 | 3 | amp |
| TCGA-04-1542 | 9 | 123737012 | 123780145 | 0.714894286 | 3 | amp |
| TCGA-04-1542 | 9 | 124544573 | 125282370 | 0.686737221 | 3 | amp |
| TCGA-04-1542 | 9 | 130116113 | 130442538 | 0.703819041 | 3 | amp |
| TCGA-04-1542 | 9 | 131741513 | 131768702 | 0.707536731 | 3 | amp |
| TCGA-04-1542 | 9 | 133989924 | 134008604 | 0.765217483 | 3 | amp |
| TCGA-04-1542 | X | 41025115 | 41031270 | 0.65951975 | 3 | amp |
| TCGA-04-1542 | X | 134852665 | 134872360 | 0.656045559 | 3 | amp |
| TCGA-04-1542 | X | 134887196 | 134928978 | 0.676198008 | 3 | amp |
| TCGA-04-1542 | Y | 4966220 | 28680670 | 0.879589061 | 4 | amp |
| TCGA-09-0365 | 1 | 14642 | 741285 | 1.608656646 | 5 | amp |
| TCGA-09-0365 | 1 | 745438 | 7731153 | 2.537781688 | 6 | amp |
| TCGA-09-0365 | 1 | 7737605 | 8716467 | 1.659895824 | 5 | amp |
| TCGA-09-0365 | 1 | 8921363 | 9801352 | 2.122027205 | 6 | amp |
| TCGA-09-0365 | 1 | 9803884 | 10460659 | 1.348935535 | 5 | amp |
| TCGA-09-0365 | 1 | 10463100 | 11187253 | 2.157763737 | 6 | amp |
| TCGA-09-0365 | 1 | 11187651 | 11294380 | 1.389176983 | 5 | amp |
| TCGA-09-0365 | 1 | 11297882 | 12254110 | 2.383104099 | 6 | amp |
| TCGA-09-0365 | 1 | 12254597 | 14113075 | 1.40710377 | 5 | amp |
| TCGA-09-0365 | 1 | 14142861 | 15871105 | 2.188838208 | 6 | amp |
| TCGA-09-0365 | 1 | 15873253 | 15988250 | 1.684672817 | 5 | amp |
| TCGA-09-0365 | 1 | 16042724 | 16875383 | 2.407851724 | 6 | amp |
| TCGA-09-0365 | 1 | 16890379 | 16918548 | 1.16504962 | 4 | amp |
| TCGA-09-0365 | 1 | 16945531 | 19421579 | 2.326651339 | 6 | amp |
| TCGA-09-0365 | 1 | 19422032 | 19559649 | 1.303256484 | 5 | amp |
| TCGA-09-0365 | 1 | 19561564 | 21050779 | 2.132684226 | 6 | amp |
| TCGA-09-0365 | 1 | 21071256 | 21329280 | 1.280712907 | 5 | amp |
| TCGA-09-0365 | 1 | 21546415 | 21738157 | 2.358513783 | 6 | amp |

| | | | | | | |
|---|---|---|---|---|---|---|
| TCGA-09-0365 | 1 | 21751054 | 21809937 | 1.344543559 | 5 | amp |
| TCGA-09-0365 | 1 | 21880514 | 22013767 | 2.536384331 | 6 | amp |
| TCGA-09-0365 | 1 | 22016409 | 22084321 | 1.120585596 | 4 | amp |
| TCGA-09-0365 | 1 | 22138896 | 22336393 | 2.69132351 | 6 | amp |
| TCGA-09-0365 | 1 | 22338921 | 22848972 | 1.30378026 | 5 | amp |
| TCGA-09-0365 | 1 | 22850703 | 23111586 | 2.119656739 | 6 | amp |
| TCGA-09-0365 | 1 | 23189487 | 23693717 | 1.457097371 | 5 | amp |
| TCGA-09-0365 | 1 | 23694391 | 24696366 | 2.242187308 | 6 | amp |
| TCGA-09-0365 | 1 | 24700155 | 25812314 | 1.641089065 | 5 | amp |
| TCGA-09-0365 | 1 | 25815611 | 27736708 | 2.229270453 | 6 | amp |
| TCGA-09-0365 | 1 | 27739023 | 27755455 | 1.196767317 | 5 | amp |
| TCGA-09-0365 | 1 | 27873772 | 28071363 | 2.465040675 | 6 | amp |
| TCGA-09-0365 | 1 | 28075561 | 28823113 | 1.608213844 | 5 | amp |
| TCGA-09-0365 | 1 | 28833829 | 28939875 | 2.066216114 | 6 | amp |
| TCGA-09-0365 | 1 | 28944413 | 29481434 | 1.524775999 | 5 | amp |
| TCGA-09-0365 | 1 | 29485881 | 31351648 | 2.18190601 | 6 | amp |
| TCGA-09-0365 | 1 | 31406003 | 31842437 | 1.483145961 | 5 | amp |
| TCGA-09-0365 | 1 | 31845734 | 32280962 | 2.702162544 | 6 | amp |
| TCGA-09-0365 | 1 | 32374446 | 32625167 | 1.633502609 | 5 | amp |
| TCGA-09-0365 | 1 | 32626199 | 32860069 | 2.262476877 | 6 | amp |
| TCGA-09-0365 | 1 | 33058523 | 33160765 | 1.528371539 | 5 | amp |
| TCGA-09-0365 | 1 | 33233322 | 34035180 | 1.974871686 | 6 | amp |
| TCGA-09-0365 | 1 | 34037108 | 36521351 | 1.470194547 | 5 | amp |
| TCGA-09-0365 | 1 | 36550450 | 36645913 | 2.352952439 | 6 | amp |
| TCGA-09-0365 | 1 | 36748112 | 36762397 | 1.291231543 | 5 | amp |
| TCGA-09-0365 | 1 | 36766435 | 38298015 | 2.142180092 | 6 | amp |
| TCGA-09-0365 | 1 | 38300698 | 38453357 | 1.685280498 | 5 | amp |
| TCGA-09-0365 | 1 | 38453421 | 39361750 | 2.105599997 | 6 | amp |
| TCGA-09-0365 | 1 | 39376989 | 40318574 | 1.443382947 | 5 | amp |
| TCGA-09-0365 | 1 | 40319630 | 40434448 | 2.183438761 | 6 | amp |
| TCGA-09-0365 | 1 | 40435118 | 40882050 | 1.500794685 | 5 | amp |
| TCGA-09-0365 | 1 | 40882429 | 41494446 | 2.062263321 | 6 | amp |
| TCGA-09-0365 | 1 | 41499565 | 43166688 | 1.523196283 | 5 | amp |
| TCGA-09-0365 | 1 | 43203851 | 43638622 | 2.150040272 | 6 | amp |
| TCGA-09-0365 | 1 | 43647182 | 43675755 | 1.183851825 | 5 | amp |
| TCGA-09-0365 | 1 | 43738363 | 45316708 | 2.193138018 | 6 | amp |
| TCGA-09-0365 | 1 | 45323367 | 46827559 | 1.551386717 | 5 | amp |
| TCGA-09-0365 | 1 | 46870632 | 47080755 | 2.092002269 | 6 | amp |
| TCGA-09-0365 | 1 | 47101424 | 55555416 | 1.302869664 | 5 | amp |
| TCGA-09-0365 | 1 | 55557687 | 109566175 | 0.862872802 | 3 | amp |
| TCGA-09-0365 | 1 | 109607109 | 113456829 | 1.361764822 | 5 | amp |
| TCGA-09-0365 | 1 | 113459722 | 118584725 | 0.948901983 | 3 | amp |
| TCGA-09-0365 | 1 | 118596620 | 145368622 | 1.284624255 | 5 | amp |
| TCGA-09-0365 | 1 | 145414739 | 145646203 | 2.738205798 | 6 | amp |
| TCGA-09-0365 | 1 | 145650451 | 147083721 | 1.354121406 | 5 | amp |
| TCGA-09-0365 | 1 | 147084629 | 147416221 | 2.130749645 | 6 | amp |
| TCGA-09-0365 | 1 | 147425511 | 148888263 | 1.251075922 | 5 | amp |
| TCGA-09-0365 | 1 | 148889575 | 150598318 | 2.342569837 | 6 | amp |
| TCGA-09-0365 | 1 | 150598908 | 150801755 | 1.669515703 | 5 | amp |
| TCGA-09-0365 | 1 | 150801956 | 151372253 | 2.562453353 | 6 | amp |

| | | | | | | |
|---|---|---|---|---|---|---|
| TCGA-09-0365 | 1 | 151372409 | 151413602 | 1.664379862 | 5 | amp |
| TCGA-09-0365 | 1 | 151414498 | 152944374 | 2.434916902 | 6 | amp |
| TCGA-09-0365 | 1 | 152944402 | 153177511 | 1.44333432 | 5 | amp |
| TCGA-09-0365 | 1 | 153270400 | 153995805 | 2.84145091 | 6 | amp |
| TCGA-09-0365 | 1 | 153997984 | 154130215 | 1.534162493 | 5 | amp |
| TCGA-09-0365 | 1 | 154131394 | 155308210 | 2.931969031 | 6 | amp |
| TCGA-09-0365 | 1 | 155309048 | 155491340 | 1.710845157 | 5 | amp |
| TCGA-09-0365 | 1 | 155560722 | 157494387 | 2.752610051 | 6 | amp |
| TCGA-09-0365 | 1 | 157497365 | 157773947 | 1.563458428 | 5 | amp |
| TCGA-09-0365 | 1 | 157776841 | 158326721 | 2.244914454 | 6 | amp |
| TCGA-09-0365 | 1 | 158368280 | 159043338 | 1.448751034 | 5 | amp |
| TCGA-09-0365 | 1 | 159159551 | 159176285 | 2.598176369 | 6 | amp |
| TCGA-09-0365 | 1 | 159272091 | 159683981 | 1.483885788 | 5 | amp |
| TCGA-09-0365 | 1 | 159684184 | 160456580 | 2.443123025 | 6 | amp |
| TCGA-09-0365 | 1 | 160456869 | 160849212 | 1.600238263 | 5 | amp |
| TCGA-09-0365 | 1 | 160850287 | 161569651 | 2.538187096 | 6 | amp |
| TCGA-09-0365 | 1 | 161576026 | 165860570 | 1.6868793 | 5 | amp |
| TCGA-09-0365 | 1 | 165865347 | 167097877 | 2.213648427 | 6 | amp |
| TCGA-09-0365 | 1 | 167301653 | 167385071 | 1.526443323 | 5 | amp |
| TCGA-09-0365 | 1 | 167400860 | 167805782 | 2.03657724 | 6 | amp |
| TCGA-09-0365 | 1 | 167806431 | 174927094 | 1.452454503 | 5 | amp |
| TCGA-09-0365 | 1 | 174973709 | 175088008 | 2.238560032 | 6 | amp |
| TCGA-09-0365 | 1 | 175092457 | 179019516 | 1.543915166 | 5 | amp |
| TCGA-09-0365 | 1 | 179023628 | 179102505 | 2.244056492 | 6 | amp |
| TCGA-09-0365 | 1 | 179112063 | 180080333 | 1.47573028 | 5 | amp |
| TCGA-09-0365 | 1 | 180135585 | 180257694 | 2.340414704 | 6 | amp |
| TCGA-09-0365 | 1 | 180283781 | 180833046 | 1.325829054 | 5 | amp |
| TCGA-09-0365 | 1 | 180842888 | 182369629 | 2.109760341 | 6 | amp |
| TCGA-09-0365 | 1 | 182544469 | 200827214 | 1.373591271 | 5 | amp |
| TCGA-09-0365 | 1 | 200867388 | 202318304 | 2.652018419 | 6 | amp |
| TCGA-09-0365 | 1 | 202385898 | 202544316 | 1.673624613 | 5 | amp |
| TCGA-09-0365 | 1 | 202549554 | 202715480 | 2.154726214 | 6 | amp |
| TCGA-09-0365 | 1 | 202718049 | 202746243 | 1.531092043 | 5 | amp |
| TCGA-09-0365 | 1 | 202777781 | 203652549 | 2.626929455 | 6 | amp |
| TCGA-09-0365 | 1 | 203667263 | 203787877 | 1.578286863 | 5 | amp |
| TCGA-09-0365 | 1 | 203797401 | 205042898 | 2.444313934 | 6 | amp |
| TCGA-09-0365 | 1 | 205052644 | 205156940 | 1.587868659 | 5 | amp |
| TCGA-09-0365 | 1 | 205238034 | 207244623 | 2.24903338 | 6 | amp |
| TCGA-09-0365 | 1 | 207244825 | 207975300 | 1.626813676 | 5 | amp |
| TCGA-09-0365 | 1 | 207975767 | 209950884 | 2.521310023 | 6 | amp |
| TCGA-09-0365 | 1 | 209951426 | 212553427 | 1.599291149 | 5 | amp |
| TCGA-09-0365 | 1 | 212558541 | 212870414 | 2.278686884 | 6 | amp |
| TCGA-09-0365 | 1 | 212911708 | 223168335 | 1.463501098 | 5 | amp |
| TCGA-09-0365 | 1 | 223175690 | 223949980 | 2.09141024 | 6 | amp |
| TCGA-09-0365 | 1 | 223951820 | 226016641 | 1.577119109 | 5 | amp |
| TCGA-09-0365 | 1 | 226019449 | 227182722 | 2.420138245 | 6 | amp |
| TCGA-09-0365 | 1 | 227192603 | 227843562 | 1.334748265 | 5 | amp |
| TCGA-09-0365 | 1 | 227922314 | 229431685 | 2.876357949 | 6 | amp |
| TCGA-09-0365 | 1 | 229433185 | 230821026 | 1.707696093 | 5 | amp |
| TCGA-09-0365 | 1 | 230822659 | 230991528 | 2.110296352 | 6 | amp |

| | | | | | | |
|---|---|---|---|---|---|---|
| TCGA-09-0365 | 1 | 231042617 | 234603441 | 1.689925197 | 5 | amp |
| TCGA-09-0365 | 1 | 234606922 | 235324414 | 2.128180669 | 6 | amp |
| TCGA-09-0365 | 1 | 235324417 | 247079750 | 1.560139864 | 5 | amp |
| TCGA-09-0365 | 1 | 247081491 | 247729306 | 2.305342055 | 6 | amp |
| TCGA-09-0365 | 1 | 247737384 | 248814188 | 1.563322158 | 5 | amp |
| TCGA-09-0365 | 1 | 248844657 | 249231325 | 2.526132024 | 6 | amp |
| TCGA-09-0365 | 10 | 92880 | 298468 | 1.419999029 | 5 | amp |
| TCGA-09-0365 | 10 | 323180 | 5005738 | 2.030121964 | 6 | amp |
| TCGA-09-0365 | 10 | 5005980 | 5254693 | 1.316432483 | 5 | amp |
| TCGA-09-0365 | 10 | 5254919 | 5693370 | 2.022687176 | 6 | amp |
| TCGA-09-0365 | 10 | 5762558 | 5842674 | 1.427830196 | 5 | amp |
| TCGA-09-0365 | 10 | 5920021 | 7788671 | 1.87093458 | 6 | amp |
| TCGA-09-0365 | 10 | 7791103 | 7816883 | 1.200827881 | 5 | amp |
| TCGA-09-0365 | 10 | 7817677 | 7866581 | 1.809588585 | 6 | amp |
| TCGA-09-0365 | 10 | 8005848 | 8019304 | 1.203237801 | 5 | amp |
| TCGA-09-0365 | 10 | 8050984 | 11963361 | 1.781832128 | 6 | amp |
| TCGA-09-0365 | 10 | 11971803 | 12071553 | 1.159409325 | 4 | amp |
| TCGA-09-0365 | 10 | 12076990 | 12185214 | 1.834544173 | 6 | amp |
| TCGA-09-0365 | 10 | 12191539 | 43319264 | 1.376778116 | 5 | amp |
| TCGA-09-0365 | 10 | 43325669 | 43623758 | 2.20487477 | 6 | amp |
| TCGA-09-0365 | 10 | 43650567 | 43679023 | 1.2284664 | 5 | amp |
| TCGA-09-0365 | 10 | 43691613 | 45959790 | 1.998164873 | 6 | amp |
| TCGA-09-0365 | 10 | 45984779 | 46673730 | 1.36845459 | 5 | amp |
| TCGA-09-0365 | 10 | 46675443 | 47241968 | 1.883274494 | 6 | amp |
| TCGA-09-0365 | 10 | 47379708 | 47420476 | 1.266748193 | 5 | amp |
| TCGA-09-0365 | 10 | 47708444 | 47901471 | 1.784532718 | 6 | amp |
| TCGA-09-0365 | 10 | 47903122 | 48255499 | 1.339036371 | 5 | amp |
| TCGA-09-0365 | 10 | 48259458 | 48739478 | 1.917958866 | 6 | amp |
| TCGA-09-0365 | 10 | 48751794 | 50599954 | 1.483763642 | 5 | amp |
| TCGA-09-0365 | 10 | 50666830 | 50732293 | 1.059380969 | 4 | amp |
| TCGA-09-0365 | 10 | 50732404 | 51065547 | 1.887883381 | 6 | amp |
| TCGA-09-0365 | 10 | 51069611 | 51613324 | 1.479580708 | 5 | amp |
| TCGA-09-0365 | 10 | 51620292 | 51949363 | 1.770848526 | 6 | amp |
| TCGA-09-0365 | 10 | 51952382 | 52103922 | 1.335705919 | 5 | amp |
| TCGA-09-0365 | 10 | 52436801 | 52502782 | 2.329184647 | 6 | amp |
| TCGA-09-0365 | 10 | 52504841 | 55755586 | 1.261489529 | 5 | amp |
| TCGA-09-0365 | 10 | 55779943 | 69970247 | 1.050486176 | 4 | amp |
| TCGA-09-0365 | 10 | 70043899 | 70706426 | 1.246560054 | 5 | amp |
| TCGA-09-0365 | 10 | 70715964 | 70765699 | 1.798439808 | 6 | amp |
| TCGA-09-0365 | 10 | 70768578 | 70918004 | 1.304431725 | 5 | amp |
| TCGA-09-0365 | 10 | 70922154 | 71921702 | 1.889744517 | 6 | amp |
| TCGA-09-0365 | 10 | 71962866 | 71974358 | 1.199343311 | 5 | amp |
| TCGA-09-0365 | 10 | 71977507 | 73832343 | 2.145000492 | 6 | amp |
| TCGA-09-0365 | 10 | 73856999 | 73921483 | 1.075429865 | 4 | amp |
| TCGA-09-0365 | 10 | 73950121 | 74135645 | 1.841746038 | 6 | amp |
| TCGA-09-0365 | 10 | 74167680 | 75391923 | 1.148402981 | 4 | amp |
| TCGA-09-0365 | 10 | 75393620 | 75676354 | 1.996108701 | 6 | amp |
| TCGA-09-0365 | 10 | 75802815 | 76790845 | 1.146171823 | 4 | amp |
| TCGA-09-0365 | 10 | 76797504 | 76868960 | 1.985074794 | 6 | amp |
| TCGA-09-0365 | 10 | 76910275 | 79011043 | 1.380486201 | 5 | amp |

| | | | | | | |
|---|---|---|---|---|---|---|
| TCGA-09-0365 | 10 | 79163550 | 79740084 | 1.910827652 | 6 | amp |
| TCGA-09-0365 | 10 | 79741131 | 79785568 | 1.319977991 | 5 | amp |
| TCGA-09-0365 | 10 | 79785769 | 84118679 | 1.87157398 | 6 | amp |
| TCGA-09-0365 | 10 | 84498309 | 84745407 | 1.208101403 | 5 | amp |
| TCGA-09-0365 | 10 | 85901200 | 86018449 | 1.954164698 | 6 | amp |
| TCGA-09-0365 | 10 | 86130796 | 88260527 | 1.360841491 | 5 | amp |
| TCGA-09-0365 | 10 | 88277279 | 88492748 | 2.256050402 | 6 | amp |
| TCGA-09-0365 | 10 | 88635748 | 88950460 | 1.412483749 | 5 | amp |
| TCGA-09-0365 | 10 | 88971862 | 89125623 | 1.861768953 | 6 | amp |
| TCGA-09-0365 | 10 | 89125868 | 89272989 | 1.39818539 | 5 | amp |
| TCGA-09-0365 | 10 | 89280859 | 94774077 | 1.044540633 | 4 | amp |
| TCGA-09-0365 | 10 | 94816676 | 95385428 | 1.361065553 | 5 | amp |
| TCGA-09-0365 | 10 | 95386368 | 96979757 | 1.081622156 | 4 | amp |
| TCGA-09-0365 | 10 | 96997624 | 98416742 | 1.311248343 | 5 | amp |
| TCGA-09-0365 | 10 | 98469261 | 98985943 | 2.012740615 | 6 | amp |
| TCGA-09-0365 | 10 | 98988882 | 99025929 | 1.174268883 | 4 | amp |
| TCGA-09-0365 | 10 | 99116841 | 99771114 | 1.991559663 | 6 | amp |
| TCGA-09-0365 | 10 | 99967863 | 99995970 | 1.123786111 | 4 | amp |
| TCGA-09-0365 | 10 | 100003821 | 100221619 | 1.988219427 | 6 | amp |
| TCGA-09-0365 | 10 | 100242315 | 101961935 | 1.40298998 | 5 | amp |
| TCGA-09-0365 | 10 | 101964218 | 102677111 | 1.782953866 | 6 | amp |
| TCGA-09-0365 | 10 | 102678109 | 102719278 | 1.375705947 | 5 | amp |
| TCGA-09-0365 | 10 | 102721591 | 102987586 | 2.353266322 | 6 | amp |
| TCGA-09-0365 | 10 | 103174362 | 103310656 | 1.242468176 | 5 | amp |
| TCGA-09-0365 | 10 | 103339121 | 103609710 | 1.934597645 | 6 | amp |
| TCGA-09-0365 | 10 | 103649117 | 103769816 | 1.139721941 | 4 | amp |
| TCGA-09-0365 | 10 | 103771449 | 103912347 | 1.943179106 | 6 | amp |
| TCGA-09-0365 | 10 | 103916743 | 104122446 | 1.44286925 | 5 | amp |
| TCGA-09-0365 | 10 | 104122932 | 104636811 | 1.909374215 | 6 | amp |
| TCGA-09-0365 | 10 | 104638081 | 105074045 | 1.311402732 | 5 | amp |
| TCGA-09-0365 | 10 | 105086279 | 105648925 | 1.784612316 | 6 | amp |
| TCGA-09-0365 | 10 | 105651828 | 105781494 | 1.172671991 | 4 | amp |
| TCGA-09-0365 | 10 | 105785284 | 105824394 | 1.809839612 | 6 | amp |
| TCGA-09-0365 | 10 | 105830153 | 105974220 | 1.188747058 | 5 | amp |
| TCGA-09-0365 | 10 | 105978533 | 106039238 | 1.84767051 | 6 | amp |
| TCGA-09-0365 | 10 | 106057328 | 115618482 | 1.23530898 | 5 | amp |
| TCGA-09-0365 | 10 | 115636271 | 115982523 | 0.932805794 | 4 | amp |
| TCGA-09-0365 | 10 | 115985771 | 115987064 | 1.406705559 | 5 | amp |
| TCGA-09-0365 | 10 | 115987631 | 116100645 | 1.942934065 | 6 | amp |
| TCGA-09-0365 | 10 | 116196015 | 116595407 | 1.212815714 | 5 | amp |
| TCGA-09-0365 | 10 | 116595823 | 117704349 | 0.901753146 | 4 | amp |
| TCGA-09-0365 | 10 | 117823861 | 120900852 | 1.419310872 | 5 | amp |
| TCGA-09-0365 | 10 | 120905686 | 121285646 | 1.87388429 | 6 | amp |
| TCGA-09-0365 | 10 | 121286756 | 128776291 | 1.423257433 | 5 | amp |
| TCGA-09-0365 | 10 | 128780149 | 135516111 | 2.058485127 | 6 | amp |
| TCGA-09-0365 | 11 | 86637 | 3700980 | 1.695180406 | 5 | amp |
| TCGA-09-0365 | 11 | 3704370 | 3803369 | 0.796301304 | 4 | amp |
| TCGA-09-0365 | 11 | 3832421 | 4360362 | 1.462369845 | 5 | amp |
| TCGA-09-0365 | 11 | 4388572 | 5411658 | 0.996191457 | 4 | amp |
| TCGA-09-0365 | 11 | 5443427 | 6226941 | 1.185635469 | 5 | amp |

| | | | | | | |
|---|---|---|---|---|---|---|
| TCGA-09-0365 | 11 | 6231028 | 6503482 | 1.867342777 | 6 | amp |
| TCGA-09-0365 | 11 | 6519368 | 6541387 | 0.944372407 | 4 | amp |
| TCGA-09-0365 | 11 | 6587785 | 6704560 | 1.840496877 | 6 | amp |
| TCGA-09-0365 | 11 | 6789232 | 7509681 | 0.919969383 | 4 | amp |
| TCGA-09-0365 | 11 | 7530603 | 9082132 | 1.36257049 | 5 | amp |
| TCGA-09-0365 | 11 | 9087422 | 10215551 | 0.971135398 | 4 | amp |
| TCGA-09-0365 | 11 | 10327206 | 11643142 | 1.211025269 | 5 | amp |
| TCGA-09-0365 | 11 | 11985971 | 12549493 | 1.390952608 | 5 | amp |
| TCGA-09-0365 | 11 | 12785752 | 14907512 | 1.009821319 | 4 | amp |
| TCGA-09-0365 | 11 | 14989200 | 15267641 | 1.432226311 | 5 | amp |
| TCGA-09-0365 | 11 | 15994326 | 17337037 | 0.964175528 | 4 | amp |
| TCGA-09-0365 | 11 | 17351638 | 18308317 | 1.487234845 | 5 | amp |
| TCGA-09-0365 | 11 | 18309009 | 18638538 | 1.030640747 | 4 | amp |
| TCGA-09-0365 | 11 | 18655684 | 18765769 | 1.797831027 | 6 | amp |
| TCGA-09-0365 | 11 | 18770927 | 20907097 | 1.245147838 | 5 | amp |
| TCGA-09-0365 | 11 | 20939703 | 31805126 | 0.88531018 | 3 | amp |
| TCGA-09-0365 | 11 | 31811453 | 32623397 | 1.402654286 | 5 | amp |
| TCGA-09-0365 | 11 | 32623764 | 33566978 | 0.937655615 | 4 | amp |
| TCGA-09-0365 | 11 | 33569307 | 34664285 | 1.192316778 | 5 | amp |
| TCGA-09-0365 | 11 | 34667898 | 43876805 | 0.984477256 | 4 | amp |
| TCGA-09-0365 | 11 | 43904181 | 44265887 | 1.327837367 | 5 | amp |
| TCGA-09-0365 | 11 | 44288957 | 45967697 | 1.801545448 | 6 | amp |
| TCGA-09-0365 | 11 | 45970376 | 46105803 | 0.926222644 | 4 | amp |
| TCGA-09-0365 | 11 | 46321479 | 46431931 | 1.758077575 | 5 | amp |
| TCGA-09-0365 | 11 | 46439412 | 46693903 | 1.170496355 | 4 | amp |
| TCGA-09-0365 | 11 | 46694192 | 46751140 | 1.689031531 | 5 | amp |
| TCGA-09-0365 | 11 | 46760572 | 46890730 | 0.847639953 | 4 | amp |
| TCGA-09-0365 | 11 | 46893069 | 47810167 | 1.417635758 | 5 | amp |
| TCGA-09-0365 | 11 | 47813421 | 50252738 | 0.95685136 | 4 | amp |
| TCGA-09-0365 | 11 | 51411441 | 56237991 | 0.796000998 | 3 | amp |
| TCGA-09-0365 | 11 | 56257898 | 56786270 | 0.959222303 | 4 | amp |
| TCGA-09-0365 | 11 | 56949312 | 57574480 | 1.709453049 | 5 | amp |
| TCGA-09-0365 | 11 | 57575580 | 58604926 | 1.007775268 | 4 | amp |
| TCGA-09-0365 | 11 | 58605727 | 59597047 | 1.415744882 | 5 | amp |
| TCGA-09-0365 | 11 | 59599100 | 60198452 | 1.002474298 | 4 | amp |
| TCGA-09-0365 | 11 | 60199851 | 60482610 | 1.390846064 | 5 | amp |
| TCGA-09-0365 | 11 | 60482744 | 61919460 | 1.835659066 | 6 | amp |
| TCGA-09-0365 | 11 | 61957723 | 62066514 | 1.075169878 | 4 | amp |
| TCGA-09-0365 | 11 | 62123754 | 62760818 | 1.823907616 | 6 | amp |
| TCGA-09-0365 | 11 | 62760847 | 62782443 | 1.368583428 | 5 | amp |
| TCGA-09-0365 | 11 | 62931210 | 63177393 | 0.735619324 | 3 | amp |
| TCGA-09-0365 | 11 | 63230930 | 63585621 | 1.389777322 | 5 | amp |
| TCGA-09-0365 | 11 | 63585642 | 67829520 | 1.804769073 | 6 | amp |
| TCGA-09-0365 | 11 | 67831984 | 67957583 | 1.113250429 | 4 | amp |
| TCGA-09-0365 | 11 | 68029089 | 68216587 | 1.74356754 | 5 | amp |
| TCGA-09-0365 | 11 | 68305125 | 68377551 | 0.942239231 | 4 | amp |
| TCGA-09-0365 | 11 | 68380499 | 71548644 | 1.626990377 | 5 | amp |
| TCGA-09-0365 | 11 | 71567291 | 71712644 | 1.155479611 | 4 | amp |
| TCGA-09-0365 | 11 | 71712778 | 72552022 | 1.694282079 | 5 | amp |
| TCGA-09-0365 | 11 | 72552443 | 72946371 | 1.077076026 | 4 | amp |

| | | | | | | |
|---|---|---|---|---|---|---|
| TCGA-09-0365 | 11 | 73007486 | 73105830 | 1.769085588 | 6 | amp |
| TCGA-09-0365 | 11 | 73106102 | 73718144 | 1.354676903 | 5 | amp |
| TCGA-09-0365 | 11 | 73745587 | 74015505 | 0.970811705 | 4 | amp |
| TCGA-09-0365 | 11 | 74047687 | 76175174 | 1.355956666 | 5 | amp |
| TCGA-09-0365 | 11 | 76183565 | 76731404 | 0.941613579 | 4 | amp |
| TCGA-09-0365 | 11 | 76750546 | 76928406 | 1.906465045 | 6 | amp |
| TCGA-09-0365 | 11 | 76938874 | 77580875 | 0.918685684 | 4 | amp |
| TCGA-09-0365 | 11 | 77583140 | 82895948 | 1.227736607 | 5 | amp |
| TCGA-09-0365 | 11 | 82905681 | 86126364 | 0.966295935 | 4 | amp |
| TCGA-09-0365 | 11 | 86130882 | 86778919 | 1.418091133 | 5 | amp |
| TCGA-09-0365 | 11 | 86782550 | 89487143 | 0.848015055 | 3 | amp |
| TCGA-09-0365 | 11 | 89531247 | 92919038 | 1.098149119 | 4 | amp |
| TCGA-09-0365 | 11 | 93065379 | 94134459 | 1.192772041 | 5 | amp |
| TCGA-09-0365 | 11 | 94153258 | 103031776 | 0.974604271 | 4 | amp |
| TCGA-09-0365 | 11 | 103033756 | 108332310 | 0.860165988 | 3 | amp |
| TCGA-09-0365 | 11 | 108345496 | 110697364 | 0.996548615 | 4 | amp |
| TCGA-09-0365 | 11 | 111152071 | 111904310 | 1.344563177 | 5 | amp |
| TCGA-09-0365 | 11 | 111907970 | 111956222 | 0.882602582 | 4 | amp |
| TCGA-09-0365 | 11 | 111957345 | 113577004 | 1.394495598 | 5 | amp |
| TCGA-09-0365 | 11 | 113604355 | 113803856 | 1.004068257 | 4 | amp |
| TCGA-09-0365 | 11 | 113813688 | 114318634 | 1.362753991 | 5 | amp |
| TCGA-09-0365 | 11 | 114320512 | 115111186 | 0.853188419 | 4 | amp |
| TCGA-09-0365 | 11 | 116619184 | 116741141 | 1.60335975 | 5 | amp |
| TCGA-09-0365 | 11 | 116744154 | 116827841 | 0.86276184 | 4 | amp |
| TCGA-09-0365 | 11 | 117006204 | 117117714 | 1.933909739 | 6 | amp |
| TCGA-09-0365 | 11 | 117150611 | 117261942 | 1.370974646 | 5 | amp |
| TCGA-09-0365 | 11 | 117262890 | 118047171 | 1.794099498 | 6 | amp |
| TCGA-09-0365 | 11 | 118065025 | 118267211 | 1.015802382 | 4 | amp |
| TCGA-09-0365 | 11 | 118272316 | 118463617 | 1.333131084 | 5 | amp |
| TCGA-09-0365 | 11 | 118464268 | 118534157 | 1.864881748 | 6 | amp |
| TCGA-09-0365 | 11 | 118625388 | 118639115 | 0.955364379 | 4 | amp |
| TCGA-09-0365 | 11 | 118650332 | 119063965 | 1.87864078 | 6 | amp |
| TCGA-09-0365 | 11 | 119103071 | 119168202 | 0.978700093 | 4 | amp |
| TCGA-09-0365 | 11 | 119169038 | 120180334 | 1.967656023 | 6 | amp |
| TCGA-09-0365 | 11 | 120186042 | 120208028 | 1.38188229 | 5 | amp |
| TCGA-09-0365 | 11 | 120276778 | 120355241 | 0.807783129 | 4 | amp |
| TCGA-09-0365 | 11 | 120355690 | 121037507 | 1.34554617 | 5 | amp |
| TCGA-09-0365 | 11 | 121038724 | 121448169 | 0.974884824 | 4 | amp |
| TCGA-09-0365 | 11 | 121454109 | 121500298 | 1.31259643 | 5 | amp |
| TCGA-09-0365 | 11 | 121970418 | 122830174 | 0.95946057 | 4 | amp |
| TCGA-09-0365 | 11 | 122848267 | 123625236 | 1.408133997 | 5 | amp |
| TCGA-09-0365 | 11 | 123676106 | 124483066 | 0.947061822 | 4 | amp |
| TCGA-09-0365 | 11 | 124487126 | 124530731 | 1.327740934 | 5 | amp |
| TCGA-09-0365 | 11 | 124539216 | 125281821 | 1.861563077 | 6 | amp |
| TCGA-09-0365 | 11 | 125298885 | 125848298 | 1.384232621 | 5 | amp |
| TCGA-09-0365 | 11 | 125850903 | 125893393 | 0.800818911 | 4 | amp |
| TCGA-09-0365 | 11 | 126073252 | 134252931 | 1.457177185 | 5 | amp |
| TCGA-09-0365 | 12 | 73256 | 133768601 | 1.919795775 | 6 | amp |
| TCGA-09-0365 | 12 | 133778675 | 133779395 | 1.682882728 | 5 | amp |
| TCGA-09-0365 | 13 | 19240876 | 19414327 | 1.419043486 | 5 | amp |

| | | | | | | |
|---|---|---|---|---|---|---|
| TCGA-09-0365 | 13 | 19419858 | 19419978 | 1.085559277 | 4 | amp |
| TCGA-09-0365 | 13 | 19705370 | 19753711 | 1.411901581 | 5 | amp |
| TCGA-09-0365 | 13 | 19775749 | 115064514 | 1.174816951 | 4 | amp |
| TCGA-09-0365 | 13 | 115067142 | 115091796 | 1.371323824 | 5 | amp |
| TCGA-09-0365 | 14 | 19377543 | 21561807 | 1.40504924 | 5 | amp |
| TCGA-09-0365 | 14 | 21623039 | 23013107 | 0.995678847 | 4 | amp |
| TCGA-09-0365 | 14 | 23013951 | 23424395 | 1.391347314 | 5 | amp |
| TCGA-09-0365 | 14 | 23442594 | 23938990 | 1.830988624 | 6 | amp |
| TCGA-09-0365 | 14 | 23939190 | 23947285 | 0.758257474 | 3 | amp |
| TCGA-09-0365 | 14 | 24025909 | 24910180 | 1.997915089 | 6 | amp |
| TCGA-09-0365 | 14 | 24910820 | 25078851 | 1.139176272 | 4 | amp |
| TCGA-09-0365 | 14 | 25100228 | 47600991 | 0.887110346 | 3 | amp |
| TCGA-09-0365 | 14 | 50044490 | 50587107 | 1.013710757 | 4 | amp |
| TCGA-09-0365 | 14 | 50596639 | 51221641 | 0.878105723 | 3 | amp |
| TCGA-09-0365 | 14 | 51223153 | 52794175 | 1.04896372 | 4 | amp |
| TCGA-09-0365 | 14 | 52898983 | 60938483 | 0.889812869 | 3 | amp |
| TCGA-09-0365 | 14 | 60945002 | 64066678 | 1.030802957 | 4 | amp |
| TCGA-09-0365 | 14 | 64152798 | 64634404 | 0.846081235 | 3 | amp |
| TCGA-09-0365 | 14 | 64635581 | 65568336 | 1.361808768 | 5 | amp |
| TCGA-09-0365 | 14 | 66028262 | 67790533 | 0.993830159 | 4 | amp |
| TCGA-09-0365 | 14 | 67799464 | 68280801 | 1.283860322 | 5 | amp |
| TCGA-09-0365 | 14 | 68282463 | 68935066 | 0.762646825 | 3 | amp |
| TCGA-09-0365 | 14 | 68935756 | 70714463 | 1.271269431 | 5 | amp |
| TCGA-09-0365 | 14 | 70793107 | 71267830 | 1.091114216 | 4 | amp |
| TCGA-09-0365 | 14 | 71413615 | 72169279 | 0.771216826 | 3 | amp |
| TCGA-09-0365 | 14 | 72171408 | 72985271 | 1.072007361 | 4 | amp |
| TCGA-09-0365 | 14 | 73002848 | 75070432 | 1.353837247 | 5 | amp |
| TCGA-09-0365 | 14 | 75130383 | 75323720 | 0.970508682 | 4 | amp |
| TCGA-09-0365 | 14 | 75325100 | 76105809 | 1.30738604 | 5 | amp |
| TCGA-09-0365 | 14 | 76107290 | 76420837 | 0.884116598 | 4 | amp |
| TCGA-09-0365 | 14 | 76425488 | 77978806 | 1.437991061 | 5 | amp |
| TCGA-09-0365 | 14 | 77984295 | 91633765 | 1.002172149 | 4 | amp |
| TCGA-09-0365 | 14 | 91636295 | 91875116 | 1.448672288 | 5 | amp |
| TCGA-09-0365 | 14 | 91925029 | 92071074 | 0.974429848 | 4 | amp |
| TCGA-09-0365 | 14 | 92074602 | 92474209 | 0.801513846 | 3 | amp |
| TCGA-09-0365 | 14 | 92477312 | 92628101 | 1.011304003 | 4 | amp |
| TCGA-09-0365 | 14 | 92792206 | 93715030 | 1.317435283 | 5 | amp |
| TCGA-09-0365 | 14 | 93717783 | 94171051 | 0.819171853 | 3 | amp |
| TCGA-09-0365 | 14 | 94172999 | 94693716 | 1.409778489 | 5 | amp |
| TCGA-09-0365 | 14 | 94696877 | 94752639 | 0.766939657 | 3 | amp |
| TCGA-09-0365 | 14 | 94754579 | 95111388 | 1.259492607 | 5 | amp |
| TCGA-09-0365 | 14 | 95192939 | 95677239 | 0.930928549 | 4 | amp |
| TCGA-09-0365 | 14 | 95679530 | 96752326 | 1.314290544 | 5 | amp |
| TCGA-09-0365 | 14 | 96755917 | 99932150 | 0.865647744 | 3 | amp |
| TCGA-09-0365 | 14 | 99958992 | 101393773 | 1.567818018 | 5 | amp |
| TCGA-09-0365 | 14 | 101396239 | 101459669 | 0.728396898 | 3 | amp |
| TCGA-09-0365 | 14 | 101488345 | 104508524 | 1.379437636 | 5 | amp |
| TCGA-09-0365 | 14 | 104515920 | 106111159 | 1.924236845 | 6 | amp |
| TCGA-09-0365 | 14 | 106134683 | 106552766 | 1.476307007 | 5 | amp |
| TCGA-09-0365 | 14 | 106573203 | 107283263 | 1.055006592 | 4 | amp |

| | | | | | | |
|---|---|---|---|---|---|---|
| TCGA-09-0365 | 15 | 20169886 | 23356222 | 1.002035421 | 4 | amp |
| TCGA-09-0365 | 15 | 23377399 | 23610309 | 1.202531435 | 5 | amp |
| TCGA-09-0365 | 15 | 23810911 | 25328020 | 0.934695788 | 4 | amp |
| TCGA-09-0365 | 15 | 25328720 | 25420174 | 0.684380625 | 3 | amp |
| TCGA-09-0365 | 15 | 25421958 | 25481332 | 1.42070859 | 5 | amp |
| TCGA-09-0365 | 15 | 25483113 | 25523582 | 1.079279645 | 4 | amp |
| TCGA-09-0365 | 15 | 25654184 | 28390004 | 0.957508588 | 4 | amp |
| TCGA-09-0365 | 15 | 28391376 | 28506139 | 0.80782033 | 3 | amp |
| TCGA-09-0365 | 15 | 28508009 | 28835430 | 1.048675979 | 4 | amp |
| TCGA-09-0365 | 15 | 28857202 | 30092936 | 0.793207454 | 3 | amp |
| TCGA-09-0365 | 15 | 30337369 | 30919207 | 0.998787973 | 4 | amp |
| TCGA-09-0365 | 15 | 30922880 | 34610940 | 0.778967121 | 3 | amp |
| TCGA-09-0365 | 15 | 34628565 | 35087064 | 1.094834054 | 4 | amp |
| TCGA-09-0365 | 15 | 35148969 | 40318293 | 0.782081734 | 3 | amp |
| TCGA-09-0365 | 15 | 40321575 | 40398321 | 1.204259464 | 5 | amp |
| TCGA-09-0365 | 15 | 40457205 | 40512982 | 0.697737702 | 3 | amp |
| TCGA-09-0365 | 15 | 40556938 | 40757643 | 1.392352671 | 5 | amp |
| TCGA-09-0365 | 15 | 40758084 | 40901135 | 1.055125084 | 4 | amp |
| TCGA-09-0365 | 15 | 40902408 | 40993450 | 0.731099931 | 3 | amp |
| TCGA-09-0365 | 15 | 40998313 | 41280212 | 1.209468175 | 5 | amp |
| TCGA-09-0365 | 15 | 41289702 | 41388556 | 0.646174638 | 3 | amp |
| TCGA-09-0365 | 15 | 41455382 | 41768742 | 0.999826924 | 4 | amp |
| TCGA-09-0365 | 15 | 41769303 | 41870518 | 1.220305839 | 5 | amp |
| TCGA-09-0365 | 15 | 41961084 | 42104359 | 0.797999929 | 3 | amp |
| TCGA-09-0365 | 15 | 42104662 | 42297215 | 1.403126915 | 5 | amp |
| TCGA-09-0365 | 15 | 42298133 | 42455630 | 1.123446094 | 4 | amp |
| TCGA-09-0365 | 15 | 42455784 | 42837487 | 0.692959116 | 3 | amp |
| TCGA-09-0365 | 15 | 42839412 | 43038463 | 1.120820211 | 4 | amp |
| TCGA-09-0365 | 15 | 43044148 | 43270171 | 0.66297962 | 3 | amp |
| TCGA-09-0365 | 15 | 43276082 | 43317662 | 1.091823787 | 4 | amp |
| TCGA-09-0365 | 15 | 43318749 | 43444185 | 0.768151575 | 3 | amp |
| TCGA-09-0365 | 15 | 43446829 | 43675721 | 1.107027506 | 4 | amp |
| TCGA-09-0365 | 15 | 43677951 | 43749472 | 0.619658824 | 3 | amp |
| TCGA-09-0365 | 15 | 43762039 | 43875164 | 0.982742383 | 4 | amp |
| TCGA-09-0365 | 15 | 43875549 | 43902692 | 1.239019869 | 5 | amp |
| TCGA-09-0365 | 15 | 43903077 | 44116787 | 1.084762414 | 4 | amp |
| TCGA-09-0365 | 15 | 44120123 | 45357647 | 0.731321266 | 3 | amp |
| TCGA-09-0365 | 15 | 45360351 | 45453231 | 1.380716334 | 5 | amp |
| TCGA-09-0365 | 15 | 45453931 | 45777557 | 1.042150286 | 4 | amp |
| TCGA-09-0365 | 15 | 45778755 | 45983259 | 0.780408145 | 3 | amp |
| TCGA-09-0365 | 15 | 51980407 | 52606419 | 0.828303301 | 3 | amp |
| TCGA-09-0365 | 15 | 55488957 | 56665730 | 0.763958239 | 3 | amp |
| TCGA-09-0365 | 15 | 57808930 | 58889852 | 0.792713986 | 3 | amp |
| TCGA-09-0365 | 15 | 59377800 | 59944500 | 0.770653703 | 3 | amp |
| TCGA-09-0365 | 15 | 59961079 | 60716016 | 0.979587424 | 4 | amp |
| TCGA-09-0365 | 15 | 60720576 | 60919629 | 0.745844506 | 3 | amp |
| TCGA-09-0365 | 15 | 62967407 | 63667926 | 0.953559844 | 4 | amp |
| TCGA-09-0365 | 15 | 63824815 | 64217063 | 0.678834405 | 3 | amp |
| TCGA-09-0365 | 15 | 64218065 | 64495394 | 0.998334699 | 4 | amp |
| TCGA-09-0365 | 15 | 64496593 | 64973587 | 0.779198617 | 3 | amp |

| | | | | | | |
|---|---|---|---|---|---|---|
| TCGA-09-0365 | 15 | 64980847 | 65218376 | 1.2497637 | 5 | amp |
| TCGA-09-0365 | 15 | 65219058 | 65421558 | 1.015713771 | 4 | amp |
| TCGA-09-0365 | 15 | 65443115 | 65459165 | 0.7175636 | 3 | amp |
| TCGA-09-0365 | 15 | 65471184 | 65684769 | 1.166659932 | 4 | amp |
| TCGA-09-0365 | 15 | 65685663 | 66191288 | 0.729358003 | 3 | amp |
| TCGA-09-0365 | 15 | 66198390 | 66587517 | 1.098709991 | 4 | amp |
| TCGA-09-0365 | 15 | 66599105 | 66850406 | 0.781120879 | 3 | amp |
| TCGA-09-0365 | 15 | 66853301 | 67496493 | 1.044502373 | 4 | amp |
| TCGA-09-0365 | 15 | 67500887 | 68065127 | 0.669197652 | 3 | amp |
| TCGA-09-0365 | 15 | 68099145 | 68126186 | 1.188453859 | 4 | amp |
| TCGA-09-0365 | 15 | 68378625 | 68486461 | 0.718771184 | 3 | amp |
| TCGA-09-0365 | 15 | 68489751 | 70386952 | 1.09662332 | 4 | amp |
| TCGA-09-0365 | 15 | 71124352 | 72023567 | 0.804627946 | 3 | amp |
| TCGA-09-0365 | 15 | 72029998 | 72109956 | 1.113585908 | 4 | amp |
| TCGA-09-0365 | 15 | 72454261 | 72691301 | 1.087504207 | 4 | amp |
| TCGA-09-0365 | 15 | 72698897 | 72875650 | 0.634942161 | 3 | amp |
| TCGA-09-0365 | 15 | 72879545 | 72958739 | 1.240868511 | 5 | amp |
| TCGA-09-0365 | 15 | 72987483 | 73889716 | 0.721929876 | 3 | amp |
| TCGA-09-0365 | 15 | 73991929 | 75674059 | 1.247058198 | 5 | amp |
| TCGA-09-0365 | 15 | 75676541 | 75704138 | 0.725577811 | 3 | amp |
| TCGA-09-0365 | 15 | 75705002 | 76146837 | 1.099707233 | 4 | amp |
| TCGA-09-0365 | 15 | 76152210 | 76430261 | 0.769506753 | 3 | amp |
| TCGA-09-0365 | 15 | 76448871 | 76673957 | 0.990385439 | 4 | amp |
| TCGA-09-0365 | 15 | 76696822 | 77771708 | 0.729033694 | 3 | amp |
| TCGA-09-0365 | 15 | 77906473 | 78732239 | 1.11251748 | 4 | amp |
| TCGA-09-0365 | 15 | 78755226 | 78786664 | 0.784096156 | 3 | amp |
| TCGA-09-0365 | 15 | 78789440 | 81654661 | 1.016321287 | 4 | amp |
| TCGA-09-0365 | 15 | 81660571 | 82545133 | 0.612459018 | 3 | amp |
| TCGA-09-0365 | 15 | 82551402 | 83679171 | 1.038714174 | 4 | amp |
| TCGA-09-0365 | 15 | 83686747 | 83788441 | 0.824760709 | 3 | amp |
| TCGA-09-0365 | 15 | 84859539 | 85056549 | 1.223613588 | 5 | amp |
| TCGA-09-0365 | 15 | 85057178 | 85488453 | 1.017586902 | 4 | amp |
| TCGA-09-0365 | 15 | 85607568 | 88669612 | 0.760188347 | 3 | amp |
| TCGA-09-0365 | 15 | 88670364 | 89762292 | 1.088860032 | 4 | amp |
| TCGA-09-0365 | 15 | 89790830 | 89828488 | 0.619789815 | 3 | amp |
| TCGA-09-0365 | 15 | 89833417 | 90129175 | 1.172758433 | 4 | amp |
| TCGA-09-0365 | 15 | 90135222 | 90152219 | 0.699075016 | 3 | amp |
| TCGA-09-0365 | 15 | 90159590 | 90281489 | 0.98103593 | 4 | amp |
| TCGA-09-0365 | 15 | 90286482 | 90934115 | 1.223235166 | 5 | amp |
| TCGA-09-0365 | 15 | 90969299 | 91354655 | 0.713711924 | 3 | amp |
| TCGA-09-0365 | 15 | 91358300 | 91550810 | 1.241516021 | 5 | amp |
| TCGA-09-0365 | 15 | 91551056 | 93444558 | 0.942927755 | 4 | amp |
| TCGA-09-0365 | 15 | 93467546 | 95020033 | 0.672367141 | 3 | amp |
| TCGA-09-0365 | 15 | 95022168 | 99715367 | 1.091565286 | 4 | amp |
| TCGA-09-0365 | 15 | 99740092 | 100215693 | 0.685209075 | 3 | amp |
| TCGA-09-0365 | 15 | 100230419 | 100943002 | 1.076387474 | 4 | amp |
| TCGA-09-0365 | 15 | 100996054 | 101121087 | 0.648006467 | 3 | amp |
| TCGA-09-0365 | 15 | 101152406 | 102516522 | 1.08930044 | 4 | amp |
| TCGA-09-0365 | 16 | 66517 | 701108 | 1.663023847 | 5 | amp |
| TCGA-09-0365 | 16 | 701605 | 3340575 | 1.983118809 | 6 | amp |

| | | | | | | |
|---|---|---|---|---|---|---|
| TCGA-09-0365 | 16 | 3348919 | 3860811 | 1.565271393 | 5 | amp |
| TCGA-09-0365 | 16 | 3900263 | 9210826 | 4.579608252 | 6 | amp |
| TCGA-09-0365 | 16 | 9250495 | 10637531 | 1.453834614 | 5 | amp |
| TCGA-09-0365 | 16 | 10641375 | 11781827 | 1.928341282 | 6 | amp |
| TCGA-09-0365 | 16 | 11782109 | 15781419 | 1.427656601 | 5 | amp |
| TCGA-09-0365 | 16 | 15784970 | 16346394 | 1.856251737 | 6 | amp |
| TCGA-09-0365 | 16 | 16349569 | 23366834 | 1.415588782 | 5 | amp |
| TCGA-09-0365 | 16 | 23379168 | 23848771 | 1.98890585 | 6 | amp |
| TCGA-09-0365 | 16 | 23999811 | 26147610 | 1.424739874 | 5 | amp |
| TCGA-09-0365 | 16 | 27078094 | 28124417 | 1.904263518 | 6 | amp |
| TCGA-09-0365 | 16 | 28128590 | 28192366 | 1.175369628 | 4 | amp |
| TCGA-09-0365 | 16 | 28328675 | 29370694 | 2.077076349 | 6 | amp |
| TCGA-09-0365 | 16 | 29372513 | 29647709 | 1.440494733 | 5 | amp |
| TCGA-09-0365 | 16 | 29674960 | 30233259 | 2.276821862 | 6 | amp |
| TCGA-09-0365 | 16 | 30236800 | 30317312 | 1.104118328 | 4 | amp |
| TCGA-09-0365 | 16 | 30354558 | 31228394 | 1.930152345 | 6 | amp |
| TCGA-09-0365 | 16 | 31230574 | 31367326 | 1.480942682 | 5 | amp |
| TCGA-09-0365 | 16 | 31370965 | 31885348 | 1.988741868 | 6 | amp |
| TCGA-09-0365 | 16 | 31895761 | 66850589 | 1.344128838 | 5 | amp |
| TCGA-09-0365 | 16 | 66850839 | 67333490 | 1.872589473 | 6 | amp |
| TCGA-09-0365 | 16 | 67335609 | 67839446 | 1.560310322 | 5 | amp |
| TCGA-09-0365 | 16 | 67854715 | 68024931 | 1.838387787 | 6 | amp |
| TCGA-09-0365 | 16 | 68024962 | 85105506 | 1.35874208 | 5 | amp |
| TCGA-09-0365 | 16 | 85106507 | 90244214 | 1.779770129 | 6 | amp |
| TCGA-09-0365 | 17 | 63618 | 680287 | 1.067165678 | 4 | amp |
| TCGA-09-0365 | 17 | 681869 | 2935768 | 1.249641993 | 5 | amp |
| TCGA-09-0365 | 17 | 2965951 | 3427694 | 0.897683046 | 4 | amp |
| TCGA-09-0365 | 17 | 3430124 | 3917492 | 1.349992794 | 5 | amp |
| TCGA-09-0365 | 17 | 3917604 | 4027392 | 0.710989641 | 3 | amp |
| TCGA-09-0365 | 17 | 4046964 | 5045804 | 1.42486758 | 5 | amp |
| TCGA-09-0365 | 17 | 5047951 | 5138099 | 1.004538412 | 4 | amp |
| TCGA-09-0365 | 17 | 5211932 | 5320037 | 0.73555431 | 3 | amp |
| TCGA-09-0365 | 17 | 5322641 | 6441414 | 1.290997703 | 5 | amp |
| TCGA-09-0365 | 17 | 6482975 | 6526919 | 0.705935089 | 3 | amp |
| TCGA-09-0365 | 17 | 6528067 | 8398566 | 1.403401111 | 5 | amp |
| TCGA-09-0365 | 17 | 8402540 | 8508341 | 0.936156775 | 4 | amp |
| TCGA-09-0365 | 17 | 8526211 | 9124581 | 1.430859217 | 5 | amp |
| TCGA-09-0365 | 17 | 9281859 | 9503558 | 0.822845646 | 4 | amp |
| TCGA-09-0365 | 17 | 9511425 | 9939811 | 1.235467771 | 5 | amp |
| TCGA-09-0365 | 17 | 10204214 | 10233891 | 1.037153995 | 4 | amp |
| TCGA-09-0365 | 17 | 10531892 | 12823169 | 0.859864058 | 3 | amp |
| TCGA-09-0365 | 17 | 12832244 | 15907232 | 1.083950909 | 4 | amp |
| TCGA-09-0365 | 17 | 15907455 | 16090084 | 0.751424447 | 3 | amp |
| TCGA-09-0365 | 17 | 16097769 | 16520462 | 1.232209562 | 5 | amp |
| TCGA-09-0365 | 17 | 16525572 | 16594066 | 1.124805253 | 4 | amp |
| TCGA-09-0365 | 17 | 16596267 | 16705659 | 0.809440374 | 3 | amp |
| TCGA-09-0365 | 17 | 16734996 | 18419111 | 1.389773797 | 5 | amp |
| TCGA-09-0365 | 17 | 18420432 | 18525907 | 0.788386889 | 3 | amp |
| TCGA-09-0365 | 17 | 18528408 | 18585006 | 1.397204418 | 5 | amp |
| TCGA-09-0365 | 17 | 18625537 | 18682620 | 1.027090289 | 4 | amp |

| | | | | | | |
|---|---|---|---|---|---|---|
| TCGA-09-0365 | 17 | 18692646 | 18834051 | 0.738929979 | 3 | amp |
| TCGA-09-0365 | 17 | 18855513 | 19684477 | 1.380269742 | 5 | amp |
| TCGA-09-0365 | 17 | 19685170 | 19835324 | 0.714747412 | 3 | amp |
| TCGA-09-0365 | 17 | 19839550 | 20227610 | 1.151817135 | 4 | amp |
| TCGA-09-0365 | 17 | 20239088 | 20334237 | 0.768384948 | 3 | amp |
| TCGA-09-0365 | 17 | 20353238 | 26925998 | 1.428459936 | 5 | amp |
| TCGA-09-0365 | 17 | 26938532 | 26951449 | 1.020748127 | 4 | amp |
| TCGA-09-0365 | 17 | 26955249 | 27573956 | 1.394609651 | 5 | amp |
| TCGA-09-0365 | 17 | 27576131 | 27849597 | 0.788591359 | 3 | amp |
| TCGA-09-0365 | 17 | 27857380 | 27959983 | 1.31625386 | 5 | amp |
| TCGA-09-0365 | 17 | 27962952 | 29365692 | 0.822156635 | 3 | amp |
| TCGA-09-0365 | 17 | 29663308 | 30510300 | 1.06288014 | 4 | amp |
| TCGA-09-0365 | 17 | 30594872 | 30688008 | 1.273519058 | 5 | amp |
| TCGA-09-0365 | 17 | 30688450 | 33285765 | 0.833565173 | 3 | amp |
| TCGA-09-0365 | 17 | 33286577 | 33348834 | 1.058382597 | 4 | amp |
| TCGA-09-0365 | 17 | 33353362 | 33510643 | 1.325747401 | 5 | amp |
| TCGA-09-0365 | 17 | 33513289 | 34044410 | 0.813473979 | 3 | amp |
| TCGA-09-0365 | 17 | 34050620 | 34144799 | 1.363540492 | 5 | amp |
| TCGA-09-0365 | 17 | 34146993 | 34207331 | 1.023107786 | 4 | amp |
| TCGA-09-0365 | 17 | 34245336 | 34433721 | 0.878967402 | 3 | amp |
| TCGA-09-0365 | 17 | 34493274 | 35311258 | 1.217998531 | 5 | amp |
| TCGA-09-0365 | 17 | 35733003 | 36002357 | 0.880169484 | 3 | amp |
| TCGA-09-0365 | 17 | 36003315 | 37373437 | 1.221999658 | 5 | amp |
| TCGA-09-0365 | 17 | 37417676 | 37588291 | 0.890581754 | 3 | amp |
| TCGA-09-0365 | 17 | 37761643 | 37903196 | 1.47598507 | 5 | amp |
| TCGA-09-0365 | 17 | 37922034 | 38073571 | 0.966981339 | 4 | amp |
| TCGA-09-0365 | 17 | 38078720 | 38350097 | 1.379548721 | 5 | amp |
| TCGA-09-0365 | 17 | 38412652 | 38546436 | 0.993164959 | 4 | amp |
| TCGA-09-0365 | 17 | 38547704 | 39504932 | 0.831276854 | 3 | amp |
| TCGA-09-0365 | 17 | 39505578 | 40469280 | 1.274128799 | 5 | amp |
| TCGA-09-0365 | 17 | 40474285 | 40485796 | 1.079714446 | 4 | amp |
| TCGA-09-0365 | 17 | 40485867 | 40666512 | 0.844905339 | 3 | amp |
| TCGA-09-0365 | 17 | 40673001 | 41026428 | 1.274712665 | 5 | amp |
| TCGA-09-0365 | 17 | 41052888 | 41599624 | 1.041786607 | 4 | amp |
| TCGA-09-0365 | 17 | 41600955 | 43580944 | 1.631995214 | 5 | amp |
| TCGA-09-0365 | 17 | 43664066 | 44110857 | 1.592381457 | 5 | amp |
| TCGA-09-0365 | 17 | 44111438 | 44382970 | 0.938844872 | 4 | amp |
| TCGA-09-0365 | 17 | 44383574 | 45099311 | 1.292777134 | 5 | amp |
| TCGA-09-0365 | 17 | 45102021 | 45219818 | 0.783143069 | 3 | amp |
| TCGA-09-0365 | 17 | 45221208 | 47304090 | 1.384215612 | 5 | amp |
| TCGA-09-0365 | 17 | 47375722 | 47395117 | 0.700820713 | 3 | amp |
| TCGA-09-0365 | 17 | 47482339 | 48785029 | 1.669964369 | 5 | amp |
| TCGA-09-0365 | 17 | 48814293 | 49157124 | 0.859483298 | 3 | amp |
| TCGA-09-0365 | 17 | 49231701 | 53046064 | 1.079549155 | 4 | amp |
| TCGA-09-0365 | 17 | 53063543 | 54559932 | 0.864609193 | 3 | amp |
| TCGA-09-0365 | 17 | 54671531 | 56621606 | 1.485814406 | 5 | amp |
| TCGA-09-0365 | 17 | 56634329 | 57684583 | 1.020195664 | 4 | amp |
| TCGA-09-0365 | 17 | 57721590 | 58035848 | 0.805529405 | 3 | amp |
| TCGA-09-0365 | 17 | 58037418 | 58236807 | 1.403929565 | 5 | amp |
| TCGA-09-0365 | 17 | 58256582 | 59560928 | 0.865079808 | 3 | amp |

| | | | | | | |
|---|---|---|---|---|---|---|
| TCGA-09-0365 | 17 | 60318917 | 61907320 | 0.898236899 | 3 | amp |
| TCGA-09-0365 | 17 | 61907460 | 62045728 | 1.251022117 | 5 | amp |
| TCGA-09-0365 | 17 | 62049480 | 63632150 | 0.874685676 | 3 | amp |
| TCGA-09-0365 | 17 | 64179304 | 64685176 | 0.827432691 | 3 | amp |
| TCGA-09-0365 | 17 | 64728724 | 65052393 | 1.258842289 | 5 | amp |
| TCGA-09-0365 | 17 | 65074354 | 65989268 | 0.789637119 | 3 | amp |
| TCGA-09-0365 | 17 | 66033225 | 66352996 | 1.420242202 | 5 | amp |
| TCGA-09-0365 | 17 | 66364636 | 66551911 | 1.11436213 | 4 | amp |
| TCGA-09-0365 | 17 | 66864184 | 66890502 | 0.854133066 | 3 | amp |
| TCGA-09-0365 | 17 | 67513637 | 68172482 | 0.838742033 | 3 | amp |
| TCGA-09-0365 | 17 | 70118806 | 81188237 | 1.595762162 | 5 | amp |
| TCGA-09-0365 | 18 | 47273 | 179044 | 1.088476625 | 4 | amp |
| TCGA-09-0365 | 18 | 180227 | 196821 | 0.703236885 | 3 | amp |
| TCGA-09-0365 | 18 | 197595 | 77891090 | 1.019784699 | 4 | amp |
| TCGA-09-0365 | 18 | 77893473 | 77960823 | 1.440041856 | 5 | amp |
| TCGA-09-0365 | 19 | 71882 | 5727376 | 1.917942561 | 6 | amp |
| TCGA-09-0365 | 19 | 5729858 | 5773007 | 1.294821532 | 5 | amp |
| TCGA-09-0365 | 19 | 5776098 | 6042154 | 1.985895494 | 6 | amp |
| TCGA-09-0365 | 19 | 6141497 | 6374657 | 1.440779317 | 5 | amp |
| TCGA-09-0365 | 19 | 6374752 | 6733692 | 1.862332118 | 6 | amp |
| TCGA-09-0365 | 19 | 6733799 | 7570517 | 1.478406552 | 5 | amp |
| TCGA-09-0365 | 19 | 7570800 | 7797020 | 1.948978573 | 6 | amp |
| TCGA-09-0365 | 19 | 7807875 | 7833918 | 1.231219129 | 5 | amp |
| TCGA-09-0365 | 19 | 7911346 | 8503470 | 1.87095454 | 6 | amp |
| TCGA-09-0365 | 19 | 8520253 | 8539149 | 1.339314504 | 5 | amp |
| TCGA-09-0365 | 19 | 8548008 | 8809085 | 1.892406294 | 6 | amp |
| TCGA-09-0365 | 19 | 8841352 | 10292824 | 1.402808224 | 5 | amp |
| TCGA-09-0365 | 19 | 10334480 | 11725524 | 1.942993166 | 6 | amp |
| TCGA-09-0365 | 19 | 11727476 | 12740084 | 1.348385834 | 5 | amp |
| TCGA-09-0365 | 19 | 12754045 | 13255690 | 1.915478908 | 6 | amp |
| TCGA-09-0365 | 19 | 13260269 | 13566033 | 1.561730109 | 5 | amp |
| TCGA-09-0365 | 19 | 13862563 | 14516775 | 2.027152084 | 6 | amp |
| TCGA-09-0365 | 19 | 14517484 | 17308693 | 1.519797578 | 5 | amp |
| TCGA-09-0365 | 19 | 17308934 | 19767047 | 1.866282548 | 6 | amp |
| TCGA-09-0365 | 19 | 19770384 | 30099649 | 1.133247635 | 4 | amp |
| TCGA-09-0365 | 19 | 30101307 | 30314742 | 2.21745806 | 6 | amp |
| TCGA-09-0365 | 19 | 30462056 | 32968613 | 1.261044131 | 5 | amp |
| TCGA-09-0365 | 19 | 32971263 | 34302761 | 2.145219668 | 6 | amp |
| TCGA-09-0365 | 19 | 34685344 | 34818783 | 1.378239577 | 5 | amp |
| TCGA-09-0365 | 19 | 34818825 | 36900746 | 2.649716668 | 6 | amp |
| TCGA-09-0365 | 19 | 36939850 | 38134264 | 1.483084929 | 5 | amp |
| TCGA-09-0365 | 19 | 38135453 | 44733987 | 3.016841882 | 6 | amp |
| TCGA-09-0365 | 19 | 44738838 | 45179737 | 1.44131519 | 5 | amp |
| TCGA-09-0365 | 19 | 45182110 | 46326118 | 1.937855559 | 6 | amp |
| TCGA-09-0365 | 19 | 46326589 | 49387305 | 1.612871809 | 5 | amp |
| TCGA-09-0365 | 19 | 49388700 | 49714533 | 1.914889453 | 6 | amp |
| TCGA-09-0365 | 19 | 49714692 | 49842688 | 1.342863358 | 5 | amp |
| TCGA-09-0365 | 19 | 49843456 | 51329247 | 1.995338323 | 6 | amp |
| TCGA-09-0365 | 19 | 51329824 | 54618103 | 1.434538777 | 5 | amp |
| TCGA-09-0365 | 19 | 54618577 | 54784394 | 1.989708342 | 6 | amp |

| | | | | | | |
|---|---|---|---|---|---|---|
| TCGA-09-0365 | 19 | 54799984 | 54868261 | 1.484549172 | 5 | amp |
| TCGA-09-0365 | 19 | 54868495 | 54969206 | 1.939506591 | 6 | amp |
| TCGA-09-0365 | 19 | 54969226 | 55485949 | 1.387042398 | 5 | amp |
| TCGA-09-0365 | 19 | 55489065 | 56185486 | 1.998980168 | 6 | amp |
| TCGA-09-0365 | 19 | 56189987 | 59110878 | 1.354941888 | 5 | amp |
| TCGA-09-0365 | 2 | 41527 | 8823060 | 2.127018002 | 6 | amp |
| TCGA-09-0365 | 2 | 8870820 | 10126450 | 1.47408663 | 5 | amp |
| TCGA-09-0365 | 2 | 10130789 | 10585167 | 2.028500935 | 6 | amp |
| TCGA-09-0365 | 2 | 10712196 | 10811867 | 1.464984005 | 5 | amp |
| TCGA-09-0365 | 2 | 10813604 | 11312230 | 2.057892087 | 6 | amp |
| TCGA-09-0365 | 2 | 11315054 | 11696938 | 1.267385306 | 5 | amp |
| TCGA-09-0365 | 2 | 11702498 | 13555122 | 2.212040795 | 6 | amp |
| TCGA-09-0365 | 2 | 14774059 | 20174431 | 1.373763748 | 5 | amp |
| TCGA-09-0365 | 2 | 20175237 | 20405204 | 2.077997425 | 6 | amp |
| TCGA-09-0365 | 2 | 20451291 | 24207741 | 1.463193217 | 5 | amp |
| TCGA-09-0365 | 2 | 24222489 | 24427320 | 2.106955897 | 6 | amp |
| TCGA-09-0365 | 2 | 24428034 | 25037423 | 1.302307574 | 5 | amp |
| TCGA-09-0365 | 2 | 25038289 | 25642453 | 2.098042139 | 6 | amp |
| TCGA-09-0365 | 2 | 25650391 | 26512863 | 1.575226517 | 5 | amp |
| TCGA-09-0365 | 2 | 26531904 | 27826210 | 2.379826127 | 6 | amp |
| TCGA-09-0365 | 2 | 27830658 | 28748200 | 1.455943374 | 5 | amp |
| TCGA-09-0365 | 2 | 28748731 | 28848009 | 1.876031833 | 6 | amp |
| TCGA-09-0365 | 2 | 28849244 | 29221123 | 1.544594398 | 5 | amp |
| TCGA-09-0365 | 2 | 29222010 | 29297153 | 2.152986531 | 6 | amp |
| TCGA-09-0365 | 2 | 29344200 | 29420577 | 0.99210361 | 4 | amp |
| TCGA-09-0365 | 2 | 29429997 | 29498374 | 2.096679018 | 6 | amp |
| TCGA-09-0365 | 2 | 29519718 | 30953698 | 1.498561085 | 5 | amp |
| TCGA-09-0365 | 2 | 30954177 | 31610779 | 1.832646998 | 6 | amp |
| TCGA-09-0365 | 2 | 31611066 | 46819727 | 1.264720693 | 5 | amp |
| TCGA-09-0365 | 2 | 46839424 | 47277196 | 2.222801617 | 6 | amp |
| TCGA-09-0365 | 2 | 47278801 | 70457996 | 1.324078217 | 5 | amp |
| TCGA-09-0365 | 2 | 70463198 | 71375242 | 2.093917983 | 6 | amp |
| TCGA-09-0365 | 2 | 71376347 | 71730456 | 1.369861951 | 5 | amp |
| TCGA-09-0365 | 2 | 71740798 | 72371349 | 2.49225515 | 6 | amp |
| TCGA-09-0365 | 2 | 72406406 | 73053042 | 1.242863274 | 5 | amp |
| TCGA-09-0365 | 2 | 73115407 | 73498056 | 2.77033982 | 6 | amp |
| TCGA-09-0365 | 2 | 73635692 | 74002225 | 1.283171469 | 5 | amp |
| TCGA-09-0365 | 2 | 74005405 | 74072401 | 1.978240435 | 6 | amp |
| TCGA-09-0365 | 2 | 74074456 | 74185909 | 1.561069558 | 5 | amp |
| TCGA-09-0365 | 2 | 74273413 | 75426105 | 2.668459206 | 6 | amp |
| TCGA-09-0365 | 2 | 75720278 | 85276809 | 1.540998587 | 5 | amp |
| TCGA-09-0365 | 2 | 85280221 | 86014070 | 2.430624689 | 6 | amp |
| TCGA-09-0365 | 2 | 86067210 | 86090666 | 1.320567682 | 5 | amp |
| TCGA-09-0365 | 2 | 86094673 | 86315800 | 2.366800931 | 6 | amp |
| TCGA-09-0365 | 2 | 86315860 | 90260280 | 1.405364561 | 5 | amp |
| TCGA-09-0365 | 2 | 90273672 | 92006318 | 2.002655154 | 6 | amp |
| TCGA-09-0365 | 2 | 92012985 | 95513950 | 1.409582378 | 5 | amp |
| TCGA-09-0365 | 2 | 95518913 | 96116883 | 2.715402847 | 6 | amp |
| TCGA-09-0365 | 2 | 96142852 | 96265380 | 1.491083955 | 5 | amp |
| TCGA-09-0365 | 2 | 96291348 | 96516991 | 2.37567711 | 6 | amp |

| | | | | | | |
|---|---|---|---|---|---|---|
| TCGA-09-0365 | 2 | 96517848 | 96679657 | 1.118625537 | 4 | amp |
| TCGA-09-0365 | 2 | 96680844 | 97019149 | 1.990818353 | 6 | amp |
| TCGA-09-0365 | 2 | 97019873 | 97285308 | 1.591062329 | 5 | amp |
| TCGA-09-0365 | 2 | 97285393 | 97784290 | 2.016684643 | 6 | amp |
| TCGA-09-0365 | 2 | 97845429 | 98177368 | 0.947275098 | 4 | amp |
| TCGA-09-0365 | 2 | 98195341 | 98201610 | 1.574592911 | 5 | amp |
| TCGA-09-0365 | 2 | 98201744 | 98275997 | 2.06936086 | 6 | amp |
| TCGA-09-0365 | 2 | 98276981 | 119729152 | 1.318593677 | 5 | amp |
| TCGA-09-0365 | 2 | 119731902 | 120252183 | 1.859385398 | 6 | amp |
| TCGA-09-0365 | 2 | 120362281 | 121555090 | 1.084903595 | 4 | amp |
| TCGA-09-0365 | 2 | 121684899 | 122135269 | 2.111720311 | 6 | amp |
| TCGA-09-0365 | 2 | 122139781 | 127447918 | 1.333127815 | 5 | amp |
| TCGA-09-0365 | 2 | 127451421 | 127961156 | 2.279927091 | 6 | amp |
| TCGA-09-0365 | 2 | 128015087 | 128317366 | 1.562791337 | 5 | amp |
| TCGA-09-0365 | 2 | 128321724 | 128467169 | 2.074576424 | 6 | amp |
| TCGA-09-0365 | 2 | 128467198 | 130872904 | 1.370548838 | 5 | amp |
| TCGA-09-0365 | 2 | 130877527 | 131223405 | 2.212878952 | 6 | amp |
| TCGA-09-0365 | 2 | 131231913 | 131261225 | 0.993864452 | 4 | amp |
| TCGA-09-0365 | 2 | 131261349 | 131374480 | 2.079077552 | 6 | amp |
| TCGA-09-0365 | 2 | 131374604 | 131403920 | 0.996095294 | 4 | amp |
| TCGA-09-0365 | 2 | 131412433 | 131813316 | 2.025518474 | 6 | amp |
| TCGA-09-0365 | 2 | 131829409 | 132219701 | 1.465511913 | 5 | amp |
| TCGA-09-0365 | 2 | 132235727 | 132354827 | 2.263298897 | 6 | amp |
| TCGA-09-0365 | 2 | 132357570 | 136664994 | 1.353956174 | 5 | amp |
| TCGA-09-0365 | 2 | 136668684 | 174104237 | 1.080309471 | 4 | amp |
| TCGA-09-0365 | 2 | 174123364 | 177135202 | 1.335531568 | 5 | amp |
| TCGA-09-0365 | 2 | 177161551 | 197750233 | 1.058388343 | 4 | amp |
| TCGA-09-0365 | 2 | 197750520 | 209142466 | 1.28291658 | 5 | amp |
| TCGA-09-0365 | 2 | 209150440 | 216274852 | 1.074110106 | 4 | amp |
| TCGA-09-0365 | 2 | 216279500 | 217364772 | 1.321348966 | 5 | amp |
| TCGA-09-0365 | 2 | 217366005 | 219300204 | 2.265132558 | 6 | amp |
| TCGA-09-0365 | 2 | 219301126 | 219677493 | 1.427312203 | 5 | amp |
| TCGA-09-0365 | 2 | 219677582 | 220506471 | 2.530623834 | 6 | amp |
| TCGA-09-0365 | 2 | 222290714 | 223783970 | 1.333561896 | 5 | amp |
| TCGA-09-0365 | 2 | 223785952 | 230695564 | 1.125581132 | 4 | amp |
| TCGA-09-0365 | 2 | 230701481 | 232156170 | 1.307128805 | 5 | amp |
| TCGA-09-0365 | 2 | 232160878 | 232577597 | 1.950155592 | 6 | amp |
| TCGA-09-0365 | 2 | 232578005 | 233245082 | 1.540965674 | 5 | amp |
| TCGA-09-0365 | 2 | 233245105 | 233537205 | 2.209453363 | 6 | amp |
| TCGA-09-0365 | 2 | 233546211 | 238940938 | 1.467020145 | 5 | amp |
| TCGA-09-0365 | 2 | 238944489 | 243037201 | 1.899773551 | 6 | amp |
| TCGA-09-0365 | 2 | 243061101 | 243160772 | 1.315632753 | 5 | amp |
| TCGA-09-0365 | 20 | 68319 | 239952 | 0.914265241 | 3 | amp |
| TCGA-09-0365 | 20 | 256517 | 464722 | 1.666242042 | 5 | amp |
| TCGA-09-0365 | 20 | 467002 | 489204 | 0.809893135 | 4 | amp |
| TCGA-09-0365 | 20 | 629309 | 2560708 | 1.388075023 | 5 | amp |
| TCGA-09-0365 | 20 | 2572944 | 2616670 | 0.92006832 | 4 | amp |
| TCGA-09-0365 | 20 | 2618028 | 2847267 | 1.740512051 | 5 | amp |
| TCGA-09-0365 | 20 | 2944899 | 2996572 | 1.080721387 | 4 | amp |
| TCGA-09-0365 | 20 | 2998420 | 3245186 | 1.699481308 | 5 | amp |

| | | | | | | |
|---|---|---|---|---|---|---|
| TCGA-09-0365 | 20 | 3251020 | 3624917 | 0.923756807 | 4 | amp |
| TCGA-09-0365 | 20 | 3649538 | 3846801 | 1.892451175 | 6 | amp |
| TCGA-09-0365 | 20 | 3888568 | 3944712 | 0.916957314 | 4 | amp |
| TCGA-09-0365 | 20 | 4155656 | 5149333 | 1.298047675 | 5 | amp |
| TCGA-09-0365 | 20 | 5154116 | 5566937 | 0.947961272 | 4 | amp |
| TCGA-09-0365 | 20 | 5573954 | 6100245 | 1.233197679 | 5 | amp |
| TCGA-09-0365 | 20 | 6194716 | 9353059 | 0.900938463 | 4 | amp |
| TCGA-09-0365 | 20 | 9353662 | 9386037 | 1.441488239 | 5 | amp |
| TCGA-09-0365 | 20 | 9388514 | 10622344 | 0.980518593 | 4 | amp |
| TCGA-09-0365 | 20 | 10622395 | 11899838 | 1.381310424 | 5 | amp |
| TCGA-09-0365 | 20 | 11900304 | 13714548 | 0.851193 | 4 | amp |
| TCGA-09-0365 | 20 | 13740299 | 13789577 | 1.400076508 | 5 | amp |
| TCGA-09-0365 | 20 | 13797090 | 16731811 | 0.830114326 | 3 | amp |
| TCGA-09-0365 | 20 | 17207888 | 17594905 | 1.299051954 | 5 | amp |
| TCGA-09-0365 | 20 | 17595333 | 17641187 | 1.770291361 | 6 | amp |
| TCGA-09-0365 | 20 | 17705613 | 18541399 | 1.110432111 | 4 | amp |
| TCGA-09-0365 | 20 | 18574359 | 20003171 | 1.221851063 | 5 | amp |
| TCGA-09-0365 | 20 | 20006275 | 21362753 | 0.95702308 | 4 | amp |
| TCGA-09-0365 | 20 | 21367424 | 25394517 | 1.528334487 | 5 | amp |
| TCGA-09-0365 | 20 | 25397698 | 25434302 | 0.926574779 | 4 | amp |
| TCGA-09-0365 | 20 | 25436305 | 25597248 | 1.709175839 | 5 | amp |
| TCGA-09-0365 | 20 | 25655650 | 30038024 | 1.026929985 | 4 | amp |
| TCGA-09-0365 | 20 | 30053237 | 30253940 | 1.72148741 | 5 | amp |
| TCGA-09-0365 | 20 | 30309409 | 30388887 | 1.03216241 | 4 | amp |
| TCGA-09-0365 | 20 | 30407868 | 32883415 | 1.526498635 | 5 | amp |
| TCGA-09-0365 | 20 | 32981592 | 33033298 | 0.773287881 | 3 | amp |
| TCGA-09-0365 | 20 | 33037190 | 33609195 | 1.47887241 | 5 | amp |
| TCGA-09-0365 | 20 | 33622898 | 33722766 | 0.968693659 | 4 | amp |
| TCGA-09-0365 | 20 | 33725625 | 33894585 | 1.672561252 | 5 | amp |
| TCGA-09-0365 | 20 | 33902439 | 33999814 | 0.867593708 | 4 | amp |
| TCGA-09-0365 | 20 | 34021633 | 34293259 | 1.47629717 | 5 | amp |
| TCGA-09-0365 | 20 | 34295022 | 34502156 | 0.911798026 | 4 | amp |
| TCGA-09-0365 | 20 | 34505344 | 35812799 | 1.404296478 | 5 | amp |
| TCGA-09-0365 | 20 | 35826787 | 35865167 | 0.838230037 | 4 | amp |
| TCGA-09-0365 | 20 | 35866768 | 36361524 | 1.473195351 | 5 | amp |
| TCGA-09-0365 | 20 | 36365742 | 36470840 | 0.76008685 | 3 | amp |
| TCGA-09-0365 | 20 | 36488219 | 37150360 | 1.488961942 | 5 | amp |
| TCGA-09-0365 | 20 | 37153384 | 37210075 | 0.877971446 | 4 | amp |
| TCGA-09-0365 | 20 | 37214670 | 37597910 | 1.621373403 | 5 | amp |
| TCGA-09-0365 | 20 | 37601187 | 39750442 | 1.005410774 | 4 | amp |
| TCGA-09-0365 | 20 | 39750570 | 39993855 | 1.675870285 | 5 | amp |
| TCGA-09-0365 | 20 | 40033200 | 40162248 | 0.897893086 | 4 | amp |
| TCGA-09-0365 | 20 | 40179899 | 43585136 | 1.567791666 | 5 | amp |
| TCGA-09-0365 | 20 | 43600698 | 43681828 | 0.780716623 | 4 | amp |
| TCGA-09-0365 | 20 | 43703587 | 43743741 | 1.74630087 | 5 | amp |
| TCGA-09-0365 | 20 | 43752392 | 43883201 | 0.972634435 | 4 | amp |
| TCGA-09-0365 | 20 | 43922511 | 44108777 | 1.689756063 | 5 | amp |
| TCGA-09-0365 | 20 | 44163034 | 44238834 | 0.891655165 | 4 | amp |
| TCGA-09-0365 | 20 | 44258437 | 45891221 | 1.658604006 | 5 | amp |
| TCGA-09-0365 | 20 | 45904968 | 46281865 | 0.995550189 | 4 | amp |

| | | | | | | |
|---|---|---|---|---|---|---|
| TCGA-09-0365 | 20 | 46282095 | 47592764 | 1.584126614 | 5 | amp |
| TCGA-09-0365 | 20 | 47601231 | 47634119 | 0.91871325 | 4 | amp |
| TCGA-09-0365 | 20 | 47635387 | 47679929 | 1.504807845 | 5 | amp |
| TCGA-09-0365 | 20 | 47682719 | 47782755 | 0.836178486 | 4 | amp |
| TCGA-09-0365 | 20 | 47835835 | 48166784 | 1.565491484 | 5 | amp |
| TCGA-09-0365 | 20 | 48252802 | 48497576 | 1.107902228 | 4 | amp |
| TCGA-09-0365 | 20 | 48500342 | 56886198 | 1.46171899 | 5 | amp |
| TCGA-09-0365 | 20 | 56918724 | 57019331 | 0.947056286 | 4 | amp |
| TCGA-09-0365 | 20 | 57035777 | 58425556 | 1.44558358 | 5 | amp |
| TCGA-09-0365 | 20 | 58439344 | 58482508 | 0.627511138 | 3 | amp |
| TCGA-09-0365 | 20 | 58486792 | 60573279 | 1.315306049 | 5 | amp |
| TCGA-09-0365 | 20 | 60573951 | 62926333 | 1.865608064 | 6 | amp |
| TCGA-09-0365 | 21 | 9483321 | 9590404 | 1.092429197 | 4 | amp |
| TCGA-09-0365 | 21 | 9755657 | 48056942 | 1.504706869 | 5 | amp |
| TCGA-09-0365 | 21 | 48063408 | 48111215 | 1.596981496 | 6 | amp |
| TCGA-09-0365 | 22 | 16084594 | 17688190 | 0.857285708 | 4 | amp |
| TCGA-09-0365 | 22 | 18220738 | 21088881 | 0.838151163 | 4 | amp |
| TCGA-09-0365 | 22 | 21322144 | 22330106 | 0.84372691 | 4 | amp |
| TCGA-09-0365 | 22 | 23252698 | 24645496 | 0.881247119 | 4 | amp |
| TCGA-09-0365 | 22 | 29438459 | 29763273 | 0.844775994 | 4 | amp |
| TCGA-09-0365 | 22 | 29938836 | 30218508 | 0.841924166 | 3 | amp |
| TCGA-09-0365 | 22 | 30639604 | 31302374 | 0.93103578 | 4 | amp |
| TCGA-09-0365 | 22 | 31345692 | 31796787 | 0.876156278 | 4 | amp |
| TCGA-09-0365 | 22 | 35689561 | 36013316 | 0.953599112 | 4 | amp |
| TCGA-09-0365 | 22 | 36653095 | 38870637 | 0.911809132 | 4 | amp |
| TCGA-09-0365 | 22 | 38964175 | 40078703 | 0.897957513 | 4 | amp |
| TCGA-09-0365 | 22 | 41601318 | 43870848 | 0.852703426 | 4 | amp |
| TCGA-09-0365 | 22 | 43924673 | 44645624 | 0.802154544 | 3 | amp |
| TCGA-09-0365 | 22 | 44681286 | 45724375 | 0.928498727 | 4 | amp |
| TCGA-09-0365 | 22 | 45813447 | 45973042 | 0.951833543 | 3 | amp |
| TCGA-09-0365 | 22 | 46239489 | 51237627 | 0.92393283 | 4 | amp |
| TCGA-09-0365 | 3 | 361444 | 9719764 | 0.802344795 | 3 | amp |
| TCGA-09-0365 | 3 | 9724804 | 11276168 | 1.200266639 | 5 | amp |
| TCGA-09-0365 | 3 | 11300675 | 12660236 | 0.823596875 | 3 | amp |
| TCGA-09-0365 | 3 | 12776192 | 14696044 | 1.268380833 | 5 | amp |
| TCGA-09-0365 | 3 | 14696995 | 37845493 | 0.714962763 | 3 | amp |
| TCGA-09-0365 | 3 | 37860340 | 38674841 | 1.225388586 | 5 | amp |
| TCGA-09-0365 | 3 | 38738786 | 46479645 | 0.932423709 | 3 | amp |
| TCGA-09-0365 | 3 | 46480758 | 48610531 | 1.230061142 | 5 | amp |
| TCGA-09-0365 | 3 | 48610547 | 48621540 | 2.270423107 | 6 | amp |
| TCGA-09-0365 | 3 | 48621709 | 49899663 | 1.269578389 | 5 | amp |
| TCGA-09-0365 | 3 | 49899683 | 50879185 | 3.328315243 | 6 | amp |
| TCGA-09-0365 | 3 | 50900801 | 51691700 | 1.755425251 | 5 | amp |
| TCGA-09-0365 | 3 | 51694027 | 52588937 | 3.300734574 | 6 | amp |
| TCGA-09-0365 | 3 | 52595733 | 52696340 | 1.38660842 | 5 | amp |
| TCGA-09-0365 | 3 | 52702467 | 52741885 | 2.49780235 | 6 | amp |
| TCGA-09-0365 | 3 | 52745748 | 52800016 | 1.697623184 | 5 | amp |
| TCGA-09-0365 | 3 | 52800172 | 54420079 | 2.532068911 | 6 | amp |
| TCGA-09-0365 | 3 | 54420711 | 62204710 | 1.308004801 | 5 | amp |
| TCGA-09-0365 | 3 | 62216881 | 90251665 | 0.963803227 | 3 | amp |

| | | | | | | |
|---|---|---|---|---|---|---|
| TCGA-09-0365 | 3 | 90257348 | 112711981 | 1.484408253 | 5 | amp |
| TCGA-09-0365 | 3 | 112713926 | 113005733 | 2.485265057 | 6 | amp |
| TCGA-09-0365 | 3 | 113081945 | 122598227 | 1.674809634 | 5 | amp |
| TCGA-09-0365 | 3 | 122628948 | 129304979 | 2.443043968 | 6 | amp |
| TCGA-09-0365 | 3 | 129370270 | 129547263 | 1.270995908 | 5 | amp |
| TCGA-09-0365 | 3 | 129695446 | 129822776 | 3.42509478 | 6 | amp |
| TCGA-09-0365 | 3 | 130159007 | 133472552 | 1.493842212 | 5 | amp |
| TCGA-09-0365 | 3 | 133473306 | 133698513 | 2.415848988 | 6 | amp |
| TCGA-09-0365 | 3 | 133876992 | 133941442 | 1.585457262 | 5 | amp |
| TCGA-09-0365 | 3 | 134076515 | 134090328 | 2.62247335 | 6 | amp |
| TCGA-09-0365 | 3 | 134197344 | 138033271 | 1.671152305 | 5 | amp |
| TCGA-09-0365 | 3 | 138036935 | 138191752 | 2.478690406 | 6 | amp |
| TCGA-09-0365 | 3 | 138192336 | 183801767 | 1.540986007 | 5 | amp |
| TCGA-09-0365 | 3 | 183818145 | 184429622 | 3.459073891 | 6 | amp |
| TCGA-09-0365 | 3 | 184542376 | 194309372 | 1.619416397 | 5 | amp |
| TCGA-09-0365 | 3 | 194313695 | 195000166 | 2.554284645 | 6 | amp |
| TCGA-09-0365 | 3 | 195006495 | 195346160 | 1.474376924 | 5 | amp |
| TCGA-09-0365 | 3 | 195346298 | 196054480 | 2.562840465 | 6 | amp |
| TCGA-09-0365 | 3 | 196083516 | 196214479 | 1.709609171 | 5 | amp |
| TCGA-09-0365 | 3 | 196215455 | 196457972 | 2.345445143 | 6 | amp |
| TCGA-09-0365 | 3 | 196460583 | 196666332 | 1.573923249 | 5 | amp |
| TCGA-09-0365 | 3 | 196674011 | 196746693 | 2.698204184 | 6 | amp |
| TCGA-09-0365 | 3 | 196749755 | 197955154 | 1.730679849 | 5 | amp |
| TCGA-09-0365 | 4 | 53323 | 466440 | 0.861119489 | 4 | amp |
| TCGA-09-0365 | 4 | 494167 | 1903039 | 1.263575781 | 5 | amp |
| TCGA-09-0365 | 4 | 1905903 | 2514364 | 0.986946966 | 4 | amp |
| TCGA-09-0365 | 4 | 2514773 | 2695636 | 0.772303198 | 3 | amp |
| TCGA-09-0365 | 4 | 2696644 | 2834806 | 1.211722186 | 5 | amp |
| TCGA-09-0365 | 4 | 2877589 | 2909580 | 0.772645014 | 3 | amp |
| TCGA-09-0365 | 4 | 2910226 | 2959519 | 1.227627994 | 5 | amp |
| TCGA-09-0365 | 4 | 2986197 | 3123176 | 1.012496819 | 4 | amp |
| TCGA-09-0365 | 4 | 3124579 | 3158982 | 0.751228332 | 3 | amp |
| TCGA-09-0365 | 4 | 3174025 | 6296961 | 1.074581113 | 4 | amp |
| TCGA-09-0365 | 4 | 6302329 | 8621346 | 1.263108226 | 5 | amp |
| TCGA-09-0365 | 4 | 9024980 | 9222074 | 0.9650584 | 4 | amp |
| TCGA-09-0365 | 4 | 9222193 | 9356519 | 0.828570293 | 3 | amp |
| TCGA-09-0365 | 4 | 9356759 | 10503077 | 0.942763769 | 4 | amp |
| TCGA-09-0365 | 4 | 10509561 | 17493968 | 0.74400469 | 3 | amp |
| TCGA-09-0365 | 4 | 17503291 | 17832799 | 0.96019533 | 4 | amp |
| TCGA-09-0365 | 4 | 17835883 | 17836183 | 0.689167862 | 3 | amp |
| TCGA-09-0365 | 4 | 24572253 | 26614905 | 0.758011314 | 3 | amp |
| TCGA-09-0365 | 4 | 36216012 | 38893467 | 0.815656211 | 3 | amp |
| TCGA-09-0365 | 4 | 39435784 | 39843681 | 0.82773603 | 3 | amp |
| TCGA-09-0365 | 4 | 40133329 | 44724282 | 0.75314123 | 3 | amp |
| TCGA-09-0365 | 4 | 48894727 | 52883831 | 0.784598201 | 3 | amp |
| TCGA-09-0365 | 4 | 52890074 | 54280895 | 1.04788595 | 4 | amp |
| TCGA-09-0365 | 4 | 54292017 | 55968721 | 0.838910053 | 3 | amp |
| TCGA-09-0365 | 4 | 55970800 | 57164575 | 0.935075673 | 4 | amp |
| TCGA-09-0365 | 4 | 57173662 | 57865984 | 1.188744079 | 5 | amp |
| TCGA-09-0365 | 4 | 57871389 | 57883919 | 0.925320001 | 4 | amp |

| | | | | | | |
|---|---|---|---|---|---|---|
| TCGA-09-0365 | 4 | 57887052 | 69434260 | 0.828842869 | 3 | amp |
| TCGA-09-0365 | 4 | 70592801 | 71532235 | 0.813746572 | 3 | amp |
| TCGA-09-0365 | 4 | 71554379 | 71889469 | 0.945308707 | 4 | amp |
| TCGA-09-0365 | 4 | 71891500 | 73414637 | 0.762516287 | 3 | amp |
| TCGA-09-0365 | 4 | 73433044 | 73968349 | 0.985249776 | 4 | amp |
| TCGA-09-0365 | 4 | 73979474 | 74459358 | 0.811518682 | 3 | amp |
| TCGA-09-0365 | 4 | 74464335 | 75040450 | 1.030052219 | 4 | amp |
| TCGA-09-0365 | 4 | 75040897 | 75178919 | 0.672954831 | 3 | amp |
| TCGA-09-0365 | 4 | 75230998 | 76584127 | 1.025072806 | 4 | amp |
| TCGA-09-0365 | 4 | 76587101 | 76788578 | 0.794791585 | 3 | amp |
| TCGA-09-0365 | 4 | 76793128 | 77234420 | 0.953173437 | 4 | amp |
| TCGA-09-0365 | 4 | 77244448 | 77324385 | 0.762010356 | 3 | amp |
| TCGA-09-0365 | 4 | 77357170 | 77692094 | 1.044026961 | 4 | amp |
| TCGA-09-0365 | 4 | 77699905 | 78873821 | 0.843572096 | 3 | amp |
| TCGA-09-0365 | 4 | 78979141 | 79494286 | 0.928745656 | 4 | amp |
| TCGA-09-0365 | 4 | 79494317 | 79845115 | 0.770834628 | 3 | amp |
| TCGA-09-0365 | 4 | 79847623 | 81257037 | 0.965332924 | 4 | amp |
| TCGA-09-0365 | 4 | 81283822 | 82064144 | 0.730157497 | 3 | amp |
| TCGA-09-0365 | 4 | 82065374 | 85678316 | 0.934547921 | 4 | amp |
| TCGA-09-0365 | 4 | 85686951 | 99363324 | 0.837010551 | 3 | amp |
| TCGA-09-0365 | 4 | 99393615 | 101111154 | 0.935440096 | 4 | amp |
| TCGA-09-0365 | 4 | 101331434 | 103870618 | 0.809957809 | 3 | amp |
| TCGA-09-0365 | 4 | 103910907 | 104044256 | 0.988874642 | 4 | amp |
| TCGA-09-0365 | 4 | 104053831 | 108829923 | 0.853322507 | 3 | amp |
| TCGA-09-0365 | 4 | 108831487 | 109684086 | 1.096904024 | 4 | amp |
| TCGA-09-0365 | 4 | 109735013 | 109895731 | 0.760783255 | 3 | amp |
| TCGA-09-0365 | 4 | 109931462 | 110459760 | 0.967248725 | 4 | amp |
| TCGA-09-0365 | 4 | 110460713 | 110678988 | 1.231098657 | 5 | amp |
| TCGA-09-0365 | 4 | 110681420 | 110754509 | 0.964418394 | 4 | amp |
| TCGA-09-0365 | 4 | 110756511 | 111480901 | 0.82056273 | 3 | amp |
| TCGA-09-0365 | 4 | 111482517 | 114257227 | 0.970634033 | 4 | amp |
| TCGA-09-0365 | 4 | 114257714 | 119148148 | 0.834440787 | 3 | amp |
| TCGA-09-0365 | 4 | 119154108 | 119626990 | 0.970251486 | 4 | amp |
| TCGA-09-0365 | 4 | 119631124 | 119689516 | 0.755158681 | 3 | amp |
| TCGA-09-0365 | 4 | 119718781 | 120240280 | 1.024319726 | 4 | amp |
| TCGA-09-0365 | 4 | 120240651 | 120463821 | 0.752760862 | 3 | amp |
| TCGA-09-0365 | 4 | 120473688 | 122756488 | 0.981285875 | 4 | amp |
| TCGA-09-0365 | 4 | 122760728 | 123222563 | 0.831996057 | 3 | amp |
| TCGA-09-0365 | 4 | 123541962 | 123542202 | 0.707376507 | 3 | amp |
| TCGA-09-0365 | 4 | 123663033 | 126315184 | 1.012217459 | 4 | amp |
| TCGA-09-0365 | 4 | 126319884 | 128637595 | 0.770523867 | 3 | amp |
| TCGA-09-0365 | 4 | 128651696 | 142642375 | 0.962501493 | 4 | amp |
| TCGA-09-0365 | 4 | 142643058 | 144387436 | 0.796313968 | 3 | amp |
| TCGA-09-0365 | 4 | 144390142 | 144474364 | 0.970072741 | 4 | amp |
| TCGA-09-0365 | 4 | 144797897 | 146077177 | 0.825484386 | 3 | amp |
| TCGA-09-0365 | 4 | 146080598 | 148407283 | 1.015616288 | 4 | amp |
| TCGA-09-0365 | 4 | 148440946 | 148575719 | 0.759009399 | 3 | amp |
| TCGA-09-0365 | 4 | 148578914 | 151207195 | 1.006687803 | 4 | amp |
| TCGA-09-0365 | 4 | 151223740 | 151604905 | 0.862722345 | 3 | amp |
| TCGA-09-0365 | 4 | 151849627 | 151850221 | 0.71415161 | 3 | amp |

| | | | | | | |
|---|---|---|---|---|---|---|
| TCGA-09-0365 | 4 | 151935566 | 152682166 | 1.034138587 | 4 | amp |
| TCGA-09-0365 | 4 | 153243986 | 153549716 | 0.651981221 | 3 | amp |
| TCGA-09-0365 | 4 | 153562048 | 154502076 | 1.044073666 | 4 | amp |
| TCGA-09-0365 | 4 | 154502515 | 154525627 | 0.758575406 | 3 | amp |
| TCGA-09-0365 | 4 | 154533357 | 154710036 | 1.070884296 | 4 | amp |
| TCGA-09-0365 | 4 | 155155679 | 156787418 | 0.848374619 | 3 | amp |
| TCGA-09-0365 | 4 | 156824867 | 156864421 | 1.005999755 | 4 | amp |
| TCGA-09-0365 | 4 | 157684179 | 159048782 | 0.80212462 | 3 | amp |
| TCGA-09-0365 | 4 | 159051930 | 160243686 | 0.955178979 | 4 | amp |
| TCGA-09-0365 | 4 | 160244569 | 164067034 | 0.842592298 | 3 | amp |
| TCGA-09-0365 | 4 | 164069479 | 164416014 | 1.306457067 | 5 | amp |
| TCGA-09-0365 | 4 | 164428180 | 166914055 | 0.971905508 | 4 | amp |
| TCGA-09-0365 | 4 | 166915458 | 169227880 | 0.765173956 | 3 | amp |
| TCGA-09-0365 | 4 | 169229131 | 176556251 | 0.958199971 | 4 | amp |
| TCGA-09-0365 | 4 | 176561222 | 177071796 | 0.785881842 | 3 | amp |
| TCGA-09-0365 | 4 | 177072920 | 178283653 | 0.97645271 | 4 | amp |
| TCGA-09-0365 | 4 | 178352851 | 178361606 | 1.349126751 | 5 | amp |
| TCGA-09-0365 | 4 | 178881971 | 187560966 | 1.047960415 | 4 | amp |
| TCGA-09-0365 | 4 | 187584429 | 191010188 | 1.184855745 | 5 | amp |
| TCGA-09-0365 | 5 | 151610 | 895104 | 1.142528557 | 4 | amp |
| TCGA-09-0365 | 5 | 896724 | 1060572 | 0.681098084 | 3 | amp |
| TCGA-09-0365 | 5 | 1065345 | 1225792 | 1.604807047 | 5 | amp |
| TCGA-09-0365 | 5 | 1232814 | 10394910 | 1.02925339 | 4 | amp |
| TCGA-09-0365 | 5 | 10397370 | 11397348 | 0.818102706 | 3 | amp |
| TCGA-09-0365 | 5 | 13900297 | 14331067 | 0.775605278 | 3 | amp |
| TCGA-09-0365 | 5 | 14336590 | 17354033 | 0.907799276 | 4 | amp |
| TCGA-09-0365 | 5 | 31449336 | 34954858 | 0.774563679 | 3 | amp |
| TCGA-09-0365 | 5 | 37180912 | 38493917 | 0.771685566 | 3 | amp |
| TCGA-09-0365 | 5 | 41850086 | 45267392 | 0.764641724 | 3 | amp |
| TCGA-09-0365 | 5 | 66438244 | 68881532 | 0.805356226 | 3 | amp |
| TCGA-09-0365 | 5 | 70798401 | 74001154 | 0.762842489 | 3 | amp |
| TCGA-09-0365 | 5 | 75996849 | 76029333 | 0.822563305 | 3 | amp |
| TCGA-09-0365 | 5 | 76128470 | 76351439 | 1.033670293 | 4 | amp |
| TCGA-09-0365 | 5 | 79057586 | 79089466 | 0.783641999 | 3 | amp |
| TCGA-09-0365 | 5 | 79095135 | 79443147 | 0.937074457 | 4 | amp |
| TCGA-09-0365 | 5 | 79446677 | 81283503 | 0.732652606 | 3 | amp |
| TCGA-09-0365 | 5 | 114598358 | 115299021 | 0.850328253 | 3 | amp |
| TCGA-09-0365 | 5 | 123966326 | 127450385 | 0.770869323 | 3 | amp |
| TCGA-09-0365 | 5 | 131283282 | 131298419 | 0.805952309 | 3 | amp |
| TCGA-09-0365 | 5 | 131302046 | 131893200 | 1.045575762 | 4 | amp |
| TCGA-09-0365 | 5 | 131894957 | 132088669 | 0.713111722 | 3 | amp |
| TCGA-09-0365 | 5 | 132094136 | 132101280 | 1.338400865 | 5 | amp |
| TCGA-09-0365 | 5 | 132158897 | 132433035 | 0.783771995 | 3 | amp |
| TCGA-09-0365 | 5 | 132435230 | 134002719 | 0.935585353 | 4 | amp |
| TCGA-09-0365 | 5 | 134007477 | 134147580 | 0.728813415 | 3 | amp |
| TCGA-09-0365 | 5 | 134152056 | 135416356 | 0.9136596 | 4 | amp |
| TCGA-09-0365 | 5 | 135489410 | 137542424 | 0.753772315 | 3 | amp |
| TCGA-09-0365 | 5 | 137548665 | 139828932 | 1.019088608 | 4 | amp |
| TCGA-09-0365 | 5 | 139838148 | 139919033 | 0.673854797 | 3 | amp |
| TCGA-09-0365 | 5 | 139921721 | 140058769 | 1.352722098 | 5 | amp |

| | | | | | | |
|---|---|---|---|---|---|---|
| TCGA-09-0365 | 5 | 140059360 | 140914010 | 1.105807297 | 4 | amp |
| TCGA-09-0365 | 5 | 140950938 | 140966806 | 0.719504921 | 3 | amp |
| TCGA-09-0365 | 5 | 140967743 | 141359921 | 1.474440623 | 5 | amp |
| TCGA-09-0365 | 5 | 141362881 | 141391658 | 1.009519577 | 4 | amp |
| TCGA-09-0365 | 5 | 141511326 | 147015875 | 0.761220658 | 3 | amp |
| TCGA-09-0365 | 5 | 147803500 | 148016624 | 0.737119917 | 3 | amp |
| TCGA-09-0365 | 5 | 148206325 | 149509545 | 1.03800666 | 4 | amp |
| TCGA-09-0365 | 5 | 149510013 | 150080580 | 1.320153986 | 5 | amp |
| TCGA-09-0365 | 5 | 150090697 | 151169984 | 1.047328357 | 4 | amp |
| TCGA-09-0365 | 5 | 151170536 | 153407895 | 0.760981708 | 3 | amp |
| TCGA-09-0365 | 5 | 153409004 | 157182306 | 0.926132447 | 4 | amp |
| TCGA-09-0365 | 5 | 157214646 | 158621837 | 0.724412384 | 3 | amp |
| TCGA-09-0365 | 5 | 158630414 | 159912467 | 0.883417205 | 4 | amp |
| TCGA-09-0365 | 5 | 159992443 | 161580416 | 0.670007721 | 3 | amp |
| TCGA-09-0365 | 5 | 162866184 | 169021343 | 0.942601039 | 4 | amp |
| TCGA-09-0365 | 5 | 169021356 | 169454969 | 0.678685099 | 3 | amp |
| TCGA-09-0365 | 5 | 169461330 | 170235799 | 0.968043979 | 4 | amp |
| TCGA-09-0365 | 5 | 170236545 | 170692887 | 0.693648849 | 3 | amp |
| TCGA-09-0365 | 5 | 170720875 | 175775114 | 0.931023252 | 4 | amp |
| TCGA-09-0365 | 5 | 175775215 | 175837340 | 1.236071032 | 5 | amp |
| TCGA-09-0365 | 5 | 175906162 | 175933979 | 0.720431575 | 3 | amp |
| TCGA-09-0365 | 5 | 175992289 | 176323172 | 1.339777257 | 5 | amp |
| TCGA-09-0365 | 5 | 176332267 | 176524722 | 1.041237773 | 4 | amp |
| TCGA-09-0365 | 5 | 176562087 | 176715932 | 0.746599737 | 3 | amp |
| TCGA-09-0365 | 5 | 176718906 | 177036738 | 1.342562337 | 5 | amp |
| TCGA-09-0365 | 5 | 177053411 | 177180252 | 0.721111618 | 3 | amp |
| TCGA-09-0365 | 5 | 177303491 | 180432902 | 1.101904525 | 4 | amp |
| TCGA-09-0365 | 5 | 180477048 | 180899507 | 1.222127491 | 5 | amp |
| TCGA-09-0365 | 6 | 105907 | 29524131 | 0.967200804 | 4 | amp |
| TCGA-09-0365 | 6 | 29527374 | 32191657 | 1.545794246 | 5 | amp |
| TCGA-09-0365 | 6 | 32260716 | 32709321 | 0.883070903 | 4 | amp |
| TCGA-09-0365 | 6 | 32712879 | 33054065 | 1.198921212 | 5 | amp |
| TCGA-09-0365 | 6 | 33095674 | 33153576 | 1.774919261 | 6 | amp |
| TCGA-09-0365 | 6 | 33154349 | 34512254 | 1.50227105 | 5 | amp |
| TCGA-09-0365 | 6 | 34558299 | 34985842 | 0.896282122 | 4 | amp |
| TCGA-09-0365 | 6 | 35021848 | 35480493 | 1.330615868 | 5 | amp |
| TCGA-09-0365 | 6 | 35543605 | 36569812 | 1.000553771 | 4 | amp |
| TCGA-09-0365 | 6 | 36641600 | 37631932 | 1.202758844 | 5 | amp |
| TCGA-09-0365 | 6 | 37897642 | 39868039 | 0.89491539 | 4 | amp |
| TCGA-09-0365 | 6 | 39869042 | 39884013 | 1.210343716 | 5 | amp |
| TCGA-09-0365 | 6 | 40359626 | 42237353 | 1.819539815 | 6 | amp |
| TCGA-09-0365 | 6 | 42541391 | 42638510 | 0.718257157 | 3 | amp |
| TCGA-09-0365 | 6 | 42641516 | 42833210 | 1.395175033 | 5 | amp |
| TCGA-09-0365 | 6 | 42847592 | 44311021 | 1.976489592 | 6 | amp |
| TCGA-09-0365 | 6 | 44320386 | 52130967 | 0.987888893 | 4 | amp |
| TCGA-09-0365 | 6 | 52131361 | 52962633 | 1.249541162 | 5 | amp |
| TCGA-09-0365 | 6 | 52992923 | 62390976 | 0.913645261 | 4 | amp |
| TCGA-09-0365 | 6 | 62407068 | 64290140 | 0.768843368 | 3 | amp |
| TCGA-09-0365 | 6 | 73835082 | 74407328 | 0.836901613 | 3 | amp |
| TCGA-09-0365 | 6 | 89793423 | 90406305 | 0.806823306 | 3 | amp |

| | | | | | | |
|---|---|---|---|---|---|---|
| TCGA-09-0365 | 6 | 108246018 | 109820464 | 0.861035419 | 3 | amp |
| TCGA-09-0365 | 6 | 148792549 | 151785790 | 0.820259951 | 3 | amp |
| TCGA-09-0365 | 6 | 155532329 | 160679822 | 0.876506955 | 3 | amp |
| TCGA-09-0365 | 6 | 161518099 | 168347603 | 0.781967647 | 3 | amp |
| TCGA-09-0365 | 6 | 168348518 | 171055029 | 1.014200206 | 4 | amp |
| TCGA-09-0365 | 7 | 540048 | 22349673 | 1.418855106 | 5 | amp |
| TCGA-09-0365 | 7 | 22532146 | 27497851 | 5.31753767 | 6 | amp |
| TCGA-09-0365 | 7 | 27565761 | 158935247 | 1.507629503 | 5 | amp |
| TCGA-09-0365 | 8 | 116074 | 642664 | 1.4309372 | 5 | amp |
| TCGA-09-0365 | 8 | 665813 | 2089141 | 1.827543327 | 6 | amp |
| TCGA-09-0365 | 8 | 2090250 | 6614916 | 1.157699091 | 4 | amp |
| TCGA-09-0365 | 8 | 6673323 | 7147503 | 1.917374088 | 6 | amp |
| TCGA-09-0365 | 8 | 7147863 | 7421932 | 1.414312443 | 5 | amp |
| TCGA-09-0365 | 8 | 7429457 | 7629569 | 1.77362526 | 6 | amp |
| TCGA-09-0365 | 8 | 7669280 | 11996312 | 1.503387558 | 5 | amp |
| TCGA-09-0365 | 8 | 12032443 | 12291651 | 2.082791096 | 6 | amp |
| TCGA-09-0365 | 8 | 12435418 | 12973190 | 1.542445277 | 5 | amp |
| TCGA-09-0365 | 8 | 13072058 | 19266224 | 1.081639001 | 4 | amp |
| TCGA-09-0365 | 8 | 19276153 | 21861597 | 1.367135607 | 5 | amp |
| TCGA-09-0365 | 8 | 21862492 | 22105565 | 2.307855082 | 6 | amp |
| TCGA-09-0365 | 8 | 22105578 | 22397056 | 1.339354427 | 5 | amp |
| TCGA-09-0365 | 8 | 22398101 | 23539182 | 1.899562861 | 6 | amp |
| TCGA-09-0365 | 8 | 23702237 | 23712097 | 1.450900779 | 5 | amp |
| TCGA-09-0365 | 8 | 24151625 | 24366118 | 0.963959472 | 4 | amp |
| TCGA-09-0365 | 8 | 24773059 | 28645435 | 1.401079052 | 5 | amp |
| TCGA-09-0365 | 8 | 28651263 | 32621952 | 1.134521595 | 4 | amp |
| TCGA-09-0365 | 8 | 33230088 | 33311060 | 1.24230934 | 5 | amp |
| TCGA-09-0365 | 8 | 33318869 | 33455042 | 1.788555441 | 6 | amp |
| TCGA-09-0365 | 8 | 33827032 | 35407038 | 1.224380899 | 5 | amp |
| TCGA-09-0365 | 8 | 35425537 | 36793474 | 1.001368653 | 4 | amp |
| TCGA-09-0365 | 8 | 37556009 | 37595553 | 1.353558858 | 5 | amp |
| TCGA-09-0365 | 8 | 37597833 | 38146319 | 1.85820154 | 6 | amp |
| TCGA-09-0365 | 8 | 38146906 | 38184430 | 1.333802458 | 5 | amp |
| TCGA-09-0365 | 8 | 38186853 | 38646339 | 1.897709441 | 6 | amp |
| TCGA-09-0365 | 8 | 38676996 | 38700919 | 1.302601362 | 5 | amp |
| TCGA-09-0365 | 8 | 38704156 | 38865513 | 1.978730206 | 6 | amp |
| TCGA-09-0365 | 8 | 38869145 | 39080777 | 1.265551232 | 5 | amp |
| TCGA-09-0365 | 8 | 39089539 | 39679202 | 0.961604492 | 4 | amp |
| TCGA-09-0365 | 8 | 39682316 | 40554961 | 1.329840663 | 5 | amp |
| TCGA-09-0365 | 8 | 40625144 | 41791828 | 2.123948363 | 6 | amp |
| TCGA-09-0365 | 8 | 41792077 | 41845116 | 1.241161496 | 5 | amp |
| TCGA-09-0365 | 8 | 41905835 | 42317521 | 1.840010772 | 6 | amp |
| TCGA-09-0365 | 8 | 42320505 | 53050071 | 1.466052669 | 5 | amp |
| TCGA-09-0365 | 8 | 53055457 | 53596629 | 0.991089679 | 4 | amp |
| TCGA-09-0365 | 8 | 53597933 | 68004137 | 1.304207491 | 5 | amp |
| TCGA-09-0365 | 8 | 68005766 | 69688748 | 0.995437705 | 4 | amp |
| TCGA-09-0365 | 8 | 69699664 | 75276650 | 1.27562254 | 5 | amp |
| TCGA-09-0365 | 8 | 75737410 | 79652336 | 1.049641245 | 4 | amp |
| TCGA-09-0365 | 8 | 79672321 | 88364027 | 1.346791738 | 5 | amp |
| TCGA-09-0365 | 8 | 88365818 | 91075721 | 1.049364539 | 4 | amp |

| | | | | | | |
|---|---|---|---|---|---|---|
| TCGA-09-0365 | 8 | 91078074 | 95161156 | 1.265329487 | 5 | amp |
| TCGA-09-0365 | 8 | 95164037 | 95556172 | 1.029022538 | 4 | amp |
| TCGA-09-0365 | 8 | 95565553 | 97285662 | 1.300092371 | 5 | amp |
| TCGA-09-0365 | 8 | 97296268 | 100050842 | 1.096528996 | 4 | amp |
| TCGA-09-0365 | 8 | 100108483 | 100847986 | 0.75694058 | 3 | amp |
| TCGA-09-0365 | 8 | 100860942 | 133858216 | 1.049270982 | 4 | amp |
| TCGA-09-0365 | 8 | 133879218 | 139768109 | 1.368273723 | 5 | amp |
| TCGA-09-0365 | 8 | 139774592 | 141595433 | 1.769104296 | 6 | amp |
| TCGA-09-0365 | 8 | 141669550 | 142148238 | 1.153049307 | 4 | amp |
| TCGA-09-0365 | 8 | 142151242 | 146279593 | 2.002774632 | 6 | amp |
| TCGA-09-0365 | 9 | 17322 | 135064 | 0.910305806 | 3 | amp |
| TCGA-09-0365 | 9 | 32526021 | 34372948 | 0.708622635 | 3 | amp |
| TCGA-09-0365 | 9 | 34379066 | 35108304 | 0.916315197 | 4 | amp |
| TCGA-09-0365 | 9 | 35546505 | 35826219 | 1.024895006 | 4 | amp |
| TCGA-09-0365 | 9 | 35842364 | 65635954 | 0.679869857 | 3 | amp |
| TCGA-09-0365 | 9 | 65647173 | 67930504 | 1.047387877 | 4 | amp |
| TCGA-09-0365 | 9 | 67930630 | 88650585 | 0.716903089 | 3 | amp |
| TCGA-09-0365 | 9 | 88651222 | 93976728 | 0.929364058 | 4 | amp |
| TCGA-09-0365 | 9 | 93978304 | 95280182 | 0.72801853 | 3 | amp |
| TCGA-09-0365 | 9 | 95284841 | 98643602 | 0.980733037 | 4 | amp |
| TCGA-09-0365 | 9 | 98740299 | 101830954 | 0.932819361 | 4 | amp |
| TCGA-09-0365 | 9 | 101831911 | 116041437 | 0.703589169 | 3 | amp |
| TCGA-09-0365 | 9 | 116044909 | 116116634 | 0.980511562 | 4 | amp |
| TCGA-09-0365 | 9 | 116122781 | 117386744 | 1.248009204 | 5 | amp |
| TCGA-09-0365 | 9 | 117389142 | 123688403 | 0.861417056 | 4 | amp |
| TCGA-09-0365 | 9 | 123714931 | 123954535 | 0.654977659 | 3 | amp |
| TCGA-09-0365 | 9 | 123955604 | 125747033 | 1.021337202 | 4 | amp |
| TCGA-09-0365 | 9 | 125748475 | 125946471 | 0.657324414 | 3 | amp |
| TCGA-09-0365 | 9 | 126125095 | 130117783 | 0.98460412 | 4 | amp |
| TCGA-09-0365 | 9 | 130119456 | 131258429 | 1.409016719 | 5 | amp |
| TCGA-09-0365 | 9 | 131260689 | 131392524 | 0.978482788 | 4 | amp |
| TCGA-09-0365 | 9 | 131392536 | 131718730 | 1.422225472 | 5 | amp |
| TCGA-09-0365 | 9 | 131719210 | 131760528 | 0.997734843 | 4 | amp |
| TCGA-09-0365 | 9 | 131760799 | 132686241 | 1.375965872 | 5 | amp |
| TCGA-09-0365 | 9 | 132687217 | 132891099 | 0.868203589 | 4 | amp |
| TCGA-09-0365 | 9 | 132897291 | 133995725 | 1.330002202 | 5 | amp |
| TCGA-09-0365 | 9 | 134002858 | 134098360 | 0.69547492 | 3 | amp |
| TCGA-09-0365 | 9 | 134103512 | 134769385 | 1.285881469 | 5 | amp |
| TCGA-09-0365 | 9 | 134814754 | 135862857 | 0.90166083 | 4 | amp |
| TCGA-09-0365 | 9 | 135863539 | 141071671 | 1.423043595 | 5 | amp |
| TCGA-09-0365 | X | 200797 | 10153264 | 0.70948082 | 3 | amp |
| TCGA-09-0365 | X | 13057816 | 13765105 | 0.676533263 | 3 | amp |
| TCGA-09-0365 | X | 18911567 | 19024231 | 0.691318531 | 3 | amp |
| TCGA-09-0365 | X | 19367399 | 19389675 | 0.717168791 | 3 | amp |
| TCGA-09-0365 | X | 23685824 | 23855392 | 0.680441076 | 3 | amp |
| TCGA-09-0365 | X | 46940504 | 47106820 | 0.91430776 | 4 | amp |
| TCGA-09-0365 | X | 47106993 | 48025262 | 0.758436792 | 3 | amp |
| TCGA-09-0365 | X | 48317309 | 48325282 | 0.759267062 | 3 | amp |
| TCGA-09-0365 | X | 48325346 | 48776140 | 0.911643516 | 4 | amp |
| TCGA-09-0365 | X | 48780391 | 48891328 | 0.787343307 | 3 | amp |

| | | | | | | |
|---|---|---|---|---|---|---|
| TCGA-09-0365 | X | 48891587 | 49055519 | 0.932479084 | 4 | amp |
| TCGA-09-0365 | X | 49061591 | 49079611 | 1.193591998 | 5 | amp |
| TCGA-09-0365 | X | 49081215 | 49111989 | 0.901874038 | 4 | amp |
| TCGA-09-0365 | X | 49113199 | 49368453 | 0.719030509 | 3 | amp |
| TCGA-09-0365 | X | 50136121 | 51935217 | 0.724365679 | 3 | amp |
| TCGA-09-0365 | X | 52789410 | 53570985 | 0.677325538 | 3 | amp |
| TCGA-09-0365 | X | 54467107 | 54959925 | 0.683808686 | 3 | amp |
| TCGA-09-0365 | X | 67935116 | 68836590 | 0.999323676 | 4 | amp |
| TCGA-09-0365 | X | 68837519 | 69496372 | 0.692629592 | 3 | amp |
| TCGA-09-0365 | X | 69496492 | 69507215 | 0.951568145 | 4 | amp |
| TCGA-09-0365 | X | 69507570 | 70518671 | 0.74932586 | 3 | amp |
| TCGA-09-0365 | X | 70641132 | 71549603 | 0.660294955 | 3 | amp |
| TCGA-09-0365 | X | 100537257 | 103499549 | 0.721540391 | 3 | amp |
| TCGA-09-0365 | X | 117900465 | 119438394 | 0.682348261 | 3 | amp |
| TCGA-09-0365 | X | 119671931 | 120007931 | 0.638501372 | 3 | amp |
| TCGA-09-0365 | X | 120009329 | 120119075 | 1.191718891 | 5 | amp |
| TCGA-09-0365 | X | 120119188 | 122319882 | 0.745409701 | 3 | amp |
| TCGA-09-0365 | X | 128641875 | 129318726 | 0.709956423 | 3 | amp |
| TCGA-09-0365 | X | 130215555 | 130423278 | 0.751599305 | 3 | amp |
| TCGA-09-0365 | X | 134852665 | 135068003 | 0.747869579 | 3 | amp |
| TCGA-09-0365 | X | 148564242 | 149678311 | 0.667255303 | 3 | amp |
| TCGA-09-0365 | X | 150840908 | 151304146 | 0.688344118 | 3 | amp |
| TCGA-09-0365 | X | 151815368 | 153770716 | 1.000329573 | 4 | amp |
| TCGA-09-0365 | X | 153774164 | 154132391 | 0.706663748 | 3 | amp |
| TCGA-09-0365 | X | 155227378 | 155252897 | 0.742145607 | 3 | amp |
| TCGA-09-0366 | 1 | 16834 | 6706063 | 2.263913977 | 6 | amp |
| TCGA-09-0366 | 1 | 6711038 | 11562197 | 1.62827733 | 5 | amp |
| TCGA-09-0366 | 1 | 11562694 | 12033111 | 2.48065016 | 6 | amp |
| TCGA-09-0366 | 1 | 12049192 | 16355357 | 1.645848922 | 5 | amp |
| TCGA-09-0366 | 1 | 16356200 | 16475567 | 3.159621653 | 6 | amp |
| TCGA-09-0366 | 1 | 16477334 | 17303077 | 1.574165161 | 5 | amp |
| TCGA-09-0366 | 1 | 17303233 | 19441493 | 2.043687082 | 6 | amp |
| TCGA-09-0366 | 1 | 19441831 | 19514044 | 0.886461765 | 3 | amp |
| TCGA-09-0366 | 1 | 19518626 | 19581364 | 1.711405697 | 5 | amp |
| TCGA-09-0366 | 1 | 19582433 | 19651311 | 2.932986002 | 6 | amp |
| TCGA-09-0366 | 1 | 19651947 | 21809937 | 1.633506797 | 5 | amp |
| TCGA-09-0366 | 1 | 21880514 | 21936163 | 3.191790801 | 6 | amp |
| TCGA-09-0366 | 1 | 21936540 | 22160153 | 1.586624622 | 5 | amp |
| TCGA-09-0366 | 1 | 22161124 | 22313219 | 3.216616595 | 6 | amp |
| TCGA-09-0366 | 1 | 22315733 | 26090471 | 1.705887564 | 5 | amp |
| TCGA-09-0366 | 1 | 26098145 | 27661973 | 2.195644689 | 6 | amp |
| TCGA-09-0366 | 1 | 27673850 | 31842437 | 1.634713833 | 5 | amp |
| TCGA-09-0366 | 1 | 31845734 | 32044896 | 2.980792665 | 6 | amp |
| TCGA-09-0366 | 1 | 32048705 | 32119298 | 1.804482034 | 5 | amp |
| TCGA-09-0366 | 1 | 32119405 | 32696633 | 2.582235288 | 6 | amp |
| TCGA-09-0366 | 1 | 32696661 | 39761566 | 1.757081095 | 5 | amp |
| TCGA-09-0366 | 1 | 39763234 | 39920794 | 0.925034343 | 3 | amp |
| TCGA-09-0366 | 1 | 39923981 | 43825823 | 1.617130929 | 5 | amp |
| TCGA-09-0366 | 1 | 43825841 | 44433365 | 2.263345691 | 6 | amp |
| TCGA-09-0366 | 1 | 44435770 | 51831722 | 1.699465526 | 5 | amp |

| | | | | | | |
|---|---|---|---|---|---|---|
| TCGA-09-0366 | 1 | 51860025 | 52301903 | 0.756415921 | 3 | amp |
| TCGA-09-0366 | 1 | 52301985 | 52823632 | 1.551944335 | 5 | amp |
| TCGA-09-0366 | 1 | 52823947 | 52826278 | 3.750571082 | 6 | amp |
| TCGA-09-0366 | 1 | 52826574 | 52880367 | 1.698631312 | 5 | amp |
| TCGA-09-0366 | 1 | 52880392 | 52954763 | 0.680735422 | 3 | amp |
| TCGA-09-0366 | 1 | 52956378 | 53516418 | 1.31640843 | 5 | amp |
| TCGA-09-0366 | 1 | 53540219 | 53571514 | 3.072732317 | 6 | amp |
| TCGA-09-0366 | 1 | 53600001 | 54683943 | 1.730640554 | 5 | amp |
| TCGA-09-0366 | 1 | 54692728 | 55073797 | 2.749834923 | 6 | amp |
| TCGA-09-0366 | 1 | 55074557 | 55545391 | 1.869953504 | 5 | amp |
| TCGA-09-0366 | 1 | 55546985 | 60359531 | 0.913599583 | 3 | amp |
| TCGA-09-0366 | 1 | 60366555 | 62913216 | 1.153676807 | 5 | amp |
| TCGA-09-0366 | 1 | 62914116 | 65270790 | 0.823757009 | 3 | amp |
| TCGA-09-0366 | 1 | 65272842 | 65325986 | 1.506912736 | 5 | amp |
| TCGA-09-0366 | 1 | 65330442 | 89481153 | 0.925394626 | 3 | amp |
| TCGA-09-0366 | 1 | 89486189 | 90470809 | 1.239461252 | 5 | amp |
| TCGA-09-0366 | 1 | 90472836 | 94466669 | 0.851791498 | 3 | amp |
| TCGA-09-0366 | 1 | 94467360 | 94578635 | 1.346360844 | 5 | amp |
| TCGA-09-0366 | 1 | 94586508 | 108691014 | 0.821891744 | 3 | amp |
| TCGA-09-0366 | 1 | 108697560 | 109812498 | 1.391194569 | 5 | amp |
| TCGA-09-0366 | 1 | 109812534 | 109815688 | 3.099327023 | 6 | amp |
| TCGA-09-0366 | 1 | 109815722 | 110022240 | 1.465552187 | 5 | amp |
| TCGA-09-0366 | 1 | 110028621 | 110774946 | 2.133503185 | 6 | amp |
| TCGA-09-0366 | 1 | 110775503 | 113616327 | 1.455251434 | 5 | amp |
| TCGA-09-0366 | 1 | 113633912 | 114301362 | 0.77840067 | 3 | amp |
| TCGA-09-0366 | 1 | 114308598 | 115322843 | 1.188120614 | 5 | amp |
| TCGA-09-0366 | 1 | 115322992 | 115603181 | 0.794806319 | 3 | amp |
| TCGA-09-0366 | 1 | 115604744 | 117638019 | 1.178627878 | 5 | amp |
| TCGA-09-0366 | 1 | 117638725 | 119474351 | 0.825195147 | 3 | amp |
| TCGA-09-0366 | 1 | 119575442 | 144919031 | 1.766732899 | 5 | amp |
| TCGA-09-0366 | 1 | 144921820 | 152060670 | 3.818084424 | 6 | amp |
| TCGA-09-0366 | 1 | 152079799 | 153654367 | 1.617389339 | 5 | amp |
| TCGA-09-0366 | 1 | 153654959 | 153661845 | 3.543836495 | 6 | amp |
| TCGA-09-0366 | 1 | 153661902 | 155001910 | 1.546416385 | 5 | amp |
| TCGA-09-0366 | 1 | 155002488 | 155261754 | 3.925781597 | 6 | amp |
| TCGA-09-0366 | 1 | 155262900 | 157488312 | 1.666168473 | 5 | amp |
| TCGA-09-0366 | 1 | 157488470 | 157773947 | 0.795755414 | 3 | amp |
| TCGA-09-0366 | 1 | 157776841 | 158326721 | 1.542096412 | 5 | amp |
| TCGA-09-0366 | 1 | 158368280 | 159043338 | 0.865542058 | 3 | amp |
| TCGA-09-0366 | 1 | 159159551 | 160456580 | 1.378117715 | 5 | amp |
| TCGA-09-0366 | 1 | 160456869 | 160850557 | 0.882895754 | 3 | amp |
| TCGA-09-0366 | 1 | 160850902 | 161021512 | 1.351691164 | 5 | amp |
| TCGA-09-0366 | 1 | 161022054 | 161039425 | 4.15392106 | 6 | amp |
| TCGA-09-0366 | 1 | 161042442 | 162372620 | 1.590851303 | 5 | amp |
| TCGA-09-0366 | 1 | 162381619 | 164815926 | 0.804358942 | 3 | amp |
| TCGA-09-0366 | 1 | 165173046 | 167793821 | 1.203358432 | 5 | amp |
| TCGA-09-0366 | 1 | 167793870 | 180065365 | 0.858299123 | 3 | amp |
| TCGA-09-0366 | 1 | 180067938 | 182616080 | 1.323375925 | 5 | amp |
| TCGA-09-0366 | 1 | 182617204 | 200822632 | 0.712580932 | 3 | amp |
| TCGA-09-0366 | 1 | 200823890 | 207237205 | 1.609902293 | 5 | amp |

| | | | | | | |
|---|---|---|---|---|---|---|
| TCGA-09-0366 | 1 | 207238292 | 207966961 | 0.836309367 | 3 | amp |
| TCGA-09-0366 | 1 | 207975180 | 209936944 | 1.551710881 | 5 | amp |
| TCGA-09-0366 | 1 | 209946238 | 212560457 | 0.856900424 | 3 | amp |
| TCGA-09-0366 | 1 | 212583724 | 212968512 | 1.562389291 | 5 | amp |
| TCGA-09-0366 | 1 | 212969855 | 220826748 | 0.736815504 | 3 | amp |
| TCGA-09-0366 | 1 | 220831093 | 222721405 | 1.351661063 | 5 | amp |
| TCGA-09-0366 | 1 | 222731997 | 223168335 | 0.739330161 | 3 | amp |
| TCGA-09-0366 | 1 | 223175690 | 223957636 | 1.428485277 | 5 | amp |
| TCGA-09-0366 | 1 | 223958127 | 225755118 | 0.818407666 | 3 | amp |
| TCGA-09-0366 | 1 | 225965618 | 227182722 | 1.502546105 | 5 | amp |
| TCGA-09-0366 | 1 | 227192603 | 227843562 | 0.670536807 | 3 | amp |
| TCGA-09-0366 | 1 | 227922314 | 229431685 | 1.893156797 | 6 | amp |
| TCGA-09-0366 | 1 | 229433185 | 229676545 | 0.819698924 | 3 | amp |
| TCGA-09-0366 | 1 | 229677930 | 233193066 | 1.119794472 | 5 | amp |
| TCGA-09-0366 | 1 | 233225751 | 247163414 | 0.859411166 | 3 | amp |
| TCGA-09-0366 | 1 | 247171148 | 247712567 | 1.415598464 | 5 | amp |
| TCGA-09-0366 | 1 | 247719642 | 248814188 | 0.782698324 | 3 | amp |
| TCGA-09-0366 | 1 | 248844657 | 249231325 | 1.806958636 | 5 | amp |
| TCGA-09-0366 | 10 | 92880 | 1054996 | 1.053429896 | 4 | amp |
| TCGA-09-0366 | 10 | 1055434 | 4884714 | 1.36619004 | 5 | amp |
| TCGA-09-0366 | 10 | 4889320 | 5965681 | 0.906398788 | 4 | amp |
| TCGA-09-0366 | 10 | 5966222 | 6266011 | 1.578688765 | 5 | amp |
| TCGA-09-0366 | 10 | 6266053 | 6273306 | 2.309805963 | 6 | amp |
| TCGA-09-0366 | 10 | 6274821 | 7788671 | 1.179685194 | 5 | amp |
| TCGA-09-0366 | 10 | 7791103 | 8019304 | 0.903806476 | 4 | amp |
| TCGA-09-0366 | 10 | 8050984 | 11963361 | 1.197409636 | 5 | amp |
| TCGA-09-0366 | 10 | 11971803 | 12071553 | 0.531959076 | 3 | amp |
| TCGA-09-0366 | 10 | 12076990 | 12803135 | 1.000521294 | 4 | amp |
| TCGA-09-0366 | 10 | 12811614 | 13166112 | 1.570787824 | 5 | amp |
| TCGA-09-0366 | 10 | 13167342 | 13251328 | 0.955138588 | 4 | amp |
| TCGA-09-0366 | 10 | 13264091 | 13481501 | 1.38958015 | 5 | amp |
| TCGA-09-0366 | 10 | 13483656 | 15600256 | 0.944691636 | 4 | amp |
| TCGA-09-0366 | 10 | 15614168 | 17272738 | 0.684709019 | 3 | amp |
| TCGA-09-0366 | 10 | 17275573 | 17278395 | 1.710037918 | 5 | amp |
| TCGA-09-0366 | 10 | 17279189 | 27353053 | 0.721853437 | 3 | amp |
| TCGA-09-0366 | 10 | 27355386 | 27798891 | 0.93460135 | 4 | amp |
| TCGA-09-0366 | 10 | 27815704 | 28905316 | 0.636195452 | 3 | amp |
| TCGA-09-0366 | 10 | 28906529 | 31750185 | 0.978789492 | 4 | amp |
| TCGA-09-0366 | 10 | 31784679 | 34985356 | 0.674118045 | 3 | amp |
| TCGA-09-0366 | 10 | 35299184 | 45959790 | 0.867266295 | 4 | amp |
| TCGA-09-0366 | 10 | 45984779 | 46938387 | 0.719515212 | 3 | amp |
| TCGA-09-0366 | 10 | 46959933 | 47929905 | 1.025189284 | 4 | amp |
| TCGA-09-0366 | 10 | 47933762 | 48050564 | 0.526364934 | 3 | amp |
| TCGA-09-0366 | 10 | 48187381 | 49259739 | 0.965674556 | 4 | amp |
| TCGA-09-0366 | 10 | 49265409 | 50725169 | 0.726606665 | 3 | amp |
| TCGA-09-0366 | 10 | 50732065 | 50965026 | 1.066110931 | 4 | amp |
| TCGA-09-0366 | 10 | 50966386 | 51838525 | 0.803037195 | 3 | amp |
| TCGA-09-0366 | 10 | 69966443 | 70968803 | 0.736120068 | 3 | amp |
| TCGA-09-0366 | 10 | 70980162 | 71917631 | 1.173821089 | 5 | amp |
| TCGA-09-0366 | 10 | 71978486 | 73832343 | 1.414871918 | 5 | amp |

| | | | | | | |
|---|---|---|---|---|---|---|
| TCGA-09-0366 | 10 | 73856999 | 74135645 | 1.00883221 | 4 | amp |
| TCGA-09-0366 | 10 | 74167680 | 75239294 | 0.658783315 | 3 | amp |
| TCGA-09-0366 | 10 | 75258336 | 75391923 | 0.903597851 | 4 | amp |
| TCGA-09-0366 | 10 | 75393620 | 75676354 | 1.368341912 | 5 | amp |
| TCGA-09-0366 | 10 | 75802815 | 76790845 | 0.683255179 | 3 | amp |
| TCGA-09-0366 | 10 | 76797504 | 76924510 | 1.834469835 | 5 | amp |
| TCGA-09-0366 | 10 | 76928237 | 80961479 | 0.916341774 | 4 | amp |
| TCGA-09-0366 | 10 | 80968089 | 81904158 | 1.196431791 | 5 | amp |
| TCGA-09-0366 | 10 | 81915548 | 85962927 | 0.952154688 | 4 | amp |
| TCGA-09-0366 | 10 | 85964261 | 86007544 | 1.309581056 | 5 | amp |
| TCGA-09-0366 | 10 | 86008582 | 88260527 | 0.859423448 | 4 | amp |
| TCGA-09-0366 | 10 | 88277279 | 88417027 | 2.861103529 | 6 | amp |
| TCGA-09-0366 | 10 | 88417705 | 88492748 | 1.752770935 | 5 | amp |
| TCGA-09-0366 | 10 | 88635748 | 89272989 | 0.914198311 | 4 | amp |
| TCGA-09-0366 | 10 | 94816676 | 95385428 | 0.796066727 | 3 | amp |
| TCGA-09-0366 | 10 | 97096244 | 98762784 | 0.711110883 | 3 | amp |
| TCGA-09-0366 | 10 | 98763751 | 98985943 | 1.334021907 | 5 | amp |
| TCGA-09-0366 | 10 | 99052266 | 99219320 | 1.28648332 | 5 | amp |
| TCGA-09-0366 | 10 | 99219396 | 99330327 | 0.860885302 | 4 | amp |
| TCGA-09-0366 | 10 | 99341031 | 100195219 | 1.208787564 | 5 | amp |
| TCGA-09-0366 | 10 | 100195310 | 101579055 | 0.718597459 | 3 | amp |
| TCGA-09-0366 | 10 | 101590006 | 102259377 | 0.933186681 | 4 | amp |
| TCGA-09-0366 | 10 | 102261975 | 102584515 | 1.287440262 | 5 | amp |
| TCGA-09-0366 | 10 | 102584639 | 102733079 | 0.77544115 | 4 | amp |
| TCGA-09-0366 | 10 | 102733244 | 102987586 | 1.615471636 | 5 | amp |
| TCGA-09-0366 | 10 | 103174362 | 103310656 | 0.729263897 | 4 | amp |
| TCGA-09-0366 | 10 | 103339938 | 103347119 | 1.953248747 | 6 | amp |
| TCGA-09-0366 | 10 | 103354364 | 103534698 | 0.878325793 | 4 | amp |
| TCGA-09-0366 | 10 | 103541401 | 103543208 | 3.777653575 | 6 | amp |
| TCGA-09-0366 | 10 | 103546155 | 104161300 | 0.889520677 | 4 | amp |
| TCGA-09-0366 | 10 | 104161467 | 104594776 | 1.19656845 | 5 | amp |
| TCGA-09-0366 | 10 | 104594959 | 105074045 | 0.73329962 | 3 | amp |
| TCGA-09-0366 | 10 | 105086279 | 105642640 | 0.990199357 | 4 | amp |
| TCGA-09-0366 | 10 | 105648805 | 105795336 | 0.656685244 | 3 | amp |
| TCGA-09-0366 | 10 | 105796169 | 105822111 | 1.356686549 | 5 | amp |
| TCGA-09-0366 | 10 | 105823480 | 115537241 | 0.664162582 | 3 | amp |
| TCGA-09-0366 | 10 | 116014620 | 116062290 | 1.590537392 | 5 | amp |
| TCGA-09-0366 | 10 | 116067525 | 116444140 | 0.76373972 | 3 | amp |
| TCGA-09-0366 | 10 | 118353780 | 120820847 | 0.784591251 | 3 | amp |
| TCGA-09-0366 | 10 | 120824876 | 121285646 | 0.982036507 | 4 | amp |
| TCGA-09-0366 | 10 | 121286756 | 123683872 | 0.642167317 | 3 | amp |
| TCGA-09-0366 | 10 | 123718797 | 133950835 | 0.935946462 | 4 | amp |
| TCGA-09-0366 | 10 | 133978146 | 135516111 | 1.29408045 | 5 | amp |
| TCGA-09-0366 | 11 | 86637 | 3697983 | 1.493193731 | 5 | amp |
| TCGA-09-0366 | 11 | 3700613 | 3803369 | 0.490270345 | 3 | amp |
| TCGA-09-0366 | 11 | 3832421 | 3877689 | 1.651852741 | 5 | amp |
| TCGA-09-0366 | 11 | 3988726 | 6221474 | 0.731543798 | 3 | amp |
| TCGA-09-0366 | 11 | 6226791 | 6662676 | 1.38071257 | 5 | amp |
| TCGA-09-0366 | 11 | 6703310 | 7982878 | 0.763938158 | 3 | amp |
| TCGA-09-0366 | 11 | 7984717 | 9009866 | 1.125126461 | 5 | amp |

| | | | | | | |
|---|---|---|---|---|---|---|
| TCGA-09-0366 | 11 | 9042547 | 11977754 | 0.701886125 | 3 | amp |
| TCGA-09-0366 | 11 | 11985971 | 12284106 | 1.511614156 | 5 | amp |
| TCGA-09-0366 | 11 | 12313582 | 17352549 | 0.714036733 | 3 | amp |
| TCGA-09-0366 | 11 | 17352902 | 17899832 | 1.393623365 | 5 | amp |
| TCGA-09-0366 | 11 | 17980979 | 18638538 | 0.762702823 | 3 | amp |
| TCGA-09-0366 | 11 | 18655684 | 18793555 | 1.794993213 | 5 | amp |
| TCGA-09-0366 | 11 | 18955276 | 20127294 | 0.816589734 | 3 | amp |
| TCGA-09-0366 | 11 | 20129118 | 20413841 | 1.461563129 | 5 | amp |
| TCGA-09-0366 | 11 | 20414433 | 34155976 | 0.665955195 | 3 | amp |
| TCGA-09-0366 | 11 | 34156035 | 34226283 | 1.310720056 | 5 | amp |
| TCGA-09-0366 | 11 | 34470704 | 44265887 | 0.716626248 | 3 | amp |
| TCGA-09-0366 | 11 | 44288957 | 46431931 | 1.526308686 | 5 | amp |
| TCGA-09-0366 | 11 | 46439412 | 46691137 | 0.765580674 | 3 | amp |
| TCGA-09-0366 | 11 | 46693783 | 46751140 | 2.085078023 | 6 | amp |
| TCGA-09-0366 | 11 | 46760572 | 46905534 | 0.595085818 | 3 | amp |
| TCGA-09-0366 | 11 | 46907592 | 47606069 | 1.45282282 | 5 | amp |
| TCGA-09-0366 | 11 | 47640354 | 56954956 | 0.588086548 | 3 | amp |
| TCGA-09-0366 | 11 | 57003276 | 57317632 | 1.161457671 | 5 | amp |
| TCGA-09-0366 | 11 | 57319756 | 57583834 | 0.748322824 | 3 | amp |
| TCGA-09-0366 | 11 | 57798421 | 58893793 | 7.913949139 | 6 | amp |
| TCGA-09-0366 | 11 | 58916295 | 61071541 | 0.747466414 | 3 | amp |
| TCGA-09-0366 | 11 | 61076441 | 61647627 | 1.234389161 | 5 | amp |
| TCGA-09-0366 | 11 | 61665700 | 62301580 | 0.789874159 | 3 | amp |
| TCGA-09-0366 | 11 | 62302418 | 62569750 | 1.278726642 | 5 | amp |
| TCGA-09-0366 | 11 | 62570847 | 63756234 | 0.70143634 | 3 | amp |
| TCGA-09-0366 | 11 | 63763940 | 65827486 | 1.360244376 | 5 | amp |
| TCGA-09-0366 | 11 | 65828007 | 66106283 | 0.845315076 | 3 | amp |
| TCGA-09-0366 | 11 | 66107547 | 66834323 | 1.222114331 | 5 | amp |
| TCGA-09-0366 | 11 | 66837812 | 67059265 | 0.696330024 | 3 | amp |
| TCGA-09-0366 | 11 | 67066471 | 67120277 | 1.566600342 | 5 | amp |
| TCGA-09-0366 | 11 | 67120731 | 72145556 | 0.679467303 | 3 | amp |
| TCGA-09-0366 | 11 | 72288412 | 72539857 | 1.334760568 | 5 | amp |
| TCGA-09-0366 | 11 | 75715000 | 79113076 | 15.77513208 | 6 | amp |
| TCGA-09-0366 | 12 | 73256 | 310002 | 2.796703459 | 6 | amp |
| TCGA-09-0366 | 12 | 310911 | 133438256 | 1.523621236 | 5 | amp |
| TCGA-09-0366 | 12 | 133447249 | 133522273 | 2.244324895 | 6 | amp |
| TCGA-09-0366 | 12 | 133583570 | 133779395 | 1.172901903 | 5 | amp |
| TCGA-09-0366 | 13 | 19240876 | 21171265 | 0.674611424 | 3 | amp |
| TCGA-09-0366 | 13 | 21535765 | 24455941 | 0.710873025 | 3 | amp |
| TCGA-09-0366 | 13 | 24460408 | 24893047 | 1.171124223 | 5 | amp |
| TCGA-09-0366 | 13 | 24895099 | 25052036 | 0.635475133 | 3 | amp |
| TCGA-09-0366 | 13 | 25052202 | 25341516 | 0.896238605 | 4 | amp |
| TCGA-09-0366 | 13 | 25348906 | 28602429 | 0.65512586 | 3 | amp |
| TCGA-09-0366 | 13 | 28862039 | 31821257 | 0.728538705 | 3 | amp |
| TCGA-09-0366 | 13 | 38923997 | 41768400 | 0.678997292 | 3 | amp |
| TCGA-09-0366 | 13 | 43500458 | 47224527 | 0.686879364 | 3 | amp |
| TCGA-09-0366 | 13 | 50025977 | 51935516 | 0.677828981 | 3 | amp |
| TCGA-09-0366 | 13 | 52325438 | 52348177 | 0.885017277 | 3 | amp |
| TCGA-09-0366 | 13 | 52351166 | 52524591 | 1.35827059 | 5 | amp |
| TCGA-09-0366 | 13 | 52531637 | 53042507 | 0.874898729 | 4 | amp |

| | | | | | | |
|---|---|---|---|---|---|---|
| TCGA-09-0366 | 13 | 53047911 | 58207868 | 0.711819585 | 3 | amp |
| TCGA-09-0366 | 13 | 80910886 | 96361595 | 0.667028762 | 3 | amp |
| TCGA-09-0366 | 13 | 96577890 | 98673436 | 0.673603688 | 3 | amp |
| TCGA-09-0366 | 13 | 98673972 | 99338557 | 0.996252231 | 4 | amp |
| TCGA-09-0366 | 13 | 99339756 | 100169961 | 0.682507452 | 3 | amp |
| TCGA-09-0366 | 13 | 101077875 | 101721232 | 0.767013461 | 3 | amp |
| TCGA-09-0366 | 13 | 103346633 | 110864066 | 0.688762758 | 3 | amp |
| TCGA-09-0366 | 13 | 110864152 | 111319873 | 1.205477848 | 5 | amp |
| TCGA-09-0366 | 13 | 111329235 | 111920075 | 0.915738318 | 4 | amp |
| TCGA-09-0366 | 13 | 111926109 | 113803890 | 1.250527332 | 5 | amp |
| TCGA-09-0366 | 13 | 113814259 | 113917938 | 0.851727052 | 4 | amp |
| TCGA-09-0366 | 13 | 113960740 | 114766414 | 1.316569519 | 5 | amp |
| TCGA-09-0366 | 13 | 114786871 | 115091796 | 0.916786754 | 4 | amp |
| TCGA-09-0366 | 14 | 19377543 | 20770177 | 0.932560689 | 4 | amp |
| TCGA-09-0366 | 14 | 20774010 | 21779134 | 1.49836109 | 5 | amp |
| TCGA-09-0366 | 14 | 21779935 | 21798476 | 0.992148927 | 4 | amp |
| TCGA-09-0366 | 14 | 21802753 | 22932242 | 0.864755505 | 3 | amp |
| TCGA-09-0366 | 14 | 22932738 | 23008106 | 0.980410765 | 4 | amp |
| TCGA-09-0366 | 14 | 23009159 | 23299195 | 1.169780861 | 5 | amp |
| TCGA-09-0366 | 14 | 23299198 | 23345612 | 2.02737573 | 6 | amp |
| TCGA-09-0366 | 14 | 23345842 | 23421940 | 1.135104367 | 5 | amp |
| TCGA-09-0366 | 14 | 23424275 | 23519211 | 2.071854645 | 6 | amp |
| TCGA-09-0366 | 14 | 23523323 | 23826891 | 1.589504981 | 5 | amp |
| TCGA-09-0366 | 14 | 23828039 | 23899880 | 2.058605503 | 6 | amp |
| TCGA-09-0366 | 14 | 23900068 | 23947285 | 1.183972934 | 5 | amp |
| TCGA-09-0366 | 14 | 24025909 | 24662524 | 2.203448725 | 6 | amp |
| TCGA-09-0366 | 14 | 24663842 | 24684872 | 1.240093355 | 5 | amp |
| TCGA-09-0366 | 14 | 24684882 | 24806718 | 2.066843675 | 6 | amp |
| TCGA-09-0366 | 14 | 24806739 | 24976756 | 1.35427024 | 5 | amp |
| TCGA-09-0366 | 14 | 24977322 | 25103398 | 1.027255015 | 4 | amp |
| TCGA-09-0366 | 14 | 25281836 | 64644258 | 0.767825691 | 3 | amp |
| TCGA-09-0366 | 14 | 64653069 | 65056082 | 1.034483254 | 4 | amp |
| TCGA-09-0366 | 14 | 65066939 | 65568336 | 1.323545095 | 5 | amp |
| TCGA-09-0366 | 14 | 66028262 | 67849355 | 0.899092596 | 4 | amp |
| TCGA-09-0366 | 14 | 67849944 | 68215374 | 1.306735576 | 5 | amp |
| TCGA-09-0366 | 14 | 68217730 | 68935066 | 0.977567098 | 4 | amp |
| TCGA-09-0366 | 14 | 68935756 | 69866194 | 1.307896154 | 5 | amp |
| TCGA-09-0366 | 14 | 69890774 | 71267830 | 1.076205978 | 4 | amp |
| TCGA-09-0366 | 14 | 71413615 | 72172117 | 0.626277263 | 3 | amp |
| TCGA-09-0366 | 14 | 72175919 | 73002998 | 0.982502208 | 4 | amp |
| TCGA-09-0366 | 14 | 73006721 | 75070432 | 1.327589001 | 5 | amp |
| TCGA-09-0366 | 14 | 75130383 | 76129589 | 1.062336068 | 4 | amp |
| TCGA-09-0366 | 14 | 76135751 | 76349256 | 0.608430003 | 3 | amp |
| TCGA-09-0366 | 14 | 76353012 | 76668233 | 1.076294887 | 4 | amp |
| TCGA-09-0366 | 14 | 76905624 | 77580694 | 1.598982556 | 5 | amp |
| TCGA-09-0366 | 14 | 77599978 | 77733057 | 2.691958756 | 6 | amp |
| TCGA-09-0366 | 14 | 77734748 | 77797594 | 1.29026502 | 5 | amp |
| TCGA-09-0366 | 14 | 77808102 | 80130326 | 1.035643199 | 4 | amp |
| TCGA-09-0366 | 14 | 80158500 | 89747385 | 0.741260625 | 3 | amp |
| TCGA-09-0366 | 14 | 89816963 | 92071074 | 1.050033974 | 4 | amp |

| | | | | | | |
|---|---|---|---|---|---|---|
| TCGA-09-0366 | 14 | 92074602 | 92482242 | 0.71129277 | 3 | amp |
| TCGA-09-0366 | 14 | 92483999 | 92900353 | 1.014097546 | 4 | amp |
| TCGA-09-0366 | 14 | 92905655 | 93715030 | 1.285581557 | 5 | amp |
| TCGA-09-0366 | 14 | 93717783 | 94160779 | 0.735529873 | 3 | amp |
| TCGA-09-0366 | 14 | 94170931 | 94693716 | 1.414123878 | 5 | amp |
| TCGA-09-0366 | 14 | 94696877 | 95677239 | 0.928084171 | 4 | amp |
| TCGA-09-0366 | 14 | 95679530 | 96752326 | 1.475886124 | 5 | amp |
| TCGA-09-0366 | 14 | 96755917 | 97342501 | 0.706083942 | 3 | amp |
| TCGA-09-0366 | 14 | 97347444 | 99927722 | 1.071400183 | 4 | amp |
| TCGA-09-0366 | 14 | 99929808 | 101377538 | 1.523278923 | 5 | amp |
| TCGA-09-0366 | 14 | 101391132 | 101420807 | 0.753877395 | 3 | amp |
| TCGA-09-0366 | 14 | 101488345 | 101507800 | 2.725392526 | 6 | amp |
| TCGA-09-0366 | 14 | 101512750 | 104460957 | 1.234482588 | 5 | amp |
| TCGA-09-0366 | 14 | 104462055 | 105175098 | 0.737802798 | 4 | amp |
| TCGA-09-0366 | 14 | 105181588 | 105421167 | 1.630811353 | 5 | amp |
| TCGA-09-0366 | 14 | 105421265 | 105695263 | 2.525169737 | 6 | amp |
| TCGA-09-0366 | 14 | 105715952 | 107283263 | 1.179404727 | 5 | amp |
| TCGA-09-0366 | 15 | 20169886 | 22705528 | 1.270674159 | 5 | amp |
| TCGA-09-0366 | 15 | 22705541 | 22742458 | 1.922248259 | 6 | amp |
| TCGA-09-0366 | 15 | 22744206 | 25331779 | 1.311842323 | 5 | amp |
| TCGA-09-0366 | 15 | 25415850 | 32908596 | 1.218027167 | 5 | amp |
| TCGA-09-0366 | 15 | 32912283 | 34638288 | 0.872344349 | 3 | amp |
| TCGA-09-0366 | 15 | 34640163 | 34824240 | 1.920822912 | 6 | amp |
| TCGA-09-0366 | 15 | 34824989 | 35087064 | 1.304280677 | 5 | amp |
| TCGA-09-0366 | 15 | 35148969 | 40512982 | 0.852412159 | 3 | amp |
| TCGA-09-0366 | 15 | 40556938 | 40627929 | 2.383285 | 6 | amp |
| TCGA-09-0366 | 15 | 40628729 | 41280212 | 1.384061184 | 5 | amp |
| TCGA-09-0366 | 15 | 41455382 | 41797783 | 1.310448829 | 5 | amp |
| TCGA-09-0366 | 15 | 41797852 | 41870518 | 1.945484751 | 6 | amp |
| TCGA-09-0366 | 15 | 41961084 | 42105327 | 1.024819428 | 4 | amp |
| TCGA-09-0366 | 15 | 42105476 | 42442687 | 1.934125718 | 6 | amp |
| TCGA-09-0366 | 15 | 42445428 | 42453985 | 1.162292137 | 5 | amp |
| TCGA-09-0366 | 15 | 42454176 | 42840492 | 0.818754758 | 3 | amp |
| TCGA-09-0366 | 15 | 42840530 | 43028294 | 2.109395199 | 6 | amp |
| TCGA-09-0366 | 15 | 43037983 | 43038463 | 1.655183936 | 5 | amp |
| TCGA-09-0366 | 15 | 43044148 | 43489700 | 0.847173208 | 3 | amp |
| TCGA-09-0366 | 15 | 43493958 | 43627396 | 1.963736994 | 6 | amp |
| TCGA-09-0366 | 15 | 43627838 | 43678542 | 1.21132858 | 5 | amp |
| TCGA-09-0366 | 15 | 43687269 | 43739685 | 0.757886305 | 4 | amp |
| TCGA-09-0366 | 15 | 43748032 | 43875164 | 1.12402751 | 5 | amp |
| TCGA-09-0366 | 15 | 43875549 | 43897065 | 2.357937464 | 6 | amp |
| TCGA-09-0366 | 15 | 43897409 | 43974999 | 1.230764253 | 5 | amp |
| TCGA-09-0366 | 15 | 43975384 | 44010043 | 1.976731713 | 6 | amp |
| TCGA-09-0366 | 15 | 44010207 | 44066585 | 1.391765066 | 5 | amp |
| TCGA-09-0366 | 15 | 44066634 | 44089150 | 1.989980968 | 6 | amp |
| TCGA-09-0366 | 15 | 44090074 | 44131939 | 1.329721114 | 5 | amp |
| TCGA-09-0366 | 15 | 44134573 | 45250760 | 0.852173386 | 3 | amp |
| TCGA-09-0366 | 15 | 45253691 | 45386944 | 1.333526734 | 5 | amp |
| TCGA-09-0366 | 15 | 45387091 | 45446233 | 1.973667261 | 6 | amp |
| TCGA-09-0366 | 15 | 45447913 | 45554318 | 1.048756094 | 4 | amp |

| | | | | | | |
|---|---|---|---|---|---|---|
| TCGA-09-0366 | 15 | 45555200 | 45777557 | 1.013892228 | 3 | amp |
| TCGA-09-0366 | 15 | 62336372 | 62336492 | 1.22955596 | 4 | amp |
| TCGA-09-0366 | 15 | 62939457 | 63419899 | 1.20023943 | 5 | amp |
| TCGA-09-0366 | 15 | 63421646 | 64422533 | 0.834299743 | 3 | amp |
| TCGA-09-0366 | 15 | 64423868 | 65563461 | 1.247725826 | 5 | amp |
| TCGA-09-0366 | 15 | 65621156 | 65677492 | 1.923928586 | 6 | amp |
| TCGA-09-0366 | 15 | 65678132 | 65703712 | 1.586155466 | 5 | amp |
| TCGA-09-0366 | 15 | 65739162 | 66855968 | 0.892589452 | 3 | amp |
| TCGA-09-0366 | 15 | 66856202 | 67459213 | 1.963316447 | 6 | amp |
| TCGA-09-0366 | 15 | 67462856 | 67496493 | 1.314123241 | 5 | amp |
| TCGA-09-0366 | 15 | 67500887 | 68065127 | 0.770345555 | 3 | amp |
| TCGA-09-0366 | 15 | 68099145 | 69672405 | 1.392274898 | 5 | amp |
| TCGA-09-0366 | 15 | 69676998 | 69696220 | 2.482490988 | 6 | amp |
| TCGA-09-0366 | 15 | 69708307 | 70368525 | 1.323298704 | 5 | amp |
| TCGA-09-0366 | 15 | 72454261 | 72949018 | 1.159683651 | 5 | amp |
| TCGA-09-0366 | 15 | 72950878 | 72955138 | 2.14066301 | 6 | amp |
| TCGA-09-0366 | 15 | 72955351 | 72958739 | 1.289046863 | 5 | amp |
| TCGA-09-0366 | 15 | 72987483 | 73889716 | 0.79582905 | 3 | amp |
| TCGA-09-0366 | 15 | 73991929 | 76146837 | 1.619787271 | 5 | amp |
| TCGA-09-0366 | 15 | 76152210 | 78191180 | 0.915976915 | 3 | amp |
| TCGA-09-0366 | 15 | 78207469 | 78215372 | 2.1414161 | 6 | amp |
| TCGA-09-0366 | 15 | 78217231 | 81630285 | 1.247172048 | 5 | amp |
| TCGA-09-0366 | 15 | 81630938 | 82597150 | 0.783517529 | 3 | amp |
| TCGA-09-0366 | 15 | 82620489 | 83296150 | 1.439134073 | 5 | amp |
| TCGA-09-0366 | 15 | 83328266 | 83335711 | 1.932743305 | 6 | amp |
| TCGA-09-0366 | 15 | 83345180 | 83679171 | 1.204259664 | 5 | amp |
| TCGA-09-0366 | 15 | 83686747 | 84795403 | 0.719200831 | 3 | amp |
| TCGA-09-0366 | 15 | 84859539 | 85488102 | 1.366802119 | 5 | amp |
| TCGA-09-0366 | 15 | 85488303 | 88669612 | 0.860384655 | 3 | amp |
| TCGA-09-0366 | 15 | 88670364 | 90934115 | 1.415595579 | 5 | amp |
| TCGA-09-0366 | 15 | 90969299 | 91354655 | 0.813654778 | 3 | amp |
| TCGA-09-0366 | 15 | 91358300 | 91491562 | 2.526044693 | 6 | amp |
| TCGA-09-0366 | 15 | 91491803 | 93444558 | 1.30998674 | 5 | amp |
| TCGA-09-0366 | 15 | 93467546 | 95020033 | 0.805795807 | 3 | amp |
| TCGA-09-0366 | 15 | 95022168 | 101552401 | 1.155115774 | 5 | amp |
| TCGA-09-0366 | 15 | 101554486 | 101588900 | 2.265441451 | 6 | amp |
| TCGA-09-0366 | 15 | 101589768 | 102514208 | 1.358221912 | 5 | amp |
| TCGA-09-0366 | 16 | 66517 | 511510 | 1.486764267 | 5 | amp |
| TCGA-09-0366 | 16 | 546781 | 984308 | 2.908566282 | 6 | amp |
| TCGA-09-0366 | 16 | 1245914 | 1798959 | 1.56345562 | 5 | amp |
| TCGA-09-0366 | 16 | 1811205 | 2764381 | 1.905835758 | 6 | amp |
| TCGA-09-0366 | 16 | 2806306 | 2818124 | 1.180683494 | 5 | amp |
| TCGA-09-0366 | 16 | 2818166 | 3296595 | 2.222288343 | 6 | amp |
| TCGA-09-0366 | 16 | 3297010 | 4484571 | 1.478058396 | 5 | amp |
| TCGA-09-0366 | 16 | 4487384 | 4851354 | 2.315442849 | 6 | amp |
| TCGA-09-0366 | 16 | 4855213 | 4936096 | 1.294354987 | 5 | amp |
| TCGA-09-0366 | 16 | 4937091 | 5145543 | 2.188808315 | 6 | amp |
| TCGA-09-0366 | 16 | 7102025 | 8992315 | 1.362551829 | 5 | amp |
| TCGA-09-0366 | 16 | 8992351 | 10576139 | 0.845686874 | 4 | amp |
| TCGA-09-0366 | 16 | 10626705 | 11824638 | 1.463992716 | 5 | amp |

| | | | | | | |
|---|---|---|---|---|---|---|
| TCGA-09-0366 | 16 | 11827826 | 14693848 | 0.940413028 | 3 | amp |
| TCGA-09-0366 | 16 | 14697982 | 14711536 | 1.09108879 | 4 | amp |
| TCGA-09-0366 | 16 | 14720943 | 15818856 | 1.189944902 | 5 | amp |
| TCGA-09-0366 | 16 | 15820657 | 15854575 | 1.99473513 | 6 | amp |
| TCGA-09-0366 | 16 | 15857610 | 16251708 | 1.493250244 | 5 | amp |
| TCGA-09-0366 | 16 | 16253299 | 16315716 | 1.996942835 | 6 | amp |
| TCGA-09-0366 | 16 | 16330742 | 18820989 | 1.28785189 | 5 | amp |
| TCGA-09-0366 | 16 | 18823075 | 18907571 | 0.72115438 | 3 | amp |
| TCGA-09-0366 | 16 | 18908076 | 19884192 | 1.151221375 | 4 | amp |
| TCGA-09-0366 | 16 | 20043043 | 22120959 | 0.908304261 | 3 | amp |
| TCGA-09-0366 | 16 | 22122201 | 22339929 | 1.304908779 | 5 | amp |
| TCGA-09-0366 | 16 | 22343323 | 23119555 | 0.872996163 | 3 | amp |
| TCGA-09-0366 | 16 | 23197540 | 23695420 | 1.417728368 | 5 | amp |
| TCGA-09-0366 | 16 | 23698717 | 23721913 | 1.903137857 | 6 | amp |
| TCGA-09-0366 | 16 | 23722226 | 27375201 | 1.152032316 | 5 | amp |
| TCGA-09-0366 | 16 | 27445628 | 27499761 | 2.01100037 | 6 | amp |
| TCGA-09-0366 | 16 | 27499825 | 28474412 | 1.362220427 | 5 | amp |
| TCGA-09-0366 | 16 | 28488746 | 28631478 | 2.079476223 | 6 | amp |
| TCGA-09-0366 | 16 | 28649681 | 28738574 | 1.45151399 | 5 | amp |
| TCGA-09-0366 | 16 | 28743091 | 29001353 | 2.177690953 | 6 | amp |
| TCGA-09-0366 | 16 | 29057320 | 29465821 | 1.159758898 | 5 | amp |
| TCGA-09-0366 | 16 | 29466037 | 29493919 | 2.429981536 | 6 | amp |
| TCGA-09-0366 | 16 | 29495113 | 29577608 | 0.912455395 | 4 | amp |
| TCGA-09-0366 | 16 | 29630027 | 30233259 | 2.124556773 | 6 | amp |
| TCGA-09-0366 | 16 | 30236800 | 30370180 | 1.007755105 | 4 | amp |
| TCGA-09-0366 | 16 | 30376419 | 31271459 | 1.929878854 | 6 | amp |
| TCGA-09-0366 | 16 | 31273005 | 31371433 | 1.328414174 | 5 | amp |
| TCGA-09-0366 | 16 | 31371557 | 31512089 | 2.205125792 | 6 | amp |
| TCGA-09-0366 | 16 | 31539437 | 34324982 | 1.174910108 | 5 | amp |
| TCGA-09-0366 | 16 | 34326294 | 46962923 | 0.96823514 | 3 | amp |
| TCGA-09-0366 | 16 | 54145600 | 56602858 | 0.848738081 | 3 | amp |
| TCGA-09-0366 | 16 | 56623427 | 57757097 | 1.218187277 | 5 | amp |
| TCGA-09-0366 | 16 | 57758516 | 57785253 | 2.81159554 | 6 | amp |
| TCGA-09-0366 | 16 | 57785504 | 58076323 | 1.499371924 | 5 | amp |
| TCGA-09-0366 | 16 | 58147871 | 58554960 | 1.04332137 | 4 | amp |
| TCGA-09-0366 | 16 | 58555033 | 65397177 | 0.70501772 | 3 | amp |
| TCGA-09-0366 | 16 | 66413195 | 66850589 | 1.102611327 | 4 | amp |
| TCGA-09-0366 | 16 | 66850839 | 67860980 | 1.293417986 | 5 | amp |
| TCGA-09-0366 | 16 | 67861094 | 67865556 | 2.116493695 | 6 | amp |
| TCGA-09-0366 | 16 | 67865604 | 68057133 | 1.371127049 | 5 | amp |
| TCGA-09-0366 | 16 | 68071869 | 68732318 | 1.05972151 | 4 | amp |
| TCGA-09-0366 | 16 | 68835534 | 70180143 | 0.909210605 | 3 | amp |
| TCGA-09-0366 | 16 | 70182351 | 70605765 | 1.086954162 | 4 | amp |
| TCGA-09-0366 | 16 | 70688387 | 71013000 | 1.332168127 | 5 | amp |
| TCGA-09-0366 | 16 | 71015232 | 71516060 | 1.062699758 | 4 | amp |
| TCGA-09-0366 | 16 | 71570560 | 81142909 | 0.873160696 | 3 | amp |
| TCGA-09-0366 | 16 | 81145752 | 81953298 | 1.167656569 | 4 | amp |
| TCGA-09-0366 | 16 | 81954778 | 83817087 | 0.71505856 | 3 | amp |
| TCGA-09-0366 | 16 | 83828571 | 89758950 | 1.286949085 | 5 | amp |
| TCGA-09-0366 | 16 | 89759647 | 89813103 | 2.447887433 | 6 | amp |

| | | | | | | |
|---|---|---|---|---|---|---|
| TCGA-09-0366 | 16 | 89816103 | 90244214 | 1.478487911 | 5 | amp |
| TCGA-09-0366 | 17 | 69410 | 2935768 | 1.316252044 | 5 | amp |
| TCGA-09-0366 | 17 | 2965951 | 3417308 | 0.726681001 | 3 | amp |
| TCGA-09-0366 | 17 | 3417866 | 3913094 | 1.337891987 | 5 | amp |
| TCGA-09-0366 | 17 | 3916734 | 4060387 | 0.669484586 | 3 | amp |
| TCGA-09-0366 | 17 | 4071018 | 5045804 | 1.409759426 | 5 | amp |
| TCGA-09-0366 | 17 | 5047951 | 5314125 | 0.740370732 | 3 | amp |
| TCGA-09-0366 | 17 | 5317271 | 6441414 | 1.154218992 | 4 | amp |
| TCGA-09-0366 | 17 | 6482975 | 6538436 | 0.552031153 | 3 | amp |
| TCGA-09-0366 | 17 | 6545053 | 6616661 | 1.353790984 | 5 | amp |
| TCGA-09-0366 | 17 | 6659323 | 6733720 | 0.73809259 | 3 | amp |
| TCGA-09-0366 | 17 | 6901778 | 8398566 | 1.395227958 | 5 | amp |
| TCGA-09-0366 | 17 | 8402540 | 8526571 | 0.874075869 | 3 | amp |
| TCGA-09-0366 | 17 | 8633426 | 9124581 | 1.465929432 | 5 | amp |
| TCGA-09-0366 | 17 | 9281859 | 9532182 | 0.684815893 | 3 | amp |
| TCGA-09-0366 | 17 | 9536157 | 10223860 | 1.065790012 | 4 | amp |
| TCGA-09-0366 | 17 | 10224816 | 10235597 | 0.832977595 | 3 | amp |
| TCGA-09-0366 | 17 | 10532832 | 12847005 | 0.808490438 | 3 | amp |
| TCGA-09-0366 | 17 | 12847329 | 12916617 | 1.327576524 | 5 | amp |
| TCGA-09-0366 | 17 | 12917724 | 15554963 | 0.811337541 | 3 | amp |
| TCGA-09-0366 | 17 | 15603601 | 15907232 | 1.173379784 | 4 | amp |
| TCGA-09-0366 | 17 | 15907455 | 16090084 | 0.672259 | 3 | amp |
| TCGA-09-0366 | 17 | 16097769 | 16527852 | 1.087444568 | 4 | amp |
| TCGA-09-0366 | 17 | 16537163 | 16705659 | 0.822749601 | 3 | amp |
| TCGA-09-0366 | 17 | 16734996 | 18396212 | 1.26286544 | 5 | amp |
| TCGA-09-0366 | 17 | 18397457 | 18525907 | 0.668565082 | 3 | amp |
| TCGA-09-0366 | 17 | 18528408 | 18678536 | 1.095487628 | 4 | amp |
| TCGA-09-0366 | 17 | 18681780 | 18834051 | 0.619905292 | 3 | amp |
| TCGA-09-0366 | 17 | 18855513 | 19689444 | 1.208618885 | 5 | amp |
| TCGA-09-0366 | 17 | 19698896 | 20334237 | 0.727749092 | 3 | amp |
| TCGA-09-0366 | 17 | 20353238 | 20654793 | 1.024522985 | 4 | amp |
| TCGA-09-0366 | 17 | 20768714 | 26925998 | 1.389079016 | 5 | amp |
| TCGA-09-0366 | 17 | 26938532 | 26951449 | 0.720824764 | 3 | amp |
| TCGA-09-0366 | 17 | 26955249 | 27028202 | 1.05168587 | 4 | amp |
| TCGA-09-0366 | 17 | 27028399 | 27573956 | 1.400875651 | 5 | amp |
| TCGA-09-0366 | 17 | 27576131 | 27849597 | 0.683509233 | 3 | amp |
| TCGA-09-0366 | 17 | 27857380 | 27959983 | 1.157075129 | 4 | amp |
| TCGA-09-0366 | 17 | 27962952 | 29070412 | 0.737453327 | 3 | amp |
| TCGA-09-0366 | 17 | 29663308 | 30551813 | 0.832442562 | 3 | amp |
| TCGA-09-0366 | 17 | 30594872 | 30690027 | 1.213424927 | 4 | amp |
| TCGA-09-0366 | 17 | 30692307 | 33285765 | 0.749956718 | 3 | amp |
| TCGA-09-0366 | 17 | 33286577 | 33592918 | 1.112379943 | 4 | amp |
| TCGA-09-0366 | 17 | 33679346 | 34037355 | 0.68819885 | 3 | amp |
| TCGA-09-0366 | 17 | 34044170 | 34160987 | 1.199147829 | 4 | amp |
| TCGA-09-0366 | 17 | 34161525 | 34433721 | 0.832167183 | 3 | amp |
| TCGA-09-0366 | 17 | 34493274 | 35310630 | 1.198337662 | 5 | amp |
| TCGA-09-0366 | 17 | 35733003 | 36002357 | 0.719489142 | 3 | amp |
| TCGA-09-0366 | 17 | 36003315 | 36462608 | 1.067349483 | 4 | amp |
| TCGA-09-0366 | 17 | 36474549 | 37373437 | 1.323103223 | 5 | amp |
| TCGA-09-0366 | 17 | 37417676 | 37682582 | 0.785362126 | 3 | amp |

| | | | | | | |
|---|---|---|---|---|---|---|
| TCGA-09-0366 | 17 | 37686822 | 37903196 | 1.541576149 | 5 | amp |
| TCGA-09-0366 | 17 | 37922034 | 38073571 | 0.777831399 | 3 | amp |
| TCGA-09-0366 | 17 | 38078720 | 38290175 | 1.5878859 | 5 | amp |
| TCGA-09-0366 | 17 | 38297413 | 38325922 | 0.744486722 | 3 | amp |
| TCGA-09-0366 | 17 | 38340392 | 38712131 | 1.043460333 | 4 | amp |
| TCGA-09-0366 | 17 | 38715109 | 39150380 | 0.669251226 | 3 | amp |
| TCGA-09-0366 | 17 | 39155776 | 39524281 | 1.012679116 | 4 | amp |
| TCGA-09-0366 | 17 | 39525648 | 40481839 | 1.330161926 | 5 | amp |
| TCGA-09-0366 | 17 | 40483459 | 40666512 | 0.749846273 | 3 | amp |
| TCGA-09-0366 | 17 | 40673001 | 41180752 | 1.317468574 | 5 | amp |
| TCGA-09-0366 | 17 | 41197636 | 41258571 | 0.703889269 | 3 | amp |
| TCGA-09-0366 | 17 | 41267709 | 41561599 | 1.186753145 | 4 | amp |
| TCGA-09-0366 | 17 | 41566799 | 41601225 | 0.647560421 | 3 | amp |
| TCGA-09-0366 | 17 | 41605848 | 42466867 | 1.416209392 | 5 | amp |
| TCGA-09-0366 | 17 | 42474898 | 42552293 | 0.557418284 | 3 | amp |
| TCGA-09-0366 | 17 | 42635108 | 42979073 | 1.049789467 | 4 | amp |
| TCGA-09-0366 | 17 | 42979688 | 44116606 | 1.266685755 | 5 | amp |
| TCGA-09-0366 | 17 | 44117038 | 45247465 | 0.829932972 | 3 | amp |
| TCGA-09-0366 | 17 | 45249236 | 45387597 | 1.106976826 | 4 | amp |
| TCGA-09-0366 | 17 | 45405644 | 45759829 | 0.835864624 | 3 | amp |
| TCGA-09-0366 | 17 | 45773470 | 46148988 | 1.315351555 | 5 | amp |
| TCGA-09-0366 | 17 | 46152324 | 46474154 | 0.667933281 | 3 | amp |
| TCGA-09-0366 | 17 | 46606892 | 47810117 | 1.127065478 | 4 | amp |
| TCGA-09-0366 | 17 | 47869201 | 47904873 | 0.603990685 | 3 | amp |
| TCGA-09-0366 | 17 | 47915959 | 48777219 | 1.683615839 | 5 | amp |
| TCGA-09-0366 | 17 | 48777882 | 54559932 | 0.639823004 | 3 | amp |
| TCGA-09-0366 | 17 | 54671531 | 56292210 | 1.034461165 | 4 | amp |
| TCGA-09-0366 | 17 | 56293406 | 56621606 | 1.331353868 | 5 | amp |
| TCGA-09-0366 | 17 | 56634329 | 57298161 | 0.730785446 | 3 | amp |
| TCGA-09-0366 | 17 | 58034543 | 58079788 | 0.725669309 | 3 | amp |
| TCGA-09-0366 | 17 | 58085697 | 58236807 | 1.135975583 | 4 | amp |
| TCGA-09-0366 | 17 | 59469261 | 59560928 | 1.708356864 | 5 | amp |
| TCGA-09-0366 | 17 | 60140424 | 60689959 | 0.709036614 | 3 | amp |
| TCGA-09-0366 | 17 | 60741839 | 60879111 | 1.335226202 | 5 | amp |
| TCGA-09-0366 | 17 | 61021556 | 61495777 | 0.693087341 | 3 | amp |
| TCGA-09-0366 | 17 | 61497573 | 61790946 | 1.111072963 | 4 | amp |
| TCGA-09-0366 | 17 | 61791326 | 61902525 | 0.707251865 | 3 | amp |
| TCGA-09-0366 | 17 | 61902578 | 62034879 | 1.363048232 | 5 | amp |
| TCGA-09-0366 | 17 | 62036591 | 62506404 | 1.009770454 | 4 | amp |
| TCGA-09-0366 | 17 | 62512833 | 62758710 | 0.574228525 | 3 | amp |
| TCGA-09-0366 | 17 | 62788443 | 62968716 | 1.066440233 | 4 | amp |
| TCGA-09-0366 | 17 | 63010360 | 65052393 | 0.770231812 | 3 | amp |
| TCGA-09-0366 | 17 | 65978325 | 66303863 | 1.141510623 | 4 | amp |
| TCGA-09-0366 | 17 | 66339718 | 66551911 | 0.799939576 | 3 | amp |
| TCGA-09-0366 | 17 | 68171162 | 71192947 | 1.033047815 | 3 | amp |
| TCGA-09-0366 | 17 | 71193009 | 81188237 | 1.309007491 | 5 | amp |
| TCGA-09-0366 | 18 | 225309 | 724647 | 1.041369918 | 4 | amp |
| TCGA-09-0366 | 18 | 2795905 | 2928683 | 1.365507688 | 5 | amp |
| TCGA-09-0366 | 18 | 2929049 | 5969596 | 0.973097129 | 4 | amp |
| TCGA-09-0366 | 18 | 6046701 | 6462950 | 0.64459645 | 3 | amp |

| | | | | | | |
|---|---|---|---|---|---|---|
| TCGA-09-0366 | 18 | 6837290 | 9195742 | 0.99444114 | 4 | amp |
| TCGA-09-0366 | 18 | 9280911 | 10546421 | 0.96146364 | 4 | amp |
| TCGA-09-0366 | 18 | 10689606 | 12452557 | 1.381626178 | 5 | amp |
| TCGA-09-0366 | 18 | 12453041 | 12785892 | 0.920583456 | 4 | amp |
| TCGA-09-0366 | 18 | 12794230 | 13073215 | 0.738493691 | 3 | amp |
| TCGA-09-0366 | 18 | 13086965 | 14543184 | 1.042560049 | 4 | amp |
| TCGA-09-0366 | 18 | 19120479 | 20602252 | 0.754402678 | 3 | amp |
| TCGA-09-0366 | 18 | 20606097 | 21481308 | 0.977457225 | 4 | amp |
| TCGA-09-0366 | 18 | 21482910 | 22932159 | 0.836192823 | 3 | amp |
| TCGA-09-0366 | 18 | 23637509 | 32418814 | 0.776678648 | 3 | amp |
| TCGA-09-0366 | 18 | 32428243 | 33059379 | 1.094270857 | 4 | amp |
| TCGA-09-0366 | 18 | 33060411 | 33747185 | 0.757301041 | 3 | amp |
| TCGA-09-0366 | 18 | 33749972 | 37202172 | 0.999625067 | 4 | amp |
| TCGA-09-0366 | 18 | 40857169 | 43467913 | 0.962263488 | 4 | amp |
| TCGA-09-0366 | 18 | 43469713 | 43833844 | 0.833641617 | 3 | amp |
| TCGA-09-0366 | 18 | 43842866 | 44336478 | 1.21981038 | 4 | amp |
| TCGA-09-0366 | 18 | 44392365 | 45377731 | 0.814546795 | 3 | amp |
| TCGA-09-0366 | 18 | 45566179 | 46570619 | 1.987406576 | 6 | amp |
| TCGA-09-0366 | 18 | 47009885 | 47373707 | 1.241309355 | 5 | amp |
| TCGA-09-0366 | 18 | 47375893 | 47787621 | 1.00510422 | 4 | amp |
| TCGA-09-0366 | 18 | 47788354 | 47908607 | 1.445588563 | 5 | amp |
| TCGA-09-0366 | 18 | 47911504 | 48422304 | 1.046391791 | 4 | amp |
| TCGA-09-0366 | 18 | 48434379 | 54694456 | 0.751872009 | 3 | amp |
| TCGA-09-0366 | 18 | 55020016 | 56171391 | 1.048518278 | 4 | amp |
| TCGA-09-0366 | 18 | 56182208 | 67788854 | 0.788432215 | 3 | amp |
| TCGA-09-0366 | 18 | 67863660 | 74649325 | 1.048601055 | 4 | amp |
| TCGA-09-0366 | 18 | 74659392 | 76757346 | 1.455754283 | 5 | amp |
| TCGA-09-0366 | 18 | 76936799 | 77960823 | 1.424126472 | 5 | amp |
| TCGA-09-0366 | 19 | 71882 | 4323644 | 1.162182641 | 5 | amp |
| TCGA-09-0366 | 19 | 4325198 | 9968583 | 0.844897749 | 3 | amp |
| TCGA-09-0366 | 19 | 10071039 | 10670239 | 1.837546704 | 5 | amp |
| TCGA-09-0366 | 19 | 10670276 | 10673518 | 3.095582581 | 6 | amp |
| TCGA-09-0366 | 19 | 10675567 | 10737012 | 1.801234532 | 5 | amp |
| TCGA-09-0366 | 19 | 10738344 | 10754067 | 3.250492007 | 6 | amp |
| TCGA-09-0366 | 19 | 10781222 | 10800041 | 1.52171286 | 5 | amp |
| TCGA-09-0366 | 19 | 10800072 | 11327800 | 2.152230618 | 6 | amp |
| TCGA-09-0366 | 19 | 11332491 | 11560000 | 1.64566843 | 5 | amp |
| TCGA-09-0366 | 19 | 11560003 | 15295303 | 0.897181103 | 3 | amp |
| TCGA-09-0366 | 19 | 15295993 | 16001302 | 1.848807443 | 5 | amp |
| TCGA-09-0366 | 19 | 16003066 | 16688505 | 2.670331567 | 6 | amp |
| TCGA-09-0366 | 19 | 16689092 | 17088383 | 1.790866171 | 5 | amp |
| TCGA-09-0366 | 19 | 17091281 | 17213367 | 2.501079778 | 6 | amp |
| TCGA-09-0366 | 19 | 17256193 | 17267903 | 1.368985152 | 5 | amp |
| TCGA-09-0366 | 19 | 17270189 | 19756798 | 3.364592255 | 6 | amp |
| TCGA-09-0366 | 19 | 19756964 | 30106303 | 1.310241838 | 5 | amp |
| TCGA-09-0366 | 19 | 30193541 | 30314742 | 6.048021935 | 6 | amp |
| TCGA-09-0366 | 19 | 30462056 | 30528881 | 1.445229794 | 5 | amp |
| TCGA-09-0366 | 19 | 30934384 | 31770676 | 4.432349965 | 6 | amp |
| TCGA-09-0366 | 19 | 32843719 | 35993121 | 1.525057102 | 5 | amp |
| TCGA-09-0366 | 19 | 35993669 | 36033947 | 2.659173092 | 6 | amp |

| | | | | | | |
|---|---|---|---|---|---|---|
| TCGA-09-0366 | 19 | 36034167 | 36215998 | 1.640212888 | 5 | amp |
| TCGA-09-0366 | 19 | 36216117 | 36271576 | 2.612167657 | 6 | amp |
| TCGA-09-0366 | 19 | 36271598 | 38966143 | 1.539830607 | 5 | amp |
| TCGA-09-0366 | 19 | 38968279 | 38987234 | 4.295819101 | 6 | amp |
| TCGA-09-0366 | 19 | 38989695 | 39329714 | 1.649250261 | 5 | amp |
| TCGA-09-0366 | 19 | 39334457 | 39338134 | 2.948016921 | 6 | amp |
| TCGA-09-0366 | 19 | 39360128 | 41084467 | 1.385484318 | 5 | amp |
| TCGA-09-0366 | 19 | 41086190 | 41096817 | 2.815881453 | 6 | amp |
| TCGA-09-0366 | 19 | 41096847 | 44339760 | 1.303544305 | 5 | amp |
| TCGA-09-0366 | 19 | 44341147 | 44972056 | 0.69726785 | 3 | amp |
| TCGA-09-0366 | 19 | 44976032 | 45735125 | 1.56519727 | 5 | amp |
| TCGA-09-0366 | 19 | 45766510 | 46027910 | 2.135428512 | 6 | amp |
| TCGA-09-0366 | 19 | 46029172 | 46299137 | 1.751484398 | 5 | amp |
| TCGA-09-0366 | 19 | 46299150 | 46314132 | 2.936627237 | 6 | amp |
| TCGA-09-0366 | 19 | 46317719 | 54611561 | 1.437539549 | 5 | amp |
| TCGA-09-0366 | 19 | 54611607 | 54649832 | 3.089109173 | 6 | amp |
| TCGA-09-0366 | 19 | 54650274 | 54657634 | 1.621822598 | 5 | amp |
| TCGA-09-0366 | 19 | 54660421 | 54850214 | 2.097132355 | 6 | amp |
| TCGA-09-0366 | 19 | 54850287 | 59063199 | 1.325924671 | 5 | amp |
| TCGA-09-0366 | 19 | 59063243 | 59110878 | 2.46522808 | 6 | amp |
| TCGA-09-0366 | 2 | 41527 | 1457650 | 1.306073497 | 5 | amp |
| TCGA-09-0366 | 2 | 1459830 | 1890404 | 2.22401953 | 6 | amp |
| TCGA-09-0366 | 2 | 1891200 | 8890473 | 1.540549478 | 5 | amp |
| TCGA-09-0366 | 2 | 8891564 | 9098818 | 0.704392311 | 3 | amp |
| TCGA-09-0366 | 2 | 9419421 | 11706813 | 1.21702438 | 5 | amp |
| TCGA-09-0366 | 2 | 11716452 | 11928607 | 2.186590931 | 6 | amp |
| TCGA-09-0366 | 2 | 11932025 | 15307489 | 1.585309122 | 5 | amp |
| TCGA-09-0366 | 2 | 15319055 | 20166697 | 0.874143524 | 3 | amp |
| TCGA-09-0366 | 2 | 20169153 | 24443994 | 1.250337451 | 5 | amp |
| TCGA-09-0366 | 2 | 24468973 | 24991294 | 0.840583364 | 3 | amp |
| TCGA-09-0366 | 2 | 25013244 | 25180880 | 1.550429544 | 5 | amp |
| TCGA-09-0366 | 2 | 25186248 | 25642453 | 1.94256762 | 6 | amp |
| TCGA-09-0366 | 2 | 25650391 | 26534970 | 1.152076925 | 5 | amp |
| TCGA-09-0366 | 2 | 26535780 | 26644271 | 2.121444308 | 6 | amp |
| TCGA-09-0366 | 2 | 26647080 | 26663435 | 1.257903482 | 5 | amp |
| TCGA-09-0366 | 2 | 26667006 | 27545409 | 2.267611337 | 6 | amp |
| TCGA-09-0366 | 2 | 27549530 | 27560899 | 1.369672557 | 5 | amp |
| TCGA-09-0366 | 2 | 27564777 | 27670793 | 2.393222625 | 6 | amp |
| TCGA-09-0366 | 2 | 27671689 | 27717335 | 1.438998568 | 5 | amp |
| TCGA-09-0366 | 2 | 27719711 | 27731136 | 1.94958289 | 6 | amp |
| TCGA-09-0366 | 2 | 27741609 | 31610779 | 1.32319348 | 5 | amp |
| TCGA-09-0366 | 2 | 31611066 | 45812949 | 0.917789543 | 3 | amp |
| TCGA-09-0366 | 2 | 45826540 | 47184181 | 1.38435464 | 5 | amp |
| TCGA-09-0366 | 2 | 47202026 | 47288128 | 2.164533534 | 6 | amp |
| TCGA-09-0366 | 2 | 47357039 | 47657100 | 1.145103203 | 5 | amp |
| TCGA-09-0366 | 2 | 47672681 | 69590859 | 0.917140333 | 3 | amp |
| TCGA-09-0366 | 2 | 69597126 | 71047084 | 1.227351813 | 5 | amp |
| TCGA-09-0366 | 2 | 71047564 | 71300727 | 1.88650056 | 6 | amp |
| TCGA-09-0366 | 2 | 71302668 | 71730456 | 1.163720788 | 5 | amp |
| TCGA-09-0366 | 2 | 71740798 | 71913662 | 2.194129124 | 6 | amp |

| | | | | | | |
|---|---|---|---|---|---|---|
| TCGA-09-0366 | 2 | 72360113 | 73316464 | 1.337822664 | 5 | amp |
| TCGA-09-0366 | 2 | 73441352 | 73478576 | 2.578145677 | 6 | amp |
| TCGA-09-0366 | 2 | 73479758 | 74329311 | 1.167733948 | 5 | amp |
| TCGA-09-0366 | 2 | 74362596 | 74789566 | 2.551295317 | 6 | amp |
| TCGA-09-0366 | 2 | 74802515 | 74842303 | 1.18620402 | 5 | amp |
| TCGA-09-0366 | 2 | 74867102 | 75081620 | 2.225547543 | 6 | amp |
| TCGA-09-0366 | 2 | 75094686 | 85536608 | 1.226833293 | 5 | amp |
| TCGA-09-0366 | 2 | 85542837 | 85852857 | 2.350787014 | 6 | amp |
| TCGA-09-0366 | 2 | 85857806 | 85875997 | 1.451192678 | 5 | amp |
| TCGA-09-0366 | 2 | 85890421 | 86014070 | 2.886144813 | 6 | amp |
| TCGA-09-0366 | 2 | 86067210 | 86090666 | 0.91554136 | 4 | amp |
| TCGA-09-0366 | 2 | 86094673 | 86276205 | 2.336449249 | 6 | amp |
| TCGA-09-0366 | 2 | 86276233 | 87178510 | 1.130727901 | 5 | amp |
| TCGA-09-0366 | 2 | 87180060 | 88116500 | 0.851443749 | 3 | amp |
| TCGA-09-0366 | 2 | 88118050 | 95943774 | 1.175408348 | 5 | amp |
| TCGA-09-0366 | 2 | 95944410 | 96116643 | 3.224742374 | 6 | amp |
| TCGA-09-0366 | 2 | 96116763 | 96595060 | 1.017121912 | 4 | amp |
| TCGA-09-0366 | 2 | 96652462 | 96801180 | 2.529858289 | 6 | amp |
| TCGA-09-0366 | 2 | 96803226 | 96859094 | 1.387364851 | 5 | amp |
| TCGA-09-0366 | 2 | 96860660 | 96951117 | 2.103954226 | 6 | amp |
| TCGA-09-0366 | 2 | 96951985 | 97279410 | 1.429930694 | 5 | amp |
| TCGA-09-0366 | 2 | 97285068 | 97399356 | 1.931431297 | 6 | amp |
| TCGA-09-0366 | 2 | 97400084 | 97463436 | 1.331096028 | 5 | amp |
| TCGA-09-0366 | 2 | 97464728 | 97533628 | 2.323928931 | 6 | amp |
| TCGA-09-0366 | 2 | 97691652 | 97756073 | 1.674372825 | 5 | amp |
| TCGA-09-0366 | 2 | 97757140 | 98253638 | 0.784143269 | 3 | amp |
| TCGA-09-0366 | 2 | 98263506 | 98341754 | 2.04178978 | 6 | amp |
| TCGA-09-0366 | 2 | 98349294 | 99636963 | 1.30156022 | 5 | amp |
| TCGA-09-0366 | 2 | 99651677 | 101126087 | 0.92006688 | 3 | amp |
| TCGA-09-0366 | 2 | 101182907 | 101885598 | 1.508134821 | 5 | amp |
| TCGA-09-0366 | 2 | 101885656 | 105705544 | 0.89203275 | 3 | amp |
| TCGA-09-0366 | 2 | 105706325 | 109089378 | 1.154642617 | 5 | amp |
| TCGA-09-0366 | 2 | 109091980 | 113514832 | 0.861342573 | 3 | amp |
| TCGA-09-0366 | 2 | 113518255 | 114475449 | 1.280088176 | 5 | amp |
| TCGA-09-0366 | 2 | 114476687 | 119699984 | 0.904905572 | 3 | amp |
| TCGA-09-0366 | 2 | 119726696 | 120252183 | 1.369254973 | 5 | amp |
| TCGA-09-0366 | 2 | 120362281 | 121050896 | 0.693905878 | 3 | amp |
| TCGA-09-0366 | 2 | 121106642 | 128631844 | 1.24352981 | 5 | amp |
| TCGA-09-0366 | 2 | 128699538 | 130869681 | 0.862738818 | 3 | amp |
| TCGA-09-0366 | 2 | 130872420 | 133489646 | 1.277771668 | 5 | amp |
| TCGA-09-0366 | 2 | 133531367 | 206418147 | 0.790236866 | 3 | amp |
| TCGA-09-0366 | 2 | 206480108 | 207027649 | 1.17375036 | 5 | amp |
| TCGA-09-0366 | 2 | 207040897 | 217069977 | 0.808349143 | 3 | amp |
| TCGA-09-0366 | 2 | 217123948 | 219110325 | 1.544464506 | 5 | amp |
| TCGA-09-0366 | 2 | 219114076 | 219269203 | 2.355644407 | 6 | amp |
| TCGA-09-0366 | 2 | 219288448 | 219679813 | 1.268087403 | 5 | amp |
| TCGA-09-0366 | 2 | 219688392 | 220112021 | 2.257167977 | 6 | amp |
| TCGA-09-0366 | 2 | 220112046 | 220309894 | 1.495397683 | 5 | amp |
| TCGA-09-0366 | 2 | 220315779 | 220506471 | 2.228588416 | 6 | amp |
| TCGA-09-0366 | 2 | 222290714 | 223436785 | 1.129320299 | 5 | amp |

| | | | | | | |
|---|---|---|---|---|---|---|
| TCGA-09-0366 | 2 | 223464604 | 232081548 | 0.810357449 | 3 | amp |
| TCGA-09-0366 | 2 | 232087396 | 233349805 | 1.21671438 | 5 | amp |
| TCGA-09-0366 | 2 | 233349852 | 233410459 | 2.202662501 | 6 | amp |
| TCGA-09-0366 | 2 | 233421122 | 234377253 | 1.255276619 | 5 | amp |
| TCGA-09-0366 | 2 | 234389835 | 234602560 | 0.654849775 | 3 | amp |
| TCGA-09-0366 | 2 | 234621590 | 241069506 | 1.221426811 | 5 | amp |
| TCGA-09-0366 | 2 | 241073325 | 241468929 | 2.107238683 | 6 | amp |
| TCGA-09-0366 | 2 | 241492252 | 241537851 | 1.318693415 | 5 | amp |
| TCGA-09-0366 | 2 | 241555716 | 241700839 | 2.259104717 | 6 | amp |
| TCGA-09-0366 | 2 | 241712481 | 242149993 | 1.458944128 | 5 | amp |
| TCGA-09-0366 | 2 | 242151504 | 242169745 | 2.324146664 | 6 | amp |
| TCGA-09-0366 | 2 | 242170146 | 243160772 | 1.412092951 | 5 | amp |
| TCGA-09-0366 | 20 | 68319 | 210465 | 0.995576136 | 4 | amp |
| TCGA-09-0366 | 20 | 238388 | 3285198 | 1.754012876 | 5 | amp |
| TCGA-09-0366 | 20 | 3295633 | 3619588 | 0.80860863 | 4 | amp |
| TCGA-09-0366 | 20 | 3624797 | 6759749 | 1.607180726 | 5 | amp |
| TCGA-09-0366 | 20 | 7864214 | 9459671 | 1.007538267 | 4 | amp |
| TCGA-09-0366 | 20 | 9496065 | 10644696 | 1.288396194 | 5 | amp |
| TCGA-09-0366 | 20 | 11898876 | 17339088 | 0.90595213 | 4 | amp |
| TCGA-09-0366 | 20 | 17341170 | 18365245 | 1.465815574 | 5 | amp |
| TCGA-09-0366 | 20 | 18370288 | 23425521 | 0.998188823 | 4 | amp |
| TCGA-09-0366 | 20 | 23431124 | 32883415 | 1.900738552 | 6 | amp |
| TCGA-09-0366 | 20 | 32981592 | 33370160 | 1.02149871 | 4 | amp |
| TCGA-09-0366 | 20 | 33433092 | 34215388 | 1.46084448 | 5 | amp |
| TCGA-09-0366 | 20 | 34218296 | 34502156 | 0.997901487 | 4 | amp |
| TCGA-09-0366 | 20 | 34505344 | 37667223 | 2.56073861 | 6 | amp |
| TCGA-09-0366 | 20 | 39316511 | 42157400 | 0.982775053 | 4 | amp |
| TCGA-09-0366 | 20 | 42157895 | 44108777 | 2.360459179 | 6 | amp |
| TCGA-09-0366 | 20 | 44163034 | 44354330 | 0.87403109 | 4 | amp |
| TCGA-09-0366 | 20 | 44402984 | 47364441 | 1.701774763 | 5 | amp |
| TCGA-09-0366 | 20 | 47557690 | 47782755 | 0.866759334 | 4 | amp |
| TCGA-09-0366 | 20 | 47835835 | 58420341 | 1.52541644 | 5 | amp |
| TCGA-09-0366 | 20 | 58422087 | 58490622 | 0.609520215 | 3 | amp |
| TCGA-09-0366 | 20 | 58491523 | 58559868 | 0.95991055 | 4 | amp |
| TCGA-09-0366 | 20 | 58559999 | 62926333 | 2.072877077 | 6 | amp |
| TCGA-09-0366 | 21 | 11097535 | 32127790 | 0.836144269 | 3 | amp |
| TCGA-09-0366 | 21 | 32185292 | 35209351 | 1.094520414 | 4 | amp |
| TCGA-09-0366 | 21 | 35228980 | 38460213 | 1.304482039 | 5 | amp |
| TCGA-09-0366 | 21 | 38560755 | 40668299 | 0.903127085 | 3 | amp |
| TCGA-09-0366 | 21 | 40670275 | 42598296 | 1.03160554 | 4 | amp |
| TCGA-09-0366 | 21 | 42609427 | 48111215 | 1.762677768 | 5 | amp |
| TCGA-09-0366 | 22 | 16084594 | 16984326 | 0.911047803 | 3 | amp |
| TCGA-09-0366 | 22 | 16988940 | 21106062 | 1.333423355 | 5 | amp |
| TCGA-09-0366 | 22 | 21107132 | 21304185 | 0.805612143 | 3 | amp |
| TCGA-09-0366 | 22 | 21329933 | 25115616 | 1.32259668 | 5 | amp |
| TCGA-09-0366 | 22 | 25115668 | 25240961 | 0.746775082 | 3 | amp |
| TCGA-09-0366 | 22 | 25243561 | 25756106 | 1.285459921 | 5 | amp |
| TCGA-09-0366 | 22 | 26157017 | 26399312 | 1.28543851 | 5 | amp |
| TCGA-09-0366 | 22 | 26400632 | 29414060 | 0.90880559 | 3 | amp |
| TCGA-09-0366 | 22 | 29438459 | 30202567 | 1.150737042 | 5 | amp |

| | | | | | | |
|---|---|---|---|---|---|---|
| TCGA-09-0366 | 22 | 30202749 | 30572163 | 0.744816912 | 3 | amp |
| TCGA-09-0366 | 22 | 30639604 | 31796787 | 1.347636798 | 5 | amp |
| TCGA-09-0366 | 22 | 31798982 | 35481746 | 0.832242596 | 3 | amp |
| TCGA-09-0366 | 22 | 35658308 | 35948114 | 1.297540957 | 5 | amp |
| TCGA-09-0366 | 22 | 36003266 | 36629263 | 0.732093179 | 3 | amp |
| TCGA-09-0366 | 22 | 36649962 | 38710184 | 1.505444629 | 5 | amp |
| TCGA-09-0366 | 22 | 38757447 | 38951447 | 0.696673899 | 3 | amp |
| TCGA-09-0366 | 22 | 38958272 | 40078703 | 1.604159712 | 5 | amp |
| TCGA-09-0366 | 22 | 40139625 | 41556745 | 0.905693752 | 3 | amp |
| TCGA-09-0366 | 22 | 41601318 | 43936241 | 1.422621166 | 5 | amp |
| TCGA-09-0366 | 22 | 43950742 | 44161288 | 0.639690628 | 3 | amp |
| TCGA-09-0366 | 22 | 44168757 | 45691640 | 1.537523684 | 5 | amp |
| TCGA-09-0366 | 22 | 45718223 | 45802749 | 0.826892849 | 3 | amp |
| TCGA-09-0366 | 22 | 45804589 | 51237627 | 1.317308477 | 5 | amp |
| TCGA-09-0366 | 3 | 361444 | 8579178 | 0.820391782 | 3 | amp |
| TCGA-09-0366 | 3 | 8590241 | 9746686 | 1.159580468 | 5 | amp |
| TCGA-09-0366 | 3 | 9747312 | 9757748 | 2.286633234 | 6 | amp |
| TCGA-09-0366 | 3 | 9758704 | 9799591 | 1.805002762 | 5 | amp |
| TCGA-09-0366 | 3 | 9800830 | 9960115 | 2.10186557 | 6 | amp |
| TCGA-09-0366 | 3 | 9960148 | 15265084 | 1.431334484 | 5 | amp |
| TCGA-09-0366 | 3 | 15269408 | 32181908 | 0.786263126 | 3 | amp |
| TCGA-09-0366 | 3 | 32188109 | 33183973 | 1.232200665 | 5 | amp |
| TCGA-09-0366 | 3 | 33194219 | 37476654 | 0.699572707 | 3 | amp |
| TCGA-09-0366 | 3 | 37512441 | 38802898 | 1.376458211 | 5 | amp |
| TCGA-09-0366 | 3 | 38804951 | 39136276 | 0.752459931 | 3 | amp |
| TCGA-09-0366 | 3 | 39139643 | 40574323 | 1.296608405 | 5 | amp |
| TCGA-09-0366 | 3 | 40803147 | 41979699 | 0.721377047 | 3 | amp |
| TCGA-09-0366 | 3 | 41996062 | 43122951 | 1.214009629 | 5 | amp |
| TCGA-09-0366 | 3 | 43344689 | 46619536 | 0.885937799 | 3 | amp |
| TCGA-09-0366 | 3 | 46620550 | 47632358 | 1.223710036 | 5 | amp |
| TCGA-09-0366 | 3 | 47651506 | 48436134 | 0.895281462 | 3 | amp |
| TCGA-09-0366 | 3 | 48445854 | 48602434 | 1.422459806 | 5 | amp |
| TCGA-09-0366 | 3 | 48602527 | 48626485 | 2.903695767 | 6 | amp |
| TCGA-09-0366 | 3 | 48626971 | 50385380 | 1.31668327 | 5 | amp |
| TCGA-09-0366 | 3 | 50385493 | 50418611 | 2.481963771 | 6 | amp |
| TCGA-09-0366 | 3 | 50421547 | 50683238 | 1.383874525 | 5 | amp |
| TCGA-09-0366 | 3 | 50683482 | 51718659 | 0.793047709 | 3 | amp |
| TCGA-09-0366 | 3 | 51733355 | 52588937 | 1.405558092 | 5 | amp |
| TCGA-09-0366 | 3 | 52595733 | 52802666 | 0.826449979 | 3 | amp |
| TCGA-09-0366 | 3 | 52811569 | 52819272 | 1.235396226 | 5 | amp |
| TCGA-09-0366 | 3 | 52820887 | 52824970 | 4.268617432 | 6 | amp |
| TCGA-09-0366 | 3 | 52825528 | 52858616 | 1.467898218 | 5 | amp |
| TCGA-09-0366 | 3 | 52858765 | 53215312 | 0.772367625 | 3 | amp |
| TCGA-09-0366 | 3 | 53215390 | 53535754 | 1.3510497 | 5 | amp |
| TCGA-09-0366 | 3 | 53684755 | 89499543 | 0.698469157 | 3 | amp |
| TCGA-09-0366 | 3 | 89521571 | 99536915 | 1.139868582 | 5 | amp |
| TCGA-09-0366 | 3 | 99552031 | 101212832 | 0.938897412 | 3 | amp |
| TCGA-09-0366 | 3 | 101219878 | 105243362 | 1.421436381 | 5 | amp |
| TCGA-09-0366 | 3 | 105250787 | 108822789 | 0.895499714 | 3 | amp |
| TCGA-09-0366 | 3 | 108829552 | 112992231 | 1.140111068 | 5 | amp |

| | | | | | | |
|---|---|---|---|---|---|---|
| TCGA-09-0366 | 3 | 112993165 | 112999517 | 2.420392423 | 6 | amp |
| TCGA-09-0366 | 3 | 112999818 | 120315416 | 1.147977801 | 5 | amp |
| TCGA-09-0366 | 3 | 120319910 | 121212626 | 0.814387259 | 3 | amp |
| TCGA-09-0366 | 3 | 121215596 | 122634798 | 1.193999169 | 5 | amp |
| TCGA-09-0366 | 3 | 122642411 | 122680168 | 2.332947099 | 6 | amp |
| TCGA-09-0366 | 3 | 122807927 | 125509489 | 1.350660668 | 5 | amp |
| TCGA-09-0366 | 3 | 125639647 | 127393470 | 2.481045499 | 6 | amp |
| TCGA-09-0366 | 3 | 127395074 | 127774614 | 1.492231824 | 5 | amp |
| TCGA-09-0366 | 3 | 127775497 | 127816361 | 2.222743428 | 6 | amp |
| TCGA-09-0366 | 3 | 127817696 | 127831916 | 1.377397887 | 5 | amp |
| TCGA-09-0366 | 3 | 127838153 | 128349009 | 2.466506666 | 6 | amp |
| TCGA-09-0366 | 3 | 128350745 | 128532277 | 1.496742698 | 5 | amp |
| TCGA-09-0366 | 3 | 128584087 | 128852945 | 2.267775329 | 6 | amp |
| TCGA-09-0366 | 3 | 128852975 | 129234446 | 1.731415794 | 5 | amp |
| TCGA-09-0366 | 3 | 129236254 | 129304979 | 2.366963712 | 6 | amp |
| TCGA-09-0366 | 3 | 129370270 | 129547263 | 0.82750936 | 3 | amp |
| TCGA-09-0366 | 3 | 129694644 | 129812033 | 2.366963712 | 6 | amp |
| TCGA-09-0366 | 3 | 129817111 | 130399570 | 1.228770267 | 5 | amp |
| TCGA-09-0366 | 3 | 130399804 | 132418953 | 0.891564314 | 3 | amp |
| TCGA-09-0366 | 3 | 132419174 | 138119475 | 1.228142917 | 5 | amp |
| TCGA-09-0366 | 3 | 138121001 | 138183391 | 2.74961423 | 6 | amp |
| TCGA-09-0366 | 3 | 138186336 | 138384081 | 1.436564832 | 5 | amp |
| TCGA-09-0366 | 3 | 138400787 | 182770047 | 0.949845838 | 3 | amp |
| TCGA-09-0366 | 3 | 182775094 | 183824486 | 1.239536542 | 5 | amp |
| TCGA-09-0366 | 3 | 183854341 | 184298976 | 2.935689234 | 6 | amp |
| TCGA-09-0366 | 3 | 184298998 | 190282170 | 1.28551583 | 5 | amp |
| TCGA-09-0366 | 3 | 190321879 | 194336481 | 0.960168058 | 3 | amp |
| TCGA-09-0366 | 3 | 194346582 | 195802250 | 1.398218431 | 5 | amp |
| TCGA-09-0366 | 3 | 195803892 | 196053924 | 2.097721577 | 6 | amp |
| TCGA-09-0366 | 3 | 196054210 | 197955154 | 1.302724666 | 5 | amp |
| TCGA-09-0366 | 4 | 53323 | 2160969 | 1.557364924 | 5 | amp |
| TCGA-09-0366 | 4 | 2172393 | 2231011 | 0.805996195 | 4 | amp |
| TCGA-09-0366 | 4 | 2233650 | 3123176 | 1.267242535 | 5 | amp |
| TCGA-09-0366 | 4 | 3124579 | 3190868 | 0.896137446 | 4 | amp |
| TCGA-09-0366 | 4 | 3201442 | 6580232 | 1.417085968 | 5 | amp |
| TCGA-09-0366 | 4 | 6588688 | 6602536 | 3.140007912 | 6 | amp |
| TCGA-09-0366 | 4 | 6606708 | 9177142 | 1.599564882 | 5 | amp |
| TCGA-09-0366 | 4 | 9212938 | 9558031 | 1.005098168 | 4 | amp |
| TCGA-09-0366 | 4 | 9699827 | 10503077 | 1.40667175 | 5 | amp |
| TCGA-09-0366 | 4 | 10509561 | 17830015 | 0.821888234 | 4 | amp |
| TCGA-09-0366 | 4 | 24854687 | 26388053 | 0.834344766 | 4 | amp |
| TCGA-09-0366 | 4 | 36195174 | 38937434 | 0.894234045 | 4 | amp |
| TCGA-09-0366 | 4 | 39328131 | 48514721 | 0.803251838 | 4 | amp |
| TCGA-09-0366 | 4 | 52895816 | 57873194 | 0.829643936 | 4 | amp |
| TCGA-09-0366 | 4 | 73433044 | 85676605 | 0.798289525 | 4 | amp |
| TCGA-09-0366 | 4 | 186296763 | 190948390 | 0.789845119 | 4 | amp |
| TCGA-09-0366 | 5 | 151610 | 884238 | 1.895521324 | 6 | amp |
| TCGA-09-0366 | 5 | 886674 | 7875549 | 1.344216846 | 5 | amp |
| TCGA-09-0366 | 5 | 7878005 | 14331067 | 0.985554338 | 4 | amp |
| TCGA-09-0366 | 5 | 14336590 | 16711411 | 1.374193842 | 5 | amp |

| | | | | | | |
|---|---|---|---|---|---|---|
| TCGA-09-0366 | 5 | 16754916 | 34829929 | 0.982676563 | 4 | amp |
| TCGA-09-0366 | 5 | 34830741 | 34950480 | 1.316033829 | 5 | amp |
| TCGA-09-0366 | 5 | 34954008 | 38418382 | 0.981307932 | 4 | amp |
| TCGA-09-0366 | 5 | 38425011 | 38438586 | 1.409122209 | 5 | amp |
| TCGA-09-0366 | 5 | 38445732 | 38463121 | 2.372175096 | 6 | amp |
| TCGA-09-0366 | 5 | 38463860 | 38943123 | 0.87472863 | 4 | amp |
| TCGA-09-0366 | 5 | 38944489 | 39138835 | 0.714057016 | 3 | amp |
| TCGA-09-0366 | 5 | 39139284 | 43619277 | 0.906527247 | 4 | amp |
| TCGA-09-0366 | 5 | 43624117 | 45304000 | 0.813231128 | 3 | amp |
| TCGA-09-0366 | 5 | 66389404 | 79084980 | 0.676984761 | 3 | amp |
| TCGA-09-0366 | 5 | 79086782 | 79647792 | 1.046318669 | 4 | amp |
| TCGA-09-0366 | 5 | 79654965 | 81283503 | 0.687262679 | 3 | amp |
| TCGA-09-0366 | 5 | 123966326 | 126887623 | 0.766859671 | 3 | amp |
| TCGA-09-0366 | 5 | 131046252 | 131298419 | 0.71392585 | 3 | amp |
| TCGA-09-0366 | 5 | 131302046 | 131822853 | 1.067000233 | 4 | amp |
| TCGA-09-0366 | 5 | 131877491 | 132433035 | 0.727264644 | 3 | amp |
| TCGA-09-0366 | 5 | 132435230 | 134002719 | 0.952823803 | 4 | amp |
| TCGA-09-0366 | 5 | 134007477 | 134154741 | 0.646067341 | 3 | amp |
| TCGA-09-0366 | 5 | 134162613 | 137235435 | 0.942822999 | 4 | amp |
| TCGA-09-0366 | 5 | 137241888 | 137527648 | 0.660796329 | 3 | amp |
| TCGA-09-0366 | 5 | 137527897 | 137756702 | 0.967181214 | 4 | amp |
| TCGA-09-0366 | 5 | 137759686 | 137844094 | 1.519245575 | 5 | amp |
| TCGA-09-0366 | 5 | 137844311 | 138665118 | 0.994081325 | 4 | amp |
| TCGA-09-0366 | 5 | 138699422 | 139494642 | 1.310458484 | 5 | amp |
| TCGA-09-0366 | 5 | 139508022 | 139828932 | 1.056520445 | 4 | amp |
| TCGA-09-0366 | 5 | 139838148 | 139928695 | 0.589009789 | 3 | amp |
| TCGA-09-0366 | 5 | 139928742 | 140890746 | 1.309563696 | 5 | amp |
| TCGA-09-0366 | 5 | 140896376 | 140896616 | 1.077858594 | 4 | amp |
| TCGA-09-0366 | 5 | 140903662 | 140966806 | 0.576215737 | 3 | amp |
| TCGA-09-0366 | 5 | 140967743 | 141309345 | 1.629462926 | 5 | amp |
| TCGA-09-0366 | 5 | 141309506 | 141391658 | 1.089492725 | 4 | amp |
| TCGA-09-0366 | 5 | 141511326 | 148386661 | 0.66924694 | 3 | amp |
| TCGA-09-0366 | 5 | 148388368 | 149435962 | 1.096223285 | 4 | amp |
| TCGA-09-0366 | 5 | 149436808 | 150056472 | 1.483682779 | 5 | amp |
| TCGA-09-0366 | 5 | 150071227 | 150282769 | 0.936449765 | 4 | amp |
| TCGA-09-0366 | 5 | 150404874 | 150567043 | 1.258027328 | 5 | amp |
| TCGA-09-0366 | 5 | 150578511 | 151055780 | 0.966246145 | 4 | amp |
| TCGA-09-0366 | 5 | 151125827 | 153760219 | 0.721735131 | 3 | amp |
| TCGA-09-0366 | 5 | 153765811 | 156464374 | 0.94168649 | 4 | amp |
| TCGA-09-0366 | 5 | 156469605 | 161580416 | 0.663090212 | 3 | amp |
| TCGA-09-0366 | 5 | 162866184 | 168100378 | 1.053937673 | 4 | amp |
| TCGA-09-0366 | 5 | 168110885 | 168181060 | 1.537750393 | 5 | amp |
| TCGA-09-0366 | 5 | 168187843 | 175775114 | 1.002937213 | 4 | amp |
| TCGA-09-0366 | 5 | 175775215 | 175933979 | 1.265605201 | 5 | amp |
| TCGA-09-0366 | 5 | 175992289 | 176323172 | 2.177240203 | 6 | amp |
| TCGA-09-0366 | 5 | 176332267 | 176733197 | 0.83135324 | 4 | amp |
| TCGA-09-0366 | 5 | 176733376 | 177054619 | 1.307298555 | 5 | amp |
| TCGA-09-0366 | 5 | 177058481 | 177474135 | 0.927576045 | 4 | amp |
| TCGA-09-0366 | 5 | 177482599 | 177666220 | 1.486651469 | 5 | amp |
| TCGA-09-0366 | 5 | 177669308 | 177987775 | 1.958154072 | 6 | amp |

| | | | | | | |
|---|---|---|---|---|---|---|
| TCGA-09-0366 | 5 | 178030567 | 180899507 | 1.206714666 | 5 | amp |
| TCGA-09-0366 | 6 | 105907 | 171055029 | 1.396215347 | 5 | amp |
| TCGA-09-0366 | 7 | 540503 | 5267012 | 1.752866222 | 5 | amp |
| TCGA-09-0366 | 7 | 5269183 | 5643307 | 2.270475511 | 6 | amp |
| TCGA-09-0366 | 7 | 5643427 | 6826700 | 1.479959355 | 5 | amp |
| TCGA-09-0366 | 7 | 6829205 | 6970595 | 1.107905493 | 4 | amp |
| TCGA-09-0366 | 7 | 7118571 | 22196526 | 0.757717442 | 3 | amp |
| TCGA-09-0366 | 7 | 22197427 | 30402111 | 0.922895853 | 4 | amp |
| TCGA-09-0366 | 7 | 30465199 | 31144562 | 1.540341749 | 5 | amp |
| TCGA-09-0366 | 7 | 31146083 | 39046518 | 0.766920168 | 3 | amp |
| TCGA-09-0366 | 7 | 39125425 | 43846884 | 1.009773107 | 4 | amp |
| TCGA-09-0366 | 7 | 43906259 | 44047397 | 1.386174844 | 5 | amp |
| TCGA-09-0366 | 7 | 44054171 | 44620884 | 1.97775509 | 6 | amp |
| TCGA-09-0366 | 7 | 44620990 | 47451422 | 1.409923923 | 5 | amp |
| TCGA-09-0366 | 7 | 47453499 | 48147150 | 1.121292656 | 4 | amp |
| TCGA-09-0366 | 7 | 48147734 | 50097780 | 0.727271953 | 3 | amp |
| TCGA-09-0366 | 7 | 50121312 | 50660841 | 1.09185546 | 4 | amp |
| TCGA-09-0366 | 7 | 50663090 | 55002403 | 1.321247166 | 5 | amp |
| TCGA-09-0366 | 7 | 55002883 | 55273370 | 2.115976967 | 6 | amp |
| TCGA-09-0366 | 7 | 55459424 | 56066792 | 1.458600025 | 5 | amp |
| TCGA-09-0366 | 7 | 56079448 | 57142378 | 2.262439098 | 6 | amp |
| TCGA-09-0366 | 7 | 57187542 | 65429497 | 1.092297929 | 5 | amp |
| TCGA-09-0366 | 7 | 65432655 | 65592768 | 2.163843436 | 6 | amp |
| TCGA-09-0366 | 7 | 65595719 | 66490067 | 1.262158908 | 5 | amp |
| TCGA-09-0366 | 7 | 66514874 | 72341127 | 1.034720002 | 4 | amp |
| TCGA-09-0366 | 7 | 72361109 | 72470093 | 1.932747373 | 6 | amp |
| TCGA-09-0366 | 7 | 72470272 | 72619511 | 1.248978414 | 5 | amp |
| TCGA-09-0366 | 7 | 72634656 | 72648759 | 2.193040707 | 6 | amp |
| TCGA-09-0366 | 7 | 72657262 | 72685718 | 1.274239855 | 5 | amp |
| TCGA-09-0366 | 7 | 72693953 | 72754856 | 1.992874102 | 6 | amp |
| TCGA-09-0366 | 7 | 72755236 | 72925243 | 1.150648784 | 5 | amp |
| TCGA-09-0366 | 7 | 72951583 | 74016785 | 2.061777414 | 6 | amp |
| TCGA-09-0366 | 7 | 74103421 | 74328258 | 1.345975602 | 5 | amp |
| TCGA-09-0366 | 7 | 74329226 | 74486638 | 2.327543413 | 6 | amp |
| TCGA-09-0366 | 7 | 74524745 | 74558500 | 1.214990533 | 5 | amp |
| TCGA-09-0366 | 7 | 74559710 | 74587771 | 2.072405432 | 6 | amp |
| TCGA-09-0366 | 7 | 74602928 | 74714699 | 1.206612591 | 5 | amp |
| TCGA-09-0366 | 7 | 74765929 | 74824247 | 2.168137349 | 6 | amp |
| TCGA-09-0366 | 7 | 74911430 | 76247656 | 1.598725806 | 5 | amp |
| TCGA-09-0366 | 7 | 76254799 | 76687189 | 1.005446017 | 4 | amp |
| TCGA-09-0366 | 7 | 76688446 | 96639413 | 0.672360033 | 3 | amp |
| TCGA-09-0366 | 7 | 96650032 | 99235946 | 1.54806619 | 5 | amp |
| TCGA-09-0366 | 7 | 99245915 | 99461359 | 0.738723936 | 4 | amp |
| TCGA-09-0366 | 7 | 99463516 | 99669840 | 1.398992934 | 5 | amp |
| TCGA-09-0366 | 7 | 99672670 | 99772407 | 2.115114925 | 6 | amp |
| TCGA-09-0366 | 7 | 99773145 | 100014867 | 1.150657085 | 5 | amp |
| TCGA-09-0366 | 7 | 100016682 | 100198579 | 2.334904486 | 6 | amp |
| TCGA-09-0366 | 7 | 100200066 | 100238521 | 1.521643387 | 5 | amp |
| TCGA-09-0366 | 7 | 100238551 | 100486060 | 2.312441431 | 6 | amp |
| TCGA-09-0366 | 7 | 100486072 | 100780764 | 1.429892546 | 5 | amp |

| | | | | | | |
|---|---|---|---|---|---|---|
| TCGA-09-0366 | 7 | 100799843 | 101961003 | 2.028266509 | 6 | amp |
| TCGA-09-0366 | 7 | 101967609 | 102045147 | 1.344126775 | 5 | amp |
| TCGA-09-0366 | 7 | 102047852 | 102343959 | 2.255738119 | 6 | amp |
| TCGA-09-0366 | 7 | 102453763 | 104909331 | 0.722359549 | 3 | amp |
| TCGA-09-0366 | 7 | 105098184 | 106523662 | 1.202644393 | 4 | amp |
| TCGA-09-0366 | 7 | 106524565 | 127229653 | 0.700037667 | 3 | amp |
| TCGA-09-0366 | 7 | 127230065 | 127638092 | 1.074875207 | 4 | amp |
| TCGA-09-0366 | 7 | 127668682 | 128852312 | 1.293160466 | 5 | amp |
| TCGA-09-0366 | 7 | 129008303 | 131172546 | 1.054534895 | 4 | amp |
| TCGA-09-0366 | 7 | 131189048 | 132070060 | 1.52901853 | 5 | amp |
| TCGA-09-0366 | 7 | 132169553 | 134813739 | 0.912114805 | 4 | amp |
| TCGA-09-0366 | 7 | 134849142 | 135073655 | 1.626708955 | 5 | amp |
| TCGA-09-0366 | 7 | 135078588 | 140043407 | 0.953690709 | 4 | amp |
| TCGA-09-0366 | 7 | 140044949 | 140302234 | 1.360155053 | 5 | amp |
| TCGA-09-0366 | 7 | 140373116 | 140394652 | 2.214623709 | 6 | amp |
| TCGA-09-0366 | 7 | 140397974 | 142460914 | 0.931277333 | 4 | amp |
| TCGA-09-0366 | 7 | 142471717 | 142499157 | 1.511962662 | 5 | amp |
| TCGA-09-0366 | 7 | 142499576 | 142637676 | 2.042943384 | 6 | amp |
| TCGA-09-0366 | 7 | 142638299 | 142651642 | 1.33606297 | 5 | amp |
| TCGA-09-0366 | 7 | 142654866 | 142659351 | 2.295729579 | 6 | amp |
| TCGA-09-0366 | 7 | 142723272 | 144228221 | 1.546228379 | 5 | amp |
| TCGA-09-0366 | 7 | 144245579 | 148544451 | 1.028646451 | 4 | amp |
| TCGA-09-0366 | 7 | 148700823 | 149492810 | 1.566748212 | 5 | amp |
| TCGA-09-0366 | 7 | 149502465 | 149572770 | 2.388478835 | 6 | amp |
| TCGA-09-0366 | 7 | 149588275 | 150491163 | 1.153892106 | 5 | amp |
| TCGA-09-0366 | 7 | 150493405 | 150939330 | 2.179800002 | 6 | amp |
| TCGA-09-0366 | 7 | 150939507 | 151135385 | 1.358277211 | 5 | amp |
| TCGA-09-0366 | 7 | 151164160 | 158672707 | 1.027850698 | 4 | amp |
| TCGA-09-0366 | 7 | 158677222 | 158935247 | 1.339303677 | 5 | amp |
| TCGA-09-0366 | 8 | 116074 | 413198 | 0.701053596 | 3 | amp |
| TCGA-09-0366 | 8 | 417672 | 1824938 | 0.915495205 | 4 | amp |
| TCGA-09-0366 | 8 | 1828121 | 2071241 | 1.146307745 | 5 | amp |
| TCGA-09-0366 | 8 | 2071381 | 6783580 | 0.659820688 | 3 | amp |
| TCGA-09-0366 | 8 | 6793452 | 7438186 | 0.916355612 | 4 | amp |
| TCGA-09-0366 | 8 | 7574954 | 7614273 | 1.487704056 | 5 | amp |
| TCGA-09-0366 | 8 | 7621321 | 7809920 | 0.709841269 | 3 | amp |
| TCGA-09-0366 | 8 | 7825148 | 8097806 | 1.081106667 | 4 | amp |
| TCGA-09-0366 | 8 | 8175629 | 10383292 | 0.64496768 | 3 | amp |
| TCGA-09-0366 | 8 | 10386992 | 12175856 | 0.961797653 | 4 | amp |
| TCGA-09-0366 | 8 | 12211888 | 12291651 | 1.145447483 | 5 | amp |
| TCGA-09-0366 | 8 | 12435418 | 12968351 | 0.718890858 | 3 | amp |
| TCGA-09-0366 | 8 | 19263255 | 20077963 | 0.623186174 | 3 | amp |
| TCGA-09-0366 | 8 | 20107163 | 21892097 | 0.912006992 | 4 | amp |
| TCGA-09-0366 | 8 | 21903593 | 22102575 | 1.43897928 | 5 | amp |
| TCGA-09-0366 | 8 | 22104695 | 22386114 | 0.686569705 | 3 | amp |
| TCGA-09-0366 | 8 | 22389718 | 22426843 | 0.913029524 | 4 | amp |
| TCGA-09-0366 | 8 | 22429222 | 23057474 | 1.206844497 | 5 | amp |
| TCGA-09-0366 | 8 | 23057975 | 23301474 | 0.952225145 | 4 | amp |
| TCGA-09-0366 | 8 | 23301923 | 23712097 | 0.76244571 | 3 | amp |
| TCGA-09-0366 | 8 | 24773059 | 27099293 | 0.684038511 | 3 | amp |

| | | | | | | |
|---|---|---|---|---|---|---|
| TCGA-09-0366 | 8 | 27099871 | 27316038 | 1.218856259 | 5 | amp |
| TCGA-09-0366 | 8 | 27319088 | 27528882 | 0.979772123 | 4 | amp |
| TCGA-09-0366 | 8 | 27533983 | 30690880 | 0.673640177 | 3 | amp |
| TCGA-09-0366 | 8 | 33310700 | 33356802 | 0.744083266 | 3 | amp |
| TCGA-09-0366 | 8 | 33357776 | 33455042 | 1.206020564 | 4 | amp |
| TCGA-09-0366 | 8 | 36768422 | 36793474 | 0.640072437 | 3 | amp |
| TCGA-09-0366 | 8 | 37595433 | 37635676 | 0.951916752 | 4 | amp |
| TCGA-09-0366 | 8 | 37672359 | 37707344 | 1.144024259 | 5 | amp |
| TCGA-09-0366 | 8 | 37720371 | 38264990 | 0.91786367 | 4 | amp |
| TCGA-09-0366 | 8 | 38265700 | 38458768 | 1.252091663 | 5 | amp |
| TCGA-09-0366 | 8 | 38646219 | 38834275 | 0.8680279 | 4 | amp |
| TCGA-09-0366 | 8 | 38835448 | 38853049 | 1.580270035 | 5 | amp |
| TCGA-09-0366 | 8 | 39682316 | 40554961 | 0.79195653 | 3 | amp |
| TCGA-09-0366 | 8 | 40625144 | 41364661 | 0.914739388 | 4 | amp |
| TCGA-09-0366 | 8 | 41387709 | 41799020 | 1.615683833 | 5 | amp |
| TCGA-09-0366 | 8 | 41800294 | 42036617 | 0.943429618 | 4 | amp |
| TCGA-09-0366 | 8 | 42037393 | 42403980 | 1.418472579 | 5 | amp |
| TCGA-09-0366 | 8 | 42407658 | 43171108 | 0.908330368 | 4 | amp |
| TCGA-09-0366 | 8 | 43172458 | 53079598 | 0.725410596 | 3 | amp |
| TCGA-09-0366 | 8 | 53597933 | 68018234 | 0.677988157 | 3 | amp |
| TCGA-09-0366 | 8 | 69699664 | 75898531 | 0.659108744 | 3 | amp |
| TCGA-09-0366 | 8 | 79672321 | 88364027 | 0.689391523 | 3 | amp |
| TCGA-09-0366 | 8 | 95541182 | 99057644 | 0.692391246 | 3 | amp |
| TCGA-09-0366 | 8 | 124383872 | 126023886 | 0.667593403 | 3 | amp |
| TCGA-09-0366 | 8 | 133879218 | 139729170 | 0.745967171 | 3 | amp |
| TCGA-09-0366 | 8 | 139732918 | 141449343 | 0.892751187 | 4 | amp |
| TCGA-09-0366 | 8 | 141460855 | 141669790 | 1.170238898 | 5 | amp |
| TCGA-09-0366 | 8 | 141674995 | 141696821 | 0.728868083 | 3 | amp |
| TCGA-09-0366 | 8 | 142173389 | 145541525 | 1.174381918 | 5 | amp |
| TCGA-09-0366 | 8 | 145544967 | 146279593 | 0.98152456 | 4 | amp |
| TCGA-09-0366 | 9 | 17322 | 32500989 | 0.756025684 | 3 | amp |
| TCGA-09-0366 | 9 | 32526021 | 33364116 | 1.048097873 | 4 | amp |
| TCGA-09-0366 | 9 | 33364696 | 33935916 | 1.382682828 | 5 | amp |
| TCGA-09-0366 | 9 | 33941633 | 34242124 | 0.83460533 | 4 | amp |
| TCGA-09-0366 | 9 | 34249747 | 34397683 | 1.347753176 | 5 | amp |
| TCGA-09-0366 | 9 | 34401630 | 35103128 | 1.943346679 | 6 | amp |
| TCGA-09-0366 | 9 | 35105144 | 35608508 | 1.22499878 | 5 | amp |
| TCGA-09-0366 | 9 | 35608818 | 35853849 | 1.967182423 | 6 | amp |
| TCGA-09-0366 | 9 | 35869448 | 43129920 | 1.051500839 | 4 | amp |
| TCGA-09-0366 | 9 | 43164462 | 66962667 | 1.34237712 | 5 | amp |
| TCGA-09-0366 | 9 | 67026619 | 67281892 | 2.146873529 | 6 | amp |
| TCGA-09-0366 | 9 | 67293644 | 72435982 | 0.996822832 | 4 | amp |
| TCGA-09-0366 | 9 | 72459426 | 88642829 | 0.774933799 | 3 | amp |
| TCGA-09-0366 | 9 | 88648193 | 90273052 | 0.995849138 | 4 | amp |
| TCGA-09-0366 | 9 | 90283490 | 93636589 | 1.278521305 | 5 | amp |
| TCGA-09-0366 | 9 | 93636892 | 95285182 | 0.870763001 | 4 | amp |
| TCGA-09-0366 | 9 | 95298155 | 98238498 | 1.407298933 | 5 | amp |
| TCGA-09-0366 | 9 | 98239029 | 100761018 | 1.030068302 | 4 | amp |
| TCGA-09-0366 | 9 | 100767190 | 101807094 | 1.378814175 | 5 | amp |
| TCGA-09-0366 | 9 | 101810004 | 102609927 | 0.963613464 | 4 | amp |

| | | | | | | |
|---|---|---|---|---|---|---|
| TCGA-09-0366 | 9 | 102625845 | 115626722 | 0.79211969 | 3 | amp |
| TCGA-09-0366 | 9 | 115631033 | 116049166 | 0.990396127 | 4 | amp |
| TCGA-09-0366 | 9 | 116050409 | 117386744 | 1.762935187 | 5 | amp |
| TCGA-09-0366 | 9 | 117389142 | 123595781 | 0.934567636 | 4 | amp |
| TCGA-09-0366 | 9 | 123620212 | 123688403 | 1.539194863 | 5 | amp |
| TCGA-09-0366 | 9 | 123714931 | 123949323 | 0.734239148 | 4 | amp |
| TCGA-09-0366 | 9 | 123952800 | 125747033 | 1.304206813 | 5 | amp |
| TCGA-09-0366 | 9 | 125748475 | 126641324 | 0.943044217 | 4 | amp |
| TCGA-09-0366 | 9 | 126776220 | 130155567 | 1.230049253 | 5 | amp |
| TCGA-09-0366 | 9 | 130168249 | 130341277 | 1.978049976 | 6 | amp |
| TCGA-09-0366 | 9 | 130413846 | 130432266 | 0.955083571 | 4 | amp |
| TCGA-09-0366 | 9 | 130434302 | 131190760 | 2.167369603 | 6 | amp |
| TCGA-09-0366 | 9 | 131190983 | 131337143 | 1.304571141 | 5 | amp |
| TCGA-09-0366 | 9 | 131337400 | 131369992 | 0.79216577 | 4 | amp |
| TCGA-09-0366 | 9 | 131370109 | 131756776 | 1.589719677 | 5 | amp |
| TCGA-09-0366 | 9 | 131757108 | 132620393 | 2.0128498 | 6 | amp |
| TCGA-09-0366 | 9 | 132620691 | 134003128 | 1.537712814 | 5 | amp |
| TCGA-09-0366 | 9 | 134003674 | 134090796 | 0.749775946 | 4 | amp |
| TCGA-09-0366 | 9 | 134098120 | 134362737 | 1.42376306 | 5 | amp |
| TCGA-09-0366 | 9 | 134363182 | 134471782 | 2.115627201 | 6 | amp |
| TCGA-09-0366 | 9 | 134473581 | 135862175 | 1.030504699 | 4 | amp |
| TCGA-09-0366 | 9 | 135862617 | 141071671 | 1.897255668 | 6 | amp |
| TCGA-09-0366 | X | 200797 | 2876515 | 1.513166063 | 5 | amp |
| TCGA-09-0366 | X | 2878383 | 9000561 | 0.982165891 | 4 | amp |
| TCGA-09-0366 | X | 9000953 | 10107636 | 1.230973045 | 5 | amp |
| TCGA-09-0366 | X | 10109447 | 13765105 | 0.900595856 | 4 | amp |
| TCGA-09-0366 | X | 13767538 | 15605988 | 0.733417806 | 3 | amp |
| TCGA-09-0366 | X | 15607462 | 16847915 | 0.917386584 | 4 | amp |
| TCGA-09-0366 | X | 16850655 | 18631453 | 0.666100951 | 3 | amp |
| TCGA-09-0366 | X | 18637976 | 18845619 | 0.974949301 | 4 | amp |
| TCGA-09-0366 | X | 18911567 | 18949872 | 1.608564145 | 5 | amp |
| TCGA-09-0366 | X | 18954160 | 19626169 | 0.962899521 | 4 | amp |
| TCGA-09-0366 | X | 19649938 | 23020082 | 0.685917092 | 3 | amp |
| TCGA-09-0366 | X | 23352987 | 23953547 | 0.975818009 | 4 | amp |
| TCGA-09-0366 | X | 23956620 | 30719048 | 0.781860544 | 3 | amp |
| TCGA-09-0366 | X | 45010886 | 46918503 | 0.655938419 | 3 | amp |
| TCGA-09-0366 | X | 46940504 | 47039478 | 0.945437232 | 4 | amp |
| TCGA-09-0366 | X | 47039801 | 47500855 | 1.357284919 | 5 | amp |
| TCGA-09-0366 | X | 47509809 | 48047176 | 1.013913332 | 4 | amp |
| TCGA-09-0366 | X | 48049516 | 48270840 | 0.705152418 | 3 | amp |
| TCGA-09-0366 | X | 48300718 | 48462838 | 1.420730626 | 5 | amp |
| TCGA-09-0366 | X | 48542188 | 48547295 | 2.488124938 | 6 | amp |
| TCGA-09-0366 | X | 48547392 | 49143583 | 1.349260544 | 5 | amp |
| TCGA-09-0366 | X | 49161318 | 49364879 | 1.011016547 | 4 | amp |
| TCGA-09-0366 | X | 49365266 | 50134624 | 0.646158458 | 3 | amp |
| TCGA-09-0366 | X | 50135332 | 53222838 | 0.908576312 | 4 | amp |
| TCGA-09-0366 | X | 53222933 | 53407655 | 1.220864273 | 5 | amp |
| TCGA-09-0366 | X | 53407913 | 53575270 | 0.934634293 | 4 | amp |
| TCGA-09-0366 | X | 53575892 | 54360137 | 0.714029067 | 3 | amp |
| TCGA-09-0366 | X | 54469818 | 55046964 | 1.016221712 | 4 | amp |

| | | | | | | |
|---|---|---|---|---|---|---|
| TCGA-09-0366 | X | 55047475 | 67518950 | 0.707160557 | 3 | amp |
| TCGA-09-0366 | X | 67652665 | 67885417 | 0.908370522 | 4 | amp |
| TCGA-09-0366 | X | 67932731 | 68749817 | 1.318796544 | 5 | amp |
| TCGA-09-0366 | X | 68836110 | 69490031 | 0.949404797 | 4 | amp |
| TCGA-09-0366 | X | 69495911 | 69510488 | 1.450919064 | 5 | amp |
| TCGA-09-0366 | X | 69510537 | 70341718 | 0.957892846 | 4 | amp |
| TCGA-09-0366 | X | 70342002 | 70461255 | 1.284818609 | 5 | amp |
| TCGA-09-0366 | X | 70461966 | 70588081 | 0.945804933 | 4 | amp |
| TCGA-09-0366 | X | 70594956 | 73811703 | 0.775556229 | 3 | amp |
| TCGA-09-0366 | X | 96684636 | 100809314 | 0.742238269 | 3 | amp |
| TCGA-09-0366 | X | 100852501 | 102333583 | 0.976722991 | 4 | amp |
| TCGA-09-0366 | X | 102334109 | 102338638 | 1.629855742 | 5 | amp |
| TCGA-09-0366 | X | 102339226 | 102471502 | 0.966038682 | 4 | amp |
| TCGA-09-0366 | X | 102508595 | 105199604 | 0.738784929 | 3 | amp |
| TCGA-09-0366 | X | 106358533 | 107435816 | 0.749032307 | 3 | amp |
| TCGA-09-0366 | X | 117900465 | 118986933 | 0.754374071 | 3 | amp |
| TCGA-09-0366 | X | 119004490 | 119500653 | 1.012969622 | 4 | amp |
| TCGA-09-0366 | X | 119504483 | 119675635 | 0.591011738 | 3 | amp |
| TCGA-09-0366 | X | 119676794 | 120337025 | 0.978594755 | 4 | amp |
| TCGA-09-0366 | X | 122251517 | 122319882 | 0.744931765 | 3 | amp |
| TCGA-09-0366 | X | 128641875 | 128724304 | 0.697589458 | 3 | amp |
| TCGA-09-0366 | X | 128873123 | 129215326 | 0.954713043 | 4 | amp |
| TCGA-09-0366 | X | 129263477 | 135591354 | 0.694813389 | 3 | amp |
| TCGA-09-0366 | X | 148044235 | 150844661 | 0.721518098 | 3 | amp |
| TCGA-09-0366 | X | 150867199 | 151562983 | 0.980625008 | 4 | amp |
| TCGA-09-0366 | X | 151806580 | 153780430 | 1.27860952 | 5 | amp |
| TCGA-09-0366 | X | 153789890 | 154020142 | 0.856906028 | 4 | amp |
| TCGA-09-0366 | X | 154020338 | 155252520 | 0.688592596 | 3 | amp |
| TCGA-09-0369 | 1 | 16834 | 745558 | 1.476461356 | 4 | amp |
| TCGA-09-0369 | 1 | 809477 | 1634451 | 4.147905485 | 5 | amp |
| TCGA-09-0369 | 1 | 1634868 | 1639055 | 1.985674261 | 4 | amp |
| TCGA-09-0369 | 1 | 1639591 | 2519920 | 3.511626465 | 5 | amp |
| TCGA-09-0369 | 1 | 2519936 | 2526816 | 1.779365756 | 4 | amp |
| TCGA-09-0369 | 1 | 2527436 | 5965887 | 3.38344892 | 5 | amp |
| TCGA-09-0369 | 1 | 5967168 | 6100726 | 2.048094914 | 4 | amp |
| TCGA-09-0369 | 1 | 6101851 | 6727879 | 3.995172647 | 5 | amp |
| TCGA-09-0369 | 1 | 6738400 | 6885330 | 1.551550961 | 4 | amp |
| TCGA-09-0369 | 1 | 7151337 | 7798580 | 2.834336968 | 5 | amp |
| TCGA-09-0369 | 1 | 7804880 | 7815772 | 1.9315982 | 4 | amp |
| TCGA-09-0369 | 1 | 7826474 | 7854103 | 2.63895447 | 5 | amp |
| TCGA-09-0369 | 1 | 7858508 | 7998894 | 1.697443803 | 4 | amp |
| TCGA-09-0369 | 1 | 7999944 | 8418256 | 2.988542678 | 5 | amp |
| TCGA-09-0369 | 1 | 8419890 | 8716467 | 1.902027119 | 4 | amp |
| TCGA-09-0369 | 1 | 8921363 | 10075924 | 3.015713379 | 5 | amp |
| TCGA-09-0369 | 1 | 10093680 | 10423431 | 1.536562432 | 4 | amp |
| TCGA-09-0369 | 1 | 10425110 | 11204818 | 3.190387286 | 5 | amp |
| TCGA-09-0369 | 1 | 11205003 | 11298686 | 1.722699232 | 4 | amp |
| TCGA-09-0369 | 1 | 11300271 | 12267116 | 3.517381069 | 5 | amp |
| TCGA-09-0369 | 1 | 12294311 | 12942265 | 1.744815474 | 4 | amp |
| TCGA-09-0369 | 1 | 12942858 | 12980252 | 2.71633273 | 5 | amp |

| | | | | | | |
|---|---|---|---|---|---|---|
| TCGA-09-0369 | 1 | 12987861 | 13352274 | 2.027488123 | 4 | amp |
| TCGA-09-0369 | 1 | 13353863 | 13427878 | 2.754856121 | 5 | amp |
| TCGA-09-0369 | 1 | 13448028 | 13477558 | 1.783383674 | 4 | amp |
| TCGA-09-0369 | 1 | 13495246 | 13669484 | 2.768672499 | 5 | amp |
| TCGA-09-0369 | 1 | 13670340 | 14149726 | 2.000669568 | 4 | amp |
| TCGA-09-0369 | 1 | 15361192 | 15855803 | 3.1177828 | 5 | amp |
| TCGA-09-0369 | 1 | 15860704 | 15890875 | 2.149085092 | 4 | amp |
| TCGA-09-0369 | 1 | 15892393 | 15978415 | 6.215560391 | 5 | amp |
| TCGA-09-0369 | 1 | 15983059 | 15988250 | 1.493007816 | 4 | amp |
| TCGA-09-0369 | 1 | 16042724 | 16133969 | 5.208015741 | 5 | amp |
| TCGA-09-0369 | 1 | 16199290 | 16262457 | 1.867346223 | 4 | amp |
| TCGA-09-0369 | 1 | 16262493 | 16528376 | 3.942266818 | 5 | amp |
| TCGA-09-0369 | 1 | 16528869 | 16578960 | 2.092416472 | 4 | amp |
| TCGA-09-0369 | 1 | 16579558 | 16893879 | 2.784938849 | 5 | amp |
| TCGA-09-0369 | 1 | 16894439 | 16918548 | 1.548638841 | 4 | amp |
| TCGA-09-0369 | 1 | 16945531 | 16973873 | 3.476921887 | 5 | amp |
| TCGA-09-0369 | 1 | 16973897 | 16974935 | 1.404855679 | 4 | amp |
| TCGA-09-0369 | 1 | 16975091 | 17023346 | 3.017058788 | 5 | amp |
| TCGA-09-0369 | 1 | 17029181 | 17087643 | 1.88111801 | 4 | amp |
| TCGA-09-0369 | 1 | 17249075 | 17355282 | 4.019422449 | 5 | amp |
| TCGA-09-0369 | 1 | 17359537 | 17396766 | 1.970300919 | 4 | amp |
| TCGA-09-0369 | 1 | 17397883 | 17720992 | 3.309705617 | 5 | amp |
| TCGA-09-0369 | 1 | 17721404 | 17725382 | 1.59826249 | 4 | amp |
| TCGA-09-0369 | 1 | 17727697 | 19414426 | 3.138841741 | 5 | amp |
| TCGA-09-0369 | 1 | 19415241 | 19528320 | 1.670265604 | 4 | amp |
| TCGA-09-0369 | 1 | 19545736 | 19712278 | 3.855368014 | 5 | amp |
| TCGA-09-0369 | 1 | 19746139 | 19952947 | 1.679137959 | 4 | amp |
| TCGA-09-0369 | 1 | 19969788 | 20063966 | 2.701554679 | 5 | amp |
| TCGA-09-0369 | 1 | 20066234 | 20466744 | 2.052688648 | 4 | amp |
| TCGA-09-0369 | 1 | 20469860 | 21083763 | 2.917546394 | 5 | amp |
| TCGA-09-0369 | 1 | 21091826 | 21097614 | 1.642487557 | 4 | amp |
| TCGA-09-0369 | 1 | 21099903 | 21144051 | 2.88004952 | 5 | amp |
| TCGA-09-0369 | 1 | 21151581 | 21329280 | 1.39594306 | 4 | amp |
| TCGA-09-0369 | 1 | 21546415 | 21771765 | 3.601653213 | 5 | amp |
| TCGA-09-0369 | 1 | 21795135 | 21806749 | 1.785675509 | 4 | amp |
| TCGA-09-0369 | 1 | 21807388 | 22016649 | 3.991793335 | 5 | amp |
| TCGA-09-0369 | 1 | 22021516 | 22084321 | 1.26038452 | 4 | amp |
| TCGA-09-0369 | 1 | 22138896 | 22339041 | 3.696861416 | 5 | amp |
| TCGA-09-0369 | 1 | 22404959 | 22987891 | 1.989804038 | 4 | amp |
| TCGA-09-0369 | 1 | 23101655 | 23233506 | 2.740143386 | 5 | amp |
| TCGA-09-0369 | 1 | 23234433 | 23664360 | 1.601182371 | 4 | amp |
| TCGA-09-0369 | 1 | 23664921 | 23698070 | 2.624517228 | 5 | amp |
| TCGA-09-0369 | 1 | 23707902 | 23710934 | 1.458307966 | 4 | amp |
| TCGA-09-0369 | 1 | 23713718 | 24124723 | 3.320877626 | 5 | amp |
| TCGA-09-0369 | 1 | 24125072 | 24131072 | 1.464631693 | 4 | amp |
| TCGA-09-0369 | 1 | 24133464 | 24192167 | 4.316711342 | 5 | amp |
| TCGA-09-0369 | 1 | 24200935 | 24301575 | 2.030487158 | 4 | amp |
| TCGA-09-0369 | 1 | 24305194 | 24766755 | 3.106523907 | 5 | amp |
| TCGA-09-0369 | 1 | 24768480 | 27686915 | 1.672054026 | 4 | amp |
| TCGA-09-0369 | 1 | 27687974 | 27706730 | 2.693373069 | 5 | amp |

| | | | | | | |
|---|---|---|---|---|---|---|
| TCGA-09-0369 | 1 | 27708220 | 28301076 | 1.529271022 | 4 | amp |
| TCGA-09-0369 | 1 | 28304874 | 28823113 | 0.953214199 | 3 | amp |
| TCGA-09-0369 | 1 | 28833829 | 31351648 | 1.489307389 | 4 | amp |
| TCGA-09-0369 | 1 | 31406003 | 31845884 | 1.009084723 | 3 | amp |
| TCGA-09-0369 | 1 | 31896500 | 32131261 | 3.521665235 | 5 | amp |
| TCGA-09-0369 | 1 | 32131449 | 32150499 | 1.946645434 | 4 | amp |
| TCGA-09-0369 | 1 | 32151155 | 32162746 | 2.529223641 | 5 | amp |
| TCGA-09-0369 | 1 | 32162800 | 32688103 | 1.651331874 | 4 | amp |
| TCGA-09-0369 | 1 | 32688124 | 32745865 | 2.418935866 | 5 | amp |
| TCGA-09-0369 | 1 | 32751065 | 34291396 | 1.466060166 | 4 | amp |
| TCGA-09-0369 | 1 | 34312450 | 35972774 | 0.948782876 | 3 | amp |
| TCGA-09-0369 | 1 | 36019901 | 36024897 | 1.40146648 | 4 | amp |
| TCGA-09-0369 | 1 | 36025840 | 36055709 | 2.479060705 | 5 | amp |
| TCGA-09-0369 | 1 | 36056155 | 36349094 | 0.987569712 | 3 | amp |
| TCGA-09-0369 | 1 | 36353999 | 39715790 | 1.541457593 | 4 | amp |
| TCGA-09-0369 | 1 | 39719948 | 39930835 | 0.798288329 | 3 | amp |
| TCGA-09-0369 | 1 | 39934225 | 40525134 | 1.360521736 | 4 | amp |
| TCGA-09-0369 | 1 | 40525700 | 40747226 | 0.872488566 | 3 | amp |
| TCGA-09-0369 | 1 | 40751588 | 41449165 | 1.436199035 | 4 | amp |
| TCGA-09-0369 | 1 | 41450457 | 42896587 | 1.038387951 | 3 | amp |
| TCGA-09-0369 | 1 | 42898766 | 42923029 | 1.801242503 | 4 | amp |
| TCGA-09-0369 | 1 | 42925255 | 43204293 | 0.925054209 | 3 | amp |
| TCGA-09-0369 | 1 | 43212502 | 43826637 | 1.422879838 | 4 | amp |
| TCGA-09-0369 | 1 | 43826699 | 43831071 | 3.422232149 | 5 | amp |
| TCGA-09-0369 | 1 | 43831167 | 43880938 | 1.554635803 | 4 | amp |
| TCGA-09-0369 | 1 | 43881581 | 43893791 | 0.852008254 | 3 | amp |
| TCGA-09-0369 | 1 | 43893823 | 44426945 | 1.586464502 | 4 | amp |
| TCGA-09-0369 | 1 | 44429906 | 44437760 | 3.033204456 | 5 | amp |
| TCGA-09-0369 | 1 | 44437797 | 46501753 | 1.51097093 | 4 | amp |
| TCGA-09-0369 | 1 | 46509323 | 46654701 | 0.839100405 | 3 | amp |
| TCGA-09-0369 | 1 | 46654895 | 47310305 | 1.475652396 | 4 | amp |
| TCGA-09-0369 | 1 | 47311308 | 52819374 | 0.920668295 | 3 | amp |
| TCGA-09-0369 | 1 | 52820234 | 52849286 | 2.186498863 | 5 | amp |
| TCGA-09-0369 | 1 | 52849495 | 53504773 | 0.927494517 | 3 | amp |
| TCGA-09-0369 | 1 | 53513509 | 53720882 | 2.198727422 | 5 | amp |
| TCGA-09-0369 | 1 | 78272602 | 78399185 | 1.480997921 | 4 | amp |
| TCGA-09-0369 | 1 | 78401498 | 84815505 | 0.889388591 | 3 | amp |
| TCGA-09-0369 | 1 | 84864255 | 84991761 | 1.508700364 | 4 | amp |
| TCGA-09-0369 | 1 | 84999360 | 94033061 | 1.06797718 | 3 | amp |
| TCGA-09-0369 | 1 | 94033292 | 94586628 | 1.393368854 | 4 | amp |
| TCGA-09-0369 | 1 | 94639384 | 109566175 | 0.790801239 | 3 | amp |
| TCGA-09-0369 | 1 | 109607109 | 109836925 | 1.697339372 | 4 | amp |
| TCGA-09-0369 | 1 | 109837705 | 109958041 | 0.896830032 | 3 | amp |
| TCGA-09-0369 | 1 | 109964428 | 110028771 | 1.601314546 | 4 | amp |
| TCGA-09-0369 | 1 | 110029089 | 110034361 | 2.7904881 | 5 | amp |
| TCGA-09-0369 | 1 | 110035124 | 110087042 | 1.372144911 | 4 | amp |
| TCGA-09-0369 | 1 | 110116319 | 110172597 | 0.951716705 | 3 | amp |
| TCGA-09-0369 | 1 | 110172791 | 110889012 | 1.749374076 | 4 | amp |
| TCGA-09-0369 | 1 | 110906301 | 111731914 | 0.858284619 | 3 | amp |
| TCGA-09-0369 | 1 | 111734767 | 111784126 | 1.695485683 | 4 | amp |

| | | | | | | |
|---|---|---|---|---|---|---|
| TCGA-09-0369 | 1 | 111784767 | 113201743 | 1.013250629 | 3 | amp |
| TCGA-09-0369 | 1 | 113202301 | 113471941 | 1.782167888 | 4 | amp |
| TCGA-09-0369 | 1 | 113615967 | 115168622 | 0.920029222 | 3 | amp |
| TCGA-09-0369 | 1 | 115215693 | 115400151 | 1.456565234 | 4 | amp |
| TCGA-09-0369 | 1 | 115401129 | 115604864 | 0.925051366 | 3 | amp |
| TCGA-09-0369 | 1 | 115615486 | 117635557 | 1.358513042 | 4 | amp |
| TCGA-09-0369 | 1 | 117637899 | 118694041 | 1.009520761 | 3 | amp |
| TCGA-09-0369 | 1 | 118727646 | 121124793 | 1.388990281 | 4 | amp |
| TCGA-09-0369 | 1 | 121128357 | 143897654 | 0.837051547 | 3 | amp |
| TCGA-09-0369 | 1 | 143905954 | 145441260 | 1.511190687 | 4 | amp |
| TCGA-09-0369 | 1 | 145456589 | 145458028 | 2.966665369 | 5 | amp |
| TCGA-09-0369 | 1 | 145459566 | 145528359 | 1.972893308 | 4 | amp |
| TCGA-09-0369 | 1 | 145528566 | 145541548 | 2.416051042 | 5 | amp |
| TCGA-09-0369 | 1 | 145541798 | 145542305 | 1.501323531 | 4 | amp |
| TCGA-09-0369 | 1 | 145549275 | 145560286 | 3.11835774 | 5 | amp |
| TCGA-09-0369 | 1 | 145560847 | 145579034 | 1.762026005 | 4 | amp |
| TCGA-09-0369 | 1 | 145579226 | 145611387 | 2.56939977 | 5 | amp |
| TCGA-09-0369 | 1 | 145646083 | 149876777 | 1.509876009 | 4 | amp |
| TCGA-09-0369 | 1 | 149877361 | 149901841 | 2.525230383 | 5 | amp |
| TCGA-09-0369 | 1 | 149902227 | 150204309 | 1.738221447 | 4 | amp |
| TCGA-09-0369 | 1 | 150207964 | 150245303 | 3.171335443 | 5 | amp |
| TCGA-09-0369 | 1 | 150246441 | 150445863 | 1.910533605 | 4 | amp |
| TCGA-09-0369 | 1 | 150459869 | 150471561 | 3.220083455 | 5 | amp |
| TCGA-09-0369 | 1 | 150471640 | 150485304 | 2.077431282 | 4 | amp |
| TCGA-09-0369 | 1 | 150485707 | 150529530 | 3.526091646 | 5 | amp |
| TCGA-09-0369 | 1 | 150529568 | 150931858 | 1.521136961 | 4 | amp |
| TCGA-09-0369 | 1 | 150932962 | 150990390 | 3.178521401 | 5 | amp |
| TCGA-09-0369 | 1 | 150990777 | 151001460 | 1.927374681 | 4 | amp |
| TCGA-09-0369 | 1 | 151006255 | 151060779 | 2.851331043 | 5 | amp |
| TCGA-09-0369 | 1 | 151062869 | 151076154 | 1.910167402 | 4 | amp |
| TCGA-09-0369 | 1 | 151079485 | 151131750 | 3.100371683 | 5 | amp |
| TCGA-09-0369 | 1 | 151133306 | 151140836 | 1.917269935 | 4 | amp |
| TCGA-09-0369 | 1 | 151141451 | 151147064 | 3.21042735 | 5 | amp |
| TCGA-09-0369 | 1 | 151147139 | 151265538 | 1.988979914 | 4 | amp |
| TCGA-09-0369 | 1 | 151266433 | 151298711 | 2.554294628 | 5 | amp |
| TCGA-09-0369 | 1 | 151314617 | 151318798 | 1.87452282 | 4 | amp |
| TCGA-09-0369 | 1 | 151336949 | 151345175 | 2.546666057 | 5 | amp |
| TCGA-09-0369 | 1 | 151371983 | 151414738 | 1.421081703 | 4 | amp |
| TCGA-09-0369 | 1 | 151490951 | 151508177 | 2.422318392 | 5 | amp |
| TCGA-09-0369 | 1 | 151508194 | 151665105 | 1.840478363 | 4 | amp |
| TCGA-09-0369 | 1 | 151665330 | 151682332 | 2.575423306 | 5 | amp |
| TCGA-09-0369 | 1 | 151688273 | 151739412 | 1.769674354 | 4 | amp |
| TCGA-09-0369 | 1 | 151739586 | 151826099 | 2.621161005 | 5 | amp |
| TCGA-09-0369 | 1 | 151847293 | 153431538 | 1.690497942 | 4 | amp |
| TCGA-09-0369 | 1 | 153507088 | 153617762 | 2.914714243 | 5 | amp |
| TCGA-09-0369 | 1 | 153631546 | 153635611 | 2.053197962 | 4 | amp |
| TCGA-09-0369 | 1 | 153635631 | 153665918 | 3.388469031 | 5 | amp |
| TCGA-09-0369 | 1 | 153701037 | 153740346 | 1.980765621 | 4 | amp |
| TCGA-09-0369 | 1 | 153741312 | 153782839 | 2.859566058 | 5 | amp |
| TCGA-09-0369 | 1 | 153784115 | 153800835 | 1.768098581 | 4 | amp |

| | | | | | | |
|---|---|---|---|---|---|---|
| TCGA-09-0369 | 1 | 153902725 | 153958770 | 3.081992082 | 5 | amp |
| TCGA-09-0369 | 1 | 153963215 | 154241436 | 1.579091282 | 4 | amp |
| TCGA-09-0369 | 1 | 154242633 | 154318029 | 2.875719222 | 5 | amp |
| TCGA-09-0369 | 1 | 154318257 | 154420683 | 2.006177685 | 4 | amp |
| TCGA-09-0369 | 1 | 154422361 | 154480987 | 3.09337232 | 5 | amp |
| TCGA-09-0369 | 1 | 154492754 | 154527295 | 1.875649806 | 4 | amp |
| TCGA-09-0369 | 1 | 154527865 | 155282235 | 3.28962982 | 5 | amp |
| TCGA-09-0369 | 1 | 155287711 | 155295737 | 1.912524631 | 4 | amp |
| TCGA-09-0369 | 1 | 155296316 | 155308210 | 2.764708457 | 5 | amp |
| TCGA-09-0369 | 1 | 155309048 | 155580924 | 1.985795525 | 4 | amp |
| TCGA-09-0369 | 1 | 155580984 | 155618262 | 2.908979567 | 5 | amp |
| TCGA-09-0369 | 1 | 155629388 | 155642556 | 1.779884194 | 4 | amp |
| TCGA-09-0369 | 1 | 155644753 | 155691418 | 2.590255046 | 5 | amp |
| TCGA-09-0369 | 1 | 155695166 | 155715983 | 2.138731364 | 4 | amp |
| TCGA-09-0369 | 1 | 155716384 | 155730453 | 2.494603504 | 5 | amp |
| TCGA-09-0369 | 1 | 155731952 | 155921081 | 2.098562305 | 4 | amp |
| TCGA-09-0369 | 1 | 155921238 | 156128622 | 4.150582746 | 5 | amp |
| TCGA-09-0369 | 1 | 156130171 | 156132929 | 1.540438916 | 4 | amp |
| TCGA-09-0369 | 1 | 156142586 | 156921547 | 3.444391829 | 5 | amp |
| TCGA-09-0369 | 1 | 156924638 | 157014166 | 2.003943336 | 4 | amp |
| TCGA-09-0369 | 1 | 157030398 | 157491124 | 2.791010479 | 5 | amp |
| TCGA-09-0369 | 1 | 157493997 | 157801815 | 1.608357822 | 4 | amp |
| TCGA-09-0369 | 1 | 157802931 | 158150981 | 3.336374572 | 5 | amp |
| TCGA-09-0369 | 1 | 158151167 | 158299960 | 1.931201277 | 4 | amp |
| TCGA-09-0369 | 1 | 158300538 | 158326721 | 3.053059835 | 5 | amp |
| TCGA-09-0369 | 1 | 158368280 | 158617539 | 1.369931502 | 4 | amp |
| TCGA-09-0369 | 1 | 158618220 | 158626459 | 2.768646818 | 5 | amp |
| TCGA-09-0369 | 1 | 158627225 | 159043338 | 1.654925361 | 4 | amp |
| TCGA-09-0369 | 1 | 159159551 | 159176285 | 2.568005972 | 5 | amp |
| TCGA-09-0369 | 1 | 159272091 | 159785508 | 1.657767643 | 4 | amp |
| TCGA-09-0369 | 1 | 159796618 | 159832346 | 3.430672572 | 5 | amp |
| TCGA-09-0369 | 1 | 159842223 | 159857835 | 1.950313633 | 4 | amp |
| TCGA-09-0369 | 1 | 159858093 | 160123044 | 2.953431432 | 5 | amp |
| TCGA-09-0369 | 1 | 160124786 | 160136872 | 1.850955403 | 4 | amp |
| TCGA-09-0369 | 1 | 160136984 | 160188818 | 3.071501437 | 5 | amp |
| TCGA-09-0369 | 1 | 160192355 | 160314623 | 1.879707878 | 4 | amp |
| TCGA-09-0369 | 1 | 160318730 | 160394077 | 2.674505599 | 5 | amp |
| TCGA-09-0369 | 1 | 160394857 | 160769887 | 1.615825678 | 4 | amp |
| TCGA-09-0369 | 1 | 160771572 | 160788118 | 2.586690404 | 5 | amp |
| TCGA-09-0369 | 1 | 160789047 | 160851142 | 1.776821669 | 4 | amp |
| TCGA-09-0369 | 1 | 160851660 | 161293483 | 3.175118009 | 5 | amp |
| TCGA-09-0369 | 1 | 161298146 | 161479916 | 1.913920496 | 4 | amp |
| TCGA-09-0369 | 1 | 161480564 | 161719943 | 2.496669462 | 5 | amp |
| TCGA-09-0369 | 1 | 161721424 | 161928459 | 1.61967859 | 4 | amp |
| TCGA-09-0369 | 1 | 161953418 | 162124290 | 2.611705017 | 5 | amp |
| TCGA-09-0369 | 1 | 162257119 | 162313815 | 1.964439136 | 4 | amp |
| TCGA-09-0369 | 1 | 162324939 | 162352029 | 3.318235633 | 5 | amp |
| TCGA-09-0369 | 1 | 162352922 | 169096673 | 1.60417262 | 4 | amp |
| TCGA-09-0369 | 1 | 169099227 | 171605876 | 1.071972114 | 3 | amp |
| TCGA-09-0369 | 1 | 171607649 | 173916733 | 1.416958904 | 4 | amp |

| | | | | | | |
|---|---|---|---|---|---|---|
| TCGA-09-0369 | 1 | 173921079 | 174606629 | 0.940062718 | 3 | amp |
| TCGA-09-0369 | 1 | 174652636 | 175049610 | 1.459983033 | 4 | amp |
| TCGA-09-0369 | 1 | 175052858 | 175067779 | 2.581480544 | 5 | amp |
| TCGA-09-0369 | 1 | 175086027 | 175914357 | 1.469224066 | 4 | amp |
| TCGA-09-0369 | 1 | 175916292 | 176918523 | 1.085356818 | 3 | amp |
| TCGA-09-0369 | 1 | 176926768 | 180151501 | 1.375141064 | 4 | amp |
| TCGA-09-0369 | 1 | 180152967 | 180163557 | 2.935615536 | 5 | amp |
| TCGA-09-0369 | 1 | 180165334 | 180853231 | 1.346509636 | 4 | amp |
| TCGA-09-0369 | 1 | 180885252 | 180934852 | 2.669929195 | 5 | amp |
| TCGA-09-0369 | 1 | 180945653 | 182829343 | 1.748218942 | 4 | amp |
| TCGA-09-0369 | 1 | 182835529 | 182898894 | 0.830685363 | 3 | amp |
| TCGA-09-0369 | 1 | 182908299 | 183387419 | 1.352016077 | 4 | amp |
| TCGA-09-0369 | 1 | 183481927 | 183520372 | 0.822915648 | 3 | amp |
| TCGA-09-0369 | 1 | 183520949 | 183942936 | 1.400469037 | 4 | amp |
| TCGA-09-0369 | 1 | 183944169 | 186324953 | 1.018093169 | 3 | amp |
| TCGA-09-0369 | 1 | 186325379 | 186360935 | 1.614397343 | 4 | amp |
| TCGA-09-0369 | 1 | 186367388 | 197641346 | 0.98850154 | 3 | amp |
| TCGA-09-0369 | 1 | 197643214 | 197898431 | 1.76192077 | 4 | amp |
| TCGA-09-0369 | 1 | 198201678 | 200573144 | 0.976256583 | 3 | amp |
| TCGA-09-0369 | 1 | 200574374 | 200869409 | 1.341186322 | 4 | amp |
| TCGA-09-0369 | 1 | 200876928 | 200978624 | 3.090835401 | 5 | amp |
| TCGA-09-0369 | 1 | 201008904 | 201021790 | 1.853581042 | 4 | amp |
| TCGA-09-0369 | 1 | 201022279 | 201687940 | 2.692067487 | 5 | amp |
| TCGA-09-0369 | 1 | 201749467 | 201842142 | 1.627781845 | 4 | amp |
| TCGA-09-0369 | 1 | 201843318 | 201915517 | 2.650459092 | 5 | amp |
| TCGA-09-0369 | 1 | 201924577 | 201958221 | 2.085328846 | 4 | amp |
| TCGA-09-0369 | 1 | 201958434 | 202274904 | 3.050489071 | 5 | amp |
| TCGA-09-0369 | 1 | 202275941 | 202914323 | 1.640966126 | 4 | amp |
| TCGA-09-0369 | 1 | 202915532 | 203155798 | 2.590024673 | 5 | amp |
| TCGA-09-0369 | 1 | 203174918 | 203276578 | 1.556909105 | 4 | amp |
| TCGA-09-0369 | 1 | 203311396 | 203652549 | 3.578133725 | 5 | amp |
| TCGA-09-0369 | 1 | 203667263 | 203803009 | 1.61250025 | 4 | amp |
| TCGA-09-0369 | 1 | 203807052 | 203819176 | 2.748207906 | 5 | amp |
| TCGA-09-0369 | 1 | 203819575 | 204009457 | 1.808176214 | 4 | amp |
| TCGA-09-0369 | 1 | 204010180 | 204227038 | 2.594589027 | 5 | amp |
| TCGA-09-0369 | 1 | 204228326 | 204917842 | 1.805602833 | 4 | amp |
| TCGA-09-0369 | 1 | 204919633 | 205035096 | 2.640776514 | 5 | amp |
| TCGA-09-0369 | 1 | 205035527 | 205156940 | 1.498952435 | 4 | amp |
| TCGA-09-0369 | 1 | 205238034 | 205312784 | 3.25236739 | 5 | amp |
| TCGA-09-0369 | 1 | 205350773 | 205389683 | 1.279785504 | 4 | amp |
| TCGA-09-0369 | 1 | 205417387 | 205553286 | 2.800892133 | 5 | amp |
| TCGA-09-0369 | 1 | 205553971 | 205569621 | 2.026098437 | 4 | amp |
| TCGA-09-0369 | 1 | 205585662 | 205633818 | 2.501623089 | 5 | amp |
| TCGA-09-0369 | 1 | 205687357 | 205741719 | 1.537195461 | 4 | amp |
| TCGA-09-0369 | 1 | 205743992 | 205797929 | 2.623130798 | 5 | amp |
| TCGA-09-0369 | 1 | 205799397 | 205884568 | 1.857161437 | 4 | amp |
| TCGA-09-0369 | 1 | 205886356 | 205905005 | 2.409097801 | 5 | amp |
| TCGA-09-0369 | 1 | 206139260 | 206628418 | 1.62314216 | 4 | amp |
| TCGA-09-0369 | 1 | 206631950 | 206758047 | 2.756636166 | 5 | amp |
| TCGA-09-0369 | 1 | 206758479 | 207071214 | 1.939883062 | 4 | amp |

| | | | | | | |
|---|---|---|---|---|---|---|
| TCGA-09-0369 | 1 | 207072612 | 207095240 | 3.018686183 | 5 | amp |
| TCGA-09-0369 | 1 | 207103650 | 208073376 | 1.427031151 | 4 | amp |
| TCGA-09-0369 | 1 | 208200545 | 208207947 | 2.999537761 | 5 | amp |
| TCGA-09-0369 | 1 | 208211677 | 209785588 | 1.992055214 | 4 | amp |
| TCGA-09-0369 | 1 | 209786114 | 209797079 | 2.736078503 | 5 | amp |
| TCGA-09-0369 | 1 | 209797144 | 215775312 | 1.422189098 | 4 | amp |
| TCGA-09-0369 | 1 | 215775364 | 220198656 | 1.001408002 | 3 | amp |
| TCGA-09-0369 | 1 | 220203714 | 220291600 | 1.538394463 | 4 | amp |
| TCGA-09-0369 | 1 | 220298539 | 220835510 | 1.014613899 | 3 | amp |
| TCGA-09-0369 | 1 | 220869893 | 222721405 | 1.686699598 | 4 | amp |
| TCGA-09-0369 | 1 | 222731997 | 222849618 | 1.014030221 | 3 | amp |
| TCGA-09-0369 | 1 | 222860264 | 223905558 | 1.379676294 | 4 | amp |
| TCGA-09-0369 | 1 | 223931740 | 223949980 | 3.026346247 | 5 | amp |
| TCGA-09-0369 | 1 | 223951820 | 225977121 | 1.30624769 | 4 | amp |
| TCGA-09-0369 | 1 | 226016401 | 226027753 | 2.742662133 | 5 | amp |
| TCGA-09-0369 | 1 | 226030060 | 226788430 | 1.824386022 | 4 | amp |
| TCGA-09-0369 | 1 | 226825355 | 227174475 | 3.104922289 | 5 | amp |
| TCGA-09-0369 | 1 | 227181950 | 227198791 | 1.586067417 | 4 | amp |
| TCGA-09-0369 | 1 | 227203719 | 227843562 | 0.943888549 | 3 | amp |
| TCGA-09-0369 | 1 | 227922314 | 228284985 | 2.65952202 | 5 | amp |
| TCGA-09-0369 | 1 | 228285007 | 228476637 | 1.791928499 | 4 | amp |
| TCGA-09-0369 | 1 | 228479550 | 228495275 | 3.453559369 | 5 | amp |
| TCGA-09-0369 | 1 | 228497162 | 228596476 | 2.043532543 | 4 | amp |
| TCGA-09-0369 | 1 | 228596834 | 228873508 | 2.507210796 | 5 | amp |
| TCGA-09-0369 | 1 | 228878989 | 232651123 | 1.502397845 | 4 | amp |
| TCGA-09-0369 | 1 | 232940731 | 233137474 | 3.048801736 | 5 | amp |
| TCGA-09-0369 | 1 | 233150406 | 234596172 | 1.686288546 | 4 | amp |
| TCGA-09-0369 | 1 | 234599571 | 235318431 | 2.720634759 | 5 | amp |
| TCGA-09-0369 | 1 | 235323773 | 242451867 | 1.479029915 | 4 | amp |
| TCGA-09-0369 | 1 | 242511365 | 244769106 | 1.117567114 | 3 | amp |
| TCGA-09-0369 | 1 | 244773524 | 245809622 | 1.678878292 | 4 | amp |
| TCGA-09-0369 | 1 | 245847495 | 245927456 | 4.302401611 | 5 | amp |
| TCGA-09-0369 | 1 | 246021764 | 247201779 | 1.536120737 | 4 | amp |
| TCGA-09-0369 | 1 | 247202081 | 247265471 | 3.840542173 | 5 | amp |
| TCGA-09-0369 | 1 | 247319833 | 247420291 | 1.93747921 | 4 | amp |
| TCGA-09-0369 | 1 | 247463749 | 247582405 | 3.084424166 | 5 | amp |
| TCGA-09-0369 | 1 | 247586471 | 248814188 | 1.576721391 | 4 | amp |
| TCGA-09-0369 | 1 | 248844657 | 249231325 | 2.886308537 | 5 | amp |
| TCGA-09-0369 | 10 | 92880 | 5965681 | 1.613845348 | 4 | amp |
| TCGA-09-0369 | 10 | 5966222 | 6274941 | 2.59238446 | 5 | amp |
| TCGA-09-0369 | 10 | 6470096 | 11963361 | 1.548139953 | 4 | amp |
| TCGA-09-0369 | 10 | 11971803 | 12077479 | 0.911966345 | 3 | amp |
| TCGA-09-0369 | 10 | 12123428 | 15600256 | 1.632696264 | 4 | amp |
| TCGA-09-0369 | 10 | 15614168 | 17216664 | 1.058530769 | 3 | amp |
| TCGA-09-0369 | 10 | 17272588 | 17279309 | 1.831518126 | 4 | amp |
| TCGA-09-0369 | 10 | 17362870 | 22209929 | 1.04972687 | 3 | amp |
| TCGA-09-0369 | 10 | 22217397 | 24822202 | 1.326634215 | 4 | amp |
| TCGA-09-0369 | 10 | 24825642 | 26465821 | 1.009852408 | 3 | amp |
| TCGA-09-0369 | 10 | 26482126 | 27054256 | 1.375179765 | 4 | amp |
| TCGA-09-0369 | 10 | 27057700 | 27353053 | 0.875095706 | 3 | amp |

| | | | | | | |
|---|---|---|---|---|---|---|
| TCGA-09-0369 | 10 | 27355386 | 27817305 | 1.377995116 | 4 | amp |
| TCGA-09-0369 | 10 | 27821411 | 28900896 | 1.04202359 | 3 | amp |
| TCGA-09-0369 | 10 | 28903404 | 31750185 | 1.39351359 | 4 | amp |
| TCGA-09-0369 | 10 | 31784679 | 33221545 | 1.023650148 | 3 | amp |
| TCGA-09-0369 | 10 | 33224392 | 42772288 | 1.269708097 | 4 | amp |
| TCGA-09-0369 | 10 | 43015939 | 43319264 | 0.936395443 | 3 | amp |
| TCGA-09-0369 | 10 | 43325669 | 45959790 | 1.451406607 | 4 | amp |
| TCGA-09-0369 | 10 | 45984779 | 46673730 | 1.012841071 | 3 | amp |
| TCGA-09-0369 | 10 | 46675443 | 47894662 | 1.492192801 | 4 | amp |
| TCGA-09-0369 | 10 | 47896622 | 48050564 | 0.757032383 | 3 | amp |
| TCGA-09-0369 | 10 | 48187381 | 48429583 | 1.464428267 | 4 | amp |
| TCGA-09-0369 | 10 | 48738593 | 48860629 | 0.906147123 | 3 | amp |
| TCGA-09-0369 | 10 | 48862343 | 49233992 | 1.442852978 | 4 | amp |
| TCGA-09-0369 | 10 | 49236562 | 49634604 | 0.99830779 | 3 | amp |
| TCGA-09-0369 | 10 | 49635064 | 50599389 | 1.377292122 | 4 | amp |
| TCGA-09-0369 | 10 | 50666830 | 50732293 | 0.736340584 | 3 | amp |
| TCGA-09-0369 | 10 | 50732404 | 50960794 | 1.401750112 | 4 | amp |
| TCGA-09-0369 | 10 | 50964786 | 51877862 | 1.036487585 | 3 | amp |
| TCGA-09-0369 | 10 | 70043899 | 70728924 | 0.994900244 | 3 | amp |
| TCGA-09-0369 | 10 | 70729911 | 74128152 | 1.549230367 | 4 | amp |
| TCGA-09-0369 | 10 | 74135525 | 75335462 | 0.952553892 | 3 | amp |
| TCGA-09-0369 | 10 | 75391653 | 75676354 | 1.725422103 | 4 | amp |
| TCGA-09-0369 | 10 | 75802815 | 76790845 | 0.848708293 | 3 | amp |
| TCGA-09-0369 | 10 | 76797504 | 78084260 | 1.40113973 | 4 | amp |
| TCGA-09-0369 | 10 | 78316946 | 79493777 | 1.007949396 | 3 | amp |
| TCGA-09-0369 | 10 | 79552159 | 79750928 | 1.37390455 | 4 | amp |
| TCGA-09-0369 | 10 | 79752919 | 79782202 | 0.889365116 | 3 | amp |
| TCGA-09-0369 | 10 | 79784289 | 86004945 | 1.310136305 | 4 | amp |
| TCGA-09-0369 | 10 | 86007274 | 88260527 | 1.038102339 | 3 | amp |
| TCGA-09-0369 | 10 | 88277279 | 88492748 | 1.992469227 | 4 | amp |
| TCGA-09-0369 | 10 | 88635748 | 89272989 | 1.136688741 | 3 | amp |
| TCGA-09-0369 | 10 | 94816676 | 95373022 | 1.095493027 | 3 | amp |
| TCGA-09-0369 | 10 | 97096244 | 98416742 | 1.025505803 | 3 | amp |
| TCGA-09-0369 | 10 | 98469261 | 100221619 | 1.485237318 | 4 | amp |
| TCGA-09-0369 | 10 | 100242315 | 101579055 | 1.030387077 | 3 | amp |
| TCGA-09-0369 | 10 | 101590006 | 103609710 | 1.44402492 | 4 | amp |
| TCGA-09-0369 | 10 | 103649117 | 103769816 | 0.820571487 | 3 | amp |
| TCGA-09-0369 | 10 | 103771449 | 105420926 | 1.422516794 | 4 | amp |
| TCGA-09-0369 | 10 | 105483983 | 105781494 | 0.897791739 | 3 | amp |
| TCGA-09-0369 | 10 | 105785284 | 105821256 | 1.597646958 | 4 | amp |
| TCGA-09-0369 | 10 | 105821991 | 105891973 | 1.064215158 | 3 | amp |
| TCGA-09-0369 | 10 | 114201909 | 115531871 | 1.05647064 | 3 | amp |
| TCGA-09-0369 | 10 | 115985771 | 116100645 | 1.587203813 | 4 | amp |
| TCGA-09-0369 | 10 | 116196015 | 116590709 | 1.02652385 | 3 | amp |
| TCGA-09-0369 | 10 | 118327218 | 120820376 | 1.114759714 | 3 | amp |
| TCGA-09-0369 | 10 | 120820697 | 121286996 | 1.500210324 | 4 | amp |
| TCGA-09-0369 | 10 | 121335119 | 123719122 | 0.971422696 | 3 | amp |
| TCGA-09-0369 | 10 | 123719836 | 124376819 | 1.285545614 | 4 | amp |
| TCGA-09-0369 | 10 | 124377514 | 125558732 | 1.144779353 | 3 | amp |
| TCGA-09-0369 | 10 | 125601784 | 126523545 | 1.392220173 | 4 | amp |

| | | | | | | |
|---|---|---|---|---|---|---|
| TCGA-09-0369 | 10 | 126631049 | 128147817 | 1.13238551 | 3 | amp |
| TCGA-09-0369 | 10 | 128149911 | 135516111 | 1.446757353 | 4 | amp |
| TCGA-09-0369 | 11 | 86637 | 219056 | 1.536626763 | 4 | amp |
| TCGA-09-0369 | 11 | 224008 | 247482 | 2.21222916 | 5 | amp |
| TCGA-09-0369 | 11 | 248725 | 700720 | 1.842671767 | 4 | amp |
| TCGA-09-0369 | 11 | 755820 | 1213787 | 2.28057845 | 5 | amp |
| TCGA-09-0369 | 11 | 1260611 | 3000503 | 1.449486768 | 4 | amp |
| TCGA-09-0369 | 11 | 3022290 | 3033525 | 2.410099865 | 5 | amp |
| TCGA-09-0369 | 11 | 3036954 | 3392989 | 1.457628657 | 4 | amp |
| TCGA-09-0369 | 11 | 3424792 | 3435279 | 2.521494246 | 5 | amp |
| TCGA-09-0369 | 11 | 3578477 | 3578797 | 0.94486423 | 3 | amp |
| TCGA-09-0369 | 11 | 3659905 | 3697854 | 2.383628015 | 5 | amp |
| TCGA-09-0369 | 11 | 3697863 | 3712767 | 1.54245391 | 4 | amp |
| TCGA-09-0369 | 11 | 3832421 | 3832691 | 1.595983755 | 4 | amp |
| TCGA-09-0369 | 11 | 3838523 | 3877689 | 3.069364836 | 5 | amp |
| TCGA-09-0369 | 11 | 3988726 | 4133330 | 1.327501996 | 4 | amp |
| TCGA-09-0369 | 11 | 4139500 | 6221474 | 0.817740545 | 3 | amp |
| TCGA-09-0369 | 11 | 6226791 | 6662676 | 1.509176437 | 4 | amp |
| TCGA-09-0369 | 11 | 6703310 | 7649615 | 0.855655861 | 3 | amp |
| TCGA-09-0369 | 11 | 7650667 | 7686810 | 1.291628434 | 4 | amp |
| TCGA-09-0369 | 11 | 7687641 | 7693777 | 2.278676574 | 5 | amp |
| TCGA-09-0369 | 11 | 7693927 | 8720560 | 1.1246609 | 3 | amp |
| TCGA-09-0369 | 11 | 8720728 | 9168757 | 1.383358831 | 4 | amp |
| TCGA-09-0369 | 11 | 9171541 | 11977754 | 0.920052782 | 3 | amp |
| TCGA-09-0369 | 11 | 11985971 | 12280118 | 1.483636779 | 4 | amp |
| TCGA-09-0369 | 11 | 12281302 | 17409651 | 0.838303541 | 3 | amp |
| TCGA-09-0369 | 11 | 17414486 | 17809975 | 1.479767192 | 4 | amp |
| TCGA-09-0369 | 11 | 17899712 | 18655834 | 0.954782234 | 3 | amp |
| TCGA-09-0369 | 11 | 18722401 | 18793555 | 1.911374274 | 4 | amp |
| TCGA-09-0369 | 11 | 18955276 | 20117325 | 0.966384096 | 3 | amp |
| TCGA-09-0369 | 11 | 20119086 | 20629231 | 1.321946573 | 4 | amp |
| TCGA-09-0369 | 11 | 20636175 | 31805126 | 0.735935699 | 3 | amp |
| TCGA-09-0369 | 11 | 31811453 | 32617612 | 1.275808302 | 4 | amp |
| TCGA-09-0369 | 11 | 32622186 | 34135443 | 0.889655163 | 3 | amp |
| TCGA-09-0369 | 11 | 34137310 | 34473808 | 1.24964586 | 4 | amp |
| TCGA-09-0369 | 11 | 34474510 | 43436352 | 0.888860222 | 3 | amp |
| TCGA-09-0369 | 11 | 43464836 | 44089501 | 1.579057454 | 4 | amp |
| TCGA-09-0369 | 11 | 44092745 | 44105170 | 2.313542623 | 5 | amp |
| TCGA-09-0369 | 11 | 44105203 | 44265887 | 1.3113938 | 4 | amp |
| TCGA-09-0369 | 11 | 44288957 | 45957291 | 2.410202727 | 5 | amp |
| TCGA-09-0369 | 11 | 45957992 | 46105803 | 1.190658791 | 3 | amp |
| TCGA-09-0369 | 11 | 46321479 | 46430279 | 3.326065822 | 5 | amp |
| TCGA-09-0369 | 11 | 46431781 | 46686513 | 1.537338123 | 4 | amp |
| TCGA-09-0369 | 11 | 46686880 | 46750409 | 2.786455088 | 5 | amp |
| TCGA-09-0369 | 11 | 46750900 | 46761006 | 1.517762922 | 4 | amp |
| TCGA-09-0369 | 11 | 46765513 | 46802066 | 0.932449501 | 3 | amp |
| TCGA-09-0369 | 11 | 46804810 | 46905534 | 1.359913802 | 4 | amp |
| TCGA-09-0369 | 11 | 46907592 | 47314210 | 2.4138522 | 5 | amp |
| TCGA-09-0369 | 11 | 47315423 | 47355325 | 1.427007305 | 4 | amp |
| TCGA-09-0369 | 11 | 47358915 | 47469712 | 2.990058491 | 5 | amp |

| | | | | | | |
|---|---|---|---|---|---|---|
| TCGA-09-0369 | 11 | 47470270 | 47510597 | 1.171166186 | 3 | amp |
| TCGA-09-0369 | 11 | 47591197 | 47660363 | 2.982538435 | 5 | amp |
| TCGA-09-0369 | 11 | 47660470 | 49884094 | 1.296528301 | 4 | amp |
| TCGA-09-0369 | 11 | 49893373 | 56786270 | 1.040916659 | 3 | amp |
| TCGA-09-0369 | 11 | 56949312 | 57506742 | 2.693854335 | 5 | amp |
| TCGA-09-0369 | 11 | 57507493 | 59376261 | 1.265284962 | 4 | amp |
| TCGA-09-0369 | 11 | 59377837 | 59425218 | 2.325412828 | 5 | amp |
| TCGA-09-0369 | 11 | 59480357 | 60235951 | 1.29669152 | 4 | amp |
| TCGA-09-0369 | 11 | 60264749 | 60274604 | 2.624753001 | 5 | amp |
| TCGA-09-0369 | 11 | 60285500 | 60541426 | 1.504030818 | 4 | amp |
| TCGA-09-0369 | 11 | 60543042 | 60617509 | 2.637957883 | 5 | amp |
| TCGA-09-0369 | 11 | 60617588 | 60658746 | 1.527616402 | 4 | amp |
| TCGA-09-0369 | 11 | 60665280 | 60971779 | 2.812252136 | 5 | amp |
| TCGA-09-0369 | 11 | 60973920 | 60978917 | 1.549543654 | 4 | amp |
| TCGA-09-0369 | 11 | 60979979 | 61919460 | 2.64556094 | 5 | amp |
| TCGA-09-0369 | 11 | 61957723 | 62012216 | 1.241006027 | 3 | amp |
| TCGA-09-0369 | 11 | 62037625 | 62475876 | 3.184668512 | 5 | amp |
| TCGA-09-0369 | 11 | 62476093 | 62491950 | 1.696172541 | 4 | amp |
| TCGA-09-0369 | 11 | 62496259 | 62602008 | 3.17217318 | 5 | amp |
| TCGA-09-0369 | 11 | 62602893 | 62623129 | 1.657768791 | 4 | amp |
| TCGA-09-0369 | 11 | 62623707 | 62752217 | 2.152082376 | 5 | amp |
| TCGA-09-0369 | 11 | 62760698 | 62782443 | 1.583253891 | 4 | amp |
| TCGA-09-0369 | 11 | 62931210 | 63177393 | 0.890824583 | 3 | amp |
| TCGA-09-0369 | 11 | 63230930 | 63274051 | 1.794374792 | 4 | amp |
| TCGA-09-0369 | 11 | 63275978 | 63330851 | 2.355861566 | 5 | amp |
| TCGA-09-0369 | 11 | 63342377 | 63523672 | 1.472309864 | 4 | amp |
| TCGA-09-0369 | 11 | 63525567 | 64854103 | 2.838487958 | 5 | amp |
| TCGA-09-0369 | 11 | 64854167 | 64882885 | 1.498830911 | 4 | amp |
| TCGA-09-0369 | 11 | 64882888 | 65036232 | 2.528844845 | 5 | amp |
| TCGA-09-0369 | 11 | 65043325 | 65050030 | 1.502450671 | 4 | amp |
| TCGA-09-0369 | 11 | 65055128 | 66278581 | 2.753613744 | 5 | amp |
| TCGA-09-0369 | 11 | 66278602 | 66318826 | 1.63771328 | 4 | amp |
| TCGA-09-0369 | 11 | 66318998 | 66568199 | 3.42296296 | 5 | amp |
| TCGA-09-0369 | 11 | 66568477 | 66595845 | 1.550507784 | 4 | amp |
| TCGA-09-0369 | 11 | 66605816 | 66888868 | 2.922767692 | 5 | amp |
| TCGA-09-0369 | 11 | 66947498 | 66983433 | 1.430754773 | 4 | amp |
| TCGA-09-0369 | 11 | 66985158 | 67353688 | 2.84321359 | 5 | amp |
| TCGA-09-0369 | 11 | 67353833 | 67379783 | 1.38147616 | 4 | amp |
| TCGA-09-0369 | 11 | 67379795 | 67412629 | 3.60854859 | 5 | amp |
| TCGA-09-0369 | 11 | 67430627 | 67434451 | 1.536337943 | 4 | amp |
| TCGA-09-0369 | 11 | 67557509 | 67832104 | 2.930882757 | 5 | amp |
| TCGA-09-0369 | 11 | 67833216 | 67957583 | 1.399930949 | 4 | amp |
| TCGA-09-0369 | 11 | 68029089 | 68216587 | 2.822343269 | 5 | amp |
| TCGA-09-0369 | 11 | 68305125 | 68453148 | 1.31604606 | 4 | amp |
| TCGA-09-0369 | 11 | 68455464 | 68478549 | 3.62630539 | 5 | amp |
| TCGA-09-0369 | 11 | 68480656 | 68514874 | 1.452737604 | 4 | amp |
| TCGA-09-0369 | 11 | 68522211 | 68837990 | 2.387907867 | 5 | amp |
| TCGA-09-0369 | 11 | 68839350 | 68846291 | 1.216798578 | 3 | amp |
| TCGA-09-0369 | 11 | 68846303 | 70118590 | 5.311298908 | 5 | amp |
| TCGA-09-0369 | 11 | 70130158 | 70261855 | 1.468192715 | 4 | amp |

| | | | | | | |
|---|---|---|---|---|---|---|
| TCGA-09-0369 | 11 | 70263083 | 71548644 | 2.299560347 | 5 | amp |
| TCGA-09-0369 | 11 | 71567291 | 71707420 | 1.367682989 | 4 | amp |
| TCGA-09-0369 | 11 | 71710136 | 72019669 | 2.221479259 | 5 | amp |
| TCGA-09-0369 | 11 | 72028116 | 72070107 | 1.107498356 | 3 | amp |
| TCGA-09-0369 | 11 | 72083923 | 72539598 | 2.750866015 | 5 | amp |
| TCGA-09-0369 | 11 | 72539737 | 73008596 | 1.238884283 | 3 | amp |
| TCGA-09-0369 | 11 | 73019659 | 73122629 | 2.973571434 | 5 | amp |
| TCGA-09-0369 | 11 | 73130831 | 73598510 | 1.46457714 | 4 | amp |
| TCGA-09-0369 | 11 | 73602141 | 73718144 | 2.510819857 | 5 | amp |
| TCGA-09-0369 | 11 | 73745587 | 74351829 | 1.207631778 | 3 | amp |
| TCGA-09-0369 | 11 | 74407526 | 74429880 | 2.521822247 | 5 | amp |
| TCGA-09-0369 | 11 | 74500617 | 74875196 | 1.50109461 | 4 | amp |
| TCGA-09-0369 | 11 | 74876762 | 75508409 | 2.434945613 | 5 | amp |
| TCGA-09-0369 | 11 | 75509222 | 76731404 | 1.272567686 | 4 | amp |
| TCGA-09-0369 | 11 | 76750546 | 76925024 | 2.568457153 | 5 | amp |
| TCGA-09-0369 | 11 | 76925636 | 82936116 | 1.381795299 | 4 | amp |
| TCGA-09-0369 | 11 | 82938731 | 85342855 | 0.956162707 | 3 | amp |
| TCGA-09-0369 | 11 | 85345106 | 85670155 | 1.289991814 | 4 | amp |
| TCGA-09-0369 | 11 | 85685444 | 86120416 | 0.996209465 | 3 | amp |
| TCGA-09-0369 | 11 | 86123394 | 86161451 | 2.245573617 | 5 | amp |
| TCGA-09-0369 | 11 | 86176127 | 93472496 | 1.238993446 | 3 | amp |
| TCGA-09-0369 | 11 | 93480526 | 93488583 | 2.200876839 | 5 | amp |
| TCGA-09-0369 | 11 | 93490436 | 94134459 | 1.364439785 | 4 | amp |
| TCGA-09-0369 | 11 | 94153258 | 94224184 | 0.862173827 | 3 | amp |
| TCGA-09-0369 | 11 | 94225897 | 95546812 | 1.250792093 | 4 | amp |
| TCGA-09-0369 | 11 | 95550888 | 111155131 | 0.820964293 | 3 | amp |
| TCGA-09-0369 | 11 | 111156338 | 111908210 | 1.337780598 | 4 | amp |
| TCGA-09-0369 | 11 | 111909894 | 112071535 | 0.845739473 | 3 | amp |
| TCGA-09-0369 | 11 | 112072709 | 113577004 | 1.322592564 | 4 | amp |
| TCGA-09-0369 | 11 | 113604355 | 116631679 | 0.882358928 | 3 | amp |
| TCGA-09-0369 | 11 | 116633265 | 116741141 | 1.705106004 | 4 | amp |
| TCGA-09-0369 | 11 | 116744154 | 116798135 | 0.58234153 | 1 | loss |
| TCGA-09-0369 | 11 | 116824731 | 117052858 | 1.517866344 | 4 | amp |
| TCGA-09-0369 | 11 | 117053225 | 117117714 | 2.25065773 | 5 | amp |
| TCGA-09-0369 | 11 | 117150611 | 117282691 | 1.292613074 | 4 | amp |
| TCGA-09-0369 | 11 | 117282775 | 117710590 | 2.182942641 | 5 | amp |
| TCGA-09-0369 | 11 | 117710968 | 118047171 | 1.479238026 | 4 | amp |
| TCGA-09-0369 | 11 | 118065025 | 118372599 | 0.975577534 | 3 | amp |
| TCGA-09-0369 | 11 | 118373076 | 118471444 | 1.365398481 | 4 | amp |
| TCGA-09-0369 | 11 | 118485185 | 118521260 | 2.410050616 | 5 | amp |
| TCGA-09-0369 | 11 | 118526267 | 118779411 | 1.279086721 | 4 | amp |
| TCGA-09-0369 | 11 | 118780575 | 118852004 | 2.457590757 | 5 | amp |
| TCGA-09-0369 | 11 | 118869709 | 118889712 | 1.529933199 | 4 | amp |
| TCGA-09-0369 | 11 | 118889819 | 118923193 | 2.158651622 | 5 | amp |
| TCGA-09-0369 | 11 | 118923294 | 118949083 | 1.738271538 | 4 | amp |
| TCGA-09-0369 | 11 | 118949224 | 118962913 | 2.539200449 | 5 | amp |
| TCGA-09-0369 | 11 | 118963023 | 118971164 | 1.561475631 | 4 | amp |
| TCGA-09-0369 | 11 | 118971296 | 118986980 | 2.626593412 | 5 | amp |
| TCGA-09-0369 | 11 | 118997604 | 119050999 | 1.611890017 | 4 | amp |
| TCGA-09-0369 | 11 | 119051832 | 119063965 | 2.321822902 | 5 | amp |

| | | | | | | |
|---|---|---|---|---|---|---|
| TCGA-09-0369 | 11 | 119103071 | 119170527 | 0.860822363 | 3 | amp |
| TCGA-09-0369 | 11 | 119180967 | 120139995 | 2.211679551 | 5 | amp |
| TCGA-09-0369 | 11 | 120168886 | 120186162 | 1.40315913 | 4 | amp |
| TCGA-09-0369 | 11 | 120187855 | 120690666 | 0.791014957 | 3 | amp |
| TCGA-09-0369 | 11 | 120702499 | 121037507 | 1.348938364 | 4 | amp |
| TCGA-09-0369 | 11 | 121038724 | 122830174 | 0.966534085 | 3 | amp |
| TCGA-09-0369 | 11 | 122848267 | 123524541 | 1.2457485 | 4 | amp |
| TCGA-09-0369 | 11 | 123596661 | 124617616 | 0.903648207 | 3 | amp |
| TCGA-09-0369 | 11 | 124618237 | 124742482 | 1.929443383 | 4 | amp |
| TCGA-09-0369 | 11 | 124742762 | 124757742 | 2.175752007 | 5 | amp |
| TCGA-09-0369 | 11 | 124761116 | 124793342 | 1.840230618 | 4 | amp |
| TCGA-09-0369 | 11 | 124793585 | 124910666 | 1.027288134 | 3 | amp |
| TCGA-09-0369 | 11 | 124946603 | 124955058 | 2.737383435 | 5 | amp |
| TCGA-09-0369 | 11 | 124955239 | 125497733 | 1.507432903 | 4 | amp |
| TCGA-09-0369 | 11 | 125499098 | 125548241 | 0.75525863 | 3 | amp |
| TCGA-09-0369 | 11 | 125550559 | 125848298 | 1.520441444 | 4 | amp |
| TCGA-09-0369 | 11 | 125850903 | 125893393 | 0.752251094 | 3 | amp |
| TCGA-09-0369 | 11 | 126073252 | 126137269 | 1.474307994 | 4 | amp |
| TCGA-09-0369 | 11 | 126137375 | 126870437 | 2.382850965 | 5 | amp |
| TCGA-09-0369 | 11 | 128332243 | 128807729 | 1.500186072 | 4 | amp |
| TCGA-09-0369 | 11 | 128838787 | 129062095 | 0.89089486 | 3 | amp |
| TCGA-09-0369 | 11 | 129245873 | 134031790 | 1.383521421 | 4 | amp |
| TCGA-09-0369 | 11 | 134037870 | 134087030 | 0.901706953 | 3 | amp |
| TCGA-09-0369 | 11 | 134090422 | 134177119 | 1.642085055 | 4 | amp |
| TCGA-09-0369 | 11 | 134180444 | 134252931 | 2.157197821 | 5 | amp |
| TCGA-09-0369 | 12 | 73256 | 351959 | 3.484697248 | 5 | amp |
| TCGA-09-0369 | 12 | 352791 | 551132 | 1.353900687 | 4 | amp |
| TCGA-09-0369 | 12 | 644293 | 674603 | 3.80273757 | 5 | amp |
| TCGA-09-0369 | 12 | 675109 | 1890268 | 1.693752857 | 4 | amp |
| TCGA-09-0369 | 12 | 1893022 | 2024120 | 3.340418193 | 5 | amp |
| TCGA-09-0369 | 12 | 2055321 | 2224731 | 1.749559737 | 4 | amp |
| TCGA-09-0369 | 12 | 2229484 | 4433769 | 2.643101323 | 5 | amp |
| TCGA-09-0369 | 12 | 4440404 | 5860188 | 1.639233818 | 4 | amp |
| TCGA-09-0369 | 12 | 5908644 | 7361803 | 3.059159962 | 5 | amp |
| TCGA-09-0369 | 12 | 7362237 | 7656323 | 1.427928483 | 4 | amp |
| TCGA-09-0369 | 12 | 7802097 | 8197574 | 2.416192343 | 5 | amp |
| TCGA-09-0369 | 12 | 8200379 | 9440993 | 1.80825531 | 4 | amp |
| TCGA-09-0369 | 12 | 9446201 | 9596322 | 2.793247985 | 5 | amp |
| TCGA-09-0369 | 12 | 9633110 | 20709678 | 1.47441053 | 4 | amp |
| TCGA-09-0369 | 12 | 20766325 | 21624591 | 1.097013863 | 3 | amp |
| TCGA-09-0369 | 12 | 21626470 | 30906727 | 1.325703034 | 4 | amp |
| TCGA-09-0369 | 12 | 31106907 | 31283542 | 2.891222006 | 5 | amp |
| TCGA-09-0369 | 12 | 31284251 | 38715028 | 1.565382853 | 4 | amp |
| TCGA-09-0369 | 12 | 39047627 | 39688332 | 0.947763272 | 3 | amp |
| TCGA-09-0369 | 12 | 48095196 | 48391395 | 1.665437911 | 4 | amp |
| TCGA-09-0369 | 12 | 48391439 | 49094915 | 1.061679455 | 3 | amp |
| TCGA-09-0369 | 12 | 49099494 | 50369470 | 1.593431981 | 4 | amp |
| TCGA-09-0369 | 12 | 50383998 | 52188469 | 1.044908945 | 3 | amp |
| TCGA-09-0369 | 12 | 52200039 | 53697017 | 1.393244458 | 4 | amp |
| TCGA-09-0369 | 12 | 53699647 | 53715307 | 2.506693787 | 5 | amp |

| | | | | | | |
|---|---|---|---|---|---|---|
| TCGA-09-0369 | 12 | 53721906 | 56524761 | 1.701085834 | 4 | amp |
| TCGA-09-0369 | 12 | 56524770 | 56531199 | 2.555897073 | 5 | amp |
| TCGA-09-0369 | 12 | 56531220 | 56537686 | 1.663377974 | 4 | amp |
| TCGA-09-0369 | 12 | 56547669 | 56716590 | 2.417191718 | 5 | amp |
| TCGA-09-0369 | 12 | 56716831 | 57604001 | 1.644436939 | 4 | amp |
| TCGA-09-0369 | 12 | 57604026 | 57660623 | 2.430951881 | 5 | amp |
| TCGA-09-0369 | 12 | 57662042 | 57921835 | 1.96342517 | 4 | amp |
| TCGA-09-0369 | 12 | 57921866 | 57969080 | 2.538906271 | 5 | amp |
| TCGA-09-0369 | 12 | 57969331 | 57976458 | 1.680035734 | 4 | amp |
| TCGA-09-0369 | 12 | 57976862 | 58140058 | 2.311644943 | 5 | amp |
| TCGA-09-0369 | 12 | 58140317 | 58191781 | 1.926493012 | 4 | amp |
| TCGA-09-0369 | 12 | 58193520 | 69121209 | 1.154946615 | 3 | amp |
| TCGA-09-0369 | 12 | 69124844 | 70209244 | 1.527580282 | 4 | amp |
| TCGA-09-0369 | 12 | 70671970 | 94965541 | 1.081678587 | 3 | amp |
| TCGA-09-0369 | 12 | 94972087 | 100988751 | 1.320196934 | 4 | amp |
| TCGA-09-0369 | 12 | 100994123 | 102811711 | 1.131452553 | 3 | amp |
| TCGA-09-0369 | 12 | 102813257 | 109522937 | 1.512185839 | 4 | amp |
| TCGA-09-0369 | 12 | 109523423 | 109724568 | 2.219015381 | 5 | amp |
| TCGA-09-0369 | 12 | 109826466 | 109872939 | 1.811105753 | 4 | amp |
| TCGA-09-0369 | 12 | 109874266 | 109880109 | 3.808148273 | 5 | amp |
| TCGA-09-0369 | 12 | 109881266 | 109961971 | 1.816820367 | 4 | amp |
| TCGA-09-0369 | 12 | 109962215 | 110346768 | 3.065406855 | 5 | amp |
| TCGA-09-0369 | 12 | 110350776 | 110824322 | 1.600250765 | 4 | amp |
| TCGA-09-0369 | 12 | 110825470 | 110893737 | 2.452867195 | 5 | amp |
| TCGA-09-0369 | 12 | 110895290 | 111162576 | 1.670927107 | 4 | amp |
| TCGA-09-0369 | 12 | 111168298 | 111786148 | 2.30426606 | 5 | amp |
| TCGA-09-0369 | 12 | 111884547 | 112147528 | 1.698725215 | 4 | amp |
| TCGA-09-0369 | 12 | 112150261 | 112237908 | 2.477704055 | 5 | amp |
| TCGA-09-0369 | 12 | 112241659 | 113448293 | 1.535337804 | 4 | amp |
| TCGA-09-0369 | 12 | 113515221 | 114261137 | 2.887620075 | 5 | amp |
| TCGA-09-0369 | 12 | 114282382 | 120117831 | 1.640786834 | 4 | amp |
| TCGA-09-0369 | 12 | 120118031 | 120156704 | 2.550478619 | 5 | amp |
| TCGA-09-0369 | 12 | 120158276 | 120510543 | 1.415521015 | 4 | amp |
| TCGA-09-0369 | 12 | 120518635 | 120576699 | 2.455104419 | 5 | amp |
| TCGA-09-0369 | 12 | 120578594 | 120634851 | 1.926611328 | 4 | amp |
| TCGA-09-0369 | 12 | 120635074 | 120650429 | 2.616433094 | 5 | amp |
| TCGA-09-0369 | 12 | 120653353 | 121019215 | 1.818366952 | 4 | amp |
| TCGA-09-0369 | 12 | 121088372 | 121165030 | 2.591291035 | 5 | amp |
| TCGA-09-0369 | 12 | 121175154 | 121432267 | 1.600763316 | 4 | amp |
| TCGA-09-0369 | 12 | 121434020 | 121444228 | 3.035678546 | 5 | amp |
| TCGA-09-0369 | 12 | 121448619 | 121868301 | 1.749258533 | 4 | amp |
| TCGA-09-0369 | 12 | 121877648 | 122497093 | 2.601528051 | 5 | amp |
| TCGA-09-0369 | 12 | 122611789 | 122657303 | 1.794356412 | 4 | amp |
| TCGA-09-0369 | 12 | 122658344 | 122701420 | 2.506778231 | 5 | amp |
| TCGA-09-0369 | 12 | 122702779 | 124382535 | 1.528687813 | 4 | amp |
| TCGA-09-0369 | 12 | 124395030 | 125621428 | 2.564407614 | 5 | amp |
| TCGA-09-0369 | 12 | 125626556 | 132380251 | 1.58472172 | 4 | amp |
| TCGA-09-0369 | 12 | 132380258 | 132530164 | 2.430335315 | 5 | amp |
| TCGA-09-0369 | 12 | 132530208 | 133252794 | 1.743304653 | 4 | amp |
| TCGA-09-0369 | 12 | 133253095 | 133435853 | 2.302565006 | 5 | amp |

| | | | | | | |
|---|---|---|---|---|---|---|
| TCGA-09-0369 | 12 | 133438016 | 133779395 | 1.66361941 | 4 | amp |
| TCGA-09-0369 | 13 | 19240876 | 98673436 | 0.913385738 | 3 | amp |
| TCGA-09-0369 | 13 | 98673972 | 99134606 | 1.919366307 | 4 | amp |
| TCGA-09-0369 | 13 | 99171491 | 110895111 | 1.204676197 | 3 | amp |
| TCGA-09-0369 | 13 | 110960352 | 113219518 | 1.880237059 | 4 | amp |
| TCGA-09-0369 | 13 | 113223459 | 114751297 | 2.265717757 | 5 | amp |
| TCGA-09-0369 | 13 | 114766294 | 115091796 | 1.487015549 | 4 | amp |
| TCGA-09-0369 | 14 | 19377543 | 107042692 | 1.242273522 | 3 | amp |
| TCGA-09-0369 | 14 | 107048641 | 107283263 | 1.323912786 | 4 | amp |
| TCGA-09-0369 | 15 | 20169886 | 20434059 | 1.417511155 | 4 | amp |
| TCGA-09-0369 | 15 | 20444702 | 20495466 | 3.196571366 | 5 | amp |
| TCGA-09-0369 | 15 | 20588526 | 25299463 | 1.661267634 | 4 | amp |
| TCGA-09-0369 | 15 | 25301993 | 25322303 | 2.507530376 | 5 | amp |
| TCGA-09-0369 | 15 | 25324190 | 25351768 | 1.168776724 | 3 | amp |
| TCGA-09-0369 | 15 | 25415850 | 25486993 | 2.361693218 | 5 | amp |
| TCGA-09-0369 | 15 | 25488741 | 32908596 | 1.389901743 | 4 | amp |
| TCGA-09-0369 | 15 | 32912283 | 34115294 | 1.155587772 | 3 | amp |
| TCGA-09-0369 | 15 | 34117780 | 34647399 | 1.30169742 | 4 | amp |
| TCGA-09-0369 | 15 | 34647670 | 34656510 | 2.316659734 | 5 | amp |
| TCGA-09-0369 | 15 | 34657198 | 40512982 | 1.326601941 | 4 | amp |
| TCGA-09-0369 | 15 | 40556938 | 40675260 | 2.813082642 | 5 | amp |
| TCGA-09-0369 | 15 | 40675385 | 41106019 | 1.651797062 | 4 | amp |
| TCGA-09-0369 | 15 | 41106054 | 41248011 | 2.292299801 | 5 | amp |
| TCGA-09-0369 | 15 | 41272379 | 41768023 | 1.615915937 | 4 | amp |
| TCGA-09-0369 | 15 | 41768592 | 41870518 | 2.838202554 | 5 | amp |
| TCGA-09-0369 | 15 | 41961084 | 42092126 | 1.49853937 | 4 | amp |
| TCGA-09-0369 | 15 | 42103051 | 42377374 | 2.368476911 | 5 | amp |
| TCGA-09-0369 | 15 | 42377618 | 43020012 | 1.376312248 | 4 | amp |
| TCGA-09-0369 | 15 | 43020101 | 43027575 | 2.569438624 | 5 | amp |
| TCGA-09-0369 | 15 | 43027672 | 43473557 | 1.37839581 | 4 | amp |
| TCGA-09-0369 | 15 | 43476440 | 43627396 | 2.153350315 | 5 | amp |
| TCGA-09-0369 | 15 | 43627838 | 43875739 | 1.44182781 | 4 | amp |
| TCGA-09-0369 | 15 | 43876508 | 43908919 | 2.275990958 | 5 | amp |
| TCGA-09-0369 | 15 | 43909607 | 43976583 | 1.48292032 | 4 | amp |
| TCGA-09-0369 | 15 | 43986202 | 44010043 | 2.351933589 | 5 | amp |
| TCGA-09-0369 | 15 | 44010207 | 44065583 | 1.724611483 | 4 | amp |
| TCGA-09-0369 | 15 | 44066315 | 44093009 | 2.514540554 | 5 | amp |
| TCGA-09-0369 | 15 | 44093267 | 45365765 | 1.444586059 | 4 | amp |
| TCGA-09-0369 | 15 | 45386258 | 45545733 | 2.339984847 | 5 | amp |
| TCGA-09-0369 | 15 | 45554168 | 45981445 | 1.51111589 | 4 | amp |
| TCGA-09-0369 | 15 | 45983139 | 62333603 | 1.065823109 | 3 | amp |
| TCGA-09-0369 | 15 | 62336372 | 63915111 | 1.496640261 | 4 | amp |
| TCGA-09-0369 | 15 | 63915886 | 64050590 | 1.078124993 | 3 | amp |
| TCGA-09-0369 | 15 | 64056282 | 64428628 | 1.536758265 | 4 | amp |
| TCGA-09-0369 | 15 | 64429035 | 64495394 | 2.273926137 | 5 | amp |
| TCGA-09-0369 | 15 | 64496593 | 64973587 | 1.459636885 | 4 | amp |
| TCGA-09-0369 | 15 | 64980847 | 65242247 | 2.37668323 | 5 | amp |
| TCGA-09-0369 | 15 | 65255868 | 65621951 | 1.751770512 | 4 | amp |
| TCGA-09-0369 | 15 | 65621982 | 65702706 | 2.173313231 | 5 | amp |
| TCGA-09-0369 | 15 | 65703322 | 68492068 | 1.337177486 | 4 | amp |

| | | | | | | |
|---|---|---|---|---|---|---|
| TCGA-09-0369 | 15 | 68497383 | 69349090 | 2.26481261 | 5 | amp |
| TCGA-09-0369 | 15 | 69548138 | 70368525 | 1.859094344 | 4 | amp |
| TCGA-09-0369 | 15 | 70949370 | 71128858 | 0.726958231 | 3 | amp |
| TCGA-09-0369 | 15 | 71144007 | 72122670 | 1.45409894 | 4 | amp |
| TCGA-09-0369 | 15 | 72141127 | 72432643 | 0.866669902 | 3 | amp |
| TCGA-09-0369 | 15 | 72454261 | 72932255 | 1.640981199 | 4 | amp |
| TCGA-09-0369 | 15 | 72947060 | 72958739 | 2.289322677 | 5 | amp |
| TCGA-09-0369 | 15 | 72987483 | 74003553 | 1.229180538 | 3 | amp |
| TCGA-09-0369 | 15 | 74005195 | 75664554 | 2.39264311 | 5 | amp |
| TCGA-09-0369 | 15 | 75667989 | 75715195 | 1.408161257 | 4 | amp |
| TCGA-09-0369 | 15 | 75722471 | 76075727 | 2.259123451 | 5 | amp |
| TCGA-09-0369 | 15 | 76077825 | 78894640 | 1.628342166 | 4 | amp |
| TCGA-09-0369 | 15 | 78909330 | 79090488 | 2.322764786 | 5 | amp |
| TCGA-09-0369 | 15 | 79170517 | 82665047 | 1.559839252 | 4 | amp |
| TCGA-09-0369 | 15 | 82724742 | 83350371 | 2.384913928 | 5 | amp |
| TCGA-09-0369 | 15 | 83357475 | 83788441 | 1.535551523 | 4 | amp |
| TCGA-09-0369 | 15 | 83790653 | 84795403 | 1.031769196 | 3 | amp |
| TCGA-09-0369 | 15 | 84859539 | 89009030 | 1.500633949 | 4 | amp |
| TCGA-09-0369 | 15 | 89010344 | 89453208 | 2.162517404 | 5 | amp |
| TCGA-09-0369 | 15 | 89659505 | 89849451 | 1.614678752 | 4 | amp |
| TCGA-09-0369 | 15 | 89850673 | 90135462 | 2.565775611 | 5 | amp |
| TCGA-09-0369 | 15 | 90137595 | 90278812 | 1.611332616 | 4 | amp |
| TCGA-09-0369 | 15 | 90280761 | 90934115 | 2.35864132 | 5 | amp |
| TCGA-09-0369 | 15 | 90969299 | 91354655 | 1.277165769 | 4 | amp |
| TCGA-09-0369 | 15 | 91358300 | 91553113 | 2.957330985 | 5 | amp |
| TCGA-09-0369 | 15 | 91556977 | 96875792 | 1.35430292 | 4 | amp |
| TCGA-09-0369 | 15 | 96877238 | 98514015 | 2.387741917 | 5 | amp |
| TCGA-09-0369 | 15 | 98514298 | 100533445 | 1.453120792 | 4 | amp |
| TCGA-09-0369 | 15 | 100537529 | 100739635 | 0.950962342 | 3 | amp |
| TCGA-09-0369 | 16 | 66517 | 3795376 | 2.72755956 | 5 | amp |
| TCGA-09-0369 | 16 | 3799595 | 4029316 | 1.574387159 | 4 | amp |
| TCGA-09-0369 | 16 | 4038955 | 7102145 | 2.883392076 | 5 | amp |
| TCGA-09-0369 | 16 | 7382955 | 19056384 | 1.39223623 | 4 | amp |
| TCGA-09-0369 | 16 | 19056651 | 19191906 | 2.533810492 | 5 | amp |
| TCGA-09-0369 | 16 | 19194799 | 21136713 | 1.527075944 | 4 | amp |
| TCGA-09-0369 | 16 | 21138920 | 21211251 | 2.500894949 | 5 | amp |
| TCGA-09-0369 | 16 | 21212664 | 22149876 | 1.384789379 | 4 | amp |
| TCGA-09-0369 | 16 | 22151459 | 22328541 | 2.359965983 | 5 | amp |
| TCGA-09-0369 | 16 | 22330086 | 23387237 | 1.331873339 | 4 | amp |
| TCGA-09-0369 | 16 | 23388433 | 23404670 | 2.888719639 | 5 | amp |
| TCGA-09-0369 | 16 | 23409348 | 23504789 | 1.703896284 | 4 | amp |
| TCGA-09-0369 | 16 | 23505571 | 23598698 | 2.59784086 | 5 | amp |
| TCGA-09-0369 | 16 | 23614734 | 23695420 | 1.660229756 | 4 | amp |
| TCGA-09-0369 | 16 | 23698717 | 23848771 | 2.779041003 | 5 | amp |
| TCGA-09-0369 | 16 | 23999811 | 27777832 | 1.682168465 | 4 | amp |
| TCGA-09-0369 | 16 | 27781110 | 27840313 | 2.788989258 | 5 | amp |
| TCGA-09-0369 | 16 | 27856236 | 28603088 | 1.751310586 | 4 | amp |
| TCGA-09-0369 | 16 | 28603344 | 28723118 | 3.083361178 | 5 | amp |
| TCGA-09-0369 | 16 | 28724994 | 28743571 | 1.620802912 | 4 | amp |
| TCGA-09-0369 | 16 | 28745800 | 29065167 | 2.874820035 | 5 | amp |

| | | | | | | |
|---|---|---|---|---|---|---|
| TCGA-09-0369 | 16 | 29089190 | 29458354 | 1.605089134 | 4 | amp |
| TCGA-09-0369 | 16 | 29464943 | 29495233 | 2.869357876 | 5 | amp |
| TCGA-09-0369 | 16 | 29496773 | 29690584 | 1.350913033 | 4 | amp |
| TCGA-09-0369 | 16 | 29705922 | 30225952 | 2.917686284 | 5 | amp |
| TCGA-09-0369 | 16 | 30233139 | 30364420 | 1.164294428 | 3 | amp |
| TCGA-09-0369 | 16 | 30364494 | 31283360 | 2.835140607 | 5 | amp |
| TCGA-09-0369 | 16 | 31284642 | 31342600 | 1.80302513 | 4 | amp |
| TCGA-09-0369 | 16 | 31367176 | 31505281 | 3.329278122 | 5 | amp |
| TCGA-09-0369 | 16 | 31510615 | 56602858 | 1.487611936 | 4 | amp |
| TCGA-09-0369 | 16 | 56623427 | 56717169 | 2.505474446 | 5 | amp |
| TCGA-09-0369 | 16 | 56717825 | 56878525 | 1.327777645 | 4 | amp |
| TCGA-09-0369 | 16 | 56892381 | 56933544 | 3.649153586 | 5 | amp |
| TCGA-09-0369 | 16 | 56936232 | 57110832 | 1.907582541 | 4 | amp |
| TCGA-09-0369 | 16 | 57111159 | 57180294 | 2.749335747 | 5 | amp |
| TCGA-09-0369 | 16 | 57181426 | 57718408 | 2.005489584 | 4 | amp |
| TCGA-09-0369 | 16 | 57719486 | 58076323 | 2.733497678 | 5 | amp |
| TCGA-09-0369 | 16 | 58147871 | 66421035 | 1.478727818 | 4 | amp |
| TCGA-09-0369 | 16 | 66422164 | 66551795 | 3.340913189 | 5 | amp |
| TCGA-09-0369 | 16 | 66562873 | 66852545 | 1.758615844 | 4 | amp |
| TCGA-09-0369 | 16 | 66855347 | 67315100 | 2.644844592 | 5 | amp |
| TCGA-09-0369 | 16 | 67315593 | 67686218 | 1.882066844 | 4 | amp |
| TCGA-09-0369 | 16 | 67689891 | 68057133 | 2.887697564 | 5 | amp |
| TCGA-09-0369 | 16 | 68071869 | 68266594 | 1.815250046 | 4 | amp |
| TCGA-09-0369 | 16 | 68266600 | 68321831 | 2.752290387 | 5 | amp |
| TCGA-09-0369 | 16 | 68324266 | 69304236 | 1.736720391 | 4 | amp |
| TCGA-09-0369 | 16 | 69317928 | 69377546 | 2.6084406 | 5 | amp |
| TCGA-09-0369 | 16 | 69381644 | 69951818 | 1.574161498 | 4 | amp |
| TCGA-09-0369 | 16 | 69959269 | 70012215 | 2.352903981 | 5 | amp |
| TCGA-09-0369 | 16 | 70029971 | 70208955 | 1.611474461 | 4 | amp |
| TCGA-09-0369 | 16 | 70211053 | 70530371 | 2.614309704 | 5 | amp |
| TCGA-09-0369 | 16 | 70531056 | 74425676 | 1.684709752 | 4 | amp |
| TCGA-09-0369 | 16 | 74426966 | 74487270 | 2.744685649 | 5 | amp |
| TCGA-09-0369 | 16 | 74490539 | 81116579 | 1.683310366 | 4 | amp |
| TCGA-09-0369 | 16 | 81118013 | 81204733 | 2.572276926 | 5 | amp |
| TCGA-09-0369 | 16 | 81208301 | 83817087 | 1.815479849 | 4 | amp |
| TCGA-09-0369 | 16 | 83828571 | 84070525 | 2.384302166 | 5 | amp |
| TCGA-09-0369 | 16 | 84075517 | 84135426 | 1.565198214 | 4 | amp |
| TCGA-09-0369 | 16 | 84158193 | 84495428 | 2.389029081 | 5 | amp |
| TCGA-09-0369 | 16 | 84495514 | 85011630 | 1.755425339 | 4 | amp |
| TCGA-09-0369 | 16 | 85012782 | 90141940 | 2.762526789 | 5 | amp |
| TCGA-09-0369 | 16 | 90142223 | 90244214 | 1.498328112 | 4 | amp |
| TCGA-09-0369 | 17 | 69410 | 722791 | 1.526742494 | 4 | amp |
| TCGA-09-0369 | 17 | 725556 | 910598 | 5.638124831 | 5 | amp |
| TCGA-09-0369 | 17 | 913885 | 1268372 | 1.751464304 | 4 | amp |
| TCGA-09-0369 | 17 | 1303282 | 1417279 | 2.601322735 | 5 | amp |
| TCGA-09-0369 | 17 | 1424856 | 1481640 | 1.928754837 | 4 | amp |
| TCGA-09-0369 | 17 | 1486443 | 1560058 | 2.87229257 | 5 | amp |
| TCGA-09-0369 | 17 | 1561490 | 1582235 | 1.83677509 | 4 | amp |
| TCGA-09-0369 | 17 | 1582255 | 1657861 | 2.318163142 | 5 | amp |
| TCGA-09-0369 | 17 | 1673094 | 1798405 | 1.474095843 | 4 | amp |

| | | | | | | |
|---|---|---|---|---|---|---|
| TCGA-09-0369 | 17 | 1839785 | 1946448 | 2.512569876 | 5 | amp |
| TCGA-09-0369 | 17 | 1959561 | 2279612 | 1.591697386 | 4 | amp |
| TCGA-09-0369 | 17 | 2279977 | 2310530 | 3.140220638 | 5 | amp |
| TCGA-09-0369 | 17 | 2317666 | 2966911 | 1.521106544 | 4 | amp |
| TCGA-09-0369 | 17 | 2995340 | 3366090 | 0.901047924 | 3 | amp |
| TCGA-09-0369 | 17 | 3370663 | 3631490 | 1.814169705 | 4 | amp |
| TCGA-09-0369 | 17 | 3632750 | 3643195 | 2.484842093 | 5 | amp |
| TCGA-09-0369 | 17 | 3646696 | 3779736 | 1.722180589 | 4 | amp |
| TCGA-09-0369 | 17 | 3785517 | 3846082 | 2.676433604 | 5 | amp |
| TCGA-09-0369 | 17 | 3846600 | 3913094 | 1.749828104 | 4 | amp |
| TCGA-09-0369 | 17 | 3916734 | 4085681 | 0.940295832 | 3 | amp |
| TCGA-09-0369 | 17 | 4086619 | 4446513 | 1.648124776 | 4 | amp |
| TCGA-09-0369 | 17 | 4451731 | 4500662 | 2.31930678 | 5 | amp |
| TCGA-09-0369 | 17 | 4510568 | 4689349 | 1.78860208 | 4 | amp |
| TCGA-09-0369 | 17 | 4689404 | 4764116 | 2.962496888 | 5 | amp |
| TCGA-09-0369 | 17 | 4778345 | 4841738 | 1.978428239 | 4 | amp |
| TCGA-09-0369 | 17 | 4841761 | 4858600 | 2.61462544 | 5 | amp |
| TCGA-09-0369 | 17 | 4858650 | 4873408 | 1.958299182 | 4 | amp |
| TCGA-09-0369 | 17 | 4873582 | 4936477 | 2.589217633 | 5 | amp |
| TCGA-09-0369 | 17 | 4936497 | 5138099 | 1.532448805 | 4 | amp |
| TCGA-09-0369 | 17 | 5211932 | 5320037 | 0.853838425 | 3 | amp |
| TCGA-09-0369 | 17 | 5322641 | 6441414 | 1.470504458 | 4 | amp |
| TCGA-09-0369 | 17 | 6482975 | 6538436 | 0.803592433 | 3 | amp |
| TCGA-09-0369 | 17 | 6545053 | 7012261 | 1.581031807 | 4 | amp |
| TCGA-09-0369 | 17 | 7017377 | 7127573 | 2.517766 | 5 | amp |
| TCGA-09-0369 | 17 | 7128075 | 7141023 | 1.961316031 | 4 | amp |
| TCGA-09-0369 | 17 | 7144145 | 7162254 | 2.410684897 | 5 | amp |
| TCGA-09-0369 | 17 | 7162856 | 7218036 | 1.948541796 | 4 | amp |
| TCGA-09-0369 | 17 | 7219393 | 7247287 | 2.67173835 | 5 | amp |
| TCGA-09-0369 | 17 | 7249498 | 7307052 | 1.650856104 | 4 | amp |
| TCGA-09-0369 | 17 | 7315440 | 7366338 | 3.00628723 | 5 | amp |
| TCGA-09-0369 | 17 | 7366410 | 7454337 | 1.427158779 | 4 | amp |
| TCGA-09-0369 | 17 | 7460006 | 7477661 | 2.454583182 | 5 | amp |
| TCGA-09-0369 | 17 | 7477809 | 7490926 | 1.914473213 | 4 | amp |
| TCGA-09-0369 | 17 | 7491629 | 7576700 | 2.467831732 | 5 | amp |
| TCGA-09-0369 | 17 | 7576799 | 7592650 | 1.683078315 | 4 | amp |
| TCGA-09-0369 | 17 | 7592903 | 7606172 | 2.553319856 | 5 | amp |
| TCGA-09-0369 | 17 | 7606227 | 7728069 | 1.590467566 | 4 | amp |
| TCGA-09-0369 | 17 | 7733593 | 7749298 | 2.38393069 | 5 | amp |
| TCGA-09-0369 | 17 | 7749673 | 7754739 | 1.383774124 | 4 | amp |
| TCGA-09-0369 | 17 | 7754771 | 7807345 | 2.674244544 | 5 | amp |
| TCGA-09-0369 | 17 | 7807728 | 7827642 | 1.767116937 | 4 | amp |
| TCGA-09-0369 | 17 | 7827715 | 7835157 | 2.340415287 | 5 | amp |
| TCGA-09-0369 | 17 | 7836352 | 8018435 | 1.971812747 | 4 | amp |
| TCGA-09-0369 | 17 | 8018905 | 8050327 | 2.332912615 | 5 | amp |
| TCGA-09-0369 | 17 | 8050513 | 8195933 | 1.676781301 | 4 | amp |
| TCGA-09-0369 | 17 | 8197667 | 8243836 | 3.02727758 | 5 | amp |
| TCGA-09-0369 | 17 | 8248577 | 10222541 | 1.601508433 | 4 | amp |
| TCGA-09-0369 | 17 | 10223454 | 10544705 | 0.817482797 | 3 | amp |
| TCGA-09-0369 | 17 | 10545501 | 11532937 | 1.545871083 | 4 | amp |

| | | | | | | |
|---|---|---|---|---|---|---|
| TCGA-09-0369 | 17 | 11535841 | 12847005 | 0.979759091 | 3 | amp |
| TCGA-09-0369 | 17 | 12847329 | 15907232 | 1.563920008 | 4 | amp |
| TCGA-09-0369 | 17 | 15907455 | 16090084 | 0.926096378 | 3 | amp |
| TCGA-09-0369 | 17 | 16097769 | 17480347 | 1.633517253 | 4 | amp |
| TCGA-09-0369 | 17 | 17488797 | 17764887 | 2.773153484 | 5 | amp |
| TCGA-09-0369 | 17 | 17765953 | 18010570 | 1.897509638 | 4 | amp |
| TCGA-09-0369 | 17 | 18028453 | 18040058 | 2.960664631 | 5 | amp |
| TCGA-09-0369 | 17 | 18040885 | 18045145 | 1.784503182 | 4 | amp |
| TCGA-09-0369 | 17 | 18045360 | 18098368 | 2.319042188 | 5 | amp |
| TCGA-09-0369 | 17 | 18110056 | 18178324 | 1.95179369 | 4 | amp |
| TCGA-09-0369 | 17 | 18180969 | 18252028 | 2.465623958 | 5 | amp |
| TCGA-09-0369 | 17 | 18256908 | 18922939 | 1.524051864 | 4 | amp |
| TCGA-09-0369 | 17 | 18922983 | 19093526 | 2.389014471 | 5 | amp |
| TCGA-09-0369 | 17 | 19186429 | 19232231 | 1.588725259 | 4 | amp |
| TCGA-09-0369 | 17 | 19232826 | 19317001 | 2.390118274 | 5 | amp |
| TCGA-09-0369 | 17 | 19317372 | 19689444 | 1.647588514 | 4 | amp |
| TCGA-09-0369 | 17 | 19698896 | 20334237 | 1.084765533 | 3 | amp |
| TCGA-09-0369 | 17 | 20353238 | 20602223 | 2.351550779 | 5 | amp |
| TCGA-09-0369 | 17 | 20619869 | 25924497 | 1.635150141 | 4 | amp |
| TCGA-09-0369 | 17 | 25928346 | 25944507 | 2.705581561 | 5 | amp |
| TCGA-09-0369 | 17 | 25948897 | 26657571 | 1.887196578 | 4 | amp |
| TCGA-09-0369 | 17 | 26658797 | 26723104 | 2.466049288 | 5 | amp |
| TCGA-09-0369 | 17 | 26723120 | 26823656 | 1.860243837 | 4 | amp |
| TCGA-09-0369 | 17 | 26824051 | 26882093 | 2.505811724 | 5 | amp |
| TCGA-09-0369 | 17 | 26883173 | 27030894 | 1.65850847 | 4 | amp |
| TCGA-09-0369 | 17 | 27030971 | 27065047 | 3.937131666 | 5 | amp |
| TCGA-09-0369 | 17 | 27065067 | 27188653 | 1.712284859 | 4 | amp |
| TCGA-09-0369 | 17 | 27207507 | 27235909 | 2.514109908 | 5 | amp |
| TCGA-09-0369 | 17 | 27237195 | 27383377 | 1.733187491 | 4 | amp |
| TCGA-09-0369 | 17 | 27400833 | 27442140 | 2.706515396 | 5 | amp |
| TCGA-09-0369 | 17 | 27442353 | 27870049 | 1.377608806 | 4 | amp |
| TCGA-09-0369 | 17 | 27889511 | 27939332 | 2.445393679 | 5 | amp |
| TCGA-09-0369 | 17 | 27943642 | 27948460 | 1.609780267 | 4 | amp |
| TCGA-09-0369 | 17 | 27957823 | 29226645 | 0.985982268 | 3 | amp |
| TCGA-09-0369 | 17 | 29227355 | 29324364 | 1.756687592 | 4 | amp |
| TCGA-09-0369 | 17 | 29325614 | 29665159 | 0.730965855 | 3 | amp |
| TCGA-09-0369 | 17 | 29665712 | 30503067 | 1.339407336 | 4 | amp |
| TCGA-09-0369 | 17 | 30503132 | 30551813 | 0.814227163 | 3 | amp |
| TCGA-09-0369 | 17 | 30594872 | 30688008 | 1.883897049 | 4 | amp |
| TCGA-09-0369 | 17 | 30688450 | 31107817 | 0.968845036 | 3 | amp |
| TCGA-09-0369 | 17 | 31260137 | 32904732 | 1.297563692 | 4 | amp |
| TCGA-09-0369 | 17 | 32905824 | 32965263 | 2.604536371 | 5 | amp |
| TCGA-09-0369 | 17 | 33255041 | 33286697 | 0.559548012 | 1 | loss |
| TCGA-09-0369 | 17 | 33288193 | 33348834 | 1.661258361 | 4 | amp |
| TCGA-09-0369 | 17 | 33353362 | 33445698 | 2.361274694 | 5 | amp |
| TCGA-09-0369 | 17 | 33446070 | 33463568 | 1.765106737 | 4 | amp |
| TCGA-09-0369 | 17 | 33463965 | 33480016 | 2.921402299 | 5 | amp |
| TCGA-09-0369 | 17 | 33481556 | 33592918 | 1.782676209 | 4 | amp |
| TCGA-09-0369 | 17 | 33679346 | 34037355 | 0.974788095 | 3 | amp |
| TCGA-09-0369 | 17 | 34044170 | 34067609 | 1.685758167 | 4 | amp |

| | | | | | | |
|---|---|---|---|---|---|---|
| TCGA-09-0369 | 17 | 34071977 | 34095443 | 3.015175187 | 5 | amp |
| TCGA-09-0369 | 17 | 34100176 | 34160987 | 1.550161341 | 4 | amp |
| TCGA-09-0369 | 17 | 34161525 | 34252699 | 0.988261634 | 3 | amp |
| TCGA-09-0369 | 17 | 34257019 | 34859048 | 1.627694535 | 4 | amp |
| TCGA-09-0369 | 17 | 34860953 | 34864997 | 2.488193407 | 5 | amp |
| TCGA-09-0369 | 17 | 34869159 | 35311258 | 1.487960703 | 4 | amp |
| TCGA-09-0369 | 17 | 35343912 | 36287227 | 0.877705468 | 3 | amp |
| TCGA-09-0369 | 17 | 36287649 | 36486509 | 1.460279386 | 4 | amp |
| TCGA-09-0369 | 17 | 36489056 | 36499695 | 2.541065004 | 5 | amp |
| TCGA-09-0369 | 17 | 36517565 | 37373437 | 1.53505546 | 4 | amp |
| TCGA-09-0369 | 17 | 37417676 | 37687572 | 1.052718471 | 3 | amp |
| TCGA-09-0369 | 17 | 37762158 | 37883281 | 1.647355358 | 4 | amp |
| TCGA-09-0369 | 17 | 37883493 | 37903196 | 2.317501319 | 5 | amp |
| TCGA-09-0369 | 17 | 37922034 | 38152633 | 1.306853337 | 4 | amp |
| TCGA-09-0369 | 17 | 38153517 | 38175959 | 2.515641915 | 5 | amp |
| TCGA-09-0369 | 17 | 38178167 | 38209906 | 1.624951028 | 4 | amp |
| TCGA-09-0369 | 17 | 38230707 | 38253674 | 2.762642658 | 5 | amp |
| TCGA-09-0369 | 17 | 38256271 | 38511713 | 1.576444626 | 4 | amp |
| TCGA-09-0369 | 17 | 38545714 | 39616546 | 0.923288293 | 3 | amp |
| TCGA-09-0369 | 17 | 39619071 | 39974826 | 1.676401364 | 4 | amp |
| TCGA-09-0369 | 17 | 39975406 | 39988746 | 2.52961545 | 5 | amp |
| TCGA-09-0369 | 17 | 39991284 | 40173702 | 1.656374044 | 4 | amp |
| TCGA-09-0369 | 17 | 40173832 | 40263946 | 2.656645198 | 5 | amp |
| TCGA-09-0369 | 17 | 40265613 | 40477089 | 1.673347969 | 4 | amp |
| TCGA-09-0369 | 17 | 40478085 | 40666512 | 0.981474266 | 3 | amp |
| TCGA-09-0369 | 17 | 40673001 | 40947568 | 1.6847101 | 4 | amp |
| TCGA-09-0369 | 17 | 40947576 | 40951289 | 3.322402103 | 5 | amp |
| TCGA-09-0369 | 17 | 40955615 | 41566919 | 1.295812896 | 4 | amp |
| TCGA-09-0369 | 17 | 41567704 | 41599624 | 0.884476096 | 3 | amp |
| TCGA-09-0369 | 17 | 41600955 | 43102126 | 1.692449546 | 4 | amp |
| TCGA-09-0369 | 17 | 43107413 | 43318037 | 2.483816975 | 5 | amp |
| TCGA-09-0369 | 17 | 43318486 | 43322932 | 1.32850195 | 4 | amp |
| TCGA-09-0369 | 17 | 43322959 | 43344095 | 2.377958164 | 5 | amp |
| TCGA-09-0369 | 17 | 43344769 | 43480191 | 1.872256878 | 4 | amp |
| TCGA-09-0369 | 17 | 43480939 | 44110857 | 2.842889282 | 5 | amp |
| TCGA-09-0369 | 17 | 44111438 | 45758074 | 1.509808016 | 4 | amp |
| TCGA-09-0369 | 17 | 45759679 | 45785878 | 2.470796688 | 5 | amp |
| TCGA-09-0369 | 17 | 45787807 | 45891256 | 1.482205967 | 4 | amp |
| TCGA-09-0369 | 17 | 45892499 | 45916384 | 2.50921734 | 5 | amp |
| TCGA-09-0369 | 17 | 45916759 | 46023410 | 1.829650172 | 4 | amp |
| TCGA-09-0369 | 17 | 46023663 | 46137043 | 2.719457864 | 5 | amp |
| TCGA-09-0369 | 17 | 46148748 | 46474154 | 1.352226823 | 4 | amp |
| TCGA-09-0369 | 17 | 46606892 | 46700499 | 2.669677616 | 5 | amp |
| TCGA-09-0369 | 17 | 46709826 | 46973133 | 1.56106486 | 4 | amp |
| TCGA-09-0369 | 17 | 46988145 | 47008025 | 2.494406446 | 5 | amp |
| TCGA-09-0369 | 17 | 47008951 | 47904873 | 1.772210096 | 4 | amp |
| TCGA-09-0369 | 17 | 47915959 | 48185791 | 3.371061456 | 5 | amp |
| TCGA-09-0369 | 17 | 48185969 | 48195727 | 1.427854215 | 4 | amp |
| TCGA-09-0369 | 17 | 48202211 | 48274064 | 2.651704021 | 5 | amp |
| TCGA-09-0369 | 17 | 48274337 | 48457887 | 1.744418805 | 4 | amp |

| | | | | | | |
|---|---|---|---|---|---|---|
| TCGA-09-0369 | 17 | 48458091 | 48650244 | 2.745629177 | 5 | amp |
| TCGA-09-0369 | 17 | 48655496 | 48678593 | 1.683491938 | 4 | amp |
| TCGA-09-0369 | 17 | 48680154 | 48733400 | 2.66128113 | 5 | amp |
| TCGA-09-0369 | 17 | 48733975 | 54912628 | 1.377646633 | 4 | amp |
| TCGA-09-0369 | 17 | 54921339 | 55075862 | 2.494553669 | 5 | amp |
| TCGA-09-0369 | 17 | 55078164 | 55083624 | 1.215421793 | 3 | amp |
| TCGA-09-0369 | 17 | 55182752 | 56059325 | 2.919915854 | 5 | amp |
| TCGA-09-0369 | 17 | 56060050 | 56247958 | 1.874852245 | 4 | amp |
| TCGA-09-0369 | 17 | 56270155 | 56293646 | 2.623562321 | 5 | amp |
| TCGA-09-0369 | 17 | 56294001 | 56343720 | 1.987616799 | 4 | amp |
| TCGA-09-0369 | 17 | 56344648 | 56448452 | 2.885107162 | 5 | amp |
| TCGA-09-0369 | 17 | 56492602 | 56540703 | 1.400459103 | 4 | amp |
| TCGA-09-0369 | 17 | 56544232 | 56621606 | 4.151497343 | 5 | amp |
| TCGA-09-0369 | 17 | 56634329 | 57262930 | 1.704496219 | 4 | amp |
| TCGA-09-0369 | 17 | 57270805 | 57292384 | 2.672095452 | 5 | amp |
| TCGA-09-0369 | 17 | 57297892 | 57941236 | 1.438679458 | 4 | amp |
| TCGA-09-0369 | 17 | 57943919 | 57968397 | 2.616406422 | 5 | amp |
| TCGA-09-0369 | 17 | 57987887 | 58086467 | 1.702343062 | 4 | amp |
| TCGA-09-0369 | 17 | 58087346 | 58235845 | 2.582197155 | 5 | amp |
| TCGA-09-0369 | 17 | 58256582 | 59445891 | 1.519984459 | 4 | amp |
| TCGA-09-0369 | 17 | 59457839 | 59560928 | 3.430662027 | 5 | amp |
| TCGA-09-0369 | 17 | 59667857 | 60343047 | 1.352678059 | 4 | amp |
| TCGA-09-0369 | 17 | 60343926 | 60351500 | 2.745196248 | 5 | amp |
| TCGA-09-0369 | 17 | 60360048 | 60678216 | 1.611564576 | 4 | amp |
| TCGA-09-0369 | 17 | 60679351 | 60879111 | 2.875471712 | 5 | amp |
| TCGA-09-0369 | 17 | 61021556 | 61514927 | 1.515915094 | 4 | amp |
| TCGA-09-0369 | 17 | 61557081 | 61824340 | 2.850364933 | 5 | amp |
| TCGA-09-0369 | 17 | 61829263 | 61902818 | 1.717061845 | 4 | amp |
| TCGA-09-0369 | 17 | 61902885 | 62023038 | 3.348813494 | 5 | amp |
| TCGA-09-0369 | 17 | 62024345 | 62272474 | 1.841385084 | 4 | amp |
| TCGA-09-0369 | 17 | 62289897 | 62463767 | 2.55726038 | 5 | amp |
| TCGA-09-0369 | 17 | 62473902 | 62496482 | 1.895633117 | 4 | amp |
| TCGA-09-0369 | 17 | 62496658 | 62500997 | 2.737733734 | 5 | amp |
| TCGA-09-0369 | 17 | 62504653 | 62797056 | 1.57400194 | 4 | amp |
| TCGA-09-0369 | 17 | 62801022 | 62865308 | 2.607626472 | 5 | amp |
| TCGA-09-0369 | 17 | 62882183 | 64728994 | 1.710096153 | 4 | amp |
| TCGA-09-0369 | 17 | 64731573 | 65052393 | 2.438768999 | 5 | amp |
| TCGA-09-0369 | 17 | 65074354 | 65989268 | 1.444819112 | 4 | amp |
| TCGA-09-0369 | 17 | 66033225 | 66274465 | 2.850552691 | 5 | amp |
| TCGA-09-0369 | 17 | 66303623 | 66422325 | 1.959825976 | 4 | amp |
| TCGA-09-0369 | 17 | 66423214 | 66520247 | 2.345564471 | 5 | amp |
| TCGA-09-0369 | 17 | 66521015 | 66914376 | 1.504225082 | 4 | amp |
| TCGA-09-0369 | 17 | 66915409 | 67152125 | 1.042729724 | 3 | amp |
| TCGA-09-0369 | 17 | 67152958 | 71166613 | 1.432900242 | 4 | amp |
| TCGA-09-0369 | 17 | 71192557 | 74161664 | 3.292546402 | 5 | amp |
| TCGA-09-0369 | 17 | 74162489 | 74273437 | 1.933596361 | 4 | amp |
| TCGA-09-0369 | 17 | 74274019 | 74309996 | 3.23502846 | 5 | amp |
| TCGA-09-0369 | 17 | 74324752 | 74328549 | 1.579177225 | 4 | amp |
| TCGA-09-0369 | 17 | 74340694 | 74465029 | 2.522307374 | 5 | amp |
| TCGA-09-0369 | 17 | 74465181 | 74470908 | 1.900208886 | 4 | amp |

| | | | | | | |
|---|---|---|---|---|---|---|
| TCGA-09-0369 | 17 | 74471107 | 74750208 | 3.267943648 | 5 | amp |
| TCGA-09-0369 | 17 | 74763425 | 74774471 | 1.598977111 | 4 | amp |
| TCGA-09-0369 | 17 | 74868898 | 75139769 | 3.10517258 | 5 | amp |
| TCGA-09-0369 | 17 | 75186839 | 75196780 | 1.507722656 | 4 | amp |
| TCGA-09-0369 | 17 | 75199605 | 76061030 | 2.414175328 | 5 | amp |
| TCGA-09-0369 | 17 | 76063800 | 76094640 | 1.742114491 | 4 | amp |
| TCGA-09-0369 | 17 | 76099444 | 76794687 | 3.474664944 | 5 | amp |
| TCGA-09-0369 | 17 | 76794987 | 76802422 | 1.82266984 | 4 | amp |
| TCGA-09-0369 | 17 | 76802989 | 78343677 | 2.883975244 | 5 | amp |
| TCGA-09-0369 | 17 | 78345669 | 78359024 | 1.949117582 | 4 | amp |
| TCGA-09-0369 | 17 | 78359319 | 78363189 | 2.983951915 | 5 | amp |
| TCGA-09-0369 | 17 | 78363612 | 78367341 | 1.481200897 | 4 | amp |
| TCGA-09-0369 | 17 | 78406896 | 78797070 | 2.62459413 | 5 | amp |
| TCGA-09-0369 | 17 | 78811699 | 78854311 | 1.709269055 | 4 | amp |
| TCGA-09-0369 | 17 | 78857191 | 81188237 | 3.223293023 | 5 | amp |
| TCGA-09-0369 | 18 | 47273 | 179044 | 1.524585512 | 4 | amp |
| TCGA-09-0369 | 18 | 180227 | 216679 | 0.7526292 | 3 | amp |
| TCGA-09-0369 | 18 | 218867 | 724647 | 1.443398512 | 4 | amp |
| TCGA-09-0369 | 18 | 732779 | 2778261 | 1.061877182 | 3 | amp |
| TCGA-09-0369 | 18 | 2784413 | 6171943 | 1.401054136 | 4 | amp |
| TCGA-09-0369 | 18 | 6213143 | 6462950 | 0.892730304 | 3 | amp |
| TCGA-09-0369 | 18 | 6837290 | 7232069 | 1.333625507 | 4 | amp |
| TCGA-09-0369 | 18 | 7774087 | 8376238 | 1.068732219 | 3 | amp |
| TCGA-09-0369 | 18 | 8376407 | 8384725 | 2.750632738 | 5 | amp |
| TCGA-09-0369 | 18 | 8387031 | 9134290 | 1.668585929 | 4 | amp |
| TCGA-09-0369 | 18 | 9182413 | 9360079 | 0.899280684 | 3 | amp |
| TCGA-09-0369 | 18 | 9396230 | 10546421 | 1.413970395 | 4 | amp |
| TCGA-09-0369 | 18 | 10548275 | 10681763 | 0.873464369 | 3 | amp |
| TCGA-09-0369 | 18 | 10689606 | 12363877 | 1.774002182 | 4 | amp |
| TCGA-09-0369 | 18 | 12366919 | 12429435 | 2.324592443 | 5 | amp |
| TCGA-09-0369 | 18 | 12449585 | 12814397 | 1.328851023 | 4 | amp |
| TCGA-09-0369 | 18 | 12817109 | 13073215 | 1.097198414 | 3 | amp |
| TCGA-09-0369 | 18 | 13086965 | 14543184 | 1.427272874 | 4 | amp |
| TCGA-09-0369 | 18 | 14613239 | 19148057 | 0.995392178 | 3 | amp |
| TCGA-09-0369 | 18 | 19153259 | 19353696 | 1.418374372 | 4 | amp |
| TCGA-09-0369 | 18 | 19358036 | 20573866 | 1.115474559 | 3 | amp |
| TCGA-09-0369 | 18 | 20576320 | 22932159 | 1.438270292 | 4 | amp |
| TCGA-09-0369 | 18 | 23637509 | 28581707 | 1.177867364 | 3 | amp |
| TCGA-09-0369 | 18 | 28584099 | 29771853 | 1.730973822 | 4 | amp |
| TCGA-09-0369 | 18 | 29772570 | 33740990 | 1.154366021 | 3 | amp |
| TCGA-09-0369 | 18 | 33744413 | 34359511 | 1.347647927 | 4 | amp |
| TCGA-09-0369 | 18 | 34376680 | 44015365 | 1.13121882 | 3 | amp |
| TCGA-09-0369 | 18 | 44027470 | 44589753 | 1.407032134 | 4 | amp |
| TCGA-09-0369 | 18 | 44593380 | 44656716 | 0.929021233 | 3 | amp |
| TCGA-09-0369 | 18 | 44660850 | 45423129 | 1.421264051 | 4 | amp |
| TCGA-09-0369 | 18 | 45556088 | 46474840 | 2.175799966 | 5 | amp |
| TCGA-09-0369 | 18 | 46570349 | 46956814 | 0.955308628 | 3 | amp |
| TCGA-09-0369 | 18 | 47008657 | 47018126 | 1.420874638 | 4 | amp |
| TCGA-09-0369 | 18 | 47088636 | 47310318 | 2.313850735 | 5 | amp |
| TCGA-09-0369 | 18 | 47311523 | 48422304 | 1.554161677 | 4 | amp |

| | | | | | | |
|---|---|---|---|---|---|---|
| TCGA-09-0369 | 18 | 48434379 | 55274035 | 1.101342633 | 3 | amp |
| TCGA-09-0369 | 18 | 55274315 | 56182328 | 1.377232251 | 4 | amp |
| TCGA-09-0369 | 18 | 56184096 | 61602513 | 1.123916577 | 3 | amp |
| TCGA-09-0369 | 18 | 61620665 | 63430303 | 1.589309965 | 4 | amp |
| TCGA-09-0369 | 18 | 63476906 | 70461678 | 1.051232242 | 3 | amp |
| TCGA-09-0369 | 18 | 70502461 | 77137434 | 1.46827562 | 4 | amp |
| TCGA-09-0369 | 18 | 77170035 | 77960823 | 2.454583586 | 5 | amp |
| TCGA-09-0369 | 19 | 71882 | 1110935 | 1.557955657 | 4 | amp |
| TCGA-09-0369 | 19 | 1218397 | 1358487 | 2.379368454 | 5 | amp |
| TCGA-09-0369 | 19 | 1360102 | 4844862 | 1.476989784 | 4 | amp |
| TCGA-09-0369 | 19 | 4847682 | 4930927 | 2.343554389 | 5 | amp |
| TCGA-09-0369 | 19 | 4932740 | 5208444 | 1.851761416 | 4 | amp |
| TCGA-09-0369 | 19 | 5210422 | 5592947 | 2.529177678 | 5 | amp |
| TCGA-09-0369 | 19 | 5595289 | 5694600 | 1.849918587 | 4 | amp |
| TCGA-09-0369 | 19 | 5700748 | 5737242 | 2.626199658 | 5 | amp |
| TCGA-09-0369 | 19 | 5739333 | 6430215 | 1.641709932 | 4 | amp |
| TCGA-09-0369 | 19 | 6432017 | 6444356 | 2.65774947 | 5 | amp |
| TCGA-09-0369 | 19 | 6452286 | 6735733 | 1.569269563 | 4 | amp |
| TCGA-09-0369 | 19 | 6735883 | 6763784 | 2.270670991 | 5 | amp |
| TCGA-09-0369 | 19 | 6772770 | 7593153 | 1.397756392 | 4 | amp |
| TCGA-09-0369 | 19 | 7593475 | 7606971 | 2.60174644 | 5 | amp |
| TCGA-09-0369 | 19 | 7607580 | 7625711 | 1.579342886 | 4 | amp |
| TCGA-09-0369 | 19 | 7625887 | 7744051 | 2.329250981 | 5 | amp |
| TCGA-09-0369 | 19 | 7754017 | 8056733 | 1.74084725 | 4 | amp |
| TCGA-09-0369 | 19 | 8122745 | 8154870 | 2.440997702 | 5 | amp |
| TCGA-09-0369 | 19 | 8154908 | 8322016 | 1.731357152 | 4 | amp |
| TCGA-09-0369 | 19 | 8322725 | 8503470 | 2.550605588 | 5 | amp |
| TCGA-09-0369 | 19 | 8520253 | 8551993 | 1.365057257 | 4 | amp |
| TCGA-09-0369 | 19 | 8553536 | 8668809 | 2.513528754 | 5 | amp |
| TCGA-09-0369 | 19 | 8807735 | 10473155 | 1.456013875 | 4 | amp |
| TCGA-09-0369 | 19 | 10475218 | 10504145 | 2.511005031 | 5 | amp |
| TCGA-09-0369 | 19 | 10505607 | 10665888 | 1.615093835 | 4 | amp |
| TCGA-09-0369 | 19 | 10665926 | 10754067 | 2.722305107 | 5 | amp |
| TCGA-09-0369 | 19 | 10781222 | 10812980 | 1.51292498 | 4 | amp |
| TCGA-09-0369 | 19 | 10817956 | 10823958 | 2.503872649 | 5 | amp |
| TCGA-09-0369 | 19 | 10870360 | 10886600 | 1.36459314 | 4 | amp |
| TCGA-09-0369 | 19 | 10887752 | 10939947 | 2.720427887 | 5 | amp |
| TCGA-09-0369 | 19 | 10940718 | 11211047 | 1.684701687 | 4 | amp |
| TCGA-09-0369 | 19 | 11213290 | 11339723 | 2.345905242 | 5 | amp |
| TCGA-09-0369 | 19 | 11343840 | 11354429 | 1.204418946 | 3 | amp |
| TCGA-09-0369 | 19 | 11354457 | 11448081 | 2.428381379 | 5 | amp |
| TCGA-09-0369 | 19 | 11453433 | 11527374 | 1.947818634 | 4 | amp |
| TCGA-09-0369 | 19 | 11527471 | 11553385 | 2.622112042 | 5 | amp |
| TCGA-09-0369 | 19 | 11559339 | 12758454 | 1.449187254 | 4 | amp |
| TCGA-09-0369 | 19 | 12759899 | 12775822 | 2.38701799 | 5 | amp |
| TCGA-09-0369 | 19 | 12776102 | 12792596 | 1.567663968 | 4 | amp |
| TCGA-09-0369 | 19 | 12799976 | 12813744 | 3.087529027 | 5 | amp |
| TCGA-09-0369 | 19 | 12814230 | 12830172 | 1.631512737 | 4 | amp |
| TCGA-09-0369 | 19 | 12841768 | 12865887 | 2.51717715 | 5 | amp |
| TCGA-09-0369 | 19 | 12866141 | 12867151 | 1.619297612 | 4 | amp |

| | | | | | | |
|---|---|---|---|---|---|---|
| TCGA-09-0369 | 19 | 12874020 | 12924330 | 2.551637205 | 5 | amp |
| TCGA-09-0369 | 19 | 12939465 | 12995906 | 1.753476018 | 4 | amp |
| TCGA-09-0369 | 19 | 12997820 | 13007246 | 3.744532929 | 5 | amp |
| TCGA-09-0369 | 19 | 13007715 | 13063704 | 1.828580546 | 4 | amp |
| TCGA-09-0369 | 19 | 13063716 | 13226306 | 2.7865471 | 5 | amp |
| TCGA-09-0369 | 19 | 13226360 | 13988620 | 1.623253113 | 4 | amp |
| TCGA-09-0369 | 19 | 13991226 | 14040283 | 2.467193999 | 5 | amp |
| TCGA-09-0369 | 19 | 14065110 | 14204641 | 1.760817895 | 4 | amp |
| TCGA-09-0369 | 19 | 14208083 | 14247273 | 2.332316497 | 5 | amp |
| TCGA-09-0369 | 19 | 14262893 | 14578793 | 1.686916909 | 4 | amp |
| TCGA-09-0369 | 19 | 14580120 | 14589596 | 2.588966157 | 5 | amp |
| TCGA-09-0369 | 19 | 14591056 | 15224812 | 1.379904805 | 4 | amp |
| TCGA-09-0369 | 19 | 15226031 | 15230337 | 2.872986873 | 5 | amp |
| TCGA-09-0369 | 19 | 15233469 | 15483213 | 1.671907505 | 4 | amp |
| TCGA-09-0369 | 19 | 15485369 | 15572127 | 2.342968914 | 5 | amp |
| TCGA-09-0369 | 19 | 15579458 | 16212174 | 1.600211035 | 4 | amp |
| TCGA-09-0369 | 19 | 16222623 | 16259742 | 2.366734974 | 5 | amp |
| TCGA-09-0369 | 19 | 16263346 | 16553069 | 1.665787321 | 4 | amp |
| TCGA-09-0369 | 19 | 16589893 | 16631366 | 2.326542106 | 5 | amp |
| TCGA-09-0369 | 19 | 16631589 | 17294717 | 1.522917123 | 4 | amp |
| TCGA-09-0369 | 19 | 17295614 | 17411975 | 2.508086139 | 5 | amp |
| TCGA-09-0369 | 19 | 17420424 | 17516421 | 1.965472633 | 4 | amp |
| TCGA-09-0369 | 19 | 17531080 | 17611722 | 2.308463787 | 5 | amp |
| TCGA-09-0369 | 19 | 17612004 | 17760416 | 1.901991603 | 4 | amp |
| TCGA-09-0369 | 19 | 17763402 | 17885936 | 2.571857502 | 5 | amp |
| TCGA-09-0369 | 19 | 17886203 | 17985566 | 1.778856931 | 4 | amp |
| TCGA-09-0369 | 19 | 17988451 | 18096311 | 3.031406715 | 5 | amp |
| TCGA-09-0369 | 19 | 18100468 | 18274255 | 1.955566908 | 4 | amp |
| TCGA-09-0369 | 19 | 18276890 | 18327747 | 2.295852456 | 5 | amp |
| TCGA-09-0369 | 19 | 18328918 | 18583695 | 1.917413364 | 4 | amp |
| TCGA-09-0369 | 19 | 18643389 | 18895206 | 2.34139727 | 5 | amp |
| TCGA-09-0369 | 19 | 18895653 | 19030214 | 1.911956283 | 4 | amp |
| TCGA-09-0369 | 19 | 19031337 | 19038678 | 2.436104056 | 5 | amp |
| TCGA-09-0369 | 19 | 19038719 | 19215794 | 1.644224775 | 4 | amp |
| TCGA-09-0369 | 19 | 19216985 | 19375697 | 2.359217607 | 5 | amp |
| TCGA-09-0369 | 19 | 19377208 | 30308500 | 1.260001088 | 4 | amp |
| TCGA-09-0369 | 19 | 30311531 | 30314742 | 2.822591003 | 5 | amp |
| TCGA-09-0369 | 19 | 30462056 | 33110489 | 1.376231875 | 4 | amp |
| TCGA-09-0369 | 19 | 33113276 | 33359456 | 2.545848439 | 5 | amp |
| TCGA-09-0369 | 19 | 33370025 | 33451004 | 1.539863408 | 4 | amp |
| TCGA-09-0369 | 19 | 33457258 | 33465161 | 2.463336135 | 5 | amp |
| TCGA-09-0369 | 19 | 33467252 | 33499122 | 1.788202784 | 4 | amp |
| TCGA-09-0369 | 19 | 33502526 | 33579227 | 2.513970811 | 5 | amp |
| TCGA-09-0369 | 19 | 33581638 | 33605361 | 1.560396005 | 4 | amp |
| TCGA-09-0369 | 19 | 33608688 | 34297959 | 2.368627436 | 5 | amp |
| TCGA-09-0369 | 19 | 34302641 | 34833383 | 0.968172083 | 3 | amp |
| TCGA-09-0369 | 19 | 34839839 | 34872457 | 2.546705854 | 5 | amp |
| TCGA-09-0369 | 19 | 34884122 | 34960155 | 1.374800108 | 4 | amp |
| TCGA-09-0369 | 19 | 34981271 | 34986701 | 2.757436922 | 5 | amp |
| TCGA-09-0369 | 19 | 35033441 | 35451954 | 1.566148206 | 4 | amp |

| | | | | | | |
|---|---|---|---|---|---|---|
| TCGA-09-0369 | 19 | 35499977 | 35823020 | 2.620766229 | 5 | amp |
| TCGA-09-0369 | 19 | 35823371 | 35832909 | 1.644604306 | 4 | amp |
| TCGA-09-0369 | 19 | 35835689 | 35863321 | 4.751153823 | 5 | amp |
| TCGA-09-0369 | 19 | 35940572 | 35981390 | 1.609991997 | 4 | amp |
| TCGA-09-0369 | 19 | 35989551 | 36036739 | 2.970591351 | 5 | amp |
| TCGA-09-0369 | 19 | 36037397 | 36110707 | 1.897780378 | 4 | amp |
| TCGA-09-0369 | 19 | 36110879 | 36674661 | 2.749696816 | 5 | amp |
| TCGA-09-0369 | 19 | 36685122 | 38579545 | 1.359992924 | 4 | amp |
| TCGA-09-0369 | 19 | 38590575 | 38939457 | 2.754872808 | 5 | amp |
| TCGA-09-0369 | 19 | 38942314 | 38948991 | 1.57229343 | 4 | amp |
| TCGA-09-0369 | 19 | 38949761 | 39104763 | 2.923088265 | 5 | amp |
| TCGA-09-0369 | 19 | 39104818 | 39108545 | 1.714674656 | 4 | amp |
| TCGA-09-0369 | 19 | 39109877 | 39322446 | 2.9386657 | 5 | amp |
| TCGA-09-0369 | 19 | 39327300 | 39328322 | 1.504303335 | 4 | amp |
| TCGA-09-0369 | 19 | 39329017 | 39860769 | 3.046180123 | 5 | amp |
| TCGA-09-0369 | 19 | 39866229 | 39871811 | 1.642620563 | 4 | amp |
| TCGA-09-0369 | 19 | 39873799 | 40023499 | 2.657404321 | 5 | amp |
| TCGA-09-0369 | 19 | 40029973 | 40321228 | 1.300266591 | 4 | amp |
| TCGA-09-0369 | 19 | 40321243 | 40480748 | 2.514640453 | 5 | amp |
| TCGA-09-0369 | 19 | 40485687 | 40589094 | 1.234306553 | 3 | amp |
| TCGA-09-0369 | 19 | 40704221 | 40748637 | 2.780843127 | 5 | amp |
| TCGA-09-0369 | 19 | 40761060 | 40763016 | 1.101121498 | 3 | amp |
| TCGA-09-0369 | 19 | 40771091 | 41332596 | 2.567919862 | 5 | amp |
| TCGA-09-0369 | 19 | 41349671 | 41601084 | 1.49339315 | 4 | amp |
| TCGA-09-0369 | 19 | 41601633 | 41631588 | 2.802852872 | 5 | amp |
| TCGA-09-0369 | 19 | 41700381 | 41707326 | 1.48111675 | 4 | amp |
| TCGA-09-0369 | 19 | 41709298 | 41763585 | 2.742246047 | 5 | amp |
| TCGA-09-0369 | 19 | 41765423 | 41785091 | 1.783303448 | 4 | amp |
| TCGA-09-0369 | 19 | 41787033 | 41945855 | 2.785149547 | 5 | amp |
| TCGA-09-0369 | 19 | 42012013 | 42314962 | 1.709976187 | 4 | amp |
| TCGA-09-0369 | 19 | 42315152 | 42741111 | 2.875421606 | 5 | amp |
| TCGA-09-0369 | 19 | 42744080 | 42794111 | 1.784032619 | 4 | amp |
| TCGA-09-0369 | 19 | 42794311 | 42911970 | 2.61790269 | 5 | amp |
| TCGA-09-0369 | 19 | 42912170 | 43859993 | 1.587819411 | 4 | amp |
| TCGA-09-0369 | 19 | 43860141 | 44131988 | 2.467026953 | 5 | amp |
| TCGA-09-0369 | 19 | 44152988 | 44169659 | 1.495787029 | 4 | amp |
| TCGA-09-0369 | 19 | 44171697 | 44249039 | 2.742277525 | 5 | amp |
| TCGA-09-0369 | 19 | 44251552 | 45164634 | 1.371485001 | 4 | amp |
| TCGA-09-0369 | 19 | 45164952 | 46457240 | 3.038613334 | 5 | amp |
| TCGA-09-0369 | 19 | 46464219 | 46652338 | 1.611975077 | 4 | amp |
| TCGA-09-0369 | 19 | 46663638 | 47349444 | 2.741999775 | 5 | amp |
| TCGA-09-0369 | 19 | 47421912 | 47440712 | 1.1461637 | 3 | amp |
| TCGA-09-0369 | 19 | 47491224 | 47597875 | 2.795702604 | 5 | amp |
| TCGA-09-0369 | 19 | 47646746 | 47712521 | 1.481294002 | 4 | amp |
| TCGA-09-0369 | 19 | 47761568 | 48339695 | 2.778555025 | 5 | amp |
| TCGA-09-0369 | 19 | 48342457 | 48593707 | 1.747490351 | 4 | amp |
| TCGA-09-0369 | 19 | 48598640 | 48647239 | 2.672203099 | 5 | amp |
| TCGA-09-0369 | 19 | 48652998 | 48737757 | 1.681731943 | 4 | amp |
| TCGA-09-0369 | 19 | 48782970 | 49336009 | 2.758769285 | 5 | amp |
| TCGA-09-0369 | 19 | 49337446 | 49341387 | 1.732580613 | 4 | amp |

| | | | | | | |
|---|---|---|---|---|---|---|
| TCGA-09-0369 | 19 | 49342430 | 49644769 | 2.86957457 | 5 | amp |
| TCGA-09-0369 | 19 | 49646023 | 49843606 | 1.887383272 | 4 | amp |
| TCGA-09-0369 | 19 | 49844547 | 49920363 | 2.754749161 | 5 | amp |
| TCGA-09-0369 | 19 | 49920409 | 49938603 | 1.561279391 | 4 | amp |
| TCGA-09-0369 | 19 | 49939721 | 50185544 | 2.942131671 | 5 | amp |
| TCGA-09-0369 | 19 | 50188146 | 50212137 | 1.802911294 | 4 | amp |
| TCGA-09-0369 | 19 | 50213538 | 50266539 | 2.670423158 | 5 | amp |
| TCGA-09-0369 | 19 | 50284987 | 50303435 | 1.776091346 | 4 | amp |
| TCGA-09-0369 | 19 | 50304642 | 50658039 | 2.822764301 | 5 | amp |
| TCGA-09-0369 | 19 | 50661504 | 50666092 | 1.640118364 | 4 | amp |
| TCGA-09-0369 | 19 | 50666185 | 50902744 | 2.660519295 | 5 | amp |
| TCGA-09-0369 | 19 | 50904957 | 50917187 | 1.720876032 | 4 | amp |
| TCGA-09-0369 | 19 | 50918039 | 51217552 | 3.052559615 | 5 | amp |
| TCGA-09-0369 | 19 | 51218891 | 51226848 | 1.632522631 | 4 | amp |
| TCGA-09-0369 | 19 | 51226915 | 51410385 | 2.24569622 | 5 | amp |
| TCGA-09-0369 | 19 | 51411562 | 51466861 | 1.775184541 | 4 | amp |
| TCGA-09-0369 | 19 | 51470382 | 51518859 | 2.842103428 | 5 | amp |
| TCGA-09-0369 | 19 | 51525756 | 51629421 | 1.983263996 | 4 | amp |
| TCGA-09-0369 | 19 | 51630282 | 51650585 | 2.39252754 | 5 | amp |
| TCGA-09-0369 | 19 | 51656260 | 51742977 | 1.67078386 | 4 | amp |
| TCGA-09-0369 | 19 | 51767247 | 51917782 | 2.386699907 | 5 | amp |
| TCGA-09-0369 | 19 | 51918009 | 51920876 | 1.603000442 | 4 | amp |
| TCGA-09-0369 | 19 | 51955581 | 51986620 | 2.495718819 | 5 | amp |
| TCGA-09-0369 | 19 | 51994868 | 52091759 | 1.651696097 | 4 | amp |
| TCGA-09-0369 | 19 | 52115429 | 52132369 | 2.529562857 | 5 | amp |
| TCGA-09-0369 | 19 | 52132569 | 54260099 | 1.517920686 | 4 | amp |
| TCGA-09-0369 | 19 | 54261438 | 54401907 | 2.705367666 | 5 | amp |
| TCGA-09-0369 | 19 | 54403442 | 54406426 | 1.351985539 | 4 | amp |
| TCGA-09-0369 | 19 | 54407882 | 54544366 | 2.664619869 | 5 | amp |
| TCGA-09-0369 | 19 | 54544991 | 54603092 | 1.748677469 | 4 | amp |
| TCGA-09-0369 | 19 | 54606398 | 54783530 | 2.812589591 | 5 | amp |
| TCGA-09-0369 | 19 | 54783577 | 54804020 | 1.827386952 | 4 | amp |
| TCGA-09-0369 | 19 | 54804096 | 54969716 | 2.753887238 | 5 | amp |
| TCGA-09-0369 | 19 | 54973179 | 55420922 | 1.604537986 | 4 | amp |
| TCGA-09-0369 | 19 | 55421341 | 55568158 | 2.770040564 | 5 | amp |
| TCGA-09-0369 | 19 | 55590336 | 55607331 | 1.446981983 | 4 | amp |
| TCGA-09-0369 | 19 | 55607332 | 55658442 | 2.423843889 | 5 | amp |
| TCGA-09-0369 | 19 | 55658449 | 55687552 | 1.870528745 | 4 | amp |
| TCGA-09-0369 | 19 | 55689529 | 56220463 | 2.696836213 | 5 | amp |
| TCGA-09-0369 | 19 | 56228058 | 57059283 | 1.609568674 | 4 | amp |
| TCGA-09-0369 | 19 | 57060258 | 57176880 | 3.976270489 | 5 | amp |
| TCGA-09-0369 | 19 | 57184176 | 57890202 | 1.869175688 | 4 | amp |
| TCGA-09-0369 | 19 | 57901414 | 57929462 | 2.674573391 | 5 | amp |
| TCGA-09-0369 | 19 | 57930965 | 57985815 | 1.729917679 | 4 | amp |
| TCGA-09-0369 | 19 | 57986302 | 59110878 | 4.303018664 | 5 | amp |
| TCGA-09-0369 | 2 | 41527 | 1437414 | 2.059548194 | 4 | amp |
| TCGA-09-0369 | 2 | 1439969 | 7023702 | 2.746870325 | 5 | amp |
| TCGA-09-0369 | 2 | 7027060 | 9499071 | 1.618309917 | 4 | amp |
| TCGA-09-0369 | 2 | 9508540 | 9593155 | 2.542162181 | 5 | amp |
| TCGA-09-0369 | 2 | 9595746 | 10140837 | 1.764718866 | 4 | amp |

| | | | | | | |
|---|---|---|---|---|---|---|
| TCGA-09-0369 | 2 | 10186201 | 10585167 | 2.79028487 | 5 | amp |
| TCGA-09-0369 | 2 | 10712196 | 10863159 | 1.700163856 | 4 | amp |
| TCGA-09-0369 | 2 | 10866601 | 11300845 | 3.649106824 | 5 | amp |
| TCGA-09-0369 | 2 | 11304298 | 11706813 | 1.47052019 | 4 | amp |
| TCGA-09-0369 | 2 | 11716452 | 11932145 | 3.197933971 | 5 | amp |
| TCGA-09-0369 | 2 | 11935513 | 20240831 | 1.612907328 | 4 | amp |
| TCGA-09-0369 | 2 | 20401986 | 20451531 | 3.114047538 | 5 | amp |
| TCGA-09-0369 | 2 | 20453558 | 24194334 | 1.628739636 | 4 | amp |
| TCGA-09-0369 | 2 | 24199801 | 24413510 | 2.689299172 | 5 | amp |
| TCGA-09-0369 | 2 | 24426423 | 25047445 | 1.631856283 | 4 | amp |
| TCGA-09-0369 | 2 | 25048864 | 25065307 | 3.133447382 | 5 | amp |
| TCGA-09-0369 | 2 | 25095363 | 25180880 | 1.585686585 | 4 | amp |
| TCGA-09-0369 | 2 | 25186248 | 25523119 | 3.066433961 | 5 | amp |
| TCGA-09-0369 | 2 | 25551489 | 26512863 | 1.860028749 | 4 | amp |
| TCGA-09-0369 | 2 | 26531904 | 27825438 | 3.223790782 | 5 | amp |
| TCGA-09-0369 | 2 | 27825940 | 28550340 | 1.795554143 | 4 | amp |
| TCGA-09-0369 | 2 | 28561258 | 28865945 | 2.497263068 | 5 | amp |
| TCGA-09-0369 | 2 | 28999662 | 29222370 | 1.732551324 | 4 | amp |
| TCGA-09-0369 | 2 | 29225387 | 29297153 | 2.509302858 | 5 | amp |
| TCGA-09-0369 | 2 | 29344200 | 29420577 | 1.163166914 | 3 | amp |
| TCGA-09-0369 | 2 | 29429997 | 29498374 | 2.496817203 | 5 | amp |
| TCGA-09-0369 | 2 | 29519718 | 30863533 | 1.817841759 | 4 | amp |
| TCGA-09-0369 | 2 | 30953578 | 31467347 | 2.514068442 | 5 | amp |
| TCGA-09-0369 | 2 | 31472225 | 46987314 | 1.489314099 | 4 | amp |
| TCGA-09-0369 | 2 | 47132547 | 47288128 | 2.500900384 | 5 | amp |
| TCGA-09-0369 | 2 | 47300801 | 70915264 | 1.48052027 | 4 | amp |
| TCGA-09-0369 | 2 | 70917869 | 71304786 | 2.880127234 | 5 | amp |
| TCGA-09-0369 | 2 | 71305422 | 71661964 | 1.642182159 | 4 | amp |
| TCGA-09-0369 | 2 | 71707980 | 71913662 | 2.925135338 | 5 | amp |
| TCGA-09-0369 | 2 | 72360113 | 72968635 | 1.622823535 | 4 | amp |
| TCGA-09-0369 | 2 | 73052922 | 73477619 | 3.289031587 | 5 | amp |
| TCGA-09-0369 | 2 | 73478306 | 74307731 | 1.709215507 | 4 | amp |
| TCGA-09-0369 | 2 | 74316978 | 75112728 | 3.445785208 | 5 | amp |
| TCGA-09-0369 | 2 | 75113410 | 85276809 | 1.694407027 | 4 | amp |
| TCGA-09-0369 | 2 | 85280221 | 86014070 | 3.077042118 | 5 | amp |
| TCGA-09-0369 | 2 | 86067210 | 86090666 | 1.592385016 | 4 | amp |
| TCGA-09-0369 | 2 | 86094673 | 86281457 | 2.81273183 | 5 | amp |
| TCGA-09-0369 | 2 | 86292342 | 95942154 | 1.606195132 | 4 | amp |
| TCGA-09-0369 | 2 | 95942283 | 95976305 | 3.151291839 | 5 | amp |
| TCGA-09-0369 | 2 | 96039955 | 96687436 | 1.491751393 | 4 | amp |
| TCGA-09-0369 | 2 | 96687844 | 96943484 | 2.572563624 | 5 | amp |
| TCGA-09-0369 | 2 | 96943496 | 97369106 | 1.857667534 | 4 | amp |
| TCGA-09-0369 | 2 | 97369254 | 97531285 | 2.652747098 | 5 | amp |
| TCGA-09-0369 | 2 | 97533478 | 110559453 | 1.414336749 | 4 | amp |
| TCGA-09-0369 | 2 | 110561004 | 111419468 | 1.01950297 | 3 | amp |
| TCGA-09-0369 | 2 | 111423765 | 121107482 | 1.403016599 | 4 | amp |
| TCGA-09-0369 | 2 | 121554820 | 122098539 | 2.821241945 | 5 | amp |
| TCGA-09-0369 | 2 | 122104611 | 128327544 | 1.703173651 | 4 | amp |
| TCGA-09-0369 | 2 | 128331445 | 128397049 | 2.625114519 | 5 | amp |
| TCGA-09-0369 | 2 | 128397592 | 130912870 | 1.564232556 | 4 | amp |

| | | | | | | |
|---|---|---|---|---|---|---|
| TCGA-09-0369 | 2 | 130918741 | 131117259 | 2.677661548 | 5 | amp |
| TCGA-09-0369 | 2 | 131126649 | 131266714 | 1.472324216 | 4 | amp |
| TCGA-09-0369 | 2 | 131279008 | 131356909 | 4.401760558 | 5 | amp |
| TCGA-09-0369 | 2 | 131369199 | 132237075 | 1.639500636 | 4 | amp |
| TCGA-09-0369 | 2 | 132237621 | 132287332 | 2.655160359 | 5 | amp |
| TCGA-09-0369 | 2 | 132288095 | 136598582 | 1.407299524 | 4 | amp |
| TCGA-09-0369 | 2 | 136602064 | 149539400 | 0.968447898 | 3 | amp |
| TCGA-09-0369 | 2 | 149541125 | 153616367 | 1.30195753 | 4 | amp |
| TCGA-09-0369 | 2 | 154800961 | 170163916 | 1.115365963 | 3 | amp |
| TCGA-09-0369 | 2 | 170175212 | 179355583 | 1.358368416 | 4 | amp |
| TCGA-09-0369 | 2 | 179358527 | 217069977 | 0.906179822 | 3 | amp |
| TCGA-09-0369 | 2 | 217123948 | 219137508 | 1.602441914 | 4 | amp |
| TCGA-09-0369 | 2 | 219140120 | 219247729 | 2.408126402 | 5 | amp |
| TCGA-09-0369 | 2 | 219247755 | 219825530 | 1.370660346 | 4 | amp |
| TCGA-09-0369 | 2 | 219854927 | 219871268 | 3.125817077 | 5 | amp |
| TCGA-09-0369 | 2 | 219878527 | 220032735 | 1.981916248 | 4 | amp |
| TCGA-09-0369 | 2 | 220032872 | 220104848 | 3.05799487 | 5 | amp |
| TCGA-09-0369 | 2 | 220104899 | 220345465 | 1.703706189 | 4 | amp |
| TCGA-09-0369 | 2 | 220345981 | 220402063 | 2.588428316 | 5 | amp |
| TCGA-09-0369 | 2 | 220402315 | 220506471 | 1.892567539 | 4 | amp |
| TCGA-09-0369 | 2 | 222290714 | 232127124 | 0.884599358 | 3 | amp |
| TCGA-09-0369 | 2 | 232135679 | 234173843 | 1.534753726 | 4 | amp |
| TCGA-09-0369 | 2 | 234178590 | 238263619 | 1.073457543 | 3 | amp |
| TCGA-09-0369 | 2 | 238265917 | 243160772 | 1.494009737 | 4 | amp |
| TCGA-09-0369 | 20 | 68319 | 238508 | 1.577884904 | 4 | amp |
| TCGA-09-0369 | 20 | 239682 | 464722 | 2.78683054 | 5 | amp |
| TCGA-09-0369 | 20 | 467002 | 485877 | 1.310720076 | 4 | amp |
| TCGA-09-0369 | 20 | 489084 | 1472022 | 2.428738263 | 5 | amp |
| TCGA-09-0369 | 20 | 1514975 | 1630115 | 1.64195799 | 4 | amp |
| TCGA-09-0369 | 20 | 1638233 | 2413332 | 2.215446315 | 5 | amp |
| TCGA-09-0369 | 20 | 2442360 | 2448448 | 1.0846981 | 3 | amp |
| TCGA-09-0369 | 20 | 2451274 | 3285198 | 2.987929519 | 5 | amp |
| TCGA-09-0369 | 20 | 3295633 | 3615056 | 1.468004414 | 4 | amp |
| TCGA-09-0369 | 20 | 3619468 | 3846801 | 3.691538816 | 5 | amp |
| TCGA-09-0369 | 20 | 3888568 | 3944712 | 1.211573338 | 3 | amp |
| TCGA-09-0369 | 20 | 4155656 | 5154356 | 2.245423306 | 5 | amp |
| TCGA-09-0369 | 20 | 5155816 | 5974396 | 1.634057673 | 4 | amp |
| TCGA-09-0369 | 20 | 5974900 | 6058026 | 2.382535562 | 5 | amp |
| TCGA-09-0369 | 20 | 6060003 | 9353059 | 1.597269695 | 4 | amp |
| TCGA-09-0369 | 20 | 9353662 | 9386037 | 2.498780002 | 5 | amp |
| TCGA-09-0369 | 20 | 9388514 | 9459671 | 1.390159901 | 4 | amp |
| TCGA-09-0369 | 20 | 9495471 | 9498900 | 3.585276602 | 5 | amp |
| TCGA-09-0369 | 20 | 9510257 | 10622545 | 1.697244016 | 4 | amp |
| TCGA-09-0369 | 20 | 10623132 | 10644696 | 2.632255612 | 5 | amp |
| TCGA-09-0369 | 20 | 11898876 | 17434614 | 1.483829526 | 4 | amp |
| TCGA-09-0369 | 20 | 17436952 | 17641187 | 2.374719677 | 5 | amp |
| TCGA-09-0369 | 20 | 17705613 | 18541399 | 1.849739276 | 4 | amp |
| TCGA-09-0369 | 20 | 18574359 | 20003171 | 2.376102151 | 5 | amp |
| TCGA-09-0369 | 20 | 20006275 | 23383729 | 1.656073236 | 4 | amp |
| TCGA-09-0369 | 20 | 23420863 | 25597248 | 2.768428598 | 5 | amp |

| | | | | | | |
|---|---|---|---|---|---|---|
| TCGA-09-0369 | 20 | 25655650 | 30028590 | 1.65080888 | 4 | amp |
| TCGA-09-0369 | 20 | 30037784 | 30310039 | 4.236719584 | 5 | amp |
| TCGA-09-0369 | 20 | 30345272 | 30388887 | 1.434117465 | 4 | amp |
| TCGA-09-0369 | 20 | 30407868 | 31380623 | 2.631260276 | 5 | amp |
| TCGA-09-0369 | 20 | 31381311 | 31573014 | 1.809887692 | 4 | amp |
| TCGA-09-0369 | 20 | 31573475 | 31768405 | 2.253679452 | 5 | amp |
| TCGA-09-0369 | 20 | 31805285 | 31891857 | 1.710714245 | 4 | amp |
| TCGA-09-0369 | 20 | 31892614 | 32850728 | 2.247479701 | 5 | amp |
| TCGA-09-0369 | 20 | 32868755 | 33370160 | 1.710995148 | 4 | amp |
| TCGA-09-0369 | 20 | 33433092 | 33502242 | 2.71407551 | 5 | amp |
| TCGA-09-0369 | 20 | 33502979 | 33517418 | 1.365352182 | 4 | amp |
| TCGA-09-0369 | 20 | 33519089 | 33600899 | 2.490112847 | 5 | amp |
| TCGA-09-0369 | 20 | 33603756 | 33722766 | 1.237413822 | 3 | amp |
| TCGA-09-0369 | 20 | 33725625 | 33894585 | 2.325690846 | 5 | amp |
| TCGA-09-0369 | 20 | 33902439 | 33999814 | 1.259535489 | 4 | amp |
| TCGA-09-0369 | 20 | 34021633 | 34215388 | 2.171422725 | 5 | amp |
| TCGA-09-0369 | 20 | 34218296 | 34527064 | 1.603121777 | 4 | amp |
| TCGA-09-0369 | 20 | 34528727 | 35509165 | 2.227071288 | 5 | amp |
| TCGA-09-0369 | 20 | 35515804 | 35569537 | 1.347393378 | 4 | amp |
| TCGA-09-0369 | 20 | 35575083 | 35651260 | 2.60178206 | 5 | amp |
| TCGA-09-0369 | 20 | 35661053 | 35690699 | 1.488096713 | 4 | amp |
| TCGA-09-0369 | 20 | 35693761 | 35812799 | 2.57486087 | 5 | amp |
| TCGA-09-0369 | 20 | 35826787 | 35867243 | 1.180514502 | 3 | amp |
| TCGA-09-0369 | 20 | 35869651 | 36361524 | 2.46227695 | 5 | amp |
| TCGA-09-0369 | 20 | 36365742 | 36488769 | 1.29413222 | 4 | amp |
| TCGA-09-0369 | 20 | 36500280 | 36662544 | 2.444971327 | 5 | amp |
| TCGA-09-0369 | 20 | 36668781 | 36718322 | 1.171857272 | 3 | amp |
| TCGA-09-0369 | 20 | 36758575 | 37078199 | 2.597205272 | 5 | amp |
| TCGA-09-0369 | 20 | 37117048 | 39802977 | 1.545660068 | 4 | amp |
| TCGA-09-0369 | 20 | 39803067 | 39993855 | 2.282125297 | 5 | amp |
| TCGA-09-0369 | 20 | 40033200 | 42169790 | 1.477114114 | 4 | amp |
| TCGA-09-0369 | 20 | 42194913 | 43115318 | 2.348580307 | 5 | amp |
| TCGA-09-0369 | 20 | 43117862 | 43247025 | 1.580236749 | 4 | amp |
| TCGA-09-0369 | 20 | 43248391 | 43571861 | 2.2990175 | 5 | amp |
| TCGA-09-0369 | 20 | 43572036 | 44354330 | 1.792981885 | 4 | amp |
| TCGA-09-0369 | 20 | 44402984 | 44996218 | 2.564879572 | 5 | amp |
| TCGA-09-0369 | 20 | 44997478 | 46282245 | 1.6285618 | 4 | amp |
| TCGA-09-0369 | 20 | 46287060 | 47247358 | 2.862554391 | 5 | amp |
| TCGA-09-0369 | 20 | 47248781 | 47782755 | 1.402019028 | 4 | amp |
| TCGA-09-0369 | 20 | 47835835 | 47855883 | 2.32016354 | 5 | amp |
| TCGA-09-0369 | 20 | 47858416 | 48497576 | 1.759341142 | 4 | amp |
| TCGA-09-0369 | 20 | 48500342 | 50235597 | 2.624295448 | 5 | amp |
| TCGA-09-0369 | 20 | 50238601 | 50314051 | 1.693922544 | 4 | amp |
| TCGA-09-0369 | 20 | 50329498 | 50776967 | 2.400487007 | 5 | amp |
| TCGA-09-0369 | 20 | 50781194 | 55206946 | 1.539176166 | 4 | amp |
| TCGA-09-0369 | 20 | 55208396 | 56099289 | 2.194396098 | 5 | amp |
| TCGA-09-0369 | 20 | 56136429 | 60573279 | 1.573607297 | 4 | amp |
| TCGA-09-0369 | 20 | 60573951 | 62926333 | 2.557946746 | 5 | amp |
| TCGA-09-0369 | 21 | 32513350 | 38460213 | 1.090835715 | 3 | amp |
| TCGA-09-0369 | 21 | 38597795 | 38612988 | 2.758691795 | 5 | amp |

| | | | | | | |
|---|---|---|---|---|---|---|
| TCGA-09-0369 | 21 | 39492381 | 40553810 | 1.131630374 | 3 | amp |
| TCGA-09-0369 | 21 | 40670275 | 42080718 | 0.950926486 | 3 | amp |
| TCGA-09-0369 | 21 | 42551458 | 48111215 | 1.507301054 | 4 | amp |
| TCGA-09-0369 | 22 | 16100468 | 17414938 | 1.101640922 | 3 | amp |
| TCGA-09-0369 | 22 | 17443544 | 25121048 | 1.660082795 | 4 | amp |
| TCGA-09-0369 | 22 | 25121386 | 25240961 | 0.845159493 | 3 | amp |
| TCGA-09-0369 | 22 | 25243561 | 25756106 | 1.511998093 | 4 | amp |
| TCGA-09-0369 | 22 | 25851594 | 26118456 | 0.908306714 | 3 | amp |
| TCGA-09-0369 | 22 | 26157017 | 30228333 | 1.458888463 | 4 | amp |
| TCGA-09-0369 | 22 | 30366989 | 30572163 | 0.923517433 | 3 | amp |
| TCGA-09-0369 | 22 | 30639604 | 31796787 | 1.69150064 | 4 | amp |
| TCGA-09-0369 | 22 | 31798982 | 31998298 | 0.907450766 | 3 | amp |
| TCGA-09-0369 | 22 | 31998531 | 32174179 | 1.610614913 | 4 | amp |
| TCGA-09-0369 | 22 | 32179871 | 35481746 | 1.107668966 | 3 | amp |
| TCGA-09-0369 | 22 | 35658308 | 35948114 | 1.792274287 | 4 | amp |
| TCGA-09-0369 | 22 | 36003266 | 36591539 | 1.016309032 | 3 | amp |
| TCGA-09-0369 | 22 | 36595338 | 38870637 | 1.868082205 | 4 | amp |
| TCGA-09-0369 | 22 | 38875556 | 38951447 | 0.876885911 | 3 | amp |
| TCGA-09-0369 | 22 | 38958272 | 40078703 | 1.761958588 | 4 | amp |
| TCGA-09-0369 | 22 | 40139625 | 41575008 | 1.034525859 | 3 | amp |
| TCGA-09-0369 | 22 | 41601318 | 41616924 | 1.499107843 | 4 | amp |
| TCGA-09-0369 | 22 | 41620019 | 41790329 | 2.273974559 | 5 | amp |
| TCGA-09-0369 | 22 | 41791736 | 43926889 | 1.576159798 | 4 | amp |
| TCGA-09-0369 | 22 | 43930539 | 44178248 | 0.79533398 | 3 | amp |
| TCGA-09-0369 | 22 | 44221876 | 45724375 | 1.514288906 | 4 | amp |
| TCGA-09-0369 | 22 | 45726427 | 45804805 | 0.817046282 | 3 | amp |
| TCGA-09-0369 | 22 | 45813447 | 50591680 | 1.508819744 | 4 | amp |
| TCGA-09-0369 | 22 | 50596458 | 50663040 | 2.892950072 | 5 | amp |
| TCGA-09-0369 | 22 | 50664147 | 51237627 | 1.646281702 | 4 | amp |
| TCGA-09-0369 | 3 | 9512137 | 9726371 | 1.146859202 | 3 | amp |
| TCGA-09-0369 | 3 | 9726519 | 9971892 | 1.589075027 | 4 | amp |
| TCGA-09-0369 | 3 | 9972023 | 10107187 | 0.983977356 | 3 | amp |
| TCGA-09-0369 | 3 | 10107545 | 10320758 | 1.572907204 | 4 | amp |
| TCGA-09-0369 | 3 | 10327478 | 11350556 | 1.199211141 | 3 | amp |
| TCGA-09-0369 | 3 | 12776192 | 12791354 | 0.987477747 | 3 | amp |
| TCGA-09-0369 | 3 | 12828859 | 14175333 | 1.454761365 | 4 | amp |
| TCGA-09-0369 | 3 | 14176210 | 15046151 | 1.070260316 | 3 | amp |
| TCGA-09-0369 | 3 | 37725280 | 38038682 | 1.085466654 | 3 | amp |
| TCGA-09-0369 | 3 | 38042865 | 38232335 | 1.493427328 | 4 | amp |
| TCGA-09-0369 | 3 | 38240163 | 38802898 | 1.124569181 | 3 | amp |
| TCGA-09-0369 | 3 | 39139643 | 40574323 | 1.052228785 | 3 | amp |
| TCGA-09-0369 | 3 | 42236214 | 46717201 | 0.98475162 | 3 | amp |
| TCGA-09-0369 | 3 | 46717663 | 47043367 | 1.712052284 | 4 | amp |
| TCGA-09-0369 | 3 | 47043815 | 48436134 | 1.139512178 | 3 | amp |
| TCGA-09-0369 | 3 | 48445854 | 50684273 | 1.613223366 | 4 | amp |
| TCGA-09-0369 | 3 | 51679572 | 52561955 | 1.685269482 | 4 | amp |
| TCGA-09-0369 | 3 | 52562823 | 52802666 | 0.962904773 | 3 | amp |
| TCGA-09-0369 | 3 | 52811569 | 52860085 | 1.590827021 | 4 | amp |
| TCGA-09-0369 | 3 | 52860521 | 54420831 | 1.152440903 | 3 | amp |
| TCGA-09-0369 | 3 | 58106888 | 58852472 | 1.088954439 | 3 | amp |

| | | | | | | |
|---|---|---|---|---|---|---|
| TCGA-09-0369 | 3 | 90276503 | 112650045 | 1.113950683 | 3 | amp |
| TCGA-09-0369 | 3 | 112666646 | 113346583 | 1.448590801 | 4 | amp |
| TCGA-09-0369 | 3 | 113373741 | 115561484 | 1.155106613 | 3 | amp |
| TCGA-09-0369 | 3 | 122403978 | 125298916 | 1.042701778 | 3 | amp |
| TCGA-09-0369 | 3 | 125301697 | 126217074 | 1.508970783 | 4 | amp |
| TCGA-09-0369 | 3 | 126218867 | 126224910 | 3.864028376 | 5 | amp |
| TCGA-09-0369 | 3 | 126226525 | 129822776 | 1.587996843 | 4 | amp |
| TCGA-09-0369 | 3 | 130159007 | 130394263 | 1.009814855 | 3 | amp |
| TCGA-09-0369 | 3 | 133368585 | 134911721 | 1.103165056 | 3 | amp |
| TCGA-09-0369 | 3 | 138007865 | 138119475 | 1.078341782 | 3 | amp |
| TCGA-09-0369 | 3 | 138121001 | 138187051 | 1.833155295 | 4 | amp |
| TCGA-09-0369 | 3 | 138187467 | 138351966 | 1.047609848 | 3 | amp |
| TCGA-09-0369 | 3 | 139195204 | 154898258 | 1.136602257 | 3 | amp |
| TCGA-09-0369 | 3 | 155198688 | 182858414 | 1.707906866 | 4 | amp |
| TCGA-09-0369 | 3 | 182870106 | 196771606 | 3.625304 | 5 | amp |
| TCGA-09-0369 | 3 | 196778433 | 197241330 | 2.078170389 | 4 | amp |
| TCGA-09-0369 | 3 | 197249461 | 197955154 | 2.844073945 | 5 | amp |
| TCGA-09-0369 | 4 | 53323 | 628650 | 1.299587269 | 4 | amp |
| TCGA-09-0369 | 4 | 629653 | 663925 | 3.312325874 | 5 | amp |
| TCGA-09-0369 | 4 | 667080 | 1838347 | 1.748980151 | 4 | amp |
| TCGA-09-0369 | 4 | 1843028 | 2698324 | 1.205497366 | 3 | amp |
| TCGA-09-0369 | 4 | 2701332 | 2834806 | 2.158002556 | 5 | amp |
| TCGA-09-0369 | 4 | 2877589 | 2900268 | 1.186489298 | 3 | amp |
| TCGA-09-0369 | 4 | 2900953 | 10447959 | 1.804052982 | 4 | amp |
| TCGA-09-0369 | 4 | 10492043 | 17503531 | 1.213338475 | 3 | amp |
| TCGA-09-0369 | 4 | 17505989 | 17816998 | 1.842303046 | 4 | amp |
| TCGA-09-0369 | 4 | 17818859 | 39449123 | 1.100880736 | 3 | amp |
| TCGA-09-0369 | 4 | 39449843 | 39505609 | 1.923308541 | 4 | amp |
| TCGA-09-0369 | 4 | 39505992 | 48424120 | 1.190206598 | 3 | amp |
| TCGA-09-0369 | 4 | 48486933 | 48496309 | 2.383802894 | 5 | amp |
| TCGA-09-0369 | 4 | 48501387 | 55161485 | 1.135999077 | 3 | amp |
| TCGA-09-0369 | 4 | 55561632 | 57907104 | 1.773497309 | 4 | amp |
| TCGA-09-0369 | 4 | 75312194 | 75484613 | 1.76880586 | 4 | amp |
| TCGA-09-0369 | 4 | 185552886 | 187126464 | 1.022132178 | 3 | amp |
| TCGA-09-0369 | 4 | 189061669 | 190948390 | 1.417449973 | 4 | amp |
| TCGA-09-0369 | 5 | 143116 | 11111182 | 1.580304152 | 4 | amp |
| TCGA-09-0369 | 5 | 11117522 | 13920777 | 0.971933867 | 3 | amp |
| TCGA-09-0369 | 5 | 13922176 | 17354033 | 1.404731541 | 4 | amp |
| TCGA-09-0369 | 5 | 18049639 | 31799863 | 0.94030485 | 3 | amp |
| TCGA-09-0369 | 5 | 31983181 | 32789930 | 1.281083891 | 4 | amp |
| TCGA-09-0369 | 5 | 32888069 | 33751700 | 1.011473844 | 3 | amp |
| TCGA-09-0369 | 5 | 33881164 | 34950480 | 1.279337883 | 4 | amp |
| TCGA-09-0369 | 5 | 34954008 | 45304000 | 1.034190469 | 3 | amp |
| TCGA-09-0369 | 5 | 66438244 | 72168566 | 0.937698496 | 3 | amp |
| TCGA-09-0369 | 5 | 75996849 | 79647792 | 0.948286435 | 3 | amp |
| TCGA-09-0369 | 5 | 131283282 | 131321185 | 1.043324767 | 3 | amp |
| TCGA-09-0369 | 5 | 131322434 | 131755668 | 1.26211631 | 4 | amp |
| TCGA-09-0369 | 5 | 131785301 | 132070213 | 0.842043047 | 3 | amp |
| TCGA-09-0369 | 5 | 132083952 | 132216934 | 1.464412504 | 4 | amp |
| TCGA-09-0369 | 5 | 132219021 | 132556618 | 0.951207945 | 3 | amp |

| | | | | | | |
|---|---|---|---|---|---|---|
| TCGA-09-0369 | 5 | 132559834 | 133915016 | 1.24414765 | 4 | amp |
| TCGA-09-0369 | 5 | 133942547 | 137542424 | 1.04877679 | 3 | amp |
| TCGA-09-0369 | 5 | 137548665 | 137781465 | 1.349198997 | 4 | amp |
| TCGA-09-0369 | 5 | 137801413 | 138715089 | 1.094974791 | 3 | amp |
| TCGA-09-0369 | 5 | 138715324 | 139828932 | 1.503349103 | 4 | amp |
| TCGA-09-0369 | 5 | 139838148 | 139930759 | 0.84063375 | 3 | amp |
| TCGA-09-0369 | 5 | 139931517 | 140051346 | 1.738757542 | 4 | amp |
| TCGA-09-0369 | 5 | 140052764 | 140966806 | 1.129556385 | 3 | amp |
| TCGA-09-0369 | 5 | 140967743 | 141387474 | 1.70089006 | 4 | amp |
| TCGA-09-0369 | 5 | 141391388 | 145442306 | 0.918361405 | 3 | amp |
| TCGA-09-0369 | 5 | 147819948 | 148422448 | 1.065510349 | 3 | amp |
| TCGA-09-0369 | 5 | 148424058 | 150920345 | 1.412899646 | 4 | amp |
| TCGA-09-0369 | 5 | 150921801 | 159782080 | 1.014427412 | 3 | amp |
| TCGA-09-0369 | 5 | 162866184 | 169697941 | 1.009906746 | 3 | amp |
| TCGA-09-0369 | 5 | 169701263 | 170159915 | 1.657124011 | 4 | amp |
| TCGA-09-0369 | 5 | 170160810 | 171384711 | 0.936883678 | 3 | amp |
| TCGA-09-0369 | 5 | 171471805 | 172507842 | 1.268512956 | 4 | amp |
| TCGA-09-0369 | 5 | 172513446 | 175775114 | 1.035349814 | 3 | amp |
| TCGA-09-0369 | 5 | 175775215 | 176323172 | 1.573929105 | 4 | amp |
| TCGA-09-0369 | 5 | 176332267 | 176722486 | 1.003387689 | 3 | amp |
| TCGA-09-0369 | 5 | 176728685 | 176939691 | 1.514620096 | 4 | amp |
| TCGA-09-0369 | 5 | 176939738 | 176951055 | 2.330928822 | 5 | amp |
| TCGA-09-0369 | 5 | 176951155 | 177054619 | 1.480526895 | 4 | amp |
| TCGA-09-0369 | 5 | 177058481 | 177474135 | 0.999585688 | 3 | amp |
| TCGA-09-0369 | 5 | 177482599 | 180220022 | 1.443718567 | 4 | amp |
| TCGA-09-0369 | 5 | 180326274 | 180432902 | 1.010642835 | 3 | amp |
| TCGA-09-0369 | 5 | 180477048 | 180899507 | 1.600364436 | 4 | amp |
| TCGA-09-0369 | 6 | 105907 | 17616961 | 1.715055806 | 4 | amp |
| TCGA-09-0369 | 6 | 17624747 | 26452615 | 1.060412701 | 3 | amp |
| TCGA-09-0369 | 6 | 26458843 | 33145275 | 1.818935898 | 4 | amp |
| TCGA-09-0369 | 6 | 33145400 | 33153576 | 3.243280358 | 5 | amp |
| TCGA-09-0369 | 6 | 33154349 | 33381620 | 1.951125935 | 4 | amp |
| TCGA-09-0369 | 6 | 33381791 | 33385484 | 3.74102224 | 5 | amp |
| TCGA-09-0369 | 6 | 33385822 | 37631932 | 1.487229451 | 4 | amp |
| TCGA-09-0369 | 6 | 37897642 | 39682564 | 0.940100608 | 3 | amp |
| TCGA-09-0369 | 6 | 39688472 | 41703953 | 2.007366886 | 4 | amp |
| TCGA-09-0369 | 6 | 41704488 | 41712573 | 3.303806163 | 5 | amp |
| TCGA-09-0369 | 6 | 41714952 | 42646461 | 1.884988936 | 4 | amp |
| TCGA-09-0369 | 6 | 42647455 | 44360604 | 3.883226142 | 5 | amp |
| TCGA-09-0369 | 6 | 44361096 | 108243116 | 0.888201779 | 3 | amp |
| TCGA-09-0369 | 6 | 108246018 | 109763517 | 1.394801986 | 4 | amp |
| TCGA-09-0369 | 6 | 109763726 | 109772912 | 3.975660064 | 5 | amp |
| TCGA-09-0369 | 6 | 109773406 | 109803233 | 2.002243766 | 4 | amp |
| TCGA-09-0369 | 6 | 109814552 | 157431710 | 0.917306535 | 3 | amp |
| TCGA-09-0369 | 6 | 157454131 | 160638556 | 1.24769411 | 4 | amp |
| TCGA-09-0369 | 6 | 160645696 | 161537951 | 0.906025711 | 3 | amp |
| TCGA-09-0369 | 6 | 161551848 | 169067169 | 1.500184239 | 4 | amp |
| TCGA-09-0369 | 6 | 169619843 | 169628416 | 3.807043844 | 5 | amp |
| TCGA-09-0369 | 6 | 169629660 | 171055029 | 1.752181299 | 4 | amp |
| TCGA-09-0369 | 7 | 538145 | 1195274 | 2.832387598 | 5 | amp |

| | | | | | | |
|---|---|---|---|---|---|---|
| TCGA-09-0369 | 7 | 1197248 | 1539003 | 1.710570485 | 4 | amp |
| TCGA-09-0369 | 7 | 1607259 | 2773169 | 3.075040079 | 5 | amp |
| TCGA-09-0369 | 7 | 2793390 | 2946493 | 2.057642997 | 4 | amp |
| TCGA-09-0369 | 7 | 2949651 | 2976889 | 3.33028069 | 5 | amp |
| TCGA-09-0369 | 7 | 2977483 | 5239307 | 2.115002932 | 4 | amp |
| TCGA-09-0369 | 7 | 5253921 | 5681015 | 3.222290928 | 5 | amp |
| TCGA-09-0369 | 7 | 5692032 | 5983641 | 1.887261731 | 4 | amp |
| TCGA-09-0369 | 7 | 5984691 | 6004227 | 2.725356104 | 5 | amp |
| TCGA-09-0369 | 7 | 6005245 | 6456441 | 1.977877152 | 4 | amp |
| TCGA-09-0369 | 7 | 6456451 | 6472648 | 2.830588826 | 5 | amp |
| TCGA-09-0369 | 7 | 6474311 | 6485779 | 1.713018538 | 4 | amp |
| TCGA-09-0369 | 7 | 6487365 | 6641811 | 3.120234813 | 5 | amp |
| TCGA-09-0369 | 7 | 6730446 | 6737715 | 1.710280884 | 4 | amp |
| TCGA-09-0369 | 7 | 6744736 | 6816202 | 3.01883045 | 5 | amp |
| TCGA-09-0369 | 7 | 6819237 | 11076724 | 1.371928764 | 4 | amp |
| TCGA-09-0369 | 7 | 11078374 | 22190133 | 1.050319542 | 3 | amp |
| TCGA-09-0369 | 7 | 22194027 | 23766956 | 1.400863796 | 4 | amp |
| TCGA-09-0369 | 7 | 23768668 | 24727264 | 0.887413939 | 3 | amp |
| TCGA-09-0369 | 7 | 24738641 | 27150309 | 1.407903068 | 4 | amp |
| TCGA-09-0369 | 7 | 27168727 | 27238118 | 2.531148101 | 5 | amp |
| TCGA-09-0369 | 7 | 27284584 | 30878993 | 1.460634944 | 4 | amp |
| TCGA-09-0369 | 7 | 30880329 | 31117719 | 2.636903495 | 5 | amp |
| TCGA-09-0369 | 7 | 31120177 | 31736774 | 1.636040718 | 4 | amp |
| TCGA-09-0369 | 7 | 31746797 | 37934238 | 1.105853954 | 3 | amp |
| TCGA-09-0369 | 7 | 37936432 | 38393822 | 1.49833564 | 4 | amp |
| TCGA-09-0369 | 7 | 38398087 | 40900116 | 1.048902087 | 3 | amp |
| TCGA-09-0369 | 7 | 41729216 | 44047228 | 1.590031058 | 4 | amp |
| TCGA-09-0369 | 7 | 44047277 | 44302693 | 3.606235976 | 5 | amp |
| TCGA-09-0369 | 7 | 44425604 | 44575622 | 2.016431568 | 4 | amp |
| TCGA-09-0369 | 7 | 44575790 | 44619272 | 2.426818057 | 5 | amp |
| TCGA-09-0369 | 7 | 44620614 | 44875312 | 1.975472804 | 4 | amp |
| TCGA-09-0369 | 7 | 44880494 | 45145381 | 2.852489333 | 5 | amp |
| TCGA-09-0369 | 7 | 45145482 | 50596026 | 1.746407269 | 4 | amp |
| TCGA-09-0369 | 7 | 50596852 | 51094422 | 2.575741277 | 5 | amp |
| TCGA-09-0369 | 7 | 51095358 | 55210174 | 1.67355434 | 4 | amp |
| TCGA-09-0369 | 7 | 55210969 | 55234054 | 2.483063895 | 5 | amp |
| TCGA-09-0369 | 7 | 55236158 | 56127344 | 1.839681915 | 4 | amp |
| TCGA-09-0369 | 7 | 56127915 | 56174141 | 2.556534823 | 5 | amp |
| TCGA-09-0369 | 7 | 56358801 | 65426086 | 1.664321602 | 4 | amp |
| TCGA-09-0369 | 7 | 65429257 | 65444925 | 3.057341392 | 5 | amp |
| TCGA-09-0369 | 7 | 65469009 | 65469129 | 1.138525207 | 3 | amp |
| TCGA-09-0369 | 7 | 65551520 | 65580013 | 2.961292251 | 5 | amp |
| TCGA-09-0369 | 7 | 65592648 | 65817576 | 1.621500647 | 4 | amp |
| TCGA-09-0369 | 7 | 65821760 | 66019640 | 2.994983262 | 5 | amp |
| TCGA-09-0369 | 7 | 66038454 | 72730745 | 1.682740918 | 4 | amp |
| TCGA-09-0369 | 7 | 72732634 | 72745839 | 2.524650772 | 5 | amp |
| TCGA-09-0369 | 7 | 72754616 | 72951733 | 1.30481054 | 4 | amp |
| TCGA-09-0369 | 7 | 72952243 | 73602154 | 2.335855162 | 5 | amp |
| TCGA-09-0369 | 7 | 73603974 | 73944256 | 1.787425 | 4 | amp |
| TCGA-09-0369 | 7 | 73949395 | 74015427 | 2.973680677 | 5 | amp |

| | | | | | | |
|---|---|---|---|---|---|---|
| TCGA-09-0369 | 7 | 74016665 | 74248099 | 1.492964216 | 4 | amp |
| TCGA-09-0369 | 7 | 74251390 | 74300649 | 2.72325865 | 5 | amp |
| TCGA-09-0369 | 7 | 74301078 | 74328258 | 1.448482398 | 4 | amp |
| TCGA-09-0369 | 7 | 74329226 | 74482649 | 3.827291757 | 5 | amp |
| TCGA-09-0369 | 7 | 74486371 | 74994083 | 1.583961614 | 4 | amp |
| TCGA-09-0369 | 7 | 74994512 | 75028634 | 2.602399567 | 5 | amp |
| TCGA-09-0369 | 7 | 75032915 | 75399118 | 1.864805565 | 4 | amp |
| TCGA-09-0369 | 7 | 75401173 | 75633184 | 2.493279306 | 5 | amp |
| TCGA-09-0369 | 7 | 75634550 | 76659870 | 1.836021768 | 4 | amp |
| TCGA-09-0369 | 7 | 76669217 | 97487773 | 0.823347491 | 3 | amp |
| TCGA-09-0369 | 7 | 97488156 | 99027439 | 1.482159033 | 4 | amp |
| TCGA-09-0369 | 7 | 99030856 | 99051793 | 2.717859552 | 5 | amp |
| TCGA-09-0369 | 7 | 99054028 | 99246035 | 1.575644533 | 4 | amp |
| TCGA-09-0369 | 7 | 99247655 | 99461359 | 0.790453809 | 3 | amp |
| TCGA-09-0369 | 7 | 99463516 | 99704141 | 1.838241507 | 4 | amp |
| TCGA-09-0369 | 7 | 99704242 | 99720587 | 3.007008853 | 5 | amp |
| TCGA-09-0369 | 7 | 99722075 | 100179512 | 1.574994462 | 4 | amp |
| TCGA-09-0369 | 7 | 100179576 | 100193383 | 2.578711116 | 5 | amp |
| TCGA-09-0369 | 7 | 100197618 | 100282275 | 1.67477674 | 4 | amp |
| TCGA-09-0369 | 7 | 100282281 | 100391660 | 2.859840139 | 5 | amp |
| TCGA-09-0369 | 7 | 100391690 | 102113545 | 1.852317068 | 4 | amp |
| TCGA-09-0369 | 7 | 102114774 | 102143710 | 4.588007974 | 5 | amp |
| TCGA-09-0369 | 7 | 102194714 | 102208664 | 1.263034247 | 4 | amp |
| TCGA-09-0369 | 7 | 102210266 | 102235870 | 3.320929672 | 5 | amp |
| TCGA-09-0369 | 7 | 102240293 | 102343959 | 1.727316686 | 4 | amp |
| TCGA-09-0369 | 7 | 102453763 | 117420689 | 0.884664411 | 3 | amp |
| TCGA-09-0369 | 7 | 117422905 | 127229653 | 1.416838188 | 4 | amp |
| TCGA-09-0369 | 7 | 127230065 | 127240470 | 2.780940324 | 5 | amp |
| TCGA-09-0369 | 7 | 127251509 | 127670731 | 1.591847345 | 4 | amp |
| TCGA-09-0369 | 7 | 127721362 | 127894876 | 3.226746726 | 5 | amp |
| TCGA-09-0369 | 7 | 127950796 | 127979885 | 1.649699389 | 4 | amp |
| TCGA-09-0369 | 7 | 127983638 | 128455012 | 2.375987589 | 5 | amp |
| TCGA-09-0369 | 7 | 128455635 | 128457922 | 1.710595487 | 4 | amp |
| TCGA-09-0369 | 7 | 128475293 | 128505651 | 3.03771659 | 5 | amp |
| TCGA-09-0369 | 7 | 128582082 | 128694884 | 1.764966728 | 4 | amp |
| TCGA-09-0369 | 7 | 128801458 | 128852312 | 3.284736458 | 5 | amp |
| TCGA-09-0369 | 7 | 129008303 | 129053541 | 1.594386017 | 4 | amp |
| TCGA-09-0369 | 7 | 129062669 | 129104635 | 2.794896504 | 5 | amp |
| TCGA-09-0369 | 7 | 129107180 | 129940764 | 1.763861173 | 4 | amp |
| TCGA-09-0369 | 7 | 129944308 | 130021039 | 2.861111473 | 5 | amp |
| TCGA-09-0369 | 7 | 130021437 | 131833413 | 1.852516202 | 4 | amp |
| TCGA-09-0369 | 7 | 131844161 | 131911029 | 2.503679605 | 5 | amp |
| TCGA-09-0369 | 7 | 131912166 | 134878186 | 1.694166388 | 4 | amp |
| TCGA-09-0369 | 7 | 134878316 | 135047967 | 2.961569181 | 5 | amp |
| TCGA-09-0369 | 7 | 135048591 | 139102499 | 1.612892715 | 4 | amp |
| TCGA-09-0369 | 7 | 139106877 | 139833517 | 2.982467289 | 5 | amp |
| TCGA-09-0369 | 7 | 139838887 | 140037176 | 1.54936274 | 4 | amp |
| TCGA-09-0369 | 7 | 140043137 | 140439798 | 3.707894916 | 5 | amp |
| TCGA-09-0369 | 7 | 140449032 | 141366275 | 1.783148932 | 4 | amp |
| TCGA-09-0369 | 7 | 141385236 | 141419077 | 3.03209174 | 5 | amp |

| | | | | | | |
|---|---|---|---|---|---|---|
| TCGA-09-0369 | 7 | 141420675 | 141736819 | 2.046938624 | 4 | amp |
| TCGA-09-0369 | 7 | 141738312 | 142364317 | 2.573619983 | 5 | amp |
| TCGA-09-0369 | 7 | 142364382 | 142423720 | 1.801735216 | 4 | amp |
| TCGA-09-0369 | 7 | 142428467 | 143104797 | 4.129327887 | 5 | amp |
| TCGA-09-0369 | 7 | 143140453 | 143271277 | 1.643570182 | 4 | amp |
| TCGA-09-0369 | 7 | 143295091 | 143827151 | 3.530709688 | 5 | amp |
| TCGA-09-0369 | 7 | 143880563 | 143966376 | 2.071992967 | 4 | amp |
| TCGA-09-0369 | 7 | 143969513 | 144708153 | 6.939910443 | 5 | amp |
| TCGA-09-0369 | 7 | 146471357 | 148705446 | 0.881066815 | 3 | amp |
| TCGA-09-0369 | 7 | 148708896 | 150749348 | 1.610542269 | 4 | amp |
| TCGA-09-0369 | 7 | 150749441 | 150754262 | 5.105762397 | 5 | amp |
| TCGA-09-0369 | 7 | 150761553 | 151164310 | 1.65937194 | 4 | amp |
| TCGA-09-0369 | 7 | 151167637 | 158935247 | 0.889935974 | 3 | amp |
| TCGA-09-0369 | 8 | 116074 | 363266 | 2.136994239 | 4 | amp |
| TCGA-09-0369 | 8 | 381332 | 57890716 | 2.394538888 | 5 | amp |
| TCGA-09-0369 | 8 | 57892554 | 59404363 | 1.272405046 | 4 | amp |
| TCGA-09-0369 | 8 | 59404879 | 146112315 | 0.928758641 | 3 | amp |
| TCGA-09-0369 | 8 | 146114962 | 146279593 | 1.800861382 | 4 | amp |
| TCGA-09-0369 | 9 | 17322 | 2088616 | 1.038648333 | 3 | amp |
| TCGA-09-0369 | 9 | 32553847 | 34372948 | 1.180864242 | 3 | amp |
| TCGA-09-0369 | 9 | 34379066 | 35102871 | 1.82198344 | 4 | amp |
| TCGA-09-0369 | 9 | 35103008 | 35607683 | 1.178474046 | 3 | amp |
| TCGA-09-0369 | 9 | 35607906 | 35826219 | 1.989622334 | 4 | amp |
| TCGA-09-0369 | 9 | 35842364 | 67930870 | 0.993424998 | 3 | amp |
| TCGA-09-0369 | 9 | 88636755 | 95792336 | 1.026644995 | 3 | amp |
| TCGA-09-0369 | 9 | 95795012 | 96062441 | 1.984570586 | 4 | amp |
| TCGA-09-0369 | 9 | 96069020 | 101825399 | 1.151143755 | 3 | amp |
| TCGA-09-0369 | 9 | 115598441 | 116136676 | 1.11672343 | 3 | amp |
| TCGA-09-0369 | 9 | 116150550 | 117094217 | 1.690286443 | 4 | amp |
| TCGA-09-0369 | 9 | 117095321 | 130432266 | 1.07904834 | 3 | amp |
| TCGA-09-0369 | 9 | 130434302 | 131223314 | 2.01813243 | 4 | amp |
| TCGA-09-0369 | 9 | 131231426 | 131392686 | 1.234319184 | 3 | amp |
| TCGA-09-0369 | 9 | 131394353 | 131718730 | 1.776209646 | 4 | amp |
| TCGA-09-0369 | 9 | 131719210 | 131760528 | 1.219286098 | 3 | amp |
| TCGA-09-0369 | 9 | 131760799 | 132637939 | 1.86769926 | 4 | amp |
| TCGA-09-0369 | 9 | 132641797 | 132897411 | 1.107835339 | 3 | amp |
| TCGA-09-0369 | 9 | 132963158 | 133995725 | 1.705435162 | 4 | amp |
| TCGA-09-0369 | 9 | 134002858 | 134365975 | 1.155009257 | 3 | amp |
| TCGA-09-0369 | 9 | 134366769 | 134471782 | 2.359362621 | 5 | amp |
| TCGA-09-0369 | 9 | 134473581 | 135927558 | 1.162900256 | 3 | amp |
| TCGA-09-0369 | 9 | 135929211 | 141071671 | 2.080397772 | 4 | amp |
| TCGA-09-0369 | X | 200797 | 1746690 | 0.938788657 | 3 | amp |
| TCGA-09-0369 | X | 2828646 | 2876515 | 1.153400418 | 3 | amp |
| TCGA-09-0369 | X | 13773225 | 18646718 | 0.49262557 | 1 | loss |
| TCGA-09-0369 | X | 19725142 | 46918503 | 0.472953096 | 1 | loss |
| TCGA-09-0369 | X | 47035881 | 47046055 | 0.942025834 | 3 | amp |
| TCGA-09-0369 | X | 47058139 | 47063137 | 1.420965412 | 4 | amp |
| TCGA-09-0369 | X | 47065309 | 47230163 | 0.994061202 | 3 | amp |
| TCGA-09-0369 | X | 48320587 | 48463447 | 0.940299546 | 3 | amp |
| TCGA-09-0369 | X | 48542188 | 48546885 | 1.767263263 | 4 | amp |

| | | | | | | |
|---|---|---|---|---|---|---|
| TCGA-09-0369 | X | 48547015 | 49061831 | 0.873877832 | 3 | amp |
| TCGA-09-0369 | X | 49062967 | 49082751 | 1.365544137 | 4 | amp |
| TCGA-09-0369 | X | 49208251 | 49242970 | 1.004561112 | 3 | amp |
| TCGA-09-0369 | X | 52936213 | 53285292 | 0.920316144 | 3 | amp |
| TCGA-09-0369 | X | 62944381 | 67935236 | 0.810638128 | 3 | amp |
| TCGA-09-0369 | X | 67936184 | 68749817 | 1.806049933 | 4 | amp |
| TCGA-09-0369 | X | 68836110 | 69243139 | 1.059245757 | 3 | amp |
| TCGA-09-0369 | X | 69247811 | 69424380 | 1.366783406 | 4 | amp |
| TCGA-09-0369 | X | 69424758 | 69490031 | 0.962312718 | 3 | amp |
| TCGA-09-0369 | X | 69495911 | 69510657 | 1.753621113 | 4 | amp |
| TCGA-09-0369 | X | 69516822 | 69640160 | 1.014249468 | 3 | amp |
| TCGA-09-0369 | X | 69644826 | 69715360 | 1.49105202 | 4 | amp |
| TCGA-09-0369 | X | 69716990 | 70127680 | 0.844892345 | 3 | amp |
| TCGA-09-0369 | X | 70145607 | 70469063 | 1.341588985 | 4 | amp |
| TCGA-09-0369 | X | 70469299 | 73811703 | 0.928600272 | 3 | amp |
| TCGA-09-0369 | X | 77359809 | 86869624 | 0.916323364 | 3 | amp |
| TCGA-09-0369 | X | 91066212 | 100536795 | 0.508937631 | 1 | loss |
| TCGA-09-0369 | X | 103903545 | 117907949 | 0.451018776 | 1 | loss |
| TCGA-09-0369 | X | 120007692 | 120112951 | 0.939935183 | 3 | amp |
| TCGA-09-0369 | X | 122387093 | 128724304 | 0.416314841 | 1 | loss |
| TCGA-09-0369 | X | 129338822 | 134714139 | 0.500918438 | 1 | loss |
| TCGA-09-0369 | X | 135076924 | 148072921 | 0.469930837 | 1 | loss |
| TCGA-09-0369 | X | 151806580 | 152730539 | 0.903688887 | 3 | amp |
| TCGA-09-0369 | X | 152734571 | 152807035 | 1.631953509 | 4 | amp |
| TCGA-09-0369 | X | 152807045 | 153196423 | 1.131664063 | 3 | amp |
| TCGA-09-0369 | X | 153197455 | 153228899 | 1.304411898 | 4 | amp |
| TCGA-09-0369 | X | 153229534 | 153425995 | 0.835709812 | 3 | amp |
| TCGA-09-0369 | X | 153524129 | 153871036 | 1.034529015 | 3 | amp |
| TCGA-09-1664 | 1 | 16834 | 31214584 | 0.595994502 | 1 | loss |
| TCGA-09-1664 | 1 | 32158663 | 32162920 | 1.344053907 | 3 | amp |
| TCGA-09-1664 | 1 | 55524125 | 57161839 | 0.695227579 | 1 | loss |
| TCGA-09-1664 | 1 | 84946606 | 85136940 | 0.745104044 | 1 | loss |
| TCGA-09-1664 | 1 | 91727753 | 91788762 | 0.692731102 | 1 | loss |
| TCGA-09-1664 | 1 | 97216958 | 97279016 | 0.671269244 | 1 | loss |
| TCGA-09-1664 | 1 | 103427388 | 103474155 | 0.708821441 | 1 | loss |
| TCGA-09-1664 | 1 | 109839621 | 109859585 | 1.313263178 | 3 | amp |
| TCGA-09-1664 | 1 | 110199808 | 110217473 | 1.223676788 | 3 | amp |
| TCGA-09-1664 | 1 | 156446235 | 156905880 | 1.158276569 | 3 | amp |
| TCGA-09-1664 | 1 | 161069812 | 161198935 | 1.171794127 | 3 | amp |
| TCGA-09-1664 | 1 | 186269163 | 186301520 | 0.71659158 | 1 | loss |
| TCGA-09-1664 | 1 | 200957362 | 201113120 | 1.21228675 | 3 | amp |
| TCGA-09-1664 | 1 | 207715605 | 207871226 | 0.738004257 | 1 | loss |
| TCGA-09-1664 | 1 | 208062767 | 209785588 | 1.20041032 | 3 | amp |
| TCGA-09-1664 | 1 | 237586349 | 237923173 | 0.748316723 | 1 | loss |
| TCGA-09-1664 | 10 | 15838044 | 15883631 | 0.714244248 | 1 | loss |
| TCGA-09-1664 | 10 | 17702414 | 17735325 | 0.692869246 | 1 | loss |
| TCGA-09-1664 | 10 | 22615374 | 22616992 | 0.53791451 | 1 | loss |
| TCGA-09-1664 | 10 | 27322109 | 27332545 | 0.749280577 | 1 | loss |
| TCGA-09-1664 | 10 | 27342224 | 27355506 | 0.744027941 | 1 | loss |
| TCGA-09-1664 | 10 | 28347387 | 28420638 | 0.744919805 | 1 | loss |

| | | | | | | |
|---|---|---|---|---|---|---|
| TCGA-09-1664 | 10 | 32128141 | 32143183 | 0.704855298 | 1 | loss |
| TCGA-09-1664 | 10 | 32742257 | 32807448 | 0.772614013 | 1 | loss |
| TCGA-09-1664 | 10 | 32863299 | 33094044 | 0.714524137 | 1 | loss |
| TCGA-09-1664 | 10 | 33208792 | 33221545 | 0.764644261 | 1 | loss |
| TCGA-09-1664 | 10 | 37440958 | 37508846 | 0.729017118 | 1 | loss |
| TCGA-09-1664 | 10 | 47194144 | 47233591 | 0.729294983 | 1 | loss |
| TCGA-09-1664 | 10 | 48259458 | 48268159 | 1.224858458 | 3 | amp |
| TCGA-09-1664 | 10 | 54040499 | 54053670 | 0.721565458 | 1 | loss |
| TCGA-09-1664 | 10 | 70182256 | 70197002 | 0.715102309 | 1 | loss |
| TCGA-09-1664 | 10 | 72192647 | 72297674 | 1.216058479 | 3 | amp |
| TCGA-09-1664 | 10 | 73822407 | 73848111 | 1.298386219 | 3 | amp |
| TCGA-09-1664 | 10 | 75206230 | 75260624 | 0.743339842 | 1 | loss |
| TCGA-09-1664 | 10 | 75984262 | 76360253 | 0.703928287 | 1 | loss |
| TCGA-09-1664 | 10 | 91477156 | 91483910 | 0.642324826 | 1 | loss |
| TCGA-09-1664 | 10 | 91522373 | 91533826 | 0.723772364 | 1 | loss |
| TCGA-09-1664 | 10 | 92634593 | 92655715 | 0.739313466 | 1 | loss |
| TCGA-09-1664 | 10 | 92982624 | 93221968 | 0.70607901 | 1 | loss |
| TCGA-09-1664 | 10 | 93255929 | 93261139 | 0.697029402 | 1 | loss |
| TCGA-09-1664 | 10 | 94653041 | 94675712 | 0.736446546 | 1 | loss |
| TCGA-09-1664 | 10 | 104859639 | 104866524 | 0.701707692 | 1 | loss |
| TCGA-09-1664 | 10 | 112642747 | 112769649 | 0.729227595 | 1 | loss |
| TCGA-09-1664 | 10 | 115636271 | 115663510 | 0.695916449 | 1 | loss |
| TCGA-09-1664 | 10 | 116590589 | 116608510 | 0.748822864 | 1 | loss |
| TCGA-09-1664 | 10 | 116879930 | 117045936 | 0.753771018 | 1 | loss |
| TCGA-09-1664 | 10 | 119768472 | 119805677 | 0.700129293 | 1 | loss |
| TCGA-09-1664 | 10 | 121608892 | 121619413 | 0.743789061 | 1 | loss |
| TCGA-09-1664 | 10 | 122618111 | 122626689 | 0.741612324 | 1 | loss |
| TCGA-09-1664 | 11 | 5655021 | 5664119 | 0.720979587 | 1 | loss |
| TCGA-09-1664 | 11 | 6964764 | 7092565 | 0.771095935 | 1 | loss |
| TCGA-09-1664 | 11 | 10011032 | 10064579 | 0.715974643 | 1 | loss |
| TCGA-09-1664 | 11 | 11906010 | 11977754 | 0.657663721 | 1 | loss |
| TCGA-09-1664 | 11 | 14793447 | 14856644 | 0.727320155 | 1 | loss |
| TCGA-09-1664 | 11 | 16863061 | 16877465 | 1.272926334 | 3 | amp |
| TCGA-09-1664 | 11 | 28045282 | 30358347 | 0.772116965 | 1 | loss |
| TCGA-09-1664 | 11 | 30946845 | 31128545 | 0.714775282 | 1 | loss |
| TCGA-09-1664 | 11 | 31455001 | 31805126 | 0.753238531 | 1 | loss |
| TCGA-09-1664 | 11 | 34093243 | 34104474 | 0.735680499 | 1 | loss |
| TCGA-09-1664 | 11 | 34163254 | 34378589 | 1.208329112 | 3 | amp |
| TCGA-09-1664 | 11 | 57367274 | 57374070 | 1.339367082 | 3 | amp |
| TCGA-09-1664 | 11 | 60973920 | 61035034 | 1.246492653 | 3 | amp |
| TCGA-09-1664 | 11 | 62400006 | 62401874 | 1.294632979 | 3 | amp |
| TCGA-09-1664 | 11 | 62606950 | 62622860 | 1.239901618 | 3 | amp |
| TCGA-09-1664 | 11 | 71529806 | 71589587 | 0.745304482 | 1 | loss |
| TCGA-09-1664 | 11 | 72553655 | 72598626 | 0.722085139 | 1 | loss |
| TCGA-09-1664 | 11 | 73849708 | 73941486 | 0.75377387 | 1 | loss |
| TCGA-09-1664 | 11 | 74013375 | 74085663 | 0.723709297 | 1 | loss |
| TCGA-09-1664 | 11 | 82922254 | 82985794 | 0.766784186 | 1 | loss |
| TCGA-09-1664 | 11 | 83877942 | 85361402 | 0.763784629 | 1 | loss |
| TCGA-09-1664 | 11 | 86782550 | 87020750 | 0.718054392 | 1 | loss |
| TCGA-09-1664 | 11 | 89372397 | 89424704 | 0.769326457 | 1 | loss |

| | | | | | | |
|---|---|---|---|---|---|---|
| TCGA-09-1664 | 11 | 89595601 | 89666103 | 0.772686746 | 1 | loss |
| TCGA-09-1664 | 11 | 89819108 | 89944494 | 0.722646899 | 1 | loss |
| TCGA-09-1664 | 11 | 92085244 | 92526157 | 0.739501823 | 1 | loss |
| TCGA-09-1664 | 11 | 92881646 | 93170916 | 0.736514138 | 1 | loss |
| TCGA-09-1664 | 11 | 93471229 | 93493085 | 0.758834243 | 1 | loss |
| TCGA-09-1664 | 11 | 93754499 | 93800968 | 0.6880452 | 1 | loss |
| TCGA-09-1664 | 11 | 93826616 | 93845078 | 0.656575853 | 1 | loss |
| TCGA-09-1664 | 11 | 95546035 | 100211940 | 0.765922831 | 1 | loss |
| TCGA-09-1664 | 11 | 101793281 | 101868441 | 0.72554326 | 1 | loss |
| TCGA-09-1664 | 11 | 102233605 | 102248937 | 0.72868522 | 1 | loss |
| TCGA-09-1664 | 11 | 102709757 | 102815104 | 0.7793588 | 1 | loss |
| TCGA-09-1664 | 11 | 102953462 | 103229088 | 0.687422249 | 1 | loss |
| TCGA-09-1664 | 11 | 105961224 | 107300210 | 0.756241549 | 1 | loss |
| TCGA-09-1664 | 11 | 107506306 | 107535956 | 0.728177688 | 1 | loss |
| TCGA-09-1664 | 11 | 107948884 | 107966457 | 0.708685613 | 1 | loss |
| TCGA-09-1664 | 11 | 108114762 | 108218108 | 0.785821401 | 1 | loss |
| TCGA-09-1664 | 11 | 111909894 | 111922075 | 0.719528035 | 1 | loss |
| TCGA-09-1664 | 11 | 112103830 | 113078101 | 0.712009351 | 1 | loss |
| TCGA-09-1664 | 11 | 118512925 | 118531659 | 1.190055278 | 3 | amp |
| TCGA-09-1664 | 11 | 120292476 | 120345366 | 0.774036604 | 1 | loss |
| TCGA-09-1664 | 11 | 124085879 | 124135782 | 0.600530704 | 1 | loss |
| TCGA-09-1664 | 11 | 124856986 | 124909010 | 0.742285602 | 1 | loss |
| TCGA-09-1664 | 11 | 134076410 | 134087030 | 0.623995584 | 1 | loss |
| TCGA-09-1664 | 14 | 19377543 | 19378624 | 0.792501929 | 1 | loss |
| TCGA-09-1664 | 14 | 107131398 | 107283263 | 0.675645101 | 1 | loss |
| TCGA-09-1664 | 16 | 4895024 | 4911123 | 0.769571276 | 1 | loss |
| TCGA-09-1664 | 16 | 10551157 | 10626975 | 0.771919642 | 1 | loss |
| TCGA-09-1664 | 16 | 11870155 | 11875360 | 0.685406074 | 1 | loss |
| TCGA-09-1664 | 16 | 14280711 | 14334695 | 0.756183145 | 1 | loss |
| TCGA-09-1664 | 16 | 14540721 | 14711536 | 0.770202603 | 1 | loss |
| TCGA-09-1664 | 16 | 15164922 | 15178590 | 0.784100431 | 1 | loss |
| TCGA-09-1664 | 16 | 15812100 | 15835802 | 1.260006679 | 3 | amp |
| TCGA-09-1664 | 16 | 18823075 | 18866281 | 0.691567944 | 1 | loss |
| TCGA-09-1664 | 16 | 18874952 | 18880645 | 0.747495381 | 1 | loss |
| TCGA-09-1664 | 16 | 18887400 | 18907571 | 0.753034416 | 1 | loss |
| TCGA-09-1664 | 16 | 19556079 | 19559396 | 0.763884566 | 1 | loss |
| TCGA-09-1664 | 16 | 19746663 | 19867899 | 0.766020173 | 1 | loss |
| TCGA-09-1664 | 16 | 21464003 | 21467800 | 0.71642128 | 1 | loss |
| TCGA-09-1664 | 16 | 21890717 | 21926519 | 0.775109677 | 1 | loss |
| TCGA-09-1664 | 16 | 22466848 | 22497542 | 0.73992339 | 1 | loss |
| TCGA-09-1664 | 16 | 23226359 | 23436288 | 1.252905627 | 3 | amp |
| TCGA-09-1664 | 16 | 24564795 | 24583564 | 0.716875073 | 1 | loss |
| TCGA-09-1664 | 16 | 28601361 | 28994270 | 1.19080119 | 3 | amp |
| TCGA-09-1664 | 16 | 29544466 | 29566369 | 0.793219171 | 1 | loss |
| TCGA-09-1664 | 16 | 29569972 | 30208694 | 1.113074132 | 3 | amp |
| TCGA-09-1664 | 16 | 30208712 | 30215036 | 1.371908889 | 4 | amp |
| TCGA-09-1664 | 16 | 31925779 | 32163867 | 0.707358174 | 1 | loss |
| TCGA-09-1664 | 16 | 32165226 | 32170570 | 1.166746651 | 3 | amp |
| TCGA-09-1664 | 16 | 32176937 | 32265095 | 0.770607036 | 1 | loss |
| TCGA-09-1664 | 16 | 32926761 | 33122010 | 0.769111748 | 1 | loss |

| | | | | | | |
|---|---|---|---|---|---|---|
| TCGA-09-1664 | 16 | 33490850 | 33741019 | 0.724071576 | 1 | loss |
| TCGA-09-1664 | 16 | 47156511 | 47252877 | 0.674596925 | 1 | loss |
| TCGA-09-1664 | 16 | 50257071 | 50261951 | 0.570291696 | 1 | loss |
| TCGA-09-1664 | 16 | 50367426 | 50388888 | 0.719269144 | 1 | loss |
| TCGA-09-1664 | 16 | 50783561 | 50826621 | 0.743425952 | 1 | loss |
| TCGA-09-1664 | 16 | 52472791 | 53501085 | 0.74291363 | 1 | loss |
| TCGA-09-1664 | 16 | 53652908 | 53907797 | 0.736872033 | 1 | loss |
| TCGA-09-1664 | 16 | 55568284 | 55617037 | 0.789469865 | 1 | loss |
| TCGA-09-1664 | 16 | 56875560 | 57207784 | 1.169170984 | 3 | amp |
| TCGA-09-1664 | 16 | 57238535 | 57254761 | 0.757207414 | 1 | loss |
| TCGA-09-1664 | 16 | 57255135 | 57973366 | 1.165304549 | 3 | amp |
| TCGA-09-1664 | 16 | 57973488 | 57984484 | 1.307164608 | 4 | amp |
| TCGA-09-1664 | 16 | 71748626 | 71790111 | 0.769494549 | 1 | loss |
| TCGA-09-1664 | 16 | 74426966 | 74493693 | 1.238093534 | 3 | amp |
| TCGA-09-1664 | 16 | 76513315 | 76573802 | 0.682654983 | 1 | loss |
| TCGA-09-1664 | 16 | 83250905 | 83704605 | 0.704665225 | 1 | loss |
| TCGA-09-1664 | 16 | 84767014 | 84793584 | 0.700255113 | 1 | loss |
| TCGA-09-1664 | 17 | 9066073 | 21731390 | 0.542025257 | 1 | loss |
| TCGA-09-1664 | 17 | 27244289 | 45699329 | 0.567204297 | 1 | loss |
| TCGA-09-1664 | 17 | 67148103 | 67303100 | 0.75081203 | 1 | loss |
| TCGA-09-1664 | 19 | 71882 | 8999102 | 0.514996671 | 1 | loss |
| TCGA-09-1664 | 19 | 8999357 | 9091816 | 0.410439428 | 0 | loss |
| TCGA-09-1664 | 19 | 20278094 | 23837547 | 0.783647917 | 1 | loss |
| TCGA-09-1664 | 19 | 33406215 | 55241285 | 0.63484836 | 1 | loss |
| TCGA-09-1664 | 19 | 55246711 | 55299739 | 0.189373861 | 0 | loss |
| TCGA-09-1664 | 19 | 55358559 | 59110878 | 0.581984999 | 1 | loss |
| TCGA-09-1664 | 2 | 11315054 | 11348063 | 0.755586315 | 1 | loss |
| TCGA-09-1664 | 2 | 15746055 | 15770293 | 0.708897693 | 1 | loss |
| TCGA-09-1664 | 2 | 25875435 | 26022448 | 0.66017573 | 1 | loss |
| TCGA-09-1664 | 2 | 29344200 | 29397334 | 0.737288843 | 1 | loss |
| TCGA-09-1664 | 2 | 32530506 | 32832735 | 0.678411297 | 1 | loss |
| TCGA-09-1664 | 2 | 38177639 | 38294188 | 0.737693276 | 1 | loss |
| TCGA-09-1664 | 2 | 43019586 | 43933567 | 0.745542019 | 1 | loss |
| TCGA-09-1664 | 2 | 61447398 | 61610497 | 0.776737076 | 1 | loss |
| TCGA-09-1664 | 2 | 71592517 | 71661964 | 0.772841561 | 1 | loss |
| TCGA-09-1664 | 2 | 72406406 | 72968635 | 0.716141738 | 1 | loss |
| TCGA-09-1664 | 2 | 74592164 | 74604873 | 1.238878322 | 3 | amp |
| TCGA-09-1664 | 2 | 87180060 | 87209829 | 0.750043973 | 1 | loss |
| TCGA-09-1664 | 2 | 88084580 | 88116500 | 0.750864465 | 1 | loss |
| TCGA-09-1664 | 2 | 89160356 | 90260280 | 0.750242362 | 1 | loss |
| TCGA-09-1664 | 2 | 96517848 | 96595060 | 0.776828398 | 1 | loss |
| TCGA-09-1664 | 2 | 97275207 | 97279410 | 0.617250737 | 1 | loss |
| TCGA-09-1664 | 2 | 97779454 | 98273431 | 0.678972669 | 1 | loss |
| TCGA-09-1664 | 2 | 98413783 | 98475948 | 0.748815775 | 1 | loss |
| TCGA-09-1664 | 2 | 107042436 | 107074138 | 0.757974851 | 1 | loss |
| TCGA-09-1664 | 2 | 111293979 | 111407002 | 0.756991293 | 1 | loss |
| TCGA-09-1664 | 2 | 114697487 | 114713301 | 0.669603194 | 1 | loss |
| TCGA-09-1664 | 2 | 120567378 | 120714712 | 0.749897895 | 1 | loss |
| TCGA-09-1664 | 2 | 122159046 | 122227908 | 0.755030935 | 1 | loss |
| TCGA-09-1664 | 2 | 128065147 | 128186527 | 0.739599822 | 1 | loss |

| | | | | | | |
|---|---|---|---|---|---|---|
| TCGA-09-1664 | 2 | 133483145 | 134275122 | 0.744965488 | 1 | loss |
| TCGA-09-1664 | 2 | 136740935 | 138727820 | 0.738919279 | 1 | loss |
| TCGA-09-1664 | 2 | 139326457 | 141245366 | 0.775319007 | 1 | loss |
| TCGA-09-1664 | 2 | 148696702 | 148733558 | 0.708479443 | 1 | loss |
| TCGA-09-1664 | 2 | 152316453 | 155115712 | 0.73845606 | 1 | loss |
| TCGA-09-1664 | 2 | 162081139 | 162834297 | 0.745249906 | 1 | loss |
| TCGA-09-1664 | 2 | 162895404 | 163005702 | 0.748667659 | 1 | loss |
| TCGA-09-1664 | 2 | 163134656 | 163236566 | 0.744053814 | 1 | loss |
| TCGA-09-1664 | 2 | 166770059 | 166866409 | 0.758794025 | 1 | loss |
| TCGA-09-1664 | 2 | 166905381 | 167334209 | 0.705310367 | 1 | loss |
| TCGA-09-1664 | 2 | 169780111 | 169874657 | 0.734242746 | 1 | loss |
| TCGA-09-1664 | 2 | 170344280 | 170676171 | 0.739020227 | 1 | loss |
| TCGA-09-1664 | 2 | 171854187 | 172779073 | 0.769202978 | 1 | loss |
| TCGA-09-1664 | 2 | 175673581 | 176043132 | 0.742700321 | 1 | loss |
| TCGA-09-1664 | 2 | 177162539 | 177195409 | 0.65649048 | 1 | loss |
| TCGA-09-1664 | 2 | 179334325 | 179629086 | 0.653485315 | 1 | loss |
| TCGA-09-1664 | 2 | 180809848 | 182396563 | 0.759894839 | 1 | loss |
| TCGA-09-1664 | 2 | 182761602 | 183011881 | 0.736250745 | 1 | loss |
| TCGA-09-1664 | 2 | 183859500 | 183948318 | 0.733781875 | 1 | loss |
| TCGA-09-1664 | 2 | 187455037 | 188245518 | 0.771730936 | 1 | loss |
| TCGA-09-1664 | 2 | 189850357 | 189964908 | 0.78247516 | 1 | loss |
| TCGA-09-1664 | 2 | 190439816 | 190729011 | 0.781235496 | 1 | loss |
| TCGA-09-1664 | 2 | 191300711 | 191797634 | 0.748442705 | 1 | loss |
| TCGA-09-1664 | 2 | 192194617 | 192275904 | 0.745639019 | 1 | loss |
| TCGA-09-1664 | 2 | 197504436 | 197597287 | 0.71993277 | 1 | loss |
| TCGA-09-1664 | 2 | 202340253 | 202469436 | 0.750527973 | 1 | loss |
| TCGA-09-1664 | 2 | 225651735 | 225750960 | 0.770648757 | 1 | loss |
| TCGA-09-1664 | 2 | 230633293 | 230683235 | 0.773863392 | 1 | loss |
| TCGA-09-1664 | 2 | 234386033 | 234433232 | 0.707956692 | 1 | loss |
| TCGA-09-1664 | 20 | 5952607 | 5968025 | 0.775517789 | 1 | loss |
| TCGA-09-1664 | 20 | 8608889 | 8665771 | 0.78520702 | 1 | loss |
| TCGA-09-1664 | 20 | 13747347 | 13763827 | 0.739372865 | 1 | loss |
| TCGA-09-1664 | 20 | 15843370 | 15967468 | 0.767142669 | 1 | loss |
| TCGA-09-1664 | 20 | 20517243 | 23016226 | 0.770566303 | 1 | loss |
| TCGA-09-1664 | 20 | 25733291 | 29632725 | 0.761133611 | 1 | loss |
| TCGA-09-1664 | 20 | 31879693 | 31893899 | 1.387905571 | 4 | amp |
| TCGA-09-1664 | 20 | 43058083 | 43129947 | 1.338870923 | 3 | amp |
| TCGA-09-1664 | 20 | 43584986 | 43615974 | 0.745684186 | 1 | loss |
| TCGA-09-1664 | 20 | 47688786 | 47693618 | 0.744393618 | 1 | loss |
| TCGA-09-1664 | 20 | 49518509 | 49565242 | 0.760107404 | 1 | loss |
| TCGA-09-1664 | 20 | 55940394 | 55949879 | 0.783177244 | 1 | loss |
| TCGA-09-1664 | 20 | 57473927 | 57564738 | 0.756008215 | 1 | loss |
| TCGA-09-1664 | 20 | 58415394 | 58453141 | 0.71427881 | 1 | loss |
| TCGA-09-1664 | 20 | 58456968 | 58494659 | 0.717476832 | 1 | loss |
| TCGA-09-1664 | 20 | 62891291 | 62905003 | 0.680418909 | 1 | loss |
| TCGA-09-1664 | 21 | 17183402 | 17603465 | 0.763859567 | 1 | loss |
| TCGA-09-1664 | 21 | 22696641 | 22910282 | 0.770479422 | 1 | loss |
| TCGA-09-1664 | 21 | 30400204 | 30414880 | 0.734071784 | 1 | loss |
| TCGA-09-1664 | 21 | 34931488 | 34957104 | 0.726593384 | 1 | loss |
| TCGA-09-1664 | 21 | 40572108 | 40597127 | 0.754010452 | 1 | loss |

| | | | | | | |
|---|---|---|---|---|---|---|
| TCGA-09-1664 | 21 | 40648058 | 40715121 | 0.739124599 | 1 | loss |
| TCGA-09-1664 | 21 | 45993548 | 46032651 | 1.31149173 | 3 | amp |
| TCGA-09-1664 | 22 | 16084594 | 18003367 | 0.559371249 | 1 | loss |
| TCGA-09-1664 | 22 | 18016733 | 18020534 | 0.356897126 | 0 | loss |
| TCGA-09-1664 | 22 | 18021471 | 19217549 | 0.565528511 | 1 | loss |
| TCGA-09-1664 | 22 | 19219939 | 19230498 | 0.360938771 | 0 | loss |
| TCGA-09-1664 | 22 | 19241435 | 22698106 | 0.578292002 | 1 | loss |
| TCGA-09-1664 | 22 | 22707445 | 22786835 | 0.373952949 | 0 | loss |
| TCGA-09-1664 | 22 | 22842067 | 24325812 | 0.563372657 | 1 | loss |
| TCGA-09-1664 | 22 | 24431906 | 51237627 | 0.571447921 | 1 | loss |
| TCGA-09-1664 | 3 | 9483230 | 9512617 | 0.73266021 | 1 | loss |
| TCGA-09-1664 | 3 | 15711739 | 15793351 | 0.736983861 | 1 | loss |
| TCGA-09-1664 | 3 | 16535183 | 16640151 | 0.751744734 | 1 | loss |
| TCGA-09-1664 | 3 | 17299950 | 17448023 | 0.753311937 | 1 | loss |
| TCGA-09-1664 | 3 | 19921089 | 20019908 | 0.75976146 | 1 | loss |
| TCGA-09-1664 | 3 | 25646245 | 25761158 | 0.705262543 | 1 | loss |
| TCGA-09-1664 | 3 | 27329151 | 28533700 | 0.772017015 | 1 | loss |
| TCGA-09-1664 | 3 | 33540105 | 33866875 | 0.701185709 | 1 | loss |
| TCGA-09-1664 | 3 | 42727086 | 42739205 | 1.186577776 | 3 | amp |
| TCGA-09-1664 | 3 | 48606753 | 48675711 | 1.148018826 | 3 | amp |
| TCGA-09-1664 | 3 | 50681751 | 51697478 | 0.731716914 | 1 | loss |
| TCGA-09-1664 | 3 | 53919526 | 56655662 | 0.766907681 | 1 | loss |
| TCGA-09-1664 | 3 | 57280056 | 57302633 | 0.747410657 | 1 | loss |
| TCGA-09-1664 | 3 | 69084129 | 69117195 | 0.761067283 | 1 | loss |
| TCGA-09-1664 | 3 | 77656906 | 81695647 | 0.743222381 | 1 | loss |
| TCGA-09-1664 | 3 | 96585640 | 97516940 | 0.73694183 | 1 | loss |
| TCGA-09-1664 | 3 | 98512471 | 98600617 | 0.711047347 | 1 | loss |
| TCGA-09-1664 | 3 | 100025243 | 100039837 | 0.672782539 | 1 | loss |
| TCGA-09-1664 | 3 | 100447508 | 100945878 | 0.786085039 | 1 | loss |
| TCGA-09-1664 | 3 | 101060436 | 101091030 | 0.735116123 | 1 | loss |
| TCGA-09-1664 | 3 | 113563302 | 113655320 | 0.758220062 | 1 | loss |
| TCGA-09-1664 | 3 | 122422586 | 122459964 | 0.758933846 | 1 | loss |
| TCGA-09-1664 | 3 | 129195133 | 129290723 | 1.145422875 | 3 | amp |
| TCGA-09-1664 | 3 | 129817111 | 130383953 | 0.715342321 | 1 | loss |
| TCGA-09-1664 | 3 | 132086546 | 132230119 | 0.713703971 | 1 | loss |
| TCGA-09-1664 | 3 | 132409282 | 132434115 | 0.759557775 | 1 | loss |
| TCGA-09-1664 | 3 | 138218918 | 138248320 | 0.734341399 | 1 | loss |
| TCGA-09-1664 | 3 | 141230943 | 141295954 | 0.712133704 | 1 | loss |
| TCGA-09-1664 | 3 | 142396824 | 142523039 | 0.740278144 | 1 | loss |
| TCGA-09-1664 | 3 | 142735095 | 142751826 | 0.705030536 | 1 | loss |
| TCGA-09-1664 | 3 | 145788461 | 146311936 | 0.785272412 | 1 | loss |
| TCGA-09-1664 | 3 | 149677822 | 149701129 | 0.688327769 | 1 | loss |
| TCGA-09-1664 | 3 | 155615712 | 155654332 | 0.660676181 | 1 | loss |
| TCGA-09-1664 | 3 | 160137118 | 160148578 | 0.729854384 | 1 | loss |
| TCGA-09-1664 | 3 | 160243549 | 160254628 | 0.650590381 | 1 | loss |
| TCGA-09-1664 | 3 | 160945022 | 160955999 | 0.694645867 | 1 | loss |
| TCGA-09-1664 | 3 | 164704909 | 167196844 | 0.772562951 | 1 | loss |
| TCGA-09-1664 | 3 | 169814974 | 169896748 | 0.736797652 | 1 | loss |
| TCGA-09-1664 | 3 | 170783925 | 171087501 | 0.781163224 | 1 | loss |
| TCGA-09-1664 | 3 | 174577158 | 176782796 | 0.714491815 | 1 | loss |

| | | | | | | |
|---|---|---|---|---|---|---|
| TCGA-09-1664 | 3 | 180349198 | 180680878 | 0.747258934 | 1 | loss |
| TCGA-09-1664 | 3 | 182537985 | 182587157 | 0.770162806 | 1 | loss |
| TCGA-09-1664 | 3 | 184573492 | 184700454 | 0.753594886 | 1 | loss |
| TCGA-09-1664 | 3 | 195015403 | 195057742 | 0.695331607 | 1 | loss |
| TCGA-09-1664 | 4 | 82348873 | 191006895 | 0.454761871 | 1 | loss |
| TCGA-09-1664 | 5 | 233555 | 236748 | 1.120351851 | 3 | amp |
| TCGA-09-1664 | 6 | 4052109 | 4077678 | 0.739171176 | 1 | loss |
| TCGA-09-1664 | 6 | 25279983 | 25606527 | 0.728895436 | 1 | loss |
| TCGA-09-1664 | 6 | 27293046 | 27425244 | 0.681576294 | 1 | loss |
| TCGA-09-1664 | 6 | 30550220 | 31752284 | 1.166400525 | 3 | amp |
| TCGA-09-1664 | 6 | 31994224 | 32010661 | 1.168920171 | 3 | amp |
| TCGA-09-1664 | 6 | 32317501 | 32339536 | 0.71436078 | 1 | loss |
| TCGA-09-1664 | 6 | 33652329 | 33662901 | 1.167028448 | 3 | amp |
| TCGA-09-1664 | 6 | 38913153 | 38952092 | 0.721147445 | 1 | loss |
| TCGA-09-1664 | 6 | 44115905 | 44122644 | 1.231565286 | 3 | amp |
| TCGA-09-1664 | 6 | 49399384 | 49423991 | 0.721067274 | 1 | loss |
| TCGA-09-1664 | 6 | 55929337 | 56490068 | 0.684307378 | 1 | loss |
| TCGA-09-1664 | 6 | 56510607 | 56857414 | 0.719242654 | 1 | loss |
| TCGA-09-1664 | 6 | 57183200 | 62611332 | 0.755324035 | 1 | loss |
| TCGA-09-1664 | 6 | 64029773 | 64412583 | 0.726140869 | 1 | loss |
| TCGA-09-1664 | 6 | 66044826 | 66547312 | 0.745476271 | 1 | loss |
| TCGA-09-1664 | 6 | 66804596 | 70064232 | 0.780947186 | 1 | loss |
| TCGA-09-1664 | 6 | 70744103 | 70852001 | 0.751397918 | 1 | loss |
| TCGA-09-1664 | 6 | 72002978 | 72011780 | 0.635871898 | 1 | loss |
| TCGA-09-1664 | 6 | 72957713 | 73110426 | 0.701605354 | 1 | loss |
| TCGA-09-1664 | 6 | 79654995 | 79770579 | 0.754266064 | 1 | loss |
| TCGA-09-1664 | 6 | 83806645 | 83831828 | 0.713298333 | 1 | loss |
| TCGA-09-1664 | 6 | 83861284 | 83867088 | 0.738368735 | 1 | loss |
| TCGA-09-1664 | 6 | 84862340 | 84873045 | 0.680141266 | 1 | loss |
| TCGA-09-1664 | 6 | 84910504 | 84930891 | 0.704441936 | 1 | loss |
| TCGA-09-1664 | 6 | 100957185 | 101077093 | 0.763263279 | 1 | loss |
| TCGA-09-1664 | 6 | 105219754 | 105259316 | 0.732433505 | 1 | loss |
| TCGA-09-1664 | 6 | 122734611 | 122779865 | 0.76116272 | 1 | loss |
| TCGA-09-1664 | 6 | 128329151 | 128404974 | 0.692473039 | 1 | loss |
| TCGA-09-1664 | 6 | 135306422 | 135318117 | 0.703825836 | 1 | loss |
| TCGA-09-1664 | 6 | 135611539 | 135811942 | 0.777997728 | 1 | loss |
| TCGA-09-1664 | 6 | 144768707 | 144784015 | 0.725295166 | 1 | loss |
| TCGA-09-1664 | 6 | 170110259 | 170143374 | 0.710763067 | 1 | loss |
| TCGA-09-1664 | 7 | 7572324 | 7646734 | 0.701702405 | 1 | loss |
| TCGA-09-1664 | 7 | 10977655 | 16446003 | 0.674887004 | 1 | loss |
| TCGA-09-1664 | 7 | 16655328 | 20706445 | 0.742452084 | 1 | loss |
| TCGA-09-1664 | 7 | 21521521 | 22349673 | 0.750254216 | 1 | loss |
| TCGA-09-1664 | 7 | 24663314 | 24727264 | 0.768015937 | 1 | loss |
| TCGA-09-1664 | 7 | 26232106 | 26237127 | 0.70977932 | 1 | loss |
| TCGA-09-1664 | 7 | 27668930 | 27827249 | 0.757174846 | 1 | loss |
| TCGA-09-1664 | 7 | 33217086 | 33545285 | 0.775175919 | 1 | loss |
| TCGA-09-1664 | 7 | 35053128 | 35204510 | 0.77494236 | 1 | loss |
| TCGA-09-1664 | 7 | 40037057 | 40498808 | 0.760201494 | 1 | loss |
| TCGA-09-1664 | 7 | 48258906 | 50358737 | 0.770814251 | 1 | loss |
| TCGA-09-1664 | 7 | 73111889 | 74005369 | 1.180621869 | 3 | amp |

| | | | | | | |
|---|---|---|---|---|---|---|
| TCGA-09-1664 | 7 | 77469534 | 77649332 | 0.691732186 | 1 | loss |
| TCGA-09-1664 | 7 | 77807283 | 83606539 | 0.761426172 | 1 | loss |
| TCGA-09-1664 | 7 | 87056030 | 87133781 | 0.785318418 | 1 | loss |
| TCGA-09-1664 | 7 | 87280135 | 87537530 | 0.754939299 | 1 | loss |
| TCGA-09-1664 | 7 | 89906350 | 92027224 | 0.793107393 | 1 | loss |
| TCGA-09-1664 | 7 | 94059511 | 94913548 | 0.77704919 | 1 | loss |
| TCGA-09-1664 | 7 | 100187738 | 100281550 | 1.185118523 | 3 | amp |
| TCGA-09-1664 | 7 | 100780263 | 100876062 | 1.30093161 | 3 | amp |
| TCGA-09-1664 | 7 | 103473931 | 104742666 | 0.794865557 | 1 | loss |
| TCGA-09-1664 | 7 | 106921734 | 107240955 | 0.728169718 | 1 | loss |
| TCGA-09-1664 | 7 | 107874989 | 111970324 | 0.789721615 | 1 | loss |
| TCGA-09-1664 | 7 | 114303479 | 116146238 | 0.794449469 | 1 | loss |
| TCGA-09-1664 | 7 | 121960185 | 122377125 | 0.758967441 | 1 | loss |
| TCGA-09-1664 | 7 | 123269976 | 123514919 | 0.771807424 | 1 | loss |
| TCGA-09-1664 | 7 | 130290040 | 130296020 | 0.637093921 | 1 | loss |
| TCGA-09-1664 | 7 | 141362433 | 141442058 | 0.776846749 | 1 | loss |
| TCGA-09-1664 | 7 | 141643639 | 141795558 | 0.77524605 | 1 | loss |
| TCGA-09-1664 | 7 | 141957417 | 142423720 | 0.793982056 | 1 | loss |
| TCGA-09-1664 | 7 | 151877788 | 151919178 | 0.772549825 | 1 | loss |
| TCGA-09-1664 | 7 | 154672564 | 155473622 | 0.787287329 | 1 | loss |
| TCGA-09-1664 | 8 | 2112069 | 3470858 | 0.722308549 | 1 | loss |
| TCGA-09-1664 | 8 | 7309747 | 7437706 | 0.710063303 | 1 | loss |
| TCGA-09-1664 | 8 | 7669280 | 41123026 | 0.54423855 | 1 | loss |
| TCGA-09-1664 | 8 | 71056859 | 71556508 | 0.699214149 | 1 | loss |
| TCGA-09-1664 | 8 | 76468200 | 79652336 | 0.730184474 | 1 | loss |
| TCGA-09-1664 | 8 | 87386224 | 87470261 | 0.66876327 | 1 | loss |
| TCGA-09-1664 | 8 | 110374785 | 110575694 | 0.726433054 | 1 | loss |
| TCGA-09-1664 | 9 | 33385605 | 33386590 | 1.083785596 | 3 | amp |
| TCGA-09-1672 | 1 | 8715977 | 12988102 | 0.987043148 | 3 | amp |
| TCGA-09-1672 | 1 | 12998485 | 13219590 | 1.352386369 | 4 | amp |
| TCGA-09-1672 | 1 | 13328819 | 16875383 | 0.999556062 | 3 | amp |
| TCGA-09-1672 | 1 | 16890379 | 16902989 | 1.603702257 | 4 | amp |
| TCGA-09-1672 | 1 | 16903786 | 17395775 | 0.852390942 | 3 | amp |
| TCGA-09-1672 | 1 | 17396526 | 17727937 | 1.394320651 | 4 | amp |
| TCGA-09-1672 | 1 | 17735547 | 19401417 | 1.10522931 | 3 | amp |
| TCGA-09-1672 | 1 | 19403164 | 19441493 | 1.387816947 | 4 | amp |
| TCGA-09-1672 | 1 | 19441831 | 22307425 | 0.961492467 | 3 | amp |
| TCGA-09-1672 | 1 | 22307447 | 22339041 | 1.8730755 | 5 | amp |
| TCGA-09-1672 | 1 | 22404959 | 24022908 | 0.989118445 | 3 | amp |
| TCGA-09-1672 | 1 | 24402645 | 35223762 | 0.955359865 | 3 | amp |
| TCGA-09-1672 | 1 | 46806454 | 48713230 | 0.975855273 | 3 | amp |
| TCGA-09-1672 | 1 | 54561901 | 55174777 | 1.168509297 | 3 | amp |
| TCGA-09-1672 | 1 | 57252774 | 58522158 | 1.228115196 | 4 | amp |
| TCGA-09-1672 | 1 | 63991186 | 65877140 | 1.017542482 | 3 | amp |
| TCGA-09-1672 | 1 | 70555395 | 86891210 | 0.610021023 | 1 | loss |
| TCGA-09-1672 | 1 | 90463630 | 94370171 | 0.523117061 | 1 | loss |
| TCGA-09-1672 | 1 | 100316528 | 100921064 | 0.561232511 | 1 | loss |
| TCGA-09-1672 | 1 | 107961174 | 109836925 | 0.564318742 | 1 | loss |
| TCGA-09-1672 | 1 | 109944583 | 111785037 | 0.56649287 | 1 | loss |
| TCGA-09-1672 | 1 | 115236002 | 115537691 | 0.527513789 | 1 | loss |

| | | | | | | |
|---|---|---|---|---|---|---|
| TCGA-09-1672 | 1 | 121129473 | 144094452 | 1.066401515 | 3 | amp |
| TCGA-09-1672 | 1 | 144146796 | 144158263 | 1.51437868 | 4 | amp |
| TCGA-09-1672 | 1 | 144158836 | 144224209 | 2.025242935 | 5 | amp |
| TCGA-09-1672 | 1 | 144363623 | 144906598 | 1.478297067 | 4 | amp |
| TCGA-09-1672 | 1 | 144909854 | 145209464 | 0.895250444 | 3 | amp |
| TCGA-09-1672 | 1 | 145248793 | 145301847 | 1.568545812 | 4 | amp |
| TCGA-09-1672 | 1 | 145302640 | 145368622 | 2.272202423 | 5 | amp |
| TCGA-09-1672 | 1 | 145414739 | 145508105 | 0.995958722 | 3 | amp |
| TCGA-09-1672 | 1 | 145508185 | 145538322 | 1.626466217 | 4 | amp |
| TCGA-09-1672 | 1 | 145538705 | 145539799 | 1.061878168 | 3 | amp |
| TCGA-09-1672 | 1 | 145686973 | 146024395 | 1.017986274 | 3 | amp |
| TCGA-09-1672 | 1 | 146034106 | 146038304 | 1.93928247 | 5 | amp |
| TCGA-09-1672 | 1 | 146038955 | 146080046 | 1.45305056 | 4 | amp |
| TCGA-09-1672 | 1 | 146215016 | 146234585 | 1.990673514 | 5 | amp |
| TCGA-09-1672 | 1 | 146238428 | 146414253 | 1.532322149 | 4 | amp |
| TCGA-09-1672 | 1 | 146418099 | 146466179 | 1.954870112 | 5 | amp |
| TCGA-09-1672 | 1 | 146491172 | 146747133 | 0.972945312 | 3 | amp |
| TCGA-09-1672 | 1 | 146747724 | 147381393 | 1.406529391 | 4 | amp |
| TCGA-09-1672 | 1 | 147400610 | 147566175 | 0.963620131 | 3 | amp |
| TCGA-09-1672 | 1 | 147575887 | 147580973 | 1.955004289 | 5 | amp |
| TCGA-09-1672 | 1 | 147581536 | 147955385 | 1.405087281 | 4 | amp |
| TCGA-09-1672 | 1 | 148004489 | 148024938 | 1.937997867 | 5 | amp |
| TCGA-09-1672 | 1 | 148025735 | 148252749 | 1.283171357 | 4 | amp |
| TCGA-09-1672 | 1 | 148253516 | 148328426 | 2.064007788 | 5 | amp |
| TCGA-09-1672 | 1 | 148328995 | 148754920 | 1.462057062 | 4 | amp |
| TCGA-09-1672 | 1 | 148755608 | 150234067 | 1.063750265 | 3 | amp |
| TCGA-09-1672 | 1 | 150234477 | 150236294 | 2.302480539 | 5 | amp |
| TCGA-09-1672 | 1 | 150236965 | 150464985 | 1.452630048 | 4 | amp |
| TCGA-09-1672 | 1 | 150468910 | 150471790 | 2.332224794 | 5 | amp |
| TCGA-09-1672 | 1 | 150476770 | 150484359 | 1.426267594 | 4 | amp |
| TCGA-09-1672 | 1 | 150484817 | 150830940 | 1.018318294 | 3 | amp |
| TCGA-09-1672 | 1 | 150899208 | 151006735 | 1.478935209 | 4 | amp |
| TCGA-09-1672 | 1 | 151009161 | 151137764 | 1.073522271 | 3 | amp |
| TCGA-09-1672 | 1 | 151138593 | 151317682 | 1.486064624 | 4 | amp |
| TCGA-09-1672 | 1 | 151318360 | 151345175 | 2.102529942 | 5 | amp |
| TCGA-09-1672 | 1 | 151371983 | 151542249 | 1.364496727 | 4 | amp |
| TCGA-09-1672 | 1 | 151546689 | 151755556 | 0.990701912 | 3 | amp |
| TCGA-09-1672 | 1 | 151773389 | 152857253 | 1.327769857 | 4 | amp |
| TCGA-09-1672 | 1 | 152882265 | 153012832 | 1.997160721 | 5 | amp |
| TCGA-09-1672 | 1 | 153028981 | 153615891 | 1.439097535 | 4 | amp |
| TCGA-09-1672 | 1 | 153655799 | 153660307 | 1.933458842 | 5 | amp |
| TCGA-09-1672 | 1 | 153660462 | 153740346 | 1.423349142 | 4 | amp |
| TCGA-09-1672 | 1 | 153741312 | 154141879 | 1.002600985 | 3 | amp |
| TCGA-09-1672 | 1 | 154142850 | 154173119 | 2.019461211 | 5 | amp |
| TCGA-09-1672 | 1 | 154178003 | 154247973 | 1.382366399 | 4 | amp |
| TCGA-09-1672 | 1 | 154248044 | 155208494 | 1.023619447 | 3 | amp |
| TCGA-09-1672 | 1 | 155307406 | 155311929 | 1.225261579 | 4 | amp |
| TCGA-09-1672 | 1 | 155313071 | 155348185 | 1.832333901 | 5 | amp |
| TCGA-09-1672 | 1 | 155348256 | 155727873 | 1.070731064 | 3 | amp |
| TCGA-09-1672 | 1 | 155730213 | 155886531 | 1.407097146 | 4 | amp |

| | | | | | | |
|---|---|---|---|---|---|---|
| TCGA-09-1672 | 1 | 155887224 | 156498910 | 1.028146048 | 3 | amp |
| TCGA-09-1672 | 1 | 156499882 | 156542362 | 1.965111799 | 5 | amp |
| TCGA-09-1672 | 1 | 156552131 | 156905880 | 0.963087413 | 3 | amp |
| TCGA-09-1672 | 1 | 156905889 | 157504722 | 1.470984832 | 4 | amp |
| TCGA-09-1672 | 1 | 157508804 | 157557387 | 1.930805392 | 5 | amp |
| TCGA-09-1672 | 1 | 157557601 | 159038510 | 1.541968584 | 4 | amp |
| TCGA-09-1672 | 1 | 159042978 | 159803165 | 1.794865278 | 5 | amp |
| TCGA-09-1672 | 1 | 159804921 | 159847255 | 1.224537293 | 4 | amp |
| TCGA-09-1672 | 1 | 159850296 | 159863152 | 1.987728309 | 5 | amp |
| TCGA-09-1672 | 1 | 159869826 | 160065087 | 0.979886734 | 3 | amp |
| TCGA-09-1672 | 1 | 160083249 | 160151830 | 2.336396071 | 5 | amp |
| TCGA-09-1672 | 1 | 160156021 | 160254939 | 1.482302211 | 4 | amp |
| TCGA-09-1672 | 1 | 160259886 | 160275425 | 1.957304482 | 5 | amp |
| TCGA-09-1672 | 1 | 160275460 | 160593990 | 1.479848647 | 4 | amp |
| TCGA-09-1672 | 1 | 160604364 | 160721246 | 1.921806733 | 5 | amp |
| TCGA-09-1672 | 1 | 160721946 | 160968916 | 1.307029712 | 4 | amp |
| TCGA-09-1672 | 1 | 160969101 | 160970613 | 2.36709682 | 5 | amp |
| TCGA-09-1672 | 1 | 160970773 | 161145903 | 0.854951471 | 3 | amp |
| TCGA-09-1672 | 1 | 161160870 | 161205821 | 2.147629042 | 5 | amp |
| TCGA-09-1672 | 1 | 161206241 | 161600924 | 1.341779918 | 4 | amp |
| TCGA-09-1672 | 1 | 161633027 | 161643071 | 2.211618563 | 5 | amp |
| TCGA-09-1672 | 1 | 161643222 | 161683202 | 1.414803683 | 4 | amp |
| TCGA-09-1672 | 1 | 161692387 | 161753892 | 0.931077111 | 3 | amp |
| TCGA-09-1672 | 1 | 161761142 | 162381859 | 1.419658807 | 4 | amp |
| TCGA-09-1672 | 1 | 162467714 | 162549425 | 0.86923483 | 3 | amp |
| TCGA-09-1672 | 1 | 162551042 | 163138162 | 1.465311302 | 4 | amp |
| TCGA-09-1672 | 1 | 163172543 | 164781426 | 0.945262438 | 3 | amp |
| TCGA-09-1672 | 1 | 164789304 | 165370693 | 1.396549529 | 4 | amp |
| TCGA-09-1672 | 1 | 165376011 | 165533081 | 2.070847467 | 5 | amp |
| TCGA-09-1672 | 1 | 165601433 | 167042818 | 1.00047739 | 3 | amp |
| TCGA-09-1672 | 1 | 167059412 | 167742658 | 1.329362249 | 4 | amp |
| TCGA-09-1672 | 1 | 167745291 | 167815125 | 0.902048986 | 3 | amp |
| TCGA-09-1672 | 1 | 167815181 | 167874424 | 1.527429994 | 4 | amp |
| TCGA-09-1672 | 1 | 167887518 | 168208443 | 0.920127757 | 3 | amp |
| TCGA-09-1672 | 1 | 168211698 | 169100841 | 1.401897245 | 4 | amp |
| TCGA-09-1672 | 1 | 169101365 | 169483743 | 1.072322898 | 3 | amp |
| TCGA-09-1672 | 1 | 169484622 | 169564142 | 1.390697922 | 4 | amp |
| TCGA-09-1672 | 1 | 169565117 | 169581667 | 2.136309565 | 5 | amp |
| TCGA-09-1672 | 1 | 169582139 | 169701089 | 1.438088721 | 4 | amp |
| TCGA-09-1672 | 1 | 169701677 | 170024631 | 1.028322567 | 3 | amp |
| TCGA-09-1672 | 1 | 170043532 | 171311002 | 1.407360468 | 4 | amp |
| TCGA-09-1672 | 1 | 171481223 | 173840288 | 1.071453283 | 3 | amp |
| TCGA-09-1672 | 1 | 173842535 | 173939758 | 1.492571142 | 4 | amp |
| TCGA-09-1672 | 1 | 173941585 | 175105088 | 0.945917431 | 3 | amp |
| TCGA-09-1672 | 1 | 175105920 | 175335322 | 2.118326085 | 5 | amp |
| TCGA-09-1672 | 1 | 175336328 | 175937724 | 1.639470983 | 4 | amp |
| TCGA-09-1672 | 1 | 175956038 | 176176150 | 0.885401312 | 3 | amp |
| TCGA-09-1672 | 1 | 176525407 | 176984028 | 1.914102206 | 5 | amp |
| TCGA-09-1672 | 1 | 176992503 | 178490437 | 1.476435367 | 4 | amp |
| TCGA-09-1672 | 1 | 178491457 | 181453193 | 1.077425392 | 3 | amp |

| | | | | | | |
|---|---|---|---|---|---|---|
| TCGA-09-1672 | 1 | 181479605 | 181680207 | 2.246208856 | 5 | amp |
| TCGA-09-1672 | 1 | 181684440 | 181719677 | 1.367942308 | 4 | amp |
| TCGA-09-1672 | 1 | 181721266 | 181752927 | 1.918305175 | 5 | amp |
| TCGA-09-1672 | 1 | 181753790 | 182642016 | 1.331877388 | 4 | amp |
| TCGA-09-1672 | 1 | 182763450 | 182921999 | 1.001956449 | 3 | amp |
| TCGA-09-1672 | 1 | 183072404 | 183091411 | 1.832342486 | 5 | amp |
| TCGA-09-1672 | 1 | 183093708 | 183111976 | 1.514610067 | 4 | amp |
| TCGA-09-1672 | 1 | 183176989 | 183230230 | 1.935443771 | 5 | amp |
| TCGA-09-1672 | 1 | 183230327 | 183525368 | 1.373459514 | 4 | amp |
| TCGA-09-1672 | 1 | 183529169 | 183559497 | 2.032440002 | 5 | amp |
| TCGA-09-1672 | 1 | 183596607 | 184672157 | 1.406960822 | 4 | amp |
| TCGA-09-1672 | 1 | 184675739 | 184723696 | 0.933280299 | 3 | amp |
| TCGA-09-1672 | 1 | 184764070 | 184868496 | 1.574191628 | 4 | amp |
| TCGA-09-1672 | 1 | 185056668 | 185704225 | 0.85289982 | 3 | amp |
| TCGA-09-1672 | 1 | 185815132 | 186159066 | 1.45551495 | 4 | amp |
| TCGA-09-1672 | 1 | 186265985 | 201027637 | 1.026665837 | 3 | amp |
| TCGA-09-1672 | 1 | 201028281 | 201253051 | 1.311532971 | 4 | amp |
| TCGA-09-1672 | 1 | 201263061 | 201297986 | 1.984765796 | 5 | amp |
| TCGA-09-1672 | 1 | 201328300 | 202573780 | 1.432420055 | 4 | amp |
| TCGA-09-1672 | 1 | 202574691 | 203098319 | 0.946105268 | 3 | amp |
| TCGA-09-1672 | 1 | 203134318 | 203311666 | 1.529342426 | 4 | amp |
| TCGA-09-1672 | 1 | 203316368 | 203743825 | 2.198796183 | 5 | amp |
| TCGA-09-1672 | 1 | 203786165 | 205156940 | 1.440240478 | 4 | amp |
| TCGA-09-1672 | 1 | 205210599 | 205801929 | 1.144360596 | 3 | amp |
| TCGA-09-1672 | 1 | 205809325 | 205905005 | 2.018989781 | 5 | amp |
| TCGA-09-1672 | 1 | 206139260 | 206240224 | 0.886305577 | 3 | amp |
| TCGA-09-1672 | 1 | 206241484 | 206331244 | 1.902831732 | 5 | amp |
| TCGA-09-1672 | 1 | 206516160 | 206758629 | 1.473659967 | 4 | amp |
| TCGA-09-1672 | 1 | 206760083 | 206902471 | 0.949015354 | 3 | amp |
| TCGA-09-1672 | 1 | 206902690 | 207072882 | 1.903507958 | 5 | amp |
| TCGA-09-1672 | 1 | 207073610 | 207495939 | 1.417699792 | 4 | amp |
| TCGA-09-1672 | 1 | 207497849 | 207685016 | 0.921178216 | 3 | amp |
| TCGA-09-1672 | 1 | 207696914 | 207753966 | 2.206722894 | 5 | amp |
| TCGA-09-1672 | 1 | 207755245 | 207791623 | 1.438177456 | 4 | amp |
| TCGA-09-1672 | 1 | 207793197 | 207857319 | 0.844884412 | 3 | amp |
| TCGA-09-1672 | 1 | 207867656 | 207891042 | 1.831862557 | 5 | amp |
| TCGA-09-1672 | 1 | 207896936 | 208050384 | 0.830007865 | 3 | amp |
| TCGA-09-1672 | 1 | 208061055 | 208200695 | 1.572982326 | 4 | amp |
| TCGA-09-1672 | 1 | 208201308 | 208391273 | 1.992262428 | 5 | amp |
| TCGA-09-1672 | 1 | 208870809 | 210016043 | 1.41014744 | 4 | amp |
| TCGA-09-1672 | 1 | 210016738 | 215785274 | 1.049927393 | 3 | amp |
| TCGA-09-1672 | 1 | 215792199 | 217604716 | 1.425528906 | 4 | amp |
| TCGA-09-1672 | 1 | 217622574 | 222825681 | 1.072768167 | 3 | amp |
| TCGA-09-1672 | 1 | 222826290 | 222843653 | 1.515144026 | 4 | amp |
| TCGA-09-1672 | 1 | 222846595 | 226550890 | 1.052947045 | 3 | amp |
| TCGA-09-1672 | 1 | 226551587 | 227239693 | 1.281224633 | 4 | amp |
| TCGA-09-1672 | 1 | 227247032 | 227300672 | 1.044955105 | 3 | amp |
| TCGA-09-1672 | 1 | 229661622 | 229676545 | 1.055164203 | 3 | amp |
| TCGA-09-1672 | 1 | 229677930 | 230979670 | 1.423173795 | 4 | amp |
| TCGA-09-1672 | 1 | 230991378 | 231700702 | 1.09898444 | 3 | amp |

| | | | | | | |
|---|---|---|---|---|---|---|
| TCGA-09-1672 | 1 | 231829521 | 234528349 | 1.367944271 | 4 | amp |
| TCGA-09-1672 | 1 | 234529072 | 235506126 | 0.955677132 | 3 | amp |
| TCGA-09-1672 | 1 | 235543354 | 235904944 | 1.410785816 | 4 | amp |
| TCGA-09-1672 | 1 | 235907247 | 236590807 | 1.157050674 | 3 | amp |
| TCGA-09-1672 | 1 | 236631493 | 237780805 | 1.297277515 | 4 | amp |
| TCGA-09-1672 | 1 | 237787057 | 241752150 | 1.161707564 | 3 | amp |
| TCGA-09-1672 | 1 | 241753304 | 241958608 | 1.504594988 | 4 | amp |
| TCGA-09-1672 | 1 | 242052714 | 243349762 | 1.355903255 | 4 | amp |
| TCGA-09-1672 | 1 | 243354317 | 247582405 | 1.008451909 | 3 | amp |
| TCGA-09-1672 | 1 | 247586471 | 247607478 | 2.040709839 | 5 | amp |
| TCGA-09-1672 | 1 | 247607887 | 248814188 | 1.585757426 | 4 | amp |
| TCGA-09-1672 | 10 | 92880 | 4889760 | 1.071647697 | 3 | amp |
| TCGA-09-1672 | 10 | 5005618 | 5255159 | 1.647943165 | 4 | amp |
| TCGA-09-1672 | 10 | 5258654 | 5842674 | 1.07061361 | 3 | amp |
| TCGA-09-1672 | 10 | 5920021 | 6104142 | 1.431140065 | 4 | amp |
| TCGA-09-1672 | 10 | 6138969 | 6266011 | 1.011166072 | 3 | amp |
| TCGA-09-1672 | 10 | 6266053 | 7791343 | 1.457260723 | 4 | amp |
| TCGA-09-1672 | 10 | 7797958 | 7866581 | 0.950399102 | 3 | amp |
| TCGA-09-1672 | 10 | 8005848 | 12858347 | 1.372993933 | 4 | amp |
| TCGA-09-1672 | 10 | 12866417 | 13225136 | 1.10795038 | 3 | amp |
| TCGA-09-1672 | 10 | 13228101 | 14867668 | 1.549593129 | 4 | amp |
| TCGA-09-1672 | 10 | 14870087 | 14970202 | 0.985897548 | 3 | amp |
| TCGA-09-1672 | 10 | 14974843 | 15858933 | 1.361489918 | 4 | amp |
| TCGA-09-1672 | 10 | 15863599 | 16556736 | 1.075579889 | 3 | amp |
| TCGA-09-1672 | 10 | 16562474 | 18903585 | 1.441075628 | 4 | amp |
| TCGA-09-1672 | 10 | 20290689 | 21124556 | 1.503403214 | 4 | amp |
| TCGA-09-1672 | 10 | 21129662 | 23257427 | 1.003023371 | 3 | amp |
| TCGA-09-1672 | 10 | 23270224 | 24820960 | 1.448914362 | 4 | amp |
| TCGA-09-1672 | 10 | 24821932 | 24875087 | 2.088517228 | 5 | amp |
| TCGA-09-1672 | 10 | 24878125 | 24889896 | 1.364016918 | 4 | amp |
| TCGA-09-1672 | 10 | 24890819 | 26558134 | 1.130159176 | 3 | amp |
| TCGA-09-1672 | 10 | 26559506 | 26802648 | 1.592361822 | 4 | amp |
| TCGA-09-1672 | 10 | 26822350 | 26998751 | 0.96107292 | 3 | amp |
| TCGA-09-1672 | 10 | 27009067 | 27035451 | 1.576474667 | 4 | amp |
| TCGA-09-1672 | 10 | 27037466 | 27456236 | 1.068320938 | 3 | amp |
| TCGA-09-1672 | 10 | 27458842 | 27508838 | 1.42454146 | 4 | amp |
| TCGA-09-1672 | 10 | 27512263 | 28971360 | 1.164076443 | 3 | amp |
| TCGA-09-1672 | 10 | 29188170 | 32120757 | 1.47796631 | 4 | amp |
| TCGA-09-1672 | 10 | 32128141 | 33221545 | 1.040377779 | 3 | amp |
| TCGA-09-1672 | 10 | 33224392 | 34759221 | 1.409252654 | 4 | amp |
| TCGA-09-1672 | 10 | 34805876 | 46240359 | 1.115210978 | 3 | amp |
| TCGA-09-1672 | 10 | 46242011 | 46322880 | 1.56811506 | 4 | amp |
| TCGA-09-1672 | 10 | 46323442 | 46657982 | 1.102520021 | 3 | amp |
| TCGA-09-1672 | 10 | 46663895 | 46675574 | 1.852486308 | 5 | amp |
| TCGA-09-1672 | 10 | 46678632 | 47727853 | 1.04058708 | 3 | amp |
| TCGA-09-1672 | 10 | 47728415 | 48190077 | 1.411888026 | 4 | amp |
| TCGA-09-1672 | 10 | 48235776 | 48902932 | 1.338418235 | 4 | amp |
| TCGA-09-1672 | 10 | 48903504 | 49348127 | 1.110296894 | 3 | amp |
| TCGA-09-1672 | 10 | 49365328 | 50945927 | 1.42453507 | 4 | amp |
| TCGA-09-1672 | 10 | 50945935 | 51845347 | 1.138529233 | 3 | amp |

| | | | | | | |
|---|---|---|---|---|---|---|
| TCGA-09-1672 | 10 | 51846998 | 53667358 | 1.400989264 | 4 | amp |
| TCGA-09-1672 | 10 | 53814222 | 60154205 | 1.083415699 | 3 | amp |
| TCGA-09-1672 | 10 | 60154640 | 63959626 | 1.455788478 | 4 | amp |
| TCGA-09-1672 | 10 | 63964548 | 67748562 | 1.166924799 | 3 | amp |
| TCGA-09-1672 | 10 | 67829036 | 69571457 | 1.393045991 | 4 | amp |
| TCGA-09-1672 | 10 | 69583050 | 69882126 | 1.080198975 | 3 | amp |
| TCGA-09-1672 | 10 | 69902634 | 69970247 | 1.538848361 | 4 | amp |
| TCGA-09-1672 | 10 | 70043899 | 71026559 | 1.124909225 | 3 | amp |
| TCGA-09-1672 | 10 | 71048388 | 71634961 | 1.58841949 | 4 | amp |
| TCGA-09-1672 | 10 | 71640221 | 71678890 | 2.096022692 | 5 | amp |
| TCGA-09-1672 | 10 | 71686794 | 71912409 | 1.541230993 | 4 | amp |
| TCGA-09-1672 | 10 | 71913539 | 72192887 | 1.129603739 | 3 | amp |
| TCGA-09-1672 | 10 | 72195030 | 72543174 | 1.339402753 | 4 | amp |
| TCGA-09-1672 | 10 | 72576532 | 72637141 | 0.92526604 | 3 | amp |
| TCGA-09-1672 | 10 | 72643665 | 74769672 | 1.43423844 | 4 | amp |
| TCGA-09-1672 | 10 | 74770700 | 75286591 | 1.157834134 | 3 | amp |
| TCGA-09-1672 | 10 | 75289403 | 75397710 | 1.668165122 | 4 | amp |
| TCGA-09-1672 | 10 | 75399678 | 75802965 | 1.053784189 | 3 | amp |
| TCGA-09-1672 | 10 | 75830382 | 75877973 | 1.483826987 | 4 | amp |
| TCGA-09-1672 | 10 | 75883557 | 76732450 | 1.06206364 | 3 | amp |
| TCGA-09-1672 | 10 | 76735142 | 76936037 | 1.348737129 | 4 | amp |
| TCGA-09-1672 | 10 | 76971961 | 77159838 | 0.926478288 | 3 | amp |
| TCGA-09-1672 | 10 | 77160028 | 77807111 | 1.491011709 | 4 | amp |
| TCGA-09-1672 | 10 | 77818332 | 78729838 | 1.987480462 | 5 | amp |
| TCGA-09-1672 | 10 | 78771702 | 81373920 | 1.509394004 | 4 | amp |
| TCGA-09-1672 | 10 | 81463316 | 81666538 | 0.975323719 | 3 | amp |
| TCGA-09-1672 | 10 | 81697555 | 85944539 | 1.319348169 | 4 | amp |
| TCGA-09-1672 | 10 | 85955237 | 86008852 | 1.137733938 | 3 | amp |
| TCGA-09-1672 | 10 | 86012569 | 88197784 | 1.486816904 | 4 | amp |
| TCGA-09-1672 | 10 | 88203015 | 88277876 | 0.871636463 | 3 | amp |
| TCGA-09-1672 | 10 | 88414462 | 88425548 | 1.636489194 | 4 | amp |
| TCGA-09-1672 | 10 | 88428434 | 88730345 | 1.071637127 | 3 | amp |
| TCGA-09-1672 | 10 | 88760130 | 88811687 | 1.693189114 | 5 | amp |
| TCGA-09-1672 | 10 | 88813069 | 89419811 | 0.929265033 | 3 | amp |
| TCGA-09-1672 | 10 | 89463455 | 89516732 | 1.465016652 | 4 | amp |
| TCGA-09-1672 | 10 | 89527386 | 90365485 | 1.146012037 | 3 | amp |
| TCGA-09-1672 | 10 | 90366427 | 90733124 | 1.3736759 | 4 | amp |
| TCGA-09-1672 | 10 | 90749220 | 91061942 | 0.997379217 | 3 | amp |
| TCGA-09-1672 | 10 | 91065675 | 91465244 | 1.368899857 | 4 | amp |
| TCGA-09-1672 | 10 | 91468876 | 91522613 | 1.068155703 | 3 | amp |
| TCGA-09-1672 | 10 | 91528037 | 92680135 | 1.326276847 | 4 | amp |
| TCGA-09-1672 | 10 | 92680710 | 94071112 | 1.082559475 | 3 | amp |
| TCGA-09-1672 | 10 | 94100374 | 94268685 | 1.531062108 | 4 | amp |
| TCGA-09-1672 | 10 | 94269772 | 95069970 | 1.044645202 | 3 | amp |
| TCGA-09-1672 | 10 | 95070285 | 95088713 | 2.289328046 | 5 | amp |
| TCGA-09-1672 | 10 | 95089385 | 95216726 | 1.616954278 | 4 | amp |
| TCGA-09-1672 | 10 | 95241847 | 95373022 | 1.012305652 | 3 | amp |
| TCGA-09-1672 | 10 | 95380344 | 95429663 | 1.45698953 | 4 | amp |
| TCGA-09-1672 | 10 | 95430505 | 95720612 | 0.968062331 | 3 | amp |
| TCGA-09-1672 | 10 | 95790746 | 96084862 | 1.674077195 | 4 | amp |

| | | | | | | |
|---|---|---|---|---|---|---|
| TCGA-09-1672 | 10 | 96087680 | 96541785 | 1.062885949 | 3 | amp |
| TCGA-09-1672 | 10 | 96580203 | 97192388 | 1.46006696 | 4 | amp |
| TCGA-09-1672 | 10 | 97194366 | 97373940 | 2.082003455 | 5 | amp |
| TCGA-09-1672 | 10 | 97376182 | 98064475 | 1.536991071 | 4 | amp |
| TCGA-09-1672 | 10 | 98078045 | 98282113 | 1.853161916 | 5 | amp |
| TCGA-09-1672 | 10 | 98286947 | 98355422 | 1.083719252 | 3 | amp |
| TCGA-09-1672 | 10 | 98361975 | 98380331 | 2.221795351 | 5 | amp |
| TCGA-09-1672 | 10 | 98383171 | 98989635 | 1.615223812 | 4 | amp |
| TCGA-09-1672 | 10 | 98994932 | 99161038 | 1.924713528 | 5 | amp |
| TCGA-09-1672 | 10 | 99186013 | 99205786 | 0.863047178 | 3 | amp |
| TCGA-09-1672 | 10 | 99211343 | 100022817 | 1.444866852 | 4 | amp |
| TCGA-09-1672 | 10 | 100143499 | 100152919 | 2.380118862 | 5 | amp |
| TCGA-09-1672 | 10 | 100154941 | 101579055 | 1.429023833 | 4 | amp |
| TCGA-09-1672 | 10 | 101590006 | 101668926 | 0.901509044 | 3 | amp |
| TCGA-09-1672 | 10 | 101714946 | 101912118 | 1.600291314 | 4 | amp |
| TCGA-09-1672 | 10 | 101914596 | 102050336 | 0.889800946 | 3 | amp |
| TCGA-09-1672 | 10 | 102050970 | 102059531 | 1.537932971 | 4 | amp |
| TCGA-09-1672 | 10 | 102088964 | 103904871 | 1.13027418 | 3 | amp |
| TCGA-09-1672 | 10 | 103906368 | 104019899 | 1.353110141 | 4 | amp |
| TCGA-09-1672 | 10 | 104103753 | 104130247 | 2.030381213 | 5 | amp |
| TCGA-09-1672 | 10 | 104130420 | 104139462 | 1.449426116 | 4 | amp |
| TCGA-09-1672 | 10 | 104139600 | 104269177 | 0.951054396 | 3 | amp |
| TCGA-09-1672 | 10 | 104309674 | 104486948 | 1.451873124 | 4 | amp |
| TCGA-09-1672 | 10 | 104488146 | 104614235 | 2.03343782 | 5 | amp |
| TCGA-09-1672 | 10 | 104620059 | 105160339 | 0.951076251 | 3 | amp |
| TCGA-09-1672 | 10 | 105162838 | 105215561 | 1.475463051 | 4 | amp |
| TCGA-09-1672 | 10 | 105217900 | 105452859 | 1.009570745 | 3 | amp |
| TCGA-09-1672 | 10 | 105483983 | 105838381 | 1.348251381 | 4 | amp |
| TCGA-09-1672 | 10 | 105840345 | 106118419 | 1.094765673 | 3 | amp |
| TCGA-09-1672 | 10 | 106121734 | 106581917 | 1.54673072 | 4 | amp |
| TCGA-09-1672 | 10 | 106602523 | 106918760 | 2.023660923 | 5 | amp |
| TCGA-09-1672 | 10 | 106924038 | 107023159 | 1.59816344 | 4 | amp |
| TCGA-09-1672 | 10 | 107446166 | 108434920 | 2.018739916 | 5 | amp |
| TCGA-09-1672 | 10 | 108437026 | 111890302 | 1.538051157 | 4 | amp |
| TCGA-09-1672 | 10 | 111892020 | 113935474 | 1.055529062 | 3 | amp |
| TCGA-09-1672 | 10 | 113937718 | 114925762 | 1.557927738 | 4 | amp |
| TCGA-09-1672 | 10 | 115312854 | 115417340 | 2.124017161 | 5 | amp |
| TCGA-09-1672 | 10 | 115422421 | 115540420 | 1.561261412 | 4 | amp |
| TCGA-09-1672 | 10 | 115594870 | 115639513 | 0.844728775 | 3 | amp |
| TCGA-09-1672 | 10 | 115643954 | 115897056 | 1.410692814 | 4 | amp |
| TCGA-09-1672 | 10 | 115904214 | 115982523 | 1.077508688 | 3 | amp |
| TCGA-09-1672 | 10 | 115985771 | 116444140 | 1.568354758 | 4 | amp |
| TCGA-09-1672 | 10 | 116590589 | 117185845 | 1.040001723 | 3 | amp |
| TCGA-09-1672 | 10 | 117221407 | 118352110 | 1.338040288 | 4 | amp |
| TCGA-09-1672 | 10 | 118353780 | 121652495 | 1.074916099 | 3 | amp |
| TCGA-09-1672 | 10 | 121657904 | 123549863 | 1.433654163 | 4 | amp |
| TCGA-09-1672 | 10 | 123596222 | 123727365 | 0.969300121 | 3 | amp |
| TCGA-09-1672 | 10 | 123730368 | 124320376 | 1.583262476 | 4 | amp |
| TCGA-09-1672 | 10 | 124325437 | 124390816 | 2.805727676 | 5 | amp |
| TCGA-09-1672 | 10 | 124391320 | 124672493 | 1.582448058 | 4 | amp |

| | | | | | | |
|---|---|---|---|---|---|---|
| TCGA-09-1672 | 10 | 124691939 | 124923403 | 1.019682306 | 3 | amp |
| TCGA-09-1672 | 10 | 124924507 | 126395303 | 1.404613055 | 4 | amp |
| TCGA-09-1672 | 10 | 126449011 | 127526985 | 1.183578455 | 3 | amp |
| TCGA-09-1672 | 10 | 127527513 | 129910114 | 1.366442332 | 4 | amp |
| TCGA-09-1672 | 10 | 129910129 | 135516111 | 0.902336331 | 3 | amp |
| TCGA-09-1672 | 11 | 86637 | 1580351 | 0.611878214 | 1 | loss |
| TCGA-09-1672 | 11 | 2980949 | 3988966 | 0.993434586 | 3 | amp |
| TCGA-09-1672 | 11 | 4045099 | 4123326 | 1.380473474 | 4 | amp |
| TCGA-09-1672 | 11 | 4388572 | 5686631 | 1.34279958 | 4 | amp |
| TCGA-09-1672 | 11 | 5686674 | 5730880 | 0.918516799 | 3 | amp |
| TCGA-09-1672 | 11 | 5757663 | 6078950 | 1.465772713 | 4 | amp |
| TCGA-09-1672 | 11 | 6079190 | 6245797 | 1.072954106 | 3 | amp |
| TCGA-09-1672 | 11 | 6704290 | 7631737 | 1.293258514 | 4 | amp |
| TCGA-09-1672 | 11 | 7642121 | 9052508 | 1.011104335 | 3 | amp |
| TCGA-09-1672 | 11 | 9055107 | 9228334 | 1.411394415 | 4 | amp |
| TCGA-09-1672 | 11 | 9229083 | 10585955 | 0.993276427 | 3 | amp |
| TCGA-09-1672 | 11 | 10589976 | 10649605 | 1.345786998 | 4 | amp |
| TCGA-09-1672 | 11 | 10651051 | 10800678 | 1.060038623 | 3 | amp |
| TCGA-09-1672 | 11 | 10819305 | 10828881 | 0.458613524 | 1 | loss |
| TCGA-09-1672 | 11 | 11292643 | 11362578 | 1.718246857 | 5 | amp |
| TCGA-09-1672 | 11 | 11373458 | 11454370 | 1.380692409 | 4 | amp |
| TCGA-09-1672 | 11 | 11642902 | 12030142 | 0.918861959 | 3 | amp |
| TCGA-09-1672 | 11 | 12183654 | 13410712 | 1.423368472 | 4 | amp |
| TCGA-09-1672 | 11 | 13424679 | 14510143 | 1.050770623 | 3 | amp |
| TCGA-09-1672 | 11 | 14991400 | 17139247 | 1.264607027 | 4 | amp |
| TCGA-09-1672 | 11 | 17140178 | 17419347 | 0.9595087 | 3 | amp |
| TCGA-09-1672 | 11 | 17419876 | 17527556 | 1.432499576 | 4 | amp |
| TCGA-09-1672 | 11 | 17530843 | 18010342 | 1.022291729 | 3 | amp |
| TCGA-09-1672 | 11 | 18339291 | 18387432 | 1.267984081 | 4 | amp |
| TCGA-09-1672 | 11 | 18497839 | 19914179 | 1.014558686 | 3 | amp |
| TCGA-09-1672 | 11 | 19954647 | 20005851 | 1.51943817 | 4 | amp |
| TCGA-09-1672 | 11 | 20057423 | 20071474 | 1.988287613 | 5 | amp |
| TCGA-09-1672 | 11 | 20072796 | 20139861 | 1.491253601 | 4 | amp |
| TCGA-09-1672 | 11 | 20639288 | 26669449 | 1.054377898 | 3 | amp |
| TCGA-09-1672 | 11 | 26677651 | 27137111 | 1.297703563 | 4 | amp |
| TCGA-09-1672 | 11 | 27141176 | 32460746 | 1.005568524 | 3 | amp |
| TCGA-09-1672 | 11 | 33083053 | 33375175 | 0.94964239 | 3 | amp |
| TCGA-09-1672 | 11 | 33563990 | 33581495 | 1.861739233 | 5 | amp |
| TCGA-09-1672 | 11 | 33583191 | 33640263 | 1.488542296 | 4 | amp |
| TCGA-09-1672 | 11 | 34097721 | 34145415 | 1.32088823 | 4 | amp |
| TCGA-09-1672 | 11 | 34145837 | 34165093 | 1.760986572 | 5 | amp |
| TCGA-09-1672 | 11 | 34167624 | 34504071 | 1.073913599 | 3 | amp |
| TCGA-09-1672 | 11 | 34511490 | 34680297 | 1.396686883 | 4 | amp |
| TCGA-09-1672 | 11 | 34680368 | 35161197 | 0.981982198 | 3 | amp |
| TCGA-09-1672 | 11 | 35198054 | 36484328 | 1.379366324 | 4 | amp |
| TCGA-09-1672 | 11 | 36511343 | 45893832 | 1.089202208 | 3 | amp |
| TCGA-09-1672 | 11 | 46430039 | 46773109 | 1.257909619 | 4 | amp |
| TCGA-09-1672 | 11 | 46774162 | 47238624 | 1.069821338 | 3 | amp |
| TCGA-09-1672 | 11 | 47254317 | 47270343 | 1.726753874 | 5 | amp |
| TCGA-09-1672 | 11 | 47280698 | 47442007 | 1.025924739 | 3 | amp |

| | | | | | | |
|---|---|---|---|---|---|---|
| TCGA-09-1672 | 11 | 47444048 | 47505104 | 1.338822886 | 4 | amp |
| TCGA-09-1672 | 11 | 47505887 | 47681931 | 0.917254458 | 3 | amp |
| TCGA-09-1672 | 11 | 47684530 | 47732115 | 1.375431159 | 4 | amp |
| TCGA-09-1672 | 11 | 47735807 | 47869991 | 1.063706115 | 3 | amp |
| TCGA-09-1672 | 11 | 48131558 | 48632473 | 1.349897368 | 4 | amp |
| TCGA-09-1672 | 11 | 51458668 | 55607260 | 1.276688028 | 4 | amp |
| TCGA-09-1672 | 11 | 55648292 | 57135937 | 1.08266642 | 3 | amp |
| TCGA-09-1672 | 11 | 57136782 | 57182612 | 1.470098845 | 4 | amp |
| TCGA-09-1672 | 11 | 57296170 | 57583489 | 1.035146693 | 3 | amp |
| TCGA-09-1672 | 11 | 57583714 | 58275593 | 1.426620602 | 4 | amp |
| TCGA-09-1672 | 11 | 58294881 | 58962881 | 1.058821501 | 3 | amp |
| TCGA-09-1672 | 11 | 58967141 | 59211595 | 1.511662658 | 4 | amp |
| TCGA-09-1672 | 11 | 59224425 | 59283337 | 1.849563314 | 5 | amp |
| TCGA-09-1672 | 11 | 59554499 | 59630244 | 1.311549521 | 4 | amp |
| TCGA-09-1672 | 11 | 59631349 | 60048228 | 1.003113397 | 3 | amp |
| TCGA-09-1672 | 11 | 60059657 | 60160268 | 1.31195418 | 4 | amp |
| TCGA-09-1672 | 11 | 60468322 | 60971779 | 0.971829621 | 3 | amp |
| TCGA-09-1672 | 11 | 60973920 | 61018798 | 2.11595171 | 5 | amp |
| TCGA-09-1672 | 11 | 61106666 | 61179444 | 1.458207284 | 4 | amp |
| TCGA-09-1672 | 11 | 61183076 | 61205590 | 0.924546169 | 3 | amp |
| TCGA-09-1672 | 11 | 61213357 | 61258067 | 1.554910774 | 4 | amp |
| TCGA-09-1672 | 11 | 61548560 | 62186654 | 1.005751166 | 3 | amp |
| TCGA-09-1672 | 11 | 62189666 | 62363839 | 1.337821187 | 4 | amp |
| TCGA-09-1672 | 11 | 62520289 | 62533791 | 1.462791656 | 4 | amp |
| TCGA-09-1672 | 11 | 62572754 | 62760818 | 1.056935335 | 3 | amp |
| TCGA-09-1672 | 11 | 62760847 | 62931600 | 1.50294324 | 4 | amp |
| TCGA-09-1672 | 11 | 62931991 | 63381538 | 1.004533192 | 3 | amp |
| TCGA-09-1672 | 11 | 65046147 | 65063517 | 1.422661325 | 4 | amp |
| TCGA-09-1672 | 11 | 68312248 | 71715448 | 0.945885857 | 3 | amp |
| TCGA-09-1672 | 11 | 71715653 | 71822382 | 1.329308148 | 4 | amp |
| TCGA-09-1672 | 11 | 71846962 | 71907260 | 2.106874784 | 5 | amp |
| TCGA-09-1672 | 11 | 72005640 | 72028266 | 1.875733999 | 5 | amp |
| TCGA-09-1672 | 11 | 72040739 | 72289389 | 1.366135185 | 4 | amp |
| TCGA-09-1672 | 11 | 72289478 | 73712573 | 1.007354388 | 3 | amp |
| TCGA-09-1672 | 11 | 73714811 | 73805072 | 1.369038865 | 4 | amp |
| TCGA-09-1672 | 11 | 73879343 | 74000209 | 1.265280535 | 4 | amp |
| TCGA-09-1672 | 11 | 74109090 | 75001127 | 1.275697921 | 4 | amp |
| TCGA-09-1672 | 11 | 75316830 | 75511595 | 1.275408661 | 4 | amp |
| TCGA-09-1672 | 11 | 75562866 | 78413489 | 1.042234366 | 3 | amp |
| TCGA-09-1672 | 11 | 78419362 | 78489719 | 1.768232399 | 5 | amp |
| TCGA-09-1672 | 11 | 78497915 | 85687755 | 0.979674957 | 3 | amp |
| TCGA-09-1672 | 11 | 85987980 | 89431792 | 1.011843357 | 3 | amp |
| TCGA-09-1672 | 11 | 89644704 | 89666103 | 1.410285478 | 4 | amp |
| TCGA-09-1672 | 11 | 90016384 | 92577918 | 1.375345122 | 4 | amp |
| TCGA-09-1672 | 11 | 92590326 | 94349753 | 0.926774493 | 3 | amp |
| TCGA-09-1672 | 11 | 94351076 | 94599339 | 1.359877631 | 4 | amp |
| TCGA-09-1672 | 11 | 94602320 | 102100731 | 1.038534538 | 3 | amp |
| TCGA-09-1672 | 11 | 102391460 | 102826494 | 1.150936959 | 3 | amp |
| TCGA-09-1672 | 11 | 103780364 | 104034713 | 1.075800417 | 3 | amp |
| TCGA-09-1672 | 11 | 104815468 | 105010509 | 1.308594653 | 4 | amp |

| | | | | | | |
|---|---|---|---|---|---|---|
| TCGA-09-1672 | 11 | 105481708 | 107501335 | 0.956519899 | 3 | amp |
| TCGA-09-1672 | 11 | 107501379 | 107686723 | 1.359530001 | 4 | amp |
| TCGA-09-1672 | 11 | 107729378 | 111728512 | 0.937632288 | 3 | amp |
| TCGA-09-1672 | 11 | 113073077 | 113283621 | 1.088137886 | 3 | amp |
| TCGA-09-1672 | 11 | 113283999 | 113614822 | 1.318828626 | 4 | amp |
| TCGA-09-1672 | 11 | 113618223 | 113705073 | 0.934517508 | 3 | amp |
| TCGA-09-1672 | 11 | 113711279 | 114118123 | 1.390609846 | 4 | amp |
| TCGA-09-1672 | 11 | 114121012 | 114320752 | 1.024024915 | 3 | amp |
| TCGA-09-1672 | 11 | 114392657 | 115102261 | 1.362869838 | 4 | amp |
| TCGA-09-1672 | 11 | 115109145 | 116717310 | 0.973880545 | 3 | amp |
| TCGA-09-1672 | 11 | 116718137 | 116824851 | 1.420961118 | 4 | amp |
| TCGA-09-1672 | 11 | 116827571 | 117151020 | 1.036294937 | 3 | amp |
| TCGA-09-1672 | 11 | 117152014 | 117234243 | 1.262192852 | 4 | amp |
| TCGA-09-1672 | 11 | 117241748 | 117258143 | 1.861565922 | 5 | amp |
| TCGA-09-1672 | 11 | 117261408 | 117948078 | 1.242725288 | 4 | amp |
| TCGA-09-1672 | 11 | 117965490 | 117988214 | 1.864372268 | 5 | amp |
| TCGA-09-1672 | 11 | 117988532 | 118133848 | 1.418134904 | 4 | amp |
| TCGA-09-1672 | 11 | 118134779 | 118242424 | 1.085283819 | 3 | amp |
| TCGA-09-1672 | 11 | 118243191 | 118250492 | 1.853155106 | 5 | amp |
| TCGA-09-1672 | 11 | 118252051 | 119228790 | 1.009910229 | 3 | amp |
| TCGA-09-1672 | 11 | 119228820 | 120180334 | 1.313732576 | 4 | amp |
| TCGA-09-1672 | 11 | 120186042 | 120312530 | 0.926292255 | 3 | amp |
| TCGA-09-1672 | 11 | 120312801 | 120973496 | 1.29687483 | 4 | amp |
| TCGA-09-1672 | 11 | 120976486 | 121023809 | 1.803568243 | 5 | amp |
| TCGA-09-1672 | 11 | 121028471 | 121349009 | 0.88761668 | 3 | amp |
| TCGA-09-1672 | 11 | 121358671 | 122720968 | 1.390576368 | 4 | amp |
| TCGA-09-1672 | 11 | 122722356 | 122929558 | 1.061309048 | 3 | amp |
| TCGA-09-1672 | 11 | 122929747 | 123989447 | 1.309374604 | 4 | amp |
| TCGA-09-1672 | 11 | 123989678 | 124086239 | 1.027231822 | 3 | amp |
| TCGA-09-1672 | 11 | 124095374 | 124539456 | 1.308696993 | 4 | amp |
| TCGA-09-1672 | 11 | 125255412 | 125333482 | 1.32572487 | 4 | amp |
| TCGA-09-1672 | 11 | 125542429 | 125648702 | 1.400467917 | 4 | amp |
| TCGA-09-1672 | 11 | 125786907 | 126132081 | 1.205490606 | 4 | amp |
| TCGA-09-1672 | 11 | 126133724 | 126145332 | 1.796879372 | 5 | amp |
| TCGA-09-1672 | 11 | 126145666 | 126333246 | 1.034947317 | 3 | amp |
| TCGA-09-1672 | 11 | 126396387 | 128841067 | 1.27147281 | 4 | amp |
| TCGA-09-1672 | 11 | 128842297 | 132016411 | 0.976251651 | 3 | amp |
| TCGA-09-1672 | 11 | 132081858 | 132399137 | 1.472079252 | 4 | amp |
| TCGA-09-1672 | 11 | 132526977 | 134078861 | 0.94146589 | 3 | amp |
| TCGA-09-1672 | 12 | 73256 | 319165 | 1.059603919 | 3 | amp |
| TCGA-09-1672 | 12 | 330024 | 427629 | 1.426320756 | 4 | amp |
| TCGA-09-1672 | 12 | 430127 | 518628 | 0.910664385 | 3 | amp |
| TCGA-09-1672 | 12 | 520908 | 3662908 | 1.327085743 | 4 | amp |
| TCGA-09-1672 | 12 | 3677822 | 3701577 | 2.145835581 | 5 | amp |
| TCGA-09-1672 | 12 | 3702216 | 3806171 | 1.603883885 | 4 | amp |
| TCGA-09-1672 | 12 | 3921266 | 4668210 | 0.99515828 | 3 | amp |
| TCGA-09-1672 | 12 | 4700255 | 6345459 | 1.454192945 | 4 | amp |
| TCGA-09-1672 | 12 | 6346901 | 6679910 | 1.078085123 | 3 | amp |
| TCGA-09-1672 | 12 | 6679996 | 6697176 | 1.441386891 | 4 | amp |
| TCGA-09-1672 | 12 | 6697375 | 6887141 | 1.021568613 | 3 | amp |

| | | | | | | |
|---|---|---|---|---|---|---|
| TCGA-09-1672 | 12 | 6887518 | 6970813 | 1.31833943 | 4 | amp |
| TCGA-09-1672 | 12 | 6971587 | 7078760 | 1.038860113 | 3 | amp |
| TCGA-09-1672 | 12 | 7079340 | 7276803 | 1.32409454 | 4 | amp |
| TCGA-09-1672 | 12 | 7277215 | 7295941 | 1.989804238 | 5 | amp |
| TCGA-09-1672 | 12 | 7301560 | 7362477 | 1.10768717 | 3 | amp |
| TCGA-09-1672 | 12 | 7362568 | 7559467 | 1.386165578 | 4 | amp |
| TCGA-09-1672 | 12 | 7584991 | 7635640 | 0.908525627 | 3 | amp |
| TCGA-09-1672 | 12 | 7635906 | 8248721 | 1.450944519 | 4 | amp |
| TCGA-09-1672 | 12 | 8276455 | 8330186 | 0.95830639 | 3 | amp |
| TCGA-09-1672 | 12 | 8374358 | 8990194 | 1.372966262 | 4 | amp |
| TCGA-09-1672 | 12 | 8990898 | 9021009 | 1.967734516 | 5 | amp |
| TCGA-09-1672 | 12 | 9021087 | 9083570 | 1.437753437 | 4 | amp |
| TCGA-09-1672 | 12 | 9085120 | 9307443 | 1.110565892 | 3 | amp |
| TCGA-09-1672 | 12 | 9309763 | 9578426 | 1.32345508 | 4 | amp |
| TCGA-09-1672 | 12 | 9578637 | 10750758 | 1.156430375 | 3 | amp |
| TCGA-09-1672 | 12 | 10758863 | 12251962 | 1.515590725 | 4 | amp |
| TCGA-09-1672 | 12 | 12283711 | 12340074 | 1.372334128 | 4 | amp |
| TCGA-09-1672 | 12 | 12356115 | 12640161 | 0.995432536 | 3 | amp |
| TCGA-09-1672 | 12 | 12653414 | 13724900 | 1.434094017 | 4 | amp |
| TCGA-09-1672 | 12 | 13761501 | 14019176 | 1.992085487 | 5 | amp |
| TCGA-09-1672 | 12 | 14576788 | 14609591 | 0.88965543 | 3 | amp |
| TCGA-09-1672 | 12 | 14610124 | 16048481 | 1.426897032 | 4 | amp |
| TCGA-09-1672 | 12 | 16050733 | 20709678 | 1.149297587 | 3 | amp |
| TCGA-09-1672 | 12 | 20766325 | 20886060 | 1.355573153 | 4 | amp |
| TCGA-09-1672 | 12 | 20890003 | 21970252 | 1.060992971 | 3 | amp |
| TCGA-09-1672 | 12 | 21971044 | 22014006 | 1.508071004 | 4 | amp |
| TCGA-09-1672 | 12 | 22015847 | 22837911 | 1.043301335 | 3 | amp |
| TCGA-09-1672 | 12 | 23687124 | 25255213 | 1.30256985 | 4 | amp |
| TCGA-09-1672 | 12 | 25256741 | 26377328 | 1.073967957 | 3 | amp |
| TCGA-09-1672 | 12 | 26383586 | 26878700 | 1.305436865 | 4 | amp |
| TCGA-09-1672 | 12 | 26943064 | 27475444 | 1.092805793 | 3 | amp |
| TCGA-09-1672 | 12 | 27521149 | 27863892 | 1.467382124 | 4 | amp |
| TCGA-09-1672 | 12 | 27867672 | 29598393 | 1.081871658 | 3 | amp |
| TCGA-09-1672 | 12 | 29604269 | 30790138 | 1.498720798 | 4 | amp |
| TCGA-09-1672 | 12 | 30792438 | 30906727 | 1.057530352 | 3 | amp |
| TCGA-09-1672 | 12 | 31106907 | 39087669 | 1.325905062 | 4 | amp |
| TCGA-09-1672 | 12 | 39096639 | 39266888 | 0.94985509 | 3 | amp |
| TCGA-09-1672 | 12 | 39268232 | 40020209 | 1.285173227 | 4 | amp |
| TCGA-09-1672 | 12 | 40034716 | 40734295 | 1.148641333 | 3 | amp |
| TCGA-09-1672 | 12 | 40740519 | 41967708 | 1.344735758 | 4 | amp |
| TCGA-09-1672 | 12 | 42481548 | 44605181 | 1.105038387 | 3 | amp |
| TCGA-09-1672 | 12 | 44693322 | 46592586 | 1.284287123 | 4 | amp |
| TCGA-09-1672 | 12 | 47160474 | 47173832 | 1.48967447 | 4 | amp |
| TCGA-09-1672 | 12 | 47178280 | 48238838 | 1.127994969 | 3 | amp |
| TCGA-09-1672 | 12 | 48240025 | 48723878 | 1.469012352 | 4 | amp |
| TCGA-09-1672 | 12 | 48736712 | 49415668 | 1.081365056 | 3 | amp |
| TCGA-09-1672 | 12 | 49415819 | 49439962 | 1.310434568 | 4 | amp |
| TCGA-09-1672 | 12 | 49440004 | 49485221 | 0.932139214 | 3 | amp |
| TCGA-09-1672 | 12 | 49487978 | 49504362 | 1.42617398 | 4 | amp |
| TCGA-09-1672 | 12 | 49521690 | 49854867 | 1.047271951 | 3 | amp |

| | | | | | | |
|---|---|---|---|---|---|---|
| TCGA-09-1672 | 12 | 49878352 | 50386445 | 1.390428079 | 4 | amp |
| TCGA-09-1672 | 12 | 50387892 | 50501919 | 1.063200607 | 3 | amp |
| TCGA-09-1672 | 12 | 50503193 | 52374987 | 1.416969617 | 4 | amp |
| TCGA-09-1672 | 12 | 52377746 | 52471011 | 1.013767057 | 3 | amp |
| TCGA-09-1672 | 12 | 52565111 | 52574404 | 1.784657923 | 5 | amp |
| TCGA-09-1672 | 12 | 52574656 | 52779396 | 0.995139262 | 3 | amp |
| TCGA-09-1672 | 12 | 52788718 | 52797708 | 1.228923323 | 4 | amp |
| TCGA-09-1672 | 12 | 52799585 | 52843649 | 1.929225285 | 5 | amp |
| TCGA-09-1672 | 12 | 52843765 | 52887002 | 1.570107737 | 4 | amp |
| TCGA-09-1672 | 12 | 52908649 | 52979980 | 2.349637812 | 5 | amp |
| TCGA-09-1672 | 12 | 52980656 | 53045871 | 1.367758648 | 4 | amp |
| TCGA-09-1672 | 12 | 53074030 | 53189871 | 1.392187414 | 4 | amp |
| TCGA-09-1672 | 12 | 53200823 | 53242762 | 2.13127564 | 5 | amp |
| TCGA-09-1672 | 12 | 53291159 | 53648015 | 1.041753891 | 3 | amp |
| TCGA-09-1672 | 12 | 53662530 | 53690103 | 1.508165633 | 4 | amp |
| TCGA-09-1672 | 12 | 53691610 | 53714532 | 2.132006028 | 5 | amp |
| TCGA-09-1672 | 12 | 53715037 | 53825288 | 1.443767093 | 4 | amp |
| TCGA-09-1672 | 12 | 53836447 | 53879299 | 0.998216548 | 3 | amp |
| TCGA-09-1672 | 12 | 53879815 | 54105936 | 1.411522826 | 4 | amp |
| TCGA-09-1672 | 12 | 54106534 | 54116007 | 2.061415006 | 5 | amp |
| TCGA-09-1672 | 12 | 54117321 | 54448991 | 0.969641324 | 3 | amp |
| TCGA-09-1672 | 12 | 54575366 | 54678129 | 1.426236888 | 4 | amp |
| TCGA-09-1672 | 12 | 54686121 | 54744343 | 1.992971958 | 5 | amp |
| TCGA-09-1672 | 12 | 54756409 | 54769742 | 1.441798724 | 4 | amp |
| TCGA-09-1672 | 12 | 54790058 | 54852960 | 1.065784328 | 3 | amp |
| TCGA-09-1672 | 12 | 54854147 | 54963178 | 1.564769243 | 4 | amp |
| TCGA-09-1672 | 12 | 54963302 | 55042118 | 1.896672319 | 5 | amp |
| TCGA-09-1672 | 12 | 55248335 | 55726432 | 1.51747283 | 4 | amp |
| TCGA-09-1672 | 12 | 55758883 | 56120770 | 1.125461865 | 3 | amp |
| TCGA-09-1672 | 12 | 56120878 | 56211527 | 1.302483372 | 4 | amp |
| TCGA-09-1672 | 12 | 56212750 | 56297277 | 1.971489354 | 5 | amp |
| TCGA-09-1672 | 12 | 56330229 | 56398826 | 1.175428395 | 3 | amp |
| TCGA-09-1672 | 12 | 56415260 | 56491766 | 1.456850626 | 4 | amp |
| TCGA-09-1672 | 12 | 56492231 | 56511043 | 1.070981727 | 3 | amp |
| TCGA-09-1672 | 12 | 56511226 | 56562030 | 1.51348483 | 4 | amp |
| TCGA-09-1672 | 12 | 56563204 | 56583245 | 0.912220982 | 3 | amp |
| TCGA-09-1672 | 12 | 56600196 | 56642374 | 1.457410688 | 4 | amp |
| TCGA-09-1672 | 12 | 56642538 | 56680463 | 1.016779579 | 3 | amp |
| TCGA-09-1672 | 12 | 56704297 | 56717703 | 1.591632184 | 4 | amp |
| TCGA-09-1672 | 12 | 56717750 | 56827965 | 2.155384471 | 5 | amp |
| TCGA-09-1672 | 12 | 56845036 | 56962847 | 1.385284067 | 4 | amp |
| TCGA-09-1672 | 12 | 56963638 | 57007949 | 2.023483805 | 5 | amp |
| TCGA-09-1672 | 12 | 57008794 | 57058332 | 0.860265566 | 3 | amp |
| TCGA-09-1672 | 12 | 57058511 | 57106388 | 1.380919198 | 4 | amp |
| TCGA-09-1672 | 12 | 57106510 | 57115268 | 2.054260071 | 5 | amp |
| TCGA-09-1672 | 12 | 57118210 | 57409584 | 1.395433606 | 4 | amp |
| TCGA-09-1672 | 12 | 57422481 | 57453871 | 2.245452505 | 5 | amp |
| TCGA-09-1672 | 12 | 57454491 | 57502063 | 1.419115386 | 4 | amp |
| TCGA-09-1672 | 12 | 57532152 | 57560049 | 2.145546333 | 5 | amp |
| TCGA-09-1672 | 12 | 57560650 | 57571411 | 1.534470813 | 4 | amp |

| | | | | | | |
|---|---|---|---|---|---|---|
| TCGA-09-1672 | 12 | 57572083 | 57638183 | 1.098207569 | 3 | amp |
| TCGA-09-1672 | 12 | 57638272 | 57829005 | 2.01923491 | 5 | amp |
| TCGA-09-1672 | 12 | 57842982 | 58180883 | 0.980369335 | 3 | amp |
| TCGA-09-1672 | 12 | 58186752 | 58200352 | 1.307824088 | 4 | amp |
| TCGA-09-1672 | 12 | 58201072 | 58207227 | 1.967600345 | 5 | amp |
| TCGA-09-1672 | 12 | 58207881 | 58223424 | 1.308557583 | 4 | amp |
| TCGA-09-1672 | 12 | 59266295 | 59280691 | 1.464341424 | 4 | amp |
| TCGA-09-1672 | 12 | 59281522 | 64238689 | 1.094698806 | 3 | amp |
| TCGA-09-1672 | 12 | 64377704 | 64536503 | 1.524296791 | 4 | amp |
| TCGA-09-1672 | 12 | 64587609 | 65016500 | 1.06963497 | 3 | amp |
| TCGA-09-1672 | 12 | 65078560 | 65137124 | 1.508694052 | 4 | amp |
| TCGA-09-1672 | 12 | 65138577 | 66642012 | 1.04718261 | 3 | amp |
| TCGA-09-1672 | 12 | 66696326 | 67072716 | 1.423529412 | 4 | amp |
| TCGA-09-1672 | 12 | 67675684 | 68595890 | 1.077604589 | 3 | amp |
| TCGA-09-1672 | 12 | 68618875 | 68647255 | 1.678469617 | 5 | amp |
| TCGA-09-1672 | 12 | 68688999 | 69229806 | 1.094948966 | 3 | amp |
| TCGA-09-1672 | 12 | 69230430 | 69279680 | 1.615282099 | 4 | amp |
| TCGA-09-1672 | 12 | 69326387 | 70004621 | 1.007635447 | 3 | amp |
| TCGA-09-1672 | 12 | 70048659 | 70091622 | 1.633297526 | 4 | amp |
| TCGA-09-1672 | 12 | 70133547 | 70760862 | 0.886689989 | 3 | amp |
| TCGA-09-1672 | 12 | 70793932 | 70981096 | 1.541411188 | 4 | amp |
| TCGA-09-1672 | 12 | 70983698 | 71834086 | 1.125464226 | 3 | amp |
| TCGA-09-1672 | 12 | 71898339 | 72004565 | 1.449492176 | 4 | amp |
| TCGA-09-1672 | 12 | 72004758 | 72335558 | 1.145483167 | 3 | amp |
| TCGA-09-1672 | 12 | 72338045 | 72372919 | 2.050528294 | 5 | amp |
| TCGA-09-1672 | 12 | 72388161 | 77458428 | 1.162639231 | 3 | amp |
| TCGA-09-1672 | 12 | 78225152 | 78584001 | 1.30693252 | 4 | amp |
| TCGA-09-1672 | 12 | 78590983 | 91498138 | 1.137858531 | 3 | amp |
| TCGA-09-1672 | 12 | 91501845 | 93139370 | 1.378344108 | 4 | amp |
| TCGA-09-1672 | 12 | 93147802 | 94620491 | 1.047700138 | 3 | amp |
| TCGA-09-1672 | 12 | 94620877 | 94703908 | 1.497987285 | 4 | amp |
| TCGA-09-1672 | 12 | 94706681 | 95488478 | 1.097587169 | 3 | amp |
| TCGA-09-1672 | 12 | 95498777 | 96131962 | 1.324757914 | 4 | amp |
| TCGA-09-1672 | 12 | 96180681 | 99145336 | 1.049536786 | 3 | amp |
| TCGA-09-1672 | 12 | 99166768 | 100433590 | 1.348298817 | 4 | amp |
| TCGA-09-1672 | 12 | 100441301 | 100709543 | 1.046283693 | 3 | amp |
| TCGA-09-1672 | 12 | 100711491 | 101520872 | 1.360727048 | 4 | amp |
| TCGA-09-1672 | 12 | 101550954 | 101587599 | 0.925033911 | 3 | amp |
| TCGA-09-1672 | 12 | 101588846 | 102072067 | 1.357778759 | 4 | amp |
| TCGA-09-1672 | 12 | 102074093 | 102591603 | 1.067919801 | 3 | amp |
| TCGA-09-1672 | 12 | 102796224 | 104077090 | 1.502786511 | 4 | amp |
| TCGA-09-1672 | 12 | 104078754 | 104157447 | 2.204840289 | 5 | amp |
| TCGA-09-1672 | 12 | 104160028 | 104370886 | 1.410064547 | 4 | amp |
| TCGA-09-1672 | 12 | 104373549 | 105571081 | 1.027846282 | 3 | amp |
| TCGA-09-1672 | 12 | 105582017 | 108686775 | 1.43450248 | 4 | amp |
| TCGA-09-1672 | 12 | 108910689 | 109246493 | 1.11824008 | 3 | amp |
| TCGA-09-1672 | 12 | 109278729 | 109332786 | 1.501109418 | 4 | amp |
| TCGA-09-1672 | 12 | 109494451 | 109614072 | 1.049096369 | 3 | amp |
| TCGA-09-1672 | 12 | 109616877 | 109941031 | 1.553159894 | 4 | amp |
| TCGA-09-1672 | 12 | 109945334 | 109968467 | 1.927407212 | 5 | amp |

| | | | | | | |
|---|---|---|---|---|---|---|
| TCGA-09-1672 | 12 | 109971226 | 110503731 | 1.386864351 | 4 | amp |
| TCGA-09-1672 | 12 | 110565137 | 110734592 | 1.009397031 | 3 | amp |
| TCGA-09-1672 | 12 | 110760776 | 111947447 | 1.397238483 | 4 | amp |
| TCGA-09-1672 | 12 | 111991931 | 112375065 | 1.365860578 | 4 | amp |
| TCGA-09-1672 | 12 | 112375929 | 112617209 | 1.138218107 | 3 | amp |
| TCGA-09-1672 | 12 | 112620846 | 113314678 | 1.462403781 | 4 | amp |
| TCGA-09-1672 | 12 | 113316873 | 113349067 | 2.010148485 | 5 | amp |
| TCGA-09-1672 | 12 | 113354278 | 113416621 | 1.50403863 | 4 | amp |
| TCGA-09-1672 | 12 | 113424797 | 113496306 | 2.174565323 | 5 | amp |
| TCGA-09-1672 | 12 | 113515221 | 113559457 | 0.991030295 | 3 | amp |
| TCGA-09-1672 | 12 | 113565587 | 113837008 | 1.390041028 | 4 | amp |
| TCGA-09-1672 | 12 | 113865751 | 113875907 | 2.15853216 | 5 | amp |
| TCGA-09-1672 | 12 | 113905023 | 114374996 | 1.573745911 | 4 | amp |
| TCGA-09-1672 | 12 | 114377714 | 114804228 | 1.877781859 | 5 | amp |
| TCGA-09-1672 | 12 | 115120570 | 117593794 | 1.491282377 | 4 | amp |
| TCGA-09-1672 | 12 | 117653031 | 117969551 | 2.041986326 | 5 | amp |
| TCGA-09-1672 | 12 | 117977487 | 119773086 | 1.321969231 | 4 | amp |
| TCGA-09-1672 | 12 | 119866467 | 119978529 | 2.365409211 | 5 | amp |
| TCGA-09-1672 | 12 | 120106008 | 120575858 | 1.456596002 | 4 | amp |
| TCGA-09-1672 | 12 | 120576112 | 120622100 | 1.915879104 | 5 | amp |
| TCGA-09-1672 | 12 | 120622568 | 121003341 | 1.326470455 | 4 | amp |
| TCGA-09-1672 | 12 | 121004585 | 121165030 | 1.027741573 | 3 | amp |
| TCGA-09-1672 | 12 | 121175154 | 121454324 | 1.422488579 | 4 | amp |
| TCGA-09-1672 | 12 | 121458282 | 121598800 | 2.239998684 | 5 | amp |
| TCGA-09-1672 | 12 | 121600214 | 122611909 | 1.352005559 | 4 | amp |
| TCGA-09-1672 | 12 | 122612412 | 122727096 | 1.090246756 | 3 | amp |
| TCGA-09-1672 | 12 | 122729092 | 123042251 | 1.39281461 | 4 | amp |
| TCGA-09-1672 | 12 | 123046434 | 123749895 | 1.05587127 | 3 | amp |
| TCGA-09-1672 | 12 | 123751690 | 123835042 | 1.467622776 | 4 | amp |
| TCGA-09-1672 | 12 | 123875104 | 124284964 | 1.033208949 | 3 | amp |
| TCGA-09-1672 | 12 | 124285753 | 124383400 | 1.493357184 | 4 | amp |
| TCGA-09-1672 | 12 | 124395030 | 125614063 | 1.141458245 | 3 | amp |
| TCGA-09-1672 | 12 | 125618493 | 129822390 | 1.405285338 | 4 | amp |
| TCGA-09-1672 | 12 | 130015526 | 133779395 | 1.036020574 | 3 | amp |
| TCGA-09-1672 | 13 | 24995234 | 25021358 | 0.960169951 | 3 | amp |
| TCGA-09-1672 | 13 | 25023845 | 25033484 | 1.331752583 | 4 | amp |
| TCGA-09-1672 | 13 | 25034071 | 25068905 | 0.947647702 | 3 | amp |
| TCGA-09-1672 | 13 | 26104688 | 26535816 | 1.060927343 | 3 | amp |
| TCGA-09-1672 | 13 | 28122389 | 28578369 | 0.867400187 | 3 | amp |
| TCGA-09-1672 | 13 | 28588551 | 28647587 | 1.315381526 | 4 | amp |
| TCGA-09-1672 | 13 | 28748413 | 28931876 | 0.921916341 | 3 | amp |
| TCGA-09-1672 | 13 | 28958974 | 29041765 | 1.238951654 | 4 | amp |
| TCGA-09-1672 | 13 | 29598782 | 30423721 | 0.936748162 | 3 | amp |
| TCGA-09-1672 | 13 | 31221011 | 32954332 | 1.009993399 | 3 | amp |
| TCGA-09-1672 | 13 | 33327451 | 36367619 | 0.945896226 | 3 | amp |
| TCGA-09-1672 | 13 | 36379785 | 36521604 | 1.213270562 | 4 | amp |
| TCGA-09-1672 | 13 | 37804292 | 39564892 | 0.978441797 | 3 | amp |
| TCGA-09-1672 | 13 | 41704577 | 41835075 | 1.019292337 | 3 | amp |
| TCGA-09-1672 | 13 | 42161606 | 42393557 | 1.017013329 | 3 | amp |
| TCGA-09-1672 | 13 | 42524050 | 43986240 | 0.991234887 | 3 | amp |

| | | | | | | |
|---|---|---|---|---|---|---|
| TCGA-09-1672 | 13 | 46092880 | 46708443 | 0.904563863 | 3 | amp |
| TCGA-09-1672 | 13 | 46917515 | 48528731 | 0.947935893 | 3 | amp |
| TCGA-09-1672 | 13 | 49070271 | 50129849 | 0.885428128 | 3 | amp |
| TCGA-09-1672 | 13 | 50586025 | 52549567 | 0.90013438 | 3 | amp |
| TCGA-09-1672 | 13 | 53602911 | 53624924 | 1.765074259 | 5 | amp |
| TCGA-09-1672 | 13 | 58206673 | 72204794 | 0.91378181 | 3 | amp |
| TCGA-09-1672 | 13 | 73637931 | 75930394 | 0.998680135 | 3 | amp |
| TCGA-09-1672 | 13 | 76370768 | 76445112 | 1.270204141 | 4 | amp |
| TCGA-09-1672 | 13 | 77459263 | 95705453 | 0.943779374 | 3 | amp |
| TCGA-09-1672 | 13 | 95714885 | 95859084 | 1.221224178 | 4 | amp |
| TCGA-09-1672 | 13 | 95859996 | 96485027 | 0.912815188 | 3 | amp |
| TCGA-09-1672 | 13 | 96577890 | 96599478 | 0.246556669 | 1 | loss |
| TCGA-09-1672 | 13 | 97999009 | 99090125 | 0.931741749 | 3 | amp |
| TCGA-09-1672 | 13 | 99336953 | 99460107 | 1.422308632 | 4 | amp |
| TCGA-09-1672 | 13 | 99460851 | 103346903 | 1.013395923 | 3 | amp |
| TCGA-09-1672 | 13 | 103474371 | 103698666 | 1.001918418 | 3 | amp |
| TCGA-09-1672 | 13 | 103701597 | 107148201 | 1.206890865 | 4 | amp |
| TCGA-09-1672 | 13 | 107164838 | 111138212 | 1.001554993 | 3 | amp |
| TCGA-09-1672 | 13 | 113815213 | 113917938 | 0.945401659 | 3 | amp |
| TCGA-09-1672 | 14 | 19377543 | 19583149 | 0.522707497 | 1 | loss |
| TCGA-09-1672 | 14 | 20181044 | 20612837 | 0.984368928 | 3 | amp |
| TCGA-09-1672 | 14 | 20813527 | 20837945 | 0.97303688 | 3 | amp |
| TCGA-09-1672 | 14 | 20839364 | 20854413 | 1.271603978 | 4 | amp |
| TCGA-09-1672 | 14 | 20854543 | 20876614 | 0.928196508 | 3 | amp |
| TCGA-09-1672 | 14 | 21024529 | 21900032 | 0.930070105 | 3 | amp |
| TCGA-09-1672 | 14 | 21928317 | 21936929 | 0.497862494 | 1 | loss |
| TCGA-09-1672 | 14 | 21942999 | 22315435 | 1.03093435 | 3 | amp |
| TCGA-09-1672 | 14 | 22320697 | 22994712 | 1.40722751 | 4 | amp |
| TCGA-09-1672 | 14 | 22995782 | 23005181 | 1.083305354 | 3 | amp |
| TCGA-09-1672 | 14 | 23235671 | 23282657 | 1.045716978 | 3 | amp |
| TCGA-09-1672 | 14 | 23378657 | 23397884 | 1.034451956 | 3 | amp |
| TCGA-09-1672 | 14 | 23598813 | 23746409 | 1.004669646 | 3 | amp |
| TCGA-09-1672 | 14 | 23872515 | 23946804 | 0.941513541 | 3 | amp |
| TCGA-09-1672 | 14 | 24675121 | 24685032 | 1.045146409 | 3 | amp |
| TCGA-09-1672 | 14 | 24975180 | 25326417 | 0.954545557 | 3 | amp |
| TCGA-09-1672 | 14 | 31535351 | 31598715 | 0.95607662 | 3 | amp |
| TCGA-09-1672 | 14 | 31774066 | 31856633 | 0.906677483 | 3 | amp |
| TCGA-09-1672 | 14 | 32902681 | 34243759 | 0.96536396 | 3 | amp |
| TCGA-09-1672 | 14 | 38678582 | 39565331 | 0.42025124 | 1 | loss |
| TCGA-09-1672 | 14 | 39601160 | 39685443 | 0.934429923 | 3 | amp |
| TCGA-09-1672 | 14 | 39721895 | 39871719 | 0.444943606 | 1 | loss |
| TCGA-09-1672 | 14 | 50067251 | 50304950 | 0.515701611 | 1 | loss |
| TCGA-09-1672 | 14 | 51223729 | 51259634 | 0.966755234 | 3 | amp |
| TCGA-09-1672 | 14 | 51370730 | 51387789 | 1.021850925 | 3 | amp |
| TCGA-09-1672 | 14 | 55817066 | 55852875 | 1.10986643 | 3 | amp |
| TCGA-09-1672 | 14 | 60921670 | 61858034 | 0.52891281 | 1 | loss |
| TCGA-09-1672 | 14 | 61909753 | 62014652 | 1.146131202 | 3 | amp |
| TCGA-09-1672 | 14 | 62536306 | 63863488 | 0.963096477 | 3 | amp |
| TCGA-09-1672 | 14 | 64564615 | 64637182 | 0.908624052 | 3 | amp |
| TCGA-09-1672 | 14 | 64641638 | 64656991 | 0.493614811 | 1 | loss |

| | | | | | | |
|---|---|---|---|---|---|---|
| TCGA-09-1672 | 14 | 64669449 | 64911491 | 0.930869044 | 3 | amp |
| TCGA-09-1672 | 14 | 65246395 | 65528084 | 1.003035038 | 3 | amp |
| TCGA-09-1672 | 14 | 67849944 | 68260520 | 0.942644296 | 3 | amp |
| TCGA-09-1672 | 14 | 68582502 | 70635147 | 0.917786954 | 3 | amp |
| TCGA-09-1672 | 14 | 71067283 | 71478297 | 0.922847064 | 3 | amp |
| TCGA-09-1672 | 14 | 72090708 | 73181229 | 1.007149957 | 3 | amp |
| TCGA-09-1672 | 14 | 74436680 | 75349370 | 0.90628574 | 3 | amp |
| TCGA-09-1672 | 14 | 76088372 | 76621397 | 0.912597704 | 3 | amp |
| TCGA-09-1672 | 14 | 77274227 | 77327228 | 1.009015401 | 3 | amp |
| TCGA-09-1672 | 14 | 77915607 | 77929163 | 1.090356553 | 3 | amp |
| TCGA-09-1672 | 14 | 78285269 | 80319996 | 0.961080096 | 3 | amp |
| TCGA-09-1672 | 14 | 90745351 | 90833396 | 1.033055431 | 3 | amp |
| TCGA-09-1672 | 14 | 91110321 | 91681944 | 0.885716294 | 3 | amp |
| TCGA-09-1672 | 14 | 92336528 | 92357742 | 1.096635499 | 3 | amp |
| TCGA-09-1672 | 14 | 92920208 | 93081847 | 1.051600982 | 3 | amp |
| TCGA-09-1672 | 14 | 94038214 | 94203771 | 0.934721443 | 3 | amp |
| TCGA-09-1672 | 14 | 94780288 | 95088872 | 1.199954056 | 4 | amp |
| TCGA-09-1672 | 14 | 95089929 | 95942206 | 0.947375725 | 3 | amp |
| TCGA-09-1672 | 14 | 101195187 | 101348328 | 0.396213843 | 1 | loss |
| TCGA-09-1672 | 14 | 101488345 | 102348612 | 0.884083159 | 3 | amp |
| TCGA-09-1672 | 14 | 102460470 | 102500538 | 1.02771982 | 3 | amp |
| TCGA-09-1672 | 14 | 103425947 | 103852451 | 0.478470213 | 1 | loss |
| TCGA-09-1672 | 14 | 106452641 | 106494614 | 1.083371681 | 3 | amp |
| TCGA-09-1672 | 14 | 106518370 | 106552766 | 1.615204698 | 4 | amp |
| TCGA-09-1672 | 14 | 106573203 | 107199428 | 1.02818205 | 3 | amp |
| TCGA-09-1672 | 15 | 20169886 | 20667707 | 1.24900228 | 4 | amp |
| TCGA-09-1672 | 15 | 20668018 | 20778092 | 1.050924972 | 3 | amp |
| TCGA-09-1672 | 15 | 22140237 | 22567092 | 1.577713745 | 4 | amp |
| TCGA-09-1672 | 15 | 25219541 | 25299463 | 1.315291545 | 4 | amp |
| TCGA-09-1672 | 15 | 25301993 | 25328020 | 2.083575315 | 5 | amp |
| TCGA-09-1672 | 15 | 25328720 | 25423985 | 1.508150137 | 4 | amp |
| TCGA-09-1672 | 15 | 25425624 | 25474214 | 2.439410184 | 5 | amp |
| TCGA-09-1672 | 15 | 25475965 | 25485085 | 1.288668959 | 4 | amp |
| TCGA-09-1672 | 15 | 25605481 | 26828579 | 1.127941979 | 3 | amp |
| TCGA-09-1672 | 15 | 28857202 | 28908715 | 1.076679115 | 3 | amp |
| TCGA-09-1672 | 15 | 31319098 | 32404105 | 1.019820909 | 3 | amp |
| TCGA-09-1672 | 15 | 32935786 | 34131215 | 1.359866782 | 4 | amp |
| TCGA-09-1672 | 15 | 34132994 | 34643088 | 1.078598231 | 3 | amp |
| TCGA-09-1672 | 15 | 34657198 | 34849021 | 1.1246717 | 3 | amp |
| TCGA-09-1672 | 15 | 38228474 | 39544912 | 1.185299335 | 3 | amp |
| TCGA-09-1672 | 15 | 41020875 | 41120891 | 1.082921655 | 3 | amp |
| TCGA-09-1672 | 15 | 41227085 | 41810411 | 0.999296686 | 3 | amp |
| TCGA-09-1672 | 15 | 41812516 | 41829352 | 1.428295275 | 4 | amp |
| TCGA-09-1672 | 15 | 41849448 | 42067590 | 1.032556216 | 3 | amp |
| TCGA-09-1672 | 15 | 42092006 | 42132451 | 1.431015065 | 4 | amp |
| TCGA-09-1672 | 15 | 42166462 | 42641696 | 1.092644913 | 3 | amp |
| TCGA-09-1672 | 15 | 42643518 | 42749448 | 1.507845985 | 4 | amp |
| TCGA-09-1672 | 15 | 43348530 | 43476800 | 0.471465079 | 1 | loss |
| TCGA-09-1672 | 15 | 43544925 | 43579922 | 1.55227461 | 4 | amp |
| TCGA-09-1672 | 15 | 43678345 | 43705544 | 1.077549689 | 3 | amp |

| | | | | | | |
|---|---|---|---|---|---|---|
| TCGA-09-1672 | 15 | 43707779 | 43724915 | 1.475960701 | 4 | amp |
| TCGA-09-1672 | 15 | 43784186 | 43910865 | 1.104902462 | 3 | amp |
| TCGA-09-1672 | 15 | 43941283 | 44153617 | 1.044324938 | 3 | amp |
| TCGA-09-1672 | 15 | 44158277 | 44216514 | 1.500296437 | 4 | amp |
| TCGA-09-1672 | 15 | 44581180 | 45398842 | 1.136165567 | 3 | amp |
| TCGA-09-1672 | 15 | 45490940 | 45564986 | 1.180476378 | 3 | amp |
| TCGA-09-1672 | 15 | 45847739 | 45983259 | 1.048203257 | 3 | amp |
| TCGA-09-1672 | 15 | 48051989 | 48426743 | 1.44331461 | 4 | amp |
| TCGA-09-1672 | 15 | 48499886 | 48624553 | 1.126247925 | 3 | amp |
| TCGA-09-1672 | 15 | 48626599 | 48634306 | 0.442702896 | 1 | loss |
| TCGA-09-1672 | 15 | 48703141 | 48736917 | 1.512055614 | 4 | amp |
| TCGA-09-1672 | 15 | 48737516 | 49292226 | 1.087923809 | 3 | amp |
| TCGA-09-1672 | 15 | 50087607 | 50521250 | 0.992935025 | 3 | amp |
| TCGA-09-1672 | 15 | 50526009 | 50555637 | 1.421794425 | 4 | amp |
| TCGA-09-1672 | 15 | 50557744 | 50838755 | 1.108366165 | 3 | amp |
| TCGA-09-1672 | 15 | 51294666 | 51751008 | 1.157291583 | 3 | amp |
| TCGA-09-1672 | 15 | 51973933 | 56209035 | 1.056307164 | 3 | amp |
| TCGA-09-1672 | 15 | 57355924 | 57731838 | 1.155734723 | 3 | amp |
| TCGA-09-1672 | 15 | 57732561 | 58855932 | 1.389064048 | 4 | amp |
| TCGA-09-1672 | 15 | 59322981 | 59931403 | 1.117003592 | 3 | amp |
| TCGA-09-1672 | 15 | 60715776 | 62165602 | 1.10893636 | 3 | amp |
| TCGA-09-1672 | 15 | 62333483 | 65667767 | 1.06347258 | 3 | amp |
| TCGA-09-1672 | 15 | 65703322 | 66645338 | 1.087124821 | 3 | amp |
| TCGA-09-1672 | 15 | 66727319 | 66779657 | 1.628656108 | 4 | amp |
| TCGA-09-1672 | 15 | 66781523 | 68603434 | 1.053726042 | 3 | amp |
| TCGA-09-1672 | 15 | 68605095 | 69080303 | 1.366810616 | 4 | amp |
| TCGA-09-1672 | 15 | 69113003 | 69696220 | 1.067082421 | 3 | amp |
| TCGA-09-1672 | 15 | 71329474 | 72932485 | 0.999232744 | 3 | amp |
| TCGA-09-1672 | 15 | 72947060 | 72958739 | 1.604963764 | 4 | amp |
| TCGA-09-1672 | 15 | 72987483 | 74033003 | 1.125268833 | 3 | amp |
| TCGA-09-1672 | 15 | 74043273 | 74180138 | 1.621238818 | 4 | amp |
| TCGA-09-1672 | 15 | 74363194 | 74636351 | 1.44158966 | 4 | amp |
| TCGA-09-1672 | 15 | 74637364 | 74967503 | 1.125632421 | 3 | amp |
| TCGA-09-1672 | 15 | 75304961 | 75586872 | 1.478167532 | 4 | amp |
| TCGA-09-1672 | 15 | 75630348 | 76077945 | 0.989818989 | 3 | amp |
| TCGA-09-1672 | 15 | 77287872 | 78416139 | 1.148965483 | 3 | amp |
| TCGA-09-1672 | 15 | 79277327 | 79356874 | 1.359851361 | 4 | amp |
| TCGA-09-1672 | 15 | 79382522 | 80460691 | 0.993844552 | 3 | amp |
| TCGA-09-1672 | 15 | 80464450 | 81221557 | 1.400513599 | 4 | amp |
| TCGA-09-1672 | 15 | 81224139 | 81552226 | 1.06306748 | 3 | amp |
| TCGA-09-1672 | 15 | 81557950 | 81611858 | 1.403327839 | 4 | amp |
| TCGA-09-1672 | 15 | 81614694 | 82532947 | 1.160312072 | 3 | amp |
| TCGA-09-1672 | 15 | 82894359 | 83561634 | 1.016627689 | 3 | amp |
| TCGA-09-1672 | 15 | 83807964 | 85147601 | 1.054813843 | 3 | amp |
| TCGA-09-1672 | 15 | 85259220 | 86201854 | 1.116367964 | 3 | amp |
| TCGA-09-1672 | 15 | 86205591 | 89009030 | 1.277518212 | 4 | amp |
| TCGA-09-1672 | 15 | 89010344 | 89453208 | 0.980017538 | 3 | amp |
| TCGA-09-1672 | 15 | 89659505 | 89761979 | 1.346538957 | 4 | amp |
| TCGA-09-1672 | 15 | 89857830 | 90039803 | 1.088193638 | 3 | amp |
| TCGA-09-1672 | 15 | 90226563 | 90281489 | 1.139478105 | 3 | amp |

| | | | | | | |
|---|---|---|---|---|---|---|
| TCGA-09-1672 | 15 | 90969299 | 90992892 | 1.032533729 | 3 | amp |
| TCGA-09-1672 | 15 | 90995968 | 91016276 | 1.51974488 | 4 | amp |
| TCGA-09-1672 | 15 | 91016898 | 91334096 | 1.045300882 | 3 | amp |
| TCGA-09-1672 | 15 | 91542184 | 91795794 | 1.150001372 | 3 | amp |
| TCGA-09-1672 | 15 | 91801597 | 92690421 | 1.603170901 | 4 | amp |
| TCGA-09-1672 | 15 | 92694151 | 101024933 | 1.053459991 | 3 | amp |
| TCGA-09-1672 | 16 | 66517 | 3367889 | 0.995420446 | 3 | amp |
| TCGA-09-1672 | 16 | 3405929 | 9210826 | 1.474289986 | 4 | amp |
| TCGA-09-1672 | 16 | 9250495 | 10032411 | 2.388599375 | 5 | amp |
| TCGA-09-1672 | 16 | 10273791 | 11010329 | 1.611599897 | 4 | amp |
| TCGA-09-1672 | 16 | 11012249 | 11770228 | 1.955042343 | 5 | amp |
| TCGA-09-1672 | 16 | 11773011 | 12172790 | 1.491066228 | 4 | amp |
| TCGA-09-1672 | 16 | 12220461 | 12798933 | 2.123981656 | 5 | amp |
| TCGA-09-1672 | 16 | 12875000 | 14749079 | 1.589266654 | 4 | amp |
| TCGA-09-1672 | 16 | 14755715 | 14859274 | 2.160005046 | 5 | amp |
| TCGA-09-1672 | 16 | 14860251 | 14947898 | 1.47987329 | 4 | amp |
| TCGA-09-1672 | 16 | 14951066 | 15046984 | 2.455284846 | 5 | amp |
| TCGA-09-1672 | 16 | 15082200 | 15165162 | 1.134676365 | 3 | amp |
| TCGA-09-1672 | 16 | 15166751 | 15494738 | 2.05857974 | 5 | amp |
| TCGA-09-1672 | 16 | 15501635 | 15733138 | 1.488371088 | 4 | amp |
| TCGA-09-1672 | 16 | 15758586 | 15917309 | 2.286241385 | 5 | amp |
| TCGA-09-1672 | 16 | 15931756 | 16108536 | 1.237886677 | 4 | amp |
| TCGA-09-1672 | 16 | 16110310 | 16315716 | 2.873632226 | 5 | amp |
| TCGA-09-1672 | 16 | 16330742 | 16346394 | 1.404863582 | 4 | amp |
| TCGA-09-1672 | 16 | 16349569 | 18555061 | 2.340368656 | 5 | amp |
| TCGA-09-1672 | 16 | 18558525 | 19191906 | 1.605852837 | 4 | amp |
| TCGA-09-1672 | 16 | 19194799 | 19516428 | 2.290802759 | 5 | amp |
| TCGA-09-1672 | 16 | 19518994 | 19586491 | 1.362294849 | 4 | amp |
| TCGA-09-1672 | 16 | 19590351 | 20486783 | 1.974392052 | 5 | amp |
| TCGA-09-1672 | 16 | 20486913 | 20563662 | 1.516528112 | 4 | amp |
| TCGA-09-1672 | 16 | 20565050 | 20696739 | 2.087869682 | 5 | amp |
| TCGA-09-1672 | 16 | 20702294 | 20856589 | 1.425694485 | 4 | amp |
| TCGA-09-1672 | 16 | 20857451 | 20981332 | 2.167384698 | 5 | amp |
| TCGA-09-1672 | 16 | 20986525 | 21063209 | 1.511623226 | 4 | amp |
| TCGA-09-1672 | 16 | 21065737 | 21273538 | 2.149590836 | 5 | amp |
| TCGA-09-1672 | 16 | 21278846 | 21690578 | 1.542437348 | 4 | amp |
| TCGA-09-1672 | 16 | 21693042 | 21768620 | 2.297019151 | 5 | amp |
| TCGA-09-1672 | 16 | 21771765 | 22000137 | 1.667827327 | 4 | amp |
| TCGA-09-1672 | 16 | 22120839 | 22285128 | 2.093973451 | 5 | amp |
| TCGA-09-1672 | 16 | 22291487 | 22477614 | 1.037391035 | 3 | amp |
| TCGA-09-1672 | 16 | 22480310 | 23479025 | 1.611199898 | 4 | amp |
| TCGA-09-1672 | 16 | 23480201 | 23546684 | 2.059584805 | 5 | amp |
| TCGA-09-1672 | 16 | 23555809 | 24185929 | 1.394018554 | 4 | amp |
| TCGA-09-1672 | 16 | 24192059 | 24373228 | 2.384662136 | 5 | amp |
| TCGA-09-1672 | 16 | 24551910 | 24769730 | 0.83771836 | 3 | amp |
| TCGA-09-1672 | 16 | 24788215 | 25252077 | 1.932337662 | 5 | amp |
| TCGA-09-1672 | 16 | 25255051 | 27268943 | 1.657950709 | 4 | amp |
| TCGA-09-1672 | 16 | 27351499 | 27761685 | 2.190372429 | 5 | amp |
| TCGA-09-1672 | 16 | 27763022 | 27789305 | 1.582070247 | 4 | amp |
| TCGA-09-1672 | 16 | 27789832 | 28137209 | 2.034558541 | 5 | amp |

| | | | | | | |
|---|---|---|---|---|---|---|
| TCGA-09-1672 | 16 | 28143629 | 28785254 | 1.490800797 | 4 | amp |
| TCGA-09-1672 | 16 | 28836614 | 29332332 | 1.188823752 | 3 | amp |
| TCGA-09-1672 | 16 | 29367047 | 29562715 | 1.400769466 | 4 | amp |
| TCGA-09-1672 | 16 | 29564395 | 30457094 | 1.195441471 | 3 | amp |
| TCGA-09-1672 | 16 | 30484128 | 30510594 | 1.536614069 | 4 | amp |
| TCGA-09-1672 | 16 | 30510614 | 30525189 | 2.127142116 | 5 | amp |
| TCGA-09-1672 | 16 | 30528260 | 30721490 | 1.025367417 | 3 | amp |
| TCGA-09-1672 | 16 | 30721771 | 30745996 | 1.559386223 | 4 | amp |
| TCGA-09-1672 | 16 | 30747497 | 47630482 | 1.101607543 | 3 | amp |
| TCGA-09-1672 | 16 | 47644723 | 47733328 | 1.451484179 | 4 | amp |
| TCGA-09-1672 | 16 | 48117578 | 48261915 | 2.361850337 | 5 | amp |
| TCGA-09-1672 | 16 | 48264295 | 53358849 | 1.348703811 | 4 | amp |
| TCGA-09-1672 | 16 | 53396278 | 55518121 | 1.079960932 | 3 | amp |
| TCGA-09-1672 | 16 | 55519154 | 55719243 | 1.348620614 | 4 | amp |
| TCGA-09-1672 | 16 | 55725776 | 56310033 | 2.464370678 | 5 | amp |
| TCGA-09-1672 | 16 | 56362472 | 56832478 | 1.36632769 | 4 | amp |
| TCGA-09-1672 | 16 | 56839359 | 56918158 | 2.22060962 | 5 | amp |
| TCGA-09-1672 | 16 | 56920271 | 57017600 | 1.483816893 | 4 | amp |
| TCGA-09-1672 | 16 | 57054561 | 57101753 | 2.349385101 | 5 | amp |
| TCGA-09-1672 | 16 | 57104428 | 57466495 | 1.471872288 | 4 | amp |
| TCGA-09-1672 | 16 | 57467919 | 57555093 | 1.036224958 | 3 | amp |
| TCGA-09-1672 | 16 | 57559775 | 57719876 | 1.555228691 | 4 | amp |
| TCGA-09-1672 | 16 | 57722213 | 57757097 | 2.826825058 | 5 | amp |
| TCGA-09-1672 | 16 | 57765033 | 57935540 | 1.29470964 | 4 | amp |
| TCGA-09-1672 | 16 | 57937655 | 58010576 | 2.082047621 | 5 | amp |
| TCGA-09-1672 | 16 | 58011680 | 66565387 | 1.466272097 | 4 | amp |
| TCGA-09-1672 | 16 | 66570803 | 66860691 | 1.029100699 | 3 | amp |
| TCGA-09-1672 | 16 | 66861887 | 67000817 | 1.456038522 | 4 | amp |
| TCGA-09-1672 | 16 | 67003531 | 67702010 | 1.07523121 | 3 | amp |
| TCGA-09-1672 | 16 | 67708996 | 67778394 | 2.064521631 | 5 | amp |
| TCGA-09-1672 | 16 | 67805872 | 67877428 | 1.612837641 | 4 | amp |
| TCGA-09-1672 | 16 | 67899022 | 67919810 | 1.000238156 | 3 | amp |
| TCGA-09-1672 | 16 | 67919839 | 68716417 | 1.422518537 | 4 | amp |
| TCGA-09-1672 | 16 | 68718396 | 68867417 | 2.358960795 | 5 | amp |
| TCGA-09-1672 | 16 | 68877507 | 70154630 | 1.430776438 | 4 | amp |
| TCGA-09-1672 | 16 | 70161109 | 70190819 | 2.047539029 | 5 | amp |
| TCGA-09-1672 | 16 | 70208123 | 70348891 | 1.282698222 | 4 | amp |
| TCGA-09-1672 | 16 | 70349819 | 70405490 | 2.416571402 | 5 | amp |
| TCGA-09-1672 | 16 | 70405798 | 70524323 | 1.640965862 | 4 | amp |
| TCGA-09-1672 | 16 | 70530131 | 70605765 | 2.151822906 | 5 | amp |
| TCGA-09-1672 | 16 | 70688387 | 70866980 | 1.476116452 | 4 | amp |
| TCGA-09-1672 | 16 | 70867748 | 71015502 | 1.971727901 | 5 | amp |
| TCGA-09-1672 | 16 | 71019036 | 71411669 | 1.669929005 | 4 | amp |
| TCGA-09-1672 | 16 | 71416589 | 71423829 | 2.290793716 | 5 | amp |
| TCGA-09-1672 | 16 | 71481990 | 71955360 | 1.520745886 | 4 | amp |
| TCGA-09-1672 | 16 | 71956335 | 72170774 | 2.1827145 | 5 | amp |
| TCGA-09-1672 | 16 | 72173182 | 74487270 | 1.549132238 | 4 | amp |
| TCGA-09-1672 | 16 | 74490539 | 74990560 | 1.921986034 | 5 | amp |
| TCGA-09-1672 | 16 | 74999605 | 77317970 | 1.331173644 | 4 | amp |
| TCGA-09-1672 | 16 | 77323084 | 77390025 | 2.142642331 | 5 | amp |

| | | | | | | |
|---|---|---|---|---|---|---|
| TCGA-09-1672 | 16 | 77393207 | 80719030 | 1.649441133 | 4 | amp |
| TCGA-09-1672 | 16 | 81009972 | 81078630 | 0.897568905 | 3 | amp |
| TCGA-09-1672 | 16 | 81087627 | 81142909 | 1.445897581 | 4 | amp |
| TCGA-09-1672 | 16 | 81145752 | 82132100 | 2.126196921 | 5 | amp |
| TCGA-09-1672 | 16 | 82182281 | 82892082 | 1.018927509 | 3 | amp |
| TCGA-09-1672 | 16 | 83065598 | 83817087 | 2.274051654 | 5 | amp |
| TCGA-09-1672 | 16 | 83828571 | 84442115 | 1.536989686 | 4 | amp |
| TCGA-09-1672 | 16 | 84444101 | 84476220 | 2.191718593 | 5 | amp |
| TCGA-09-1672 | 16 | 84479925 | 85141562 | 1.49302601 | 4 | amp |
| TCGA-09-1672 | 16 | 85141580 | 90244214 | 1.108247771 | 3 | amp |
| TCGA-09-1672 | 17 | 1985026 | 2091870 | 1.298307676 | 4 | amp |
| TCGA-09-1672 | 17 | 2898575 | 3337189 | 0.96826006 | 3 | amp |
| TCGA-09-1672 | 17 | 3925912 | 4020482 | 0.920114461 | 3 | amp |
| TCGA-09-1672 | 17 | 4619237 | 4648709 | 0.499980483 | 1 | loss |
| TCGA-09-1672 | 17 | 4718735 | 4764116 | 0.974458487 | 3 | amp |
| TCGA-09-1672 | 17 | 4841253 | 4864174 | 0.487242189 | 1 | loss |
| TCGA-09-1672 | 17 | 5253714 | 5312260 | 0.94097212 | 3 | amp |
| TCGA-09-1672 | 17 | 5433762 | 5463410 | 1.031042071 | 3 | amp |
| TCGA-09-1672 | 17 | 6978641 | 6982157 | 1.038902107 | 3 | amp |
| TCGA-09-1672 | 17 | 7004856 | 7224788 | 0.509685264 | 1 | loss |
| TCGA-09-1672 | 17 | 7411561 | 7509500 | 0.50008643 | 1 | loss |
| TCGA-09-1672 | 17 | 7559010 | 7593023 | 1.0484353 | 3 | amp |
| TCGA-09-1672 | 17 | 7643003 | 7678305 | 1.217901057 | 4 | amp |
| TCGA-09-1672 | 17 | 7678549 | 7728069 | 1.004007462 | 3 | amp |
| TCGA-09-1672 | 17 | 7748820 | 7836712 | 0.513845243 | 1 | loss |
| TCGA-09-1672 | 17 | 7846655 | 7852885 | 1.003714774 | 3 | amp |
| TCGA-09-1672 | 17 | 7907111 | 8077615 | 0.537175735 | 1 | loss |
| TCGA-09-1672 | 17 | 8131417 | 8139697 | 0.961838817 | 3 | amp |
| TCGA-09-1672 | 17 | 8248577 | 8396394 | 0.480983566 | 1 | loss |
| TCGA-09-1672 | 17 | 8416801 | 8424626 | 1.049309515 | 3 | amp |
| TCGA-09-1672 | 17 | 9615246 | 10352392 | 0.977133189 | 3 | amp |
| TCGA-09-1672 | 17 | 10353747 | 10364421 | 1.244353439 | 4 | amp |
| TCGA-09-1672 | 17 | 10366173 | 10370080 | 1.074676825 | 3 | amp |
| TCGA-09-1672 | 17 | 10415110 | 10545651 | 1.074411953 | 3 | amp |
| TCGA-09-1672 | 17 | 10545706 | 10555026 | 1.342504984 | 4 | amp |
| TCGA-09-1672 | 17 | 11514911 | 11845830 | 1.064235872 | 3 | amp |
| TCGA-09-1672 | 17 | 12013657 | 12847005 | 0.972000367 | 3 | amp |
| TCGA-09-1672 | 17 | 15492161 | 15522874 | 1.105564074 | 3 | amp |
| TCGA-09-1672 | 17 | 15928287 | 15976932 | 0.945022722 | 3 | amp |
| TCGA-09-1672 | 17 | 15978811 | 16056740 | 0.483969135 | 1 | loss |
| TCGA-09-1672 | 17 | 18389228 | 18420703 | 0.947390628 | 3 | amp |
| TCGA-09-1672 | 17 | 18630834 | 18675792 | 1.106216884 | 3 | amp |
| TCGA-09-1672 | 17 | 20319092 | 20363770 | 0.961065923 | 3 | amp |
| TCGA-09-1672 | 17 | 25944267 | 26096209 | 1.02060013 | 3 | amp |
| TCGA-09-1672 | 17 | 26905212 | 26913539 | 0.999220395 | 3 | amp |
| TCGA-09-1672 | 17 | 27001532 | 27024754 | 1.055868774 | 3 | amp |
| TCGA-09-1672 | 17 | 27093584 | 27211347 | 1.063290243 | 3 | amp |
| TCGA-09-1672 | 17 | 27287786 | 27437666 | 0.939353439 | 3 | amp |
| TCGA-09-1672 | 17 | 27957823 | 28030088 | 1.136641778 | 3 | amp |
| TCGA-09-1672 | 17 | 29552070 | 29576189 | 0.914738716 | 3 | amp |

| | | | | | | |
|---|---|---|---|---|---|---|
| TCGA-09-1672 | 17 | 31075898 | 31098250 | 0.995346536 | 3 | amp |
| TCGA-09-1672 | 17 | 31322400 | 31439129 | 1.052842304 | 3 | amp |
| TCGA-09-1672 | 17 | 33317937 | 33341830 | 1.067472468 | 3 | amp |
| TCGA-09-1672 | 17 | 34171031 | 34264960 | 0.913081297 | 3 | amp |
| TCGA-09-1672 | 17 | 34266140 | 34310940 | 1.490241236 | 4 | amp |
| TCGA-09-1672 | 17 | 34311370 | 34433721 | 1.0288084 | 3 | amp |
| TCGA-09-1672 | 17 | 35343912 | 35631196 | 0.916219663 | 3 | amp |
| TCGA-09-1672 | 17 | 35921267 | 35984494 | 0.459994962 | 1 | loss |
| TCGA-09-1672 | 17 | 35985922 | 36070710 | 0.942835182 | 3 | amp |
| TCGA-09-1672 | 17 | 36454383 | 36492052 | 0.93893467 | 3 | amp |
| TCGA-09-1672 | 17 | 36918620 | 36936900 | 1.037636933 | 3 | amp |
| TCGA-09-1672 | 17 | 36971111 | 37009376 | 1.086523919 | 3 | amp |
| TCGA-09-1672 | 17 | 37234113 | 37264556 | 1.075327761 | 3 | amp |
| TCGA-09-1672 | 17 | 38187741 | 38253403 | 0.87813795 | 3 | amp |
| TCGA-09-1672 | 17 | 38253421 | 38340662 | 0.48790885 | 1 | loss |
| TCGA-09-1672 | 17 | 38416738 | 38457909 | 1.005568846 | 3 | amp |
| TCGA-09-1672 | 17 | 38633790 | 38721716 | 1.139970882 | 3 | amp |
| TCGA-09-1672 | 17 | 38854480 | 38907617 | 0.964573406 | 3 | amp |
| TCGA-09-1672 | 17 | 39084430 | 39412202 | 0.929951113 | 3 | amp |
| TCGA-09-1672 | 17 | 39577608 | 39673435 | 0.95654011 | 3 | amp |
| TCGA-09-1672 | 17 | 40481361 | 40500590 | 0.958422019 | 3 | amp |
| TCGA-09-1672 | 17 | 40847505 | 40864524 | 0.916970209 | 3 | amp |
| TCGA-09-1672 | 17 | 41026068 | 41108043 | 1.064488181 | 3 | amp |
| TCGA-09-1672 | 17 | 41180392 | 41613880 | 0.96940908 | 3 | amp |
| TCGA-09-1672 | 17 | 42273714 | 42294104 | 0.431372342 | 1 | loss |
| TCGA-09-1672 | 17 | 42800168 | 42825890 | 0.946721574 | 3 | amp |
| TCGA-09-1672 | 17 | 42928599 | 42964070 | 1.091848488 | 3 | amp |
| TCGA-09-1672 | 17 | 43112137 | 43190677 | 0.913388439 | 3 | amp |
| TCGA-09-1672 | 17 | 43192757 | 43333822 | 0.537288664 | 1 | loss |
| TCGA-09-1672 | 17 | 43472790 | 43528176 | 0.527929324 | 1 | loss |
| TCGA-09-1672 | 17 | 43530699 | 43596393 | 0.993961896 | 3 | amp |
| TCGA-09-1672 | 17 | 45009378 | 45127752 | 0.922402204 | 3 | amp |
| TCGA-09-1672 | 17 | 45286679 | 45360937 | 1.082897395 | 3 | amp |
| TCGA-09-1672 | 17 | 45361754 | 45369959 | 1.462328674 | 4 | amp |
| TCGA-09-1672 | 17 | 45376664 | 45518118 | 0.410373389 | 1 | loss |
| TCGA-09-1672 | 17 | 46052829 | 46058895 | 0.992813544 | 3 | amp |
| TCGA-09-1672 | 17 | 46196023 | 46441223 | 1.039931979 | 3 | amp |
| TCGA-09-1672 | 17 | 46847004 | 46940403 | 0.95843255 | 3 | amp |
| TCGA-09-1672 | 17 | 47677661 | 47700193 | 1.014697531 | 3 | amp |
| TCGA-09-1672 | 17 | 48733975 | 48761508 | 1.091317597 | 3 | amp |
| TCGA-09-1672 | 17 | 49054465 | 49072621 | 1.012795802 | 3 | amp |
| TCGA-09-1672 | 17 | 49270062 | 49280298 | 1.026677148 | 3 | amp |
| TCGA-09-1672 | 17 | 49708865 | 50008540 | 1.239282658 | 4 | amp |
| TCGA-09-1672 | 17 | 53007380 | 53038016 | 0.967939238 | 3 | amp |
| TCGA-09-1672 | 17 | 53797988 | 54559932 | 0.950115231 | 3 | amp |
| TCGA-09-1672 | 17 | 55189101 | 55196439 | 1.023731442 | 3 | amp |
| TCGA-09-1672 | 17 | 56246968 | 56290456 | 0.96558173 | 3 | amp |
| TCGA-09-1672 | 17 | 56646526 | 56671028 | 0.893076981 | 3 | amp |
| TCGA-09-1672 | 17 | 56676181 | 56700410 | 1.371224703 | 4 | amp |
| TCGA-09-1672 | 17 | 59115182 | 59155911 | 1.063597632 | 3 | amp |

| | | | | | | |
|---|---|---|---|---|---|---|
| TCGA-09-1672 | 17 | 60503820 | 60526132 | 1.075044854 | 3 | amp |
| TCGA-09-1672 | 17 | 61483456 | 61499373 | 0.999664518 | 3 | amp |
| TCGA-09-1672 | 17 | 61972329 | 61987723 | 1.170169892 | 3 | amp |
| TCGA-09-1672 | 17 | 61987777 | 62007769 | 1.428542211 | 4 | amp |
| TCGA-09-1672 | 17 | 62175420 | 62386102 | 0.951160298 | 3 | amp |
| TCGA-09-1672 | 17 | 63149497 | 63204142 | 1.050477912 | 3 | amp |
| TCGA-09-1672 | 17 | 64492299 | 64738928 | 1.309583266 | 4 | amp |
| TCGA-09-1672 | 17 | 64770054 | 65103842 | 0.944498813 | 3 | amp |
| TCGA-09-1672 | 17 | 65871615 | 65962789 | 0.88655567 | 3 | amp |
| TCGA-09-1672 | 17 | 65971860 | 66303863 | 0.495604302 | 1 | loss |
| TCGA-09-1672 | 17 | 66339718 | 66416646 | 1.10546654 | 3 | amp |
| TCGA-09-1672 | 17 | 66864409 | 66878182 | 0.996233443 | 3 | amp |
| TCGA-09-1672 | 17 | 67285257 | 67310565 | 0.406648096 | 1 | loss |
| TCGA-09-1672 | 17 | 67411073 | 68129516 | 1.027916512 | 3 | amp |
| TCGA-09-1672 | 17 | 70732763 | 71084909 | 1.115478239 | 3 | amp |
| TCGA-09-1672 | 17 | 71379962 | 72301589 | 0.936328737 | 3 | amp |
| TCGA-09-1672 | 17 | 72477858 | 72700965 | 1.035104486 | 3 | amp |
| TCGA-09-1672 | 17 | 73221114 | 73231305 | 0.995275204 | 3 | amp |
| TCGA-09-1672 | 17 | 73647215 | 73667979 | 1.451751115 | 4 | amp |
| TCGA-09-1672 | 17 | 73689480 | 73720882 | 0.961707919 | 3 | amp |
| TCGA-09-1672 | 17 | 78264327 | 78287009 | 0.954794894 | 3 | amp |
| TCGA-09-1672 | 17 | 79567349 | 81188237 | 0.522333619 | 1 | loss |
| TCGA-09-1672 | 18 | 3567473 | 5445250 | 0.960260023 | 3 | amp |
| TCGA-09-1672 | 18 | 6982452 | 7888419 | 0.8960607 | 3 | amp |
| TCGA-09-1672 | 18 | 13072975 | 13105090 | 0.291474597 | 1 | loss |
| TCGA-09-1672 | 18 | 20581500 | 20945674 | 0.914303367 | 3 | amp |
| TCGA-09-1672 | 18 | 21422301 | 21694624 | 0.923197074 | 3 | amp |
| TCGA-09-1672 | 18 | 29116158 | 29175270 | 1.005671834 | 3 | amp |
| TCGA-09-1672 | 18 | 29866949 | 30260330 | 1.013838557 | 3 | amp |
| TCGA-09-1672 | 18 | 31311919 | 32720355 | 0.921701593 | 3 | amp |
| TCGA-09-1672 | 18 | 33716244 | 33734974 | 0.434069977 | 1 | loss |
| TCGA-09-1672 | 18 | 42449161 | 43329949 | 1.073930789 | 3 | amp |
| TCGA-09-1672 | 18 | 43467673 | 43502616 | 0.916575491 | 3 | amp |
| TCGA-09-1672 | 18 | 43664228 | 43671852 | 0.328534981 | 1 | loss |
| TCGA-09-1672 | 18 | 44542698 | 44559533 | 0.189326133 | 1 | loss |
| TCGA-09-1672 | 18 | 46917928 | 47329374 | 0.483186284 | 1 | loss |
| TCGA-09-1672 | 18 | 47438397 | 47777356 | 1.095815034 | 3 | amp |
| TCGA-09-1672 | 18 | 47787400 | 47809155 | 0.518904141 | 1 | loss |
| TCGA-09-1672 | 18 | 47902184 | 47918662 | 0.430626242 | 1 | loss |
| TCGA-09-1672 | 18 | 48190271 | 48333266 | 1.056637936 | 3 | amp |
| TCGA-09-1672 | 18 | 50918020 | 51692553 | 0.9387818 | 3 | amp |
| TCGA-09-1672 | 18 | 55351225 | 55373830 | 0.99260123 | 3 | amp |
| TCGA-09-1672 | 18 | 56149012 | 56274732 | 1.054253371 | 3 | amp |
| TCGA-09-1672 | 18 | 56807163 | 57021841 | 0.537805454 | 1 | loss |
| TCGA-09-1672 | 18 | 57637180 | 57863893 | 0.405225029 | 1 | loss |
| TCGA-09-1672 | 18 | 58038542 | 58039622 | 1.584808497 | 4 | amp |
| TCGA-09-1672 | 18 | 61228256 | 61328487 | 0.930063236 | 3 | amp |
| TCGA-09-1672 | 18 | 65178180 | 66381192 | 0.494932204 | 1 | loss |
| TCGA-09-1672 | 18 | 76903770 | 78005275 | 0.474217425 | 1 | loss |
| TCGA-09-1672 | 19 | 5724785 | 5778716 | 1.060198468 | 3 | amp |

| | | | | | | |
|---|---|---|---|---|---|---|
| TCGA-09-1672 | 19 | 6141497 | 6190691 | 0.942869696 | 3 | amp |
| TCGA-09-1672 | 19 | 6833497 | 6908801 | 0.956223478 | 3 | amp |
| TCGA-09-1672 | 19 | 6913632 | 6935103 | 1.544549691 | 4 | amp |
| TCGA-09-1672 | 19 | 6937187 | 7150585 | 0.932774136 | 3 | amp |
| TCGA-09-1672 | 19 | 7152687 | 7267931 | 1.279392432 | 4 | amp |
| TCGA-09-1672 | 19 | 7807875 | 7833918 | 0.968620061 | 3 | amp |
| TCGA-09-1672 | 19 | 8966612 | 8993084 | 1.158765399 | 3 | amp |
| TCGA-09-1672 | 19 | 8993338 | 9021238 | 1.719419927 | 5 | amp |
| TCGA-09-1672 | 19 | 9024106 | 9054351 | 1.248555801 | 4 | amp |
| TCGA-09-1672 | 19 | 9056158 | 9237636 | 1.811264407 | 5 | amp |
| TCGA-09-1672 | 19 | 9266644 | 9362769 | 1.007313423 | 3 | amp |
| TCGA-09-1672 | 19 | 10097165 | 10107366 | 0.890800134 | 3 | amp |
| TCGA-09-1672 | 19 | 10131872 | 10166472 | 1.48882557 | 4 | amp |
| TCGA-09-1672 | 19 | 10262027 | 10270776 | 0.952347974 | 3 | amp |
| TCGA-09-1672 | 19 | 11213290 | 11280657 | 1.098986898 | 3 | amp |
| TCGA-09-1672 | 19 | 13423478 | 13566033 | 1.306834155 | 4 | amp |
| TCGA-09-1672 | 19 | 14693933 | 14705651 | 1.579749057 | 4 | amp |
| TCGA-09-1672 | 19 | 14706046 | 14830151 | 0.981105798 | 3 | amp |
| TCGA-09-1672 | 19 | 14846910 | 15073165 | 1.238291636 | 4 | amp |
| TCGA-09-1672 | 19 | 15164221 | 15198821 | 1.077995418 | 3 | amp |
| TCGA-09-1672 | 19 | 15790990 | 15918908 | 1.019628958 | 3 | amp |
| TCGA-09-1672 | 19 | 16238778 | 16264055 | 0.939528173 | 3 | amp |
| TCGA-09-1672 | 19 | 16268012 | 16284320 | 1.364676343 | 4 | amp |
| TCGA-09-1672 | 19 | 16770730 | 16923668 | 1.130607237 | 3 | amp |
| TCGA-09-1672 | 19 | 17035943 | 17170914 | 0.914247172 | 3 | amp |
| TCGA-09-1672 | 19 | 19304698 | 19313890 | 0.990906686 | 3 | amp |
| TCGA-09-1672 | 19 | 20508127 | 20808495 | 1.122972899 | 3 | amp |
| TCGA-09-1672 | 19 | 31025679 | 31770676 | 1.432126911 | 4 | amp |
| TCGA-09-1672 | 19 | 33122263 | 33140702 | 0.980713465 | 3 | amp |
| TCGA-09-1672 | 19 | 33444463 | 33512574 | 1.091839638 | 3 | amp |
| TCGA-09-1672 | 19 | 35828610 | 35863321 | 0.849372222 | 3 | amp |
| TCGA-09-1672 | 19 | 35940572 | 35991518 | 1.337720083 | 4 | amp |
| TCGA-09-1672 | 19 | 35992971 | 36019235 | 0.943467746 | 3 | amp |
| TCGA-09-1672 | 19 | 36673341 | 36832528 | 1.263618974 | 4 | amp |
| TCGA-09-1672 | 19 | 37617974 | 37734555 | 1.056254626 | 3 | amp |
| TCGA-09-1672 | 19 | 38074814 | 38104188 | 1.083871225 | 3 | amp |
| TCGA-09-1672 | 19 | 39104818 | 39127604 | 0.935295717 | 3 | amp |
| TCGA-09-1672 | 19 | 39948257 | 39961209 | 1.089378431 | 3 | amp |
| TCGA-09-1672 | 19 | 40095219 | 40228644 | 0.965386448 | 3 | amp |
| TCGA-09-1672 | 19 | 41231204 | 41283373 | 0.981088031 | 3 | amp |
| TCGA-09-1672 | 19 | 41327339 | 41450806 | 0.955650503 | 3 | amp |
| TCGA-09-1672 | 19 | 41497175 | 41522761 | 1.409443182 | 4 | amp |
| TCGA-09-1672 | 19 | 42092755 | 42212742 | 0.867108306 | 3 | amp |
| TCGA-09-1672 | 19 | 42213538 | 42231254 | 1.36829391 | 4 | amp |
| TCGA-09-1672 | 19 | 42930379 | 43009567 | 0.877252853 | 3 | amp |
| TCGA-09-1672 | 19 | 43013200 | 43026395 | 1.38401162 | 4 | amp |
| TCGA-09-1672 | 19 | 43031132 | 43877816 | 1.033884973 | 3 | amp |
| TCGA-09-1672 | 19 | 44169444 | 44249039 | 0.906180203 | 3 | amp |
| TCGA-09-1672 | 19 | 44341147 | 44947074 | 0.960327156 | 3 | amp |
| TCGA-09-1672 | 19 | 46338290 | 46357760 | 1.046863883 | 3 | amp |

| | | | | | | |
|---|---|---|---|---|---|---|
| TCGA-09-1672 | 19 | 46375284 | 46623668 | 0.458286492 | 1 | loss |
| TCGA-09-1672 | 19 | 46627067 | 46733832 | 1.028842839 | 3 | amp |
| TCGA-09-1672 | 19 | 48374707 | 48646887 | 1.02910591 | 3 | amp |
| TCGA-09-1672 | 19 | 49388700 | 49398811 | 1.126770358 | 3 | amp |
| TCGA-09-1672 | 19 | 49793334 | 49813808 | 0.993009431 | 3 | amp |
| TCGA-09-1672 | 19 | 50491568 | 50504138 | 0.988487181 | 3 | amp |
| TCGA-09-1672 | 19 | 50510744 | 50578467 | 1.324596076 | 4 | amp |
| TCGA-09-1672 | 19 | 51358144 | 51518859 | 0.86371036 | 3 | amp |
| TCGA-09-1672 | 19 | 51628201 | 51771897 | 1.083922151 | 3 | amp |
| TCGA-09-1672 | 19 | 51979785 | 52150053 | 0.991642249 | 3 | amp |
| TCGA-09-1672 | 19 | 52195832 | 52222617 | 0.382405313 | 1 | loss |
| TCGA-09-1672 | 19 | 52249150 | 52403412 | 1.12820893 | 3 | amp |
| TCGA-09-1672 | 19 | 53268783 | 53410084 | 0.981010069 | 3 | amp |
| TCGA-09-1672 | 19 | 53878965 | 54245869 | 1.09167021 | 3 | amp |
| TCGA-09-1672 | 19 | 54544991 | 54567074 | 1.087898474 | 3 | amp |
| TCGA-09-1672 | 19 | 54778839 | 54849988 | 0.975000786 | 3 | amp |
| TCGA-09-1672 | 19 | 55174412 | 55258871 | 1.010050015 | 3 | amp |
| TCGA-09-1672 | 19 | 55267523 | 55378201 | 1.194737132 | 4 | amp |
| TCGA-09-1672 | 19 | 55385672 | 55401241 | 0.893493176 | 3 | amp |
| TCGA-09-1672 | 19 | 56235307 | 56443697 | 1.013207786 | 3 | amp |
| TCGA-09-1672 | 19 | 56459211 | 56485222 | 1.556190787 | 4 | amp |
| TCGA-09-1672 | 19 | 56487476 | 56901926 | 0.960790114 | 3 | amp |
| TCGA-09-1672 | 19 | 57866588 | 57932885 | 0.896199524 | 3 | amp |
| TCGA-09-1672 | 19 | 58212979 | 58445247 | 0.934123693 | 3 | amp |
| TCGA-09-1672 | 2 | 669434 | 7033851 | 1.048966816 | 3 | amp |
| TCGA-09-1672 | 2 | 7035870 | 8919993 | 1.309480342 | 4 | amp |
| TCGA-09-1672 | 2 | 8925835 | 9098818 | 1.007654675 | 3 | amp |
| TCGA-09-1672 | 2 | 9419421 | 9531377 | 1.524552815 | 4 | amp |
| TCGA-09-1672 | 2 | 9540116 | 16736431 | 1.032098256 | 3 | amp |
| TCGA-09-1672 | 2 | 16740670 | 17836682 | 1.531774865 | 4 | amp |
| TCGA-09-1672 | 2 | 17846762 | 20886878 | 0.97631882 | 3 | amp |
| TCGA-09-1672 | 2 | 20901280 | 21252703 | 1.422506492 | 4 | amp |
| TCGA-09-1672 | 2 | 21252708 | 24516660 | 1.026649403 | 3 | amp |
| TCGA-09-1672 | 2 | 24518482 | 24991294 | 1.279401095 | 4 | amp |
| TCGA-09-1672 | 2 | 25013244 | 25875555 | 1.064758448 | 3 | amp |
| TCGA-09-1672 | 2 | 25964861 | 26539971 | 1.29459769 | 4 | amp |
| TCGA-09-1672 | 2 | 26540336 | 26637315 | 0.957515552 | 3 | amp |
| TCGA-09-1672 | 2 | 26644121 | 27169880 | 1.342500057 | 4 | amp |
| TCGA-09-1672 | 2 | 27244996 | 27448114 | 0.923227582 | 3 | amp |
| TCGA-09-1672 | 2 | 27448533 | 27462388 | 1.317381698 | 4 | amp |
| TCGA-09-1672 | 2 | 27462525 | 27550157 | 1.118015995 | 3 | amp |
| TCGA-09-1672 | 2 | 27550859 | 27566477 | 1.614042194 | 4 | amp |
| TCGA-09-1672 | 2 | 27587246 | 27677540 | 0.863475011 | 3 | amp |
| TCGA-09-1672 | 2 | 27679298 | 27688800 | 1.38048443 | 4 | amp |
| TCGA-09-1672 | 2 | 27693758 | 27726493 | 0.954886047 | 3 | amp |
| TCGA-09-1672 | 2 | 27728523 | 27806613 | 1.400718226 | 4 | amp |
| TCGA-09-1672 | 2 | 27820907 | 27839219 | 0.932859472 | 3 | amp |
| TCGA-09-1672 | 2 | 27840328 | 28775886 | 1.459868866 | 4 | amp |
| TCGA-09-1672 | 2 | 28785846 | 28855918 | 1.961056241 | 5 | amp |
| TCGA-09-1672 | 2 | 28863769 | 29016915 | 1.535170958 | 4 | amp |

| | | | | | | |
|---|---|---|---|---|---|---|
| TCGA-09-1672 | 2 | 29022026 | 29137124 | 1.053698762 | 3 | amp |
| TCGA-09-1672 | 2 | 29140749 | 29297153 | 1.501479089 | 4 | amp |
| TCGA-09-1672 | 2 | 29344200 | 29397334 | 0.966102506 | 3 | amp |
| TCGA-09-1672 | 2 | 29404418 | 29940623 | 1.771791218 | 5 | amp |
| TCGA-09-1672 | 2 | 30142831 | 30791103 | 1.053151073 | 3 | amp |
| TCGA-09-1672 | 2 | 30862933 | 31215908 | 1.748475039 | 5 | amp |
| TCGA-09-1672 | 2 | 31360767 | 31806009 | 1.558936784 | 4 | amp |
| TCGA-09-1672 | 2 | 32093375 | 32679062 | 1.11497954 | 3 | amp |
| TCGA-09-1672 | 2 | 32688235 | 37336447 | 1.346550089 | 4 | amp |
| TCGA-09-1672 | 2 | 37341817 | 37450548 | 1.034336697 | 3 | amp |
| TCGA-09-1672 | 2 | 37454652 | 37873767 | 1.281655173 | 4 | amp |
| TCGA-09-1672 | 2 | 38177639 | 38978444 | 0.961025018 | 3 | amp |
| TCGA-09-1672 | 2 | 39006076 | 39222575 | 1.499678143 | 4 | amp |
| TCGA-09-1672 | 2 | 39224000 | 39995662 | 1.1209134 | 3 | amp |
| TCGA-09-1672 | 2 | 39996795 | 40657476 | 1.505338224 | 4 | amp |
| TCGA-09-1672 | 2 | 42280319 | 42490501 | 0.989096496 | 3 | amp |
| TCGA-09-1672 | 2 | 42491801 | 44550051 | 1.251096928 | 4 | amp |
| TCGA-09-1672 | 2 | 44550355 | 45812949 | 1.155161809 | 3 | amp |
| TCGA-09-1672 | 2 | 45826540 | 46211874 | 1.848811579 | 5 | amp |
| TCGA-09-1672 | 2 | 46228493 | 46739869 | 1.419423677 | 4 | amp |
| TCGA-09-1672 | 2 | 46803187 | 47184181 | 1.054564369 | 3 | amp |
| TCGA-09-1672 | 2 | 47202026 | 47279071 | 1.814760089 | 5 | amp |
| TCGA-09-1672 | 2 | 47287888 | 48046003 | 0.909009251 | 3 | amp |
| TCGA-09-1672 | 2 | 48046005 | 53956710 | 1.323914754 | 4 | amp |
| TCGA-09-1672 | 2 | 53977878 | 54864954 | 1.12080103 | 3 | amp |
| TCGA-09-1672 | 2 | 54870042 | 55201039 | 1.438111655 | 4 | amp |
| TCGA-09-1672 | 2 | 55201805 | 68274493 | 1.060778661 | 3 | amp |
| TCGA-09-1672 | 2 | 68352426 | 68622999 | 1.342940202 | 4 | amp |
| TCGA-09-1672 | 2 | 68691301 | 68882719 | 1.030829119 | 3 | amp |
| TCGA-09-1672 | 2 | 68962301 | 69098503 | 1.787397684 | 5 | amp |
| TCGA-09-1672 | 2 | 69172450 | 70037825 | 1.400326665 | 4 | amp |
| TCGA-09-1672 | 2 | 70039752 | 70392333 | 0.986531446 | 3 | amp |
| TCGA-09-1672 | 2 | 70392653 | 70444131 | 1.425876477 | 4 | amp |
| TCGA-09-1672 | 2 | 70451663 | 70528161 | 1.051224871 | 3 | amp |
| TCGA-09-1672 | 2 | 70529027 | 71186246 | 1.48373003 | 4 | amp |
| TCGA-09-1672 | 2 | 71187018 | 71801534 | 1.102209615 | 3 | amp |
| TCGA-09-1672 | 2 | 71816709 | 71892482 | 1.902441157 | 5 | amp |
| TCGA-09-1672 | 2 | 71894425 | 73452992 | 1.278614948 | 4 | amp |
| TCGA-09-1672 | 2 | 73455521 | 73659432 | 1.117235491 | 3 | amp |
| TCGA-09-1672 | 2 | 73675081 | 74307731 | 1.345263204 | 4 | amp |
| TCGA-09-1672 | 2 | 74316978 | 74459804 | 0.959100055 | 3 | amp |
| TCGA-09-1672 | 2 | 74460489 | 74598324 | 1.443271627 | 4 | amp |
| TCGA-09-1672 | 2 | 74598639 | 74746897 | 1.036529361 | 3 | amp |
| TCGA-09-1672 | 2 | 74776491 | 74787467 | 1.095676716 | 3 | amp |
| TCGA-09-1672 | 2 | 74789326 | 75745283 | 1.37947219 | 4 | amp |
| TCGA-09-1672 | 2 | 75879206 | 75897456 | 0.861905307 | 3 | amp |
| TCGA-09-1672 | 2 | 75899048 | 84652752 | 1.447919607 | 4 | amp |
| TCGA-09-1672 | 2 | 84658587 | 84806846 | 0.953260237 | 3 | amp |
| TCGA-09-1672 | 2 | 84811072 | 85768558 | 1.446217926 | 4 | amp |
| TCGA-09-1672 | 2 | 85768751 | 85858076 | 1.105955513 | 3 | amp |

| | | | | | | |
|---|---|---|---|---|---|---|
| TCGA-09-1672 | 2 | 85863154 | 86386846 | 1.429477476 | 4 | amp |
| TCGA-09-1672 | 2 | 86389090 | 87196454 | 1.13613865 | 3 | amp |
| TCGA-09-1672 | 2 | 87198854 | 87386791 | 1.290416181 | 4 | amp |
| TCGA-09-1672 | 2 | 87388687 | 87412758 | 1.024270661 | 3 | amp |
| TCGA-09-1672 | 2 | 87414257 | 88367571 | 1.318981271 | 4 | amp |
| TCGA-09-1672 | 2 | 88383802 | 88427562 | 1.877441863 | 5 | amp |
| TCGA-09-1672 | 2 | 88472660 | 89028885 | 1.089267306 | 3 | amp |
| TCGA-09-1672 | 2 | 89034040 | 89197332 | 1.301742652 | 4 | amp |
| TCGA-09-1672 | 2 | 89246786 | 90273797 | 1.934373258 | 5 | amp |
| TCGA-09-1672 | 2 | 90273904 | 95843410 | 0.993719033 | 3 | amp |
| TCGA-09-1672 | 2 | 95845870 | 95952332 | 1.267559523 | 4 | amp |
| TCGA-09-1672 | 2 | 95952525 | 96040225 | 1.906925205 | 5 | amp |
| TCGA-09-1672 | 2 | 96040570 | 96458297 | 1.399783593 | 4 | amp |
| TCGA-09-1672 | 2 | 96458969 | 97261417 | 1.129370925 | 3 | amp |
| TCGA-09-1672 | 2 | 97267326 | 97405803 | 1.435988866 | 4 | amp |
| TCGA-09-1672 | 2 | 97426865 | 97509775 | 0.952435204 | 3 | amp |
| TCGA-09-1672 | 2 | 97513959 | 97866286 | 1.272519907 | 4 | amp |
| TCGA-09-1672 | 2 | 97879110 | 97915952 | 1.114136244 | 3 | amp |
| TCGA-09-1672 | 2 | 98000732 | 98038159 | 2.114904876 | 5 | amp |
| TCGA-09-1672 | 2 | 98123456 | 98709773 | 1.075364891 | 3 | amp |
| TCGA-09-1672 | 2 | 98732101 | 99180133 | 1.443773772 | 4 | amp |
| TCGA-09-1672 | 2 | 99181044 | 99811675 | 0.94726329 | 3 | amp |
| TCGA-09-1672 | 2 | 99811983 | 99912171 | 1.490510385 | 4 | amp |
| TCGA-09-1672 | 2 | 99936083 | 100079140 | 1.125572252 | 3 | amp |
| TCGA-09-1672 | 2 | 100081350 | 101706830 | 1.33605081 | 4 | amp |
| TCGA-09-1672 | 2 | 101869603 | 102441895 | 0.987146968 | 3 | amp |
| TCGA-09-1672 | 2 | 102445950 | 102626335 | 1.280944375 | 4 | amp |
| TCGA-09-1672 | 2 | 102632302 | 102984586 | 1.000541746 | 3 | amp |
| TCGA-09-1672 | 2 | 102988375 | 103130749 | 1.393099191 | 4 | amp |
| TCGA-09-1672 | 2 | 103136305 | 103300813 | 1.919128494 | 5 | amp |
| TCGA-09-1672 | 2 | 103310827 | 105894159 | 1.056626421 | 3 | amp |
| TCGA-09-1672 | 2 | 105896799 | 107055648 | 1.299216932 | 4 | amp |
| TCGA-09-1672 | 2 | 107057732 | 108869909 | 1.133384164 | 3 | amp |
| TCGA-09-1672 | 2 | 108871970 | 108924923 | 1.897738017 | 5 | amp |
| TCGA-09-1672 | 2 | 108994757 | 110577376 | 1.088885119 | 3 | amp |
| TCGA-09-1672 | 2 | 110579466 | 110849383 | 1.31513542 | 4 | amp |
| TCGA-09-1672 | 2 | 110881323 | 110962570 | 0.983650607 | 3 | amp |
| TCGA-09-1672 | 2 | 111037146 | 112536359 | 1.259271182 | 4 | amp |
| TCGA-09-1672 | 2 | 112537858 | 112636587 | 1.014539258 | 3 | amp |
| TCGA-09-1672 | 2 | 112638175 | 112786484 | 1.574466964 | 4 | amp |
| TCGA-09-1672 | 2 | 112817466 | 113537277 | 1.047733035 | 3 | amp |
| TCGA-09-1672 | 2 | 113539171 | 113943882 | 1.517205652 | 4 | amp |
| TCGA-09-1672 | 2 | 113949911 | 118575371 | 1.026199427 | 3 | amp |
| TCGA-09-1672 | 2 | 118577176 | 120362401 | 1.35676007 | 4 | amp |
| TCGA-09-1672 | 2 | 120362417 | 121107482 | 1.036905989 | 3 | amp |
| TCGA-09-1672 | 2 | 121554820 | 122217748 | 1.344599849 | 4 | amp |
| TCGA-09-1672 | 2 | 122218662 | 125175213 | 1.050571142 | 3 | amp |
| TCGA-09-1672 | 2 | 125192042 | 127811069 | 1.479116089 | 4 | amp |
| TCGA-09-1672 | 2 | 127818103 | 128892552 | 1.062464322 | 3 | amp |
| TCGA-09-1672 | 2 | 128896238 | 128944434 | 1.334983018 | 4 | amp |

| | | | | | | |
|---|---|---|---|---|---|---|
| TCGA-09-1672 | 2 | 128944985 | 131098666 | 0.941430045 | 3 | amp |
| TCGA-09-1672 | 2 | 132258529 | 133427539 | 0.978951602 | 3 | amp |
| TCGA-09-1672 | 2 | 133430809 | 135309700 | 1.627354026 | 4 | amp |
| TCGA-09-1672 | 2 | 135470709 | 135892921 | 0.940648928 | 3 | amp |
| TCGA-09-1672 | 2 | 135893077 | 136609172 | 1.337533388 | 4 | amp |
| TCGA-09-1672 | 2 | 136610270 | 136627935 | 0.825692361 | 3 | amp |
| TCGA-09-1672 | 2 | 136630219 | 148680686 | 1.290176865 | 4 | amp |
| TCGA-09-1672 | 2 | 148683544 | 149221517 | 1.017152862 | 3 | amp |
| TCGA-09-1672 | 2 | 149225889 | 151343976 | 1.432385525 | 4 | amp |
| TCGA-09-1672 | 2 | 152107381 | 152319042 | 0.972203228 | 3 | amp |
| TCGA-09-1672 | 2 | 152319335 | 152586209 | 1.475688486 | 4 | amp |
| TCGA-09-1672 | 2 | 152589592 | 153378558 | 1.017757542 | 3 | amp |
| TCGA-09-1672 | 2 | 153399232 | 153526939 | 1.350281051 | 4 | amp |
| TCGA-09-1672 | 2 | 153527780 | 157332728 | 1.104712857 | 3 | amp |
| TCGA-09-1672 | 2 | 157352521 | 158991450 | 1.368166084 | 4 | amp |
| TCGA-09-1672 | 2 | 159028577 | 159459658 | 0.922078587 | 3 | amp |
| TCGA-09-1672 | 2 | 159477447 | 159518023 | 1.471399531 | 4 | amp |
| TCGA-09-1672 | 2 | 159519384 | 160031699 | 1.755649516 | 5 | amp |
| TCGA-09-1672 | 2 | 160032795 | 160092742 | 1.400369612 | 4 | amp |
| TCGA-09-1672 | 2 | 160104832 | 160243120 | 0.978255096 | 3 | amp |
| TCGA-09-1672 | 2 | 160245805 | 160605464 | 1.381583548 | 4 | amp |
| TCGA-09-1672 | 2 | 160608877 | 160692193 | 0.994810489 | 3 | amp |
| TCGA-09-1672 | 2 | 160697232 | 162036282 | 1.366503737 | 4 | amp |
| TCGA-09-1672 | 2 | 162059961 | 162933947 | 1.009099884 | 3 | amp |
| TCGA-09-1672 | 2 | 163000357 | 165365072 | 1.301753841 | 4 | amp |
| TCGA-09-1672 | 2 | 165365246 | 165476382 | 0.831610614 | 3 | amp |
| TCGA-09-1672 | 2 | 165542408 | 166756394 | 1.323786572 | 4 | amp |
| TCGA-09-1672 | 2 | 166758175 | 166806289 | 0.958155655 | 3 | amp |
| TCGA-09-1672 | 2 | 166847760 | 170003478 | 1.358723633 | 4 | amp |
| TCGA-09-1672 | 2 | 170007351 | 170027238 | 1.864942066 | 5 | amp |
| TCGA-09-1672 | 2 | 170028464 | 170151224 | 1.42330401 | 4 | amp |
| TCGA-09-1672 | 2 | 170471044 | 170815051 | 0.94582836 | 3 | amp |
| TCGA-09-1672 | 2 | 170843109 | 171715418 | 1.390409103 | 4 | amp |
| TCGA-09-1672 | 2 | 171716155 | 173330448 | 1.122593338 | 3 | amp |
| TCGA-09-1672 | 2 | 173332188 | 173848325 | 1.400762962 | 4 | amp |
| TCGA-09-1672 | 2 | 173850130 | 173901447 | 1.822640821 | 5 | amp |
| TCGA-09-1672 | 2 | 173913294 | 174068620 | 1.393035582 | 4 | amp |
| TCGA-09-1672 | 2 | 174074449 | 174097253 | 2.134391746 | 5 | amp |
| TCGA-09-1672 | 2 | 174103071 | 175446222 | 1.0135541 | 3 | amp |
| TCGA-09-1672 | 2 | 175450185 | 175673821 | 1.495060748 | 4 | amp |
| TCGA-09-1672 | 2 | 175676217 | 175962366 | 1.029719734 | 3 | amp |
| TCGA-09-1672 | 2 | 178357841 | 178378651 | 1.08051554 | 3 | amp |
| TCGA-09-1672 | 2 | 178385986 | 178937188 | 1.308631879 | 4 | amp |
| TCGA-09-1672 | 2 | 178968989 | 178990926 | 0.945526606 | 3 | amp |
| TCGA-09-1672 | 2 | 179170902 | 179257299 | 1.42325622 | 4 | amp |
| TCGA-09-1672 | 2 | 179258980 | 179392066 | 0.855808313 | 3 | amp |
| TCGA-09-1672 | 2 | 179392143 | 179407320 | 1.469394692 | 4 | amp |
| TCGA-09-1672 | 2 | 179407352 | 179528843 | 2.01060619 | 5 | amp |
| TCGA-09-1672 | 2 | 179529165 | 179566993 | 1.488587761 | 4 | amp |
| TCGA-09-1672 | 2 | 179567165 | 179665445 | 1.798746091 | 5 | amp |

| | | | | | | |
|---|---|---|---|---|---|---|
| TCGA-09-1672 | 2 | 179666846 | 180838006 | 1.343834149 | 4 | amp |
| TCGA-09-1672 | 2 | 180838324 | 189855816 | 1.107106073 | 3 | amp |
| TCGA-09-1672 | 2 | 189856174 | 190430382 | 1.236968938 | 4 | amp |
| TCGA-09-1672 | 2 | 190436481 | 196621052 | 1.080789272 | 3 | amp |
| TCGA-09-1672 | 2 | 196636378 | 196774954 | 1.294717619 | 4 | amp |
| TCGA-09-1672 | 2 | 196786806 | 197028166 | 0.996682471 | 3 | amp |
| TCGA-09-1672 | 2 | 197065996 | 197298181 | 1.419462502 | 4 | amp |
| TCGA-09-1672 | 2 | 197504436 | 197640844 | 1.113431317 | 3 | amp |
| TCGA-09-1672 | 2 | 197641131 | 197674144 | 1.385664708 | 4 | amp |
| TCGA-09-1672 | 2 | 197705876 | 197878455 | 0.96820676 | 3 | amp |
| TCGA-09-1672 | 2 | 197889865 | 198266306 | 1.393815554 | 4 | amp |
| TCGA-09-1672 | 2 | 198266418 | 198646637 | 0.967371653 | 3 | amp |
| TCGA-09-1672 | 2 | 198650656 | 200246599 | 1.592575177 | 4 | amp |
| TCGA-09-1672 | 2 | 200298028 | 200813237 | 0.890640528 | 3 | amp |
| TCGA-09-1672 | 2 | 200820469 | 201358005 | 1.332706317 | 4 | amp |
| TCGA-09-1672 | 2 | 201362462 | 201470364 | 0.926050717 | 3 | amp |
| TCGA-09-1672 | 2 | 201473662 | 201527694 | 1.390427664 | 4 | amp |
| TCGA-09-1672 | 2 | 201531382 | 201752396 | 0.86542111 | 3 | amp |
| TCGA-09-1672 | 2 | 201756714 | 201800638 | 1.362371559 | 4 | amp |
| TCGA-09-1672 | 2 | 201802598 | 202052535 | 0.990340918 | 3 | amp |
| TCGA-09-1672 | 2 | 202057654 | 202139703 | 1.362491999 | 4 | amp |
| TCGA-09-1672 | 2 | 202207063 | 202252754 | 1.483421134 | 4 | amp |
| TCGA-09-1672 | 2 | 202254004 | 202549887 | 0.974316926 | 3 | amp |
| TCGA-09-1672 | 2 | 202550587 | 203383779 | 1.336749905 | 4 | amp |
| TCGA-09-1672 | 2 | 203384806 | 204313569 | 1.034658714 | 3 | amp |
| TCGA-09-1672 | 2 | 204319117 | 206893079 | 1.433469531 | 4 | amp |
| TCGA-09-1672 | 2 | 206911221 | 207310259 | 1.079867822 | 3 | amp |
| TCGA-09-1672 | 2 | 207345903 | 209101907 | 1.351421632 | 4 | amp |
| TCGA-09-1672 | 2 | 209163313 | 209195452 | 1.357421586 | 4 | amp |
| TCGA-09-1672 | 2 | 209198007 | 209219391 | 0.992669775 | 3 | amp |
| TCGA-09-1672 | 2 | 209219911 | 210590533 | 1.366283145 | 4 | amp |
| TCGA-09-1672 | 2 | 210594569 | 211179838 | 1.023783309 | 3 | amp |
| TCGA-09-1672 | 2 | 211299188 | 214013396 | 1.342937528 | 4 | amp |
| TCGA-09-1672 | 2 | 214160750 | 215661872 | 1.115110513 | 3 | amp |
| TCGA-09-1672 | 2 | 215674094 | 215884571 | 1.416511799 | 4 | amp |
| TCGA-09-1672 | 2 | 215890389 | 216203685 | 1.08699347 | 3 | amp |
| TCGA-09-1672 | 2 | 216209487 | 218747161 | 1.461758197 | 4 | amp |
| TCGA-09-1672 | 2 | 218749702 | 219029947 | 1.971724633 | 5 | amp |
| TCGA-09-1672 | 2 | 219082134 | 219288598 | 1.166996294 | 3 | amp |
| TCGA-09-1672 | 2 | 219288976 | 219314062 | 2.093416334 | 5 | amp |
| TCGA-09-1672 | 2 | 219319636 | 219480533 | 1.332706004 | 4 | amp |
| TCGA-09-1672 | 2 | 219480585 | 219501306 | 2.017620141 | 5 | amp |
| TCGA-09-1672 | 2 | 219503056 | 219610528 | 1.479755611 | 4 | amp |
| TCGA-09-1672 | 2 | 219610825 | 219677493 | 1.971941302 | 5 | amp |
| TCGA-09-1672 | 2 | 219677582 | 219692152 | 1.337191769 | 4 | amp |
| TCGA-09-1672 | 2 | 219692234 | 220440251 | 0.97292919 | 3 | amp |
| TCGA-09-1672 | 2 | 220462581 | 220471876 | 1.998455708 | 5 | amp |
| TCGA-09-1672 | 2 | 220493089 | 228012223 | 1.39490234 | 4 | amp |
| TCGA-09-1672 | 2 | 228102621 | 228122399 | 1.82603119 | 5 | amp |
| TCGA-09-1672 | 2 | 228124490 | 231037677 | 1.280052236 | 4 | amp |

| | | | | | | |
|---|---|---|---|---|---|---|
| TCGA-09-1672 | 2 | 231041320 | 231118141 | 1.90389589 | 5 | amp |
| TCGA-09-1672 | 2 | 231120146 | 231177409 | 1.025542957 | 3 | amp |
| TCGA-09-1672 | 2 | 231191940 | 231624833 | 1.284697832 | 4 | amp |
| TCGA-09-1672 | 2 | 231655518 | 231683444 | 0.840518447 | 3 | amp |
| TCGA-09-1672 | 2 | 231738051 | 232160998 | 1.362354591 | 4 | amp |
| TCGA-09-1672 | 2 | 232196466 | 232390184 | 0.845923254 | 3 | amp |
| TCGA-09-1672 | 2 | 232392800 | 233323519 | 1.270585705 | 4 | amp |
| TCGA-09-1672 | 2 | 233344822 | 233421242 | 1.120053737 | 3 | amp |
| TCGA-09-1672 | 2 | 233422510 | 234191423 | 1.417854445 | 4 | amp |
| TCGA-09-1672 | 2 | 234197274 | 234237311 | 1.932827627 | 5 | amp |
| TCGA-09-1672 | 2 | 234238120 | 234251004 | 1.38268965 | 4 | amp |
| TCGA-09-1672 | 2 | 234254993 | 238233496 | 1.067725613 | 3 | amp |
| TCGA-09-1672 | 2 | 238234164 | 238305474 | 1.334746707 | 4 | amp |
| TCGA-09-1672 | 2 | 238402034 | 242170386 | 0.993685338 | 3 | amp |
| TCGA-09-1672 | 2 | 242173186 | 242192470 | 1.388881944 | 4 | amp |
| TCGA-09-1672 | 2 | 242192799 | 243160772 | 0.998928825 | 3 | amp |
| TCGA-09-1672 | 20 | 68319 | 1301093 | 0.995584233 | 3 | amp |
| TCGA-09-1672 | 20 | 1352699 | 2098027 | 1.227099672 | 4 | amp |
| TCGA-09-1672 | 20 | 2290239 | 2298183 | 1.865634472 | 5 | amp |
| TCGA-09-1672 | 20 | 2306524 | 2560708 | 1.067479625 | 3 | amp |
| TCGA-09-1672 | 20 | 2572944 | 2605099 | 1.493159397 | 4 | amp |
| TCGA-09-1672 | 20 | 2616520 | 2844938 | 0.962024071 | 3 | amp |
| TCGA-09-1672 | 20 | 2844969 | 2985806 | 1.278168781 | 4 | amp |
| TCGA-09-1672 | 20 | 3236604 | 3245186 | 1.079805968 | 3 | amp |
| TCGA-09-1672 | 20 | 3251020 | 3303380 | 1.390664366 | 4 | amp |
| TCGA-09-1672 | 20 | 3305486 | 5149333 | 0.992522023 | 3 | amp |
| TCGA-09-1672 | 20 | 5154116 | 5170933 | 1.439764345 | 4 | amp |
| TCGA-09-1672 | 20 | 5261071 | 9510497 | 1.01938436 | 3 | amp |
| TCGA-09-1672 | 20 | 9520036 | 9547085 | 1.72576395 | 5 | amp |
| TCGA-09-1672 | 20 | 9560764 | 10035298 | 0.845286829 | 3 | amp |
| TCGA-09-1672 | 20 | 10036078 | 13463988 | 1.284062001 | 4 | amp |
| TCGA-09-1672 | 20 | 13767917 | 15412124 | 0.996566653 | 3 | amp |
| TCGA-09-1672 | 20 | 15480395 | 17489647 | 1.204227217 | 4 | amp |
| TCGA-09-1672 | 20 | 17492576 | 21324906 | 1.032531267 | 3 | amp |
| TCGA-09-1672 | 20 | 23375537 | 23421133 | 0.918339399 | 3 | amp |
| TCGA-09-1672 | 20 | 23424565 | 23966420 | 1.261057799 | 4 | amp |
| TCGA-09-1672 | 20 | 23966464 | 25028828 | 1.041041372 | 3 | amp |
| TCGA-09-1672 | 20 | 29845453 | 30616939 | 1.023369114 | 3 | amp |
| TCGA-09-1672 | 20 | 30617493 | 30733230 | 1.296990025 | 4 | amp |
| TCGA-09-1672 | 20 | 30734532 | 30803282 | 0.916100577 | 3 | amp |
| TCGA-09-1672 | 20 | 30804407 | 30918152 | 1.626905027 | 4 | amp |
| TCGA-09-1672 | 20 | 30919013 | 31062517 | 0.87452937 | 3 | amp |
| TCGA-09-1672 | 20 | 31277973 | 31611228 | 1.250498587 | 4 | amp |
| TCGA-09-1672 | 20 | 31619411 | 31644545 | 1.742351133 | 5 | amp |
| TCGA-09-1672 | 20 | 31647175 | 31805503 | 1.249529127 | 4 | amp |
| TCGA-09-1672 | 20 | 31811588 | 31830372 | 2.036059758 | 5 | amp |
| TCGA-09-1672 | 20 | 31873876 | 31948346 | 1.262463141 | 4 | amp |
| TCGA-09-1672 | 20 | 31996298 | 32257565 | 1.266623372 | 4 | amp |
| TCGA-09-1672 | 20 | 32258453 | 33045296 | 0.90620068 | 3 | amp |
| TCGA-09-1672 | 20 | 33104174 | 33501651 | 1.054248525 | 3 | amp |

| | | | | | | |
|---|---|---|---|---|---|---|
| TCGA-09-1672 | 20 | 33501846 | 33529693 | 1.339589336 | 4 | amp |
| TCGA-09-1672 | 20 | 33530210 | 34041805 | 0.966359126 | 3 | amp |
| TCGA-09-1672 | 20 | 34051337 | 34136649 | 1.320051767 | 4 | amp |
| TCGA-09-1672 | 20 | 34319809 | 35242893 | 1.072143757 | 3 | amp |
| TCGA-09-1672 | 20 | 35243582 | 35293731 | 1.416957111 | 4 | amp |
| TCGA-09-1672 | 20 | 35294683 | 35563631 | 1.030035692 | 3 | amp |
| TCGA-09-1672 | 20 | 35786250 | 36953265 | 1.159125247 | 3 | amp |
| TCGA-09-1672 | 20 | 36954631 | 36983837 | 1.971326537 | 5 | amp |
| TCGA-09-1672 | 20 | 36989299 | 37000004 | 1.33160693 | 4 | amp |
| TCGA-09-1672 | 20 | 37001661 | 37617567 | 0.952921686 | 3 | amp |
| TCGA-09-1672 | 20 | 37620067 | 39792900 | 1.261821269 | 4 | amp |
| TCGA-09-1672 | 20 | 39793536 | 39993855 | 1.014429413 | 3 | amp |
| TCGA-09-1672 | 20 | 40033200 | 41514629 | 1.442133339 | 4 | amp |
| TCGA-09-1672 | 20 | 42086666 | 42158015 | 0.851152682 | 3 | amp |
| TCGA-09-1672 | 20 | 42158841 | 42213690 | 1.26678086 | 4 | amp |
| TCGA-09-1672 | 20 | 42252457 | 42635489 | 1.27619814 | 4 | amp |
| TCGA-09-1672 | 20 | 42885738 | 43142720 | 1.078402805 | 3 | amp |
| TCGA-09-1672 | 20 | 43243122 | 43264947 | 1.352875137 | 4 | amp |
| TCGA-09-1672 | 20 | 43348525 | 44108777 | 0.91649909 | 3 | amp |
| TCGA-09-1672 | 20 | 44163034 | 44187680 | 1.982447694 | 5 | amp |
| TCGA-09-1672 | 20 | 44190743 | 44637754 | 0.995710996 | 3 | amp |
| TCGA-09-1672 | 20 | 44707954 | 45012153 | 0.921172353 | 3 | amp |
| TCGA-09-1672 | 20 | 45014718 | 45878226 | 1.266116515 | 4 | amp |
| TCGA-09-1672 | 20 | 45890951 | 45938993 | 1.676477349 | 5 | amp |
| TCGA-09-1672 | 20 | 45947675 | 46305909 | 1.352871682 | 4 | amp |
| TCGA-09-1672 | 20 | 46307333 | 47282916 | 0.997316916 | 3 | amp |
| TCGA-09-1672 | 20 | 47292678 | 47645255 | 1.328786547 | 4 | amp |
| TCGA-09-1672 | 20 | 47648494 | 48166784 | 1.088389474 | 3 | amp |
| TCGA-09-1672 | 20 | 48252802 | 48263618 | 1.674419379 | 5 | amp |
| TCGA-09-1672 | 20 | 48273051 | 49434843 | 1.08706065 | 3 | amp |
| TCGA-09-1672 | 20 | 49446806 | 49520539 | 1.411828143 | 4 | amp |
| TCGA-09-1672 | 20 | 49551660 | 49571832 | 0.442952352 | 1 | loss |
| TCGA-09-1672 | 20 | 49574859 | 50071274 | 0.931290825 | 3 | amp |
| TCGA-09-1672 | 20 | 50090452 | 50346564 | 1.326909594 | 4 | amp |
| TCGA-09-1672 | 20 | 50400773 | 54970812 | 1.04286514 | 3 | amp |
| TCGA-09-1672 | 20 | 54972221 | 55111540 | 1.340050957 | 4 | amp |
| TCGA-09-1672 | 20 | 55204534 | 55803500 | 0.975929523 | 3 | amp |
| TCGA-09-1672 | 20 | 55949609 | 56934770 | 1.035316478 | 3 | amp |
| TCGA-09-1672 | 20 | 57781885 | 58422237 | 1.135706145 | 3 | amp |
| TCGA-09-1672 | 20 | 58533659 | 60448989 | 1.123658495 | 3 | amp |
| TCGA-09-1672 | 21 | 9483321 | 9590404 | 0.735964737 | 3 | amp |
| TCGA-09-1672 | 22 | 17385174 | 17414938 | 1.582824913 | 4 | amp |
| TCGA-09-1672 | 22 | 17443544 | 17684677 | 0.929466149 | 3 | amp |
| TCGA-09-1672 | 22 | 17687950 | 17990946 | 1.503555027 | 4 | amp |
| TCGA-09-1672 | 22 | 18003127 | 19420889 | 1.031614955 | 3 | amp |
| TCGA-09-1672 | 22 | 21068861 | 21242165 | 1.457701384 | 4 | amp |
| TCGA-09-1672 | 22 | 21272167 | 22869979 | 1.024617611 | 3 | amp |
| TCGA-09-1672 | 22 | 22890446 | 23248852 | 1.517145308 | 4 | amp |
| TCGA-09-1672 | 22 | 23252698 | 23265106 | 2.226405797 | 5 | amp |
| TCGA-09-1672 | 22 | 23401557 | 23955896 | 1.056455179 | 3 | amp |

| | | | | | | |
|---|---|---|---|---|---|---|
| TCGA-09-1672 | 22 | 23956301 | 23982852 | 1.64759987 | 4 | amp |
| TCGA-09-1672 | 22 | 24034156 | 25044351 | 1.047259417 | 3 | amp |
| TCGA-09-1672 | 22 | 25155775 | 26778061 | 1.334205251 | 4 | amp |
| TCGA-09-1672 | 22 | 26829643 | 29142043 | 0.989728662 | 3 | amp |
| TCGA-09-1672 | 22 | 29734900 | 29921940 | 1.342228754 | 4 | amp |
| TCGA-09-1672 | 22 | 29924026 | 30165747 | 1.005776269 | 3 | amp |
| TCGA-09-1672 | 22 | 30184937 | 30421824 | 1.414484339 | 4 | amp |
| TCGA-09-1672 | 22 | 30855948 | 30866591 | 1.994489465 | 5 | amp |
| TCGA-09-1672 | 22 | 30867828 | 31662144 | 0.992972766 | 3 | amp |
| TCGA-09-1672 | 22 | 31816206 | 31854668 | 1.369991376 | 4 | amp |
| TCGA-09-1672 | 22 | 31858898 | 31974462 | 1.062208379 | 3 | amp |
| TCGA-09-1672 | 22 | 32046664 | 32482320 | 1.460631142 | 4 | amp |
| TCGA-09-1672 | 22 | 32487553 | 32853431 | 1.188273217 | 3 | amp |
| TCGA-09-1672 | 22 | 32909594 | 35481746 | 1.681478784 | 5 | amp |
| TCGA-09-1672 | 22 | 35713851 | 36123289 | 1.396213448 | 4 | amp |
| TCGA-09-1672 | 22 | 36124737 | 36334979 | 0.996781714 | 3 | amp |
| TCGA-09-1672 | 22 | 36537166 | 36629263 | 2.062555587 | 5 | amp |
| TCGA-09-1672 | 22 | 36714160 | 38106623 | 0.982229364 | 3 | amp |
| TCGA-09-1672 | 22 | 38333730 | 38363201 | 1.426631241 | 4 | amp |
| TCGA-09-1672 | 22 | 38363616 | 38422358 | 1.025727095 | 3 | amp |
| TCGA-09-1672 | 22 | 39355498 | 39440291 | 1.514887856 | 4 | amp |
| TCGA-09-1672 | 22 | 39440943 | 39448760 | 2.316073097 | 5 | amp |
| TCGA-09-1672 | 22 | 39473314 | 39818023 | 0.962075421 | 3 | amp |
| TCGA-09-1672 | 22 | 40343089 | 40719291 | 1.070707661 | 3 | amp |
| TCGA-09-1672 | 22 | 41264940 | 41924085 | 1.058104605 | 3 | amp |
| TCGA-09-1672 | 22 | 42125665 | 42172308 | 1.530709891 | 4 | amp |
| TCGA-09-1672 | 22 | 42174703 | 42221914 | 1.026926268 | 3 | amp |
| TCGA-09-1672 | 22 | 42262778 | 42274156 | 1.842699205 | 5 | amp |
| TCGA-09-1672 | 22 | 42276647 | 42297041 | 1.098163214 | 3 | amp |
| TCGA-09-1672 | 22 | 43193512 | 44559833 | 0.971960358 | 3 | amp |
| TCGA-09-1672 | 3 | 361444 | 3170918 | 1.564465851 | 4 | amp |
| TCGA-09-1672 | 3 | 3178890 | 3216959 | 0.833790953 | 3 | amp |
| TCGA-09-1672 | 3 | 3886320 | 4693914 | 1.523113772 | 4 | amp |
| TCGA-09-1672 | 3 | 4695487 | 4856939 | 1.940881991 | 5 | amp |
| TCGA-09-1672 | 3 | 5241268 | 9057467 | 1.457245598 | 4 | amp |
| TCGA-09-1672 | 3 | 9066923 | 9407058 | 1.939086365 | 5 | amp |
| TCGA-09-1672 | 3 | 9408570 | 9757070 | 1.462198625 | 4 | amp |
| TCGA-09-1672 | 3 | 9757123 | 9831701 | 1.089435714 | 3 | amp |
| TCGA-09-1672 | 3 | 9832896 | 9935069 | 1.479066469 | 4 | amp |
| TCGA-09-1672 | 3 | 9944562 | 10136069 | 1.091529483 | 3 | amp |
| TCGA-09-1672 | 3 | 10136830 | 10219732 | 1.349853644 | 4 | amp |
| TCGA-09-1672 | 3 | 10241984 | 10258734 | 2.111108995 | 5 | amp |
| TCGA-09-1672 | 3 | 10261358 | 10357122 | 1.554914192 | 4 | amp |
| TCGA-09-1672 | 3 | 10359665 | 10392291 | 1.054428662 | 3 | amp |
| TCGA-09-1672 | 3 | 10400371 | 10452520 | 1.890166102 | 5 | amp |
| TCGA-09-1672 | 3 | 10490977 | 12660236 | 1.529240363 | 4 | amp |
| TCGA-09-1672 | 3 | 12776994 | 12791354 | 2.070258493 | 5 | amp |
| TCGA-09-1672 | 3 | 12828859 | 13361490 | 1.32378124 | 4 | amp |
| TCGA-09-1672 | 3 | 13362999 | 13427960 | 1.894346521 | 5 | amp |
| TCGA-09-1672 | 3 | 13429727 | 14209938 | 1.377613889 | 4 | amp |

| | | | | | | |
|---|---|---|---|---|---|---|
| TCGA-09-1672 | 3 | 14211933 | 14485376 | 0.929408279 | 3 | amp |
| TCGA-09-1672 | 3 | 14487171 | 14863042 | 1.560722066 | 4 | amp |
| TCGA-09-1672 | 3 | 14905580 | 15074066 | 1.782616077 | 5 | amp |
| TCGA-09-1672 | 3 | 15076126 | 16475570 | 1.486051182 | 4 | amp |
| TCGA-09-1672 | 3 | 16535183 | 16640151 | 0.828667699 | 3 | amp |
| TCGA-09-1672 | 3 | 16646620 | 20212321 | 1.380627777 | 4 | amp |
| TCGA-09-1672 | 3 | 20212479 | 24018931 | 1.075281077 | 3 | amp |
| TCGA-09-1672 | 3 | 24164315 | 25668368 | 1.479122636 | 4 | amp |
| TCGA-09-1672 | 3 | 25668672 | 25836023 | 1.055434953 | 3 | amp |
| TCGA-09-1672 | 3 | 26751133 | 27418350 | 1.445184467 | 4 | amp |
| TCGA-09-1672 | 3 | 27424632 | 28304886 | 1.055611154 | 3 | amp |
| TCGA-09-1672 | 3 | 28357808 | 30664853 | 1.453284493 | 4 | amp |
| TCGA-09-1672 | 3 | 30686202 | 30769940 | 1.941258194 | 5 | amp |
| TCGA-09-1672 | 3 | 30819655 | 32495799 | 1.48635186 | 4 | amp |
| TCGA-09-1672 | 3 | 32525400 | 32761784 | 0.988236065 | 3 | amp |
| TCGA-09-1672 | 3 | 32766896 | 37512681 | 1.376436427 | 4 | amp |
| TCGA-09-1672 | 3 | 37514807 | 37567599 | 1.946914054 | 5 | amp |
| TCGA-09-1672 | 3 | 37574761 | 38013003 | 1.53308967 | 4 | amp |
| TCGA-09-1672 | 3 | 38017142 | 38134385 | 1.098569273 | 3 | amp |
| TCGA-09-1672 | 3 | 38135026 | 38163315 | 1.550470449 | 4 | amp |
| TCGA-09-1672 | 3 | 38163473 | 38266248 | 1.10480786 | 3 | amp |
| TCGA-09-1672 | 3 | 38271141 | 38294404 | 1.389033844 | 4 | amp |
| TCGA-09-1672 | 3 | 38307300 | 38357245 | 1.825565625 | 5 | amp |
| TCGA-09-1672 | 3 | 38357764 | 38538059 | 1.321448776 | 4 | amp |
| TCGA-09-1672 | 3 | 38591770 | 38674841 | 1.428182783 | 4 | amp |
| TCGA-09-1672 | 3 | 38738786 | 38805101 | 1.906868615 | 5 | amp |
| TCGA-09-1672 | 3 | 38812692 | 38967012 | 1.393613814 | 4 | amp |
| TCGA-09-1672 | 3 | 38968207 | 39145063 | 0.982153864 | 3 | amp |
| TCGA-09-1672 | 3 | 39149281 | 39374890 | 1.398844222 | 4 | amp |
| TCGA-09-1672 | 3 | 39425159 | 40192726 | 1.02651729 | 3 | amp |
| TCGA-09-1672 | 3 | 40204200 | 40299700 | 1.549953062 | 4 | amp |
| TCGA-09-1672 | 3 | 40351357 | 40574323 | 1.149881628 | 3 | amp |
| TCGA-09-1672 | 3 | 40803147 | 41657243 | 1.454874372 | 4 | amp |
| TCGA-09-1672 | 3 | 41705053 | 41996302 | 1.100744188 | 3 | amp |
| TCGA-09-1672 | 3 | 42132946 | 42448793 | 1.356169027 | 4 | amp |
| TCGA-09-1672 | 3 | 42560708 | 44449215 | 1.152031441 | 3 | amp |
| TCGA-09-1672 | 3 | 44487978 | 44493009 | 2.252598542 | 5 | amp |
| TCGA-09-1672 | 3 | 44496556 | 44541702 | 1.479629144 | 4 | amp |
| TCGA-09-1672 | 3 | 44544442 | 44906721 | 1.046110928 | 3 | amp |
| TCGA-09-1672 | 3 | 44916119 | 44935247 | 1.583582548 | 4 | amp |
| TCGA-09-1672 | 3 | 44942952 | 44975487 | 1.950818447 | 5 | amp |
| TCGA-09-1672 | 3 | 44986571 | 45755614 | 1.535933768 | 4 | amp |
| TCGA-09-1672 | 3 | 45760982 | 45874637 | 1.005283341 | 3 | amp |
| TCGA-09-1672 | 3 | 45875699 | 46245810 | 1.600482569 | 4 | amp |
| TCGA-09-1672 | 3 | 46306613 | 46450627 | 2.258383751 | 5 | amp |
| TCGA-09-1672 | 3 | 46477642 | 46901171 | 1.388271865 | 4 | amp |
| TCGA-09-1672 | 3 | 46902090 | 47049677 | 1.132475285 | 3 | amp |
| TCGA-09-1672 | 3 | 47049731 | 47676876 | 1.306367826 | 4 | amp |
| TCGA-09-1672 | 3 | 47677508 | 47910820 | 1.170987796 | 3 | amp |
| TCGA-09-1672 | 3 | 47912238 | 48209444 | 1.371128428 | 4 | amp |

| | | | | | | |
|---|---|---|---|---|---|---|
| TCGA-09-1672 | 3 | 48215710 | 48369857 | 1.044954347 | 3 | amp |
| TCGA-09-1672 | 3 | 48414212 | 48423613 | 1.561701674 | 4 | amp |
| TCGA-09-1672 | 3 | 48436014 | 48493339 | 1.110791963 | 3 | amp |
| TCGA-09-1672 | 3 | 48495638 | 48603111 | 1.471240049 | 4 | amp |
| TCGA-09-1672 | 3 | 48603663 | 48614216 | 2.101559533 | 5 | amp |
| TCGA-09-1672 | 3 | 48614274 | 48669873 | 1.438257404 | 4 | amp |
| TCGA-09-1672 | 3 | 48675591 | 48789799 | 1.109131212 | 3 | amp |
| TCGA-09-1672 | 3 | 48793784 | 49006147 | 1.3760263 | 4 | amp |
| TCGA-09-1672 | 3 | 49007992 | 49065365 | 1.11579488 | 3 | amp |
| TCGA-09-1672 | 3 | 49065627 | 49680622 | 1.300210944 | 4 | amp |
| TCGA-09-1672 | 3 | 49688008 | 49836589 | 1.071515107 | 3 | amp |
| TCGA-09-1672 | 3 | 49836654 | 49849495 | 1.286423227 | 4 | amp |
| TCGA-09-1672 | 3 | 49849530 | 49882053 | 1.920906167 | 5 | amp |
| TCGA-09-1672 | 3 | 49884909 | 50103977 | 1.503610658 | 4 | amp |
| TCGA-09-1672 | 3 | 50106099 | 50395948 | 1.025271988 | 3 | amp |
| TCGA-09-1672 | 3 | 50403367 | 51981953 | 1.460261792 | 4 | amp |
| TCGA-09-1672 | 3 | 51982261 | 52456956 | 1.11878309 | 3 | amp |
| TCGA-09-1672 | 3 | 52457069 | 52543341 | 1.286022752 | 4 | amp |
| TCGA-09-1672 | 3 | 52543828 | 52588937 | 1.041619779 | 3 | amp |
| TCGA-09-1672 | 3 | 52595733 | 52696340 | 1.379712631 | 4 | amp |
| TCGA-09-1672 | 3 | 52771557 | 52822101 | 1.058837204 | 3 | amp |
| TCGA-09-1672 | 3 | 52822244 | 52889495 | 2.022813044 | 5 | amp |
| TCGA-09-1672 | 3 | 52946548 | 53765198 | 1.558008233 | 4 | amp |
| TCGA-09-1672 | 3 | 53765985 | 53821033 | 1.85336991 | 5 | amp |
| TCGA-09-1672 | 3 | 53834183 | 54420831 | 0.961734136 | 3 | amp |
| TCGA-09-1672 | 3 | 54537479 | 56598176 | 1.598244881 | 4 | amp |
| TCGA-09-1672 | 3 | 56600584 | 56661809 | 0.854619058 | 3 | amp |
| TCGA-09-1672 | 3 | 56662460 | 57154372 | 1.334608495 | 4 | amp |
| TCGA-09-1672 | 3 | 57203214 | 57835571 | 0.958405328 | 3 | amp |
| TCGA-09-1672 | 3 | 57843417 | 58095097 | 1.465595229 | 4 | amp |
| TCGA-09-1672 | 3 | 58095244 | 58256844 | 1.841305332 | 5 | amp |
| TCGA-09-1672 | 3 | 58260343 | 58279523 | 1.347566782 | 4 | amp |
| TCGA-09-1672 | 3 | 58355121 | 58413953 | 1.687267802 | 5 | amp |
| TCGA-09-1672 | 3 | 58502921 | 62503934 | 1.536071157 | 4 | amp |
| TCGA-09-1672 | 3 | 62518519 | 63466689 | 1.980095109 | 5 | amp |
| TCGA-09-1672 | 3 | 63542197 | 63968171 | 1.468079688 | 4 | amp |
| TCGA-09-1672 | 3 | 63968595 | 63985194 | 1.960908836 | 5 | amp |
| TCGA-09-1672 | 3 | 64084683 | 64644502 | 1.896888693 | 5 | amp |
| TCGA-09-1672 | 3 | 65365136 | 66457963 | 1.446355402 | 4 | amp |
| TCGA-09-1672 | 3 | 66460504 | 66512957 | 1.890597335 | 5 | amp |
| TCGA-09-1672 | 3 | 67049420 | 69047273 | 1.434536989 | 4 | amp |
| TCGA-09-1672 | 3 | 69050751 | 69127128 | 0.975520657 | 3 | amp |
| TCGA-09-1672 | 3 | 69134166 | 75680021 | 1.368454754 | 4 | amp |
| TCGA-09-1672 | 3 | 75986638 | 93619830 | 1.346708677 | 4 | amp |
| TCGA-09-1672 | 3 | 93624573 | 97634636 | 1.189548114 | 3 | amp |
| TCGA-09-1672 | 3 | 97652457 | 98217479 | 1.420648424 | 4 | amp |
| TCGA-09-1672 | 3 | 98235492 | 98600617 | 1.045558843 | 3 | amp |
| TCGA-09-1672 | 3 | 99509510 | 99998608 | 1.408355973 | 4 | amp |
| TCGA-09-1672 | 3 | 100000578 | 100092495 | 1.122410076 | 3 | amp |
| TCGA-09-1672 | 3 | 100093808 | 100378729 | 1.441886698 | 4 | amp |

| | | | | | | |
|---|---|---|---|---|---|---|
| TCGA-09-1672 | 3 | 100455339 | 101023192 | 1.388710639 | 4 | amp |
| TCGA-09-1672 | 3 | 101038371 | 101540822 | 1.152541802 | 3 | amp |
| TCGA-09-1672 | 3 | 102153918 | 108103641 | 1.344047794 | 4 | amp |
| TCGA-09-1672 | 3 | 108107808 | 108118137 | 2.152519098 | 5 | amp |
| TCGA-09-1672 | 3 | 108124182 | 108327030 | 1.438016744 | 4 | amp |
| TCGA-09-1672 | 3 | 108329978 | 108355581 | 0.959668078 | 3 | amp |
| TCGA-09-1672 | 3 | 108356308 | 108788661 | 1.344500246 | 4 | amp |
| TCGA-09-1672 | 3 | 108829552 | 111860183 | 1.459535243 | 4 | amp |
| TCGA-09-1672 | 3 | 111870141 | 112301610 | 1.013774631 | 3 | amp |
| TCGA-09-1672 | 3 | 112324256 | 112546552 | 1.540238695 | 4 | amp |
| TCGA-09-1672 | 3 | 112548138 | 112736491 | 1.110290251 | 3 | amp |
| TCGA-09-1672 | 3 | 112738381 | 113005733 | 1.387497385 | 4 | amp |
| TCGA-09-1672 | 3 | 113081945 | 113222095 | 1.044456888 | 3 | amp |
| TCGA-09-1672 | 3 | 113225342 | 113331046 | 1.372862478 | 4 | amp |
| TCGA-09-1672 | 3 | 113334924 | 113697900 | 0.990383991 | 3 | amp |
| TCGA-09-1672 | 3 | 113699448 | 120321145 | 1.415962435 | 4 | amp |
| TCGA-09-1672 | 3 | 120347181 | 120369726 | 1.922333893 | 5 | amp |
| TCGA-09-1672 | 3 | 120371408 | 120401010 | 1.430718663 | 4 | amp |
| TCGA-09-1672 | 3 | 120408642 | 121265522 | 1.121446371 | 3 | amp |
| TCGA-09-1672 | 3 | 121289551 | 121355368 | 2.317837399 | 5 | amp |
| TCGA-09-1672 | 3 | 121355987 | 121383513 | 1.557551949 | 4 | amp |
| TCGA-09-1672 | 3 | 121383765 | 121615356 | 0.968819542 | 3 | amp |
| TCGA-09-1672 | 3 | 121616185 | 122060469 | 1.403268251 | 4 | amp |
| TCGA-09-1672 | 3 | 122078708 | 122290619 | 1.049723343 | 3 | amp |
| TCGA-09-1672 | 3 | 122334510 | 122354982 | 1.834775806 | 5 | amp |
| TCGA-09-1672 | 3 | 122399703 | 122641310 | 0.96877751 | 3 | amp |
| TCGA-09-1672 | 3 | 122642411 | 122835171 | 1.48897096 | 4 | amp |
| TCGA-09-1672 | 3 | 122842868 | 122873912 | 2.102368885 | 5 | amp |
| TCGA-09-1672 | 3 | 122880084 | 123301177 | 1.511516344 | 4 | amp |
| TCGA-09-1672 | 3 | 123332893 | 123512725 | 2.221541181 | 5 | amp |
| TCGA-09-1672 | 3 | 123633615 | 123699330 | 1.417336391 | 4 | amp |
| TCGA-09-1672 | 3 | 123813627 | 124738453 | 1.790655419 | 5 | amp |
| TCGA-09-1672 | 3 | 124739624 | 124896822 | 1.466346644 | 4 | amp |
| TCGA-09-1672 | 3 | 124906026 | 126189922 | 1.09276289 | 3 | amp |
| TCGA-09-1672 | 3 | 126190764 | 127325676 | 1.323377893 | 4 | amp |
| TCGA-09-1672 | 3 | 127327141 | 127806659 | 1.152685761 | 3 | amp |
| TCGA-09-1672 | 3 | 127816121 | 129238049 | 1.41298155 | 4 | amp |
| TCGA-09-1672 | 3 | 129238342 | 129303081 | 1.09083441 | 3 | amp |
| TCGA-09-1672 | 3 | 129303166 | 130437425 | 1.463045411 | 4 | amp |
| TCGA-09-1672 | 3 | 130442224 | 130659564 | 0.994539594 | 3 | amp |
| TCGA-09-1672 | 3 | 130660428 | 132165418 | 1.346576918 | 4 | amp |
| TCGA-09-1672 | 3 | 132166119 | 132211478 | 0.938657428 | 3 | amp |
| TCGA-09-1672 | 3 | 132212985 | 132298428 | 1.339649544 | 4 | amp |
| TCGA-09-1672 | 3 | 132319227 | 133100020 | 1.023165939 | 3 | amp |
| TCGA-09-1672 | 3 | 133108980 | 133191418 | 1.569255438 | 4 | amp |
| TCGA-09-1672 | 3 | 133193754 | 133535808 | 1.110208734 | 3 | amp |
| TCGA-09-1672 | 3 | 133538383 | 134084764 | 1.338570676 | 4 | amp |
| TCGA-09-1672 | 3 | 134085046 | 134256113 | 0.925790627 | 3 | amp |
| TCGA-09-1672 | 3 | 134264424 | 136085974 | 1.403932227 | 4 | amp |
| TCGA-09-1672 | 3 | 136087886 | 137940863 | 1.084442034 | 3 | amp |

| | | | | | | |
|---|---|---|---|---|---|---|
| TCGA-09-1672 | 3 | 137942181 | 138189913 | 1.287320623 | 4 | amp |
| TCGA-09-1672 | 3 | 138191122 | 139063074 | 1.008855312 | 3 | amp |
| TCGA-09-1672 | 3 | 139065682 | 139894973 | 1.390677562 | 4 | amp |
| TCGA-09-1672 | 3 | 140122418 | 140283014 | 2.063455335 | 5 | amp |
| TCGA-09-1672 | 3 | 140284874 | 141689008 | 1.301815915 | 4 | amp |
| TCGA-09-1672 | 3 | 141692842 | 147131359 | 1.102303749 | 3 | amp |
| TCGA-09-1672 | 3 | 148458762 | 148614520 | 1.4625896 | 4 | amp |
| TCGA-09-1672 | 3 | 148703573 | 148891568 | 1.014416805 | 3 | amp |
| TCGA-09-1672 | 3 | 148893997 | 149290838 | 1.39629252 | 4 | amp |
| TCGA-09-1672 | 3 | 149374637 | 150344978 | 0.973648436 | 3 | amp |
| TCGA-09-1672 | 3 | 150377630 | 153973310 | 1.361228522 | 4 | amp |
| TCGA-09-1672 | 3 | 153993932 | 154878249 | 1.013278758 | 3 | amp |
| TCGA-09-1672 | 3 | 154884630 | 155314208 | 1.32559001 | 4 | amp |
| TCGA-09-1672 | 3 | 155481266 | 155762268 | 1.137826087 | 3 | amp |
| TCGA-09-1672 | 3 | 155838327 | 156241795 | 1.500535364 | 4 | amp |
| TCGA-09-1672 | 3 | 156249151 | 156877290 | 0.963535985 | 3 | amp |
| TCGA-09-1672 | 3 | 156978860 | 157146313 | 1.571189105 | 4 | amp |
| TCGA-09-1672 | 3 | 157154667 | 167728630 | 1.101996086 | 3 | amp |
| TCGA-09-1672 | 3 | 167742257 | 169498577 | 1.388646451 | 4 | amp |
| TCGA-09-1672 | 3 | 169548228 | 169646425 | 1.543356344 | 4 | amp |
| TCGA-09-1672 | 3 | 169654121 | 170110245 | 1.148076968 | 3 | amp |
| TCGA-09-1672 | 3 | 170140912 | 171417635 | 1.416184469 | 4 | amp |
| TCGA-09-1672 | 3 | 171426526 | 171455881 | 0.94346331 | 3 | amp |
| TCGA-09-1672 | 3 | 171830264 | 172472505 | 1.532426146 | 4 | amp |
| TCGA-09-1672 | 3 | 172473064 | 172538042 | 1.068299319 | 3 | amp |
| TCGA-09-1672 | 3 | 172607300 | 176755982 | 1.415402262 | 4 | amp |
| TCGA-09-1672 | 3 | 176756041 | 182683552 | 1.070121707 | 3 | amp |
| TCGA-09-1672 | 3 | 182733139 | 182759566 | 1.535205677 | 4 | amp |
| TCGA-09-1672 | 3 | 182763167 | 182812429 | 0.870623857 | 3 | amp |
| TCGA-09-1672 | 3 | 182841815 | 183535960 | 1.471972349 | 4 | amp |
| TCGA-09-1672 | 3 | 183547351 | 183585870 | 0.954536356 | 3 | amp |
| TCGA-09-1672 | 3 | 183639048 | 183756768 | 1.602283809 | 4 | amp |
| TCGA-09-1672 | 3 | 183770811 | 183778158 | 1.98927483 | 5 | amp |
| TCGA-09-1672 | 3 | 183801647 | 183857966 | 1.407671455 | 4 | amp |
| TCGA-09-1672 | 3 | 183858181 | 183904488 | 1.033934644 | 3 | amp |
| TCGA-09-1672 | 3 | 183904563 | 183961439 | 1.388796268 | 4 | amp |
| TCGA-09-1672 | 3 | 183961561 | 185135465 | 1.0714236 | 3 | amp |
| TCGA-09-1672 | 3 | 185146366 | 185766658 | 1.490672408 | 4 | amp |
| TCGA-09-1672 | 3 | 185769779 | 185799019 | 2.080785731 | 5 | amp |
| TCGA-09-1672 | 3 | 185823058 | 186272591 | 1.413079087 | 4 | amp |
| TCGA-09-1672 | 3 | 186299764 | 186384031 | 1.505658743 | 4 | amp |
| TCGA-09-1672 | 3 | 186386661 | 186395677 | 2.043169485 | 5 | amp |
| TCGA-09-1672 | 3 | 186435308 | 186501473 | 1.573432187 | 4 | amp |
| TCGA-09-1672 | 3 | 186502183 | 186938073 | 0.886865394 | 3 | amp |
| TCGA-09-1672 | 3 | 186938782 | 187003848 | 1.942748499 | 5 | amp |
| TCGA-09-1672 | 3 | 187009357 | 193188834 | 1.494928253 | 4 | amp |
| TCGA-09-1672 | 3 | 193201699 | 196460821 | 1.038537366 | 3 | amp |
| TCGA-09-1672 | 3 | 196509460 | 196630502 | 1.506733858 | 4 | amp |
| TCGA-09-1672 | 3 | 196650233 | 197362459 | 1.041895896 | 3 | amp |
| TCGA-09-1672 | 3 | 197401316 | 197445044 | 1.511377589 | 4 | amp |

| | | | | | | |
|---|---|---|---|---|---|---|
| TCGA-09-1672 | 3 | 197483242 | 197955154 | 1.153668436 | 3 | amp |
| TCGA-09-1672 | 4 | 1066715 | 1090649 | 1.374247663 | 4 | amp |
| TCGA-09-1672 | 4 | 1836520 | 1961512 | 1.107535921 | 3 | amp |
| TCGA-09-1672 | 4 | 2492164 | 2909580 | 0.980308905 | 3 | amp |
| TCGA-09-1672 | 4 | 2956100 | 9157341 | 1.023598062 | 3 | amp |
| TCGA-09-1672 | 4 | 9212938 | 9370636 | 2.55205841 | 5 | amp |
| TCGA-09-1672 | 4 | 9741213 | 10086170 | 1.228202755 | 4 | amp |
| TCGA-09-1672 | 4 | 10090266 | 25026537 | 1.050341014 | 3 | amp |
| TCGA-09-1672 | 4 | 25419179 | 25849500 | 1.206346727 | 4 | amp |
| TCGA-09-1672 | 4 | 26321443 | 39918857 | 0.976934142 | 3 | amp |
| TCGA-09-1672 | 4 | 40244994 | 40356566 | 1.391863417 | 4 | amp |
| TCGA-09-1672 | 4 | 40818072 | 41016427 | 1.292217705 | 4 | amp |
| TCGA-09-1672 | 4 | 41035201 | 47538104 | 0.941881035 | 3 | amp |
| TCGA-09-1672 | 4 | 47538397 | 47676523 | 1.255856956 | 4 | amp |
| TCGA-09-1672 | 4 | 47679892 | 48850494 | 0.975668549 | 3 | amp |
| TCGA-09-1672 | 4 | 49030638 | 53787084 | 0.926578407 | 3 | amp |
| TCGA-09-1672 | 4 | 54342913 | 54853195 | 1.358802508 | 4 | amp |
| TCGA-09-1672 | 4 | 55124899 | 68459085 | 0.960949681 | 3 | amp |
| TCGA-09-1672 | 4 | 68606139 | 69536394 | 0.914458524 | 3 | amp |
| TCGA-09-1672 | 4 | 69681736 | 70361638 | 1.223377509 | 4 | amp |
| TCGA-09-1672 | 4 | 70709848 | 79207745 | 0.987741042 | 3 | amp |
| TCGA-09-1672 | 4 | 79229171 | 79455805 | 1.206487962 | 4 | amp |
| TCGA-09-1672 | 4 | 79458131 | 89649866 | 0.96212542 | 3 | amp |
| TCGA-09-1672 | 4 | 89652468 | 89688784 | 1.769901788 | 5 | amp |
| TCGA-09-1672 | 4 | 89689111 | 100229068 | 1.035764751 | 3 | amp |
| TCGA-09-1672 | 4 | 100231870 | 100803247 | 1.202741621 | 4 | amp |
| TCGA-09-1672 | 4 | 101111034 | 104080086 | 0.97911885 | 3 | amp |
| TCGA-09-1672 | 4 | 104117291 | 110585557 | 0.986095559 | 3 | amp |
| TCGA-09-1672 | 4 | 110639786 | 110786538 | 0.978870791 | 3 | amp |
| TCGA-09-1672 | 4 | 110788769 | 111474660 | 1.20149479 | 4 | amp |
| TCGA-09-1672 | 4 | 111480751 | 113378525 | 0.932465057 | 3 | amp |
| TCGA-09-1672 | 4 | 113567908 | 114294642 | 1.214670196 | 4 | amp |
| TCGA-09-1672 | 4 | 114294702 | 122617799 | 0.969297943 | 3 | amp |
| TCGA-09-1672 | 4 | 123120499 | 128996187 | 0.93807102 | 3 | amp |
| TCGA-09-1672 | 4 | 129752849 | 129793443 | 1.400876588 | 4 | amp |
| TCGA-09-1672 | 4 | 129805602 | 139980808 | 0.958627965 | 3 | amp |
| TCGA-09-1672 | 4 | 140297512 | 141264887 | 1.09167538 | 3 | amp |
| TCGA-09-1672 | 4 | 141578195 | 153574128 | 0.958810814 | 3 | amp |
| TCGA-09-1672 | 4 | 153895740 | 170347400 | 0.996699738 | 3 | amp |
| TCGA-09-1672 | 4 | 170618312 | 187631028 | 0.9406029 | 3 | amp |
| TCGA-09-1672 | 4 | 189060646 | 189068550 | 2.084714117 | 5 | amp |
| TCGA-09-1672 | 5 | 3600668 | 10664727 | 0.938438994 | 3 | amp |
| TCGA-09-1672 | 5 | 10683571 | 13781122 | 1.347837116 | 4 | amp |
| TCGA-09-1672 | 5 | 13786223 | 33546355 | 1.084315293 | 3 | amp |
| TCGA-09-1672 | 5 | 33549279 | 33751700 | 1.337633865 | 4 | amp |
| TCGA-09-1672 | 5 | 33881164 | 34998937 | 0.928575867 | 3 | amp |
| TCGA-09-1672 | 5 | 35003826 | 35089747 | 1.295986495 | 4 | amp |
| TCGA-09-1672 | 5 | 35628552 | 37000693 | 0.990335826 | 3 | amp |
| TCGA-09-1672 | 5 | 37049187 | 37701261 | 0.934328396 | 3 | amp |
| TCGA-09-1672 | 5 | 37702999 | 38482368 | 1.277338127 | 4 | amp |

| | | | | | | |
|---|---|---|---|---|---|---|
| TCGA-09-1672 | 5 | 38482649 | 39394436 | 0.984110387 | 3 | amp |
| TCGA-09-1672 | 5 | 40832550 | 41907950 | 1.155015829 | 3 | amp |
| TCGA-09-1672 | 5 | 42565951 | 43454095 | 0.977470613 | 3 | amp |
| TCGA-09-1672 | 5 | 43486994 | 43543221 | 0.434795897 | 1 | loss |
| TCGA-09-1672 | 5 | 50090668 | 54573141 | 0.975606791 | 3 | amp |
| TCGA-09-1672 | 5 | 55147374 | 56189552 | 1.071279159 | 3 | amp |
| TCGA-09-1672 | 5 | 56246376 | 61684917 | 0.961164013 | 3 | amp |
| TCGA-09-1672 | 5 | 61856903 | 65118795 | 0.941483232 | 3 | amp |
| TCGA-09-1672 | 5 | 66055493 | 68609902 | 0.939362697 | 3 | amp |
| TCGA-09-1672 | 5 | 68720385 | 70422601 | 1.039095108 | 3 | amp |
| TCGA-09-1672 | 5 | 70922463 | 71411635 | 0.977511786 | 3 | amp |
| TCGA-09-1672 | 5 | 71479550 | 71528387 | 1.404425917 | 4 | amp |
| TCGA-09-1672 | 5 | 71529924 | 75885556 | 0.946804526 | 3 | amp |
| TCGA-09-1672 | 5 | 75886171 | 75970537 | 1.342964705 | 4 | amp |
| TCGA-09-1672 | 5 | 75972988 | 79336121 | 0.968815669 | 3 | amp |
| TCGA-09-1672 | 5 | 79351521 | 79498908 | 1.238495785 | 4 | amp |
| TCGA-09-1672 | 5 | 79616020 | 79744234 | 1.009879736 | 3 | amp |
| TCGA-09-1672 | 5 | 80149921 | 82808248 | 0.948670814 | 3 | amp |
| TCGA-09-1672 | 5 | 82815147 | 83259221 | 1.315101288 | 4 | amp |
| TCGA-09-1672 | 5 | 83356090 | 102465410 | 0.951949552 | 3 | amp |
| TCGA-09-1672 | 5 | 102894574 | 112720966 | 0.977176568 | 3 | amp |
| TCGA-09-1672 | 5 | 115320216 | 118176816 | 1.109861732 | 3 | amp |
| TCGA-09-1672 | 5 | 118865571 | 127647670 | 0.995706207 | 3 | amp |
| TCGA-09-1672 | 5 | 127648256 | 127729064 | 1.226323951 | 4 | amp |
| TCGA-09-1672 | 5 | 127730740 | 131298419 | 0.988696484 | 3 | amp |
| TCGA-09-1672 | 5 | 131302046 | 131539869 | 1.313790968 | 4 | amp |
| TCGA-09-1672 | 5 | 131543368 | 132018288 | 0.972402667 | 3 | amp |
| TCGA-09-1672 | 5 | 132087652 | 135290559 | 1.027457919 | 3 | amp |
| TCGA-09-1672 | 5 | 135369440 | 137045551 | 1.300079795 | 4 | amp |
| TCGA-09-1672 | 5 | 137056093 | 137488489 | 0.945796807 | 3 | amp |
| TCGA-09-1672 | 5 | 137492836 | 137549040 | 1.510462081 | 4 | amp |
| TCGA-09-1672 | 5 | 137588641 | 137708566 | 0.901595268 | 3 | amp |
| TCGA-09-1672 | 5 | 137710778 | 137763835 | 1.385053084 | 4 | amp |
| TCGA-09-1672 | 5 | 137765464 | 137854588 | 0.994528159 | 3 | amp |
| TCGA-09-1672 | 5 | 138208691 | 139940113 | 1.026820203 | 3 | amp |
| TCGA-09-1672 | 5 | 140059360 | 140073272 | 0.995669932 | 3 | amp |
| TCGA-09-1672 | 5 | 140073459 | 140085382 | 1.205802521 | 4 | amp |
| TCGA-09-1672 | 5 | 140165797 | 140726105 | 0.504402744 | 1 | loss |
| TCGA-09-1672 | 5 | 140903662 | 140963236 | 1.172899583 | 3 | amp |
| TCGA-09-1672 | 5 | 141511326 | 141524247 | 1.073419248 | 3 | amp |
| TCGA-09-1672 | 5 | 141693766 | 145483934 | 1.271276052 | 4 | amp |
| TCGA-09-1672 | 5 | 145493651 | 145665611 | 0.979170284 | 3 | amp |
| TCGA-09-1672 | 5 | 145872421 | 145888817 | 0.983183268 | 3 | amp |
| TCGA-09-1672 | 5 | 145889954 | 148709430 | 1.322015758 | 4 | amp |
| TCGA-09-1672 | 5 | 148712226 | 148980852 | 1.129594319 | 3 | amp |
| TCGA-09-1672 | 5 | 148989064 | 149932954 | 1.399855699 | 4 | amp |
| TCGA-09-1672 | 5 | 150027801 | 150136073 | 0.987638253 | 3 | amp |
| TCGA-09-1672 | 5 | 150138337 | 151054263 | 1.39256064 | 4 | amp |
| TCGA-09-1672 | 5 | 151055660 | 151180435 | 1.114823185 | 3 | amp |
| TCGA-09-1672 | 5 | 151183428 | 153372722 | 1.61869012 | 4 | amp |

| | | | | | | |
|---|---|---|---|---|---|---|
| TCGA-09-1672 | 5 | 153374445 | 153674486 | 1.082461215 | 3 | amp |
| TCGA-09-1672 | 5 | 153677449 | 153796572 | 1.628027828 | 4 | amp |
| TCGA-09-1672 | 5 | 153826301 | 154191235 | 0.940100667 | 3 | amp |
| TCGA-09-1672 | 5 | 154193401 | 156991475 | 1.305747469 | 4 | amp |
| TCGA-09-1672 | 5 | 156997885 | 159626198 | 0.954362041 | 3 | amp |
| TCGA-09-1672 | 5 | 159640724 | 159797701 | 1.343479734 | 4 | amp |
| TCGA-09-1672 | 5 | 159820894 | 159841481 | 0.901782381 | 3 | amp |
| TCGA-09-1672 | 5 | 159842096 | 160039959 | 1.325858791 | 4 | amp |
| TCGA-09-1672 | 5 | 160042787 | 160061666 | 1.800443895 | 5 | amp |
| TCGA-09-1672 | 5 | 160063102 | 161522594 | 1.285791264 | 4 | amp |
| TCGA-09-1672 | 5 | 161524633 | 162866574 | 1.08923829 | 3 | amp |
| TCGA-09-1672 | 5 | 162943470 | 167919877 | 1.602281193 | 4 | amp |
| TCGA-09-1672 | 5 | 167920892 | 168100378 | 1.134836149 | 3 | amp |
| TCGA-09-1672 | 5 | 168110885 | 169023754 | 1.635648791 | 4 | amp |
| TCGA-09-1672 | 5 | 169025471 | 169097658 | 0.962673907 | 3 | amp |
| TCGA-09-1672 | 5 | 169098069 | 170239290 | 1.456147414 | 4 | amp |
| TCGA-09-1672 | 5 | 171295569 | 171849519 | 1.262594517 | 4 | amp |
| TCGA-09-1672 | 5 | 172189471 | 175514306 | 1.008584599 | 3 | amp |
| TCGA-09-1672 | 5 | 175516434 | 175541065 | 1.478962388 | 4 | amp |
| TCGA-09-1672 | 5 | 175716633 | 175837340 | 1.026788884 | 3 | amp |
| TCGA-09-1672 | 5 | 175906162 | 176017352 | 1.219700561 | 4 | amp |
| TCGA-09-1672 | 5 | 176017365 | 176491616 | 1.019356569 | 3 | amp |
| TCGA-09-1672 | 5 | 176940616 | 177483199 | 1.032726599 | 3 | amp |
| TCGA-09-1672 | 6 | 105907 | 348308 | 0.996025885 | 3 | amp |
| TCGA-09-1672 | 6 | 4052109 | 4117733 | 0.46026684 | 1 | loss |
| TCGA-09-1672 | 6 | 9932970 | 10430547 | 0.491394003 | 1 | loss |
| TCGA-09-1672 | 6 | 12161586 | 13288698 | 0.988924918 | 3 | amp |
| TCGA-09-1672 | 6 | 13307754 | 13652961 | 0.500358095 | 1 | loss |
| TCGA-09-1672 | 6 | 17421726 | 17543392 | 0.990613034 | 3 | amp |
| TCGA-09-1672 | 6 | 17794432 | 17852289 | 0.922614923 | 3 | amp |
| TCGA-09-1672 | 6 | 18399727 | 20490709 | 0.949475484 | 3 | amp |
| TCGA-09-1672 | 6 | 24520550 | 24601363 | 0.958285411 | 3 | amp |
| TCGA-09-1672 | 6 | 24843075 | 24852884 | 1.504699733 | 4 | amp |
| TCGA-09-1672 | 6 | 24861172 | 24977261 | 0.922201715 | 3 | amp |
| TCGA-09-1672 | 6 | 25140443 | 25285148 | 0.411481825 | 1 | loss |
| TCGA-09-1672 | 6 | 25420306 | 26368512 | 0.926238228 | 3 | amp |
| TCGA-09-1672 | 6 | 26368786 | 26468836 | 1.307108625 | 4 | amp |
| TCGA-09-1672 | 6 | 28493743 | 28554524 | 0.988260172 | 3 | amp |
| TCGA-09-1672 | 6 | 29322949 | 29430501 | 1.039909515 | 3 | amp |
| TCGA-09-1672 | 6 | 29555710 | 29575056 | 1.113224398 | 3 | amp |
| TCGA-09-1672 | 6 | 29643153 | 30080613 | 0.529004377 | 1 | loss |
| TCGA-09-1672 | 6 | 30550753 | 30558514 | 1.047729933 | 3 | amp |
| TCGA-09-1672 | 6 | 30587671 | 30596216 | 1.029087729 | 3 | amp |
| TCGA-09-1672 | 6 | 31631582 | 31678415 | 1.004200799 | 3 | amp |
| TCGA-09-1672 | 6 | 31715126 | 31731948 | 0.951477625 | 3 | amp |
| TCGA-09-1672 | 6 | 31857144 | 31864792 | 0.36276235 | 1 | loss |
| TCGA-09-1672 | 6 | 32187833 | 32191657 | 1.44415486 | 4 | amp |
| TCGA-09-1672 | 6 | 32372709 | 32427910 | 1.039593628 | 3 | amp |
| TCGA-09-1672 | 6 | 33131374 | 33142028 | 1.040983613 | 3 | amp |
| TCGA-09-1672 | 6 | 33142270 | 33148131 | 1.370023606 | 4 | amp |

| | | | | | | |
|---|---|---|---|---|---|---|
| TCGA-09-1672 | 6 | 33166012 | 33179822 | 0.477039869 | 1 | loss |
| TCGA-09-1672 | 6 | 33391194 | 33415724 | 0.49833655 | 1 | loss |
| TCGA-09-1672 | 6 | 33419459 | 33740599 | 0.879728125 | 3 | amp |
| TCGA-09-1672 | 6 | 34789390 | 34827404 | 1.019003398 | 3 | amp |
| TCGA-09-1672 | 6 | 35195267 | 35214080 | 0.970680637 | 3 | amp |
| TCGA-09-1672 | 6 | 35543605 | 35565222 | 1.079300183 | 3 | amp |
| TCGA-09-1672 | 6 | 35911656 | 35949998 | 0.996127028 | 3 | amp |
| TCGA-09-1672 | 6 | 36269551 | 36334600 | 1.017258701 | 3 | amp |
| TCGA-09-1672 | 6 | 36446888 | 36569007 | 0.912393553 | 3 | amp |
| TCGA-09-1672 | 6 | 36720751 | 36790913 | 0.981485298 | 3 | amp |
| TCGA-09-1672 | 6 | 36976575 | 37447974 | 0.905605811 | 3 | amp |
| TCGA-09-1672 | 6 | 38759270 | 38906788 | 0.913869911 | 3 | amp |
| TCGA-09-1672 | 6 | 38957747 | 39398942 | 0.949335606 | 3 | amp |
| TCGA-09-1672 | 6 | 39824042 | 39866742 | 0.993709115 | 3 | amp |
| TCGA-09-1672 | 6 | 39867799 | 39884013 | 1.49304181 | 4 | amp |
| TCGA-09-1672 | 6 | 41117318 | 41254428 | 0.912271463 | 3 | amp |
| TCGA-09-1672 | 6 | 42796170 | 42830358 | 1.085391009 | 3 | amp |
| TCGA-09-1672 | 6 | 44106145 | 44149082 | 0.883920189 | 3 | amp |
| TCGA-09-1672 | 6 | 44201119 | 44227048 | 0.382932685 | 1 | loss |
| TCGA-09-1672 | 6 | 45459644 | 45922986 | 1.090802378 | 3 | amp |
| TCGA-09-1672 | 6 | 46609847 | 46677169 | 0.460348698 | 1 | loss |
| TCGA-09-1672 | 6 | 46825842 | 47254365 | 0.992246957 | 3 | amp |
| TCGA-09-1672 | 6 | 47591882 | 49408112 | 1.025617534 | 3 | amp |
| TCGA-09-1672 | 6 | 51483828 | 52129604 | 0.909594691 | 3 | amp |
| TCGA-09-1672 | 6 | 52344391 | 52661165 | 0.990398037 | 3 | amp |
| TCGA-09-1672 | 6 | 52761544 | 52906043 | 0.958282619 | 3 | amp |
| TCGA-09-1672 | 6 | 52992923 | 53372464 | 0.904053595 | 3 | amp |
| TCGA-09-1672 | 6 | 56469350 | 56510847 | 0.884271807 | 3 | amp |
| TCGA-09-1672 | 6 | 72002978 | 72011780 | 0.333313825 | 1 | loss |
| TCGA-09-1672 | 6 | 75827029 | 75885069 | 0.898693727 | 3 | amp |
| TCGA-09-1672 | 6 | 80715509 | 80838986 | 0.45177612 | 1 | loss |
| TCGA-09-1672 | 6 | 86277184 | 87726201 | 0.418716016 | 1 | loss |
| TCGA-09-1672 | 6 | 89888455 | 89913238 | 0.977877313 | 3 | amp |
| TCGA-09-1672 | 6 | 90096981 | 90461345 | 0.924552679 | 3 | amp |
| TCGA-09-1672 | 6 | 90494727 | 90718576 | 0.928613449 | 3 | amp |
| TCGA-09-1672 | 6 | 107008619 | 107076939 | 0.935557586 | 3 | amp |
| TCGA-09-1672 | 6 | 109786965 | 109816651 | 0.411757358 | 1 | loss |
| TCGA-09-1672 | 6 | 111737460 | 112382525 | 1.04137583 | 3 | amp |
| TCGA-09-1672 | 6 | 112421811 | 112443430 | 1.094066355 | 3 | amp |
| TCGA-09-1672 | 6 | 112471650 | 112537679 | 0.904192902 | 3 | amp |
| TCGA-09-1672 | 6 | 117859770 | 117890936 | 0.418657881 | 1 | loss |
| TCGA-09-1672 | 6 | 118320306 | 118790470 | 1.074331128 | 3 | amp |
| TCGA-09-1672 | 6 | 119324018 | 119346819 | 0.389511543 | 1 | loss |
| TCGA-09-1672 | 6 | 121481142 | 121615820 | 0.475971078 | 1 | loss |
| TCGA-09-1672 | 6 | 125613960 | 126080965 | 0.33195945 | 1 | loss |
| TCGA-09-1672 | 6 | 126206276 | 126248916 | 0.972099585 | 3 | amp |
| TCGA-09-1672 | 6 | 128040750 | 128316683 | 0.942992293 | 3 | amp |
| TCGA-09-1672 | 6 | 129748857 | 129937462 | 0.945763618 | 3 | amp |
| TCGA-09-1672 | 6 | 130761495 | 131277627 | 0.924649714 | 3 | amp |
| TCGA-09-1672 | 6 | 131958503 | 132030270 | 0.942313844 | 3 | amp |

| | | | | | | |
|---|---|---|---|---|---|---|
| TCGA-09-1672 | 6 | 132859411 | 133079035 | 0.918292879 | 3 | amp |
| TCGA-09-1672 | 6 | 134491918 | 134495967 | 0.435779718 | 1 | loss |
| TCGA-09-1672 | 6 | 136888710 | 136958593 | 1.007519604 | 3 | amp |
| TCGA-09-1672 | 6 | 137322673 | 137479671 | 1.050413101 | 3 | amp |
| TCGA-09-1672 | 6 | 138528159 | 138599790 | 0.941038408 | 3 | amp |
| TCGA-09-1672 | 6 | 139135623 | 139183869 | 0.971263474 | 3 | amp |
| TCGA-09-1672 | 6 | 144745107 | 144837563 | 0.480707929 | 1 | loss |
| TCGA-09-1672 | 6 | 151243299 | 151358296 | 0.974285802 | 3 | amp |
| TCGA-09-1672 | 6 | 151865658 | 152563594 | 0.890670174 | 3 | amp |
| TCGA-09-1672 | 6 | 152644587 | 153075481 | 0.919410163 | 3 | amp |
| TCGA-09-1672 | 6 | 153292243 | 153332922 | 0.47757823 | 1 | loss |
| TCGA-09-1672 | 6 | 153344470 | 154763442 | 0.906273665 | 3 | amp |
| TCGA-09-1672 | 6 | 155458258 | 155504682 | 0.972634427 | 3 | amp |
| TCGA-09-1672 | 6 | 155717888 | 158053949 | 0.905012424 | 3 | amp |
| TCGA-09-1672 | 6 | 158499104 | 158515100 | 0.971508715 | 3 | amp |
| TCGA-09-1672 | 6 | 159688776 | 160445789 | 0.445456084 | 1 | loss |
| TCGA-09-1672 | 6 | 160448131 | 161012174 | 0.948774231 | 3 | amp |
| TCGA-09-1672 | 6 | 161014919 | 161071569 | 1.432226979 | 4 | amp |
| TCGA-09-1672 | 6 | 161085186 | 161143639 | 0.911374223 | 3 | amp |
| TCGA-09-1672 | 6 | 168265494 | 168281238 | 1.031521148 | 3 | amp |
| TCGA-09-1672 | 6 | 170070606 | 170162635 | 0.378273865 | 1 | loss |
| TCGA-09-1672 | 7 | 2740027 | 5952589 | 1.067853363 | 3 | amp |
| TCGA-09-1672 | 7 | 5958454 | 5997718 | 1.668680448 | 4 | amp |
| TCGA-09-1672 | 7 | 5998606 | 6805439 | 1.070370673 | 3 | amp |
| TCGA-09-1672 | 7 | 6806327 | 6845711 | 1.68685529 | 5 | amp |
| TCGA-09-1672 | 7 | 6851578 | 7646734 | 1.093723208 | 3 | amp |
| TCGA-09-1672 | 7 | 7676611 | 12254664 | 1.324082592 | 4 | amp |
| TCGA-09-1672 | 7 | 12258055 | 17931273 | 1.08744256 | 3 | amp |
| TCGA-09-1672 | 7 | 17932945 | 20795268 | 1.358568559 | 4 | amp |
| TCGA-09-1672 | 7 | 21583105 | 22349673 | 1.486703126 | 4 | amp |
| TCGA-09-1672 | 7 | 22532146 | 24708318 | 1.080863461 | 3 | amp |
| TCGA-09-1672 | 7 | 24718730 | 24839909 | 1.51865091 | 4 | amp |
| TCGA-09-1672 | 7 | 24843844 | 24905892 | 1.91406484 | 5 | amp |
| TCGA-09-1672 | 7 | 24910347 | 25989632 | 1.407867641 | 4 | amp |
| TCGA-09-1672 | 7 | 26217502 | 26237127 | 0.507573237 | 1 | loss |
| TCGA-09-1672 | 7 | 26237236 | 28031623 | 1.049333521 | 3 | amp |
| TCGA-09-1672 | 7 | 28452474 | 29552385 | 1.432218728 | 4 | amp |
| TCGA-09-1672 | 7 | 29605900 | 30673533 | 1.114477631 | 3 | amp |
| TCGA-09-1672 | 7 | 30692995 | 30825666 | 1.588389916 | 4 | amp |
| TCGA-09-1672 | 7 | 30830715 | 30962294 | 1.91875484 | 5 | amp |
| TCGA-09-1672 | 7 | 30963004 | 33054452 | 1.503022957 | 4 | amp |
| TCGA-09-1672 | 7 | 33055261 | 33397656 | 1.127717932 | 3 | amp |
| TCGA-09-1672 | 7 | 33407366 | 35009154 | 1.493469566 | 4 | amp |
| TCGA-09-1672 | 7 | 35013111 | 36278709 | 1.078085786 | 3 | amp |
| TCGA-09-1672 | 7 | 36320688 | 36429689 | 1.569948157 | 4 | amp |
| TCGA-09-1672 | 7 | 36435801 | 36478839 | 0.901211936 | 3 | amp |
| TCGA-09-1672 | 7 | 36483327 | 36616280 | 1.52844847 | 4 | amp |
| TCGA-09-1672 | 7 | 36633900 | 37298990 | 1.795608677 | 5 | amp |
| TCGA-09-1672 | 7 | 37311371 | 38254727 | 1.283096068 | 4 | amp |
| TCGA-09-1672 | 7 | 38256592 | 38393650 | 1.839919528 | 5 | amp |

| | | | | | | |
|---|---|---|---|---|---|---|
| TCGA-09-1672 | 7 | 38393702 | 42188125 | 1.466883573 | 4 | amp |
| TCGA-09-1672 | 7 | 42971646 | 44114182 | 1.399775147 | 4 | amp |
| TCGA-09-1672 | 7 | 44116047 | 44524904 | 1.055113677 | 3 | amp |
| TCGA-09-1672 | 7 | 44553026 | 44685141 | 1.909961065 | 5 | amp |
| TCGA-09-1672 | 7 | 44687246 | 44747657 | 1.47050154 | 4 | amp |
| TCGA-09-1672 | 7 | 44795813 | 47341886 | 1.161849146 | 3 | amp |
| TCGA-09-1672 | 7 | 47342474 | 47944274 | 1.391146629 | 4 | amp |
| TCGA-09-1672 | 7 | 47944717 | 48321054 | 1.009488829 | 3 | amp |
| TCGA-09-1672 | 7 | 48327555 | 48556518 | 1.404272748 | 4 | amp |
| TCGA-09-1672 | 7 | 48559583 | 55005773 | 1.127387322 | 3 | amp |
| TCGA-09-1672 | 7 | 55209934 | 55273370 | 1.403377074 | 4 | amp |
| TCGA-09-1672 | 7 | 55540522 | 56088955 | 1.366014461 | 4 | amp |
| TCGA-09-1672 | 7 | 56120086 | 57142378 | 1.020689544 | 3 | amp |
| TCGA-09-1672 | 7 | 57187542 | 62917075 | 1.559456618 | 4 | amp |
| TCGA-09-1672 | 7 | 62956188 | 65413772 | 1.10668422 | 3 | amp |
| TCGA-09-1672 | 7 | 65419023 | 66751436 | 1.433181179 | 4 | amp |
| TCGA-09-1672 | 7 | 66751516 | 70800747 | 1.140092266 | 3 | amp |
| TCGA-09-1672 | 7 | 70853183 | 72341127 | 1.488514867 | 4 | amp |
| TCGA-09-1672 | 7 | 72361109 | 72483834 | 1.0691674 | 3 | amp |
| TCGA-09-1672 | 7 | 72491523 | 72597366 | 1.483216685 | 4 | amp |
| TCGA-09-1672 | 7 | 72598669 | 73457525 | 1.066315466 | 3 | amp |
| TCGA-09-1672 | 7 | 73458169 | 74251511 | 1.306652551 | 4 | amp |
| TCGA-09-1672 | 7 | 74298949 | 74312637 | 0.943277461 | 3 | amp |
| TCGA-09-1672 | 7 | 74313739 | 74982648 | 1.426372332 | 4 | amp |
| TCGA-09-1672 | 7 | 74982773 | 75190728 | 0.976464472 | 3 | amp |
| TCGA-09-1672 | 7 | 75191365 | 75659849 | 1.475269158 | 4 | amp |
| TCGA-09-1672 | 7 | 75684081 | 76033809 | 1.094572765 | 3 | amp |
| TCGA-09-1672 | 7 | 76054393 | 76069979 | 2.548292131 | 5 | amp |
| TCGA-09-1672 | 7 | 76071100 | 77003489 | 1.39678635 | 4 | amp |
| TCGA-09-1672 | 7 | 77004336 | 77583268 | 1.181973283 | 3 | amp |
| TCGA-09-1672 | 7 | 77584124 | 86527004 | 1.333300616 | 4 | amp |
| TCGA-09-1672 | 7 | 86536976 | 87011503 | 1.179195905 | 3 | amp |
| TCGA-09-1672 | 7 | 87011643 | 87215031 | 1.38613384 | 4 | amp |
| TCGA-09-1672 | 7 | 87225045 | 92985417 | 1.09665983 | 3 | amp |
| TCGA-09-1672 | 7 | 92987568 | 93116327 | 1.497954814 | 4 | amp |
| TCGA-09-1672 | 7 | 93516109 | 94048896 | 1.077298665 | 3 | amp |
| TCGA-09-1672 | 7 | 94049436 | 94146979 | 1.428518603 | 4 | amp |
| TCGA-09-1672 | 7 | 94147454 | 94540842 | 0.9998352 | 3 | amp |
| TCGA-09-1672 | 7 | 94740516 | 94935697 | 1.432776645 | 4 | amp |
| TCGA-09-1672 | 7 | 94937302 | 95439820 | 1.003032994 | 3 | amp |
| TCGA-09-1672 | 7 | 95442458 | 95614327 | 1.48415718 | 4 | amp |
| TCGA-09-1672 | 7 | 95616327 | 95668780 | 2.10964154 | 5 | amp |
| TCGA-09-1672 | 7 | 95705318 | 95800869 | 1.367141256 | 4 | amp |
| TCGA-09-1672 | 7 | 95813547 | 99045939 | 1.134461397 | 3 | amp |
| TCGA-09-1672 | 7 | 99047886 | 99459468 | 1.384766484 | 4 | amp |
| TCGA-09-1672 | 7 | 99461119 | 99527272 | 0.982646824 | 3 | amp |
| TCGA-09-1672 | 7 | 99564557 | 99673268 | 1.586784298 | 4 | amp |
| TCGA-09-1672 | 7 | 99674896 | 99793028 | 1.074371757 | 3 | amp |
| TCGA-09-1672 | 7 | 99794720 | 99918719 | 1.420491258 | 4 | amp |
| TCGA-09-1672 | 7 | 99926550 | 100356015 | 1.048564231 | 3 | amp |

| | | | | | | |
|---|---|---|---|---|---|---|
| TCGA-09-1672 | 7 | 100356153 | 100411651 | 2.082003491 | 5 | amp |
| TCGA-09-1672 | 7 | 100414746 | 100609910 | 0.969729723 | 3 | amp |
| TCGA-09-1672 | 7 | 100610017 | 100779098 | 2.030165624 | 5 | amp |
| TCGA-09-1672 | 7 | 100780263 | 100806780 | 1.355651684 | 4 | amp |
| TCGA-09-1672 | 7 | 100807557 | 100962334 | 1.000614771 | 3 | amp |
| TCGA-09-1672 | 7 | 101063198 | 102582833 | 1.313312175 | 4 | amp |
| TCGA-09-1672 | 7 | 102584615 | 102881114 | 1.028573594 | 3 | amp |
| TCGA-09-1672 | 7 | 102957248 | 103018265 | 1.200963168 | 3 | amp |
| TCGA-09-1672 | 7 | 103018886 | 105673170 | 1.415481532 | 4 | amp |
| TCGA-09-1672 | 7 | 105732197 | 107240955 | 1.044895305 | 3 | amp |
| TCGA-09-1672 | 7 | 107253736 | 107355939 | 1.43658521 | 4 | amp |
| TCGA-09-1672 | 7 | 107389288 | 107564874 | 1.031749165 | 3 | amp |
| TCGA-09-1672 | 7 | 107566595 | 107601111 | 1.623462573 | 4 | amp |
| TCGA-09-1672 | 7 | 107601592 | 107616347 | 2.172523112 | 5 | amp |
| TCGA-09-1672 | 7 | 107618431 | 112090957 | 1.377856593 | 4 | amp |
| TCGA-09-1672 | 7 | 112095809 | 112724819 | 0.990070971 | 3 | amp |
| TCGA-09-1672 | 7 | 113517718 | 116937977 | 1.309265155 | 4 | amp |
| TCGA-09-1672 | 7 | 116955049 | 121636647 | 1.106933678 | 3 | amp |
| TCGA-09-1672 | 7 | 121637876 | 121733216 | 1.428758646 | 4 | amp |
| TCGA-09-1672 | 7 | 121738446 | 122321722 | 1.091168236 | 3 | amp |
| TCGA-09-1672 | 7 | 122337806 | 123302829 | 1.331206333 | 4 | amp |
| TCGA-09-1672 | 7 | 123303060 | 127234097 | 1.117965186 | 3 | amp |
| TCGA-09-1672 | 7 | 127235348 | 127958204 | 1.459052216 | 4 | amp |
| TCGA-09-1672 | 7 | 127961277 | 128588929 | 1.132626891 | 3 | amp |
| TCGA-09-1672 | 7 | 128597248 | 130288768 | 1.35480702 | 4 | amp |
| TCGA-09-1672 | 7 | 130289551 | 130364218 | 1.001511269 | 3 | amp |
| TCGA-09-1672 | 7 | 130365759 | 131172546 | 1.374231193 | 4 | amp |
| TCGA-09-1672 | 7 | 131189048 | 131833413 | 1.973805846 | 5 | amp |
| TCGA-09-1672 | 7 | 131844161 | 134136541 | 1.391704679 | 4 | amp |
| TCGA-09-1672 | 7 | 134212636 | 134346919 | 1.94209629 | 5 | amp |
| TCGA-09-1672 | 7 | 134363573 | 134650156 | 1.453354186 | 4 | amp |
| TCGA-09-1672 | 7 | 134653003 | 134939981 | 1.134059892 | 3 | amp |
| TCGA-09-1672 | 7 | 134943146 | 137776691 | 1.441748092 | 4 | amp |
| TCGA-09-1672 | 7 | 137782578 | 138235986 | 1.05903883 | 3 | amp |
| TCGA-09-1672 | 7 | 138239396 | 139746865 | 1.399254424 | 4 | amp |
| TCGA-09-1672 | 7 | 139754422 | 140171866 | 1.063595266 | 3 | amp |
| TCGA-09-1672 | 7 | 140218432 | 140268647 | 1.461907922 | 4 | amp |
| TCGA-09-1672 | 7 | 140269401 | 140302234 | 2.111534334 | 5 | amp |
| TCGA-09-1672 | 7 | 140373116 | 140389580 | 1.361178266 | 4 | amp |
| TCGA-09-1672 | 7 | 140390496 | 140500341 | 0.902295911 | 3 | amp |
| TCGA-09-1672 | 7 | 140501165 | 141720888 | 1.359572263 | 4 | amp |
| TCGA-09-1672 | 7 | 141721341 | 142479988 | 2.166432637 | 5 | amp |
| TCGA-09-1672 | 7 | 142494208 | 142572933 | 1.470034613 | 4 | amp |
| TCGA-09-1672 | 7 | 142573176 | 142829316 | 2.063780627 | 5 | amp |
| TCGA-09-1672 | 7 | 142832249 | 142962431 | 1.519493629 | 4 | amp |
| TCGA-09-1672 | 7 | 142964617 | 142997401 | 0.981499696 | 3 | amp |
| TCGA-09-1672 | 7 | 142997443 | 143018994 | 2.046973838 | 5 | amp |
| TCGA-09-1672 | 7 | 143020350 | 143056138 | 1.548170725 | 4 | amp |
| TCGA-09-1672 | 7 | 143056405 | 143321539 | 0.977089266 | 3 | amp |
| TCGA-09-1672 | 7 | 143339645 | 143827151 | 1.431184406 | 4 | amp |

| | | | | | | |
|---|---|---|---|---|---|---|
| TCGA-09-1672 | 7 | 143880563 | 144463068 | 1.106077542 | 3 | amp |
| TCGA-09-1672 | 7 | 144702927 | 148427404 | 1.453400547 | 4 | amp |
| TCGA-09-1672 | 7 | 148451034 | 158935247 | 1.03060135 | 3 | amp |
| TCGA-09-1672 | 8 | 116074 | 2800179 | 1.038437606 | 3 | amp |
| TCGA-09-1672 | 8 | 2806804 | 6728395 | 1.321925016 | 4 | amp |
| TCGA-09-1672 | 8 | 6735258 | 7191185 | 1.7637205 | 5 | amp |
| TCGA-09-1672 | 8 | 7191305 | 7274380 | 1.325196709 | 4 | amp |
| TCGA-09-1672 | 8 | 7286458 | 7328078 | 1.879393581 | 5 | amp |
| TCGA-09-1672 | 8 | 7332501 | 7686521 | 1.459431049 | 4 | amp |
| TCGA-09-1672 | 8 | 7693975 | 7754183 | 1.866437704 | 5 | amp |
| TCGA-09-1672 | 8 | 7791897 | 10396324 | 1.354900381 | 4 | amp |
| TCGA-09-1672 | 8 | 10464396 | 17137977 | 1.150656803 | 3 | amp |
| TCGA-09-1672 | 8 | 17141933 | 17824029 | 1.397722702 | 4 | amp |
| TCGA-09-1672 | 8 | 17824463 | 17942367 | 1.104493987 | 3 | amp |
| TCGA-09-1672 | 8 | 18079512 | 19278059 | 1.362634799 | 4 | amp |
| TCGA-09-1672 | 8 | 19297331 | 19822880 | 0.956864699 | 3 | amp |
| TCGA-09-1672 | 8 | 20003270 | 20008275 | 2.236447582 | 5 | amp |
| TCGA-09-1672 | 8 | 20022337 | 20038533 | 1.429054182 | 4 | amp |
| TCGA-09-1672 | 8 | 20061962 | 21823873 | 1.001846789 | 3 | amp |
| TCGA-09-1672 | 8 | 21824320 | 21857235 | 1.399021217 | 4 | amp |
| TCGA-09-1672 | 8 | 21859582 | 22006501 | 1.034907987 | 3 | amp |
| TCGA-09-1672 | 8 | 22051478 | 23118121 | 1.069826859 | 3 | amp |
| TCGA-09-1672 | 8 | 23155535 | 23293007 | 1.518691827 | 4 | amp |
| TCGA-09-1672 | 8 | 23702237 | 24772446 | 1.502274517 | 4 | amp |
| TCGA-09-1672 | 8 | 24773059 | 24773299 | 1.175551904 | 3 | amp |
| TCGA-09-1672 | 8 | 25126320 | 25293073 | 1.633695137 | 4 | amp |
| TCGA-09-1672 | 8 | 25293708 | 25327513 | 0.867722419 | 3 | amp |
| TCGA-09-1672 | 8 | 25337414 | 25766090 | 1.501464012 | 4 | amp |
| TCGA-09-1672 | 8 | 25890544 | 26441526 | 1.037623284 | 3 | amp |
| TCGA-09-1672 | 8 | 26481590 | 27402059 | 1.742829662 | 5 | amp |
| TCGA-09-1672 | 8 | 27455702 | 27622804 | 1.334433082 | 4 | amp |
| TCGA-09-1672 | 8 | 27632997 | 27685728 | 0.961191219 | 3 | amp |
| TCGA-09-1672 | 8 | 27690541 | 30511152 | 1.462928423 | 4 | amp |
| TCGA-09-1672 | 8 | 30536991 | 30560803 | 1.924067743 | 5 | amp |
| TCGA-09-1672 | 8 | 30565563 | 30954399 | 1.113998175 | 3 | amp |
| TCGA-09-1672 | 8 | 30958327 | 32614103 | 1.340928395 | 4 | amp |
| TCGA-09-1672 | 8 | 32616768 | 33318989 | 1.908996263 | 5 | amp |
| TCGA-09-1672 | 8 | 33361221 | 36746770 | 1.406154512 | 4 | amp |
| TCGA-09-1672 | 8 | 36763213 | 36790552 | 1.959704152 | 5 | amp |
| TCGA-09-1672 | 8 | 36792994 | 37702789 | 1.105767858 | 3 | amp |
| TCGA-09-1672 | 8 | 37704322 | 38008364 | 1.328835526 | 4 | amp |
| TCGA-09-1672 | 8 | 38021122 | 38139162 | 0.918804586 | 3 | amp |
| TCGA-09-1672 | 8 | 38145959 | 38264990 | 1.365949895 | 4 | amp |
| TCGA-09-1672 | 8 | 38265700 | 38678196 | 1.719593385 | 5 | amp |
| TCGA-09-1672 | 8 | 38681465 | 38827240 | 1.436192281 | 4 | amp |
| TCGA-09-1672 | 8 | 38832509 | 39691550 | 0.977013183 | 3 | amp |
| TCGA-09-1672 | 8 | 39694632 | 41563769 | 1.366894484 | 4 | amp |
| TCGA-09-1672 | 8 | 41566244 | 42033709 | 1.024154135 | 3 | amp |
| TCGA-09-1672 | 8 | 42036377 | 42608507 | 1.252603541 | 4 | amp |
| TCGA-09-1672 | 8 | 42610907 | 42623593 | 2.023157058 | 5 | amp |

| | | | | | | |
|---|---|---|---|---|---|---|
| TCGA-09-1672 | 8 | 43196976 | 53025947 | 1.091813059 | 3 | amp |
| TCGA-09-1672 | 8 | 53028756 | 53092896 | 1.480083002 | 4 | amp |
| TCGA-09-1672 | 8 | 53124593 | 59720383 | 1.069936879 | 3 | amp |
| TCGA-09-1672 | 8 | 59720632 | 62479921 | 1.349106433 | 4 | amp |
| TCGA-09-1672 | 8 | 62489279 | 68423892 | 1.061102102 | 3 | amp |
| TCGA-09-1672 | 8 | 68430099 | 71087102 | 1.393095567 | 4 | amp |
| TCGA-09-1672 | 8 | 71126103 | 71646704 | 0.984050429 | 3 | amp |
| TCGA-09-1672 | 8 | 72111554 | 72946089 | 1.361508989 | 4 | amp |
| TCGA-09-1672 | 8 | 72946436 | 75262874 | 1.059672085 | 3 | amp |
| TCGA-09-1672 | 8 | 75263484 | 79514106 | 1.32154571 | 4 | amp |
| TCGA-09-1672 | 8 | 79588007 | 81431222 | 0.955524505 | 3 | amp |
| TCGA-09-1672 | 8 | 81431430 | 81903799 | 1.485001115 | 4 | amp |
| TCGA-09-1672 | 8 | 81905279 | 86377719 | 0.997146097 | 3 | amp |
| TCGA-09-1672 | 8 | 87060663 | 87563496 | 0.99265613 | 3 | amp |
| TCGA-09-1672 | 8 | 87567025 | 89180229 | 1.328650865 | 4 | amp |
| TCGA-09-1672 | 8 | 89198645 | 92083561 | 1.016481564 | 3 | amp |
| TCGA-09-1672 | 8 | 92086038 | 93029642 | 1.443211891 | 4 | amp |
| TCGA-09-1672 | 8 | 93074716 | 97270931 | 1.094361851 | 3 | amp |
| TCGA-09-1672 | 8 | 97274237 | 99217526 | 1.382837034 | 4 | amp |
| TCGA-09-1672 | 8 | 99224620 | 100287515 | 1.062031284 | 3 | amp |
| TCGA-09-1672 | 8 | 100396430 | 100880724 | 1.359027278 | 4 | amp |
| TCGA-09-1672 | 8 | 100882973 | 102214021 | 1.057544706 | 3 | amp |
| TCGA-09-1672 | 8 | 102504917 | 103299846 | 1.335202078 | 4 | amp |
| TCGA-09-1672 | 8 | 103300378 | 110394805 | 1.133242573 | 3 | amp |
| TCGA-09-1672 | 8 | 110396267 | 117782637 | 1.278180619 | 4 | amp |
| TCGA-09-1672 | 8 | 117783647 | 117875553 | 0.897442743 | 3 | amp |
| TCGA-09-1672 | 8 | 117878776 | 120831785 | 1.428856414 | 4 | amp |
| TCGA-09-1672 | 8 | 120843851 | 120886267 | 0.88831605 | 3 | amp |
| TCGA-09-1672 | 8 | 120940608 | 124031549 | 1.364849021 | 4 | amp |
| TCGA-09-1672 | 8 | 124033652 | 124383246 | 1.079350414 | 3 | amp |
| TCGA-09-1672 | 8 | 124383466 | 124547057 | 1.449269029 | 4 | amp |
| TCGA-09-1672 | 8 | 124657943 | 124749626 | 1.825684341 | 5 | amp |
| TCGA-09-1672 | 8 | 124810253 | 125115588 | 1.762756241 | 5 | amp |
| TCGA-09-1672 | 8 | 125131074 | 131414219 | 1.328886221 | 4 | amp |
| TCGA-09-1672 | 8 | 131792619 | 133623634 | 1.772435031 | 5 | amp |
| TCGA-09-1672 | 8 | 133627253 | 133829346 | 1.245889147 | 4 | amp |
| TCGA-09-1672 | 8 | 133829566 | 134256675 | 1.849638337 | 5 | amp |
| TCGA-09-1672 | 8 | 134258822 | 136659341 | 1.458193976 | 4 | amp |
| TCGA-09-1672 | 8 | 139144788 | 139856423 | 1.818295247 | 5 | amp |
| TCGA-09-1672 | 8 | 139889945 | 142190959 | 1.256396579 | 4 | amp |
| TCGA-09-1672 | 8 | 142195187 | 143784828 | 0.98140979 | 3 | amp |
| TCGA-09-1672 | 9 | 17322 | 175785 | 0.575179722 | 1 | loss |
| TCGA-09-1672 | 9 | 178772 | 991062 | 1.303107825 | 4 | amp |
| TCGA-09-1672 | 9 | 1053682 | 2076368 | 0.887076494 | 3 | amp |
| TCGA-09-1672 | 9 | 2077582 | 2192781 | 1.307108372 | 4 | amp |
| TCGA-09-1672 | 9 | 2635392 | 4850395 | 1.049145663 | 3 | amp |
| TCGA-09-1672 | 9 | 5462797 | 7103883 | 1.01368763 | 3 | amp |
| TCGA-09-1672 | 9 | 7128063 | 13115349 | 1.216109362 | 4 | amp |
| TCGA-09-1672 | 9 | 13119454 | 14720415 | 1.004770399 | 3 | amp |
| TCGA-09-1672 | 9 | 14722116 | 15192700 | 1.211294129 | 4 | amp |

| | | | | | | |
|---|---|---|---|---|---|---|
| TCGA-09-1672 | 9 | 17465927 | 19032947 | 1.131674994 | 3 | amp |
| TCGA-09-1672 | 9 | 19516092 | 19622337 | 1.40196405 | 4 | amp |
| TCGA-09-1672 | 9 | 19785919 | 21029405 | 0.987159894 | 3 | amp |
| TCGA-09-1672 | 9 | 21077286 | 21512208 | 1.272104831 | 4 | amp |
| TCGA-09-1672 | 9 | 21815414 | 27062794 | 0.880109281 | 3 | amp |
| TCGA-09-1672 | 9 | 27109531 | 27220154 | 1.427618039 | 4 | amp |
| TCGA-09-1672 | 9 | 27228163 | 27567136 | 0.953369456 | 3 | amp |
| TCGA-09-1672 | 9 | 27609977 | 32488251 | 1.198972546 | 4 | amp |
| TCGA-09-1672 | 9 | 32488658 | 33076653 | 0.915258548 | 3 | amp |
| TCGA-09-1672 | 9 | 33113397 | 33248506 | 1.372914116 | 4 | amp |
| TCGA-09-1672 | 9 | 33290492 | 33442527 | 1.372849131 | 4 | amp |
| TCGA-09-1672 | 9 | 33442758 | 33676700 | 0.899065246 | 3 | amp |
| TCGA-09-1672 | 9 | 33795531 | 33935916 | 1.362927631 | 4 | amp |
| TCGA-09-1672 | 9 | 33941633 | 34017156 | 0.955333774 | 3 | amp |
| TCGA-09-1672 | 9 | 34088276 | 34098538 | 1.793221794 | 5 | amp |
| TCGA-09-1672 | 9 | 34106401 | 34290399 | 1.24365009 | 4 | amp |
| TCGA-09-1672 | 9 | 35147806 | 35381214 | 1.186962898 | 3 | amp |
| TCGA-09-1672 | 9 | 35381514 | 35390728 | 1.73117763 | 5 | amp |
| TCGA-09-1672 | 9 | 35396415 | 35548545 | 1.420038995 | 4 | amp |
| TCGA-09-1672 | 9 | 35555017 | 35606161 | 0.936706506 | 3 | amp |
| TCGA-09-1672 | 9 | 35618199 | 35725746 | 0.9750678 | 3 | amp |
| TCGA-09-1672 | 9 | 36063774 | 37501895 | 1.0065138 | 3 | amp |
| TCGA-09-1672 | 9 | 37502969 | 37740897 | 1.215706152 | 4 | amp |
| TCGA-09-1672 | 9 | 37859997 | 42673569 | 0.975526724 | 3 | amp |
| TCGA-09-1672 | 9 | 42853632 | 66962667 | 0.951905971 | 3 | amp |
| TCGA-09-1672 | 9 | 67026619 | 67293764 | 1.382727193 | 4 | amp |
| TCGA-09-1672 | 9 | 71006424 | 71155771 | 1.413320802 | 4 | amp |
| TCGA-09-1672 | 9 | 71224008 | 72459546 | 0.985769369 | 3 | amp |
| TCGA-09-1672 | 9 | 72471409 | 72833562 | 1.234565988 | 4 | amp |
| TCGA-09-1672 | 9 | 72929644 | 73028326 | 0.992410295 | 3 | amp |
| TCGA-09-1672 | 9 | 73150784 | 73213640 | 1.831989777 | 5 | amp |
| TCGA-09-1672 | 9 | 73218201 | 74309533 | 1.164981594 | 3 | amp |
| TCGA-09-1672 | 9 | 74312890 | 74327207 | 1.765130785 | 5 | amp |
| TCGA-09-1672 | 9 | 74329777 | 77249737 | 0.993290067 | 3 | amp |
| TCGA-09-1672 | 9 | 77257263 | 77449095 | 1.210045148 | 4 | amp |
| TCGA-09-1672 | 9 | 77668934 | 78772139 | 1.053170987 | 3 | amp |
| TCGA-09-1672 | 9 | 78773842 | 78938205 | 1.298197044 | 4 | amp |
| TCGA-09-1672 | 9 | 78942919 | 78973873 | 1.695189799 | 5 | amp |
| TCGA-09-1672 | 9 | 79229440 | 79461632 | 1.281680504 | 4 | amp |
| TCGA-09-1672 | 9 | 79959871 | 84300839 | 0.988151041 | 3 | amp |
| TCGA-09-1672 | 9 | 84531518 | 85640840 | 1.207640174 | 4 | amp |
| TCGA-09-1672 | 9 | 85862741 | 86356965 | 0.96241539 | 3 | amp |
| TCGA-09-1672 | 9 | 86893045 | 88162232 | 1.272035384 | 4 | amp |
| TCGA-09-1672 | 9 | 88293221 | 88903685 | 0.949679732 | 3 | amp |
| TCGA-09-1672 | 9 | 89763609 | 91628545 | 0.971891185 | 3 | amp |
| TCGA-09-1672 | 9 | 91652845 | 91727565 | 1.355433415 | 4 | amp |
| TCGA-09-1672 | 9 | 91793272 | 94058390 | 1.055365761 | 3 | amp |
| TCGA-09-1672 | 9 | 95005485 | 99126848 | 0.947251181 | 3 | amp |
| TCGA-09-1672 | 9 | 100053636 | 100139214 | 1.225303291 | 4 | amp |
| TCGA-09-1672 | 9 | 100190730 | 101785744 | 0.988224523 | 3 | amp |

| | | | | | | |
|---|---|---|---|---|---|---|
| TCGA-09-1672 | 9 | 101787108 | 101824436 | 1.980786252 | 5 | amp |
| TCGA-09-1672 | 9 | 101824466 | 101832181 | 1.218158372 | 4 | amp |
| TCGA-09-1672 | 9 | 102888627 | 107549265 | 1.004544492 | 3 | amp |
| TCGA-09-1672 | 9 | 107550147 | 107595073 | 1.268971274 | 4 | amp |
| TCGA-09-1672 | 9 | 107599198 | 108536373 | 0.907221078 | 3 | amp |
| TCGA-09-1672 | 9 | 109685654 | 109765760 | 1.450232045 | 4 | amp |
| TCGA-09-1672 | 9 | 109771767 | 111976158 | 0.92336532 | 3 | amp |
| TCGA-09-1672 | 9 | 111979132 | 112969851 | 1.409930148 | 4 | amp |
| TCGA-09-1672 | 9 | 113006407 | 114246850 | 1.054057067 | 3 | amp |
| TCGA-09-1672 | 9 | 114470109 | 115920099 | 0.993953799 | 3 | amp |
| TCGA-09-1672 | 9 | 115923763 | 116029750 | 1.20359331 | 4 | amp |
| TCGA-09-1672 | 9 | 116034687 | 116984584 | 0.97947478 | 3 | amp |
| TCGA-09-1672 | 9 | 116993325 | 117086392 | 1.272204151 | 4 | amp |
| TCGA-09-1672 | 9 | 117087033 | 117186839 | 1.02467124 | 3 | amp |
| TCGA-09-1672 | 9 | 117187195 | 117783550 | 1.195551074 | 4 | amp |
| TCGA-09-1672 | 9 | 117786183 | 117822324 | 1.733770367 | 5 | amp |
| TCGA-09-1672 | 9 | 117825121 | 123220933 | 1.429393164 | 4 | amp |
| TCGA-09-1672 | 9 | 123222837 | 125154825 | 0.99439384 | 3 | amp |
| TCGA-09-1672 | 9 | 125239286 | 125585505 | 1.373406108 | 4 | amp |
| TCGA-09-1672 | 9 | 125758284 | 129728236 | 0.948736892 | 3 | amp |
| TCGA-09-1672 | 9 | 129739938 | 130127671 | 1.305528043 | 4 | amp |
| TCGA-09-1672 | 9 | 130145710 | 130341277 | 0.979066246 | 3 | amp |
| TCGA-09-1672 | 9 | 130413846 | 130453154 | 1.340227051 | 4 | amp |
| TCGA-09-1672 | 9 | 131071781 | 131285648 | 0.9534936 | 3 | amp |
| TCGA-09-1672 | 9 | 131285787 | 131394796 | 1.244013619 | 4 | amp |
| TCGA-09-1672 | 9 | 131394803 | 131735543 | 0.887241447 | 3 | amp |
| TCGA-09-1672 | 9 | 131741513 | 131768988 | 1.356656042 | 4 | amp |
| TCGA-09-1672 | 9 | 131770956 | 131909764 | 0.902222432 | 3 | amp |
| TCGA-09-1672 | 9 | 132665093 | 136031486 | 0.938306165 | 3 | amp |
| TCGA-09-1672 | X | 1314833 | 1414394 | 1.347571662 | 4 | amp |
| TCGA-09-1672 | X | 1419331 | 1471426 | 1.680240102 | 5 | amp |
| TCGA-09-1672 | X | 1475111 | 7243579 | 1.213850465 | 4 | amp |
| TCGA-09-1672 | X | 7251967 | 11790384 | 1.017570253 | 3 | amp |
| TCGA-09-1672 | X | 11790691 | 12736924 | 1.408926377 | 4 | amp |
| TCGA-09-1672 | X | 12738559 | 15526665 | 1.030702946 | 3 | amp |
| TCGA-09-1672 | X | 15527443 | 15646291 | 1.359290679 | 4 | amp |
| TCGA-09-1672 | X | 15657631 | 15870749 | 0.928136269 | 3 | amp |
| TCGA-09-1672 | X | 16142012 | 16850925 | 1.267282474 | 4 | amp |
| TCGA-09-1672 | X | 16852352 | 18836269 | 1.058959134 | 3 | amp |
| TCGA-09-1672 | X | 18842002 | 19854441 | 1.291642916 | 4 | amp |
| TCGA-09-1672 | X | 19984152 | 20082944 | 1.324640707 | 4 | amp |
| TCGA-09-1672 | X | 20146366 | 24546307 | 1.005942801 | 3 | amp |
| TCGA-09-1672 | X | 24549770 | 24690777 | 1.291832205 | 4 | amp |
| TCGA-09-1672 | X | 24766382 | 37651329 | 1.058772519 | 3 | amp |
| TCGA-09-1672 | X | 37652870 | 39934457 | 1.288131866 | 4 | amp |
| TCGA-09-1672 | X | 41554859 | 47045211 | 1.043624634 | 3 | amp |
| TCGA-09-1672 | X | 47045456 | 47070649 | 1.316384299 | 4 | amp |
| TCGA-09-1672 | X | 47657211 | 47926326 | 1.187781906 | 3 | amp |
| TCGA-09-1672 | X | 48025142 | 48326194 | 1.65069338 | 4 | amp |
| TCGA-09-1672 | X | 48326224 | 49079356 | 0.97337285 | 3 | amp |

| | | | | | | |
|---|---|---|---|---|---|---|
| TCGA-09-1672 | X | 49161822 | 49370666 | 0.402466545 | 1 | loss |
| TCGA-09-1672 | X | 49452094 | 50659661 | 1.382369352 | 4 | amp |
| TCGA-09-1672 | X | 51935092 | 52544598 | 1.368129082 | 4 | amp |
| TCGA-09-1672 | X | 52545600 | 52826426 | 1.916357728 | 5 | amp |
| TCGA-09-1672 | X | 52841566 | 52896183 | 1.160580515 | 3 | amp |
| TCGA-09-1672 | X | 53239556 | 53442143 | 1.248426769 | 4 | amp |
| TCGA-09-1672 | X | 53560260 | 53572175 | 1.716518344 | 5 | amp |
| TCGA-09-1672 | X | 53573372 | 53583302 | 1.271457855 | 4 | amp |
| TCGA-09-1672 | X | 53584103 | 54987363 | 1.014309848 | 3 | amp |
| TCGA-09-1672 | X | 54989632 | 55054271 | 1.532898607 | 4 | amp |
| TCGA-09-1672 | X | 55515063 | 62926353 | 0.961531985 | 3 | amp |
| TCGA-09-1672 | X | 62944381 | 67339191 | 1.253975321 | 4 | amp |
| TCGA-09-1672 | X | 67412737 | 69249430 | 1.079858232 | 3 | amp |
| TCGA-09-1672 | X | 69250284 | 69500146 | 1.306301645 | 4 | amp |
| TCGA-09-1672 | X | 69500360 | 70468390 | 1.03275171 | 3 | amp |
| TCGA-09-1672 | X | 70511605 | 70524153 | 1.690549151 | 5 | amp |
| TCGA-09-1672 | X | 70524362 | 70602569 | 1.237625768 | 4 | amp |
| TCGA-09-1672 | X | 70602604 | 79932900 | 1.005482254 | 3 | amp |
| TCGA-09-1672 | X | 83411020 | 99411670 | 0.953833826 | 3 | amp |
| TCGA-09-1672 | X | 99551213 | 100496783 | 1.250997335 | 4 | amp |
| TCGA-09-1672 | X | 100497236 | 100506108 | 1.706489191 | 5 | amp |
| TCGA-09-1672 | X | 100507559 | 100912630 | 1.216941431 | 4 | amp |
| TCGA-09-1672 | X | 101091107 | 101139797 | 1.770088705 | 5 | amp |
| TCGA-09-1672 | X | 101571894 | 101625098 | 1.500574497 | 4 | amp |
| TCGA-09-1672 | X | 102331133 | 102529587 | 1.167350142 | 3 | amp |
| TCGA-09-1672 | X | 102962206 | 102979237 | 1.748513773 | 5 | amp |
| TCGA-09-1672 | X | 102979412 | 103045550 | 1.414823166 | 4 | amp |
| TCGA-09-1672 | X | 103080021 | 103903695 | 0.928002723 | 3 | amp |
| TCGA-09-1672 | X | 104440113 | 105866071 | 1.2211732 | 4 | amp |
| TCGA-09-1672 | X | 105868295 | 106773749 | 0.976368286 | 3 | amp |
| TCGA-09-1672 | X | 106776697 | 108780267 | 1.330827911 | 4 | amp |
| TCGA-09-1672 | X | 109352250 | 113865442 | 1.271655439 | 4 | amp |
| TCGA-09-1672 | X | 113887133 | 118370670 | 1.053769238 | 3 | amp |
| TCGA-09-1672 | X | 118759250 | 118797654 | 1.590902131 | 4 | amp |
| TCGA-09-1672 | X | 118809483 | 119394923 | 0.957102369 | 3 | amp |
| TCGA-09-1672 | X | 119402020 | 119438394 | 1.399402378 | 4 | amp |
| TCGA-09-1672 | X | 119495964 | 119512640 | 1.70637756 | 5 | amp |
| TCGA-09-1672 | X | 119513258 | 119738156 | 1.017486799 | 3 | amp |
| TCGA-09-1672 | X | 122319642 | 122616950 | 1.300902723 | 4 | amp |
| TCGA-09-1672 | X | 123517403 | 128602935 | 1.211699348 | 4 | amp |
| TCGA-09-1672 | X | 128718242 | 129799729 | 0.98108774 | 3 | amp |
| TCGA-09-1672 | X | 129801459 | 129843300 | 1.448419793 | 4 | amp |
| TCGA-09-1672 | X | 130407687 | 132352128 | 1.263493036 | 4 | amp |
| TCGA-09-1672 | X | 132436845 | 132670356 | 1.799076939 | 5 | amp |
| TCGA-09-1672 | X | 132730427 | 133087277 | 1.259264594 | 4 | amp |
| TCGA-09-1672 | X | 134290858 | 134978504 | 1.041294052 | 3 | amp |
| TCGA-09-1672 | X | 134983663 | 134994164 | 1.730920904 | 5 | amp |
| TCGA-09-1672 | X | 135092551 | 135498691 | 1.04706195 | 3 | amp |
| TCGA-09-1672 | X | 135618136 | 135764163 | 1.307175073 | 4 | amp |
| TCGA-09-1672 | X | 135764899 | 140271250 | 1.009471289 | 3 | amp |

| | | | | | | |
|---|---|---|---|---|---|---|
| TCGA-09-1672 | X | 140335640 | 141291826 | 1.365620617 | 4 | amp |
| TCGA-09-1672 | X | 142113779 | 147582700 | 1.02181502 | 3 | amp |
| TCGA-09-1672 | X | 147733465 | 148573228 | 1.424390681 | 4 | amp |
| TCGA-09-1672 | X | 149101785 | 149639317 | 1.676662073 | 5 | amp |
| TCGA-09-1672 | X | 149639398 | 149984575 | 1.160153948 | 3 | amp |
| TCGA-09-1672 | X | 150817020 | 152037673 | 1.196075028 | 4 | amp |
| TCGA-09-1672 | X | 153416095 | 153539645 | 1.245209785 | 4 | amp |
| TCGA-09-1672 | X | 154156808 | 154227934 | 1.193285302 | 3 | amp |
| TCGA-09-1672 | X | 155227378 | 155252897 | 1.347595969 | 4 | amp |
| TCGA-10-0930 | 1 | 14642 | 11856479 | 1.64850671 | 6 | amp |
| TCGA-10-0930 | 1 | 11860233 | 16458410 | 1.306496656 | 5 | amp |
| TCGA-10-0930 | 1 | 16458543 | 28856474 | 0.769108149 | 3 | amp |
| TCGA-10-0930 | 1 | 28858258 | 29631383 | 1.002330854 | 4 | amp |
| TCGA-10-0930 | 1 | 29631841 | 35580918 | 1.286585768 | 5 | amp |
| TCGA-10-0930 | 1 | 35650005 | 36020141 | 1.015024299 | 4 | amp |
| TCGA-10-0930 | 1 | 36023747 | 39305420 | 1.202278799 | 5 | amp |
| TCGA-10-0930 | 1 | 39311564 | 40030238 | 1.003386509 | 4 | amp |
| TCGA-10-0930 | 1 | 40030341 | 40310317 | 1.218597636 | 5 | amp |
| TCGA-10-0930 | 1 | 40312840 | 47080755 | 0.730015507 | 3 | amp |
| TCGA-10-0930 | 1 | 53535620 | 55183365 | 0.76774261 | 3 | amp |
| TCGA-10-0930 | 1 | 60499110 | 60521112 | 0.748100442 | 3 | amp |
| TCGA-10-0930 | 1 | 60538201 | 94473302 | 0.930754757 | 4 | amp |
| TCGA-10-0930 | 1 | 94473761 | 94578635 | 1.244350682 | 5 | amp |
| TCGA-10-0930 | 1 | 94586508 | 109547396 | 0.921188057 | 4 | amp |
| TCGA-10-0930 | 1 | 109553518 | 110884898 | 1.187261852 | 5 | amp |
| TCGA-10-0930 | 1 | 110888135 | 144158263 | 1.022890159 | 4 | amp |
| TCGA-10-0930 | 1 | 144158836 | 144224209 | 1.552886086 | 6 | amp |
| TCGA-10-0930 | 1 | 144363623 | 144864349 | 1.138993098 | 5 | amp |
| TCGA-10-0930 | 1 | 144865749 | 145311992 | 1.024168272 | 4 | amp |
| TCGA-10-0930 | 1 | 145312643 | 145361896 | 1.533465734 | 6 | amp |
| TCGA-10-0930 | 1 | 145362944 | 145756634 | 1.292914698 | 5 | amp |
| TCGA-10-0930 | 1 | 145761169 | 146013755 | 0.960556453 | 4 | amp |
| TCGA-10-0930 | 1 | 146018952 | 146232708 | 1.27306289 | 5 | amp |
| TCGA-10-0930 | 1 | 146233255 | 146419429 | 1.007507531 | 4 | amp |
| TCGA-10-0930 | 1 | 146419985 | 146638531 | 1.324263906 | 5 | amp |
| TCGA-10-0930 | 1 | 146639278 | 147566175 | 1.012230492 | 4 | amp |
| TCGA-10-0930 | 1 | 147575887 | 148252176 | 1.204333759 | 5 | amp |
| TCGA-10-0930 | 1 | 148252628 | 148328426 | 1.537373669 | 6 | amp |
| TCGA-10-0930 | 1 | 148328995 | 158326394 | 1.270034533 | 5 | amp |
| TCGA-10-0930 | 1 | 158326488 | 158617539 | 1.014162551 | 4 | amp |
| TCGA-10-0930 | 1 | 158618220 | 161161380 | 1.267270042 | 5 | amp |
| TCGA-10-0930 | 1 | 161161792 | 161201296 | 1.556873019 | 6 | amp |
| TCGA-10-0930 | 1 | 161202546 | 161692848 | 1.219826156 | 5 | amp |
| TCGA-10-0930 | 1 | 161693073 | 166810379 | 1.017731216 | 4 | amp |
| TCGA-10-0930 | 1 | 166815791 | 167083053 | 0.719789527 | 3 | amp |
| TCGA-10-0930 | 1 | 199996947 | 200827214 | 0.757307122 | 3 | amp |
| TCGA-10-0930 | 1 | 200867388 | 202288339 | 0.998416005 | 4 | amp |
| TCGA-10-0930 | 1 | 202300905 | 202914323 | 0.785113267 | 3 | amp |
| TCGA-10-0930 | 1 | 202915532 | 207238562 | 0.953490791 | 4 | amp |
| TCGA-10-0930 | 1 | 207240827 | 207966961 | 0.782664037 | 3 | amp |

| | | | | | | |
|---|---|---|---|---|---|---|
| TCGA-10-0930 | 1 | 207975180 | 210003532 | 0.998661085 | 4 | amp |
| TCGA-10-0930 | 1 | 210004128 | 220978640 | 0.736228967 | 3 | amp |
| TCGA-10-0930 | 1 | 220986546 | 249231325 | 1.00532534 | 4 | amp |
| TCGA-10-0930 | 10 | 92880 | 5032269 | 1.01159565 | 4 | amp |
| TCGA-10-0930 | 10 | 5034003 | 5842674 | 0.866840384 | 3 | amp |
| TCGA-10-0930 | 10 | 5920021 | 11963361 | 1.002664882 | 4 | amp |
| TCGA-10-0930 | 10 | 11971803 | 12077110 | 0.785133332 | 3 | amp |
| TCGA-10-0930 | 10 | 12077175 | 12077479 | 1.006138055 | 4 | amp |
| TCGA-10-0930 | 10 | 12123428 | 12178196 | 1.211851489 | 5 | amp |
| TCGA-10-0930 | 10 | 12185094 | 14870327 | 1.046951148 | 4 | amp |
| TCGA-10-0930 | 10 | 14881883 | 14961843 | 0.849500211 | 3 | amp |
| TCGA-10-0930 | 10 | 14964933 | 15296918 | 0.975884706 | 4 | amp |
| TCGA-10-0930 | 10 | 15317839 | 22680873 | 0.874567262 | 3 | amp |
| TCGA-10-0930 | 10 | 22689997 | 24835255 | 0.98078213 | 4 | amp |
| TCGA-10-0930 | 10 | 24873257 | 26457841 | 0.856421073 | 3 | amp |
| TCGA-10-0930 | 10 | 26459286 | 27031555 | 0.988308378 | 4 | amp |
| TCGA-10-0930 | 10 | 27035211 | 29188286 | 0.86007077 | 3 | amp |
| TCGA-10-0930 | 10 | 29578042 | 29783945 | 1.20886258 | 5 | amp |
| TCGA-10-0930 | 10 | 29784005 | 31750185 | 1.041993749 | 4 | amp |
| TCGA-10-0930 | 10 | 31784679 | 35772428 | 0.823671345 | 3 | amp |
| TCGA-10-0930 | 10 | 35790385 | 50725169 | 1.014419236 | 4 | amp |
| TCGA-10-0930 | 10 | 50732065 | 51087917 | 1.180258649 | 5 | amp |
| TCGA-10-0930 | 10 | 51093235 | 51947289 | 0.996198954 | 4 | amp |
| TCGA-10-0930 | 10 | 51949242 | 64022601 | 0.883192245 | 3 | amp |
| TCGA-10-0930 | 10 | 64135903 | 64911989 | 1.038692128 | 4 | amp |
| TCGA-10-0930 | 10 | 64913234 | 69918433 | 0.869701125 | 3 | amp |
| TCGA-10-0930 | 10 | 69921437 | 70968803 | 1.01367676 | 4 | amp |
| TCGA-10-0930 | 10 | 70980162 | 74100955 | 1.18537132 | 5 | amp |
| TCGA-10-0930 | 10 | 74103076 | 75479107 | 1.007374866 | 4 | amp |
| TCGA-10-0930 | 10 | 75482241 | 75673526 | 1.241526743 | 5 | amp |
| TCGA-10-0930 | 10 | 75673691 | 75984382 | 0.959105868 | 4 | amp |
| TCGA-10-0930 | 10 | 76074403 | 76748883 | 0.819709191 | 3 | amp |
| TCGA-10-0930 | 10 | 76780305 | 79556002 | 1.045383817 | 4 | amp |
| TCGA-10-0930 | 10 | 79556130 | 86018449 | 1.142885338 | 5 | amp |
| TCGA-10-0930 | 10 | 86130796 | 88416104 | 0.979466297 | 4 | amp |
| TCGA-10-0930 | 10 | 88416907 | 88492748 | 1.234826015 | 5 | amp |
| TCGA-10-0930 | 10 | 88635748 | 88971981 | 1.018516744 | 4 | amp |
| TCGA-10-0930 | 10 | 88973843 | 89530813 | 0.856335917 | 3 | amp |
| TCGA-10-0930 | 10 | 90341274 | 90683012 | 0.880941686 | 3 | amp |
| TCGA-10-0930 | 10 | 90694901 | 92676036 | 0.963860829 | 4 | amp |
| TCGA-10-0930 | 10 | 92677477 | 93952433 | 0.878713032 | 3 | amp |
| TCGA-10-0930 | 10 | 94070872 | 94594598 | 0.966225202 | 4 | amp |
| TCGA-10-0930 | 10 | 94653041 | 94774077 | 0.831435289 | 3 | amp |
| TCGA-10-0930 | 10 | 94816676 | 94837070 | 1.01427581 | 4 | amp |
| TCGA-10-0930 | 10 | 95066648 | 95109765 | 1.200560166 | 5 | amp |
| TCGA-10-0930 | 10 | 95110942 | 95400320 | 1.048936526 | 4 | amp |
| TCGA-10-0930 | 10 | 95400671 | 95459914 | 0.808194245 | 3 | amp |
| TCGA-10-0930 | 10 | 95517888 | 96353421 | 0.985278346 | 4 | amp |
| TCGA-10-0930 | 10 | 96354411 | 96745980 | 0.868412244 | 3 | amp |
| TCGA-10-0930 | 10 | 96748574 | 98113267 | 1.008226801 | 4 | amp |

| | | | | | | |
|---|---|---|---|---|---|---|
| TCGA-10-0930 | 10 | 98114939 | 98240220 | 1.257640732 | 5 | amp |
| TCGA-10-0930 | 10 | 98281993 | 98336608 | 0.886491862 | 4 | amp |
| TCGA-10-0930 | 10 | 98355302 | 100250000 | 1.182568179 | 5 | amp |
| TCGA-10-0930 | 10 | 100374657 | 101998003 | 1.024867948 | 4 | amp |
| TCGA-10-0930 | 10 | 102003404 | 102566394 | 1.164233068 | 5 | amp |
| TCGA-10-0930 | 10 | 102568779 | 102719278 | 0.945239903 | 4 | amp |
| TCGA-10-0930 | 10 | 102721591 | 104620179 | 1.147697021 | 5 | amp |
| TCGA-10-0930 | 10 | 104620216 | 104934724 | 0.955522476 | 4 | amp |
| TCGA-10-0930 | 10 | 105046733 | 105648925 | 1.157401046 | 5 | amp |
| TCGA-10-0930 | 10 | 105651828 | 105796896 | 0.953476475 | 4 | amp |
| TCGA-10-0930 | 10 | 105797312 | 105824394 | 1.216178296 | 5 | amp |
| TCGA-10-0930 | 10 | 105830153 | 112349476 | 1.024162105 | 4 | amp |
| TCGA-10-0930 | 10 | 112349609 | 112724827 | 0.8447098 | 3 | amp |
| TCGA-10-0930 | 10 | 112745334 | 113935474 | 0.95881818 | 4 | amp |
| TCGA-10-0930 | 10 | 113937718 | 115400097 | 1.156592732 | 5 | amp |
| TCGA-10-0930 | 10 | 115401117 | 115607147 | 1.033008503 | 4 | amp |
| TCGA-10-0930 | 10 | 115608730 | 115982523 | 0.813304747 | 3 | amp |
| TCGA-10-0930 | 10 | 115985771 | 116444140 | 1.082682083 | 4 | amp |
| TCGA-10-0930 | 10 | 116590589 | 117228880 | 0.764539853 | 3 | amp |
| TCGA-10-0930 | 10 | 117278743 | 120833473 | 1.032850677 | 4 | amp |
| TCGA-10-0930 | 10 | 120867457 | 121285646 | 1.161829156 | 5 | amp |
| TCGA-10-0930 | 10 | 121286756 | 121286996 | 0.979148576 | 4 | amp |
| TCGA-10-0930 | 10 | 121335119 | 121583430 | 0.890138714 | 3 | amp |
| TCGA-10-0930 | 10 | 121652255 | 121691857 | 0.870459351 | 3 | amp |
| TCGA-10-0930 | 10 | 121692477 | 123247685 | 1.01899847 | 4 | amp |
| TCGA-10-0930 | 10 | 123255990 | 124325557 | 1.141682949 | 5 | amp |
| TCGA-10-0930 | 10 | 124329641 | 124377874 | 1.740389501 | 6 | amp |
| TCGA-10-0930 | 10 | 124378778 | 124672493 | 1.176530263 | 5 | amp |
| TCGA-10-0930 | 10 | 124691939 | 124924657 | 1.00085585 | 4 | amp |
| TCGA-10-0930 | 10 | 124931161 | 126449131 | 1.180584767 | 5 | amp |
| TCGA-10-0930 | 10 | 126453948 | 127456306 | 1.033777759 | 4 | amp |
| TCGA-10-0930 | 10 | 127458851 | 129907744 | 1.196687251 | 5 | amp |
| TCGA-10-0930 | 10 | 129908599 | 134521902 | 1.078484283 | 4 | amp |
| TCGA-10-0930 | 10 | 134521915 | 135516111 | 1.101582152 | 5 | amp |
| TCGA-10-0930 | 11 | 86637 | 9225895 | 0.660583585 | 3 | amp |
| TCGA-10-0930 | 11 | 11985971 | 25100211 | 0.654509213 | 3 | amp |
| TCGA-10-0930 | 11 | 26353755 | 34119346 | 0.931487899 | 4 | amp |
| TCGA-10-0930 | 11 | 34120821 | 34670656 | 1.182453098 | 5 | amp |
| TCGA-10-0930 | 11 | 34673062 | 44074639 | 1.004901511 | 4 | amp |
| TCGA-10-0930 | 11 | 44074945 | 46765873 | 1.141551476 | 5 | amp |
| TCGA-10-0930 | 11 | 46765931 | 46886102 | 0.946716994 | 4 | amp |
| TCGA-10-0930 | 11 | 46886643 | 47823542 | 1.20445734 | 5 | amp |
| TCGA-10-0930 | 11 | 47824978 | 56786270 | 0.968296566 | 4 | amp |
| TCGA-10-0930 | 11 | 56949312 | 57559167 | 1.223824384 | 5 | amp |
| TCGA-10-0930 | 11 | 57561457 | 60310079 | 1.015579678 | 4 | amp |
| TCGA-10-0930 | 11 | 60468322 | 60906314 | 1.182489892 | 5 | amp |
| TCGA-10-0930 | 11 | 60971004 | 61018798 | 1.562283996 | 6 | amp |
| TCGA-10-0930 | 11 | 61026075 | 62782443 | 1.280217068 | 5 | amp |
| TCGA-10-0930 | 11 | 62931210 | 63177393 | 0.84305406 | 3 | amp |
| TCGA-10-0930 | 11 | 63230930 | 73760604 | 1.238818516 | 5 | amp |

| | | | | | | |
|---|---|---|---|---|---|---|
| TCGA-10-0930 | 11 | 73765593 | 88068297 | 0.900800629 | 4 | amp |
| TCGA-10-0930 | 11 | 88258464 | 134257557 | 0.695478475 | 3 | amp |
| TCGA-10-0930 | 12 | 73256 | 369237 | 1.600809448 | 6 | amp |
| TCGA-10-0930 | 12 | 394604 | 542533 | 1.255330144 | 5 | amp |
| TCGA-10-0930 | 12 | 547460 | 675349 | 1.614221424 | 6 | amp |
| TCGA-10-0930 | 12 | 772458 | 1250989 | 1.310257356 | 5 | amp |
| TCGA-10-0930 | 12 | 1289654 | 3806171 | 1.642126192 | 6 | amp |
| TCGA-10-0930 | 12 | 3921266 | 4668210 | 1.286272691 | 5 | amp |
| TCGA-10-0930 | 12 | 4700255 | 7457147 | 1.726527091 | 6 | amp |
| TCGA-10-0930 | 12 | 7459113 | 7656323 | 1.366387378 | 5 | amp |
| TCGA-10-0930 | 12 | 7802097 | 9099032 | 1.616152721 | 6 | amp |
| TCGA-10-0930 | 12 | 9142212 | 12966404 | 1.375101314 | 5 | amp |
| TCGA-10-0930 | 12 | 12967051 | 13768632 | 1.547614703 | 6 | amp |
| TCGA-10-0930 | 12 | 13769369 | 16510645 | 1.300290612 | 5 | amp |
| TCGA-10-0930 | 12 | 16516671 | 19610155 | 1.124605475 | 4 | amp |
| TCGA-10-0930 | 12 | 19615427 | 29598393 | 0.881760447 | 3 | amp |
| TCGA-10-0930 | 12 | 29604269 | 34179863 | 1.015512055 | 4 | amp |
| TCGA-10-0930 | 12 | 47160474 | 48110302 | 0.831746982 | 3 | amp |
| TCGA-10-0930 | 12 | 48110636 | 48134635 | 0.957689525 | 4 | amp |
| TCGA-10-0930 | 12 | 48134640 | 48367382 | 1.378400357 | 5 | amp |
| TCGA-10-0930 | 12 | 48367812 | 48389739 | 1.556289468 | 6 | amp |
| TCGA-10-0930 | 12 | 48390279 | 50514033 | 1.294251055 | 5 | amp |
| TCGA-10-0930 | 12 | 50524252 | 52156493 | 1.118668577 | 4 | amp |
| TCGA-10-0930 | 12 | 52159452 | 55042118 | 1.298406756 | 5 | amp |
| TCGA-10-0930 | 12 | 55248335 | 56077894 | 1.051407165 | 4 | amp |
| TCGA-10-0930 | 12 | 56078806 | 58339493 | 1.353057159 | 5 | amp |
| TCGA-10-0930 | 12 | 58340758 | 62749303 | 0.916902184 | 4 | amp |
| TCGA-10-0930 | 12 | 62775237 | 63226096 | 1.323820178 | 5 | amp |
| TCGA-10-0930 | 12 | 63359667 | 95488478 | 1.127591575 | 4 | amp |
| TCGA-10-0930 | 12 | 95498777 | 96411406 | 1.258109925 | 5 | amp |
| TCGA-10-0930 | 12 | 96412490 | 100502364 | 1.08571795 | 4 | amp |
| TCGA-10-0930 | 12 | 100550719 | 104376764 | 1.278063251 | 5 | amp |
| TCGA-10-0930 | 12 | 104376894 | 105583970 | 1.135868911 | 4 | amp |
| TCGA-10-0930 | 12 | 105589022 | 109629763 | 1.35156829 | 5 | amp |
| TCGA-10-0930 | 12 | 109631433 | 110403627 | 1.624192831 | 6 | amp |
| TCGA-10-0930 | 12 | 110405061 | 111078374 | 1.394718939 | 5 | amp |
| TCGA-10-0930 | 12 | 111078771 | 111779900 | 1.683434146 | 6 | amp |
| TCGA-10-0930 | 12 | 111785278 | 113285693 | 1.400555234 | 5 | amp |
| TCGA-10-0930 | 12 | 113303133 | 115121050 | 1.585401421 | 6 | amp |
| TCGA-10-0930 | 12 | 116399016 | 124497504 | 1.240143661 | 5 | amp |
| TCGA-10-0930 | 12 | 124798694 | 133779395 | 1.630372818 | 6 | amp |
| TCGA-10-0930 | 13 | 26104126 | 32954332 | 0.960345742 | 4 | amp |
| TCGA-10-0930 | 13 | 32968737 | 40325276 | 0.871396753 | 3 | amp |
| TCGA-10-0930 | 13 | 77569160 | 78492766 | 0.870007952 | 3 | amp |
| TCGA-10-0930 | 13 | 87045766 | 96361595 | 1.002067489 | 4 | amp |
| TCGA-10-0930 | 13 | 96375464 | 97928696 | 0.846296005 | 3 | amp |
| TCGA-10-0930 | 13 | 97986491 | 115091796 | 1.016732592 | 4 | amp |
| TCGA-10-0930 | 14 | 19377543 | 20819303 | 0.832113013 | 3 | amp |
| TCGA-10-0930 | 14 | 20820364 | 20839484 | 1.021275928 | 4 | amp |
| TCGA-10-0930 | 14 | 20839569 | 20876614 | 1.225024244 | 5 | amp |

| | | | | | | |
|---|---|---|---|---|---|---|
| TCGA-10-0930 | 14 | 20915328 | 22134247 | 1.035436627 | 4 | amp |
| TCGA-10-0930 | 14 | 22167274 | 22447392 | 0.867036117 | 3 | amp |
| TCGA-10-0930 | 14 | 22458697 | 23073026 | 1.017265897 | 4 | amp |
| TCGA-10-0930 | 14 | 23075323 | 24879415 | 1.17834321 | 5 | amp |
| TCGA-10-0930 | 14 | 24880226 | 25103398 | 1.020784872 | 4 | amp |
| TCGA-10-0930 | 14 | 25281836 | 39601280 | 0.845989866 | 3 | amp |
| TCGA-10-0930 | 14 | 39602863 | 39684604 | 1.109247908 | 4 | amp |
| TCGA-10-0930 | 14 | 39684723 | 55434114 | 0.838948634 | 3 | amp |
| TCGA-10-0930 | 14 | 60945002 | 64634404 | 0.849781428 | 3 | amp |
| TCGA-10-0930 | 14 | 64635581 | 65220562 | 1.009851501 | 4 | amp |
| TCGA-10-0930 | 14 | 65230456 | 65453863 | 1.219220404 | 5 | amp |
| TCGA-10-0930 | 14 | 65470938 | 65568336 | 1.029589774 | 4 | amp |
| TCGA-10-0930 | 14 | 66028262 | 67849355 | 0.840163407 | 3 | amp |
| TCGA-10-0930 | 14 | 67849944 | 71267830 | 1.025968016 | 4 | amp |
| TCGA-10-0930 | 14 | 71413615 | 72125161 | 0.775760603 | 3 | amp |
| TCGA-10-0930 | 14 | 72127985 | 76129589 | 1.019025938 | 4 | amp |
| TCGA-10-0930 | 14 | 76135751 | 76242019 | 0.821190961 | 3 | amp |
| TCGA-10-0930 | 14 | 76243081 | 80130326 | 1.02582929 | 4 | amp |
| TCGA-10-0930 | 14 | 80158500 | 89343815 | 0.856671149 | 3 | amp |
| TCGA-10-0930 | 14 | 89628735 | 92105636 | 0.973211371 | 4 | amp |
| TCGA-10-0930 | 14 | 92126102 | 92280139 | 0.829278365 | 3 | amp |
| TCGA-10-0930 | 14 | 92336528 | 94693716 | 0.968938423 | 4 | amp |
| TCGA-10-0930 | 14 | 94696877 | 94750517 | 0.790747883 | 3 | amp |
| TCGA-10-0930 | 14 | 94752399 | 95267397 | 1.18840714 | 5 | amp |
| TCGA-10-0930 | 14 | 95556797 | 95566314 | 0.951524734 | 4 | amp |
| TCGA-10-0930 | 14 | 95569622 | 95599052 | 0.795901997 | 3 | amp |
| TCGA-10-0930 | 14 | 95599603 | 96768454 | 1.096824488 | 4 | amp |
| TCGA-10-0930 | 14 | 96769374 | 97342501 | 0.846354938 | 3 | amp |
| TCGA-10-0930 | 14 | 97347444 | 101393773 | 1.058533149 | 4 | amp |
| TCGA-10-0930 | 14 | 101396239 | 101459669 | 0.714083872 | 3 | amp |
| TCGA-10-0930 | 14 | 101488345 | 107283263 | 1.031276558 | 4 | amp |
| TCGA-10-0930 | 15 | 25232046 | 25523582 | 0.82854443 | 3 | amp |
| TCGA-10-0930 | 15 | 31327662 | 32930091 | 0.850379209 | 3 | amp |
| TCGA-10-0930 | 15 | 32935786 | 33895624 | 1.085919126 | 4 | amp |
| TCGA-10-0930 | 15 | 33905359 | 34123347 | 1.196227695 | 5 | amp |
| TCGA-10-0930 | 15 | 34127160 | 35083507 | 1.037314178 | 4 | amp |
| TCGA-10-0930 | 15 | 35084266 | 40557208 | 0.82329686 | 3 | amp |
| TCGA-10-0930 | 15 | 40564343 | 42462112 | 0.981358782 | 4 | amp |
| TCGA-10-0930 | 15 | 42465865 | 42641696 | 0.817837279 | 3 | amp |
| TCGA-10-0930 | 15 | 42643518 | 43038463 | 0.972038035 | 4 | amp |
| TCGA-10-0930 | 15 | 43044148 | 43773129 | 0.842323185 | 3 | amp |
| TCGA-10-0930 | 15 | 43773184 | 44671996 | 0.987781005 | 4 | amp |
| TCGA-10-0930 | 15 | 44672985 | 45059999 | 0.838025072 | 3 | amp |
| TCGA-10-0930 | 15 | 45119336 | 45399218 | 1.04723455 | 4 | amp |
| TCGA-10-0930 | 15 | 45399451 | 52902632 | 0.784775948 | 3 | amp |
| TCGA-10-0930 | 15 | 57732561 | 58855932 | 0.821414271 | 3 | amp |
| TCGA-10-0930 | 15 | 62336372 | 70386952 | 0.797176214 | 3 | amp |
| TCGA-10-0930 | 15 | 72454261 | 73067445 | 1.014138572 | 4 | amp |
| TCGA-10-0930 | 15 | 73408859 | 73889716 | 0.756028118 | 3 | amp |
| TCGA-10-0930 | 15 | 73991929 | 75651506 | 1.011650659 | 4 | amp |

| | | | | | | |
|---|---|---|---|---|---|---|
| TCGA-10-0930 | 15 | 75651918 | 79189426 | 0.830654241 | 3 | amp |
| TCGA-10-0930 | 15 | 79215247 | 83677483 | 0.998989002 | 4 | amp |
| TCGA-10-0930 | 15 | 83678931 | 84795403 | 0.851489155 | 3 | amp |
| TCGA-10-0930 | 15 | 84859539 | 86029027 | 1.032627856 | 4 | amp |
| TCGA-10-0930 | 15 | 86063852 | 86279402 | 0.801630511 | 3 | amp |
| TCGA-10-0930 | 15 | 86283417 | 87572123 | 0.929292842 | 4 | amp |
| TCGA-10-0930 | 15 | 88420138 | 89762292 | 1.525878022 | 6 | amp |
| TCGA-10-0930 | 15 | 89790830 | 89850933 | 1.20978459 | 5 | amp |
| TCGA-10-0930 | 15 | 89856108 | 90021256 | 1.613207862 | 6 | amp |
| TCGA-10-0930 | 15 | 90022515 | 90265427 | 1.313978852 | 5 | amp |
| TCGA-10-0930 | 15 | 90270353 | 90904554 | 1.508385917 | 6 | amp |
| TCGA-10-0930 | 15 | 90933995 | 91546397 | 1.335112245 | 5 | amp |
| TCGA-10-0930 | 15 | 91548073 | 93043668 | 1.5917874 | 6 | amp |
| TCGA-10-0930 | 15 | 93162627 | 102516522 | 1.295323245 | 5 | amp |
| TCGA-10-0930 | 16 | 66517 | 3350629 | 0.971577927 | 4 | amp |
| TCGA-10-0930 | 16 | 3363035 | 5145543 | 1.192301362 | 5 | amp |
| TCGA-10-0930 | 16 | 7102025 | 8990965 | 0.999822904 | 4 | amp |
| TCGA-10-0930 | 16 | 8992195 | 10576139 | 0.853917118 | 3 | amp |
| TCGA-10-0930 | 16 | 10626705 | 11824638 | 0.992343934 | 4 | amp |
| TCGA-10-0930 | 16 | 11827826 | 11981636 | 0.836616178 | 3 | amp |
| TCGA-10-0930 | 16 | 11988767 | 12798933 | 1.007552072 | 4 | amp |
| TCGA-10-0930 | 16 | 12875000 | 14693848 | 0.80098771 | 3 | amp |
| TCGA-10-0930 | 16 | 14697982 | 15703600 | 0.988935806 | 4 | amp |
| TCGA-10-0930 | 16 | 15704778 | 15733138 | 0.783692565 | 3 | amp |
| TCGA-10-0930 | 16 | 15758586 | 18806022 | 1.046666678 | 4 | amp |
| TCGA-10-0930 | 16 | 18806724 | 18907571 | 0.799861574 | 3 | amp |
| TCGA-10-0930 | 16 | 18908076 | 19516428 | 1.010582255 | 4 | amp |
| TCGA-10-0930 | 16 | 19518994 | 19584621 | 0.78585342 | 3 | amp |
| TCGA-10-0930 | 16 | 19586371 | 20638694 | 0.953219507 | 4 | amp |
| TCGA-10-0930 | 16 | 20648051 | 20931549 | 0.790676876 | 3 | amp |
| TCGA-10-0930 | 16 | 20935268 | 21850661 | 0.950472155 | 4 | amp |
| TCGA-10-0930 | 16 | 21854684 | 22000137 | 0.810130554 | 3 | amp |
| TCGA-10-0930 | 16 | 22120839 | 22336022 | 1.075104832 | 4 | amp |
| TCGA-10-0930 | 16 | 22336466 | 22501809 | 0.824628789 | 3 | amp |
| TCGA-10-0930 | 16 | 22502097 | 22926933 | 0.969668721 | 4 | amp |
| TCGA-10-0930 | 16 | 23079311 | 23119555 | 0.780384111 | 3 | amp |
| TCGA-10-0930 | 16 | 23197540 | 24560343 | 1.009119184 | 4 | amp |
| TCGA-10-0930 | 16 | 24564795 | 24830027 | 0.79523752 | 3 | amp |
| TCGA-10-0930 | 16 | 24831450 | 30391363 | 1.018159601 | 4 | amp |
| TCGA-10-0930 | 16 | 30392386 | 30525189 | 1.22518029 | 5 | amp |
| TCGA-10-0930 | 16 | 30528260 | 32177057 | 1.039902855 | 4 | amp |
| TCGA-10-0930 | 16 | 32177931 | 32772334 | 0.81466909 | 3 | amp |
| TCGA-10-0930 | 16 | 32773208 | 34339128 | 0.944233138 | 4 | amp |
| TCGA-10-0930 | 16 | 46615623 | 46727109 | 0.783693816 | 3 | amp |
| TCGA-10-0930 | 16 | 46729439 | 46993098 | 0.973331088 | 4 | amp |
| TCGA-10-0930 | 16 | 46993138 | 47733328 | 0.771399351 | 3 | amp |
| TCGA-10-0930 | 16 | 48117578 | 48286278 | 1.066975341 | 4 | amp |
| TCGA-10-0930 | 16 | 48290466 | 49313533 | 0.770512014 | 3 | amp |
| TCGA-10-0930 | 16 | 49314773 | 50348319 | 0.975571409 | 4 | amp |
| TCGA-10-0930 | 16 | 50348836 | 50402211 | 0.709526628 | 3 | amp |

| | | | | | | |
|---|---|---|---|---|---|---|
| TCGA-10-0930 | 16 | 50642177 | 50765753 | 1.337522428 | 5 | amp |
| TCGA-10-0930 | 16 | 50783561 | 51171482 | 1.014743242 | 4 | amp |
| TCGA-10-0930 | 16 | 51172558 | 52690011 | 1.224366391 | 5 | amp |
| TCGA-10-0930 | 16 | 53734532 | 54145870 | 0.802460224 | 3 | amp |
| TCGA-10-0930 | 16 | 54317564 | 55527257 | 0.991581415 | 4 | amp |
| TCGA-10-0930 | 16 | 55530785 | 55617037 | 0.6960017 | 3 | amp |
| TCGA-10-0930 | 16 | 55690533 | 56492521 | 1.010169632 | 4 | amp |
| TCGA-10-0930 | 16 | 56496435 | 56672724 | 0.806568078 | 3 | amp |
| TCGA-10-0930 | 16 | 56673118 | 56873534 | 0.929168999 | 4 | amp |
| TCGA-10-0930 | 16 | 56875560 | 56947366 | 1.168675755 | 5 | amp |
| TCGA-10-0930 | 16 | 56969125 | 57007452 | 0.956774575 | 4 | amp |
| TCGA-10-0930 | 16 | 57008947 | 57161847 | 1.153031895 | 5 | amp |
| TCGA-10-0930 | 16 | 57168622 | 57935540 | 0.981460114 | 4 | amp |
| TCGA-10-0930 | 16 | 57937655 | 58011950 | 1.20637317 | 5 | amp |
| TCGA-10-0930 | 16 | 58018177 | 58552960 | 0.972979569 | 4 | amp |
| TCGA-10-0930 | 16 | 58554810 | 58614720 | 0.735929004 | 3 | amp |
| TCGA-10-0930 | 16 | 58615213 | 58757847 | 0.972050031 | 4 | amp |
| TCGA-10-0930 | 16 | 61687458 | 69458793 | 0.788007142 | 3 | amp |
| TCGA-10-0930 | 16 | 69746892 | 71698021 | 0.770212764 | 3 | amp |
| TCGA-10-0930 | 16 | 71954623 | 75327954 | 0.745642495 | 3 | amp |
| TCGA-10-0930 | 16 | 81058291 | 90244214 | 0.769456888 | 3 | amp |
| TCGA-10-0930 | 17 | 63618 | 3917492 | 0.821878629 | 3 | amp |
| TCGA-10-0930 | 17 | 4086619 | 5045804 | 0.839776815 | 3 | amp |
| TCGA-10-0930 | 17 | 5386105 | 6493330 | 0.814228927 | 3 | amp |
| TCGA-10-0930 | 17 | 6545053 | 7577206 | 0.815660374 | 3 | amp |
| TCGA-10-0930 | 17 | 7577493 | 7736893 | 0.950469657 | 4 | amp |
| TCGA-10-0930 | 17 | 7748820 | 7803402 | 0.814925307 | 3 | amp |
| TCGA-10-0930 | 17 | 12847329 | 12909326 | 0.978880571 | 4 | amp |
| TCGA-10-0930 | 17 | 16703237 | 18397728 | 0.801250089 | 3 | amp |
| TCGA-10-0930 | 17 | 18541171 | 19689444 | 0.787729578 | 3 | amp |
| TCGA-10-0930 | 17 | 20353238 | 26512307 | 0.808237998 | 3 | amp |
| TCGA-10-0930 | 17 | 26518025 | 26907142 | 1.016077139 | 4 | amp |
| TCGA-10-0930 | 17 | 26910477 | 26913539 | 1.311999418 | 5 | amp |
| TCGA-10-0930 | 17 | 26918652 | 26982522 | 0.971522931 | 4 | amp |
| TCGA-10-0930 | 17 | 26988668 | 27426835 | 1.118649666 | 5 | amp |
| TCGA-10-0930 | 17 | 27430589 | 27963822 | 0.961938689 | 4 | amp |
| TCGA-10-0930 | 17 | 27975141 | 28378245 | 0.74231051 | 3 | amp |
| TCGA-10-0930 | 17 | 29758784 | 30498137 | 0.751663528 | 3 | amp |
| TCGA-10-0930 | 17 | 30594872 | 35512713 | 0.817419953 | 3 | amp |
| TCGA-10-0930 | 17 | 35986779 | 38715229 | 0.837529795 | 3 | amp |
| TCGA-10-0930 | 17 | 39150020 | 39847115 | 0.826812336 | 3 | amp |
| TCGA-10-0930 | 17 | 39871643 | 40107344 | 0.93635647 | 4 | amp |
| TCGA-10-0930 | 17 | 40117075 | 45387597 | 0.821576374 | 3 | amp |
| TCGA-10-0930 | 17 | 45560347 | 49076011 | 0.801316332 | 3 | amp |
| TCGA-10-0930 | 17 | 53342842 | 57060340 | 0.793468976 | 3 | amp |
| TCGA-10-0930 | 17 | 58054014 | 58236807 | 0.79191369 | 3 | amp |
| TCGA-10-0930 | 17 | 59946627 | 61483696 | 0.873680039 | 3 | amp |
| TCGA-10-0930 | 17 | 61488839 | 61903005 | 0.994225387 | 4 | amp |
| TCGA-10-0930 | 17 | 61903395 | 62158211 | 1.265533098 | 5 | amp |
| TCGA-10-0930 | 17 | 62175420 | 63637192 | 0.959808138 | 4 | amp |

| | | | | | | |
|---|---|---|---|---|---|---|
| TCGA-10-0930 | 17 | 63685231 | 64179424 | 0.782499782 | 3 | amp |
| TCGA-10-0930 | 17 | 64208218 | 65105816 | 1.027500878 | 4 | amp |
| TCGA-10-0930 | 17 | 65110389 | 65940496 | 0.84545277 | 3 | amp |
| TCGA-10-0930 | 17 | 65941468 | 66519971 | 1.013104961 | 4 | amp |
| TCGA-10-0930 | 17 | 66520097 | 70120573 | 0.785230138 | 3 | amp |
| TCGA-10-0930 | 17 | 70643701 | 72240211 | 0.957599144 | 4 | amp |
| TCGA-10-0930 | 17 | 72245094 | 73486870 | 1.140589504 | 5 | amp |
| TCGA-10-0930 | 17 | 73487121 | 73493296 | 0.984334438 | 4 | amp |
| TCGA-10-0930 | 17 | 73493842 | 73847762 | 1.128842162 | 5 | amp |
| TCGA-10-0930 | 17 | 73851258 | 76113448 | 1.053949404 | 4 | amp |
| TCGA-10-0930 | 17 | 76115335 | 76692149 | 1.17459069 | 5 | amp |
| TCGA-10-0930 | 17 | 76694346 | 81188237 | 0.986960147 | 4 | amp |
| TCGA-10-0930 | 18 | 3277728 | 5892093 | 0.717803157 | 3 | amp |
| TCGA-10-0930 | 18 | 5956152 | 6462950 | 0.989814014 | 4 | amp |
| TCGA-10-0930 | 18 | 6837290 | 7955445 | 1.151066692 | 5 | amp |
| TCGA-10-0930 | 18 | 8069657 | 20596898 | 0.944700491 | 4 | amp |
| TCGA-10-0930 | 18 | 20602042 | 21390518 | 1.098599934 | 5 | amp |
| TCGA-10-0930 | 18 | 21392983 | 29225499 | 0.9629754 | 4 | amp |
| TCGA-10-0930 | 18 | 71958925 | 72223756 | 1.095832012 | 4 | amp |
| TCGA-10-0930 | 19 | 71882 | 3382275 | 0.981700333 | 4 | amp |
| TCGA-10-0930 | 19 | 3425049 | 3982057 | 1.517829768 | 6 | amp |
| TCGA-10-0930 | 19 | 3982182 | 4355191 | 1.174526081 | 5 | amp |
| TCGA-10-0930 | 19 | 4357183 | 5729978 | 1.048142532 | 4 | amp |
| TCGA-10-0930 | 19 | 5733863 | 5771138 | 1.284484272 | 5 | amp |
| TCGA-10-0930 | 19 | 5772737 | 8464964 | 1.030861241 | 4 | amp |
| TCGA-10-0930 | 19 | 8654037 | 8933379 | 0.850004496 | 3 | amp |
| TCGA-10-0930 | 19 | 8953316 | 8987391 | 1.083907181 | 4 | amp |
| TCGA-10-0930 | 19 | 8992964 | 8998790 | 1.285008667 | 5 | amp |
| TCGA-10-0930 | 19 | 8998982 | 9006823 | 1.485453311 | 6 | amp |
| TCGA-10-0930 | 19 | 9007459 | 9018618 | 1.199333569 | 5 | amp |
| TCGA-10-0930 | 19 | 9019247 | 9362769 | 1.038572088 | 4 | amp |
| TCGA-10-0930 | 19 | 9406099 | 9585057 | 0.841649702 | 3 | amp |
| TCGA-10-0930 | 19 | 9585914 | 10102550 | 1.023342317 | 4 | amp |
| TCGA-10-0930 | 19 | 10102641 | 10166472 | 1.176371639 | 5 | amp |
| TCGA-10-0930 | 19 | 10169198 | 10260646 | 0.982971336 | 4 | amp |
| TCGA-10-0930 | 19 | 10262027 | 10279082 | 1.262020648 | 5 | amp |
| TCGA-10-0930 | 19 | 10283717 | 11892740 | 1.060664008 | 4 | amp |
| TCGA-10-0930 | 19 | 11915275 | 12639207 | 0.866972373 | 3 | amp |
| TCGA-10-0930 | 19 | 12639326 | 13414699 | 1.073338295 | 4 | amp |
| TCGA-10-0930 | 19 | 13418571 | 13476326 | 1.252303036 | 5 | amp |
| TCGA-10-0930 | 19 | 13482487 | 14705651 | 1.068426559 | 4 | amp |
| TCGA-10-0930 | 19 | 14706046 | 14758204 | 0.86414262 | 3 | amp |
| TCGA-10-0930 | 19 | 14761836 | 15640711 | 1.033423319 | 4 | amp |
| TCGA-10-0930 | 19 | 15648108 | 15660040 | 1.281405831 | 5 | amp |
| TCGA-10-0930 | 19 | 15661465 | 16263466 | 0.985229813 | 4 | amp |
| TCGA-10-0930 | 19 | 16268012 | 16280556 | 1.410998686 | 5 | amp |
| TCGA-10-0930 | 19 | 16283899 | 16551756 | 1.103399146 | 4 | amp |
| TCGA-10-0930 | 19 | 16552657 | 16620854 | 1.264763273 | 5 | amp |
| TCGA-10-0930 | 19 | 16623812 | 17783305 | 1.074918149 | 4 | amp |
| TCGA-10-0930 | 19 | 17785455 | 17881429 | 1.149934368 | 5 | amp |

| | | | | | | |
|---|---|---|---|---|---|---|
| TCGA-10-0930 | 19 | 17881562 | 19260256 | 1.066882774 | 4 | amp |
| TCGA-10-0930 | 19 | 19261457 | 19313391 | 1.322547749 | 5 | amp |
| TCGA-10-0930 | 19 | 19313584 | 19791059 | 1.057377749 | 4 | amp |
| TCGA-10-0930 | 19 | 19822129 | 30102867 | 0.808585182 | 3 | amp |
| TCGA-10-0930 | 19 | 30102964 | 30311801 | 0.999849076 | 4 | amp |
| TCGA-10-0930 | 19 | 30312616 | 30314742 | 1.220855879 | 5 | amp |
| TCGA-10-0930 | 19 | 30934384 | 31770676 | 1.228791311 | 5 | amp |
| TCGA-10-0930 | 19 | 32843719 | 33093036 | 0.76277122 | 3 | amp |
| TCGA-10-0930 | 19 | 33095129 | 33462820 | 1.027775838 | 4 | amp |
| TCGA-10-0930 | 19 | 33464095 | 33585186 | 1.284609997 | 5 | amp |
| TCGA-10-0930 | 19 | 33586636 | 34870504 | 1.008991084 | 4 | amp |
| TCGA-10-0930 | 19 | 34872337 | 35451954 | 0.867968009 | 3 | amp |
| TCGA-10-0930 | 19 | 35499977 | 35804394 | 1.019596028 | 4 | amp |
| TCGA-10-0930 | 19 | 35822870 | 36000897 | 1.166786901 | 5 | amp |
| TCGA-10-0930 | 19 | 36001029 | 36728261 | 1.027271002 | 4 | amp |
| TCGA-10-0930 | 19 | 36831088 | 38579545 | 0.880381224 | 3 | amp |
| TCGA-10-0930 | 19 | 38590575 | 40098040 | 1.03783179 | 4 | amp |
| TCGA-10-0930 | 19 | 40195115 | 40331257 | 0.824376549 | 3 | amp |
| TCGA-10-0930 | 19 | 40331343 | 43530552 | 1.002102319 | 4 | amp |
| TCGA-10-0930 | 19 | 43570640 | 43780585 | 0.84071869 | 3 | amp |
| TCGA-10-0930 | 19 | 43857832 | 44341387 | 1.033674599 | 4 | amp |
| TCGA-10-0930 | 19 | 44351041 | 45017349 | 0.863469985 | 3 | amp |
| TCGA-10-0930 | 19 | 45020965 | 49388820 | 1.027836643 | 4 | amp |
| TCGA-10-0930 | 19 | 49391182 | 49416835 | 1.250875102 | 5 | amp |
| TCGA-10-0930 | 19 | 49421921 | 51586051 | 1.025454864 | 4 | amp |
| TCGA-10-0930 | 19 | 51628201 | 51771897 | 1.178632906 | 5 | amp |
| TCGA-10-0930 | 19 | 51825296 | 52217377 | 1.020107215 | 4 | amp |
| TCGA-10-0930 | 19 | 52219454 | 52852516 | 0.833638751 | 3 | amp |
| TCGA-10-0930 | 19 | 52856877 | 54210809 | 0.972475487 | 4 | amp |
| TCGA-10-0930 | 19 | 54211978 | 54245869 | 1.216691118 | 5 | amp |
| TCGA-10-0930 | 19 | 54251842 | 54396690 | 1.062339983 | 4 | amp |
| TCGA-10-0930 | 19 | 54401075 | 54406426 | 0.797093637 | 3 | amp |
| TCGA-10-0930 | 19 | 54407882 | 55294484 | 1.027620091 | 4 | amp |
| TCGA-10-0930 | 19 | 55294903 | 55325611 | 1.362286861 | 5 | amp |
| TCGA-10-0930 | 19 | 55327912 | 56901477 | 1.019974718 | 4 | amp |
| TCGA-10-0930 | 19 | 56901656 | 57647474 | 0.837465999 | 3 | amp |
| TCGA-10-0930 | 19 | 57648229 | 58005500 | 0.968460738 | 4 | amp |
| TCGA-10-0930 | 19 | 58015969 | 58153590 | 0.838053245 | 3 | amp |
| TCGA-10-0930 | 19 | 58187471 | 59023271 | 0.990202162 | 4 | amp |
| TCGA-10-0930 | 19 | 59027740 | 59063199 | 0.774540203 | 3 | amp |
| TCGA-10-0930 | 2 | 41527 | 7023702 | 0.88217221 | 4 | amp |
| TCGA-10-0930 | 2 | 7027060 | 16747094 | 0.825600974 | 3 | amp |
| TCGA-10-0930 | 2 | 16769292 | 20647821 | 1.007409629 | 4 | amp |
| TCGA-10-0930 | 2 | 20729292 | 26512863 | 1.243580261 | 5 | amp |
| TCGA-10-0930 | 2 | 26531904 | 27686065 | 1.478000854 | 6 | amp |
| TCGA-10-0930 | 2 | 27688271 | 28762065 | 1.299855592 | 5 | amp |
| TCGA-10-0930 | 2 | 28763180 | 28865945 | 1.5195202 | 6 | amp |
| TCGA-10-0930 | 2 | 28999662 | 29164443 | 1.178631238 | 5 | amp |
| TCGA-10-0930 | 2 | 29165152 | 29297153 | 1.624116121 | 6 | amp |
| TCGA-10-0930 | 2 | 29344200 | 29420577 | 0.949266401 | 4 | amp |

| | | | | | | |
|---|---|---|---|---|---|---|
| TCGA-10-0930 | 2 | 29429997 | 32605409 | 1.700117535 | 6 | amp |
| TCGA-10-0930 | 2 | 32613794 | 32750140 | 1.286816035 | 5 | amp |
| TCGA-10-0930 | 2 | 32750492 | 51149870 | 1.54194448 | 6 | amp |
| TCGA-10-0930 | 2 | 51150487 | 68374708 | 1.083874624 | 4 | amp |
| TCGA-10-0930 | 2 | 68384333 | 71709114 | 1.241676649 | 5 | amp |
| TCGA-10-0930 | 2 | 71730306 | 72359791 | 1.520795181 | 6 | amp |
| TCGA-10-0930 | 2 | 72360113 | 74450096 | 1.332160249 | 5 | amp |
| TCGA-10-0930 | 2 | 74451894 | 75094956 | 1.478324285 | 6 | amp |
| TCGA-10-0930 | 2 | 75099410 | 85051382 | 1.233840192 | 5 | amp |
| TCGA-10-0930 | 2 | 85059163 | 86333482 | 1.548976248 | 6 | amp |
| TCGA-10-0930 | 2 | 86335375 | 95847890 | 1.196923059 | 5 | amp |
| TCGA-10-0930 | 2 | 95940275 | 96078528 | 1.640153764 | 6 | amp |
| TCGA-10-0930 | 2 | 96078613 | 102490748 | 1.257436248 | 5 | amp |
| TCGA-10-0930 | 2 | 102493416 | 113518375 | 1.031246114 | 4 | amp |
| TCGA-10-0930 | 2 | 113520052 | 114164060 | 1.246290663 | 5 | amp |
| TCGA-10-0930 | 2 | 114195402 | 128281369 | 1.066330638 | 4 | amp |
| TCGA-10-0930 | 2 | 128317246 | 130834834 | 1.31878236 | 5 | amp |
| TCGA-10-0930 | 2 | 130843349 | 135966552 | 1.067600736 | 4 | amp |
| TCGA-10-0930 | 2 | 135974985 | 138739000 | 1.217821243 | 5 | amp |
| TCGA-10-0930 | 2 | 138758451 | 160085416 | 0.995144374 | 4 | amp |
| TCGA-10-0930 | 2 | 160086085 | 243160772 | 0.705470039 | 3 | amp |
| TCGA-10-0930 | 20 | 68319 | 13415805 | 0.735498407 | 3 | amp |
| TCGA-10-0930 | 20 | 29845453 | 30070310 | 0.965677101 | 4 | amp |
| TCGA-10-0930 | 20 | 30115245 | 30147491 | 1.878294431 | 6 | amp |
| TCGA-10-0930 | 20 | 30149427 | 31044239 | 1.256903561 | 5 | amp |
| TCGA-10-0930 | 20 | 31062397 | 31946948 | 1.640818996 | 6 | amp |
| TCGA-10-0930 | 20 | 31948106 | 34761907 | 1.297237906 | 5 | amp |
| TCGA-10-0930 | 20 | 34763404 | 34843703 | 0.679237068 | 3 | amp |
| TCGA-10-0930 | 20 | 35060049 | 35155444 | 1.029475167 | 4 | amp |
| TCGA-10-0930 | 20 | 35173229 | 36624880 | 1.288482813 | 5 | amp |
| TCGA-10-0930 | 20 | 36625102 | 37078199 | 1.019774194 | 4 | amp |
| TCGA-10-0930 | 20 | 37117048 | 40162248 | 0.866516441 | 3 | amp |
| TCGA-10-0930 | 20 | 40179899 | 42907948 | 1.040213277 | 4 | amp |
| TCGA-10-0930 | 20 | 42935212 | 44421410 | 1.384405108 | 5 | amp |
| TCGA-10-0930 | 20 | 44422547 | 44434034 | 2.241793942 | 6 | amp |
| TCGA-10-0930 | 20 | 44437728 | 45797890 | 1.275120209 | 5 | amp |
| TCGA-10-0930 | 20 | 45801284 | 48732020 | 1.125444124 | 4 | amp |
| TCGA-10-0930 | 20 | 48741505 | 49493203 | 1.58540861 | 6 | amp |
| TCGA-10-0930 | 20 | 49507935 | 52570282 | 1.27114394 | 5 | amp |
| TCGA-10-0930 | 20 | 52573943 | 56098348 | 1.040422655 | 4 | amp |
| TCGA-10-0930 | 20 | 56098689 | 56190119 | 1.509171294 | 6 | amp |
| TCGA-10-0930 | 20 | 56190541 | 58425556 | 0.954306363 | 4 | amp |
| TCGA-10-0930 | 20 | 58439344 | 58559868 | 0.737340239 | 3 | amp |
| TCGA-10-0930 | 20 | 58559999 | 62926333 | 1.094572781 | 4 | amp |
| TCGA-10-0930 | 21 | 46011216 | 46047412 | 0.610359626 | 3 | amp |
| TCGA-10-0930 | 22 | 16084594 | 17119669 | 0.937991144 | 4 | amp |
| TCGA-10-0930 | 22 | 17170961 | 23223609 | 1.245143373 | 5 | amp |
| TCGA-10-0930 | 22 | 23235919 | 23401947 | 1.780673802 | 6 | amp |
| TCGA-10-0930 | 22 | 23403904 | 29730446 | 1.256453927 | 5 | amp |
| TCGA-10-0930 | 22 | 29734900 | 30421824 | 1.50879658 | 6 | amp |

| | | | | | | |
|---|---|---|---|---|---|---|
| TCGA-10-0930 | 22 | 30489906 | 32853431 | 1.252819285 | 5 | amp |
| TCGA-10-0930 | 22 | 32874934 | 38481775 | 1.088739484 | 4 | amp |
| TCGA-10-0930 | 22 | 38483186 | 39358220 | 0.796592771 | 3 | amp |
| TCGA-10-0930 | 22 | 39358446 | 39448760 | 1.079810764 | 4 | amp |
| TCGA-10-0930 | 22 | 39473314 | 51237627 | 0.73216508 | 3 | amp |
| TCGA-10-0930 | 3 | 361444 | 12232177 | 2.131240118 | 6 | amp |
| TCGA-10-0930 | 3 | 12393064 | 15687008 | 1.401180398 | 5 | amp |
| TCGA-10-0930 | 3 | 15711739 | 32586602 | 1.059159786 | 4 | amp |
| TCGA-10-0930 | 3 | 32587278 | 38307780 | 0.840263354 | 3 | amp |
| TCGA-10-0930 | 3 | 38315754 | 45877298 | 1.032821003 | 4 | amp |
| TCGA-10-0930 | 3 | 45879360 | 47044851 | 1.23754498 | 5 | amp |
| TCGA-10-0930 | 3 | 47045265 | 48369857 | 1.117106705 | 4 | amp |
| TCGA-10-0930 | 3 | 48414212 | 48602647 | 1.354747664 | 5 | amp |
| TCGA-10-0930 | 3 | 48602825 | 48628297 | 1.791892597 | 6 | amp |
| TCGA-10-0930 | 3 | 48628840 | 52598277 | 1.33720515 | 5 | amp |
| TCGA-10-0930 | 3 | 52610525 | 52800016 | 1.049661388 | 4 | amp |
| TCGA-10-0930 | 3 | 52800172 | 53886160 | 1.262899449 | 5 | amp |
| TCGA-10-0930 | 3 | 53886840 | 108822789 | 1.073939393 | 4 | amp |
| TCGA-10-0930 | 3 | 108829552 | 111342694 | 1.363624768 | 5 | amp |
| TCGA-10-0930 | 3 | 111343155 | 122634497 | 1.005973548 | 4 | amp |
| TCGA-10-0930 | 3 | 122634528 | 124732881 | 1.210588176 | 5 | amp |
| TCGA-10-0930 | 3 | 124738093 | 125509289 | 1.047219652 | 4 | amp |
| TCGA-10-0930 | 3 | 125509339 | 129304979 | 1.274495695 | 5 | amp |
| TCGA-10-0930 | 3 | 129370270 | 130399570 | 1.131361372 | 4 | amp |
| TCGA-10-0930 | 3 | 130399804 | 132418953 | 0.912427297 | 3 | amp |
| TCGA-10-0930 | 3 | 132419174 | 141934547 | 1.020864951 | 4 | amp |
| TCGA-10-0930 | 3 | 142030323 | 142537361 | 0.876749184 | 3 | amp |
| TCGA-10-0930 | 3 | 142539653 | 145828273 | 1.004717323 | 4 | amp |
| TCGA-10-0930 | 3 | 145838846 | 160951316 | 1.27582082 | 5 | amp |
| TCGA-10-0930 | 3 | 160952458 | 167078546 | 1.079630903 | 4 | amp |
| TCGA-10-0930 | 3 | 167083658 | 175473226 | 1.302386122 | 5 | amp |
| TCGA-10-0930 | 3 | 175520771 | 183683068 | 0.79287173 | 3 | amp |
| TCGA-10-0930 | 3 | 183683106 | 184298417 | 1.023107694 | 4 | amp |
| TCGA-10-0930 | 3 | 184298441 | 191888465 | 0.866753693 | 3 | amp |
| TCGA-10-0930 | 3 | 192053141 | 192053261 | 1.124856026 | 4 | amp |
| TCGA-10-0930 | 3 | 192078211 | 193082102 | 1.460031651 | 5 | amp |
| TCGA-10-0930 | 3 | 193096392 | 193184012 | 1.744889614 | 6 | amp |
| TCGA-10-0930 | 3 | 193185039 | 194181593 | 1.425025431 | 5 | amp |
| TCGA-10-0930 | 3 | 194182833 | 196771606 | 1.733419622 | 6 | amp |
| TCGA-10-0930 | 3 | 196778433 | 197009752 | 1.428809444 | 5 | amp |
| TCGA-10-0930 | 3 | 197023132 | 197955154 | 1.6354787 | 6 | amp |
| TCGA-10-0930 | 4 | 955729 | 1920408 | 0.656837113 | 3 | amp |
| TCGA-10-0930 | 4 | 3174025 | 6607098 | 0.671864956 | 3 | amp |
| TCGA-10-0930 | 4 | 6995849 | 13339015 | 0.701412154 | 3 | amp |
| TCGA-10-0930 | 4 | 69403302 | 69434260 | 0.81815181 | 3 | amp |
| TCGA-10-0930 | 4 | 76731578 | 76871986 | 0.673705195 | 3 | amp |
| TCGA-10-0930 | 4 | 160189277 | 164428300 | 0.876173442 | 4 | amp |
| TCGA-10-0930 | 4 | 164433902 | 164440695 | 0.668981285 | 3 | amp |
| TCGA-10-0930 | 4 | 164449868 | 166795260 | 0.904678303 | 4 | amp |
| TCGA-10-0930 | 4 | 166910527 | 167810397 | 0.776278324 | 3 | amp |

| | | | | | | |
|---|---|---|---|---|---|---|
| TCGA-10-0930 | 4 | 167833712 | 169108628 | 0.870354279 | 4 | amp |
| TCGA-10-0930 | 4 | 169138073 | 170634457 | 0.824146661 | 3 | amp |
| TCGA-10-0930 | 4 | 170638890 | 170927059 | 0.907802423 | 4 | amp |
| TCGA-10-0930 | 4 | 170983033 | 171009750 | 0.755227354 | 3 | amp |
| TCGA-10-0930 | 4 | 190947822 | 191013488 | 0.700238362 | 3 | amp |
| TCGA-10-0930 | 5 | 151610 | 35814670 | 1.82536601 | 6 | amp |
| TCGA-10-0930 | 5 | 35857076 | 56152621 | 1.070984322 | 4 | amp |
| TCGA-10-0930 | 5 | 56155521 | 68400560 | 0.871891684 | 3 | amp |
| TCGA-10-0930 | 5 | 68404159 | 70757788 | 1.035734001 | 4 | amp |
| TCGA-10-0930 | 5 | 70759875 | 72147168 | 0.91358186 | 3 | amp |
| TCGA-10-0930 | 5 | 96253124 | 99921946 | 0.888141261 | 3 | amp |
| TCGA-10-0930 | 5 | 100147511 | 127700475 | 1.016372213 | 4 | amp |
| TCGA-10-0930 | 5 | 127702036 | 180899507 | 0.82487469 | 3 | amp |
| TCGA-10-0930 | 6 | 105907 | 7891971 | 1.264022692 | 5 | amp |
| TCGA-10-0930 | 6 | 7895296 | 31609757 | 0.801242119 | 3 | amp |
| TCGA-10-0930 | 6 | 31609824 | 32151549 | 0.993202243 | 4 | amp |
| TCGA-10-0930 | 6 | 32151620 | 33095794 | 0.855654284 | 3 | amp |
| TCGA-10-0930 | 6 | 33131374 | 33665538 | 1.066012773 | 4 | amp |
| TCGA-10-0930 | 6 | 33668165 | 39884013 | 0.846917364 | 3 | amp |
| TCGA-10-0930 | 6 | 40359626 | 44414455 | 2.689591031 | 6 | amp |
| TCGA-10-0930 | 6 | 44797483 | 54013949 | 0.967580471 | 4 | amp |
| TCGA-10-0930 | 6 | 54025137 | 99328524 | 0.691154031 | 3 | amp |
| TCGA-10-0930 | 6 | 99347130 | 111303380 | 1.077637068 | 4 | amp |
| TCGA-10-0930 | 6 | 111306124 | 158318064 | 0.860615495 | 3 | amp |
| TCGA-10-0930 | 6 | 158322883 | 168264524 | 1.108915338 | 5 | amp |
| TCGA-10-0930 | 6 | 168264952 | 171055029 | 0.903678374 | 3 | amp |
| TCGA-10-0930 | 7 | 540048 | 5958574 | 0.941505936 | 4 | amp |
| TCGA-10-0930 | 7 | 5959418 | 5997718 | 1.231454928 | 5 | amp |
| TCGA-10-0930 | 7 | 5998606 | 11101771 | 0.885919226 | 4 | amp |
| TCGA-10-0930 | 7 | 11110833 | 20662994 | 0.727843752 | 3 | amp |
| TCGA-10-0930 | 7 | 20666146 | 27566031 | 1.164224399 | 5 | amp |
| TCGA-10-0930 | 7 | 27570768 | 28031623 | 0.900965181 | 4 | amp |
| TCGA-10-0930 | 7 | 28452474 | 31146323 | 1.161299063 | 5 | amp |
| TCGA-10-0930 | 7 | 31377848 | 39500349 | 0.94463102 | 4 | amp |
| TCGA-10-0930 | 7 | 39503732 | 40314303 | 1.267206772 | 5 | amp |
| TCGA-10-0930 | 7 | 40356346 | 45140034 | 1.608038673 | 6 | amp |
| TCGA-10-0930 | 7 | 45140823 | 50672100 | 1.117889025 | 5 | amp |
| TCGA-10-0930 | 7 | 50672894 | 51258780 | 1.017970196 | 4 | amp |
| TCGA-10-0930 | 7 | 51261060 | 57522886 | 0.698392612 | 3 | amp |
| TCGA-10-0930 | 7 | 62708567 | 65751716 | 0.871626888 | 4 | amp |
| TCGA-10-0930 | 7 | 65817426 | 66582657 | 1.238120456 | 5 | amp |
| TCGA-10-0930 | 7 | 66648107 | 73469132 | 0.987457575 | 4 | amp |
| TCGA-10-0930 | 7 | 73470562 | 76659870 | 1.146337266 | 5 | amp |
| TCGA-10-0930 | 7 | 76669217 | 76888408 | 0.944725256 | 4 | amp |
| TCGA-10-0930 | 7 | 76889319 | 97498523 | 0.690349063 | 3 | amp |
| TCGA-10-0930 | 7 | 97535388 | 100320496 | 0.946680141 | 4 | amp |
| TCGA-10-0930 | 7 | 100320528 | 100866857 | 1.136086339 | 5 | amp |
| TCGA-10-0930 | 7 | 100866888 | 102343959 | 0.9599141 | 4 | amp |
| TCGA-10-0930 | 7 | 102453763 | 127017431 | 0.728536568 | 3 | amp |
| TCGA-10-0930 | 7 | 127026101 | 135386527 | 0.880896425 | 4 | amp |

| | | | | | | |
|---|---|---|---|---|---|---|
| TCGA-10-0930 | 7 | 135387520 | 138819589 | 1.099059089 | 5 | amp |
| TCGA-10-0930 | 7 | 138822578 | 139097401 | 0.941451181 | 4 | amp |
| TCGA-10-0930 | 7 | 139102229 | 140398094 | 1.110911343 | 5 | amp |
| TCGA-10-0930 | 7 | 140402587 | 142000904 | 0.978651124 | 4 | amp |
| TCGA-10-0930 | 7 | 142000926 | 150418054 | 1.195993733 | 5 | amp |
| TCGA-10-0930 | 7 | 150437903 | 151164310 | 1.498778412 | 6 | amp |
| TCGA-10-0930 | 7 | 151167637 | 151875099 | 1.20570439 | 5 | amp |
| TCGA-10-0930 | 7 | 151876884 | 152055775 | 0.922183076 | 4 | amp |
| TCGA-10-0930 | 7 | 152142528 | 158935247 | 1.165001226 | 5 | amp |
| TCGA-10-0930 | 8 | 116074 | 7056187 | 0.760662983 | 3 | amp |
| TCGA-10-0930 | 8 | 7116415 | 8865679 | 1.034924784 | 4 | amp |
| TCGA-10-0930 | 8 | 8869006 | 33406387 | 0.730606858 | 3 | amp |
| TCGA-10-0930 | 8 | 33406878 | 36767060 | 1.003755374 | 4 | amp |
| TCGA-10-0930 | 8 | 36768422 | 37996423 | 1.185736741 | 5 | amp |
| TCGA-10-0930 | 8 | 37996475 | 77762599 | 0.928471457 | 4 | amp |
| TCGA-10-0930 | 8 | 77763111 | 88249363 | 1.155738839 | 5 | amp |
| TCGA-10-0930 | 8 | 88296854 | 97285662 | 0.887337501 | 4 | amp |
| TCGA-10-0930 | 8 | 97296268 | 126370066 | 1.181286225 | 5 | amp |
| TCGA-10-0930 | 8 | 126378948 | 146279593 | 1.736972574 | 6 | amp |
| TCGA-10-0930 | 9 | 26905491 | 32544361 | 0.788479196 | 3 | amp |
| TCGA-10-0930 | 9 | 32550748 | 42368636 | 0.949089409 | 4 | amp |
| TCGA-10-0930 | 9 | 42371921 | 89763849 | 0.747855283 | 3 | amp |
| TCGA-10-0930 | 9 | 89771564 | 93978424 | 0.916612428 | 4 | amp |
| TCGA-10-0930 | 9 | 93979493 | 95274410 | 0.823782095 | 3 | amp |
| TCGA-10-0930 | 9 | 95276882 | 101832181 | 0.924996895 | 4 | amp |
| TCGA-10-0930 | 9 | 101891079 | 111822742 | 0.810738199 | 3 | amp |
| TCGA-10-0930 | 9 | 111826711 | 112969851 | 0.972538291 | 4 | amp |
| TCGA-10-0930 | 9 | 113006407 | 115478812 | 0.785777116 | 3 | amp |
| TCGA-10-0930 | 9 | 115567031 | 125612123 | 0.929493238 | 4 | amp |
| TCGA-10-0930 | 9 | 125613313 | 125946471 | 0.775011311 | 3 | amp |
| TCGA-10-0930 | 9 | 126125095 | 133995725 | 0.944875745 | 4 | amp |
| TCGA-10-0930 | 9 | 134002858 | 134098360 | 0.691483396 | 3 | amp |
| TCGA-10-0930 | 9 | 134103512 | 135133865 | 0.907894585 | 4 | amp |
| TCGA-10-0930 | 9 | 135139578 | 135562729 | 0.766240437 | 3 | amp |
| TCGA-10-0930 | 9 | 135564209 | 137697093 | 0.953732442 | 4 | amp |
| TCGA-10-0930 | 9 | 137702037 | 139758357 | 0.720699202 | 3 | amp |
| TCGA-10-0930 | 9 | 139793136 | 141071671 | 1.494338324 | 6 | amp |
| TCGA-10-0930 | X | 2632418 | 3592843 | 0.676904647 | 3 | amp |
| TCGA-10-0930 | X | 62857890 | 63579465 | 0.751118569 | 3 | amp |
| TCGA-10-0930 | X | 63615171 | 71694598 | 0.912407073 | 4 | amp |
| TCGA-10-0930 | X | 71708776 | 83360899 | 0.744749767 | 3 | amp |
| TCGA-10-0930 | X | 91873248 | 99411670 | 0.748709911 | 3 | amp |
| TCGA-10-0930 | X | 99551213 | 101091814 | 0.867122209 | 4 | amp |
| TCGA-10-0930 | X | 101092394 | 101097798 | 1.224865602 | 5 | amp |
| TCGA-10-0930 | X | 101138477 | 101562122 | 0.770985566 | 3 | amp |
| TCGA-10-0930 | X | 101571894 | 101625098 | 1.141226831 | 5 | amp |
| TCGA-10-0930 | X | 101634875 | 103031985 | 0.90046362 | 4 | amp |
| TCGA-10-0930 | X | 103040483 | 107018669 | 0.714004402 | 3 | amp |
| TCGA-10-0933 | 1 | 16834 | 9932134 | 1.379727255 | 4 | amp |
| TCGA-10-0933 | 1 | 9990395 | 10478992 | 1.119863053 | 3 | amp |

| | | | | | | |
|---|---|---|---|---|---|---|
| TCGA-10-0933 | 1 | 10479414 | 12302668 | 1.355208223 | 4 | amp |
| TCGA-10-0933 | 1 | 12304277 | 12476916 | 1.047678391 | 3 | amp |
| TCGA-10-0933 | 1 | 12515999 | 19421579 | 1.378361098 | 4 | amp |
| TCGA-10-0933 | 1 | 19422032 | 19567692 | 1.108939426 | 3 | amp |
| TCGA-10-0933 | 1 | 19568752 | 19569022 | 8.76662121 | 5 | amp |
| TCGA-10-0933 | 1 | 19570064 | 21053590 | 1.36805753 | 4 | amp |
| TCGA-10-0933 | 1 | 21071256 | 21329280 | 1.087658559 | 3 | amp |
| TCGA-10-0933 | 1 | 21546415 | 28293722 | 1.346854766 | 4 | amp |
| TCGA-10-0933 | 1 | 28300956 | 29476719 | 1.178320308 | 3 | amp |
| TCGA-10-0933 | 1 | 29481194 | 35470922 | 1.338835275 | 4 | amp |
| TCGA-10-0933 | 1 | 35472505 | 36509158 | 1.107800134 | 3 | amp |
| TCGA-10-0933 | 1 | 36520526 | 39305420 | 1.30842599 | 4 | amp |
| TCGA-10-0933 | 1 | 39311564 | 43909487 | 1.166410134 | 3 | amp |
| TCGA-10-0933 | 1 | 43911549 | 47489660 | 1.24969432 | 4 | amp |
| TCGA-10-0933 | 1 | 47495612 | 54427810 | 1.130982885 | 3 | amp |
| TCGA-10-0933 | 1 | 54427965 | 55609897 | 1.334150879 | 4 | amp |
| TCGA-10-0933 | 1 | 55611666 | 71538002 | 1.02081474 | 3 | amp |
| TCGA-10-0933 | 1 | 110213866 | 110215891 | 4.027249322 | 5 | amp |
| TCGA-10-0933 | 1 | 120377337 | 143721103 | 1.073746529 | 3 | amp |
| TCGA-10-0933 | 1 | 143767300 | 145293614 | 1.622479731 | 4 | amp |
| TCGA-10-0933 | 1 | 145295414 | 145539096 | 1.857241143 | 5 | amp |
| TCGA-10-0933 | 1 | 145539344 | 148255347 | 1.572293824 | 4 | amp |
| TCGA-10-0933 | 1 | 148255383 | 148346789 | 1.778663216 | 5 | amp |
| TCGA-10-0933 | 1 | 148577600 | 150463258 | 1.624436912 | 4 | amp |
| TCGA-10-0933 | 1 | 150463808 | 150483692 | 2.131326601 | 5 | amp |
| TCGA-10-0933 | 1 | 150483849 | 150899328 | 1.571336052 | 4 | amp |
| TCGA-10-0933 | 1 | 150900140 | 153580208 | 1.816198418 | 5 | amp |
| TCGA-10-0933 | 1 | 153580400 | 154110767 | 1.664417778 | 4 | amp |
| TCGA-10-0933 | 1 | 154112232 | 154247973 | 1.922337925 | 5 | amp |
| TCGA-10-0933 | 1 | 154248044 | 155722424 | 1.639955812 | 4 | amp |
| TCGA-10-0933 | 1 | 155722979 | 161010175 | 1.87410368 | 5 | amp |
| TCGA-10-0933 | 1 | 161010322 | 161179743 | 1.560153573 | 4 | amp |
| TCGA-10-0933 | 1 | 161179879 | 161692848 | 1.965889971 | 5 | amp |
| TCGA-10-0933 | 1 | 161693073 | 168344870 | 1.600289878 | 4 | amp |
| TCGA-10-0933 | 1 | 168510153 | 196716461 | 1.924187837 | 5 | amp |
| TCGA-10-0933 | 1 | 196743985 | 196801147 | 1.211365826 | 4 | amp |
| TCGA-10-0933 | 1 | 196857249 | 249231325 | 2.030404623 | 5 | amp |
| TCGA-10-0933 | 10 | 92880 | 47894095 | 1.64007866 | 4 | amp |
| TCGA-10-0933 | 10 | 47894542 | 48255499 | 1.142906855 | 3 | amp |
| TCGA-10-0933 | 10 | 48259458 | 50227877 | 1.520776884 | 4 | amp |
| TCGA-10-0933 | 10 | 50254998 | 50738945 | 0.999090487 | 3 | amp |
| TCGA-10-0933 | 10 | 50740559 | 51707707 | 1.28793081 | 4 | amp |
| TCGA-10-0933 | 10 | 51748467 | 135516111 | 0.960335482 | 3 | amp |
| TCGA-10-0933 | 11 | 86637 | 9838598 | 0.963886637 | 3 | amp |
| TCGA-10-0933 | 11 | 10589976 | 10797183 | 0.945137579 | 3 | amp |
| TCGA-10-0933 | 11 | 10828731 | 11919251 | 0.975310333 | 3 | amp |
| TCGA-10-0933 | 11 | 11985971 | 13402814 | 0.950050144 | 3 | amp |
| TCGA-10-0933 | 11 | 13736021 | 14512227 | 0.939478972 | 3 | amp |
| TCGA-10-0933 | 11 | 14900614 | 17113208 | 0.938832135 | 3 | amp |
| TCGA-10-0933 | 11 | 17333387 | 31086752 | 0.941900183 | 3 | amp |

| | | | | | | |
|---|---|---|---|---|---|---|
| TCGA-10-0933 | 11 | 31811453 | 34988385 | 0.956894194 | 3 | amp |
| TCGA-10-0933 | 11 | 35302330 | 60906314 | 1.02094972 | 3 | amp |
| TCGA-10-0933 | 11 | 60971004 | 61017332 | 1.518930147 | 4 | amp |
| TCGA-10-0933 | 11 | 61017376 | 62012216 | 1.054454039 | 3 | amp |
| TCGA-10-0933 | 11 | 62037625 | 62065156 | 1.358642729 | 4 | amp |
| TCGA-10-0933 | 11 | 62066394 | 82550504 | 1.016328088 | 3 | amp |
| TCGA-10-0933 | 11 | 83962247 | 85630592 | 0.942730287 | 3 | amp |
| TCGA-10-0933 | 11 | 86123394 | 89868027 | 0.920711493 | 3 | amp |
| TCGA-10-0933 | 11 | 92498029 | 105624006 | 0.892240977 | 3 | amp |
| TCGA-10-0933 | 11 | 107427430 | 107833124 | 0.967834894 | 3 | amp |
| TCGA-10-0933 | 11 | 108302458 | 111897027 | 0.899813576 | 3 | amp |
| TCGA-10-0933 | 11 | 112104096 | 114276565 | 0.973342262 | 3 | amp |
| TCGA-10-0933 | 11 | 115085293 | 118359271 | 1.038825546 | 3 | amp |
| TCGA-10-0933 | 11 | 118372359 | 134010668 | 0.987084851 | 3 | amp |
| TCGA-10-0933 | 11 | 134076410 | 134252931 | 0.969519666 | 3 | amp |
| TCGA-10-0933 | 12 | 73256 | 38710900 | 1.762530418 | 5 | amp |
| TCGA-10-0933 | 12 | 38712041 | 133779395 | 0.991068414 | 3 | amp |
| TCGA-10-0933 | 13 | 19240876 | 21995311 | 1.286838429 | 4 | amp |
| TCGA-10-0933 | 13 | 22067379 | 22113538 | 1.068940439 | 3 | amp |
| TCGA-10-0933 | 13 | 22113751 | 24164416 | 1.241461879 | 4 | amp |
| TCGA-10-0933 | 13 | 24167478 | 24321751 | 1.071294748 | 3 | amp |
| TCGA-10-0933 | 13 | 24330660 | 26706638 | 1.323202423 | 4 | amp |
| TCGA-10-0933 | 13 | 26787944 | 26923323 | 0.998831867 | 3 | amp |
| TCGA-10-0933 | 13 | 26927826 | 31711299 | 1.237716741 | 4 | amp |
| TCGA-10-0933 | 13 | 31835030 | 39447094 | 1.256844564 | 4 | amp |
| TCGA-10-0933 | 13 | 39448610 | 39453137 | 1.002631201 | 3 | amp |
| TCGA-10-0933 | 13 | 39454342 | 77632566 | 1.265494059 | 4 | amp |
| TCGA-10-0933 | 13 | 77633619 | 77699656 | 1.089539471 | 3 | amp |
| TCGA-10-0933 | 13 | 77700444 | 77768393 | 1.183660953 | 4 | amp |
| TCGA-10-0933 | 13 | 77779337 | 77818133 | 1.0703074 | 3 | amp |
| TCGA-10-0933 | 13 | 77825267 | 79176065 | 1.261908883 | 4 | amp |
| TCGA-10-0933 | 13 | 79189705 | 79918479 | 1.095533745 | 3 | amp |
| TCGA-10-0933 | 13 | 79918746 | 115091796 | 1.310725015 | 4 | amp |
| TCGA-10-0933 | 14 | 19377543 | 53124740 | 1.331422924 | 4 | amp |
| TCGA-10-0933 | 14 | 53124794 | 101498422 | 0.966558493 | 3 | amp |
| TCGA-10-0933 | 14 | 101506355 | 107283263 | 1.389974734 | 4 | amp |
| TCGA-10-0933 | 15 | 20169886 | 102513951 | 0.952835908 | 3 | amp |
| TCGA-10-0933 | 16 | 66517 | 30712232 | 1.014955655 | 3 | amp |
| TCGA-10-0933 | 16 | 30715346 | 30734596 | 1.238475485 | 4 | amp |
| TCGA-10-0933 | 16 | 30734864 | 70815958 | 1.021514266 | 3 | amp |
| TCGA-10-0933 | 16 | 70816928 | 71052368 | 1.249028256 | 4 | amp |
| TCGA-10-0933 | 16 | 71054028 | 90244214 | 1.008158092 | 3 | amp |
| TCGA-10-0933 | 17 | 63618 | 2405681 | 1.425636047 | 4 | amp |
| TCGA-10-0933 | 17 | 2541538 | 4200120 | 1.021401638 | 3 | amp |
| TCGA-10-0933 | 17 | 4210306 | 21217560 | 0.548334341 | 1 | loss |
| TCGA-10-0933 | 17 | 21318645 | 36287227 | 0.996808929 | 3 | amp |
| TCGA-10-0933 | 17 | 36287649 | 36344976 | 1.330291465 | 4 | amp |
| TCGA-10-0933 | 17 | 36345398 | 44337262 | 1.044102559 | 3 | amp |
| TCGA-10-0933 | 17 | 44372483 | 44630834 | 1.358782855 | 4 | amp |
| TCGA-10-0933 | 17 | 44632484 | 81188237 | 1.008349884 | 3 | amp |

| | | | | | | |
|---|---|---|---|---|---|---|
| TCGA-10-0933 | 18 | 47273 | 49054 | 1.845276678 | 5 | amp |
| TCGA-10-0933 | 18 | 116821 | 6837440 | 1.309748158 | 4 | amp |
| TCGA-10-0933 | 18 | 6851018 | 6997931 | 1.015983151 | 3 | amp |
| TCGA-10-0933 | 18 | 6999420 | 13671976 | 1.240959193 | 4 | amp |
| TCGA-10-0933 | 18 | 13681599 | 77960823 | 0.908136782 | 3 | amp |
| TCGA-10-0933 | 19 | 71882 | 5788448 | 1.055956495 | 3 | amp |
| TCGA-10-0933 | 19 | 5790021 | 11031625 | 1.27632124 | 4 | amp |
| TCGA-10-0933 | 19 | 11031674 | 14030776 | 0.873445343 | 3 | amp |
| TCGA-10-0933 | 19 | 14031365 | 59110878 | 2.048514287 | 5 | amp |
| TCGA-10-0933 | 2 | 41527 | 97297230 | 1.37147772 | 4 | amp |
| TCGA-10-0933 | 2 | 97361213 | 98779496 | 3.594101249 | 5 | amp |
| TCGA-10-0933 | 2 | 98797456 | 98996847 | 1.25820766 | 4 | amp |
| TCGA-10-0933 | 2 | 98999817 | 101881527 | 0.924576568 | 3 | amp |
| TCGA-10-0933 | 2 | 101883119 | 243160772 | 1.33842639 | 4 | amp |
| TCGA-10-0933 | 20 | 68319 | 23360597 | 1.447633967 | 4 | amp |
| TCGA-10-0933 | 20 | 23361841 | 62926333 | 2.623123568 | 5 | amp |
| TCGA-10-0933 | 21 | 9483321 | 9590404 | 1.079122104 | 3 | amp |
| TCGA-10-0933 | 21 | 10996079 | 48111215 | 0.936672366 | 3 | amp |
| TCGA-10-0933 | 22 | 16084594 | 16100588 | 1.239470921 | 4 | amp |
| TCGA-10-0933 | 22 | 16101375 | 16353993 | 1.165122332 | 3 | amp |
| TCGA-10-0933 | 22 | 16414054 | 16449854 | 1.218568512 | 4 | amp |
| TCGA-10-0933 | 22 | 16914013 | 17062226 | 0.899821066 | 3 | amp |
| TCGA-10-0933 | 22 | 17170961 | 17179076 | 0.909345901 | 3 | amp |
| TCGA-10-0933 | 22 | 24509473 | 24509743 | 1.586337645 | 4 | amp |
| TCGA-10-0933 | 22 | 26861408 | 26902937 | 0.456430144 | 1 | loss |
| TCGA-10-0933 | 22 | 40696901 | 51237627 | 0.543468613 | 1 | loss |
| TCGA-10-0933 | 3 | 361444 | 90306488 | 1.029144392 | 3 | amp |
| TCGA-10-0933 | 3 | 93593048 | 197955154 | 2.059363762 | 5 | amp |
| TCGA-10-0933 | 4 | 53323 | 27024679 | 0.937713437 | 3 | amp |
| TCGA-10-0933 | 4 | 27162417 | 44724282 | 0.513469442 | 1 | loss |
| TCGA-10-0933 | 4 | 46042927 | 46264163 | 0.851608842 | 3 | amp |
| TCGA-10-0933 | 4 | 47322124 | 148605163 | 0.890522044 | 3 | amp |
| TCGA-10-0933 | 4 | 148743865 | 190948390 | 0.488655978 | 1 | loss |
| TCGA-10-0933 | 5 | 151610 | 38451608 | 1.727929914 | 5 | amp |
| TCGA-10-0933 | 5 | 38458364 | 45645738 | 1.561138928 | 4 | amp |
| TCGA-10-0933 | 5 | 49695055 | 50059088 | 0.944939627 | 3 | amp |
| TCGA-10-0933 | 5 | 50683273 | 116052262 | 0.481406785 | 1 | loss |
| TCGA-10-0933 | 5 | 118176576 | 180899507 | 0.953712993 | 3 | amp |
| TCGA-10-0933 | 6 | 105907 | 1612372 | 1.486696989 | 4 | amp |
| TCGA-10-0933 | 6 | 1726637 | 66803381 | 1.745224523 | 5 | amp |
| TCGA-10-0933 | 6 | 66803556 | 129837533 | 1.360526085 | 4 | amp |
| TCGA-10-0933 | 6 | 129899613 | 171055029 | 1.068060069 | 3 | amp |
| TCGA-10-0933 | 7 | 540048 | 30963652 | 0.931183364 | 3 | amp |
| TCGA-10-0933 | 7 | 31008439 | 31126194 | 1.206756387 | 4 | amp |
| TCGA-10-0933 | 7 | 31126496 | 72648759 | 0.975398229 | 3 | amp |
| TCGA-10-0933 | 7 | 72657262 | 72685718 | 1.205295419 | 4 | amp |
| TCGA-10-0933 | 7 | 72693953 | 76924201 | 1.02335737 | 3 | amp |
| TCGA-10-0933 | 7 | 94285295 | 101926407 | 1.003777976 | 3 | amp |
| TCGA-10-0933 | 7 | 101952082 | 101989160 | 1.278438484 | 4 | amp |
| TCGA-10-0933 | 7 | 101991150 | 103557636 | 0.987328301 | 3 | amp |

| | | | | | | |
|---|---|---|---|---|---|---|
| TCGA-10-0933 | 7 | 105098184 | 158935247 | 0.934384465 | 3 | amp |
| TCGA-10-0933 | 8 | 116074 | 33827152 | 0.983402382 | 3 | amp |
| TCGA-10-0933 | 8 | 35383169 | 39466659 | 1.356058192 | 4 | amp |
| TCGA-10-0933 | 8 | 39466981 | 42026588 | 1.074761427 | 3 | amp |
| TCGA-10-0933 | 8 | 42033439 | 42914337 | 1.31013653 | 4 | amp |
| TCGA-10-0933 | 8 | 42919240 | 43212089 | 1.087929467 | 3 | amp |
| TCGA-10-0933 | 8 | 47886143 | 146279593 | 1.313709935 | 4 | amp |
| TCGA-10-0933 | 9 | 14753 | 82324635 | 1.343112773 | 4 | amp |
| TCGA-10-0933 | 9 | 82333581 | 136216600 | 0.962873505 | 3 | amp |
| TCGA-10-0933 | 9 | 136216686 | 136219016 | 1.633634482 | 4 | amp |
| TCGA-10-0933 | 9 | 136219261 | 141071671 | 1.029936016 | 3 | amp |
| TCGA-10-0933 | X | 200797 | 155252520 | 0.962002481 | 3 | amp |
| TCGA-10-0935 | 1 | 14642 | 91383719 | 0.909535273 | 3 | amp |
| TCGA-10-0935 | 1 | 91402984 | 93744086 | 1.216230244 | 4 | amp |
| TCGA-10-0935 | 1 | 93812192 | 94517281 | 1.069554452 | 3 | amp |
| TCGA-10-0935 | 1 | 94520648 | 109566175 | 1.299834691 | 4 | amp |
| TCGA-10-0935 | 1 | 109607109 | 109823875 | 1.061357858 | 3 | amp |
| TCGA-10-0935 | 1 | 109824191 | 111785037 | 1.363771763 | 4 | amp |
| TCGA-10-0935 | 1 | 111825155 | 111970390 | 1.723670963 | 5 | amp |
| TCGA-10-0935 | 1 | 111983781 | 113636231 | 1.439983858 | 4 | amp |
| TCGA-10-0935 | 1 | 113636912 | 114281446 | 1.710223324 | 5 | amp |
| TCGA-10-0935 | 1 | 114300288 | 114951390 | 1.483972042 | 4 | amp |
| TCGA-10-0935 | 1 | 114952751 | 115168622 | 1.789468831 | 5 | amp |
| TCGA-10-0935 | 1 | 115215693 | 117663879 | 1.499985412 | 4 | amp |
| TCGA-10-0935 | 1 | 117690221 | 120057282 | 1.761743174 | 5 | amp |
| TCGA-10-0935 | 1 | 120166303 | 120478280 | 1.466331872 | 4 | amp |
| TCGA-10-0935 | 1 | 120479876 | 144917935 | 2.03094896 | 5 | amp |
| TCGA-10-0935 | 1 | 144918791 | 145139472 | 1.418702227 | 4 | amp |
| TCGA-10-0935 | 1 | 145248793 | 145368622 | 2.153100201 | 5 | amp |
| TCGA-10-0935 | 1 | 145414739 | 145684691 | 1.411666102 | 4 | amp |
| TCGA-10-0935 | 1 | 145686973 | 146466179 | 1.740128955 | 5 | amp |
| TCGA-10-0935 | 1 | 146491172 | 146728260 | 1.390692395 | 4 | amp |
| TCGA-10-0935 | 1 | 146731441 | 148890319 | 1.968326382 | 5 | amp |
| TCGA-10-0935 | 1 | 148891515 | 151373892 | 1.414964556 | 4 | amp |
| TCGA-10-0935 | 1 | 151373971 | 151413602 | 1.747669308 | 5 | amp |
| TCGA-10-0935 | 1 | 151414498 | 152883752 | 1.304147133 | 4 | amp |
| TCGA-10-0935 | 1 | 152883870 | 153318682 | 1.719478348 | 5 | amp |
| TCGA-10-0935 | 1 | 153320323 | 153995805 | 1.270649509 | 4 | amp |
| TCGA-10-0935 | 1 | 153997984 | 154062103 | 1.817805799 | 5 | amp |
| TCGA-10-0935 | 1 | 154067371 | 154207788 | 1.486136178 | 4 | amp |
| TCGA-10-0935 | 1 | 154209004 | 154241436 | 1.779399089 | 5 | amp |
| TCGA-10-0935 | 1 | 154242633 | 155309198 | 1.224545673 | 4 | amp |
| TCGA-10-0935 | 1 | 155311689 | 155348376 | 1.963851706 | 5 | amp |
| TCGA-10-0935 | 1 | 155349810 | 156450814 | 1.435351087 | 4 | amp |
| TCGA-10-0935 | 1 | 156452210 | 156568309 | 1.666407255 | 5 | amp |
| TCGA-10-0935 | 1 | 156568738 | 157494387 | 1.3675759 | 4 | amp |
| TCGA-10-0935 | 1 | 157497365 | 157803321 | 1.76561749 | 5 | amp |
| TCGA-10-0935 | 1 | 157804187 | 158154019 | 1.33086136 | 4 | amp |
| TCGA-10-0935 | 1 | 158224428 | 159856509 | 1.741824153 | 5 | amp |
| TCGA-10-0935 | 1 | 159857595 | 160090837 | 1.319393397 | 4 | amp |

| | | | | | | |
|---|---|---|---|---|---|---|
| TCGA-10-0935 | 1 | 160090921 | 160854698 | 1.683320978 | 5 | amp |
| TCGA-10-0935 | 1 | 160865711 | 161179999 | 1.258918954 | 4 | amp |
| TCGA-10-0935 | 1 | 161180076 | 161928459 | 1.652599734 | 5 | amp |
| TCGA-10-0935 | 1 | 161953418 | 162549425 | 1.452022688 | 4 | amp |
| TCGA-10-0935 | 1 | 162551042 | 165651530 | 1.639550887 | 5 | amp |
| TCGA-10-0935 | 1 | 165652176 | 167803321 | 1.436972698 | 4 | amp |
| TCGA-10-0935 | 1 | 167805542 | 167960629 | 1.674406134 | 5 | amp |
| TCGA-10-0935 | 1 | 167962450 | 168282272 | 1.39134472 | 4 | amp |
| TCGA-10-0935 | 1 | 168344720 | 171491416 | 1.728948177 | 5 | amp |
| TCGA-10-0935 | 1 | 171492306 | 172554251 | 1.483227838 | 4 | amp |
| TCGA-10-0935 | 1 | 172554932 | 173780473 | 1.665948158 | 5 | amp |
| TCGA-10-0935 | 1 | 173794310 | 173842805 | 1.376912238 | 4 | amp |
| TCGA-10-0935 | 1 | 173872964 | 174927094 | 1.639396796 | 5 | amp |
| TCGA-10-0935 | 1 | 174973709 | 175106160 | 1.383113017 | 4 | amp |
| TCGA-10-0935 | 1 | 175113484 | 176913210 | 1.731594794 | 5 | amp |
| TCGA-10-0935 | 1 | 176915008 | 180257694 | 1.510210669 | 4 | amp |
| TCGA-10-0935 | 1 | 180283781 | 180805874 | 1.812618776 | 5 | amp |
| TCGA-10-0935 | 1 | 180832806 | 182828273 | 1.447620819 | 4 | amp |
| TCGA-10-0935 | 1 | 182829103 | 186329149 | 1.702303759 | 5 | amp |
| TCGA-10-0935 | 1 | 186329360 | 186648631 | 1.392656765 | 4 | amp |
| TCGA-10-0935 | 1 | 186823442 | 192628680 | 1.731754833 | 5 | amp |
| TCGA-10-0935 | 1 | 192778196 | 193119562 | 1.375462523 | 4 | amp |
| TCGA-10-0935 | 1 | 193121481 | 197101512 | 1.722131153 | 5 | amp |
| TCGA-10-0935 | 1 | 197102422 | 197896941 | 1.491145424 | 4 | amp |
| TCGA-10-0935 | 1 | 197898092 | 200825255 | 1.735218691 | 5 | amp |
| TCGA-10-0935 | 1 | 200826372 | 202457765 | 1.344887649 | 4 | amp |
| TCGA-10-0935 | 1 | 202462193 | 202915772 | 1.734396605 | 5 | amp |
| TCGA-10-0935 | 1 | 202917369 | 203472882 | 1.428632108 | 4 | amp |
| TCGA-10-0935 | 1 | 203652279 | 203786285 | 1.757637008 | 5 | amp |
| TCGA-10-0935 | 1 | 203787637 | 205042898 | 1.312835133 | 4 | amp |
| TCGA-10-0935 | 1 | 205052644 | 205138985 | 1.721664278 | 5 | amp |
| TCGA-10-0935 | 1 | 205156499 | 207680168 | 1.35826963 | 4 | amp |
| TCGA-10-0935 | 1 | 207684896 | 207791623 | 1.834941961 | 5 | amp |
| TCGA-10-0935 | 1 | 207793197 | 209804139 | 1.441663057 | 4 | amp |
| TCGA-10-0935 | 1 | 209805874 | 211264087 | 1.770818002 | 5 | amp |
| TCGA-10-0935 | 1 | 211276830 | 212977806 | 1.513547929 | 4 | amp |
| TCGA-10-0935 | 1 | 212977905 | 220195894 | 1.663604472 | 5 | amp |
| TCGA-10-0935 | 1 | 220197581 | 220315337 | 1.402054394 | 4 | amp |
| TCGA-10-0935 | 1 | 220316260 | 220825529 | 1.682021499 | 5 | amp |
| TCGA-10-0935 | 1 | 220826388 | 222705500 | 1.405441141 | 4 | amp |
| TCGA-10-0935 | 1 | 222711915 | 222861028 | 1.693252212 | 5 | amp |
| TCGA-10-0935 | 1 | 222867097 | 223958247 | 1.437796712 | 4 | amp |
| TCGA-10-0935 | 1 | 223959448 | 225599150 | 1.645423033 | 5 | amp |
| TCGA-10-0935 | 1 | 225600131 | 233336014 | 1.345722344 | 4 | amp |
| TCGA-10-0935 | 1 | 233344229 | 234455948 | 1.731227053 | 5 | amp |
| TCGA-10-0935 | 1 | 234458751 | 235600832 | 1.450047953 | 4 | amp |
| TCGA-10-0935 | 1 | 235602040 | 236889336 | 1.685914638 | 5 | amp |
| TCGA-10-0935 | 1 | 236890956 | 240256022 | 1.505018392 | 4 | amp |
| TCGA-10-0935 | 1 | 240286441 | 244869094 | 1.62775623 | 5 | amp |
| TCGA-10-0935 | 1 | 245005242 | 247049008 | 1.473931165 | 4 | amp |

| | | | | | | |
|---|---|---|---|---|---|---|
| TCGA-10-0935 | 1 | 247050465 | 247151572 | 1.787108383 | 5 | amp |
| TCGA-10-0935 | 1 | 247162635 | 247582405 | 1.2974418 | 4 | amp |
| TCGA-10-0935 | 1 | 247586471 | 248738122 | 1.646859529 | 5 | amp |
| TCGA-10-0935 | 1 | 248756119 | 249231325 | 1.307224022 | 4 | amp |
| TCGA-10-0935 | 10 | 92880 | 1123954 | 1.184448755 | 4 | amp |
| TCGA-10-0935 | 10 | 1125938 | 4889760 | 1.074444779 | 3 | amp |
| TCGA-10-0935 | 10 | 5005618 | 11527922 | 1.38607228 | 4 | amp |
| TCGA-10-0935 | 10 | 11531040 | 28900896 | 1.634984277 | 5 | amp |
| TCGA-10-0935 | 10 | 28903404 | 37441078 | 1.534977082 | 4 | amp |
| TCGA-10-0935 | 10 | 37442484 | 38622117 | 1.69288307 | 5 | amp |
| TCGA-10-0935 | 10 | 38647221 | 47894662 | 1.357106725 | 4 | amp |
| TCGA-10-0935 | 10 | 47896622 | 48050564 | 1.838030874 | 5 | amp |
| TCGA-10-0935 | 10 | 48187381 | 49395344 | 1.34731184 | 4 | amp |
| TCGA-10-0935 | 10 | 49400711 | 61112251 | 1.701139034 | 5 | amp |
| TCGA-10-0935 | 10 | 61115614 | 61552891 | 1.408754312 | 4 | amp |
| TCGA-10-0935 | 10 | 61554142 | 94837070 | 0.942994198 | 3 | amp |
| TCGA-10-0935 | 10 | 95066648 | 95445119 | 1.70130207 | 5 | amp |
| TCGA-10-0935 | 10 | 95447123 | 95518628 | 1.343889288 | 4 | amp |
| TCGA-10-0935 | 10 | 95537111 | 97082592 | 0.983885946 | 3 | amp |
| TCGA-10-0935 | 10 | 97096244 | 97626156 | 1.281379758 | 4 | amp |
| TCGA-10-0935 | 10 | 97763934 | 119307779 | 0.887823348 | 3 | amp |
| TCGA-10-0935 | 10 | 119768472 | 121600533 | 1.278007301 | 4 | amp |
| TCGA-10-0935 | 10 | 121601895 | 122280664 | 1.682206858 | 5 | amp |
| TCGA-10-0935 | 10 | 122334607 | 124325557 | 1.396116096 | 4 | amp |
| TCGA-10-0935 | 10 | 124329641 | 124359654 | 1.741785025 | 5 | amp |
| TCGA-10-0935 | 10 | 124360462 | 129914331 | 1.322494863 | 4 | amp |
| TCGA-10-0935 | 10 | 129914717 | 135516111 | 0.981380716 | 3 | amp |
| TCGA-10-0935 | 11 | 86637 | 2981189 | 1.019319332 | 3 | amp |
| TCGA-10-0935 | 11 | 2984941 | 3000503 | 1.394946333 | 4 | amp |
| TCGA-10-0935 | 11 | 3022290 | 3431688 | 1.09251216 | 3 | amp |
| TCGA-10-0935 | 11 | 3435159 | 10794254 | 1.261092243 | 4 | amp |
| TCGA-10-0935 | 11 | 10794596 | 11292883 | 0.986403281 | 3 | amp |
| TCGA-10-0935 | 11 | 11314537 | 18655834 | 1.245987924 | 4 | amp |
| TCGA-10-0935 | 11 | 18722401 | 18754970 | 1.026502392 | 3 | amp |
| TCGA-10-0935 | 11 | 18755082 | 34226283 | 1.308446834 | 4 | amp |
| TCGA-10-0935 | 11 | 34470704 | 34664285 | 1.055914383 | 3 | amp |
| TCGA-10-0935 | 11 | 34667898 | 34910027 | 1.391589614 | 4 | amp |
| TCGA-10-0935 | 11 | 34910237 | 43333771 | 0.936316858 | 3 | amp |
| TCGA-10-0935 | 11 | 47652523 | 47862154 | 0.948777906 | 3 | amp |
| TCGA-10-0935 | 11 | 47869395 | 49104658 | 1.400476953 | 4 | amp |
| TCGA-10-0935 | 11 | 49168282 | 60638643 | 1.644367794 | 5 | amp |
| TCGA-10-0935 | 11 | 60638648 | 61042073 | 1.431787968 | 4 | amp |
| TCGA-10-0935 | 11 | 61044048 | 61919460 | 1.069296651 | 3 | amp |
| TCGA-10-0935 | 11 | 61957723 | 62066514 | 1.435195819 | 4 | amp |
| TCGA-10-0935 | 11 | 62123754 | 62656223 | 1.064027861 | 3 | amp |
| TCGA-10-0935 | 11 | 62677130 | 72552683 | 1.337158835 | 4 | amp |
| TCGA-10-0935 | 11 | 72553655 | 72695316 | 1.671836696 | 5 | amp |
| TCGA-10-0935 | 11 | 72696073 | 111728512 | 1.367861362 | 4 | amp |
| TCGA-10-0935 | 11 | 111731205 | 111908210 | 1.113360507 | 3 | amp |
| TCGA-10-0935 | 11 | 111909894 | 112021032 | 1.24722115 | 4 | amp |

| | | | | | | |
|---|---|---|---|---|---|---|
| TCGA-10-0935 | 11 | 112024260 | 113107089 | 1.082199592 | 3 | amp |
| TCGA-10-0935 | 11 | 113111471 | 114027167 | 1.211261611 | 4 | amp |
| TCGA-10-0935 | 11 | 114057656 | 114320752 | 1.037409354 | 3 | amp |
| TCGA-10-0935 | 11 | 114392657 | 115109415 | 1.422488378 | 4 | amp |
| TCGA-10-0935 | 11 | 115110946 | 116707914 | 1.035581639 | 3 | amp |
| TCGA-10-0935 | 11 | 116717070 | 116827841 | 1.253749562 | 4 | amp |
| TCGA-10-0935 | 11 | 117006204 | 117117714 | 1.071169532 | 3 | amp |
| TCGA-10-0935 | 11 | 117150611 | 117246525 | 1.339690877 | 4 | amp |
| TCGA-10-0935 | 11 | 117251315 | 117965610 | 1.090939248 | 3 | amp |
| TCGA-10-0935 | 11 | 117969696 | 118255661 | 1.308526984 | 4 | amp |
| TCGA-10-0935 | 11 | 118257119 | 119063965 | 1.107651891 | 3 | amp |
| TCGA-10-0935 | 11 | 119103071 | 124524739 | 1.243125971 | 4 | amp |
| TCGA-10-0935 | 11 | 124530491 | 125513800 | 1.078486694 | 3 | amp |
| TCGA-10-0935 | 11 | 125513954 | 126110992 | 1.289973074 | 4 | amp |
| TCGA-10-0935 | 11 | 126120394 | 134257557 | 1.101024298 | 3 | amp |
| TCGA-10-0935 | 12 | 73256 | 90681 | 1.870688305 | 5 | amp |
| TCGA-10-0935 | 12 | 208285 | 353031 | 1.027170139 | 3 | amp |
| TCGA-10-0935 | 12 | 368997 | 551132 | 1.332557559 | 4 | amp |
| TCGA-10-0935 | 12 | 644293 | 936437 | 1.093415165 | 3 | amp |
| TCGA-10-0935 | 12 | 939127 | 1890268 | 1.250145689 | 4 | amp |
| TCGA-10-0935 | 12 | 1893022 | 2997650 | 1.09642988 | 3 | amp |
| TCGA-10-0935 | 12 | 3018660 | 3048663 | 1.258727488 | 4 | amp |
| TCGA-10-0935 | 12 | 3103895 | 4554743 | 1.118912913 | 3 | amp |
| TCGA-10-0935 | 12 | 4598958 | 5722155 | 1.224353449 | 4 | amp |
| TCGA-10-0935 | 12 | 5724347 | 6604447 | 1.095209252 | 3 | amp |
| TCGA-10-0935 | 12 | 6618830 | 6636262 | 1.249632502 | 4 | amp |
| TCGA-10-0935 | 12 | 6636908 | 7361278 | 1.10398119 | 3 | amp |
| TCGA-10-0935 | 12 | 7361563 | 8091034 | 1.337967241 | 4 | amp |
| TCGA-10-0935 | 12 | 8192384 | 8330186 | 1.12267996 | 3 | amp |
| TCGA-10-0935 | 12 | 8374358 | 9221446 | 1.286008572 | 4 | amp |
| TCGA-10-0935 | 12 | 9222314 | 9248335 | 1.102579625 | 3 | amp |
| TCGA-10-0935 | 12 | 9251157 | 9392165 | 1.25097076 | 4 | amp |
| TCGA-10-0935 | 12 | 9440753 | 9724173 | 1.146367851 | 3 | amp |
| TCGA-10-0935 | 12 | 9747823 | 14942095 | 1.337595553 | 4 | amp |
| TCGA-10-0935 | 12 | 14943326 | 14959635 | 1.040730455 | 3 | amp |
| TCGA-10-0935 | 12 | 14975818 | 21657569 | 1.36178013 | 4 | amp |
| TCGA-10-0935 | 12 | 21659794 | 21695537 | 1.10557739 | 3 | amp |
| TCGA-10-0935 | 12 | 21699220 | 26493300 | 1.352918783 | 4 | amp |
| TCGA-10-0935 | 12 | 26540326 | 26875528 | 1.811949278 | 5 | amp |
| TCGA-10-0935 | 12 | 26877571 | 44190959 | 1.332456248 | 4 | amp |
| TCGA-10-0935 | 12 | 44191059 | 44238772 | 1.071232039 | 3 | amp |
| TCGA-10-0935 | 12 | 44337958 | 46755002 | 1.363407004 | 4 | amp |
| TCGA-10-0935 | 12 | 46756055 | 46765078 | 1.058089024 | 3 | amp |
| TCGA-10-0935 | 12 | 47160474 | 48091560 | 1.33799338 | 4 | amp |
| TCGA-10-0935 | 12 | 48095196 | 48393920 | 1.101757201 | 3 | amp |
| TCGA-10-0935 | 12 | 48439075 | 48483130 | 1.41131193 | 4 | amp |
| TCGA-10-0935 | 12 | 48490107 | 48516659 | 1.001223626 | 3 | amp |
| TCGA-10-0935 | 12 | 48524122 | 48537944 | 1.267850777 | 4 | amp |
| TCGA-10-0935 | 12 | 48538803 | 50369470 | 1.107846498 | 3 | amp |
| TCGA-10-0935 | 12 | 50383998 | 50400437 | 1.335874486 | 4 | amp |

| | | | | | | |
|---|---|---|---|---|---|---|
| TCGA-10-0935 | 12 | 50410395 | 50514033 | 1.083645456 | 3 | amp |
| TCGA-10-0935 | 12 | 50524252 | 51503099 | 1.263127933 | 4 | amp |
| TCGA-10-0935 | 12 | 51504652 | 51834592 | 1.12444043 | 3 | amp |
| TCGA-10-0935 | 12 | 51844612 | 52404933 | 1.245403264 | 4 | amp |
| TCGA-10-0935 | 12 | 52407457 | 53413871 | 1.09929606 | 3 | amp |
| TCGA-10-0935 | 12 | 53415556 | 53434026 | 1.279615741 | 4 | amp |
| TCGA-10-0935 | 12 | 53442889 | 54105936 | 1.116225618 | 3 | amp |
| TCGA-10-0935 | 12 | 54106534 | 54116007 | 1.337802843 | 4 | amp |
| TCGA-10-0935 | 12 | 54117321 | 54677060 | 1.062299785 | 3 | amp |
| TCGA-10-0935 | 12 | 54677553 | 54744343 | 1.270203313 | 4 | amp |
| TCGA-10-0935 | 12 | 54756409 | 54891684 | 1.074029411 | 3 | amp |
| TCGA-10-0935 | 12 | 54893133 | 54930842 | 1.402059009 | 4 | amp |
| TCGA-10-0935 | 12 | 54932652 | 55024766 | 1.122692406 | 3 | amp |
| TCGA-10-0935 | 12 | 55025512 | 55759843 | 1.401911619 | 4 | amp |
| TCGA-10-0935 | 12 | 55794301 | 56142808 | 1.129358048 | 3 | amp |
| TCGA-10-0935 | 12 | 56143235 | 56296197 | 1.241299743 | 4 | amp |
| TCGA-10-0935 | 12 | 56297157 | 56384646 | 1.136448098 | 3 | amp |
| TCGA-10-0935 | 12 | 56385130 | 56492720 | 1.22183066 | 4 | amp |
| TCGA-10-0935 | 12 | 56493390 | 56733318 | 1.076429333 | 3 | amp |
| TCGA-10-0935 | 12 | 56733397 | 56818899 | 1.293770564 | 4 | amp |
| TCGA-10-0935 | 12 | 56821984 | 56962847 | 1.054180195 | 3 | amp |
| TCGA-10-0935 | 12 | 56963638 | 56982177 | 1.315139539 | 4 | amp |
| TCGA-10-0935 | 12 | 56982665 | 57181137 | 1.115110422 | 3 | amp |
| TCGA-10-0935 | 12 | 57317575 | 57466752 | 1.349588778 | 4 | amp |
| TCGA-10-0935 | 12 | 57472314 | 57662282 | 1.096916911 | 3 | amp |
| TCGA-10-0935 | 12 | 57662634 | 57843852 | 1.364374432 | 4 | amp |
| TCGA-10-0935 | 12 | 57849258 | 57928274 | 1.089649712 | 3 | amp |
| TCGA-10-0935 | 12 | 57928827 | 57975753 | 1.224208673 | 4 | amp |
| TCGA-10-0935 | 12 | 57976308 | 58191781 | 1.108691127 | 3 | amp |
| TCGA-10-0935 | 12 | 58193520 | 68696660 | 1.324919948 | 4 | amp |
| TCGA-10-0935 | 12 | 68707217 | 68725003 | 1.055864258 | 3 | amp |
| TCGA-10-0935 | 12 | 68947227 | 69742376 | 1.268222065 | 4 | amp |
| TCGA-10-0935 | 12 | 69743849 | 69991115 | 1.091658229 | 3 | amp |
| TCGA-10-0935 | 12 | 69991361 | 70088256 | 1.30498784 | 4 | amp |
| TCGA-10-0935 | 12 | 70091382 | 70713170 | 1.109276147 | 3 | amp |
| TCGA-10-0935 | 12 | 70723156 | 96292547 | 1.325009841 | 4 | amp |
| TCGA-10-0935 | 12 | 96300136 | 96388800 | 1.09758305 | 3 | amp |
| TCGA-10-0935 | 12 | 96389384 | 98941673 | 1.296228687 | 4 | amp |
| TCGA-10-0935 | 12 | 98989152 | 99080708 | 1.101194482 | 3 | amp |
| TCGA-10-0935 | 12 | 99093146 | 102117682 | 1.319424857 | 4 | amp |
| TCGA-10-0935 | 12 | 102120066 | 102147353 | 1.021505332 | 3 | amp |
| TCGA-10-0935 | 12 | 102150953 | 108590040 | 1.290207903 | 4 | amp |
| TCGA-10-0935 | 12 | 108600062 | 109288150 | 1.10440615 | 3 | amp |
| TCGA-10-0935 | 12 | 109290732 | 109332786 | 1.243257745 | 4 | amp |
| TCGA-10-0935 | 12 | 109494451 | 109671856 | 1.121011831 | 3 | amp |
| TCGA-10-0935 | 12 | 109673099 | 109687947 | 1.275158227 | 4 | amp |
| TCGA-10-0935 | 12 | 109689845 | 109723332 | 1.089685159 | 3 | amp |
| TCGA-10-0935 | 12 | 109724448 | 109847460 | 1.241740929 | 4 | amp |
| TCGA-10-0935 | 12 | 109847760 | 110371918 | 1.095755075 | 3 | amp |
| TCGA-10-0935 | 12 | 110376158 | 110781257 | 1.231057133 | 4 | amp |

| | | | | | | |
|---|---|---|---|---|---|---|
| TCGA-10-0935 | 12 | 110782673 | 110952967 | 1.12047536 | 3 | amp |
| TCGA-10-0935 | 12 | 110956473 | 111701663 | 1.220124275 | 4 | amp |
| TCGA-10-0935 | 12 | 111729168 | 111957901 | 1.131334581 | 3 | amp |
| TCGA-10-0935 | 12 | 111958671 | 112580115 | 1.249881011 | 4 | amp |
| TCGA-10-0935 | 12 | 112583367 | 112631495 | 1.117318095 | 3 | amp |
| TCGA-10-0935 | 12 | 112632659 | 113448293 | 1.286179166 | 4 | amp |
| TCGA-10-0935 | 12 | 113495946 | 115118997 | 1.075924486 | 3 | amp |
| TCGA-10-0935 | 12 | 115120570 | 119968893 | 1.234144358 | 4 | amp |
| TCGA-10-0935 | 12 | 119978379 | 120166418 | 1.086449054 | 3 | amp |
| TCGA-10-0935 | 12 | 120168247 | 120765599 | 1.211239601 | 4 | amp |
| TCGA-10-0935 | 12 | 120783354 | 121165030 | 1.153886188 | 3 | amp |
| TCGA-10-0935 | 12 | 121175154 | 121618267 | 1.213646674 | 4 | amp |
| TCGA-10-0935 | 12 | 121622055 | 122380555 | 1.12818702 | 3 | amp |
| TCGA-10-0935 | 12 | 122380780 | 123055682 | 1.213550654 | 4 | amp |
| TCGA-10-0935 | 12 | 123057483 | 123082501 | 1.067238254 | 3 | amp |
| TCGA-10-0935 | 12 | 123087081 | 123298011 | 1.225754037 | 4 | amp |
| TCGA-10-0935 | 12 | 123307879 | 123645986 | 1.07054717 | 3 | amp |
| TCGA-10-0935 | 12 | 123646653 | 123880995 | 1.328541051 | 4 | amp |
| TCGA-10-0935 | 12 | 123888089 | 124286023 | 1.104693511 | 3 | amp |
| TCGA-10-0935 | 12 | 124288289 | 124364384 | 1.217566201 | 4 | amp |
| TCGA-10-0935 | 12 | 124371654 | 129569264 | 1.092302777 | 3 | amp |
| TCGA-10-0935 | 12 | 129694016 | 130834002 | 1.211468963 | 4 | amp |
| TCGA-10-0935 | 12 | 130834396 | 130892382 | 1.76237163 | 5 | amp |
| TCGA-10-0935 | 12 | 130897097 | 133779395 | 1.049412242 | 3 | amp |
| TCGA-10-0935 | 13 | 19240876 | 48986610 | 2.035443927 | 5 | amp |
| TCGA-10-0935 | 13 | 49027067 | 49039542 | 1.393222888 | 4 | amp |
| TCGA-10-0935 | 13 | 49047450 | 54886204 | 1.970628022 | 5 | amp |
| TCGA-10-0935 | 13 | 57715404 | 57747822 | 1.562664869 | 4 | amp |
| TCGA-10-0935 | 13 | 58206673 | 111088724 | 2.087566255 | 5 | amp |
| TCGA-10-0935 | 13 | 111090276 | 113900383 | 1.606161078 | 4 | amp |
| TCGA-10-0935 | 13 | 113907362 | 113909472 | 2.214453571 | 5 | amp |
| TCGA-10-0935 | 13 | 113914880 | 115022748 | 1.511805645 | 4 | amp |
| TCGA-10-0935 | 13 | 115024781 | 115091796 | 1.785917897 | 5 | amp |
| TCGA-10-0935 | 14 | 19377543 | 23397437 | 1.239163393 | 4 | amp |
| TCGA-10-0935 | 14 | 23397614 | 23900218 | 1.051261878 | 3 | amp |
| TCGA-10-0935 | 14 | 23900568 | 23947285 | 1.321050941 | 4 | amp |
| TCGA-10-0935 | 14 | 24025909 | 24974913 | 1.003752352 | 3 | amp |
| TCGA-10-0935 | 14 | 24975180 | 58063629 | 1.274806279 | 4 | amp |
| TCGA-10-0935 | 14 | 58471397 | 59014694 | 1.666213692 | 5 | amp |
| TCGA-10-0935 | 14 | 59107399 | 62542160 | 1.215813665 | 4 | amp |
| TCGA-10-0935 | 14 | 62547531 | 64612983 | 0.937090763 | 3 | amp |
| TCGA-10-0935 | 14 | 73783053 | 74349181 | 0.982408202 | 3 | amp |
| TCGA-10-0935 | 15 | 32976755 | 34643088 | 0.925041039 | 3 | amp |
| TCGA-10-0935 | 15 | 45983139 | 52357247 | 0.894141199 | 3 | amp |
| TCGA-10-0935 | 15 | 55965541 | 56122205 | 0.918585555 | 3 | amp |
| TCGA-10-0935 | 15 | 56122687 | 90934115 | 1.203365921 | 4 | amp |
| TCGA-10-0935 | 15 | 90969299 | 91358540 | 1.629956336 | 5 | amp |
| TCGA-10-0935 | 15 | 91419443 | 100943002 | 1.419449629 | 4 | amp |
| TCGA-10-0935 | 15 | 100996054 | 101121087 | 1.689910632 | 5 | amp |
| TCGA-10-0935 | 15 | 101152406 | 102513951 | 1.303156031 | 4 | amp |

| | | | | | | |
|---|---|---|---|---|---|---|
| TCGA-10-0935 | 16 | 7382955 | 7760805 | 0.942374354 | 3 | amp |
| TCGA-10-0935 | 16 | 9856945 | 10576139 | 0.994212519 | 3 | amp |
| TCGA-10-0935 | 16 | 11850041 | 11876236 | 0.907841893 | 3 | amp |
| TCGA-10-0935 | 16 | 14028941 | 14576709 | 0.917223156 | 3 | amp |
| TCGA-10-0935 | 16 | 14748844 | 14766654 | 1.023811373 | 3 | amp |
| TCGA-10-0935 | 16 | 14960359 | 14983105 | 0.954900478 | 3 | amp |
| TCGA-10-0935 | 16 | 18826747 | 18880645 | 0.936557852 | 3 | amp |
| TCGA-10-0935 | 16 | 19451304 | 19516428 | 0.941085629 | 3 | amp |
| TCGA-10-0935 | 16 | 19627414 | 19649018 | 0.939348474 | 3 | amp |
| TCGA-10-0935 | 16 | 19744934 | 20337766 | 0.974563545 | 3 | amp |
| TCGA-10-0935 | 16 | 20380762 | 20471645 | 0.900682057 | 3 | amp |
| TCGA-10-0935 | 16 | 20638454 | 20702534 | 0.952716504 | 3 | amp |
| TCGA-10-0935 | 16 | 20838328 | 20976756 | 0.935047523 | 3 | amp |
| TCGA-10-0935 | 16 | 21071570 | 21147859 | 0.965764056 | 3 | amp |
| TCGA-10-0935 | 16 | 21209068 | 21273538 | 0.95961112 | 3 | amp |
| TCGA-10-0935 | 16 | 21698731 | 21747766 | 0.957494701 | 3 | amp |
| TCGA-10-0935 | 16 | 21809064 | 21830089 | 0.931236592 | 3 | amp |
| TCGA-10-0935 | 16 | 21858677 | 21900041 | 0.93330198 | 3 | amp |
| TCGA-10-0935 | 16 | 21968537 | 22142622 | 0.917615387 | 3 | amp |
| TCGA-10-0935 | 16 | 22493744 | 22500639 | 0.958196672 | 3 | amp |
| TCGA-10-0935 | 16 | 23457087 | 23490277 | 0.92325799 | 3 | amp |
| TCGA-10-0935 | 16 | 23999811 | 24231477 | 0.940556339 | 3 | amp |
| TCGA-10-0935 | 16 | 24873897 | 24909482 | 0.933712157 | 3 | amp |
| TCGA-10-0935 | 16 | 27585159 | 27709854 | 0.936039038 | 3 | amp |
| TCGA-10-0935 | 16 | 28128590 | 28360572 | 0.897850555 | 3 | amp |
| TCGA-10-0935 | 16 | 29057320 | 29063596 | 0.991749357 | 3 | amp |
| TCGA-10-0935 | 16 | 30278932 | 30316573 | 0.914509495 | 3 | amp |
| TCGA-10-0935 | 16 | 32374884 | 32442118 | 1.036266998 | 3 | amp |
| TCGA-10-0935 | 16 | 47682395 | 47703346 | 0.952556635 | 3 | amp |
| TCGA-10-0935 | 16 | 48117578 | 48142455 | 0.907958172 | 3 | amp |
| TCGA-10-0935 | 16 | 48242311 | 48258386 | 0.948998628 | 3 | amp |
| TCGA-10-0935 | 16 | 50128599 | 50261951 | 0.954932113 | 3 | amp |
| TCGA-10-0935 | 16 | 50362557 | 50388424 | 0.958768594 | 3 | amp |
| TCGA-10-0935 | 16 | 50810078 | 50830446 | 0.911793242 | 3 | amp |
| TCGA-10-0935 | 16 | 51679789 | 53340348 | 0.914749289 | 3 | amp |
| TCGA-10-0935 | 16 | 53493325 | 53513161 | 0.97544749 | 3 | amp |
| TCGA-10-0935 | 16 | 53672212 | 53706974 | 0.919614169 | 3 | amp |
| TCGA-10-0935 | 16 | 53844000 | 54145870 | 0.973769777 | 3 | amp |
| TCGA-10-0935 | 16 | 55579627 | 55897371 | 0.914827667 | 3 | amp |
| TCGA-10-0935 | 16 | 56792388 | 56878525 | 0.986438597 | 3 | amp |
| TCGA-10-0935 | 16 | 58199417 | 58218248 | 0.985242445 | 3 | amp |
| TCGA-10-0935 | 16 | 58554810 | 58609091 | 1.000662271 | 3 | amp |
| TCGA-10-0935 | 16 | 58752091 | 61859119 | 0.907550824 | 3 | amp |
| TCGA-10-0935 | 16 | 65006754 | 65397177 | 0.92538827 | 3 | amp |
| TCGA-10-0935 | 16 | 66757614 | 66766429 | 0.937075613 | 3 | amp |
| TCGA-10-0935 | 16 | 67380134 | 67409352 | 0.925511916 | 3 | amp |
| TCGA-10-0935 | 16 | 68071869 | 68100563 | 1.018685461 | 3 | amp |
| TCGA-10-0935 | 16 | 68835534 | 68856135 | 0.9206056 | 3 | amp |
| TCGA-10-0935 | 16 | 68914431 | 69008120 | 0.929468364 | 3 | amp |
| TCGA-10-0935 | 16 | 70348741 | 70359593 | 0.971974565 | 3 | amp |

| | | | | | | |
|---|---|---|---|---|---|---|
| TCGA-10-0935 | 16 | 70569176 | 70601496 | 0.930262456 | 3 | amp |
| TCGA-10-0935 | 16 | 70861118 | 70868108 | 0.915874761 | 3 | amp |
| TCGA-10-0935 | 16 | 70869537 | 70884574 | 1.253276021 | 4 | amp |
| TCGA-10-0935 | 16 | 70888984 | 70993756 | 1.011553852 | 3 | amp |
| TCGA-10-0935 | 16 | 70995831 | 71007947 | 1.288050435 | 4 | amp |
| TCGA-10-0935 | 16 | 71007989 | 71220850 | 1.016660166 | 3 | amp |
| TCGA-10-0935 | 16 | 71411519 | 71423829 | 0.943098765 | 3 | amp |
| TCGA-10-0935 | 16 | 71689119 | 71715850 | 0.920748644 | 3 | amp |
| TCGA-10-0935 | 16 | 71773100 | 71799499 | 0.990081025 | 3 | amp |
| TCGA-10-0935 | 16 | 72088493 | 72111010 | 1.018229553 | 3 | amp |
| TCGA-10-0935 | 16 | 72162898 | 72205161 | 0.953643363 | 3 | amp |
| TCGA-10-0935 | 16 | 74499522 | 74511482 | 0.914909799 | 3 | amp |
| TCGA-10-0935 | 16 | 74923566 | 74983715 | 0.936534409 | 3 | amp |
| TCGA-10-0935 | 16 | 75327834 | 75446662 | 1.006078729 | 3 | amp |
| TCGA-10-0935 | 16 | 75611161 | 75646784 | 0.939591702 | 3 | amp |
| TCGA-10-0935 | 16 | 76389191 | 76573802 | 0.935805402 | 3 | amp |
| TCGA-10-0935 | 16 | 77323084 | 77359968 | 1.043762686 | 3 | amp |
| TCGA-10-0935 | 16 | 77769653 | 78064780 | 0.916925899 | 3 | amp |
| TCGA-10-0935 | 16 | 83250905 | 83817087 | 0.995443699 | 3 | amp |
| TCGA-10-0935 | 16 | 90124592 | 90128919 | 1.001676007 | 3 | amp |
| TCGA-10-0935 | 17 | 63618 | 2344878 | 1.048887513 | 3 | amp |
| TCGA-10-0935 | 17 | 2367452 | 2583655 | 1.219597139 | 4 | amp |
| TCGA-10-0935 | 17 | 2584999 | 2935768 | 1.033404744 | 3 | amp |
| TCGA-10-0935 | 17 | 2965951 | 3337189 | 1.249494497 | 4 | amp |
| TCGA-10-0935 | 17 | 3343386 | 3386899 | 0.964299567 | 3 | amp |
| TCGA-10-0935 | 17 | 3392522 | 3419866 | 1.307739031 | 4 | amp |
| TCGA-10-0935 | 17 | 3421826 | 3921317 | 1.012152485 | 3 | amp |
| TCGA-10-0935 | 17 | 3922952 | 4020482 | 1.243957962 | 4 | amp |
| TCGA-10-0935 | 17 | 4027152 | 5087599 | 1.0205591 | 3 | amp |
| TCGA-10-0935 | 17 | 5114052 | 5312260 | 1.243620308 | 4 | amp |
| TCGA-10-0935 | 17 | 5313975 | 6406930 | 1.006694664 | 3 | amp |
| TCGA-10-0935 | 17 | 6428669 | 6524332 | 1.221747149 | 4 | amp |
| TCGA-10-0935 | 17 | 6526159 | 6684238 | 1.01955859 | 3 | amp |
| TCGA-10-0935 | 17 | 6690066 | 6719336 | 1.320563935 | 4 | amp |
| TCGA-10-0935 | 17 | 6722474 | 7637615 | 0.998722326 | 3 | amp |
| TCGA-10-0935 | 17 | 7637696 | 7674280 | 1.240638014 | 4 | amp |
| TCGA-10-0935 | 17 | 7674566 | 7843128 | 1.047551969 | 3 | amp |
| TCGA-10-0935 | 17 | 7843336 | 7852885 | 1.28548378 | 4 | amp |
| TCGA-10-0935 | 17 | 7907111 | 9737219 | 1.031241376 | 3 | amp |
| TCGA-10-0935 | 17 | 9739679 | 9774167 | 1.349558328 | 4 | amp |
| TCGA-10-0935 | 17 | 9783643 | 10235597 | 1.090597501 | 3 | amp |
| TCGA-10-0935 | 17 | 10243450 | 10535351 | 1.275463135 | 4 | amp |
| TCGA-10-0935 | 17 | 10535736 | 11515151 | 1.04131373 | 3 | amp |
| TCGA-10-0935 | 17 | 11520713 | 12847005 | 1.236658429 | 4 | amp |
| TCGA-10-0935 | 17 | 12847329 | 15217595 | 1.056715796 | 3 | amp |
| TCGA-10-0935 | 17 | 15222368 | 15519161 | 1.228646628 | 4 | amp |
| TCGA-10-0935 | 17 | 15522273 | 15909894 | 1.028001955 | 3 | amp |
| TCGA-10-0935 | 17 | 15928287 | 15984100 | 1.289962114 | 4 | amp |
| TCGA-10-0935 | 17 | 15989525 | 16596407 | 1.029184044 | 3 | amp |
| TCGA-10-0935 | 17 | 16608408 | 16705659 | 1.230299653 | 4 | amp |

| | | | | | | |
|---|---|---|---|---|---|---|
| TCGA-10-0935 | 17 | 16725690 | 18397728 | 1.066106296 | 3 | amp |
| TCGA-10-0935 | 17 | 18416685 | 18509932 | 1.257162325 | 4 | amp |
| TCGA-10-0935 | 17 | 18511186 | 18585006 | 0.981473236 | 3 | amp |
| TCGA-10-0935 | 17 | 18625537 | 18739324 | 1.261107031 | 4 | amp |
| TCGA-10-0935 | 17 | 18741321 | 19451491 | 1.065067642 | 3 | amp |
| TCGA-10-0935 | 17 | 19452908 | 19474897 | 1.244527926 | 4 | amp |
| TCGA-10-0935 | 17 | 19476043 | 19679750 | 0.980632699 | 3 | amp |
| TCGA-10-0935 | 17 | 19680811 | 20330307 | 1.226415259 | 4 | amp |
| TCGA-10-0935 | 17 | 20331797 | 20602223 | 1.069579462 | 3 | amp |
| TCGA-10-0935 | 17 | 20619869 | 20768834 | 1.335067018 | 4 | amp |
| TCGA-10-0935 | 17 | 20769758 | 26925998 | 1.082742268 | 3 | amp |
| TCGA-10-0935 | 17 | 26938532 | 26960479 | 1.300363477 | 4 | amp |
| TCGA-10-0935 | 17 | 26960517 | 27000533 | 1.02647489 | 3 | amp |
| TCGA-10-0935 | 17 | 27001259 | 27015292 | 1.256988868 | 4 | amp |
| TCGA-10-0935 | 17 | 27016355 | 27182373 | 1.06453584 | 3 | amp |
| TCGA-10-0935 | 17 | 27183225 | 27187629 | 1.278203439 | 4 | amp |
| TCGA-10-0935 | 17 | 27188362 | 27621194 | 1.082552498 | 3 | amp |
| TCGA-10-0935 | 17 | 27778512 | 27838065 | 1.28385415 | 4 | amp |
| TCGA-10-0935 | 17 | 27844452 | 27959983 | 1.087535397 | 3 | amp |
| TCGA-10-0935 | 17 | 27962952 | 28525570 | 1.224896533 | 4 | amp |
| TCGA-10-0935 | 17 | 28530153 | 28548984 | 1.057233942 | 3 | amp |
| TCGA-10-0935 | 17 | 28575960 | 28791838 | 1.280203136 | 4 | amp |
| TCGA-10-0935 | 17 | 28804406 | 29070412 | 0.985305825 | 3 | amp |
| TCGA-10-0935 | 17 | 29093504 | 29219835 | 1.311530024 | 4 | amp |
| TCGA-10-0935 | 17 | 29220229 | 29365692 | 1.090240287 | 3 | amp |
| TCGA-10-0935 | 17 | 29369217 | 29657534 | 1.35153825 | 4 | amp |
| TCGA-10-0935 | 17 | 29661832 | 30190576 | 1.094006251 | 3 | amp |
| TCGA-10-0935 | 17 | 30192360 | 30216482 | 1.418262638 | 4 | amp |
| TCGA-10-0935 | 17 | 30219220 | 30293250 | 1.027800564 | 3 | amp |
| TCGA-10-0935 | 17 | 30300148 | 30320366 | 1.325827795 | 4 | amp |
| TCGA-10-0935 | 17 | 30320835 | 30521163 | 1.021387064 | 3 | amp |
| TCGA-10-0935 | 17 | 30525947 | 30551813 | 1.350575999 | 4 | amp |
| TCGA-10-0935 | 17 | 30594872 | 30796233 | 1.075704294 | 3 | amp |
| TCGA-10-0935 | 17 | 30800783 | 31105604 | 1.237116208 | 4 | amp |
| TCGA-10-0935 | 17 | 31107547 | 32483573 | 1.103100763 | 3 | amp |
| TCGA-10-0935 | 17 | 32582346 | 32688919 | 1.253681263 | 4 | amp |
| TCGA-10-0935 | 17 | 32690052 | 32965263 | 1.041621553 | 3 | amp |
| TCGA-10-0935 | 17 | 33255041 | 33353602 | 1.245671487 | 4 | amp |
| TCGA-10-0935 | 17 | 33427953 | 33935463 | 1.062795207 | 3 | amp |
| TCGA-10-0935 | 17 | 33951360 | 34050740 | 1.279915159 | 4 | amp |
| TCGA-10-0935 | 17 | 34062161 | 34247293 | 1.087326007 | 3 | amp |
| TCGA-10-0935 | 17 | 34249457 | 34431396 | 1.236111538 | 4 | amp |
| TCGA-10-0935 | 17 | 34431917 | 35311258 | 1.013017915 | 3 | amp |
| TCGA-10-0935 | 17 | 35343912 | 35914168 | 1.257694581 | 4 | amp |
| TCGA-10-0935 | 17 | 35921267 | 36916892 | 1.068622206 | 3 | amp |
| TCGA-10-0935 | 17 | 36918620 | 37009376 | 1.254599784 | 4 | amp |
| TCGA-10-0935 | 17 | 37009866 | 37373437 | 1.062599377 | 3 | amp |
| TCGA-10-0935 | 17 | 37417676 | 37676394 | 1.222942618 | 4 | amp |
| TCGA-10-0935 | 17 | 37680912 | 38033118 | 1.030666973 | 3 | amp |
| TCGA-10-0935 | 17 | 38060990 | 38073571 | 1.303560519 | 4 | amp |

| | | | | | | |
|---|---|---|---|---|---|---|
| TCGA-10-0935 | 17 | 38078720 | 38412802 | 1.03446038 | 3 | amp |
| TCGA-10-0935 | 17 | 38416738 | 38458298 | 1.228052106 | 4 | amp |
| TCGA-10-0935 | 17 | 38487409 | 38818351 | 1.07558681 | 3 | amp |
| TCGA-10-0935 | 17 | 38821243 | 38923931 | 1.275284393 | 4 | amp |
| TCGA-10-0935 | 17 | 38925119 | 39092897 | 1.040306693 | 3 | amp |
| TCGA-10-0935 | 17 | 39114801 | 39156136 | 1.319931177 | 4 | amp |
| TCGA-10-0935 | 17 | 39164997 | 40467850 | 1.014576451 | 3 | amp |
| TCGA-10-0935 | 17 | 40468802 | 40673151 | 1.209197466 | 4 | amp |
| TCGA-10-0935 | 17 | 40689369 | 41026428 | 1.004575357 | 3 | amp |
| TCGA-10-0935 | 17 | 41052888 | 41108043 | 1.274526154 | 4 | amp |
| TCGA-10-0935 | 17 | 41108158 | 41199749 | 1.099044517 | 3 | amp |
| TCGA-10-0935 | 17 | 41201114 | 41567854 | 1.29162959 | 4 | amp |
| TCGA-10-0935 | 17 | 41568515 | 41719458 | 1.940149689 | 5 | amp |
| TCGA-10-0935 | 17 | 41720839 | 41891478 | 1.242024942 | 4 | amp |
| TCGA-10-0935 | 17 | 41891496 | 42767139 | 1.010613278 | 3 | amp |
| TCGA-10-0935 | 17 | 42786612 | 42824940 | 1.241116342 | 4 | amp |
| TCGA-10-0935 | 17 | 42825650 | 42930770 | 1.027527805 | 3 | amp |
| TCGA-10-0935 | 17 | 42930793 | 42964070 | 1.310942522 | 4 | amp |
| TCGA-10-0935 | 17 | 42971776 | 43580944 | 1.029778695 | 3 | amp |
| TCGA-10-0935 | 17 | 43587590 | 43627717 | 1.391668744 | 4 | amp |
| TCGA-10-0935 | 17 | 43664066 | 44117278 | 1.01646558 | 3 | amp |
| TCGA-10-0935 | 17 | 44127864 | 44828985 | 1.408389885 | 4 | amp |
| TCGA-10-0935 | 17 | 44832647 | 45097181 | 1.06969194 | 3 | amp |
| TCGA-10-0935 | 17 | 45098908 | 45219818 | 1.261349739 | 4 | amp |
| TCGA-10-0935 | 17 | 45221208 | 45507375 | 1.077283196 | 3 | amp |
| TCGA-10-0935 | 17 | 45517758 | 45759829 | 1.210457967 | 4 | amp |
| TCGA-10-0935 | 17 | 45773470 | 46190772 | 1.004073913 | 3 | amp |
| TCGA-10-0935 | 17 | 46196023 | 46474154 | 1.325456924 | 4 | amp |
| TCGA-10-0935 | 17 | 46606892 | 46846663 | 0.991167512 | 3 | amp |
| TCGA-10-0935 | 17 | 46847004 | 46940403 | 1.262549621 | 4 | amp |
| TCGA-10-0935 | 17 | 46970738 | 47375872 | 1.031249874 | 3 | amp |
| TCGA-10-0935 | 17 | 47375949 | 47395117 | 1.340930037 | 4 | amp |
| TCGA-10-0935 | 17 | 47482339 | 47656669 | 1.003403263 | 3 | amp |
| TCGA-10-0935 | 17 | 47677661 | 47778905 | 1.469131313 | 4 | amp |
| TCGA-10-0935 | 17 | 47780146 | 47886585 | 1.027676876 | 3 | amp |
| TCGA-10-0935 | 17 | 47888826 | 47916079 | 1.355102314 | 4 | amp |
| TCGA-10-0935 | 17 | 47917181 | 48814413 | 1.033579645 | 3 | amp |
| TCGA-10-0935 | 17 | 48817626 | 49157124 | 1.283579778 | 4 | amp |
| TCGA-10-0935 | 17 | 49231701 | 49249026 | 1.047773631 | 3 | amp |
| TCGA-10-0935 | 17 | 49270062 | 54559932 | 1.305198407 | 4 | amp |
| TCGA-10-0935 | 17 | 54671531 | 56621606 | 1.034957319 | 3 | amp |
| TCGA-10-0935 | 17 | 56634329 | 57079136 | 1.309925434 | 4 | amp |
| TCGA-10-0935 | 17 | 57089634 | 57298161 | 1.117989128 | 3 | amp |
| TCGA-10-0935 | 17 | 57311813 | 58037538 | 1.271915007 | 4 | amp |
| TCGA-10-0935 | 17 | 58039878 | 58236807 | 1.026587132 | 3 | amp |
| TCGA-10-0935 | 17 | 58256582 | 59155911 | 1.258823461 | 4 | amp |
| TCGA-10-0935 | 17 | 59161816 | 59560928 | 1.055983436 | 3 | amp |
| TCGA-10-0935 | 17 | 59667857 | 60130101 | 1.228975079 | 4 | amp |
| TCGA-10-0935 | 17 | 60140424 | 60351500 | 1.029633341 | 3 | amp |
| TCGA-10-0935 | 17 | 60360048 | 60685515 | 1.259979018 | 4 | amp |

| | | | | | | |
|---|---|---|---|---|---|---|
| TCGA-10-0935 | 17 | 60689689 | 60799963 | 1.041832576 | 3 | amp |
| TCGA-10-0935 | 17 | 60802231 | 61499373 | 1.230354291 | 4 | amp |
| TCGA-10-0935 | 17 | 61512375 | 61843583 | 1.098752092 | 3 | amp |
| TCGA-10-0935 | 17 | 61864369 | 61890820 | 1.310005394 | 4 | amp |
| TCGA-10-0935 | 17 | 61892917 | 62506404 | 1.068698554 | 3 | amp |
| TCGA-10-0935 | 17 | 62512833 | 62758710 | 1.253850903 | 4 | amp |
| TCGA-10-0935 | 17 | 62788443 | 62968716 | 1.089178825 | 3 | amp |
| TCGA-10-0935 | 17 | 63010360 | 63206784 | 1.236790145 | 4 | amp |
| TCGA-10-0935 | 17 | 63221031 | 63632150 | 1.052790067 | 3 | amp |
| TCGA-10-0935 | 17 | 63633213 | 64225525 | 1.264661312 | 4 | amp |
| TCGA-10-0935 | 17 | 64298935 | 65074714 | 1.104375972 | 3 | amp |
| TCGA-10-0935 | 17 | 65082890 | 65978445 | 1.303759765 | 4 | amp |
| TCGA-10-0935 | 17 | 65988003 | 66339958 | 0.979337923 | 3 | amp |
| TCGA-10-0935 | 17 | 66343229 | 67171660 | 1.29348035 | 4 | amp |
| TCGA-10-0935 | 17 | 67178263 | 67215918 | 1.015150435 | 3 | amp |
| TCGA-10-0935 | 17 | 67217894 | 68129516 | 1.288519509 | 4 | amp |
| TCGA-10-0935 | 17 | 68171162 | 73646818 | 1.04572264 | 3 | amp |
| TCGA-10-0935 | 17 | 73647215 | 73695966 | 1.297547824 | 4 | amp |
| TCGA-10-0935 | 17 | 73698515 | 81188237 | 0.996460508 | 3 | amp |
| TCGA-10-0935 | 18 | 47273 | 46956814 | 1.249949935 | 4 | amp |
| TCGA-10-0935 | 18 | 47008657 | 47018126 | 0.892491366 | 3 | amp |
| TCGA-10-0935 | 18 | 47088636 | 56058814 | 1.25509183 | 4 | amp |
| TCGA-10-0935 | 18 | 56063389 | 61587200 | 1.709696805 | 5 | amp |
| TCGA-10-0935 | 18 | 61597229 | 76757346 | 1.518837171 | 4 | amp |
| TCGA-10-0935 | 18 | 76856442 | 76903890 | 1.773975557 | 5 | amp |
| TCGA-10-0935 | 18 | 76914469 | 77960823 | 1.230340976 | 4 | amp |
| TCGA-10-0935 | 19 | 4110459 | 5727376 | 1.011769846 | 3 | amp |
| TCGA-10-0935 | 19 | 5729858 | 5771138 | 1.288907835 | 4 | amp |
| TCGA-10-0935 | 19 | 5772737 | 6042154 | 0.992161908 | 3 | amp |
| TCGA-10-0935 | 19 | 6141497 | 6185675 | 1.3457251 | 4 | amp |
| TCGA-10-0935 | 19 | 6187243 | 6832216 | 1.008937348 | 3 | amp |
| TCGA-10-0935 | 19 | 6833155 | 6937666 | 1.403279152 | 4 | amp |
| TCGA-10-0935 | 19 | 6939977 | 7809108 | 1.030738161 | 3 | amp |
| TCGA-10-0935 | 19 | 7809752 | 7833918 | 1.252752776 | 4 | amp |
| TCGA-10-0935 | 19 | 7911346 | 8966852 | 1.025102494 | 3 | amp |
| TCGA-10-0935 | 19 | 8968853 | 8976465 | 1.361668845 | 4 | amp |
| TCGA-10-0935 | 19 | 8976554 | 9027617 | 1.649705363 | 5 | amp |
| TCGA-10-0935 | 19 | 9028189 | 9362769 | 1.281247912 | 4 | amp |
| TCGA-10-0935 | 19 | 9406099 | 9489063 | 0.986728106 | 3 | amp |
| TCGA-10-0935 | 19 | 9489580 | 9585057 | 1.242703559 | 4 | amp |
| TCGA-10-0935 | 19 | 9585914 | 10132437 | 1.051163196 | 3 | amp |
| TCGA-10-0935 | 19 | 10157421 | 10166472 | 1.287490752 | 4 | amp |
| TCGA-10-0935 | 19 | 10169198 | 10260350 | 1.007903936 | 3 | amp |
| TCGA-10-0935 | 19 | 10260526 | 10291567 | 1.205806792 | 4 | amp |
| TCGA-10-0935 | 19 | 10292674 | 13419345 | 1.012881093 | 3 | amp |
| TCGA-10-0935 | 19 | 13423478 | 13566033 | 1.35406247 | 4 | amp |
| TCGA-10-0935 | 19 | 13862563 | 14676117 | 1.032933298 | 3 | amp |
| TCGA-10-0935 | 19 | 14693933 | 14992167 | 1.291892641 | 4 | amp |
| TCGA-10-0935 | 19 | 15052210 | 15163135 | 1.051476115 | 3 | amp |
| TCGA-10-0935 | 19 | 15164221 | 15198821 | 1.315134571 | 4 | amp |

| | | | | | | |
|---|---|---|---|---|---|---|
| TCGA-10-0935 | 19 | 15219857 | 17133035 | 1.020067923 | 3 | amp |
| TCGA-10-0935 | 19 | 17160624 | 17267903 | 1.220065453 | 4 | amp |
| TCGA-10-0935 | 19 | 17270189 | 19788855 | 1.011022525 | 3 | amp |
| TCGA-10-0935 | 19 | 19789474 | 23159929 | 1.305615832 | 4 | amp |
| TCGA-10-0935 | 19 | 23542308 | 30312736 | 1.07243463 | 3 | amp |
| TCGA-10-0935 | 19 | 30312849 | 32968613 | 1.342058087 | 4 | amp |
| TCGA-10-0935 | 19 | 32971263 | 33378762 | 0.980779183 | 3 | amp |
| TCGA-10-0935 | 19 | 33390713 | 33655196 | 1.1889738 | 4 | amp |
| TCGA-10-0935 | 19 | 33663239 | 36642465 | 1.009445369 | 3 | amp |
| TCGA-10-0935 | 19 | 36673341 | 37838274 | 1.24264723 | 4 | amp |
| TCGA-10-0935 | 19 | 37838666 | 40030772 | 1.023328766 | 3 | amp |
| TCGA-10-0935 | 19 | 40093125 | 40228644 | 1.385195109 | 4 | amp |
| TCGA-10-0935 | 19 | 40316300 | 40514484 | 0.977795289 | 3 | amp |
| TCGA-10-0935 | 19 | 40519567 | 40589094 | 1.307635515 | 4 | amp |
| TCGA-10-0935 | 19 | 40704221 | 41388140 | 0.996917023 | 3 | amp |
| TCGA-10-0935 | 19 | 41441971 | 41522761 | 1.356016836 | 4 | amp |
| TCGA-10-0935 | 19 | 41530256 | 42937238 | 1.018000889 | 3 | amp |
| TCGA-10-0935 | 19 | 42937907 | 43683350 | 1.188214772 | 4 | amp |
| TCGA-10-0935 | 19 | 43687532 | 44339760 | 1.030127681 | 3 | amp |
| TCGA-10-0935 | 19 | 44341147 | 44936588 | 1.248516348 | 4 | amp |
| TCGA-10-0935 | 19 | 44946715 | 45017028 | 0.992877155 | 3 | amp |
| TCGA-10-0935 | 19 | 45017229 | 45033566 | 1.347637585 | 4 | amp |
| TCGA-10-0935 | 19 | 45150488 | 46543860 | 1.023003108 | 3 | amp |
| TCGA-10-0935 | 19 | 46544150 | 46664395 | 1.324923914 | 4 | amp |
| TCGA-10-0935 | 19 | 46732983 | 48364860 | 0.987069384 | 3 | amp |
| TCGA-10-0935 | 19 | 48374707 | 48603142 | 1.336641392 | 4 | amp |
| TCGA-10-0935 | 19 | 48607792 | 49714842 | 0.99817984 | 3 | amp |
| TCGA-10-0935 | 19 | 49793334 | 49813808 | 1.329833628 | 4 | amp |
| TCGA-10-0935 | 19 | 49814126 | 52222617 | 1.00616557 | 3 | amp |
| TCGA-10-0935 | 19 | 52249150 | 52443644 | 1.288125906 | 4 | amp |
| TCGA-10-0935 | 19 | 52443855 | 53901335 | 1.061141195 | 3 | amp |
| TCGA-10-0935 | 19 | 53905133 | 54297406 | 1.329884706 | 4 | amp |
| TCGA-10-0935 | 19 | 54299077 | 55021817 | 0.994993752 | 3 | amp |
| TCGA-10-0935 | 19 | 55043941 | 55053838 | 1.287104264 | 4 | amp |
| TCGA-10-0935 | 19 | 55085302 | 55295296 | 0.98286703 | 3 | amp |
| TCGA-10-0935 | 19 | 55299620 | 55378201 | 1.303414461 | 4 | amp |
| TCGA-10-0935 | 19 | 55385672 | 56228298 | 0.997321526 | 3 | amp |
| TCGA-10-0935 | 19 | 56235307 | 56572947 | 1.321058358 | 4 | amp |
| TCGA-10-0935 | 19 | 56599866 | 59110878 | 1.029096148 | 3 | amp |
| TCGA-10-0935 | 2 | 41527 | 8890473 | 1.370497546 | 4 | amp |
| TCGA-10-0935 | 2 | 8891564 | 8953475 | 1.812047316 | 5 | amp |
| TCGA-10-0935 | 2 | 8957736 | 9637429 | 1.538248024 | 4 | amp |
| TCGA-10-0935 | 2 | 9642263 | 9731702 | 1.733500311 | 5 | amp |
| TCGA-10-0935 | 2 | 9770253 | 11323624 | 1.510462146 | 4 | amp |
| TCGA-10-0935 | 2 | 11332260 | 11348063 | 1.738989575 | 5 | amp |
| TCGA-10-0935 | 2 | 11348372 | 15432895 | 1.462694756 | 4 | amp |
| TCGA-10-0935 | 2 | 15448288 | 15651524 | 1.748036558 | 5 | amp |
| TCGA-10-0935 | 2 | 15674655 | 15767283 | 1.41578622 | 4 | amp |
| TCGA-10-0935 | 2 | 15768544 | 16743460 | 1.734894573 | 5 | amp |
| TCGA-10-0935 | 2 | 16745249 | 17860238 | 1.523447605 | 4 | amp |

| | | | | | | |
|---|---|---|---|---|---|---|
| TCGA-10-0935 | 2 | 17864847 | 17907755 | 1.813080056 | 5 | amp |
| TCGA-10-0935 | 2 | 17912315 | 20455159 | 1.507099861 | 4 | amp |
| TCGA-10-0935 | 2 | 20455742 | 24011569 | 1.684978345 | 5 | amp |
| TCGA-10-0935 | 2 | 24020980 | 24443994 | 1.503399925 | 4 | amp |
| TCGA-10-0935 | 2 | 24468973 | 24975057 | 1.721388056 | 5 | amp |
| TCGA-10-0935 | 2 | 24980783 | 26424223 | 1.508137595 | 4 | amp |
| TCGA-10-0935 | 2 | 26426877 | 26453170 | 1.889305158 | 5 | amp |
| TCGA-10-0935 | 2 | 26454967 | 27550157 | 1.424498104 | 4 | amp |
| TCGA-10-0935 | 2 | 27550859 | 27560899 | 1.8307824 | 5 | amp |
| TCGA-10-0935 | 2 | 27564777 | 27677540 | 1.377903355 | 4 | amp |
| TCGA-10-0935 | 2 | 27679298 | 27703074 | 1.803134068 | 5 | amp |
| TCGA-10-0935 | 2 | 27703869 | 27861159 | 1.509845023 | 4 | amp |
| TCGA-10-0935 | 2 | 27861704 | 27917585 | 1.728905334 | 5 | amp |
| TCGA-10-0935 | 2 | 27994565 | 29354313 | 1.5703183 | 4 | amp |
| TCGA-10-0935 | 2 | 29355004 | 29420577 | 1.862365131 | 5 | amp |
| TCGA-10-0935 | 2 | 29429997 | 31611186 | 1.571540063 | 4 | amp |
| TCGA-10-0935 | 2 | 31620474 | 33787859 | 1.730567099 | 5 | amp |
| TCGA-10-0935 | 2 | 33809789 | 36623963 | 1.30721926 | 4 | amp |
| TCGA-10-0935 | 2 | 36668341 | 37410672 | 1.703926106 | 5 | amp |
| TCGA-10-0935 | 2 | 37414463 | 37465014 | 1.504786323 | 4 | amp |
| TCGA-10-0935 | 2 | 37468707 | 37520464 | 1.869103283 | 5 | amp |
| TCGA-10-0935 | 2 | 37543343 | 38294188 | 1.532510436 | 4 | amp |
| TCGA-10-0935 | 2 | 38297858 | 44571106 | 1.743091475 | 5 | amp |
| TCGA-10-0935 | 2 | 44571631 | 45236262 | 1.529345418 | 4 | amp |
| TCGA-10-0935 | 2 | 45616413 | 45809010 | 1.791880648 | 5 | amp |
| TCGA-10-0935 | 2 | 45812709 | 47672801 | 1.569050991 | 4 | amp |
| TCGA-10-0935 | 2 | 47690111 | 53978118 | 1.775831808 | 5 | amp |
| TCGA-10-0935 | 2 | 53992491 | 54029034 | 1.504159778 | 4 | amp |
| TCGA-10-0935 | 2 | 54035396 | 55826223 | 1.701666496 | 5 | amp |
| TCGA-10-0935 | 2 | 55831102 | 55898542 | 1.562417578 | 4 | amp |
| TCGA-10-0935 | 2 | 55899095 | 56108927 | 1.752865893 | 5 | amp |
| TCGA-10-0935 | 2 | 56144752 | 60996030 | 1.534551036 | 4 | amp |
| TCGA-10-0935 | 2 | 60997559 | 68622999 | 1.727753826 | 5 | amp |
| TCGA-10-0935 | 2 | 68691301 | 69015149 | 1.526942309 | 4 | amp |
| TCGA-10-0935 | 2 | 69034359 | 70915264 | 1.830799703 | 5 | amp |
| TCGA-10-0935 | 2 | 70917869 | 73226170 | 1.506282373 | 4 | amp |
| TCGA-10-0935 | 2 | 73228590 | 74086496 | 1.818036443 | 5 | amp |
| TCGA-10-0935 | 2 | 74087168 | 77746993 | 1.467481883 | 4 | amp |
| TCGA-10-0935 | 2 | 77748735 | 85066479 | 1.700084019 | 5 | amp |
| TCGA-10-0935 | 2 | 85097328 | 86348719 | 1.558870525 | 4 | amp |
| TCGA-10-0935 | 2 | 86350794 | 86378651 | 1.886289482 | 5 | amp |
| TCGA-10-0935 | 2 | 86380629 | 86684209 | 1.565926335 | 4 | amp |
| TCGA-10-0935 | 2 | 86691137 | 86998877 | 1.80798251 | 5 | amp |
| TCGA-10-0935 | 2 | 87000442 | 87178510 | 1.58015199 | 4 | amp |
| TCGA-10-0935 | 2 | 87180060 | 88111730 | 1.778001874 | 5 | amp |
| TCGA-10-0935 | 2 | 88115744 | 88858525 | 1.571527461 | 4 | amp |
| TCGA-10-0935 | 2 | 88861797 | 89037603 | 1.802388717 | 5 | amp |
| TCGA-10-0935 | 2 | 89049486 | 89161476 | 1.470802574 | 4 | amp |
| TCGA-10-0935 | 2 | 89185051 | 90260280 | 1.898282387 | 5 | amp |
| TCGA-10-0935 | 2 | 90273672 | 95464797 | 1.528221663 | 4 | amp |

| | | | | | | |
|---|---|---|---|---|---|---|
| TCGA-10-0935 | 2 | 95472081 | 95501899 | 1.974272965 | 5 | amp |
| TCGA-10-0935 | 2 | 95504512 | 97866286 | 1.554360843 | 4 | amp |
| TCGA-10-0935 | 2 | 97879110 | 98177368 | 1.731707681 | 5 | amp |
| TCGA-10-0935 | 2 | 98195341 | 99779581 | 1.498615723 | 4 | amp |
| TCGA-10-0935 | 2 | 99785825 | 101126087 | 1.692525385 | 5 | amp |
| TCGA-10-0935 | 2 | 101182907 | 103011127 | 1.543859893 | 4 | amp |
| TCGA-10-0935 | 2 | 103012958 | 103318972 | 1.759737839 | 5 | amp |
| TCGA-10-0935 | 2 | 103320948 | 106994560 | 1.554042844 | 4 | amp |
| TCGA-10-0935 | 2 | 106998567 | 109288960 | 1.710083178 | 5 | amp |
| TCGA-10-0935 | 2 | 109289290 | 110301953 | 1.592172697 | 4 | amp |
| TCGA-10-0935 | 2 | 110303570 | 110629063 | 1.772008901 | 5 | amp |
| TCGA-10-0935 | 2 | 110656276 | 111230384 | 1.543019713 | 4 | amp |
| TCGA-10-0935 | 2 | 111257597 | 111415232 | 1.731005187 | 5 | amp |
| TCGA-10-0935 | 2 | 111415981 | 112560103 | 1.50698341 | 4 | amp |
| TCGA-10-0935 | 2 | 112561005 | 112622575 | 1.742932642 | 5 | amp |
| TCGA-10-0935 | 2 | 112625576 | 112832573 | 1.568987941 | 4 | amp |
| TCGA-10-0935 | 2 | 112834753 | 112863705 | 1.839939817 | 5 | amp |
| TCGA-10-0935 | 2 | 112865330 | 113127832 | 1.553083764 | 4 | amp |
| TCGA-10-0935 | 2 | 113135607 | 113181910 | 1.845401253 | 5 | amp |
| TCGA-10-0935 | 2 | 113190935 | 113672862 | 1.523234172 | 4 | amp |
| TCGA-10-0935 | 2 | 113674645 | 116538630 | 1.833707813 | 5 | amp |
| TCGA-10-0935 | 2 | 116539891 | 118588348 | 1.464372319 | 4 | amp |
| TCGA-10-0935 | 2 | 118677885 | 120918563 | 1.691299243 | 5 | amp |
| TCGA-10-0935 | 2 | 120922388 | 122139901 | 1.53447015 | 4 | amp |
| TCGA-10-0935 | 2 | 122144690 | 122227908 | 1.726805221 | 5 | amp |
| TCGA-10-0935 | 2 | 122260716 | 125232505 | 1.512410616 | 4 | amp |
| TCGA-10-0935 | 2 | 125261820 | 127451541 | 1.781191218 | 5 | amp |
| TCGA-10-0935 | 2 | 127453499 | 128631844 | 1.448218378 | 4 | amp |
| TCGA-10-0935 | 2 | 128699538 | 128903578 | 1.685169158 | 5 | amp |
| TCGA-10-0935 | 2 | 128910297 | 135075197 | 1.453381226 | 4 | amp |
| TCGA-10-0935 | 2 | 135076175 | 135186032 | 1.953529191 | 5 | amp |
| TCGA-10-0935 | 2 | 135199257 | 135677480 | 1.54726644 | 4 | amp |
| TCGA-10-0935 | 2 | 135694354 | 150438832 | 1.799828121 | 5 | amp |
| TCGA-10-0935 | 2 | 150443546 | 152311720 | 1.554716517 | 4 | amp |
| TCGA-10-0935 | 2 | 152313052 | 155555998 | 1.78693409 | 5 | amp |
| TCGA-10-0935 | 2 | 155566102 | 157183450 | 1.28845099 | 4 | amp |
| TCGA-10-0935 | 2 | 157184324 | 162273627 | 1.799580091 | 5 | amp |
| TCGA-10-0935 | 2 | 162274143 | 162364001 | 1.432320328 | 4 | amp |
| TCGA-10-0935 | 2 | 162627463 | 162851905 | 1.77038522 | 5 | amp |
| TCGA-10-0935 | 2 | 162862226 | 162881450 | 1.353145254 | 4 | amp |
| TCGA-10-0935 | 2 | 162890046 | 168115899 | 1.828207929 | 5 | amp |
| TCGA-10-0935 | 2 | 168571053 | 168726579 | 1.244171093 | 4 | amp |
| TCGA-10-0935 | 2 | 168811982 | 170336152 | 1.812741213 | 5 | amp |
| TCGA-10-0935 | 2 | 170338741 | 170377563 | 1.507918392 | 4 | amp |
| TCGA-10-0935 | 2 | 170382060 | 170518987 | 1.791169435 | 5 | amp |
| TCGA-10-0935 | 2 | 170531062 | 170677812 | 1.573575172 | 4 | amp |
| TCGA-10-0935 | 2 | 170678449 | 174783536 | 1.747733645 | 5 | amp |
| TCGA-10-0935 | 2 | 174819560 | 177135202 | 1.561869336 | 4 | amp |
| TCGA-10-0935 | 2 | 177161551 | 190436601 | 1.822976522 | 5 | amp |
| TCGA-10-0935 | 2 | 190437569 | 190535469 | 1.397144049 | 4 | amp |

| | | | | | | |
|---|---|---|---|---|---|---|
| TCGA-10-0935 | 2 | 190541353 | 197763126 | 1.792627866 | 5 | amp |
| TCGA-10-0935 | 2 | 197767267 | 197878455 | 1.487317844 | 4 | amp |
| TCGA-10-0935 | 2 | 197889865 | 201334747 | 1.740466459 | 5 | amp |
| TCGA-10-0935 | 2 | 201337544 | 201468094 | 1.538418794 | 4 | amp |
| TCGA-10-0935 | 2 | 201468719 | 201535436 | 1.82539484 | 5 | amp |
| TCGA-10-0935 | 2 | 201677912 | 201746237 | 1.476704259 | 4 | amp |
| TCGA-10-0935 | 2 | 201747051 | 204062129 | 1.725429612 | 5 | amp |
| TCGA-10-0935 | 2 | 204064055 | 204306861 | 1.583475793 | 4 | amp |
| TCGA-10-0935 | 2 | 204309541 | 206581127 | 1.757765792 | 5 | amp |
| TCGA-10-0935 | 2 | 206587196 | 207310259 | 1.557309256 | 4 | amp |
| TCGA-10-0935 | 2 | 207345903 | 209049775 | 1.769438512 | 5 | amp |
| TCGA-10-0935 | 2 | 209051629 | 209142466 | 1.483244905 | 4 | amp |
| TCGA-10-0935 | 2 | 209150440 | 211477048 | 1.743151652 | 5 | amp |
| TCGA-10-0935 | 2 | 211481085 | 211533021 | 1.498198652 | 4 | amp |
| TCGA-10-0935 | 2 | 211539595 | 217235025 | 1.766078312 | 5 | amp |
| TCGA-10-0935 | 2 | 217279412 | 219557489 | 1.559941683 | 4 | amp |
| TCGA-10-0935 | 2 | 219557954 | 219619164 | 1.808428937 | 5 | amp |
| TCGA-10-0935 | 2 | 219674274 | 222294894 | 1.423216279 | 4 | amp |
| TCGA-10-0935 | 2 | 222298816 | 223097019 | 1.708065831 | 5 | amp |
| TCGA-10-0935 | 2 | 223158368 | 223560027 | 1.567998748 | 4 | amp |
| TCGA-10-0935 | 2 | 223574411 | 231135387 | 1.750074697 | 5 | amp |
| TCGA-10-0935 | 2 | 231149033 | 231282397 | 1.530428924 | 4 | amp |
| TCGA-10-0935 | 2 | 231307612 | 232035472 | 1.740149534 | 5 | amp |
| TCGA-10-0935 | 2 | 232070916 | 232578157 | 1.534391236 | 4 | amp |
| TCGA-10-0935 | 2 | 232597612 | 233075135 | 1.694829909 | 5 | amp |
| TCGA-10-0935 | 2 | 233103208 | 233626185 | 1.511754456 | 4 | amp |
| TCGA-10-0935 | 2 | 233632851 | 233709301 | 1.819966961 | 5 | amp |
| TCGA-10-0935 | 2 | 233710362 | 234173843 | 1.486489381 | 4 | amp |
| TCGA-10-0935 | 2 | 234178590 | 234227478 | 1.759405489 | 5 | amp |
| TCGA-10-0935 | 2 | 234229252 | 243160772 | 1.339348597 | 4 | amp |
| TCGA-10-0935 | 20 | 68319 | 62926333 | 2.68706073 | 5 | amp |
| TCGA-10-0935 | 21 | 9483321 | 30440071 | 1.270443914 | 4 | amp |
| TCGA-10-0935 | 21 | 30441723 | 33647233 | 0.935477254 | 3 | amp |
| TCGA-10-0935 | 21 | 33650978 | 43166340 | 1.165480996 | 4 | amp |
| TCGA-10-0935 | 21 | 43166731 | 48111215 | 0.90065897 | 3 | amp |
| TCGA-10-0935 | 22 | 16084594 | 16172039 | 1.008641758 | 3 | amp |
| TCGA-10-0935 | 22 | 16255773 | 16287929 | 1.295763587 | 4 | amp |
| TCGA-10-0935 | 22 | 16345860 | 17119669 | 1.039030616 | 3 | amp |
| TCGA-10-0935 | 22 | 18652598 | 21115692 | 1.041946803 | 3 | amp |
| TCGA-10-0935 | 22 | 21119021 | 21242165 | 1.336848152 | 4 | amp |
| TCGA-10-0935 | 22 | 21272167 | 22127276 | 1.008933565 | 3 | amp |
| TCGA-10-0935 | 22 | 22142491 | 22162163 | 1.596692731 | 4 | amp |
| TCGA-10-0935 | 22 | 22239811 | 22698106 | 1.037290498 | 3 | amp |
| TCGA-10-0935 | 22 | 22707445 | 22937561 | 1.244912819 | 4 | amp |
| TCGA-10-0935 | 22 | 22973426 | 23955896 | 1.051887669 | 3 | amp |
| TCGA-10-0935 | 22 | 23956301 | 23974226 | 1.427671517 | 4 | amp |
| TCGA-10-0935 | 22 | 23982732 | 24236775 | 1.007848353 | 3 | amp |
| TCGA-10-0935 | 22 | 24376399 | 24452858 | 1.365483797 | 4 | amp |
| TCGA-10-0935 | 22 | 24455637 | 26068348 | 1.083535256 | 3 | amp |
| TCGA-10-0935 | 22 | 26070389 | 26118456 | 1.32387165 | 4 | amp |

| | | | | | | |
|---|---|---|---|---|---|---|
| TCGA-10-0935 | 22 | 26157017 | 26695162 | 1.135090588 | 3 | amp |
| TCGA-10-0935 | 22 | 26701884 | 26778061 | 1.284416415 | 4 | amp |
| TCGA-10-0935 | 22 | 26829643 | 28250963 | 1.044664682 | 3 | amp |
| TCGA-10-0935 | 22 | 28254315 | 29108010 | 1.273186173 | 4 | amp |
| TCGA-10-0935 | 22 | 29115367 | 29755017 | 1.108821605 | 3 | amp |
| TCGA-10-0935 | 22 | 29755730 | 29940640 | 1.282688707 | 4 | amp |
| TCGA-10-0935 | 22 | 29945028 | 30204196 | 1.11764931 | 3 | amp |
| TCGA-10-0935 | 22 | 30209384 | 30500467 | 1.25180479 | 4 | amp |
| TCGA-10-0935 | 22 | 30507734 | 31807092 | 1.099628161 | 3 | amp |
| TCGA-10-0935 | 22 | 31816206 | 31981146 | 1.374206867 | 4 | amp |
| TCGA-10-0935 | 22 | 31985377 | 32161063 | 1.109941522 | 3 | amp |
| TCGA-10-0935 | 22 | 32162552 | 32789993 | 1.29224657 | 4 | amp |
| TCGA-10-0935 | 22 | 32790986 | 32827427 | 1.00349592 | 3 | amp |
| TCGA-10-0935 | 22 | 32828324 | 35689165 | 1.250506176 | 4 | amp |
| TCGA-10-0935 | 22 | 35689561 | 36003536 | 1.044649908 | 3 | amp |
| TCGA-10-0935 | 22 | 36006928 | 36662117 | 1.652956466 | 5 | amp |
| TCGA-10-0935 | 22 | 36678622 | 36684998 | 1.233130632 | 4 | amp |
| TCGA-10-0935 | 22 | 36685086 | 38247511 | 1.046604813 | 3 | amp |
| TCGA-10-0935 | 22 | 38251521 | 38284511 | 1.422745912 | 4 | amp |
| TCGA-10-0935 | 22 | 38307892 | 40080524 | 1.035058655 | 3 | amp |
| TCGA-10-0935 | 22 | 40139625 | 40356238 | 1.314263498 | 4 | amp |
| TCGA-10-0935 | 22 | 40361957 | 41258013 | 1.056020683 | 3 | amp |
| TCGA-10-0935 | 22 | 41264940 | 41575008 | 1.23816783 | 4 | amp |
| TCGA-10-0935 | 22 | 41601318 | 42112157 | 1.076434404 | 3 | amp |
| TCGA-10-0935 | 22 | 42114056 | 42172308 | 1.245168415 | 4 | amp |
| TCGA-10-0935 | 22 | 42174703 | 44011813 | 1.066073685 | 3 | amp |
| TCGA-10-0935 | 22 | 44022322 | 44112909 | 1.301778703 | 4 | amp |
| TCGA-10-0935 | 22 | 44127574 | 45732310 | 0.99643299 | 3 | amp |
| TCGA-10-0935 | 22 | 45736186 | 45789737 | 1.334170221 | 4 | amp |
| TCGA-10-0935 | 22 | 45790545 | 46189595 | 1.010062258 | 3 | amp |
| TCGA-10-0935 | 22 | 46202810 | 47193548 | 1.35103763 | 4 | amp |
| TCGA-10-0935 | 22 | 47287105 | 51237627 | 1.010203433 | 3 | amp |
| TCGA-10-0935 | 3 | 361444 | 15137673 | 1.514359001 | 4 | amp |
| TCGA-10-0935 | 3 | 15175811 | 197955154 | 1.758952313 | 5 | amp |
| TCGA-10-0935 | 4 | 146048611 | 146067606 | 0.921574229 | 3 | amp |
| TCGA-10-0935 | 4 | 190876188 | 190876308 | 1.182830855 | 4 | amp |
| TCGA-10-0935 | 4 | 190878544 | 190947944 | 0.982742457 | 3 | amp |
| TCGA-10-0935 | 5 | 143116 | 6755080 | 0.941670519 | 3 | amp |
| TCGA-10-0935 | 5 | 7414664 | 43454095 | 1.273739212 | 4 | amp |
| TCGA-10-0935 | 5 | 43486994 | 52097698 | 0.945763804 | 3 | amp |
| TCGA-10-0935 | 5 | 52145138 | 52340936 | 1.228898306 | 4 | amp |
| TCGA-10-0935 | 5 | 52344189 | 66438123 | 0.891122071 | 3 | amp |
| TCGA-10-0935 | 5 | 80932346 | 94749952 | 0.963796294 | 3 | amp |
| TCGA-10-0935 | 5 | 94755926 | 139909414 | 1.262028492 | 4 | amp |
| TCGA-10-0935 | 5 | 139914914 | 140896616 | 1.041011129 | 3 | amp |
| TCGA-10-0935 | 5 | 140903662 | 140966806 | 1.295130229 | 4 | amp |
| TCGA-10-0935 | 5 | 140967743 | 141354564 | 1.025374785 | 3 | amp |
| TCGA-10-0935 | 5 | 141357831 | 168216660 | 1.243570222 | 4 | amp |
| TCGA-10-0935 | 5 | 168222487 | 171510146 | 1.628767536 | 5 | amp |
| TCGA-10-0935 | 5 | 171517150 | 172421861 | 1.323138909 | 4 | amp |

| | | | | | | |
|---|---|---|---|---|---|---|
| TCGA-10-0935 | 5 | 172447227 | 172550263 | 1.747422667 | 5 | amp |
| TCGA-10-0935 | 5 | 172560630 | 173317898 | 1.298218108 | 4 | amp |
| TCGA-10-0935 | 5 | 173337476 | 173491336 | 1.641238991 | 5 | amp |
| TCGA-10-0935 | 5 | 173531195 | 175394311 | 1.314944811 | 4 | amp |
| TCGA-10-0935 | 5 | 175511920 | 175775114 | 1.73024875 | 5 | amp |
| TCGA-10-0935 | 5 | 175775215 | 175837340 | 1.239846809 | 4 | amp |
| TCGA-10-0935 | 5 | 175906162 | 175933979 | 1.676095454 | 5 | amp |
| TCGA-10-0935 | 5 | 175956278 | 177036738 | 1.277700771 | 4 | amp |
| TCGA-10-0935 | 5 | 177053411 | 177178101 | 1.628249644 | 5 | amp |
| TCGA-10-0935 | 5 | 177180132 | 177422939 | 1.245998673 | 4 | amp |
| TCGA-10-0935 | 5 | 177457548 | 177474135 | 1.760387858 | 5 | amp |
| TCGA-10-0935 | 5 | 177482599 | 180338656 | 1.261965485 | 4 | amp |
| TCGA-10-0935 | 5 | 180374453 | 180377583 | 1.835536919 | 5 | amp |
| TCGA-10-0935 | 5 | 180409261 | 180899507 | 1.244305883 | 4 | amp |
| TCGA-10-0935 | 6 | 105907 | 8415235 | 1.032167931 | 3 | amp |
| TCGA-10-0935 | 6 | 8417112 | 9908853 | 1.207053889 | 4 | amp |
| TCGA-10-0935 | 6 | 9932970 | 10913189 | 1.107160788 | 3 | amp |
| TCGA-10-0935 | 6 | 10924723 | 13228231 | 1.224687652 | 4 | amp |
| TCGA-10-0935 | 6 | 13230226 | 13622778 | 0.992186724 | 3 | amp |
| TCGA-10-0935 | 6 | 13625850 | 15468980 | 1.168031945 | 4 | amp |
| TCGA-10-0935 | 6 | 15487535 | 17514192 | 1.017063505 | 3 | amp |
| TCGA-10-0935 | 6 | 17539474 | 24302293 | 1.194300575 | 4 | amp |
| TCGA-10-0935 | 6 | 24353763 | 24460686 | 0.98790789 | 3 | amp |
| TCGA-10-0935 | 6 | 24462900 | 25969660 | 1.333774033 | 4 | amp |
| TCGA-10-0935 | 6 | 25972061 | 26285751 | 0.983193916 | 3 | amp |
| TCGA-10-0935 | 6 | 26368392 | 26468836 | 1.252392017 | 4 | amp |
| TCGA-10-0935 | 6 | 26501493 | 28539785 | 0.966362138 | 3 | amp |
| TCGA-10-0935 | 6 | 28539812 | 28554524 | 1.262289534 | 4 | amp |
| TCGA-10-0935 | 6 | 28751352 | 29055034 | 1.005881521 | 3 | amp |
| TCGA-10-0935 | 6 | 29079655 | 29430501 | 1.299417807 | 4 | amp |
| TCGA-10-0935 | 6 | 29454480 | 29625031 | 0.966210108 | 3 | amp |
| TCGA-10-0935 | 6 | 29627059 | 29638235 | 1.317963699 | 4 | amp |
| TCGA-10-0935 | 6 | 29638427 | 30671374 | 0.963906369 | 3 | amp |
| TCGA-10-0935 | 6 | 30671376 | 30681170 | 1.263526729 | 4 | amp |
| TCGA-10-0935 | 6 | 30681369 | 32187585 | 0.950391751 | 3 | amp |
| TCGA-10-0935 | 6 | 32187833 | 32782417 | 1.245651815 | 4 | amp |
| TCGA-10-0935 | 6 | 32782775 | 32827365 | 0.924367789 | 3 | amp |
| TCGA-10-0935 | 6 | 32902665 | 32920829 | 1.179202382 | 4 | amp |
| TCGA-10-0935 | 6 | 32940599 | 34558419 | 0.969976738 | 3 | amp |
| TCGA-10-0935 | 6 | 34574296 | 34962258 | 1.198403879 | 4 | amp |
| TCGA-10-0935 | 6 | 34985212 | 35480493 | 0.98732347 | 3 | amp |
| TCGA-10-0935 | 6 | 35480521 | 36075438 | 1.169457936 | 4 | amp |
| TCGA-10-0935 | 6 | 36076130 | 36359658 | 1.030893003 | 3 | amp |
| TCGA-10-0935 | 6 | 36368195 | 36492298 | 1.246222039 | 4 | amp |
| TCGA-10-0935 | 6 | 36493333 | 37141908 | 1.001802113 | 3 | amp |
| TCGA-10-0935 | 6 | 37182937 | 37427520 | 1.15539429 | 4 | amp |
| TCGA-10-0935 | 6 | 37429312 | 37631932 | 0.988200515 | 3 | amp |
| TCGA-10-0935 | 6 | 37897642 | 38998204 | 1.307780841 | 4 | amp |
| TCGA-10-0935 | 6 | 39024130 | 39353482 | 1.016249361 | 3 | amp |
| TCGA-10-0935 | 6 | 39387661 | 39607579 | 1.228796087 | 4 | amp |

| | | | | | | |
|---|---|---|---|---|---|---|
| TCGA-10-0935 | 6 | 39682444 | 42541781 | 1.012179629 | 3 | amp |
| TCGA-10-0935 | 6 | 42559867 | 42638510 | 1.293957122 | 4 | amp |
| TCGA-10-0935 | 6 | 42641516 | 42713830 | 0.976364474 | 3 | amp |
| TCGA-10-0935 | 6 | 42789720 | 42830358 | 1.336567855 | 4 | amp |
| TCGA-10-0935 | 6 | 42832370 | 44393939 | 1.07058668 | 3 | amp |
| TCGA-10-0935 | 6 | 44394159 | 46129516 | 1.287761295 | 4 | amp |
| TCGA-10-0935 | 6 | 46133091 | 49403372 | 0.94514155 | 3 | amp |
| TCGA-10-0935 | 6 | 51483828 | 52622761 | 0.956581061 | 3 | amp |
| TCGA-10-0935 | 6 | 52876490 | 110942619 | 0.936844922 | 3 | amp |
| TCGA-10-0935 | 6 | 111303260 | 114181813 | 0.939267798 | 3 | amp |
| TCGA-10-0935 | 6 | 116324980 | 125269280 | 0.933461777 | 3 | amp |
| TCGA-10-0935 | 6 | 125283950 | 126360036 | 1.312677033 | 4 | amp |
| TCGA-10-0935 | 6 | 126661332 | 144768460 | 0.942882529 | 3 | amp |
| TCGA-10-0935 | 6 | 144837895 | 155504682 | 0.915811032 | 3 | amp |
| TCGA-10-0935 | 6 | 160818972 | 161134207 | 1.005125105 | 3 | amp |
| TCGA-10-0935 | 7 | 195522 | 22175759 | 0.496096205 | 1 | loss |
| TCGA-10-0935 | 7 | 31015377 | 40723787 | 0.938212817 | 3 | amp |
| TCGA-10-0935 | 7 | 48141349 | 50468330 | 0.968670297 | 3 | amp |
| TCGA-10-0935 | 7 | 62910136 | 64389443 | 0.916071328 | 3 | amp |
| TCGA-10-0935 | 7 | 64438485 | 96324216 | 1.260903822 | 4 | amp |
| TCGA-10-0935 | 7 | 96338947 | 99246035 | 1.001143375 | 3 | amp |
| TCGA-10-0935 | 7 | 99247655 | 99461359 | 1.350359534 | 4 | amp |
| TCGA-10-0935 | 7 | 99463516 | 99780523 | 0.988002296 | 3 | amp |
| TCGA-10-0935 | 7 | 99783780 | 100014867 | 1.178784625 | 4 | amp |
| TCGA-10-0935 | 7 | 100016682 | 102249904 | 1.015663153 | 3 | amp |
| TCGA-10-0935 | 7 | 102293806 | 128415848 | 1.275670153 | 4 | amp |
| TCGA-10-0935 | 7 | 128432413 | 128597398 | 1.03946422 | 3 | amp |
| TCGA-10-0935 | 7 | 128607329 | 134813739 | 1.188554485 | 4 | amp |
| TCGA-10-0935 | 7 | 134849142 | 134943266 | 0.926376135 | 3 | amp |
| TCGA-10-0935 | 7 | 135047577 | 142471836 | 1.296873113 | 4 | amp |
| TCGA-10-0935 | 7 | 142479868 | 151135385 | 1.057402361 | 3 | amp |
| TCGA-10-0935 | 7 | 151164160 | 152511742 | 1.18957199 | 4 | amp |
| TCGA-10-0935 | 7 | 152513559 | 158935247 | 1.02005051 | 3 | amp |
| TCGA-10-0935 | 8 | 116074 | 2090370 | 1.362748933 | 4 | amp |
| TCGA-10-0935 | 8 | 2091292 | 6303090 | 1.801398657 | 5 | amp |
| TCGA-10-0935 | 8 | 6312628 | 7230754 | 1.602501774 | 4 | amp |
| TCGA-10-0935 | 8 | 7234908 | 7353550 | 1.8696841 | 5 | amp |
| TCGA-10-0935 | 8 | 7366553 | 7669521 | 1.523194229 | 4 | amp |
| TCGA-10-0935 | 8 | 7673055 | 7796323 | 1.863694373 | 5 | amp |
| TCGA-10-0935 | 8 | 7804889 | 12291651 | 1.603871429 | 4 | amp |
| TCGA-10-0935 | 8 | 12435418 | 12592911 | 1.854866975 | 5 | amp |
| TCGA-10-0935 | 8 | 12594197 | 12973190 | 1.566138529 | 4 | amp |
| TCGA-10-0935 | 8 | 13072058 | 19278059 | 1.797286228 | 5 | amp |
| TCGA-10-0935 | 8 | 19297331 | 19822880 | 1.598608384 | 4 | amp |
| TCGA-10-0935 | 8 | 20003270 | 20037053 | 1.80886657 | 5 | amp |
| TCGA-10-0935 | 8 | 20038263 | 22147862 | 1.595944182 | 4 | amp |
| TCGA-10-0935 | 8 | 22161507 | 22210778 | 1.925028287 | 5 | amp |
| TCGA-10-0935 | 8 | 22211777 | 23712097 | 1.503434325 | 4 | amp |
| TCGA-10-0935 | 8 | 24151625 | 24365110 | 1.857351464 | 5 | amp |
| TCGA-10-0935 | 8 | 24365968 | 25181475 | 1.614614604 | 4 | amp |

| | | | | | | |
|---|---|---|---|---|---|---|
| TCGA-10-0935 | 8 | 25182851 | 25766090 | 1.79321253 | 5 | amp |
| TCGA-10-0935 | 8 | 25890544 | 27881026 | 1.581828939 | 4 | amp |
| TCGA-10-0935 | 8 | 27884463 | 29053769 | 1.753207037 | 5 | amp |
| TCGA-10-0935 | 8 | 29102849 | 30038194 | 1.604191474 | 4 | amp |
| TCGA-10-0935 | 8 | 30040579 | 33311060 | 1.781381363 | 5 | amp |
| TCGA-10-0935 | 8 | 33318869 | 33371103 | 1.542596764 | 4 | amp |
| TCGA-10-0935 | 8 | 33406267 | 37556214 | 1.856137003 | 5 | amp |
| TCGA-10-0935 | 8 | 37595433 | 38879257 | 1.551753027 | 4 | amp |
| TCGA-10-0935 | 8 | 38880639 | 39678724 | 1.747359547 | 5 | amp |
| TCGA-10-0935 | 8 | 39679052 | 52322153 | 1.514427894 | 4 | amp |
| TCGA-10-0935 | 8 | 52323768 | 86393078 | 2.074498297 | 5 | amp |
| TCGA-10-0935 | 8 | 86554947 | 86840921 | 1.523108348 | 4 | amp |
| TCGA-10-0935 | 8 | 87060663 | 146279593 | 2.094790788 | 5 | amp |
| TCGA-10-0935 | 9 | 14753 | 34343459 | 1.695264396 | 5 | amp |
| TCGA-10-0935 | 9 | 34370788 | 43709765 | 1.407587813 | 4 | amp |
| TCGA-10-0935 | 9 | 43737303 | 44109486 | 1.675930017 | 5 | amp |
| TCGA-10-0935 | 9 | 44110469 | 110094036 | 1.243785234 | 4 | amp |
| TCGA-10-0935 | 9 | 110247969 | 123955724 | 0.889729269 | 3 | amp |
| TCGA-10-0935 | X | 200797 | 155233550 | 0.450192318 | 1 | loss |
| TCGA-10-0935 | X | 155252400 | 155254972 | 1.338208972 | 4 | amp |
| TCGA-13-0723 | 1 | 14642 | 745558 | 1.299362383 | 4 | amp |
| TCGA-13-0723 | 1 | 808997 | 7796621 | 1.91324558 | 5 | amp |
| TCGA-13-0723 | 1 | 7796969 | 15988250 | 1.528812921 | 4 | amp |
| TCGA-13-0723 | 1 | 16042724 | 16559569 | 2.122969421 | 5 | amp |
| TCGA-13-0723 | 1 | 16560032 | 19449560 | 1.639381475 | 4 | amp |
| TCGA-13-0723 | 1 | 19450989 | 19519064 | 1.176489723 | 3 | amp |
| TCGA-13-0723 | 1 | 19519868 | 21154210 | 1.547904577 | 4 | amp |
| TCGA-13-0723 | 1 | 21155582 | 21329280 | 1.129971889 | 3 | amp |
| TCGA-13-0723 | 1 | 21546415 | 22139046 | 1.529977088 | 4 | amp |
| TCGA-13-0723 | 1 | 22142381 | 22336393 | 2.116571253 | 5 | amp |
| TCGA-13-0723 | 1 | 22338921 | 26863497 | 1.547866893 | 4 | amp |
| TCGA-13-0723 | 1 | 26873295 | 26899903 | 1.875790872 | 5 | amp |
| TCGA-13-0723 | 1 | 26900510 | 32051123 | 1.562905702 | 4 | amp |
| TCGA-13-0723 | 1 | 32051295 | 32265799 | 1.964920189 | 5 | amp |
| TCGA-13-0723 | 1 | 32267225 | 35580918 | 1.479133056 | 4 | amp |
| TCGA-13-0723 | 1 | 35650005 | 35885294 | 1.145352898 | 3 | amp |
| TCGA-13-0723 | 1 | 35900438 | 36202211 | 1.352700144 | 4 | amp |
| TCGA-13-0723 | 1 | 36202445 | 36307397 | 1.151523362 | 3 | amp |
| TCGA-13-0723 | 1 | 36315748 | 39720098 | 1.471577831 | 4 | amp |
| TCGA-13-0723 | 1 | 39723593 | 39920794 | 1.191342942 | 3 | amp |
| TCGA-13-0723 | 1 | 39923981 | 42925615 | 1.413617119 | 4 | amp |
| TCGA-13-0723 | 1 | 42965822 | 43111817 | 1.068067679 | 3 | amp |
| TCGA-13-0723 | 1 | 43119020 | 43825096 | 1.517034663 | 4 | amp |
| TCGA-13-0723 | 1 | 43825114 | 43831071 | 2.150235746 | 5 | amp |
| TCGA-13-0723 | 1 | 43831167 | 45243856 | 1.642020879 | 4 | amp |
| TCGA-13-0723 | 1 | 45244005 | 45287604 | 1.897043924 | 5 | amp |
| TCGA-13-0723 | 1 | 45288003 | 47501885 | 1.471506281 | 4 | amp |
| TCGA-13-0723 | 1 | 47504294 | 47581292 | 1.085531865 | 3 | amp |
| TCGA-13-0723 | 1 | 47582302 | 51831722 | 1.333037939 | 4 | amp |
| TCGA-13-0723 | 1 | 51860025 | 52299885 | 1.116731267 | 3 | amp |

| | | | | | | |
|---|---|---|---|---|---|---|
| TCGA-13-0723 | 1 | 52301783 | 52897139 | 1.367443924 | 4 | amp |
| TCGA-13-0723 | 1 | 52900996 | 52954763 | 0.97269352 | 3 | amp |
| TCGA-13-0723 | 1 | 52956378 | 55561118 | 1.496964897 | 4 | amp |
| TCGA-13-0723 | 1 | 55562112 | 62503744 | 1.163179324 | 3 | amp |
| TCGA-13-0723 | 1 | 62516623 | 62913216 | 1.328687478 | 4 | amp |
| TCGA-13-0723 | 1 | 62914116 | 65270790 | 1.124320192 | 3 | amp |
| TCGA-13-0723 | 1 | 65272842 | 65656572 | 1.380413708 | 4 | amp |
| TCGA-13-0723 | 1 | 65684402 | 67648700 | 1.131326577 | 3 | amp |
| TCGA-13-0723 | 1 | 67666379 | 68614354 | 1.312478076 | 4 | amp |
| TCGA-13-0723 | 1 | 68615834 | 93730477 | 1.09378885 | 3 | amp |
| TCGA-13-0723 | 1 | 93743966 | 94578635 | 1.29818868 | 4 | amp |
| TCGA-13-0723 | 1 | 94586508 | 109172304 | 1.069916157 | 3 | amp |
| TCGA-13-0723 | 1 | 109177690 | 109395329 | 1.363841611 | 4 | amp |
| TCGA-13-0723 | 1 | 109428125 | 109545054 | 1.160675394 | 3 | amp |
| TCGA-13-0723 | 1 | 109547126 | 109804252 | 1.504974981 | 4 | amp |
| TCGA-13-0723 | 1 | 109804413 | 109836925 | 1.870396125 | 5 | amp |
| TCGA-13-0723 | 1 | 109837705 | 113636231 | 1.481368776 | 4 | amp |
| TCGA-13-0723 | 1 | 113636912 | 114397685 | 1.149698527 | 3 | amp |
| TCGA-13-0723 | 1 | 114398951 | 114944107 | 1.351702351 | 4 | amp |
| TCGA-13-0723 | 1 | 114945323 | 115600253 | 1.172760999 | 3 | amp |
| TCGA-13-0723 | 1 | 115601492 | 116563543 | 1.326301644 | 4 | amp |
| TCGA-13-0723 | 1 | 116569458 | 119958233 | 1.123774633 | 3 | amp |
| TCGA-13-0723 | 1 | 119962002 | 120471896 | 1.33323607 | 4 | amp |
| TCGA-13-0723 | 1 | 120478010 | 145368622 | 1.170639192 | 3 | amp |
| TCGA-13-0723 | 1 | 145414739 | 145415892 | 1.334687959 | 4 | amp |
| TCGA-13-0723 | 1 | 145416264 | 145460279 | 2.172774621 | 5 | amp |
| TCGA-13-0723 | 1 | 145473293 | 145754172 | 1.491824066 | 4 | amp |
| TCGA-13-0723 | 1 | 145756394 | 147092882 | 1.128344723 | 3 | amp |
| TCGA-13-0723 | 1 | 147094021 | 147400730 | 1.431221454 | 4 | amp |
| TCGA-13-0723 | 1 | 147408710 | 148890319 | 1.164475002 | 3 | amp |
| TCGA-13-0723 | 1 | 148891515 | 150445863 | 1.500794147 | 4 | amp |
| TCGA-13-0723 | 1 | 150459869 | 150469394 | 2.041365019 | 5 | amp |
| TCGA-13-0723 | 1 | 150469964 | 150485304 | 1.384220703 | 4 | amp |
| TCGA-13-0723 | 1 | 150485707 | 150601977 | 1.919309692 | 5 | amp |
| TCGA-13-0723 | 1 | 150620770 | 154241436 | 1.609034662 | 4 | amp |
| TCGA-13-0723 | 1 | 154242633 | 154480987 | 1.89879241 | 5 | amp |
| TCGA-13-0723 | 1 | 154492754 | 158227551 | 1.71694285 | 4 | amp |
| TCGA-13-0723 | 1 | 158259824 | 158819063 | 1.102335682 | 3 | amp |
| TCGA-13-0723 | 1 | 158906652 | 160970923 | 1.502616121 | 4 | amp |
| TCGA-13-0723 | 1 | 160971018 | 161293483 | 1.878076009 | 5 | amp |
| TCGA-13-0723 | 1 | 161298146 | 162482387 | 1.450925021 | 4 | amp |
| TCGA-13-0723 | 1 | 162482503 | 164326749 | 1.133702216 | 3 | amp |
| TCGA-13-0723 | 1 | 164532451 | 167865940 | 1.349898366 | 4 | amp |
| TCGA-13-0723 | 1 | 167868643 | 168037708 | 1.14856514 | 3 | amp |
| TCGA-13-0723 | 1 | 168044550 | 168282272 | 1.413810189 | 4 | amp |
| TCGA-13-0723 | 1 | 168344720 | 172051066 | 1.148147267 | 3 | amp |
| TCGA-13-0723 | 1 | 172061929 | 172554251 | 1.347051161 | 4 | amp |
| TCGA-13-0723 | 1 | 172554932 | 173780473 | 1.163950313 | 3 | amp |
| TCGA-13-0723 | 1 | 173794310 | 173921287 | 1.326708585 | 4 | amp |
| TCGA-13-0723 | 1 | 173930183 | 174987752 | 1.094095617 | 3 | amp |

| | | | | | | |
|---|---|---|---|---|---|---|
| TCGA-13-0723 | 1 | 174992461 | 175996885 | 1.34262836 | 4 | amp |
| TCGA-13-0723 | 1 | 176012272 | 176809417 | 1.048591898 | 3 | amp |
| TCGA-13-0723 | 1 | 176811492 | 178716471 | 1.35653745 | 4 | amp |
| TCGA-13-0723 | 1 | 178745867 | 178876016 | 1.08913278 | 3 | amp |
| TCGA-13-0723 | 1 | 178885389 | 179307061 | 1.424808844 | 4 | amp |
| TCGA-13-0723 | 1 | 179308566 | 179638553 | 1.185499572 | 3 | amp |
| TCGA-13-0723 | 1 | 179659774 | 180257694 | 1.365094541 | 4 | amp |
| TCGA-13-0723 | 1 | 180283781 | 180833046 | 1.094824826 | 3 | amp |
| TCGA-13-0723 | 1 | 180842888 | 182781379 | 1.443794592 | 4 | amp |
| TCGA-13-0723 | 1 | 182783895 | 182898894 | 1.122832485 | 3 | amp |
| TCGA-13-0723 | 1 | 182908299 | 183387419 | 1.334916223 | 4 | amp |
| TCGA-13-0723 | 1 | 183481927 | 183520372 | 0.991408302 | 3 | amp |
| TCGA-13-0723 | 1 | 183520949 | 183938624 | 1.34133 | 4 | amp |
| TCGA-13-0723 | 1 | 183942666 | 200822632 | 1.041214541 | 3 | amp |
| TCGA-13-0723 | 1 | 200823890 | 201958704 | 1.673425349 | 4 | amp |
| TCGA-13-0723 | 1 | 201965182 | 202288339 | 1.889693764 | 5 | amp |
| TCGA-13-0723 | 1 | 202300905 | 204931320 | 1.543193969 | 4 | amp |
| TCGA-13-0723 | 1 | 204937342 | 205042898 | 1.86873851 | 5 | amp |
| TCGA-13-0723 | 1 | 205052644 | 207236788 | 1.562517943 | 4 | amp |
| TCGA-13-0723 | 1 | 207237085 | 207760940 | 1.140407286 | 3 | amp |
| TCGA-13-0723 | 1 | 207762009 | 210004248 | 1.434295872 | 4 | amp |
| TCGA-13-0723 | 1 | 210004290 | 212553427 | 1.141173104 | 3 | amp |
| TCGA-13-0723 | 1 | 212558541 | 213161949 | 1.298309891 | 4 | amp |
| TCGA-13-0723 | 1 | 213168338 | 220825529 | 1.07671893 | 3 | amp |
| TCGA-13-0723 | 1 | 220826388 | 222721405 | 1.362764611 | 4 | amp |
| TCGA-13-0723 | 1 | 222731997 | 223168335 | 1.074951991 | 3 | amp |
| TCGA-13-0723 | 1 | 223175690 | 223958247 | 1.43781206 | 4 | amp |
| TCGA-13-0723 | 1 | 223959448 | 225607518 | 1.200088002 | 3 | amp |
| TCGA-13-0723 | 1 | 225609758 | 227198791 | 1.545533776 | 4 | amp |
| TCGA-13-0723 | 1 | 227203719 | 227843562 | 1.084270412 | 3 | amp |
| TCGA-13-0723 | 1 | 227922314 | 227935963 | 1.387171908 | 4 | amp |
| TCGA-13-0723 | 1 | 227946640 | 228645562 | 1.856963708 | 5 | amp |
| TCGA-13-0723 | 1 | 228645750 | 230513331 | 1.355799548 | 4 | amp |
| TCGA-13-0723 | 1 | 230795170 | 230819410 | 0.922312711 | 3 | amp |
| TCGA-13-0723 | 1 | 230820786 | 231044557 | 1.397562146 | 4 | amp |
| TCGA-13-0723 | 1 | 231044636 | 231133057 | 1.068833465 | 3 | amp |
| TCGA-13-0723 | 1 | 231155560 | 234546293 | 1.29712253 | 4 | amp |
| TCGA-13-0723 | 1 | 234553904 | 234603441 | 1.010394716 | 3 | amp |
| TCGA-13-0723 | 1 | 234606922 | 235629051 | 1.294770832 | 4 | amp |
| TCGA-13-0723 | 1 | 235634158 | 236760318 | 1.13638053 | 3 | amp |
| TCGA-13-0723 | 1 | 236761168 | 236925959 | 1.385359139 | 4 | amp |
| TCGA-13-0723 | 1 | 236966684 | 245021079 | 1.13695877 | 3 | amp |
| TCGA-13-0723 | 1 | 245021234 | 247049008 | 1.306915817 | 4 | amp |
| TCGA-13-0723 | 1 | 247050465 | 247079750 | 0.915348399 | 3 | amp |
| TCGA-13-0723 | 1 | 247081491 | 247729306 | 1.395761882 | 4 | amp |
| TCGA-13-0723 | 1 | 247737384 | 248802613 | 1.082977611 | 3 | amp |
| TCGA-13-0723 | 1 | 248813228 | 249231325 | 1.433902147 | 4 | amp |
| TCGA-13-0723 | 10 | 92880 | 7791343 | 1.332859046 | 4 | amp |
| TCGA-13-0723 | 10 | 7797958 | 8019304 | 0.942161681 | 3 | amp |
| TCGA-13-0723 | 10 | 8050984 | 11973830 | 1.11867337 | 4 | amp |

| | | | | | | |
|---|---|---|---|---|---|---|
| TCGA-13-0723 | 10 | 11978507 | 12077479 | 0.879414798 | 3 | amp |
| TCGA-13-0723 | 10 | 12123428 | 13166112 | 1.121804071 | 4 | amp |
| TCGA-13-0723 | 10 | 13167342 | 13243537 | 0.979949359 | 3 | amp |
| TCGA-13-0723 | 10 | 13246171 | 13838601 | 1.155919492 | 4 | amp |
| TCGA-13-0723 | 10 | 13852800 | 42681283 | 0.922617544 | 3 | amp |
| TCGA-13-0723 | 10 | 43325669 | 45959790 | 0.907255036 | 3 | amp |
| TCGA-13-0723 | 10 | 50736456 | 50960296 | 0.944933798 | 3 | amp |
| TCGA-13-0723 | 10 | 70980162 | 71124718 | 1.010025344 | 3 | amp |
| TCGA-13-0723 | 10 | 71128279 | 71678890 | 1.137728238 | 4 | amp |
| TCGA-13-0723 | 10 | 71683524 | 72637141 | 0.968655187 | 3 | amp |
| TCGA-13-0723 | 10 | 72643665 | 73579683 | 1.254699091 | 4 | amp |
| TCGA-13-0723 | 10 | 73579954 | 74235076 | 0.974831855 | 3 | amp |
| TCGA-13-0723 | 10 | 75391653 | 75675202 | 1.012432212 | 3 | amp |
| TCGA-13-0723 | 10 | 79552159 | 79744069 | 0.977465482 | 3 | amp |
| TCGA-13-0723 | 10 | 79789053 | 86018449 | 0.924199219 | 3 | amp |
| TCGA-13-0723 | 10 | 88414462 | 88459145 | 1.386495698 | 4 | amp |
| TCGA-13-0723 | 10 | 88466231 | 88813189 | 0.890355155 | 3 | amp |
| TCGA-13-0723 | 10 | 94816676 | 95115528 | 0.962705603 | 3 | amp |
| TCGA-13-0723 | 10 | 98405204 | 100221619 | 0.965506025 | 3 | amp |
| TCGA-13-0723 | 10 | 102040630 | 102746791 | 0.896935586 | 3 | amp |
| TCGA-13-0723 | 10 | 102746839 | 102795870 | 1.386402574 | 4 | amp |
| TCGA-13-0723 | 10 | 102796220 | 104140472 | 0.929665292 | 3 | amp |
| TCGA-13-0723 | 10 | 104140797 | 104240730 | 1.203438629 | 4 | amp |
| TCGA-13-0723 | 10 | 104240848 | 105648925 | 0.92416015 | 3 | amp |
| TCGA-13-0723 | 10 | 105785284 | 105824394 | 0.96059347 | 3 | amp |
| TCGA-13-0723 | 10 | 115987631 | 116307591 | 0.935039256 | 3 | amp |
| TCGA-13-0723 | 10 | 118354188 | 119036784 | 0.881609186 | 3 | amp |
| TCGA-13-0723 | 10 | 120820697 | 121296038 | 0.950848353 | 3 | amp |
| TCGA-13-0723 | 10 | 123255990 | 124338314 | 0.903547792 | 3 | amp |
| TCGA-13-0723 | 10 | 124339075 | 124358631 | 1.180765948 | 4 | amp |
| TCGA-13-0723 | 10 | 124359534 | 127418107 | 0.867023866 | 3 | amp |
| TCGA-13-0723 | 10 | 127705817 | 135516111 | 0.94674081 | 3 | amp |
| TCGA-13-0723 | 11 | 86637 | 3697983 | 1.611553922 | 4 | amp |
| TCGA-13-0723 | 11 | 3700613 | 6263015 | 0.898358974 | 3 | amp |
| TCGA-13-0723 | 11 | 6265168 | 6704560 | 1.476090974 | 4 | amp |
| TCGA-13-0723 | 11 | 6789232 | 11977754 | 1.032812205 | 3 | amp |
| TCGA-13-0723 | 11 | 11985971 | 12284106 | 1.57312463 | 4 | amp |
| TCGA-13-0723 | 11 | 12313582 | 17337037 | 0.919523463 | 3 | amp |
| TCGA-13-0723 | 11 | 17351638 | 17899832 | 1.567759056 | 4 | amp |
| TCGA-13-0723 | 11 | 17980979 | 18638538 | 0.900667774 | 3 | amp |
| TCGA-13-0723 | 11 | 18655684 | 19170925 | 1.590207182 | 4 | amp |
| TCGA-13-0723 | 11 | 19172245 | 44265887 | 0.919078949 | 3 | amp |
| TCGA-13-0723 | 11 | 44288957 | 46515773 | 1.656296531 | 4 | amp |
| TCGA-13-0723 | 11 | 46529710 | 46686513 | 0.890962142 | 3 | amp |
| TCGA-13-0723 | 11 | 46686880 | 46751140 | 1.627295705 | 4 | amp |
| TCGA-13-0723 | 11 | 46760572 | 46907742 | 0.824212531 | 3 | amp |
| TCGA-13-0723 | 11 | 46907859 | 47470540 | 1.498324409 | 4 | amp |
| TCGA-13-0723 | 11 | 47493695 | 57078089 | 0.837434284 | 3 | amp |
| TCGA-13-0723 | 11 | 57079965 | 57317632 | 1.431879863 | 4 | amp |
| TCGA-13-0723 | 11 | 57319756 | 60476308 | 0.852869192 | 3 | amp |

| | | | | | | |
|---|---|---|---|---|---|---|
| TCGA-13-0723 | 11 | 60482490 | 61068462 | 1.381979993 | 4 | amp |
| TCGA-13-0723 | 11 | 61069719 | 61097607 | 0.895070078 | 3 | amp |
| TCGA-13-0723 | 11 | 61098968 | 62760818 | 1.47520459 | 4 | amp |
| TCGA-13-0723 | 11 | 62760847 | 63669845 | 0.905272713 | 3 | amp |
| TCGA-13-0723 | 11 | 63670044 | 64991053 | 1.680535681 | 4 | amp |
| TCGA-13-0723 | 11 | 65000607 | 74079638 | 0.567639122 | 1 | loss |
| TCGA-13-0723 | 11 | 74081898 | 78516552 | 14.36862764 | 5 | amp |
| TCGA-13-0723 | 11 | 79112956 | 84822801 | 0.237969679 | 1 | loss |
| TCGA-13-0723 | 11 | 84996216 | 90602389 | 2.637198242 | 5 | amp |
| TCGA-13-0723 | 11 | 92085244 | 134257557 | 0.392554495 | 1 | loss |
| TCGA-13-0723 | 12 | 73256 | 351959 | 1.523200934 | 4 | amp |
| TCGA-13-0723 | 12 | 352791 | 493377 | 1.045412562 | 3 | amp |
| TCGA-13-0723 | 12 | 495011 | 966430 | 1.410449676 | 4 | amp |
| TCGA-13-0723 | 12 | 968370 | 1553941 | 1.142233113 | 3 | amp |
| TCGA-13-0723 | 12 | 1599207 | 4554743 | 1.471158426 | 4 | amp |
| TCGA-13-0723 | 12 | 4598958 | 4855426 | 1.115347517 | 3 | amp |
| TCGA-13-0723 | 12 | 4870066 | 7480985 | 1.609695124 | 4 | amp |
| TCGA-13-0723 | 12 | 7509980 | 7656323 | 1.04789535 | 3 | amp |
| TCGA-13-0723 | 12 | 7802097 | 9020665 | 1.38066011 | 4 | amp |
| TCGA-13-0723 | 12 | 9020769 | 9349040 | 1.147163766 | 3 | amp |
| TCGA-13-0723 | 12 | 9349163 | 9694480 | 1.389562077 | 4 | amp |
| TCGA-13-0723 | 12 | 9695071 | 31116976 | 1.089128211 | 3 | amp |
| TCGA-13-0723 | 12 | 31131472 | 31577664 | 1.411639744 | 4 | amp |
| TCGA-13-0723 | 12 | 31579172 | 46358009 | 1.053370963 | 3 | amp |
| TCGA-13-0723 | 12 | 48104523 | 48132062 | 1.454036149 | 4 | amp |
| TCGA-13-0723 | 12 | 48132444 | 48390429 | 1.918182257 | 5 | amp |
| TCGA-13-0723 | 12 | 48391245 | 50625530 | 1.574142481 | 4 | amp |
| TCGA-13-0723 | 12 | 50642324 | 52282656 | 1.045320534 | 3 | amp |
| TCGA-13-0723 | 12 | 52282765 | 52841399 | 1.324320363 | 4 | amp |
| TCGA-13-0723 | 12 | 52841551 | 52885532 | 1.089664234 | 3 | amp |
| TCGA-13-0723 | 12 | 52886402 | 54891684 | 1.338956477 | 4 | amp |
| TCGA-13-0723 | 12 | 54893133 | 54936473 | 0.934684721 | 3 | amp |
| TCGA-13-0723 | 12 | 54943622 | 54977073 | 1.476896016 | 4 | amp |
| TCGA-13-0723 | 12 | 55024646 | 56031626 | 1.05748975 | 3 | amp |
| TCGA-13-0723 | 12 | 56075652 | 56537686 | 1.421182377 | 4 | amp |
| TCGA-13-0723 | 12 | 56547669 | 56559532 | 1.887305337 | 5 | amp |
| TCGA-13-0723 | 12 | 56561760 | 56737363 | 1.388321637 | 4 | amp |
| TCGA-13-0723 | 12 | 56737565 | 56845276 | 1.118317935 | 3 | amp |
| TCGA-13-0723 | 12 | 56846805 | 58191781 | 1.462250393 | 4 | amp |
| TCGA-13-0723 | 12 | 58193520 | 58347490 | 1.160748055 | 3 | amp |
| TCGA-13-0723 | 12 | 94965181 | 96397716 | 1.001194756 | 3 | amp |
| TCGA-13-0723 | 12 | 97957579 | 102811711 | 1.169503902 | 3 | amp |
| TCGA-13-0723 | 12 | 102813257 | 104332140 | 1.36408014 | 4 | amp |
| TCGA-13-0723 | 12 | 104332221 | 105583970 | 1.160862572 | 3 | amp |
| TCGA-13-0723 | 12 | 105589022 | 106757723 | 1.366257935 | 4 | amp |
| TCGA-13-0723 | 12 | 106760261 | 108140246 | 1.169023314 | 3 | amp |
| TCGA-13-0723 | 12 | 108145168 | 133779395 | 1.480541639 | 4 | amp |
| TCGA-13-0723 | 13 | 19240876 | 115091796 | 0.945560124 | 3 | amp |
| TCGA-13-0723 | 14 | 19377543 | 77492332 | 0.915190662 | 3 | amp |
| TCGA-13-0723 | 14 | 77571969 | 77723088 | 5.508584594 | 5 | amp |

| | | | | | | |
|---|---|---|---|---|---|---|
| TCGA-13-0723 | 14 | 77732536 | 107283263 | 0.935751203 | 3 | amp |
| TCGA-13-0723 | 15 | 20169886 | 22567092 | 0.902744756 | 3 | amp |
| TCGA-13-0723 | 15 | 23299628 | 23932421 | 0.903077416 | 3 | amp |
| TCGA-13-0723 | 15 | 25299343 | 25334056 | 0.907653076 | 3 | amp |
| TCGA-13-0723 | 15 | 25415850 | 25423985 | 1.008753243 | 3 | amp |
| TCGA-13-0723 | 15 | 25425624 | 25523582 | 1.187712992 | 4 | amp |
| TCGA-13-0723 | 15 | 28566483 | 28798941 | 0.911595092 | 3 | amp |
| TCGA-13-0723 | 15 | 34654754 | 34828149 | 0.918317252 | 3 | amp |
| TCGA-13-0723 | 15 | 40505483 | 40901135 | 1.013083949 | 3 | amp |
| TCGA-13-0723 | 15 | 40942701 | 41165970 | 0.986821092 | 3 | amp |
| TCGA-13-0723 | 15 | 41188086 | 41275287 | 1.244926524 | 4 | amp |
| TCGA-13-0723 | 15 | 41275919 | 41280212 | 0.854180365 | 3 | amp |
| TCGA-13-0723 | 15 | 41648227 | 41770844 | 0.967577429 | 3 | amp |
| TCGA-13-0723 | 15 | 41771258 | 41816189 | 1.164780616 | 4 | amp |
| TCGA-13-0723 | 15 | 41816222 | 41870518 | 0.969492967 | 3 | amp |
| TCGA-13-0723 | 15 | 42067470 | 42104359 | 0.932274543 | 3 | amp |
| TCGA-13-0723 | 15 | 42104662 | 42169613 | 1.152569314 | 4 | amp |
| TCGA-13-0723 | 15 | 42170503 | 42462112 | 0.990101094 | 3 | amp |
| TCGA-13-0723 | 15 | 43017335 | 43038463 | 0.985973163 | 3 | amp |
| TCGA-13-0723 | 15 | 43476440 | 43628913 | 0.950008398 | 3 | amp |
| TCGA-13-0723 | 15 | 43783818 | 43831784 | 0.97468304 | 3 | amp |
| TCGA-13-0723 | 15 | 43875549 | 43910865 | 0.941955753 | 3 | amp |
| TCGA-13-0723 | 15 | 43975384 | 44010327 | 0.933511948 | 3 | amp |
| TCGA-13-0723 | 15 | 44066315 | 44705658 | 0.90140725 | 3 | amp |
| TCGA-13-0723 | 15 | 45120588 | 45460357 | 0.999725261 | 3 | amp |
| TCGA-13-0723 | 15 | 64163110 | 64464216 | 0.890956111 | 3 | amp |
| TCGA-13-0723 | 15 | 64973437 | 65703712 | 0.899864584 | 3 | amp |
| TCGA-13-0723 | 15 | 68473500 | 69329578 | 0.932550933 | 3 | amp |
| TCGA-13-0723 | 15 | 69745962 | 70386952 | 0.914993173 | 3 | amp |
| TCGA-13-0723 | 15 | 72879545 | 72958739 | 1.029286459 | 3 | amp |
| TCGA-13-0723 | 15 | 73991929 | 74369472 | 0.939799158 | 3 | amp |
| TCGA-13-0723 | 15 | 74369542 | 74738619 | 1.098293574 | 4 | amp |
| TCGA-13-0723 | 15 | 74740729 | 75044646 | 0.969043358 | 3 | amp |
| TCGA-13-0723 | 15 | 75045477 | 75144588 | 1.112962913 | 4 | amp |
| TCGA-13-0723 | 15 | 75146320 | 75541748 | 1.023827101 | 3 | amp |
| TCGA-13-0723 | 15 | 75550921 | 75585519 | 1.143768172 | 4 | amp |
| TCGA-13-0723 | 15 | 75586060 | 75676811 | 1.010334132 | 3 | amp |
| TCGA-13-0723 | 15 | 76426533 | 76523757 | 0.970354966 | 3 | amp |
| TCGA-13-0723 | 15 | 77906473 | 78889129 | 1.008731801 | 3 | amp |
| TCGA-13-0723 | 15 | 78893560 | 79172947 | 1.257441285 | 4 | amp |
| TCGA-13-0723 | 15 | 79177246 | 79189426 | 0.884776549 | 3 | amp |
| TCGA-13-0723 | 15 | 79215247 | 79614617 | 1.234250531 | 4 | amp |
| TCGA-13-0723 | 15 | 79748438 | 80430015 | 0.985387033 | 3 | amp |
| TCGA-13-0723 | 15 | 80445376 | 81654661 | 1.156243986 | 4 | amp |
| TCGA-13-0723 | 15 | 81660571 | 82422927 | 1.007824687 | 3 | amp |
| TCGA-13-0723 | 15 | 82574451 | 83449067 | 0.922775308 | 3 | amp |
| TCGA-13-0723 | 15 | 84859539 | 85607688 | 0.894210775 | 3 | amp |
| TCGA-13-0723 | 15 | 85679679 | 86063972 | 0.938200888 | 3 | amp |
| TCGA-13-0723 | 15 | 88576061 | 89762292 | 0.949513013 | 3 | amp |
| TCGA-13-0723 | 15 | 89842993 | 90039803 | 0.931345087 | 3 | amp |

| | | | | | | |
|---|---|---|---|---|---|---|
| TCGA-13-0723 | 15 | 90169109 | 90767264 | 0.925855975 | 3 | amp |
| TCGA-13-0723 | 15 | 90768184 | 90796659 | 1.222912545 | 4 | amp |
| TCGA-13-0723 | 15 | 90798901 | 90902195 | 0.900707766 | 3 | amp |
| TCGA-13-0723 | 15 | 91419443 | 91512884 | 1.113900442 | 4 | amp |
| TCGA-13-0723 | 15 | 95022168 | 99798462 | 0.917547872 | 3 | amp |
| TCGA-13-0723 | 15 | 100230419 | 100943002 | 0.959958201 | 3 | amp |
| TCGA-13-0723 | 15 | 101152406 | 102198083 | 0.967690613 | 3 | amp |
| TCGA-13-0723 | 16 | 66517 | 782396 | 0.948406567 | 3 | amp |
| TCGA-13-0723 | 16 | 789630 | 8993657 | 1.512270662 | 4 | amp |
| TCGA-13-0723 | 16 | 8994376 | 10576139 | 1.045418677 | 3 | amp |
| TCGA-13-0723 | 16 | 10626705 | 11815539 | 1.305642645 | 4 | amp |
| TCGA-13-0723 | 16 | 11824445 | 14743863 | 1.092680672 | 3 | amp |
| TCGA-13-0723 | 16 | 14748844 | 15694587 | 1.198140298 | 4 | amp |
| TCGA-13-0723 | 16 | 15695803 | 15733138 | 0.908083181 | 3 | amp |
| TCGA-13-0723 | 16 | 15758586 | 21413828 | 1.349930217 | 4 | amp |
| TCGA-13-0723 | 16 | 21414029 | 23848771 | 0.97070162 | 3 | amp |
| TCGA-13-0723 | 16 | 24268060 | 25264372 | 0.551074247 | 1 | loss |
| TCGA-13-0723 | 16 | 25266499 | 27268943 | 0.954937686 | 3 | amp |
| TCGA-13-0723 | 16 | 27351499 | 28128830 | 1.318345488 | 4 | amp |
| TCGA-13-0723 | 16 | 28132946 | 28412952 | 1.02427236 | 3 | amp |
| TCGA-13-0723 | 16 | 28414828 | 28606803 | 1.396544763 | 4 | amp |
| TCGA-13-0723 | 16 | 28606813 | 28620222 | 2.181624576 | 5 | amp |
| TCGA-13-0723 | 16 | 28631358 | 29465063 | 1.360218345 | 4 | amp |
| TCGA-13-0723 | 16 | 29465325 | 29486608 | 1.92722993 | 5 | amp |
| TCGA-13-0723 | 16 | 29493799 | 29821418 | 1.272077312 | 4 | amp |
| TCGA-13-0723 | 16 | 29824304 | 30216346 | 1.964134611 | 5 | amp |
| TCGA-13-0723 | 16 | 30225832 | 30364420 | 1.215697075 | 4 | amp |
| TCGA-13-0723 | 16 | 30364494 | 30724733 | 1.857205451 | 5 | amp |
| TCGA-13-0723 | 16 | 30724815 | 31510889 | 1.724768673 | 4 | amp |
| TCGA-13-0723 | 16 | 31511939 | 32177057 | 0.996179082 | 3 | amp |
| TCGA-13-0723 | 16 | 34326294 | 54145870 | 0.512022593 | 1 | loss |
| TCGA-13-0723 | 16 | 55690533 | 56643280 | 0.92892075 | 3 | amp |
| TCGA-13-0723 | 16 | 56659717 | 56717169 | 1.400681206 | 4 | amp |
| TCGA-13-0723 | 16 | 56717825 | 56878525 | 0.924749676 | 3 | amp |
| TCGA-13-0723 | 16 | 56892381 | 58077533 | 1.258851809 | 4 | amp |
| TCGA-13-0723 | 16 | 58147871 | 66884622 | 0.989470037 | 3 | amp |
| TCGA-13-0723 | 16 | 66885353 | 68057133 | 1.306907992 | 4 | amp |
| TCGA-13-0723 | 16 | 68071869 | 69748983 | 1.069818814 | 3 | amp |
| TCGA-13-0723 | 16 | 69751940 | 70530371 | 1.268099389 | 4 | amp |
| TCGA-13-0723 | 16 | 70531056 | 75690196 | 1.028030275 | 3 | amp |
| TCGA-13-0723 | 16 | 77769653 | 81116579 | 0.518543378 | 1 | loss |
| TCGA-13-0723 | 16 | 81529030 | 84199591 | 1.035977842 | 3 | amp |
| TCGA-13-0723 | 16 | 84203383 | 90244214 | 1.243675131 | 4 | amp |
| TCGA-13-0723 | 17 | 63618 | 663505 | 0.974820276 | 3 | amp |
| TCGA-13-0723 | 17 | 681869 | 1560058 | 1.048620306 | 3 | amp |
| TCGA-13-0723 | 17 | 1585399 | 1989226 | 1.006638455 | 3 | amp |
| TCGA-13-0723 | 17 | 2236216 | 2935768 | 1.029396829 | 3 | amp |
| TCGA-13-0723 | 17 | 3402133 | 3917844 | 1.065784853 | 3 | amp |
| TCGA-13-0723 | 17 | 4210306 | 4675425 | 1.071818455 | 3 | amp |
| TCGA-13-0723 | 17 | 4684063 | 4856197 | 1.213640924 | 4 | amp |

| | | | | | | |
|---|---|---|---|---|---|---|
| TCGA-13-0723 | 17 | 4856284 | 5042999 | 1.083536316 | 3 | amp |
| TCGA-13-0723 | 17 | 5386105 | 6406930 | 0.996260473 | 3 | amp |
| TCGA-13-0723 | 17 | 6545053 | 6659473 | 1.030785835 | 3 | amp |
| TCGA-13-0723 | 17 | 6909111 | 7188017 | 1.030093912 | 3 | amp |
| TCGA-13-0723 | 17 | 7188115 | 7370175 | 1.14638975 | 4 | amp |
| TCGA-13-0723 | 17 | 7385273 | 7415689 | 0.944473362 | 3 | amp |
| TCGA-13-0723 | 17 | 7417387 | 7417507 | 1.088505803 | 3 | amp |
| TCGA-13-0723 | 17 | 7453384 | 7489449 | 1.272267522 | 4 | amp |
| TCGA-13-0723 | 17 | 7489992 | 8132247 | 1.05660008 | 3 | amp |
| TCGA-13-0723 | 17 | 8146264 | 8285664 | 1.083316742 | 3 | amp |
| TCGA-13-0723 | 17 | 8633426 | 10235597 | 0.949359452 | 3 | amp |
| TCGA-13-0723 | 17 | 10544340 | 10555026 | 1.03896586 | 3 | amp |
| TCGA-13-0723 | 17 | 12823049 | 12916617 | 0.968020016 | 3 | amp |
| TCGA-13-0723 | 17 | 16703237 | 18457613 | 1.044255582 | 3 | amp |
| TCGA-13-0723 | 17 | 18833811 | 19689444 | 1.023515781 | 3 | amp |
| TCGA-13-0723 | 17 | 19728390 | 19845240 | 1.184460615 | 3 | amp |
| TCGA-13-0723 | 17 | 20328000 | 25621501 | 1.010679278 | 3 | amp |
| TCGA-13-0723 | 17 | 25909661 | 26109146 | 1.086099698 | 3 | amp |
| TCGA-13-0723 | 17 | 26666529 | 26902554 | 1.041384473 | 3 | amp |
| TCGA-13-0723 | 17 | 27030524 | 27621194 | 1.11035429 | 3 | amp |
| TCGA-13-0723 | 17 | 27889511 | 27946849 | 1.176811199 | 3 | amp |
| TCGA-13-0723 | 17 | 28534709 | 28548984 | 1.056183978 | 3 | amp |
| TCGA-13-0723 | 17 | 32690052 | 32965263 | 1.03817195 | 3 | amp |
| TCGA-13-0723 | 17 | 33442250 | 33510643 | 1.017519563 | 3 | amp |
| TCGA-13-0723 | 17 | 34062161 | 34106347 | 1.100780761 | 3 | amp |
| TCGA-13-0723 | 17 | 34189943 | 34313705 | 1.117788718 | 3 | amp |
| TCGA-13-0723 | 17 | 36398200 | 37373437 | 1.029063031 | 3 | amp |
| TCGA-13-0723 | 17 | 37682072 | 37762579 | 1.013330497 | 3 | amp |
| TCGA-13-0723 | 17 | 37785392 | 37903196 | 1.236162057 | 4 | amp |
| TCGA-13-0723 | 17 | 37922034 | 37922754 | 0.950894753 | 3 | amp |
| TCGA-13-0723 | 17 | 38078720 | 38547944 | 1.025437709 | 3 | amp |
| TCGA-13-0723 | 17 | 38568996 | 38715229 | 1.057140662 | 3 | amp |
| TCGA-13-0723 | 17 | 39522679 | 40613090 | 1.065314288 | 3 | amp |
| TCGA-13-0723 | 17 | 40673001 | 41203166 | 1.027239684 | 3 | amp |
| TCGA-13-0723 | 17 | 41276013 | 41561599 | 1.016711384 | 3 | amp |
| TCGA-13-0723 | 17 | 41605848 | 44117278 | 1.054383251 | 3 | amp |
| TCGA-13-0723 | 17 | 45773470 | 46134498 | 1.080064413 | 3 | amp |
| TCGA-13-0723 | 17 | 46606892 | 47656669 | 0.982518189 | 3 | amp |
| TCGA-13-0723 | 17 | 47778785 | 47810117 | 1.00314781 | 3 | amp |
| TCGA-13-0723 | 17 | 47915959 | 48538255 | 1.076787583 | 3 | amp |
| TCGA-13-0723 | 17 | 48538546 | 48695314 | 1.208538362 | 4 | amp |
| TCGA-13-0723 | 17 | 48695387 | 48768566 | 1.01200027 | 3 | amp |
| TCGA-13-0723 | 17 | 55187302 | 56606565 | 0.98806721 | 3 | amp |
| TCGA-13-0723 | 17 | 58085697 | 58236807 | 0.959153315 | 3 | amp |
| TCGA-13-0723 | 17 | 59161816 | 59560928 | 1.023288204 | 3 | amp |
| TCGA-13-0723 | 17 | 60140424 | 60361753 | 0.982920278 | 3 | amp |
| TCGA-13-0723 | 17 | 60689689 | 60866020 | 1.091426419 | 3 | amp |
| TCGA-13-0723 | 17 | 61473076 | 61788201 | 1.018933827 | 3 | amp |
| TCGA-13-0723 | 17 | 61901379 | 62149521 | 1.060274451 | 3 | amp |
| TCGA-13-0723 | 17 | 71084759 | 72349146 | 1.03938167 | 3 | amp |

| | | | | | | |
|---|---|---|---|---|---|---|
| TCGA-13-0723 | 17 | 72349616 | 73942912 | 1.235525484 | 4 | amp |
| TCGA-13-0723 | 17 | 73944314 | 74340844 | 1.04224291 | 3 | amp |
| TCGA-13-0723 | 17 | 74344595 | 74563644 | 1.241728786 | 4 | amp |
| TCGA-13-0723 | 17 | 74568667 | 76430325 | 1.038386296 | 3 | amp |
| TCGA-13-0723 | 17 | 76446285 | 76525784 | 1.209933608 | 4 | amp |
| TCGA-13-0723 | 17 | 76526389 | 77044194 | 1.013401365 | 3 | amp |
| TCGA-13-0723 | 17 | 77073485 | 78092647 | 1.190460694 | 4 | amp |
| TCGA-13-0723 | 17 | 78109236 | 78359024 | 1.044499314 | 3 | amp |
| TCGA-13-0723 | 17 | 78359319 | 80402059 | 1.21230266 | 4 | amp |
| TCGA-13-0723 | 17 | 80402244 | 81188237 | 1.070901587 | 3 | amp |
| TCGA-13-0723 | 18 | 47273 | 909661 | 16.03883788 | 5 | amp |
| TCGA-13-0723 | 18 | 2538921 | 77960823 | 0.905358813 | 3 | amp |
| TCGA-13-0723 | 19 | 71882 | 1612438 | 1.493602213 | 4 | amp |
| TCGA-13-0723 | 19 | 1615251 | 4447644 | 0.869561976 | 3 | amp |
| TCGA-13-0723 | 19 | 4452274 | 6416940 | 1.706192303 | 4 | amp |
| TCGA-13-0723 | 19 | 6416987 | 7833918 | 1.082886125 | 3 | amp |
| TCGA-13-0723 | 19 | 7911346 | 8809085 | 1.320997131 | 4 | amp |
| TCGA-13-0723 | 19 | 8841352 | 24345291 | 0.850155666 | 3 | amp |
| TCGA-13-0723 | 19 | 29698399 | 37442029 | 3.997078217 | 5 | amp |
| TCGA-13-0723 | 19 | 37579964 | 49937976 | 0.951204515 | 3 | amp |
| TCGA-13-0723 | 19 | 49937991 | 50504138 | 1.296499855 | 4 | amp |
| TCGA-13-0723 | 19 | 50510744 | 59110878 | 0.97445647 | 3 | amp |
| TCGA-13-0723 | 2 | 41527 | 905981 | 1.034209955 | 3 | amp |
| TCGA-13-0723 | 2 | 1079152 | 3730717 | 1.209968429 | 4 | amp |
| TCGA-13-0723 | 2 | 3743264 | 10133381 | 1.037100966 | 3 | amp |
| TCGA-13-0723 | 2 | 10135962 | 11315174 | 1.178220558 | 4 | amp |
| TCGA-13-0723 | 2 | 11317848 | 11590254 | 0.913334756 | 3 | amp |
| TCGA-13-0723 | 2 | 11591734 | 11905883 | 1.323956248 | 4 | amp |
| TCGA-13-0723 | 2 | 11907876 | 25046270 | 0.977785636 | 3 | amp |
| TCGA-13-0723 | 2 | 25047205 | 25095633 | 1.351043798 | 4 | amp |
| TCGA-13-0723 | 2 | 25141096 | 25384455 | 0.837648592 | 3 | amp |
| TCGA-13-0723 | 2 | 25387425 | 27668359 | 1.336958223 | 4 | amp |
| TCGA-13-0723 | 2 | 27668570 | 28521401 | 1.02464696 | 3 | amp |
| TCGA-13-0723 | 2 | 28532909 | 29222370 | 1.661962302 | 4 | amp |
| TCGA-13-0723 | 2 | 29225387 | 29297153 | 2.075138595 | 5 | amp |
| TCGA-13-0723 | 2 | 29344200 | 29420577 | 1.223696241 | 4 | amp |
| TCGA-13-0723 | 2 | 29429997 | 29451960 | 1.953669056 | 5 | amp |
| TCGA-13-0723 | 2 | 29455121 | 29606781 | 1.31668325 | 4 | amp |
| TCGA-13-0723 | 2 | 29754731 | 30953698 | 0.97043429 | 3 | amp |
| TCGA-13-0723 | 2 | 30954177 | 31611186 | 1.177965488 | 4 | amp |
| TCGA-13-0723 | 2 | 31620474 | 47220687 | 0.950730463 | 3 | amp |
| TCGA-13-0723 | 2 | 47221424 | 47357426 | 1.37869645 | 4 | amp |
| TCGA-13-0723 | 2 | 47378345 | 64808425 | 0.907764031 | 3 | amp |
| TCGA-13-0723 | 2 | 70516432 | 71361276 | 0.929698053 | 3 | amp |
| TCGA-13-0723 | 2 | 71681082 | 72371349 | 0.97502476 | 3 | amp |
| TCGA-13-0723 | 2 | 73052922 | 73498056 | 0.907562898 | 3 | amp |
| TCGA-13-0723 | 2 | 74448550 | 74653715 | 0.956697513 | 3 | amp |
| TCGA-13-0723 | 2 | 74654287 | 74759853 | 1.197326293 | 4 | amp |
| TCGA-13-0723 | 2 | 74759909 | 75109031 | 0.915450982 | 3 | amp |
| TCGA-13-0723 | 2 | 85049012 | 86335525 | 0.960791075 | 3 | amp |

| | | | | | | |
|---|---|---|---|---|---|---|
| TCGA-13-0723 | 2 | 90273672 | 91940234 | 0.905440928 | 3 | amp |
| TCGA-13-0723 | 2 | 91940354 | 95847890 | 1.266343472 | 4 | amp |
| TCGA-13-0723 | 2 | 95940275 | 96116643 | 2.230908607 | 5 | amp |
| TCGA-13-0723 | 2 | 96116763 | 96523268 | 1.52535439 | 4 | amp |
| TCGA-13-0723 | 2 | 96525595 | 96570745 | 1.005401603 | 3 | amp |
| TCGA-13-0723 | 2 | 96574370 | 99636963 | 1.320454163 | 4 | amp |
| TCGA-13-0723 | 2 | 99651677 | 99863332 | 1.01241891 | 3 | amp |
| TCGA-13-0723 | 2 | 99870641 | 103348884 | 1.200738908 | 4 | amp |
| TCGA-13-0723 | 2 | 103378742 | 105710101 | 0.963041081 | 3 | amp |
| TCGA-13-0723 | 2 | 105713577 | 106994560 | 1.203875395 | 4 | amp |
| TCGA-13-0723 | 2 | 106998567 | 113514832 | 1.065234327 | 3 | amp |
| TCGA-13-0723 | 2 | 113518255 | 114195642 | 1.248931878 | 4 | amp |
| TCGA-13-0723 | 2 | 114198926 | 119699984 | 1.054046686 | 3 | amp |
| TCGA-13-0723 | 2 | 119726696 | 120252183 | 1.293477878 | 4 | amp |
| TCGA-13-0723 | 2 | 120362281 | 121050896 | 0.992798013 | 3 | amp |
| TCGA-13-0723 | 2 | 121106642 | 128378097 | 1.289422217 | 4 | amp |
| TCGA-13-0723 | 2 | 128380743 | 128466478 | 2.218722786 | 5 | amp |
| TCGA-13-0723 | 2 | 128467019 | 128700114 | 1.288135933 | 4 | amp |
| TCGA-13-0723 | 2 | 128703021 | 130872904 | 1.082032778 | 3 | amp |
| TCGA-13-0723 | 2 | 130877527 | 131223405 | 1.37437593 | 4 | amp |
| TCGA-13-0723 | 2 | 131231913 | 131261470 | 0.92352073 | 3 | amp |
| TCGA-13-0723 | 2 | 131266594 | 131374480 | 1.36520311 | 4 | amp |
| TCGA-13-0723 | 2 | 131374604 | 131403920 | 0.837216571 | 3 | amp |
| TCGA-13-0723 | 2 | 131412433 | 131976536 | 1.240606455 | 4 | amp |
| TCGA-13-0723 | 2 | 131981165 | 132010709 | 0.926267493 | 3 | amp |
| TCGA-13-0723 | 2 | 132019238 | 135214376 | 1.184806139 | 4 | amp |
| TCGA-13-0723 | 2 | 135215523 | 159519624 | 1.015192011 | 3 | amp |
| TCGA-13-0723 | 2 | 159519720 | 160085416 | 1.190634072 | 4 | amp |
| TCGA-13-0723 | 2 | 160086085 | 175338132 | 1.013826722 | 3 | amp |
| TCGA-13-0723 | 2 | 175346214 | 175666547 | 1.238520748 | 4 | amp |
| TCGA-13-0723 | 2 | 175673581 | 176860358 | 0.940570399 | 3 | amp |
| TCGA-13-0723 | 2 | 176948050 | 177037043 | 1.424675896 | 4 | amp |
| TCGA-13-0723 | 2 | 177054492 | 201524831 | 0.969797324 | 3 | amp |
| TCGA-13-0723 | 2 | 201526267 | 202082507 | 1.182180903 | 4 | amp |
| TCGA-13-0723 | 2 | 217364652 | 219314062 | 1.034636864 | 3 | amp |
| TCGA-13-0723 | 2 | 219480413 | 220078252 | 0.966856361 | 3 | amp |
| TCGA-13-0723 | 2 | 220078259 | 220101225 | 1.227744157 | 4 | amp |
| TCGA-13-0723 | 2 | 220101787 | 220251898 | 1.060118803 | 3 | amp |
| TCGA-13-0723 | 2 | 220284786 | 220506471 | 1.215725739 | 4 | amp |
| TCGA-13-0723 | 2 | 233271547 | 234389955 | 0.962275681 | 3 | amp |
| TCGA-13-0723 | 2 | 235404404 | 243160772 | 0.9259382 | 3 | amp |
| TCGA-13-0723 | 20 | 139359 | 2622008 | 1.196555837 | 3 | amp |
| TCGA-13-0723 | 20 | 2633891 | 3285198 | 1.335462379 | 4 | amp |
| TCGA-13-0723 | 20 | 3295633 | 3627551 | 0.94506169 | 3 | amp |
| TCGA-13-0723 | 20 | 3649538 | 3675619 | 1.93543689 | 5 | amp |
| TCGA-13-0723 | 20 | 3677203 | 4854779 | 1.432077944 | 4 | amp |
| TCGA-13-0723 | 20 | 4855161 | 17410183 | 1.039957456 | 3 | amp |
| TCGA-13-0723 | 20 | 17417290 | 17640918 | 1.484204221 | 4 | amp |
| TCGA-13-0723 | 20 | 17640980 | 23546727 | 1.090566616 | 3 | amp |
| TCGA-13-0723 | 20 | 23548787 | 25028828 | 1.381149898 | 4 | amp |

| | | | | | | |
|---|---|---|---|---|---|---|
| TCGA-13-0723 | 20 | 25056831 | 29993986 | 1.167342182 | 3 | amp |
| TCGA-13-0723 | 20 | 30028440 | 36718322 | 1.460179243 | 4 | amp |
| TCGA-13-0723 | 20 | 36759412 | 36859785 | 1.975065083 | 5 | amp |
| TCGA-13-0723 | 20 | 36867878 | 39750840 | 1.429156161 | 4 | amp |
| TCGA-13-0723 | 20 | 39751825 | 40034160 | 1.942953182 | 5 | amp |
| TCGA-13-0723 | 20 | 40040723 | 43883201 | 1.60148052 | 4 | amp |
| TCGA-13-0723 | 20 | 43922375 | 44054519 | 1.878746423 | 5 | amp |
| TCGA-13-0723 | 20 | 44108264 | 44434034 | 1.382074072 | 4 | amp |
| TCGA-13-0723 | 20 | 44437728 | 44685223 | 1.959644177 | 5 | amp |
| TCGA-13-0723 | 20 | 44685482 | 44751901 | 1.496642815 | 4 | amp |
| TCGA-13-0723 | 20 | 44755249 | 44996218 | 1.923057557 | 5 | amp |
| TCGA-13-0723 | 20 | 44997478 | 45725825 | 1.432705014 | 4 | amp |
| TCGA-13-0723 | 20 | 45771682 | 47557810 | 2.049292536 | 5 | amp |
| TCGA-13-0723 | 20 | 47558342 | 48494612 | 1.582906808 | 4 | amp |
| TCGA-13-0723 | 20 | 48497456 | 49493203 | 1.878315033 | 5 | amp |
| TCGA-13-0723 | 20 | 49507935 | 58547217 | 1.607148447 | 4 | amp |
| TCGA-13-0723 | 20 | 58557931 | 62926333 | 2.27166182 | 5 | amp |
| TCGA-13-0723 | 21 | 43522271 | 45220812 | 1.188358943 | 4 | amp |
| TCGA-13-0723 | 21 | 45222151 | 48111215 | 2.042896552 | 5 | amp |
| TCGA-13-0723 | 22 | 18570699 | 19376101 | 0.993241401 | 3 | amp |
| TCGA-13-0723 | 22 | 19502190 | 24645496 | 1.027150234 | 3 | amp |
| TCGA-13-0723 | 22 | 30639604 | 31795750 | 1.033553169 | 3 | amp |
| TCGA-13-0723 | 22 | 36591299 | 38870637 | 1.102852045 | 3 | amp |
| TCGA-13-0723 | 22 | 39123926 | 40078703 | 1.192000039 | 3 | amp |
| TCGA-13-0723 | 22 | 41601318 | 44369219 | 1.039937846 | 3 | amp |
| TCGA-13-0723 | 22 | 44371848 | 44515754 | 1.373286804 | 4 | amp |
| TCGA-13-0723 | 22 | 44527321 | 45724375 | 2.091264147 | 5 | amp |
| TCGA-13-0723 | 22 | 45726427 | 45813837 | 1.527018078 | 4 | amp |
| TCGA-13-0723 | 22 | 45818078 | 45970581 | 2.225383766 | 5 | amp |
| TCGA-13-0723 | 22 | 45972816 | 47290770 | 1.646421498 | 4 | amp |
| TCGA-13-0723 | 22 | 47307966 | 47569324 | 1.963245694 | 5 | amp |
| TCGA-13-0723 | 3 | 361444 | 9771403 | 1.018508478 | 3 | amp |
| TCGA-13-0723 | 3 | 9775763 | 9956471 | 1.484777891 | 4 | amp |
| TCGA-13-0723 | 3 | 9958948 | 10219732 | 1.160619517 | 3 | amp |
| TCGA-13-0723 | 3 | 10241984 | 11060393 | 1.390023837 | 4 | amp |
| TCGA-13-0723 | 3 | 11061893 | 12777144 | 1.139676542 | 3 | amp |
| TCGA-13-0723 | 3 | 12778053 | 14562090 | 1.434488715 | 4 | amp |
| TCGA-13-0723 | 3 | 14564484 | 46580732 | 1.038989863 | 3 | amp |
| TCGA-13-0723 | 3 | 46586519 | 47099005 | 1.628358578 | 4 | amp |
| TCGA-13-0723 | 3 | 47103632 | 48436134 | 1.205061483 | 3 | amp |
| TCGA-13-0723 | 3 | 48445854 | 50608769 | 1.575405702 | 4 | amp |
| TCGA-13-0723 | 3 | 50609096 | 51929368 | 1.056887006 | 3 | amp |
| TCGA-13-0723 | 3 | 51930788 | 52582284 | 1.524073 | 4 | amp |
| TCGA-13-0723 | 3 | 52584394 | 52802666 | 1.078585705 | 3 | amp |
| TCGA-13-0723 | 3 | 52811569 | 52861007 | 1.504307003 | 4 | amp |
| TCGA-13-0723 | 3 | 52861100 | 102196494 | 1.037672521 | 3 | amp |
| TCGA-13-0723 | 3 | 105086228 | 120500343 | 2.846331965 | 5 | amp |
| TCGA-13-0723 | 3 | 120628399 | 126190914 | 1.032703759 | 3 | amp |
| TCGA-13-0723 | 3 | 126190921 | 129304979 | 1.314255766 | 4 | amp |
| TCGA-13-0723 | 3 | 129370270 | 182842055 | 1.057071976 | 3 | amp |

| | | | | | | |
|---|---|---|---|---|---|---|
| TCGA-13-0723 | 3 | 182853439 | 186038274 | 1.496354224 | 4 | amp |
| TCGA-13-0723 | 3 | 186256440 | 195256708 | 1.197829138 | 3 | amp |
| TCGA-13-0723 | 3 | 195295768 | 197955154 | 1.377946049 | 4 | amp |
| TCGA-13-0723 | 4 | 53323 | 438232 | 0.985111686 | 3 | amp |
| TCGA-13-0723 | 4 | 466320 | 2160969 | 1.292473925 | 4 | amp |
| TCGA-13-0723 | 4 | 2172393 | 2231011 | 0.927667512 | 3 | amp |
| TCGA-13-0723 | 4 | 2233650 | 2834806 | 1.199710427 | 4 | amp |
| TCGA-13-0723 | 4 | 2877589 | 2909580 | 0.962674992 | 3 | amp |
| TCGA-13-0723 | 4 | 2910226 | 3117221 | 1.254621947 | 4 | amp |
| TCGA-13-0723 | 4 | 3117780 | 3190868 | 1.010569873 | 3 | amp |
| TCGA-13-0723 | 4 | 3201442 | 4270345 | 1.352593414 | 4 | amp |
| TCGA-13-0723 | 4 | 4275292 | 4307947 | 0.856382152 | 3 | amp |
| TCGA-13-0723 | 4 | 4314744 | 9370156 | 1.275665713 | 4 | amp |
| TCGA-13-0723 | 4 | 9370395 | 9705556 | 0.99793196 | 3 | amp |
| TCGA-13-0723 | 4 | 9706485 | 10086170 | 1.15343123 | 4 | amp |
| TCGA-13-0723 | 4 | 10099304 | 17816998 | 0.942757158 | 3 | amp |
| TCGA-13-0723 | 4 | 24557833 | 44685353 | 0.927295291 | 3 | amp |
| TCGA-13-0723 | 4 | 47942772 | 57888424 | 0.900972322 | 3 | amp |
| TCGA-13-0723 | 4 | 71250194 | 71889469 | 0.930074944 | 3 | amp |
| TCGA-13-0723 | 4 | 74000787 | 85676605 | 0.91260389 | 3 | amp |
| TCGA-13-0723 | 4 | 87662757 | 104074425 | 0.888517443 | 3 | amp |
| TCGA-13-0723 | 4 | 108940638 | 122765178 | 0.903998918 | 3 | amp |
| TCGA-13-0723 | 4 | 128637475 | 142145871 | 0.915684764 | 3 | amp |
| TCGA-13-0723 | 4 | 144801552 | 151604905 | 0.916227329 | 3 | amp |
| TCGA-13-0723 | 4 | 151935566 | 154710036 | 0.961622181 | 3 | amp |
| TCGA-13-0723 | 4 | 159051930 | 166234573 | 0.89397955 | 3 | amp |
| TCGA-13-0723 | 4 | 169176820 | 184628146 | 0.884553943 | 3 | amp |
| TCGA-13-0723 | 4 | 184629523 | 185553126 | 1.125698442 | 4 | amp |
| TCGA-13-0723 | 4 | 185553365 | 190884325 | 0.980301177 | 3 | amp |
| TCGA-13-0723 | 4 | 190903651 | 190990418 | 1.175898892 | 4 | amp |
| TCGA-13-0723 | 5 | 143116 | 50137910 | 1.578081232 | 4 | amp |
| TCGA-13-0723 | 5 | 131302046 | 137506237 | 0.878069047 | 3 | amp |
| TCGA-13-0723 | 5 | 137506500 | 142435681 | 1.165899041 | 4 | amp |
| TCGA-13-0723 | 5 | 142437169 | 148016624 | 0.9436776 | 3 | amp |
| TCGA-13-0723 | 5 | 148206325 | 151131362 | 1.284757691 | 4 | amp |
| TCGA-13-0723 | 5 | 151166138 | 154202144 | 0.879279259 | 3 | amp |
| TCGA-13-0723 | 5 | 169267748 | 169468163 | 0.960384634 | 3 | amp |
| TCGA-13-0723 | 5 | 169468956 | 180899507 | 1.25061118 | 4 | amp |
| TCGA-13-0723 | 6 | 105907 | 27440129 | 1.009063898 | 3 | amp |
| TCGA-13-0723 | 6 | 27775253 | 27861670 | 1.33716465 | 4 | amp |
| TCGA-13-0723 | 6 | 27879020 | 29693863 | 1.049765934 | 3 | amp |
| TCGA-13-0723 | 6 | 29693920 | 32190615 | 1.413088519 | 4 | amp |
| TCGA-13-0723 | 6 | 32190735 | 32798250 | 1.062384449 | 3 | amp |
| TCGA-13-0723 | 6 | 32798368 | 33371945 | 1.486348171 | 4 | amp |
| TCGA-13-0723 | 6 | 33372568 | 33391434 | 1.866575777 | 5 | amp |
| TCGA-13-0723 | 6 | 33393567 | 34558419 | 1.45672629 | 4 | amp |
| TCGA-13-0723 | 6 | 34574296 | 35097018 | 1.06817931 | 3 | amp |
| TCGA-13-0723 | 6 | 35103879 | 35543725 | 1.319892824 | 4 | amp |
| TCGA-13-0723 | 6 | 35544710 | 36075438 | 1.030372175 | 3 | amp |
| TCGA-13-0723 | 6 | 36076130 | 37186828 | 1.250222765 | 4 | amp |

| | | | | | | |
|---|---|---|---|---|---|---|
| TCGA-13-0723 | 6 | 37237337 | 38994502 | 1.020446236 | 3 | amp |
| TCGA-13-0723 | 6 | 38997844 | 39353482 | 1.268761305 | 4 | amp |
| TCGA-13-0723 | 6 | 39387661 | 39682564 | 0.872913127 | 3 | amp |
| TCGA-13-0723 | 6 | 39688472 | 42237353 | 1.261232827 | 4 | amp |
| TCGA-13-0723 | 6 | 42541391 | 42646461 | 0.960269435 | 3 | amp |
| TCGA-13-0723 | 6 | 42647455 | 44344845 | 1.428621556 | 4 | amp |
| TCGA-13-0723 | 6 | 44357966 | 52962633 | 0.954454159 | 3 | amp |
| TCGA-13-0723 | 6 | 52992923 | 53883917 | 1.237353017 | 4 | amp |
| TCGA-13-0723 | 6 | 53986202 | 73787252 | 1.080153617 | 3 | amp |
| TCGA-13-0723 | 6 | 73787427 | 74491071 | 1.283998926 | 4 | amp |
| TCGA-13-0723 | 6 | 74492287 | 88294480 | 1.092125085 | 3 | amp |
| TCGA-13-0723 | 6 | 88299567 | 90411821 | 1.244191456 | 4 | amp |
| TCGA-13-0723 | 6 | 90415724 | 90486426 | 1.094500145 | 3 | amp |
| TCGA-13-0723 | 6 | 90489865 | 90661624 | 1.284693258 | 4 | amp |
| TCGA-13-0723 | 6 | 90718261 | 105291195 | 1.070600387 | 3 | amp |
| TCGA-13-0723 | 6 | 105297008 | 108071072 | 1.288017742 | 4 | amp |
| TCGA-13-0723 | 6 | 108076748 | 108234660 | 1.024931287 | 3 | amp |
| TCGA-13-0723 | 6 | 108242996 | 109748387 | 1.233720084 | 4 | amp |
| TCGA-13-0723 | 6 | 109752303 | 109773825 | 2.157343996 | 5 | amp |
| TCGA-13-0723 | 6 | 109774457 | 109797479 | 1.233955259 | 4 | amp |
| TCGA-13-0723 | 6 | 109797929 | 111737700 | 1.100197345 | 3 | amp |
| TCGA-13-0723 | 6 | 111880530 | 112375617 | 1.352374847 | 4 | amp |
| TCGA-13-0723 | 6 | 112381186 | 134323320 | 1.071940115 | 3 | amp |
| TCGA-13-0723 | 6 | 134327860 | 135286482 | 1.315827654 | 4 | amp |
| TCGA-13-0723 | 6 | 135287418 | 137219389 | 1.104208208 | 3 | amp |
| TCGA-13-0723 | 6 | 137234569 | 142384219 | 1.229851684 | 4 | amp |
| TCGA-13-0723 | 6 | 142396745 | 148808860 | 1.073758189 | 3 | amp |
| TCGA-13-0723 | 6 | 148835375 | 152694382 | 1.274714255 | 4 | amp |
| TCGA-13-0723 | 6 | 152697484 | 152847322 | 1.105130595 | 3 | amp |
| TCGA-13-0723 | 6 | 152861035 | 160969720 | 1.306051332 | 4 | amp |
| TCGA-13-0723 | 6 | 160977016 | 161513228 | 1.117916652 | 3 | amp |
| TCGA-13-0723 | 6 | 161513988 | 162622343 | 1.311531173 | 4 | amp |
| TCGA-13-0723 | 6 | 162683466 | 165989977 | 1.097967205 | 3 | amp |
| TCGA-13-0723 | 6 | 166571759 | 168264524 | 1.409535655 | 4 | amp |
| TCGA-13-0723 | 6 | 168264952 | 168312186 | 1.025340237 | 3 | amp |
| TCGA-13-0723 | 6 | 168314770 | 169067169 | 1.394775847 | 4 | amp |
| TCGA-13-0723 | 6 | 169619843 | 169628416 | 2.338677577 | 5 | amp |
| TCGA-13-0723 | 6 | 169629660 | 171055029 | 1.186859921 | 4 | amp |
| TCGA-13-0723 | 7 | 540048 | 6970595 | 1.359373536 | 4 | amp |
| TCGA-13-0723 | 7 | 7118571 | 26894516 | 0.931863863 | 3 | amp |
| TCGA-13-0723 | 7 | 26903924 | 27497851 | 1.40051861 | 4 | amp |
| TCGA-13-0723 | 7 | 27565761 | 30465349 | 0.967794728 | 3 | amp |
| TCGA-13-0723 | 7 | 30468941 | 31611818 | 1.280491611 | 4 | amp |
| TCGA-13-0723 | 7 | 31614134 | 34192914 | 0.971060811 | 3 | amp |
| TCGA-13-0723 | 7 | 34697947 | 39504332 | 1.768622094 | 4 | amp |
| TCGA-13-0723 | 7 | 39610045 | 41729666 | 0.972009958 | 3 | amp |
| TCGA-13-0723 | 7 | 41729723 | 47867126 | 1.276822614 | 4 | amp |
| TCGA-13-0723 | 7 | 47868990 | 56065160 | 1.045833063 | 3 | amp |
| TCGA-13-0723 | 7 | 56066672 | 76671422 | 1.33527487 | 4 | amp |
| TCGA-13-0723 | 7 | 76671547 | 97788753 | 0.869481339 | 3 | amp |

| | | | | | | |
|---|---|---|---|---|---|---|
| TCGA-13-0723 | 7 | 97800769 | 99235946 | 1.29787055 | 4 | amp |
| TCGA-13-0723 | 7 | 99245915 | 99461359 | 0.927184593 | 3 | amp |
| TCGA-13-0723 | 7 | 99463516 | 102343959 | 1.480100796 | 4 | amp |
| TCGA-13-0723 | 7 | 102453763 | 105429161 | 0.919877778 | 3 | amp |
| TCGA-13-0723 | 7 | 105516231 | 105673170 | 1.256422953 | 4 | amp |
| TCGA-13-0723 | 7 | 105732197 | 127569431 | 0.875675526 | 3 | amp |
| TCGA-13-0723 | 7 | 127630994 | 129008423 | 1.258309674 | 4 | amp |
| TCGA-13-0723 | 7 | 129019474 | 131172546 | 1.045288544 | 3 | amp |
| TCGA-13-0723 | 7 | 131189048 | 132169793 | 1.209907257 | 4 | amp |
| TCGA-13-0723 | 7 | 132174021 | 140082341 | 1.023402805 | 3 | amp |
| TCGA-13-0723 | 7 | 140107426 | 140398094 | 1.197879458 | 4 | amp |
| TCGA-13-0723 | 7 | 140402587 | 142423720 | 0.968417595 | 3 | amp |
| TCGA-13-0723 | 7 | 142428467 | 143531011 | 1.269438966 | 4 | amp |
| TCGA-13-0723 | 7 | 143551105 | 147914677 | 1.033670794 | 3 | amp |
| TCGA-13-0723 | 7 | 147926684 | 151664634 | 1.418386138 | 4 | amp |
| TCGA-13-0723 | 7 | 151667969 | 158935247 | 1.015285219 | 3 | amp |
| TCGA-13-0723 | 8 | 21895069 | 22021603 | 0.878465095 | 3 | amp |
| TCGA-13-0723 | 8 | 28827497 | 28908723 | 0.989516193 | 3 | amp |
| TCGA-13-0723 | 8 | 28950208 | 31077585 | 1.467028929 | 4 | amp |
| TCGA-13-0723 | 8 | 41354983 | 43212089 | 1.785395864 | 4 | amp |
| TCGA-13-0723 | 8 | 57218098 | 62578271 | 0.792629061 | 3 | amp |
| TCGA-13-0723 | 8 | 94745575 | 98155449 | 0.944600266 | 3 | amp |
| TCGA-13-0723 | 8 | 98288755 | 104664954 | 1.127028625 | 4 | amp |
| TCGA-13-0723 | 8 | 104709298 | 110985051 | 0.925064893 | 3 | amp |
| TCGA-13-0723 | 8 | 114388905 | 120818696 | 0.894497869 | 3 | amp |
| TCGA-13-0723 | 8 | 120831545 | 121327858 | 1.44552187 | 4 | amp |
| TCGA-13-0723 | 8 | 121328119 | 124142625 | 0.979759092 | 3 | amp |
| TCGA-13-0723 | 8 | 124146309 | 126017976 | 1.130356742 | 4 | amp |
| TCGA-13-0723 | 8 | 126019621 | 126445884 | 0.940455638 | 3 | amp |
| TCGA-13-0723 | 8 | 126448240 | 134063170 | 1.476224813 | 4 | amp |
| TCGA-13-0723 | 8 | 134072284 | 144413511 | 0.854037526 | 3 | amp |
| TCGA-13-0723 | 8 | 144661890 | 145781045 | 1.156332355 | 4 | amp |
| TCGA-13-0723 | 8 | 145830899 | 146279593 | 0.924724305 | 3 | amp |
| TCGA-13-0723 | 9 | 17322 | 52533 | 1.249483999 | 3 | amp |
| TCGA-13-0723 | 9 | 116709 | 44078664 | 1.293678807 | 4 | amp |
| TCGA-13-0723 | 9 | 44080010 | 69083103 | 1.18487911 | 3 | amp |
| TCGA-13-0723 | 9 | 69112797 | 141071671 | 1.401011307 | 4 | amp |
| TCGA-13-0723 | X | 7137661 | 19380971 | 0.500576569 | 1 | loss |
| TCGA-13-0723 | X | 23934329 | 39922341 | 0.439089871 | 1 | loss |
| TCGA-13-0723 | X | 44970581 | 46918503 | 0.922943662 | 3 | amp |
| TCGA-13-0723 | X | 46940504 | 47518415 | 1.316568844 | 4 | amp |
| TCGA-13-0723 | X | 47528353 | 49089818 | 1.033662512 | 3 | amp |
| TCGA-13-0723 | X | 70320668 | 70517796 | 0.957493025 | 3 | amp |
| TCGA-13-0723 | X | 119760002 | 120119075 | 0.931857525 | 3 | amp |
| TCGA-13-0723 | X | 134854733 | 134992378 | 0.928549448 | 3 | amp |
| TCGA-13-0723 | X | 151813961 | 152722700 | 0.954823238 | 3 | amp |
| TCGA-13-0723 | X | 152728038 | 153880953 | 1.535894853 | 4 | amp |
| TCGA-13-0723 | X | 153906370 | 154774966 | 0.902711319 | 3 | amp |
| TCGA-13-0723 | X | 155003486 | 155252520 | 1.547005549 | 4 | amp |
| TCGA-13-0724 | 1 | 16834 | 741285 | 1.087374725 | 3 | amp |

| | | | | | | |
|---|---|---|---|---|---|---|
| TCGA-13-0724 | 1 | 745438 | 10075924 | 1.48729984 | 4 | amp |
| TCGA-13-0724 | 1 | 10093680 | 10425766 | 1.065790164 | 3 | amp |
| TCGA-13-0724 | 1 | 10428500 | 12302668 | 1.523663036 | 4 | amp |
| TCGA-13-0724 | 1 | 12304277 | 14113075 | 1.128347002 | 3 | amp |
| TCGA-13-0724 | 1 | 14142861 | 16875383 | 1.560256212 | 4 | amp |
| TCGA-13-0724 | 1 | 16890379 | 16918548 | 0.882124905 | 3 | amp |
| TCGA-13-0724 | 1 | 16945531 | 19408074 | 1.463680951 | 4 | amp |
| TCGA-13-0724 | 1 | 19410974 | 19550096 | 1.014692051 | 3 | amp |
| TCGA-13-0724 | 1 | 19553779 | 21053590 | 1.300511068 | 4 | amp |
| TCGA-13-0724 | 1 | 21071256 | 21809937 | 1.072043373 | 3 | amp |
| TCGA-13-0724 | 1 | 21880514 | 21935445 | 1.731994026 | 4 | amp |
| TCGA-13-0724 | 1 | 21936013 | 21978046 | 2.424081497 | 5 | amp |
| TCGA-13-0724 | 1 | 21978070 | 22016649 | 1.386347635 | 4 | amp |
| TCGA-13-0724 | 1 | 22021516 | 22084321 | 0.968316009 | 3 | amp |
| TCGA-13-0724 | 1 | 22138896 | 23371009 | 1.464476291 | 4 | amp |
| TCGA-13-0724 | 1 | 23376826 | 23724486 | 1.055454922 | 3 | amp |
| TCGA-13-0724 | 1 | 23735127 | 24700305 | 1.276716955 | 4 | amp |
| TCGA-13-0724 | 1 | 24706107 | 25812314 | 1.132701735 | 3 | amp |
| TCGA-13-0724 | 1 | 25815611 | 26774160 | 1.430796512 | 4 | amp |
| TCGA-13-0724 | 1 | 26781101 | 28948629 | 1.00614138 | 3 | amp |
| TCGA-13-0724 | 1 | 29010068 | 34090300 | 1.38958451 | 4 | amp |
| TCGA-13-0724 | 1 | 34090602 | 36521351 | 1.086983767 | 3 | amp |
| TCGA-13-0724 | 1 | 36550450 | 39696944 | 1.30177613 | 4 | amp |
| TCGA-13-0724 | 1 | 39715670 | 43166688 | 1.13603359 | 3 | amp |
| TCGA-13-0724 | 1 | 43203851 | 47080755 | 1.323650447 | 4 | amp |
| TCGA-13-0724 | 1 | 47101424 | 54389658 | 1.110721755 | 3 | amp |
| TCGA-13-0724 | 1 | 54394018 | 55538092 | 1.390839906 | 4 | amp |
| TCGA-13-0724 | 1 | 55538436 | 76388084 | 0.93809212 | 3 | amp |
| TCGA-13-0724 | 1 | 91973392 | 94674917 | 0.977165162 | 3 | amp |
| TCGA-13-0724 | 1 | 118640263 | 120269735 | 1.000205364 | 3 | amp |
| TCGA-13-0724 | 1 | 120277202 | 121128477 | 1.364173881 | 4 | amp |
| TCGA-13-0724 | 1 | 143720986 | 145415892 | 1.442290313 | 4 | amp |
| TCGA-13-0724 | 1 | 145416264 | 145608711 | 2.846341785 | 5 | amp |
| TCGA-13-0724 | 1 | 145609142 | 147086440 | 1.720429244 | 4 | amp |
| TCGA-13-0724 | 1 | 147087562 | 147554034 | 2.247458272 | 5 | amp |
| TCGA-13-0724 | 1 | 147554565 | 149876777 | 1.422040981 | 4 | amp |
| TCGA-13-0724 | 1 | 149877361 | 151400527 | 2.808698436 | 5 | amp |
| TCGA-13-0724 | 1 | 151400563 | 151414738 | 1.683903484 | 4 | amp |
| TCGA-13-0724 | 1 | 151490951 | 152671717 | 2.689767692 | 5 | amp |
| TCGA-13-0724 | 1 | 152681521 | 153391822 | 1.103472665 | 3 | amp |
| TCGA-13-0724 | 1 | 153409465 | 153995805 | 1.348860304 | 4 | amp |
| TCGA-13-0724 | 1 | 153997984 | 154574958 | 1.165453093 | 3 | amp |
| TCGA-13-0724 | 1 | 154575010 | 155263170 | 1.401964887 | 4 | amp |
| TCGA-13-0724 | 1 | 155263184 | 155792291 | 1.092338627 | 3 | amp |
| TCGA-13-0724 | 1 | 155796603 | 156170310 | 3.512733178 | 5 | amp |
| TCGA-13-0724 | 1 | 156177590 | 157494387 | 1.684505242 | 4 | amp |
| TCGA-13-0724 | 1 | 157497365 | 157773947 | 1.11389299 | 3 | amp |
| TCGA-13-0724 | 1 | 157776841 | 158227315 | 1.502792434 | 4 | amp |
| TCGA-13-0724 | 1 | 158227431 | 159019425 | 1.025573355 | 3 | amp |
| TCGA-13-0724 | 1 | 159021408 | 160456580 | 1.33788114 | 4 | amp |

| | | | | | | |
|---|---|---|---|---|---|---|
| TCGA-13-0724 | 1 | 160456869 | 160802396 | 1.10729399 | 3 | amp |
| TCGA-13-0724 | 1 | 160803813 | 161736251 | 1.489249249 | 4 | amp |
| TCGA-13-0724 | 1 | 161748011 | 165664681 | 1.126390048 | 3 | amp |
| TCGA-13-0724 | 1 | 165697248 | 168201174 | 1.270221075 | 4 | amp |
| TCGA-13-0724 | 1 | 168204319 | 179638553 | 1.086519844 | 3 | amp |
| TCGA-13-0724 | 1 | 179659774 | 182781379 | 1.302137154 | 4 | amp |
| TCGA-13-0724 | 1 | 182783895 | 200827214 | 0.983522631 | 3 | amp |
| TCGA-13-0724 | 1 | 200867388 | 207244945 | 1.546051246 | 4 | amp |
| TCGA-13-0724 | 1 | 207245482 | 207966961 | 1.028135469 | 3 | amp |
| TCGA-13-0724 | 1 | 207975180 | 210004410 | 1.40055117 | 4 | amp |
| TCGA-13-0724 | 1 | 210006466 | 225598136 | 1.075324994 | 3 | amp |
| TCGA-13-0724 | 1 | 225599000 | 229584964 | 1.477747343 | 4 | amp |
| TCGA-13-0724 | 1 | 229586251 | 249231325 | 1.132723054 | 3 | amp |
| TCGA-13-0724 | 10 | 92880 | 3200348 | 1.266354613 | 4 | amp |
| TCGA-13-0724 | 10 | 3201049 | 4889440 | 0.991316236 | 3 | amp |
| TCGA-13-0724 | 10 | 5765599 | 5965681 | 0.52543497 | 1 | loss |
| TCGA-13-0724 | 10 | 7791103 | 12077479 | 0.538567795 | 1 | loss |
| TCGA-13-0724 | 10 | 13900789 | 35426845 | 0.477693903 | 1 | loss |
| TCGA-13-0724 | 10 | 35484069 | 35858159 | 0.995301915 | 3 | amp |
| TCGA-13-0724 | 10 | 37430631 | 38667006 | 0.498247842 | 1 | loss |
| TCGA-13-0724 | 10 | 43325669 | 45959790 | 0.872260843 | 3 | amp |
| TCGA-13-0724 | 10 | 46959933 | 47934003 | 0.897500794 | 3 | amp |
| TCGA-13-0724 | 10 | 48218769 | 49236682 | 0.91250627 | 3 | amp |
| TCGA-13-0724 | 10 | 70706066 | 74128152 | 0.923182141 | 3 | amp |
| TCGA-13-0724 | 10 | 75391653 | 75676354 | 0.964151474 | 3 | amp |
| TCGA-13-0724 | 10 | 76602535 | 76803784 | 0.937145089 | 3 | amp |
| TCGA-13-0724 | 10 | 76818022 | 76989520 | 1.23745292 | 4 | amp |
| TCGA-13-0724 | 10 | 78846208 | 89272989 | 0.885495583 | 3 | amp |
| TCGA-13-0724 | 10 | 94816676 | 95260019 | 0.893862847 | 3 | amp |
| TCGA-13-0724 | 10 | 98408393 | 100221619 | 0.930301035 | 3 | amp |
| TCGA-13-0724 | 10 | 102721591 | 105817985 | 0.925275074 | 3 | amp |
| TCGA-13-0724 | 10 | 105985215 | 106124688 | 0.930558645 | 3 | amp |
| TCGA-13-0724 | 10 | 114912055 | 115366048 | 0.877618724 | 3 | amp |
| TCGA-13-0724 | 10 | 115987631 | 116100645 | 0.923141301 | 3 | amp |
| TCGA-13-0724 | 10 | 120833233 | 121286996 | 0.949805156 | 3 | amp |
| TCGA-13-0724 | 10 | 124360462 | 124374867 | 1.227135937 | 4 | amp |
| TCGA-13-0724 | 10 | 127456066 | 135516111 | 0.91831354 | 3 | amp |
| TCGA-13-0724 | 11 | 86637 | 2970551 | 0.958327288 | 3 | amp |
| TCGA-13-0724 | 11 | 6591830 | 6655255 | 0.933594357 | 3 | amp |
| TCGA-13-0724 | 11 | 11988452 | 13424829 | 1.203864184 | 4 | amp |
| TCGA-13-0724 | 11 | 13427184 | 17337037 | 0.996202074 | 3 | amp |
| TCGA-13-0724 | 11 | 17351638 | 17899832 | 1.307721764 | 4 | amp |
| TCGA-13-0724 | 11 | 17980979 | 18638538 | 1.049033246 | 3 | amp |
| TCGA-13-0724 | 11 | 18655684 | 18956386 | 1.221811886 | 4 | amp |
| TCGA-13-0724 | 11 | 19076882 | 20907097 | 1.012725725 | 3 | amp |
| TCGA-13-0724 | 11 | 22296083 | 22696607 | 1.041471428 | 3 | amp |
| TCGA-13-0724 | 11 | 31811453 | 44265887 | 0.983656481 | 3 | amp |
| TCGA-13-0724 | 11 | 44288957 | 45889352 | 1.357839081 | 4 | amp |
| TCGA-13-0724 | 11 | 45891032 | 45992972 | 1.798417539 | 5 | amp |
| TCGA-13-0724 | 11 | 46001214 | 46105803 | 0.967393764 | 3 | amp |

| | | | | | | |
|---|---|---|---|---|---|---|
| TCGA-13-0724 | 11 | 46321479 | 46431931 | 1.830355593 | 5 | amp |
| TCGA-13-0724 | 11 | 46439412 | 46761006 | 1.471595697 | 4 | amp |
| TCGA-13-0724 | 11 | 46765513 | 46832762 | 0.911682459 | 3 | amp |
| TCGA-13-0724 | 11 | 46837731 | 47823542 | 1.284973655 | 4 | amp |
| TCGA-13-0724 | 11 | 47824978 | 50252738 | 1.045805329 | 3 | amp |
| TCGA-13-0724 | 11 | 55370902 | 57004506 | 1.176434493 | 3 | amp |
| TCGA-13-0724 | 11 | 57067983 | 57440674 | 1.674473175 | 4 | amp |
| TCGA-13-0724 | 11 | 57449834 | 57506742 | 1.088518101 | 3 | amp |
| TCGA-13-0724 | 11 | 60709471 | 62782443 | 1.013181448 | 3 | amp |
| TCGA-13-0724 | 11 | 63523552 | 63965075 | 0.996237704 | 3 | amp |
| TCGA-13-0724 | 11 | 63965235 | 64011530 | 1.235922688 | 4 | amp |
| TCGA-13-0724 | 11 | 64012169 | 64939003 | 1.014944775 | 3 | amp |
| TCGA-13-0724 | 11 | 64939352 | 65168386 | 1.318606417 | 4 | amp |
| TCGA-13-0724 | 11 | 65172316 | 67120277 | 1.125974325 | 3 | amp |
| TCGA-13-0724 | 11 | 67120731 | 67816774 | 1.387255739 | 4 | amp |
| TCGA-13-0724 | 11 | 67817892 | 73718144 | 1.023259911 | 3 | amp |
| TCGA-13-0724 | 11 | 74407526 | 76164454 | 0.954542661 | 3 | amp |
| TCGA-13-0724 | 11 | 76750546 | 76940309 | 1.149205398 | 3 | amp |
| TCGA-13-0724 | 11 | 113236924 | 113577004 | 1.085539635 | 3 | amp |
| TCGA-13-0724 | 11 | 113816591 | 114318634 | 0.997496202 | 3 | amp |
| TCGA-13-0724 | 11 | 116640801 | 116741141 | 1.065885259 | 3 | amp |
| TCGA-13-0724 | 11 | 117049968 | 118047171 | 1.051450336 | 3 | amp |
| TCGA-13-0724 | 11 | 118263411 | 119170527 | 1.018126762 | 3 | amp |
| TCGA-13-0724 | 11 | 119180550 | 119998280 | 1.191331893 | 4 | amp |
| TCGA-13-0724 | 11 | 119999095 | 120175949 | 1.001609885 | 3 | amp |
| TCGA-13-0724 | 11 | 120979853 | 121421396 | 0.934869744 | 3 | amp |
| TCGA-13-0724 | 11 | 124615350 | 125499380 | 0.950346559 | 3 | amp |
| TCGA-13-0724 | 11 | 126131289 | 134031790 | 0.904242899 | 3 | amp |
| TCGA-13-0724 | 11 | 134095007 | 134252931 | 0.971066468 | 3 | amp |
| TCGA-13-0724 | 12 | 73256 | 344436 | 1.249767813 | 4 | amp |
| TCGA-13-0724 | 12 | 346239 | 551132 | 0.906539839 | 3 | amp |
| TCGA-13-0724 | 12 | 644293 | 772698 | 1.331598015 | 4 | amp |
| TCGA-13-0724 | 12 | 1902812 | 2940069 | 1.019461441 | 3 | amp |
| TCGA-13-0724 | 12 | 2943768 | 3600888 | 1.573896015 | 4 | amp |
| TCGA-13-0724 | 12 | 4873105 | 7463358 | 0.991443185 | 3 | amp |
| TCGA-13-0724 | 12 | 7802097 | 9454906 | 0.898501275 | 3 | amp |
| TCGA-13-0724 | 12 | 9455198 | 9723520 | 1.243858242 | 4 | amp |
| TCGA-13-0724 | 12 | 29631744 | 30906727 | 0.964874351 | 3 | amp |
| TCGA-13-0724 | 12 | 31106907 | 31945136 | 1.514074059 | 4 | amp |
| TCGA-13-0724 | 12 | 39064507 | 48095436 | 0.923474891 | 3 | amp |
| TCGA-13-0724 | 12 | 48096426 | 50524522 | 1.455503732 | 4 | amp |
| TCGA-13-0724 | 12 | 50528256 | 51404554 | 1.009260702 | 3 | amp |
| TCGA-13-0724 | 12 | 51442080 | 54891684 | 1.3304865 | 4 | amp |
| TCGA-13-0724 | 12 | 54893133 | 54936473 | 0.88081113 | 3 | amp |
| TCGA-13-0724 | 12 | 54943622 | 55042118 | 1.283305814 | 4 | amp |
| TCGA-13-0724 | 12 | 55248335 | 56031626 | 0.895609279 | 3 | amp |
| TCGA-13-0724 | 12 | 56075652 | 58186872 | 1.53762198 | 4 | amp |
| TCGA-13-0724 | 12 | 58189922 | 75889459 | 0.904103615 | 3 | amp |
| TCGA-13-0724 | 12 | 88420204 | 108140246 | 0.986825113 | 3 | amp |
| TCGA-13-0724 | 12 | 108145168 | 112221151 | 1.21230399 | 4 | amp |

| | | | | | | |
|---|---|---|---|---|---|---|
| TCGA-13-0724 | 12 | 112223020 | 113448293 | 1.040548588 | 3 | amp |
| TCGA-13-0724 | 12 | 113495946 | 115121050 | 1.338631609 | 4 | amp |
| TCGA-13-0724 | 12 | 116399016 | 117615505 | 0.98556691 | 3 | amp |
| TCGA-13-0724 | 12 | 117624268 | 120295534 | 1.211085868 | 4 | amp |
| TCGA-13-0724 | 12 | 120306784 | 120903629 | 1.848244481 | 5 | amp |
| TCGA-13-0724 | 12 | 120934170 | 123097777 | 0.973549676 | 3 | amp |
| TCGA-13-0724 | 12 | 123098120 | 123497340 | 1.263274924 | 4 | amp |
| TCGA-13-0724 | 12 | 123498331 | 133779395 | 1.06174252 | 3 | amp |
| TCGA-13-0724 | 13 | 19419858 | 19419978 | 0.877040652 | 3 | amp |
| TCGA-13-0724 | 13 | 115089276 | 115091796 | 0.62665762 | 3 | amp |
| TCGA-13-0724 | 14 | 19377543 | 20784713 | 1.237626958 | 4 | amp |
| TCGA-13-0724 | 14 | 20794587 | 21561807 | 2.679120166 | 5 | amp |
| TCGA-13-0724 | 14 | 21623039 | 21681317 | 0.512921802 | 1 | loss |
| TCGA-13-0724 | 14 | 21698441 | 21816515 | 1.034442425 | 3 | amp |
| TCGA-13-0724 | 14 | 23013951 | 24911664 | 1.001401968 | 3 | amp |
| TCGA-13-0724 | 14 | 64653069 | 68220513 | 0.874162326 | 3 | amp |
| TCGA-13-0724 | 14 | 77236259 | 77984565 | 1.208315246 | 4 | amp |
| TCGA-13-0724 | 14 | 77987736 | 99927722 | 1.002921548 | 3 | amp |
| TCGA-13-0724 | 14 | 99929808 | 101393773 | 1.202597451 | 4 | amp |
| TCGA-13-0724 | 14 | 101488345 | 103442422 | 1.297240042 | 4 | amp |
| TCGA-13-0724 | 14 | 103444442 | 106494614 | 0.997856417 | 3 | amp |
| TCGA-13-0724 | 15 | 22929654 | 22980194 | 0.949535417 | 3 | amp |
| TCGA-13-0724 | 15 | 25420054 | 25523582 | 1.139465848 | 3 | amp |
| TCGA-13-0724 | 15 | 25936839 | 25981305 | 0.960288564 | 3 | amp |
| TCGA-13-0724 | 15 | 34675625 | 35087064 | 0.894833016 | 3 | amp |
| TCGA-13-0724 | 15 | 40556938 | 40907617 | 0.951432833 | 3 | amp |
| TCGA-13-0724 | 15 | 40942701 | 41279399 | 0.912638956 | 3 | amp |
| TCGA-13-0724 | 15 | 41768592 | 41797081 | 0.971399252 | 3 | amp |
| TCGA-13-0724 | 15 | 41797155 | 41820543 | 1.213969511 | 4 | amp |
| TCGA-13-0724 | 15 | 41821656 | 41870518 | 0.919949959 | 3 | amp |
| TCGA-13-0724 | 15 | 42103051 | 42457372 | 1.040185861 | 3 | amp |
| TCGA-13-0724 | 15 | 42820418 | 43038463 | 0.916446549 | 3 | amp |
| TCGA-13-0724 | 15 | 43476440 | 43622736 | 0.908729128 | 3 | amp |
| TCGA-13-0724 | 15 | 43772015 | 43851181 | 0.961028716 | 3 | amp |
| TCGA-13-0724 | 15 | 43874744 | 43910581 | 0.929205597 | 3 | amp |
| TCGA-13-0724 | 15 | 43975384 | 44010043 | 0.965705994 | 3 | amp |
| TCGA-13-0724 | 15 | 44036559 | 44128476 | 0.951965874 | 3 | amp |
| TCGA-13-0724 | 15 | 44175863 | 44705658 | 0.90381237 | 3 | amp |
| TCGA-13-0724 | 15 | 45357407 | 45464550 | 0.947770326 | 3 | amp |
| TCGA-13-0724 | 15 | 48051989 | 48461051 | 0.520785328 | 1 | loss |
| TCGA-13-0724 | 15 | 49284411 | 49319679 | 0.504208701 | 1 | loss |
| TCGA-13-0724 | 15 | 53809863 | 54015111 | 0.520738141 | 1 | loss |
| TCGA-13-0724 | 15 | 56676129 | 56999353 | 0.524718884 | 1 | loss |
| TCGA-13-0724 | 15 | 62146646 | 62199631 | 0.507832305 | 1 | loss |
| TCGA-13-0724 | 15 | 62226413 | 62299712 | 0.508026346 | 1 | loss |
| TCGA-13-0724 | 15 | 64983591 | 65234772 | 0.914585147 | 3 | amp |
| TCGA-13-0724 | 15 | 65471184 | 65686906 | 0.896398111 | 3 | amp |
| TCGA-13-0724 | 15 | 66206072 | 66587517 | 0.989945623 | 3 | amp |
| TCGA-13-0724 | 15 | 66629287 | 66801264 | 0.89937701 | 3 | amp |
| TCGA-13-0724 | 15 | 68486341 | 69080303 | 0.92405575 | 3 | amp |

| | | | | | | |
|---|---|---|---|---|---|---|
| TCGA-13-0724 | 15 | 69672135 | 70368525 | 0.883466163 | 3 | amp |
| TCGA-13-0724 | 15 | 70952462 | 70983729 | 0.49749075 | 1 | loss |
| TCGA-13-0724 | 15 | 71124352 | 71229190 | 1.069525285 | 3 | amp |
| TCGA-13-0724 | 15 | 72023447 | 72109956 | 0.920995966 | 3 | amp |
| TCGA-13-0724 | 15 | 72460038 | 72460188 | 1.701269974 | 4 | amp |
| TCGA-13-0724 | 15 | 72557406 | 72691301 | 0.897870583 | 3 | amp |
| TCGA-13-0724 | 15 | 72879545 | 72956874 | 0.9576436 | 3 | amp |
| TCGA-13-0724 | 15 | 73991929 | 74967503 | 1.043407163 | 3 | amp |
| TCGA-13-0724 | 15 | 75012762 | 75503513 | 1.292375327 | 4 | amp |
| TCGA-13-0724 | 15 | 75541508 | 75660033 | 0.982590055 | 3 | amp |
| TCGA-13-0724 | 15 | 75694131 | 76146837 | 0.930492927 | 3 | amp |
| TCGA-13-0724 | 15 | 76426533 | 76523757 | 0.962127349 | 3 | amp |
| TCGA-13-0724 | 15 | 77906473 | 78452550 | 0.946071977 | 3 | amp |
| TCGA-13-0724 | 15 | 78463768 | 78777302 | 0.941834993 | 3 | amp |
| TCGA-13-0724 | 15 | 78790369 | 78839098 | 0.951625093 | 3 | amp |
| TCGA-13-0724 | 15 | 78909330 | 79172947 | 0.993358728 | 3 | amp |
| TCGA-13-0724 | 15 | 79215247 | 79614617 | 0.979386795 | 3 | amp |
| TCGA-13-0724 | 15 | 88670364 | 89762292 | 0.901050083 | 3 | amp |
| TCGA-13-0724 | 15 | 89856108 | 90039803 | 0.93764148 | 3 | amp |
| TCGA-13-0724 | 15 | 90164582 | 90229779 | 0.924321242 | 3 | amp |
| TCGA-13-0724 | 15 | 90278692 | 90934115 | 1.022565493 | 3 | amp |
| TCGA-13-0724 | 15 | 91352307 | 91545465 | 0.97708552 | 3 | amp |
| TCGA-13-0724 | 15 | 92981501 | 93489132 | 0.92159542 | 3 | amp |
| TCGA-13-0724 | 15 | 95022168 | 99715367 | 0.912364886 | 3 | amp |
| TCGA-13-0724 | 15 | 100230419 | 100943002 | 0.945927848 | 3 | amp |
| TCGA-13-0724 | 15 | 101152406 | 102198083 | 0.950090955 | 3 | amp |
| TCGA-13-0724 | 15 | 102500733 | 102516522 | 1.221488117 | 4 | amp |
| TCGA-13-0724 | 16 | 66517 | 5040831 | 1.133264706 | 3 | amp |
| TCGA-13-0724 | 16 | 5046830 | 5145543 | 2.637876846 | 5 | amp |
| TCGA-13-0724 | 16 | 7102025 | 22000137 | 1.112091008 | 3 | amp |
| TCGA-13-0724 | 16 | 22120839 | 30212243 | 1.268197012 | 4 | amp |
| TCGA-13-0724 | 16 | 30212244 | 30366023 | 1.087580162 | 3 | amp |
| TCGA-13-0724 | 16 | 30369587 | 31504393 | 1.409442049 | 4 | amp |
| TCGA-13-0724 | 16 | 31504756 | 34326444 | 1.115092068 | 3 | amp |
| TCGA-13-0724 | 16 | 56601635 | 58555273 | 0.894328186 | 3 | amp |
| TCGA-13-0724 | 16 | 68071869 | 69959419 | 0.521431135 | 1 | loss |
| TCGA-13-0724 | 16 | 71209456 | 74683124 | 0.507311276 | 1 | loss |
| TCGA-13-0724 | 16 | 74719372 | 75327954 | 1.141406128 | 3 | amp |
| TCGA-13-0724 | 16 | 75338880 | 84014743 | 0.476388182 | 1 | loss |
| TCGA-13-0724 | 16 | 89629250 | 90244214 | 0.982941515 | 3 | amp |
| TCGA-13-0724 | 17 | 63618 | 2935768 | 1.024789882 | 3 | amp |
| TCGA-13-0724 | 17 | 3397608 | 3704481 | 1.055037203 | 3 | amp |
| TCGA-13-0724 | 17 | 3769161 | 3917492 | 1.232122477 | 4 | amp |
| TCGA-13-0724 | 17 | 4337211 | 5045804 | 1.025166142 | 3 | amp |
| TCGA-13-0724 | 17 | 5485130 | 6406930 | 1.031048844 | 3 | amp |
| TCGA-13-0724 | 17 | 6545546 | 6659473 | 1.062562649 | 3 | amp |
| TCGA-13-0724 | 17 | 6733480 | 7710672 | 1.058908917 | 3 | amp |
| TCGA-13-0724 | 17 | 7710695 | 7736893 | 1.861939386 | 5 | amp |
| TCGA-13-0724 | 17 | 7748820 | 10243570 | 0.992146263 | 3 | amp |
| TCGA-13-0724 | 17 | 16734996 | 18284878 | 1.015801437 | 3 | amp |

| | | | | | | |
|---|---|---|---|---|---|---|
| TCGA-13-0724 | 17 | 18785848 | 19685410 | 0.962448381 | 3 | amp |
| TCGA-13-0724 | 17 | 20641186 | 26925998 | 1.011996321 | 3 | amp |
| TCGA-13-0724 | 17 | 27008227 | 27959983 | 0.997962896 | 3 | amp |
| TCGA-13-0724 | 17 | 30611636 | 30690027 | 1.048480894 | 3 | amp |
| TCGA-13-0724 | 17 | 33326276 | 33510643 | 0.975522635 | 3 | amp |
| TCGA-13-0724 | 17 | 34062161 | 35307729 | 0.987390552 | 3 | amp |
| TCGA-13-0724 | 17 | 36003315 | 37687572 | 1.011127403 | 3 | amp |
| TCGA-13-0724 | 17 | 37762158 | 37903196 | 1.511508189 | 4 | amp |
| TCGA-13-0724 | 17 | 37922034 | 38548651 | 1.027245056 | 3 | amp |
| TCGA-13-0724 | 17 | 39471523 | 40478235 | 1.023259853 | 3 | amp |
| TCGA-13-0724 | 17 | 40673001 | 44116097 | 0.982005186 | 3 | amp |
| TCGA-13-0724 | 17 | 45773470 | 47123738 | 0.98924893 | 3 | amp |
| TCGA-13-0724 | 17 | 47126699 | 47590400 | 1.473421722 | 4 | amp |
| TCGA-13-0724 | 17 | 47655919 | 48768566 | 1.094392185 | 3 | amp |
| TCGA-13-0724 | 17 | 55191788 | 56650725 | 1.003575949 | 3 | amp |
| TCGA-13-0724 | 17 | 58079604 | 58236807 | 1.009751602 | 3 | amp |
| TCGA-13-0724 | 17 | 61495657 | 61824340 | 0.941852843 | 3 | amp |
| TCGA-13-0724 | 17 | 61902176 | 62506404 | 0.987557955 | 3 | amp |
| TCGA-13-0724 | 17 | 62602678 | 63632150 | 0.946032079 | 3 | amp |
| TCGA-13-0724 | 17 | 70643701 | 76089892 | 1.133081884 | 3 | amp |
| TCGA-13-0724 | 17 | 76094400 | 76201878 | 1.343310404 | 4 | amp |
| TCGA-13-0724 | 17 | 76201988 | 77705207 | 1.121483121 | 3 | amp |
| TCGA-13-0724 | 17 | 77707228 | 79684534 | 1.51889112 | 4 | amp |
| TCGA-13-0724 | 17 | 79686798 | 80282793 | 2.885939969 | 5 | amp |
| TCGA-13-0724 | 17 | 80319973 | 81188237 | 1.659282842 | 4 | amp |
| TCGA-13-0724 | 18 | 47273 | 47554 | 1.0397351 | 3 | amp |
| TCGA-13-0724 | 18 | 77893473 | 77960823 | 0.870016664 | 3 | amp |
| TCGA-13-0724 | 19 | 71882 | 6042154 | 1.302727073 | 4 | amp |
| TCGA-13-0724 | 19 | 6141497 | 12089967 | 1.077439994 | 3 | amp |
| TCGA-13-0724 | 19 | 12125733 | 15729002 | 3.000727952 | 5 | amp |
| TCGA-13-0724 | 19 | 15730267 | 16243127 | 1.721888846 | 4 | amp |
| TCGA-13-0724 | 19 | 16259502 | 17515027 | 2.174469306 | 5 | amp |
| TCGA-13-0724 | 19 | 17515152 | 17760210 | 1.699641207 | 4 | amp |
| TCGA-13-0724 | 19 | 17760296 | 17985388 | 2.287869286 | 5 | amp |
| TCGA-13-0724 | 19 | 17985446 | 18583695 | 1.33737611 | 4 | amp |
| TCGA-13-0724 | 19 | 18643389 | 23159929 | 0.881618935 | 3 | amp |
| TCGA-13-0724 | 19 | 23542308 | 29567193 | 1.302916638 | 4 | amp |
| TCGA-13-0724 | 19 | 29698399 | 31770676 | 3.036445701 | 5 | amp |
| TCGA-13-0724 | 19 | 32843719 | 35500247 | 1.200652623 | 4 | amp |
| TCGA-13-0724 | 19 | 35500276 | 36640568 | 2.246711131 | 5 | amp |
| TCGA-13-0724 | 19 | 36640667 | 38713110 | 1.330310442 | 4 | amp |
| TCGA-13-0724 | 19 | 38742901 | 40009809 | 2.206857477 | 5 | amp |
| TCGA-13-0724 | 19 | 40022869 | 40521709 | 1.751436007 | 4 | amp |
| TCGA-13-0724 | 19 | 40540258 | 48737757 | 0.880709085 | 3 | amp |
| TCGA-13-0724 | 19 | 48782970 | 51190094 | 1.250436793 | 4 | amp |
| TCGA-13-0724 | 19 | 51191127 | 54212128 | 0.910406026 | 3 | amp |
| TCGA-13-0724 | 19 | 54214208 | 56203451 | 1.458357706 | 4 | amp |
| TCGA-13-0724 | 19 | 56204301 | 57654063 | 0.85675515 | 3 | amp |
| TCGA-13-0724 | 19 | 57665724 | 59110878 | 1.338698119 | 4 | amp |
| TCGA-13-0724 | 2 | 41527 | 8877183 | 1.476565437 | 4 | amp |

| | | | | | | |
|---|---|---|---|---|---|---|
| TCGA-13-0724 | 2 | 8887212 | 9419541 | 1.082504497 | 3 | amp |
| TCGA-13-0724 | 2 | 9437381 | 9633130 | 1.369461376 | 4 | amp |
| TCGA-13-0724 | 2 | 9633854 | 9731702 | 1.065749137 | 3 | amp |
| TCGA-13-0724 | 2 | 9770253 | 11323624 | 1.445381201 | 4 | amp |
| TCGA-13-0724 | 2 | 11332260 | 11590254 | 1.006730502 | 3 | amp |
| TCGA-13-0724 | 2 | 11591734 | 15307489 | 1.451208279 | 4 | amp |
| TCGA-13-0724 | 2 | 15319055 | 24090820 | 1.135106247 | 3 | amp |
| TCGA-13-0724 | 2 | 24092521 | 24443994 | 1.337559067 | 4 | amp |
| TCGA-13-0724 | 2 | 24468973 | 25037423 | 1.103655396 | 3 | amp |
| TCGA-13-0724 | 2 | 25038289 | 29297153 | 1.403710361 | 4 | amp |
| TCGA-13-0724 | 2 | 29344200 | 29420577 | 0.885791009 | 3 | amp |
| TCGA-13-0724 | 2 | 29429997 | 31562568 | 1.35950917 | 4 | amp |
| TCGA-13-0724 | 2 | 31565045 | 33752521 | 1.12640239 | 3 | amp |
| TCGA-13-0724 | 2 | 33759368 | 33824321 | 1.409682007 | 4 | amp |
| TCGA-13-0724 | 2 | 36623723 | 45715484 | 1.129555758 | 3 | amp |
| TCGA-13-0724 | 2 | 45773864 | 47739627 | 1.361683442 | 4 | amp |
| TCGA-13-0724 | 2 | 48016516 | 70131472 | 1.117350603 | 3 | amp |
| TCGA-13-0724 | 2 | 70143212 | 73498056 | 1.436295601 | 4 | amp |
| TCGA-13-0724 | 2 | 73635692 | 73826670 | 0.937555797 | 3 | amp |
| TCGA-13-0724 | 2 | 73827756 | 74399915 | 1.375557345 | 4 | amp |
| TCGA-13-0724 | 2 | 74400263 | 74787467 | 2.58254985 | 5 | amp |
| TCGA-13-0724 | 2 | 74789326 | 75187783 | 1.60982781 | 4 | amp |
| TCGA-13-0724 | 2 | 75196482 | 84811432 | 1.106930707 | 3 | amp |
| TCGA-13-0724 | 2 | 85049012 | 86346225 | 1.50820554 | 4 | amp |
| TCGA-13-0724 | 2 | 86348599 | 90260280 | 1.121100567 | 3 | amp |
| TCGA-13-0724 | 2 | 90273672 | 95945788 | 1.349892627 | 4 | amp |
| TCGA-13-0724 | 2 | 95946931 | 96047457 | 2.080775827 | 5 | amp |
| TCGA-13-0724 | 2 | 96049683 | 96516991 | 1.406635821 | 4 | amp |
| TCGA-13-0724 | 2 | 96517848 | 96652702 | 0.912335749 | 3 | amp |
| TCGA-13-0724 | 2 | 96679536 | 97750020 | 1.587594234 | 4 | amp |
| TCGA-13-0724 | 2 | 97751396 | 98201610 | 0.983642695 | 3 | amp |
| TCGA-13-0724 | 2 | 98201744 | 100917297 | 1.496653624 | 4 | amp |
| TCGA-13-0724 | 2 | 100919323 | 113818531 | 1.06442278 | 3 | amp |
| TCGA-13-0724 | 2 | 113819644 | 114199047 | 1.44676066 | 4 | amp |
| TCGA-13-0724 | 2 | 114201331 | 121050896 | 1.059765516 | 3 | amp |
| TCGA-13-0724 | 2 | 121106642 | 122135269 | 1.371582869 | 4 | amp |
| TCGA-13-0724 | 2 | 122139781 | 127808107 | 1.030968793 | 3 | amp |
| TCGA-13-0724 | 2 | 127808342 | 127961156 | 1.399628348 | 4 | amp |
| TCGA-13-0724 | 2 | 128015087 | 128263333 | 1.103601796 | 3 | amp |
| TCGA-13-0724 | 2 | 128281249 | 128409349 | 1.442993434 | 4 | amp |
| TCGA-13-0724 | 2 | 128411907 | 130872904 | 1.036208668 | 3 | amp |
| TCGA-13-0724 | 2 | 130877527 | 130987236 | 1.385803411 | 4 | amp |
| TCGA-13-0724 | 2 | 131096658 | 138434194 | 1.05511827 | 3 | amp |
| TCGA-13-0724 | 2 | 149259981 | 179355583 | 0.922827105 | 3 | amp |
| TCGA-13-0724 | 2 | 191835375 | 193059284 | 1.00205462 | 3 | amp |
| TCGA-13-0724 | 2 | 196915851 | 209138820 | 0.992990733 | 3 | amp |
| TCGA-13-0724 | 2 | 216251336 | 217724807 | 1.02082621 | 3 | amp |
| TCGA-13-0724 | 2 | 218669103 | 219305634 | 1.331180963 | 4 | amp |
| TCGA-13-0724 | 2 | 219313912 | 219459034 | 1.003038778 | 3 | amp |
| TCGA-13-0724 | 2 | 219480413 | 219538700 | 1.352362048 | 4 | amp |

| | | | | | | |
|---|---|---|---|---|---|---|
| TCGA-13-0724 | 2 | 219539980 | 219677493 | 1.065984561 | 3 | amp |
| TCGA-13-0724 | 2 | 219677582 | 220506471 | 1.472850188 | 4 | amp |
| TCGA-13-0724 | 2 | 222290714 | 233244027 | 0.960594838 | 3 | amp |
| TCGA-13-0724 | 2 | 233244189 | 233527705 | 1.341931043 | 4 | amp |
| TCGA-13-0724 | 2 | 233536965 | 234237311 | 1.135986002 | 3 | amp |
| TCGA-13-0724 | 2 | 234238120 | 234377253 | 1.338047197 | 4 | amp |
| TCGA-13-0724 | 2 | 234389835 | 238256540 | 1.14221035 | 3 | amp |
| TCGA-13-0724 | 2 | 238256919 | 238683112 | 1.423589332 | 4 | amp |
| TCGA-13-0724 | 2 | 238688059 | 238925331 | 0.996723744 | 3 | amp |
| TCGA-13-0724 | 2 | 238933927 | 243160772 | 1.338008661 | 4 | amp |
| TCGA-13-0724 | 20 | 239682 | 464722 | 1.308983479 | 4 | amp |
| TCGA-13-0724 | 20 | 467002 | 2616670 | 1.112597496 | 3 | amp |
| TCGA-13-0724 | 20 | 2618028 | 2847267 | 1.292943127 | 4 | amp |
| TCGA-13-0724 | 20 | 2944899 | 3018801 | 1.098043171 | 3 | amp |
| TCGA-13-0724 | 20 | 3025017 | 3278868 | 1.456183976 | 4 | amp |
| TCGA-13-0724 | 20 | 3285048 | 3619588 | 0.958368472 | 3 | amp |
| TCGA-13-0724 | 20 | 3624797 | 3846801 | 1.50525381 | 4 | amp |
| TCGA-13-0724 | 20 | 3888568 | 3944712 | 0.916373926 | 3 | amp |
| TCGA-13-0724 | 20 | 4155656 | 4854779 | 1.39434468 | 4 | amp |
| TCGA-13-0724 | 20 | 4855161 | 17410183 | 0.974350128 | 3 | amp |
| TCGA-13-0724 | 20 | 17417290 | 21227225 | 1.51881394 | 4 | amp |
| TCGA-13-0724 | 20 | 21306860 | 21362753 | 0.990973556 | 3 | amp |
| TCGA-13-0724 | 20 | 21367424 | 23350419 | 1.534898295 | 4 | amp |
| TCGA-13-0724 | 20 | 23350722 | 23383729 | 1.026224825 | 3 | amp |
| TCGA-13-0724 | 20 | 23420863 | 25498496 | 1.537475804 | 4 | amp |
| TCGA-13-0724 | 20 | 25506983 | 30028590 | 1.072281379 | 3 | amp |
| TCGA-13-0724 | 20 | 30037784 | 33609195 | 1.447412463 | 4 | amp |
| TCGA-13-0724 | 20 | 33622898 | 33706581 | 1.009044796 | 3 | amp |
| TCGA-13-0724 | 20 | 33711614 | 34293259 | 1.36941558 | 4 | amp |
| TCGA-13-0724 | 20 | 34295022 | 34487610 | 1.099667856 | 3 | amp |
| TCGA-13-0724 | 20 | 34501024 | 35526954 | 1.351353671 | 4 | amp |
| TCGA-13-0724 | 20 | 35532545 | 35695579 | 1.118956117 | 3 | amp |
| TCGA-13-0724 | 20 | 35695607 | 35812799 | 1.389687424 | 4 | amp |
| TCGA-13-0724 | 20 | 35826787 | 35867243 | 1.015962011 | 3 | amp |
| TCGA-13-0724 | 20 | 35869651 | 36361524 | 1.507461611 | 4 | amp |
| TCGA-13-0724 | 20 | 36365742 | 36470840 | 0.890616138 | 3 | amp |
| TCGA-13-0724 | 20 | 36488219 | 36662544 | 1.460323366 | 4 | amp |
| TCGA-13-0724 | 20 | 36668781 | 36718322 | 0.904693348 | 3 | amp |
| TCGA-13-0724 | 20 | 36758575 | 37078199 | 1.437662636 | 4 | amp |
| TCGA-13-0724 | 20 | 37117048 | 37203633 | 0.927848788 | 3 | amp |
| TCGA-13-0724 | 20 | 37209955 | 37612440 | 1.427648294 | 4 | amp |
| TCGA-13-0724 | 20 | 37617417 | 39730040 | 1.033514288 | 3 | amp |
| TCGA-13-0724 | 20 | 39741373 | 40045450 | 1.440073986 | 4 | amp |
| TCGA-13-0724 | 20 | 40045793 | 40162248 | 0.924121368 | 3 | amp |
| TCGA-13-0724 | 20 | 40179899 | 40739169 | 1.519543848 | 4 | amp |
| TCGA-13-0724 | 20 | 40743787 | 42089762 | 1.088019134 | 3 | amp |
| TCGA-13-0724 | 20 | 42143573 | 43600848 | 1.476653965 | 4 | amp |
| TCGA-13-0724 | 20 | 43607027 | 43883201 | 1.07246587 | 3 | amp |
| TCGA-13-0724 | 20 | 43922375 | 44054519 | 1.579311365 | 4 | amp |
| TCGA-13-0724 | 20 | 44108264 | 44405900 | 1.044726066 | 3 | amp |

| | | | | | | |
|---|---|---|---|---|---|---|
| TCGA-13-0724 | 20 | 44416427 | 44997628 | 1.531990379 | 4 | amp |
| TCGA-13-0724 | 20 | 44999062 | 45022263 | 0.920965972 | 3 | amp |
| TCGA-13-0724 | 20 | 45022647 | 45891221 | 1.335980287 | 4 | amp |
| TCGA-13-0724 | 20 | 45904968 | 46288229 | 1.117776762 | 3 | amp |
| TCGA-13-0724 | 20 | 46288367 | 47592764 | 1.507515503 | 4 | amp |
| TCGA-13-0724 | 20 | 47601231 | 47770658 | 1.060345637 | 3 | amp |
| TCGA-13-0724 | 20 | 47782485 | 48253988 | 1.328963178 | 4 | amp |
| TCGA-13-0724 | 20 | 48256130 | 48497576 | 1.102613832 | 3 | amp |
| TCGA-13-0724 | 20 | 48500342 | 48747498 | 1.459559552 | 4 | amp |
| TCGA-13-0724 | 20 | 48759978 | 49493203 | 2.228098453 | 5 | amp |
| TCGA-13-0724 | 20 | 49507935 | 55111540 | 1.471322232 | 4 | amp |
| TCGA-13-0724 | 20 | 55206826 | 56099289 | 1.875816975 | 5 | amp |
| TCGA-13-0724 | 20 | 56136429 | 58439464 | 1.552967762 | 4 | amp |
| TCGA-13-0724 | 20 | 58440410 | 58482508 | 0.964169043 | 3 | amp |
| TCGA-13-0724 | 20 | 58486792 | 58645835 | 1.462345222 | 4 | amp |
| TCGA-13-0724 | 20 | 58883518 | 62926333 | 1.939410074 | 5 | amp |
| TCGA-13-0724 | 21 | 17763895 | 28210607 | 1.046067905 | 3 | amp |
| TCGA-13-0724 | 21 | 28210725 | 30257720 | 1.410134787 | 4 | amp |
| TCGA-13-0724 | 21 | 30302740 | 30353624 | 1.000542409 | 3 | amp |
| TCGA-13-0724 | 21 | 30354603 | 30403124 | 1.641516098 | 4 | amp |
| TCGA-13-0724 | 21 | 30407149 | 32493160 | 1.125310507 | 3 | amp |
| TCGA-13-0724 | 21 | 32496805 | 34030223 | 1.526346967 | 4 | amp |
| TCGA-13-0724 | 21 | 34037181 | 34136848 | 1.149706449 | 3 | amp |
| TCGA-13-0724 | 21 | 34142068 | 38460213 | 1.461026116 | 4 | amp |
| TCGA-13-0724 | 21 | 38460571 | 38520956 | 1.111395955 | 3 | amp |
| TCGA-13-0724 | 21 | 38522363 | 40553810 | 1.382696094 | 4 | amp |
| TCGA-13-0724 | 21 | 40558907 | 40665967 | 1.054801722 | 3 | amp |
| TCGA-13-0724 | 21 | 40667628 | 42771004 | 1.422725869 | 4 | amp |
| TCGA-13-0724 | 21 | 42771043 | 44521568 | 1.839929999 | 5 | amp |
| TCGA-13-0724 | 21 | 44524408 | 48111215 | 1.406578068 | 4 | amp |
| TCGA-13-0724 | 22 | 16084594 | 16414534 | 0.962406625 | 3 | amp |
| TCGA-13-0724 | 22 | 17052859 | 17265323 | 1.007300129 | 3 | amp |
| TCGA-13-0724 | 22 | 17443544 | 21106062 | 0.992951895 | 3 | amp |
| TCGA-13-0724 | 22 | 21322144 | 22330106 | 1.04243512 | 3 | amp |
| TCGA-13-0724 | 22 | 23237535 | 24384235 | 1.017407386 | 3 | amp |
| TCGA-13-0724 | 22 | 24455637 | 24645496 | 0.978399963 | 3 | amp |
| TCGA-13-0724 | 22 | 24807463 | 25084092 | 0.971189763 | 3 | amp |
| TCGA-13-0724 | 22 | 25158355 | 25853296 | 1.004895692 | 3 | amp |
| TCGA-13-0724 | 22 | 26157017 | 26264379 | 0.945038387 | 3 | amp |
| TCGA-13-0724 | 22 | 26902697 | 28250963 | 0.948162352 | 3 | amp |
| TCGA-13-0724 | 22 | 29537895 | 29756000 | 1.00838407 | 3 | amp |
| TCGA-13-0724 | 22 | 30060955 | 30069486 | 1.101582481 | 3 | amp |
| TCGA-13-0724 | 22 | 30074098 | 30421824 | 1.475004419 | 4 | amp |
| TCGA-13-0724 | 22 | 30489906 | 31333753 | 1.019985622 | 3 | amp |
| TCGA-13-0724 | 22 | 31346322 | 31795750 | 1.015643376 | 3 | amp |
| TCGA-13-0724 | 22 | 31985377 | 32100740 | 1.008040446 | 3 | amp |
| TCGA-13-0724 | 22 | 32297697 | 32446025 | 0.992438742 | 3 | amp |
| TCGA-13-0724 | 22 | 32579498 | 32598438 | 1.109774531 | 3 | amp |
| TCGA-13-0724 | 22 | 32804135 | 32828564 | 1.04150456 | 3 | amp |
| TCGA-13-0724 | 22 | 32909594 | 33245539 | 1.036934937 | 3 | amp |

| | | | | | | |
|---|---|---|---|---|---|---|
| TCGA-13-0724 | 22 | 35688925 | 36124977 | 1.031797517 | 3 | amp |
| TCGA-13-0724 | 22 | 36587120 | 38333880 | 1.034685862 | 3 | amp |
| TCGA-13-0724 | 22 | 38336657 | 38437146 | 1.258649047 | 4 | amp |
| TCGA-13-0724 | 22 | 38453746 | 38864343 | 1.054694581 | 3 | amp |
| TCGA-13-0724 | 22 | 38945860 | 39410435 | 0.980645405 | 3 | amp |
| TCGA-13-0724 | 22 | 39411527 | 39448760 | 1.387431581 | 4 | amp |
| TCGA-13-0724 | 22 | 39474893 | 40078703 | 1.024857215 | 3 | amp |
| TCGA-13-0724 | 22 | 40361957 | 41175190 | 0.962400423 | 3 | amp |
| TCGA-13-0724 | 22 | 41601318 | 42141082 | 1.027881474 | 3 | amp |
| TCGA-13-0724 | 22 | 42174703 | 43950982 | 0.999803354 | 3 | amp |
| TCGA-13-0724 | 22 | 44221876 | 45567582 | 1.000388305 | 3 | amp |
| TCGA-13-0724 | 22 | 45593629 | 45736336 | 0.998906558 | 3 | amp |
| TCGA-13-0724 | 22 | 45813447 | 46085807 | 1.068755897 | 3 | amp |
| TCGA-13-0724 | 22 | 46238844 | 51237627 | 1.038216033 | 3 | amp |
| TCGA-13-0724 | 3 | 9771163 | 11276168 | 1.025295832 | 3 | amp |
| TCGA-13-0724 | 3 | 12779139 | 15531147 | 1.002662463 | 3 | amp |
| TCGA-13-0724 | 3 | 38986892 | 39554953 | 1.165991552 | 3 | amp |
| TCGA-13-0724 | 3 | 46620550 | 48436134 | 1.062087303 | 3 | amp |
| TCGA-13-0724 | 3 | 48445854 | 48611027 | 1.651553586 | 4 | amp |
| TCGA-13-0724 | 3 | 48611080 | 48667169 | 1.919515742 | 5 | amp |
| TCGA-13-0724 | 3 | 48667297 | 50155882 | 1.48121078 | 4 | amp |
| TCGA-13-0724 | 3 | 50197051 | 50616364 | 2.620812281 | 5 | amp |
| TCGA-13-0724 | 3 | 50617262 | 52448097 | 1.580314962 | 4 | amp |
| TCGA-13-0724 | 3 | 52448467 | 52582284 | 2.090738723 | 5 | amp |
| TCGA-13-0724 | 3 | 52584394 | 52818522 | 1.381305398 | 4 | amp |
| TCGA-13-0724 | 3 | 52819002 | 52889495 | 1.931030103 | 5 | amp |
| TCGA-13-0724 | 3 | 52939101 | 53338323 | 1.60433275 | 4 | amp |
| TCGA-13-0724 | 3 | 53346219 | 125696047 | 1.00271929 | 3 | amp |
| TCGA-13-0724 | 3 | 125701080 | 129304979 | 1.313916871 | 4 | amp |
| TCGA-13-0724 | 3 | 129370270 | 133375730 | 0.918581897 | 3 | amp |
| TCGA-13-0724 | 3 | 133376632 | 134090328 | 1.418724875 | 4 | amp |
| TCGA-13-0724 | 3 | 134197344 | 158262077 | 1.166641213 | 3 | amp |
| TCGA-13-0724 | 3 | 158308863 | 183778158 | 1.490327632 | 4 | amp |
| TCGA-13-0724 | 3 | 183801647 | 184429622 | 2.279828523 | 5 | amp |
| TCGA-13-0724 | 3 | 184542376 | 186839130 | 1.69777425 | 4 | amp |
| TCGA-13-0724 | 3 | 186915259 | 187444722 | 1.942785376 | 5 | amp |
| TCGA-13-0724 | 3 | 187446783 | 195377354 | 1.512181556 | 4 | amp |
| TCGA-13-0724 | 3 | 195389402 | 196018281 | 1.968655474 | 5 | amp |
| TCGA-13-0724 | 3 | 196022848 | 196654768 | 1.654244193 | 4 | amp |
| TCGA-13-0724 | 3 | 196656468 | 196751384 | 2.195795738 | 5 | amp |
| TCGA-13-0724 | 3 | 196753520 | 197955154 | 1.5738907 | 4 | amp |
| TCGA-13-0724 | 4 | 53323 | 494407 | 0.921832205 | 3 | amp |
| TCGA-13-0724 | 4 | 499461 | 2133048 | 1.223187052 | 4 | amp |
| TCGA-13-0724 | 4 | 2158487 | 2916808 | 0.991466929 | 3 | amp |
| TCGA-13-0724 | 4 | 2927729 | 3101180 | 1.510826794 | 4 | amp |
| TCGA-13-0724 | 4 | 3105505 | 3190868 | 0.957467517 | 3 | amp |
| TCGA-13-0724 | 4 | 3201442 | 4199841 | 1.201494361 | 4 | amp |
| TCGA-13-0724 | 4 | 4204119 | 6578497 | 1.107967859 | 3 | amp |
| TCGA-13-0724 | 4 | 6580112 | 6607098 | 1.682870953 | 4 | amp |
| TCGA-13-0724 | 4 | 6611440 | 8010862 | 1.134508447 | 3 | amp |

| | | | | | | |
|---|---|---|---|---|---|---|
| TCGA-13-0724 | 4 | 8021311 | 8621346 | 1.288276416 | 4 | amp |
| TCGA-13-0724 | 4 | 9366250 | 10586624 | 0.955787757 | 3 | amp |
| TCGA-13-0724 | 4 | 15709081 | 17830015 | 0.888581657 | 3 | amp |
| TCGA-13-0724 | 4 | 25664088 | 26484961 | 0.947043191 | 3 | amp |
| TCGA-13-0724 | 4 | 37590415 | 38945187 | 0.868273807 | 3 | amp |
| TCGA-13-0724 | 4 | 39408561 | 41601049 | 0.880919159 | 3 | amp |
| TCGA-13-0724 | 4 | 46979058 | 47853198 | 0.9256893 | 3 | amp |
| TCGA-13-0724 | 4 | 47901355 | 57898774 | 0.942631068 | 3 | amp |
| TCGA-13-0724 | 4 | 71507681 | 72102389 | 0.909393729 | 3 | amp |
| TCGA-13-0724 | 5 | 151610 | 847739 | 0.998469764 | 3 | amp |
| TCGA-13-0724 | 5 | 864572 | 886914 | 1.278078567 | 4 | amp |
| TCGA-13-0724 | 5 | 887470 | 896964 | 0.944425284 | 3 | amp |
| TCGA-13-0724 | 5 | 907186 | 5182467 | 0.95419086 | 3 | amp |
| TCGA-13-0724 | 5 | 24511409 | 26906271 | 0.480127143 | 1 | loss |
| TCGA-13-0724 | 5 | 38435176 | 38451608 | 1.179056052 | 3 | amp |
| TCGA-13-0724 | 5 | 40730344 | 40777737 | 0.957987378 | 3 | amp |
| TCGA-13-0724 | 5 | 41142897 | 41382641 | 0.505882272 | 1 | loss |
| TCGA-13-0724 | 5 | 52337936 | 52394524 | 0.494006833 | 1 | loss |
| TCGA-13-0724 | 5 | 102493834 | 102526732 | 0.46036651 | 1 | loss |
| TCGA-13-0724 | 5 | 108704234 | 110097492 | 0.52176433 | 1 | loss |
| TCGA-13-0724 | 5 | 110439400 | 110730503 | 0.518564912 | 1 | loss |
| TCGA-13-0724 | 5 | 112899511 | 113831915 | 0.508722206 | 1 | loss |
| TCGA-13-0724 | 5 | 114462196 | 114515780 | 1.194952557 | 4 | amp |
| TCGA-13-0724 | 5 | 114548069 | 114577329 | 0.883208294 | 3 | amp |
| TCGA-13-0724 | 5 | 114588772 | 115148976 | 1.195356594 | 4 | amp |
| TCGA-13-0724 | 5 | 115151859 | 115177418 | 1.000765559 | 3 | amp |
| TCGA-13-0724 | 5 | 118532048 | 118837821 | 0.537611658 | 1 | loss |
| TCGA-13-0724 | 5 | 127497421 | 127616066 | 0.514912924 | 1 | loss |
| TCGA-13-0724 | 5 | 131321065 | 131543608 | 0.906509897 | 3 | amp |
| TCGA-13-0724 | 5 | 132084986 | 132210179 | 0.946658479 | 3 | amp |
| TCGA-13-0724 | 5 | 133695564 | 134011819 | 0.88365157 | 3 | amp |
| TCGA-13-0724 | 5 | 139188970 | 139260619 | 0.966177567 | 3 | amp |
| TCGA-13-0724 | 5 | 139714236 | 139815885 | 0.918908542 | 3 | amp |
| TCGA-13-0724 | 5 | 139918483 | 140896616 | 0.914633196 | 3 | amp |
| TCGA-13-0724 | 5 | 140967743 | 141353350 | 0.957192324 | 3 | amp |
| TCGA-13-0724 | 5 | 145539932 | 145562113 | 0.94573152 | 3 | amp |
| TCGA-13-0724 | 5 | 148998462 | 149274859 | 0.938029429 | 3 | amp |
| TCGA-13-0724 | 5 | 149294447 | 149498422 | 0.963268802 | 3 | amp |
| TCGA-13-0724 | 5 | 149498989 | 149514602 | 1.233296923 | 4 | amp |
| TCGA-13-0724 | 5 | 149515069 | 150102565 | 1.01858881 | 3 | amp |
| TCGA-13-0724 | 5 | 150404874 | 150639516 | 0.904485594 | 3 | amp |
| TCGA-13-0724 | 5 | 150885091 | 150930456 | 0.90647911 | 3 | amp |
| TCGA-13-0724 | 5 | 151043055 | 151055780 | 0.938486274 | 3 | amp |
| TCGA-13-0724 | 5 | 167631321 | 167919877 | 0.92440128 | 3 | amp |
| TCGA-13-0724 | 5 | 168093426 | 168201431 | 0.91972722 | 3 | amp |
| TCGA-13-0724 | 5 | 176005353 | 176317743 | 0.926216358 | 3 | amp |
| TCGA-13-0724 | 5 | 176728685 | 177036738 | 0.988041741 | 3 | amp |
| TCGA-13-0724 | 5 | 177473373 | 178294114 | 0.961136361 | 3 | amp |
| TCGA-13-0724 | 5 | 178408636 | 179136079 | 0.942589037 | 3 | amp |
| TCGA-13-0724 | 5 | 179153660 | 179980488 | 0.946204799 | 3 | amp |

| | | | | | | |
|---|---|---|---|---|---|---|
| TCGA-13-0724 | 5 | 180030170 | 180220022 | 0.969844925 | 3 | amp |
| TCGA-13-0724 | 5 | 180477048 | 180760833 | 0.955731673 | 3 | amp |
| TCGA-13-0724 | 6 | 105907 | 17856366 | 1.119397847 | 3 | amp |
| TCGA-13-0724 | 6 | 17873577 | 31557910 | 1.507575149 | 4 | amp |
| TCGA-13-0724 | 6 | 31560416 | 32052517 | 2.552321798 | 5 | amp |
| TCGA-13-0724 | 6 | 32053505 | 32411729 | 1.664263601 | 4 | amp |
| TCGA-13-0724 | 6 | 32427550 | 32783135 | 1.044542325 | 3 | amp |
| TCGA-13-0724 | 6 | 32784622 | 34803275 | 1.742610096 | 4 | amp |
| TCGA-13-0724 | 6 | 34803878 | 41712313 | 1.086596712 | 3 | amp |
| TCGA-13-0724 | 6 | 41712320 | 43276568 | 1.542982668 | 4 | amp |
| TCGA-13-0724 | 6 | 43304872 | 44328364 | 1.072857809 | 3 | amp |
| TCGA-13-0724 | 6 | 55263965 | 73815094 | 1.061729406 | 3 | amp |
| TCGA-13-0724 | 6 | 73821018 | 75818928 | 1.458348586 | 4 | amp |
| TCGA-13-0724 | 6 | 75822914 | 76380472 | 1.068049356 | 3 | amp |
| TCGA-13-0724 | 6 | 76385107 | 76712733 | 1.354048844 | 4 | amp |
| TCGA-13-0724 | 6 | 76713568 | 84056092 | 1.117750754 | 3 | amp |
| TCGA-13-0724 | 6 | 84061760 | 90661624 | 1.313901105 | 4 | amp |
| TCGA-13-0724 | 6 | 90718261 | 102516423 | 1.127875262 | 3 | amp |
| TCGA-13-0724 | 6 | 105177497 | 112381306 | 1.28962747 | 4 | amp |
| TCGA-13-0724 | 6 | 112382135 | 137525674 | 1.08902378 | 3 | amp |
| TCGA-13-0724 | 6 | 137527238 | 142384219 | 1.297228591 | 4 | amp |
| TCGA-13-0724 | 6 | 142396745 | 147705930 | 1.108650699 | 3 | amp |
| TCGA-13-0724 | 6 | 147728408 | 152690797 | 1.36447752 | 4 | amp |
| TCGA-13-0724 | 6 | 152694142 | 155451554 | 1.117052543 | 3 | amp |
| TCGA-13-0724 | 6 | 155458258 | 160679822 | 1.4372937 | 4 | amp |
| TCGA-13-0724 | 6 | 160818972 | 161505710 | 1.073714985 | 3 | amp |
| TCGA-13-0724 | 6 | 161507400 | 171055029 | 1.460105802 | 4 | amp |
| TCGA-13-0724 | 7 | 540048 | 5983641 | 1.070585464 | 3 | amp |
| TCGA-13-0724 | 7 | 5984691 | 6006800 | 1.436458536 | 4 | amp |
| TCGA-13-0724 | 7 | 6012981 | 6786871 | 0.965575649 | 3 | amp |
| TCGA-13-0724 | 7 | 6790831 | 6826700 | 1.349217021 | 4 | amp |
| TCGA-13-0724 | 7 | 6829205 | 6864192 | 0.9407606 | 3 | amp |
| TCGA-13-0724 | 7 | 30468941 | 31611818 | 0.997871388 | 3 | amp |
| TCGA-13-0724 | 7 | 43283457 | 44054291 | 0.90374835 | 3 | amp |
| TCGA-13-0724 | 7 | 44056017 | 47698300 | 1.235016402 | 4 | amp |
| TCGA-13-0724 | 7 | 47698455 | 48238015 | 0.935382113 | 3 | amp |
| TCGA-13-0724 | 7 | 50121312 | 51093092 | 0.996081158 | 3 | amp |
| TCGA-13-0724 | 7 | 51094182 | 57142378 | 1.319491271 | 4 | amp |
| TCGA-13-0724 | 7 | 57187542 | 69900812 | 1.11520457 | 3 | amp |
| TCGA-13-0724 | 7 | 70163520 | 72861615 | 1.294685584 | 4 | amp |
| TCGA-13-0724 | 7 | 72861657 | 72925243 | 1.12722254 | 3 | amp |
| TCGA-13-0724 | 7 | 72951583 | 75055749 | 1.407353445 | 4 | amp |
| TCGA-13-0724 | 7 | 75066758 | 75187135 | 1.156153484 | 3 | amp |
| TCGA-13-0724 | 7 | 75187454 | 76673123 | 1.36959773 | 4 | amp |
| TCGA-13-0724 | 7 | 76682064 | 77998561 | 0.964319295 | 3 | amp |
| TCGA-13-0724 | 7 | 87822372 | 92300854 | 0.908228 | 3 | amp |
| TCGA-13-0724 | 7 | 96338947 | 97814464 | 0.946059819 | 3 | amp |
| TCGA-13-0724 | 7 | 97816129 | 99235946 | 1.311378919 | 4 | amp |
| TCGA-13-0724 | 7 | 99245915 | 99514413 | 0.859998587 | 3 | amp |
| TCGA-13-0724 | 7 | 99521100 | 99986777 | 1.245383534 | 4 | amp |

| | | | | | | |
|---|---|---|---|---|---|---|
| TCGA-13-0724 | 7 | 99987469 | 100007228 | 0.876163435 | 3 | amp |
| TCGA-13-0724 | 7 | 100013537 | 102330919 | 1.373981792 | 4 | amp |
| TCGA-13-0724 | 7 | 102343839 | 103051033 | 0.932745205 | 3 | amp |
| TCGA-13-0724 | 7 | 105098184 | 111617382 | 0.898872321 | 3 | amp |
| TCGA-13-0724 | 7 | 126542544 | 127979885 | 1.004365958 | 3 | amp |
| TCGA-13-0724 | 7 | 127983638 | 129125724 | 1.271081041 | 4 | amp |
| TCGA-13-0724 | 7 | 129297182 | 140080195 | 0.989907838 | 3 | amp |
| TCGA-13-0724 | 7 | 140082221 | 140404756 | 1.193828993 | 4 | amp |
| TCGA-13-0724 | 7 | 140434333 | 142563427 | 0.945720564 | 3 | amp |
| TCGA-13-0724 | 7 | 142563656 | 143104797 | 1.231340318 | 4 | amp |
| TCGA-13-0724 | 7 | 143140453 | 150720855 | 1.09590893 | 3 | amp |
| TCGA-13-0724 | 7 | 150725589 | 151097376 | 1.430910392 | 4 | amp |
| TCGA-13-0724 | 7 | 151127079 | 158935247 | 1.011607999 | 3 | amp |
| TCGA-13-0724 | 8 | 13162734 | 39691550 | 0.514030698 | 1 | loss |
| TCGA-13-0724 | 8 | 39694632 | 93088296 | 0.960247333 | 3 | amp |
| TCGA-13-0724 | 8 | 94713448 | 95384626 | 2.511812635 | 5 | amp |
| TCGA-13-0724 | 8 | 95390387 | 95844430 | 1.616342729 | 4 | amp |
| TCGA-13-0724 | 8 | 95848715 | 102678933 | 1.068599087 | 3 | amp |
| TCGA-13-0724 | 8 | 102701495 | 105010505 | 1.488240453 | 4 | amp |
| TCGA-13-0724 | 8 | 105025639 | 113421264 | 0.942775474 | 3 | amp |
| TCGA-13-0724 | 8 | 113484757 | 114449114 | 1.552822235 | 4 | amp |
| TCGA-13-0724 | 8 | 116426241 | 125487560 | 0.977982782 | 3 | amp |
| TCGA-13-0724 | 8 | 125498018 | 132002833 | 2.578450625 | 5 | amp |
| TCGA-13-0724 | 8 | 132051529 | 133596043 | 1.054748505 | 3 | amp |
| TCGA-13-0724 | 8 | 133622399 | 133880473 | 2.210514697 | 5 | amp |
| TCGA-13-0724 | 8 | 133881962 | 142151482 | 1.091224021 | 3 | amp |
| TCGA-13-0724 | 8 | 142154242 | 145692774 | 1.412843476 | 4 | amp |
| TCGA-13-0724 | 8 | 145692790 | 146279593 | 1.109513177 | 3 | amp |
| TCGA-13-0724 | 9 | 14753 | 135064 | 0.896637113 | 3 | amp |
| TCGA-13-0724 | 9 | 32989553 | 35148642 | 0.891230002 | 3 | amp |
| TCGA-13-0724 | 9 | 35397629 | 35958080 | 0.932377411 | 3 | amp |
| TCGA-13-0724 | 9 | 43082751 | 65650110 | 0.514345714 | 1 | loss |
| TCGA-13-0724 | 9 | 67930630 | 69432768 | 0.467913982 | 1 | loss |
| TCGA-13-0724 | 9 | 116276685 | 117396162 | 0.901235897 | 3 | amp |
| TCGA-13-0724 | 9 | 126125095 | 130341277 | 0.899549046 | 3 | amp |
| TCGA-13-0724 | 9 | 130430352 | 134006247 | 1.003483702 | 3 | amp |
| TCGA-13-0724 | 9 | 134103512 | 139658511 | 1.0325579 | 3 | amp |
| TCGA-13-0724 | 9 | 139686105 | 141071671 | 1.405846653 | 4 | amp |
| TCGA-13-0724 | X | 200797 | 2945554 | 0.860263409 | 3 | amp |
| TCGA-13-0724 | X | 14926893 | 14937982 | 0.453077004 | 1 | loss |
| TCGA-13-0724 | X | 16656894 | 16701402 | 0.946335278 | 3 | amp |
| TCGA-13-0724 | X | 18660049 | 18725982 | 0.990206285 | 3 | amp |
| TCGA-13-0724 | X | 18911567 | 19023015 | 0.888605909 | 3 | amp |
| TCGA-13-0724 | X | 21444546 | 21613537 | 0.462833578 | 1 | loss |
| TCGA-13-0724 | X | 31986423 | 32486840 | 0.504200608 | 1 | loss |
| TCGA-13-0724 | X | 46952260 | 47705753 | 0.92333453 | 3 | amp |
| TCGA-13-0724 | X | 48317309 | 49364879 | 0.910172978 | 3 | amp |
| TCGA-13-0724 | X | 52240408 | 52544598 | 0.881227027 | 3 | amp |
| TCGA-13-0724 | X | 52936213 | 53308842 | 0.880479726 | 3 | amp |
| TCGA-13-0724 | X | 54469818 | 54840987 | 0.864057478 | 3 | amp |

| | | | | | | |
|---|---|---|---|---|---|---|
| TCGA-13-0724 | X | 57020571 | 69419756 | 0.915011443 | 3 | amp |
| TCGA-13-0724 | X | 69478325 | 69510657 | 0.910498713 | 3 | amp |
| TCGA-13-0724 | X | 69644826 | 70514407 | 0.863609251 | 3 | amp |
| TCGA-13-0724 | X | 74273192 | 74523352 | 0.526684623 | 1 | loss |
| TCGA-13-0724 | X | 79932058 | 79952419 | 0.465078434 | 1 | loss |
| TCGA-13-0724 | X | 106061857 | 106097536 | 0.489853226 | 1 | loss |
| TCGA-13-0724 | X | 107811820 | 108636322 | 0.548076876 | 1 | loss |
| TCGA-13-0724 | X | 114082545 | 114384507 | 0.517772086 | 1 | loss |
| TCGA-13-0724 | X | 128873123 | 129215326 | 0.894481146 | 3 | amp |
| TCGA-13-0724 | X | 134852665 | 134983903 | 0.891418754 | 3 | amp |
| TCGA-13-0724 | X | 138664585 | 138724711 | 0.495524335 | 1 | loss |
| TCGA-13-0724 | X | 150867199 | 151304146 | 0.884754697 | 3 | amp |
| TCGA-13-0724 | X | 151869142 | 153577430 | 1.01758906 | 3 | amp |
| TCGA-13-0724 | X | 153577681 | 153594876 | 1.224682148 | 4 | amp |
| TCGA-13-0724 | X | 153594878 | 154066069 | 0.982171052 | 3 | amp |
| TCGA-13-0724 | X | 155227378 | 155254972 | 0.9299921 | 3 | amp |
